# Supplementary material for: On-tissue dataset-dependent MALDI-TIMS-MS2 bioimaging
Source: Nat Commun. 2023 Nov 18;14:7495. doi: 10.1038/s41467-023-43298-9 (PMC10657435; doi:10.1038/s41467-023-43298-9)
Supplement: Supplementary file 4 — Source Data file [file merged_speclibrary_matches.pdf.pdf › merged_speclibrary_matches.pdf.pdf]

PI 38:6 **0.821**

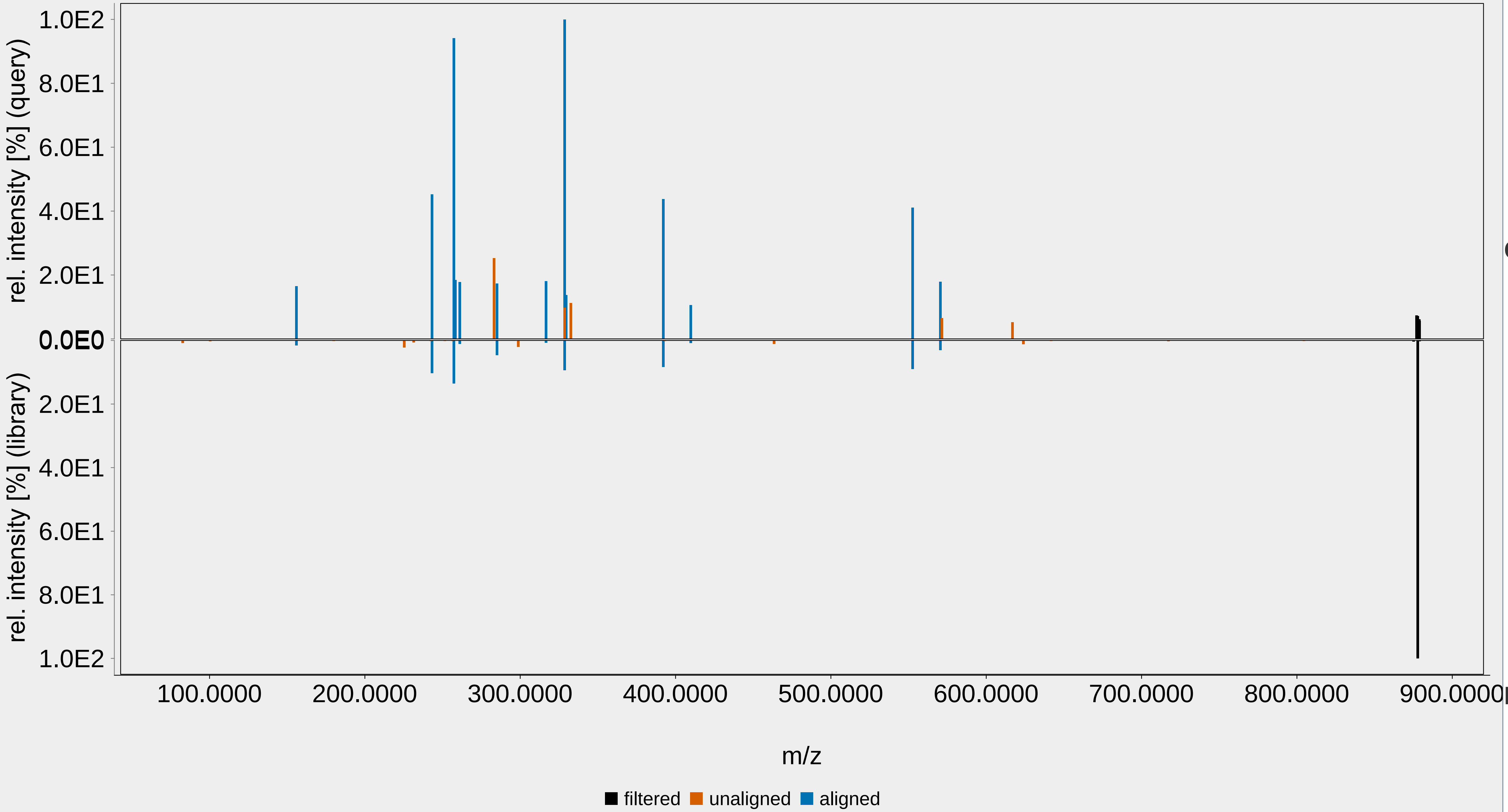

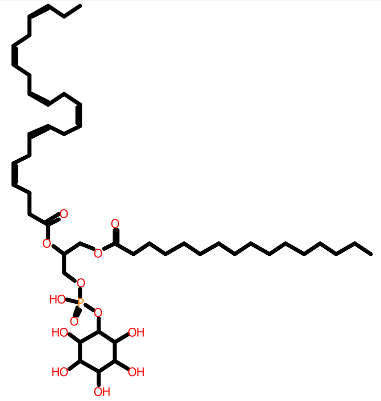

Compound information

Name: PI 38:6

Formula: C47H79O13P

Mol. weight: 883.1119999999999

Exact mass: 883.1119999999999

Ion type: [M-H]-

Precursor m/z: 881.514

RT: 8.246706

Polarity: negative

InChI:

InChI key:

SMILES:

Instrument information

Instrument type: LC-ESI-QTOF

Instrument: AB Sciex TripleTOF 5600+ system

Ms level: MS2

Collision energy: 45V

Database links

Pubchem: 138154339

MoNA ID: LQB00686

Other information

Principal investigator: Kazutaka Ikeda, Hiroshi

Data collector: Kazutaka Ikeda, Hiroshi

PI 38:6 **0.821**

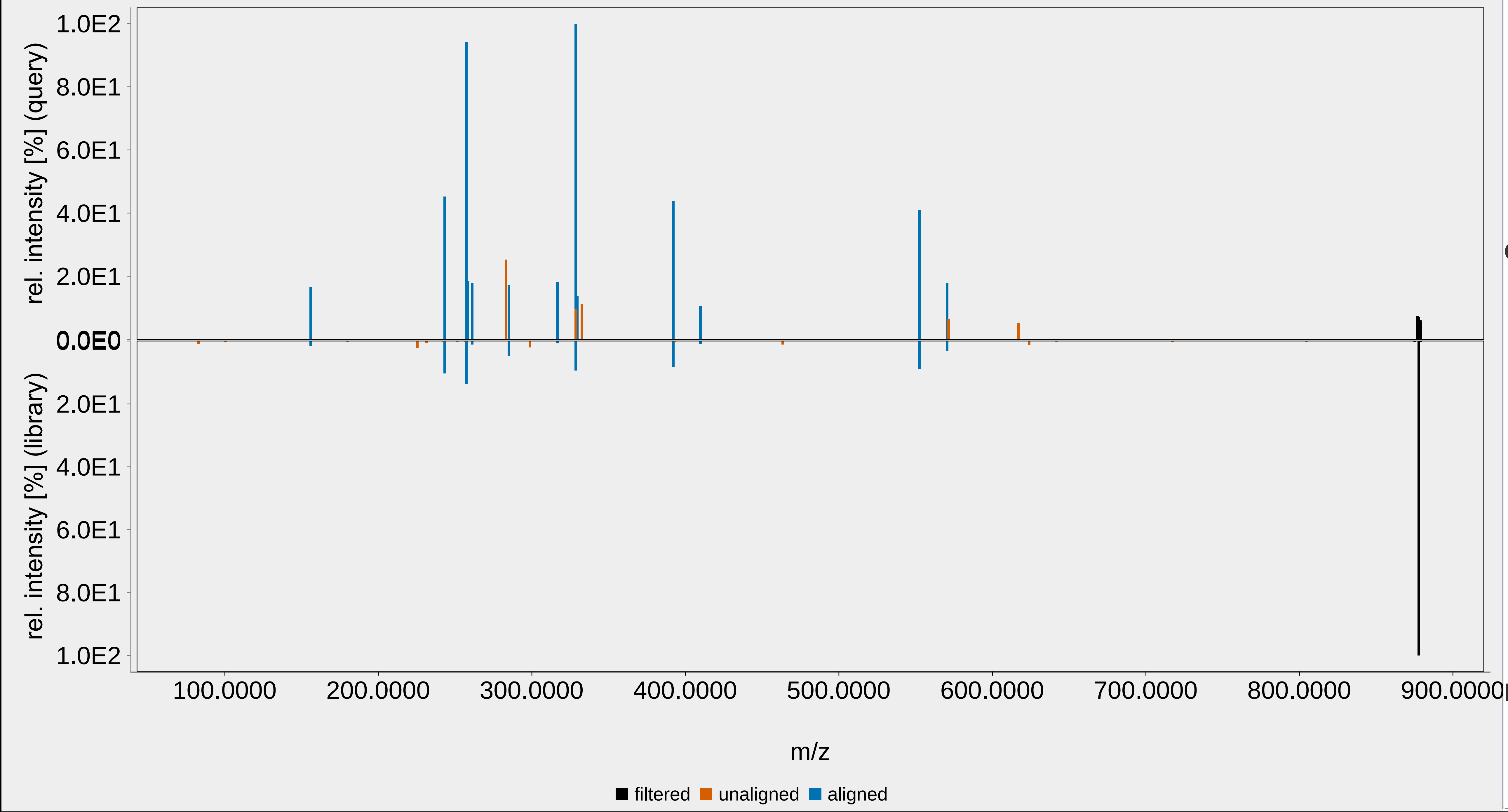

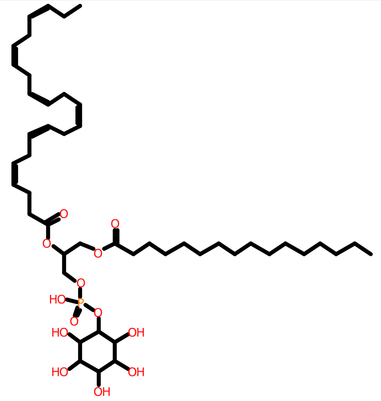

Compound information

Name: PI 38:6

Formula: C47H79O13P

Mol. weight: 883.1119999999999

Exact mass: 883.1119999999999

Ion type: [M-H]-

Precursor m/z: 881.514

RT: 8.246706

Polarity: negative

InChI:

InChI key:

SMILES:

Instrument information

Instrument type: LC-ESI-QTOF

Instrument: AB Sciex TripleTOF 5600+ system

Ms level: MS2

Collision energy: 45V

Database links

Pubchem: 138154339

MoNA ID: LQB00300

Other information

Principal investigator: Kazutaka Ikeda, Hiroshi

Data collector: Kazutaka Ikeda, Hiroshi

PI 36:4 0.864

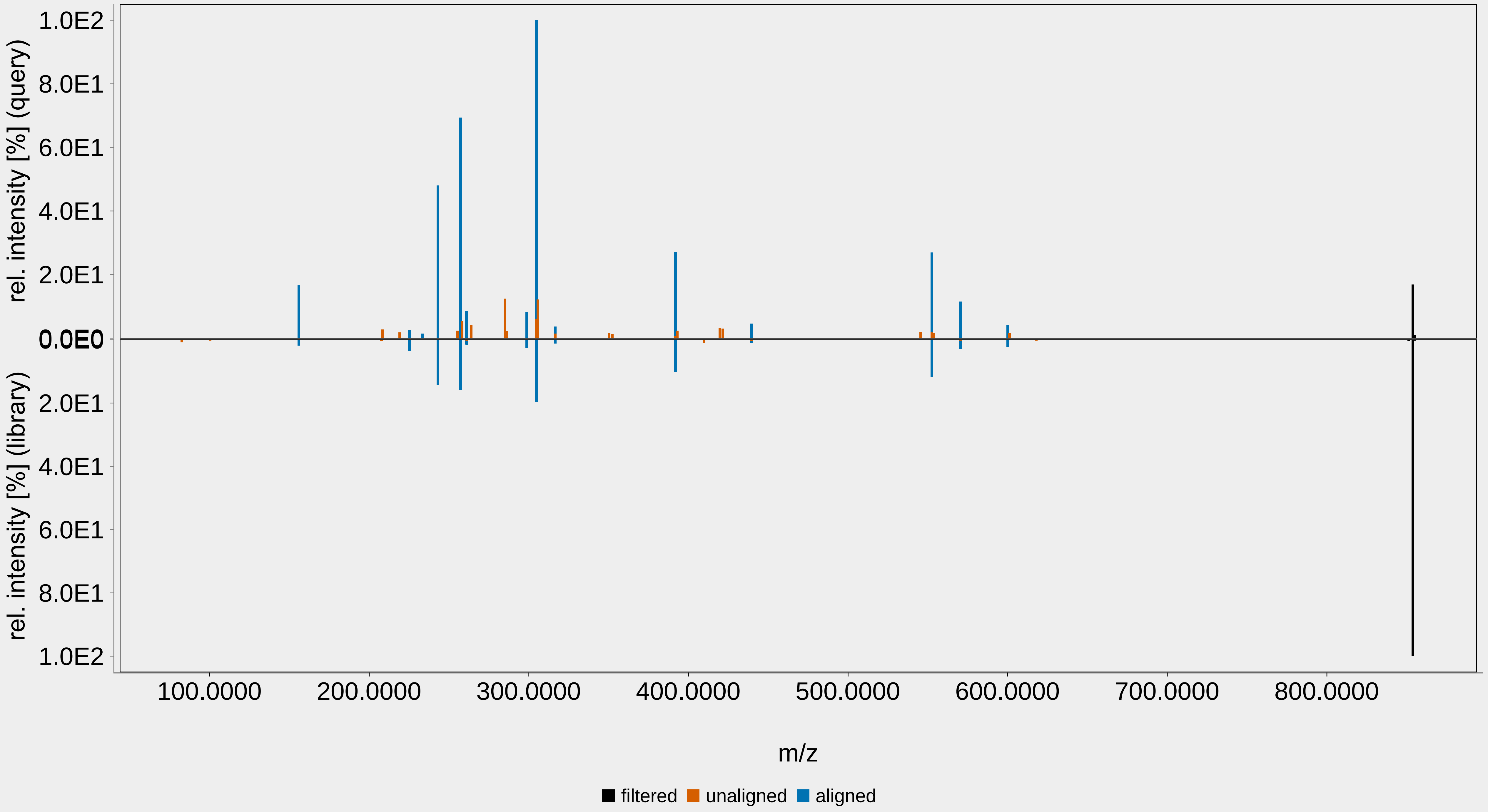

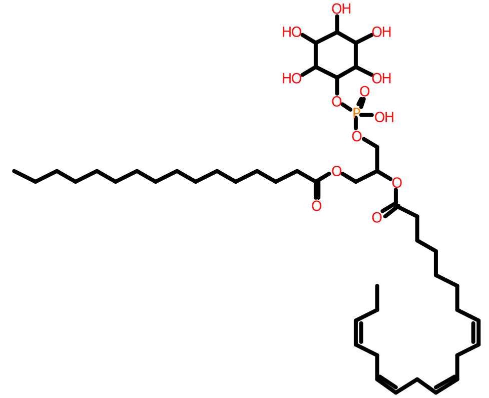

Compound information

Name: PI 36:4

Formula: C45H79O13P

Mol. weight: 859.0899999999999

Exact mass: 859.0899999999999

Ion type: [M-H]-

Precursor m/z: 857.5197

RT: 8.301669

Polarity: negative

InChI:

InChI key:

SMILES:

Instrument information

Instrument type: LC-ESI-QTOF

Instrument: AB Sciex TripleTOF 5600+ system

Ms level: MS2

Collision energy: 45V

Database links

Pubchem: 138284476

MoNA ID: LQB00683

Other information

Principal investigator: Kazutaka Ikeda, Hiroshi

Data collector: Kazutaka Ikeda, Hiroshi

PI 36:4

0.864

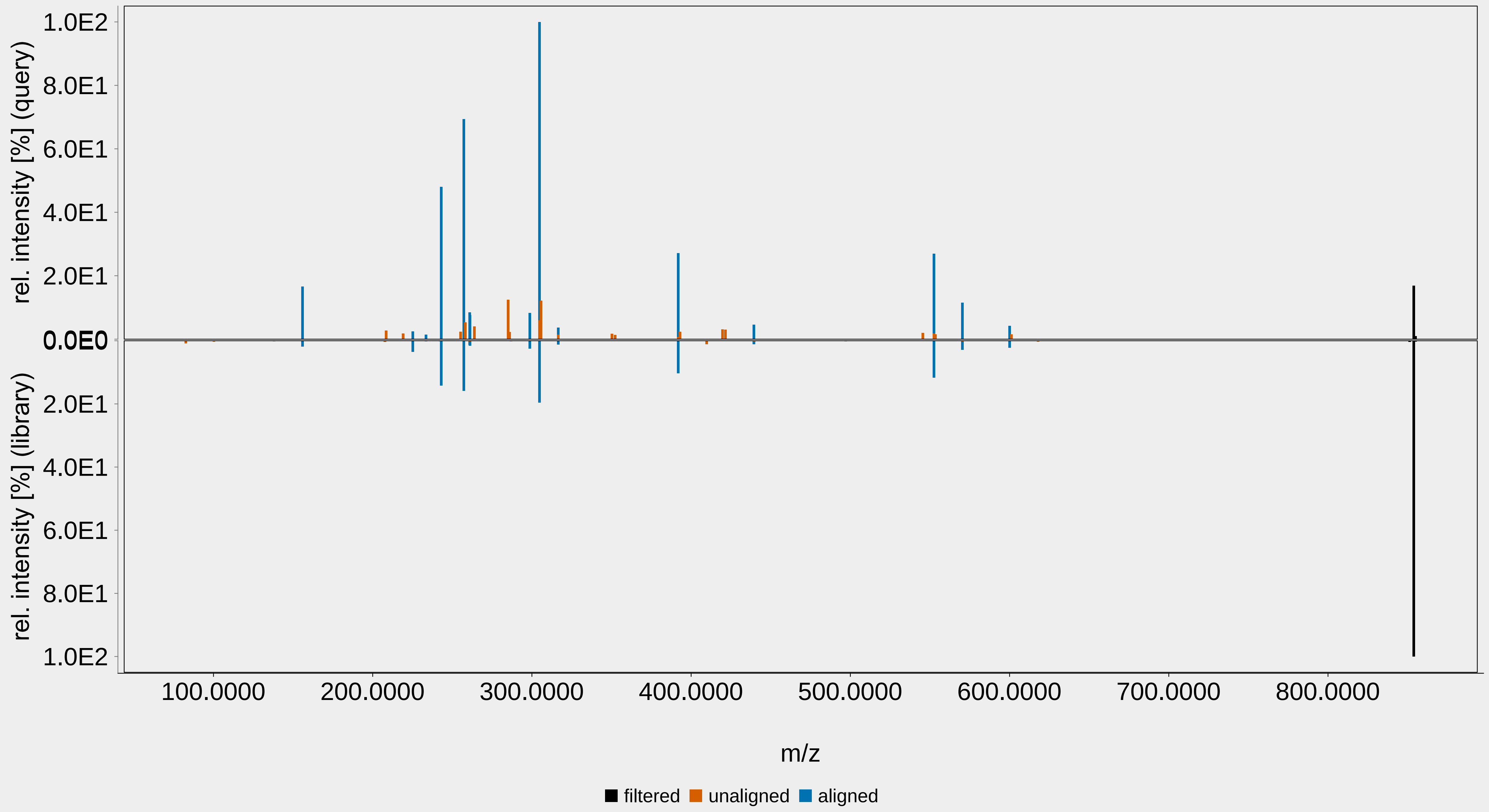

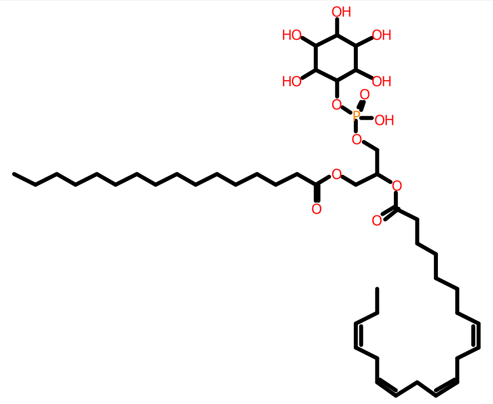

Compound information

Name: PI 36:4

Formula: C45H79O13P

Mol. weight: 859.0899999999999

Exact mass: 859.0899999999999

Ion type: [M-H]-

Precursor m/z: 857.5197

RT: 8.301669

Polarity: negative

InChI:

InChI key:

SMILES:

Instrument information

Instrument type: LC-ESI-QTOF

Instrument: AB Sciex TripleTOF 5600+ system

Ms level: MS2

Collision energy: 45V

Database links

Pubchem: 138284476

MoNA ID: LQB00297

Other information

Principal investigator: Kazutaka Ikeda, Hiroshi

Data collector: Kazutaka Ikeda, Hiroshi

Canrenone **0.939**

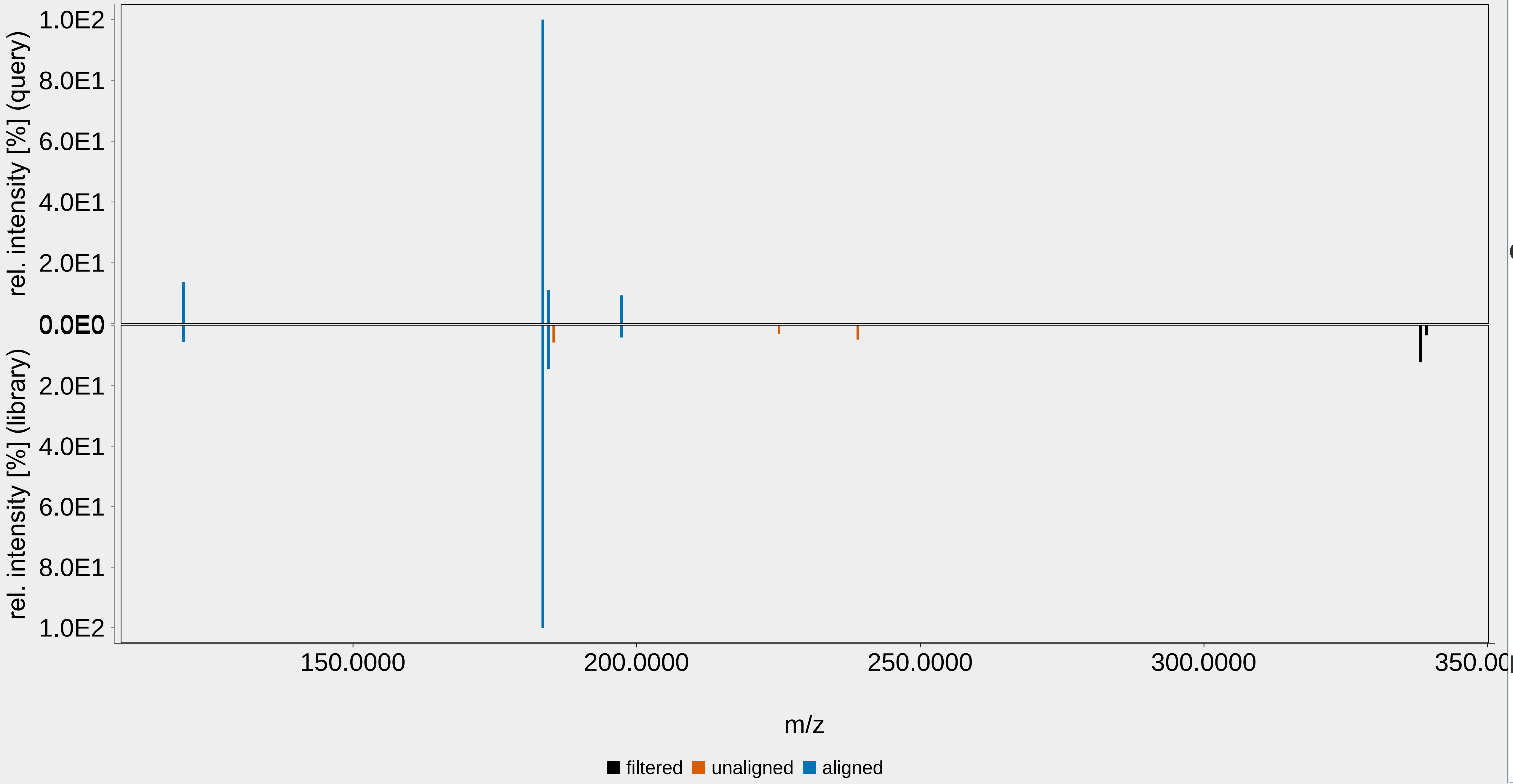

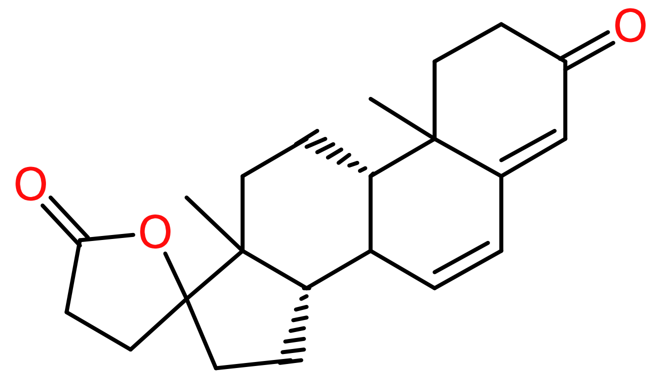

Compound information

Name: Canrenone

Formula: C22H28O3

Exact mass: 340.2038448

Ion type: [M-H]-

Precursor m/z: 339.1966

Polarity: NEGATIVE

InChI:

InChI key:

SMILES:

Num peaks: 9

Instrument information

Instrument type: LC-ESI-QTOF

Instrument: Bruker maXis Impact

Ms level: MS2

Collision energy: 40 eV

Database links

Other information

Entry ID: MSBNK-Athens_Univ-AU239460

Comment: Parent=339.1966

Canrenone, (9S,14S)-10,13-dimethylspiro[2,8,9,11,12,14,15,16-octahydro-1H-cyclopenta[a]phenanthrene-17,5`-oxolane]-2`,3-dione **0.939**

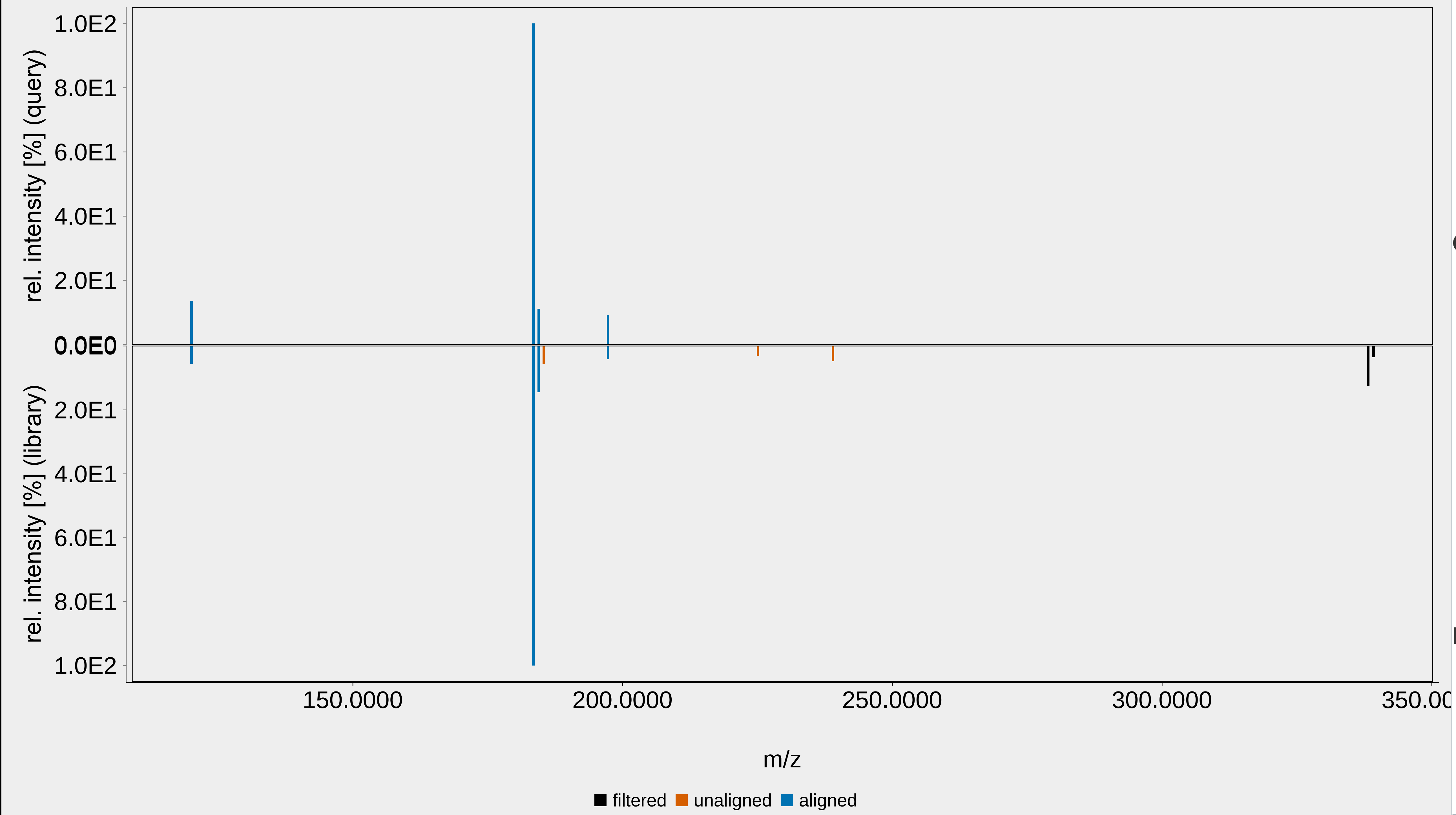

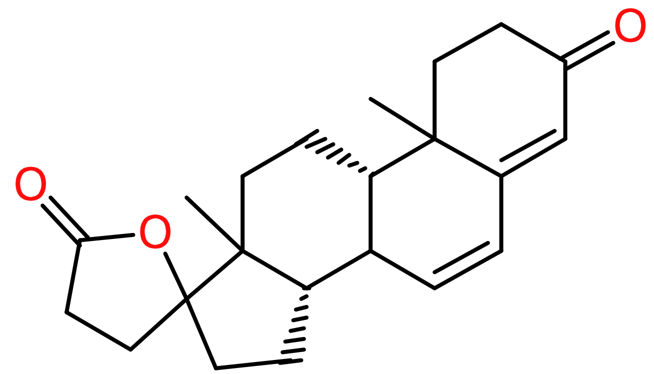

Compound information

Name: Canrenone,

Formula: C22H28O3

Mol. weight: 340.2038448

Exact mass: 340.2038448

Ion type: [M-H]-

Precursor m/z: 339.1966

Polarity: negative

InChI:

InChI key:

SMILES:

Instrument information

Instrument type: LC-ESI-QTOF

Instrument: Bruker maXis Impact

Ion source: ESI

Resolution: 35000

Ms level: MS2

Collision energy: 40 eV

Database links

Pubchem: 24728626

MoNA ID: AU239460

Chemspider: 21112020

CAS: 976-71-6

Other information

Principal investigator: Nikiforos Alygizakis,

Data collector: Nikiforos Alygizakis, Katerina

PA 18:0_22:6

0.827

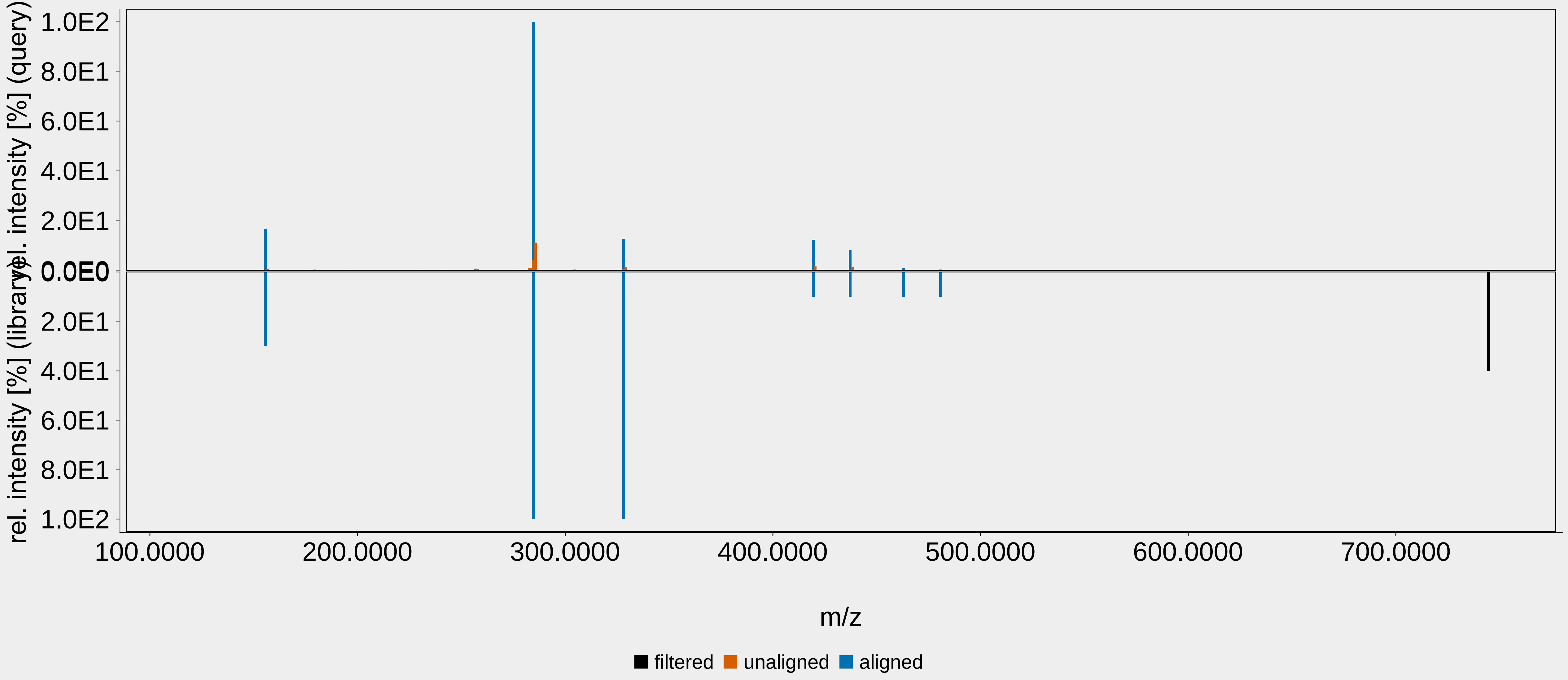

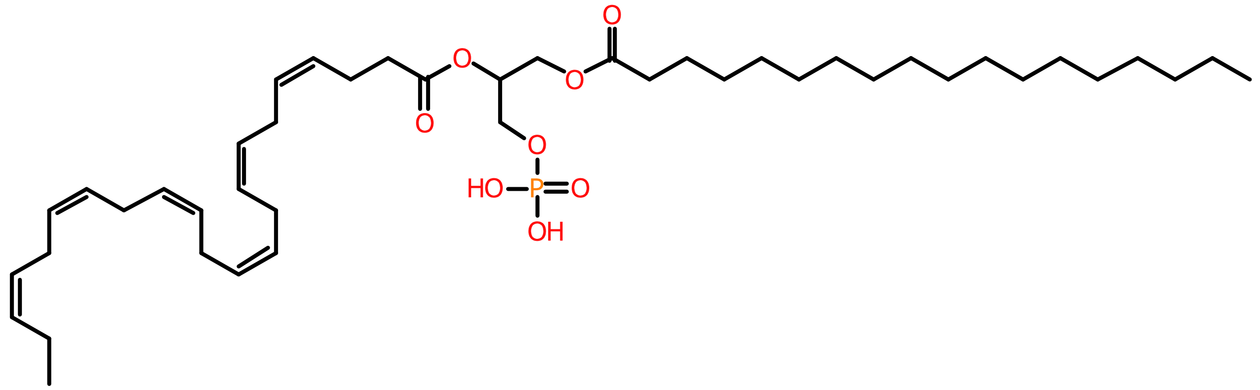

Compound information

Name: PA 18:0_22:6

Formula: C43H73O8P

Ion type: [M-H]-

Precursor m/z: 747.49703

Ccs: 266.76

InChI key:

SMILES:

Num peaks: 8

Instrument information

Database links

Other information

Spectral Match to 1-Octadecanoyl-2-(4Z,7Z,10Z,13Z,16Z,19Z-docosahexaenoyl)-sn-glycero-3-phospho-L-serine from NIST14

0.881

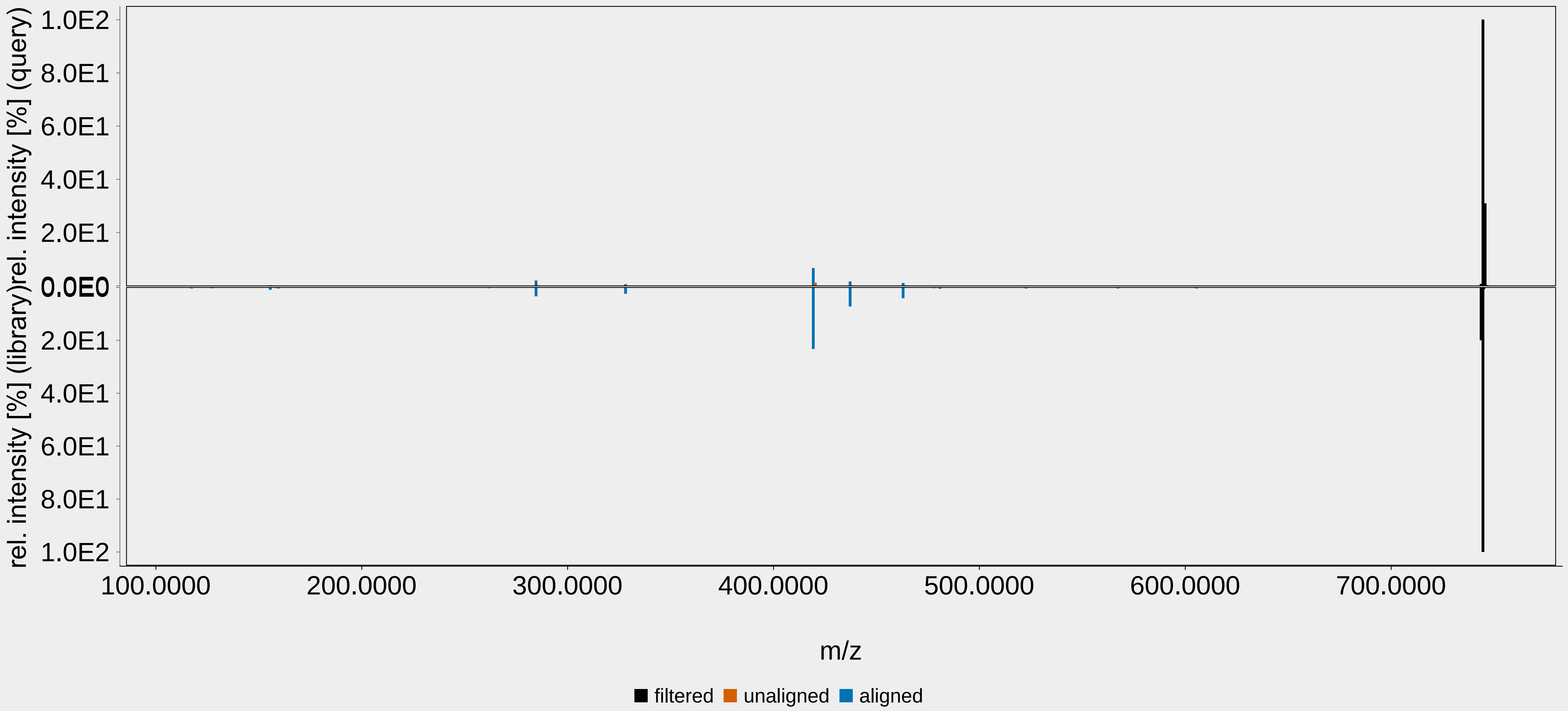

Compound information

Name: Spectral Match to

Exact mass: 0.0

Ion type: M-H-C3H5NO2

Precursor m/z: 747.498

Charge: 1

Polarity: Negative

Instrument information

Instrument type: IT/ion trap

Ion source: ESI

Ms level: 2

Acquisition: Isolated

Database links

CAS: 137173989

Other information

Principal investigator: Data from Katrina

Data collector: Data deposited by alchemistmatt

Entry ID: CCMSLIB00003134591

[1-[[2,3-dihydroxypropoxy]-hydroxyphosphoryl]oxy-3-[octadec-11-enoyl]oxypropan-2-yl] octadecanoate

0.758

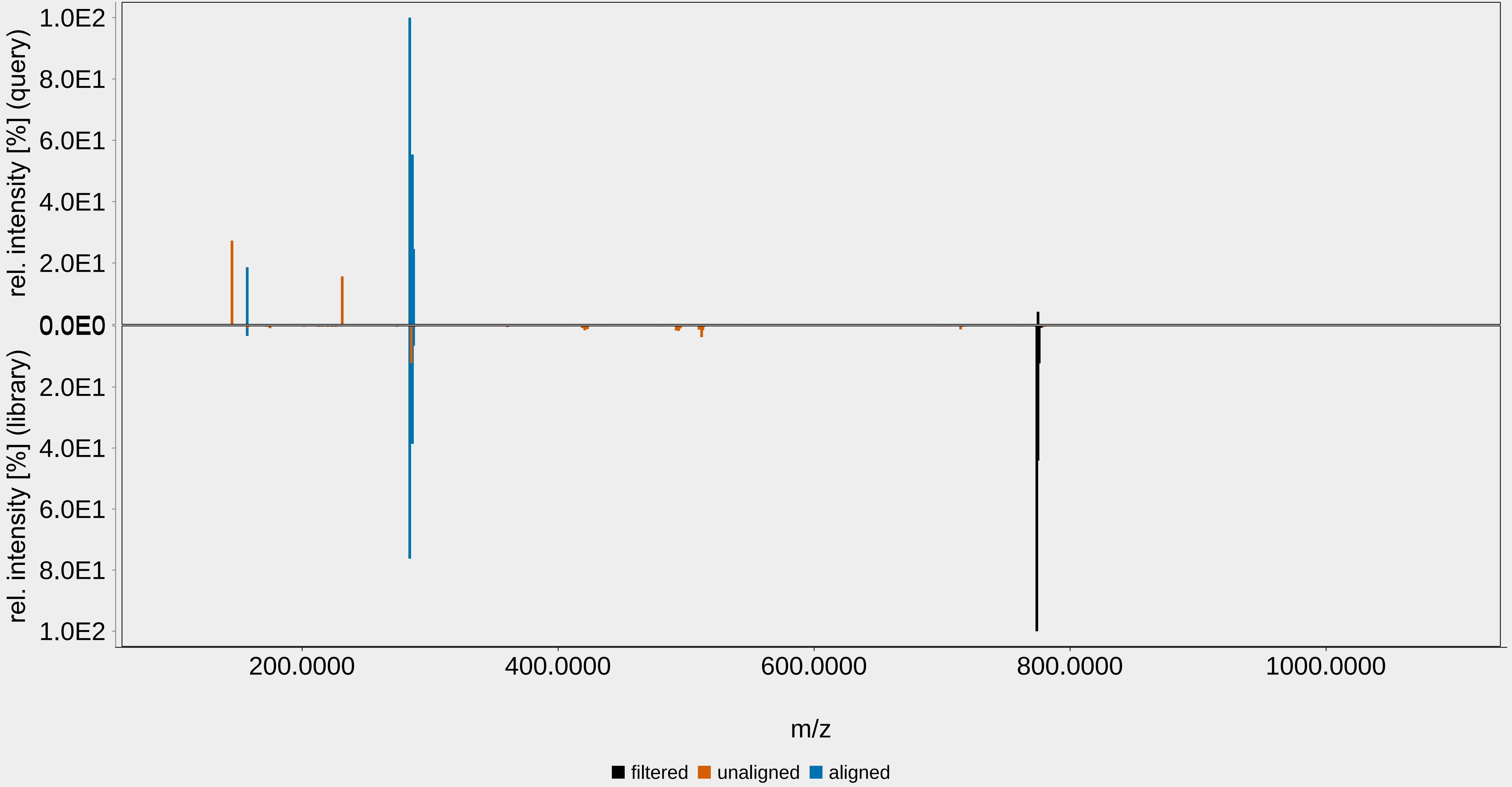

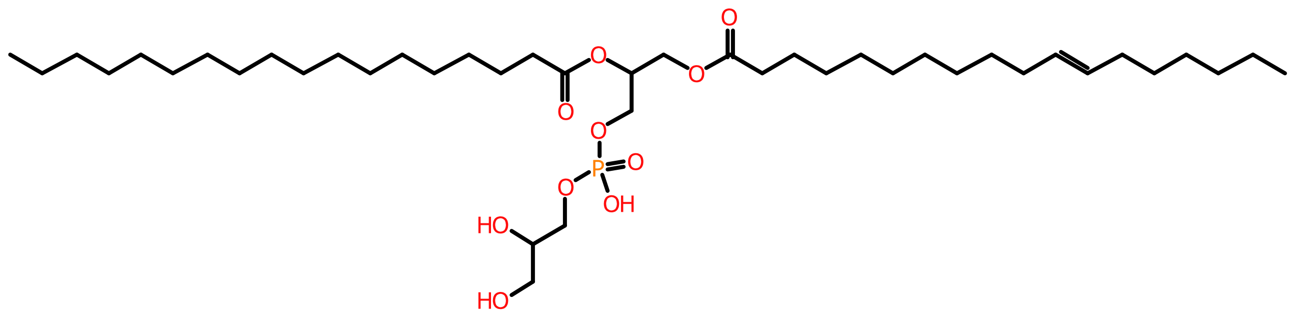

Compound information

Name:

Formula: C42H81O10P

Mol. weight: 776.557

Exact mass: 776.557

Ion type: [M-H]-

Precursor m/z: 775.549

Polarity: negative

InChI:

InChI key:

SMILES:

Instrument information

Instrument: qTof

Ms level: MS2

Database links

Other information

Principal investigator: O Laprevote, UMR 8038

Data collector: O Laprevote, UMR 8038

PG(18:0_18:1) - [2.3-dihydroxypropoxy][2-[octadec-9-enoyloxy]-3-(octadecanoyloxy)propoxy]phosphinic acid

0.775

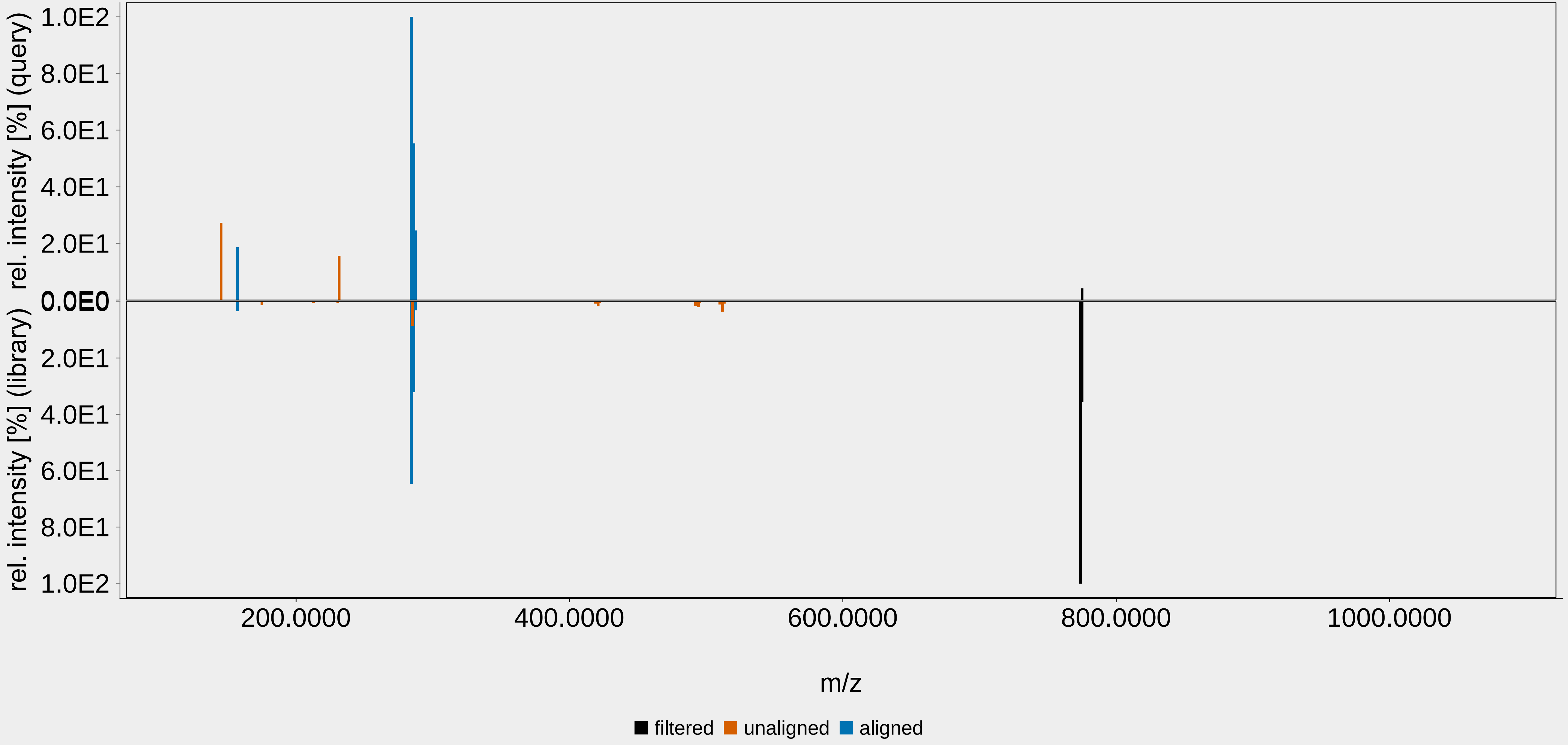

Compound information

Name: PG(18:0_18:1) -

Exact mass: 776.557

Ion type: M-H

Precursor m/z: 775.547

Charge: 1

Polarity: Negative

SMILES:

Instrument information

Instrument type: qTof

Ion source: LC-ESI

Ms level: 2

Acquisition: Lysate

Database links

Other information

Principal investigator: N Auzeil

Data collector: UMR CNRS 8038 Universite de

Entry ID: CCMSLIB00005724243

uridine 5?-diphosphate - 30.0 eV

0.882

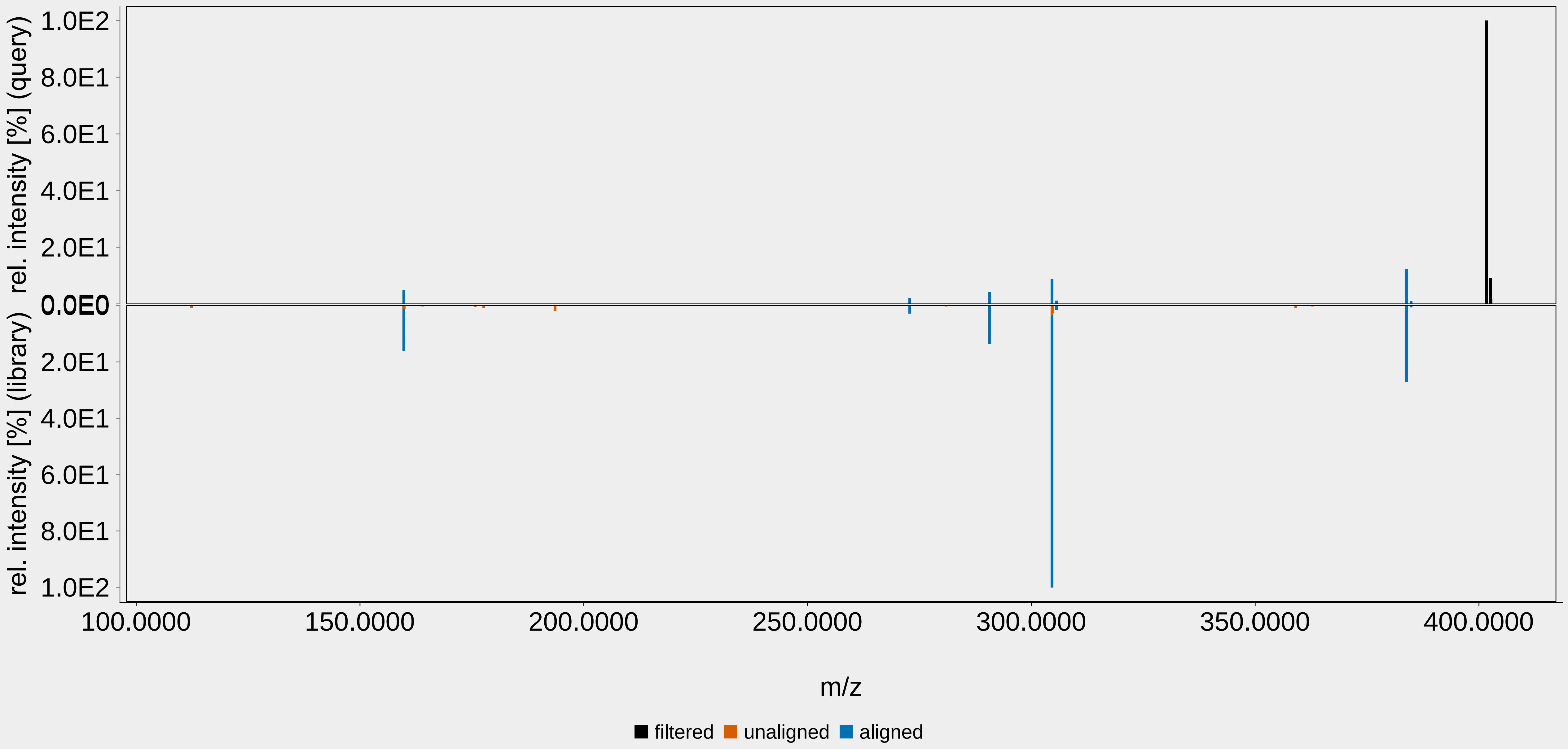

Compound information

Name: uridine 5?-diphosphate - 30.0 eV

Exact mass: 0.0

Ion type: M-H

Precursor m/z: 402.995

Charge: 1

Polarity: Negative

SMILES:

Instrument information

Instrument type: Orbitrap

Ion source: ESI

Ms level: 2

Acquisition: Isolated

Database links

CAS:

Other information

Principal investigator: PI

Data collector: Data Collector

Entry ID: CCMSLIB00006123791

uridine 5?-diphosphate - 30.0 eV

0.899

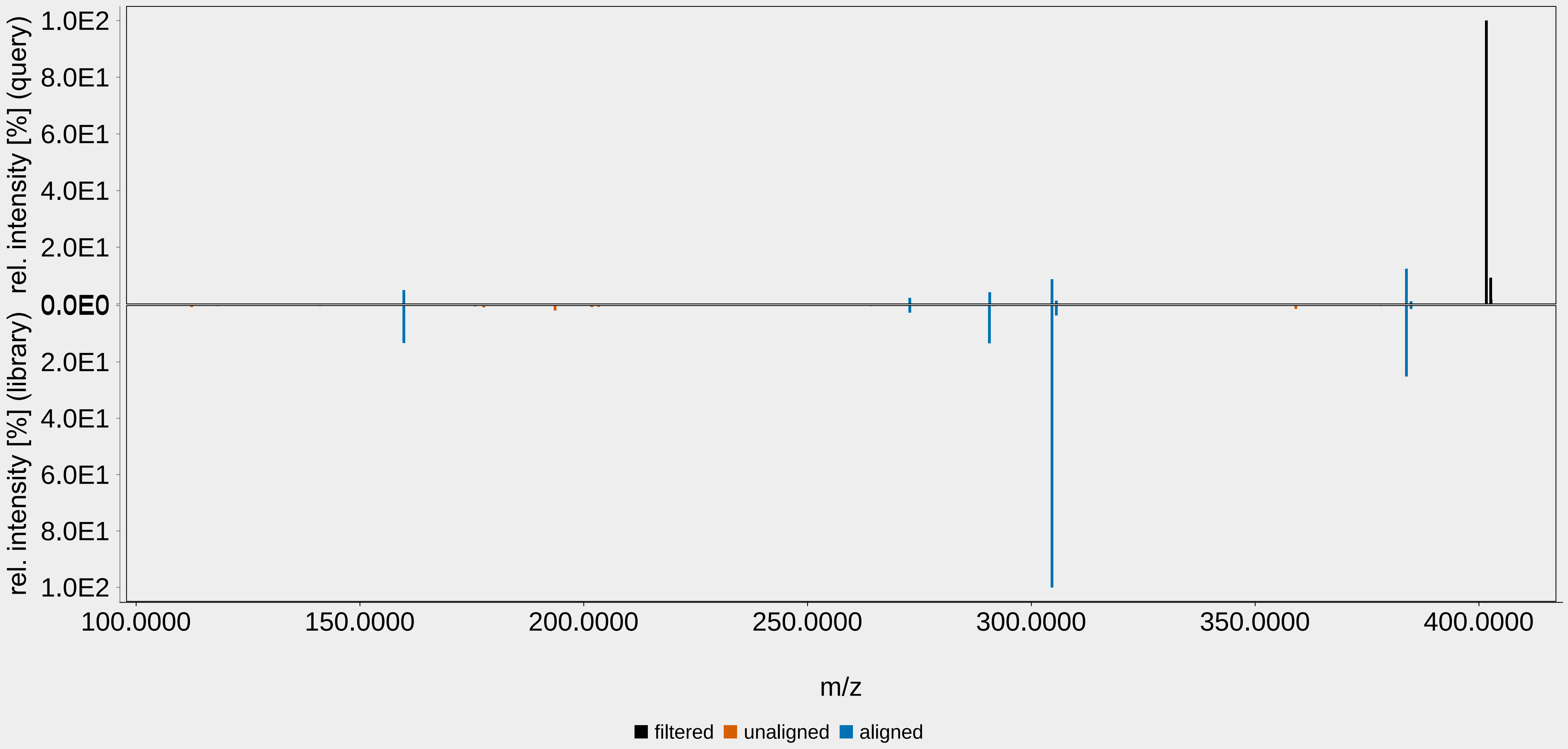

Compound information

Name: uridine 5?-diphosphate - 30.0 eV

Exact mass: 0.0

Ion type: M-H

Precursor m/z: 402.995

Charge: 1

Polarity: Negative

SMILES:

Instrument information

Instrument type: Orbitrap

Ion source: ESI

Ms level: 2

Acquisition: Isolated

Database links

CAS:

Other information

Principal investigator: PI

Data collector: Data Collector

Entry ID: CCMSLIB00006123813

PS(22:6/22:6); [M-H]- C50H73N1O10P1 **0.760**

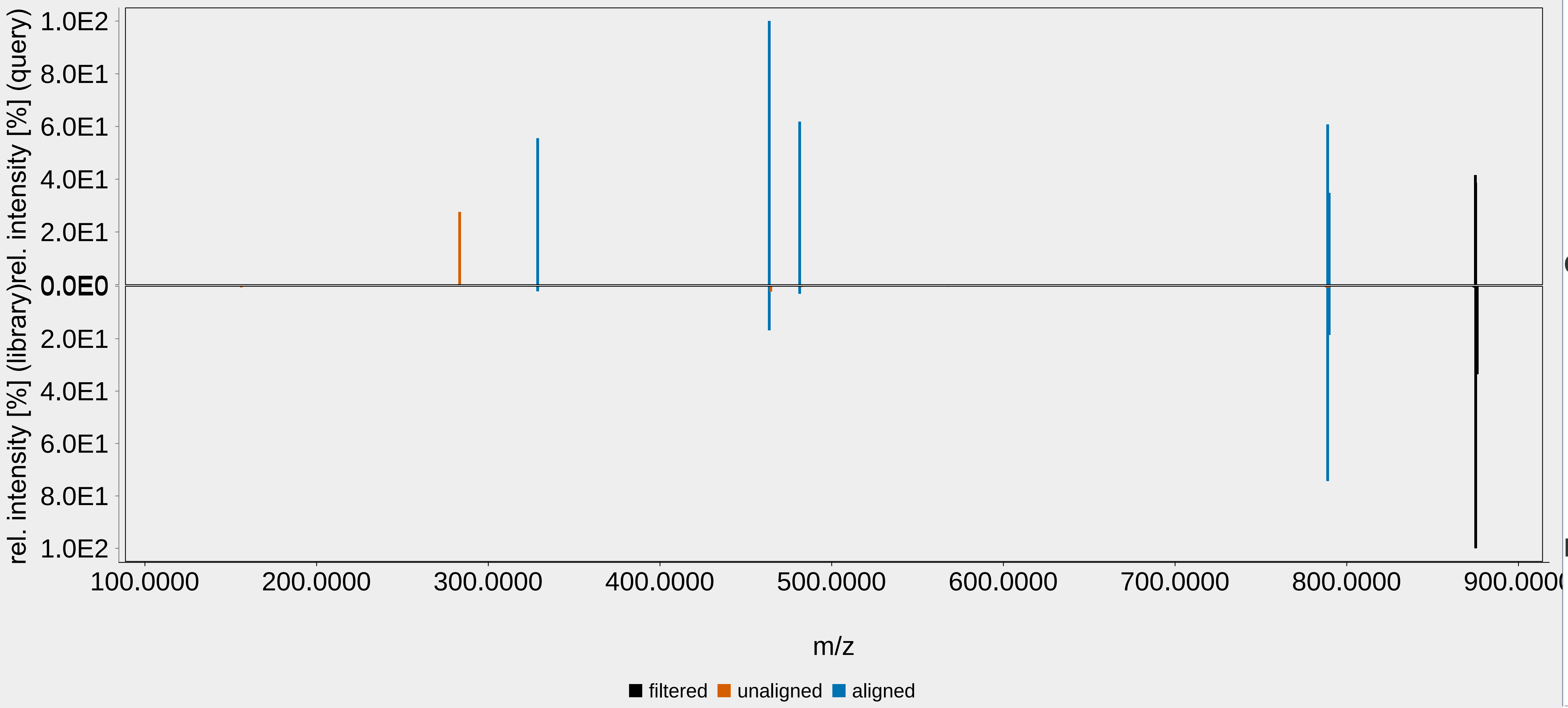

Compound information

Name: PS(22:6/22:6); [M-H]- C50H73N1O10P1

Exact mass: 0.0

Ion type: M-H

Precursor m/z: 878.497

Charge: 1

Polarity: Negative

Instrument information

Instrument type: HCD; Velos

Ion source: LC-ESI

Ms level: 2

Acquisition: Commercial

Database links

Other information

Principal investigator: Thomas Metz

Data collector: Thomas Metz

Entry ID: CCMSLIB00010083009

PS 22:6_22:6 **0.940**

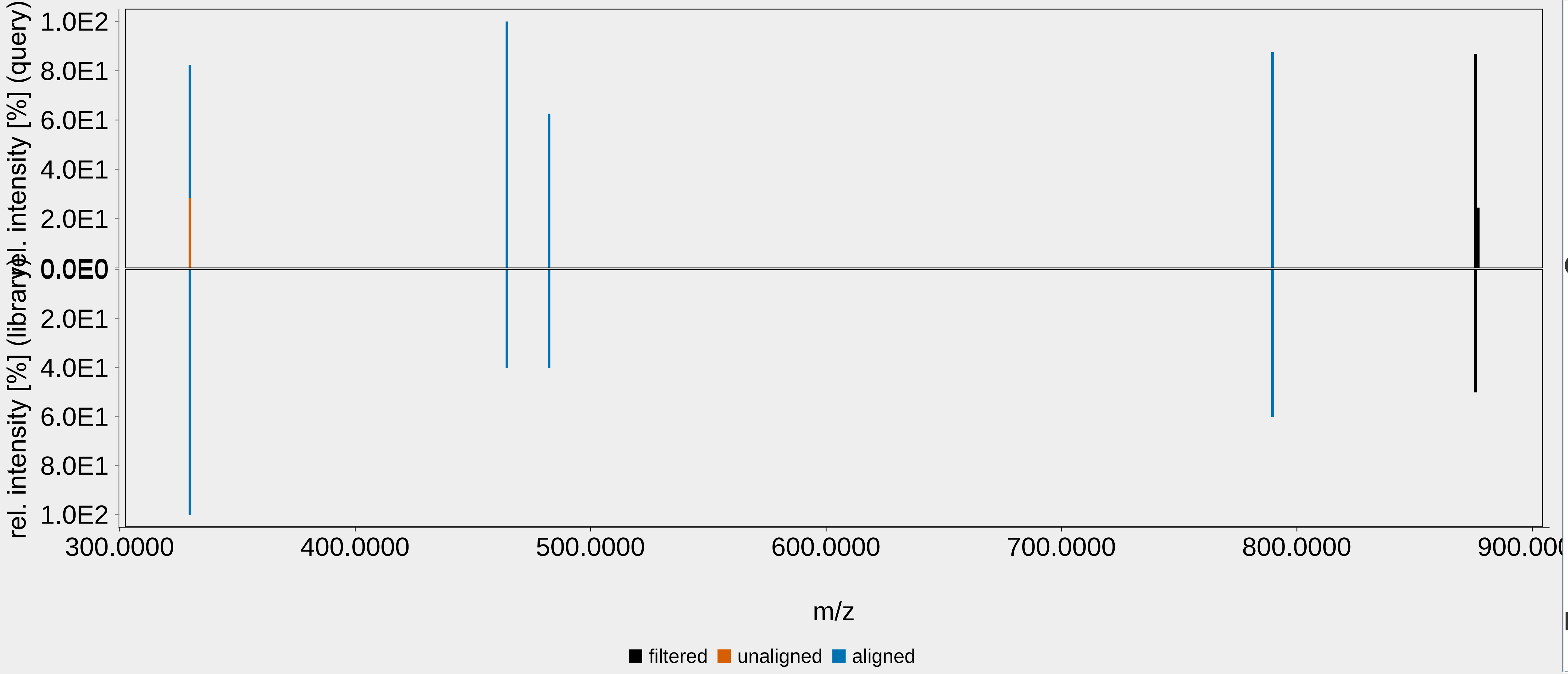

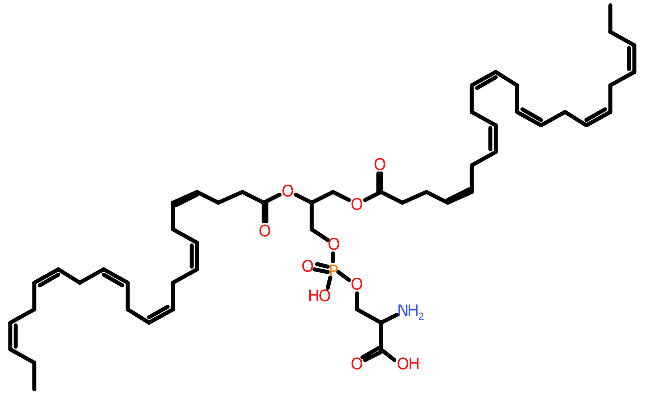

Compound information

Name: PS 22:6_22:6

Formula: C50H74NO10P

Ion type: [M-H]-

Precursor m/z: 878.49776

Ccs: 284.65

InChI key:

SMILES:

Num peaks: 5

Instrument information

Database links

Other information

PS 18:1_22:1 **0.787**

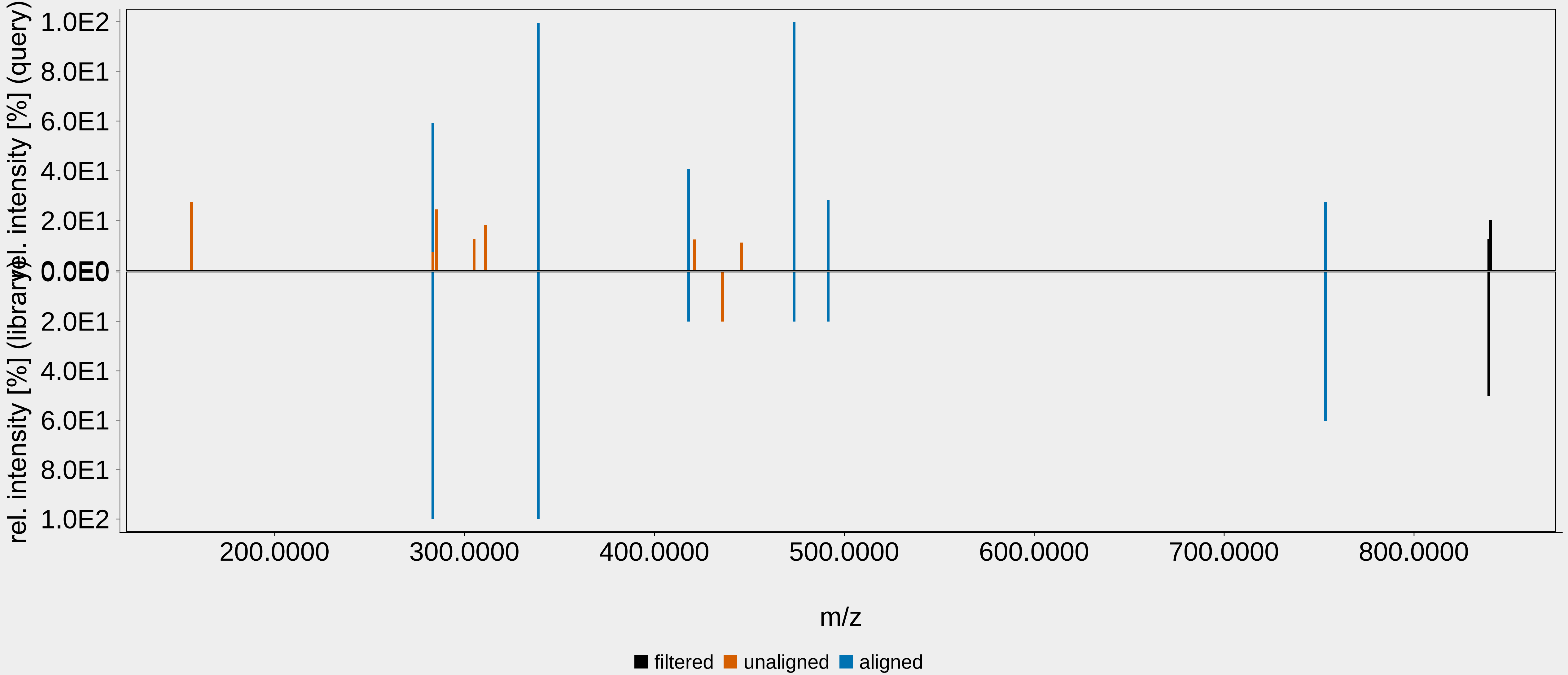

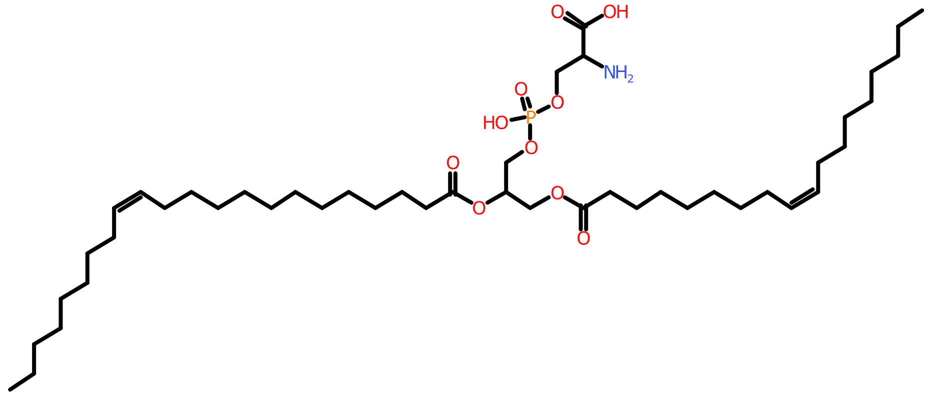

Compound information

Name: PS 18:1_22:1

Formula: C46H86NO10P

Ion type: [M-H]-

Precursor m/z: 842.59166

Ccs: 287.03

InChI key:

SMILES:

Num peaks: 8

Instrument information

Database links

Other information

PA 18:1_20:1

0.720

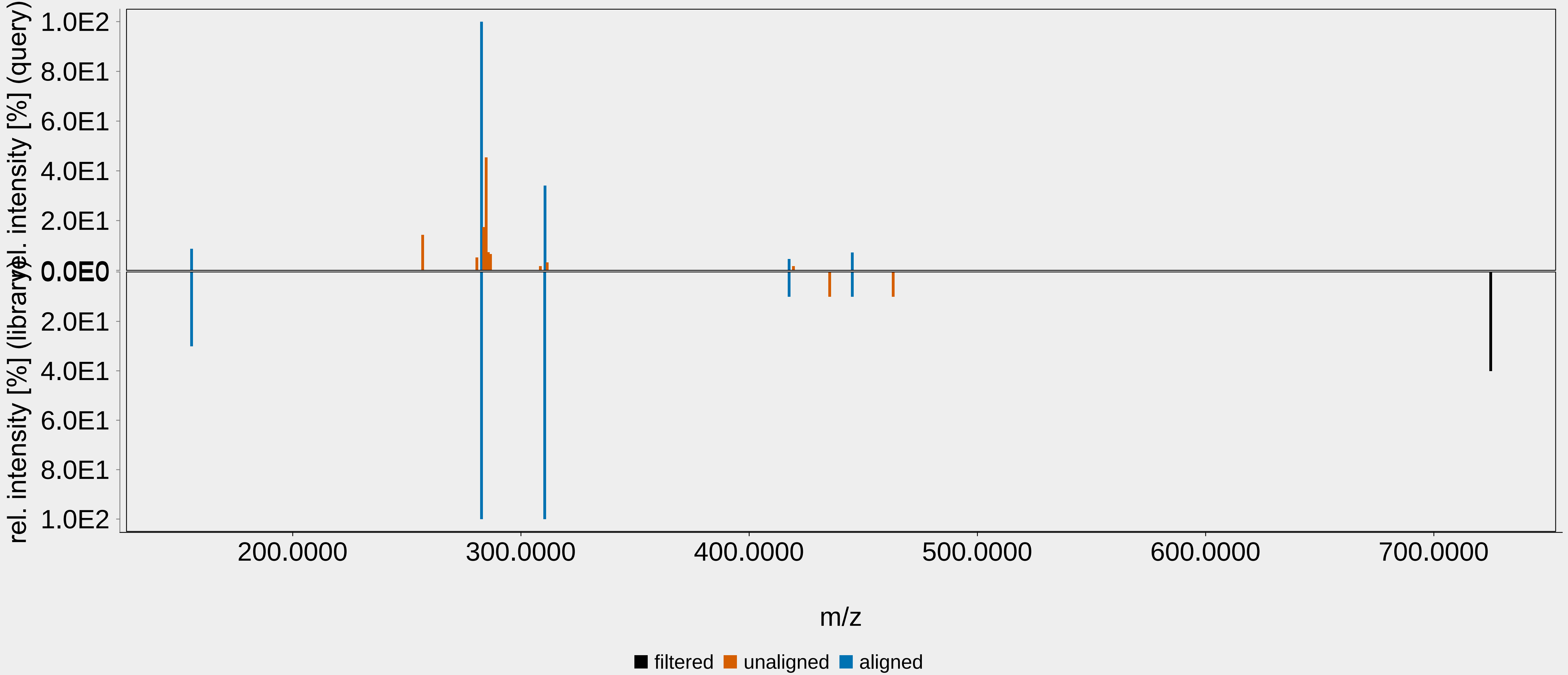

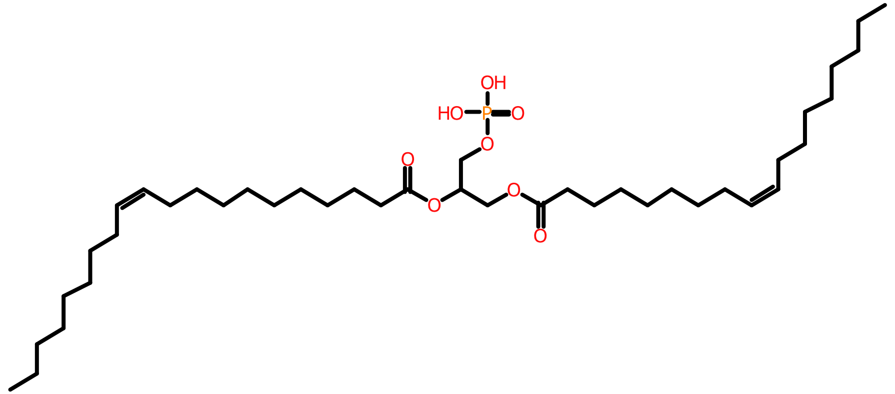

Compound information

Name: PA 18:1_20:1

Formula: C41H77O8P

Ion type: [M-H]-

Precursor m/z: 727.52833

Ccs: 265.22

InChI key:

SMILES:

Num peaks: 8

Instrument information

Database links

Other information

PG(16:1/18:1); [M-H]- C40H74O10P1 **0.779**

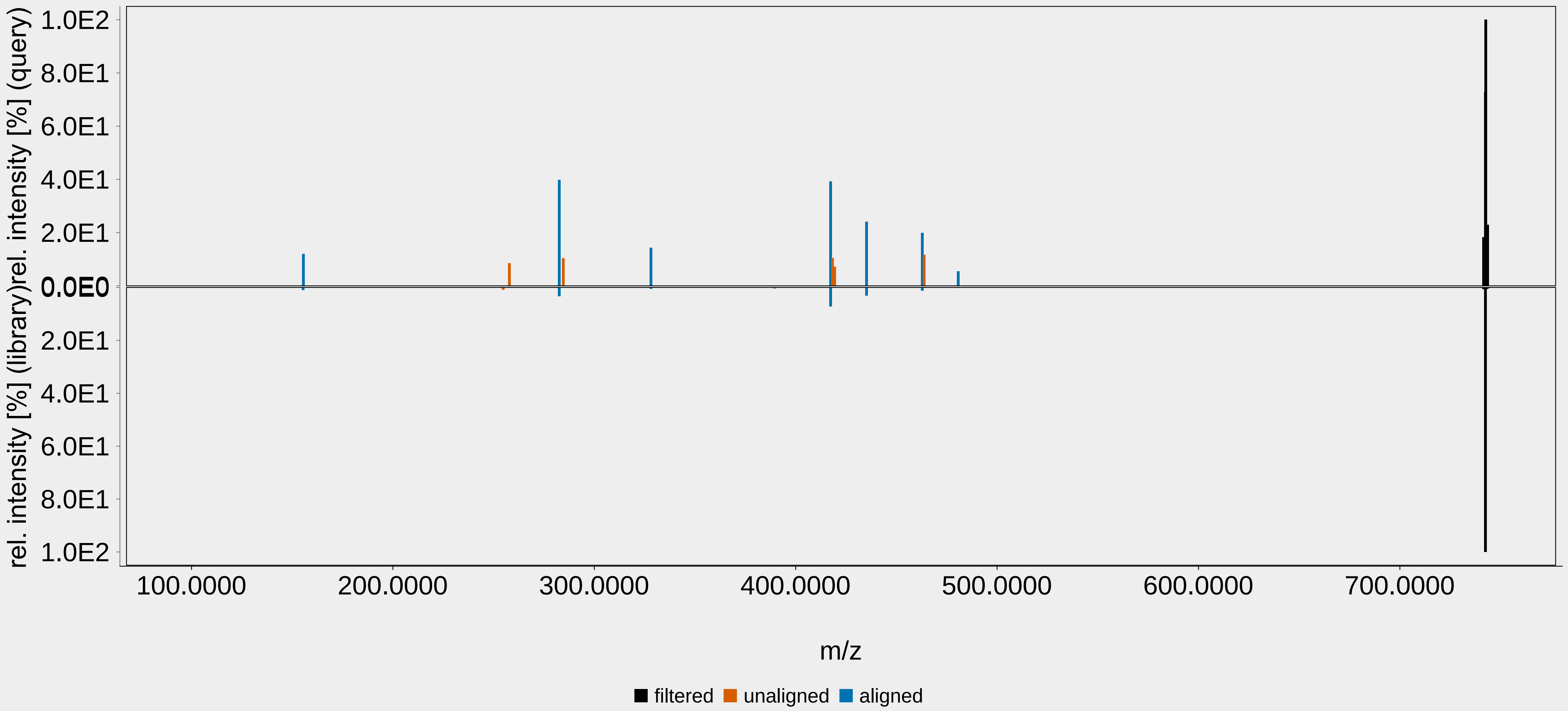

Compound information

Name: PG(16:1/18:1); [M-H]- C40H74O10P1

Exact mass: 0.0

Ion type: M-H

Precursor m/z: 745.502

Charge: 1

Polarity: Negative

Instrument information

Instrument type: HCD; Velos

Ion source: LC-ESI

Ms level: 2

Acquisition: Commercial

Database links

Other information

Principal investigator: Thomas Metz

Data collector: Thomas Metz

Entry ID: CCMSLIB00010087352

PG(16:1/18:1); [M-H]- C40H74O10P1 **0.782**

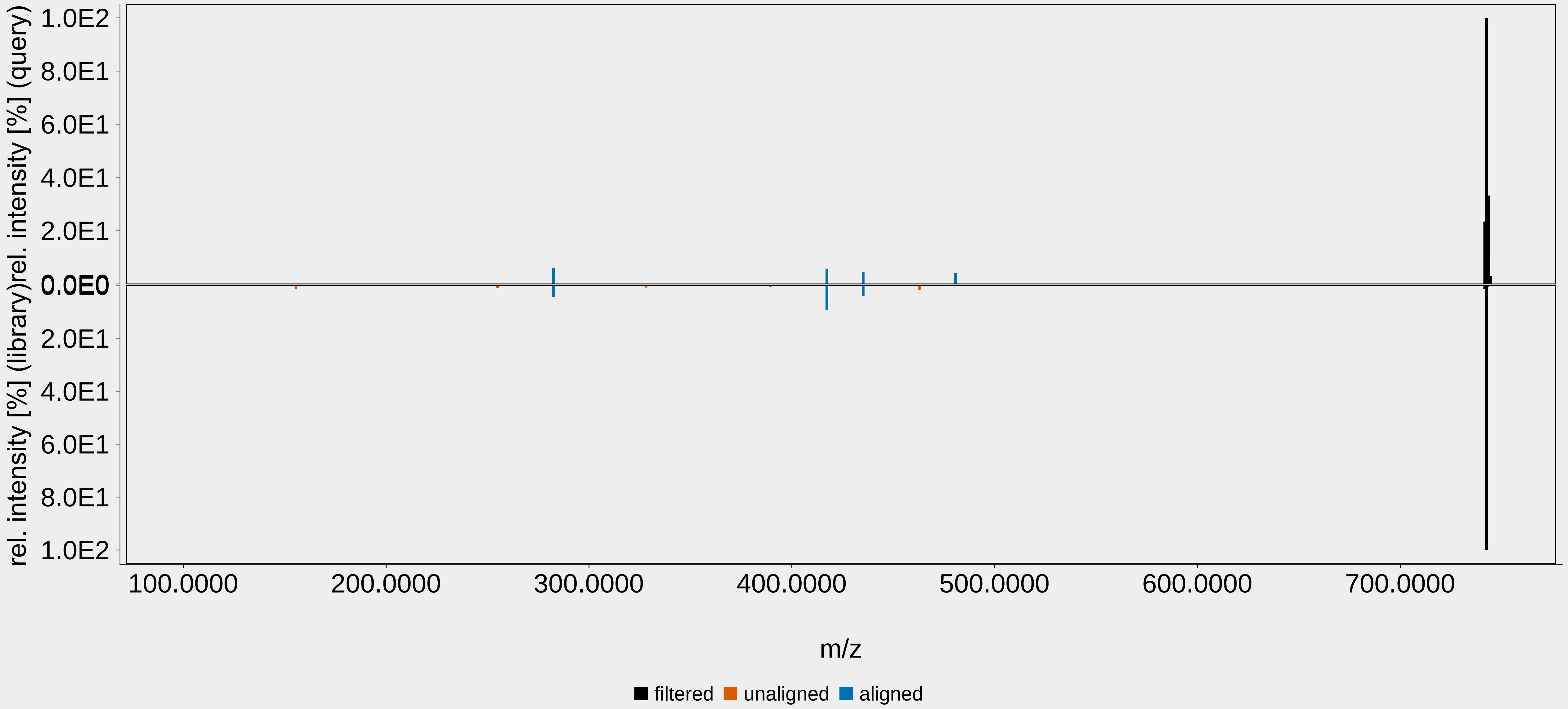

Compound information

Name: PG(16:1/18:1); [M-H]- C40H74O10P1

Exact mass: 0.0

Ion type: M-H

Precursor m/z: 745.502

Charge: 1

Polarity: Negative

Instrument information

Instrument type: HCD; Velos

Ion source: LC-ESI

Ms level: 2

Acquisition: Commercial

Database links

Other information

Principal investigator: Thomas Metz

Data collector: Thomas Metz

Entry ID: CCMSLIB00010073906

PS 40:4 **0.883**

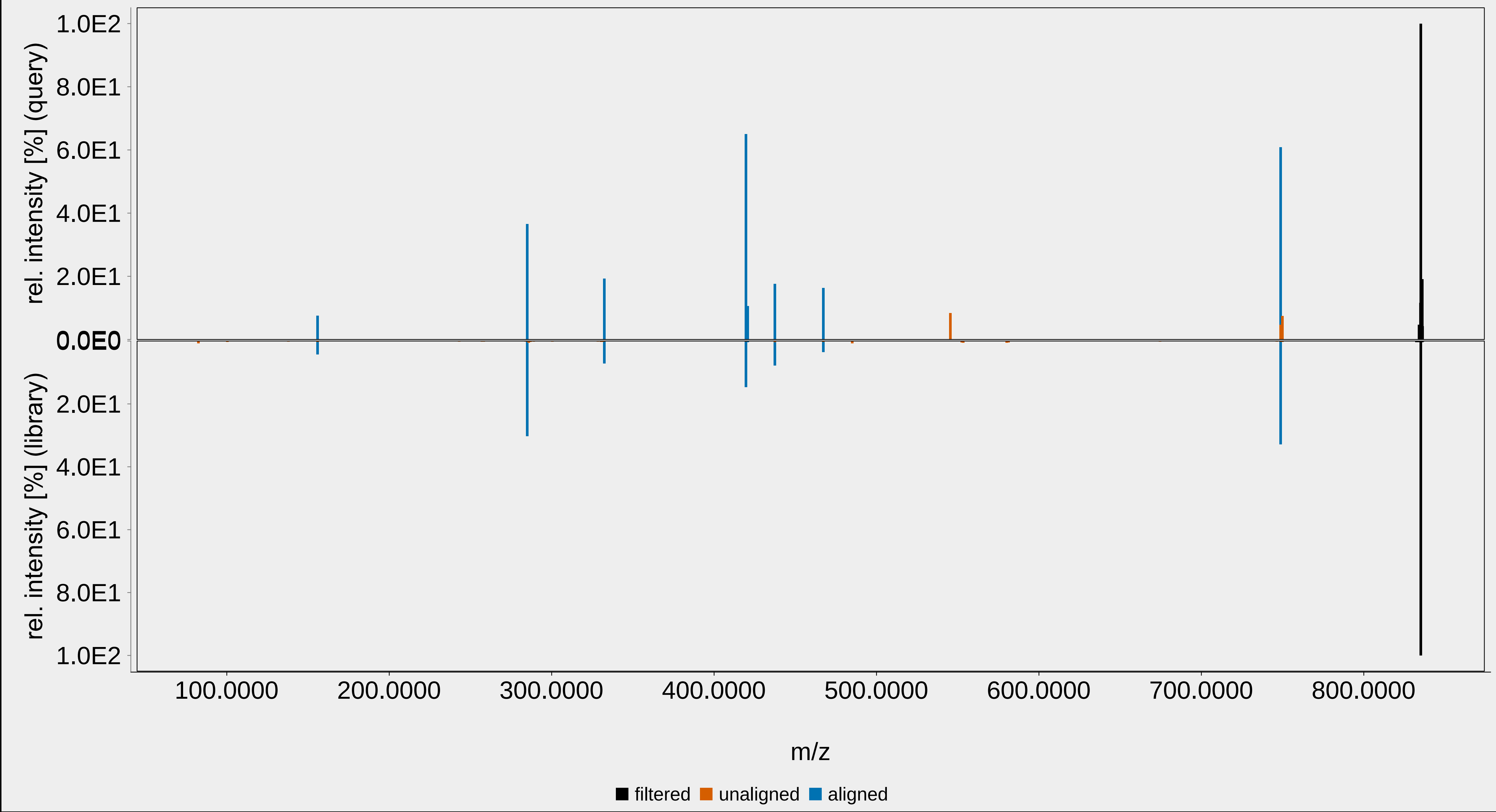

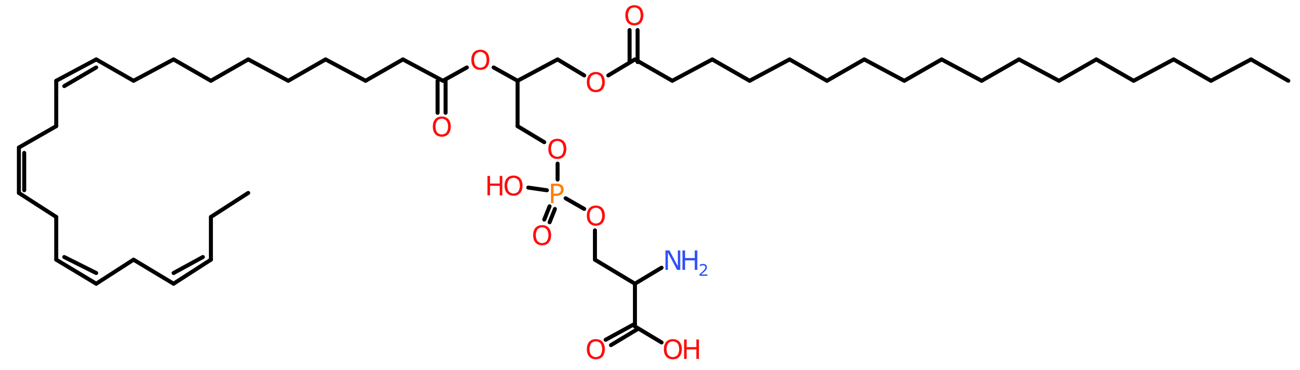

Compound information

Name: PS 40:4

Formula: C46H82NO10P

Mol. weight: 840.135

Exact mass: 840.135

Ion type: [M-H]-

Precursor m/z: 838.5547

RT: 9.189651

Polarity: negative

InChI:

InChI key:

SMILES:

Instrument information

Instrument type: LC-ESI-QTOF

Instrument: AB Sciex TripleTOF 5600+ system

Ms level: MS2

Collision energy: 45V

Database links

Pubchem: 138202734

MoNA ID: LQB00724

Other information

Principal investigator: Kazutaka Ikeda, Hiroshi

Data collector: Kazutaka Ikeda, Hiroshi

PS 40:4

0.883

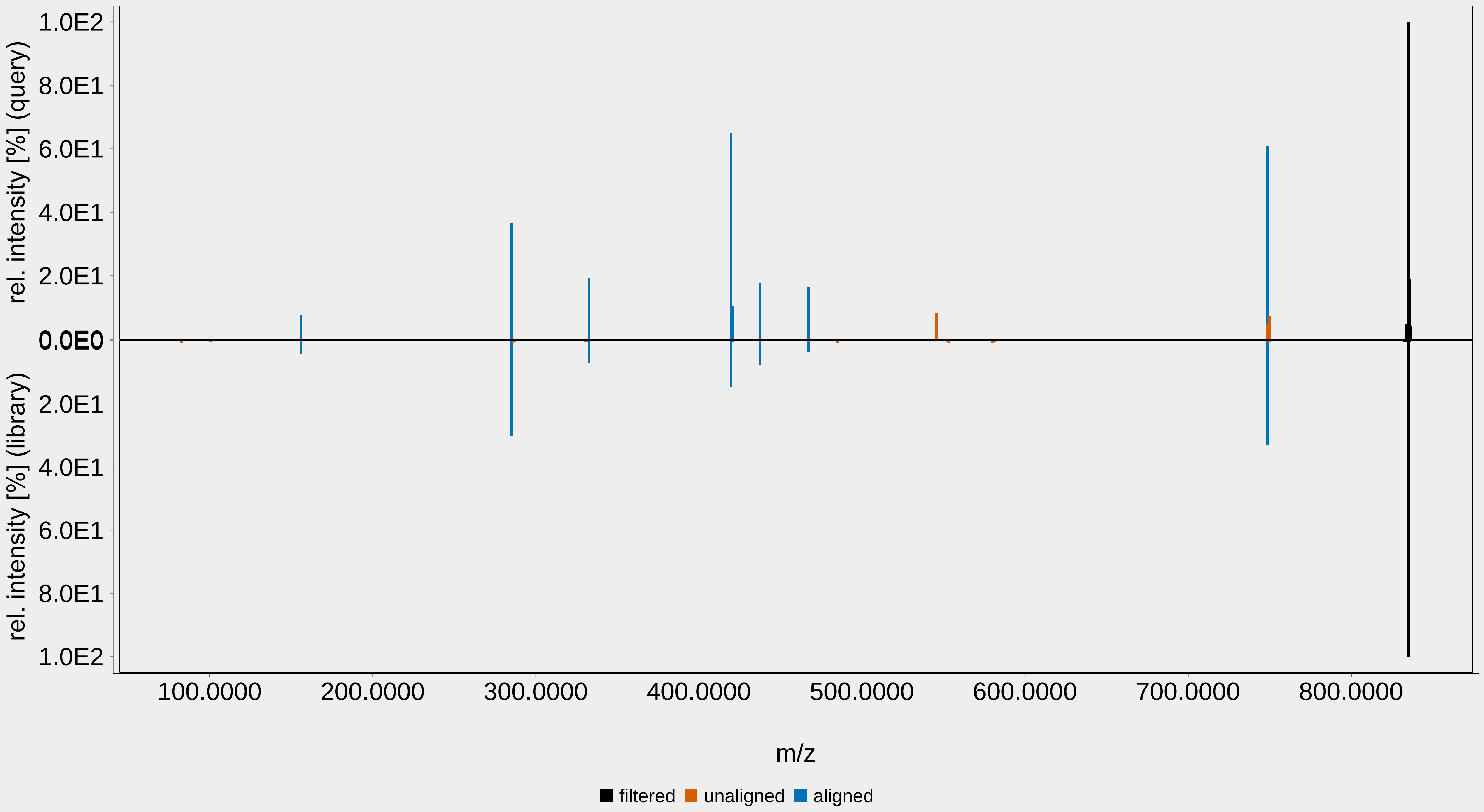

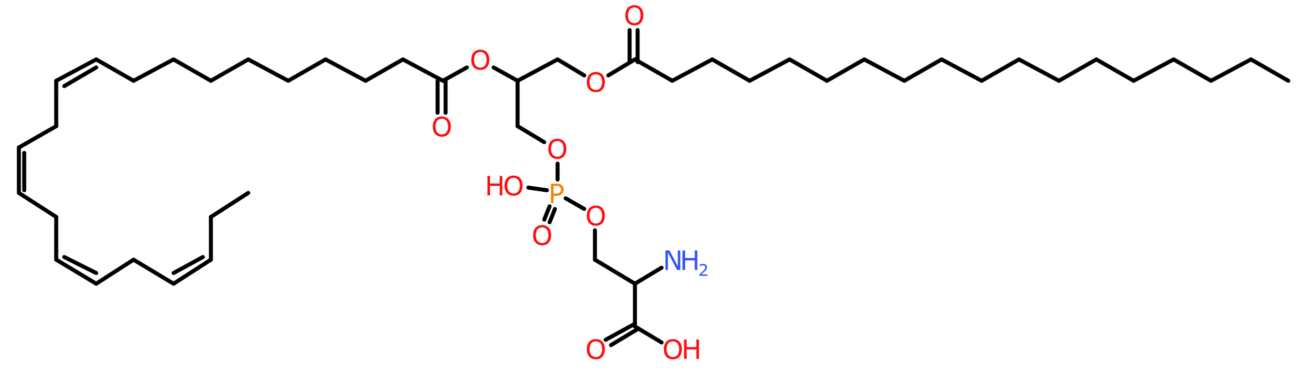

Compound information

Name: PS 40:4

Formula: C46H82NO10P

Mol. weight: 840.135

Exact mass: 840.135

Ion type: [M-H]-

Precursor m/z: 838.5547

RT: 9.189651

Polarity: negative

InChI:

InChI key:

SMILES:

Instrument information

Instrument type: LC-ESI-QTOF

Instrument: AB Sciex TripleTOF 5600+ system

Ms level: MS2

Collision energy: 45V

Database links

Pubchem: 138202734

MoNA ID: LQB00338

Other information

Principal investigator: Kazutaka Ikeda, Hiroshi

Data collector: Kazutaka Ikeda, Hiroshi

PHYTIC ACID

0.904

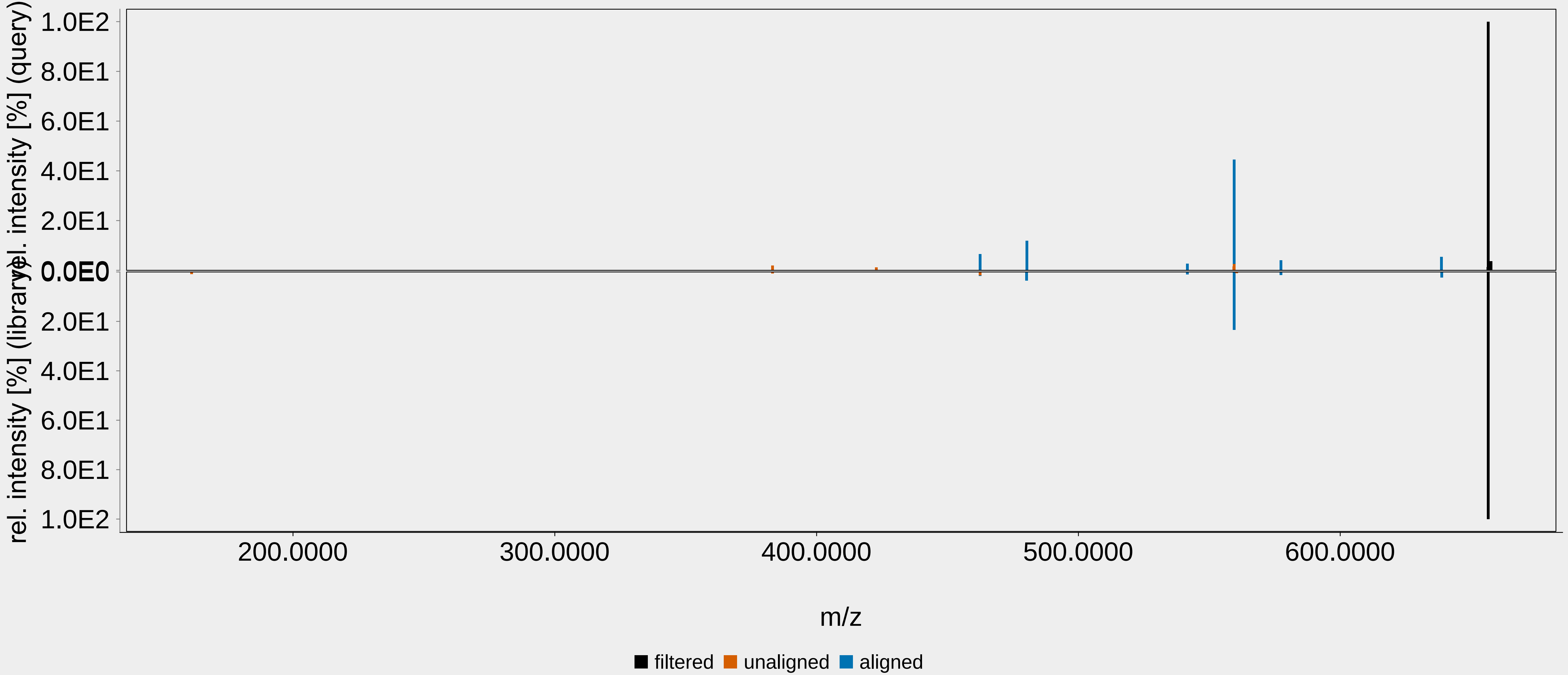

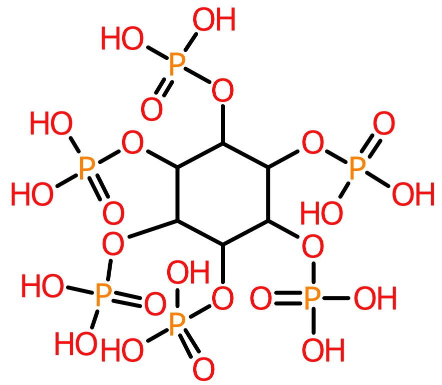

Compound information

Name: PHYTIC ACID

Formula: C6H18O24P6

Ion type: [M-H]-

Precursor m/z: 658.8540952

Polarity: negative

InChI:

InChI key: IMQLKJBTEOYOSI-UHFFFAOYSA-I

SMILES:

Instrument information

Instrument type: LC-ESI-QTOF

Instrument: Agilent qTOF 6545

Ms level: MS2

Collision energy: 20 eV

Database links

Other information

PHYTIC ACID

0.904

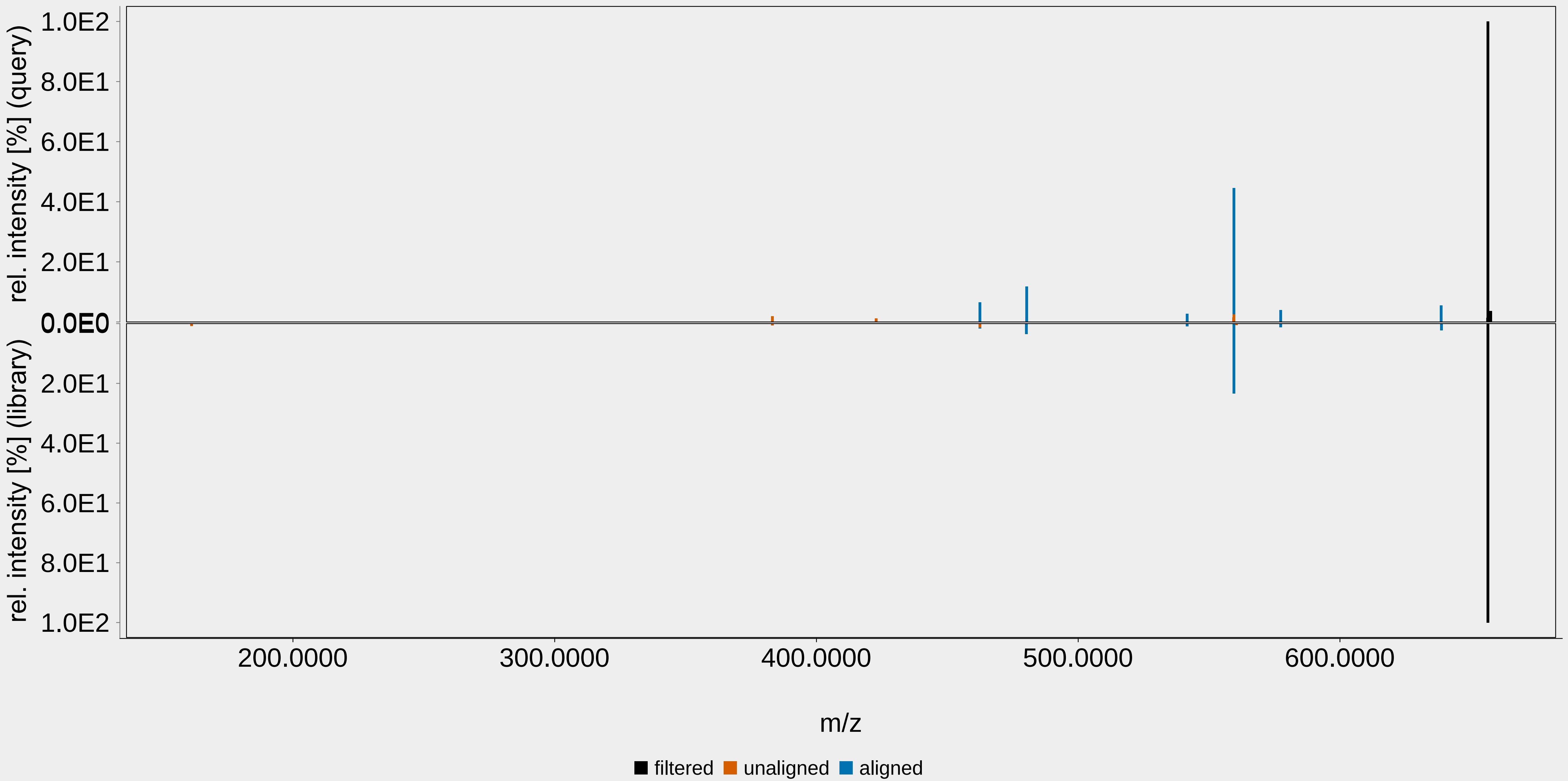

Compound information

Name: PHYTIC ACID

Exact mass: 0.0

Ion type: M-H

Precursor m/z: 658.854

Charge: 1

Polarity: negative

InChI:

SMILES:

Instrument information

Instrument type: qTof

Ion source: ESI

Ms level: 2

Acquisition: isolated

Database links

Other information

Principal investigator: MoNA

Data collector: MoNA:MoNA034158

Entry ID: CCMSLIB00006685256

PA 18:2_22:6

0.859

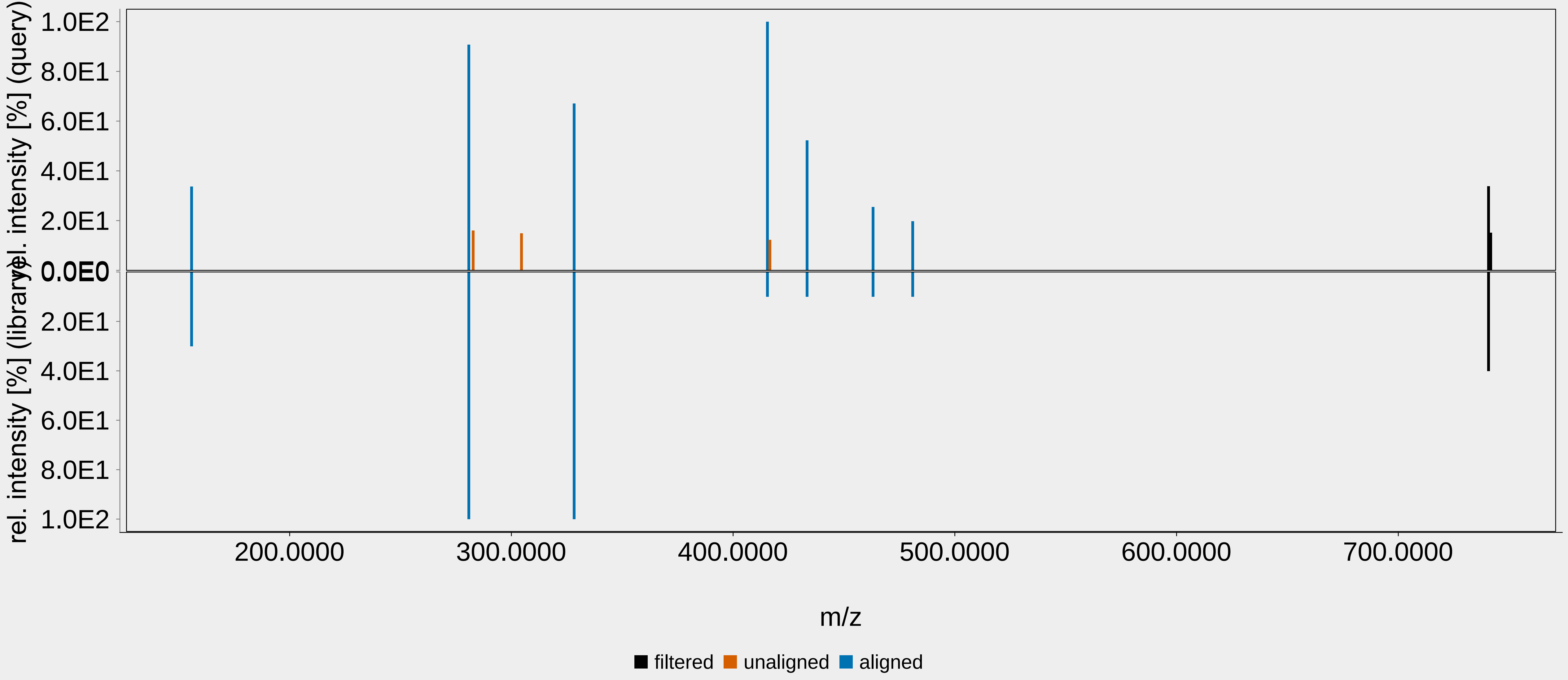

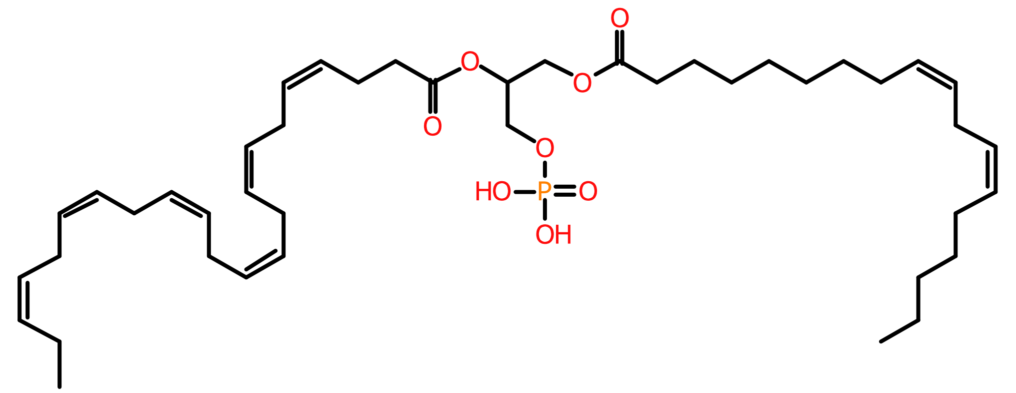

Compound information

Name: PA 18:2_22:6

Formula: C43H69O8P

Ion type: [M-H]-

Precursor m/z: 743.46573

Ccs: 264.57

InChI key:

SMILES:

Num peaks: 8

Instrument information

Database links

Other information

PA 18:1_22:6

0.796

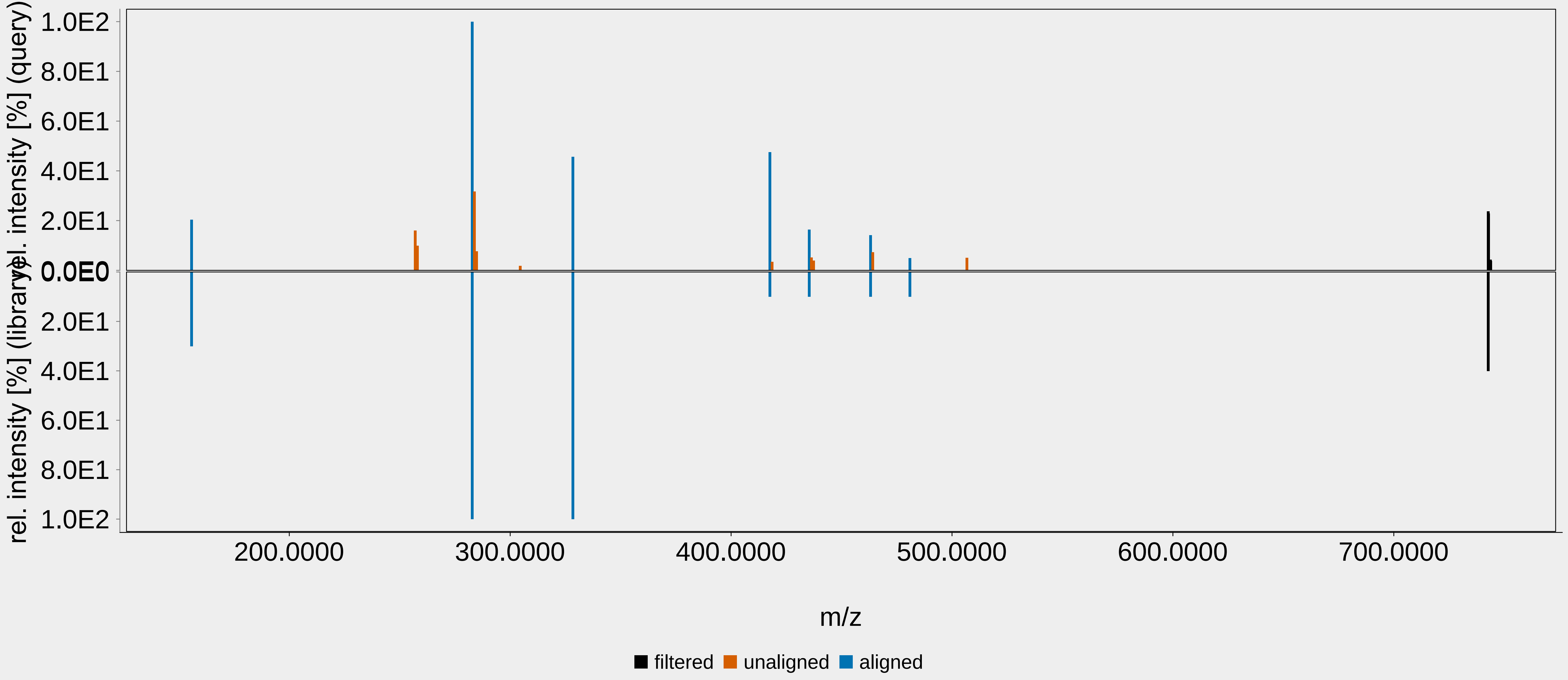

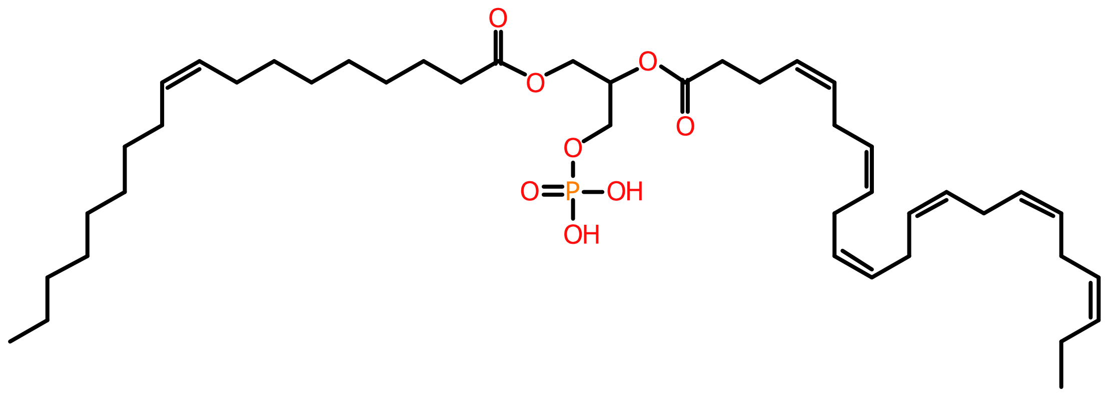

Compound information

Name: PA 18:1_22:6

Formula: C43H71O8P

Ion type: [M-H]-

Precursor m/z: 745.48138

Ccs: 264.25

InChI key:

SMILES:

Num peaks: 8

Instrument information

Database links

Other information

PE(18:0/22:6); [M-H]- C45H77N1O8P1

0.878

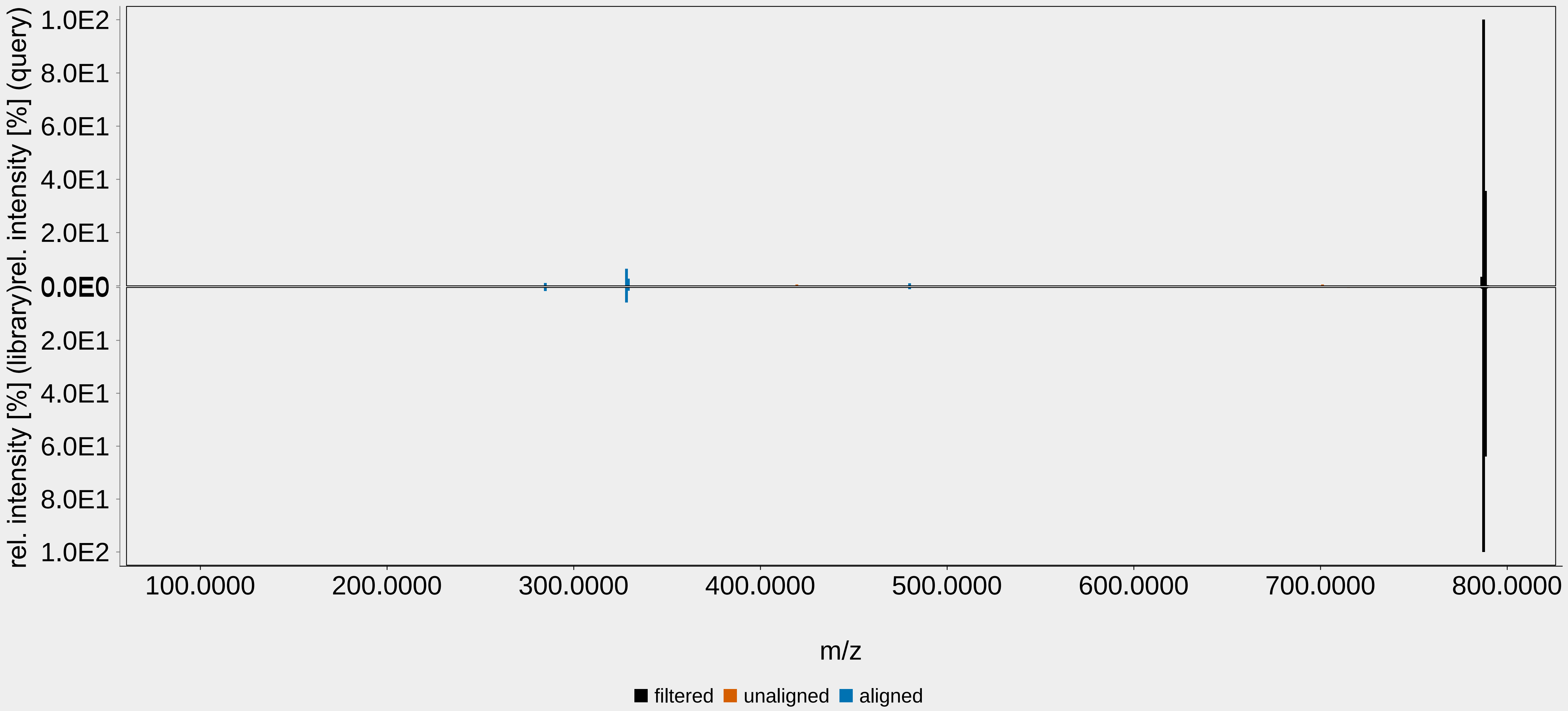

Compound information

Name: PE(18:0/22:6); [M-H]- C45H77N1O8P1

Exact mass: 0.0

Ion type: M-H

Precursor m/z: 790.539

Charge: 1

Polarity: Negative

Instrument information

Instrument type: HCD; Velos

Ion source: LC-ESI

Ms level: 2

Acquisition: Commercial

Database links

Other information

Principal investigator: Thomas Metz

Data collector: Thomas Metz

Entry ID: CCMSLIB00010085809

PE(18:0/22:6); [M-H]- C45H77N1O8P1 **0.880**

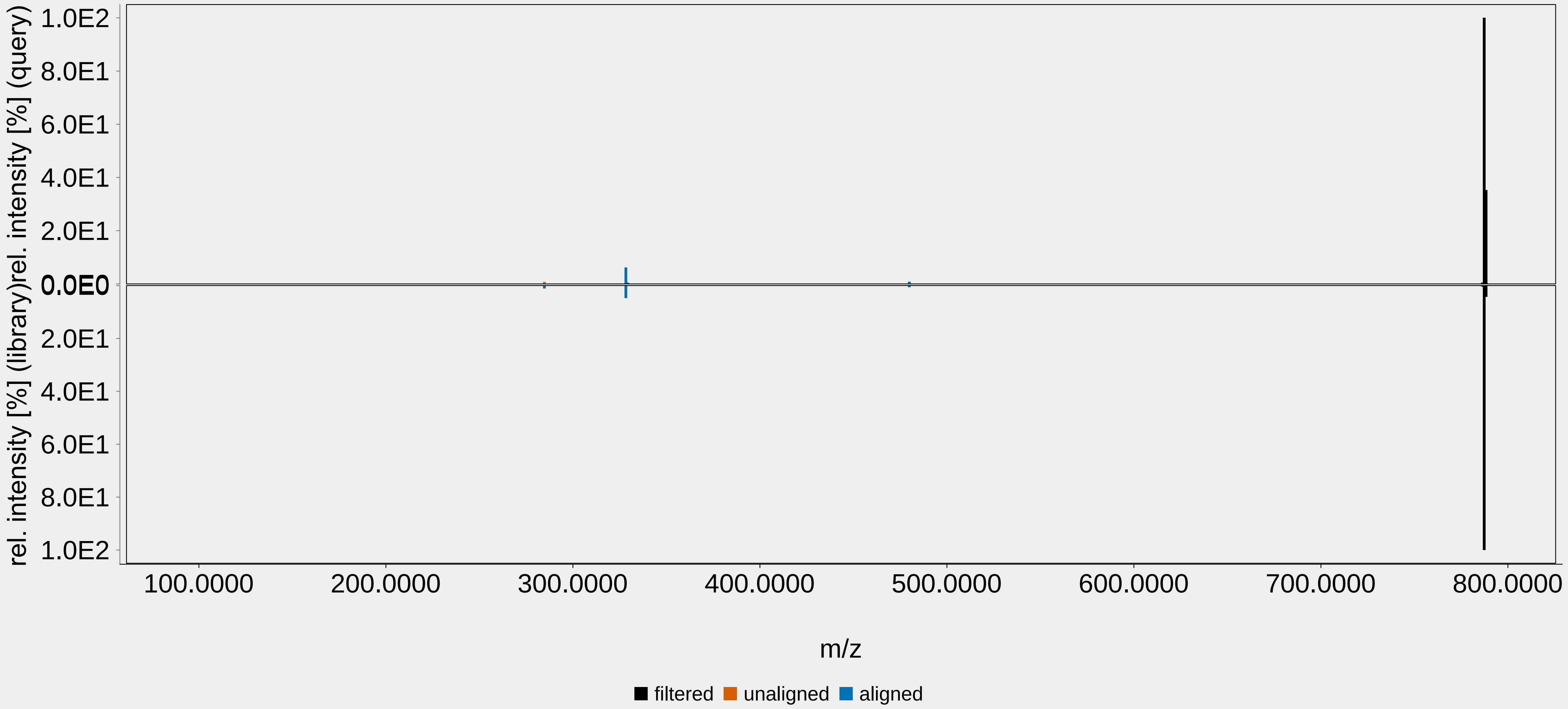

Compound information

Name: PE(18:0/22:6); [M-H]- C45H77N1O8P1

Exact mass: 0.0

Ion type: M-H

Precursor m/z: 790.539

Charge: 1

Polarity: Negative

Instrument information

Instrument type: HCD; Velos

Ion source: LC-ESI

Ms level: 2

Acquisition: Commercial

Database links

Other information

Principal investigator: Thomas Metz

Data collector: Thomas Metz

Entry ID: CCMSLIB00010073326

NADH - 30.0 eV

0.936

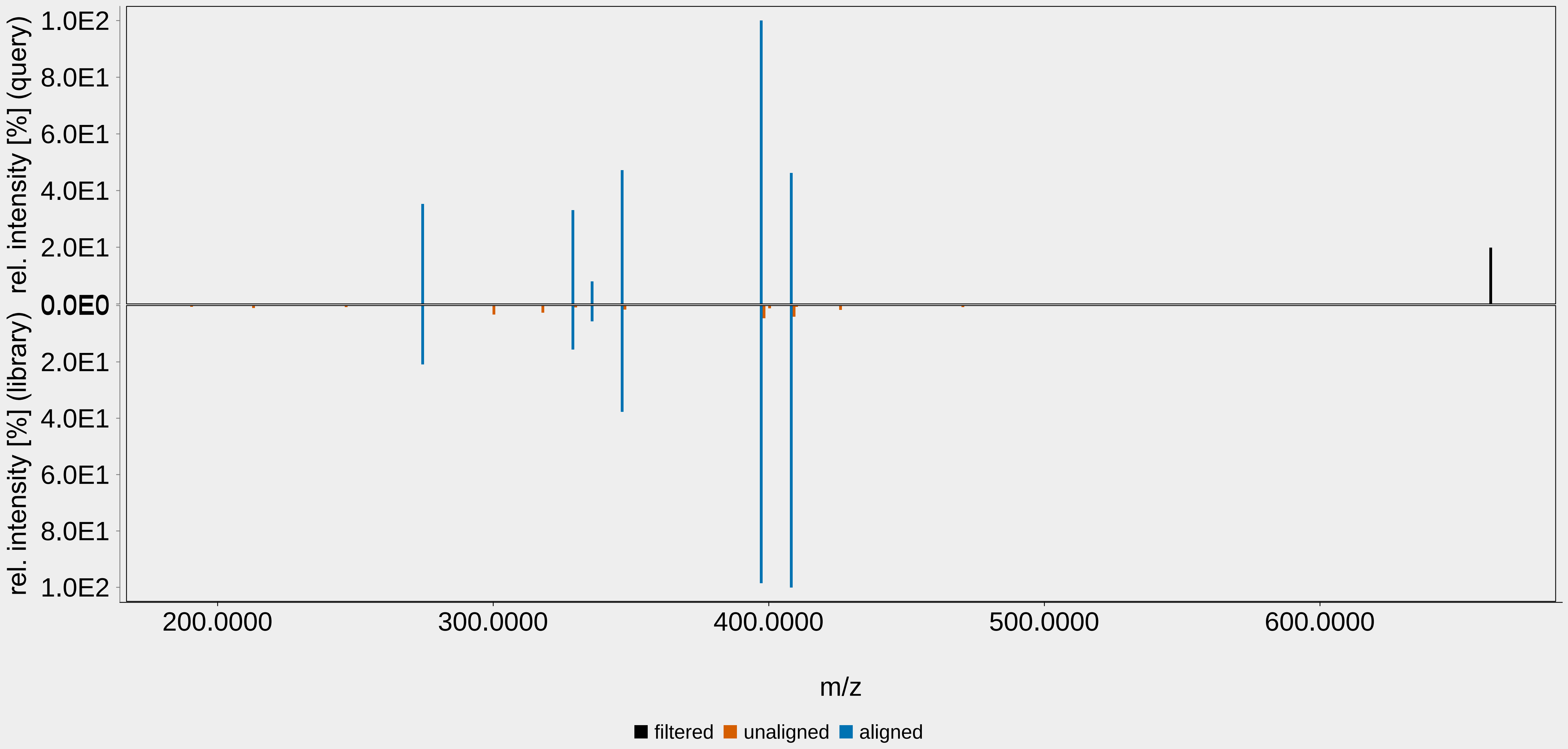

Compound information

Name: NADH - 30.0 eV

Exact mass: 0.0

Ion type: M-H

Precursor m/z: 664.118

Charge: 1

Polarity: Negative

SMILES:

Instrument information

Instrument type: Orbitrap

Ion source: ESI

Ms level: 2

Acquisition: Isolated

Database links

CAS:

Other information

Principal investigator: PI

Data collector: Data Collector

Entry ID: CCMSLIB00006123962

NADH - 30.0 eV

0.944

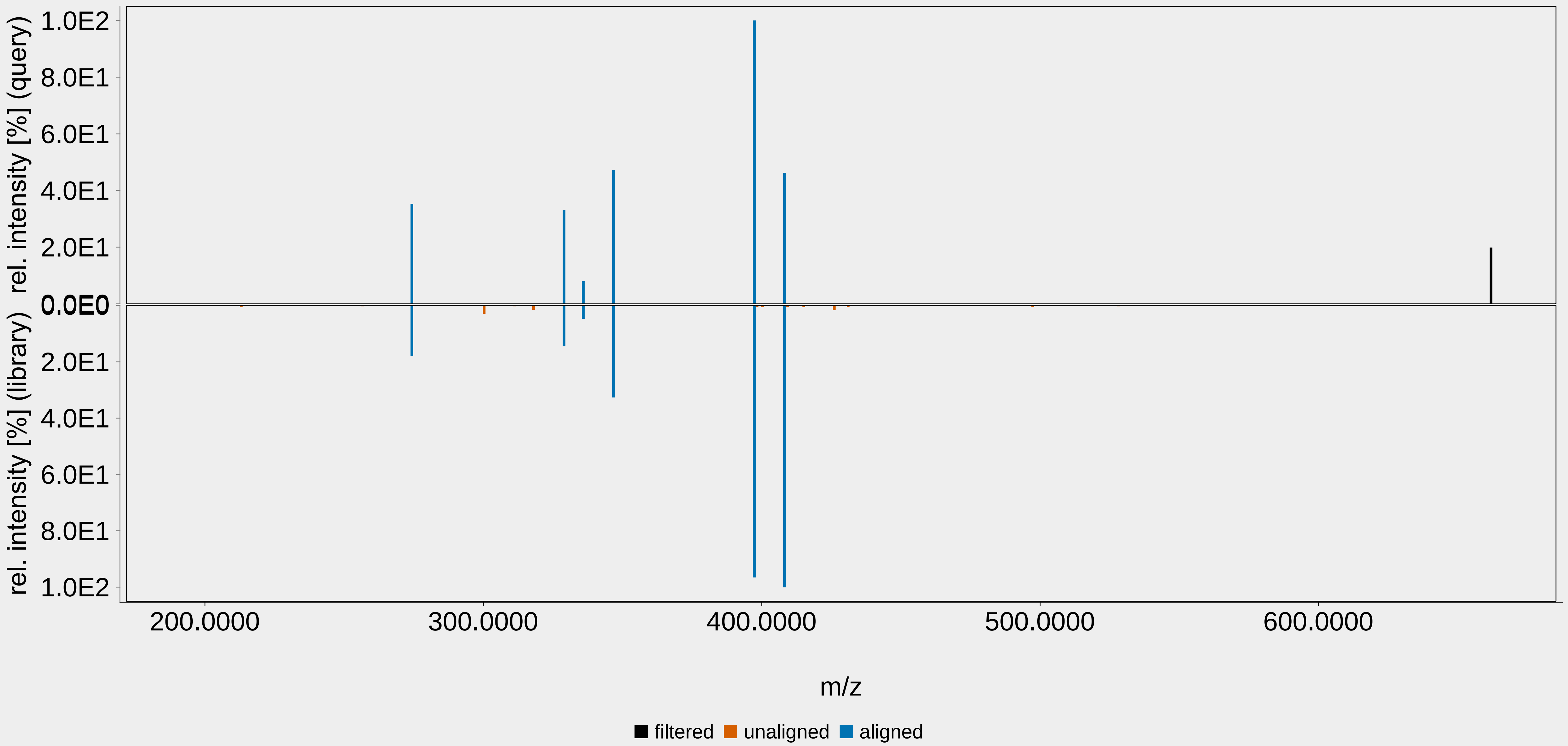

Compound information

Name: NADH - 30.0 eV

Exact mass: 0.0

Ion type: M-H

Precursor m/z: 664.117

Charge: 1

Polarity: Negative

SMILES:

Instrument information

Instrument type: Orbitrap

Ion source: ESI

Ms level: 2

Acquisition: Isolated

Database links

CAS:

Other information

Principal investigator: PI

Data collector: Data Collector

Entry ID: CCMSLIB00006123977

PI 40:6 **0.845**

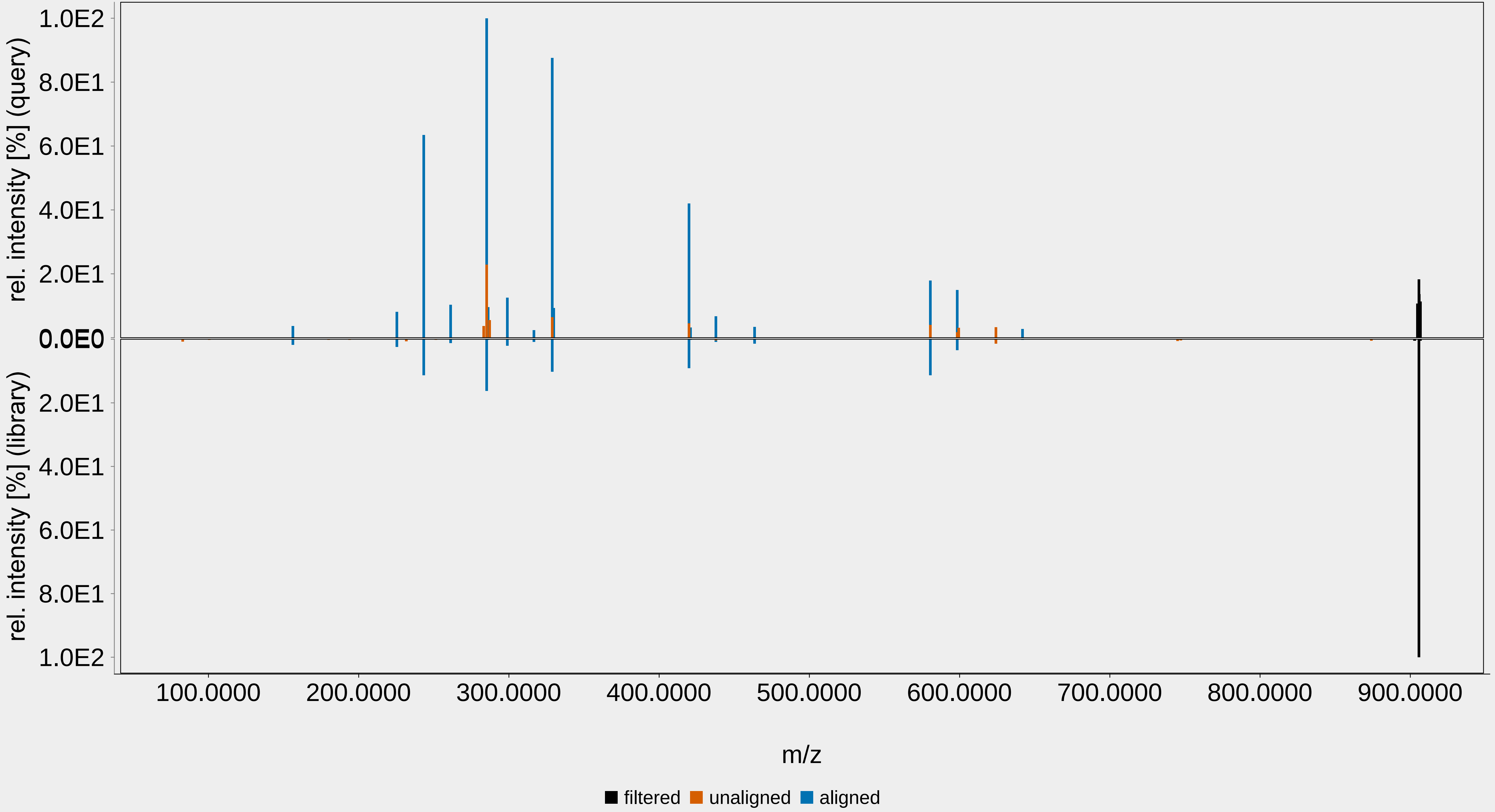

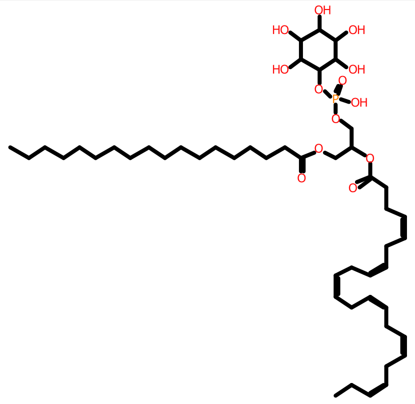

Compound information

Name: PI 40:6

Formula: C49H83O13P

Mol. weight: 911.1659999999999

Exact mass: 911.1659999999999

Ion type: [M-H]-

Precursor m/z: 909.5445

RT: 8.653857

Polarity: negative

InChI:

InChI key:

SMILES:

Instrument information

Instrument type: LC-ESI-QTOF

Instrument: AB Sciex TripleTOF 5600+ system

Ms level: MS2

Collision energy: 45V

Database links

Pubchem: 138144195

MoNA ID: LQB00699

Other information

Principal investigator: Kazutaka Ikeda, Hiroshi

Data collector: Kazutaka Ikeda, Hiroshi

PI 40:6

0.845

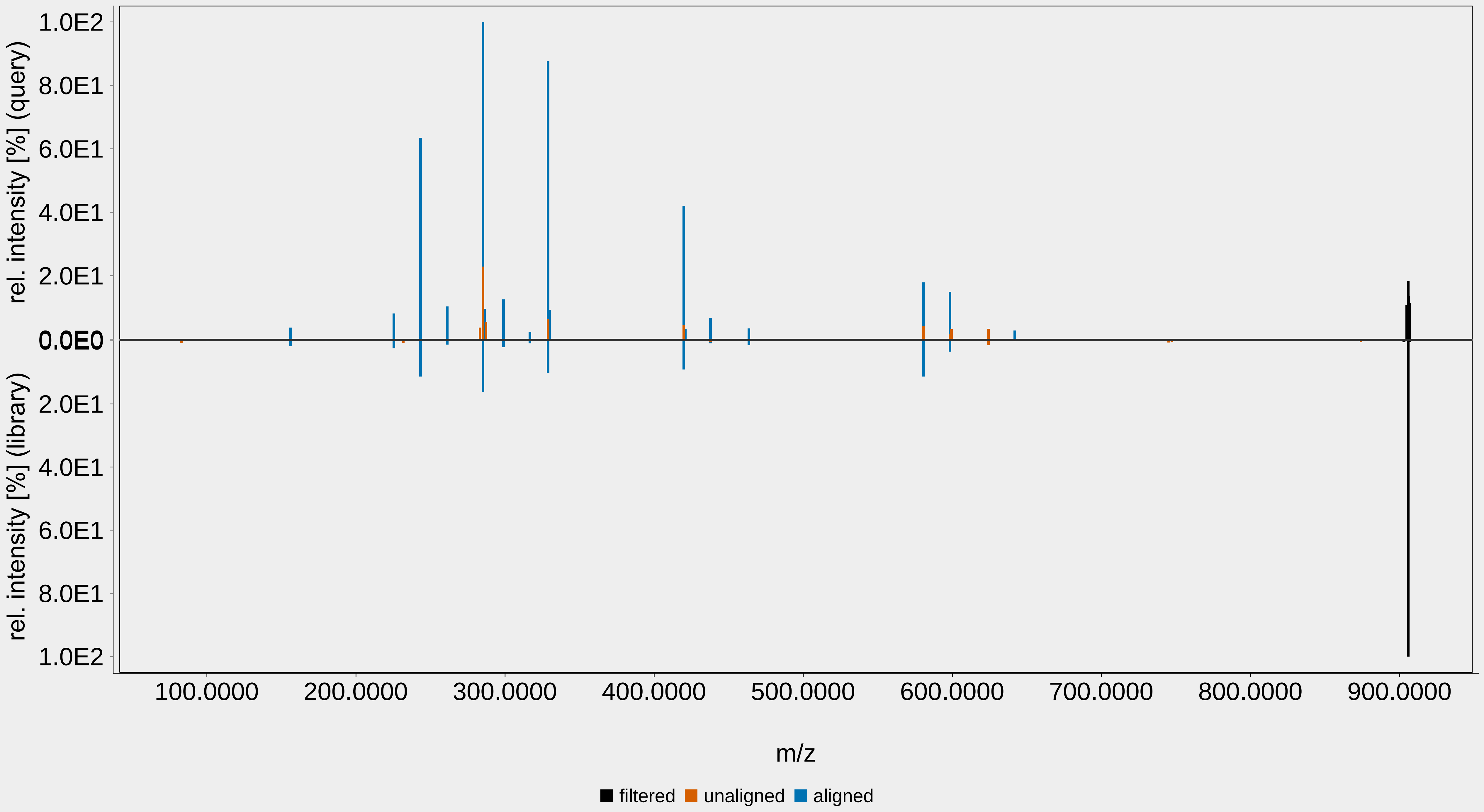

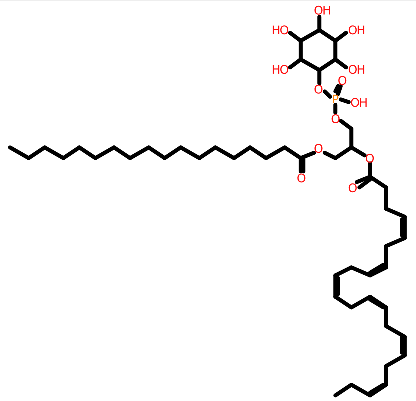

Compound information

Name: PI 40:6

Formula: C49H83O13P

Mol. weight: 911.1659999999999

Exact mass: 911.1659999999999

Ion type: [M-H]-

Precursor m/z: 909.5445

RT: 8.653857

Polarity: negative

InChI:

InChI key:

SMILES:

Instrument information

Instrument type: LC-ESI-QTOF

Instrument: AB Sciex TripleTOF 5600+ system

Ms level: MS2

Collision energy: 45V

Database links

Pubchem: 138144195

MoNA ID: LQB00313

Other information

Principal investigator: Kazutaka Ikeda, Hiroshi

Data collector: Kazutaka Ikeda, Hiroshi

PE(P-18:0/22:6); [M-H]- C45H77N1O7P1 **0.852**

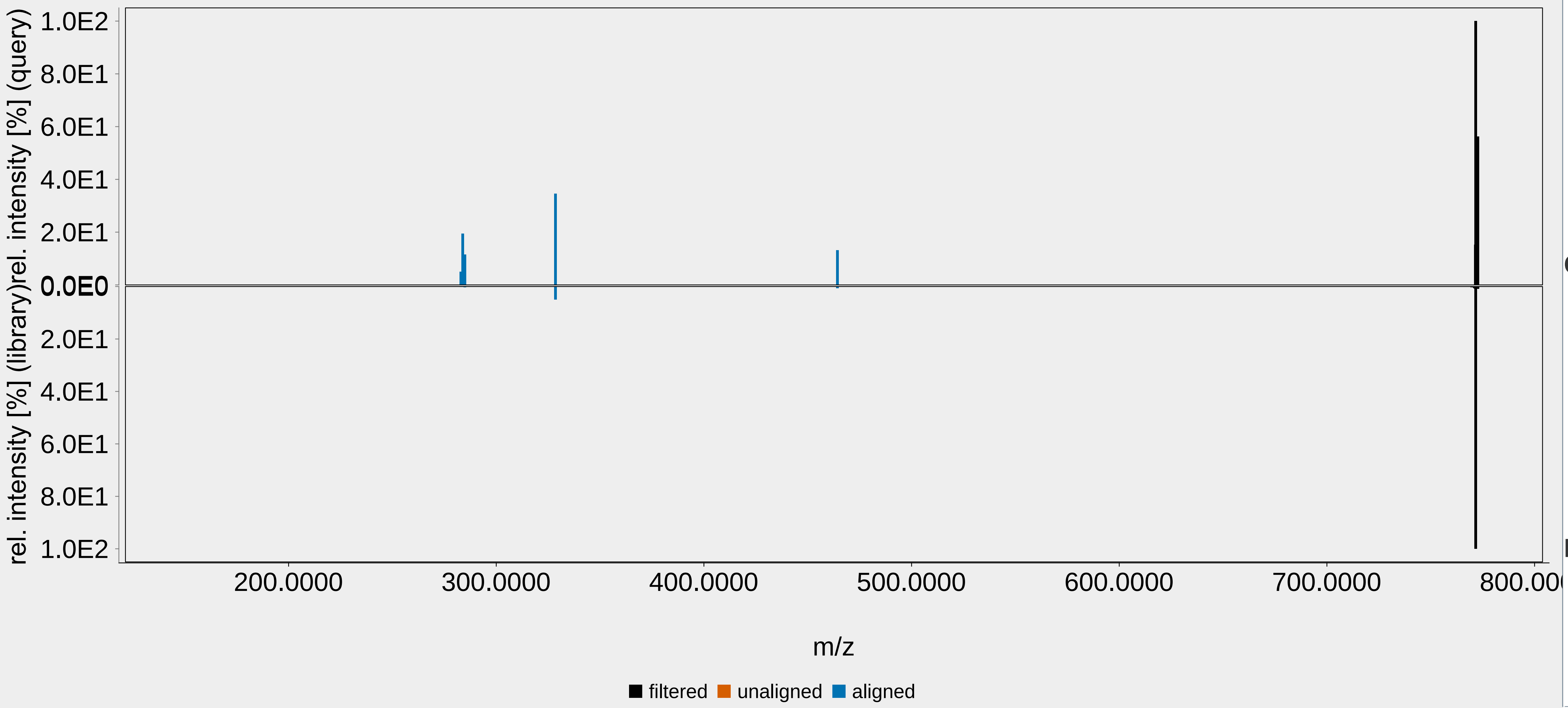

Compound information

Name: PE(P-18:0/22:6); [M-H]-

Exact mass: 0.0

Ion type: M-H

Precursor m/z: 774.544

Charge: 1

Polarity: Negative

Instrument information

Instrument type: HCD; Velos

Ion source: LC-ESI

Ms level: 2

Acquisition: Commercial

Database links

Other information

Principal investigator: Thomas Metz

Data collector: Thomas Metz

Entry ID: CCMSLIB00010087922

PE(P-18:0/22:6); [M-H]- C45H77N1O7P1

0.860

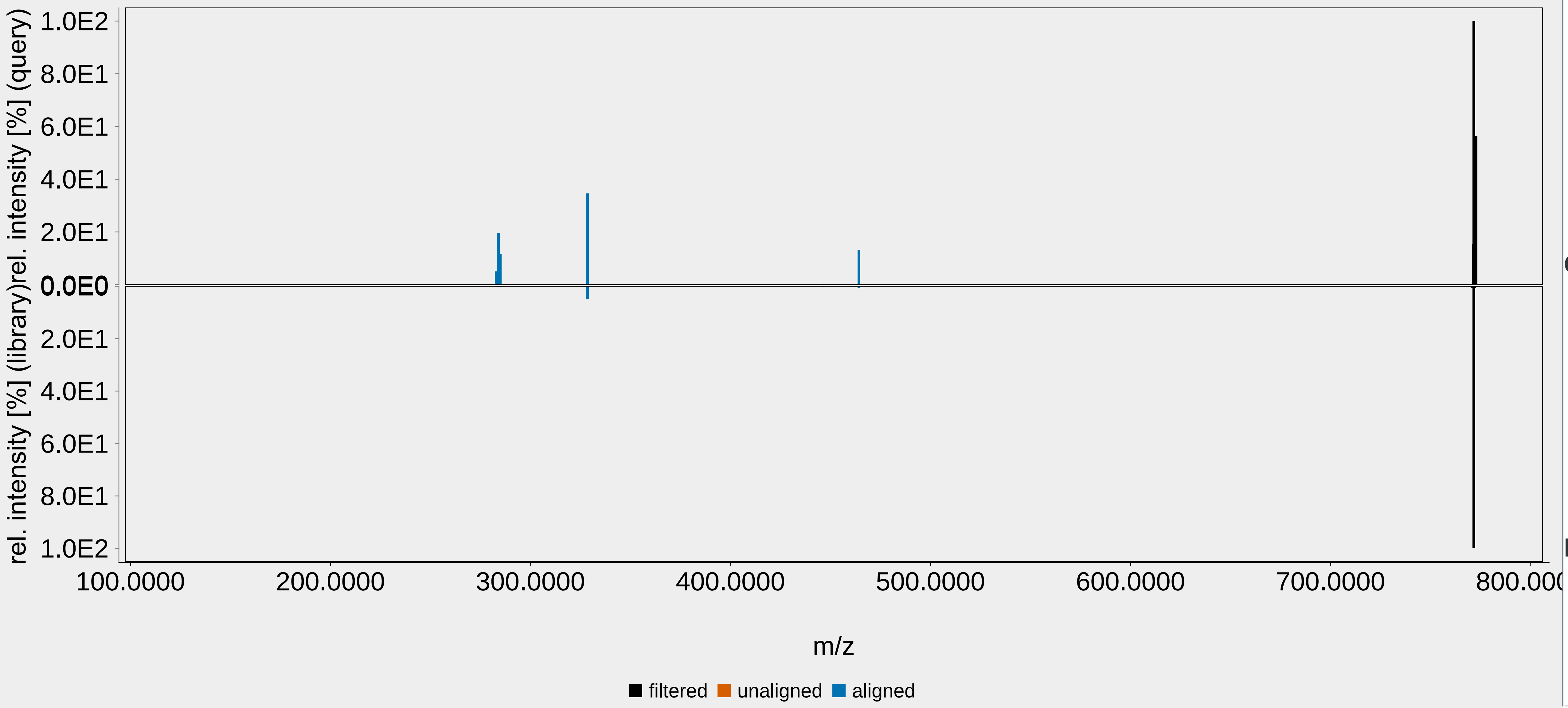

Compound information

Name: PE(P-18:0/22:6); [M-H]-

Exact mass: 0.0

Ion type: M-H

Precursor m/z: 774.544

Charge: 1

Polarity: Negative

Instrument information

Instrument type: HCD; Velos

Ion source: LC-ESI

Ms level: 2

Acquisition: Commercial

Database links

Other information

Principal investigator: Thomas Metz

Data collector: Thomas Metz

Entry ID: CCMSLIB00010088413

PA 16:0_22:6

0.729

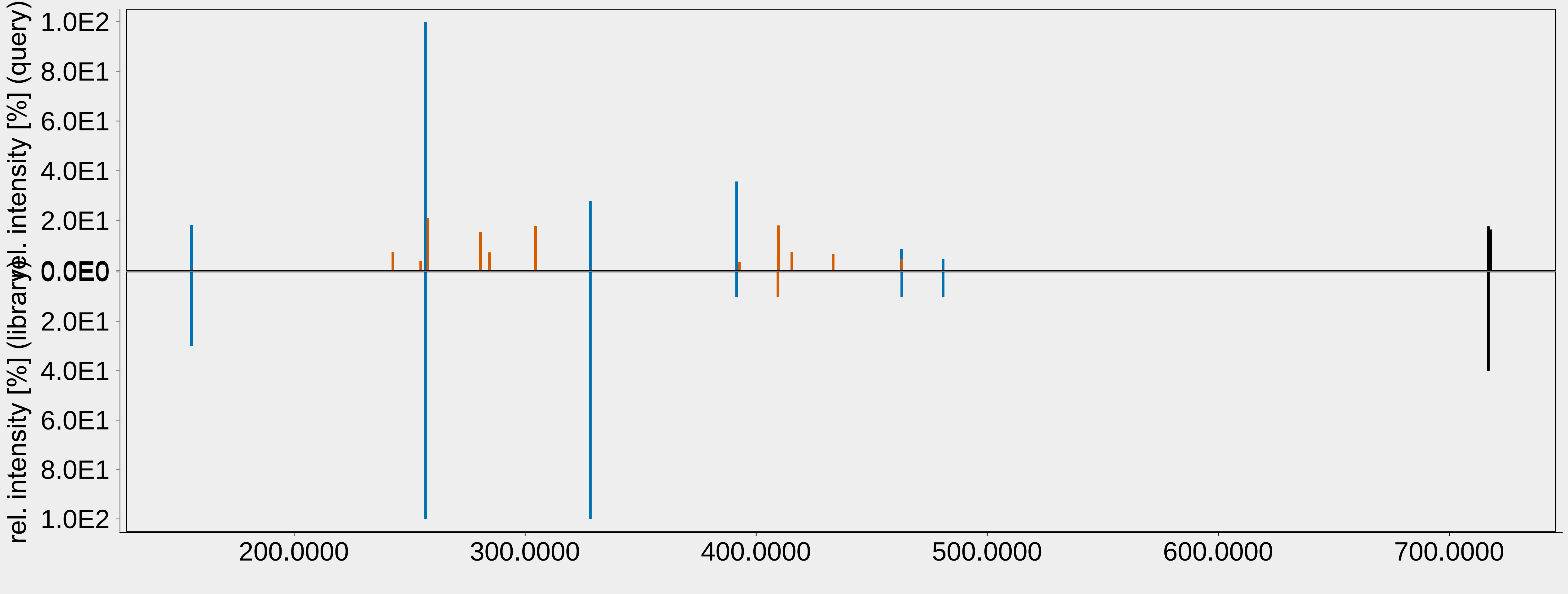

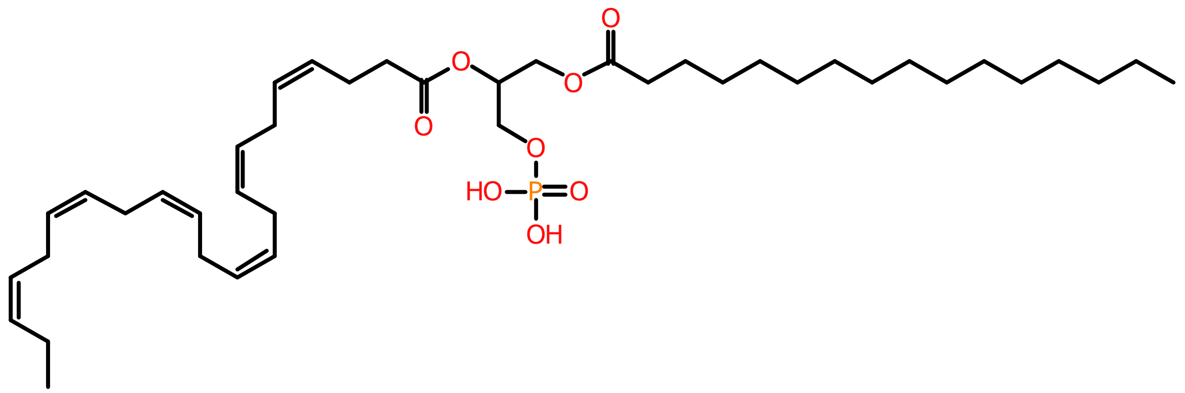

Compound information

Name: PA 16:0_22:6

Formula: C41H69O8P

Ion type: [M-H]-

Precursor m/z: 719.46573

Ccs: 257.26

InChI key:

SMILES:

Num peaks: 8

Instrument information

Database links

Other information

PG(22:6/22:6); [M-H]- C50H74O10P1

0.709

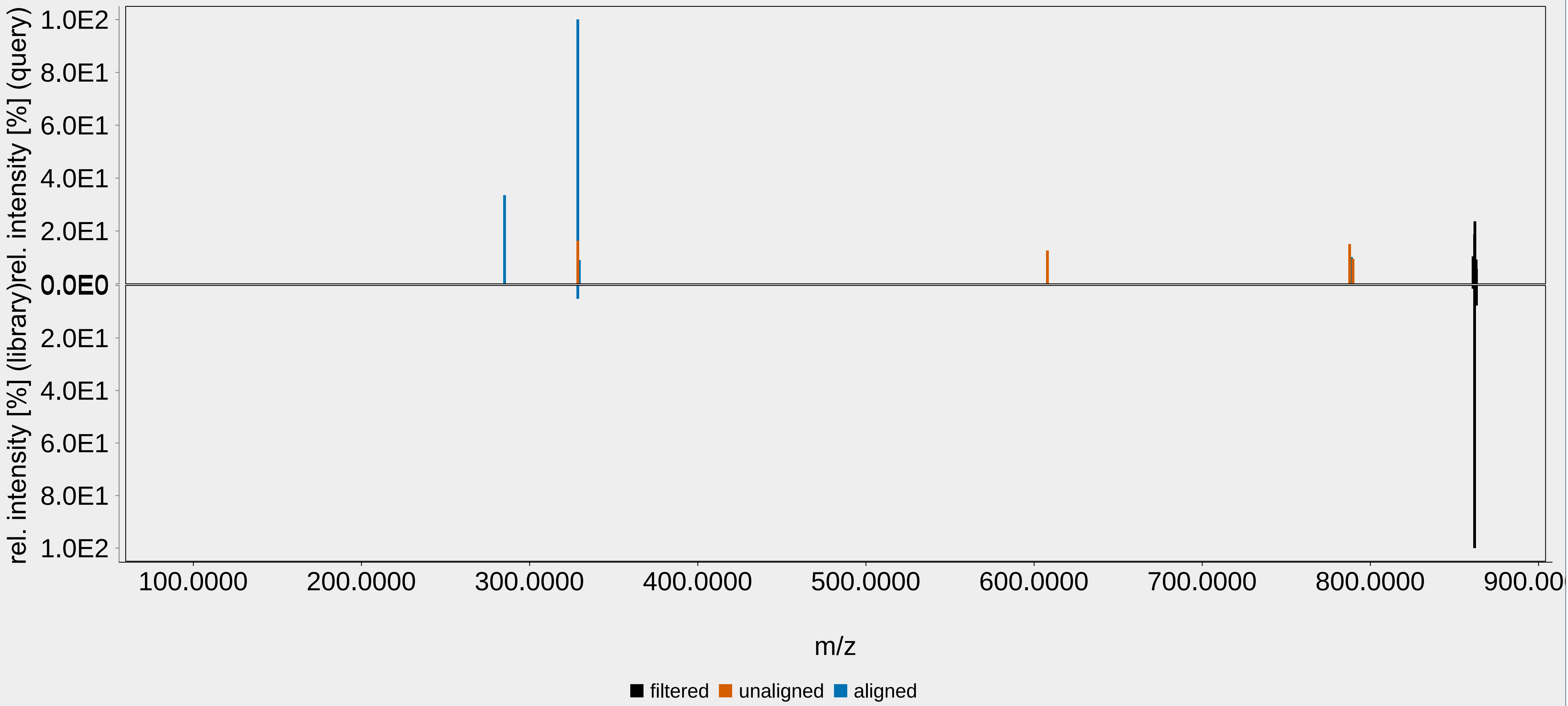

Compound information

Name: PG(22:6/22:6); [M-H]- C50H74O10P1

Exact mass: 0.0

Ion type: M-H

Precursor m/z: 865.502

Charge: 1

Polarity: Negative

Instrument information

Instrument type: HCD; Velos

Ion source: LC-ESI

Ms level: 2

Acquisition: Commercial

Database links

Other information

Principal investigator: Thomas Metz

Data collector: Thomas Metz

Entry ID: CCMSLIB00010083995

PG(22:6/22:6); [M-H]- C50H74O10P1 **0.726**

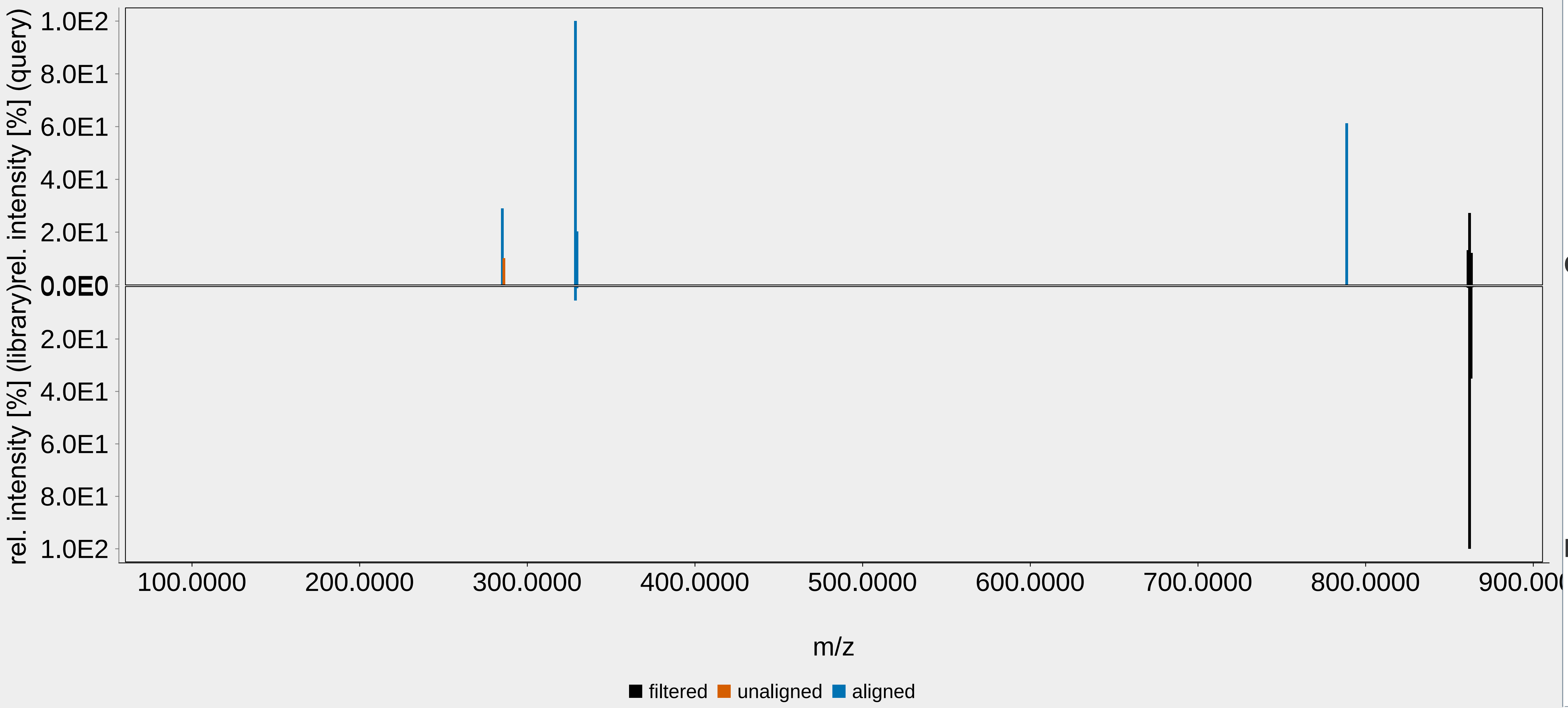

Compound information

Name: PG(22:6/22:6); [M-H]- C50H74O10P1

Exact mass: 0.0

Ion type: M-H

Precursor m/z: 865.502

Charge: 1

Polarity: Negative

Instrument information

Instrument type: HCD; Velos

Ion source: LC-ESI

Ms level: 2

Acquisition: Commercial

Database links

Other information

Principal investigator: Thomas Metz

Data collector: Thomas Metz

Entry ID: CCMSLIB00010082177

PI 40:4 **0.787**

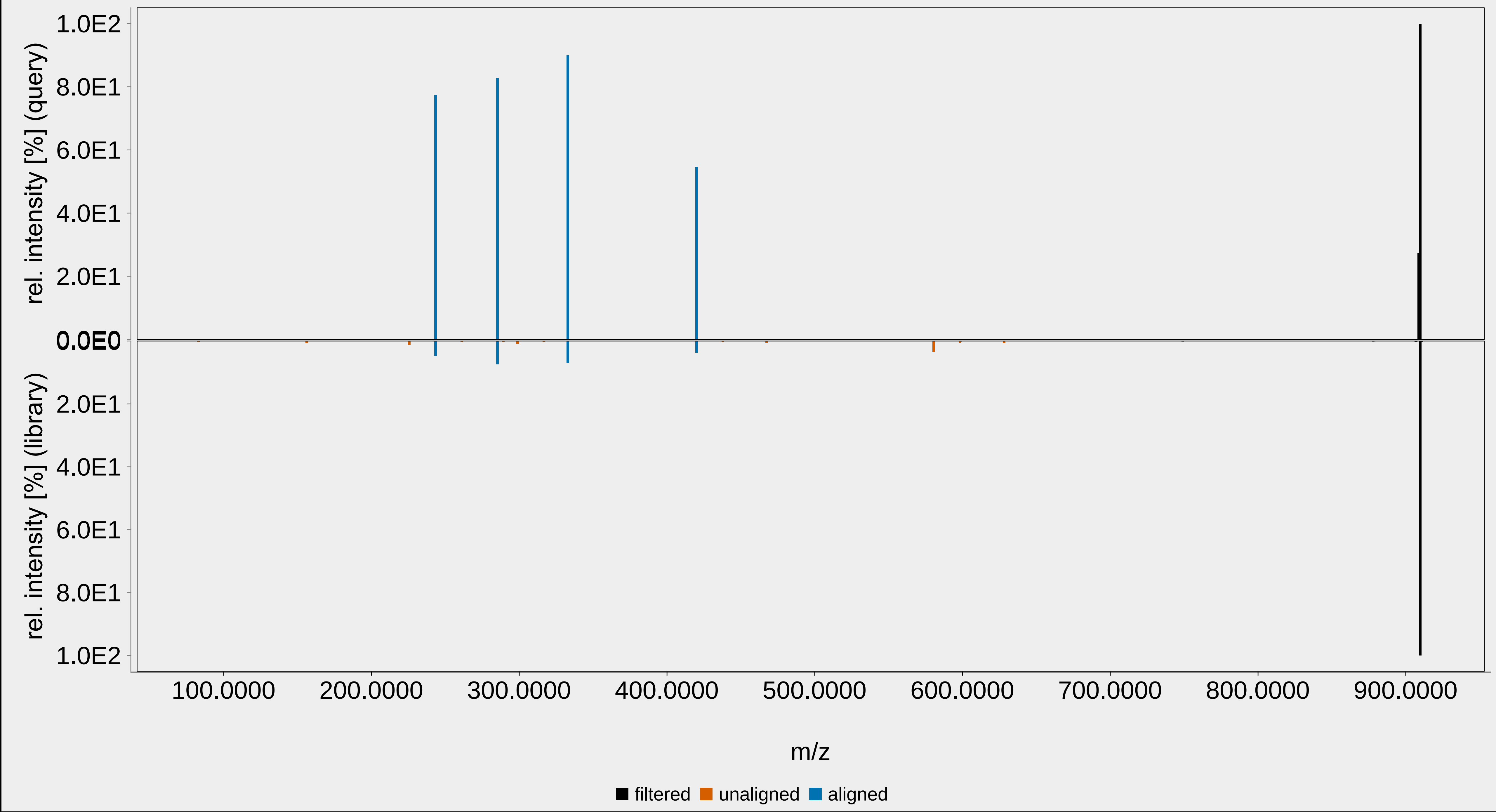

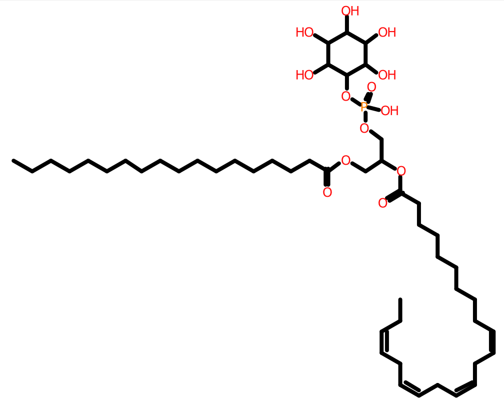

Compound information

Name: PI 40:4

Formula: C49H87O13P

Mol. weight: 915.198

Exact mass: 915.198

Ion type: [M-H]-

Precursor m/z: 913.5835

RT: 9.142293

Polarity: negative

InChI:

InChI key: VTYLJIRGLIZEIC-PYQGTGCGSA-N

SMILES:

Instrument information

Instrument type: LC-ESI-QTOF

Instrument: AB Sciex TripleTOF 5600+ system

Ms level: MS2

Collision energy: 45V

Database links

Pubchem: 138283546

MoNA ID: LQB00697

Other information

Principal investigator: Kazutaka Ikeda, Hiroshi

Data collector: Kazutaka Ikeda, Hiroshi

PI 40:4 0.787

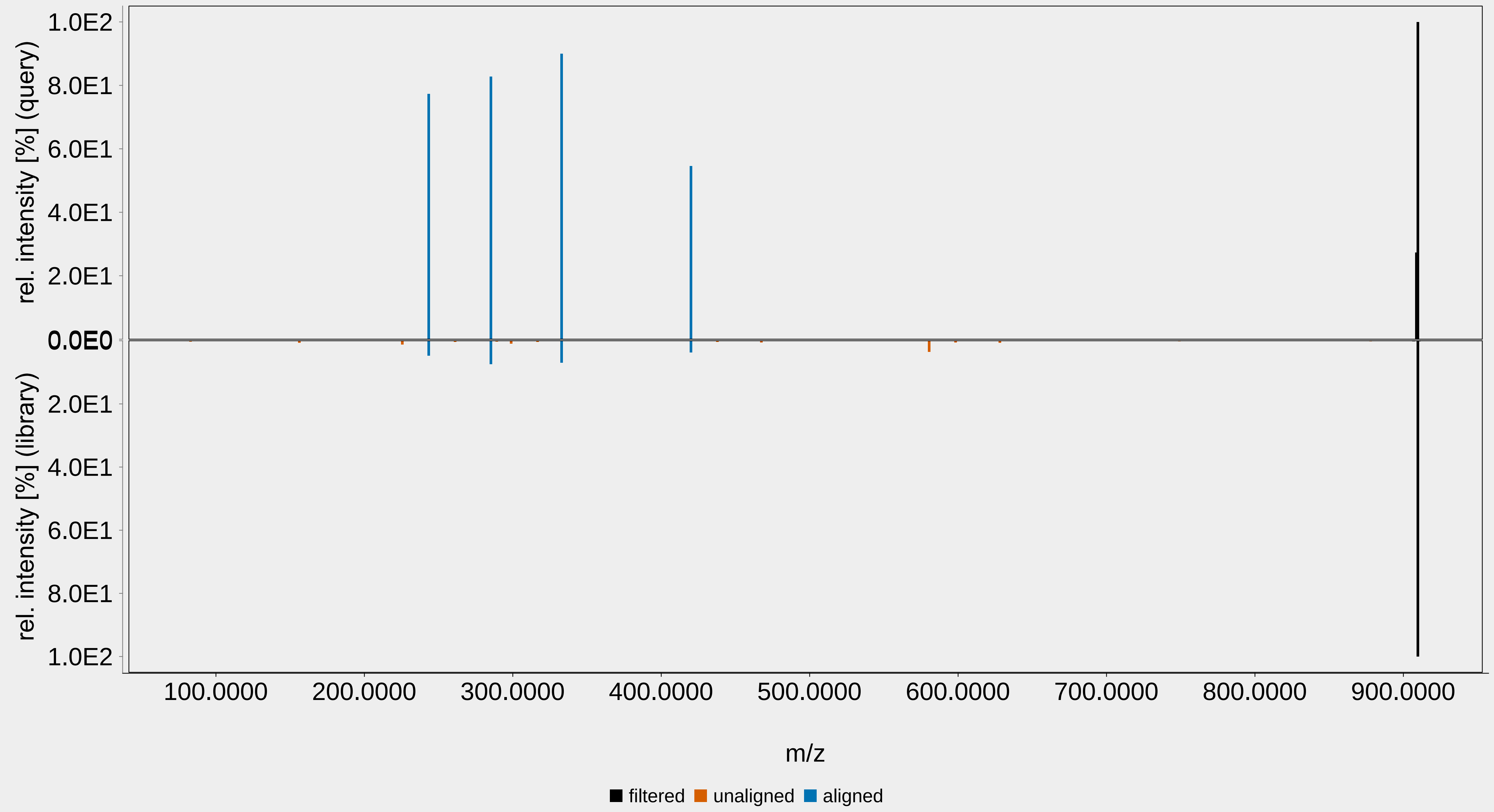

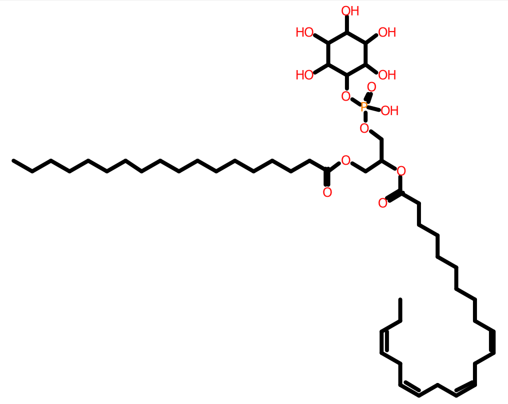

Compound information

Name: PI 40:4

Formula: C49H87O13P

Mol. weight: 915.198

Exact mass: 915.198

Ion type: [M-H]-

Precursor m/z: 913.5835

RT: 9.142293

Polarity: negative

InChI:

InChI key: VTYLJIRGLIZEIC-PYQGTGCGSA-N

SMILES:

Instrument information

Instrument type: LC-ESI-QTOF

Instrument: AB Sciex TripleTOF 5600+ system

Ms level: MS2

Collision energy: 45V

Database links

Pubchem: 138283546

MoNA ID: LQB00311

Other information

Principal investigator: Kazutaka Ikeda, Hiroshi

Data collector: Kazutaka Ikeda, Hiroshi

PE(18:1/20:4); [M-H]- C43H75N1O8P1 **0.872**

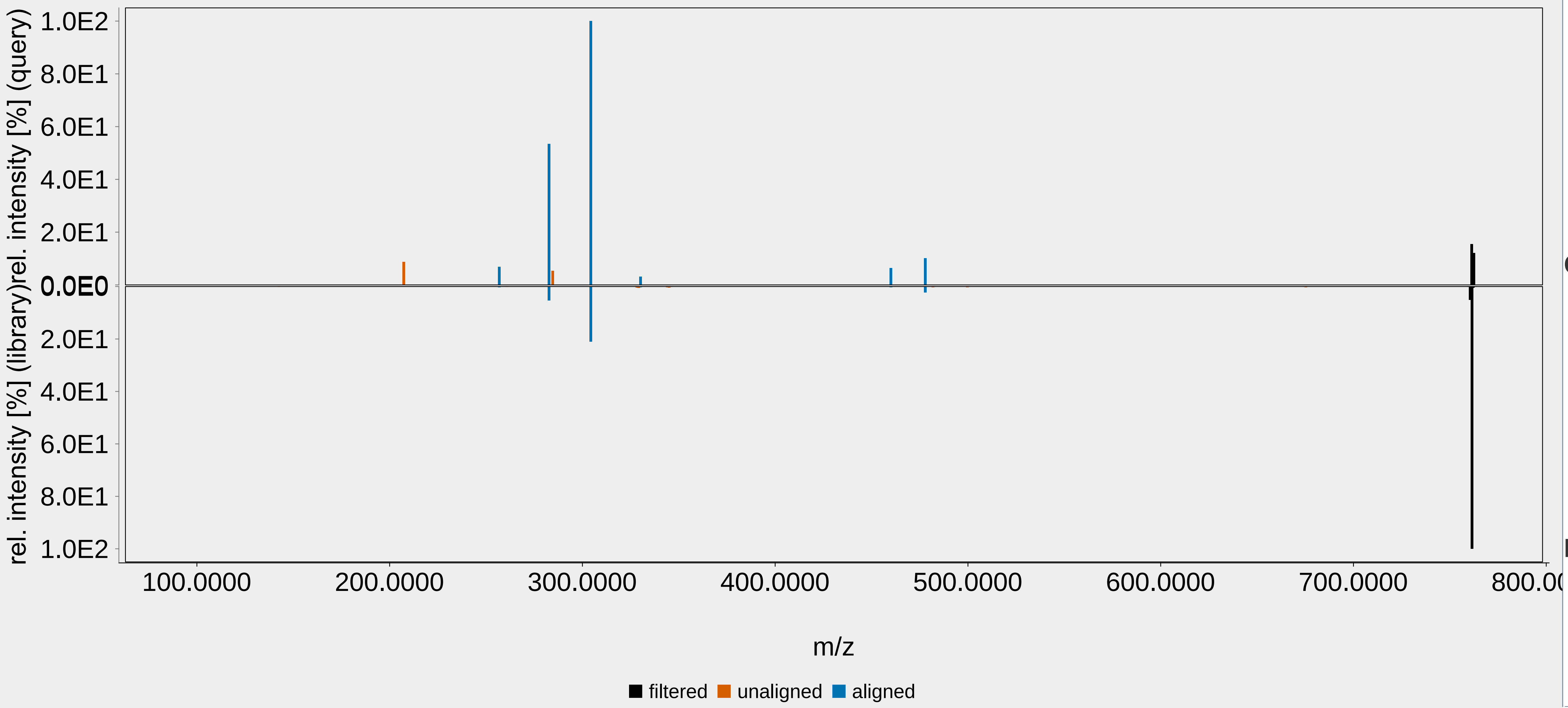

Compound information

Name: PE(18:1/20:4); [M-H]- C43H75N1O8P1

Exact mass: 0.0

Ion type: M-H

Precursor m/z: 764.523

Charge: 1

Polarity: Negative

Instrument information

Instrument type: HCD; Velos

Ion source: LC-ESI

Ms level: 2

Acquisition: Commercial

Database links

Other information

Principal investigator: Thomas Metz

Data collector: Thomas Metz

Entry ID: CCMSLIB00010063428

PE 18:1_20:4

0.880

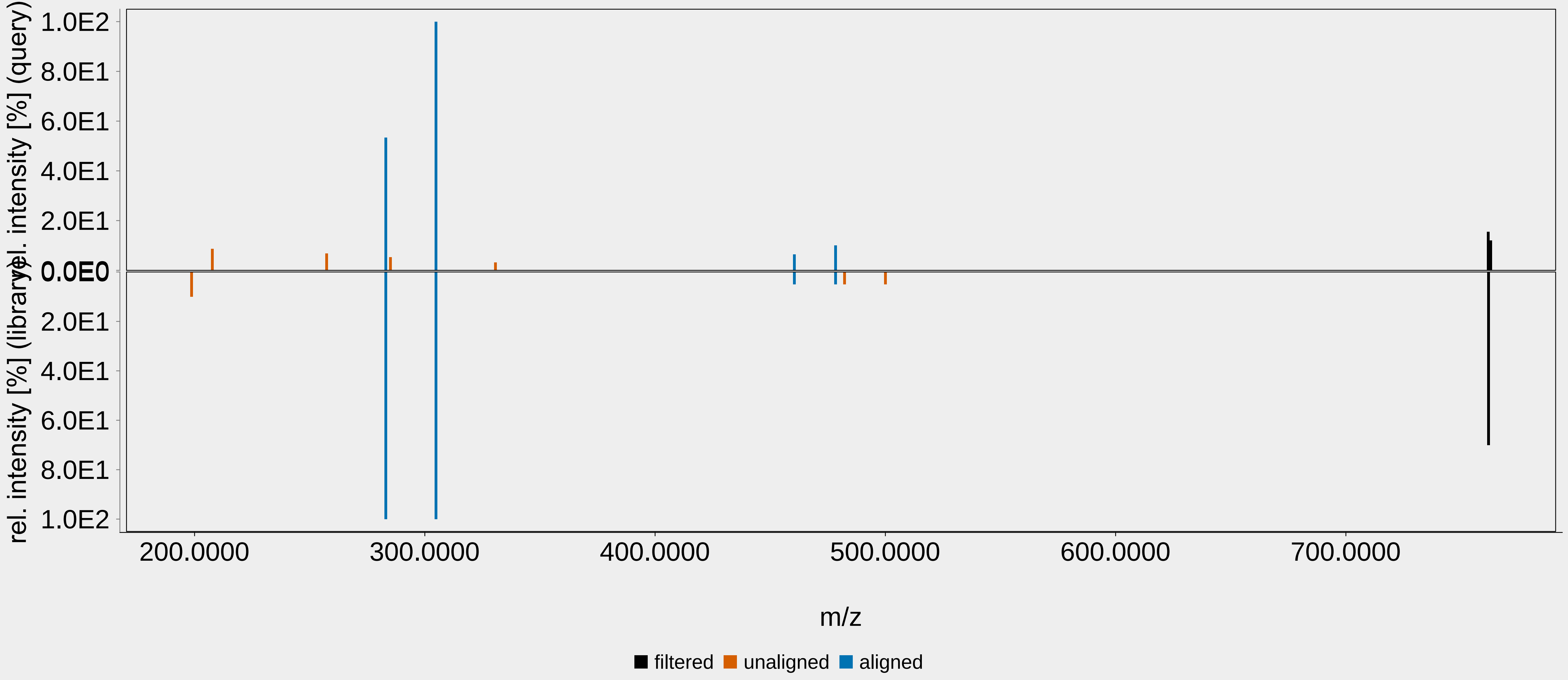

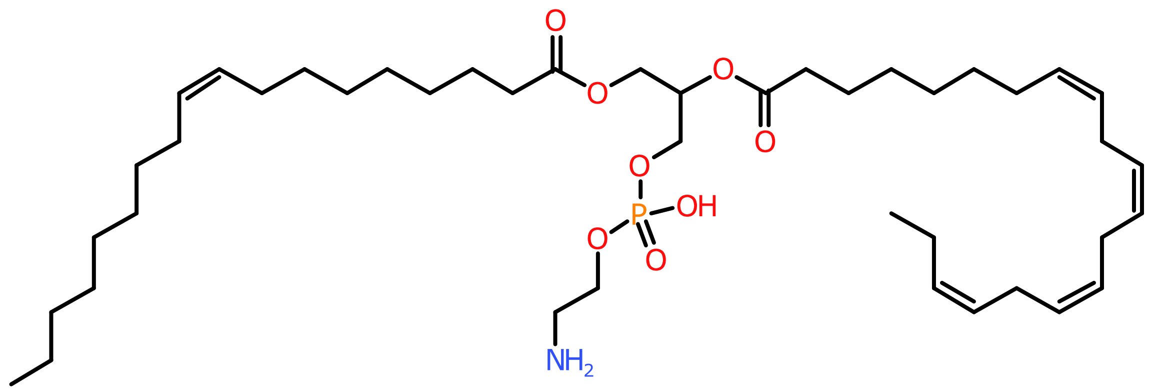

Compound information

Name: PE 18:1_20:4

Formula: C43H76NO8P

Ion type: [M-H]-

Precursor m/z: 764.52358

Ccs: 268.34

InChI key:

SMILES:

Num peaks: 8

Instrument information

Database links

Other information

PA 18:0_22:4

0.878

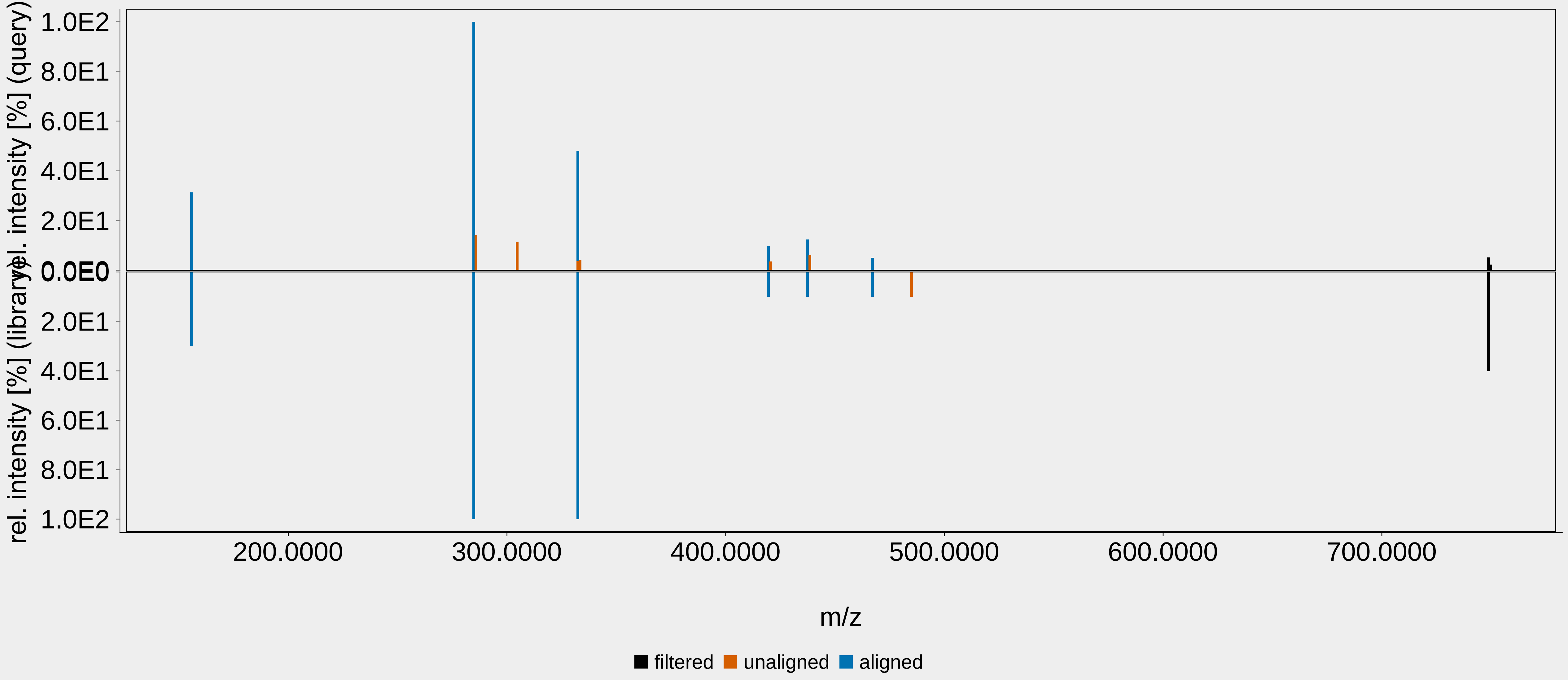

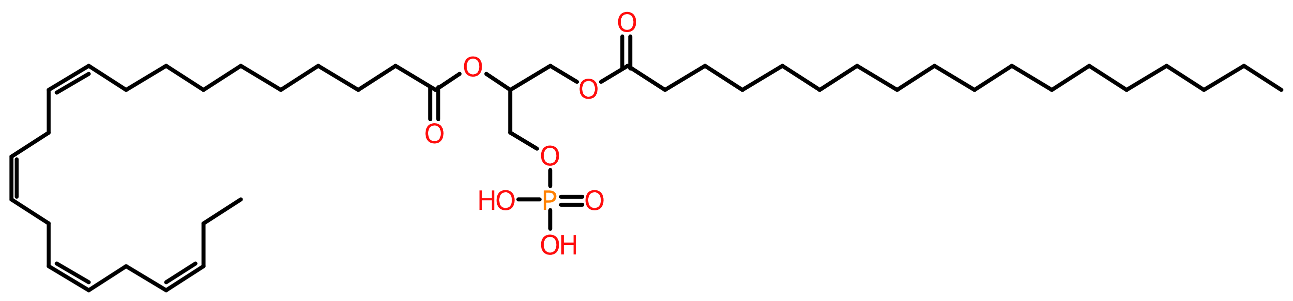

Compound information

Name: PA 18:0_22:4

Formula: C43H77O8P

Ion type: [M-H]-

Precursor m/z: 751.52833

Ccs: 267.82

InChI key:

SMILES:

Num peaks: 8

Instrument information

Database links

Other information

Arachidonic acid

0.714

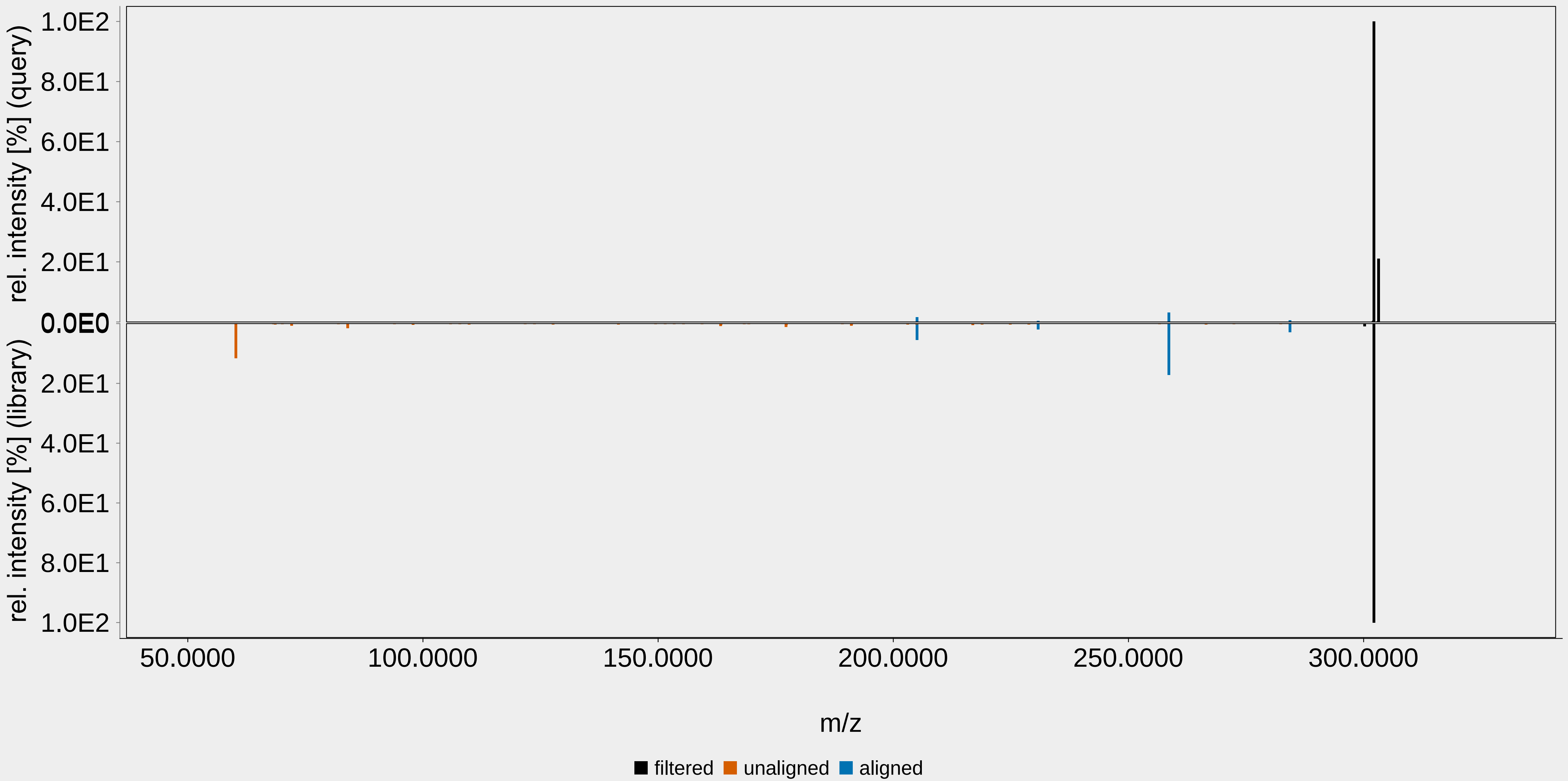

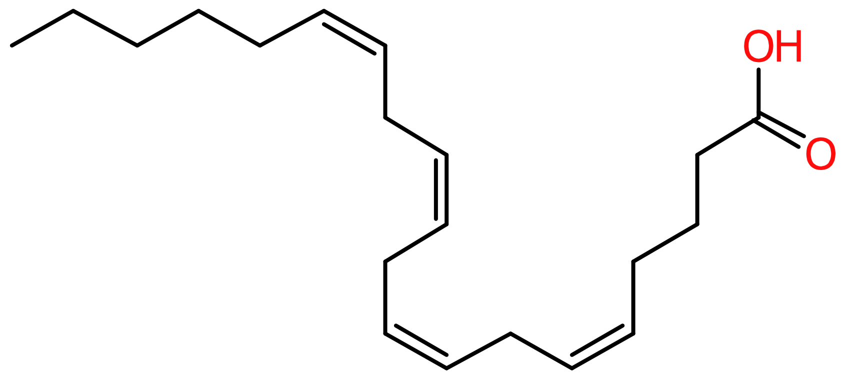

Compound information

Name: Arachidonic acid

Formula: C20H32O2

Ion type: [M-H]-

Precursor m/z: 303.232041974554

RT: 4.158056

Polarity: negative

InChI:

InChI key:

SMILES:

Instrument information

Instrument type: LC-ESI-QFT

Instrument: Thermo Q-Exactive Plus

Ms level: MS2

Collision energy: 30.0 eV

Database links

Other information

Principal investigator: Prasad Phapale

Data collector: Prasad Phapale

EicosatetraenoicAcid_Arachidonic 0.836

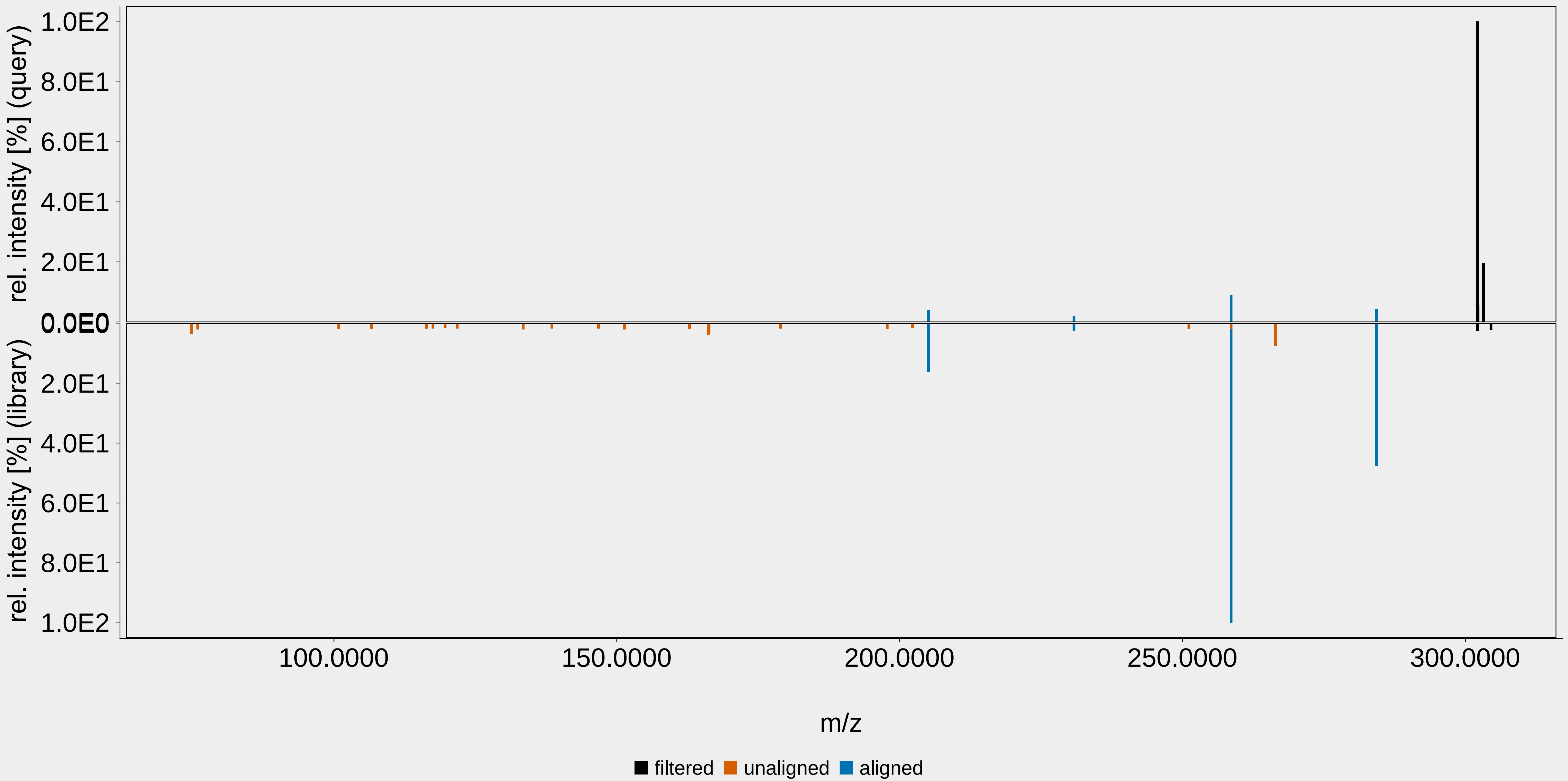

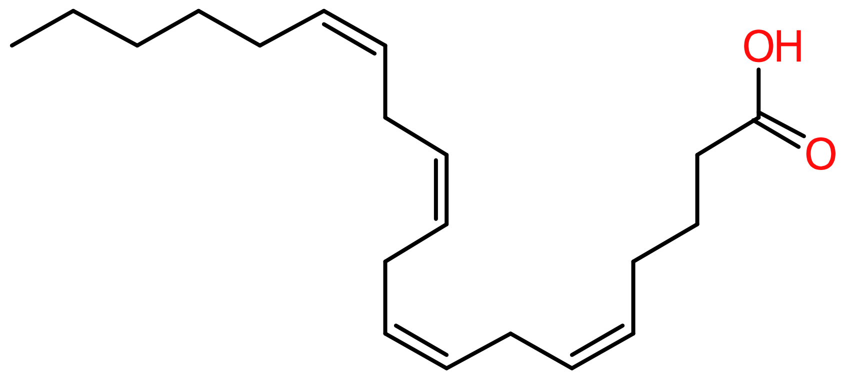

Compound information

Name: EicosatetraenoicAcid_Arachidonic

Exact mass: 304.24

Ion type: M-H

Precursor m/z: 303.233

Charge: -1

Polarity: Negative

InChI:

SMILES:

Instrument information

Instrument type: Orbitrap

Ion source: ESI

Ms level: 2

Acquisition: Commercial

Database links

Pubmed: 444899.0

CAS: 506-32-1

Other information

Principal investigator: Ulaszewska

Data collector: Marynka Ulaszewska

Entry ID: CCMSLIB00006676895

PA 16:1_20:4

0.877

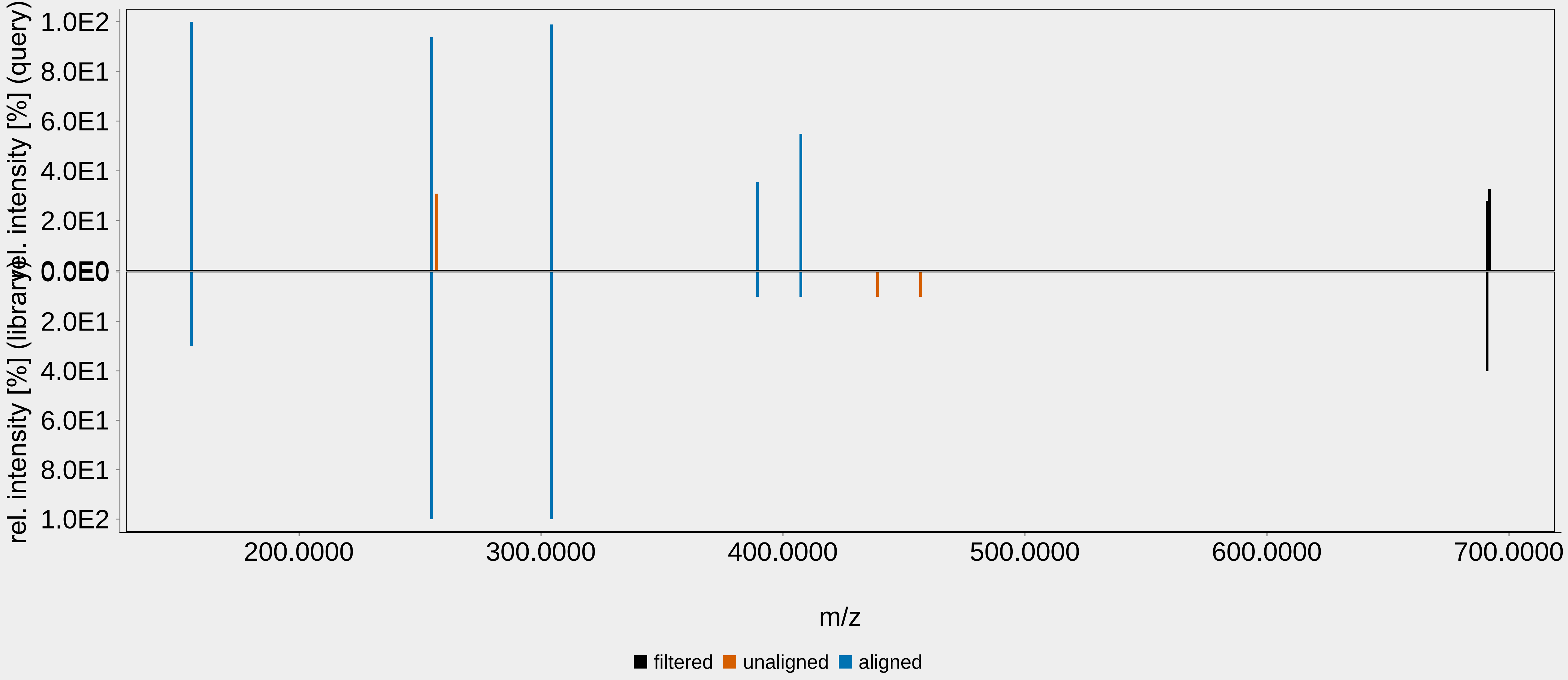

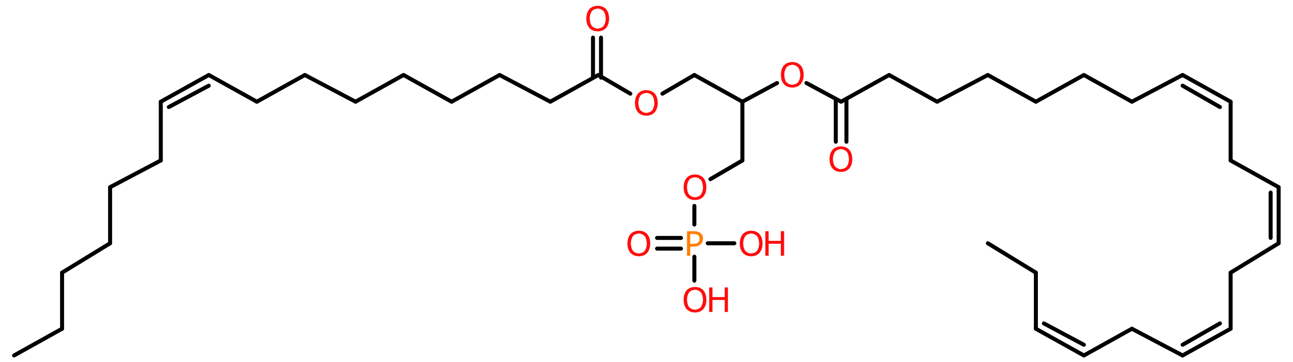

Compound information

Name: PA 16:1_20:4

Formula: C39H67O8P

Ion type: [M-H]-

Precursor m/z: 693.45008

Ccs: 253.91

InChI key:

SMILES:

Num peaks: 8

Instrument information

Database links

Other information

HYDROQUINIDINE

0.884

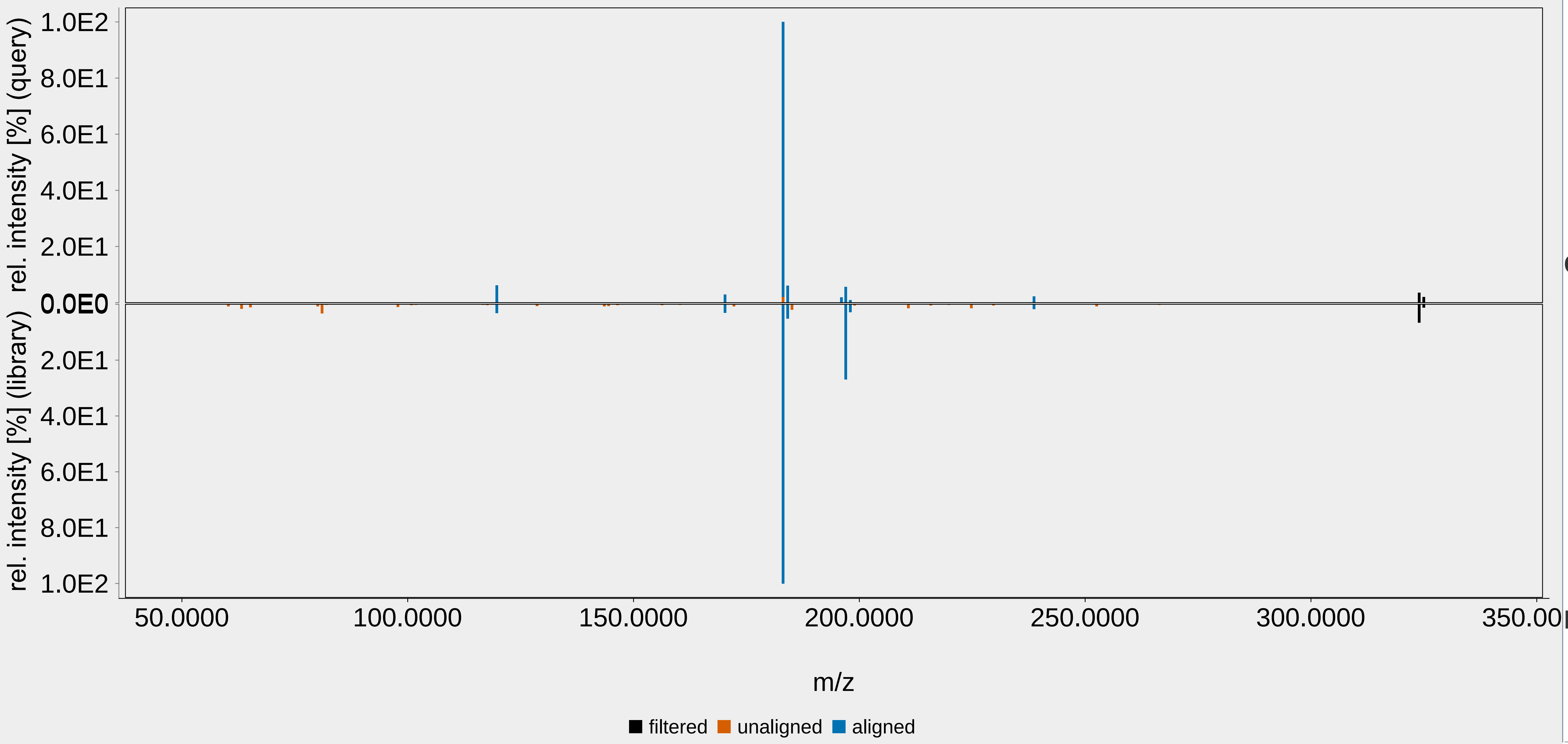

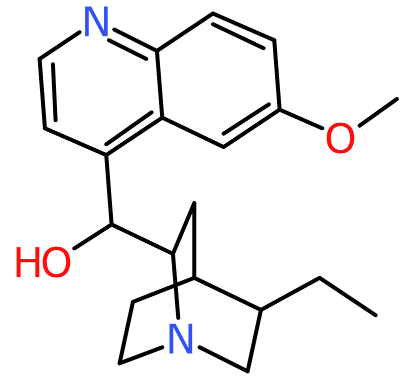

Compound information

Name: HYDROQUINIDINE

Formula: C20H26N2O2

Ion type: [M-H]-

Precursor m/z: 325.192138671875

Polarity: negative

InChI:

InChI key:

SMILES:

Instrument information

Instrument type: ESI-QFT

Instrument: Thermo Q Exactive HF

Ms level: MS2

Collision energy: 65HCD

Database links

Other information

Principal investigator: Arpana Vaniya

Data collector: Arpana Vaniya

HYDROQUINIDINE

0.884

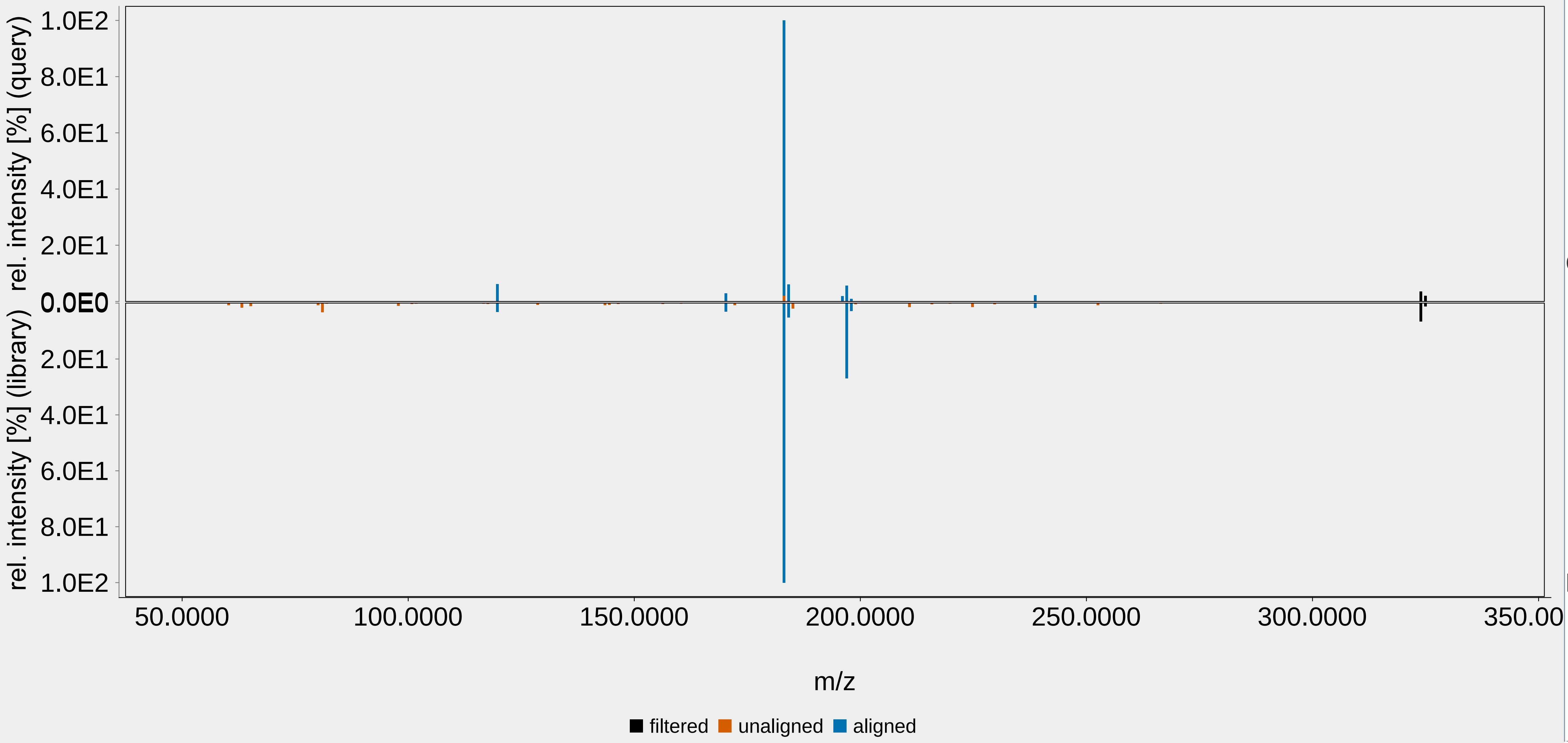

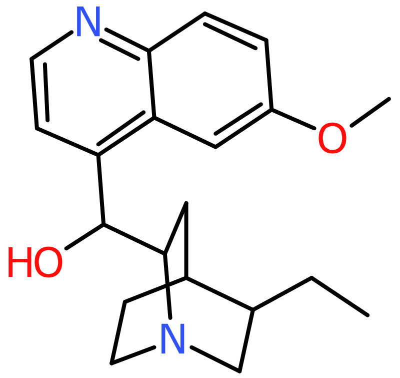

Compound information

Name: HYDROQUINIDINE

Exact mass: 0.0

Ion type: [M-H]-

Precursor m/z: 325.192

Charge: 1

Polarity: negative

InChI:

Instrument information

Instrument type: ESI-QFT

Ms level: 2

Acquisition: isolated

Database links

Other information

Principal investigator: MoNA

Data collector: MoNA:VF-NPL-QEHF003075

Entry ID: CCMSLIB00004694138

PE(18:0/20:3); [M-H]- C43H79N1O8P1 **0.730**

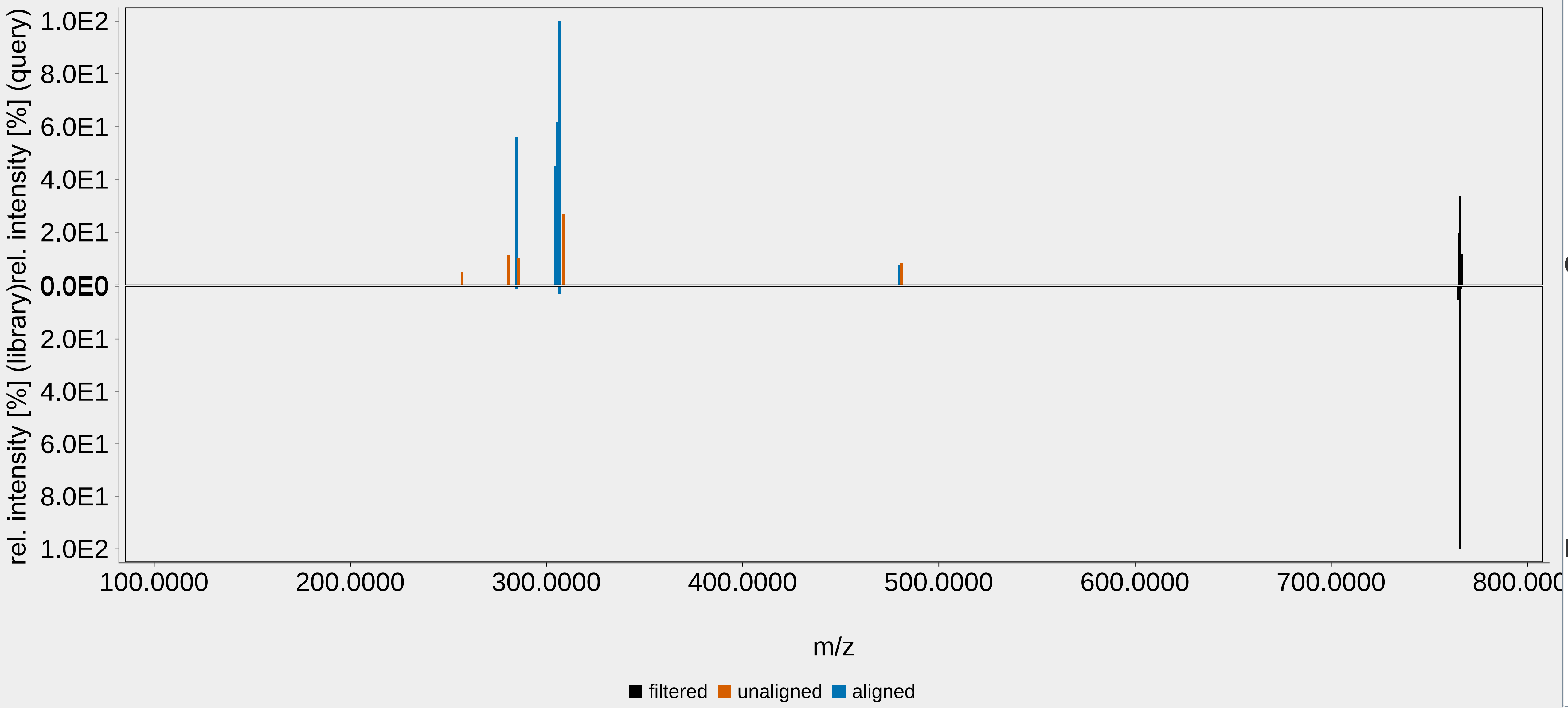

Compound information

Name: PE(18:0/20:3); [M-H]- C43H79N1O8P1

Exact mass: 0.0

Ion type: M-H

Precursor m/z: 768.554

Charge: 1

Polarity: Negative

Instrument information

Instrument type: HCD; Velos

Ion source: LC-ESI

Ms level: 2

Acquisition: Commercial

Database links

Other information

Principal investigator: Thomas Metz

Data collector: Thomas Metz

Entry ID: CCMSLIB00010079224

PE(18:0/20:3); [M-H]- C43H79N1O8P1 **0.759**

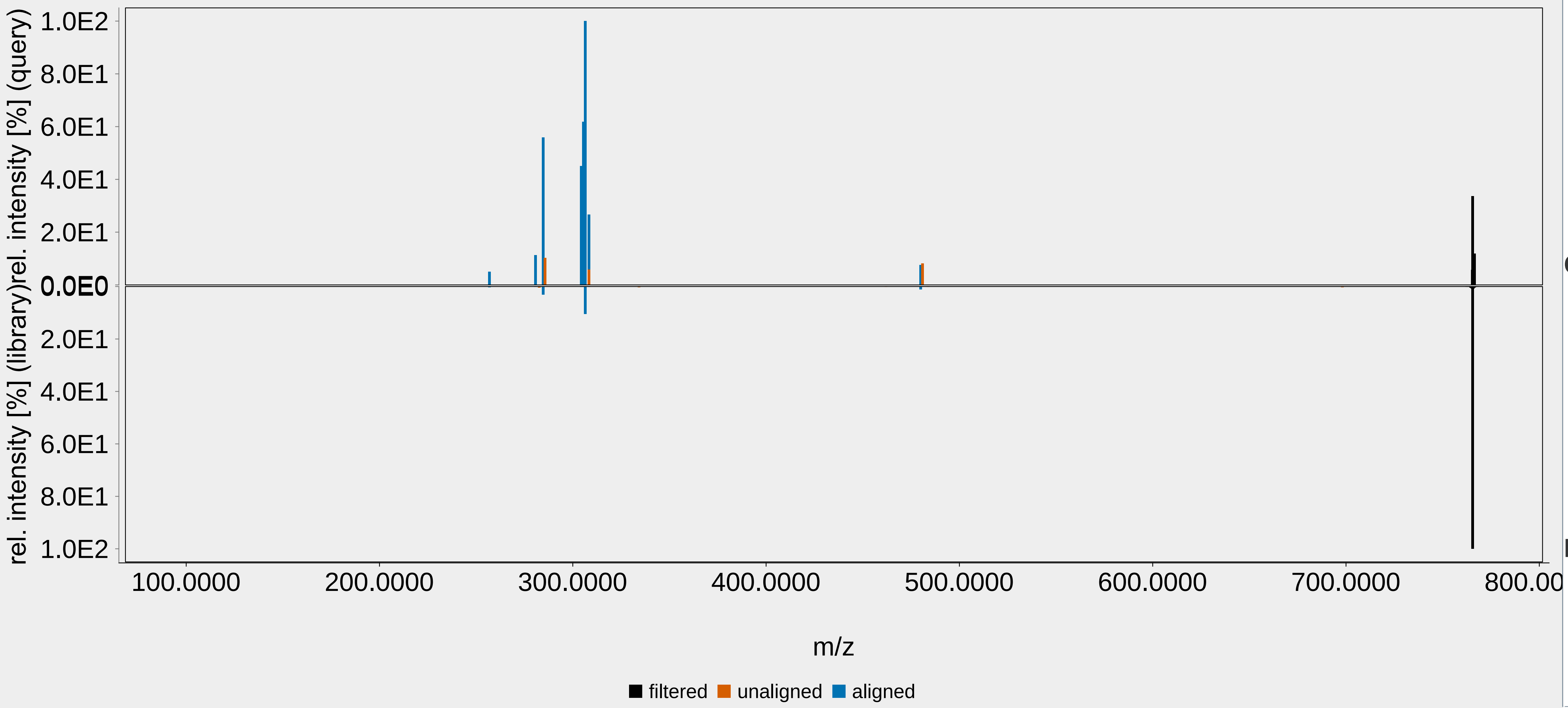

Compound information

Name: PE(18:0/20:3); [M-H]- C43H79N1O8P1

Exact mass: 0.0

Ion type: M-H

Precursor m/z: 768.554

Charge: 1

Polarity: Negative

Instrument information

Instrument type: HCD; Velos

Ion source: LC-ESI

Ms level: 2

Acquisition: Commercial

Database links

Other information

Principal investigator: Thomas Metz

Data collector: Thomas Metz

Entry ID: CCMSLIB00010071376

PA 22:6_22:6

0.946

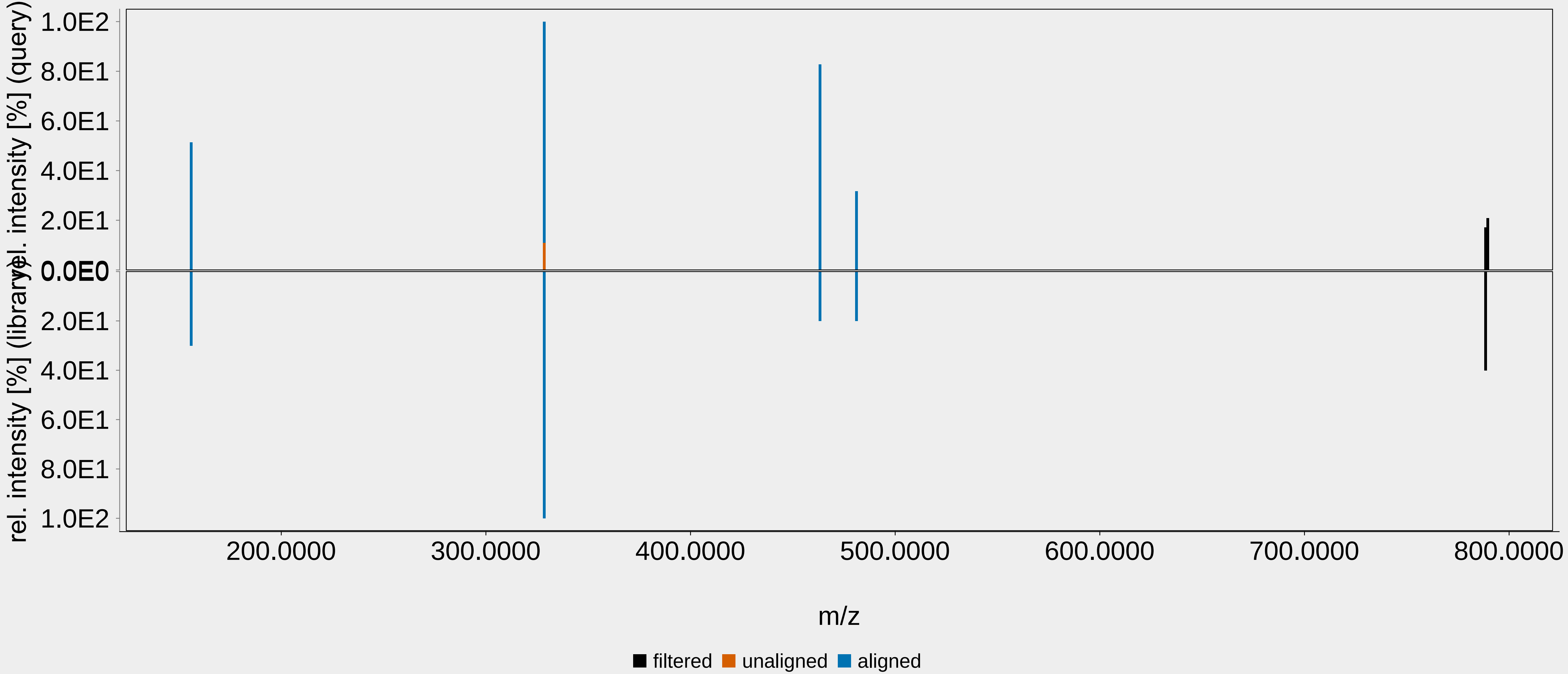

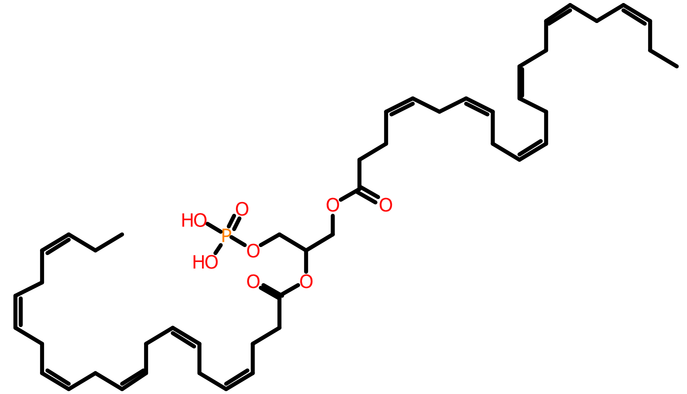

Compound information

Name: PA 22:6_22:6

Formula: C47H69O8P

Ion type: [M-H]-

Precursor m/z: 791.46573

Ccs: 269.85

InChI key: YUDIFVLBAGUYSQ-GZSOIYOPNA-

SMILES:

Num peaks: 5

Instrument information

Database links

Other information

PA(18:0/18:1); [M-H]- C39H74O8P1 **0.862**

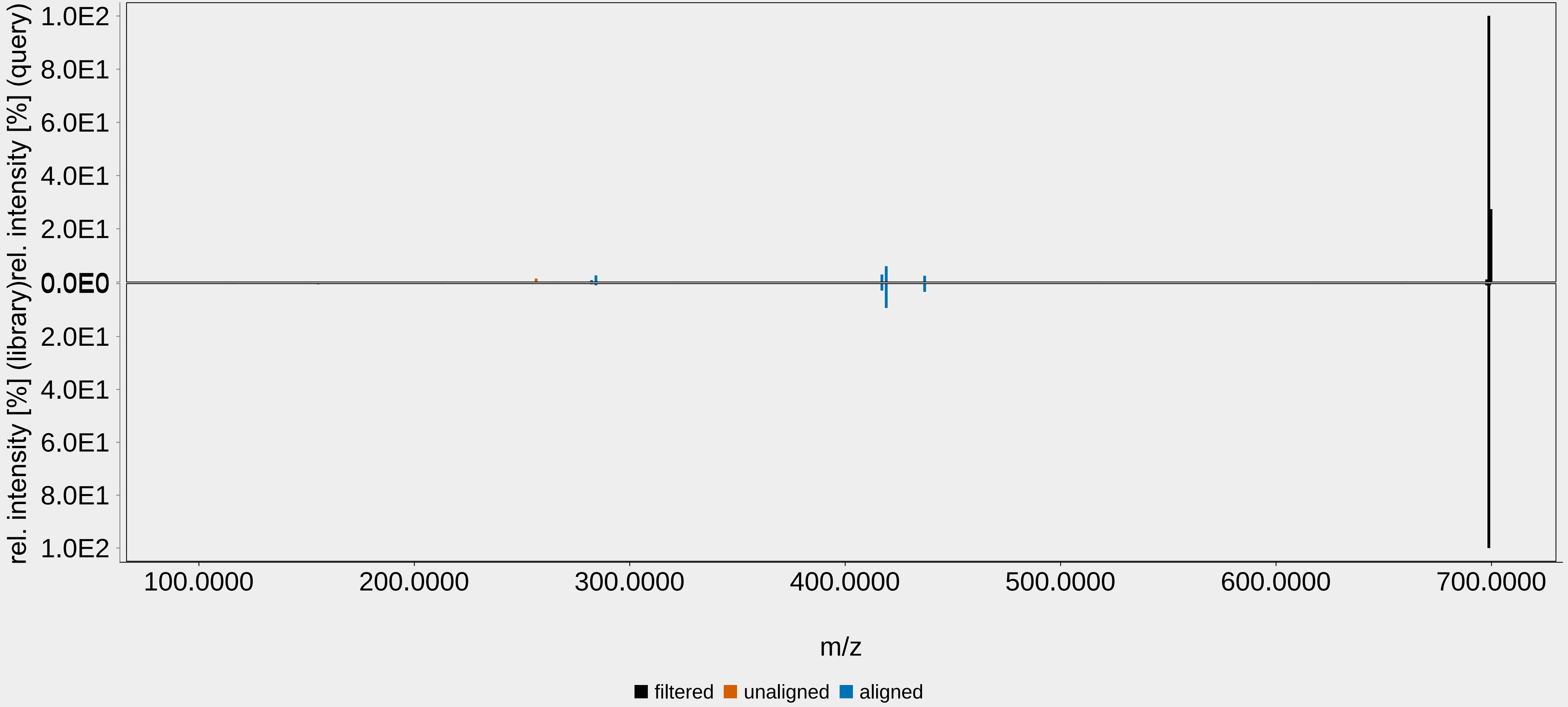

Compound information

Name: PA(18:0/18:1); [M-H]- C39H74O8P1

Exact mass: 0.0

Ion type: M-H

Precursor m/z: 701.512

Charge: 1

Polarity: Negative

Instrument information

Instrument type: HCD; Velos

Ion source: LC-ESI

Ms level: 2

Acquisition: Commercial

Database links

Other information

Principal investigator: Thomas Metz

Data collector: Thomas Metz

Entry ID: CCMSLIB00010076011

PA 18:0_18:1

0.895

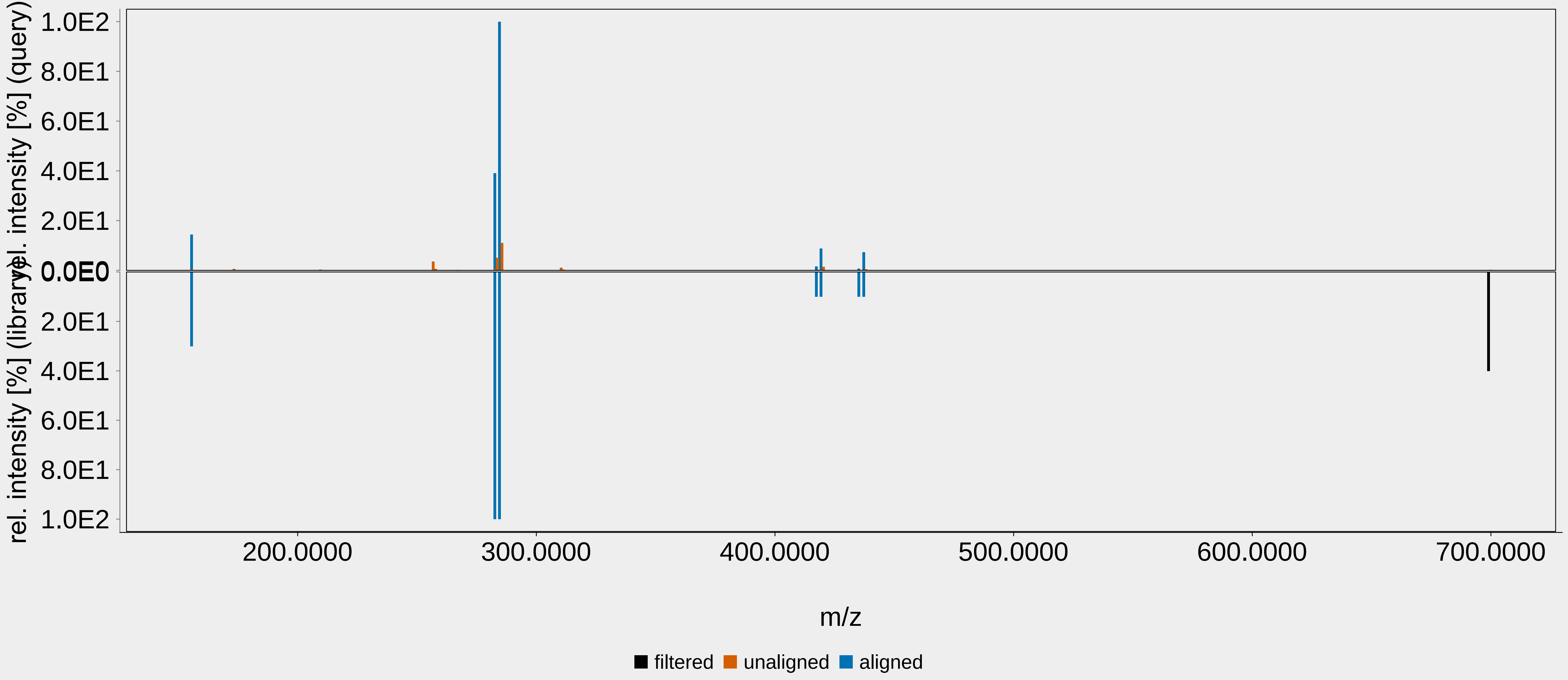

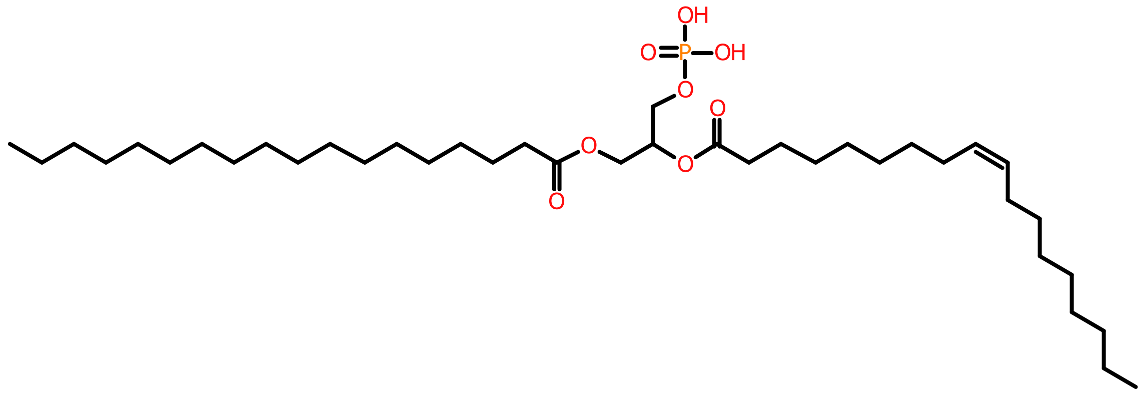

Compound information

Name: PA 18:0_18:1

Formula: C39H75O8P

Ion type: [M-H]-

Precursor m/z: 701.51268

Ccs: 258.98

InChI key:

SMILES:

Num peaks: 8

Instrument information

Database links

Other information

Glutathione - 30.0 eV

0.774

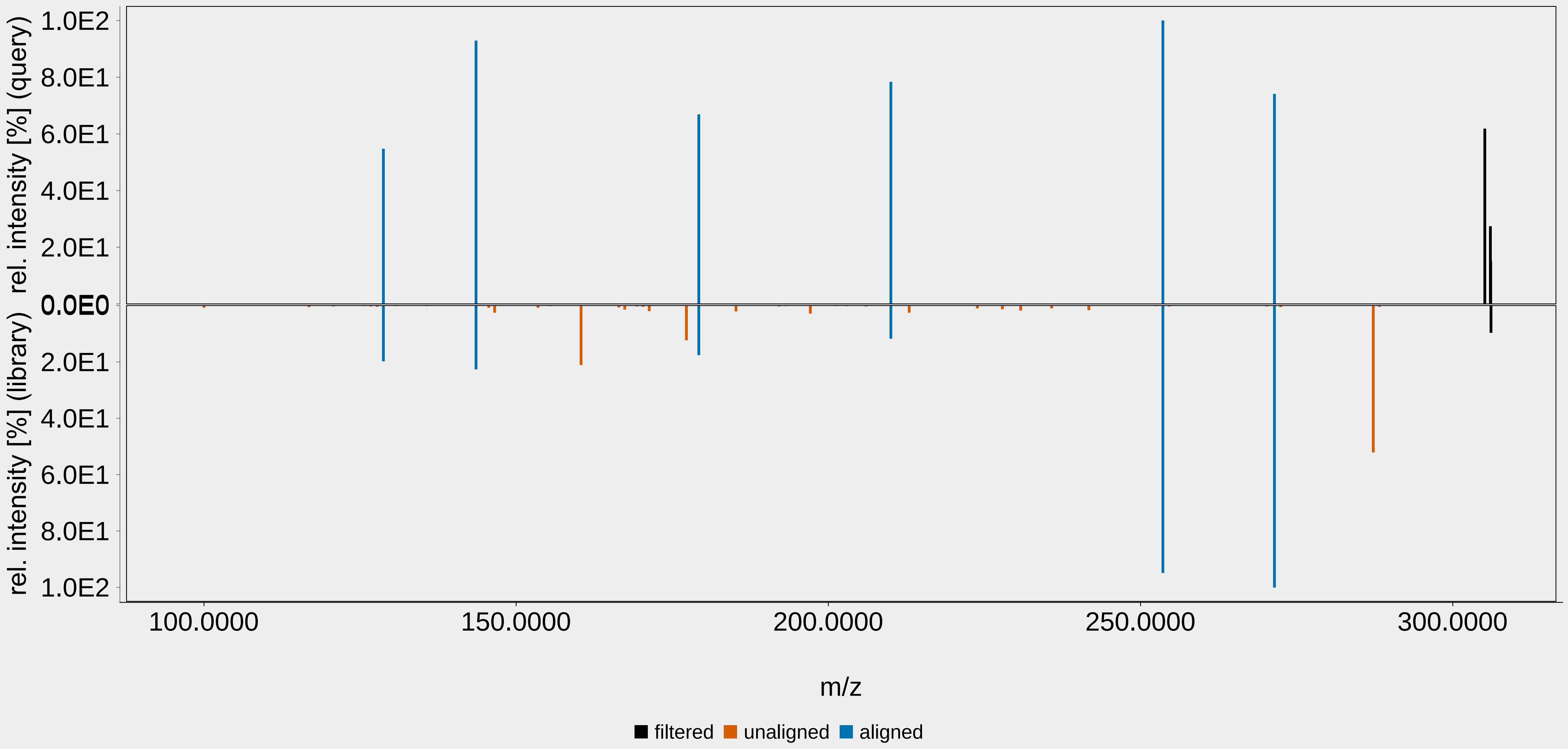

Compound information

Name: Glutathione - 30.0 eV

Exact mass: 0.0

Ion type: M-H

Precursor m/z: 306.077

Charge: 1

Polarity: Negative

SMILES:

Instrument information

Instrument type: Orbitrap

Ion source: ESI

Ms level: 2

Acquisition: Isolated

Database links

CAS:

Other information

Principal investigator: PI

Data collector: Data Collector

Entry ID: CCMSLIB00006121887

Glutathione - 30.0 eV

0.776

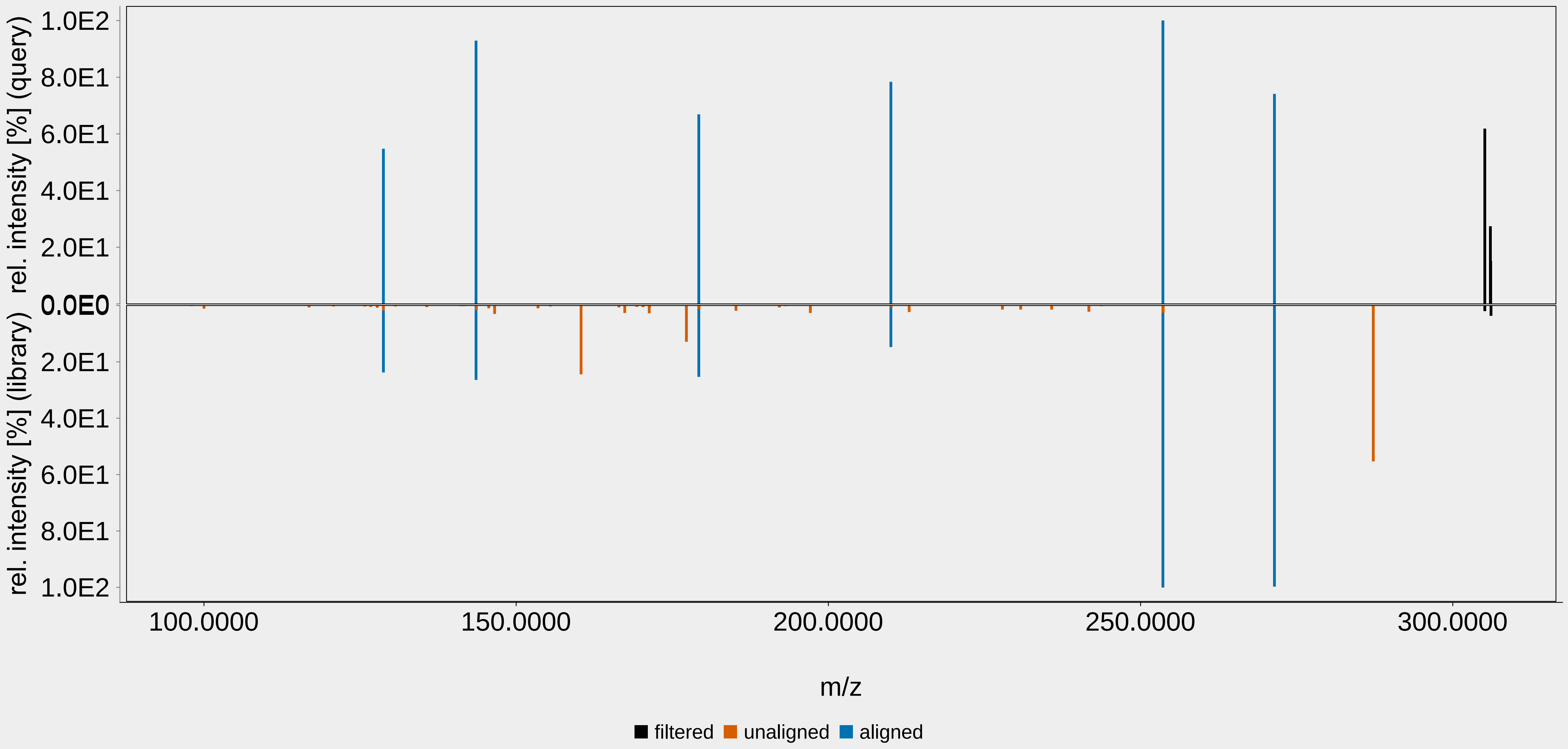

Compound information

Name: Glutathione - 30.0 eV

Exact mass: 0.0

Ion type: M-H

Precursor m/z: 306.077

Charge: 1

Polarity: Negative

SMILES:

Instrument information

Instrument type: Orbitrap

Ion source: ESI

Ms level: 2

Acquisition: Isolated

Database links

CAS:

Other information

Principal investigator: PI

Data collector: Data Collector

Entry ID: CCMSLIB00006121879

PS 24:0_18:1 **0.866**

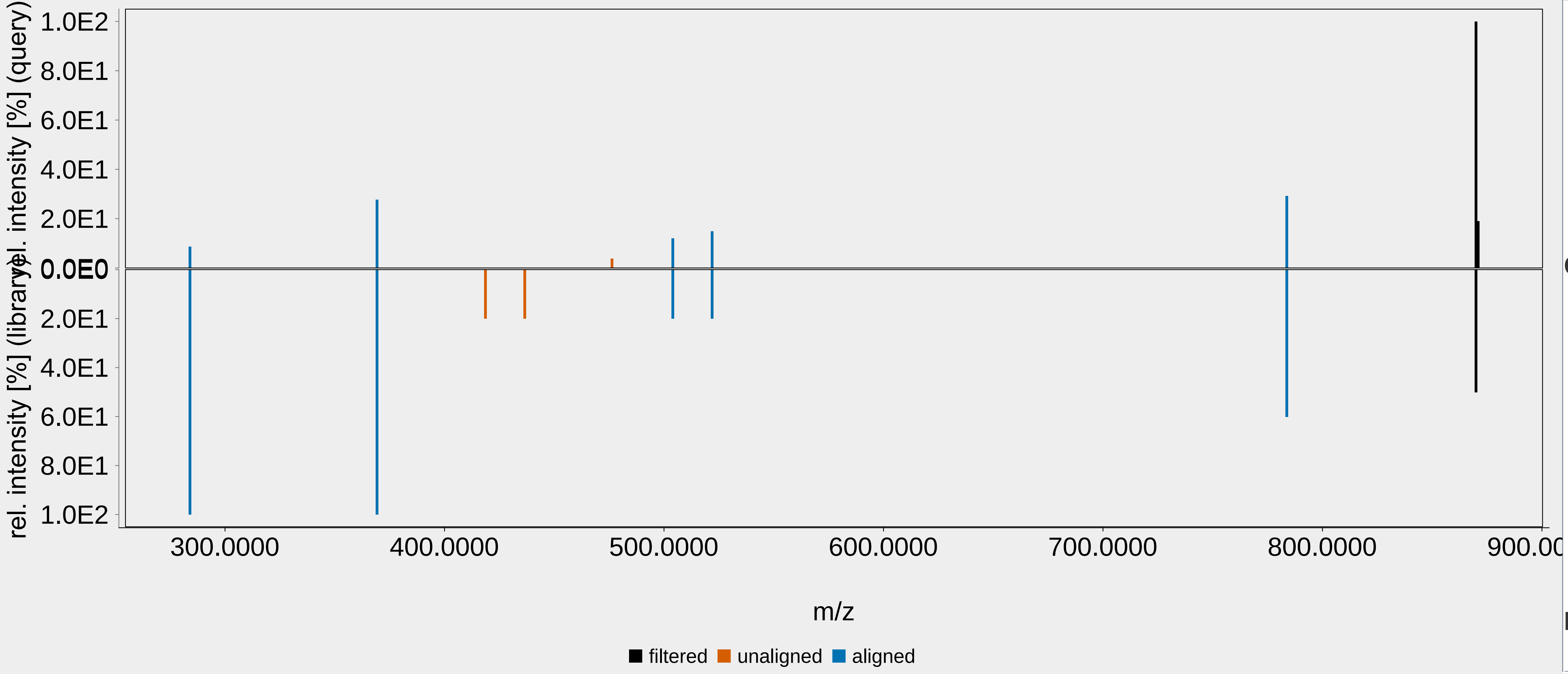

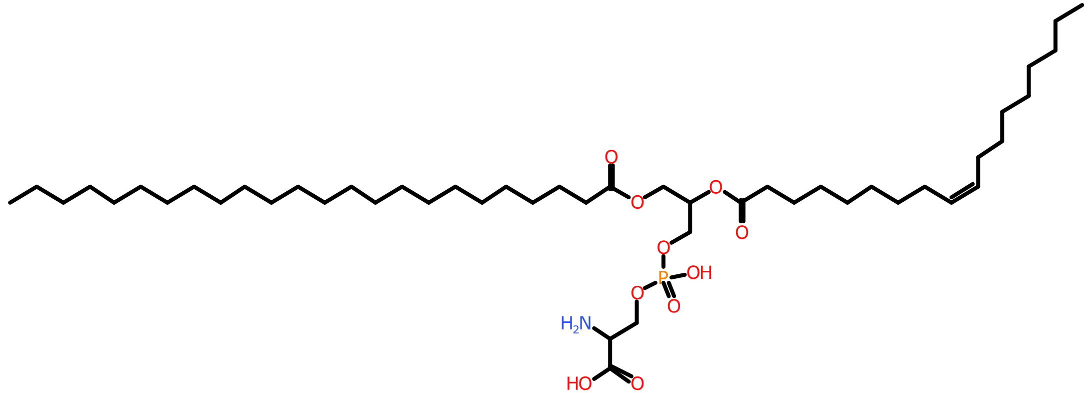

Compound information

Name: PS 24:0_18:1

Formula: C48H92NO10P

Ion type: [M-H]-

Precursor m/z: 872.63861

Ccs: 294.58

InChI key:

SMILES:

Num peaks: 8

Instrument information

Database links

Other information

PE(16:0/22:6); [M-H]- C43H73N1O8P1 **0.944**

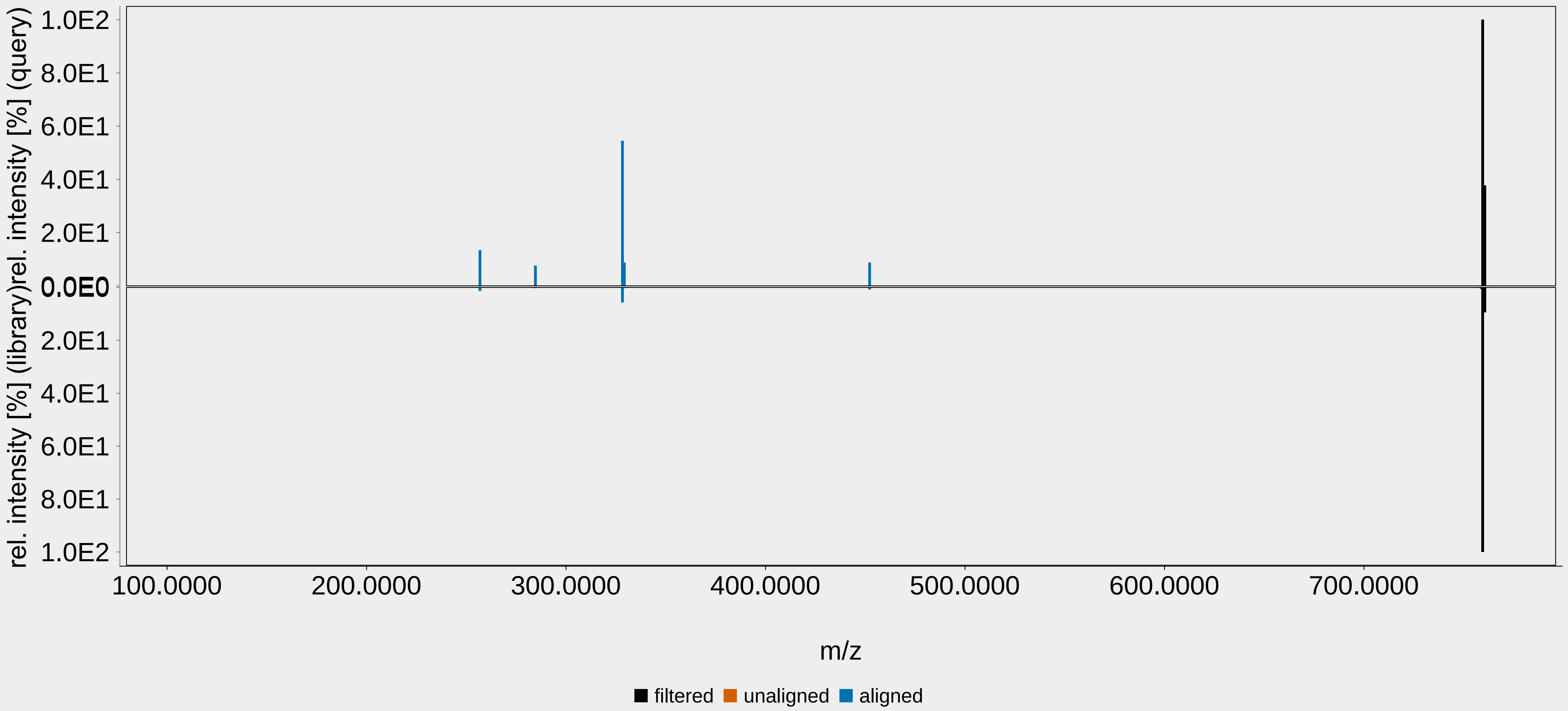

Compound information

Name: PE(16:0/22:6); [M-H]- C43H73N1O8P1

Exact mass: 0.0

Ion type: M-H

Precursor m/z: 762.507

Charge: 1

Polarity: Negative

Instrument information

Instrument type: HCD; Velos

Ion source: LC-ESI

Ms level: 2

Acquisition: Commercial

Database links

Other information

Principal investigator: Thomas Metz

Data collector: Thomas Metz

Entry ID: CCMSLIB00010088316

PE(16:0/22:6); [M-H]- C43H73N1O8P1 **0.940**

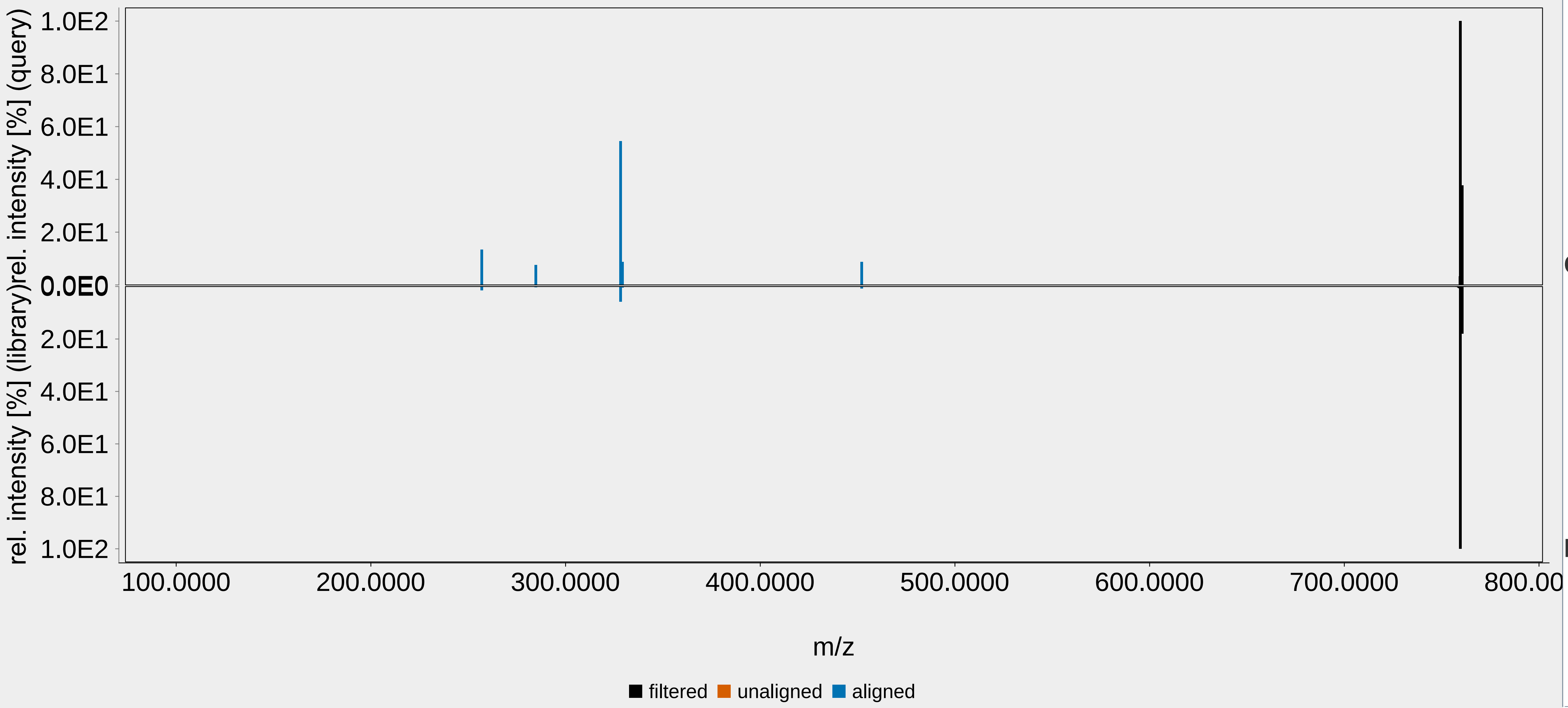

Compound information

Name: PE(16:0/22:6); [M-H]- C43H73N1O8P1

Exact mass: 0.0

Ion type: M-H

Precursor m/z: 762.507

Charge: 1

Polarity: Negative

Instrument information

Instrument type: HCD; Velos

Ion source: LC-ESI

Ms level: 2

Acquisition: Commercial

Database links

Other information

Principal investigator: Thomas Metz

Data collector: Thomas Metz

Entry ID: CCMSLIB00010064176

LPI 22:6 **0.830**

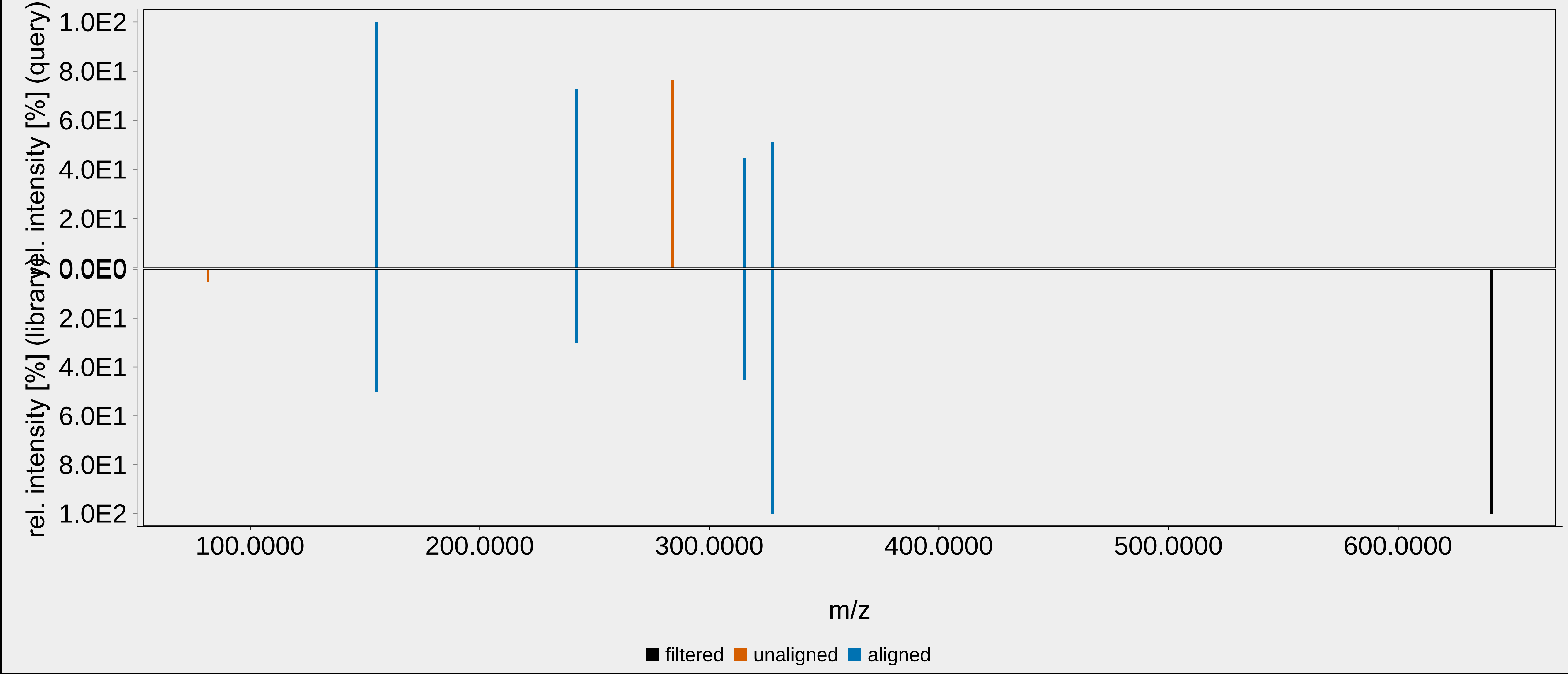

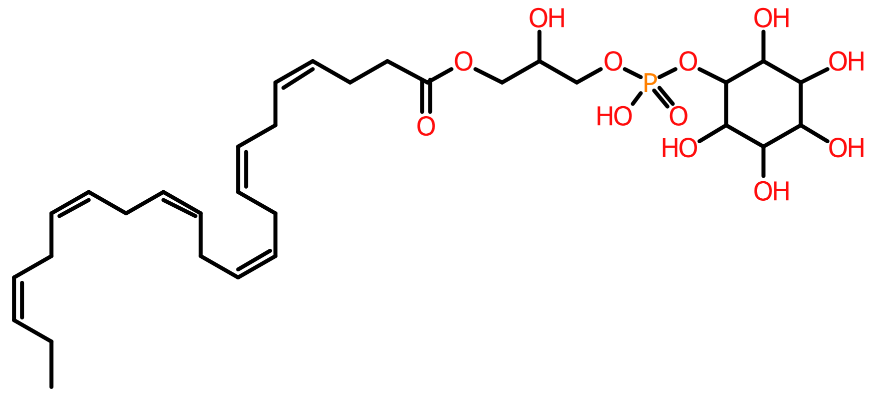

Compound information

Name: LPI 22:6

Formula: C31H49O12P

Ion type: [M-H]-

Precursor m/z: 643.28889

Ccs: 237.14

InChI key:

SMILES:

Num peaks: 6

Instrument information

Database links

Other information

PE(22:4/0:0); [M-H]- C27H47N1O7P1

0.893

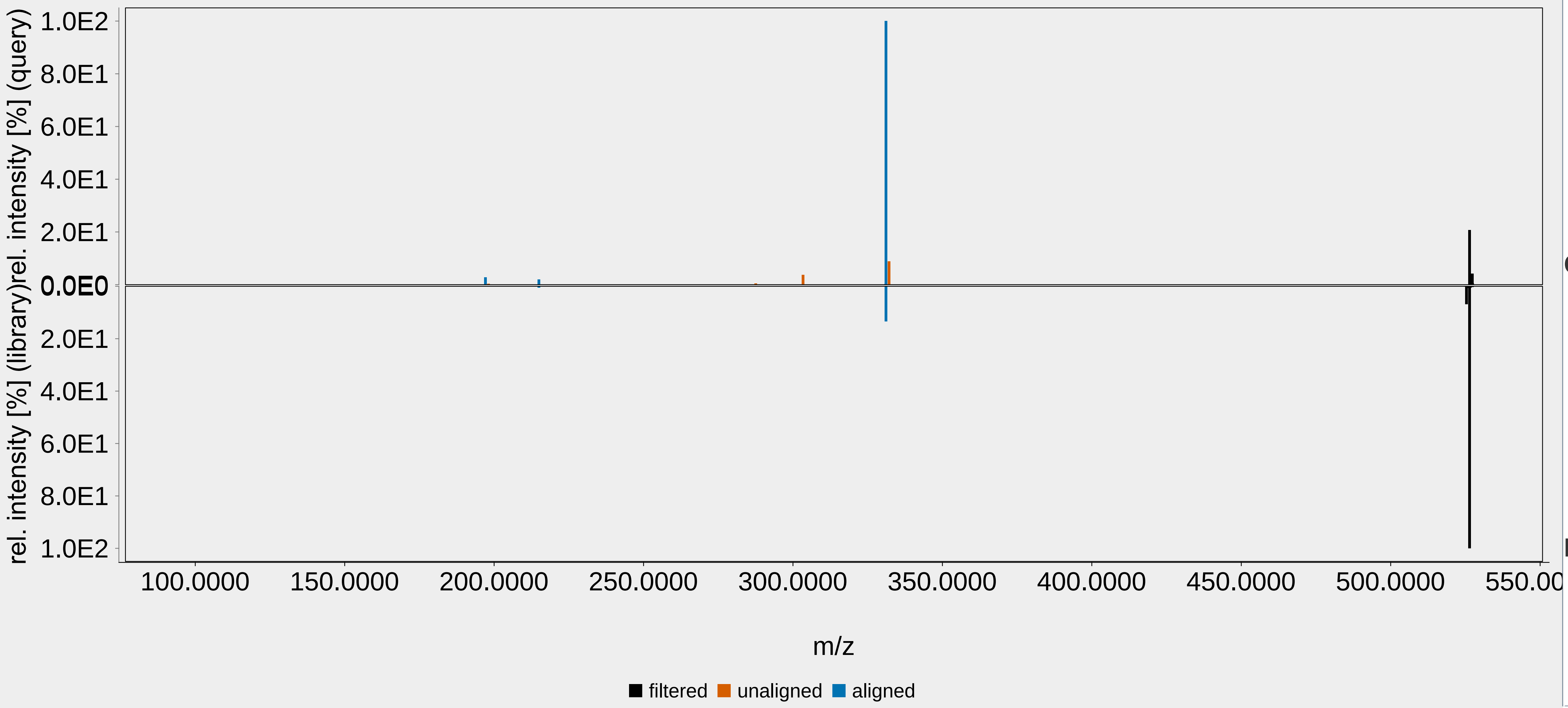

Compound information

Name: PE(22:4/0:0); [M-H]- C27H47N1O7P1

Exact mass: 0.0

Ion type: M-H

Precursor m/z: 528.309

Charge: 1

Polarity: Negative

Instrument information

Instrument type: HCD; Velos

Ion source: LC-ESI

Ms level: 2

Acquisition: Commercial

Database links

Other information

Principal investigator: Thomas Metz

Data collector: Thomas Metz

Entry ID: CCMSLIB00010086739

PE(22:4/0:0); [M-H]- C27H47N1O7P1 **0.897**

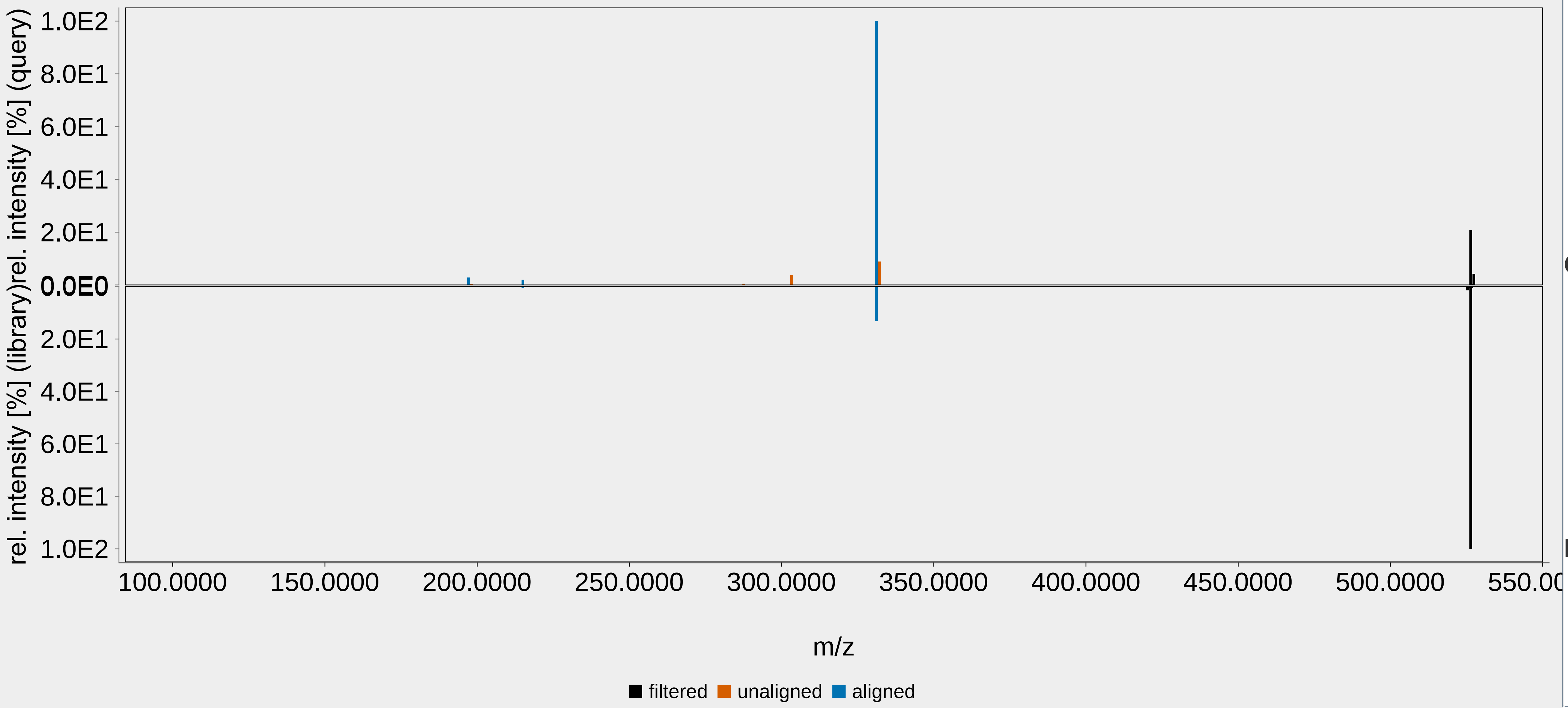

Compound information

Name: PE(22:4/0:0); [M-H]- C27H47N1O7P1

Exact mass: 0.0

Ion type: M-H

Precursor m/z: 528.309

Charge: 1

Polarity: Negative

Instrument information

Instrument type: HCD; Velos

Ion source: LC-ESI

Ms level: 2

Acquisition: Commercial

Database links

Other information

Principal investigator: Thomas Metz

Data collector: Thomas Metz

Entry ID: CCMSLIB00010063574

PC 38:6

0.734

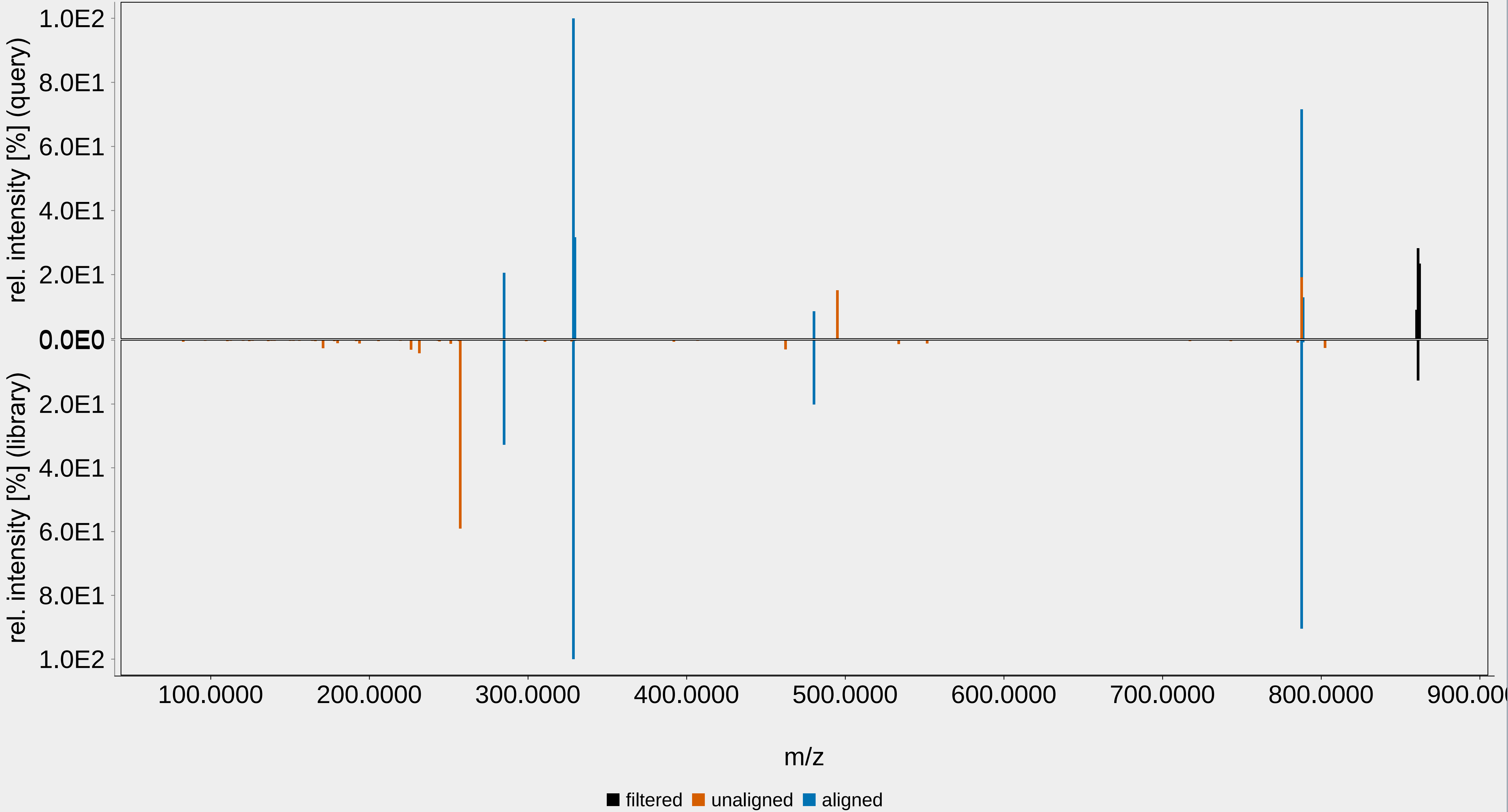

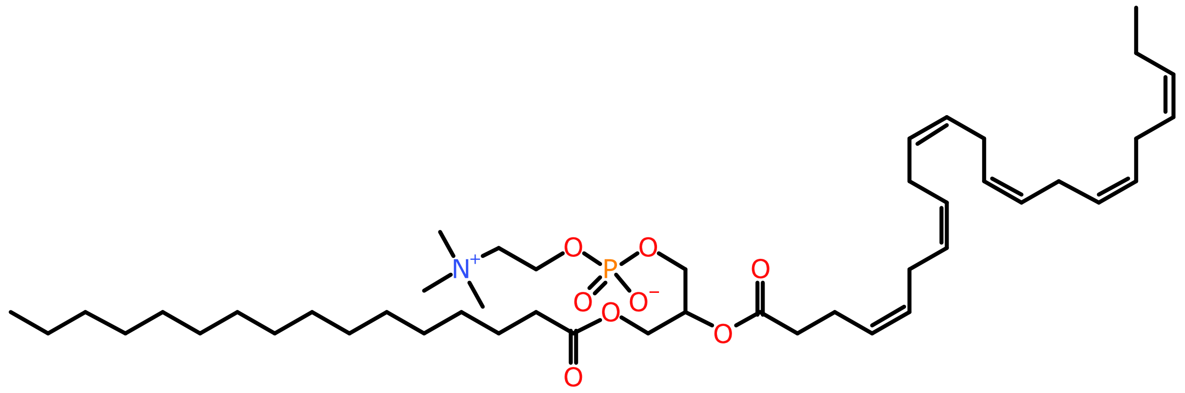

Compound information

Name: PC 38:6

Formula: C46H80NO8P

Mol. weight: 806.1209999999999

Exact mass: 806.1209999999999

Ion type: [M+CH3COOH-H]-

Precursor m/z: 864.5729

RT: 9.202155

Polarity: negative

InChI:

InChI key:

SMILES:

Instrument information

Instrument type: LC-ESI-QTOF

Instrument: AB Sciex TripleTOF 5600+ system

Ms level: MS2

Collision energy: 45V

Database links

Pubchem: 9940652

MoNA ID: LQB00502

Other information

Principal investigator: Kazutaka Ikeda, Hiroshi

Data collector: Kazutaka Ikeda, Hiroshi

PC 38:6

0.734

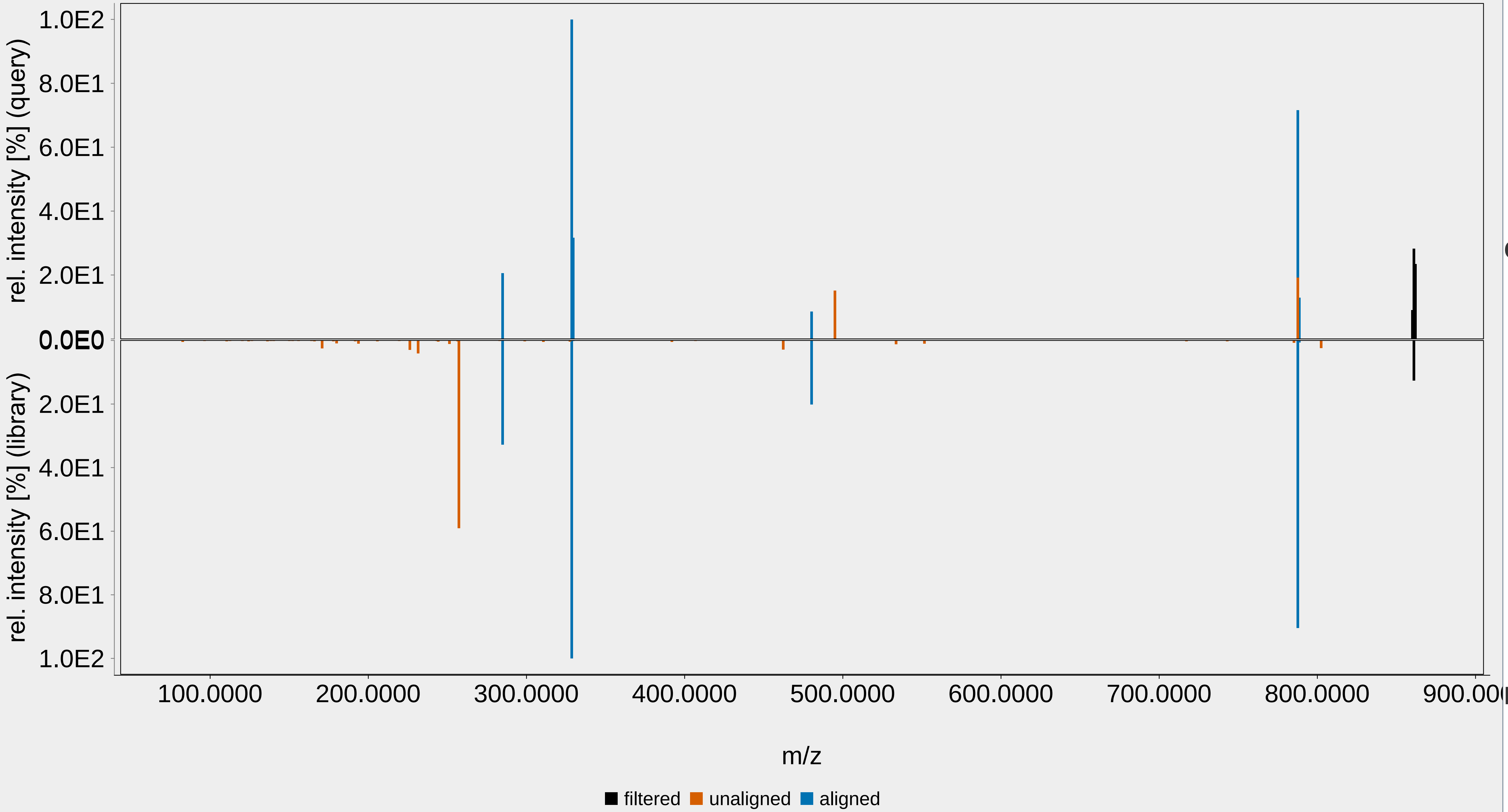

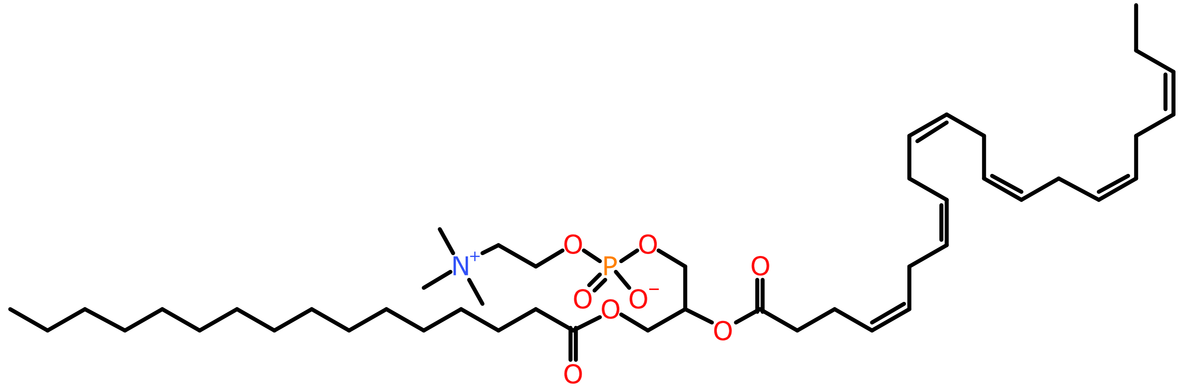

Compound information

Name: PC 38:6

Formula: C46H80NO8P

Mol. weight: 806.1209999999999

Exact mass: 806.1209999999999

Ion type: [M+CH3COOH-H]-

Precursor m/z: 864.5729

RT: 9.202155

Polarity: negative

InChI:

InChI key:

SMILES:

Instrument information

Instrument type: LC-ESI-QTOF

Instrument: AB Sciex TripleTOF 5600+ system

Ms level: MS2

Collision energy: 45V

Database links

Pubchem: 9940652

MoNA ID: LQB00116

Other information

Principal investigator: Kazutaka Ikeda, Hiroshi

Data collector: Kazutaka Ikeda, Hiroshi

PS(18:1_0:0) - 2-amino-3-[hydroxy-[2-hydroxy-3-[octadec-9-enoyl]oxypropoxy]phosphoryl]oxypropanoic acid

0.839

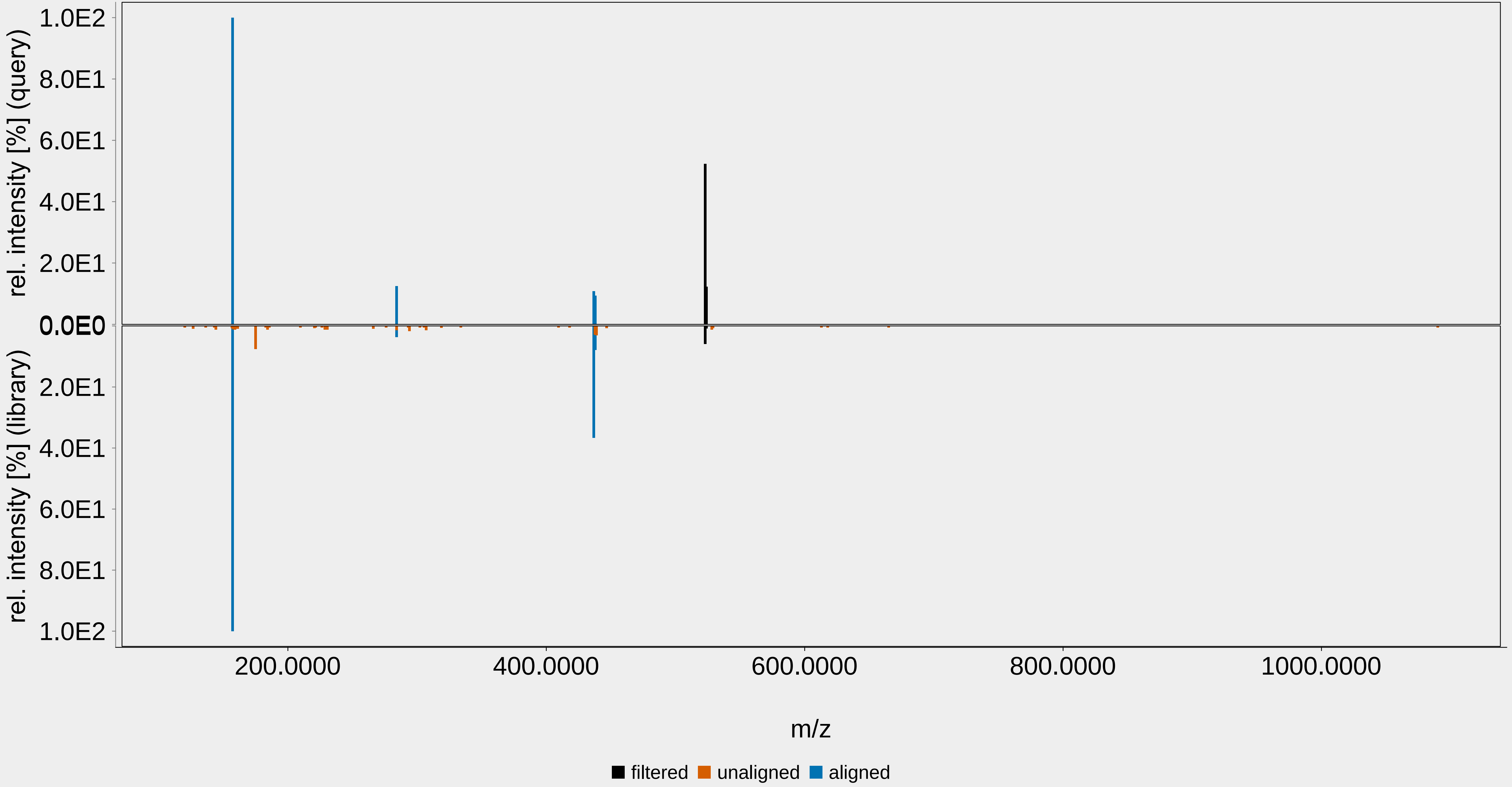

Compound information

Name: PS(18:1_0:0) -

Exact mass: 523.291

Ion type: M-H

Precursor m/z: 522.283

Charge: 1

Polarity: Negative

InChI:

InChI key:

SMILES:

Instrument information

Instrument type: qTof

Ion source: LC-ESI

Ms level: 2

Acquisition: Commercial

Database links

Pubmed:

CAS: 326589-90-6

Other information

Principal investigator: Laprevote

Data collector: UMR 8038 Universite de Paris

Entry ID: CCMSLIB00005436456

2-amino-3-[hydroxy-[2-hydroxy-3-[octadec-9-enoyl]oxypropoxy]phosphoryl]oxypropanoic acid

0.839

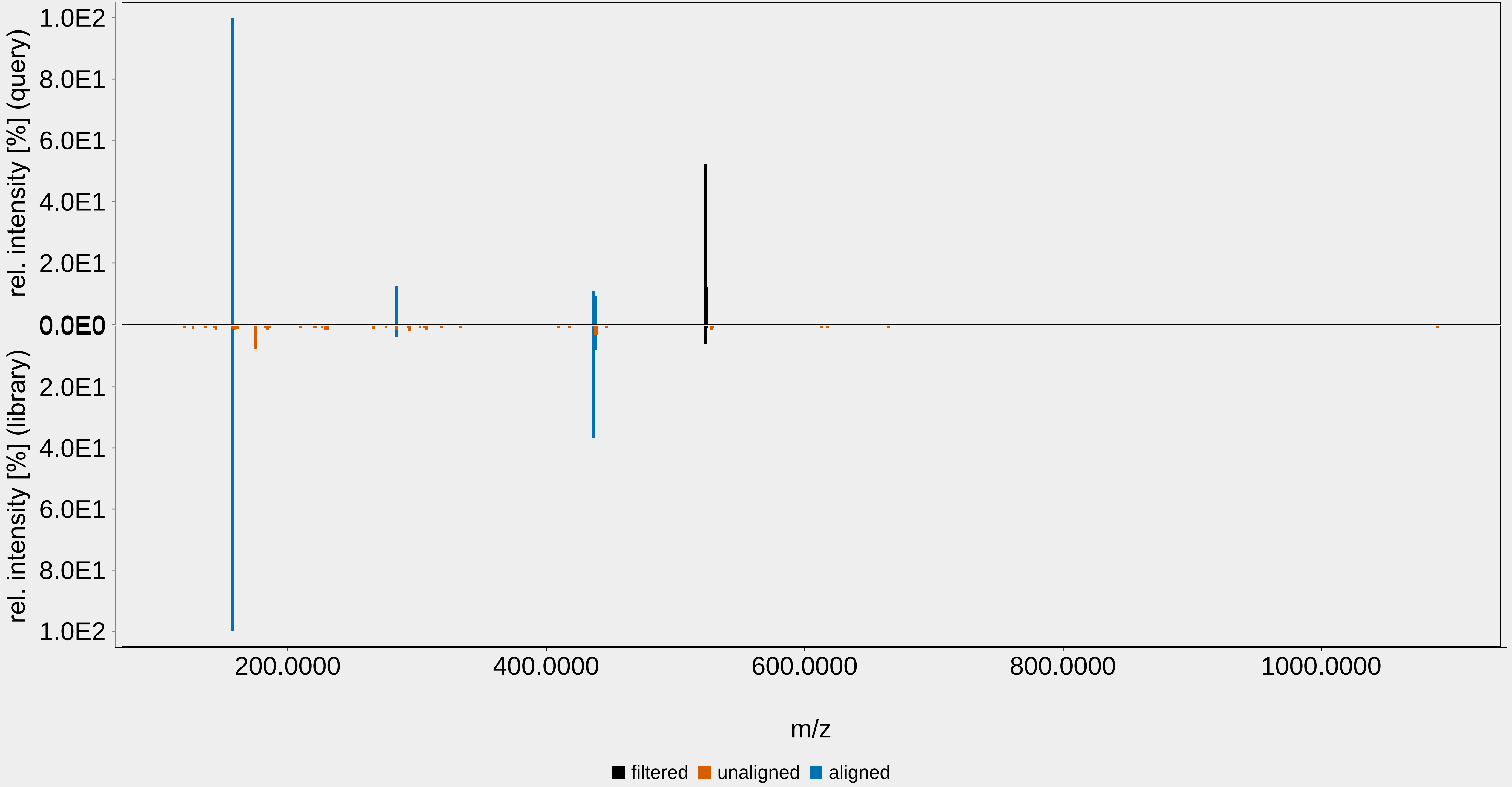

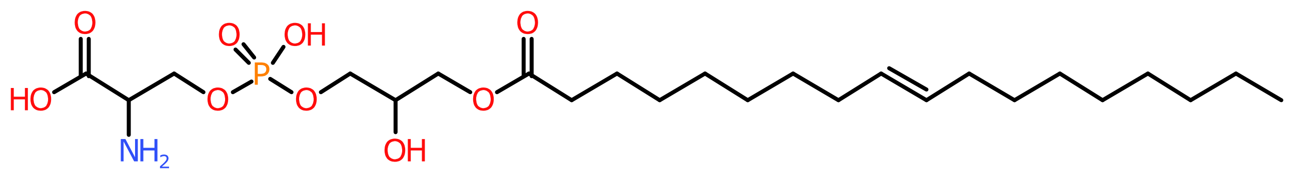

Compound information

Name:

Formula: C24H46NO9P

Mol. weight: 523.291

Exact mass: 523.291

Ion type: [M-H]-

Precursor m/z: 522.283

Polarity: negative

InChI:

InChI key:

SMILES:

Instrument information

Instrument: qTof

Ms level: MS2

Database links

Other information

Principal investigator: Laprevote, UMR 8038

Data collector: Laprevote, UMR 8038 Universite

LNAPE 16:0/N-18:0

0.906

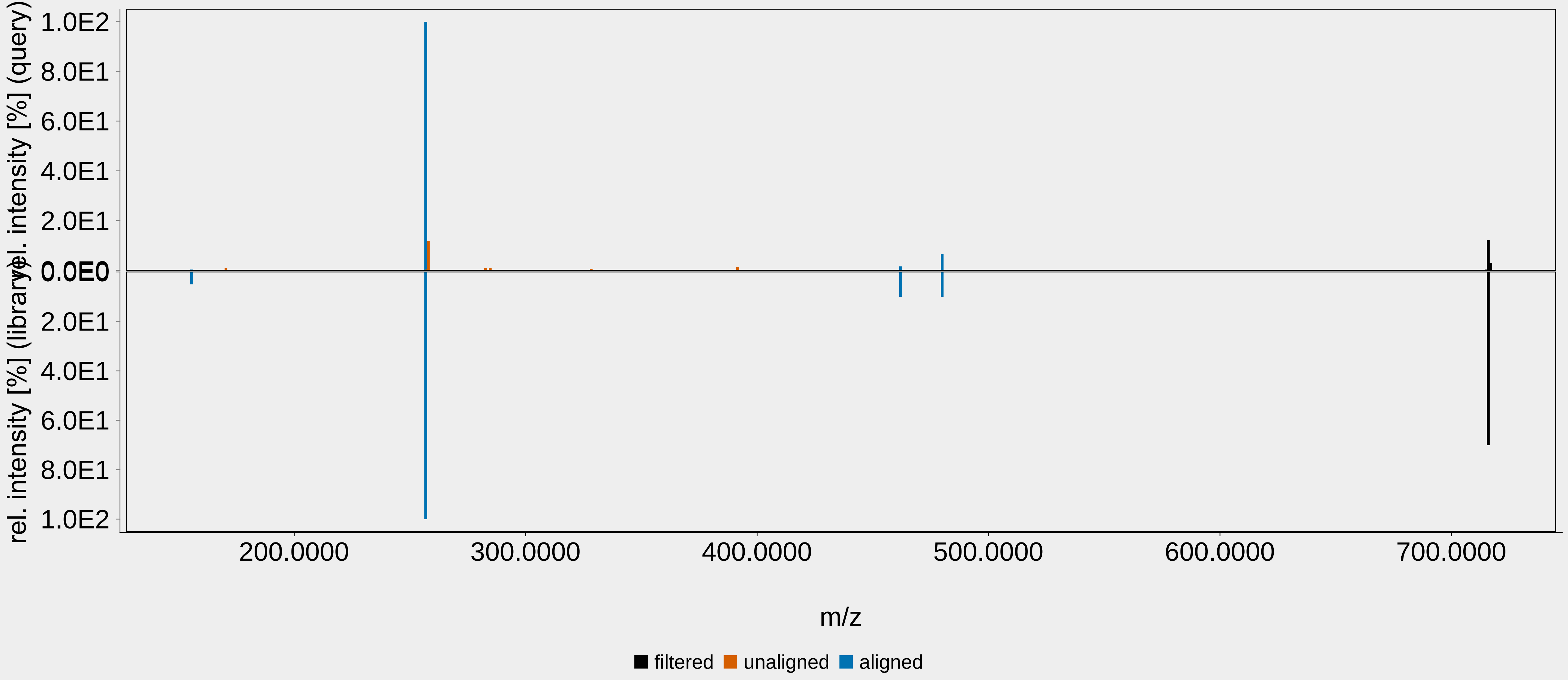

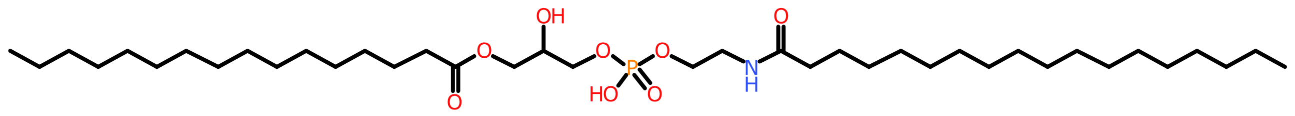

Compound information

Name: LNAPE 16:0/N-18:0

Formula: C39H78NO8P

Ion type: [M-H]-

Precursor m/z: 718.53923

Ccs: 265.73

InChI key:

SMILES:

Num peaks: 5

Instrument information

Database links

Other information

PE(16:0/18:0); [M-H]- C39H77N1O8P1 0.945

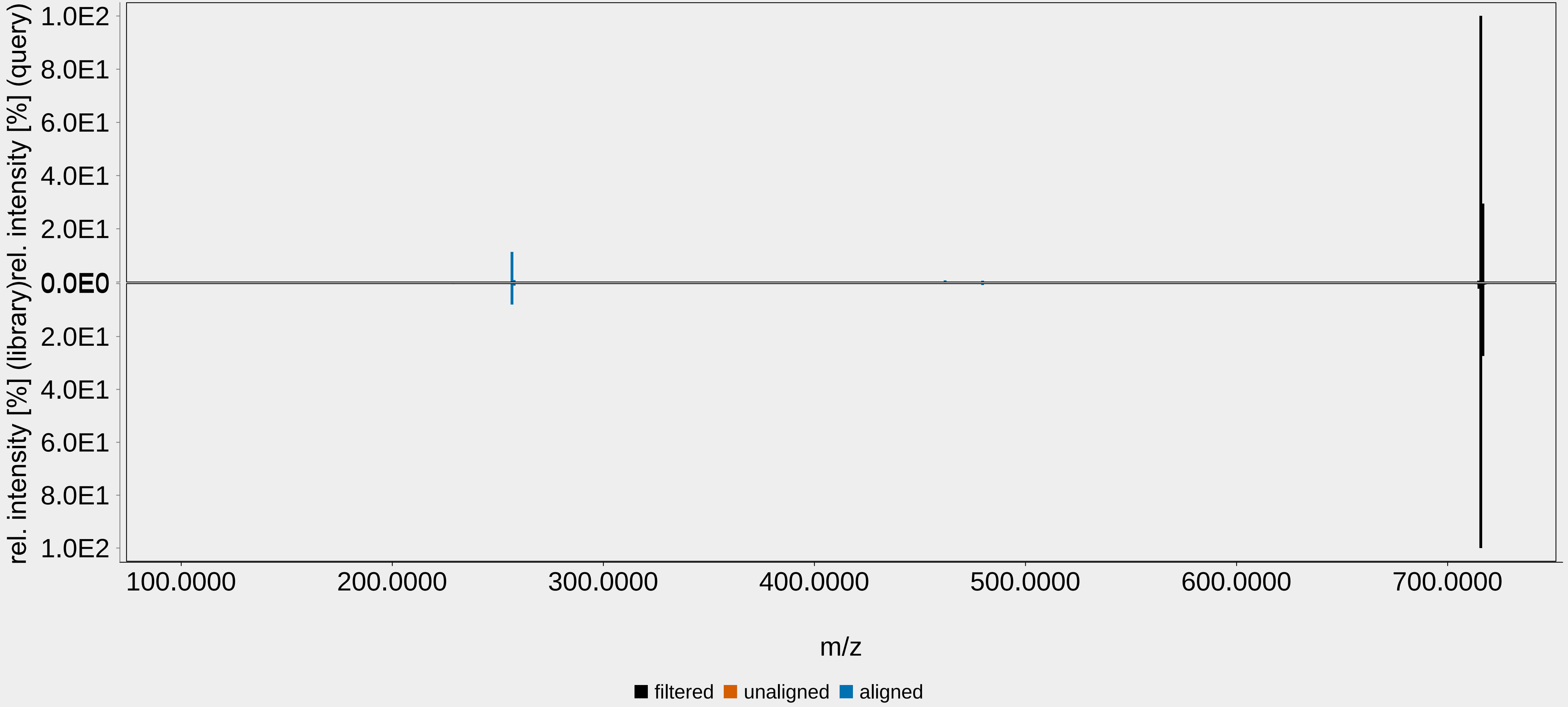

Compound information

Name: PE(16:0/18:0); [M-H]- C39H77N1O8P1

Exact mass: 0.0

Ion type: M-H

Precursor m/z: 718.539

Charge: 1

Polarity: Negative

Instrument information

Instrument type: HCD; Velos

Ion source: LC-ESI

Ms level: 2

Acquisition: Commercial

Database links

Other information

Principal investigator: Thomas Metz

Data collector: Thomas Metz

Entry ID: CCMSLIB00010073693

PE(18:1/22:0); [M-H]- C45H87N1O8P1

0.729

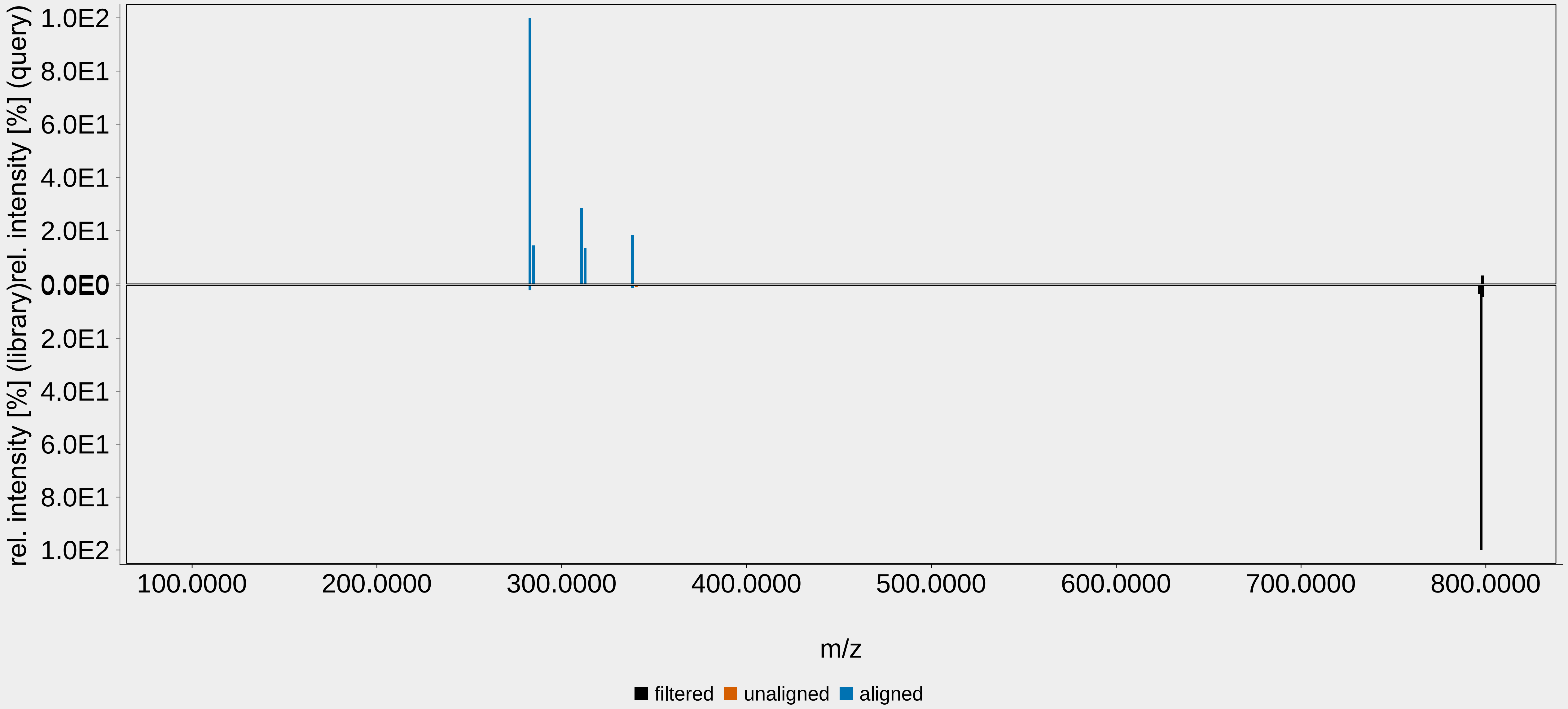

Compound information

Name: PE(18:1/22:0); [M-H]- C45H87N1O8P1

Exact mass: 0.0

Ion type: M-H

Precursor m/z: 800.617

Charge: 1

Polarity: Negative

Instrument information

Instrument type: HCD; Velos

Ion source: LC-ESI

Ms level: 2

Acquisition: Commercial

Database links

Other information

Principal investigator: Thomas Metz

Data collector: Thomas Metz

Entry ID: CCMSLIB00010074098

PE(18:1/22:0); [M-H]- C45H87N1O8P1 0.785

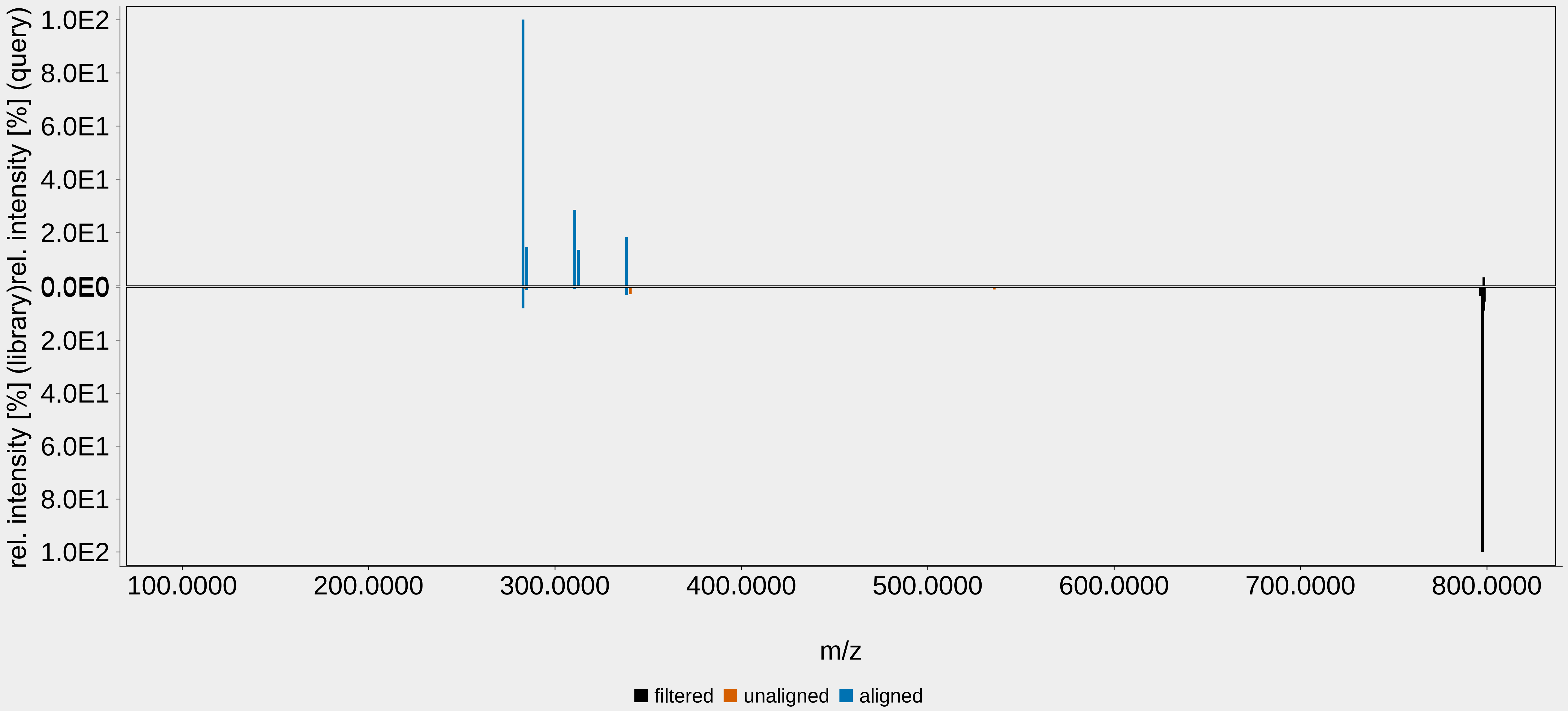

Compound information

Name: PE(18:1/22:0); [M-H]- C45H87N1O8P1

Exact mass: 0.0

Ion type: M-H

Precursor m/z: 800.617

Charge: 1

Polarity: Negative

Instrument information

Instrument type: HCD; Velos

Ion source: LC-ESI

Ms level: 2

Acquisition: Commercial

Database links

Other information

Principal investigator: Thomas Metz

Data collector: Thomas Metz

Entry ID: CCMSLIB00010087976

PE(18:0/22:4); [M-H]- C45H81N1O8P1 **0.864**

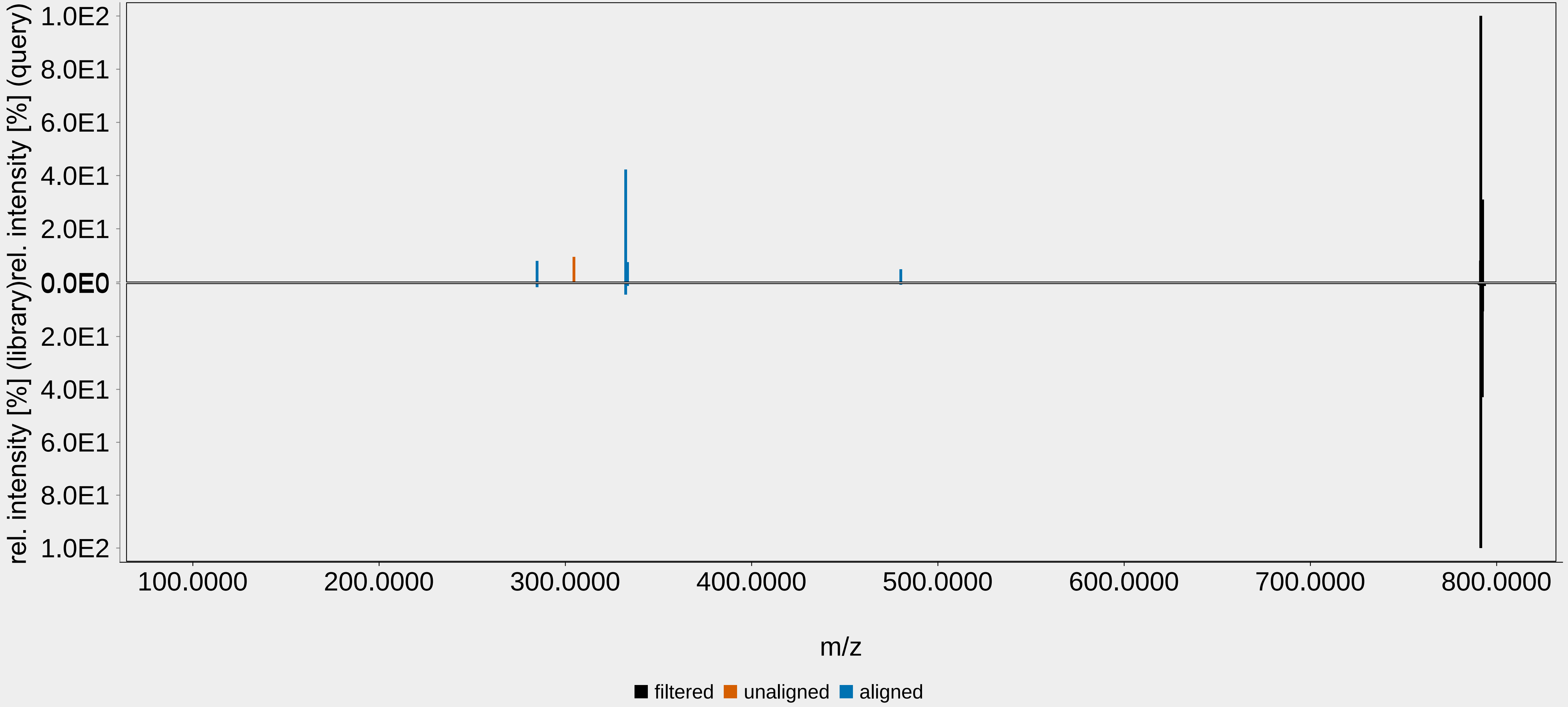

Compound information

Name: PE(18:0/22:4); [M-H]- C45H81N1O8P1

Exact mass: 0.0

Ion type: M-H

Precursor m/z: 794.57

Charge: 1

Polarity: Negative

Instrument information

Instrument type: HCD; Velos

Ion source: LC-ESI

Ms level: 2

Acquisition: Commercial

Database links

Other information

Principal investigator: Thomas Metz

Data collector: Thomas Metz

Entry ID: CCMSLIB00010085505

PE(18:0/22:4); [M-H]- C45H81N1O8P1 0.865

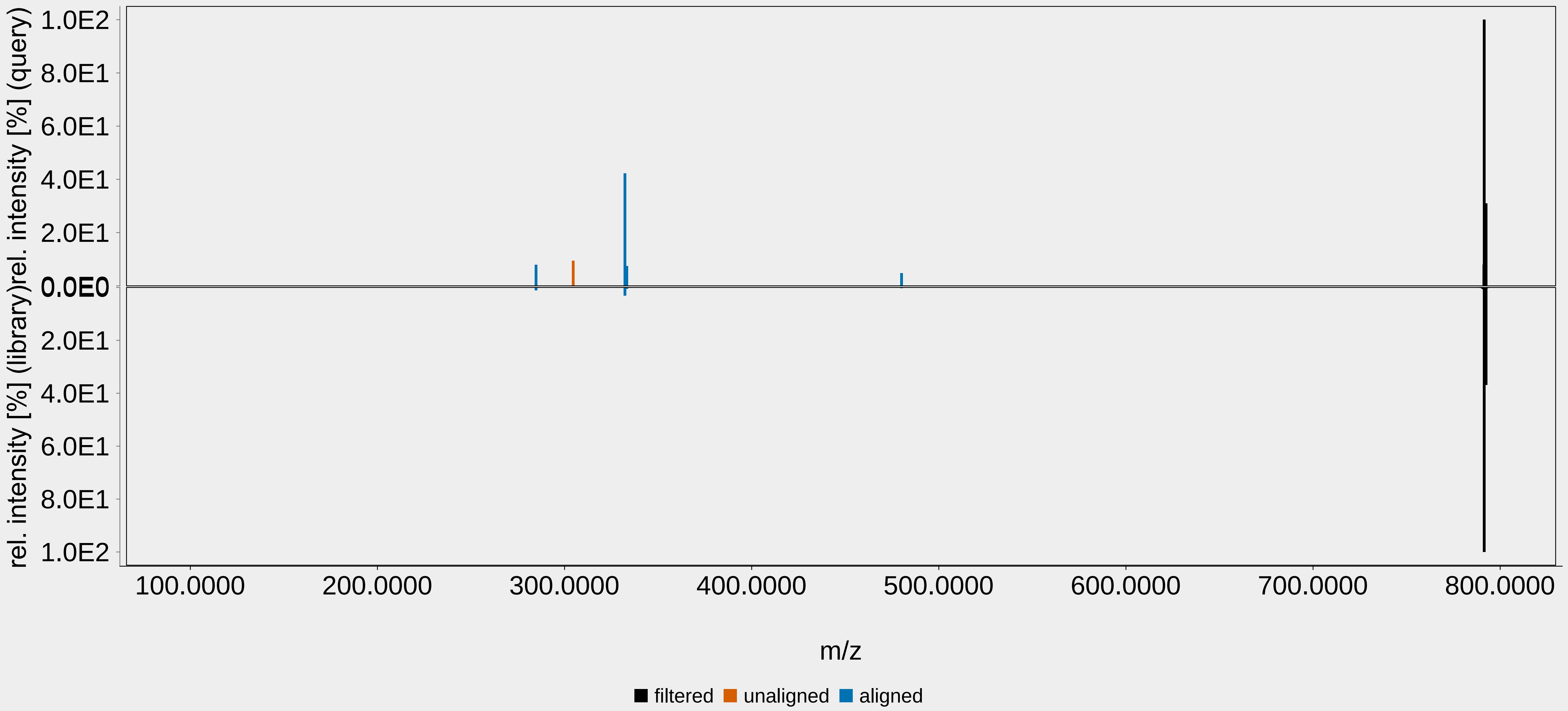

Compound information

Name: PE(18:0/22:4); [M-H]- C45H81N1O8P1

Exact mass: 0.0

Ion type: M-H

Precursor m/z: 794.57

Charge: 1

Polarity: Negative

Instrument information

Instrument type: HCD; Velos

Ion source: LC-ESI

Ms level: 2

Acquisition: Commercial

Database links

Other information

Principal investigator: Thomas Metz

Data collector: Thomas Metz

Entry ID: CCMSLIB00010083860

PS 38:4

0.855

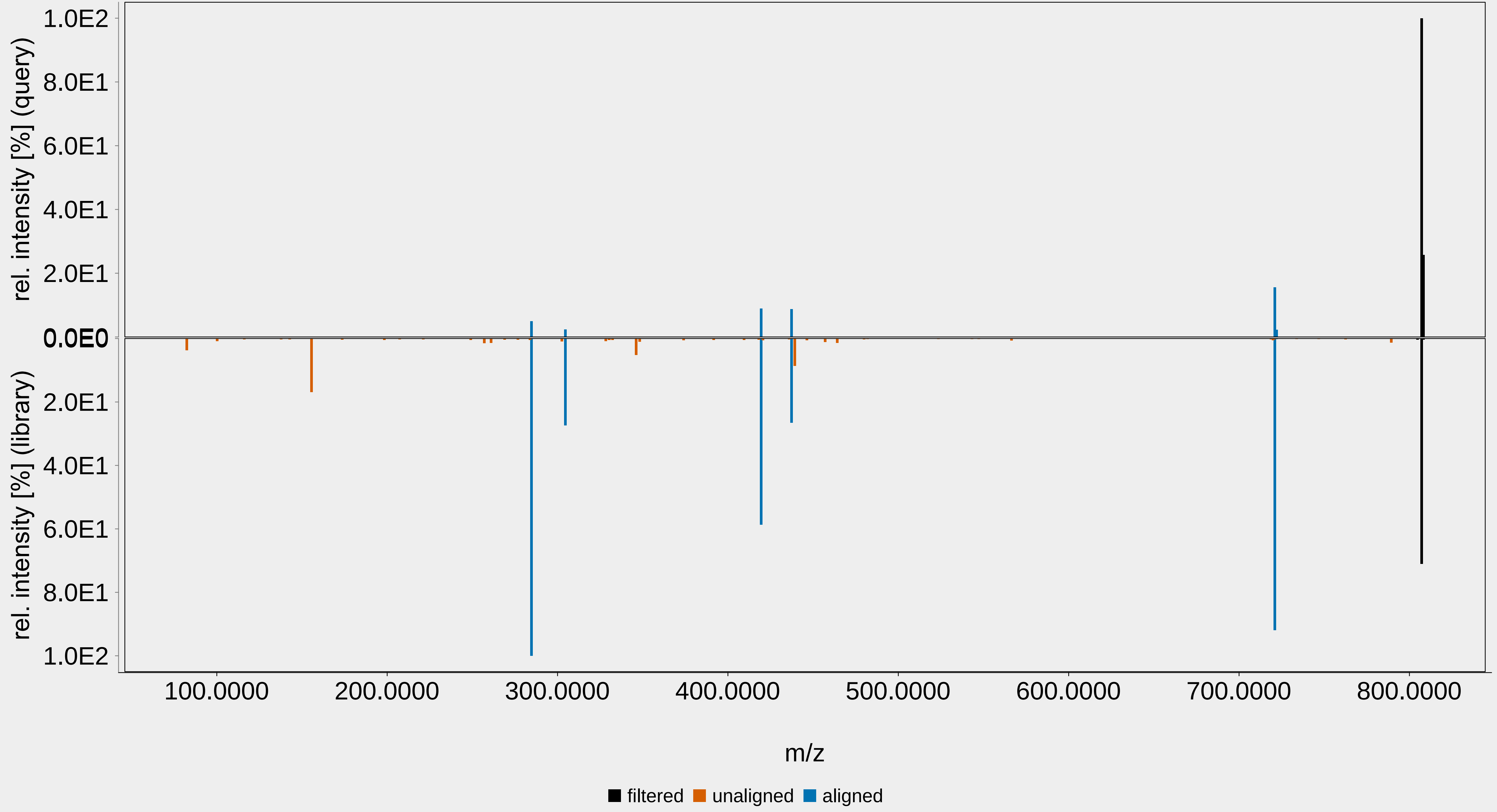

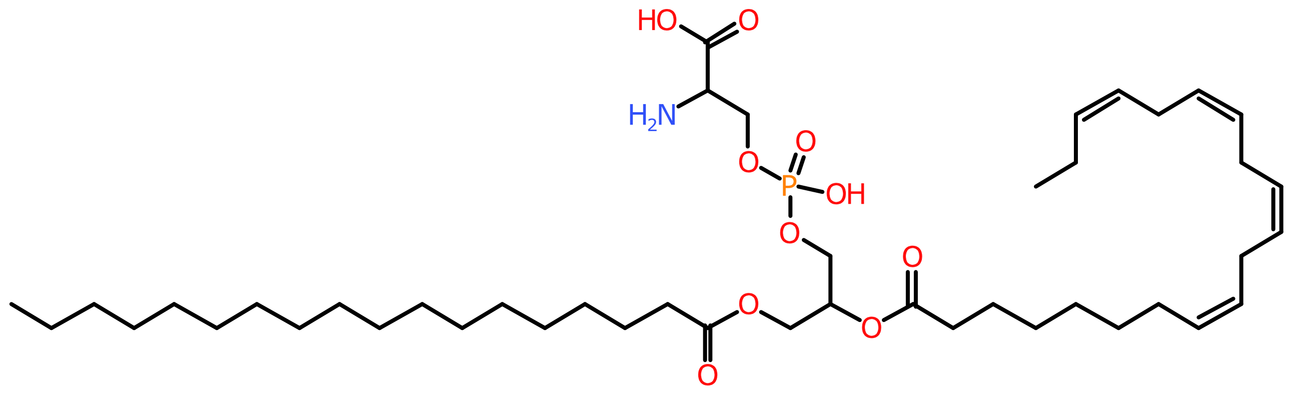

Compound information

Name: PS 38:4

Formula: C44H78NO10P

Mol. weight: 812.0809999999999

Exact mass: 812.0809999999999

Ion type: [M-H]-

Precursor m/z: 810.5303

RT: 8.778674

Polarity: negative

InChI:

InChI key:

SMILES:

Instrument information

Instrument type: LC-ESI-QTOF

Instrument: AB Sciex TripleTOF 5600+ system

Ms level: MS2

Collision energy: 45V

Database links

Pubchem: 138181120

MoNA ID: LQB00722

Other information

Principal investigator: Kazutaka Ikeda, Hiroshi

Data collector: Kazutaka Ikeda, Hiroshi

PS 38:4 **0.855**

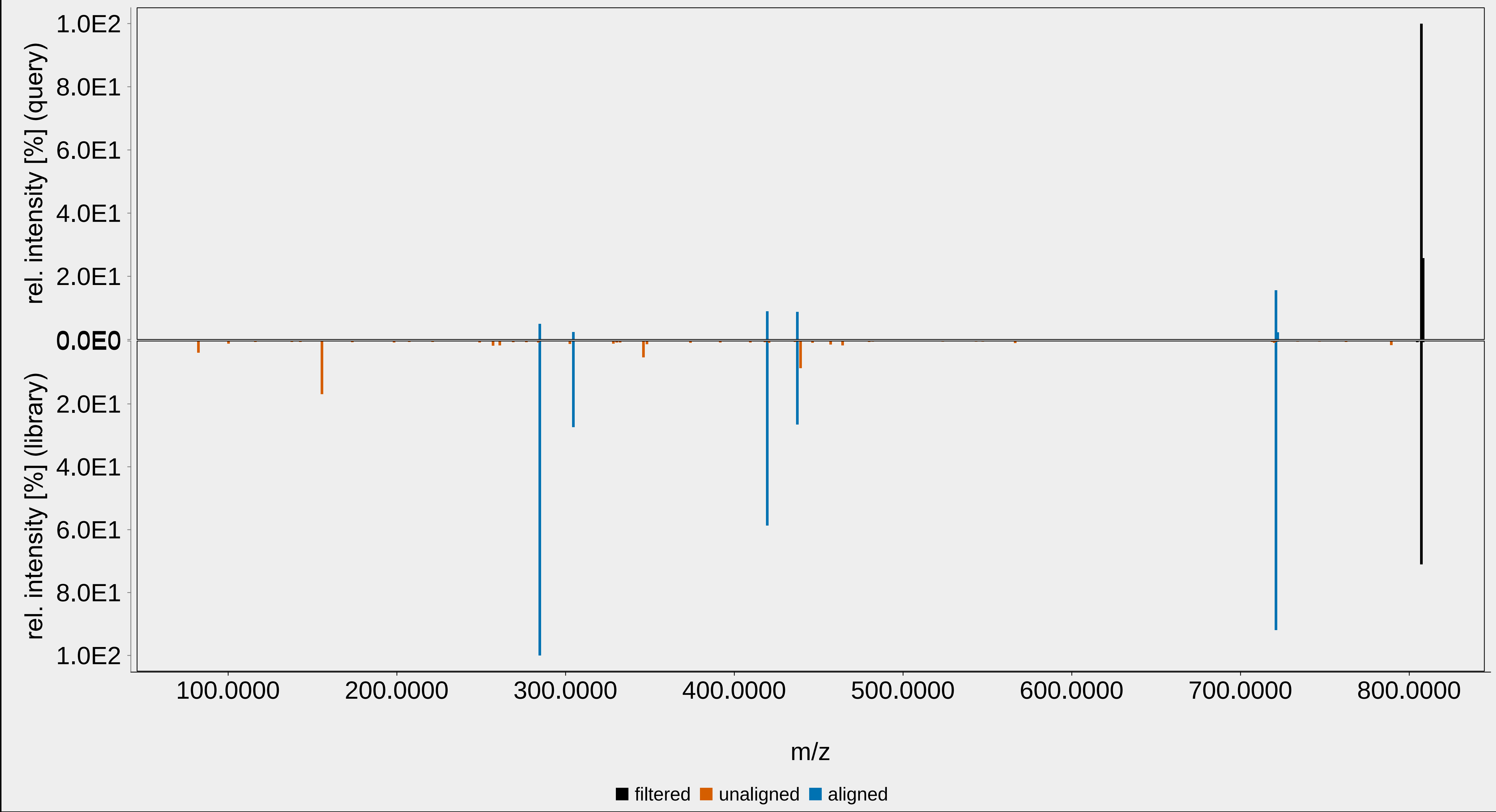

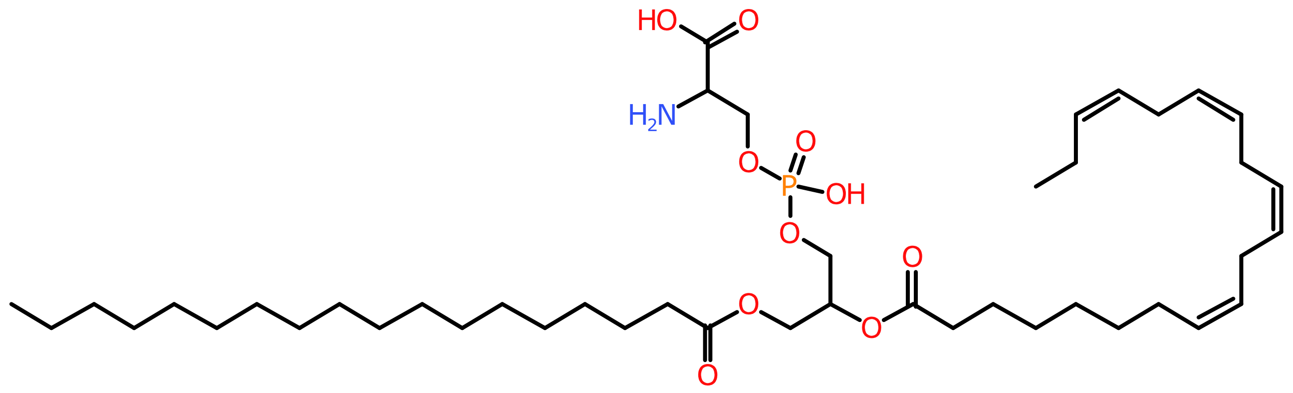

Compound information

Name: PS 38:4

Formula: C44H78NO10P

Mol. weight: 812.0809999999999

Exact mass: 812.0809999999999

Ion type: [M-H]-

Precursor m/z: 810.5303

RT: 8.778674

Polarity: negative

InChI:

InChI key:

SMILES:

Instrument information

Instrument type: LC-ESI-QTOF

Instrument: AB Sciex TripleTOF 5600+ system

Ms level: MS2

Collision energy: 45V

Database links

Pubchem: 138181120

MoNA ID: LQB00336

Other information

Principal investigator: Kazutaka Ikeda, Hiroshi

Data collector: Kazutaka Ikeda, Hiroshi

Spectral Match to cis-4,10,13,16-Docosatetraenoic acid from NIST14

0.707

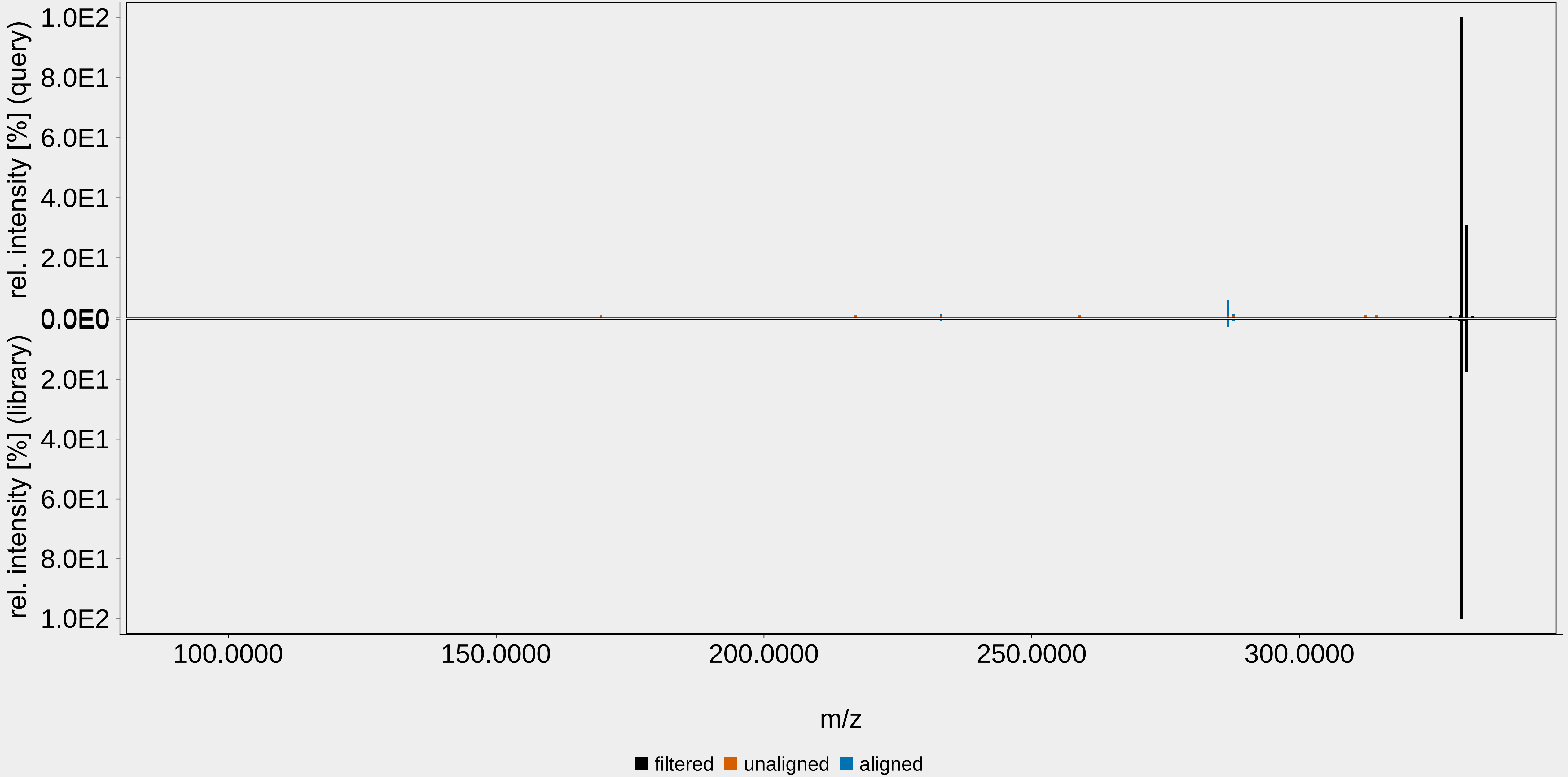

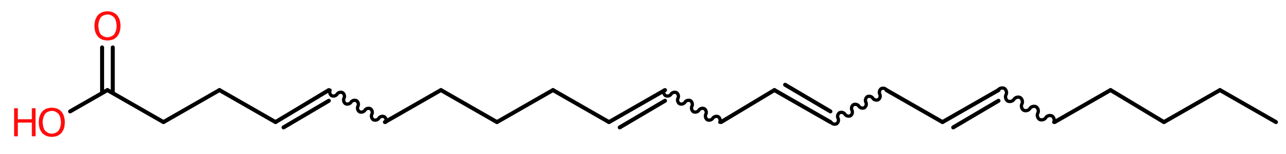

Compound information

Name: Spectral Match to

Exact mass: 332.272

Ion type: M-H

Precursor m/z: 331.263

Charge: 1

Polarity: Negative

InChI:

SMILES:

Instrument information

Instrument type: Ion Trap

Ion source: ESI

Ms level: 2

Acquisition: Isolated

Database links

CAS: 122068080

Other information

Principal investigator: Data from Charles

Data collector: Data deposited by alchemistmatt

Entry ID: CCMSLIB00003138332

PS(18:0_18:1) - 2-amino-3-({hydroxy[2-[octadec-9-enoyloxy]-3-(octadecanoyloxy)propoxy]phosphoryl}oxy)propanoic acid

0.896

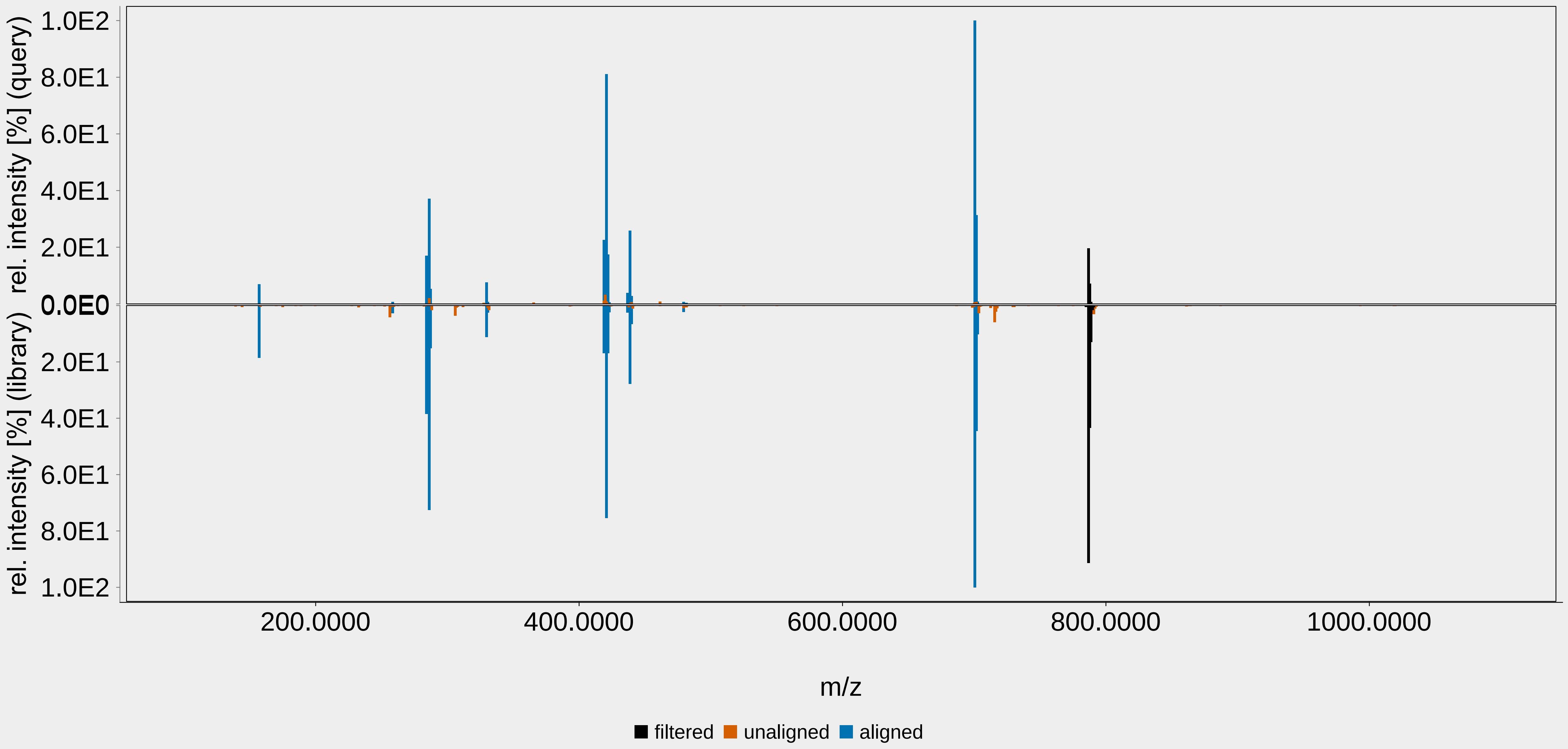

Compound information

Name: PS(18:0_18:1) -

Exact mass: 789.552

Ion type: M-H

Precursor m/z: 788.542

Charge: 1

Polarity: Negative

SMILES:

Instrument information

Instrument type: qTof

Ion source: LC-ESI

Ms level: 2

Acquisition: Lysate

Database links

CAS: 124262-93-7

Other information

Principal investigator: O Laprevote

Data collector: C-TAC

Entry ID: CCMSLIB00005720987

2-amino-3-({hydroxy[2-[octadec-9-enoyloxy]-3-(octadecanoyloxy)propoxy]phosphoryl}oxy)propanoic acid

0.896

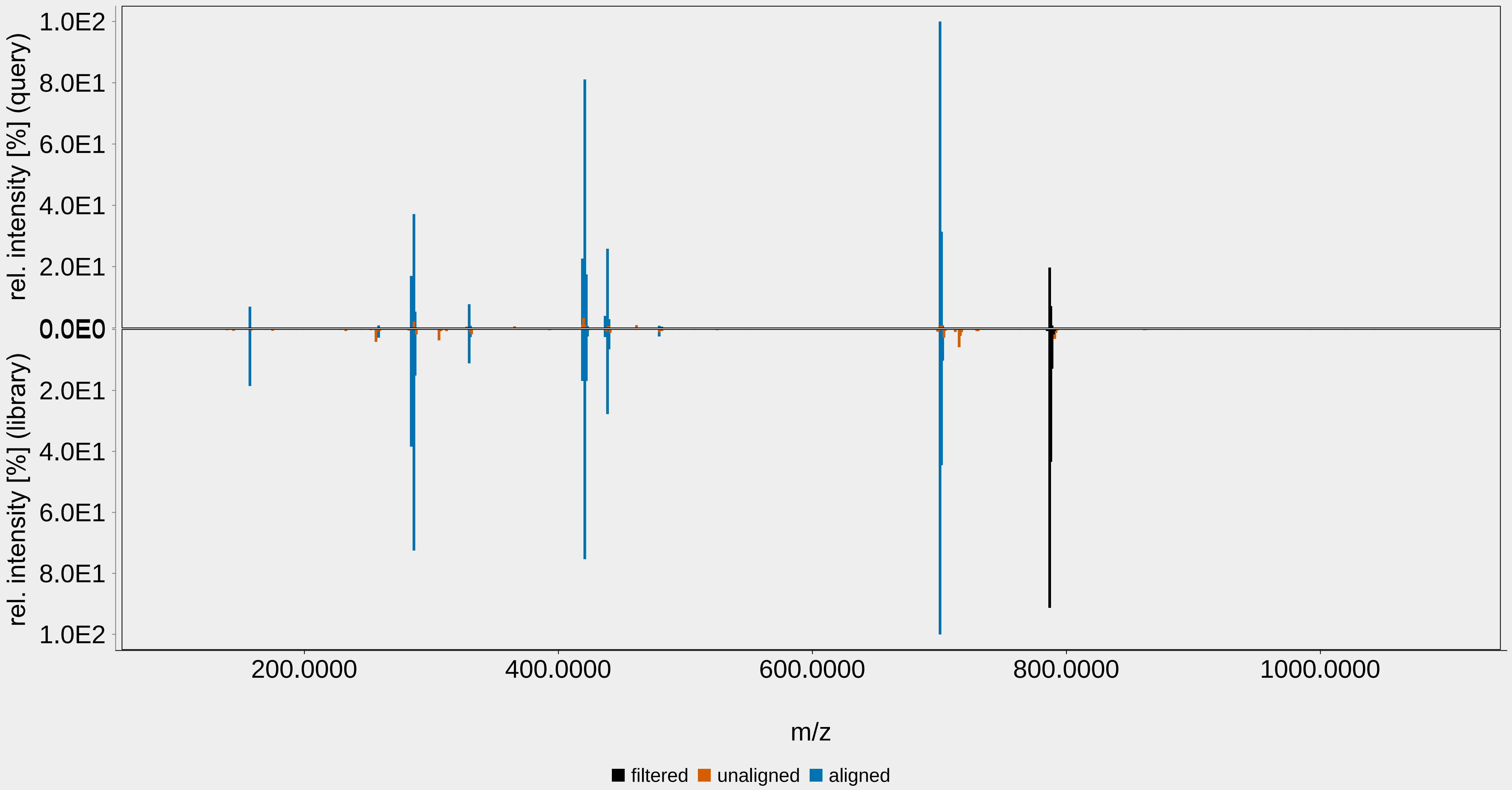

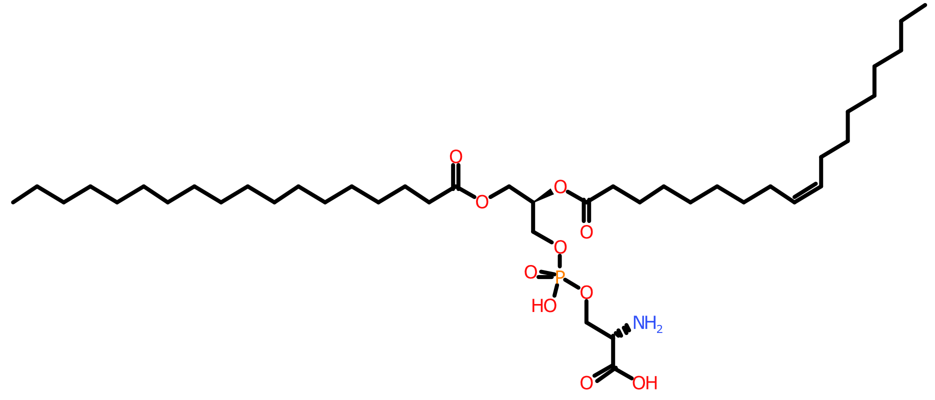

Compound information

Name:

Formula: C42H80NO10P

Mol. weight: 789.552

Exact mass: 789.552

Ion type: [M-H]-

Precursor m/z: 788.542

Polarity: negative

InChI:

InChI key:

SMILES:

Instrument information

Instrument: qTof

Ms level: MS2

Database links

Other information

Principal investigator: O Laprevote,

Data collector: O Laprevote, mpanitchpakdi,

Massbank:LQB00564 PE 36:4

0.947

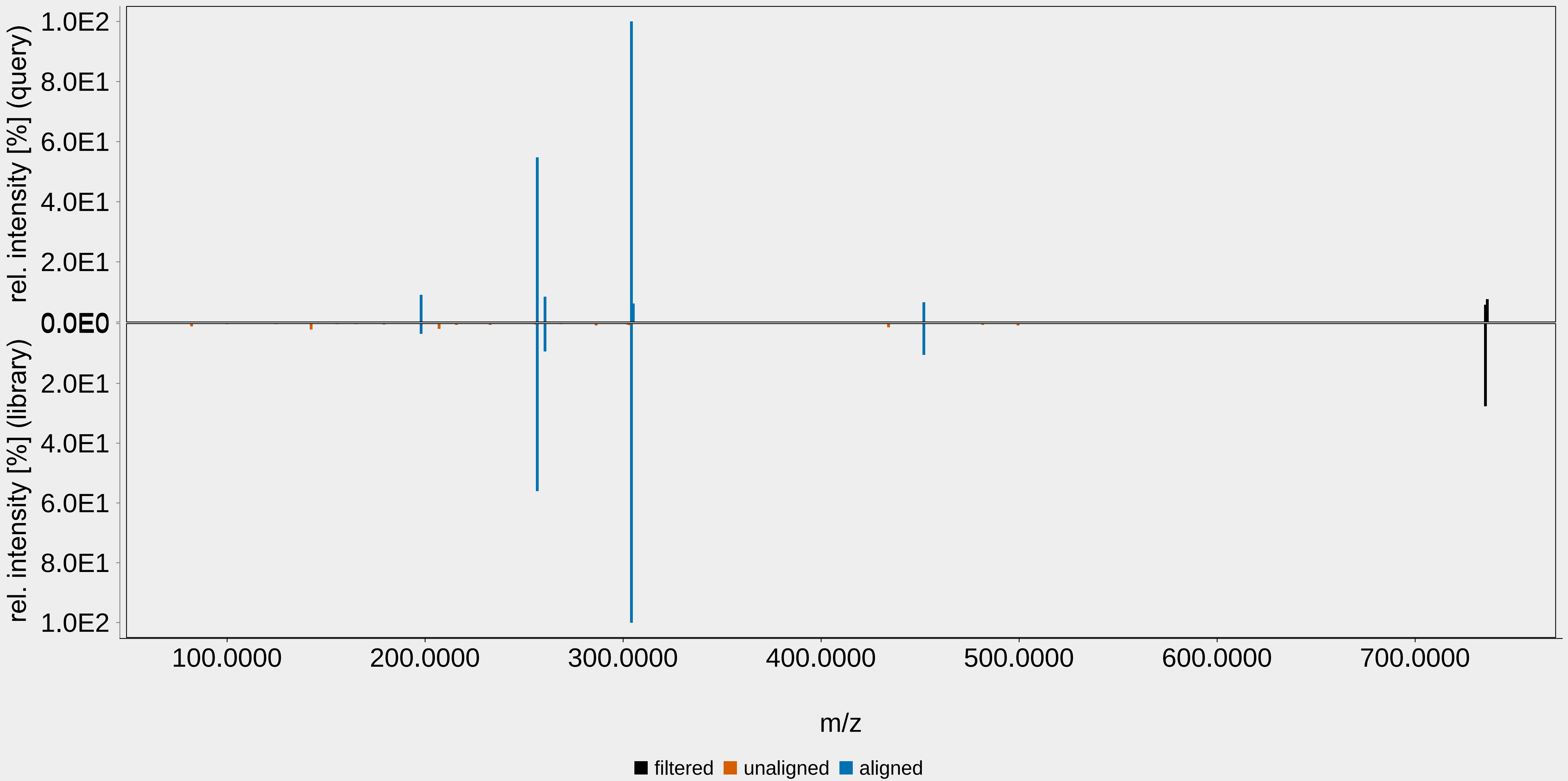

Compound information

Name: Massbank:LQB00564 PE 36:4

Exact mass: 0.0

Ion type: M-H

Precursor m/z: 738.503

Charge: 1

Polarity: Negative

InChI:

SMILES:

Instrument information

Instrument type: qTof

Ion source: ESI

Ms level: 2

Acquisition: Isolated

Database links

Other information

Principal investigator: Massbank

Data collector: Massbank

Entry ID: CCMSLIB00005737665

Massbank:LQB00178 PE 36:4

0.947

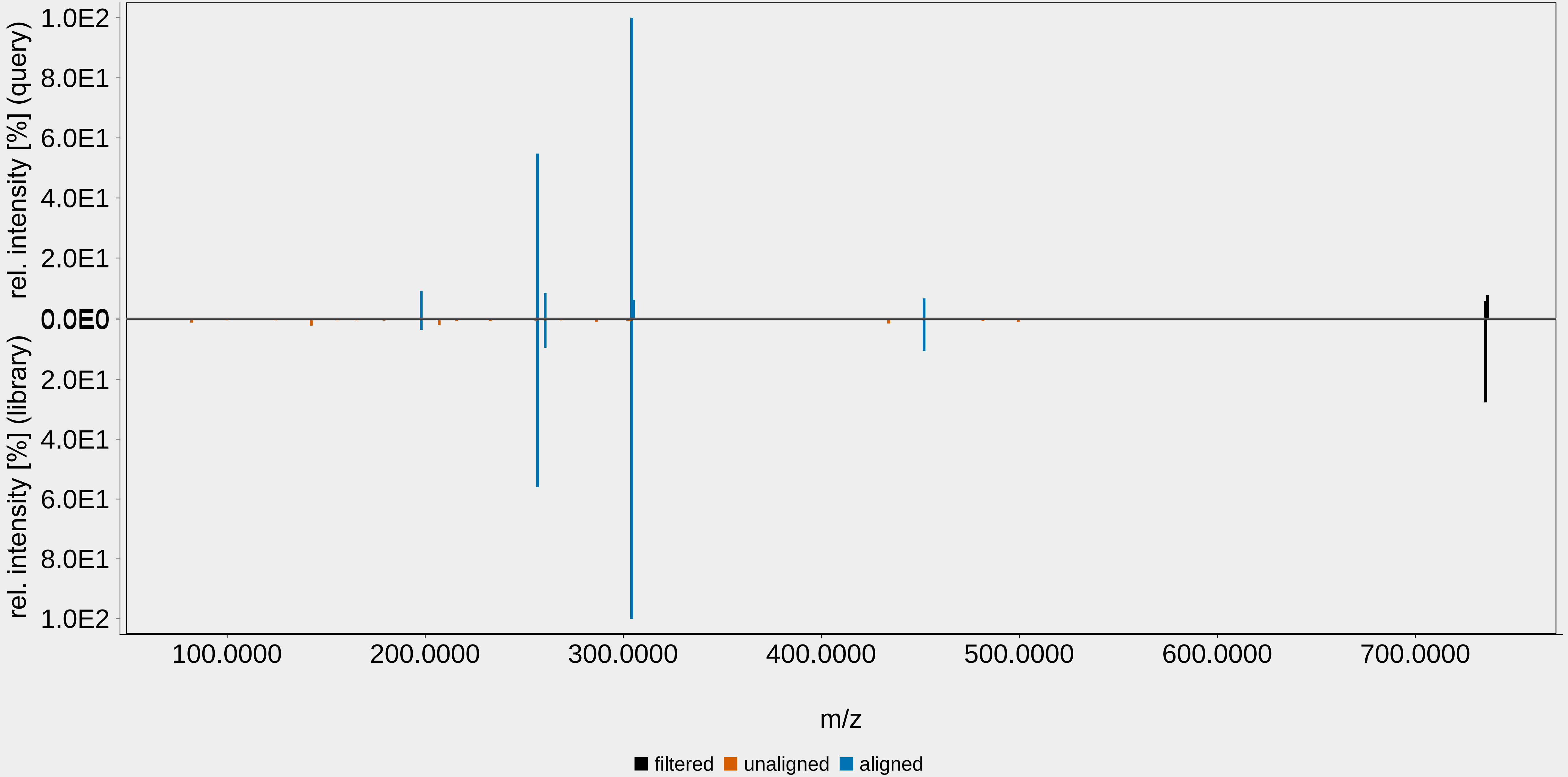

Compound information

Name: Massbank:LQB00178 PE 36:4

Exact mass: 0.0

Ion type: M-H

Precursor m/z: 738.503

Charge: 1

Polarity: Negative

InChI:

SMILES:

Instrument information

Instrument type: qTof

Ion source: ESI

Ms level: 2

Acquisition: Isolated

Database links

Other information

Principal investigator: Massbank

Data collector: Massbank

Entry ID: CCMSLIB00005737734

PE(16:0/18:1); [M-H]- C39H75N1O8P1 **0.930**

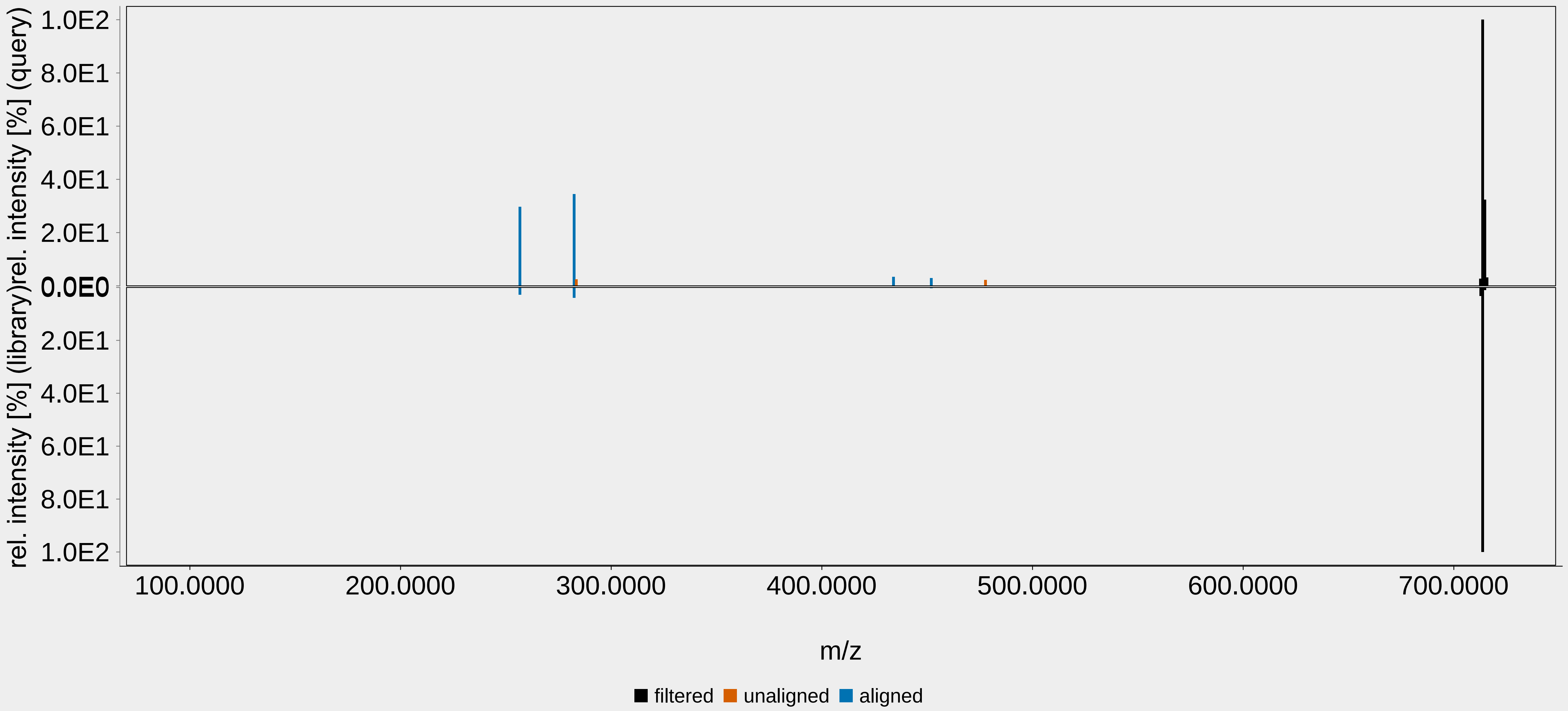

Compound information

Name: PE(16:0/18:1); [M-H]- C39H75N1O8P1

Exact mass: 0.0

Ion type: M-H

Precursor m/z: 716.523

Charge: 1

Polarity: Negative

Instrument information

Instrument type: HCD; Velos

Ion source: LC-ESI

Ms level: 2

Acquisition: Commercial

Database links

Other information

Principal investigator: Thomas Metz

Data collector: Thomas Metz

Entry ID: CCMSLIB00010072728

PE(16:0_18:1) - (2-aminoethoxy)[3-(hexadecanoyloxy)-2-[octadec-9-enoyloxy]propoxy]phosphinic acid

0.942

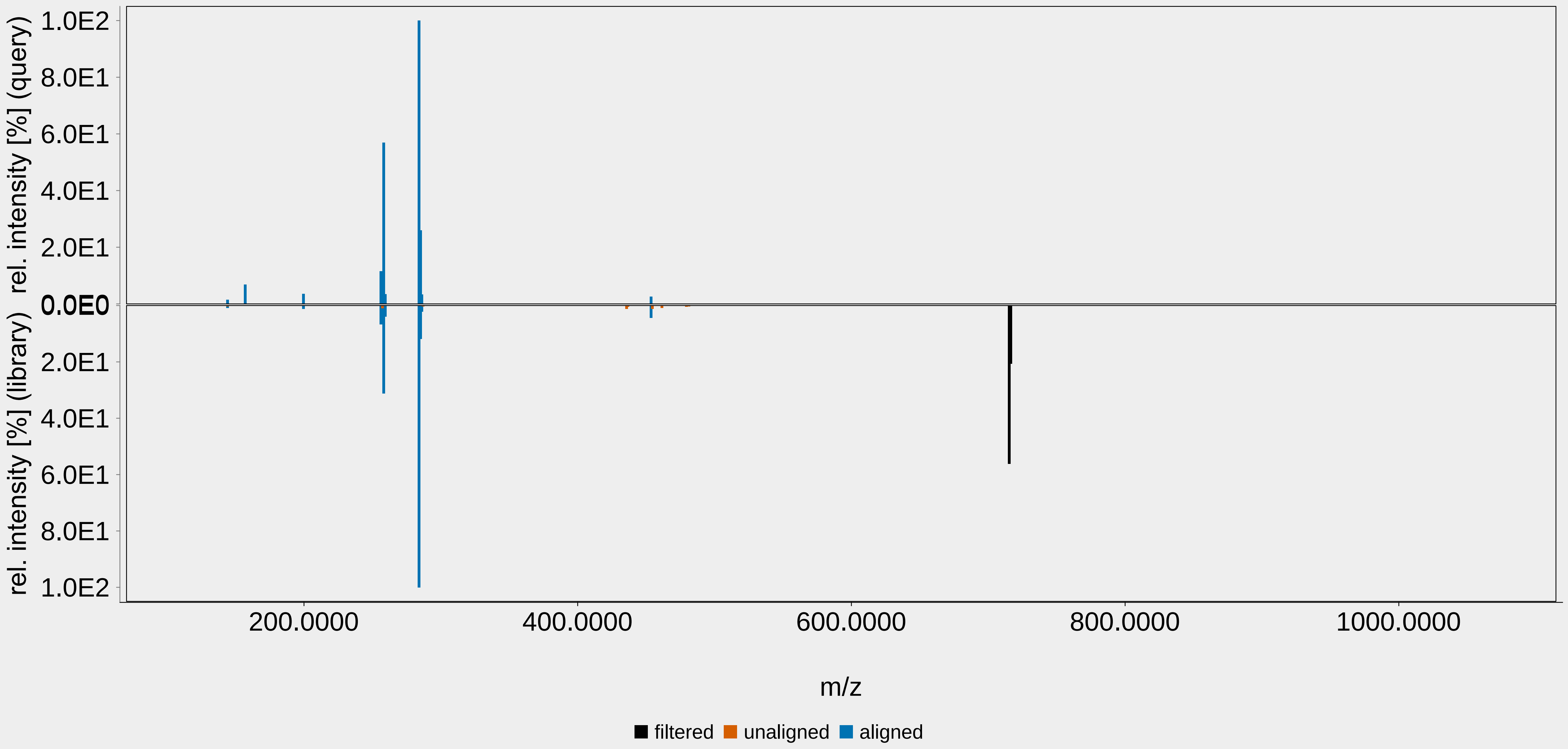

Compound information

Name: PE(16:0_18:1) -

Exact mass: 717.531

Ion type: M-H

Precursor m/z: 716.522

Charge: 1

Polarity: Negative

SMILES:

Instrument information

Instrument type: qTof

Ion source: LC-ESI

Ms level: 2

Acquisition: Lysate

Database links

CAS: 26662-94-2

Other information

Principal investigator: N Auzeil

Data collector: UMR CNRS 8038 Universite de

Entry ID: CCMSLIB00005724205

PA 22:0_18:1 **0.786**

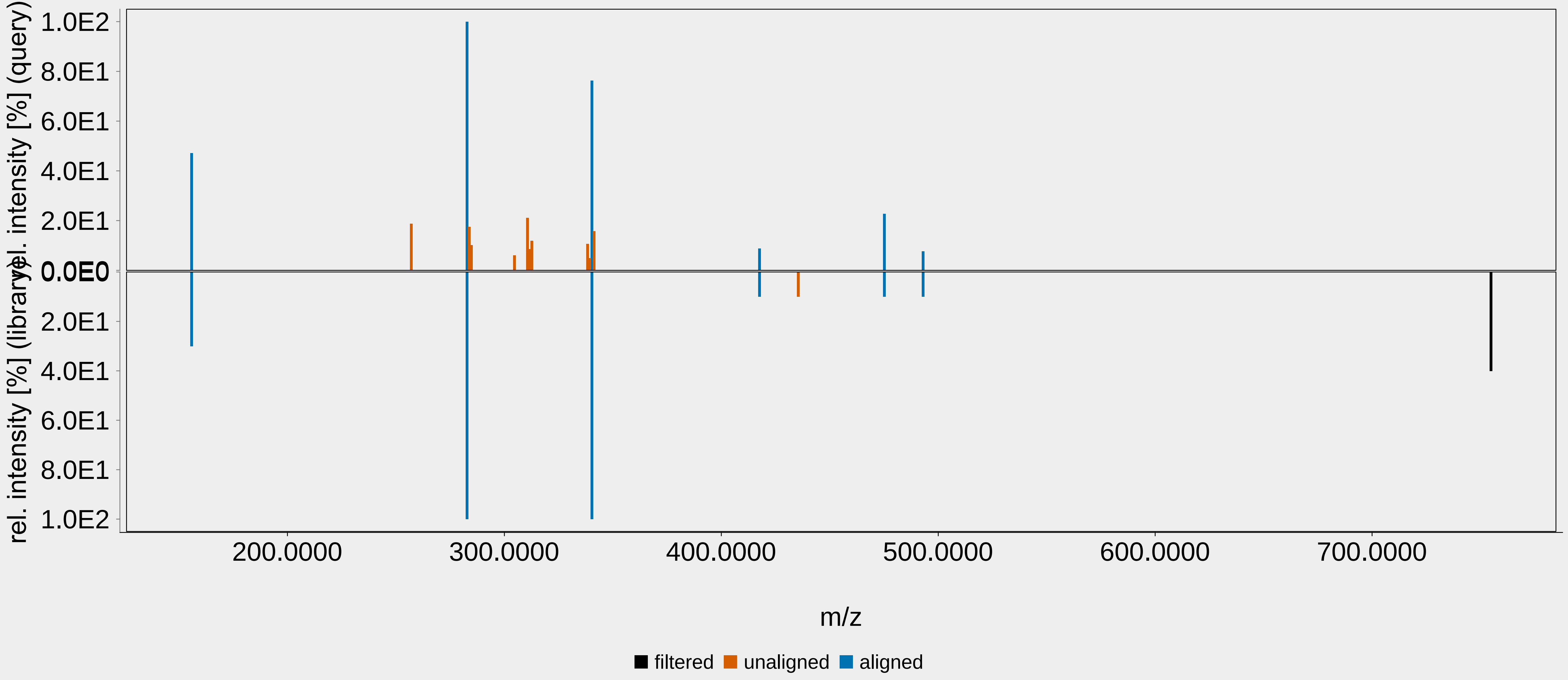

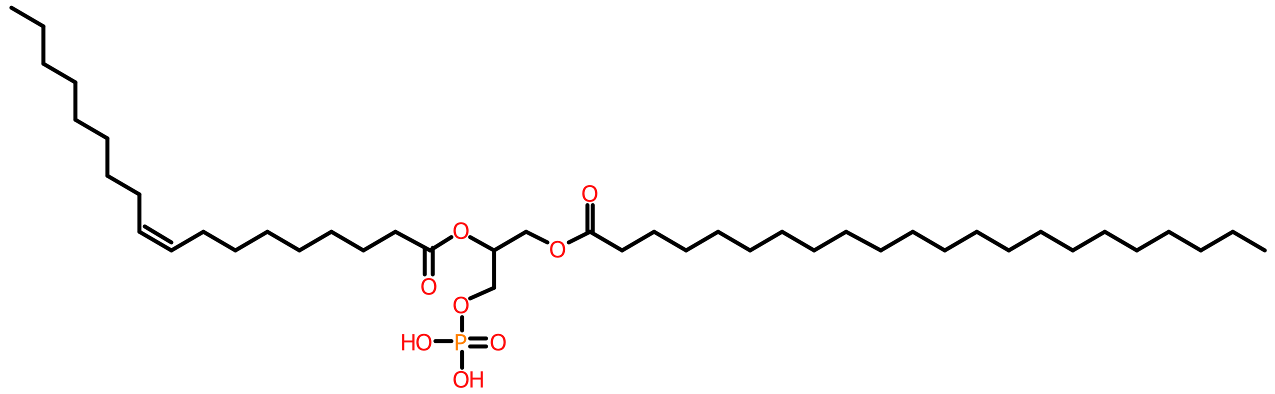

Compound information

Name: PA 22:0_18:1

Formula: C43H83O8P

Ion type: [M-H]-

Precursor m/z: 757.57528

Ccs: 273.22

InChI key:

SMILES:

Num peaks: 8

Instrument information

Database links

Other information

PS 22:4_22:6 0.706

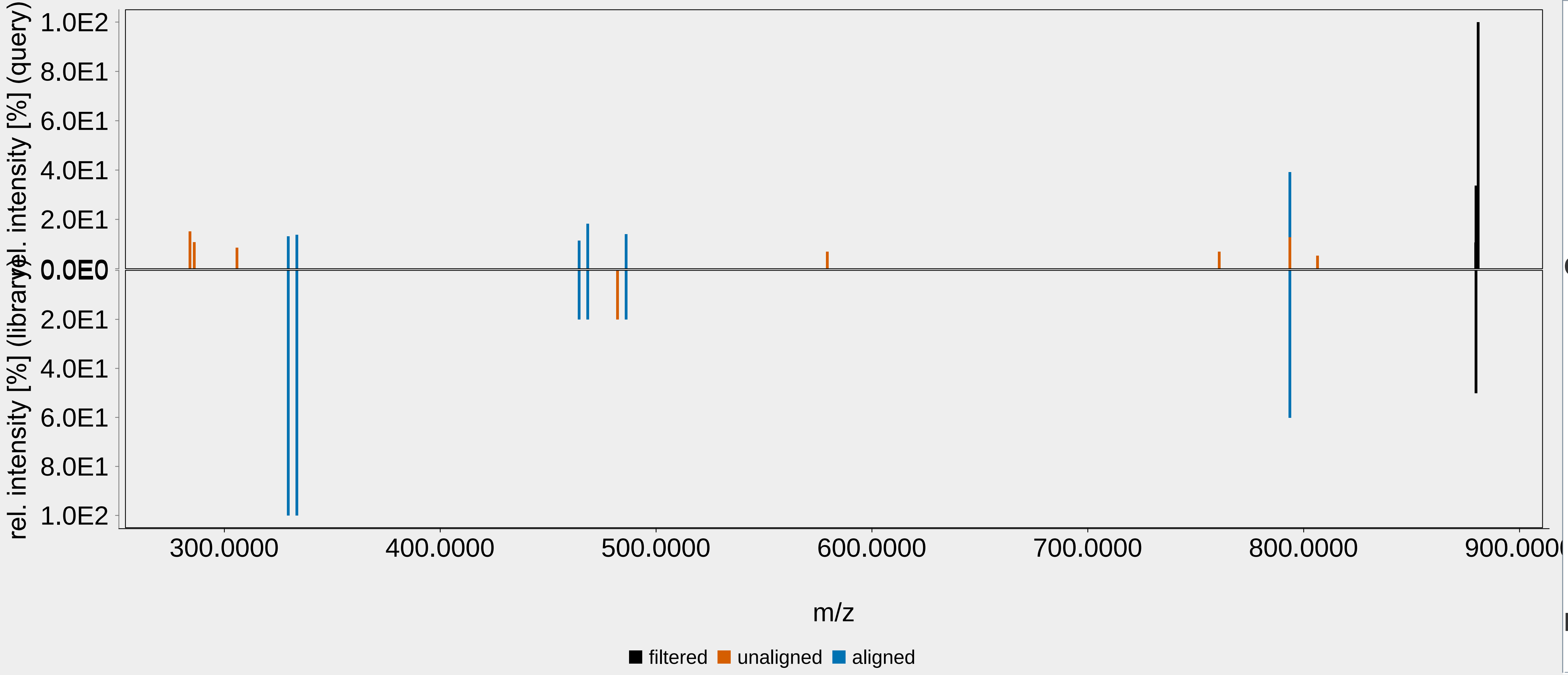

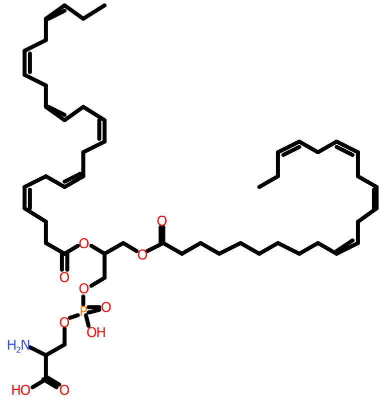

Compound information

Name: PS 22:4_22:6

Formula: C50H78NO10P

Ion type: [M-H]-

Precursor m/z: 882.52906

Ccs: 284.42

InChI key:

SMILES:

Num peaks: 8

Instrument information

Database links

Other information

PI 38:4

0.925

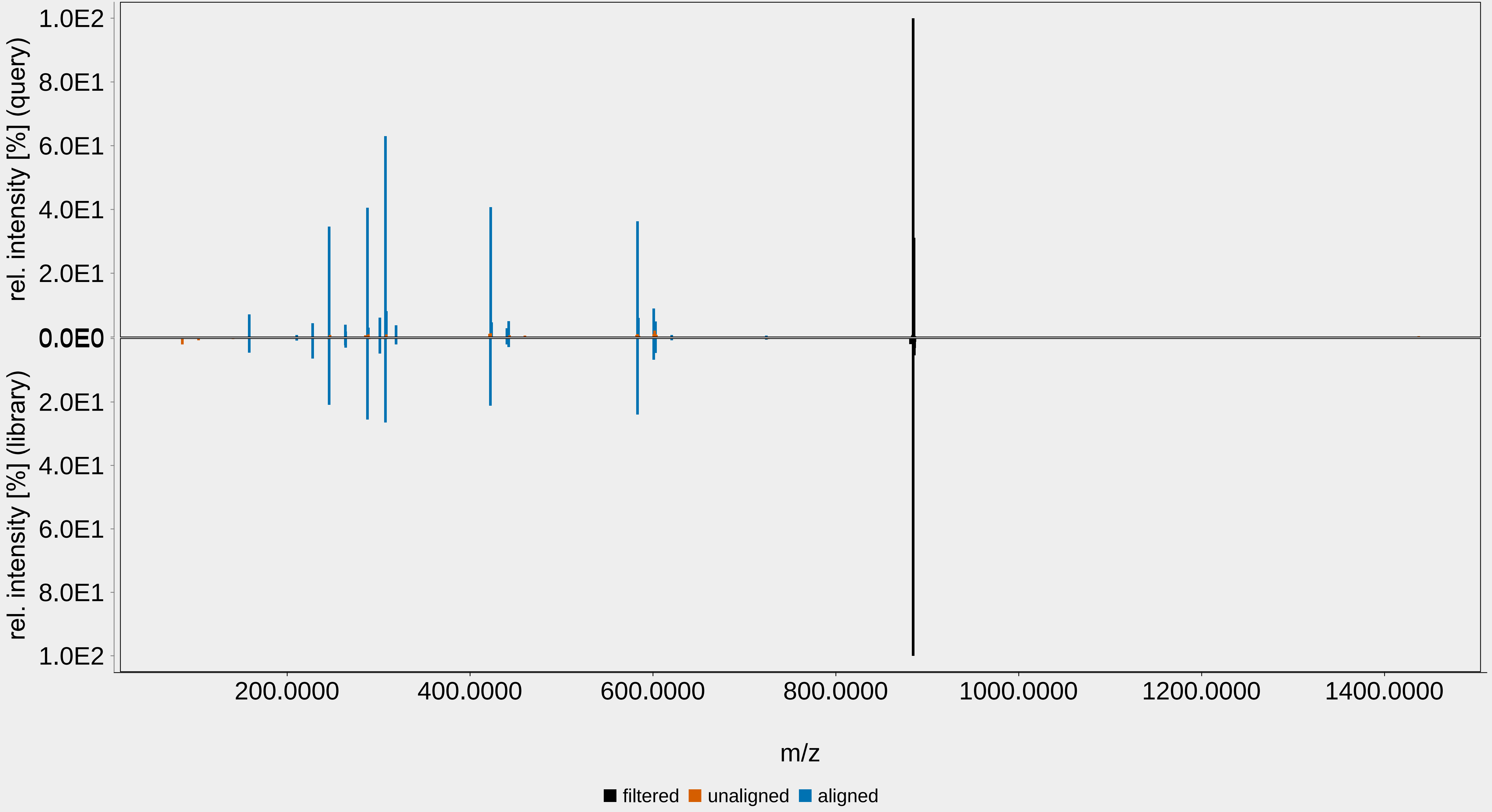

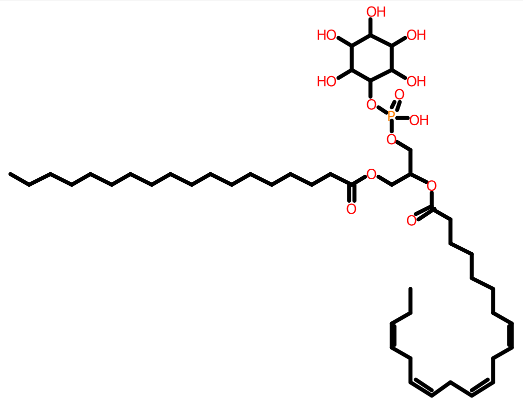

Compound information

Name: PI 38:4

Formula: C47H83O13P

Mol. weight: 887.1439999999999

Exact mass: 887.1439999999999

Ion type: [M-H]-

Precursor m/z: 885.5508

RT: 8.754183

Polarity: negative

InChI:

InChI key:

SMILES:

Instrument information

Instrument type: LC-ESI-QTOF

Instrument: AB Sciex TripleTOF 5600+ system

Ms level: MS2

Collision energy: 45V

Database links

Pubchem: 138227586

MoNA ID: LQB00694

Other information

Principal investigator: Kazutaka Ikeda, Hiroshi

Data collector: Kazutaka Ikeda, Hiroshi

PI 38:4

0.925

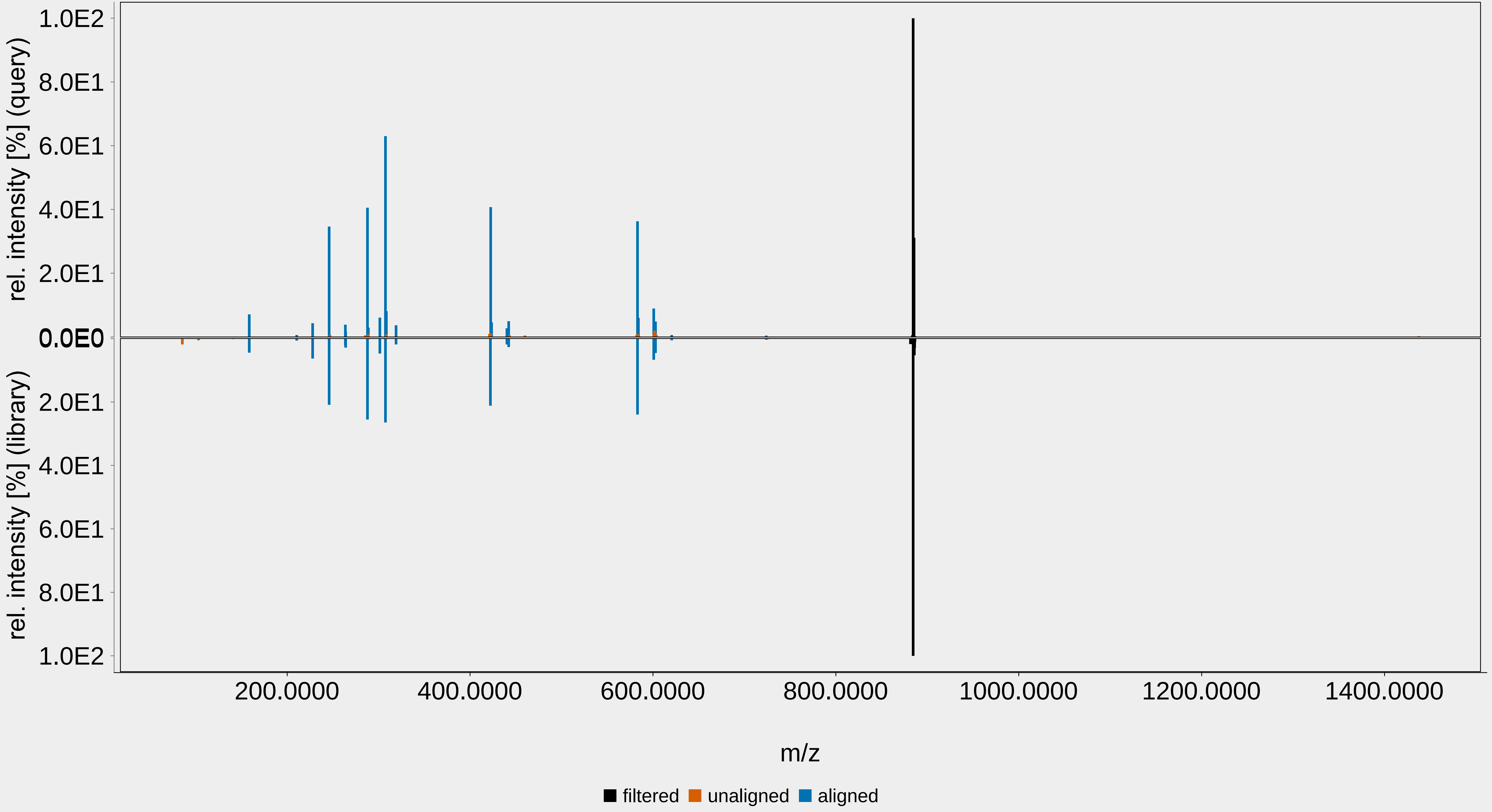

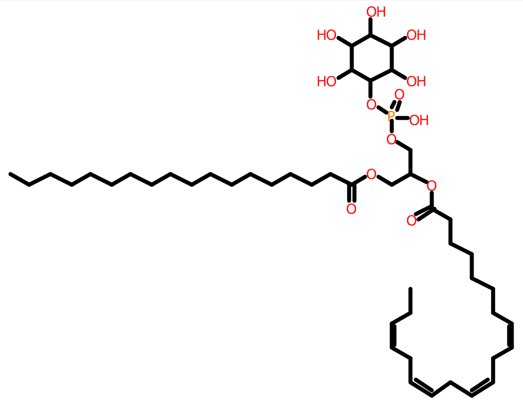

Compound information

Name: PI 38:4

Formula: C47H83O13P

Mol. weight: 887.1439999999999

Exact mass: 887.1439999999999

Ion type: [M-H]-

Precursor m/z: 885.5508

RT: 8.754183

Polarity: negative

InChI:

InChI key:

SMILES:

Instrument information

Instrument type: LC-ESI-QTOF

Instrument: AB Sciex TripleTOF 5600+ system

Ms level: MS2

Collision energy: 45V

Database links

Pubchem: 138227586

MoNA ID: LQB00308

Other information

Principal investigator: Kazutaka Ikeda, Hiroshi

Data collector: Kazutaka Ikeda, Hiroshi

Uridine 5'-diphospho-N-acetylglucosamine **0.785**

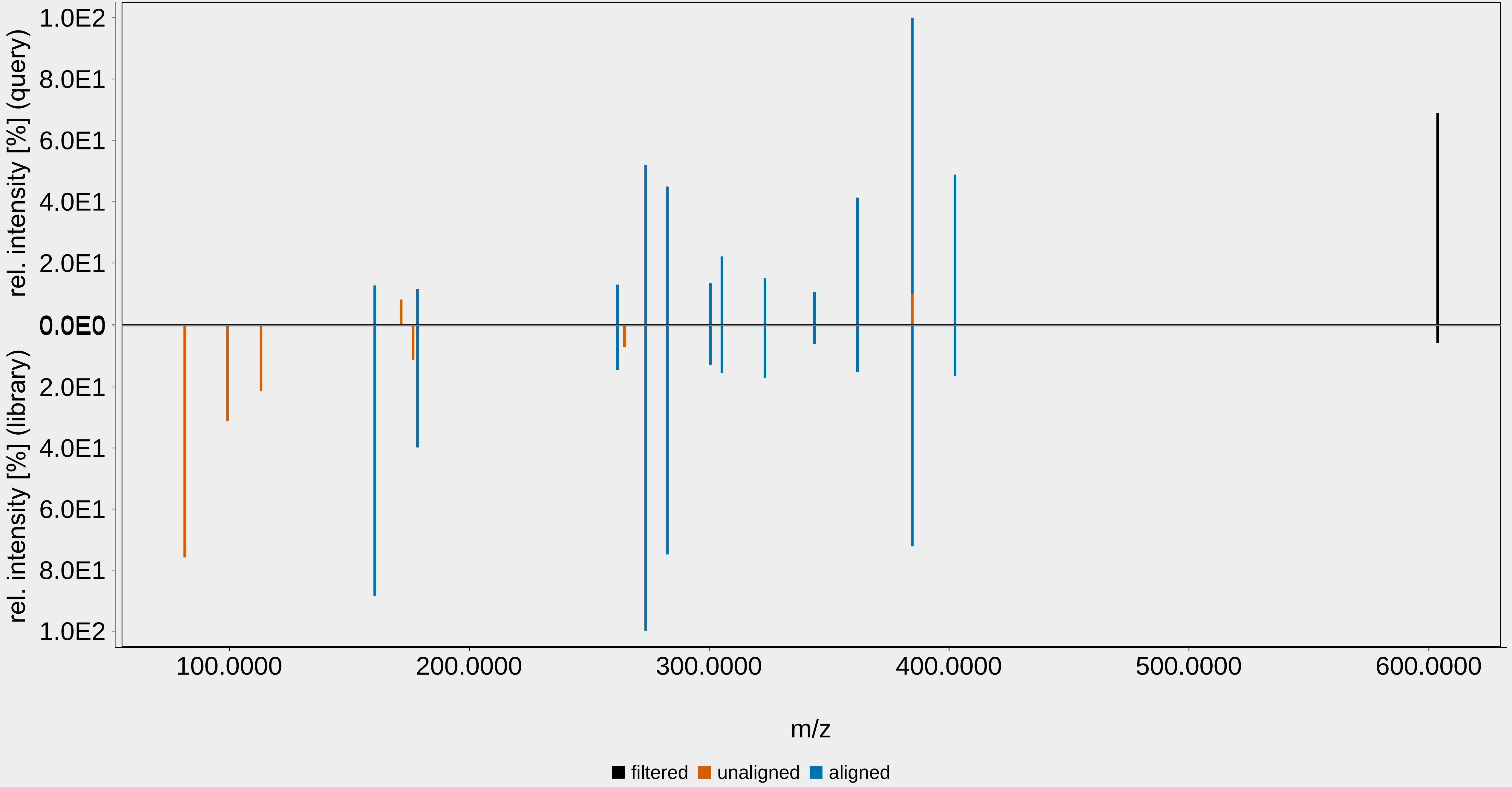

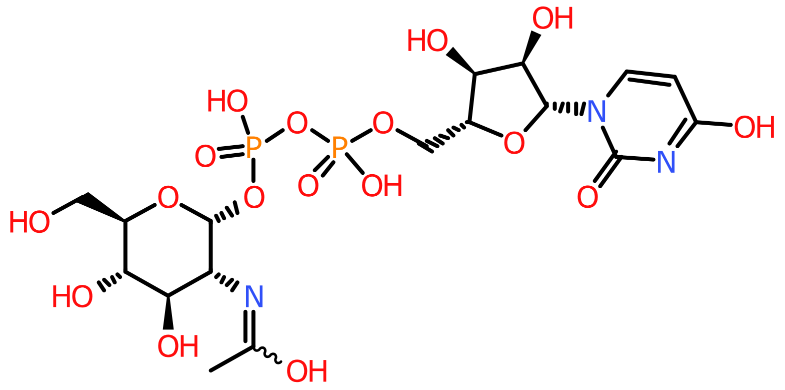

Compound information

Name: Uridine

Formula: C17H27N3O17P2

Exact mass: 607.08157

Ion type: [M-H]-

Precursor m/z: 606.07377

Polarity: NEGATIVE

InChI:

InChI key:

SMILES:

Num peaks: 18

Instrument information

Instrument type: LC-ESI-QTOF

Instrument: UPLC Q-Tof Premier, Waters

Ms level: MS2

Collision energy: 30 V

Database links

Other information

Entry ID: MSBNK-RIKEN-PR100621

Comment: Parent=606.07377

[(2R,3R,4R,5S,6R)-3-acetamido-4,5-dihydroxy-6-(hydroxymethyl)oxan-2-yl] [[(2R,3S,4R,5R)-5-(2,4-dioxopyrimidin-1-yl)-3,4-dihydroxyoxolan-2-yl]methoxy-hydroxyphosphoryl]

0.785

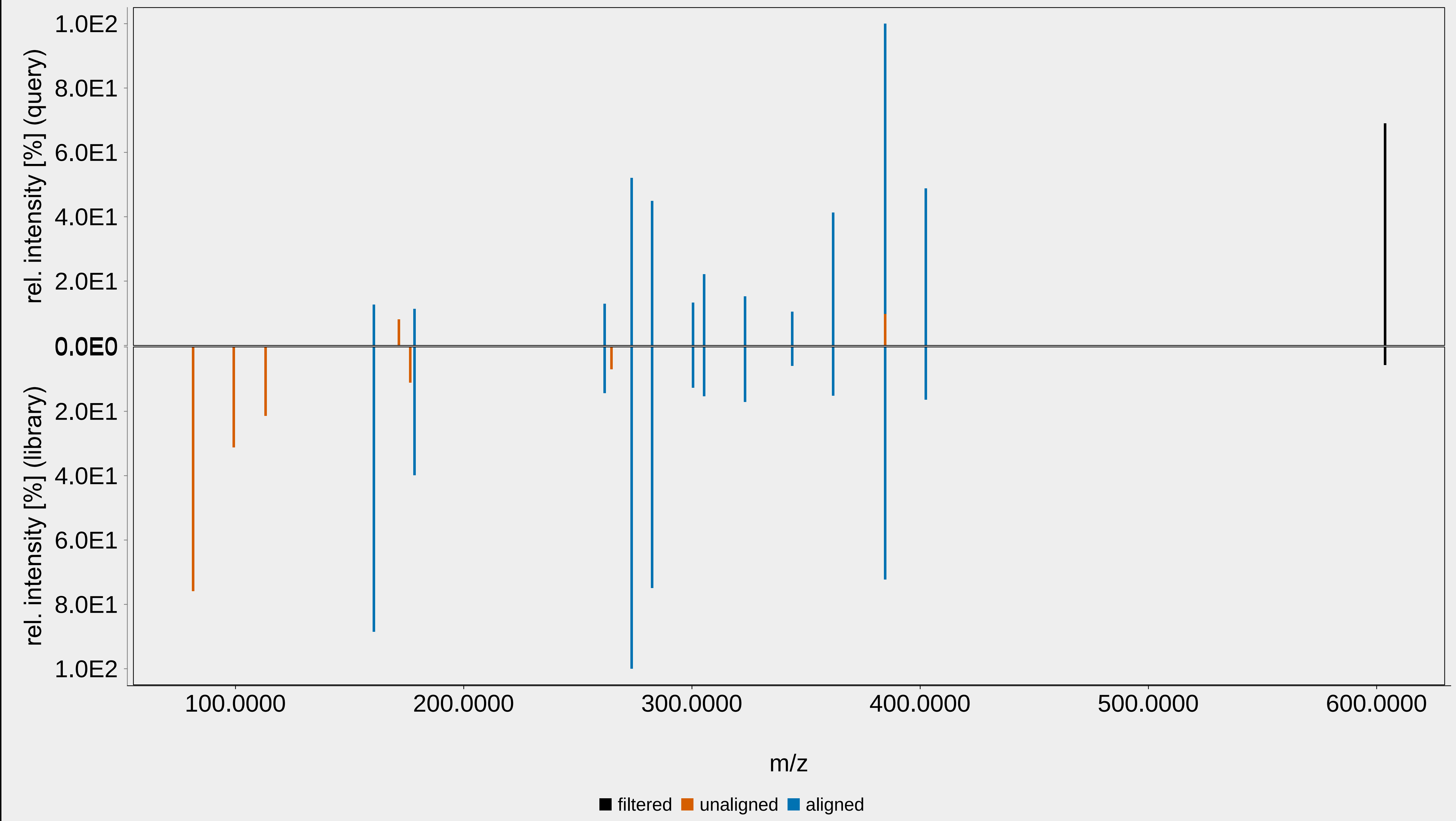

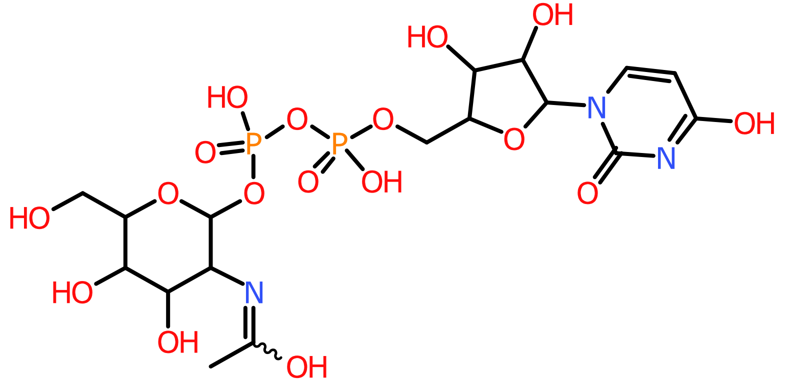

Compound information

Name:

Formula: C17H27N3O17P2

Exact mass: 607.359

Ion type: [M+H]+

Precursor m/z: 606.07377

Polarity: NEGATIVE

InChI:

InChI key:

SMILES:

Num peaks: 18

Instrument information

Instrument type: LC-ESI-QTOF

Instrument: Q-Tof Premier, Waters

Ms level: MS2

Collision energy: 30 V

Database links

Other information

Entry ID: MSBNK-RIKEN_ReSpect-PT203703

Comment: Parent=606.07377

PE(18:0/0:0); [M-H]- C23H47N1O7P1 0.746

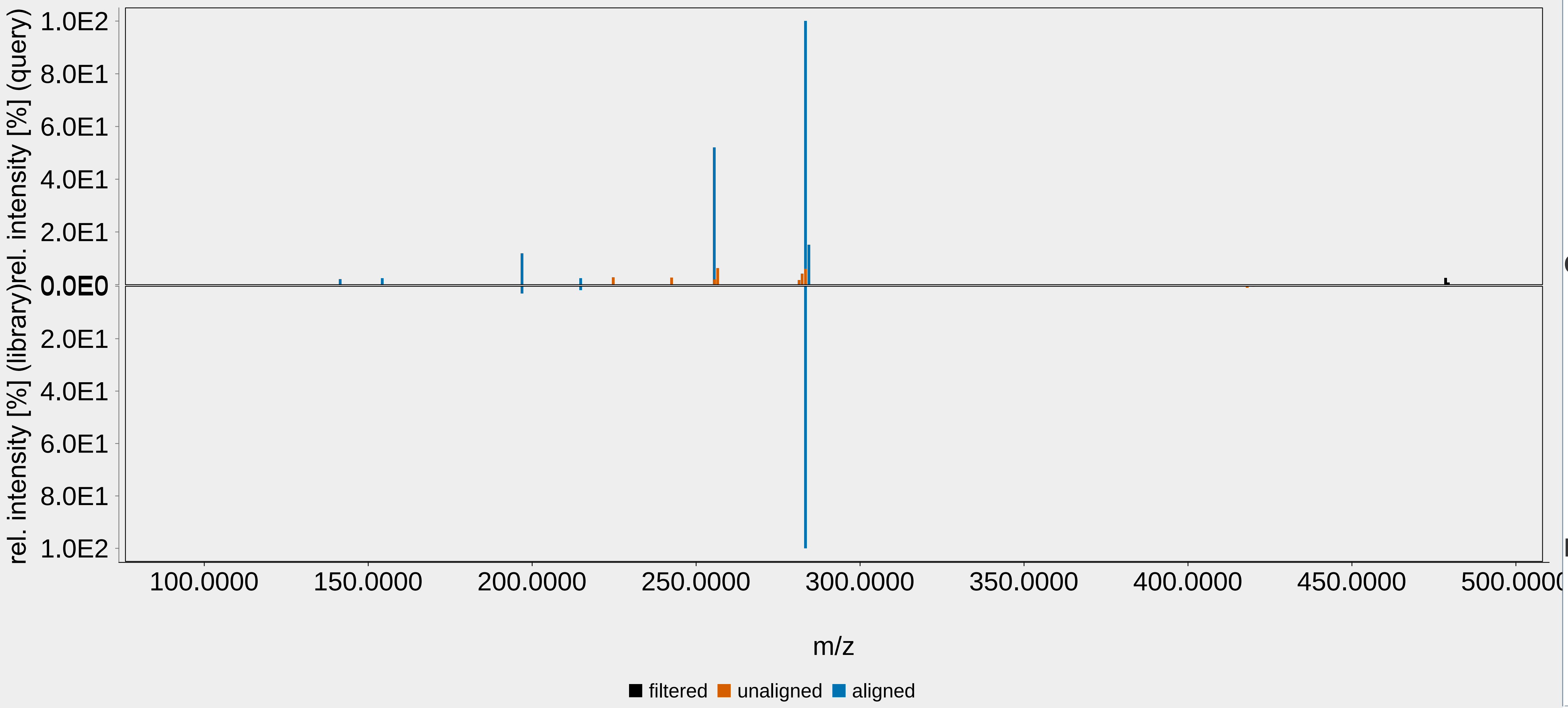

Compound information

Name: PE(18:0/0:0); [M-H]- C23H47N1O7P1

Exact mass: 0.0

Ion type: M-H

Precursor m/z: 480.309

Charge: 1

Polarity: Negative

Instrument information

Instrument type: CID; Lumos

Ion source: LC-ESI

Ms level: 2

Acquisition: Commercial

Database links

Other information

Principal investigator: Thomas Metz

Data collector: Thomas Metz

Entry ID: CCMSLIB00010059720

PE(18:0/0:0); [M-H]- C23H47N1O7P1

0.760

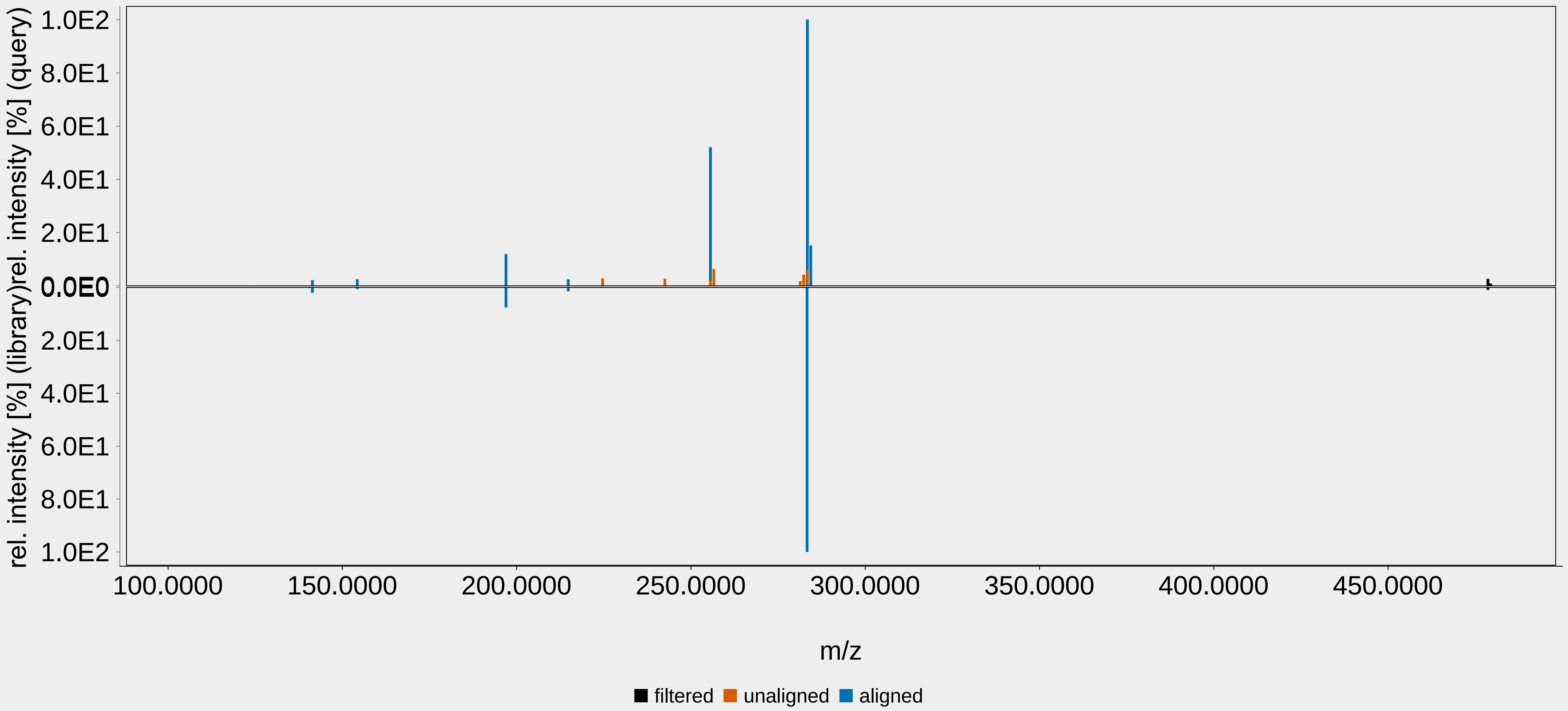

Compound information

Name: PE(18:0/0:0); [M-H]- C23H47N1O7P1

Exact mass: 0.0

Ion type: M-H

Precursor m/z: 480.309

Charge: 1

Polarity: Negative

Instrument information

Instrument type: HCD; Lumos

Ion source: LC-ESI

Ms level: 2

Acquisition: Commercial

Database links

Other information

Principal investigator: Thomas Metz

Data collector: Thomas Metz

Entry ID: CCMSLIB00010059722

FAD - 30.0 eV

0.807

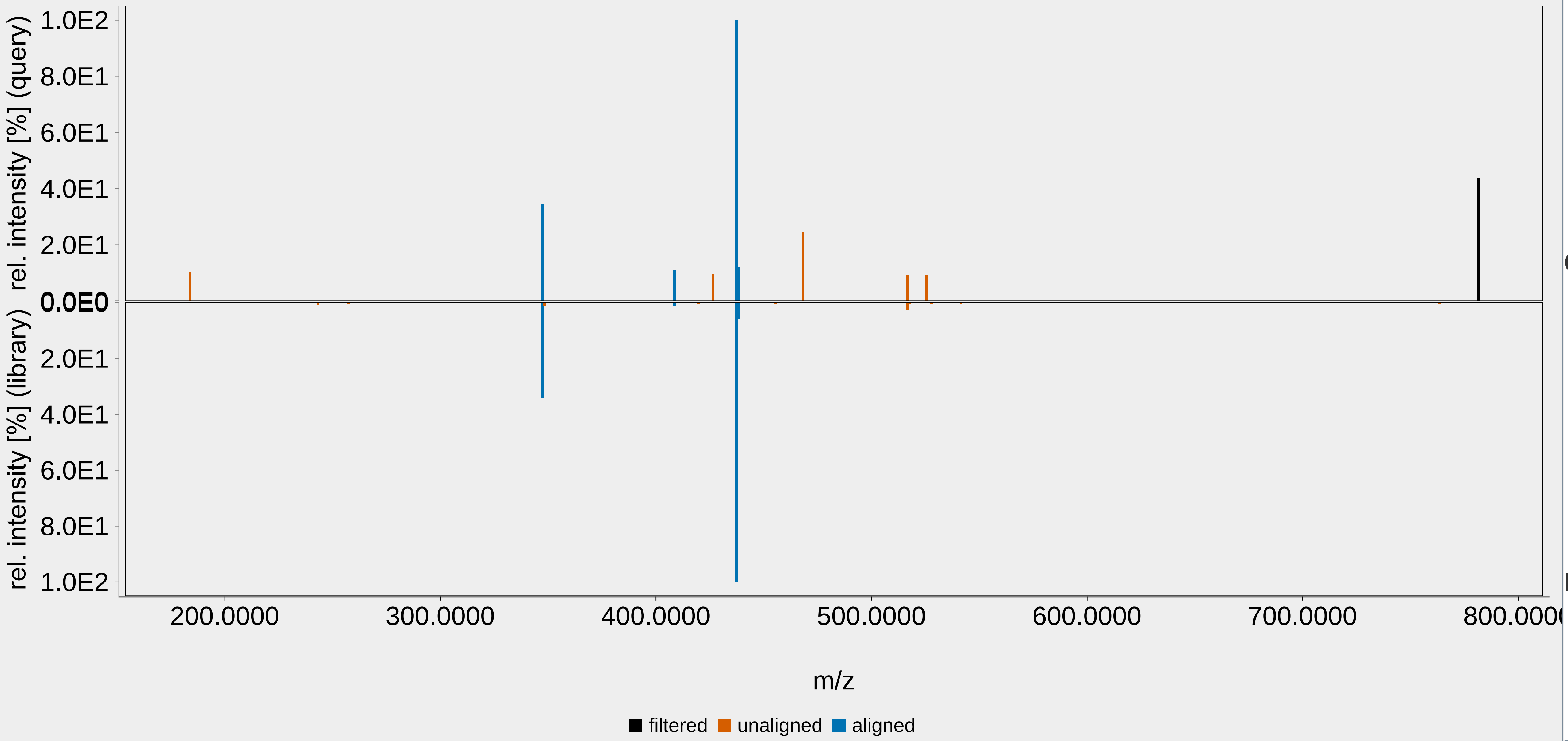

Compound information

Name: FAD - 30.0 eV

Exact mass: 0.0

Ion type: M-H

Precursor m/z: 784.15

Charge: 1

Polarity: Negative

SMILES:

Instrument information

Instrument type: Orbitrap

Ion source: ESI

Ms level: 2

Acquisition: Isolated

Database links

CAS:

Other information

Principal investigator: PI

Data collector: Data Collector

Entry ID: CCMSLIB00006113691

FAD - 30.0 eV

0.822

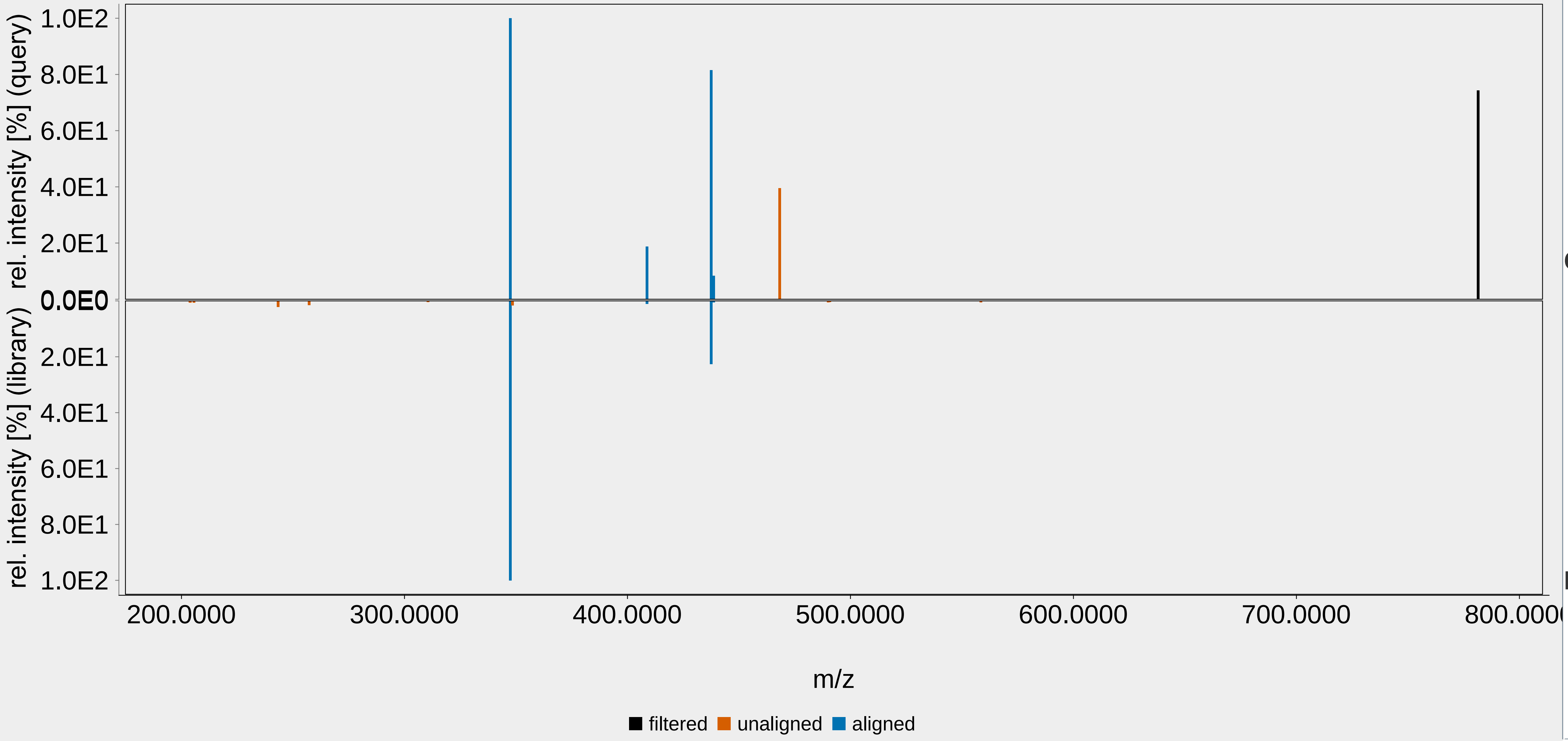

Compound information

Name: FAD - 30.0 eV

Exact mass: 0.0

Ion type: M-H

Precursor m/z: 784.149

Charge: 1

Polarity: Negative

SMILES:

Instrument information

Instrument type: Orbitrap

Ion source: ESI

Ms level: 2

Acquisition: Isolated

Database links

CAS:

Other information

Principal investigator: PI

Data collector: Data Collector

Entry ID: CCMSLIB00006113726

PA 18:1_24:1 **0.817**

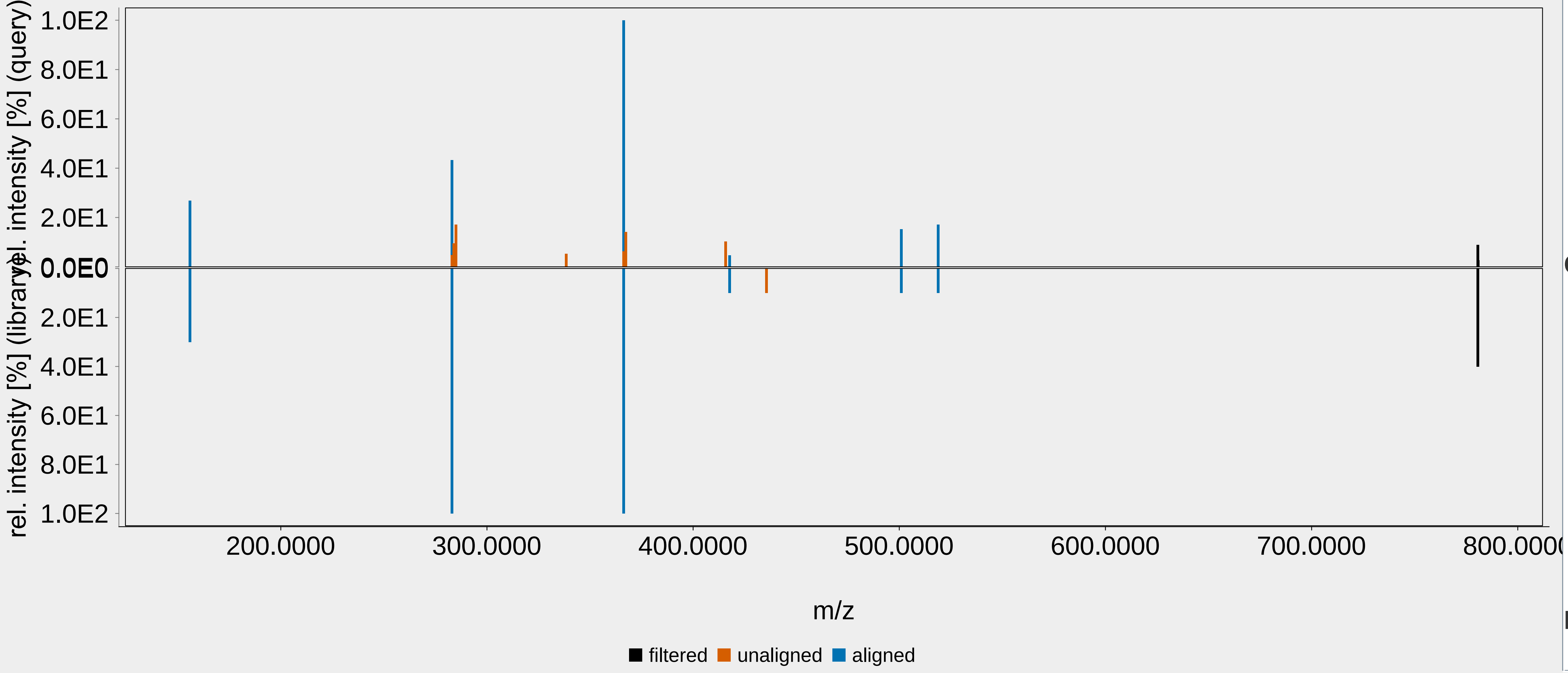

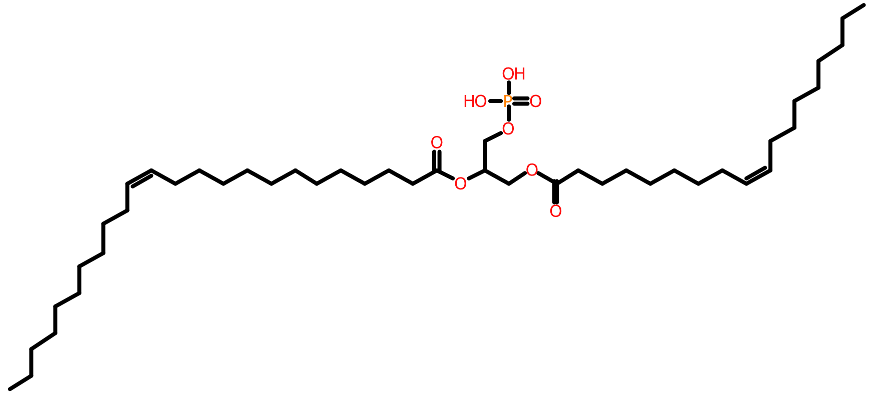

Compound information

Name: PA 18:1_24:1

Formula: C45H85O8P

Ion type: [M-H]-

Precursor m/z: 783.59093

Ccs: 279.35

InChI key:

SMILES:

Num peaks: 8

Instrument information

Database links

Other information

PE(22:6/0:0); [M-H]- C27H43N1O7P1 0.920

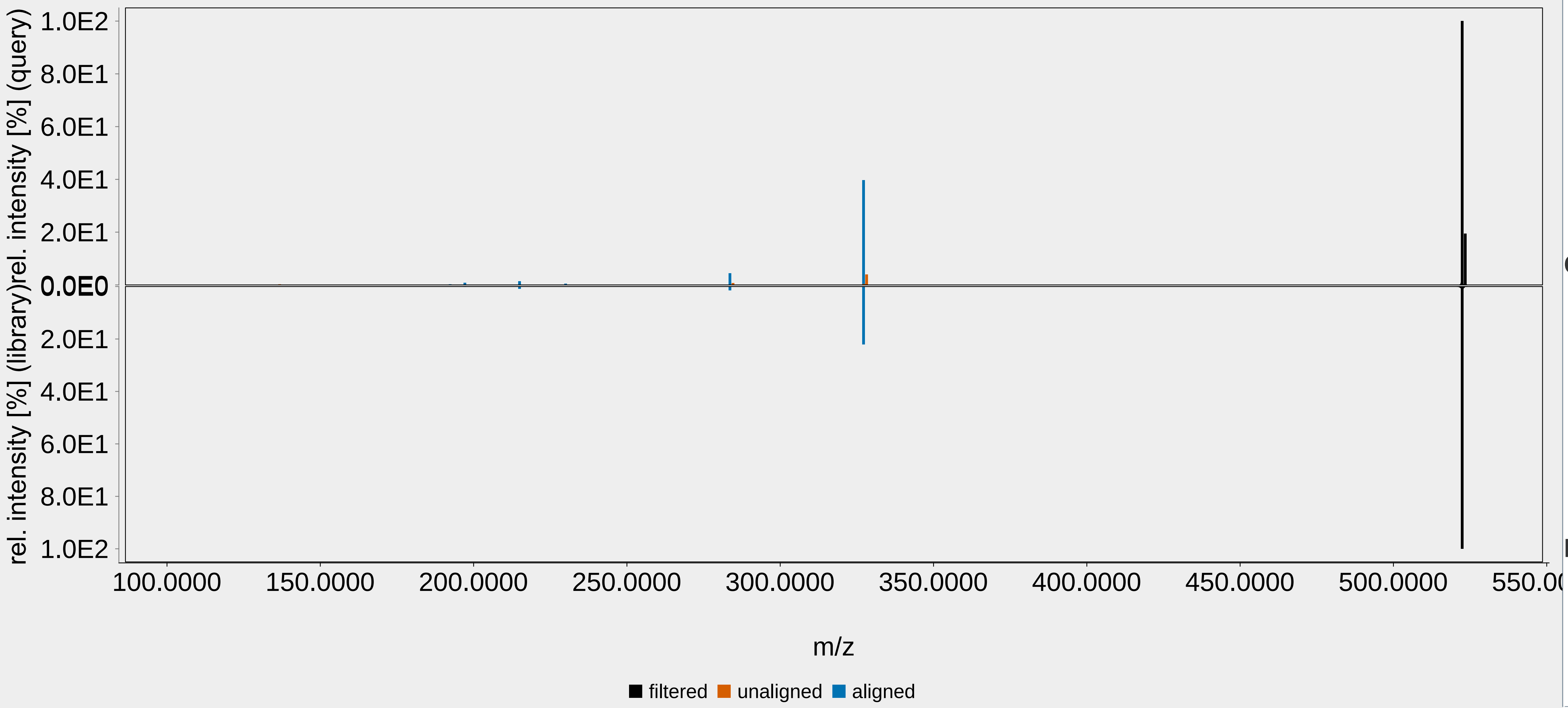

Compound information

Name: PE(22:6/0:0); [M-H]- C27H43N1O7P1

Exact mass: 0.0

Ion type: M-H

Precursor m/z: 524.278

Charge: 1

Polarity: Negative

Instrument information

Instrument type: HCD; Velos

Ion source: LC-ESI

Ms level: 2

Acquisition: Commercial

Database links

Other information

Principal investigator: Thomas Metz

Data collector: Thomas Metz

Entry ID: CCMSLIB00010073025

PE(22:6/0:0); [M-H]- C27H43N1O7P1 **0.932**

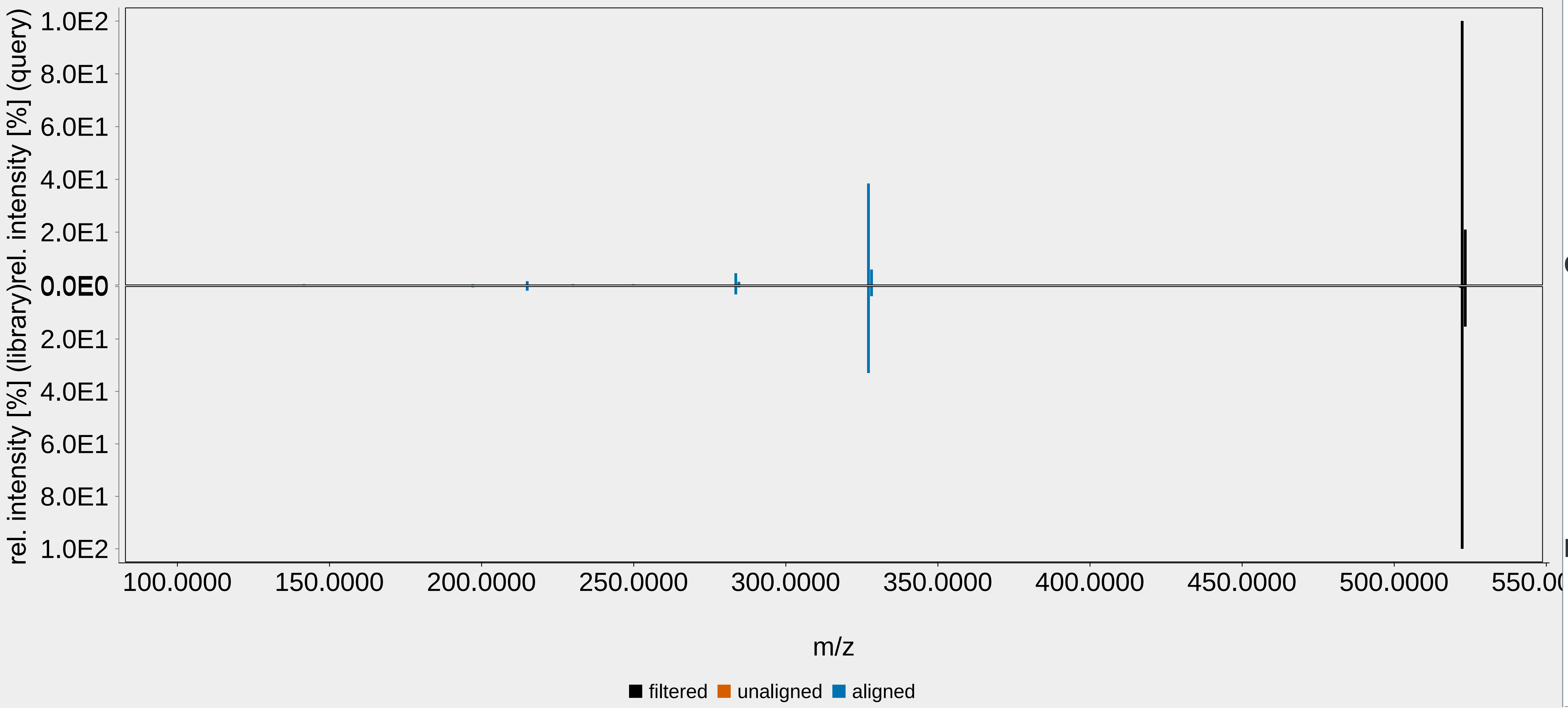

Compound information

Name: PE(22:6/0:0); [M-H]- C27H43N1O7P1

Exact mass: 0.0

Ion type: M-H

Precursor m/z: 524.278

Charge: 1

Polarity: Negative

Instrument information

Instrument type: HCD; Velos

Ion source: LC-ESI

Ms level: 2

Acquisition: Commercial

Database links

Other information

Principal investigator: Thomas Metz

Data collector: Thomas Metz

Entry ID: CCMSLIB00010086705

PE(17:0/22:6); [M-H]- C44H75N1O8P1 0.861

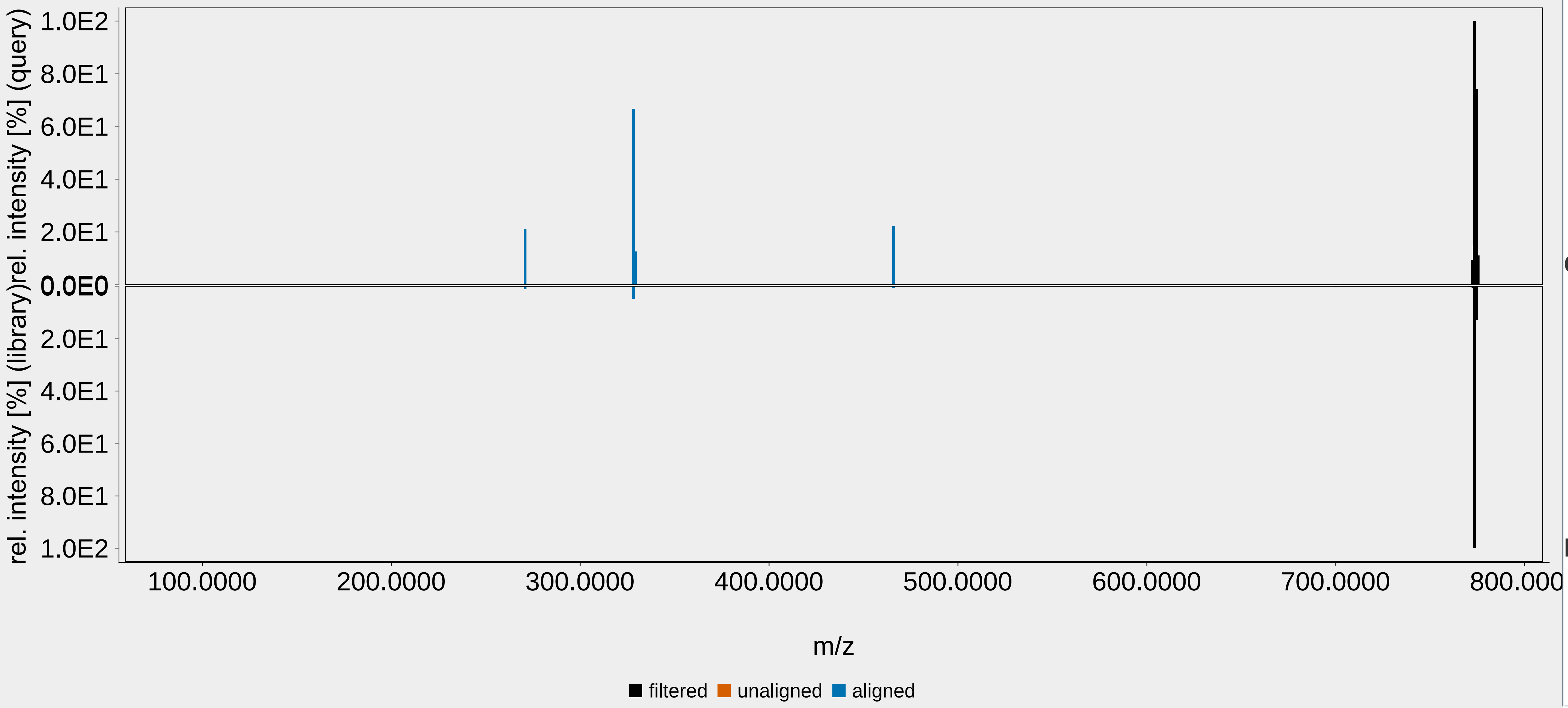

Compound information

Name: PE(17:0/22:6); [M-H]- C44H75N1O8P1

Exact mass: 0.0

Ion type: M-H

Precursor m/z: 776.523

Charge: 1

Polarity: Negative

Instrument information

Instrument type: HCD; Velos

Ion source: LC-ESI

Ms level: 2

Acquisition: Commercial

Database links

Other information

Principal investigator: Thomas Metz

Data collector: Thomas Metz

Entry ID: CCMSLIB00010086568

PE(17:0/22:6); [M-H]- C44H75N1O8P1 **0.890**

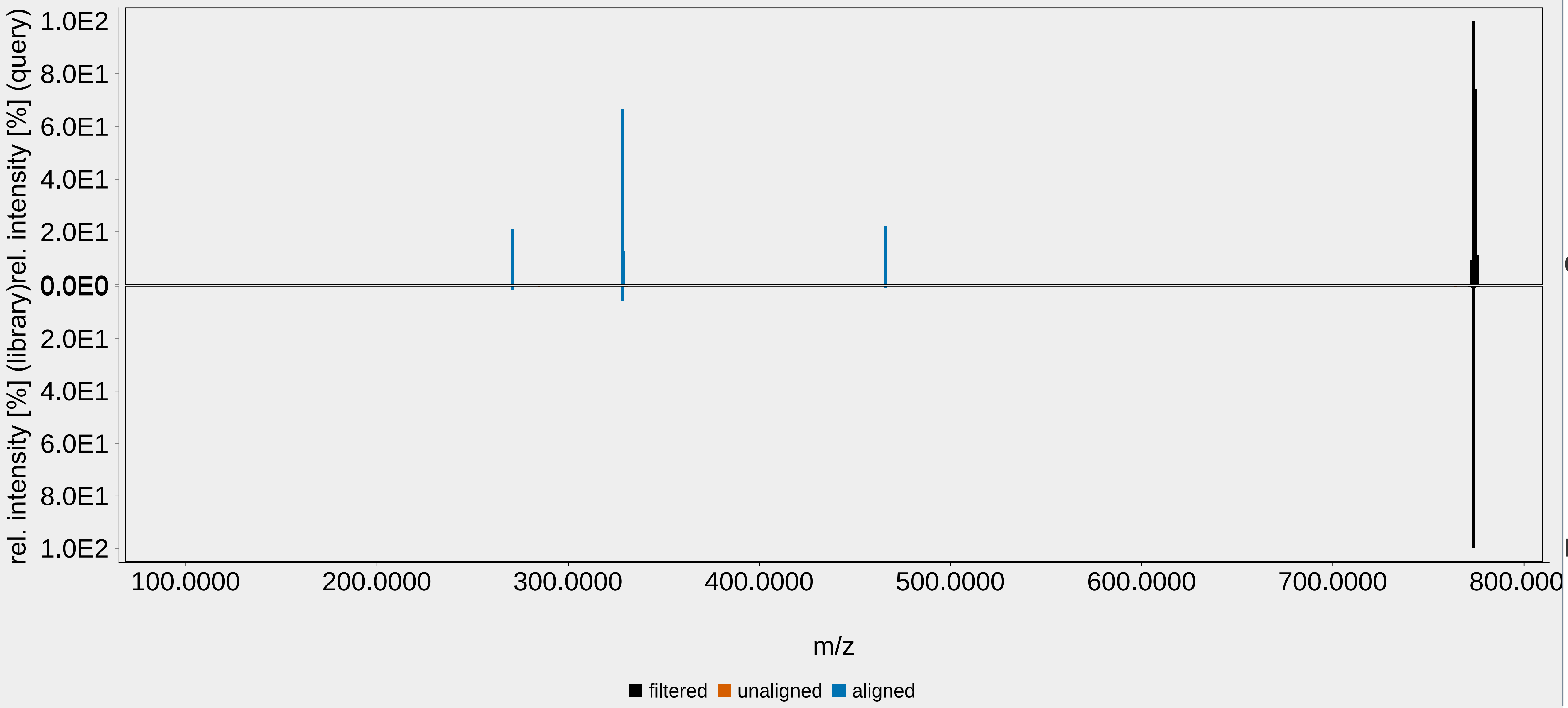

Compound information

Name: PE(17:0/22:6); [M-H]- C44H75N1O8P1

Exact mass: 0.0

Ion type: M-H

Precursor m/z: 776.523

Charge: 1

Polarity: Negative

Instrument information

Instrument type: HCD; Velos

Ion source: LC-ESI

Ms level: 2

Acquisition: Commercial

Database links

Other information

Principal investigator: Thomas Metz

Data collector: Thomas Metz

Entry ID: CCMSLIB00010087500

Spectral Match to 1-Hexadecanoyl-sn-glycero-3-phospho-(1'-myo-inositol) from NIST14

0.892

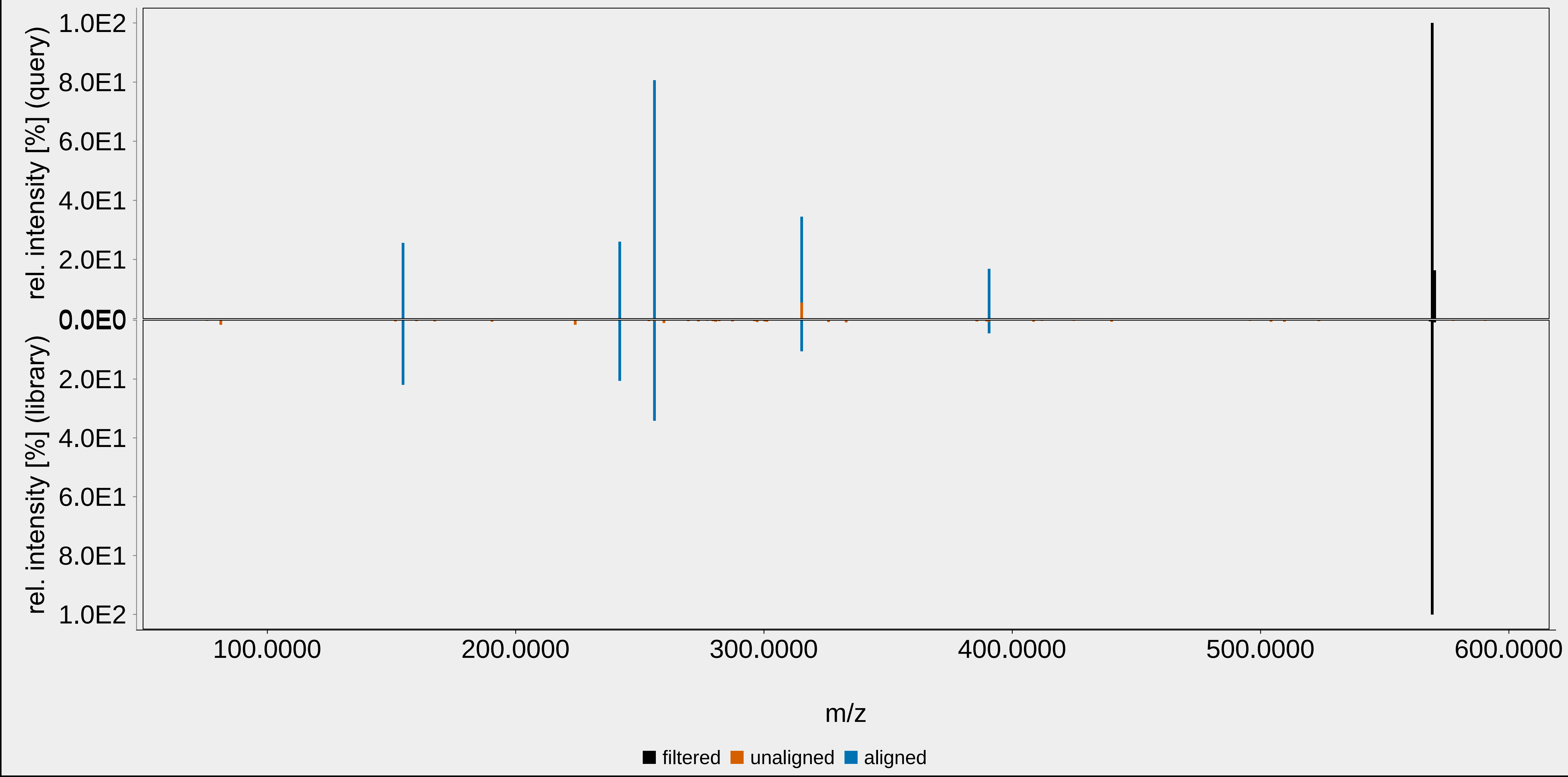

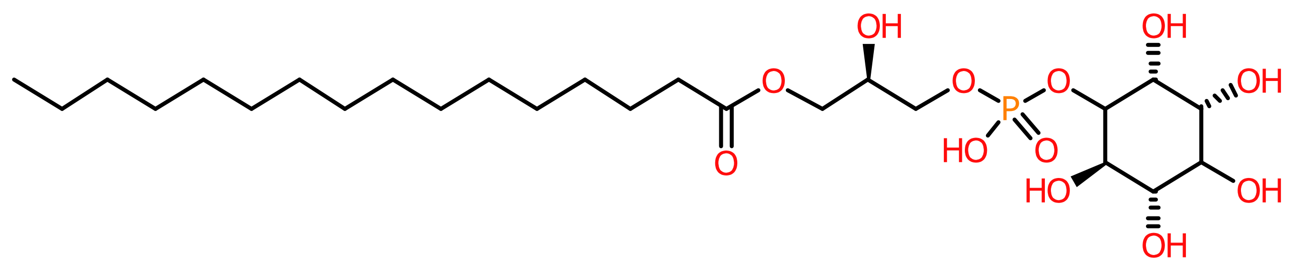

Compound information

Name: Spectral Match to

Exact mass: 572.296

Ion type: M-H

Precursor m/z: 571.294

Charge: 1

Polarity: Negative

InChI:

SMILES:

Instrument information

Instrument type: Ion Trap

Ion source: ESI

Ms level: 2

Acquisition: Isolated

Database links

CAS: 106182201

Other information

Principal investigator: Data from Pieter

Data collector: Data deposited by lfnothias

Entry ID: CCMSLIB00003136330

LPI 16:0 **0.973**

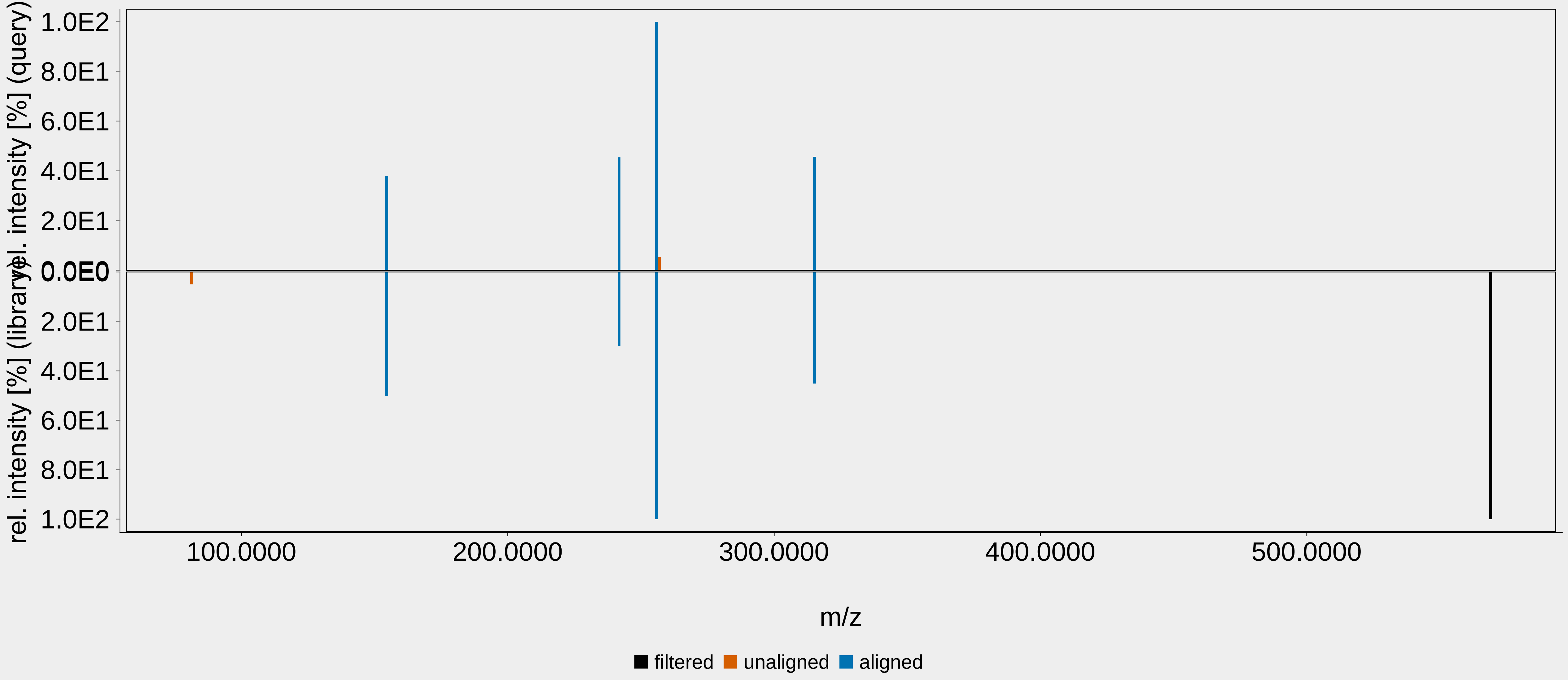

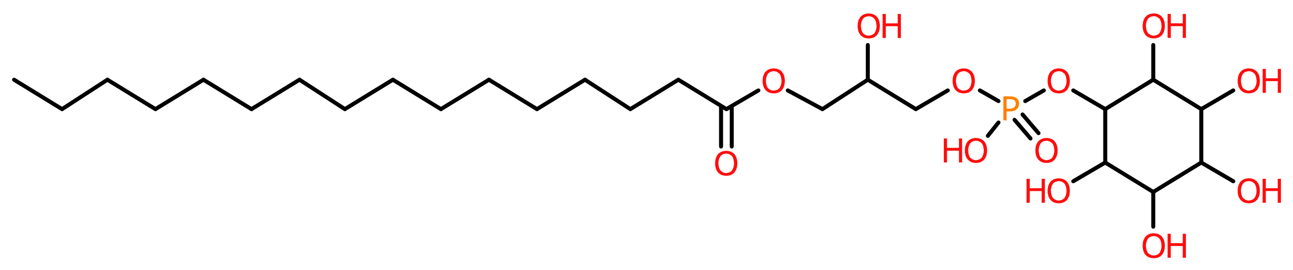

Compound information

Name: LPI 16:0

Formula: C25H49O12P

Ion type: [M-H]-

Precursor m/z: 571.28889

Ccs: 225.12

InChI key:

SMILES:

Num peaks: 6

Instrument information

Database links

Other information

Fosfructose - 30.0 eV 0.722

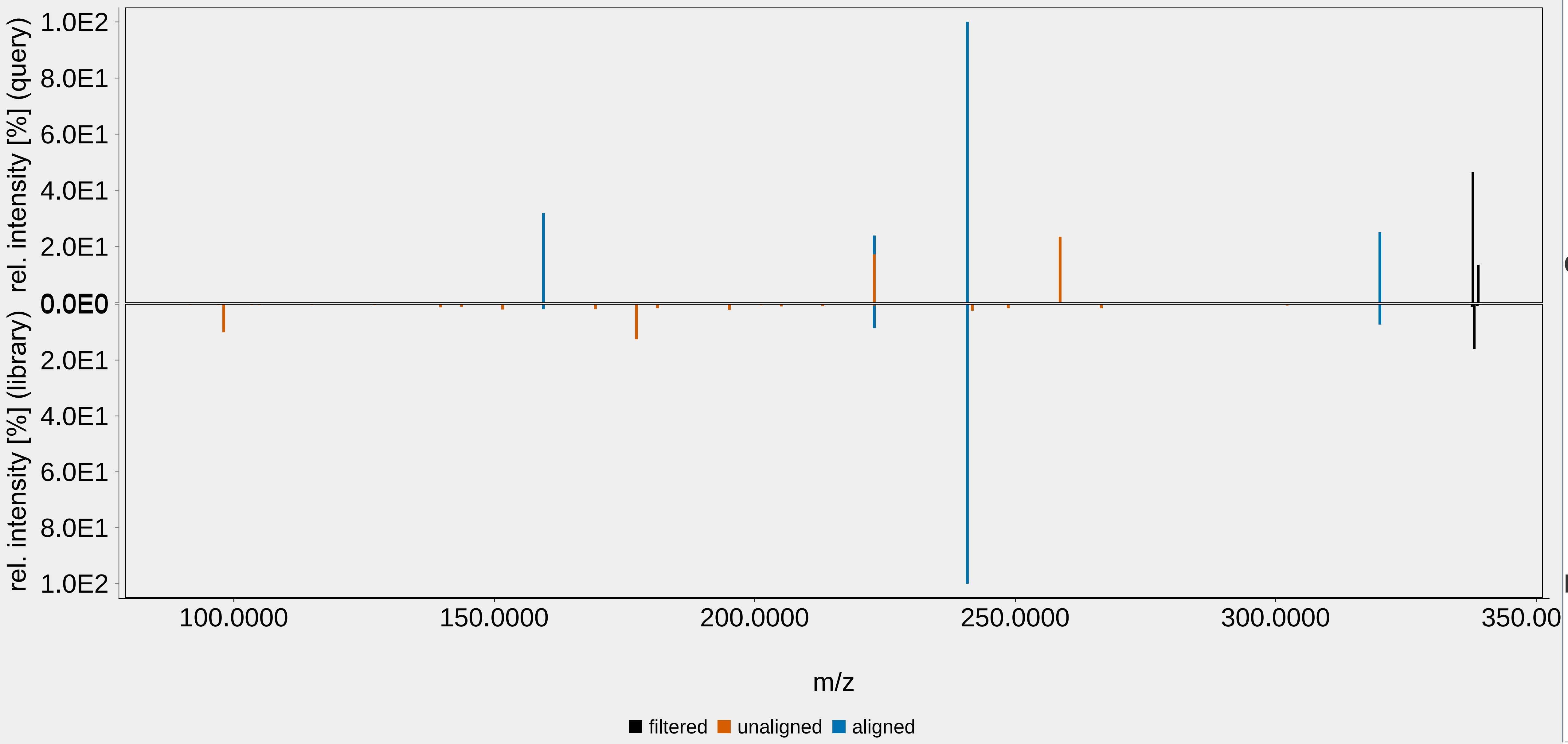

Compound information

Name: Fosfructose - 30.0 eV

Exact mass: 0.0

Ion type: M-H

Precursor m/z: 338.989

Charge: 1

Polarity: Negative

SMILES:

Instrument information

Instrument type: Orbitrap

Ion source: ESI

Ms level: 2

Acquisition: Isolated

Database links

CAS:

Other information

Principal investigator: PI

Data collector: Data Collector

Entry ID: CCMSLIB00006121372

Fosfructose - 30.0 eV

0.720

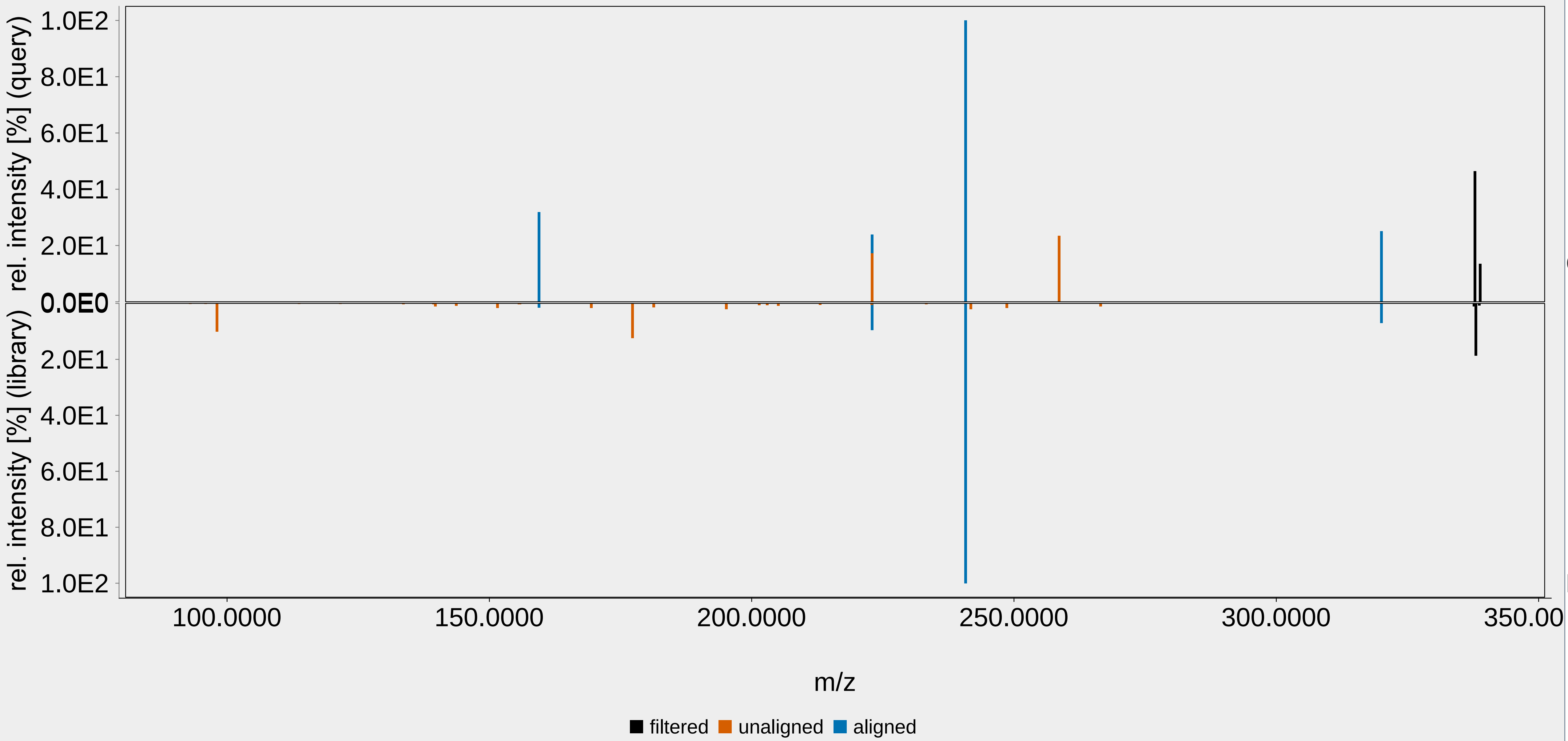

Compound information

Name: Fosfructose - 30.0 eV

Exact mass: 0.0

Ion type: M-H

Precursor m/z: 338.989

Charge: 1

Polarity: Negative

SMILES:

Instrument information

Instrument type: Orbitrap

Ion source: ESI

Ms level: 2

Acquisition: Isolated

Database links

CAS:

Other information

Principal investigator: PI

Data collector: Data Collector

Entry ID: CCMSLIB00006121374

PA 18:1_20:4 **0.891**

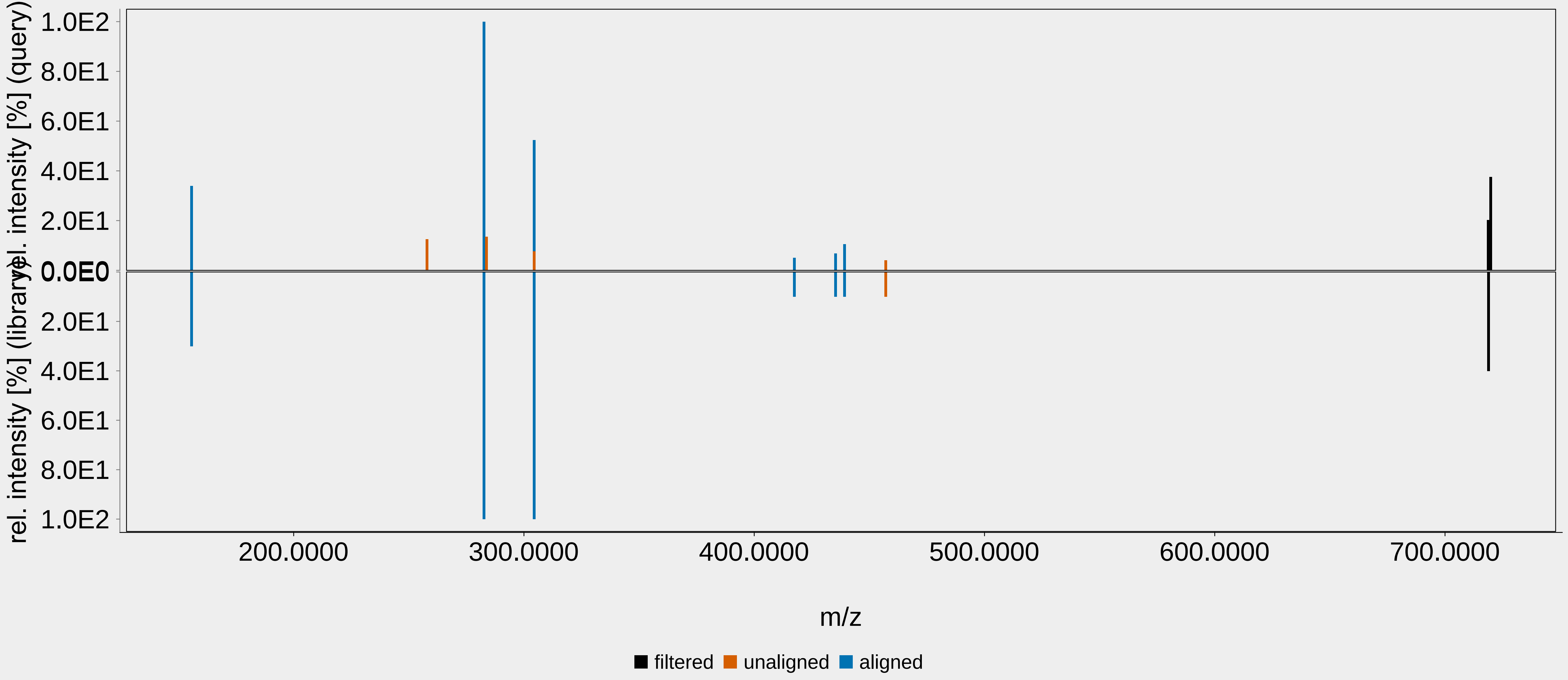

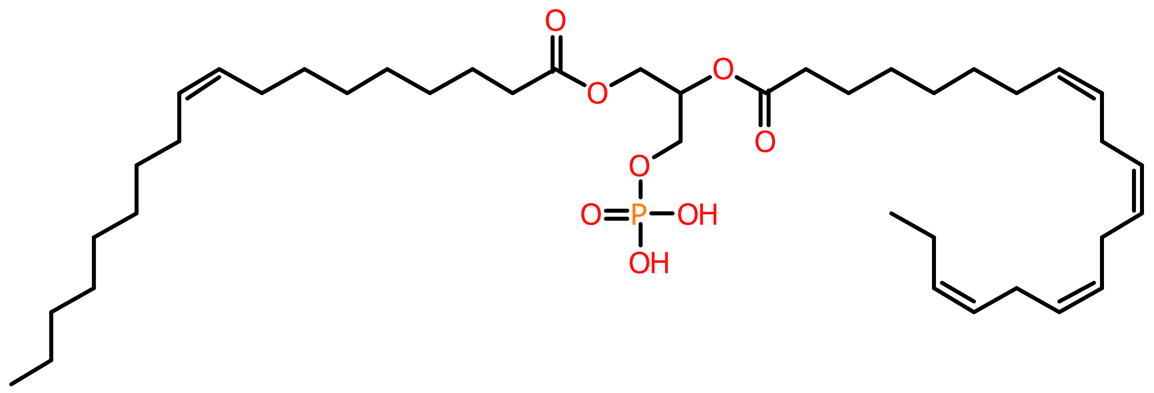

Compound information

Name: PA 18:1_20:4

Formula: C41H71O8P

Ion type: [M-H]-

Precursor m/z: 721.48138

Ccs: 261.27

InChI key:

SMILES:

Num peaks: 8

Instrument information

Database links

Other information

PE(20:1/22:6); [M-H]- C47H79N1O8P1 **0.780**

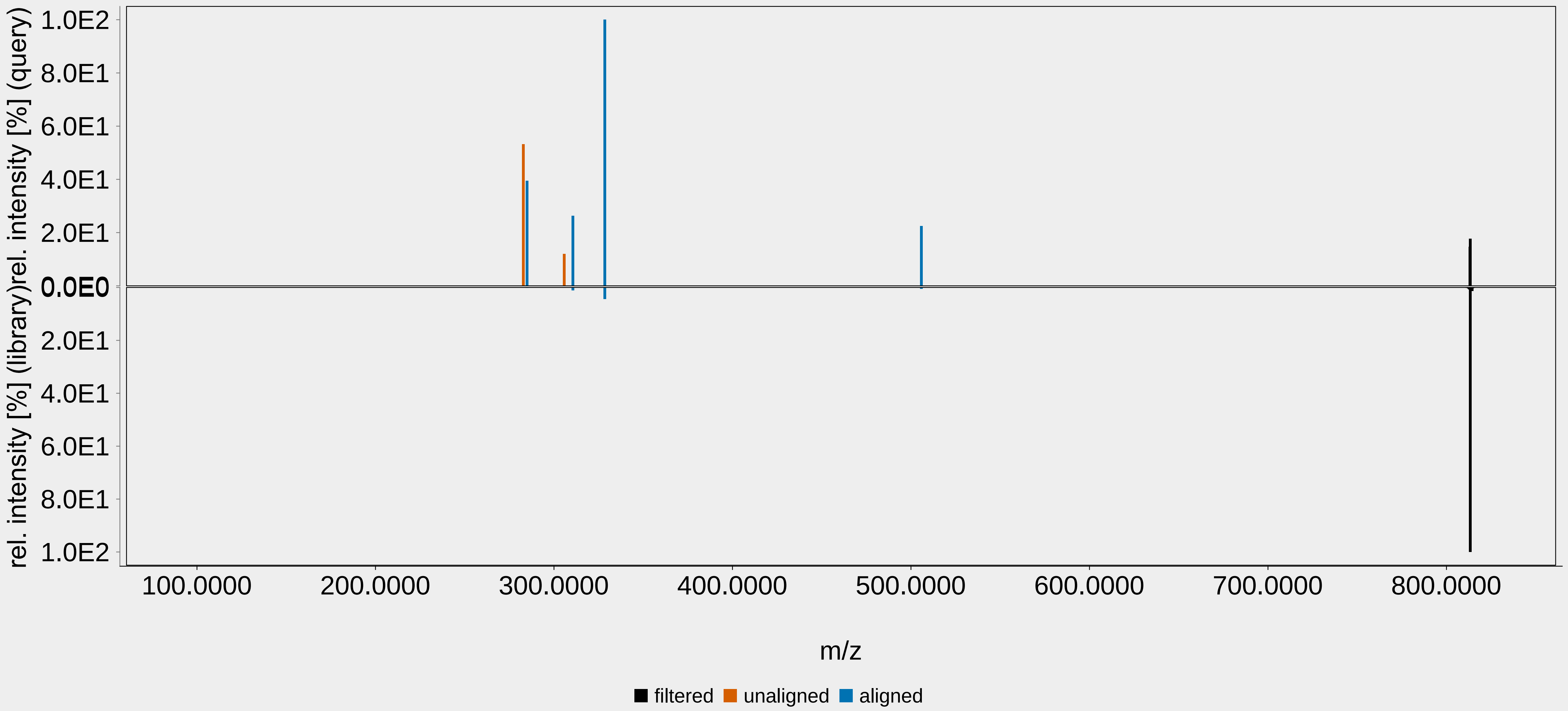

Compound information

Name: PE(20:1/22:6); [M-H]- C47H79N1O8P1

Exact mass: 0.0

Ion type: M-H

Precursor m/z: 816.554

Charge: 1

Polarity: Negative

Instrument information

Instrument type: HCD; Velos

Ion source: LC-ESI

Ms level: 2

Acquisition: Commercial

Database links

Other information

Principal investigator: Thomas Metz

Data collector: Thomas Metz

Entry ID: CCMSLIB00010073329

PS(18:1/18:1); [M-H]- C42H77N1O10P1 **0.830**

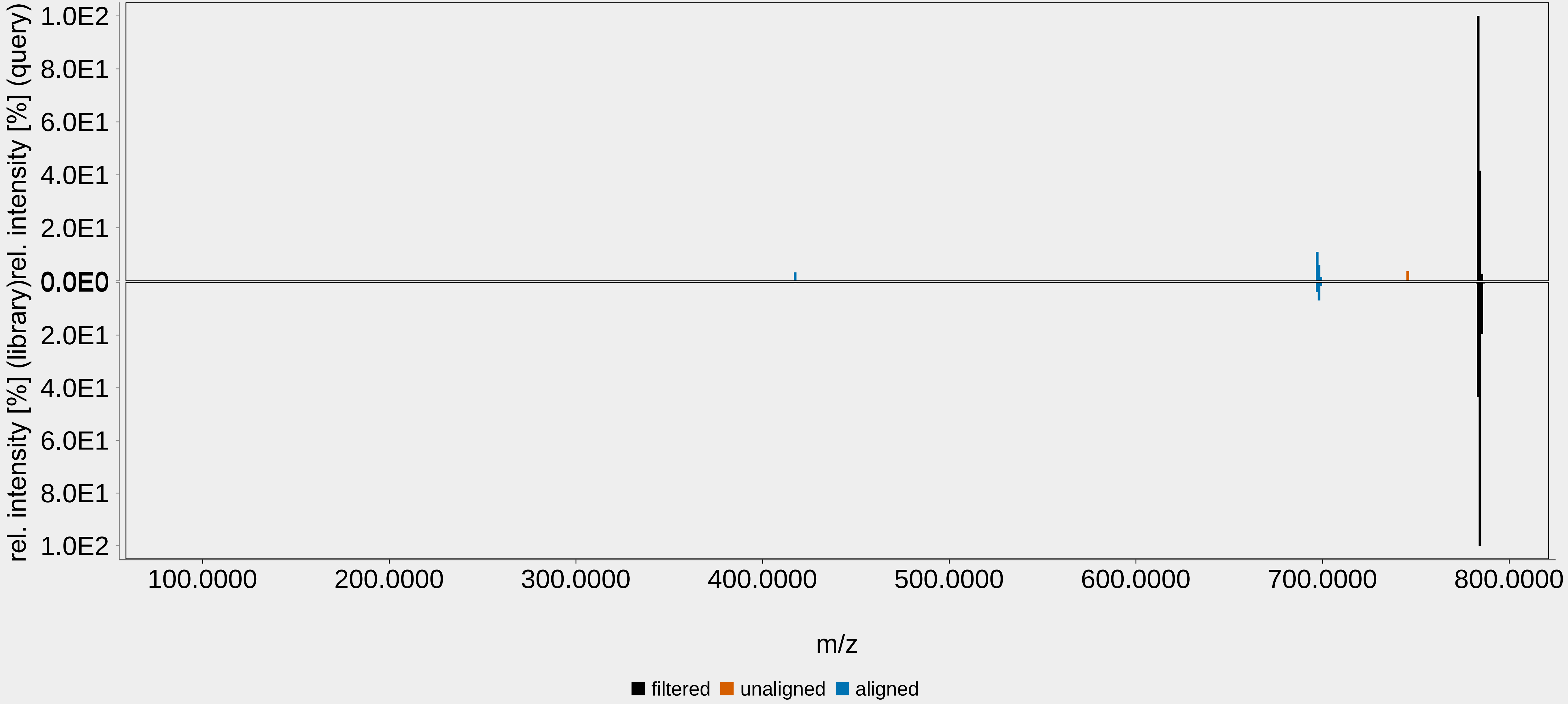

Compound information

Name: PS(18:1/18:1); [M-H]- C42H77N1O10P1

Exact mass: 0.0

Ion type: M-H

Precursor m/z: 786.529

Charge: 1

Polarity: Negative

Instrument information

Instrument type: HCD; Velos

Ion source: LC-ESI

Ms level: 2

Acquisition: Commercial

Database links

Other information

Principal investigator: Thomas Metz

Data collector: Thomas Metz

Entry ID: CCMSLIB00010084418

PS 18:1_18:1 0.869

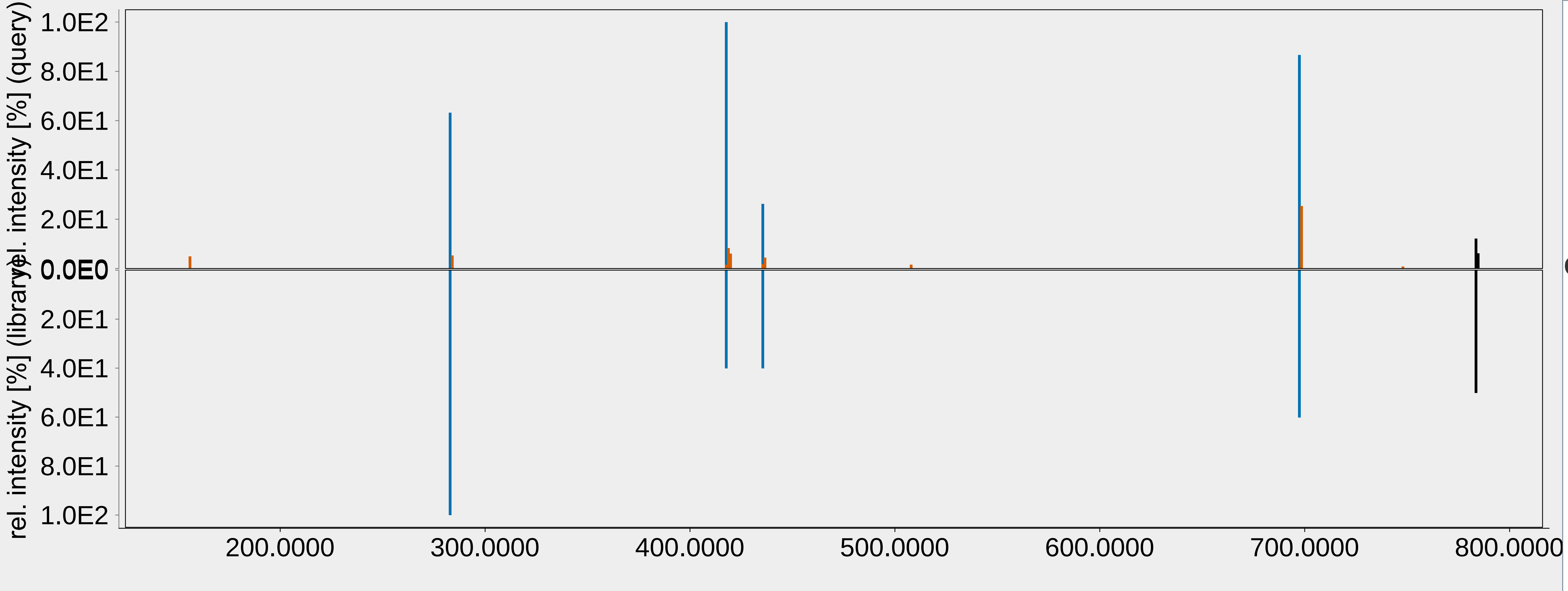

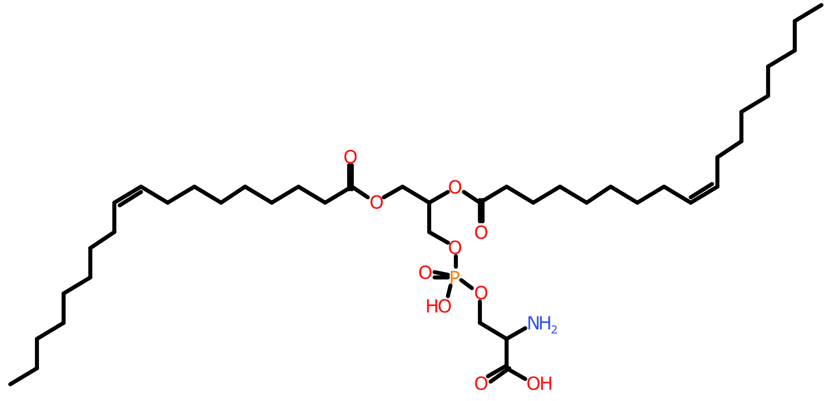

Compound information

Name: PS 18:1_18:1

Formula: C42H78NO10P

Ion type: [M-H]-

Precursor m/z: 786.52906

Ccs: 275.77

InChI key:

SMILES:

Num peaks: 5

Instrument information

Database links

Other information

UPD-alpha-D-Galactose - 30.0 eV

0.752

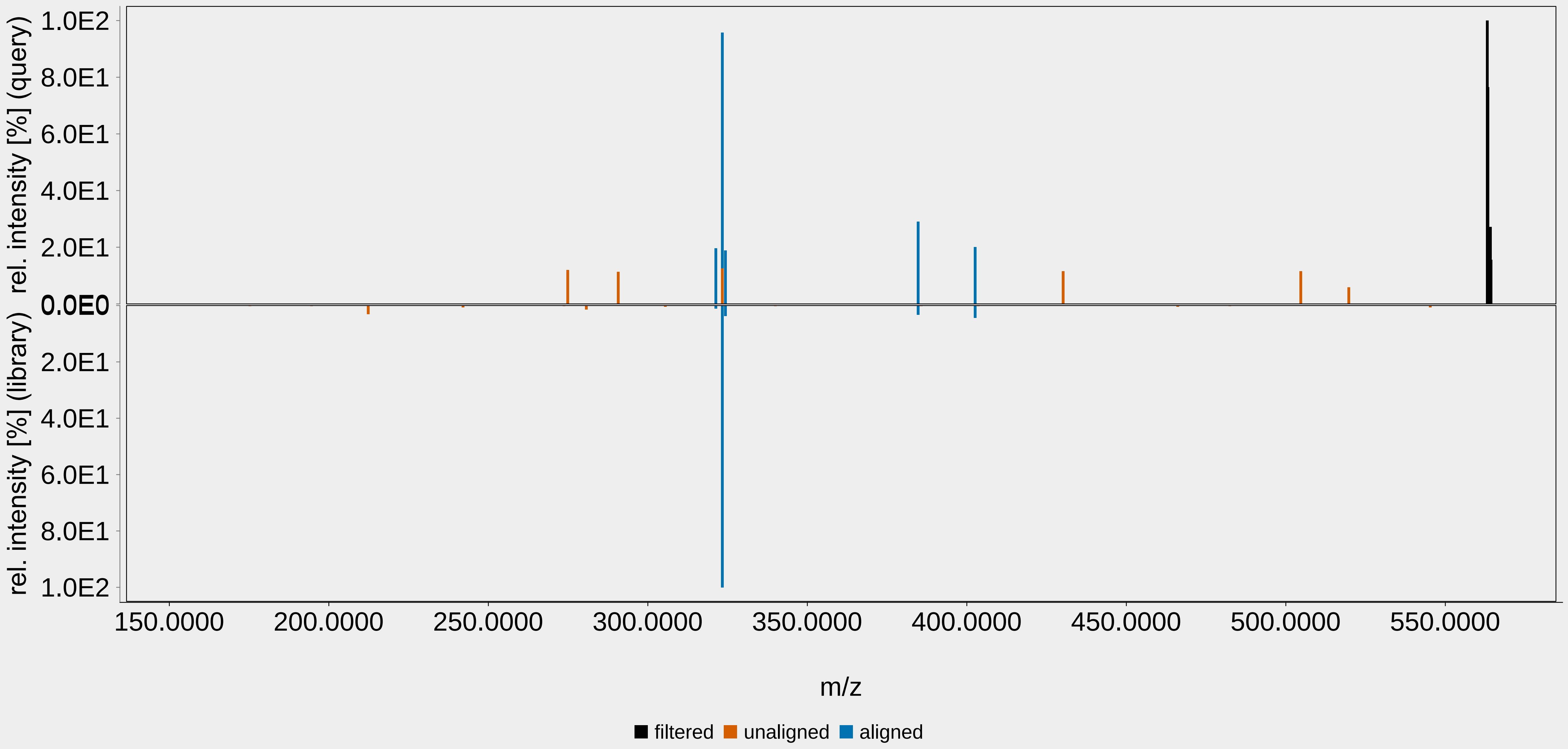

Compound information

Name: UPD-alpha-D-Galactose - 30.0 eV

Exact mass: 0.0

Ion type: Unknown

Precursor m/z: 565.048

Charge: 1

Polarity: Negative

SMILES:

Instrument information

Instrument type: Orbitrap

Ion source: ESI

Ms level: 2

Acquisition: Isolated

Database links

CAS:

Other information

Principal investigator: PI

Data collector: Data Collector

Entry ID: CCMSLIB00006113004

UPD-alpha-D-Galactose - 30.0 eV

0.757

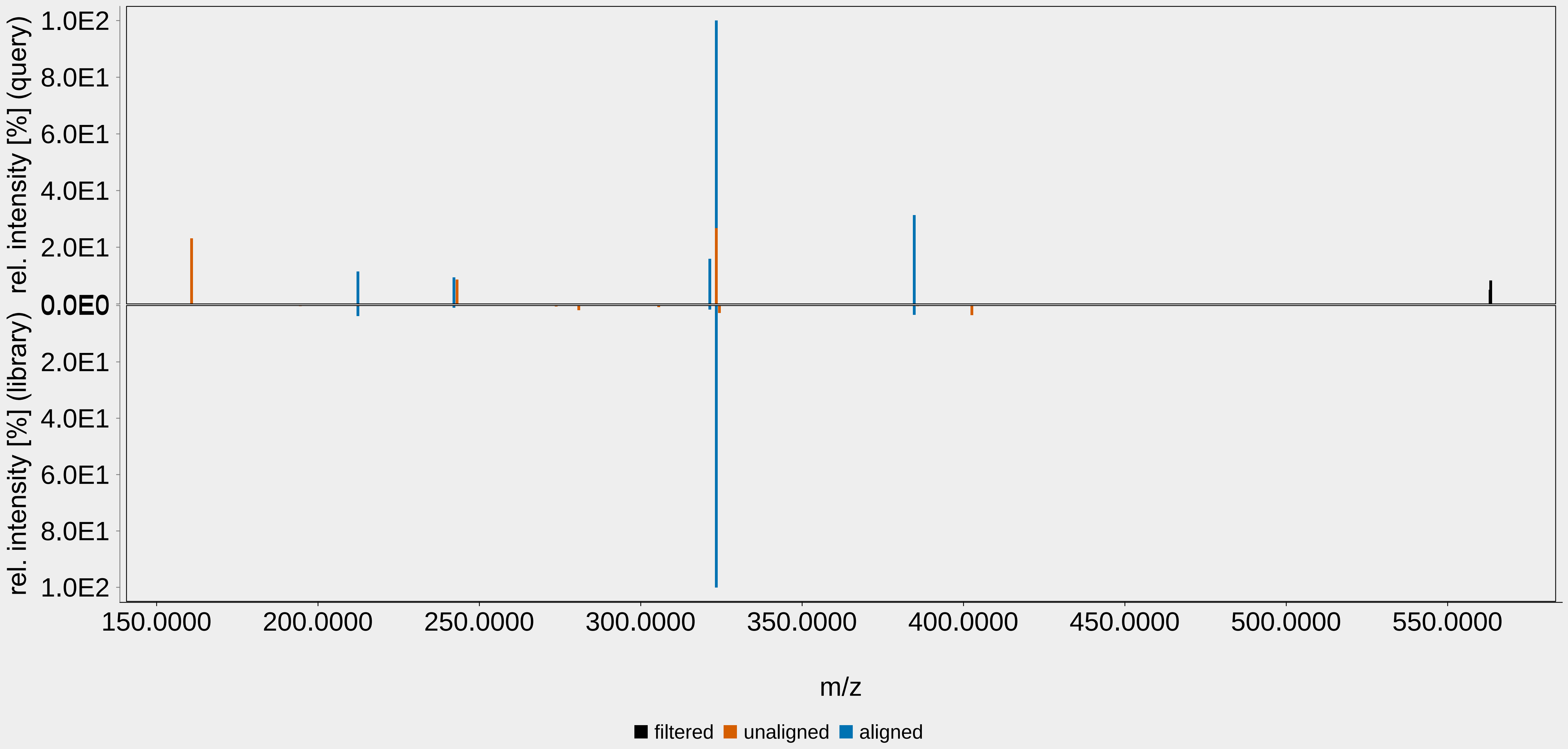

Compound information

Name: UPD-alpha-D-Galactose - 30.0 eV

Exact mass: 0.0

Ion type: Unknown

Precursor m/z: 565.048

Charge: 1

Polarity: Negative

SMILES:

Instrument information

Instrument type: Orbitrap

Ion source: ESI

Ms level: 2

Acquisition: Isolated

Database links

CAS:

Other information

Principal investigator: PI

Data collector: Data Collector

Entry ID: CCMSLIB00006113006

Spectral Match to 1,2-Di(4Z,7Z,10Z,13Z,16Z,19Z-docosahexaenoyl)-sn-glycero-3-phosphoethanolamine from NIST14

0.871

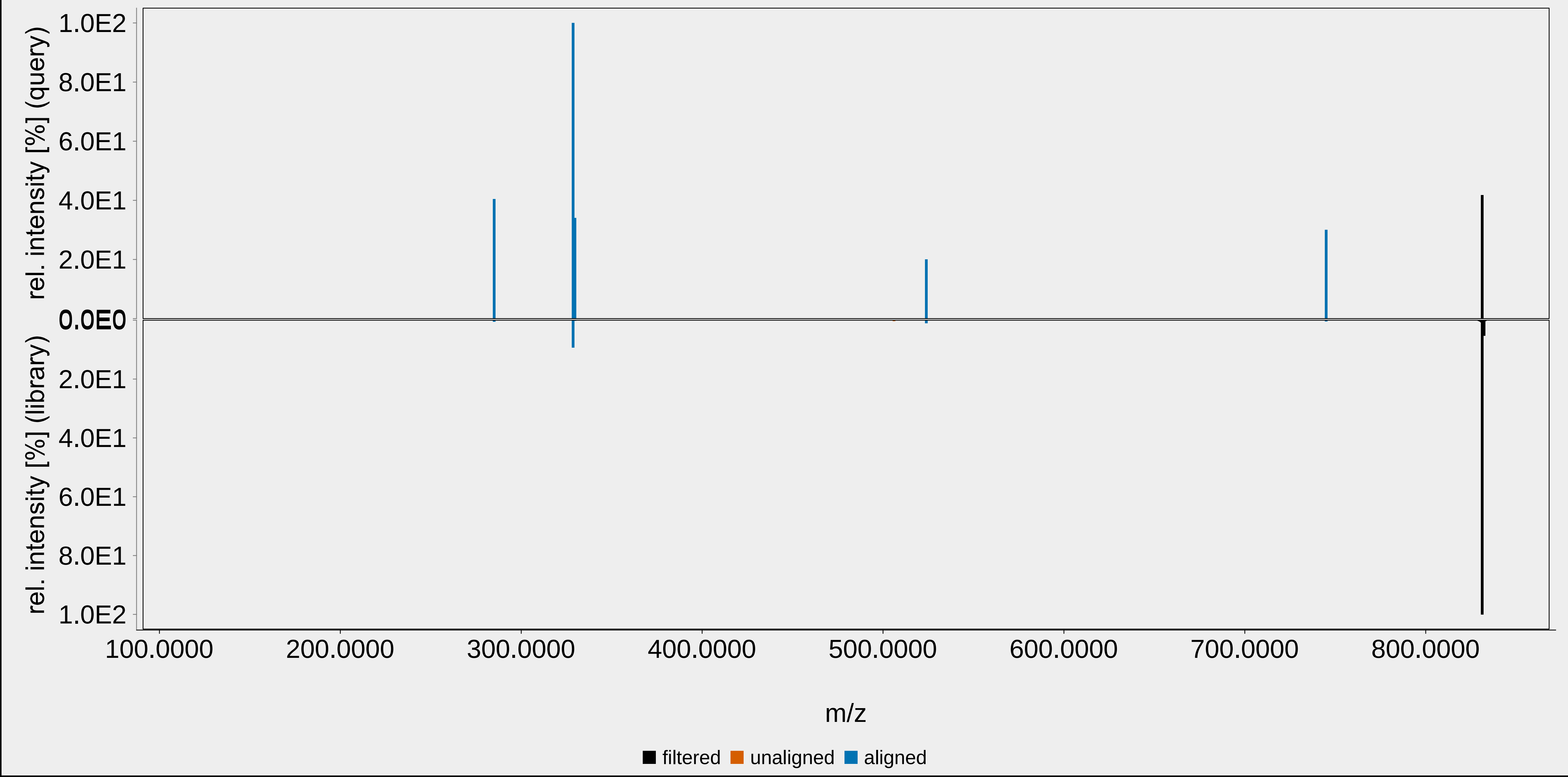

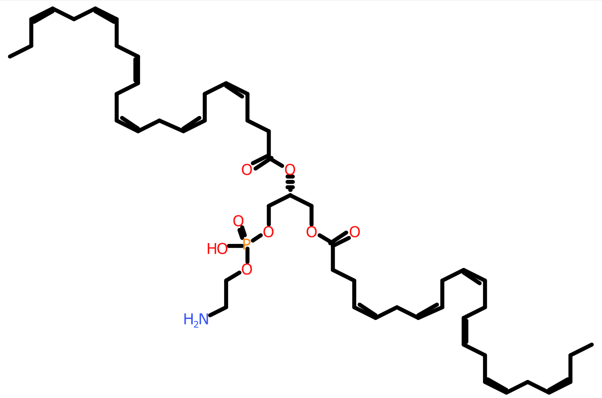

Compound information

Name: Spectral Match to

Exact mass: 835.515

Ion type: M-H

Precursor m/z: 834.512

Charge: 1

Polarity: Negative

InChI:

SMILES:

Instrument information

Instrument type: qTof

Ion source: ESI

Ms level: 2

Acquisition: Isolated

Database links

CAS: 123284811

Other information

Principal investigator: Data from Katrina

Data collector: Data deposited by alchemistmatt

Entry ID: CCMSLIB00003140104

PI(20:4/0:0); [M-H]- C29H48O12P1

0.742

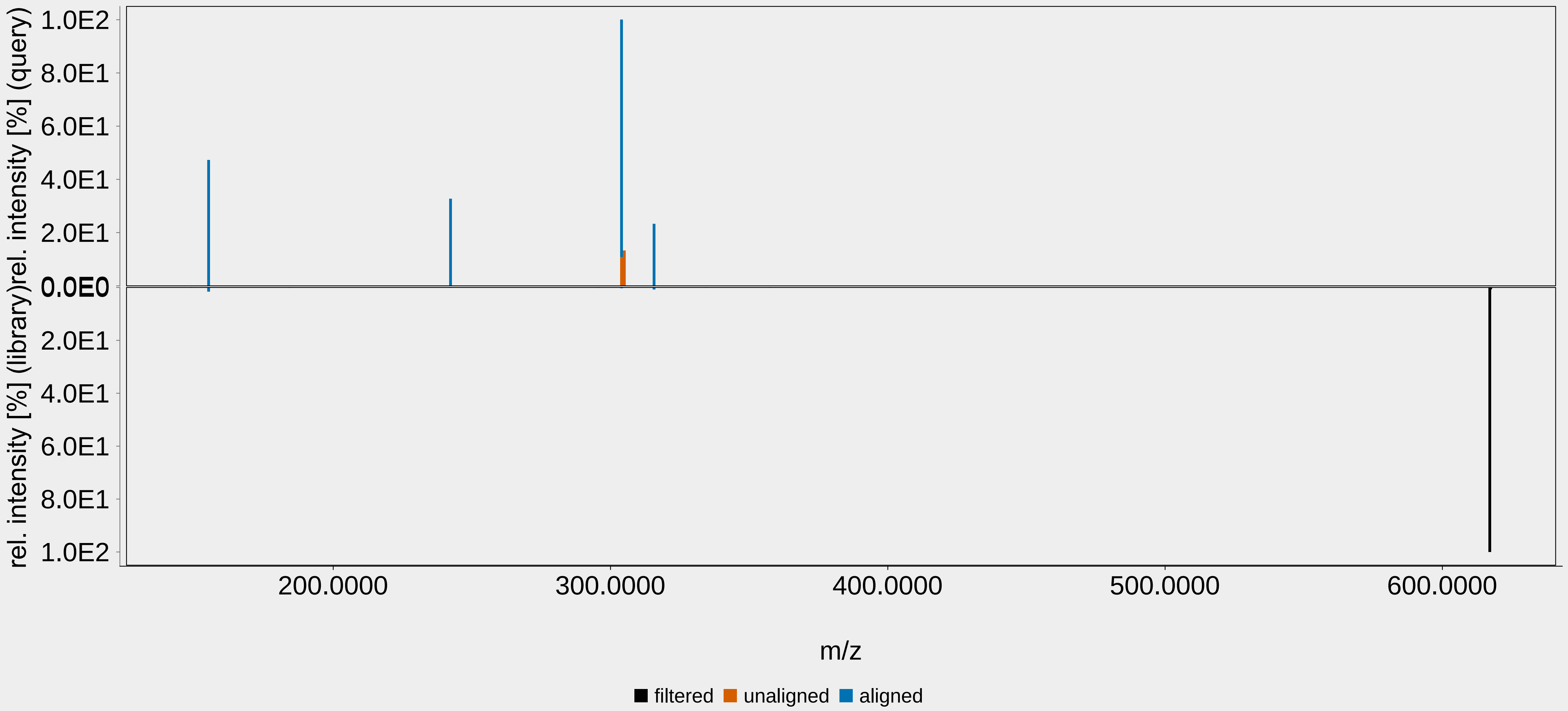

Compound information

Name: PI(20:4/0:0); [M-H]- C29H48O12P1

Exact mass: 0.0

Ion type: M-H

Precursor m/z: 619.288

Charge: 1

Polarity: Negative

Instrument information

Instrument type: HCD; Velos

Ion source: LC-ESI

Ms level: 2

Acquisition: Commercial

Database links

Other information

Principal investigator: Thomas Metz

Data collector: Thomas Metz

Entry ID: CCMSLIB00010084582

LPI 20:4

0.928

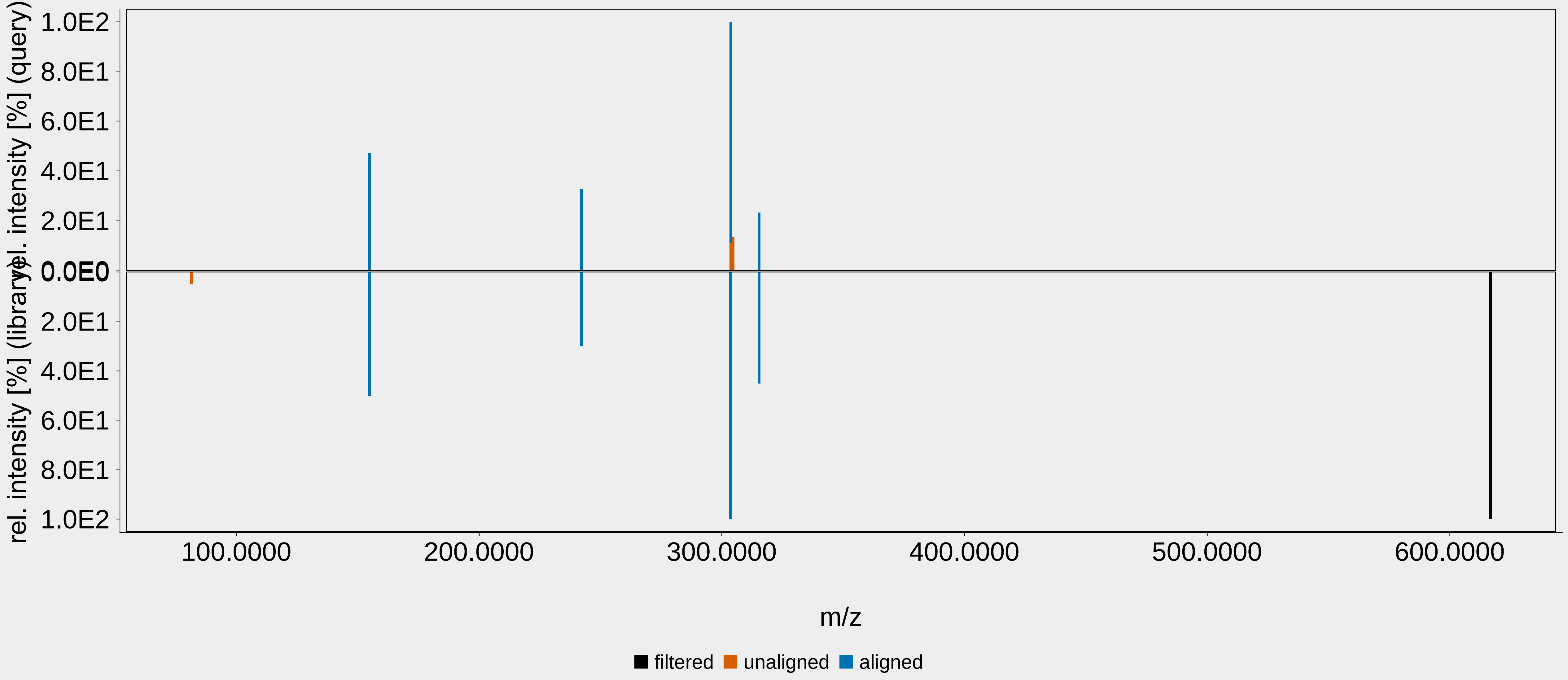

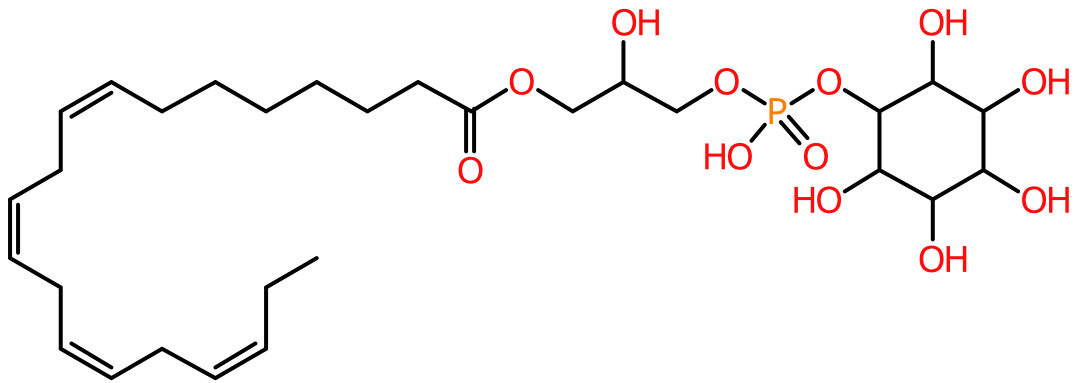

Compound information

Name: LPI 20:4

Formula: C29H49O12P

Ion type: [M-H]-

Precursor m/z: 619.28889

Ccs: 233.07

InChI key:

SMILES:

Num peaks: 6

Instrument information

Database links

Other information

PE(18:2/0:0); [M-H]- C23H43N1O7P1 0.872

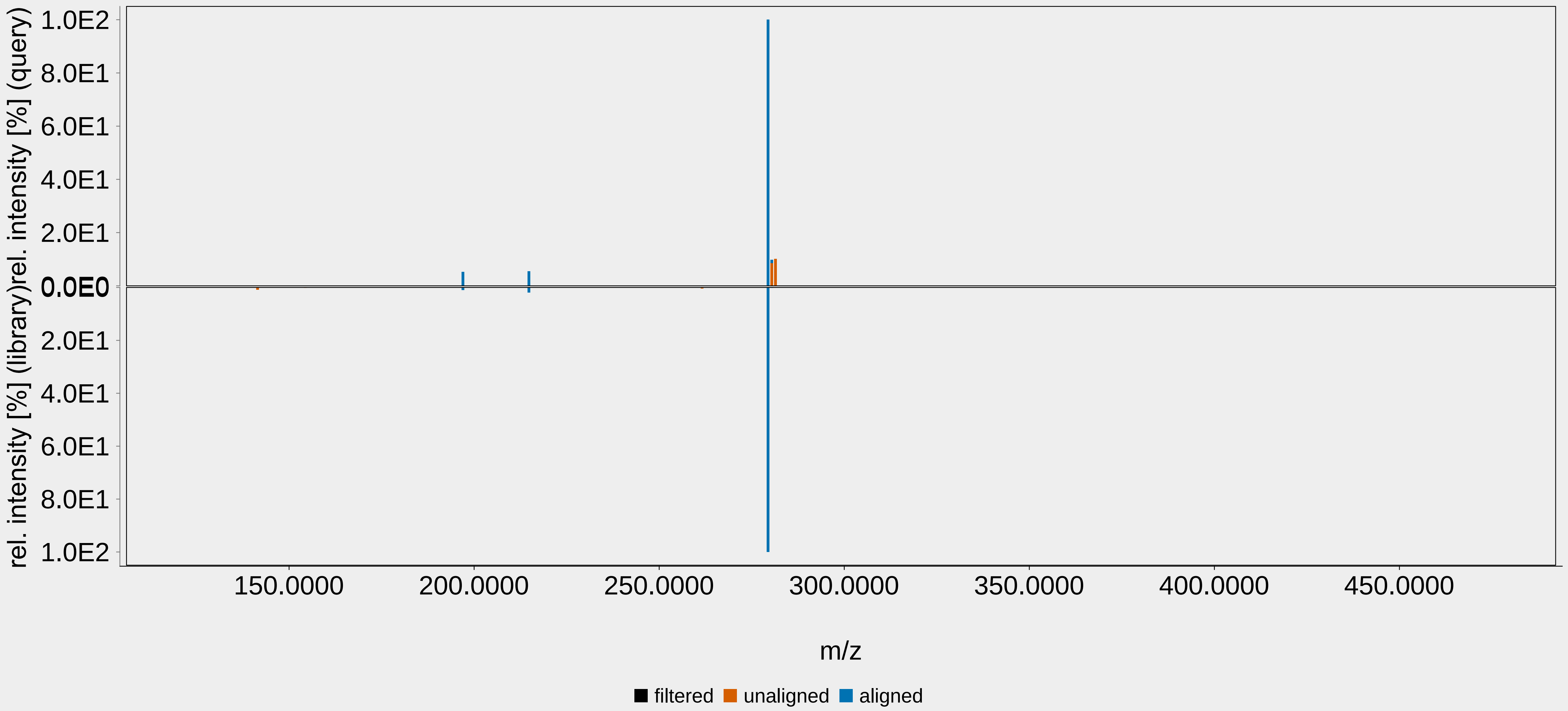

Compound information

Name: PE(18:2/0:0); [M-H]- C23H43N1O7P1

Exact mass: 0.0

Ion type: M-H

Precursor m/z: 476.278

Charge: 1

Polarity: Negative

Instrument information

Instrument type: HCD; Lumos

Ion source: LC-ESI

Ms level: 2

Acquisition: Commercial

Database links

Other information

Principal investigator: Thomas Metz

Data collector: Thomas Metz

Entry ID: CCMSLIB00010059116

PE(18:2/0:0); [M-H]- C23H43N1O7P1 **0.875**

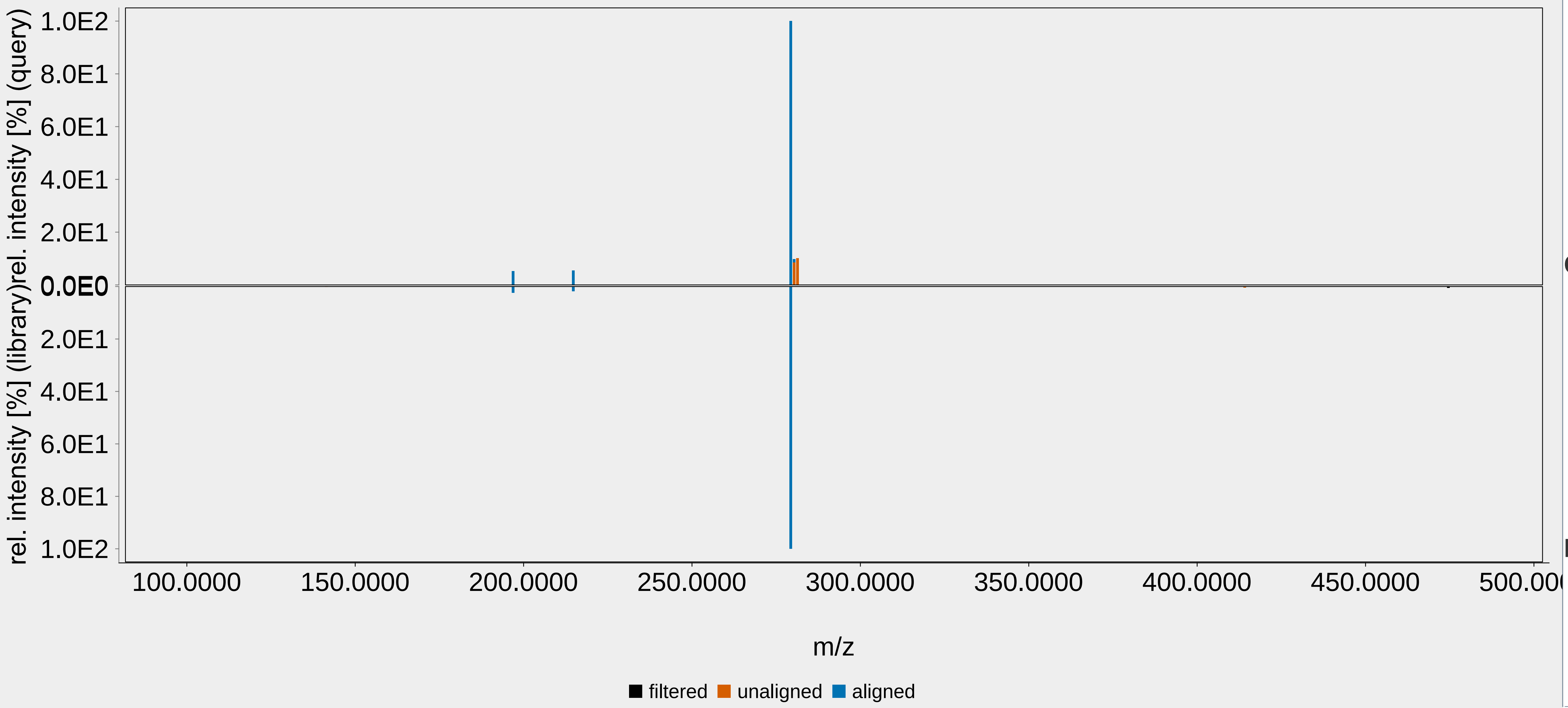

Compound information

Name: PE(18:2/0:0); [M-H]- C23H43N1O7P1

Exact mass: 0.0

Ion type: M-H

Precursor m/z: 476.278

Charge: 1

Polarity: Negative

Instrument information

Instrument type: CID; Lumos

Ion source: LC-ESI

Ms level: 2

Acquisition: Commercial

Database links

Other information

Principal investigator: Thomas Metz

Data collector: Thomas Metz

Entry ID: CCMSLIB00010059801

Guanosine triphosphate

0.779

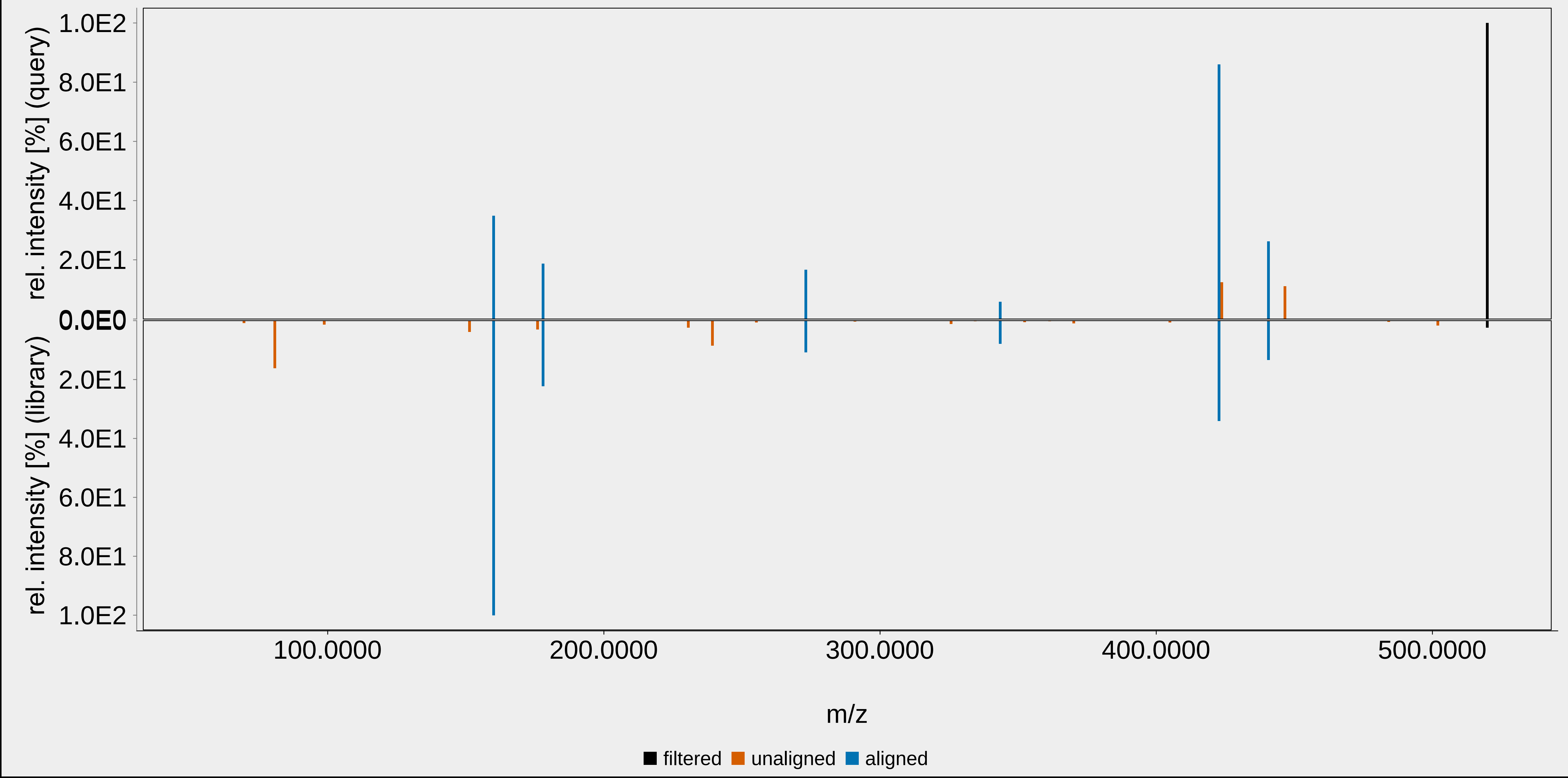

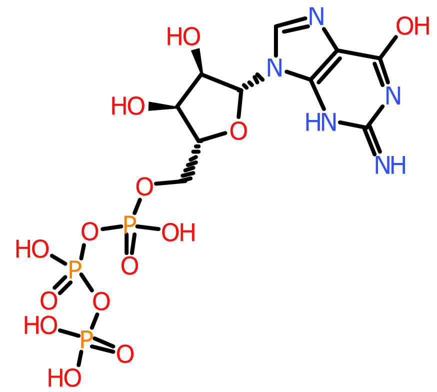

Compound information

Name: Guanosine triphosphate

Formula: C10H16N5O14P3

Ion type: [M-H]-

Precursor m/z: 521.981868542491

RT: 6.6404014

Polarity: negative

InChI:

InChI key:

SMILES:

Instrument information

Instrument type: LC-ESI-QFT

Instrument: Thermo Q-Exactive Plus

Ms level: MS2

Collision energy: 30.0 eV

Database links

Other information

Principal investigator: Prasad Phapale

Data collector: Prasad Phapale

Guanosine triphosphate

0.781

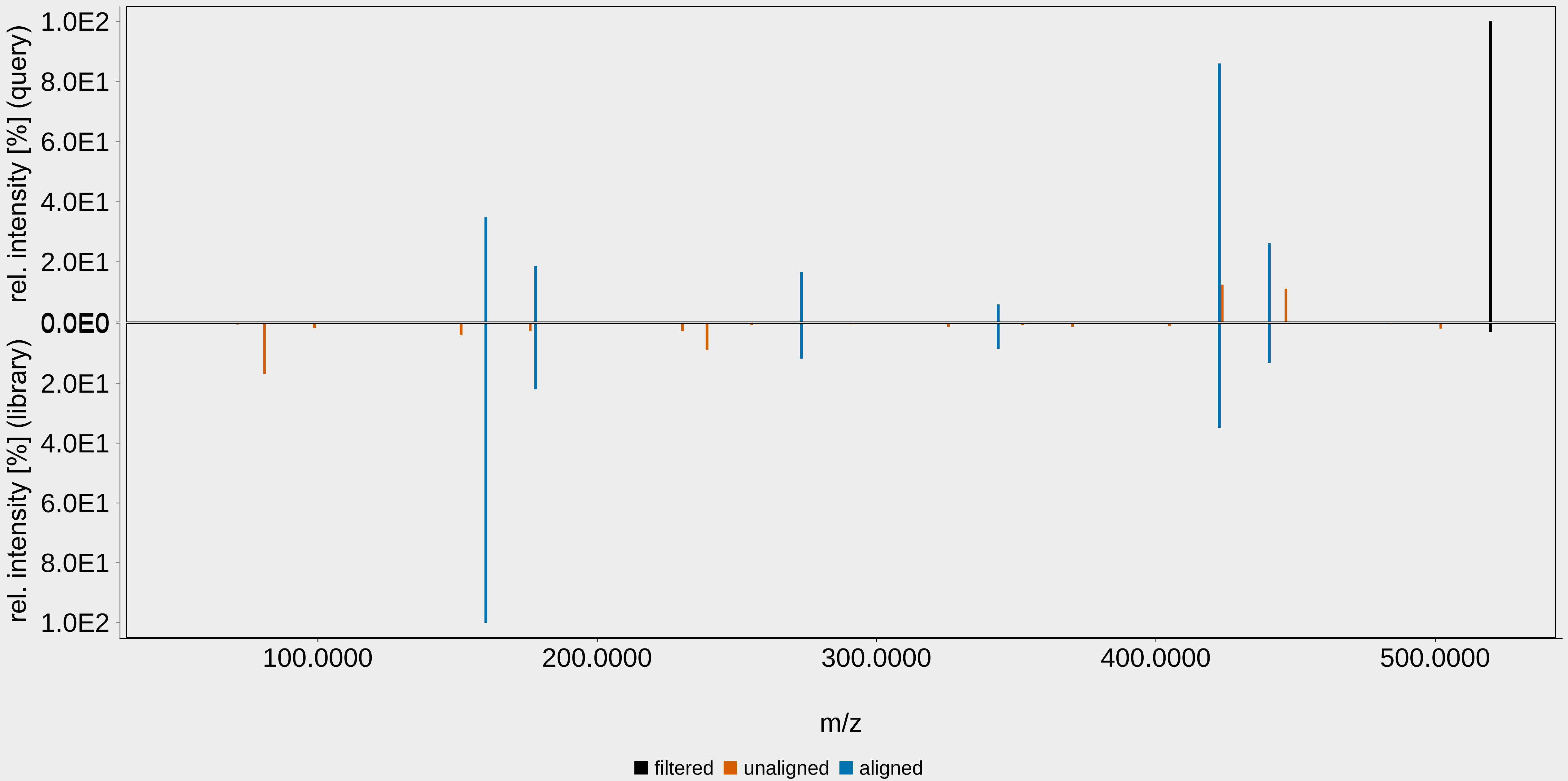

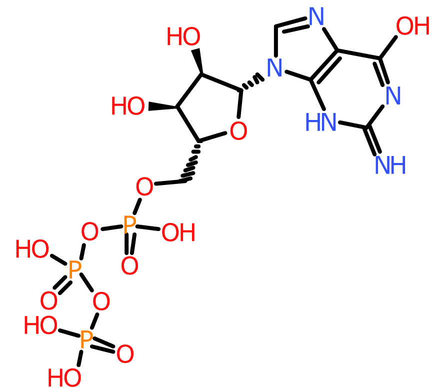

Compound information

Name: Guanosine triphosphate

Formula: C10H16N5O14P3

Ion type: [M-H]-

Precursor m/z: 521.981890480549

RT: 6.6717806

Polarity: negative

InChI:

InChI key:

SMILES:

Instrument information

Instrument type: LC-ESI-QFT

Instrument: Thermo Q-Exactive Plus

Ms level: MS2

Collision energy: 30.0 eV

Database links

Other information

Principal investigator: Prasad Phapale

Data collector: Prasad Phapale

PA 20:4_22:6 **0.934**

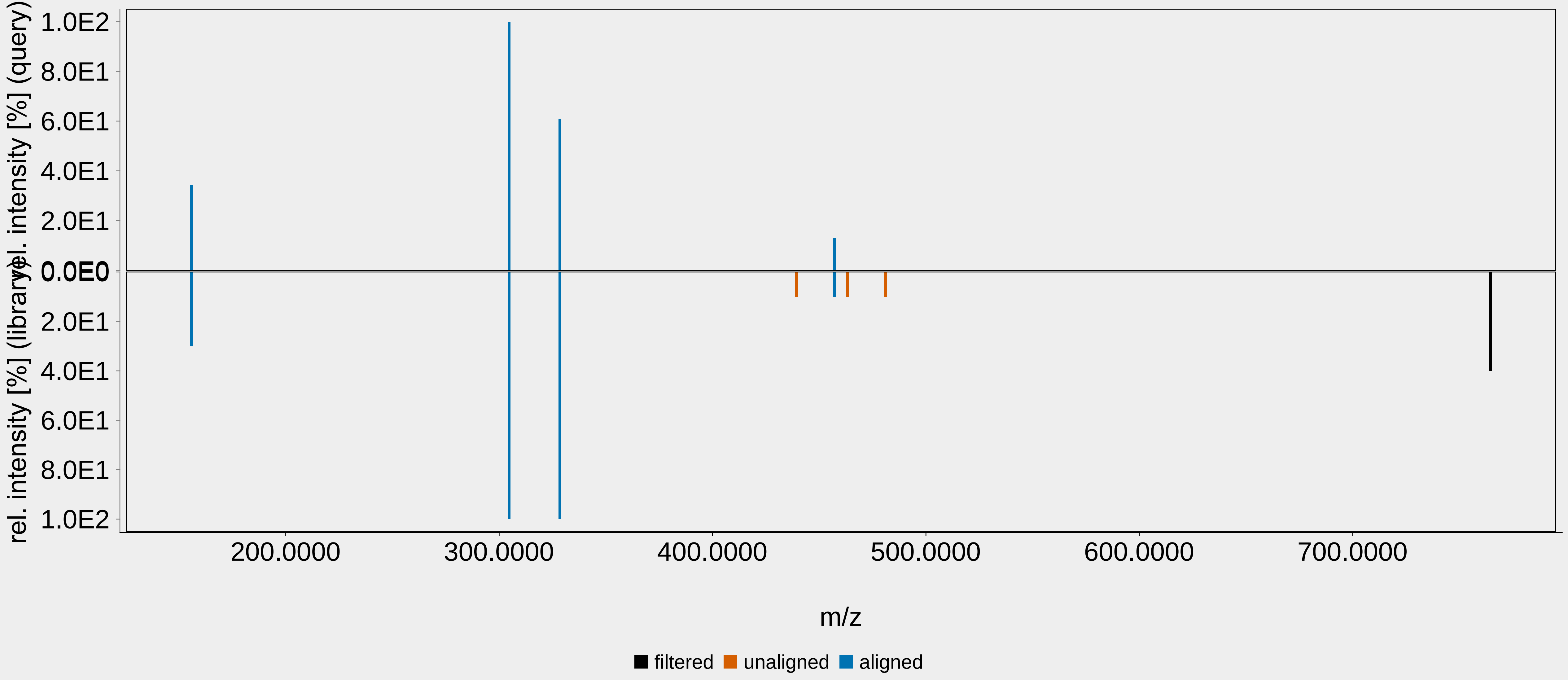

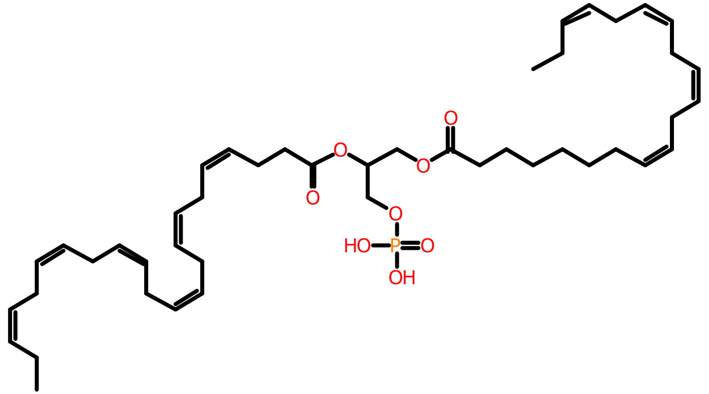

Compound information

Name: PA 20:4_22:6

Formula: C45H69O8P

Ion type: [M-H]-

Precursor m/z: 767.46573

Ccs: 264.96

InChI key:

SMILES:

Num peaks: 8

Instrument information

Database links

Other information

Spectral Match to 1-(9Z-Octadecenoyl)-sn-glycero-3-phospho-(1'-myo-inositol) from NIST14

0.804

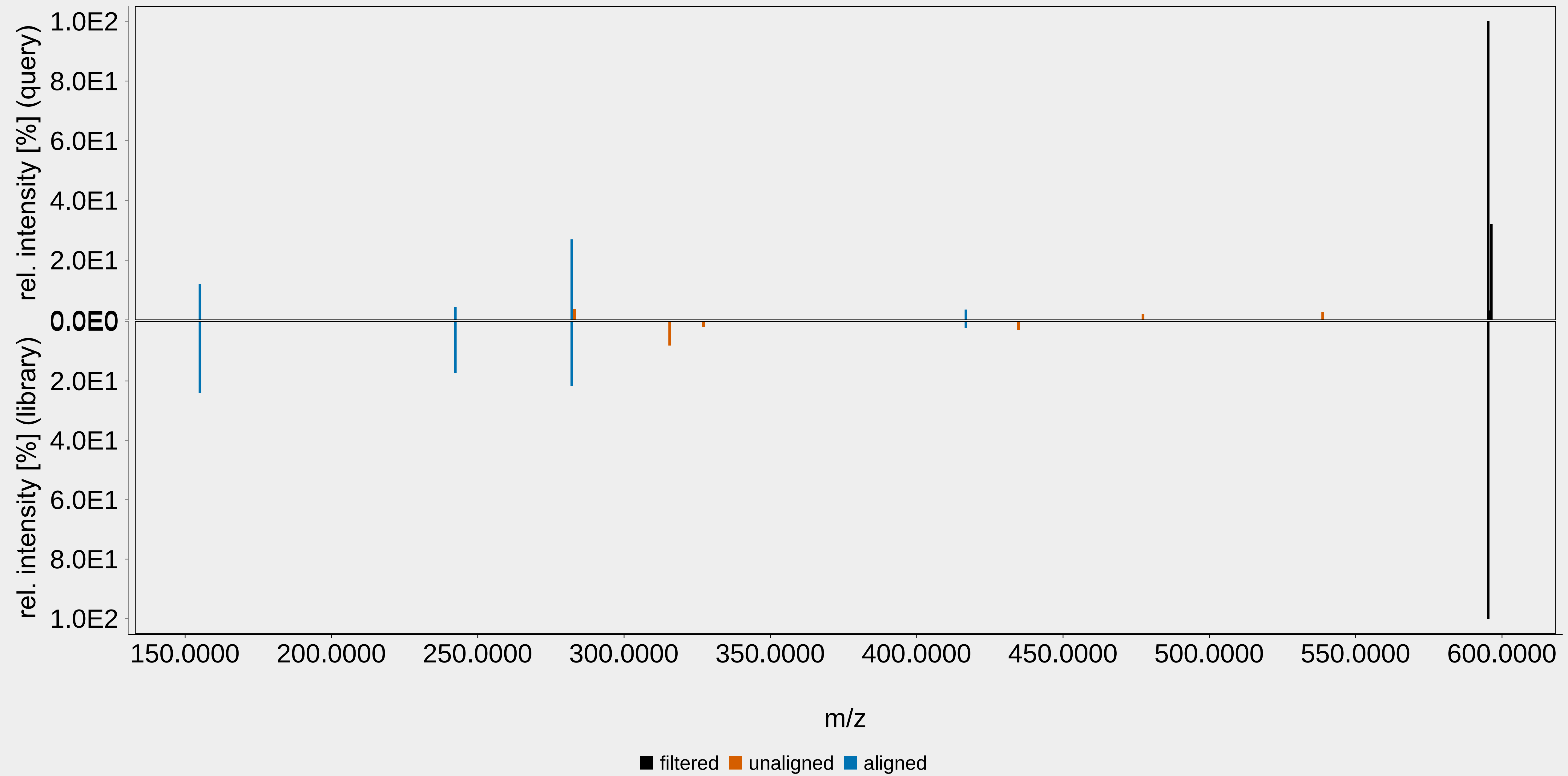

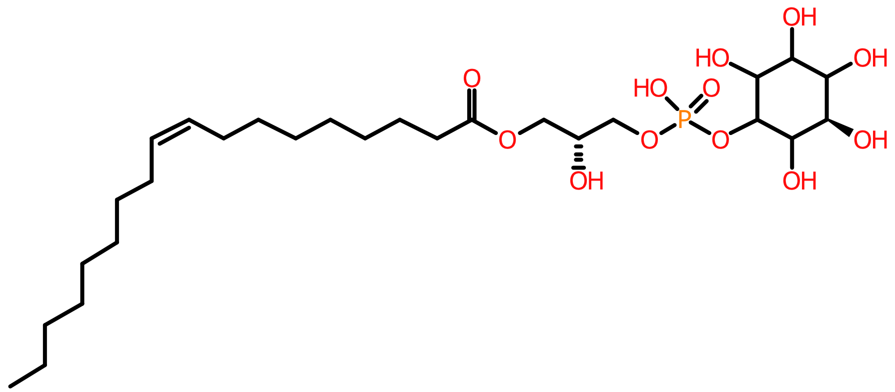

Compound information

Name: Spectral Match to

Exact mass: 598.312

Ion type: M-H

Precursor m/z: 597.3

Charge: 1

Polarity: Negative

InChI:

SMILES:

Instrument information

Instrument type: Ion Trap

Ion source: ESI

Ms level: 2

Acquisition: Isolated

Database links

CAS: 1114770158

Other information

Principal investigator: Data from Pieter

Data collector: Data deposited by lfnothias

Entry ID: CCMSLIB00003134872

LPI 18:1 **0.934**

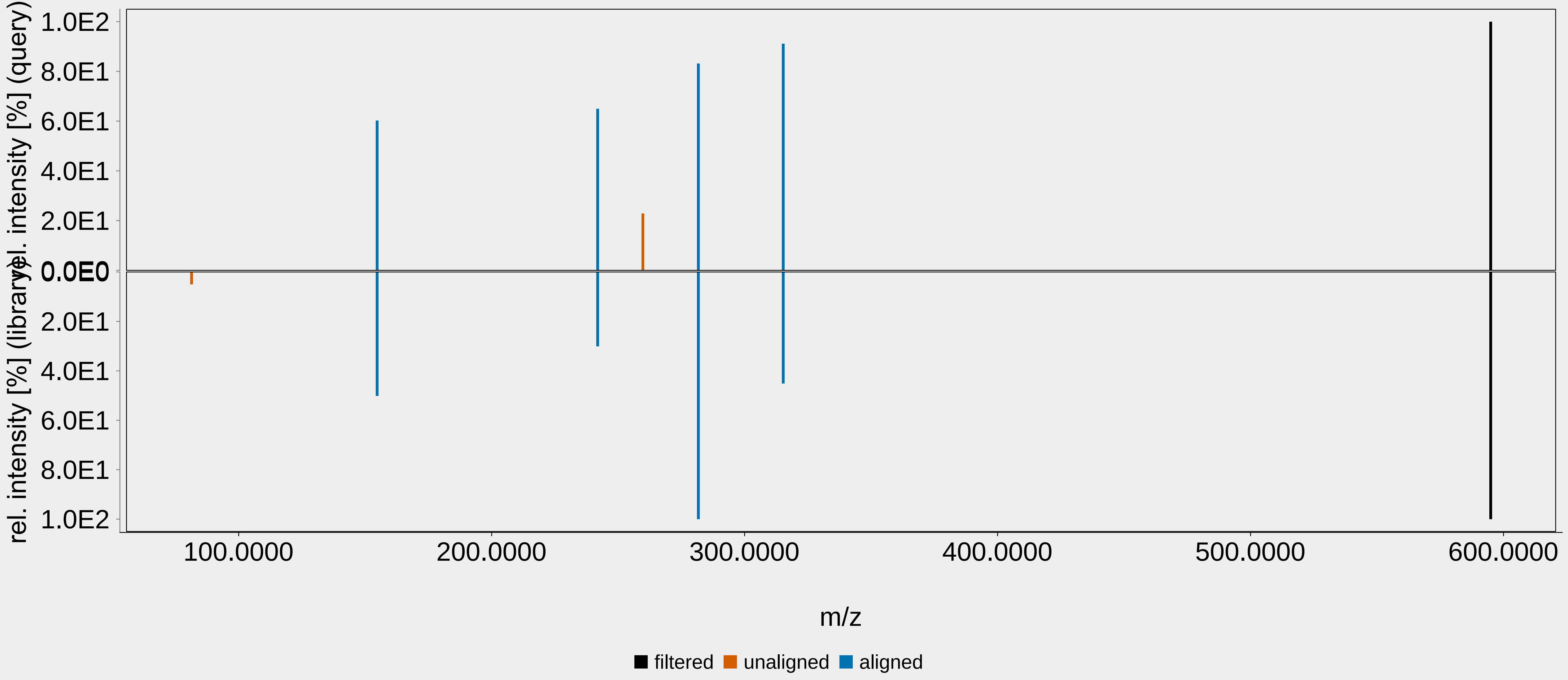

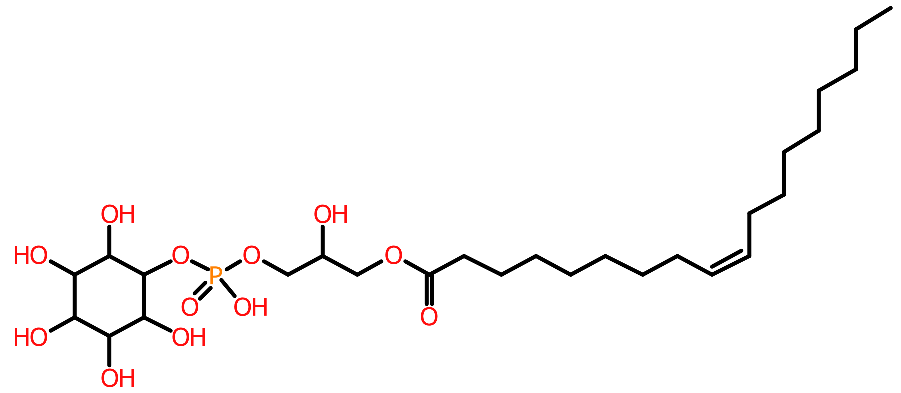

Compound information

Name: LPI 18:1

Formula: C27H51O12P

Ion type: [M-H]-

Precursor m/z: 597.30454

Ccs: 231.61

InChI key:

SMILES:

Num peaks: 6

Instrument information

Database links

Other information

PS 22:0_18:1 **0.798**

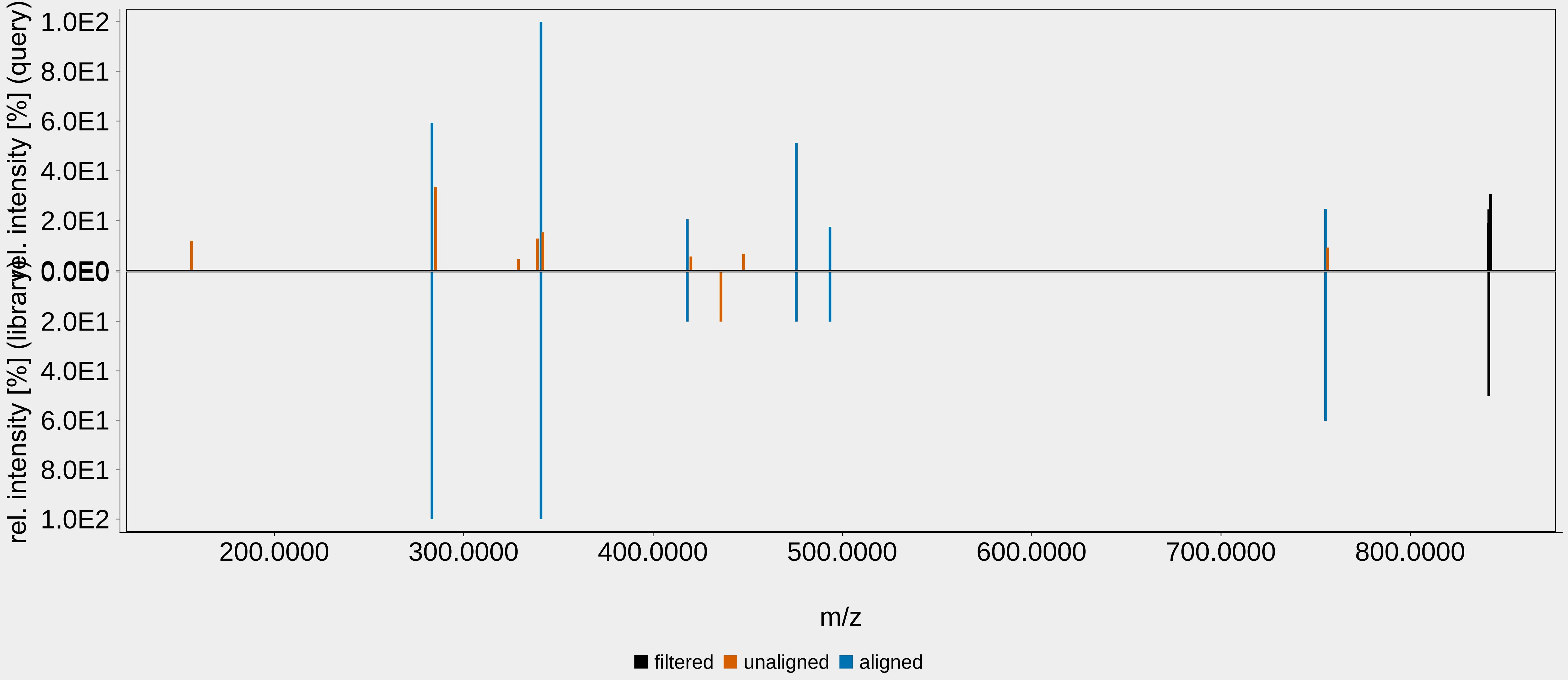

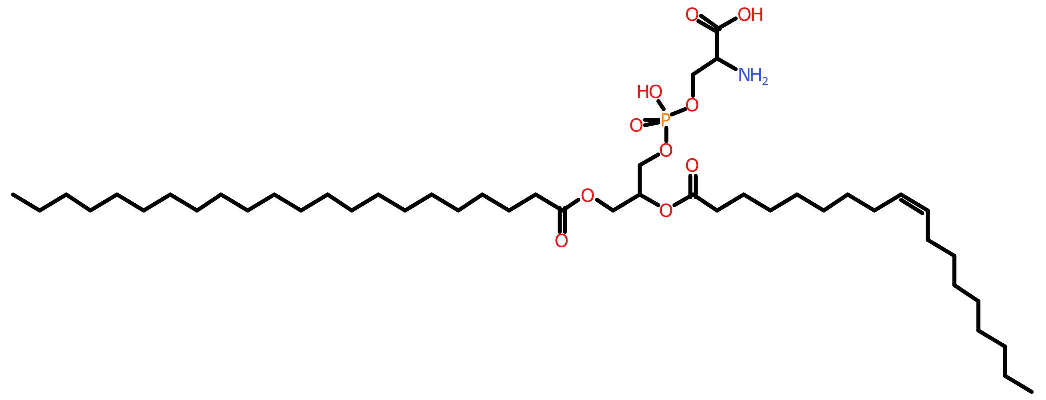

Compound information

Name: PS 22:0_18:1

Formula: C46H88NO10P

Ion type: [M-H]-

Precursor m/z: 844.60731

Ccs: 289.29

InChI key:

SMILES:

Num peaks: 8

Instrument information

Database links

Other information

PE(18:0/20:1); [M-H]- C43H83N1O8P1 0.893

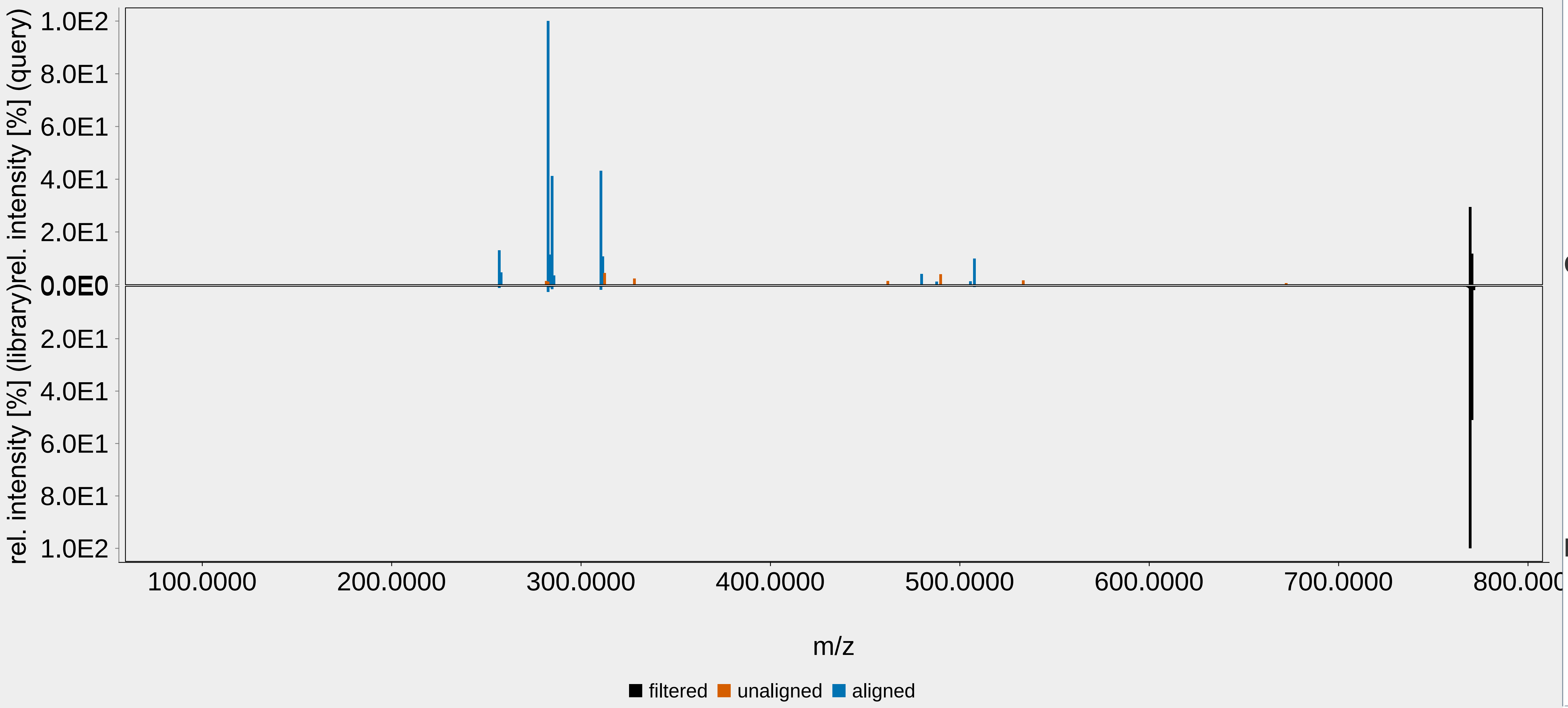

Compound information

Name: PE(18:0/20:1); [M-H]- C43H83N1O8P1

Exact mass: 0.0

Ion type: M-H

Precursor m/z: 772.586

Charge: 1

Polarity: Negative

Instrument information

Instrument type: HCD; Velos

Ion source: LC-ESI

Ms level: 2

Acquisition: Commercial

Database links

Other information

Principal investigator: Thomas Metz

Data collector: Thomas Metz

Entry ID: CCMSLIB00010068369

PE(18:0/20:1); [M-H]- C43H83N1O8P1 0.942

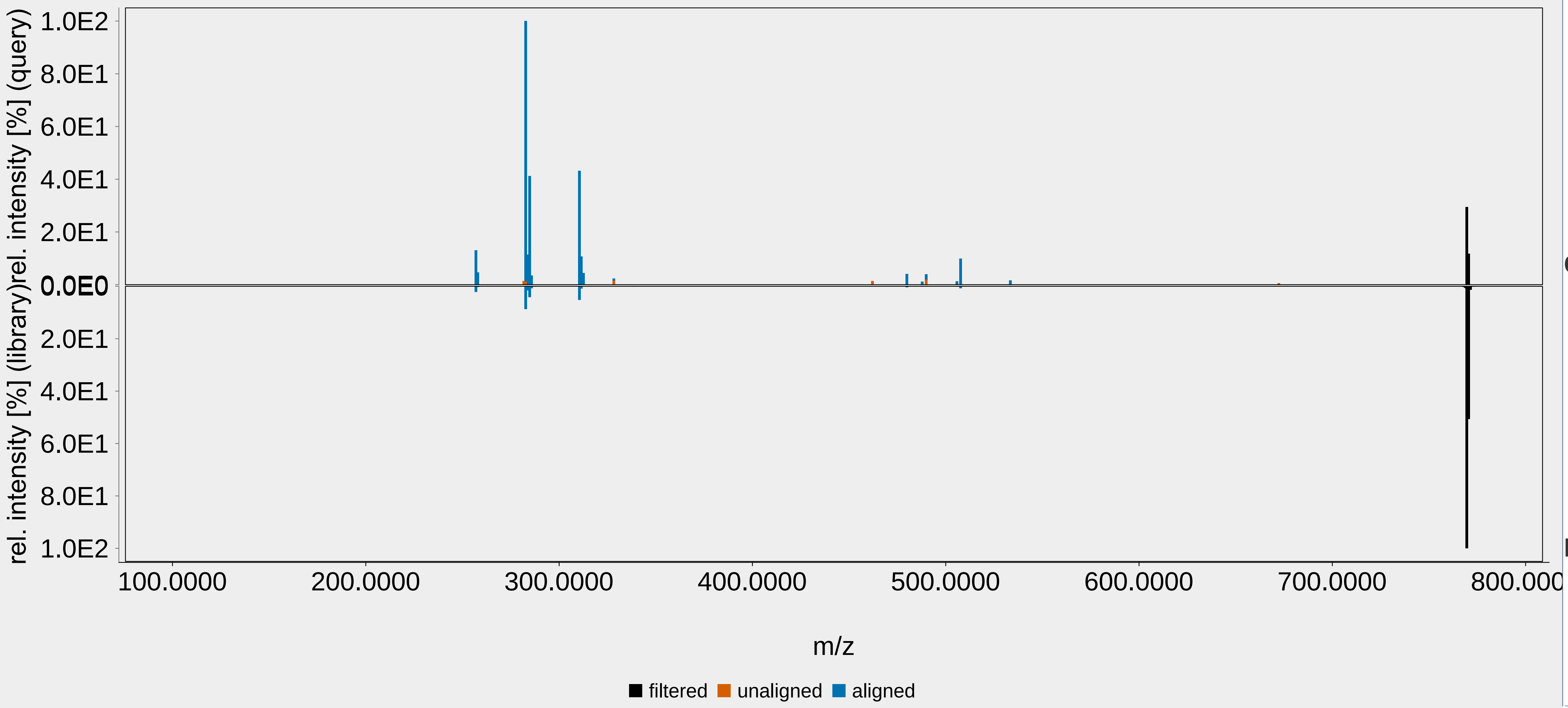

Compound information

Name: PE(18:0/20:1); [M-H]- C43H83N1O8P1

Exact mass: 0.0

Ion type: M-H

Precursor m/z: 772.586

Charge: 1

Polarity: Negative

Instrument information

Instrument type: HCD; Velos

Ion source: LC-ESI

Ms level: 2

Acquisition: Commercial

Database links

Other information

Principal investigator: Thomas Metz

Data collector: Thomas Metz

Entry ID: CCMSLIB00010068147

PI 38:5 0.825

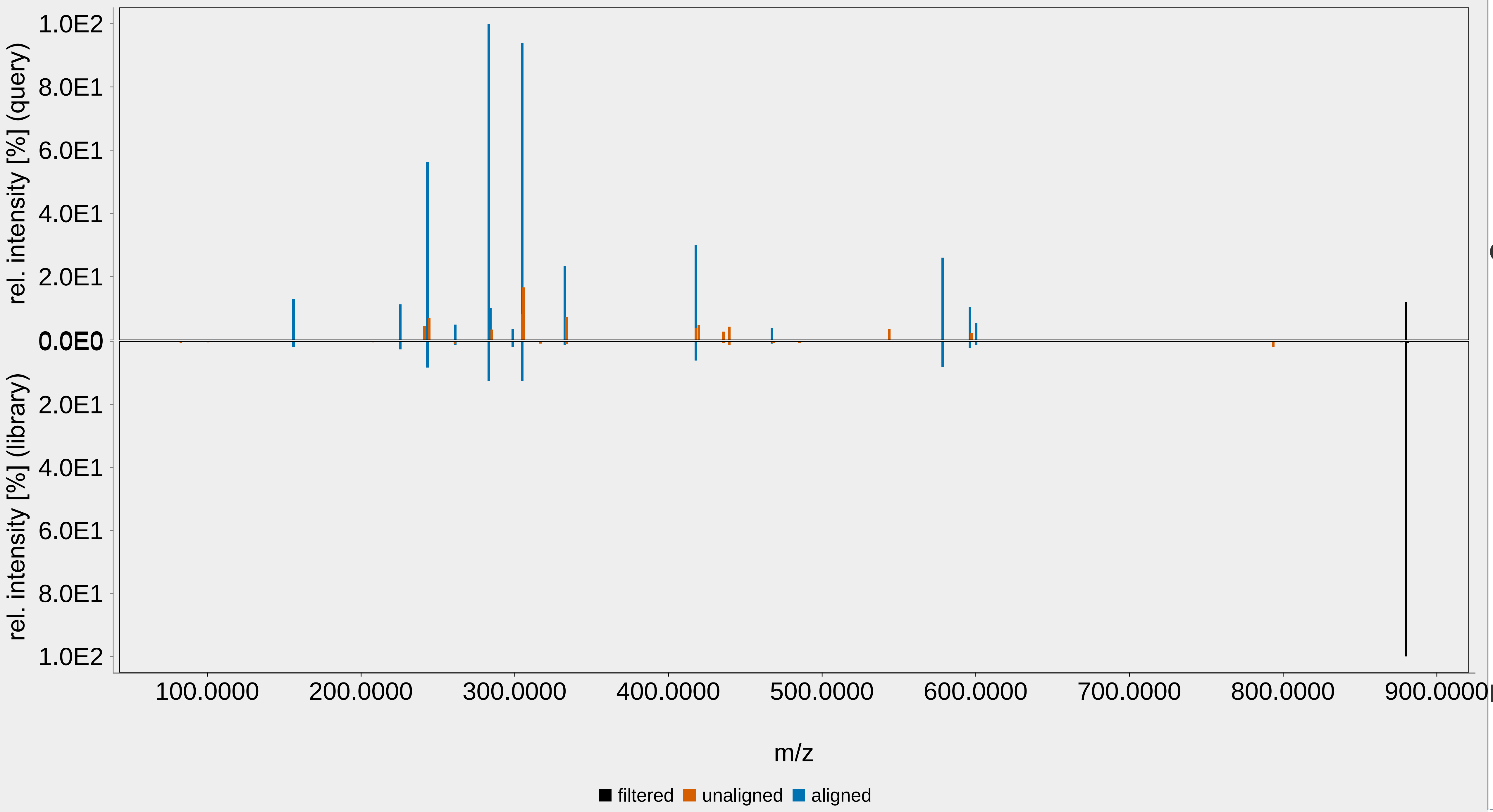

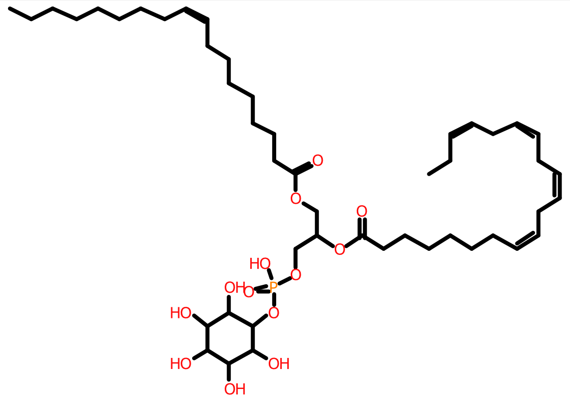

Compound information

Name: PI 38:5

Formula: C47H81O13P

Mol. weight: 885.1279999999999

Exact mass: 885.1279999999999

Ion type: [M-H]-

Precursor m/z: 883.532

RT: 8.353221

Polarity: negative

InChI:

InChI key:

SMILES:

Instrument information

Instrument type: LC-ESI-QTOF

Instrument: AB Sciex TripleTOF 5600+ system

Ms level: MS2

Collision energy: 45V

Database links

Pubchem: 138296016

MoNA ID: LQB00702

Other information

Principal investigator: Kazutaka Ikeda, Hiroshi

Data collector: Kazutaka Ikeda, Hiroshi

PI 38:5

0.825

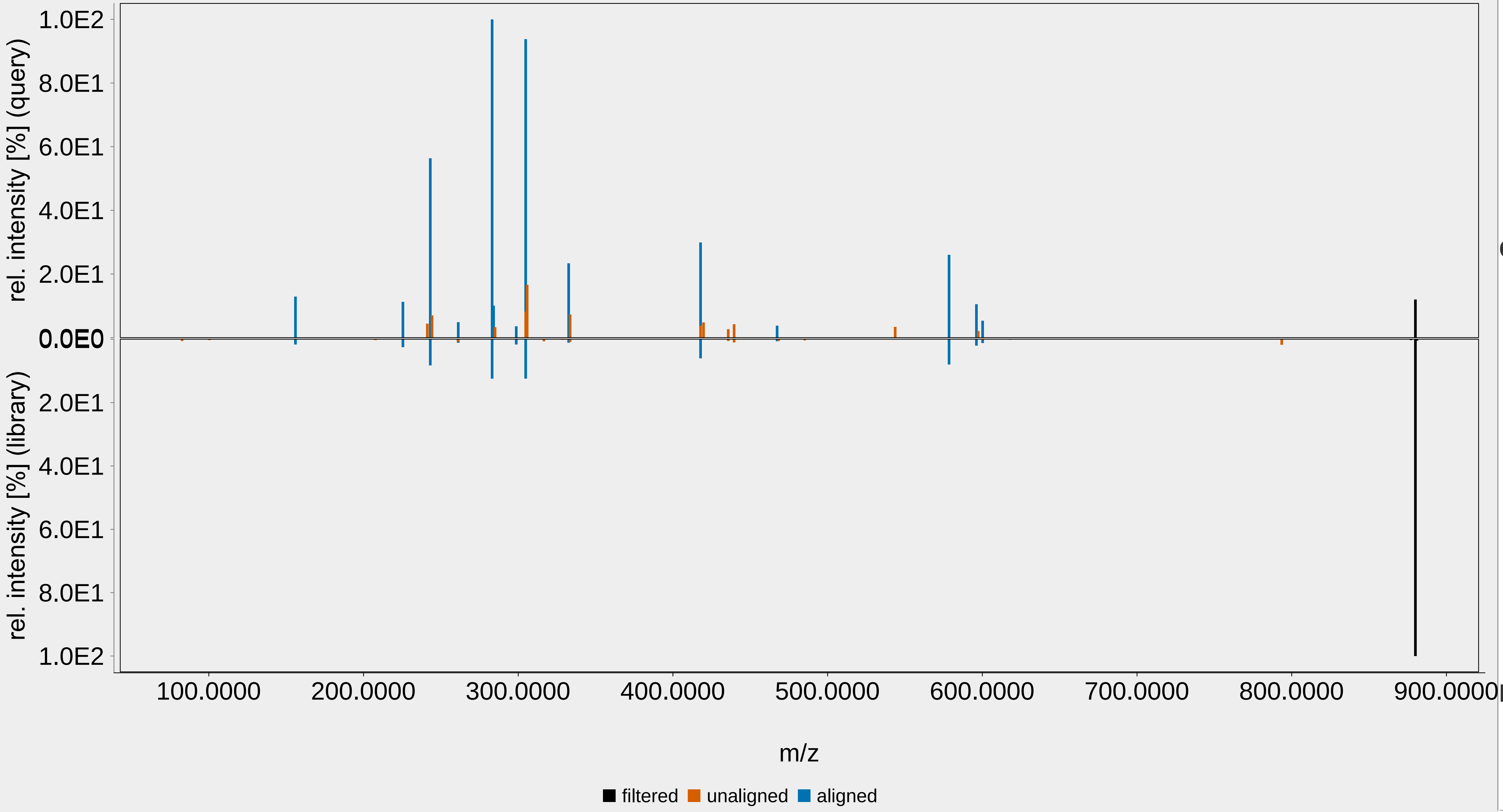

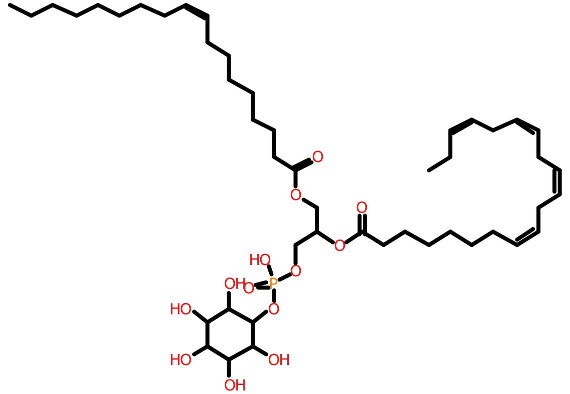

Compound information

Name: PI 38:5

Formula: C47H81O13P

Mol. weight: 885.1279999999999

Exact mass: 885.1279999999999

Ion type: [M-H]-

Precursor m/z: 883.532

RT: 8.353221

Polarity: negative

InChI:

InChI key:

SMILES:

Instrument information

Instrument type: LC-ESI-QTOF

Instrument: AB Sciex TripleTOF 5600+ system

Ms level: MS2

Collision energy: 45V

Database links

Pubchem: 138296016

MoNA ID: LQB00316

Other information

Principal investigator: Kazutaka Ikeda, Hiroshi

Data collector: Kazutaka Ikeda, Hiroshi

PI(18:0_0:0) - [2-hydroxy-3-[hydroxy-[2,3,4,5,6-pentahydroxycyclohexyl]oxyphosphoryl]oxypropyl] octadecanoate **0.778**

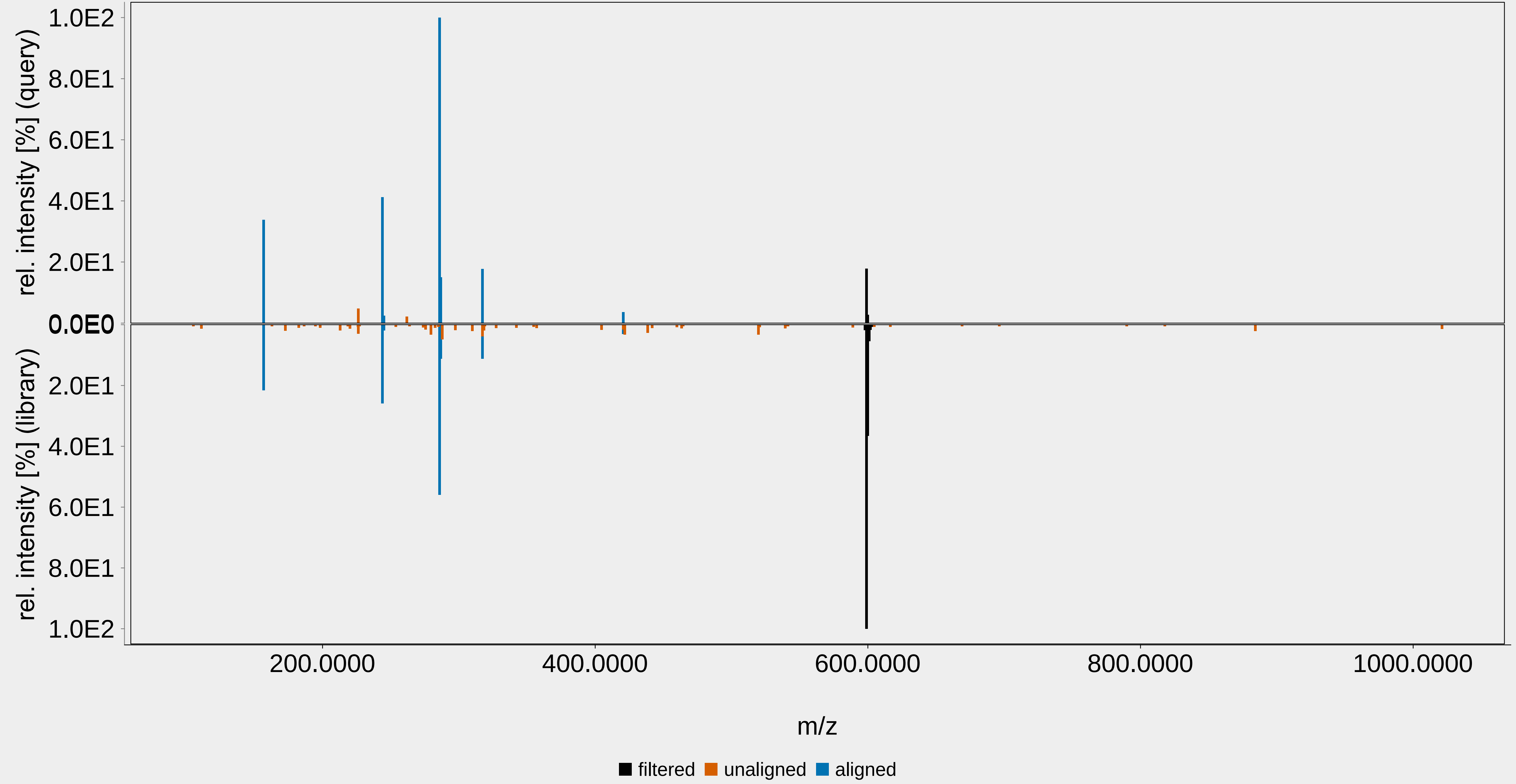

Compound information

Name: PI(18:0_0:0) -

Exact mass: 600.327

Ion type: M-H

Precursor m/z: 599.32

Charge: 1

Polarity: Negative

InChI:

InChI key:

SMILES:

Instrument information

Instrument type: qTof

Ion source: LC-ESI

Ms level: 2

Acquisition: Commercial

Database links

Pubmed:

CAS: 849412-49-3

Other information

Principal investigator: O Laprevote

Data collector: UMR 8038 Universite de Paris

Entry ID: CCMSLIB00005436451

LPI 18:0

0.948

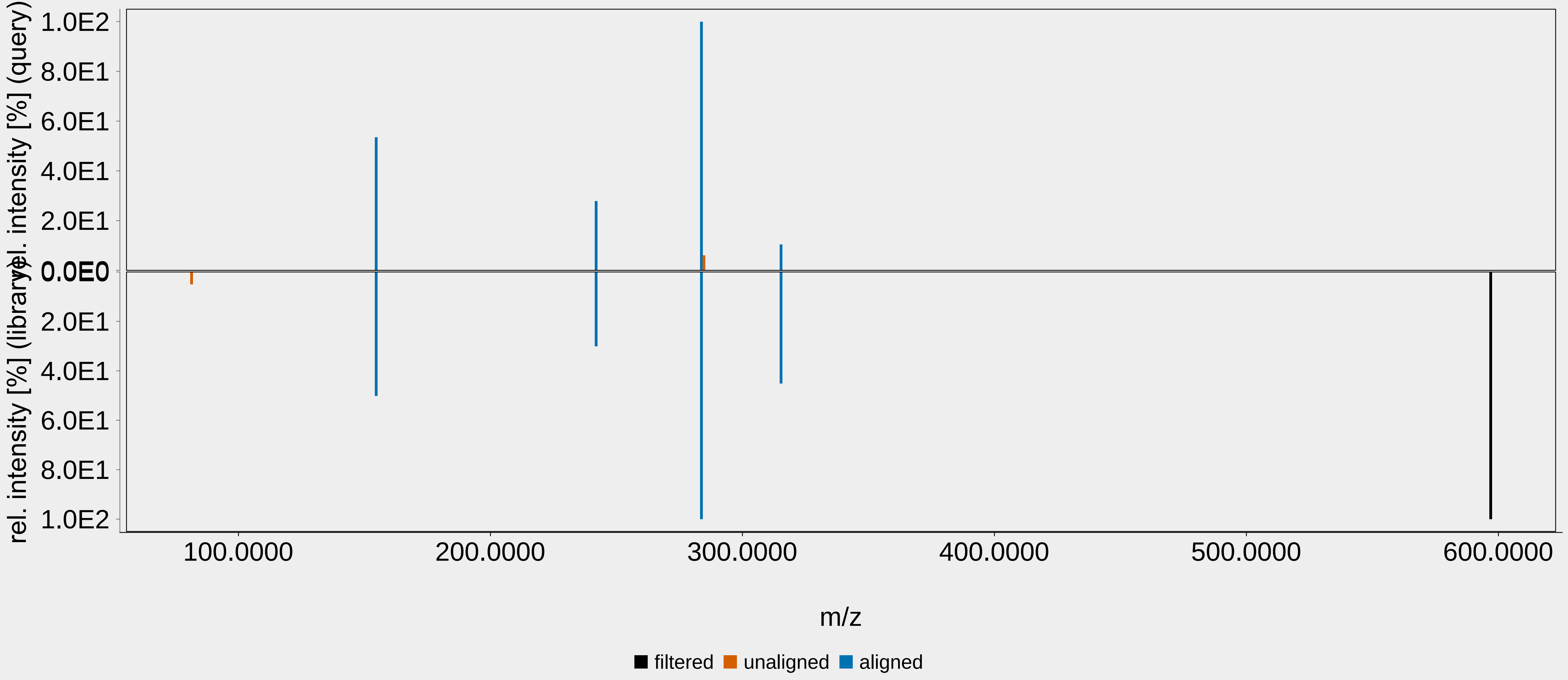

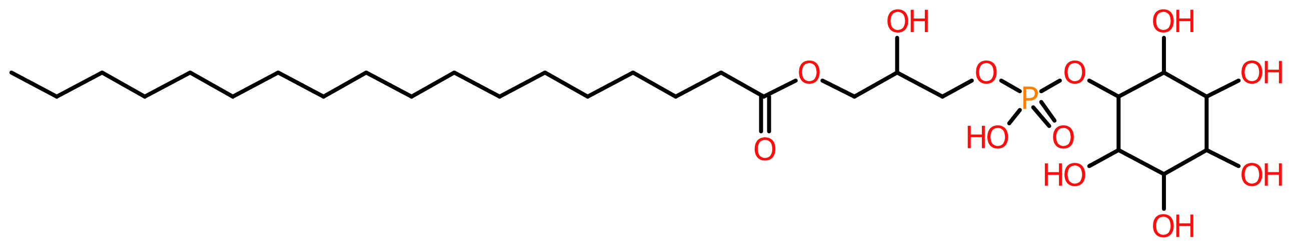

Compound information

Name: LPI 18:0

Formula: C27H53O12P

Ion type: [M-H]-

Precursor m/z: 599.32019

Ccs: 233.47

InChI key: MXAFDFDAIFZFET-UHFFFAOYNA-

SMILES:

Num peaks: 6

Instrument information

Database links

Other information

PE(18:0/18:1); [M-H]- C41H79N1O8P1

0.920

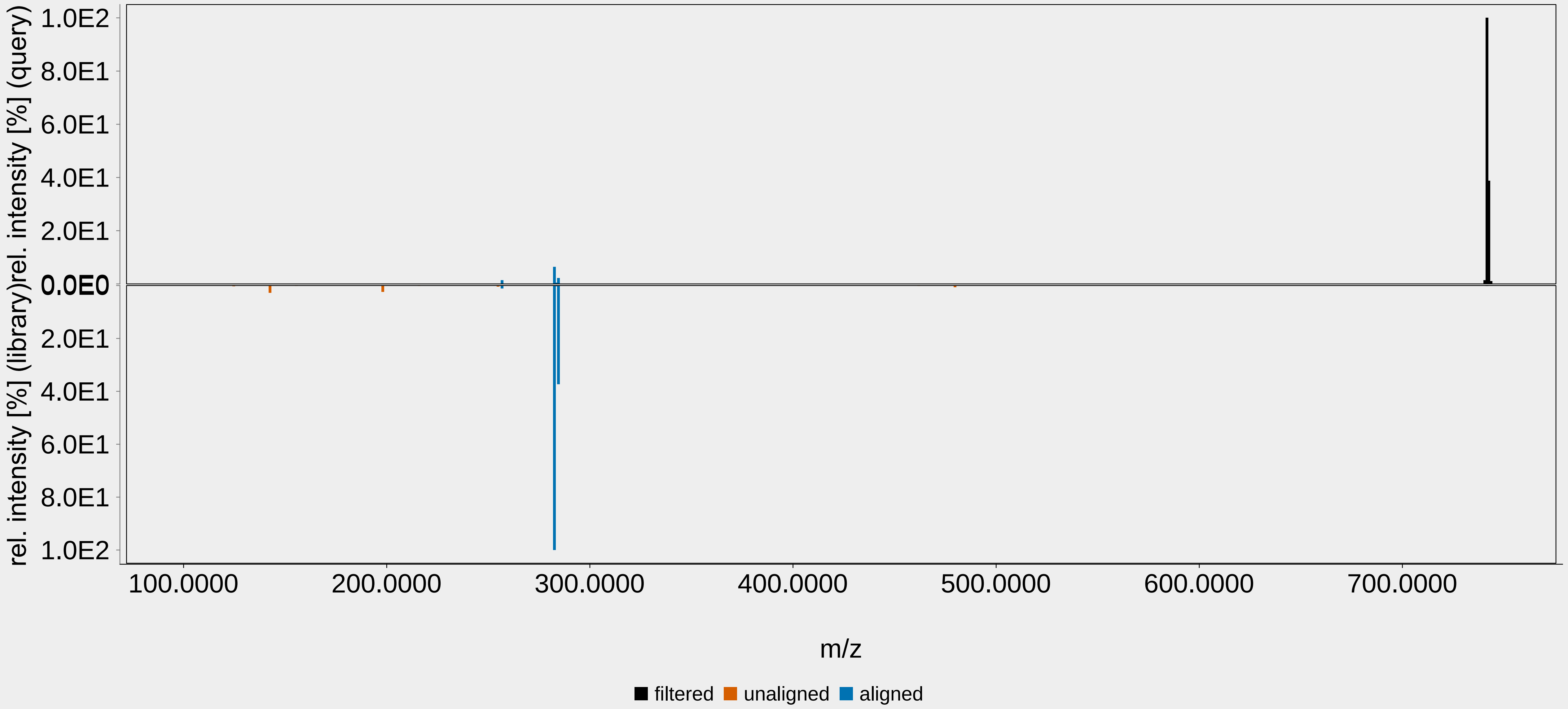

Compound information

Name: PE(18:0/18:1); [M-H]- C41H79N1O8P1

Exact mass: 0.0

Ion type: M-H

Precursor m/z: 744.554

Charge: 1

Polarity: Negative

Instrument information

Instrument type: HCD; Lumos

Ion source: LC-ESI

Ms level: 2

Acquisition: Commercial

Database links

Other information

Principal investigator: Thomas Metz

Data collector: Thomas Metz

Entry ID: CCMSLIB00010059575

PE(18:0/18:1); [M-H]- C41H79N1O8P1 0.930

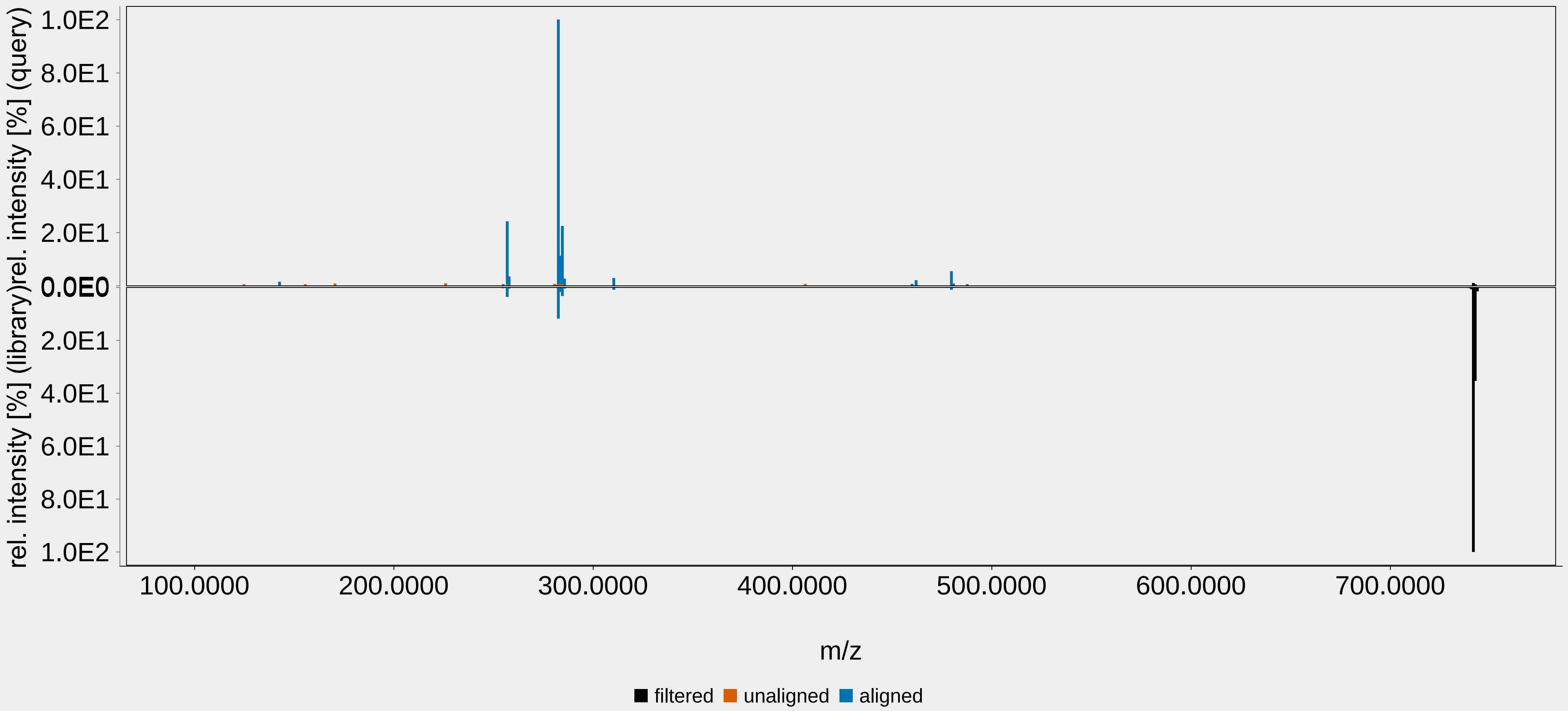

Compound information

Name: PE(18:0/18:1); [M-H]- C41H79N1O8P1

Exact mass: 0.0

Ion type: M-H

Precursor m/z: 744.554

Charge: 1

Polarity: Negative

Instrument information

Instrument type: HCD; Velos

Ion source: LC-ESI

Ms level: 2

Acquisition: Commercial

Database links

Other information

Principal investigator: Thomas Metz

Data collector: Thomas Metz

Entry ID: CCMSLIB00010072259

PE(18:1/22:6); [M-H]- C45H75N1O8P1 **0.910**

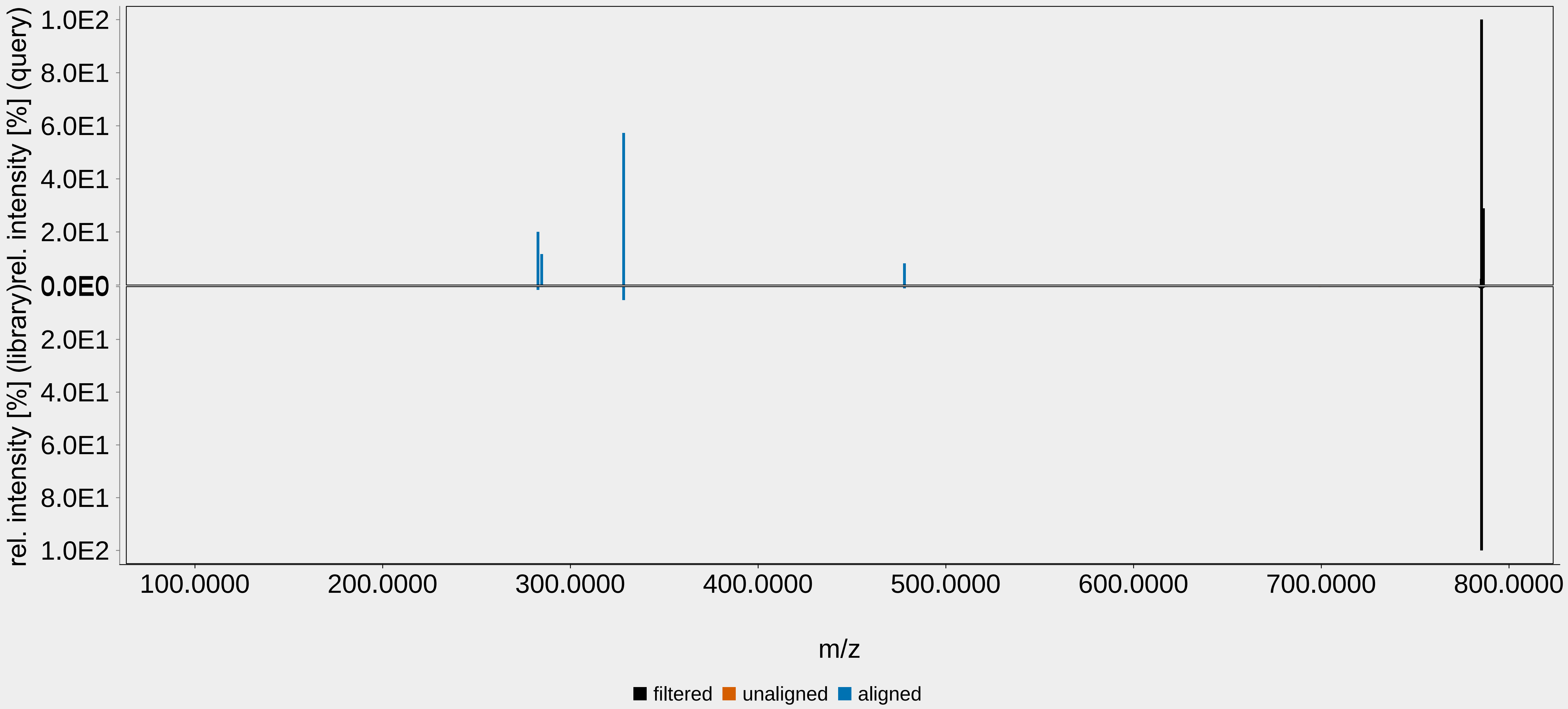

Compound information

Name: PE(18:1/22:6); [M-H]- C45H75N1O8P1

Exact mass: 0.0

Ion type: M-H

Precursor m/z: 788.523

Charge: 1

Polarity: Negative

Instrument information

Instrument type: HCD; Velos

Ion source: LC-ESI

Ms level: 2

Acquisition: Commercial

Database links

Other information

Principal investigator: Thomas Metz

Data collector: Thomas Metz

Entry ID: CCMSLIB00010063779

PE(18:1/22:6); [M-H]- C45H75N1O8P1 **0.923**

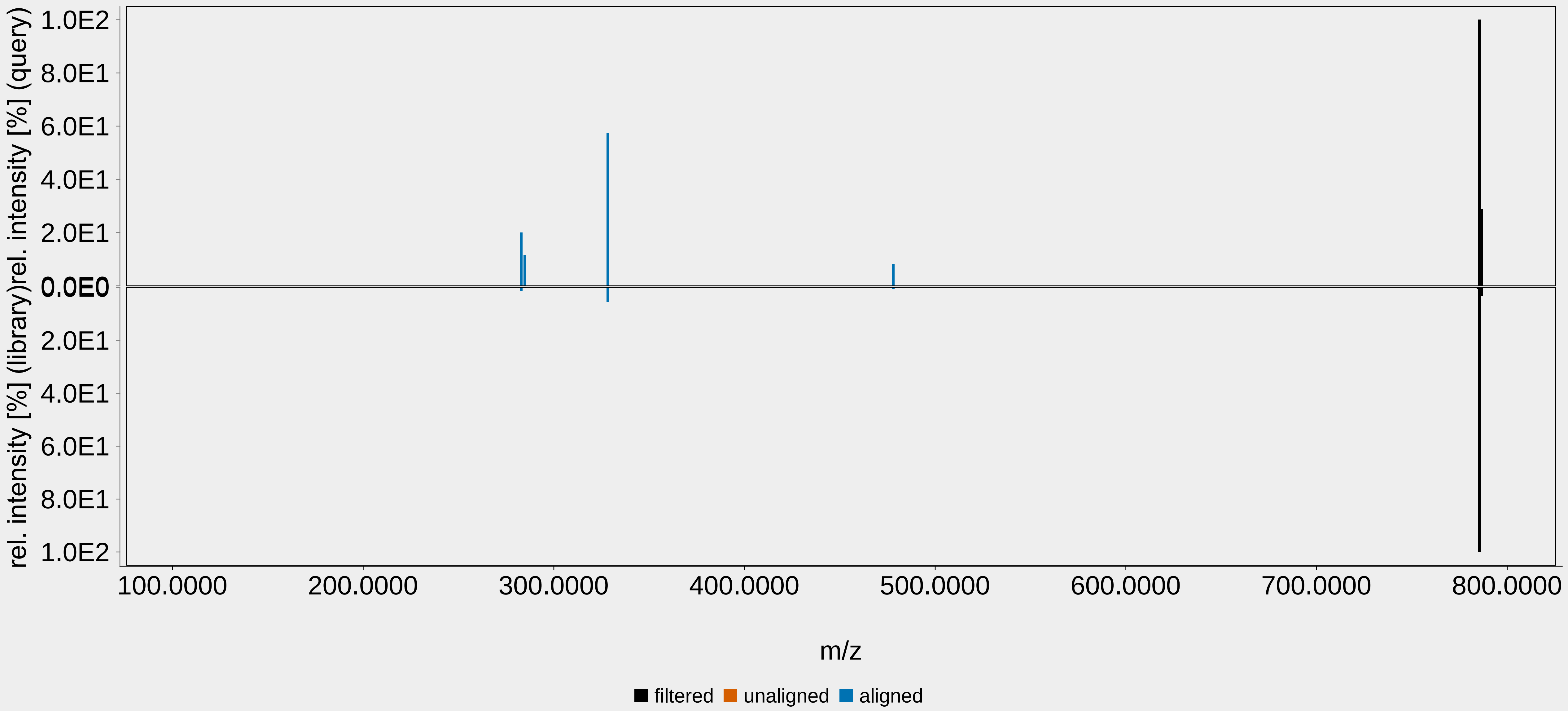

Compound information

Name: PE(18:1/22:6); [M-H]- C45H75N1O8P1

Exact mass: 0.0

Ion type: M-H

Precursor m/z: 788.523

Charge: 1

Polarity: Negative

Instrument information

Instrument type: HCD; Velos

Ion source: LC-ESI

Ms level: 2

Acquisition: Commercial

Database links

Other information

Principal investigator: Thomas Metz

Data collector: Thomas Metz

Entry ID: CCMSLIB00010073275

Adenosine monophosphate - 30.0 eV

0.806

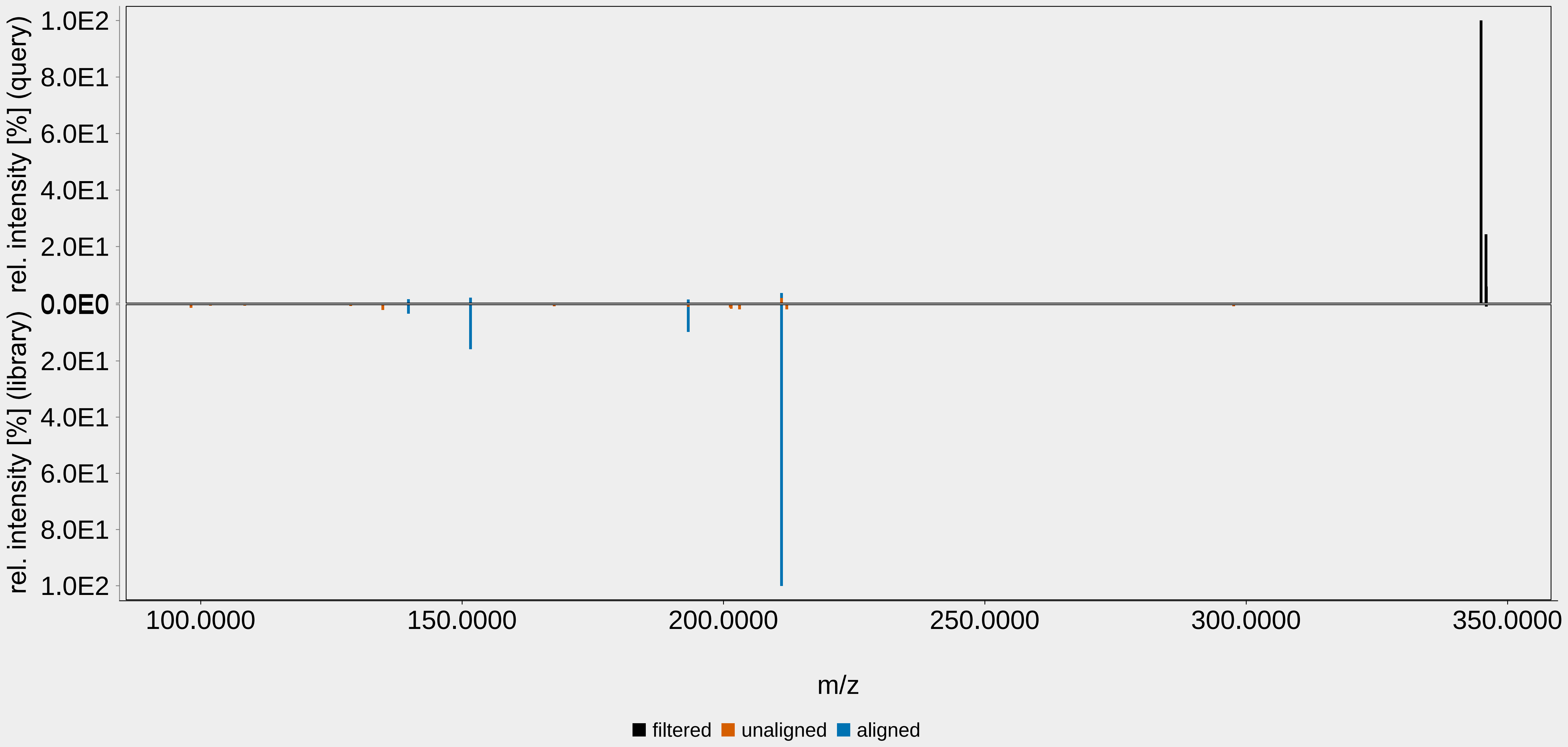

Compound information

Name: Adenosine monophosphate - 30.0 eV

Exact mass: 0.0

Ion type: M-H

Precursor m/z: 346.056

Charge: 1

Polarity: Negative

SMILES:

Instrument information

Instrument type: Orbitrap

Ion source: ESI

Ms level: 2

Acquisition: Isolated

Database links

CAS:

Other information

Principal investigator: PI

Data collector: Data Collector

Entry ID: CCMSLIB00006114160

Adenosine monophosphate - 30.0 eV

0.800

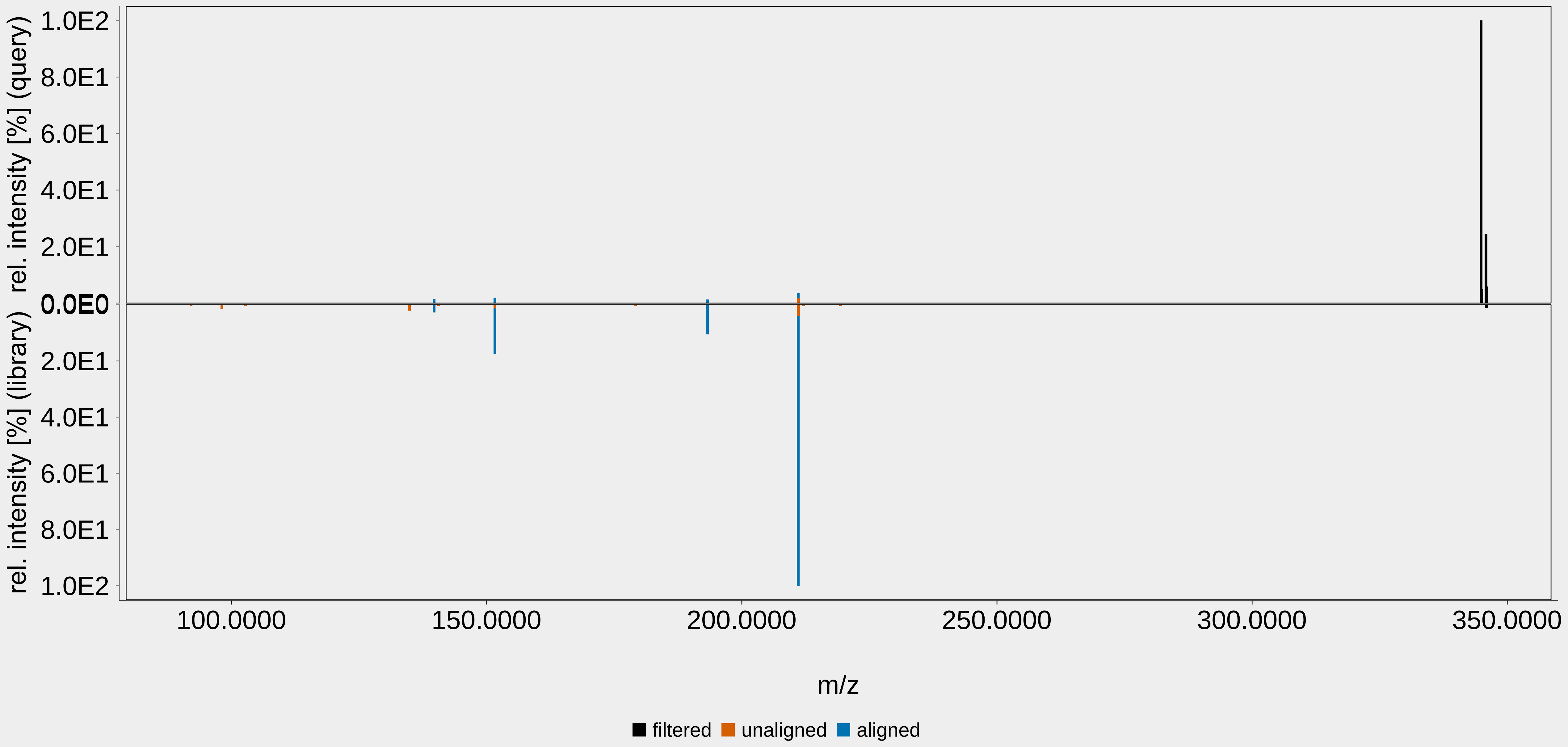

Compound information

Name: Adenosine monophosphate - 30.0 eV

Exact mass: 0.0

Ion type: M-H

Precursor m/z: 346.056

Charge: 1

Polarity: Negative

SMILES:

Instrument information

Instrument type: Orbitrap

Ion source: ESI

Ms level: 2

Acquisition: Isolated

Database links

CAS:

Other information

Principal investigator: PI

Data collector: Data Collector

Entry ID: CCMSLIB00006114141

[5-acetyloxy-3-(hydroxymethyl)-2-oxo-6-propan-2-ylcyclohex-3-en-1-yl] 3-methylbutanoate

0.793

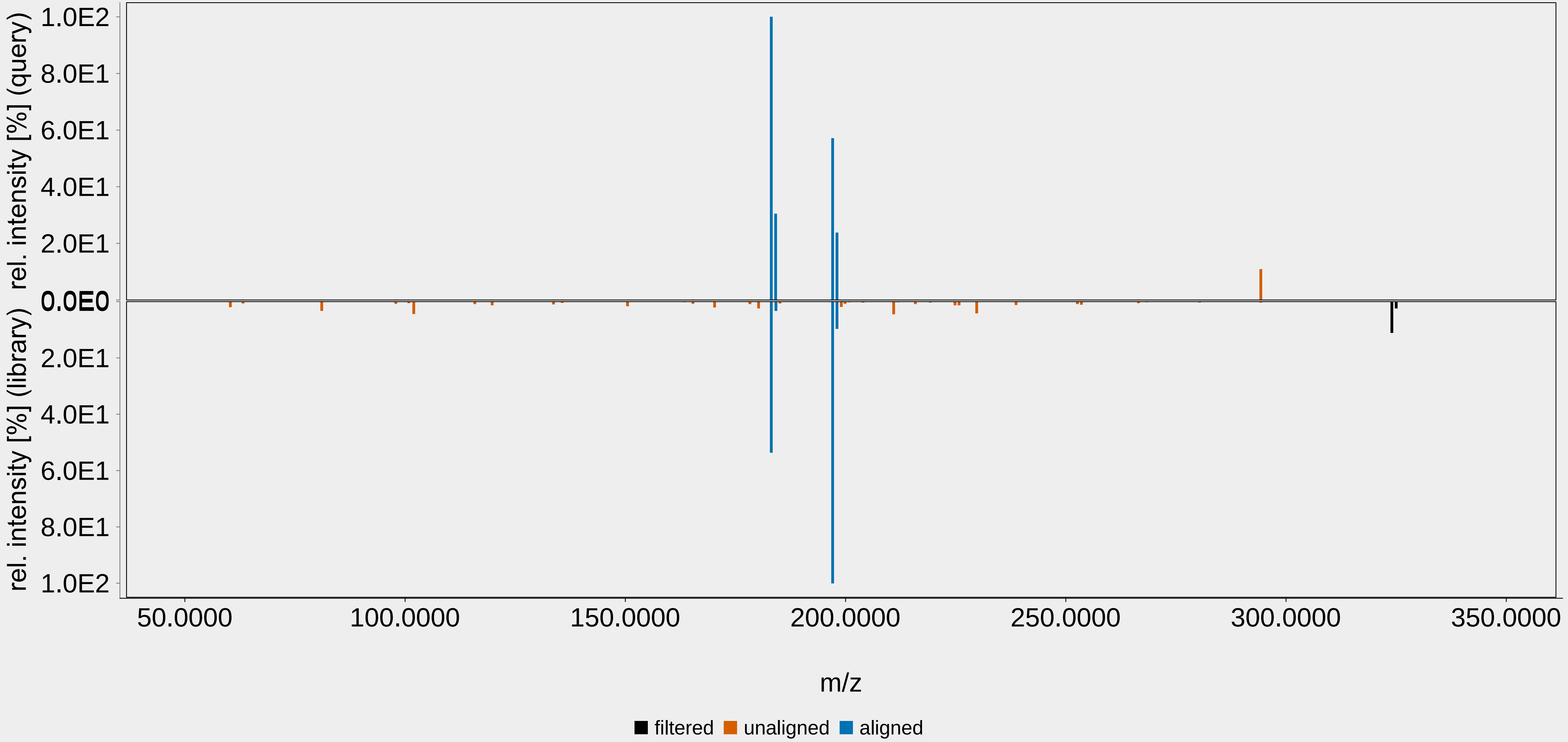

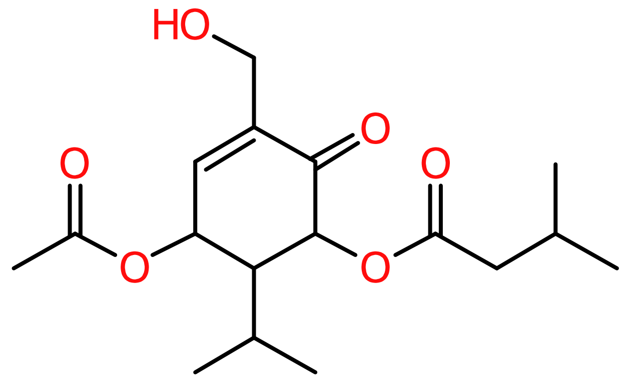

Compound information

Name:

Formula: $C_{17}H_{26}O_6$

Ion type: [M-H]-

Precursor m/z: 325.1656494140625

Polarity: negative

InChI:

InChI key: IVEJMWCGEFICJC-UHFFFAOYSA-

SMILES:

Instrument information

Instrument type: ESI-QFT

Instrument: Thermo Q Exactive HF

Ms level: MS2

Collision energy: 65HCD

Database links

Other information

Principal investigator: Arpana Vaniya

Data collector: Arpana Vaniya

[5-acetyloxy-3-(hydroxymethyl)-2-oxo-6-propan-2-ylcyclohex-3-en-1-yl] 3-methylbutanoate

0.793

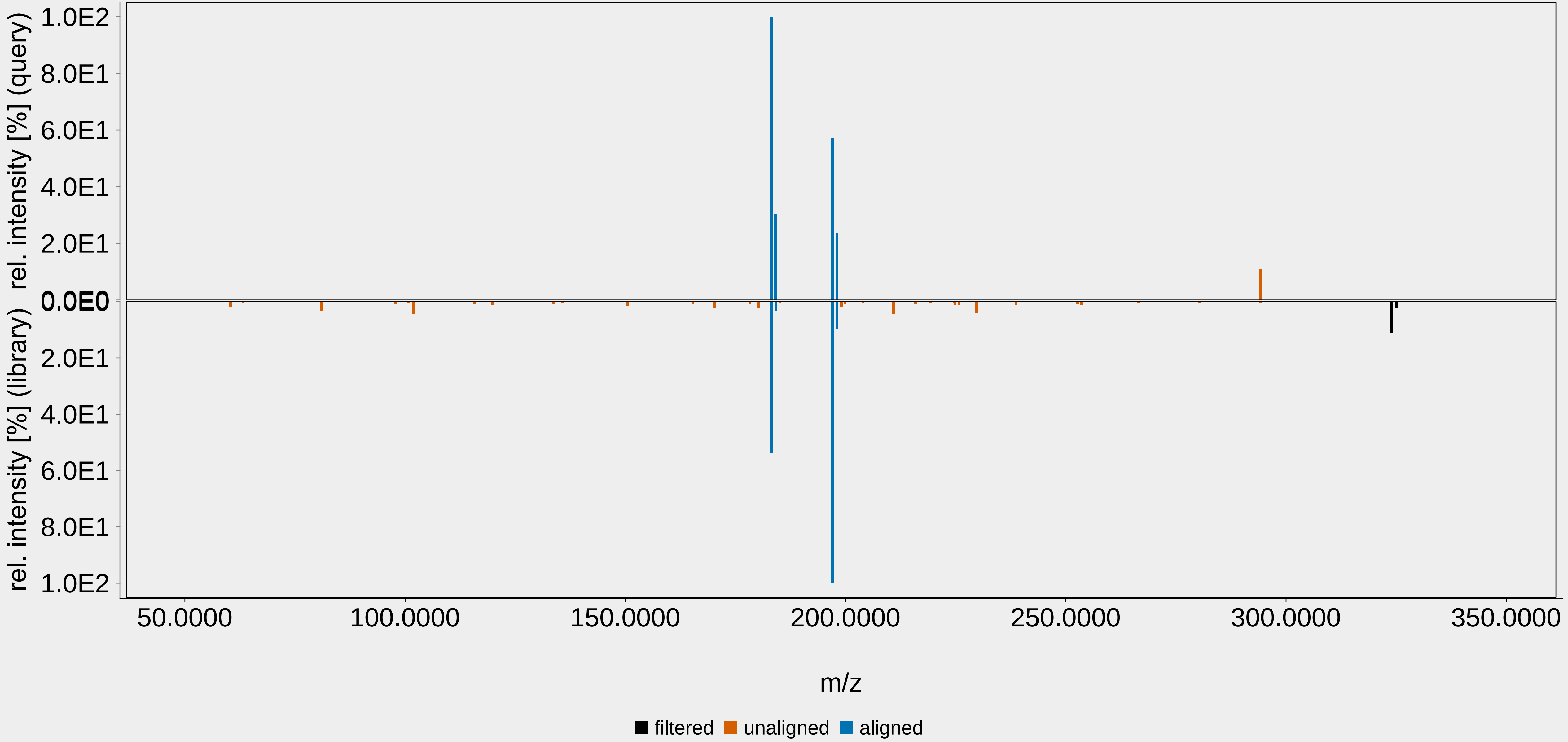

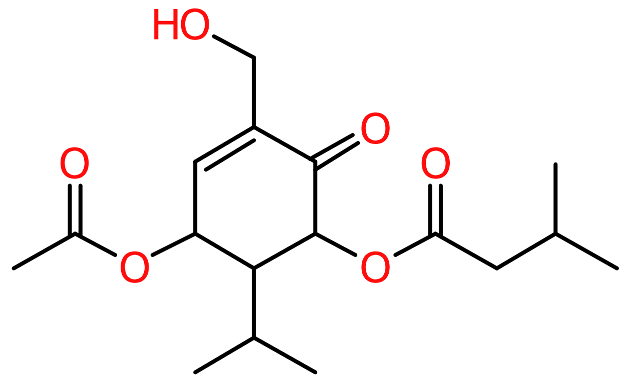

Compound information

Name:

Exact mass: 0.0

Ion type: [M-H]-

Precursor m/z: 325.166

Charge: 1

Polarity: negative

InChI:

Instrument information

Instrument type: ESI-QFT

Ms level: 2

Acquisition: isolated

Database links

Other information

Principal investigator: MoNA

Data collector: MoNA:VF-NPL-QEHF008244

Entry ID: CCMSLIB00004699307

PE(18:1/0:0); [M-H]- C23H45N1O7P1 0.949

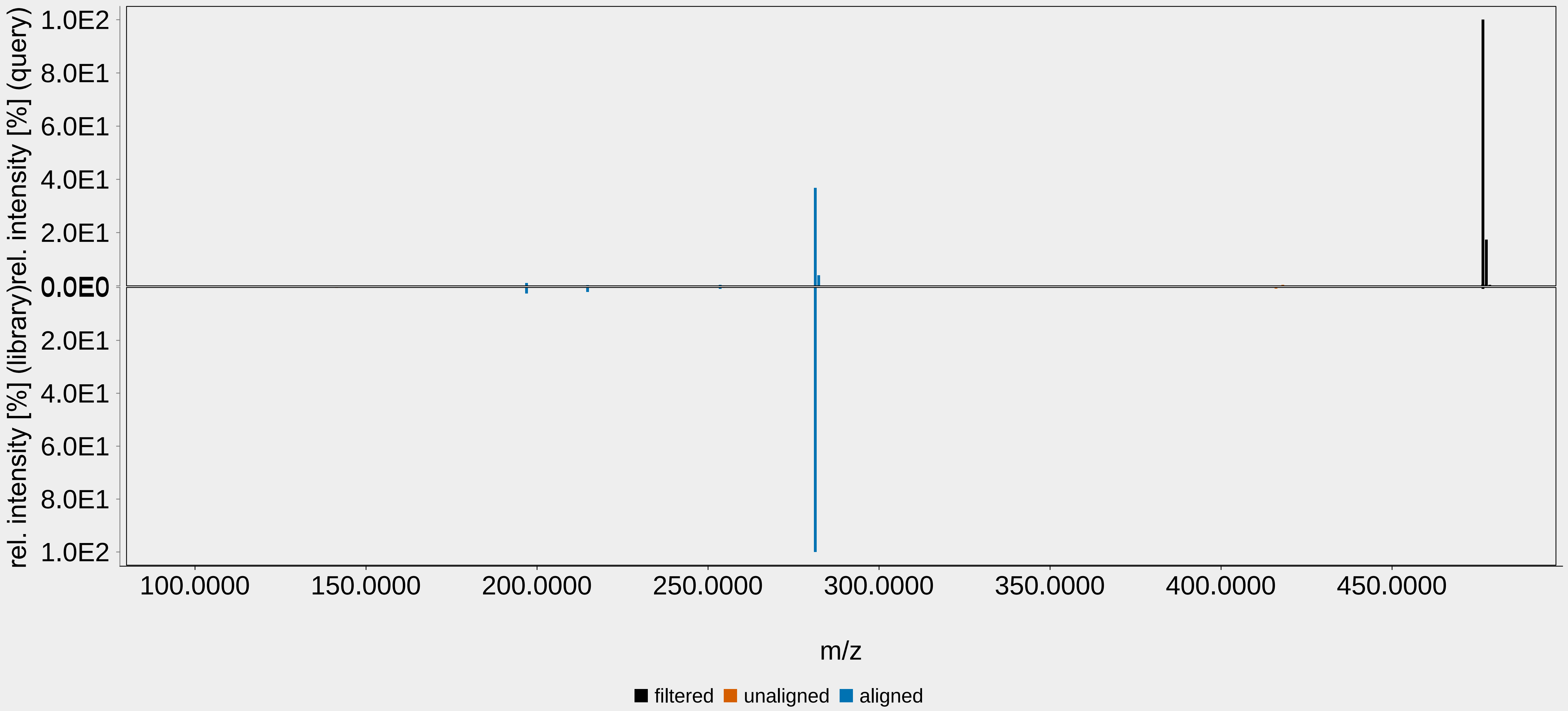

Compound information

Name: PE(18:1/0:0); [M-H]- C23H45N1O7P1

Exact mass: 0.0

Ion type: M-H

Precursor m/z: 478.293

Charge: 1

Polarity: Negative

Instrument information

Instrument type: CID; Lumos

Ion source: LC-ESI

Ms level: 2

Acquisition: Commercial

Database links

Other information

Principal investigator: Thomas Metz

Data collector: Thomas Metz

Entry ID: CCMSLIB00010059278

PE(18:1/0:0); [M-H]- C23H45N1O7P1

0.950

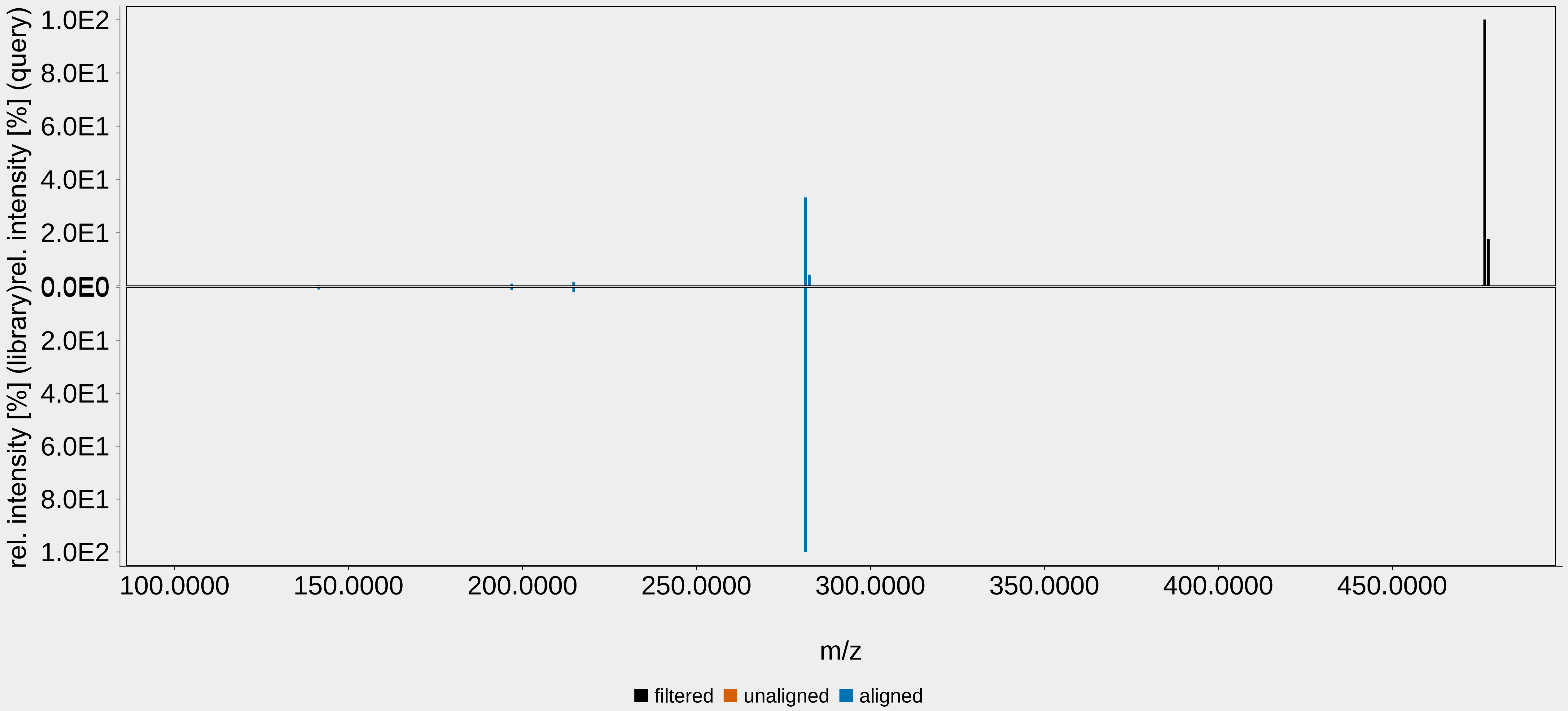

Compound information

Name: PE(18:1/0:0); [M-H]- C23H45N1O7P1

Exact mass: 0.0

Ion type: M-H

Precursor m/z: 478.293

Charge: 1

Polarity: Negative

Instrument information

Instrument type: HCD; Lumos

Ion source: LC-ESI

Ms level: 2

Acquisition: Commercial

Database links

Other information

Principal investigator: Thomas Metz

Data collector: Thomas Metz

Entry ID: CCMSLIB00010059197

C10-LAS (TENTATIVE)

0.899

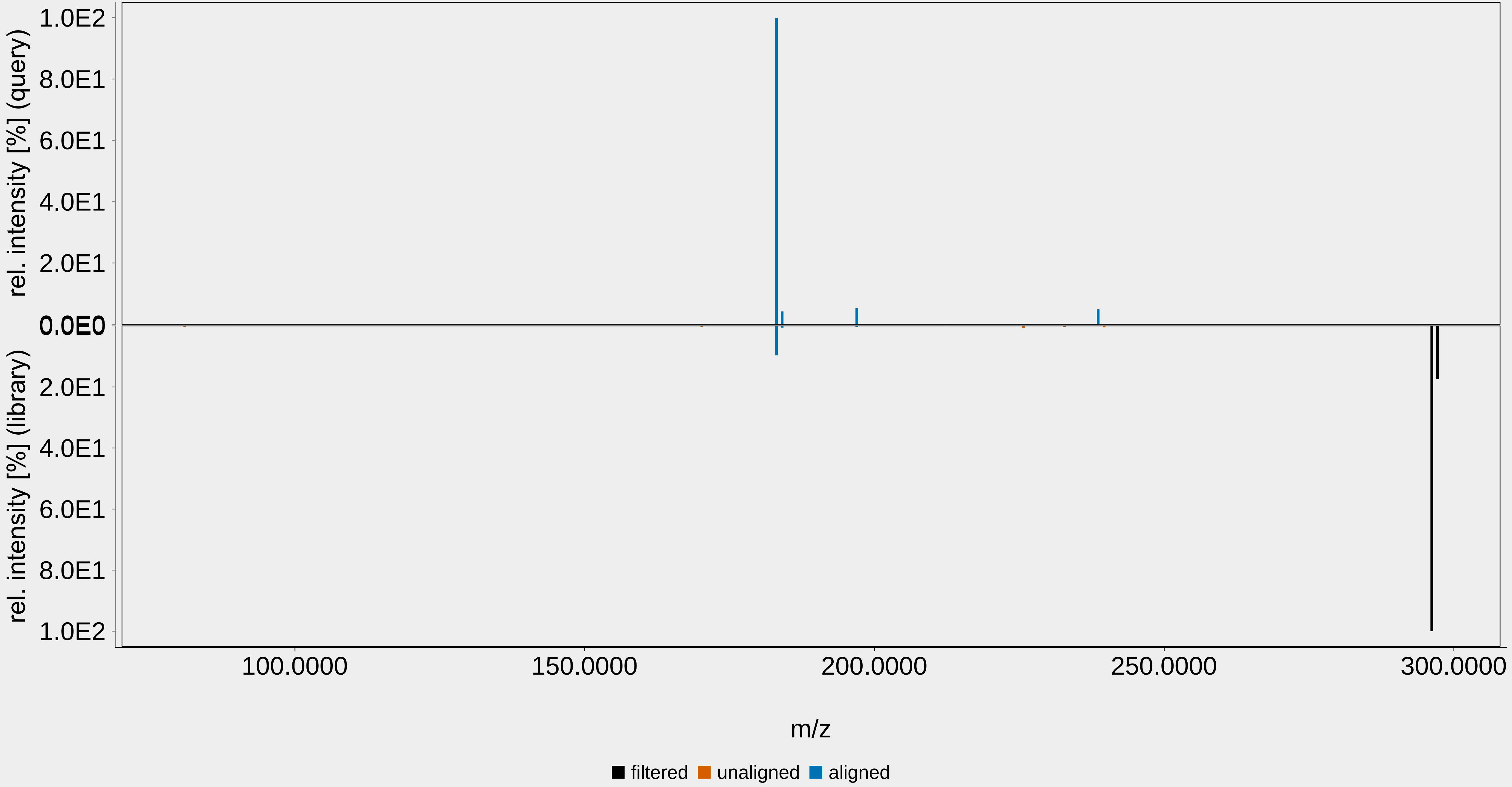

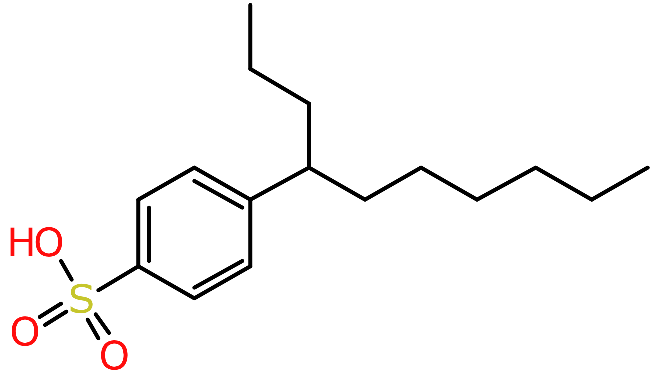

Compound information

Name: C10-LAS (TENTATIVE)

Formula: C16H26O3S

Exact mass: 298.1603

Ion type: [M-H]-

Precursor m/z: 297.1521

Polarity: NEGATIVE

InChI:

InChI key: NANHIUZYPFDGJS-UHFFFAOYSA-

SMILES:

Num peaks: 20

Instrument information

Instrument type: LC-ESI-ITFT

Instrument: LTQ Orbitrap XL Thermo Scientific

Ms level: MS2

Collision energy: 60 NCE

Database links

Other information

Entry ID:

Comment: Parent=297.1521

C10-LAS (TENTATIVE), C10-linear alkylbenzyl sulfonate, 4-(decan-4-yl)benzenesulfonic acid

0.900

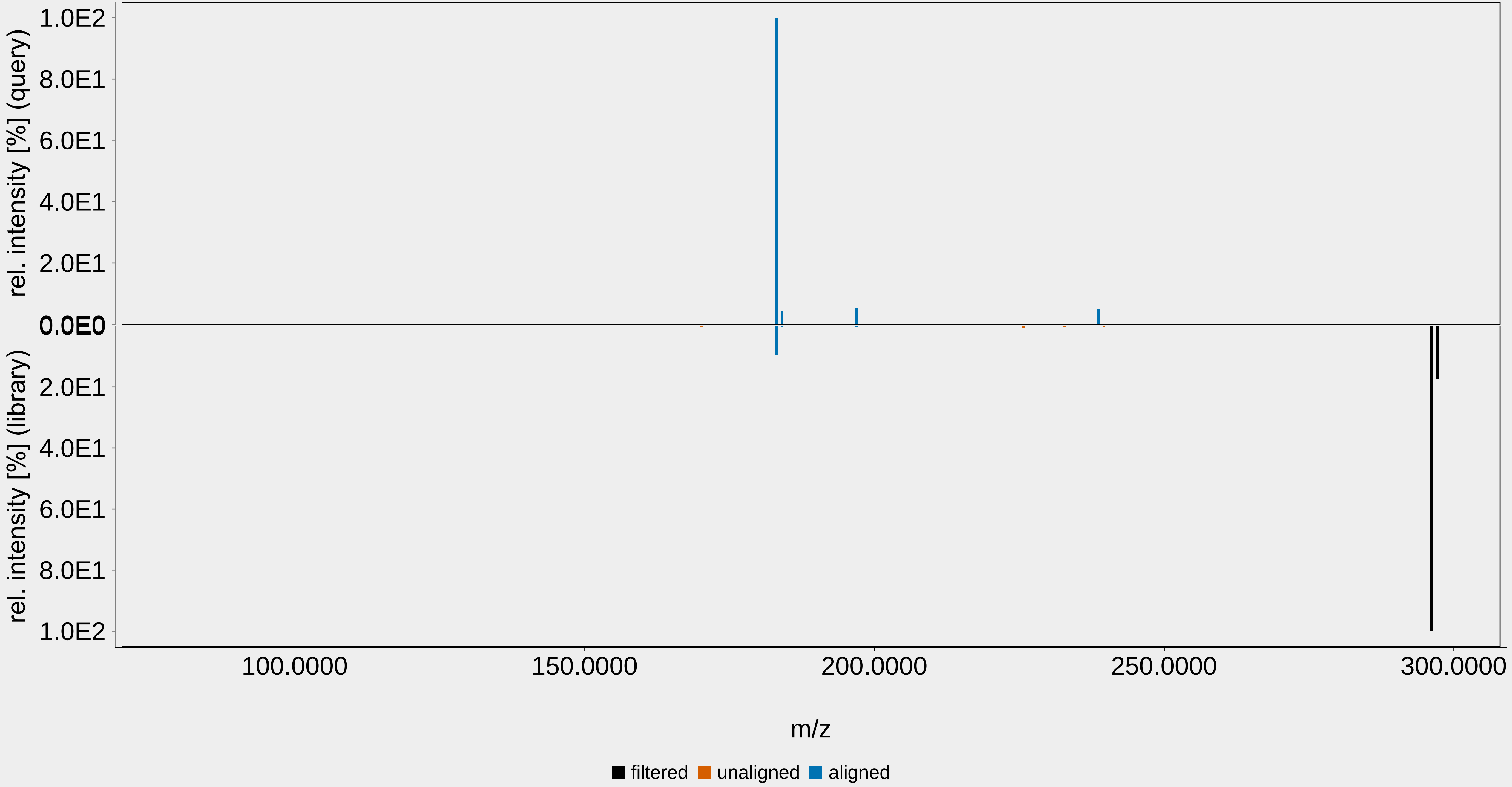

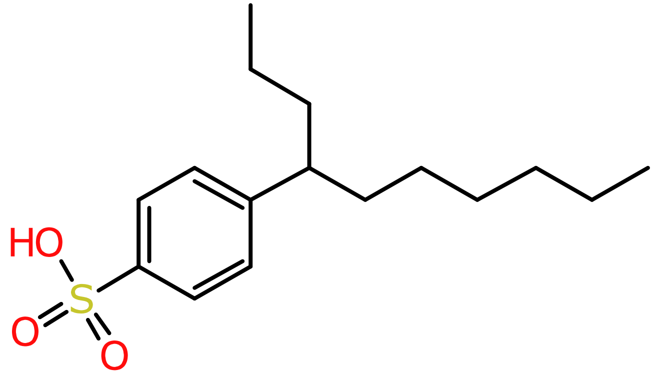

Compound information

Name: C10-LAS (TENTATIVE), C10-linear

Formula: C16H26O3S

Mol. weight: 298.1603

Exact mass: 298.1603

Ion type: [M-H]-

Precursor m/z: 297.1521

Polarity: negative

InChI:

InChI key: NANHIUZYPFDGJS-UHFFFAOYSA-

SMILES:

Instrument information

Instrument type: LC-ESI-ITFT

Instrument: LTQ Orbitrap XL Thermo Scientific

Ion source: ESI

Ms level: MS2

Collision energy: 60 NCE

Database links

Pubchem: 14921167

MoNA ID: ETS00001

Other information

Principal investigator: E. Schymanski, Dept. of

Data collector: E. Schymanski, Dept. of

Crocetin semialdehyde

0.739

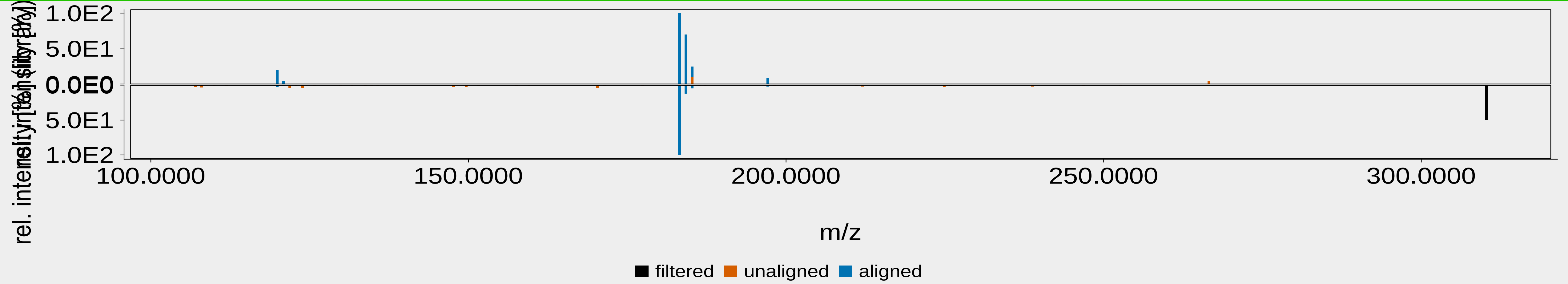

Compound information

Name: Crocetin semialdehyde

Ion type: carotenoid

Precursor m/z: 312.1725

Polarity: negative

Instrument information

Ion source: Electrospray Ionization (ESI)

Ms level: MS2

Database links

Other information

Canrenone

0.912

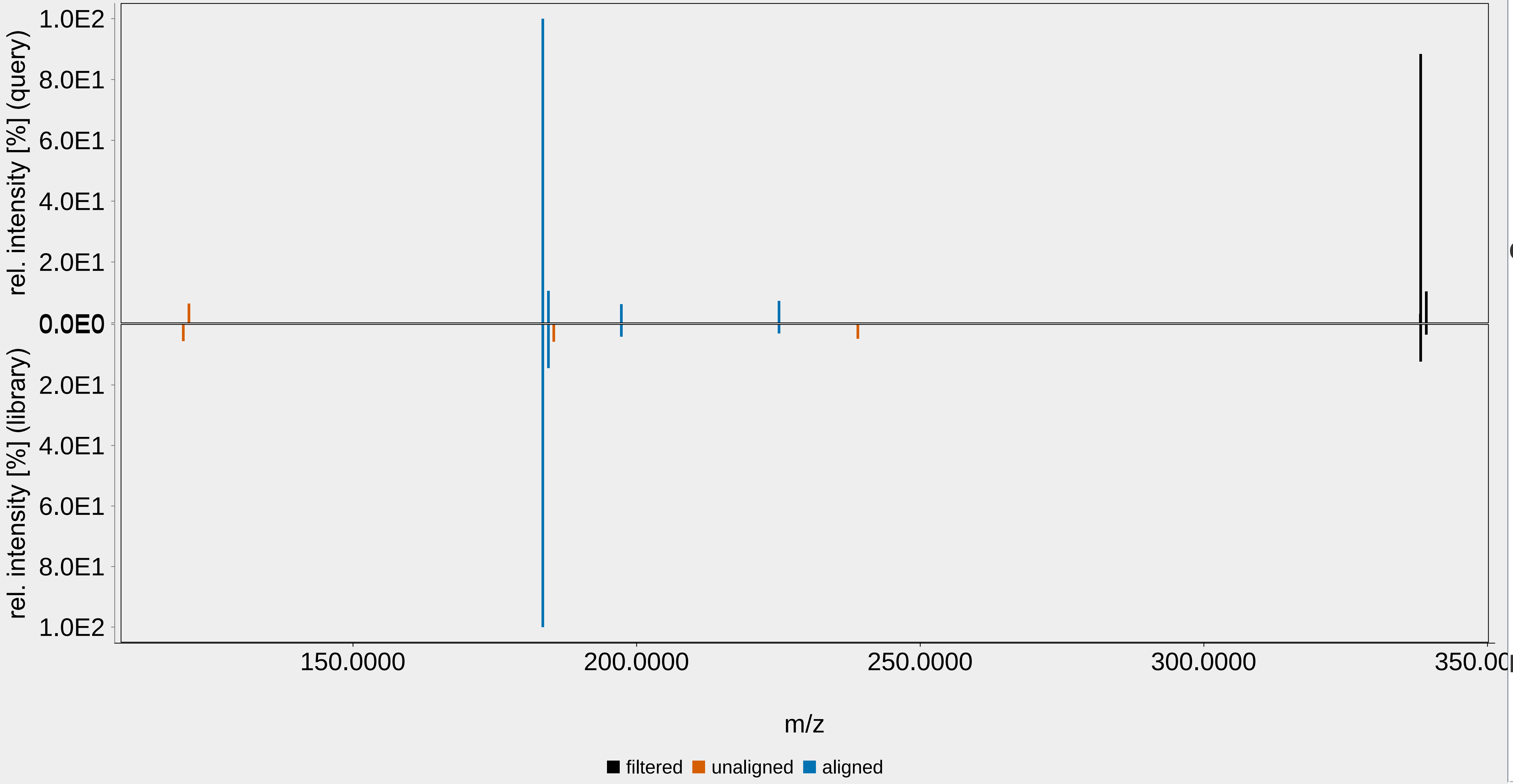

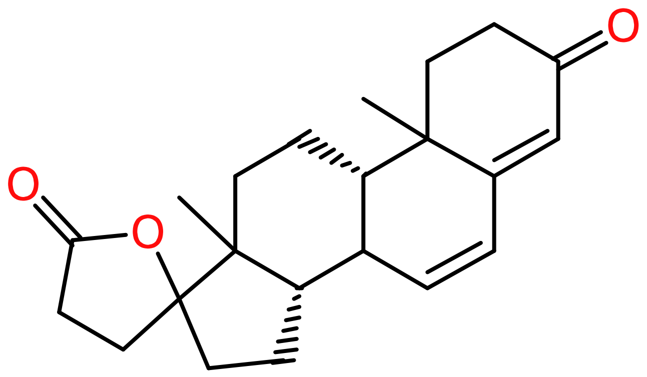

Compound information

Name: Canrenone

Formula: C22H28O3

Exact mass: 340.2038448

Ion type: [M-H]-

Precursor m/z: 339.1966

Polarity: NEGATIVE

InChI:

InChI key:

SMILES:

Num peaks: 9

Instrument information

Instrument type: LC-ESI-QTOF

Instrument: Bruker maXis Impact

Ms level: MS2

Collision energy: 40 eV

Database links

Other information

Entry ID: MSBNK-Athens_Univ-AU239460

Comment: Parent=339.1966

Canrenone, (9S,14S)-10,13-dimethylspiro[2,8,9,11,12,14,15,16-octahydro-1H-cyclopenta[a]phenanthrene-17,5`-oxolane]-2`,3-dione

0.912

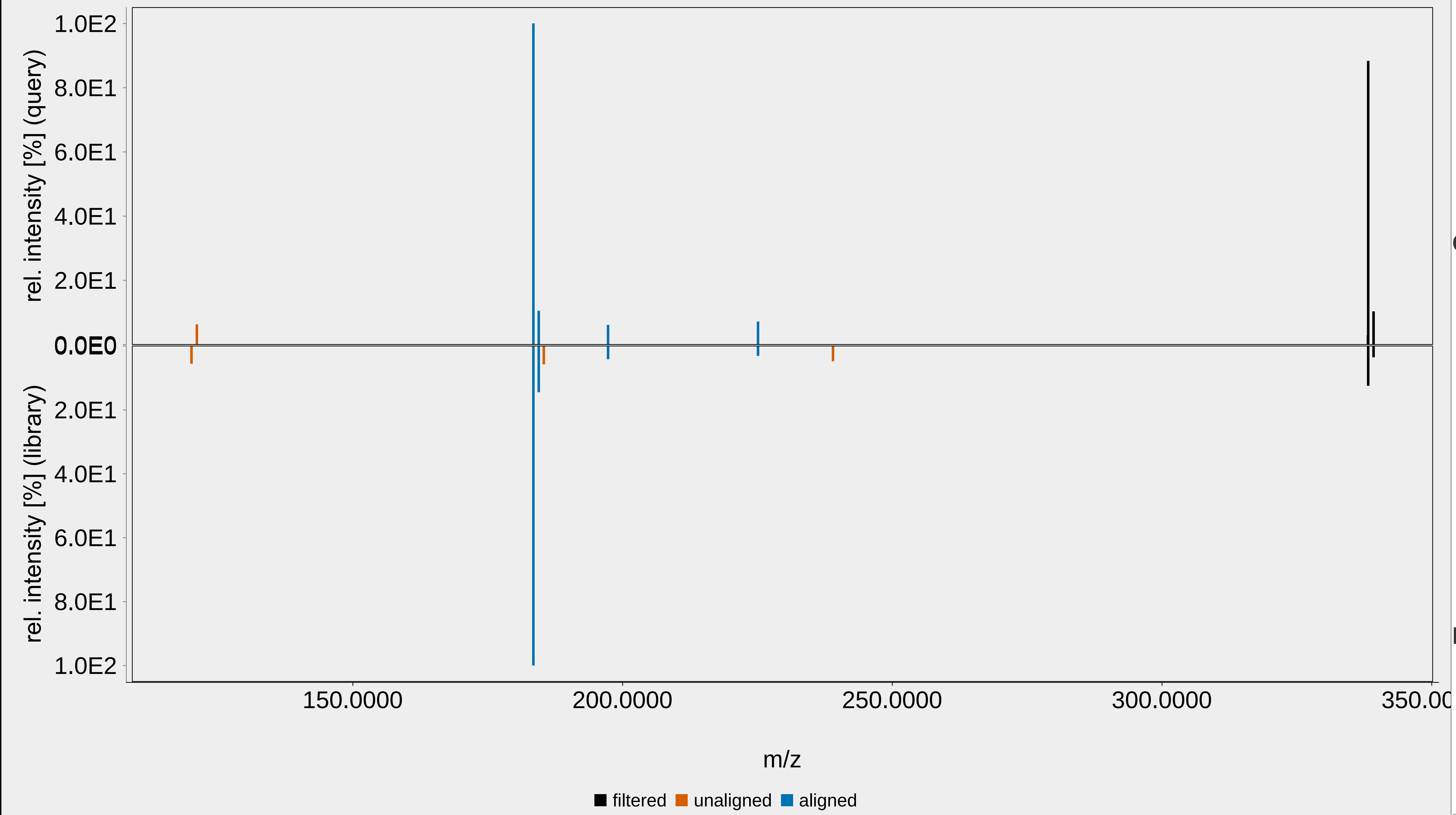

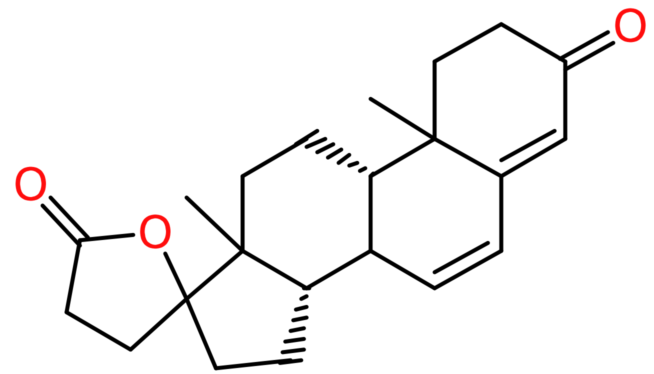

Compound information

Name: Canrenone,

Formula: C22H28O3

Mol. weight: 340.2038448

Exact mass: 340.2038448

Ion type: [M-H]-

Precursor m/z: 339.1966

Polarity: negative

InChI:

InChI key:

SMILES:

Instrument information

Instrument type: LC-ESI-QTOF

Instrument: Bruker maXis Impact

Ion source: ESI

Resolution: 35000

Ms level: MS2

Collision energy: 40 eV

Database links

Pubchem: 24728626

MoNA ID: AU239460

Chemspider: 21112020

CAS: 976-71-6

Other information

Principal investigator: Nikiforos Alygizakis,

Data collector: Nikiforos Alygizakis, Katerina

C10-LAS (TENTATIVE)

0.857

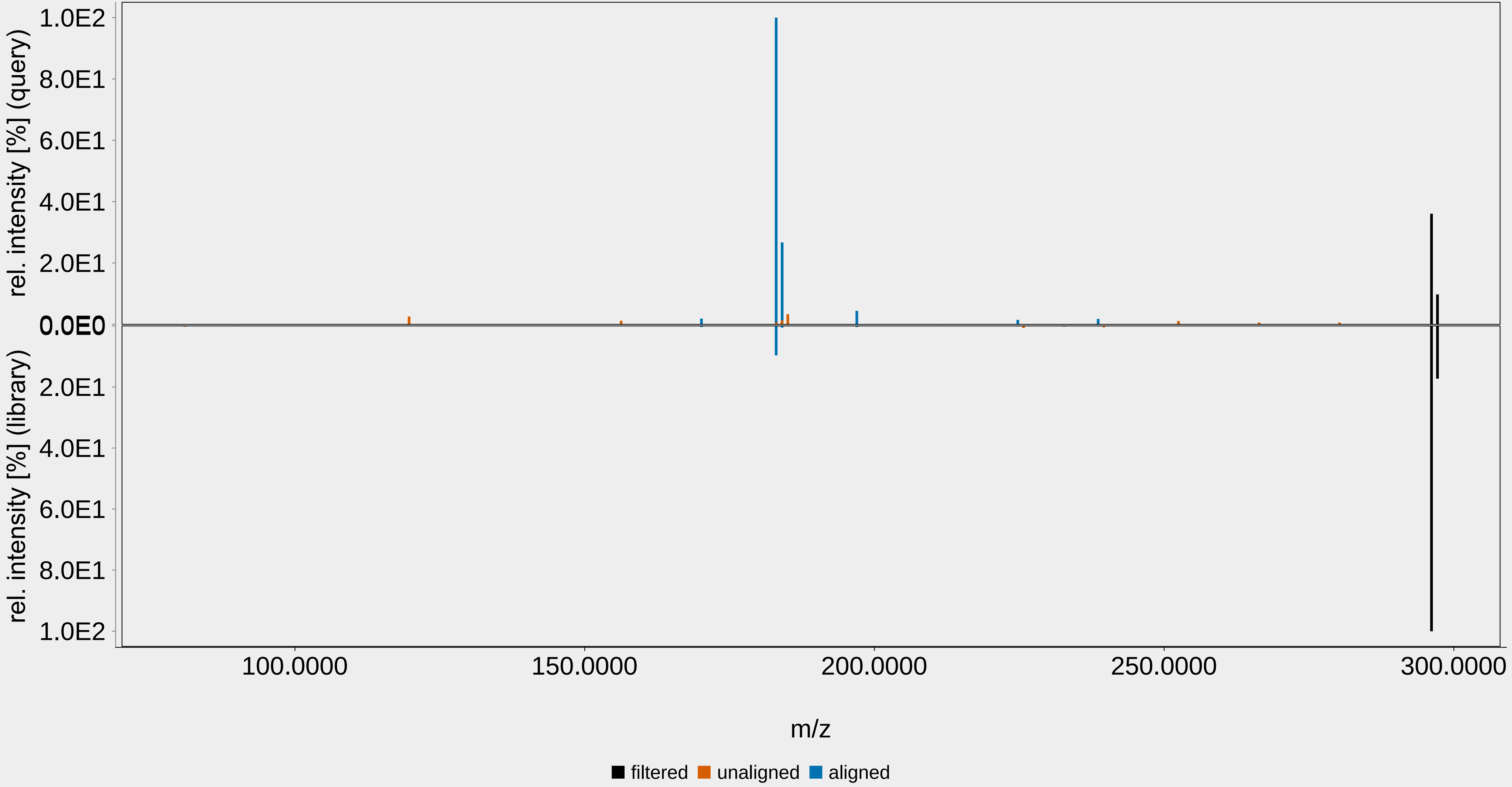

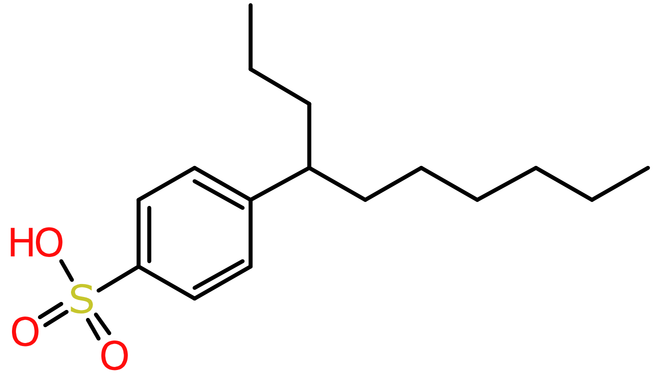

Compound information

Name: C10-LAS (TENTATIVE)

Formula: C16H26O3S

Exact mass: 298.1603

Ion type: [M-H]-

Precursor m/z: 297.1521

Polarity: NEGATIVE

InChI:

InChI key: NANHIUZYPFDGJS-UHFFFAOYSA-

SMILES:

Num peaks: 20

Instrument information

Instrument type: LC-ESI-ITFT

Instrument: LTQ Orbitrap XL Thermo Scientific

Ms level: MS2

Collision energy: 60 NCE

Database links

Other information

Entry ID:

Comment: Parent=297.1521

C10-LAS (TENTATIVE), C10-linear alkylbenzyl sulfonate, 4-(decan-4-yl)benzenesulfonic acid

0.858

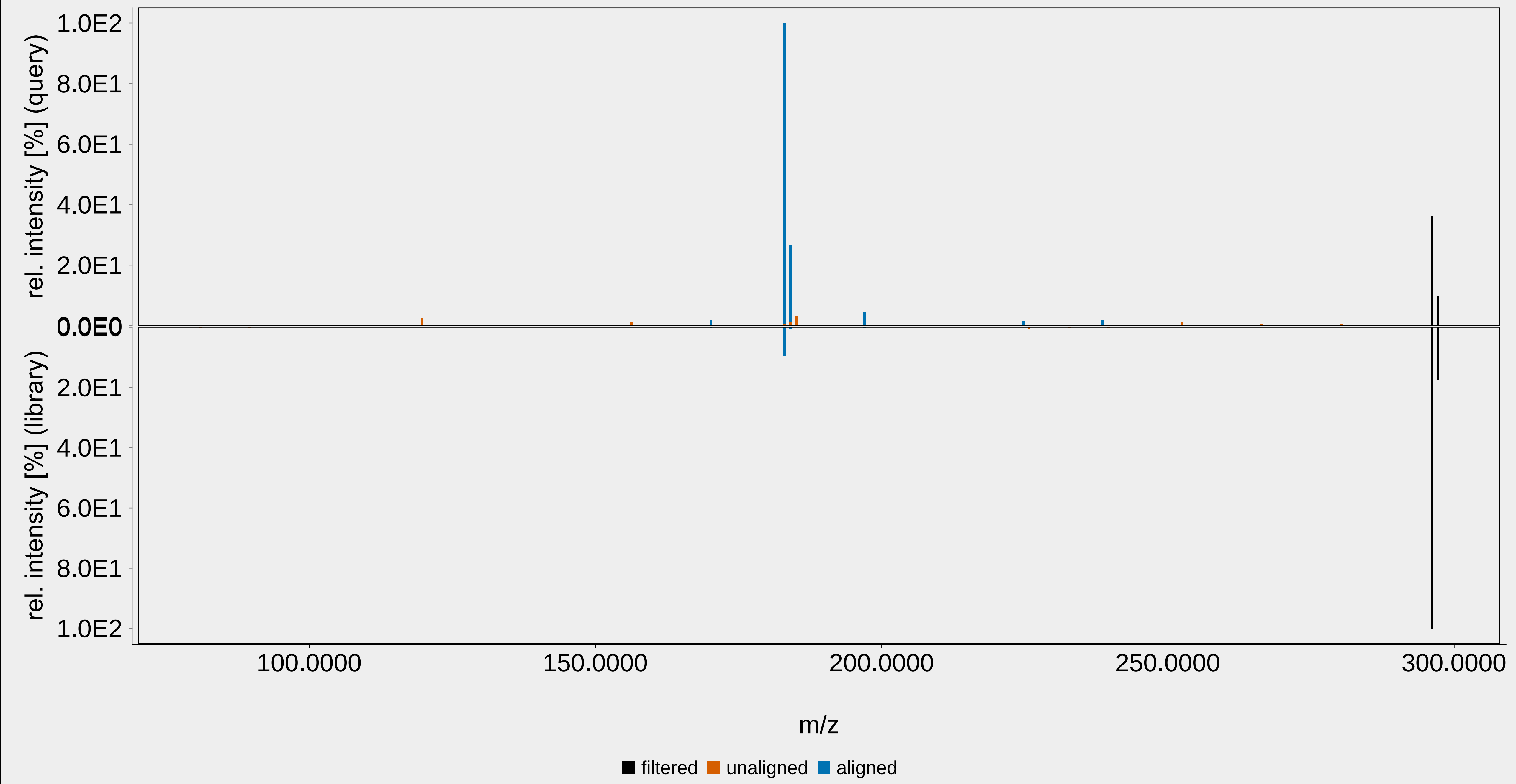

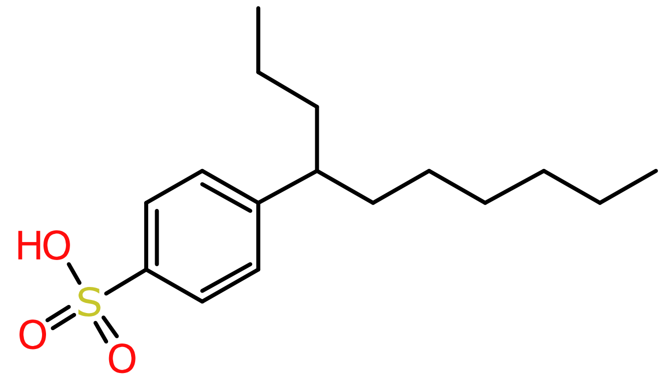

Compound information

Name: C10-LAS (TENTATIVE), C10-linear

Formula: C16H26O3S

Mol. weight: 298.1603

Exact mass: 298.1603

Ion type: [M-H]-

Precursor m/z: 297.1521

Polarity: negative

InChI:

InChI key: NANHIUZYPFDGJS-UHFFFAOYSA-

SMILES:

Instrument information

Instrument type: LC-ESI-ITFT

Instrument: LTQ Orbitrap XL Thermo Scientific

Ion source: ESI

Ms level: MS2

Collision energy: 60 NCE

Database links

Pubchem: 14921167

MoNA ID: ETS00001

Other information

Principal investigator: E. Schymanski, Dept. of

Data collector: E. Schymanski, Dept. of

C11-LAS (STANDARD MIX), C11-linear alkylbenzyl sulfonate, 4-(undecan-5-yl)benzenesulfonic acid

0.862

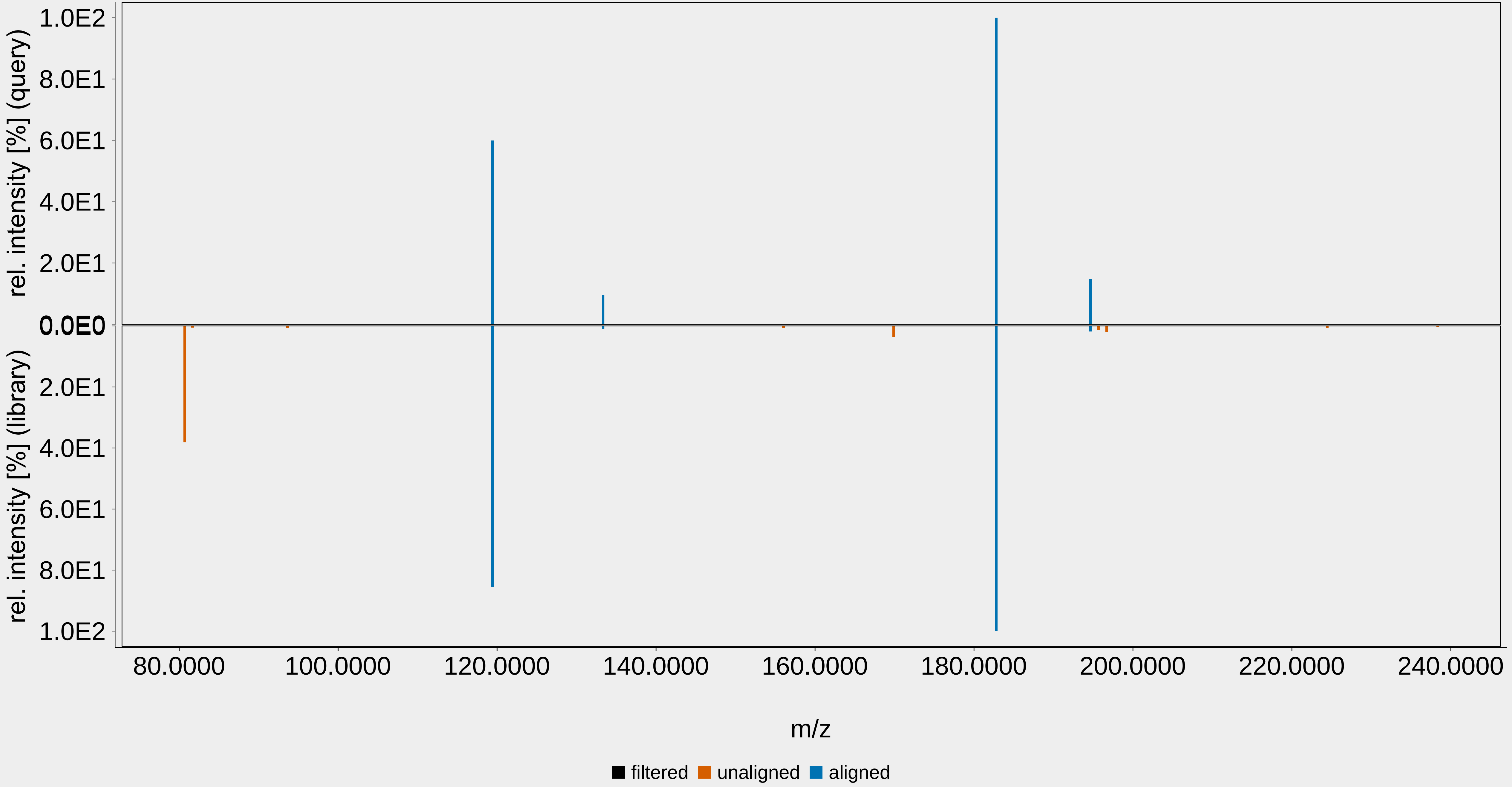

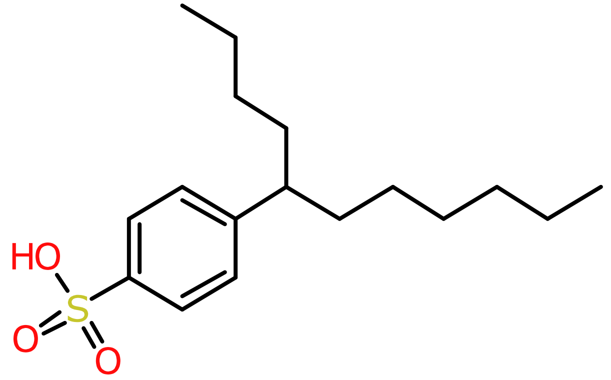

Compound information

Name: C11-LAS (STANDARD MIX), C11-linear

Formula: C17H28O3S

Mol. weight: 312.1759

Exact mass: 312.1759

Ion type: [M-H]-

Precursor m/z: 311.1686

Polarity: negative

InChI:

InChI key:

SMILES:

Instrument information

Instrument type: LC-ESI-QFT

Instrument: Q Exactive Orbitrap Thermo

Ion source: ESI

Ms level: MS2

Collision energy: 90 NCE

Database links

Pubchem: 15593874

MoNA ID: ETS00014

Other information

Principal investigator: E. Schymanski, Dept. of

Data collector: E. Schymanski, Dept. of

C11-LAS (STANDARD MIX)

0.862

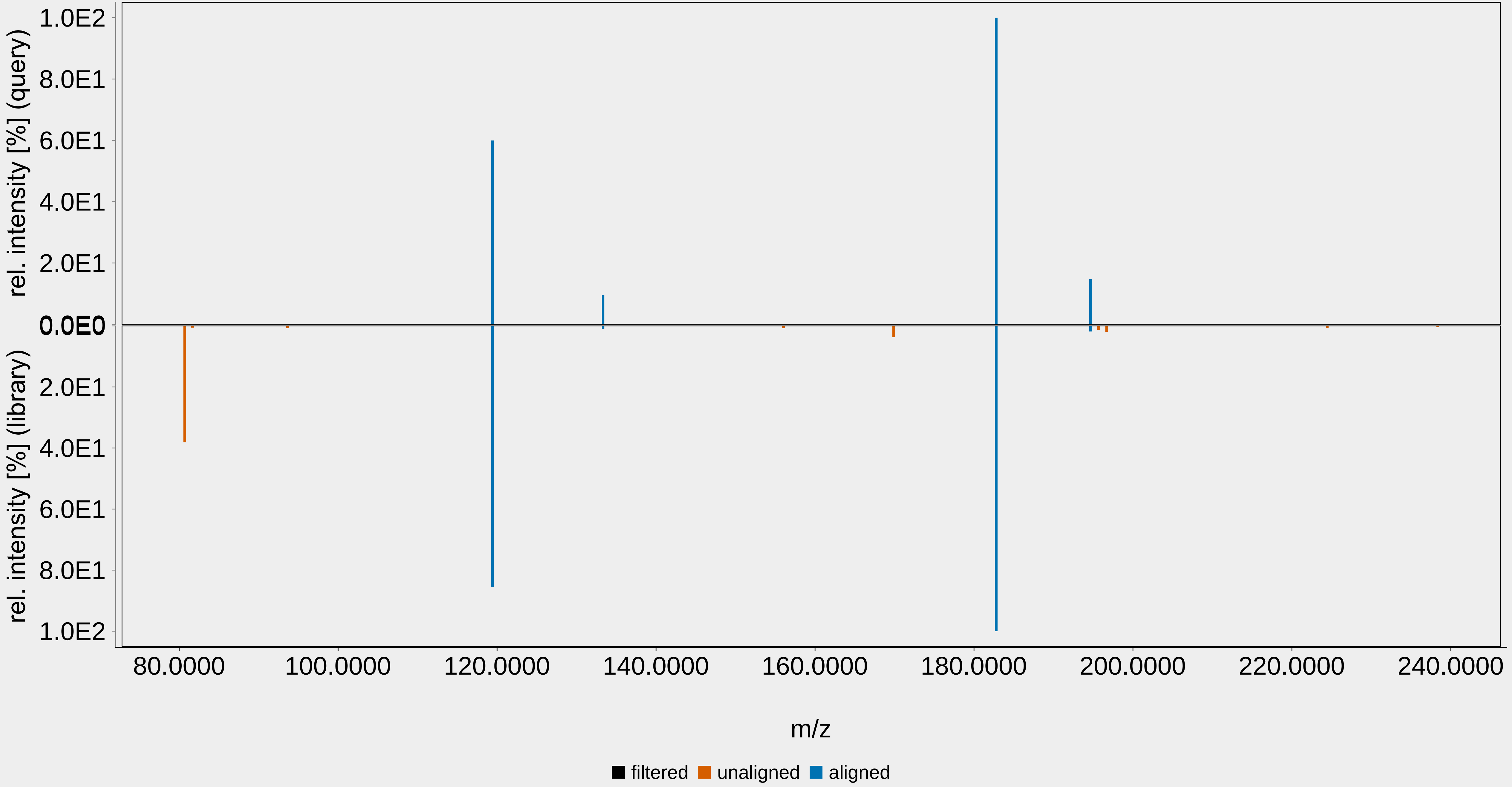

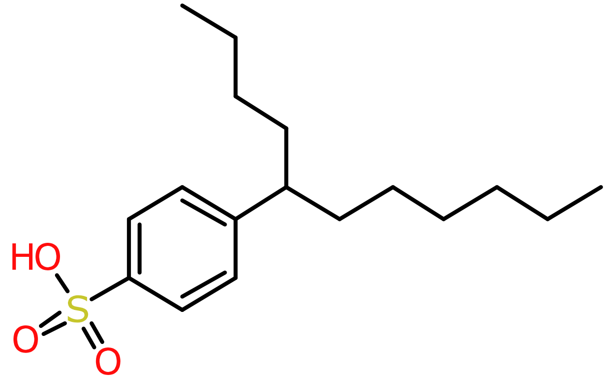

Compound information

Name: C11-LAS (STANDARD MIX)

Formula: C17H28O3S

Exact mass: 312.1759

Ion type: [M-H]-

Precursor m/z: 311.1686

Polarity: NEGATIVE

InChI:

InChI key:

SMILES:

Num peaks: 13

Instrument information

Instrument type: LC-ESI-QFT

Instrument: Q Exactive Orbitrap Thermo

Ms level: MS2

Collision energy: 90 NCE

Database links

Other information

Entry ID:

Comment: Parent=311.1686

Dodecylbenzenesulfonic acid **0.901**

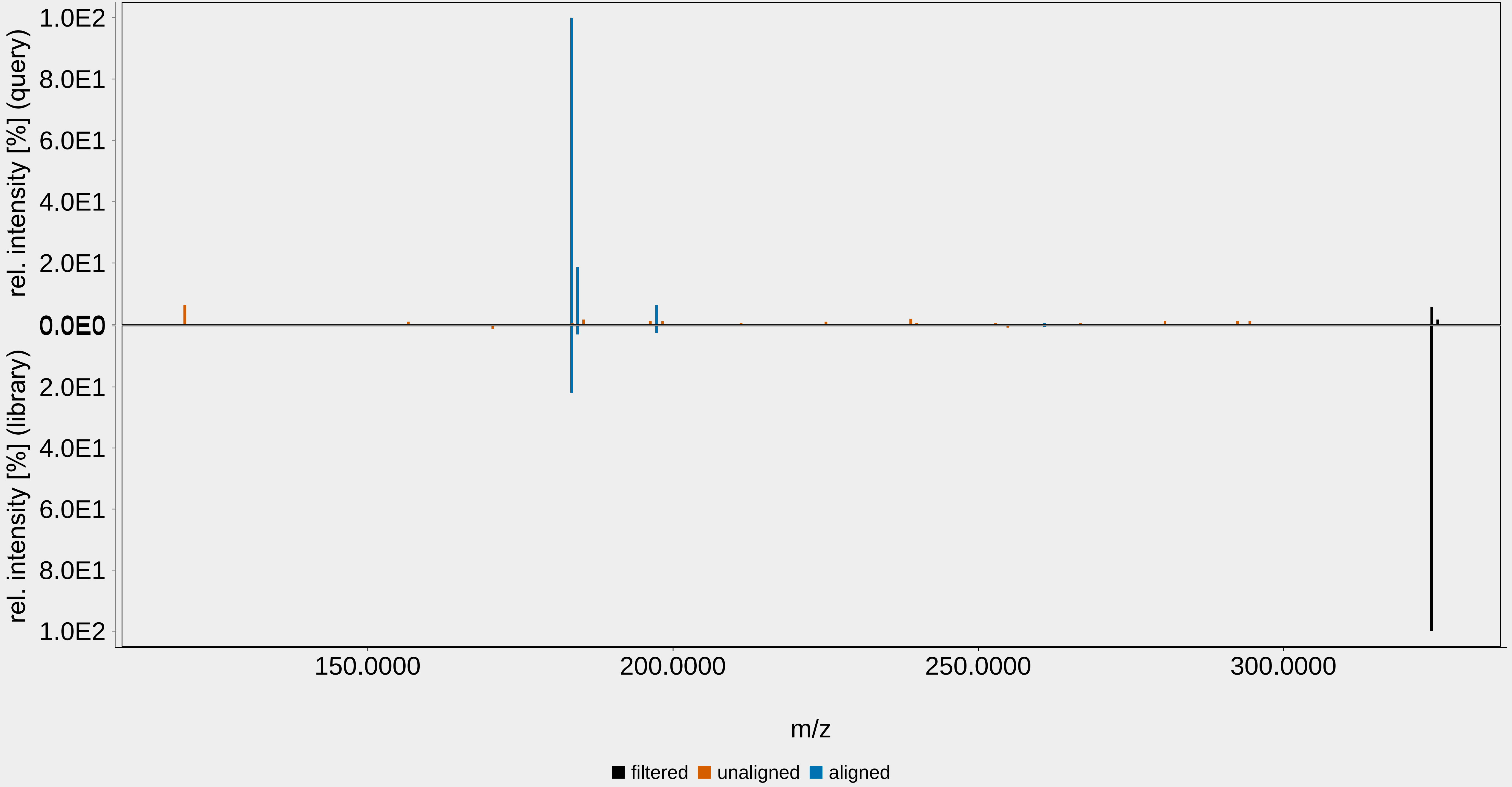

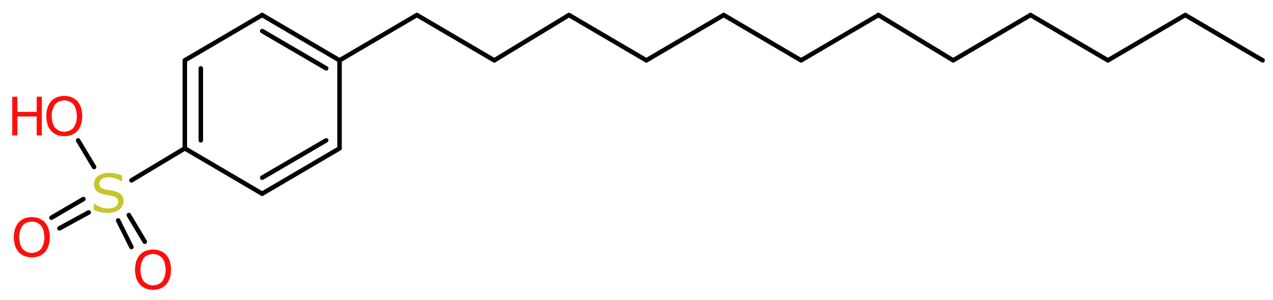

Compound information

Name: Dodecylbenzenesulfonic acid

Formula: C18H30O3S

Exact mass: 326.19157

Ion type: [M-H]-

Precursor m/z: 325.1843

Polarity: NEGATIVE

InChI:

InChI key:

SMILES:

Num peaks: 7

Instrument information

Instrument type: LC-ESI-QFT

Instrument: Q Exactive Plus Orbitrap Thermo

Ms level: MS2

Collision energy: 35 (nominal)

Database links

Other information

Entry ID: MSBNK-CASMI_2016-SM876251

Comment: Parent=325.1843

Dodecylbenzenesulfonic acid

0.900

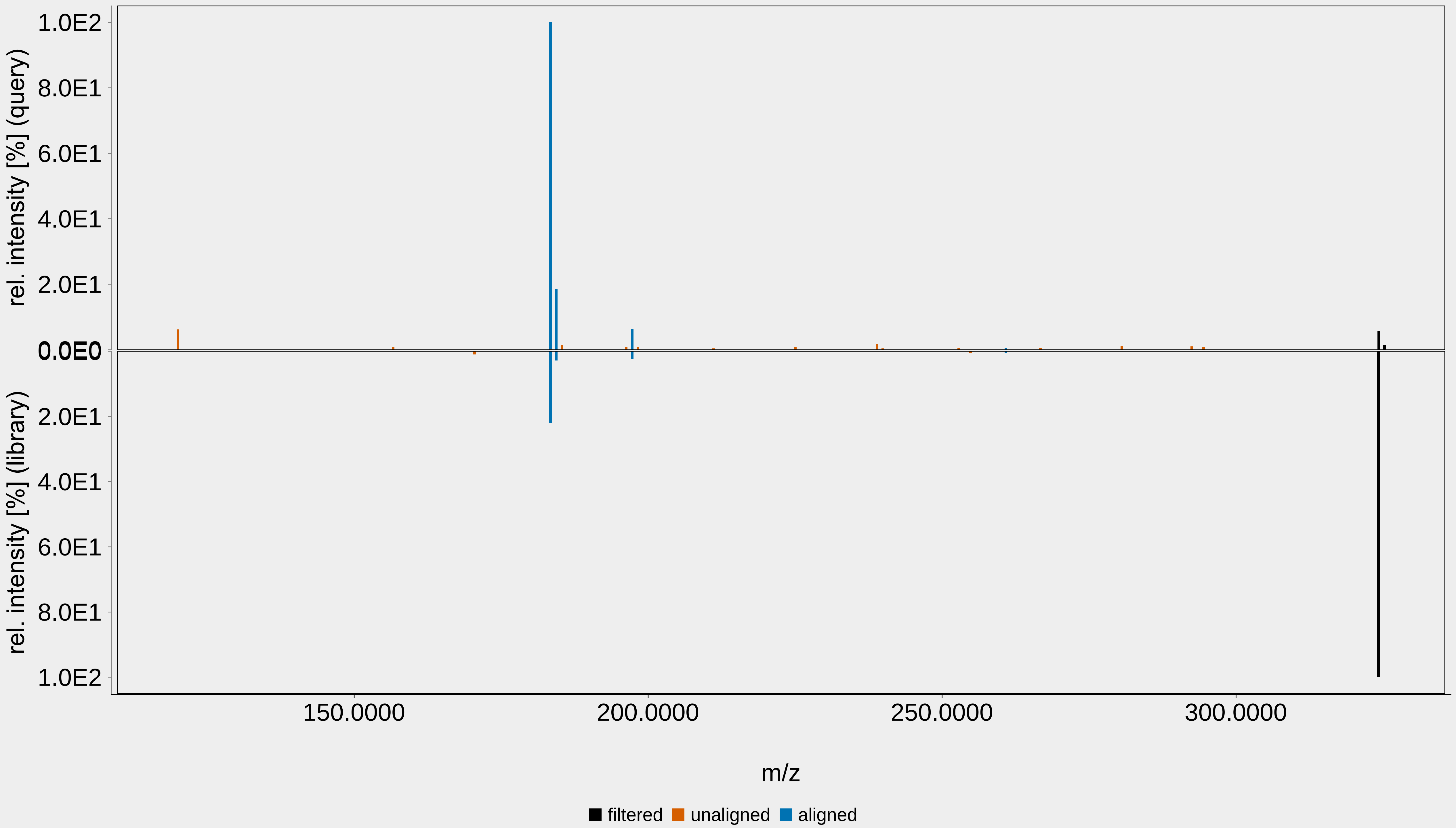

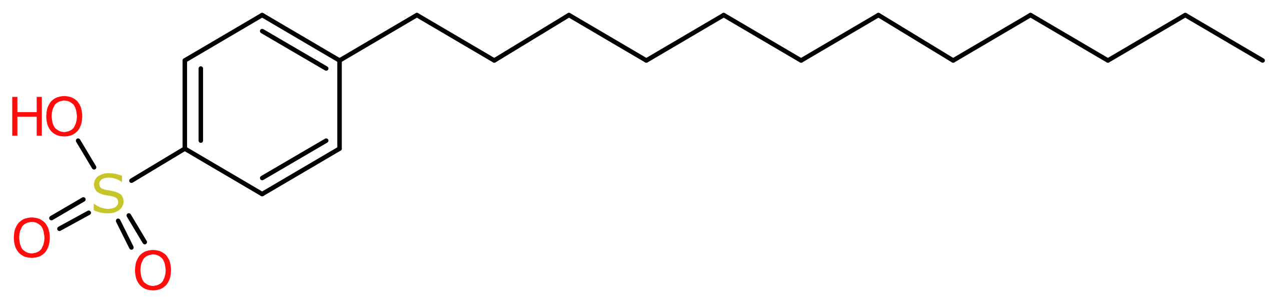

Compound information

Name: Dodecylbenzenesulfonic acid

Formula: C18H30O3S

Mol. weight: 326.19157

Exact mass: 326.19157

Ion type: [M-H]-

Precursor m/z: 325.1843

Polarity: negative

InChI:

InChI key:

SMILES:

Instrument information

Instrument type: LC-ESI-QFT

Instrument: Q Exactive Plus Orbitrap Thermo

Ion source: ESI

Resolution: 35000

Ms level: MS2

Collision energy: 35 (nominal)

Database links

Pubchem: 8485

MoNA ID: SM876251

Chemspider: 8172

CAS: 121-65-3

Other information

Principal investigator: Krauss M, Schymanski

Data collector: Krauss M, Schymanski EL,

Glyceryl phosphate - 30.0 eV

0.985

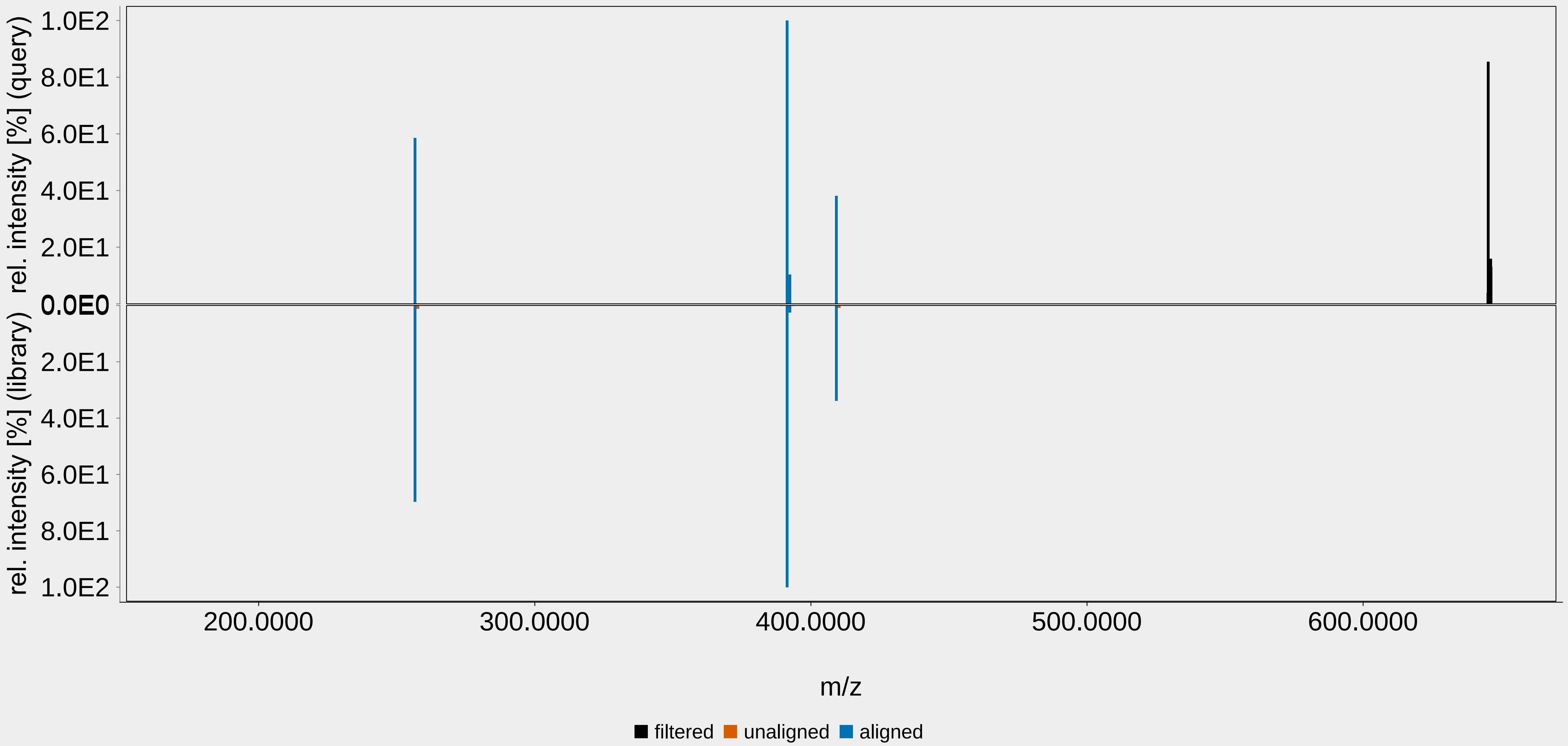

Compound information

Name: Glyceryl phosphate - 30.0 eV

Exact mass: 0.0

Ion type: Unknown

Precursor m/z: 647.466

Charge: 1

Polarity: Negative

SMILES: C([C@H](COP(=O)(O)O)O)O

Instrument information

Instrument type: Orbitrap

Ion source: ESI

Ms level: 2

Acquisition: Isolated

Database links

CAS:

Other information

Principal investigator: PI

Data collector: Data Collector

Entry ID: CCMSLIB00006126789

Glyceryl phosphate - 30.0 eV

0.986

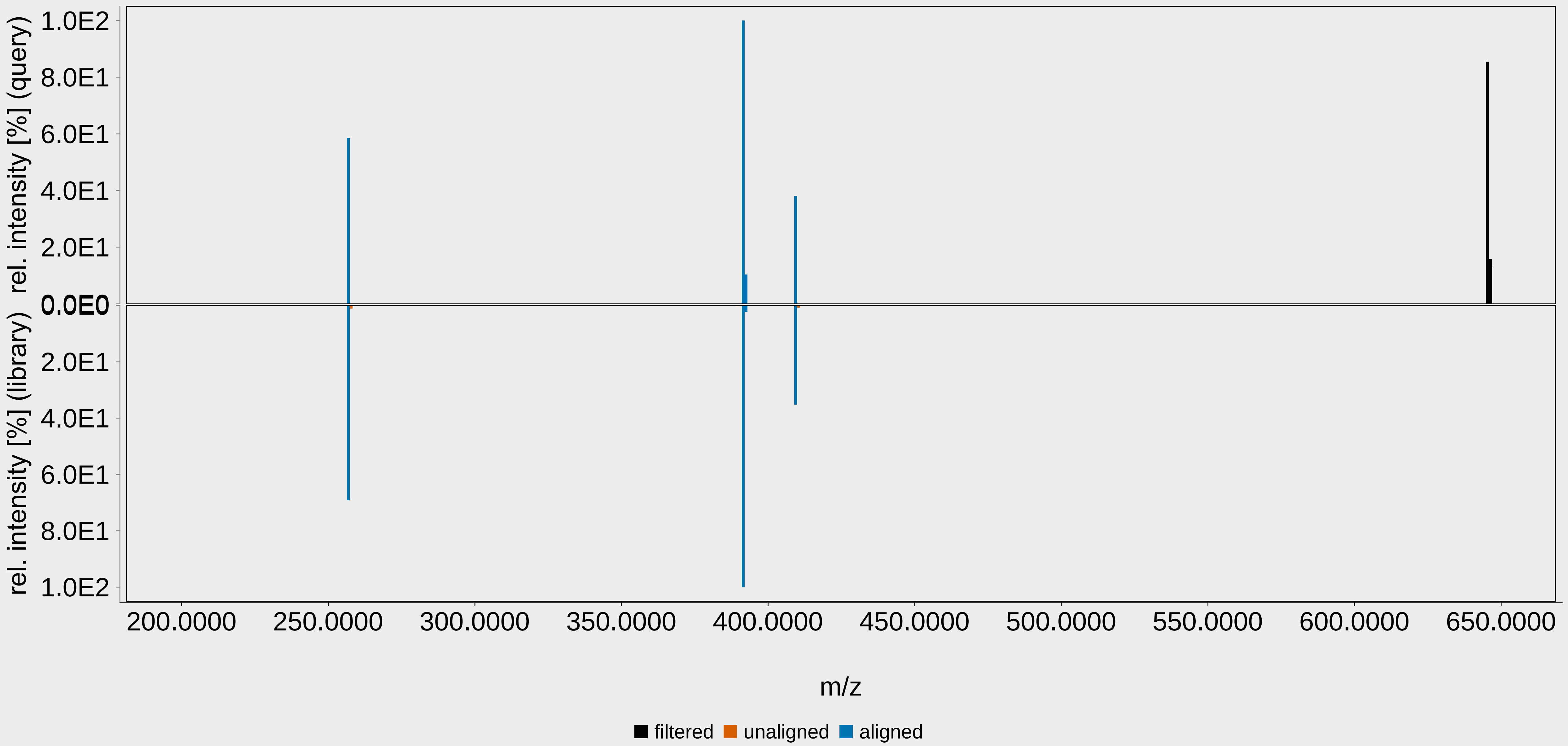

Compound information

Name: Glyceryl phosphate - 30.0 eV

Exact mass: 0.0

Ion type: Unknown

Precursor m/z: 647.466

Charge: 1

Polarity: Negative

SMILES: C([C@H](COP(=O)(O)O)O)O

Instrument information

Instrument type: Orbitrap

Ion source: ESI

Ms level: 2

Acquisition: Isolated

Database links

CAS:

Other information

Principal investigator: PI

Data collector: Data Collector

Entry ID: CCMSLIB00006126790

Dodecylbenzenesulfonic acid

0.939

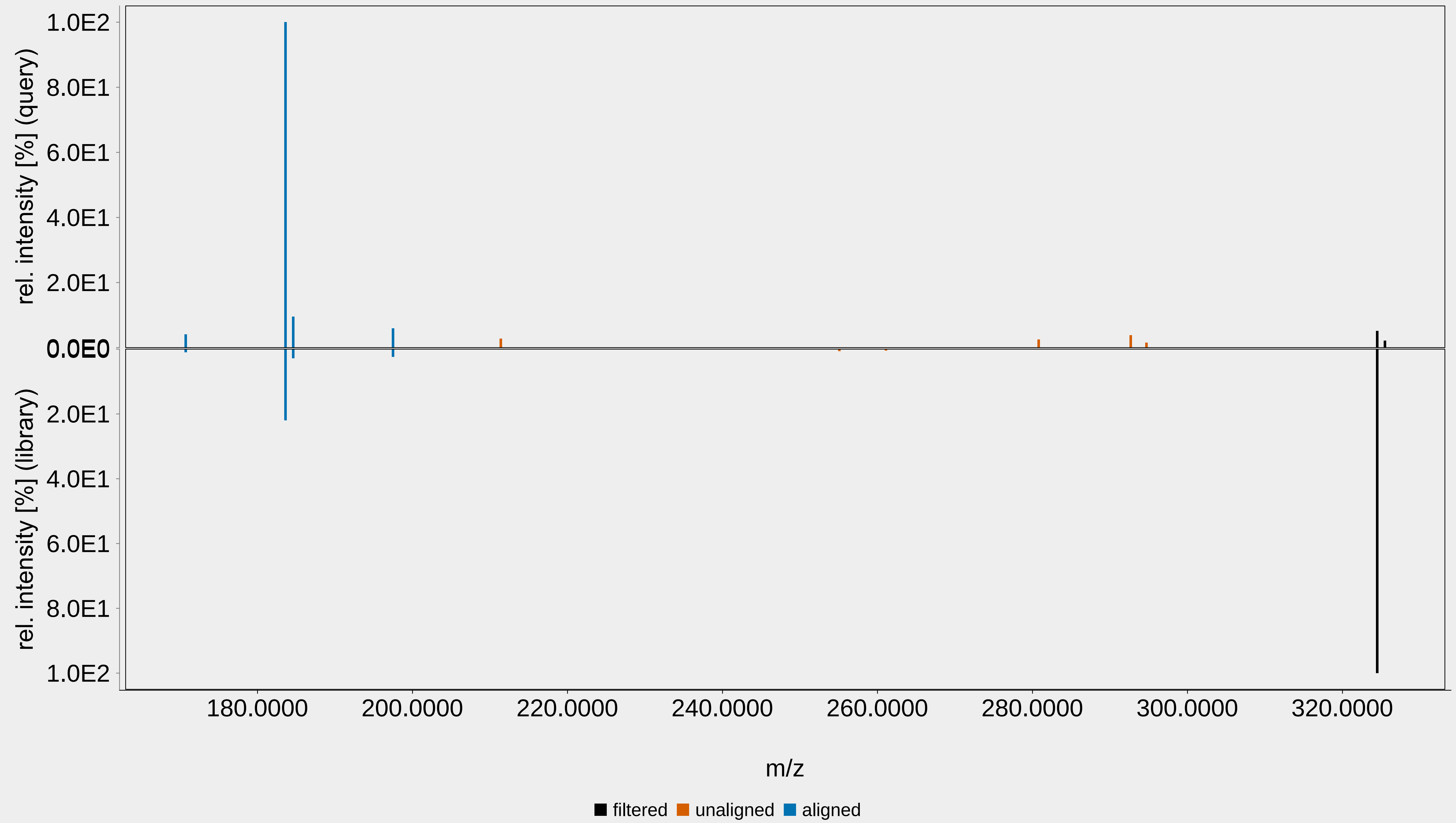

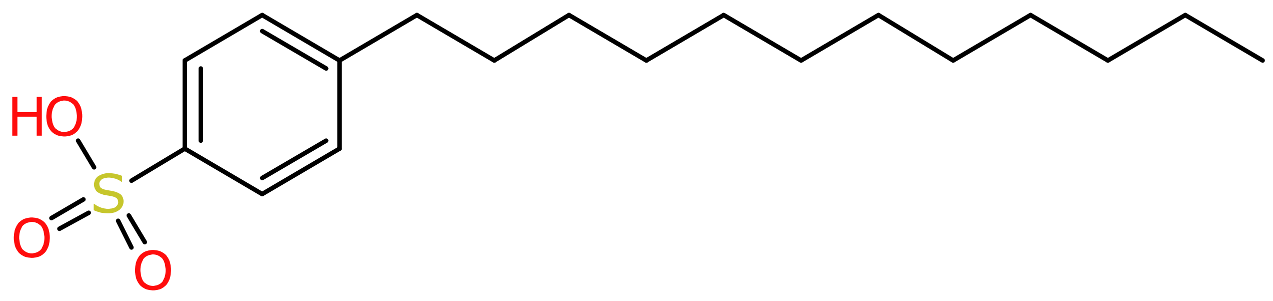

Compound information

Name: Dodecylbenzenesulfonic acid

Formula: C18H30O3S

Mol. weight: 326.19157

Exact mass: 326.19157

Ion type: [M-H]-

Precursor m/z: 325.1843

Polarity: negative

InChI:

InChI key:

SMILES:

Instrument information

Instrument type: LC-ESI-QFT

Instrument: Q Exactive Plus Orbitrap Thermo

Ion source: ESI

Resolution: 35000

Ms level: MS2

Collision energy: 35 (nominal)

Database links

Pubchem: 8485

MoNA ID: SM876251

Chemspider: 8172

CAS: 121-65-3

Other information

Principal investigator: Krauss M, Schymanski

Data collector: Krauss M, Schymanski EL,

Dodecylbenzenesulfonic acid

0.940

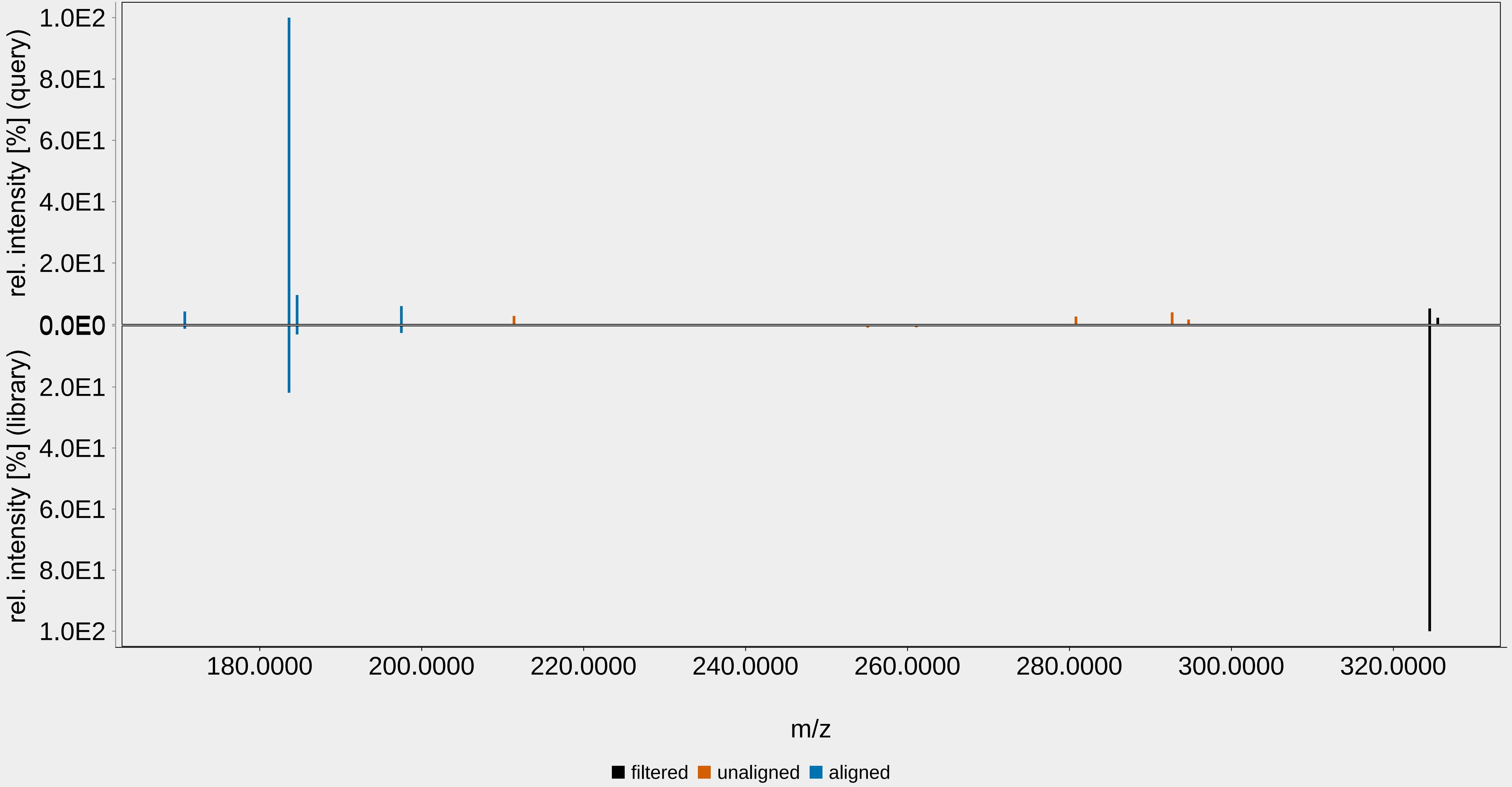

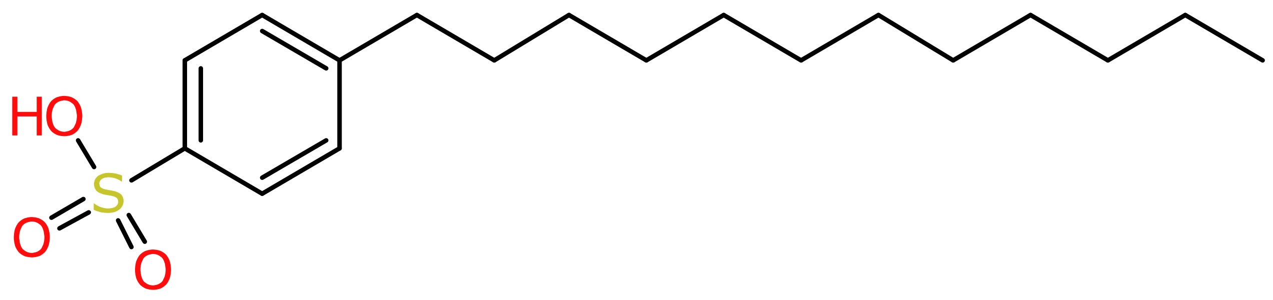

Compound information

Name: Dodecylbenzenesulfonic acid

Formula: C18H30O3S

Exact mass: 326.19157

Ion type: [M-H]-

Precursor m/z: 325.1843

Polarity: NEGATIVE

InChI:

InChI key:

SMILES:

Num peaks: 7

Instrument information

Instrument type: LC-ESI-QFT

Instrument: Q Exactive Plus Orbitrap Thermo

Ms level: MS2

Collision energy: 35 (nominal)

Database links

Other information

Entry ID: MSBNK-CASMI_2016-SM876251

Comment: Parent=325.1843

PS 16:0_22:6 **0.854**

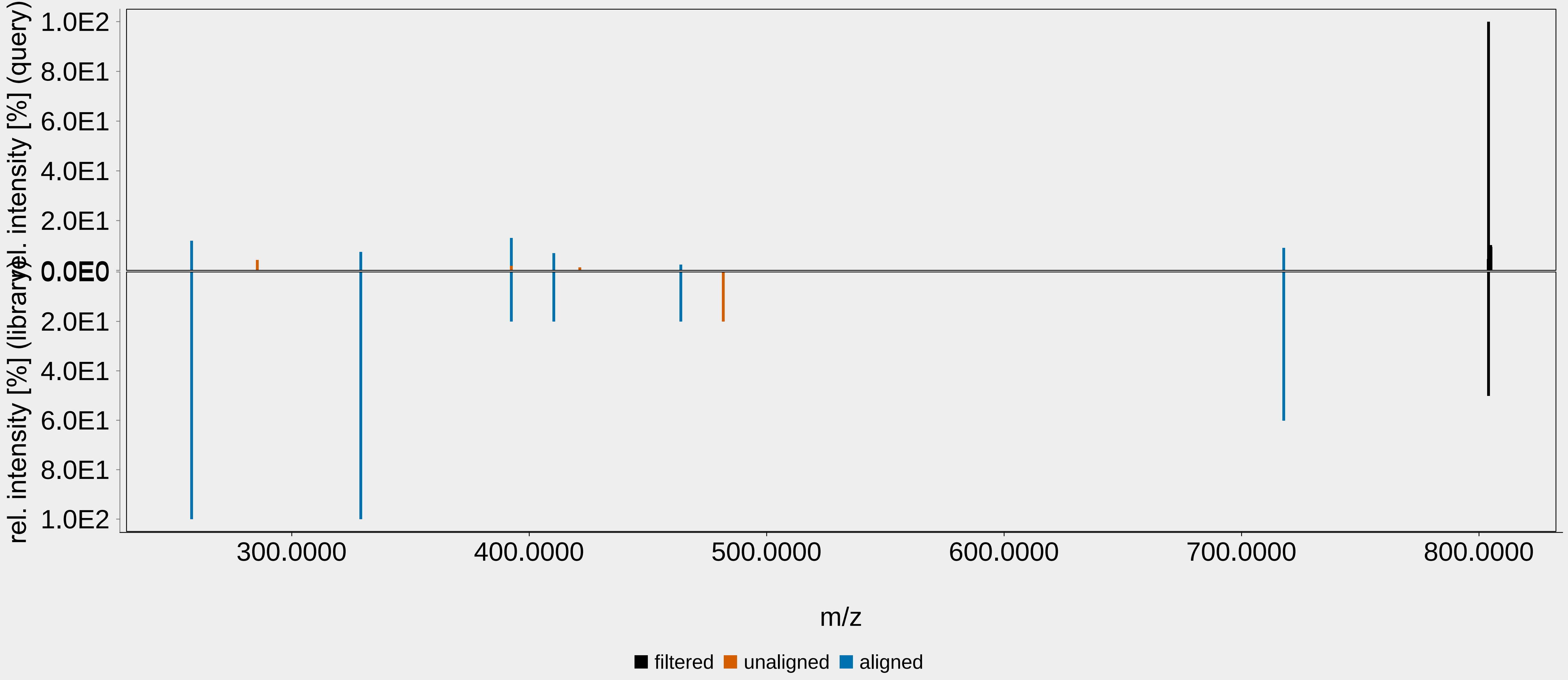

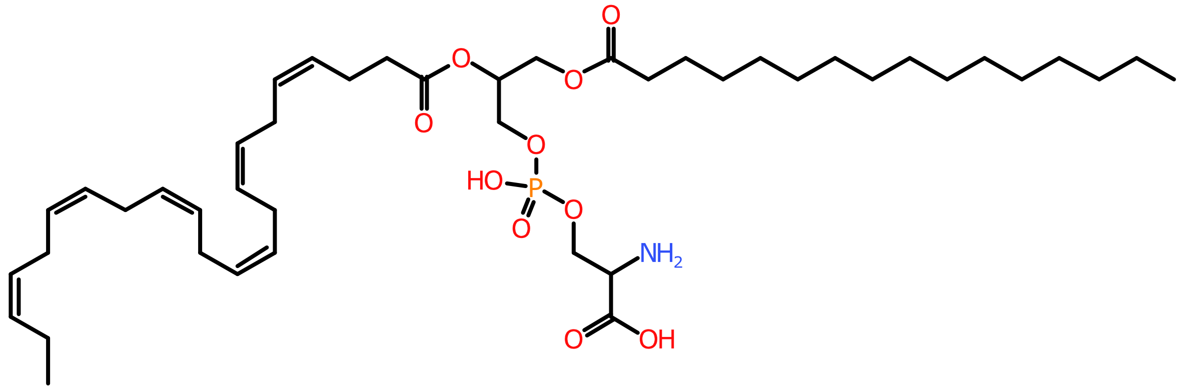

Compound information

Name: PS 16:0_22:6

Formula: C44H74NO10P

Ion type: [M-H]-

Precursor m/z: 806.49776

Ccs: 276.61

InChI key:

SMILES:

Num peaks: 8

Instrument information

Database links

Other information

PS 38:6 **0.850**

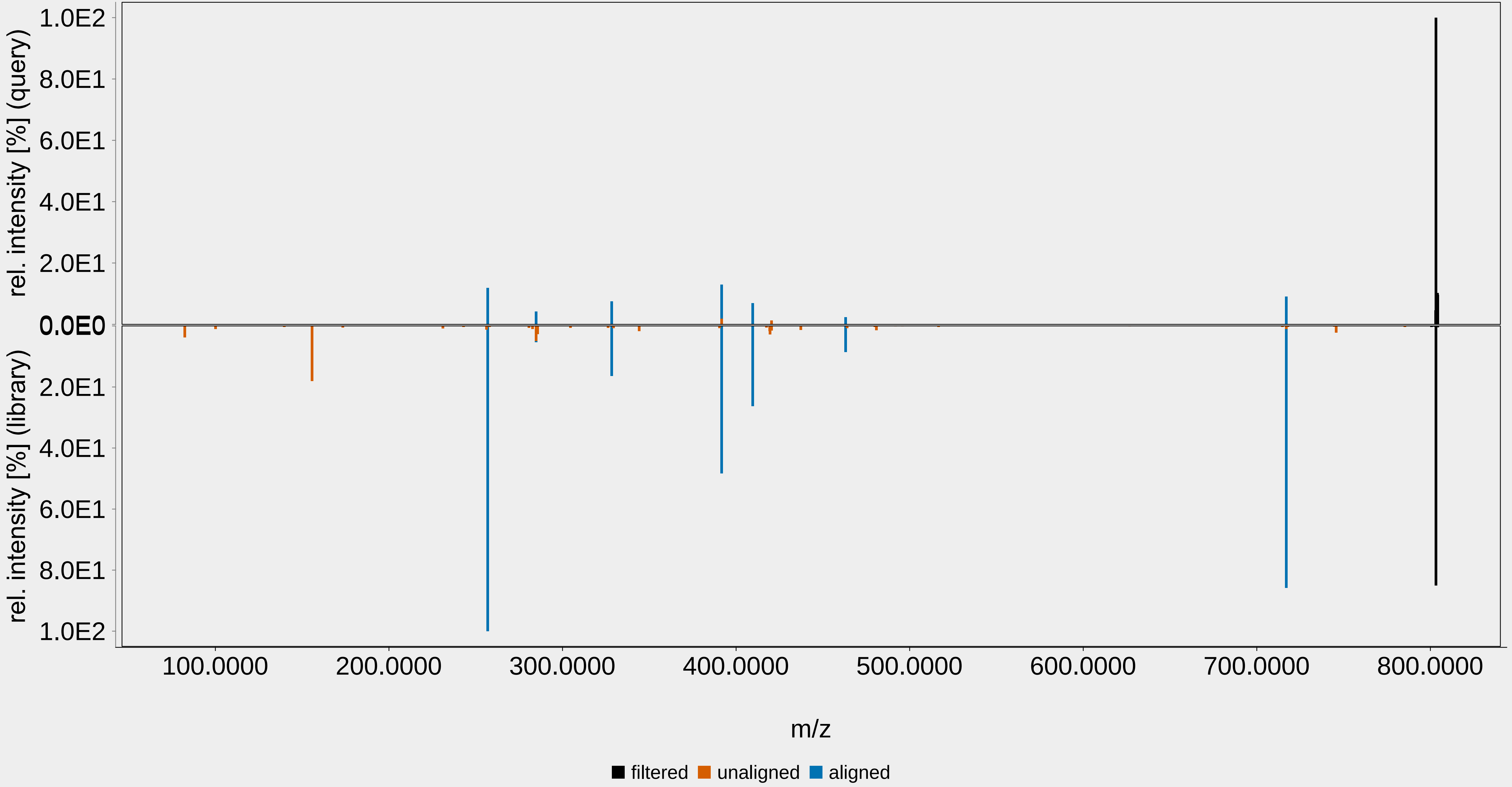

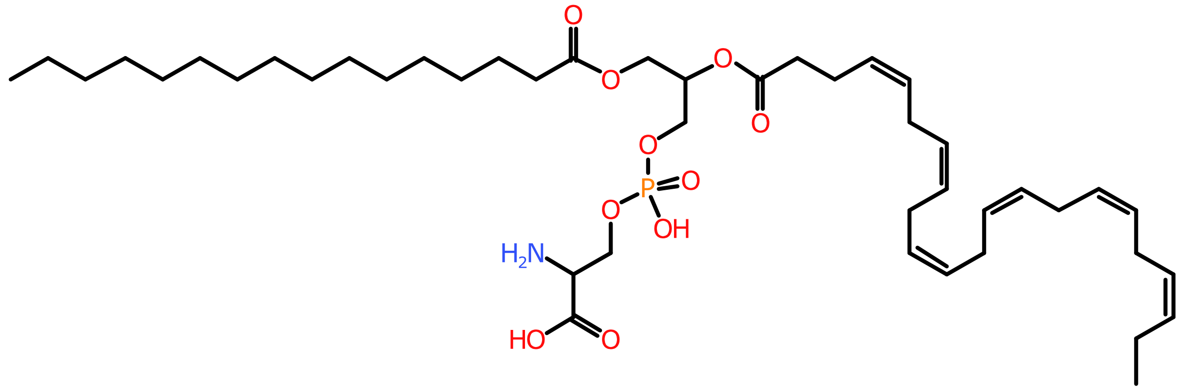

Compound information

Name: PS 38:6

Formula: C44H74NO10P

Exact mass: 808.0489999999999

Ion type: [M-H]-

Precursor m/z: 806.5011

Polarity: NEGATIVE

InChI:

InChI key:

SMILES:

Num peaks: 57

Instrument information

Instrument type: LC-ESI-QTOF

Instrument: AB Sciex TripleTOF 5600+ system

Ms level: MS2

Collision energy: 45V

Database links

Other information

Entry ID: MSBNK-RIKEN_IMS-LQB00330

Comment: Parent=806.5011

PE(16:0/16:0); [M-H]- C37H73N1O8P1

0.926

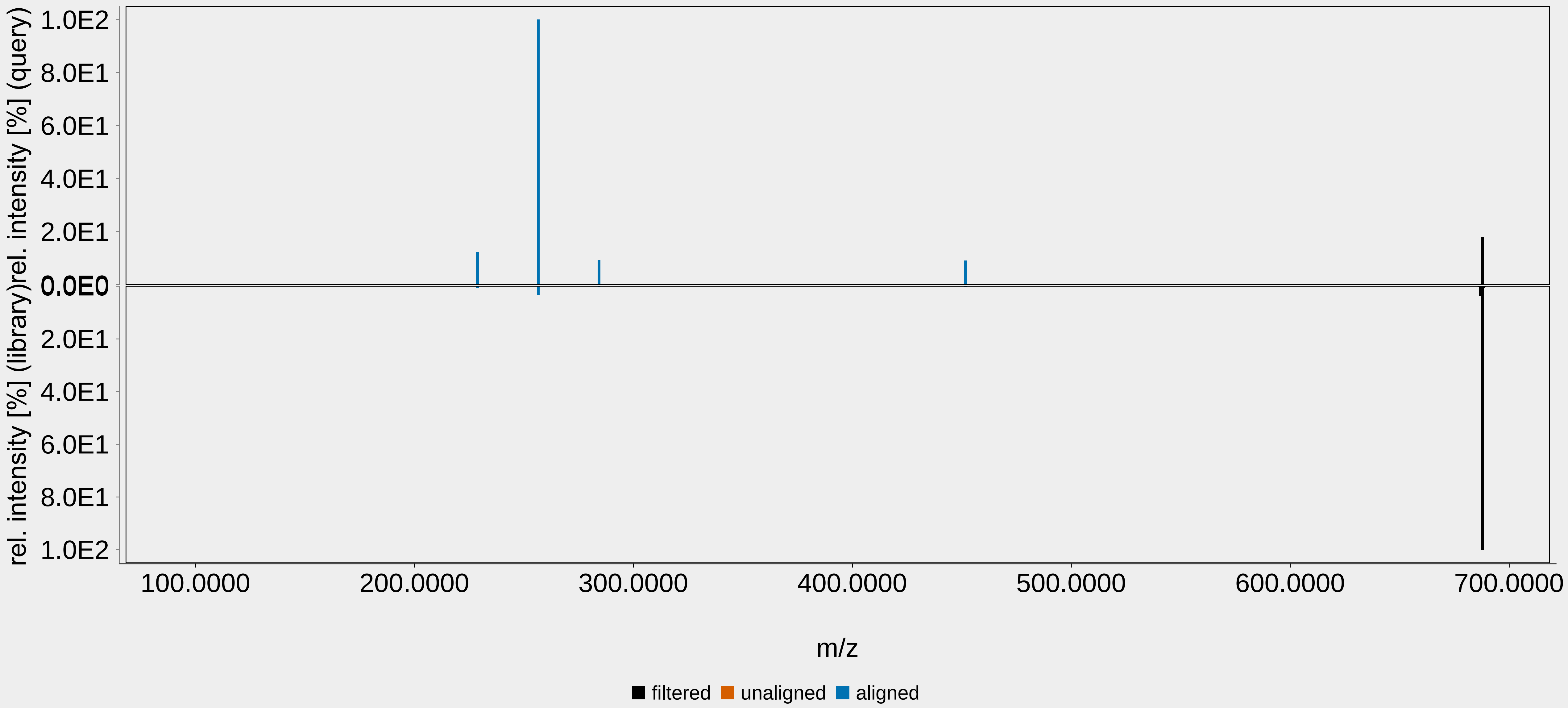

Compound information

Name: PE(16:0/16:0); [M-H]- C37H73N1O8P1

Exact mass: 0.0

Ion type: M-H

Precursor m/z: 690.507

Charge: 1

Polarity: Negative

Instrument information

Instrument type: HCD; Velos

Ion source: LC-ESI

Ms level: 2

Acquisition: Commercial

Database links

Other information

Principal investigator: Thomas Metz

Data collector: Thomas Metz

Entry ID: CCMSLIB00010069794

PE(16:0/16:0); [M-H]- C37H73N1O8P1 0.944

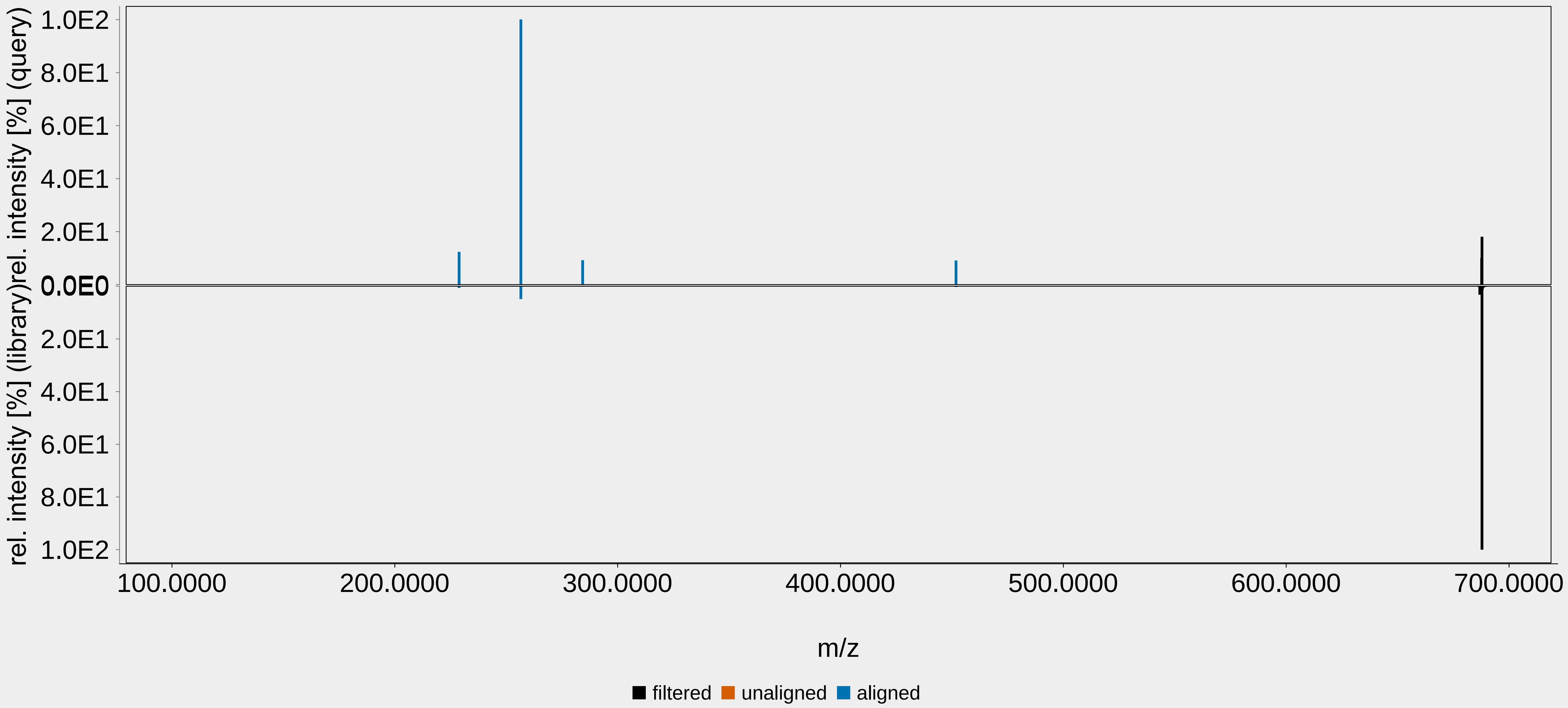

Compound information

Name: PE(16:0/16:0); [M-H]- C37H73N1O8P1

Exact mass: 0.0

Ion type: M-H

Precursor m/z: 690.507

Charge: 1

Polarity: Negative

Instrument information

Instrument type: HCD; Velos

Ion source: LC-ESI

Ms level: 2

Acquisition: Commercial

Database links

Other information

Principal investigator: Thomas Metz

Data collector: Thomas Metz

Entry ID: CCMSLIB00010068325

PA(18:2/18:2); [M-H]- C39H68O8P1 **0.721**

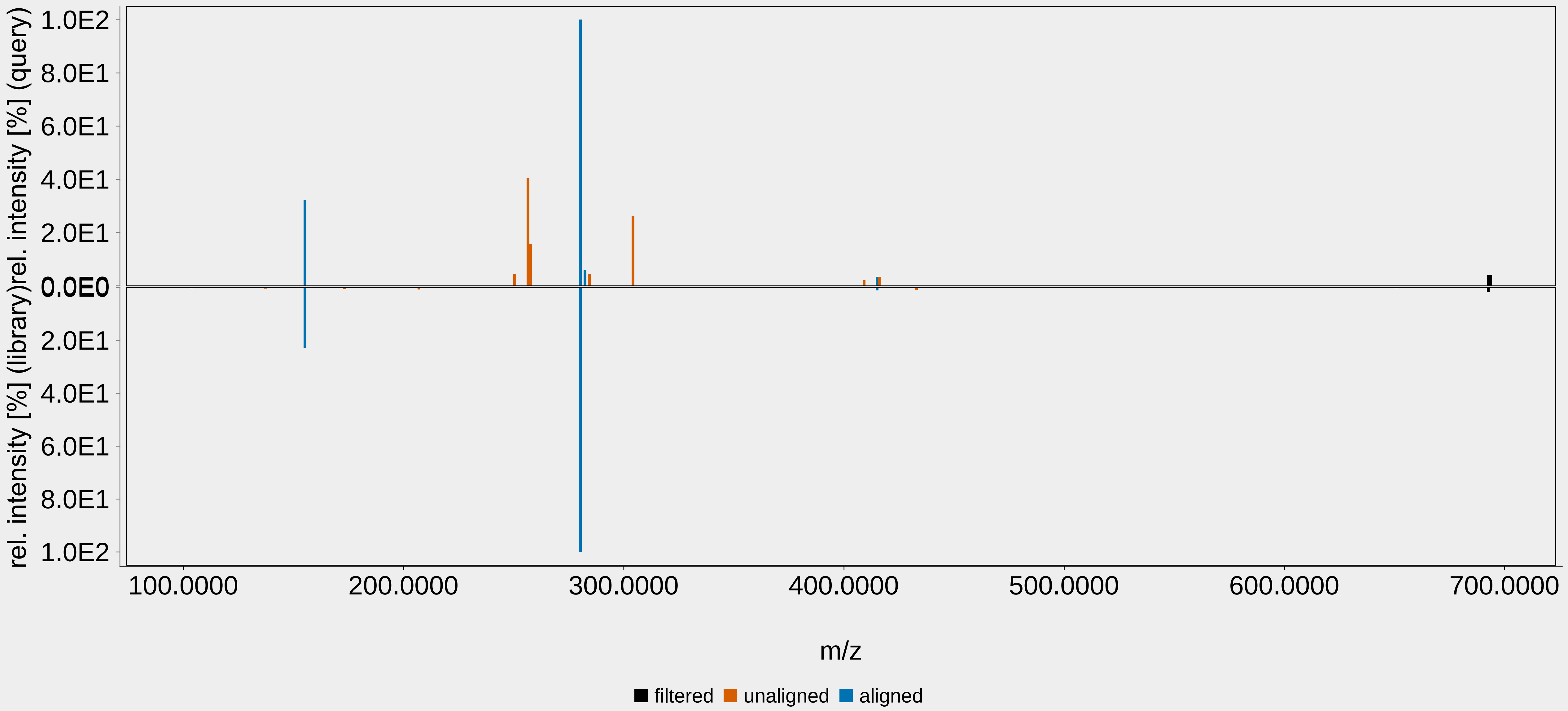

Compound information

Name: PA(18:2/18:2); [M-H]- C39H68O8P1

Exact mass: 0.0

Ion type: M-H

Precursor m/z: 695.465

Charge: 1

Polarity: Negative

Instrument information

Instrument type: HCD; Lumos

Ion source: LC-ESI

Ms level: 2

Acquisition: Commercial

Database links

Other information

Principal investigator: Thomas Metz

Data collector: Thomas Metz

Entry ID: CCMSLIB00010059139

PA 16:0_20:4 **0.903**

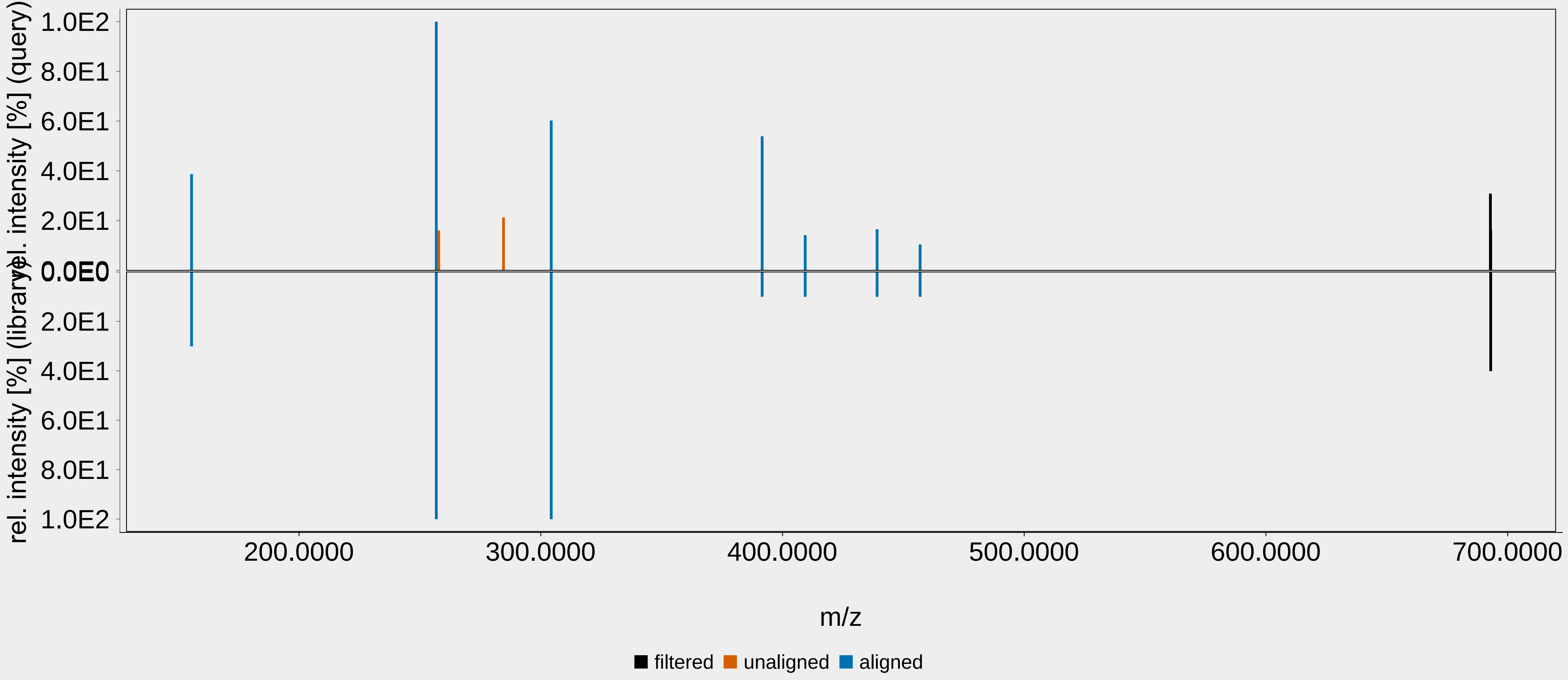

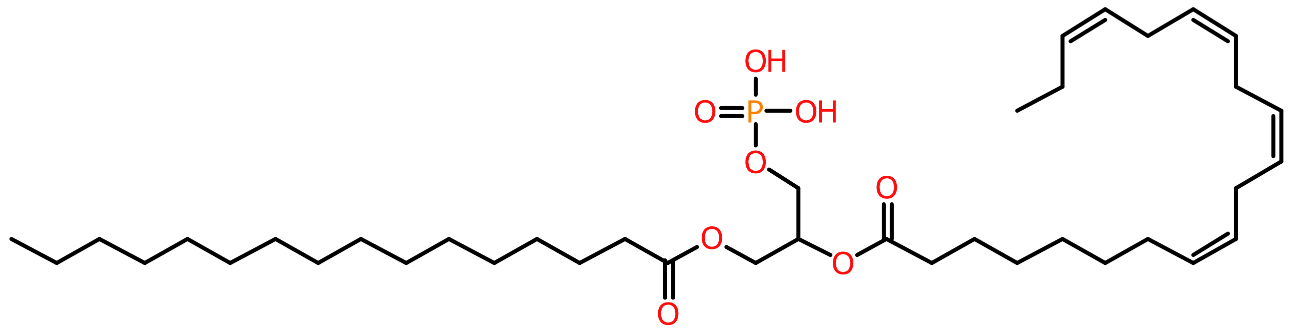

Compound information

Name: PA 16:0_20:4

Formula: C39H69O8P

Ion type: [M-H]-

Precursor m/z: 695.46573

Ccs: 255.1

InChI key:

SMILES:

Num peaks: 8

Instrument information

Database links

Other information

PS(18:1/22:6); [M-H]- C46H75N1O10P1 **0.815**

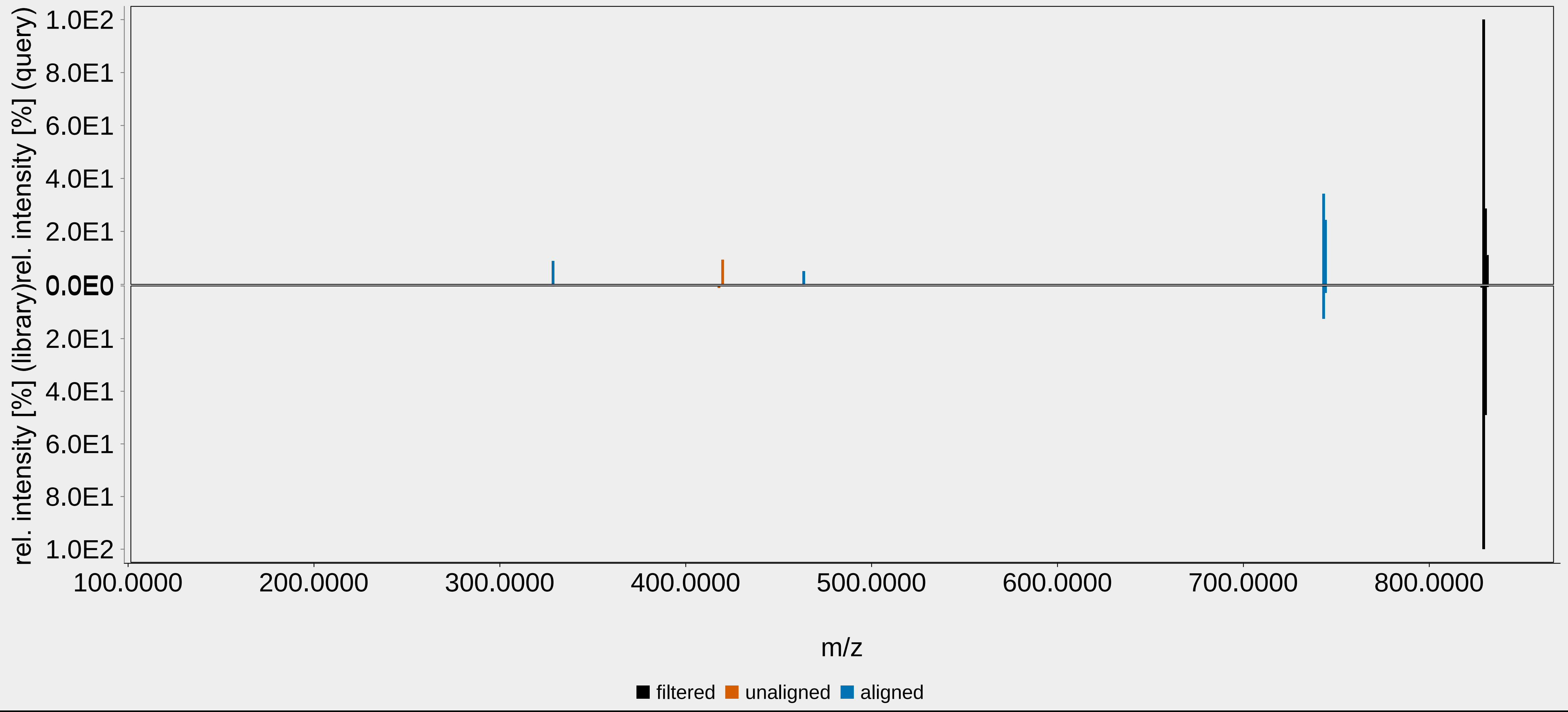

Compound information

Name: PS(18:1/22:6); [M-H]- C46H75N1O10P1

Exact mass: 0.0

Ion type: M-H

Precursor m/z: 832.513

Charge: 1

Polarity: Negative

Instrument information

Instrument type: HCD; Velos

Ion source: LC-ESI

Ms level: 2

Acquisition: Commercial

Database links

Other information

Principal investigator: Thomas Metz

Data collector: Thomas Metz

Entry ID: CCMSLIB00010083738

PS(18:1/22:6); [M-H]- C46H75N1O10P1

0.800

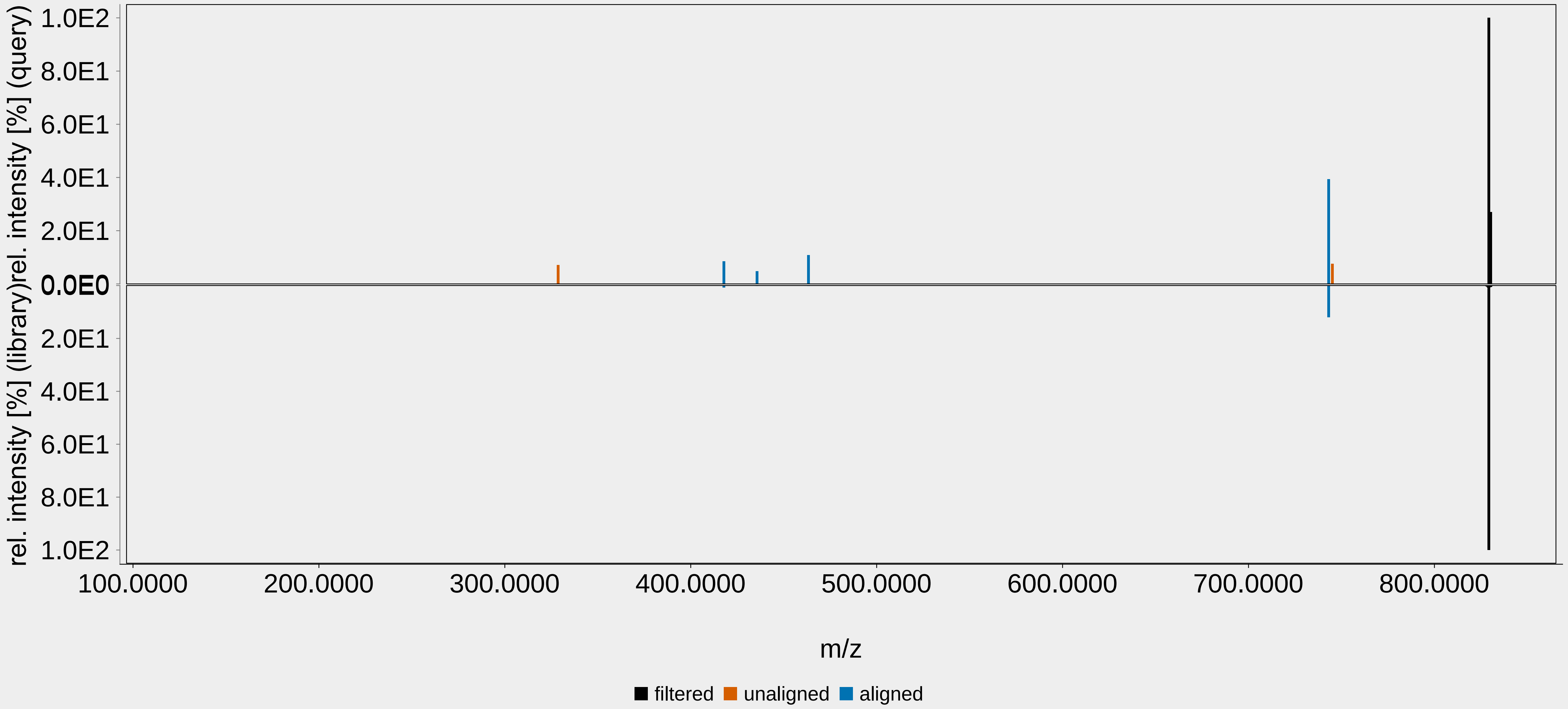

Compound information

Name: PS(18:1/22:6); [M-H]- C46H75N1O10P1

Exact mass: 0.0

Ion type: M-H

Precursor m/z: 832.513

Charge: 1

Polarity: Negative

Instrument information

Instrument type: HCD; Velos

Ion source: LC-ESI

Ms level: 2

Acquisition: Commercial

Database links

Other information

Principal investigator: Thomas Metz

Data collector: Thomas Metz

Entry ID: CCMSLIB00010063314

PS 40:6 **0.892**

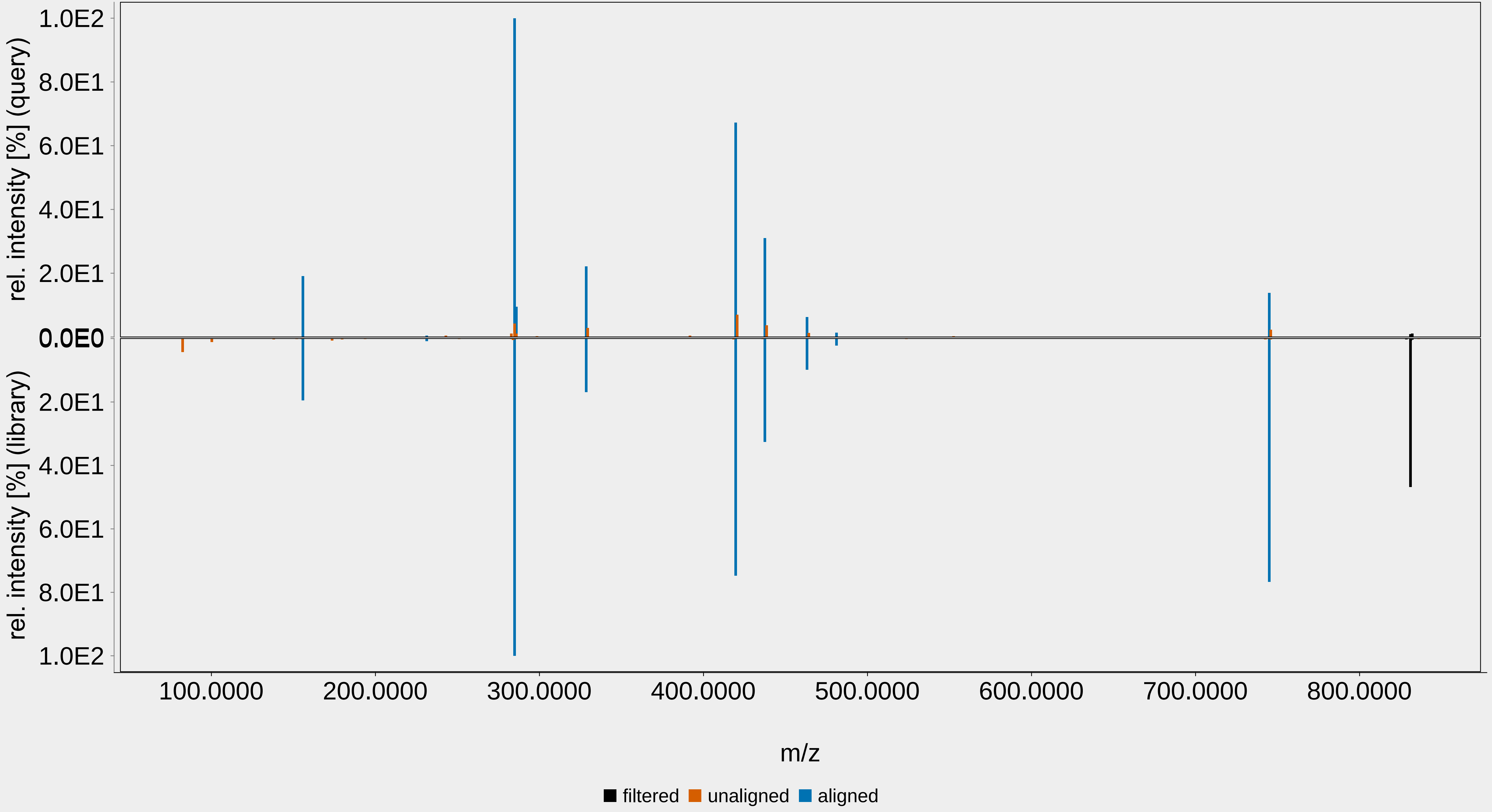

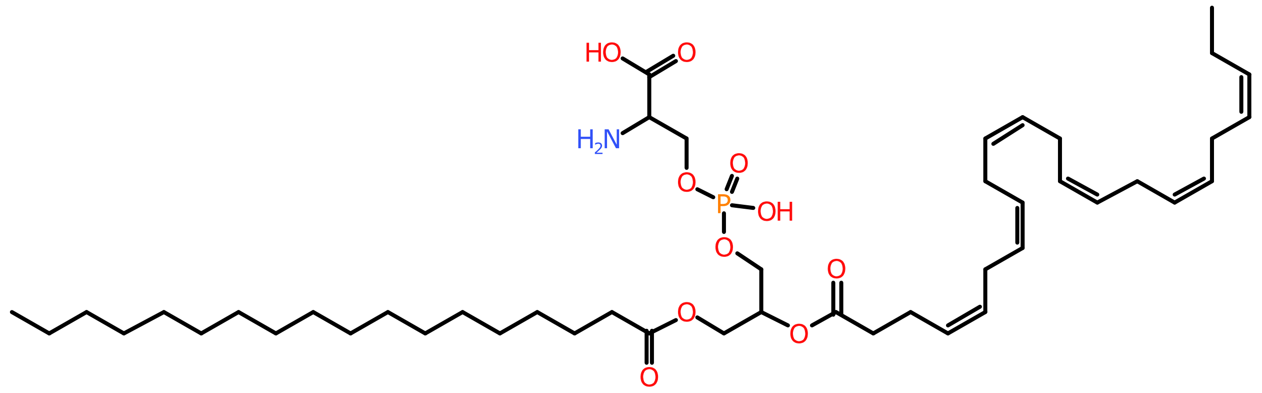

Compound information

Name: PS 40:6

Formula: C46H78NO10P

Mol. weight: 836.103

Exact mass: 836.103

Ion type: [M-H]-

Precursor m/z: 834.5288

RT: 8.68651

Polarity: negative

InChI:

InChI key:

SMILES:

Instrument information

Instrument type: LC-ESI-QTOF

Instrument: AB Sciex TripleTOF 5600+ system

Ms level: MS2

Collision energy: 45V

Database links

Pubchem: 91746251

MoNA ID: LQB00726

Other information

Principal investigator: Kazutaka Ikeda, Hiroshi

Data collector: Kazutaka Ikeda, Hiroshi

PS 40:6

0.892

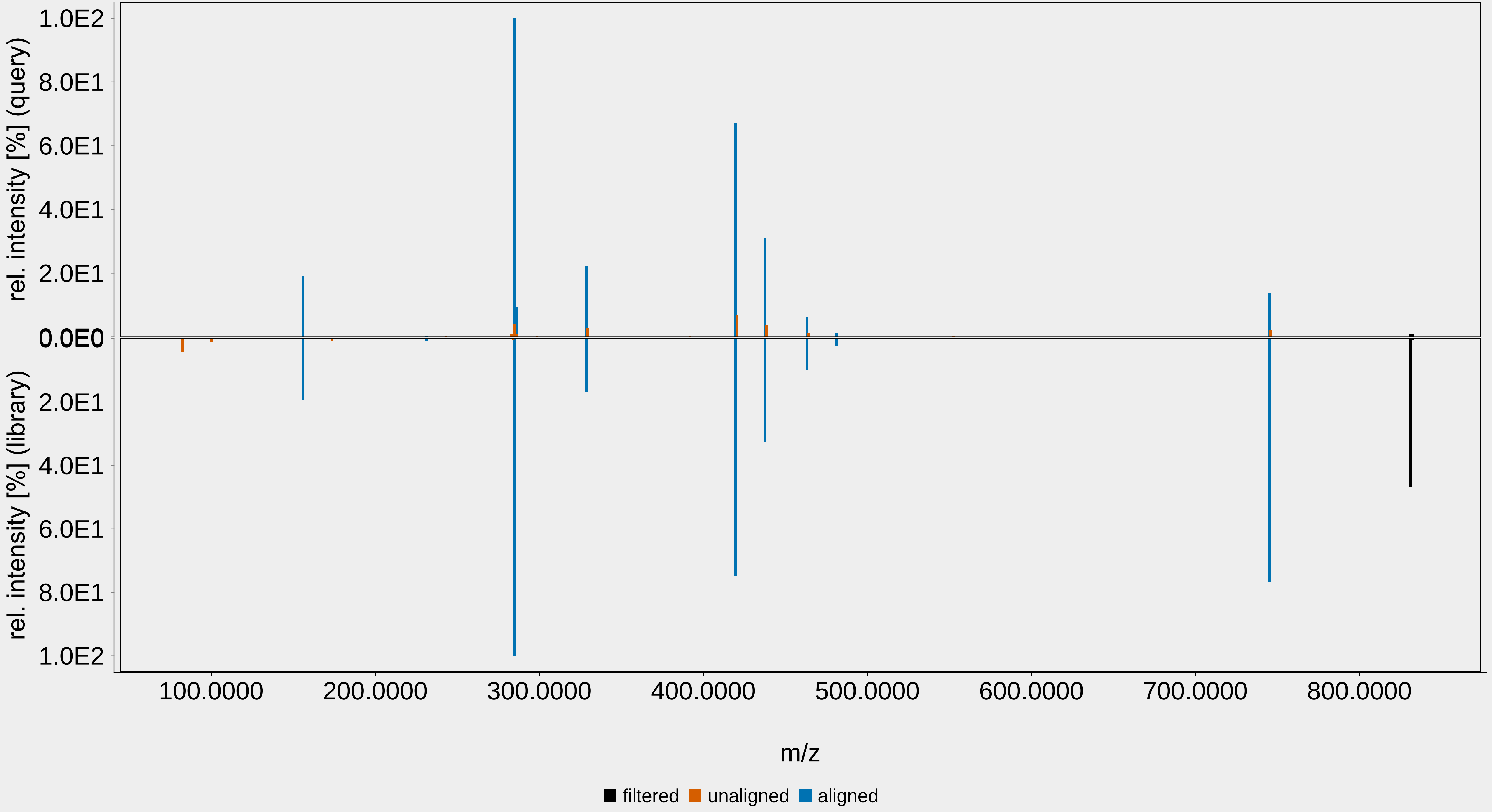

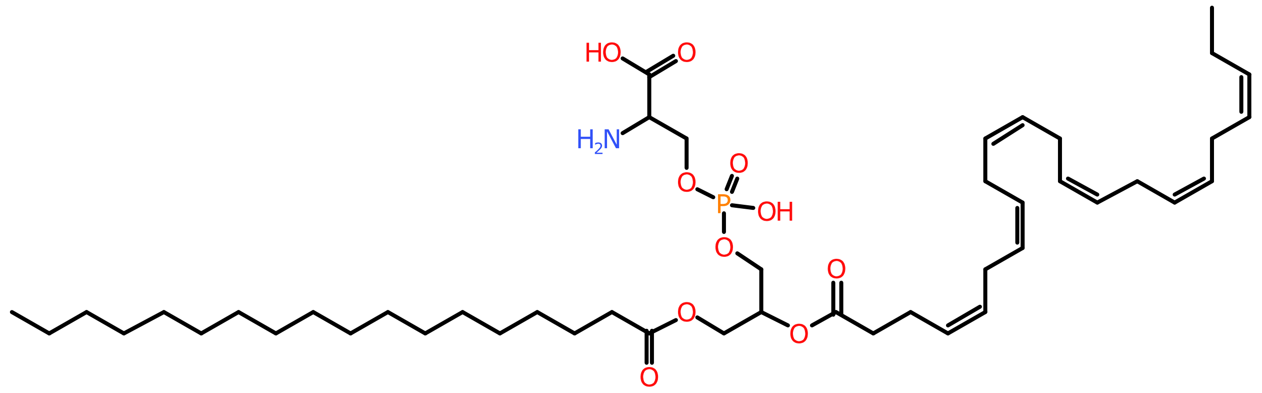

Compound information

Name: PS 40:6

Formula: C46H78NO10P

Mol. weight: 836.103

Exact mass: 836.103

Ion type: [M-H]-

Precursor m/z: 834.5288

RT: 8.68651

Polarity: negative

InChI:

InChI key:

SMILES:

Instrument information

Instrument type: LC-ESI-QTOF

Instrument: AB Sciex TripleTOF 5600+ system

Ms level: MS2

Collision energy: 45V

Database links

Pubchem: 91746251

MoNA ID: LQB00340

Other information

Principal investigator: Kazutaka Ikeda, Hiroshi

Data collector: Kazutaka Ikeda, Hiroshi

PS 18:1_20:4

0.755

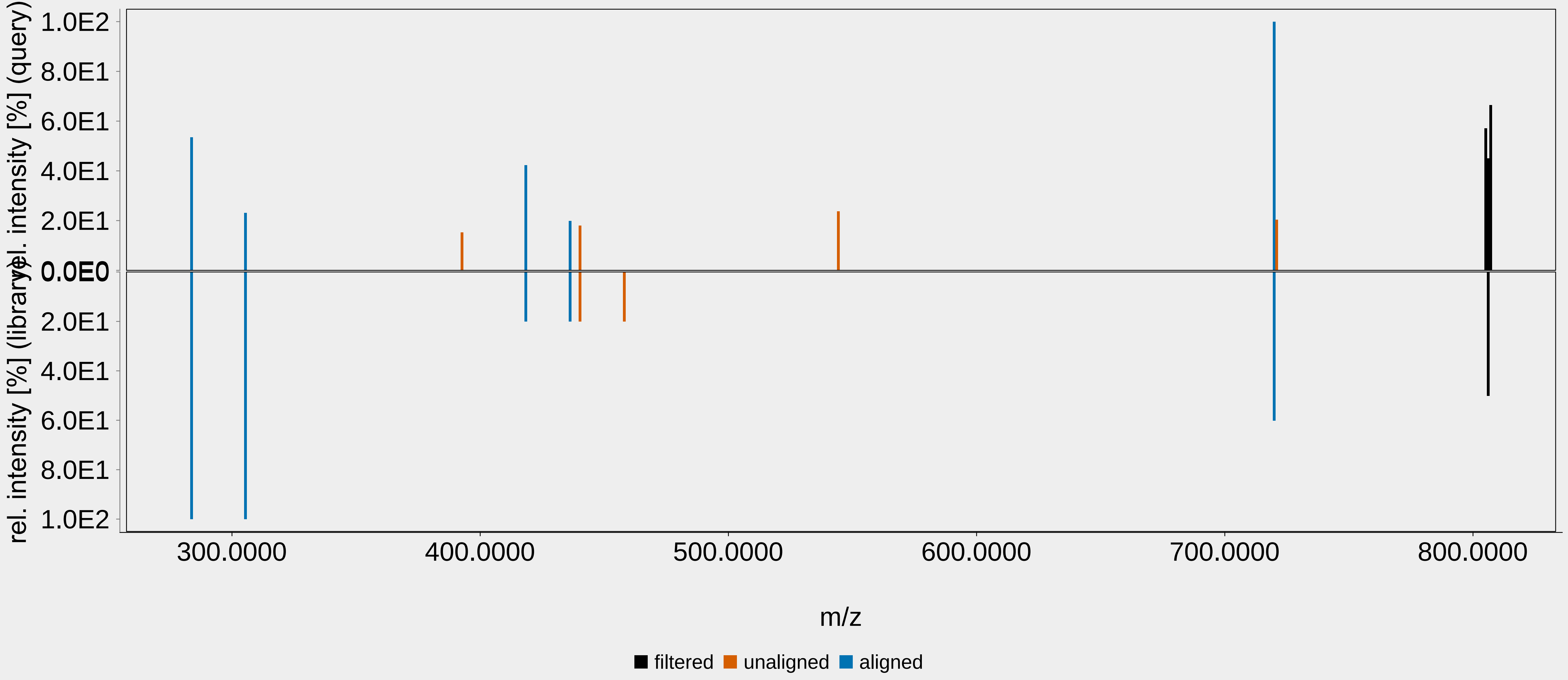

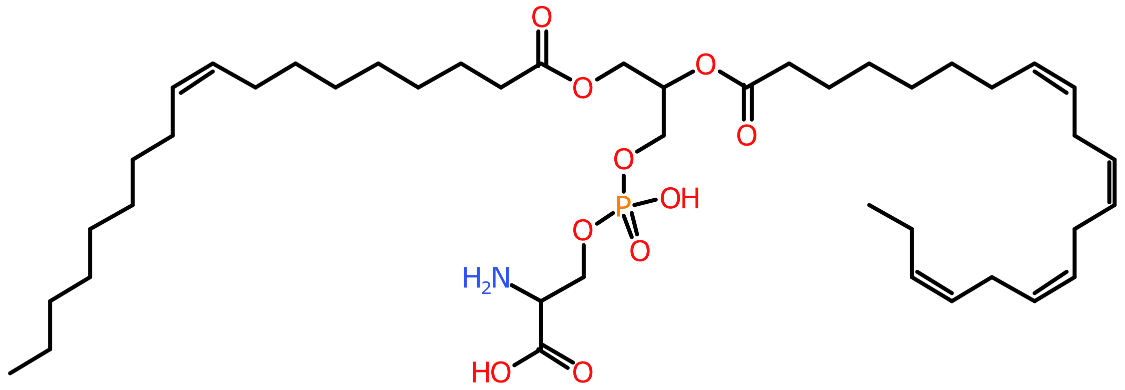

Compound information

Name: PS 18:1_20:4

Formula: C44H76NO10P

Ion type: [M-H]-

Precursor m/z: 808.51341

Ccs: 276.99

InChI key:

SMILES:

Num peaks: 8

Instrument information

Database links

Other information

PE(18:0/20:4); [M-H]- C43H77N1O8P1

0.903

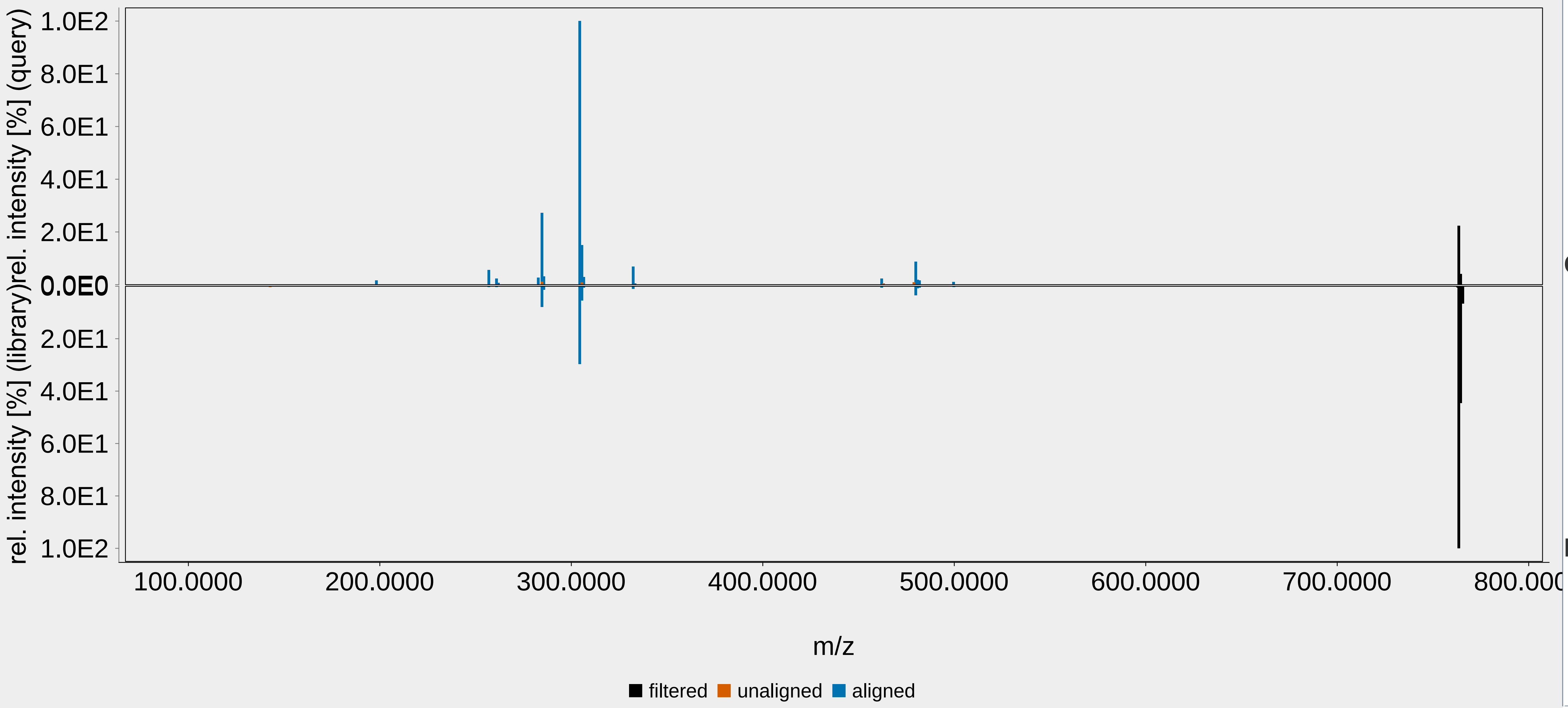

Compound information

Name: PE(18:0/20:4); [M-H]- C43H77N1O8P1

Exact mass: 0.0

Ion type: M-H

Precursor m/z: 766.539

Charge: 1

Polarity: Negative

Instrument information

Instrument type: HCD; Velos

Ion source: LC-ESI

Ms level: 2

Acquisition: Commercial

Database links

Other information

Principal investigator: Thomas Metz

Data collector: Thomas Metz

Entry ID: CCMSLIB00010074407

Spectral Match to 1-Octadecanoyl-2-(5Z,8Z,11Z,14Z-eicosatetraenoyl)-sn-glycero-3-phosphoethanolamine from NIST14

0.904

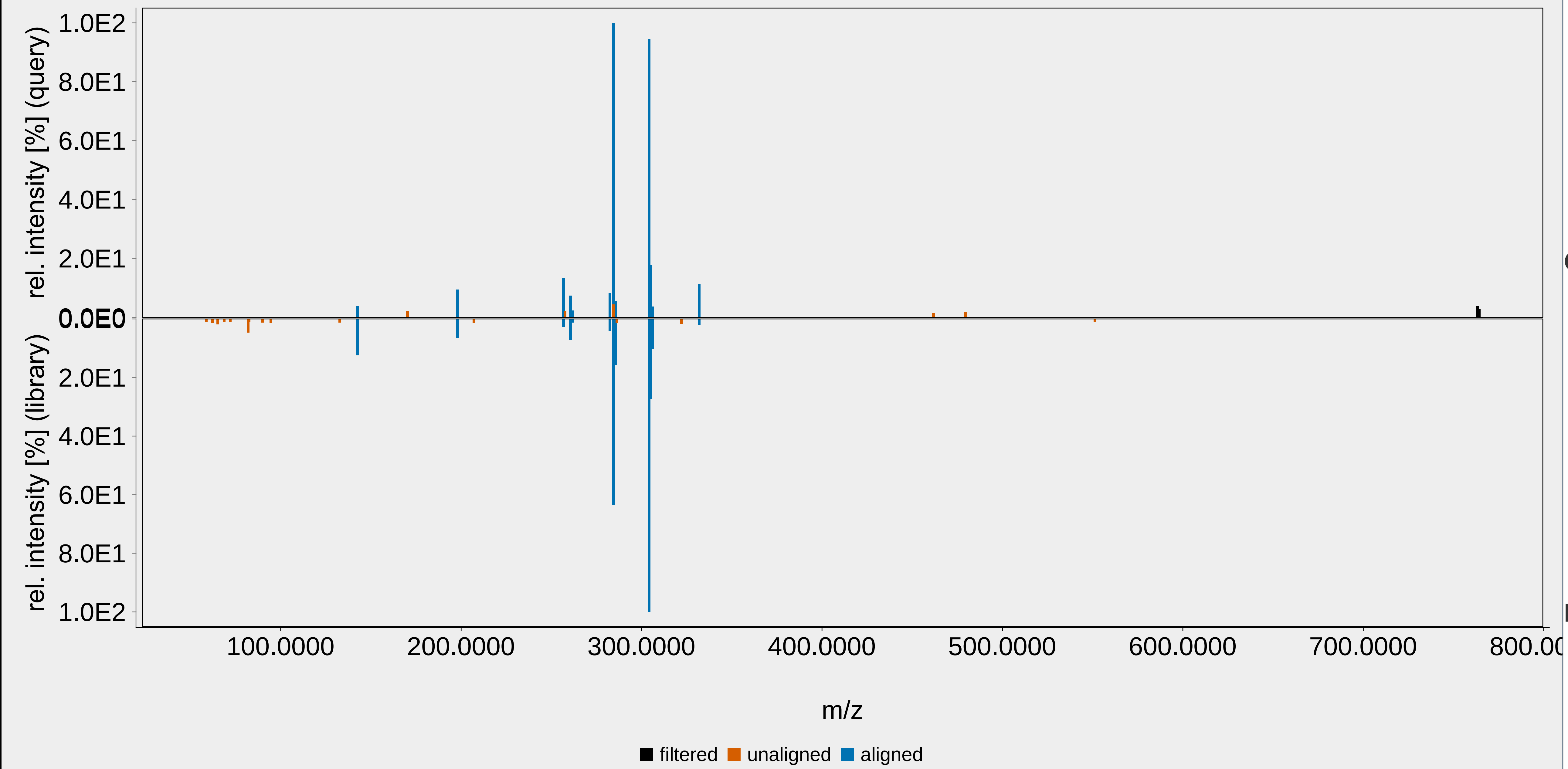

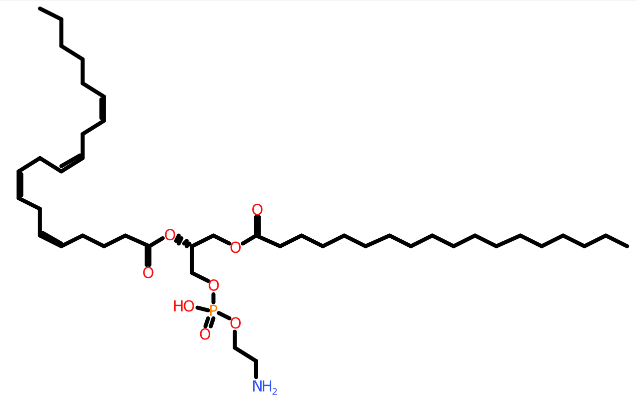

Compound information

Name: Spectral Match to

Exact mass: 767.547

Ion type: M-H

Precursor m/z: 766.541

Charge: 1

Polarity: Negative

InChI:

SMILES:

Instrument information

Instrument type: qTof

Ion source: ESI

Ms level: 2

Acquisition: Isolated

Database links

CAS: 61216624

Other information

Principal investigator: Data from Wiemann

Data collector: Data deposited by pwiemann

Entry ID: CCMSLIB00003139367

PE(16:1/18:1); [M-H]- C39H73N1O8P1

0.892

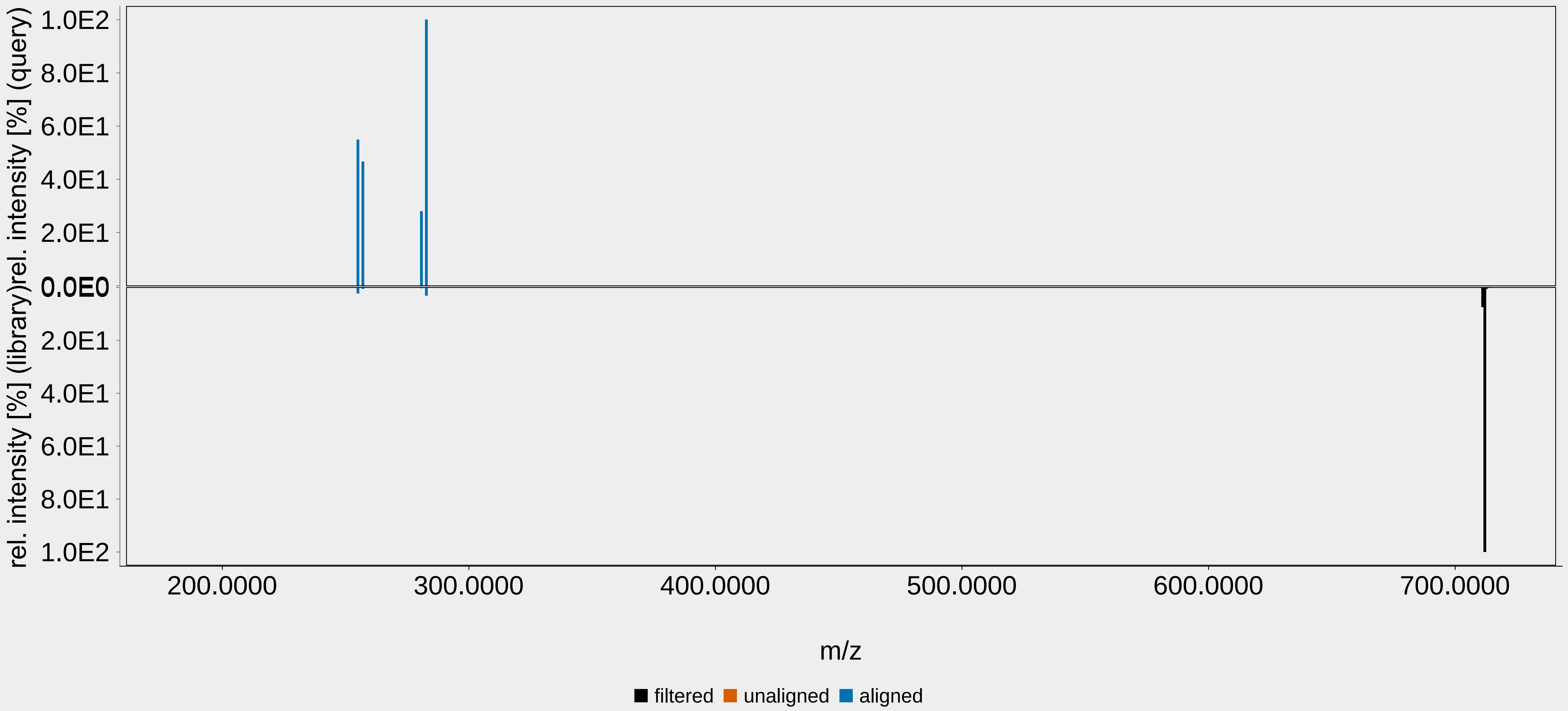

Compound information

Name: PE(16:1/18:1); [M-H]- C39H73N1O8P1

Exact mass: 0.0

Ion type: M-H

Precursor m/z: 714.507

Charge: 1

Polarity: Negative

Instrument information

Instrument type: HCD; Velos

Ion source: LC-ESI

Ms level: 2

Acquisition: Commercial

Database links

Other information

Principal investigator: Thomas Metz

Data collector: Thomas Metz

Entry ID: CCMSLIB00010060068

PE(16:1/18:1); [M-H]- C39H73N1O8P1 **0.902**

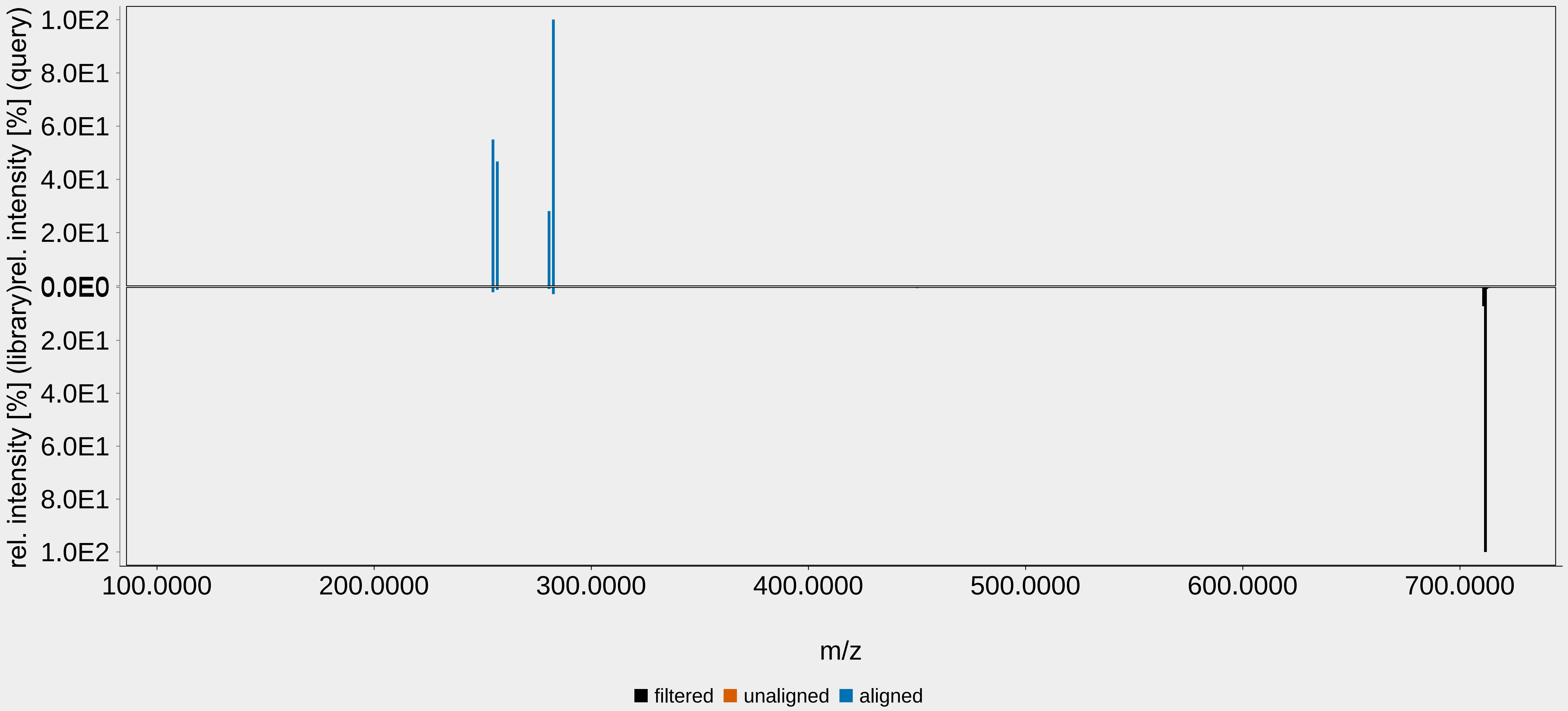

Compound information

Name: PE(16:1/18:1); [M-H]- C39H73N1O8P1

Exact mass: 0.0

Ion type: M-H

Precursor m/z: 714.507

Charge: 1

Polarity: Negative

Instrument information

Instrument type: HCD; Velos

Ion source: LC-ESI

Ms level: 2

Acquisition: Commercial

Database links

Other information

Principal investigator: Thomas Metz

Data collector: Thomas Metz

Entry ID: CCMSLIB00010060185

HYDROQUINIDINE

0.882

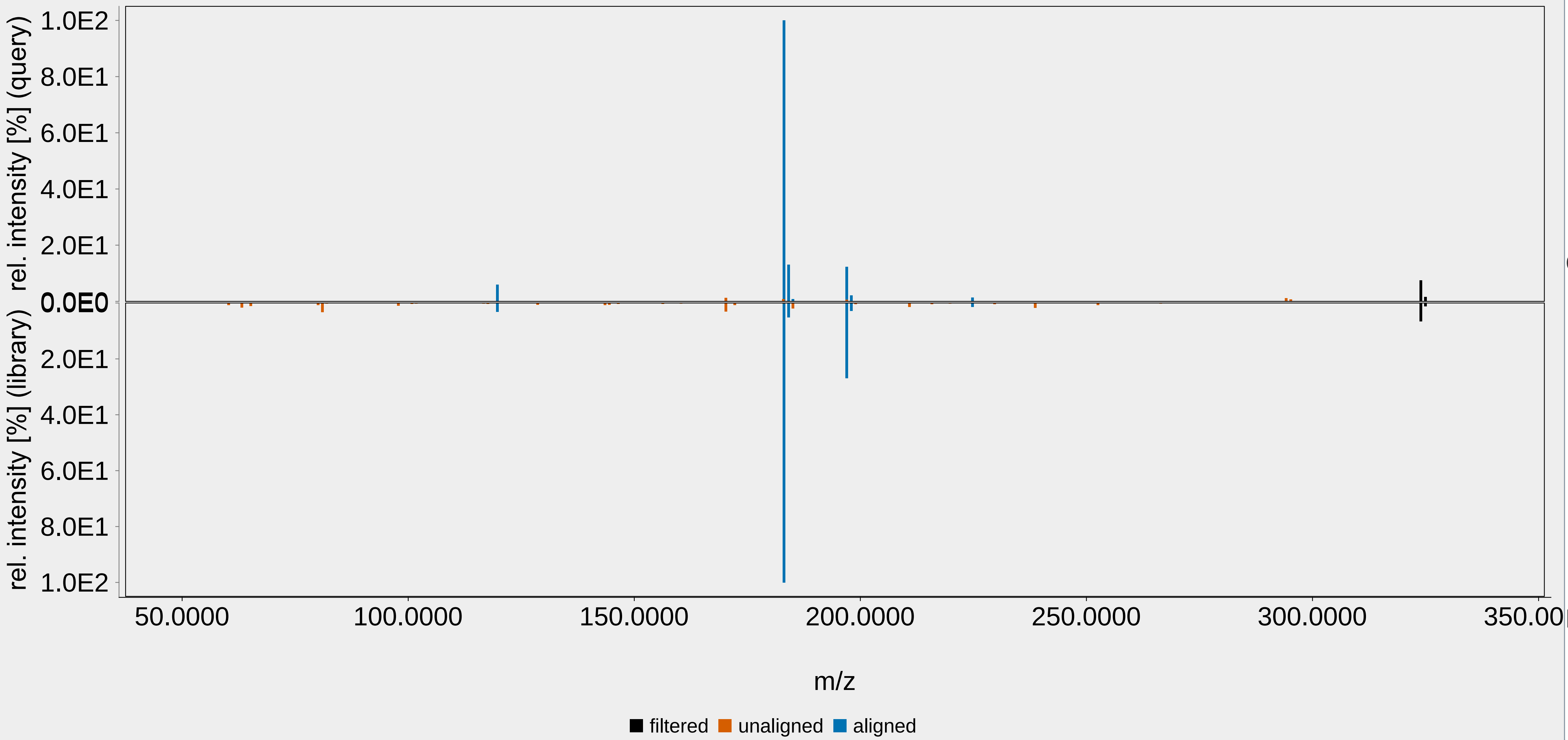

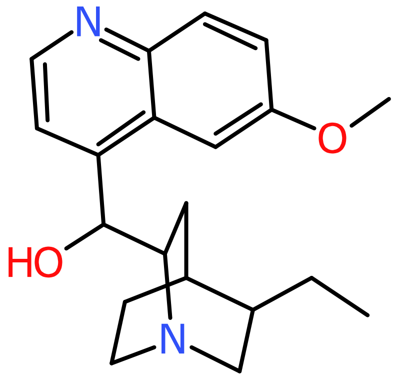

Compound information

Name: HYDROQUINIDINE

Formula: C20H26N2O2

Ion type: [M-H]-

Precursor m/z: 325.192138671875

Polarity: negative

InChI:

InChI key:

SMILES:

Instrument information

Instrument type: ESI-QFT

Instrument: Thermo Q Exactive HF

Ms level: MS2

Collision energy: 65HCD

Database links

Other information

Principal investigator: Arpana Vaniya

Data collector: Arpana Vaniya

HYDROQUINIDINE

0.882

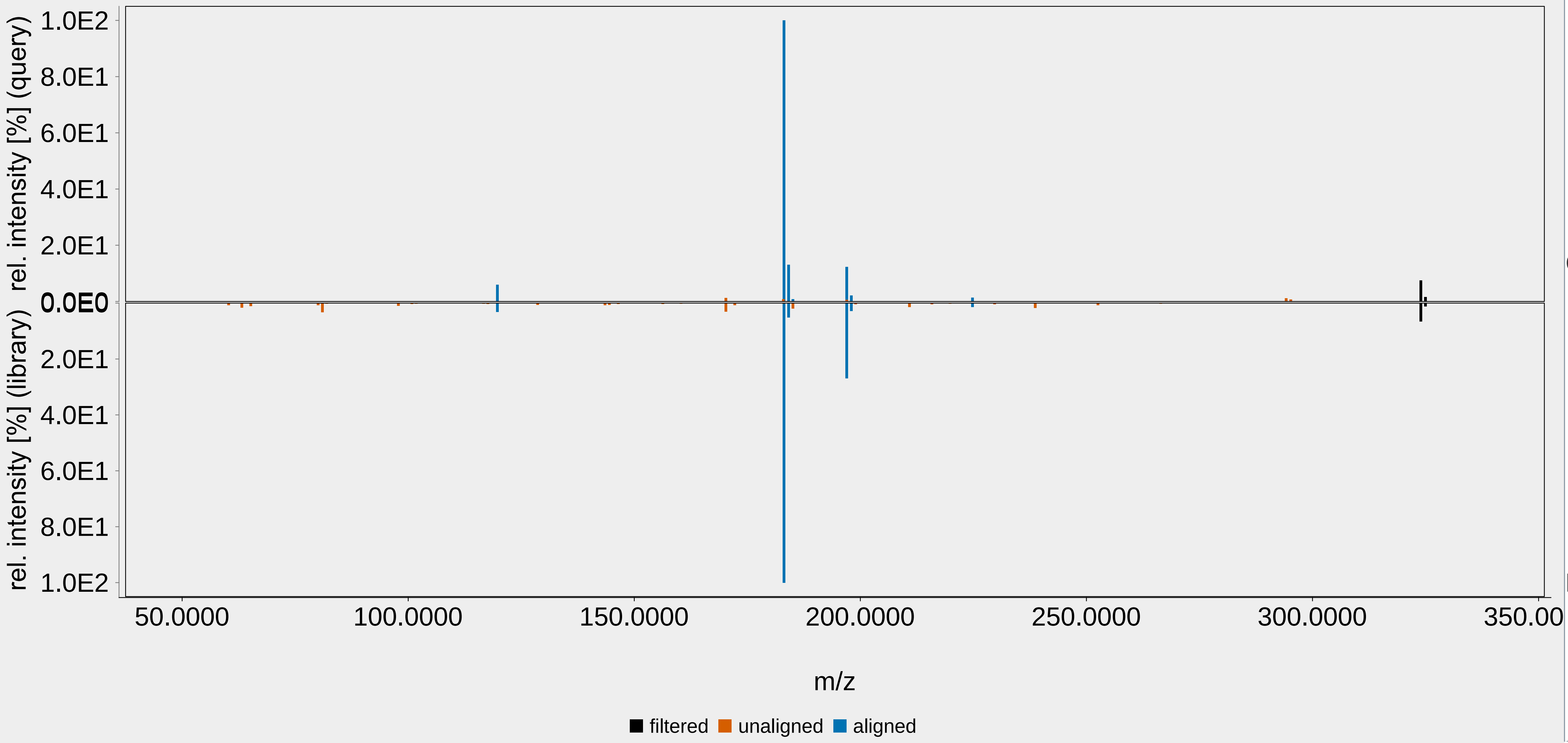

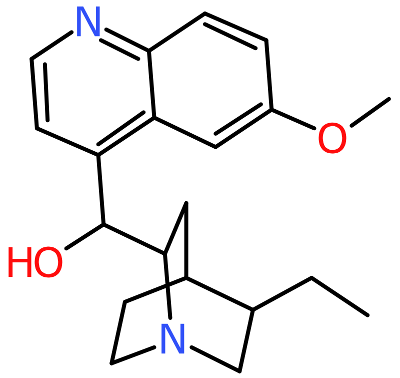

Compound information

Name: HYDROQUINIDINE

Exact mass: 0.0

Ion type: [M-H]-

Precursor m/z: 325.192

Charge: 1

Polarity: negative

InChI:

Instrument information

Instrument type: ESI-QFT

Ms level: 2

Acquisition: isolated

Database links

Other information

Principal investigator: MoNA

Data collector: MoNA:VF-NPL-QEHF003075

Entry ID: CCMSLIB00004694138

Crocetin semialdehyde

0.746

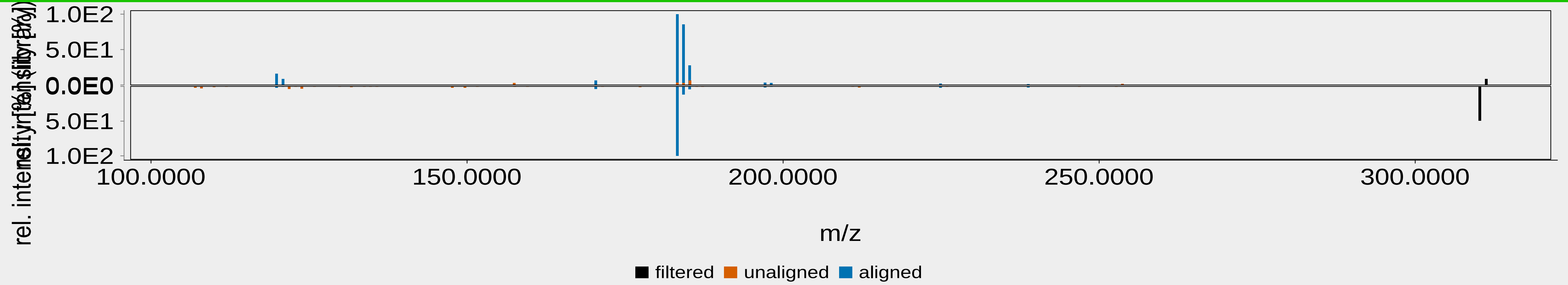

Compound information

Name: Crocetin semialdehyde

Ion type: carotenoid

Precursor m/z: 312.1725

Polarity: negative

Instrument information

Ion source: Electrospray Ionization (ESI)

Ms level: MS2

Database links

Other information

PA 24:0_18:1 **0.864**

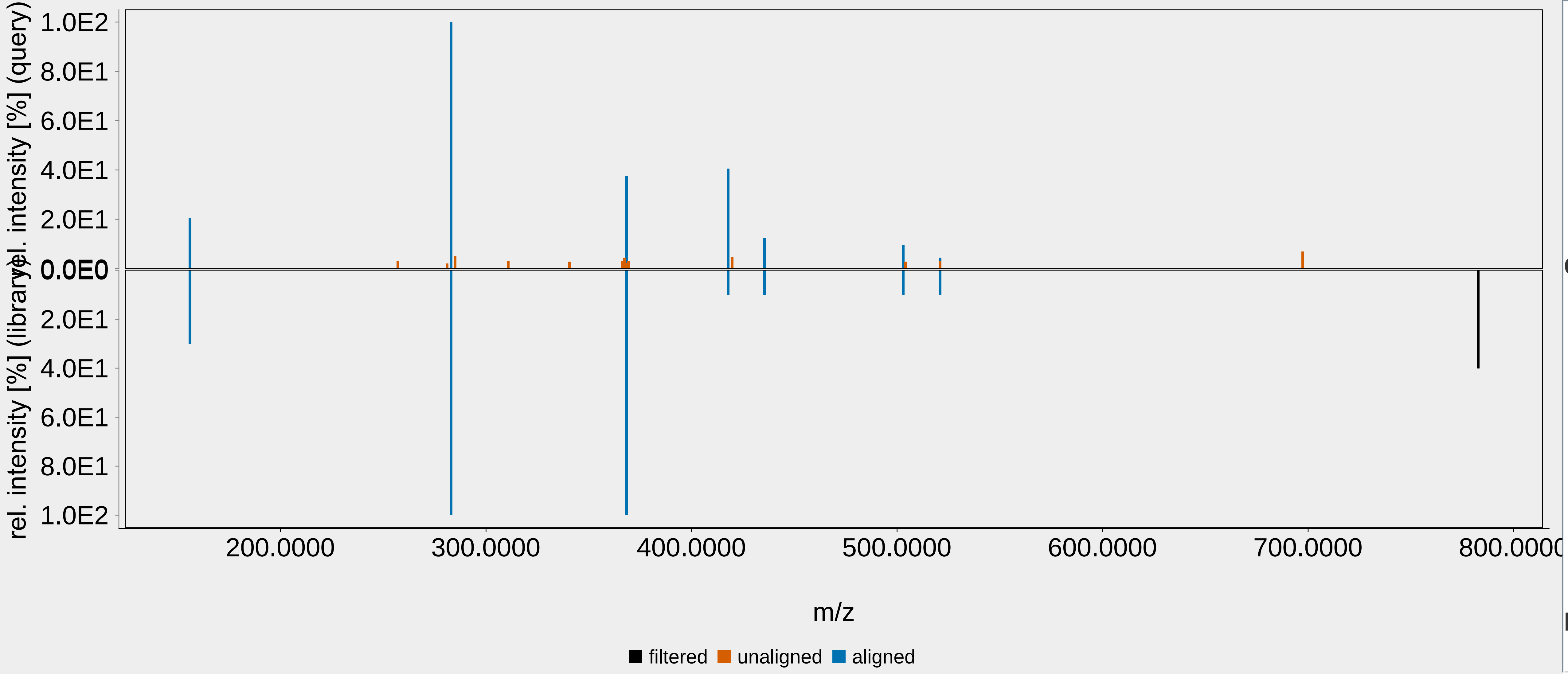

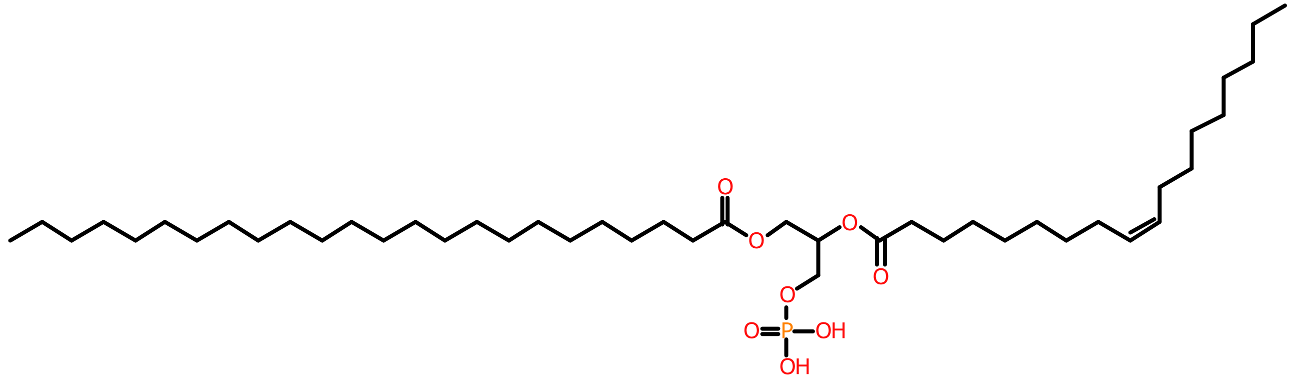

Compound information

Name: PA 24:0_18:1

Formula: C45H87O8P

Ion type: [M-H]-

Precursor m/z: 785.60658

Ccs: 278.64

InChI key:

SMILES:

Num peaks: 8

Instrument information

Database links

Other information

PS 36:1 **0.744**

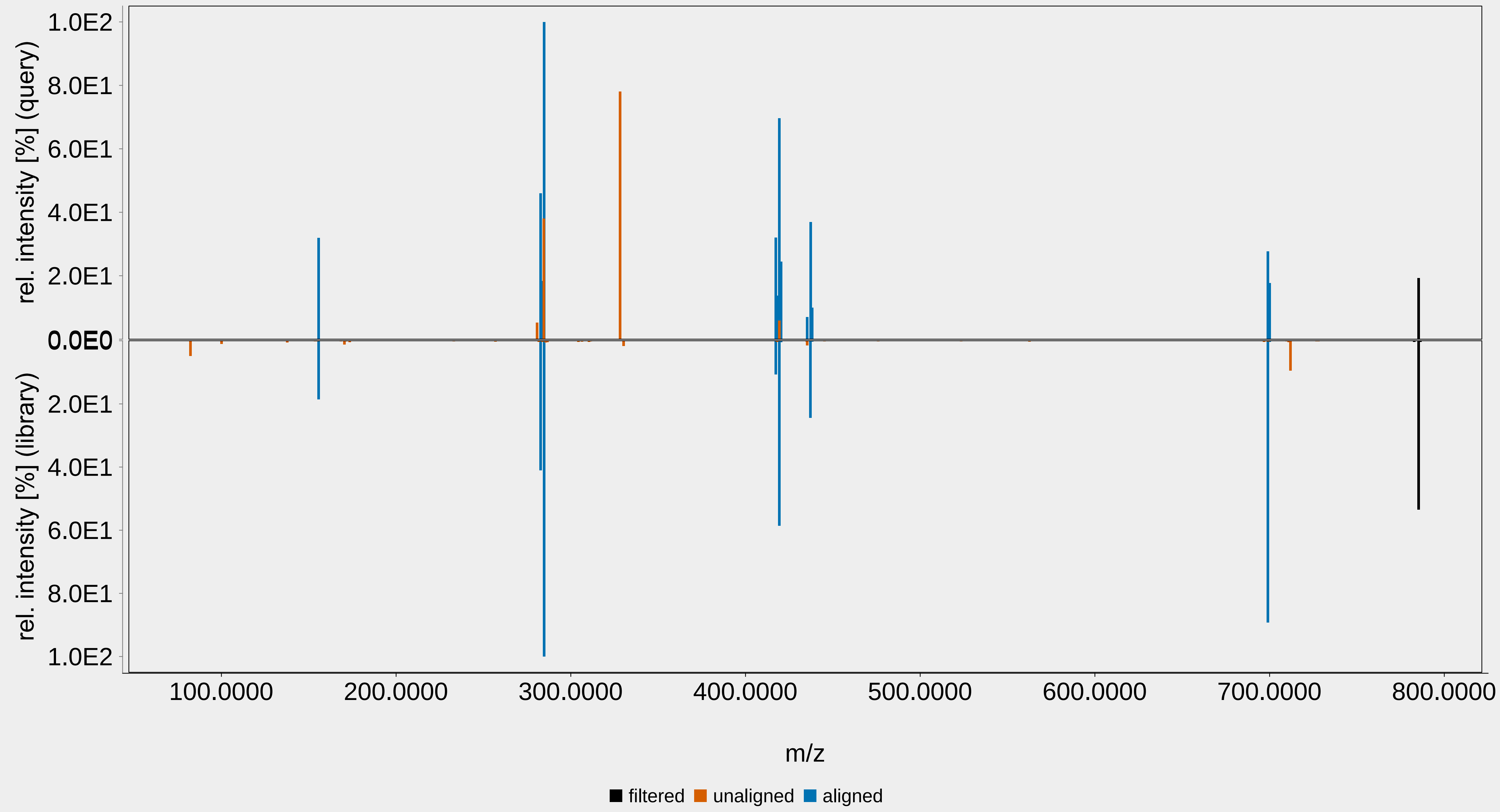

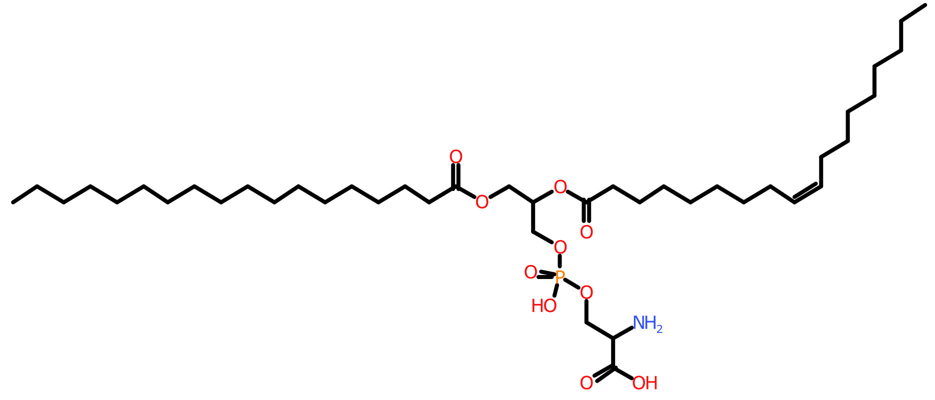

Compound information

Name: PS 36:1

Formula: C42H80NO10P

Mol. weight: 790.0749999999999

Exact mass: 790.0749999999999

Ion type: [M-H]-

Precursor m/z: 788.5428

RT: 9.185796

Polarity: negative

InChI:

InChI key: AJFWREUFUPEYII-ZZEZOPTASA-N

SMILES:

Instrument information

Instrument type: LC-ESI-QTOF

Instrument: AB Sciex TripleTOF 5600+ system

Ms level: MS2

Collision energy: 45V

Database links

Pubchem: 9962404

MoNA ID: LQB00719

Other information

Principal investigator: Kazutaka Ikeda, Hiroshi

Data collector: Kazutaka Ikeda, Hiroshi

PS 36:1 **0.744**

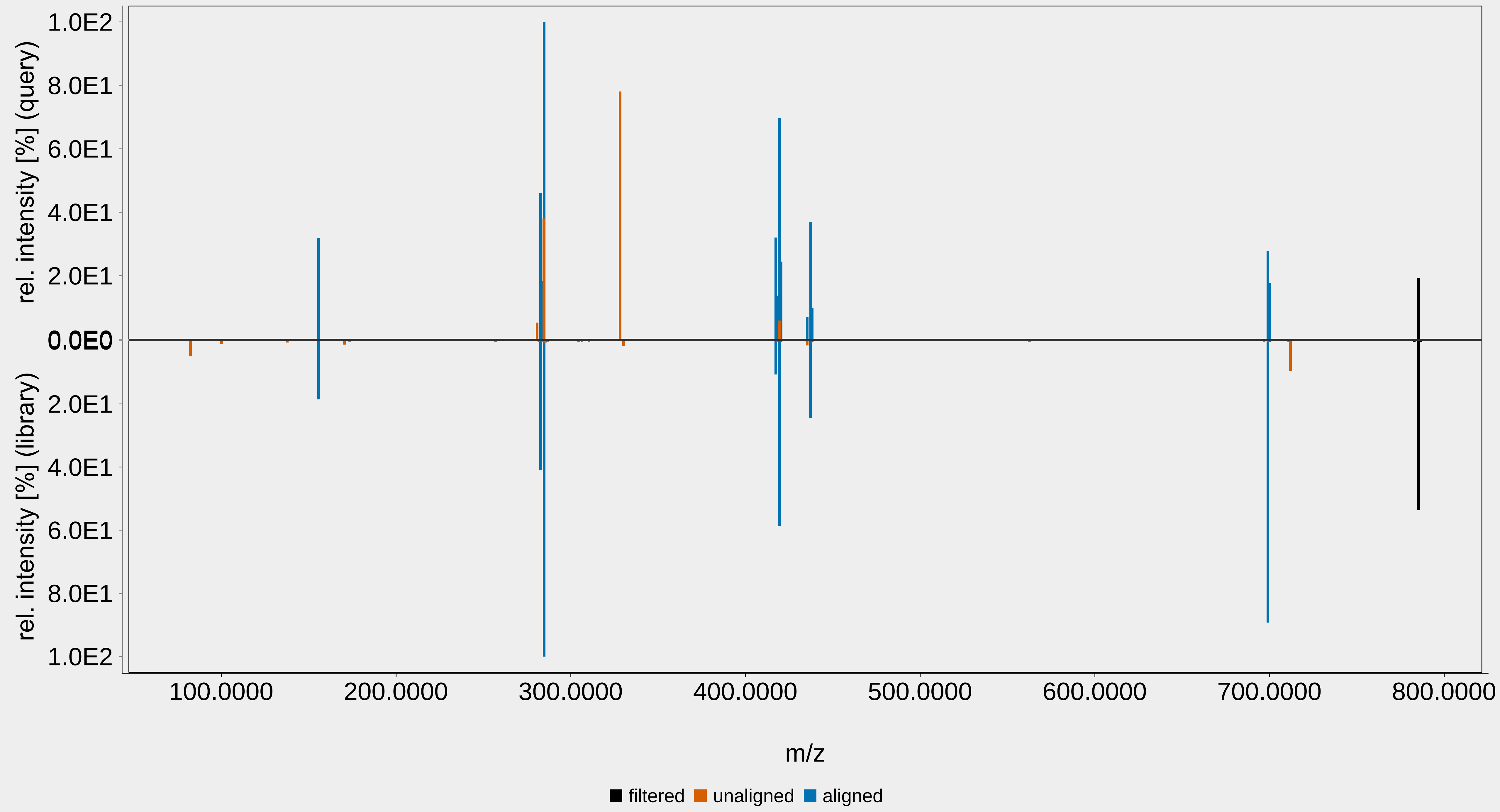

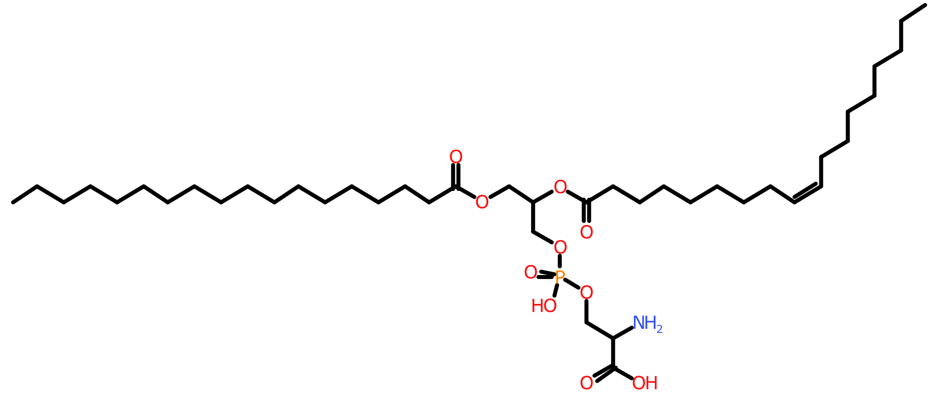

Compound information

Name: PS 36:1

Formula: C42H80NO10P

Mol. weight: 790.0749999999999

Exact mass: 790.0749999999999

Ion type: [M-H]-

Precursor m/z: 788.5428

RT: 9.185796

Polarity: negative

InChI:

InChI key: AJFWREUFUPEYII-ZZEZOPTASA-N

SMILES:

Instrument information

Instrument type: LC-ESI-QTOF

Instrument: AB Sciex TripleTOF 5600+ system

Ms level: MS2

Collision energy: 45V

Database links

Pubchem: 9962404

MoNA ID: LQB00333

Other information

Principal investigator: Kazutaka Ikeda, Hiroshi

Data collector: Kazutaka Ikeda, Hiroshi

PA 17:0_18:1

0.739

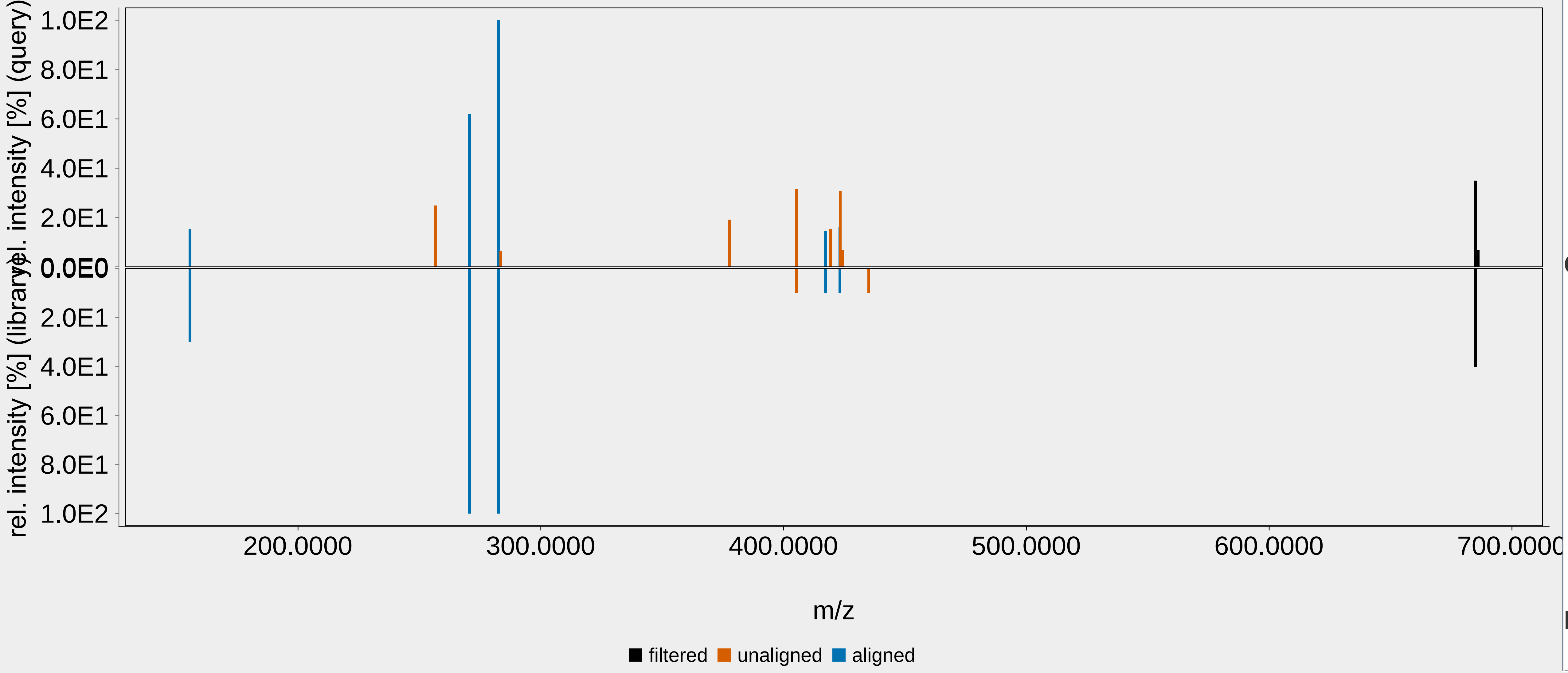

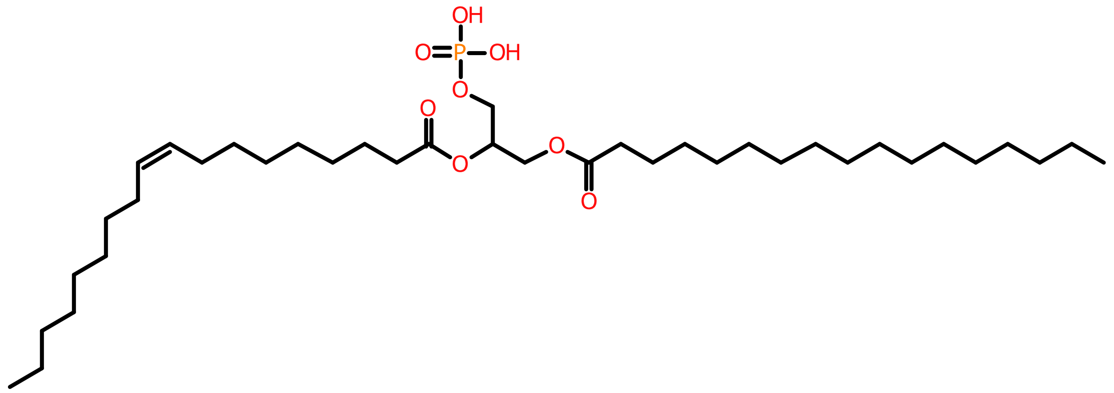

Compound information

Name: PA 17:0_18:1

Formula: C38H73O8P

Ion type: [M-H]-

Precursor m/z: 687.49703

Ccs: 257.18

InChI key:

SMILES:

Num peaks: 8

Instrument information

Database links

Other information

[3-[[2,3-dihydroxypropoxy]-hydroxyphosphoryl]oxy-2-hexadecanoyloxypropyl] hexadecanoate

0.856

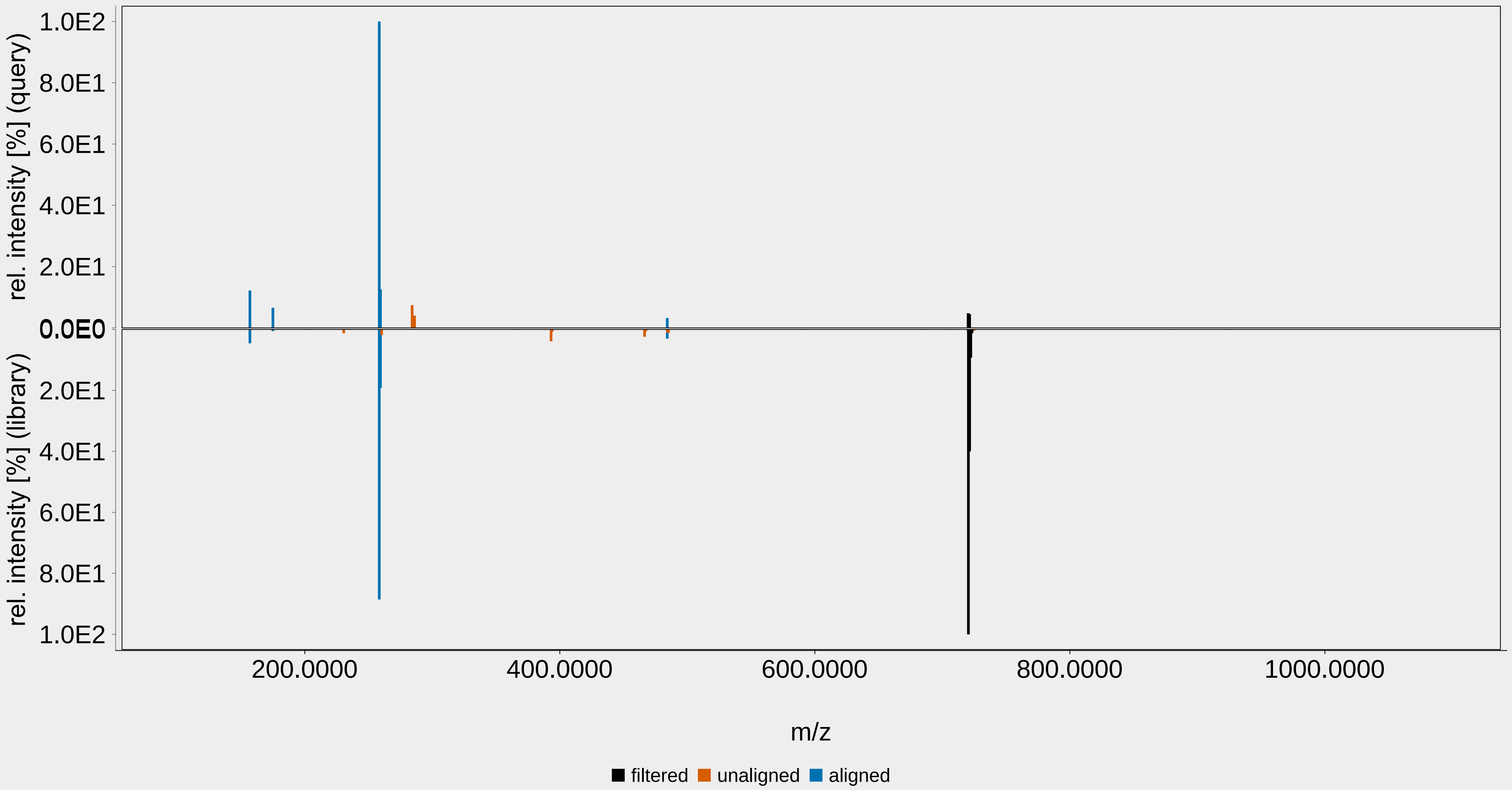

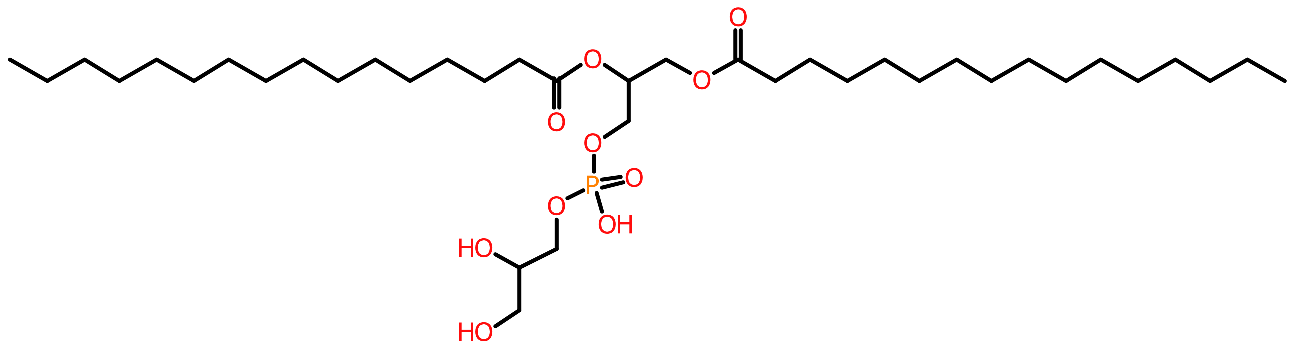

Compound information

Name:

Formula: C38H75O10P

Mol. weight: 722.51

Exact mass: 722.51

Ion type: [M-H]-

Precursor m/z: 721.502

Polarity: negative

InChI:

InChI key:

SMILES:

Instrument information

Instrument: qTof

Ms level: MS2

Database links

Other information

Principal investigator: O Laprevote, UMR 8038

Data collector: O Laprevote, UMR 8038

PG(16:0_16:0) - [3-[[2,3-dihydroxypropoxy]-hydroxyphosphoryl]oxy-2-hexadecanoyloxypropyl] hexadecanoate

0.856

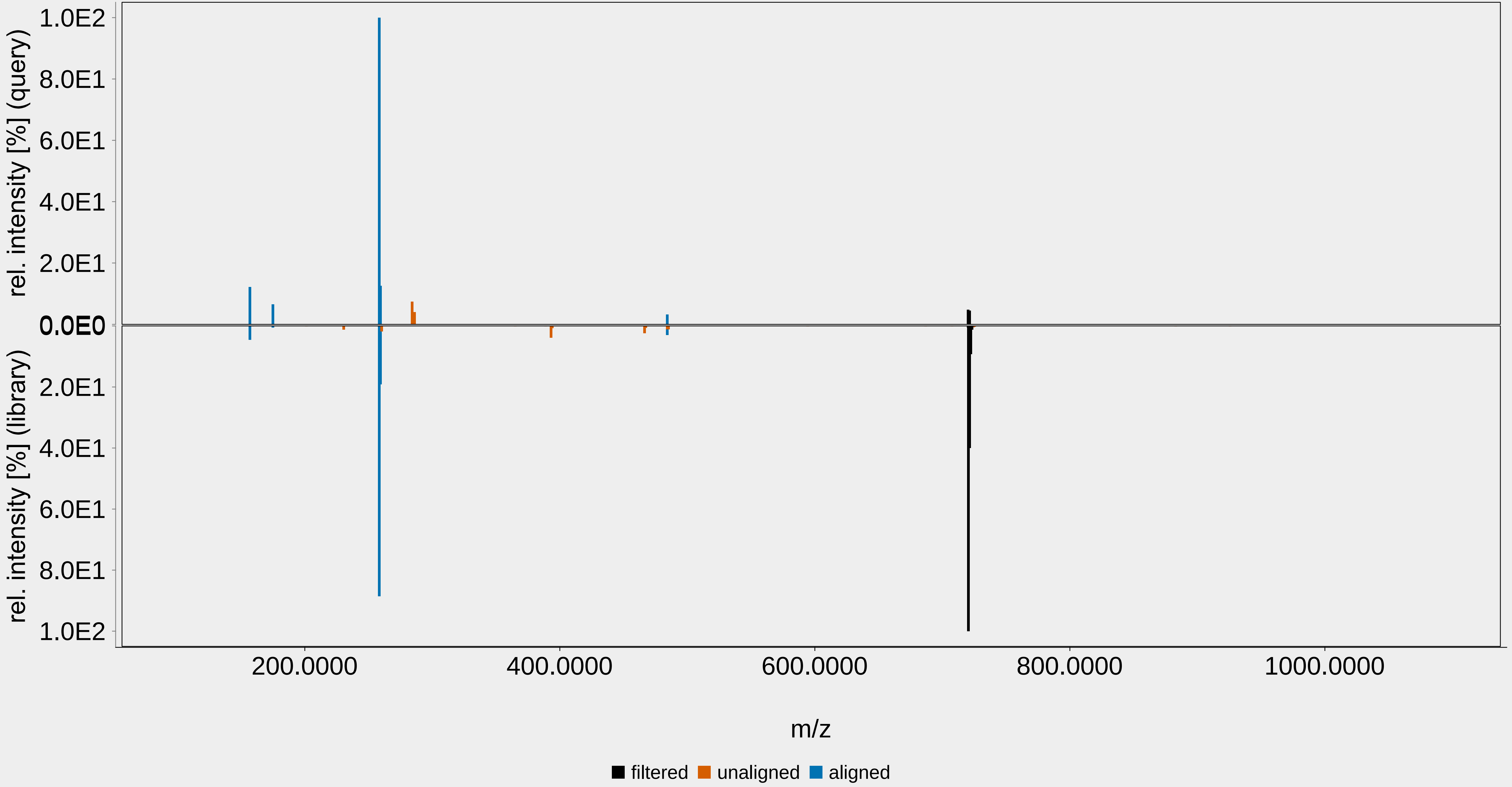

Compound information

Name: PG(16:0_16:0) -

Exact mass: 722.51

Ion type: M-H

Precursor m/z: 721.502

Charge: 1

Polarity: Negative

InChI:

InChI key:

SMILES:

Instrument information

Instrument type: qTof

Ion source: LC-ESI

Ms level: 2

Acquisition: Commercial

Database links

Pubmed:

CAS: 200880-41-7

Other information

Principal investigator: O Laprevote

Data collector: UMR 8038 Universite de Paris

Entry ID: CCMSLIB00005436447

PE(20:3/0:0); [M-H]- C25H45N1O7P1 **0.853**

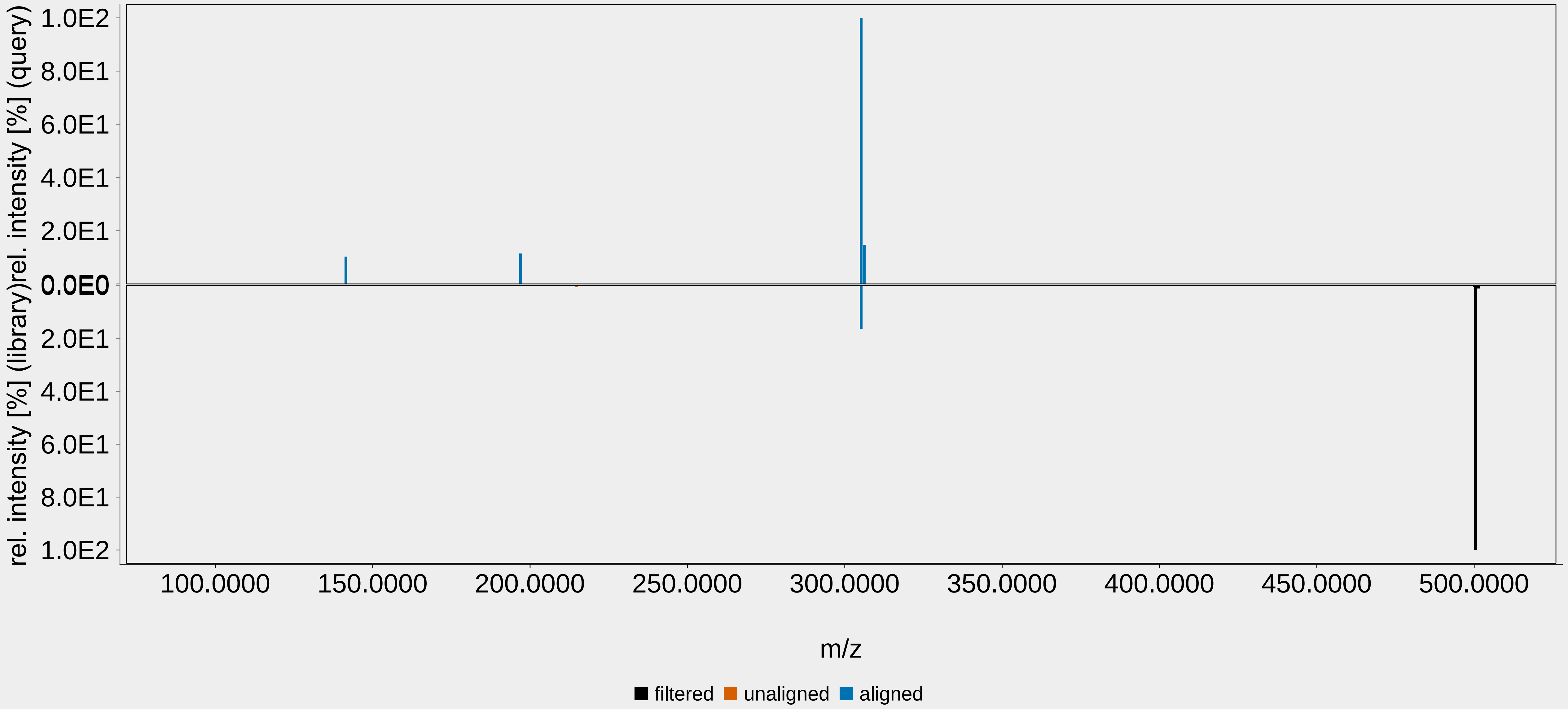

Compound information

Name: PE(20:3/0:0); [M-H]- C25H45N1O7P1

Exact mass: 0.0

Ion type: M-H

Precursor m/z: 502.293

Charge: 1

Polarity: Negative

Instrument information

Instrument type: HCD; Velos

Ion source: LC-ESI

Ms level: 2

Acquisition: Commercial

Database links

Other information

Principal investigator: Thomas Metz

Data collector: Thomas Metz

Entry ID: CCMSLIB00010080527

PE(20:3/0:0); [M-H]- C25H45N1O7P1 **0.868**

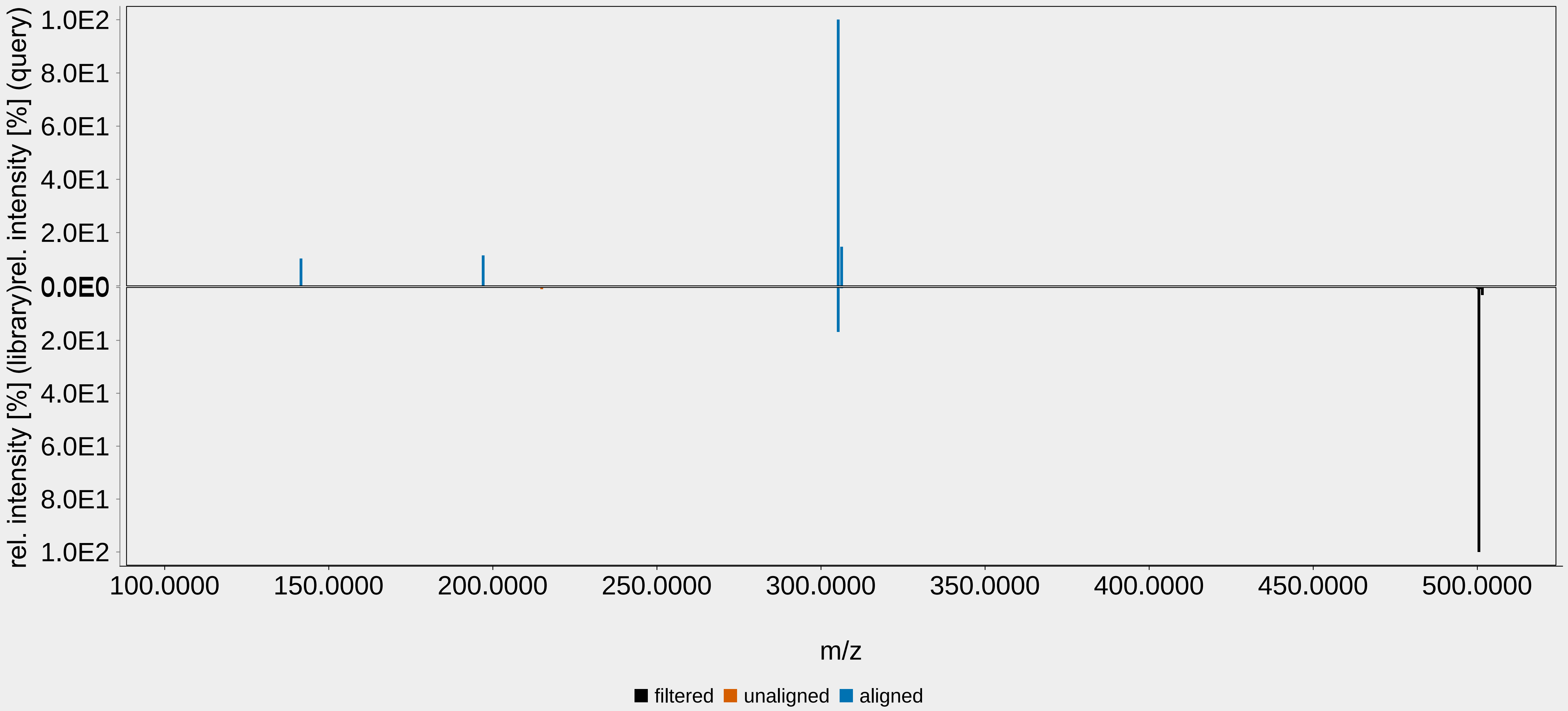

Compound information

Name: PE(20:3/0:0); [M-H]- C25H45N1O7P1

Exact mass: 0.0

Ion type: M-H

Precursor m/z: 502.293

Charge: 1

Polarity: Negative

Instrument information

Instrument type: HCD; Velos

Ion source: LC-ESI

Ms level: 2

Acquisition: Commercial

Database links

Other information

Principal investigator: Thomas Metz

Data collector: Thomas Metz

Entry ID: CCMSLIB00010079788

PE(18:1/18:2); [M-H]- C41H75N1O8P1 0.836

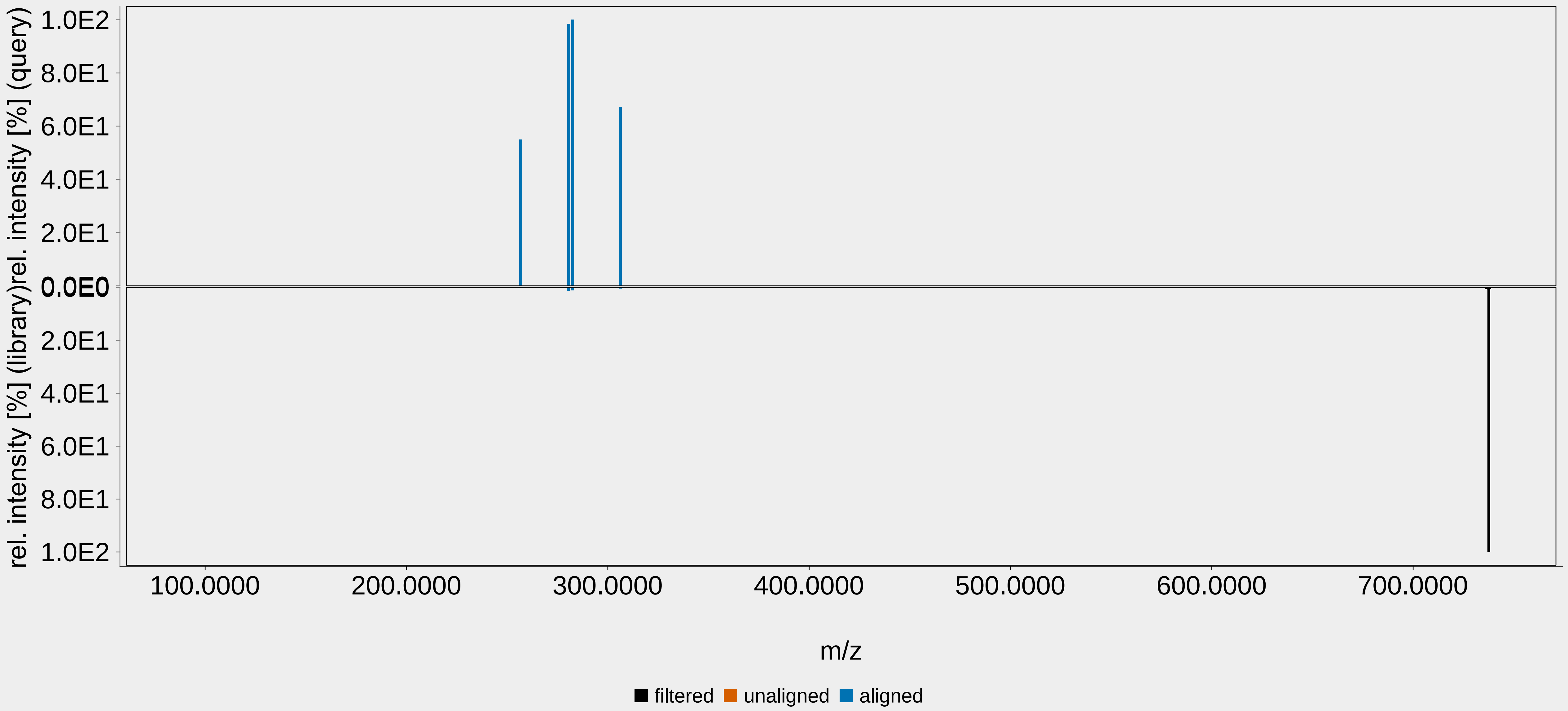

Compound information

Name: PE(18:1/18:2); [M-H]- C41H75N1O8P1

Exact mass: 0.0

Ion type: M-H

Precursor m/z: 740.523

Charge: 1

Polarity: Negative

Instrument information

Instrument type: HCD; Velos

Ion source: LC-ESI

Ms level: 2

Acquisition: Commercial

Database links

Other information

Principal investigator: Thomas Metz

Data collector: Thomas Metz

Entry ID: CCMSLIB00010076756

PE(18:1/18:2); [M-H]- C41H75N1O8P1

0.880

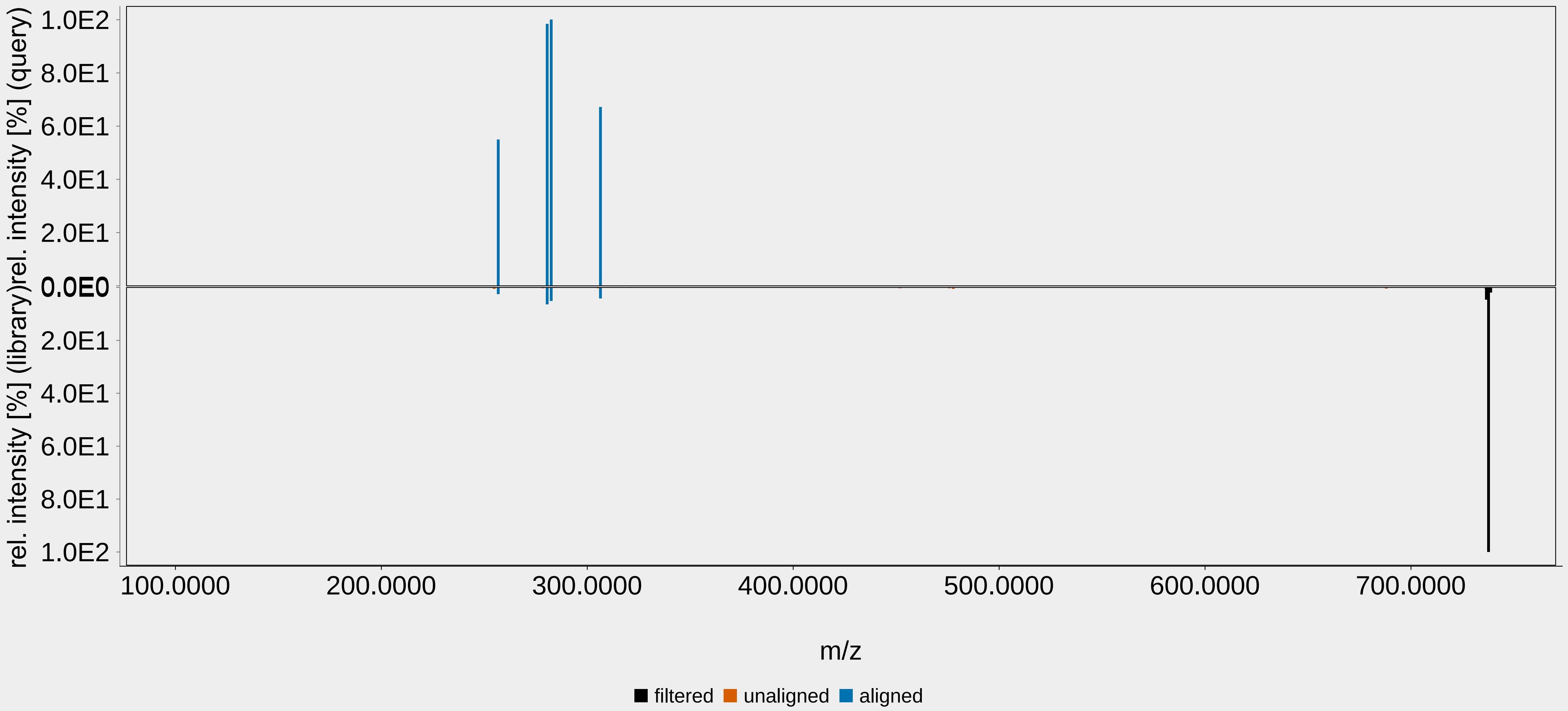

Compound information

Name: PE(18:1/18:2); [M-H]- C41H75N1O8P1

Exact mass: 0.0

Ion type: M-H

Precursor m/z: 740.523

Charge: 1

Polarity: Negative

Instrument information

Instrument type: HCD; Velos

Ion source: LC-ESI

Ms level: 2

Acquisition: Commercial

Database links

Other information

Principal investigator: Thomas Metz

Data collector: Thomas Metz

Entry ID: CCMSLIB00010071000

PS 18:0_20:3

0.753

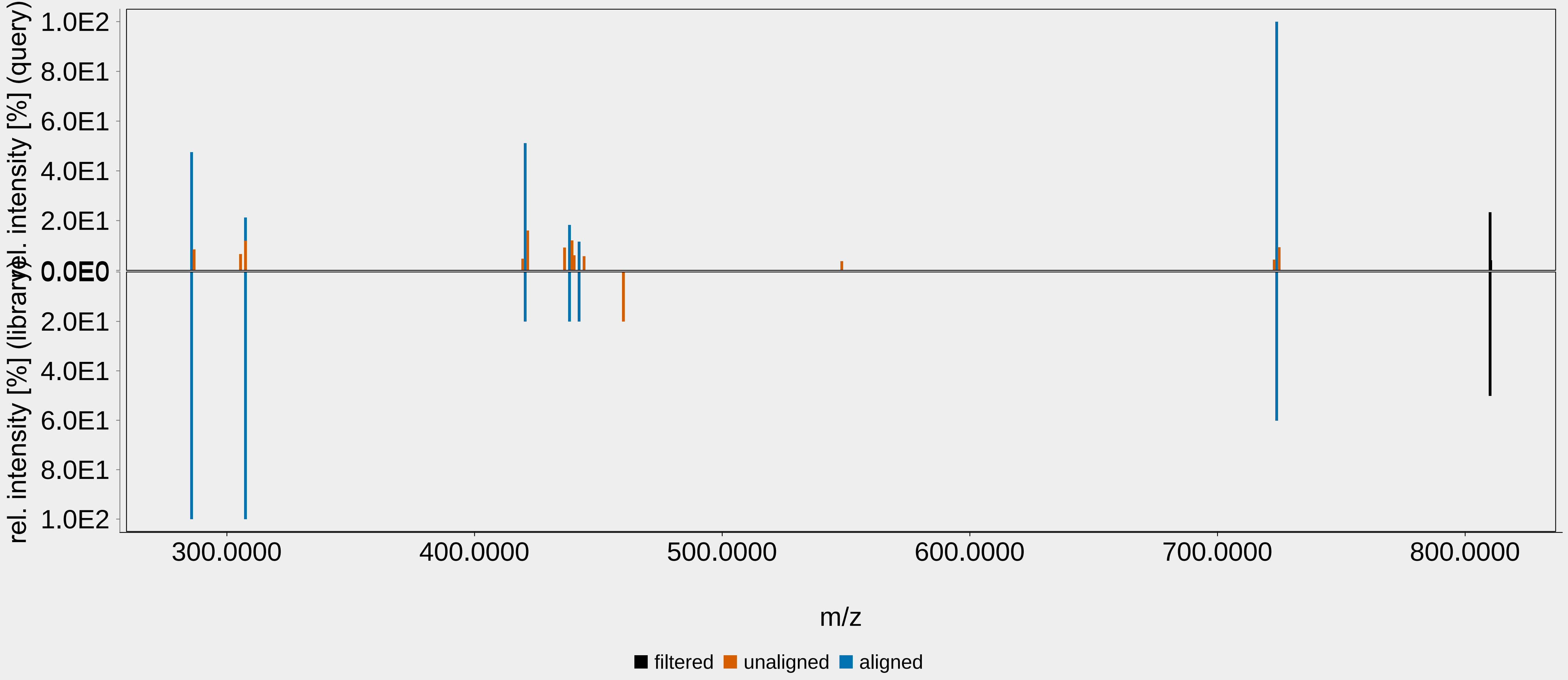

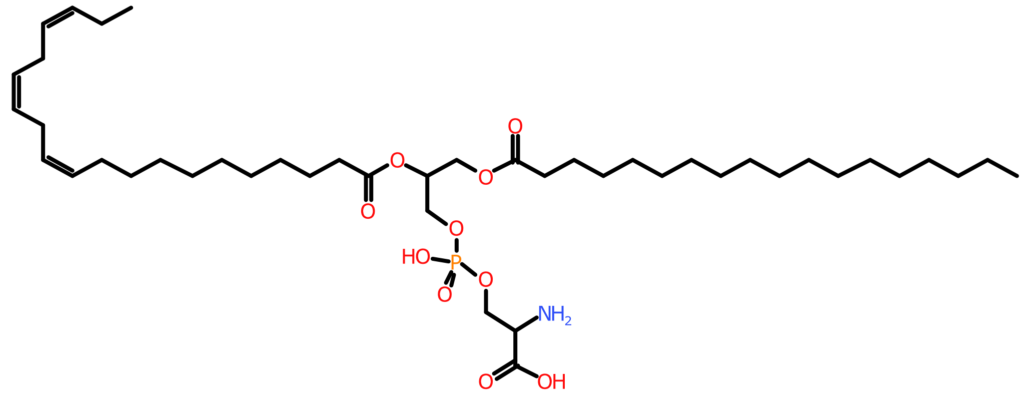

Compound information

Name: PS 18:0_20:3

Formula: C44H80NO10P

Ion type: [M-H]-

Precursor m/z: 812.54471

Ccs: 279.88

InChI key:

SMILES:

Num peaks: 8

Instrument information

Database links

Other information

PS(18:0/20:3); [M-H]- C44H79N1O10P1 **0.863**

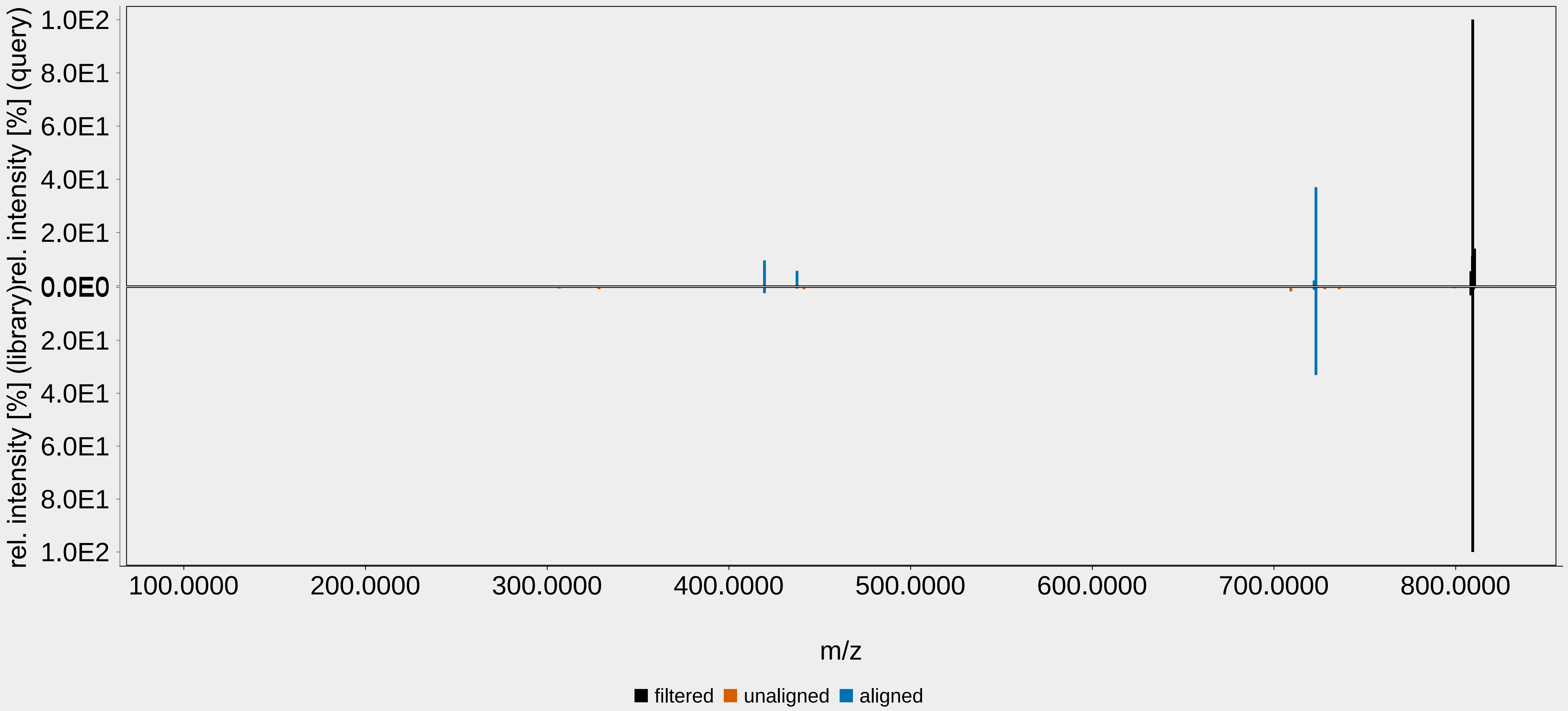

Compound information

Name: PS(18:0/20:3); [M-H]- C44H79N1O10P1

Exact mass: 0.0

Ion type: M-H

Precursor m/z: 812.544

Charge: 1

Polarity: Negative

Instrument information

Instrument type: HCD; Velos

Ion source: LC-ESI

Ms level: 2

Acquisition: Commercial

Database links

Other information

Principal investigator: Thomas Metz

Data collector: Thomas Metz

Entry ID: CCMSLIB00010077483

PE(20:2/0:0); [M-H]- C25H47N1O7P1 **0.925**

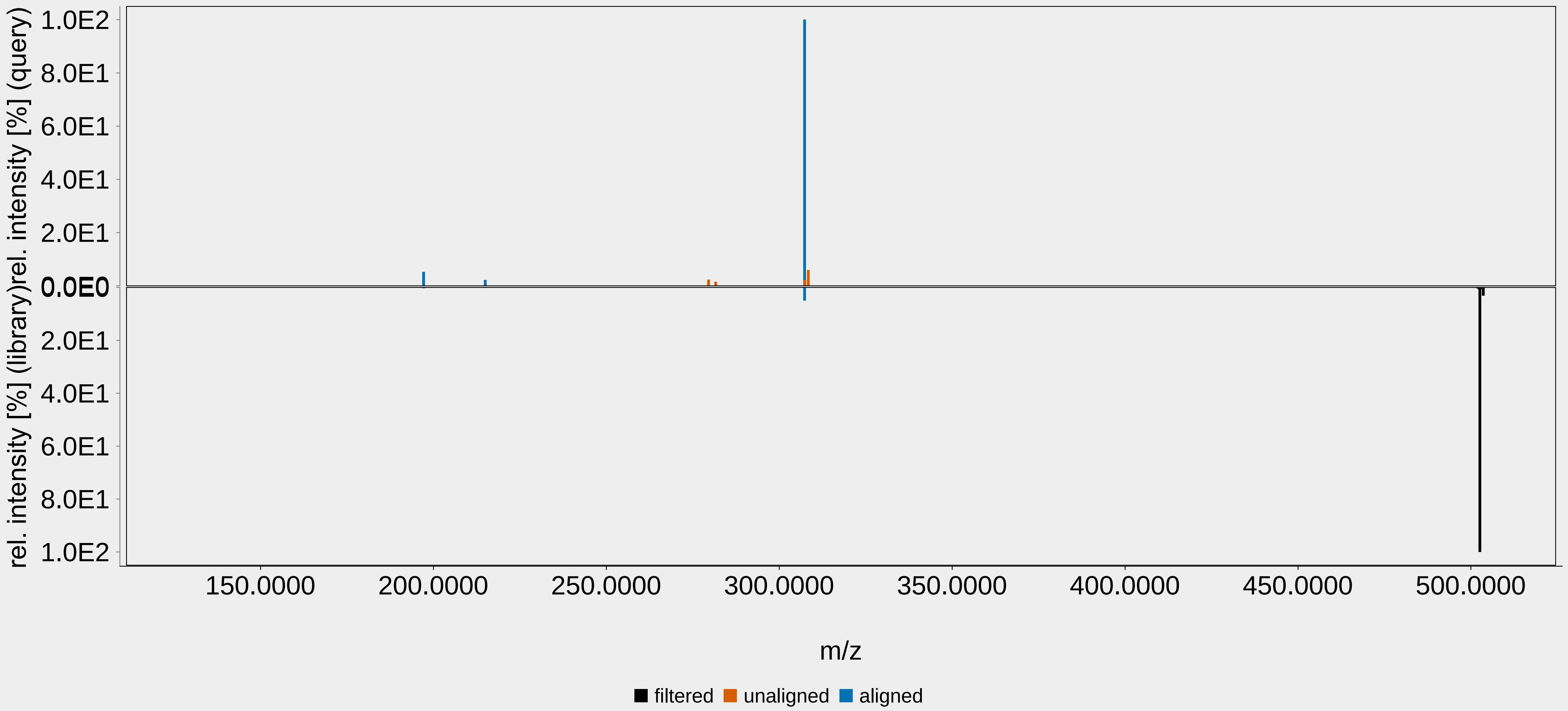

Compound information

Name: PE(20:2/0:0); [M-H]- C25H47N1O7P1

Exact mass: 0.0

Ion type: M-H

Precursor m/z: 504.309

Charge: 1

Polarity: Negative

Instrument information

Instrument type: HCD; Velos

Ion source: LC-ESI

Ms level: 2

Acquisition: Commercial

Database links

Other information

Principal investigator: Thomas Metz

Data collector: Thomas Metz

Entry ID: CCMSLIB00010086312

PS 36:4 **0.759**

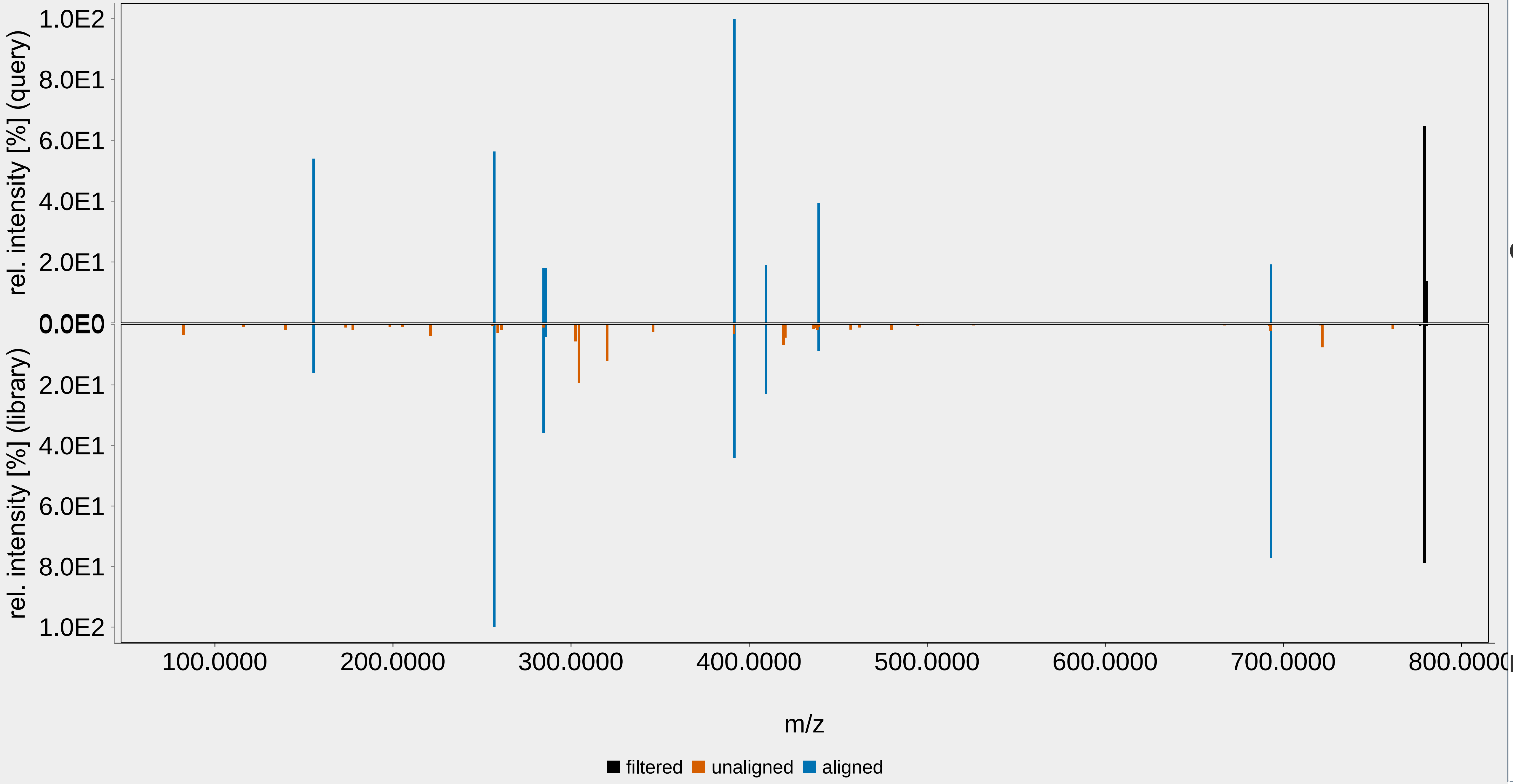

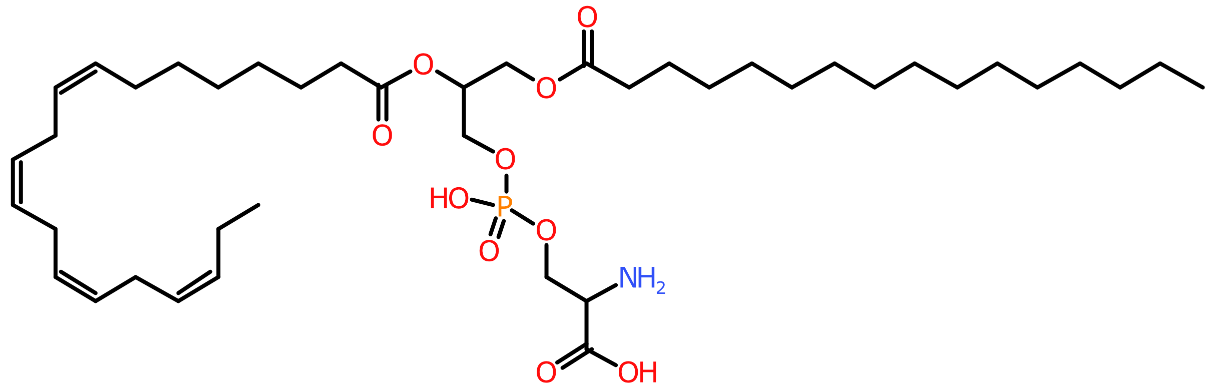

Compound information

Name: PS 36:4

Formula: C42H74NO10P

Exact mass: 784.0269999999999

Ion type: [M-H]-

Precursor m/z: 782.494

Polarity: NEGATIVE

InChI:

InChI key:

SMILES:

Num peaks: 55

Instrument information

Instrument type: LC-ESI-QTOF

Instrument: AB Sciex TripleTOF 5600+ system

Ms level: MS2

Collision energy: 45V

Database links

Other information

Entry ID: MSBNK-RIKEN_IMS-LQB00715

Comment: Parent=782.494

PS 36:4 **0.759**

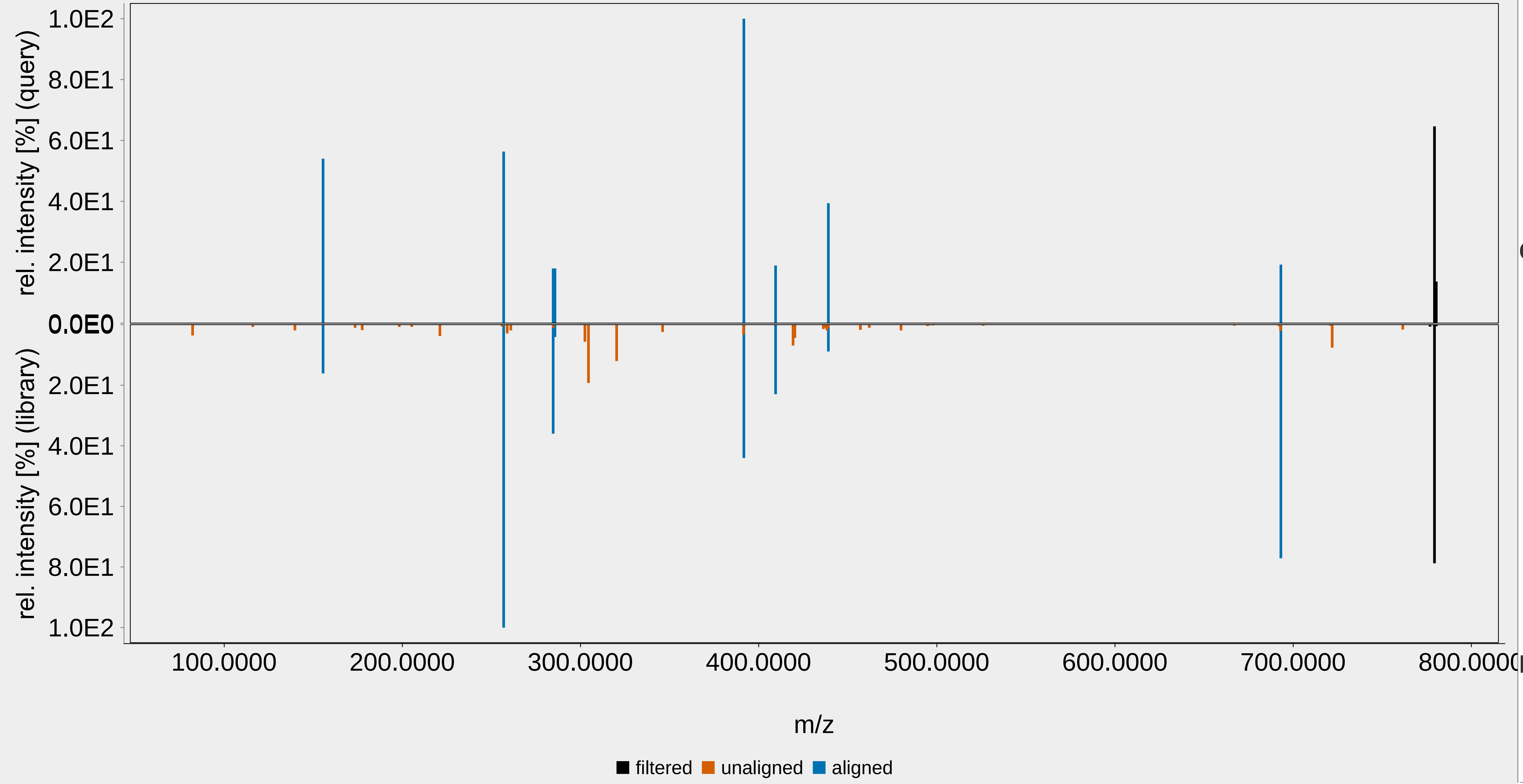

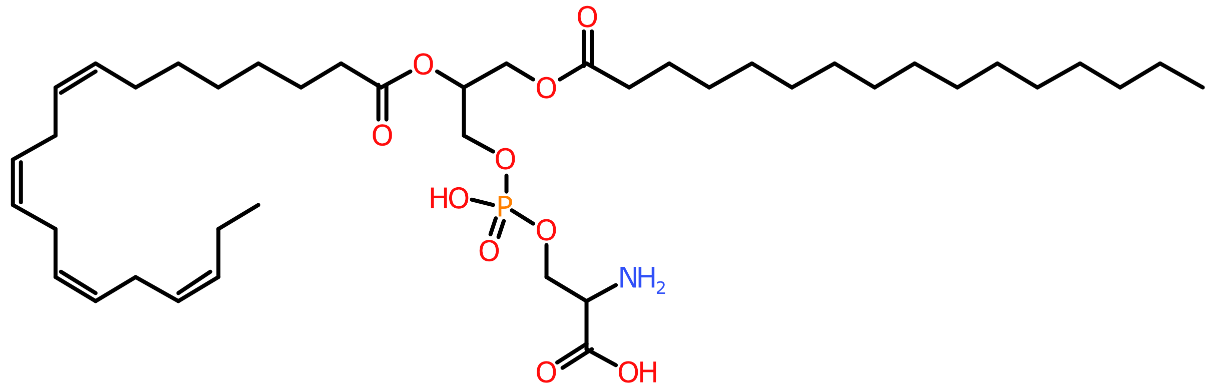

Compound information

Name: PS 36:4

Formula: C42H74NO10P

Exact mass: 784.0269999999999

Ion type: [M-H]-

Precursor m/z: 782.494

Polarity: NEGATIVE

InChI:

InChI key:

SMILES:

Num peaks: 55

Instrument information

Instrument type: LC-ESI-QTOF

Instrument: AB Sciex TripleTOF 5600+ system

Ms level: MS2

Collision energy: 45V

Database links

Other information

Entry ID: MSBNK-RIKEN_IMS-LQB00329

Comment: Parent=782.494

PS(18:0/0:0); [M-H]- C24H47N1O9P1 0.727

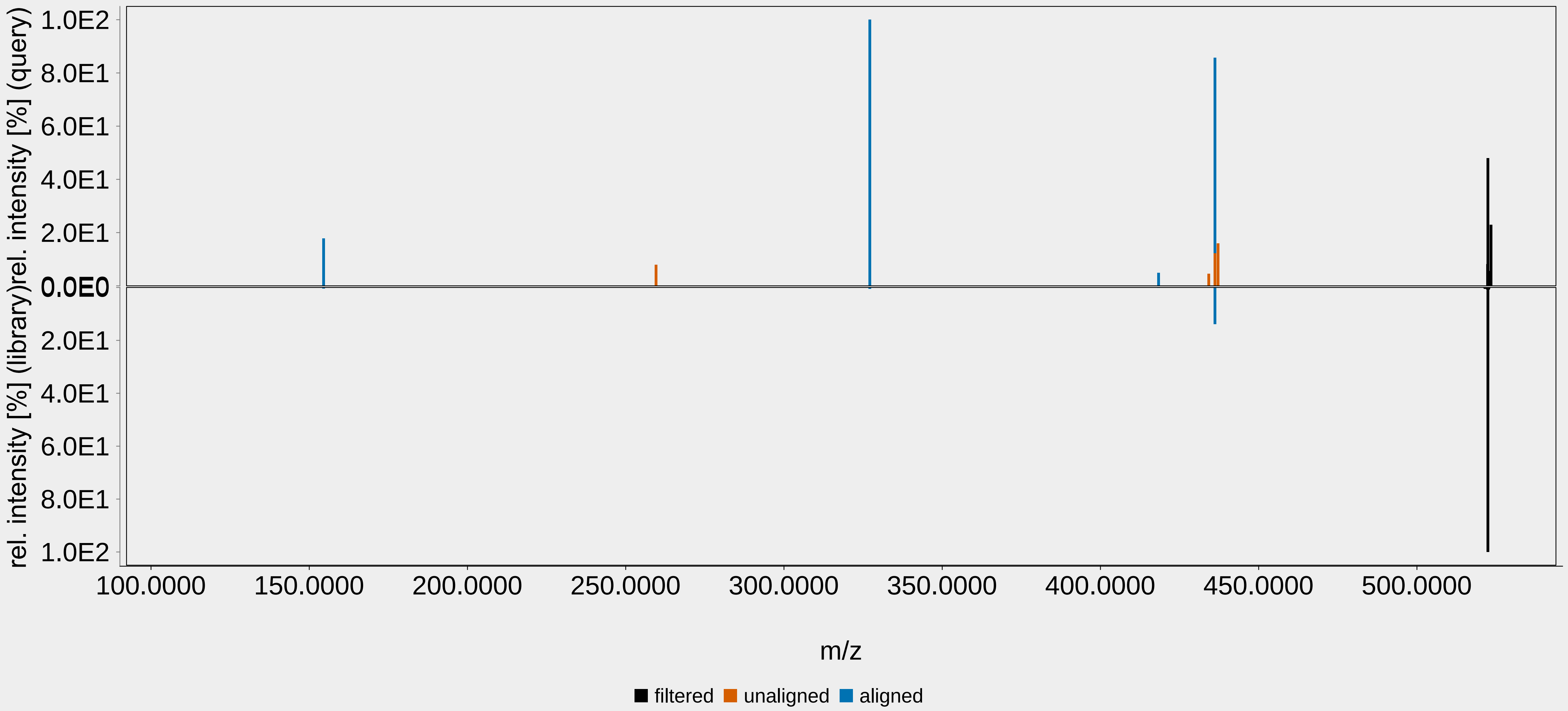

Compound information

Name: PS(18:0/0:0); [M-H]- C24H47N1O9P1

Exact mass: 0.0

Ion type: M-H

Precursor m/z: 524.299

Charge: 1

Polarity: Negative

Instrument information

Instrument type: HCD; Velos

Ion source: LC-ESI

Ms level: 2

Acquisition: Commercial

Database links

Other information

Principal investigator: Thomas Metz

Data collector: Thomas Metz

Entry ID: CCMSLIB00010074162

PE(16:1/0:0); [M-H]- C21H41N1O7P1 **0.825**

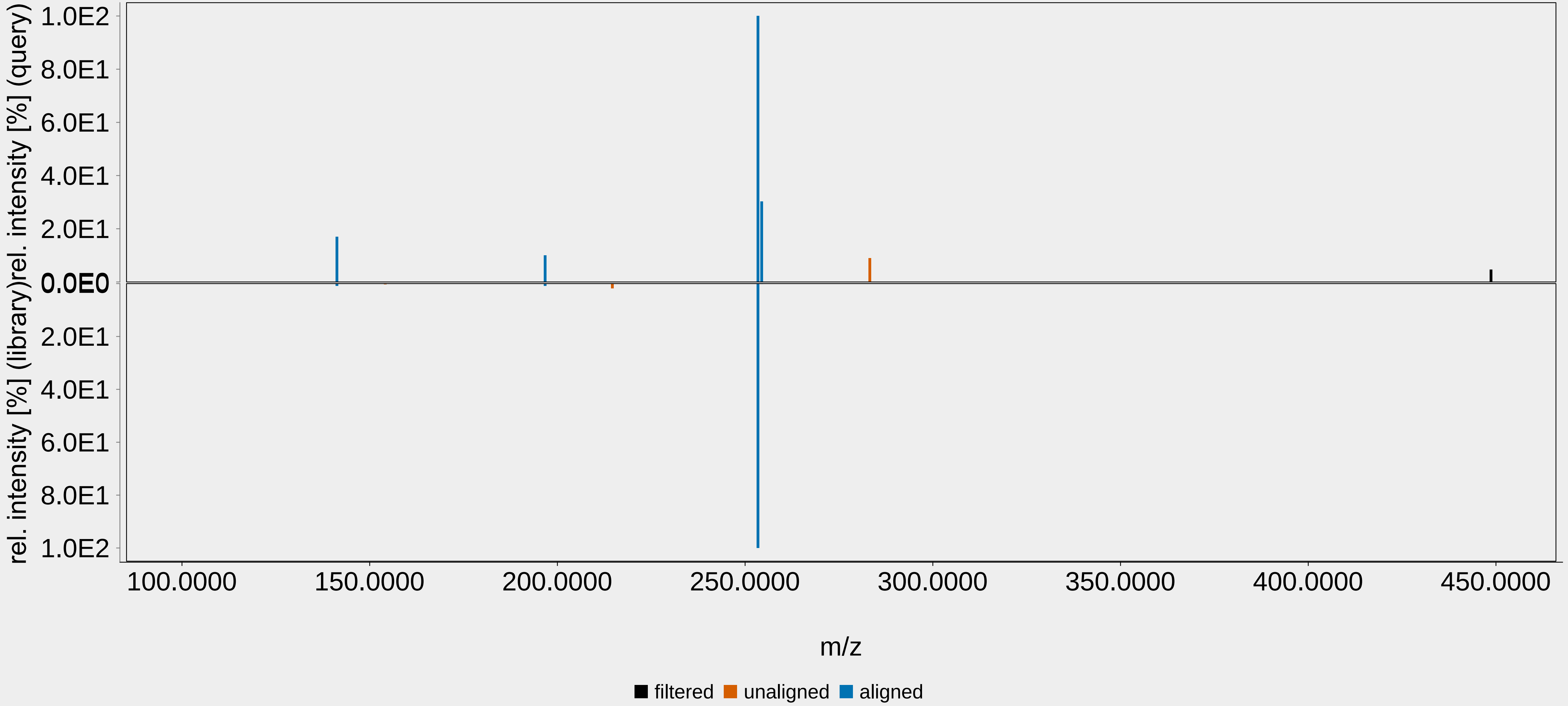

Compound information

Name: PE(16:1/0:0); [M-H]- C21H41N1O7P1

Exact mass: 0.0

Ion type: M-H

Precursor m/z: 450.262

Charge: 1

Polarity: Negative

Instrument information

Instrument type: HCD; Lumos

Ion source: LC-ESI

Ms level: 2

Acquisition: Commercial

Database links

Other information

Principal investigator: Thomas Metz

Data collector: Thomas Metz

Entry ID: CCMSLIB00010059507

PE(16:1/0:0); [M-H]- C21H41N1O7P1 0.848

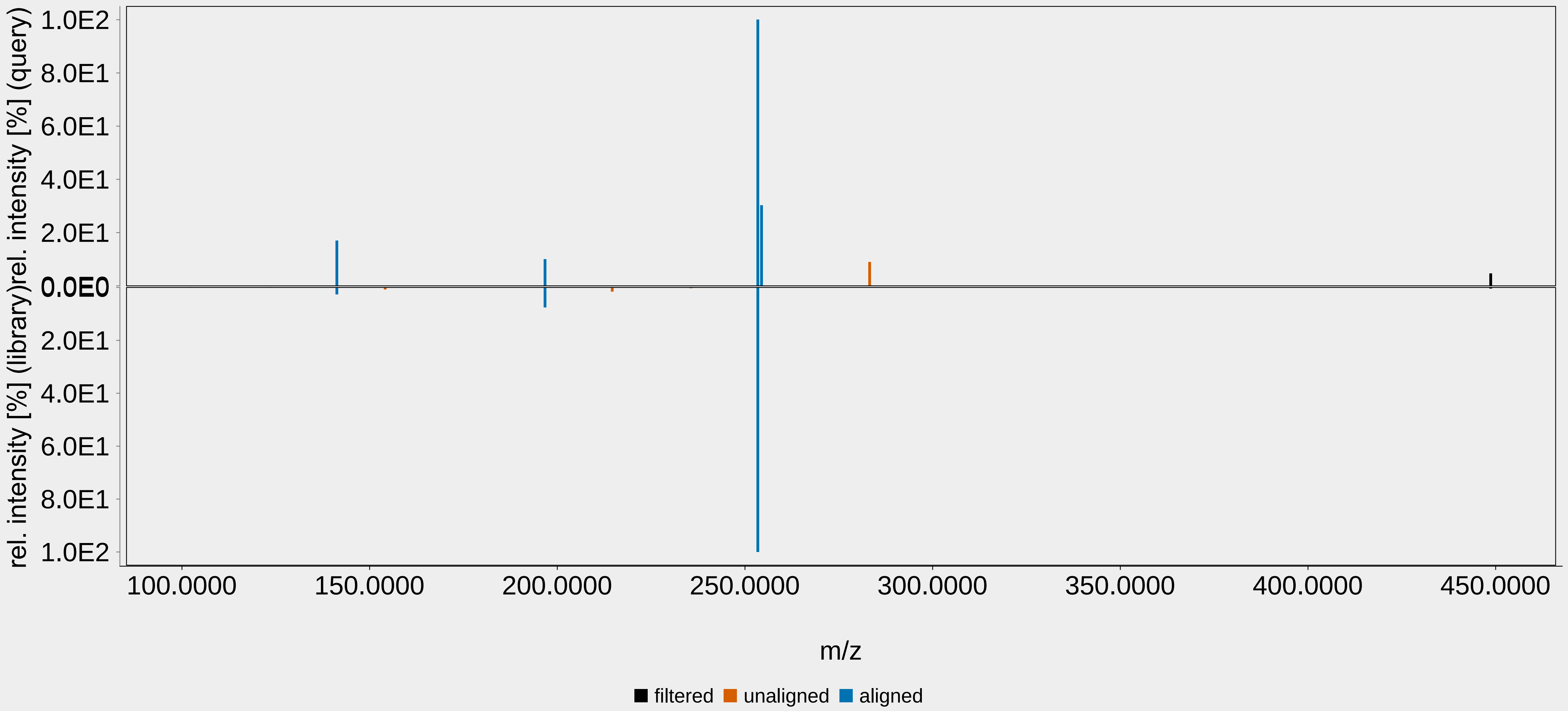

Compound information

Name: PE(16:1/0:0); [M-H]- C21H41N1O7P1

Exact mass: 0.0

Ion type: M-H

Precursor m/z: 450.262

Charge: 1

Polarity: Negative

Instrument information

Instrument type: HCD; Lumos

Ion source: LC-ESI

Ms level: 2

Acquisition: Commercial

Database links

Other information

Principal investigator: Thomas Metz

Data collector: Thomas Metz

Entry ID: CCMSLIB00010059800

PE(20:4/22:4); [M-H]- C47H77N1O8P1 **0.732**

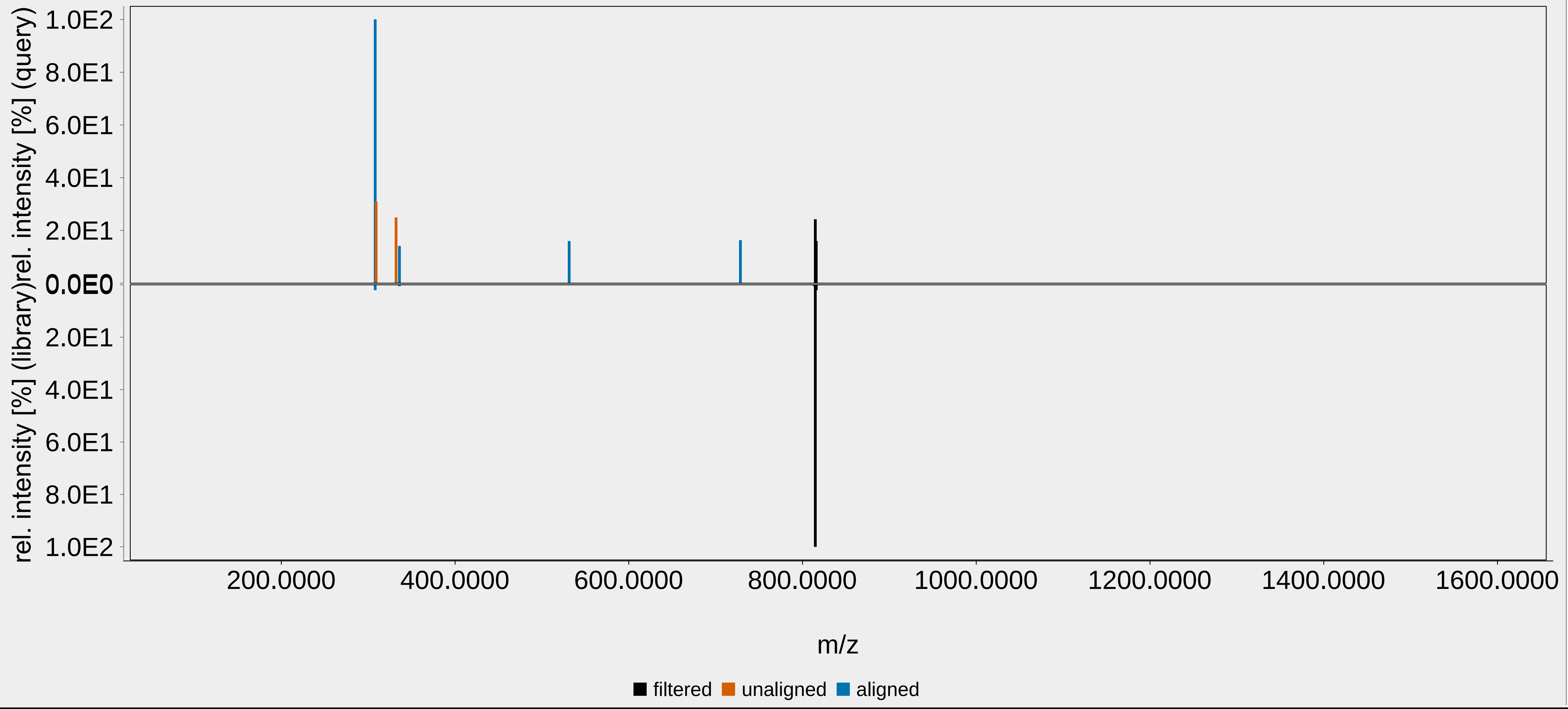

Compound information

Name: PE(20:4/22:4); [M-H]- C47H77N1O8P1

Exact mass: 0.0

Ion type: M-H

Precursor m/z: 814.539

Charge: 1

Polarity: Negative

Instrument information

Instrument type: HCD; Velos

Ion source: LC-ESI

Ms level: 2

Acquisition: Commercial

Database links

Other information

Principal investigator: Thomas Metz

Data collector: Thomas Metz

Entry ID: CCMSLIB00010063417

PE(20:4/22:4); [M-H]- C47H77N1O8P1 **0.860**

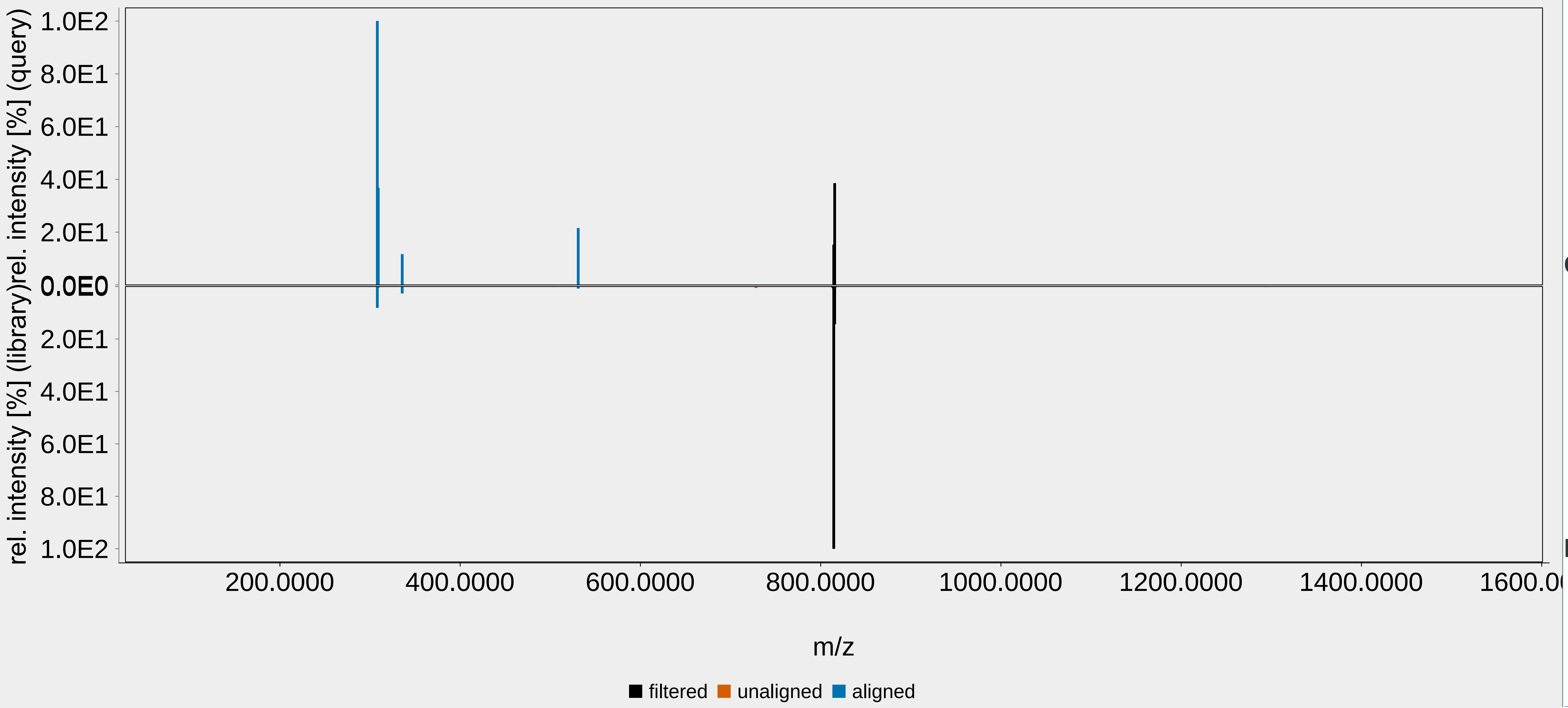

Compound information

Name: PE(20:4/22:4); [M-H]- C47H77N1O8P1

Exact mass: 0.0

Ion type: M-H

Precursor m/z: 814.539

Charge: 1

Polarity: Negative

Instrument information

Instrument type: HCD; Velos

Ion source: LC-ESI

Ms level: 2

Acquisition: Commercial

Database links

Other information

Principal investigator: Thomas Metz

Data collector: Thomas Metz

Entry ID: CCMSLIB00010087014

PA 18:0_20:3

0.742

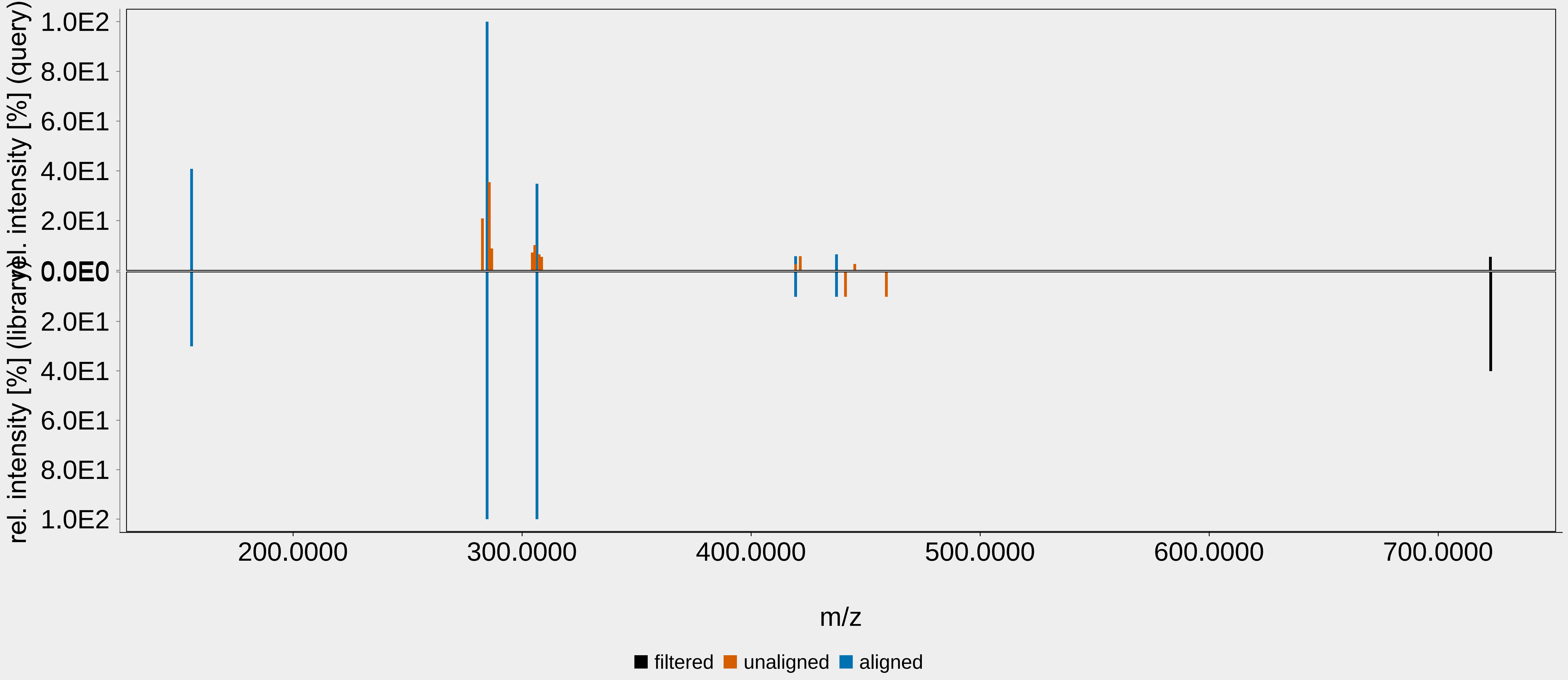

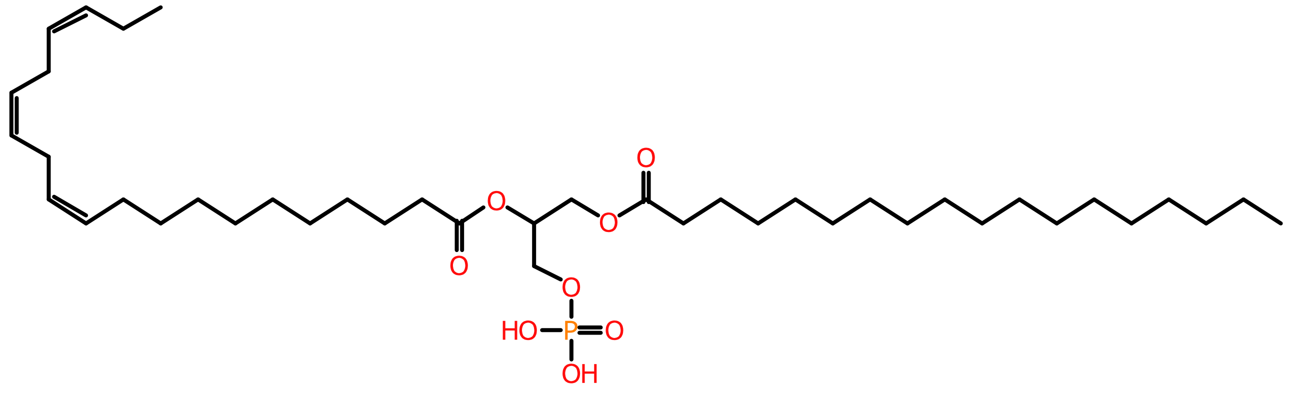

Compound information

Name: PA 18:0_20:3

Formula: C41H75O8P

Ion type: [M-H]-

Precursor m/z: 725.51268

Ccs: 263.04

InChI key: KGDFLTPILJIQMX-JTBMWNAQNA-

SMILES:

Num peaks: 8

Instrument information

Database links

Other information

PE(20:4/0:0); [M-H]- C25H43N1O7P1 **0.917**

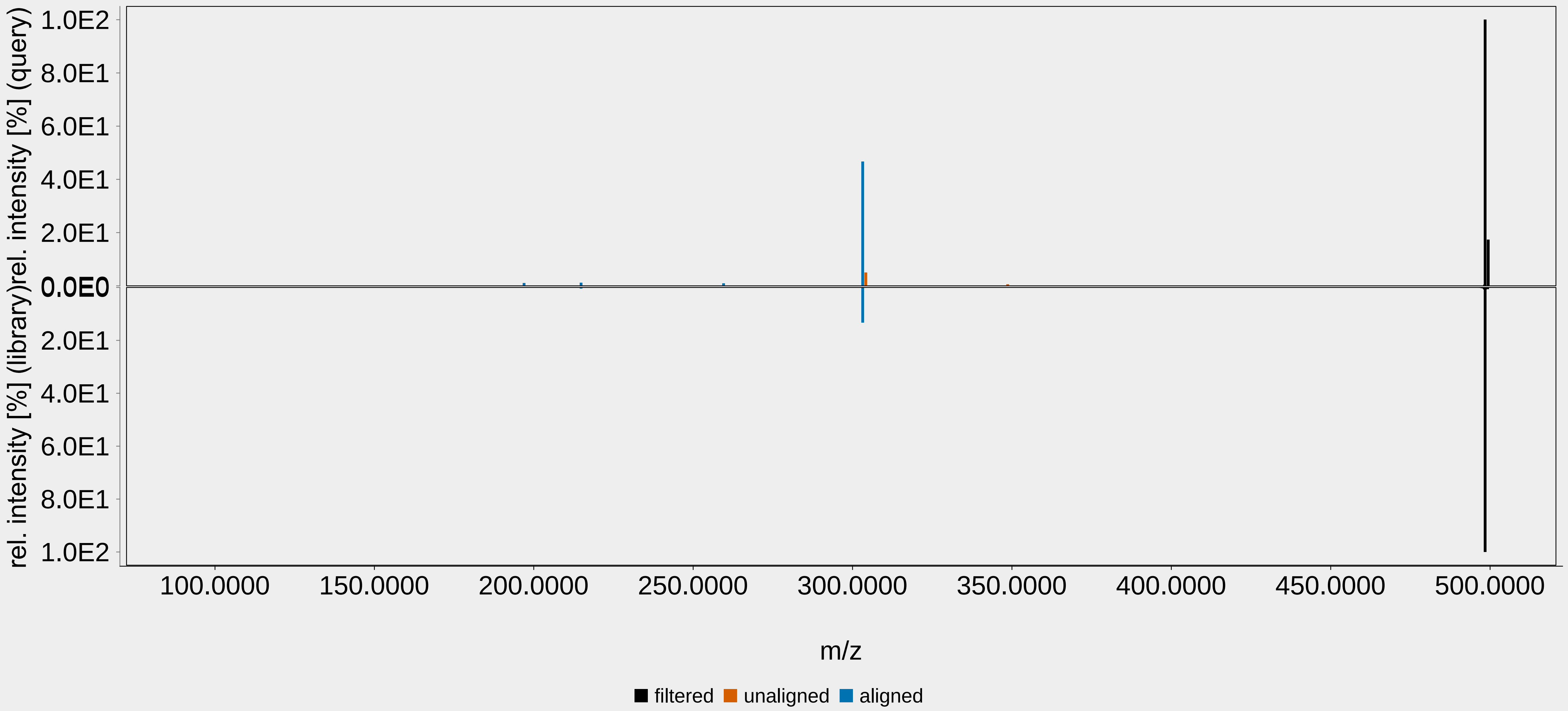

Compound information

Name: PE(20:4/0:0); [M-H]- C25H43N1O7P1

Exact mass: 0.0

Ion type: M-H

Precursor m/z: 500.278

Charge: 1

Polarity: Negative

Instrument information

Instrument type: HCD; Velos

Ion source: LC-ESI

Ms level: 2

Acquisition: Commercial

Database links

Other information

Principal investigator: Thomas Metz

Data collector: Thomas Metz

Entry ID: CCMSLIB00010067367

PE(20:4/0:0); [M-H]- C25H43N1O7P1 **0.921**

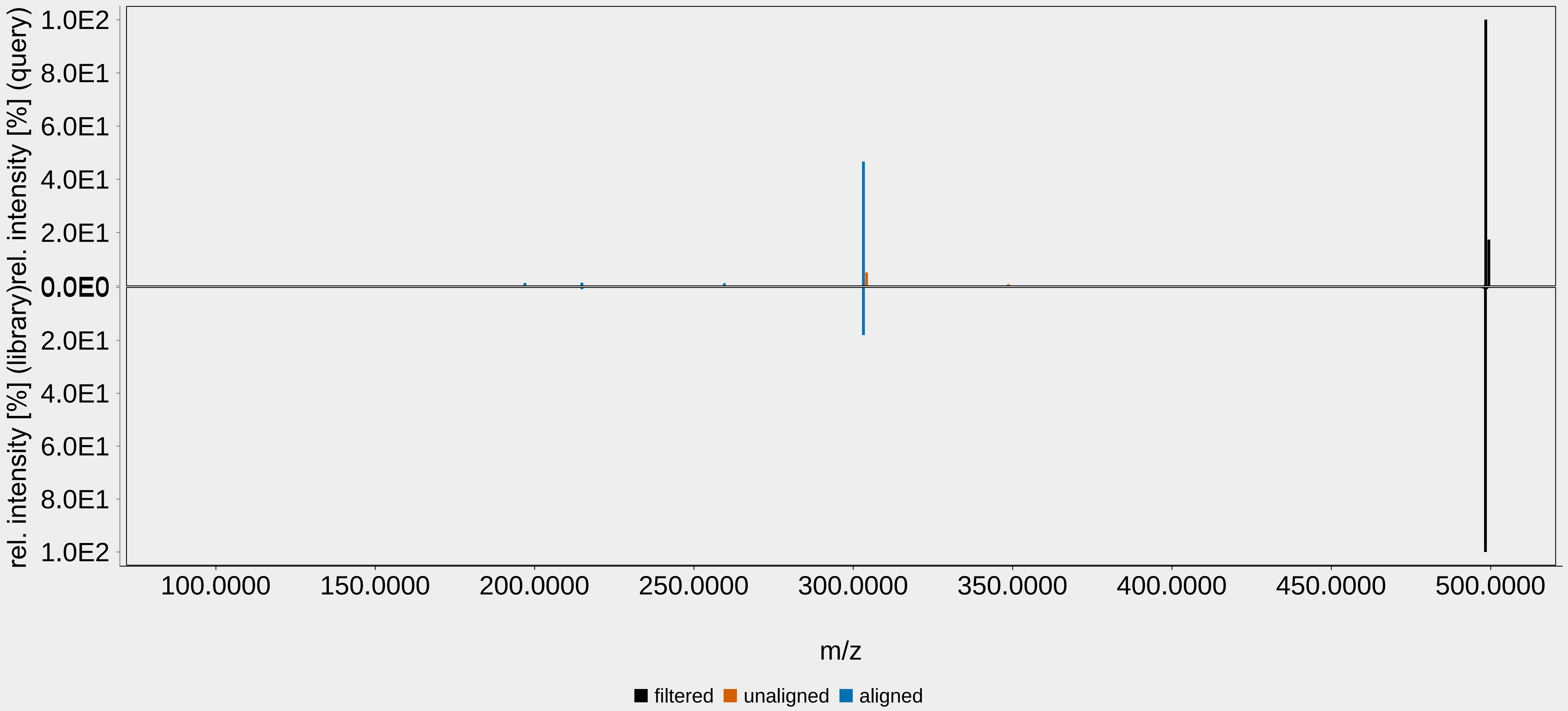

Compound information

Name: PE(20:4/0:0); [M-H]- C25H43N1O7P1

Exact mass: 0.0

Ion type: M-H

Precursor m/z: 500.278

Charge: 1

Polarity: Negative

Instrument information

Instrument type: HCD; Velos

Ion source: LC-ESI

Ms level: 2

Acquisition: Commercial

Database links

Other information

Principal investigator: Thomas Metz

Data collector: Thomas Metz

Entry ID: CCMSLIB00010085243

Dodecylbenzenesulfonic acid

0.965

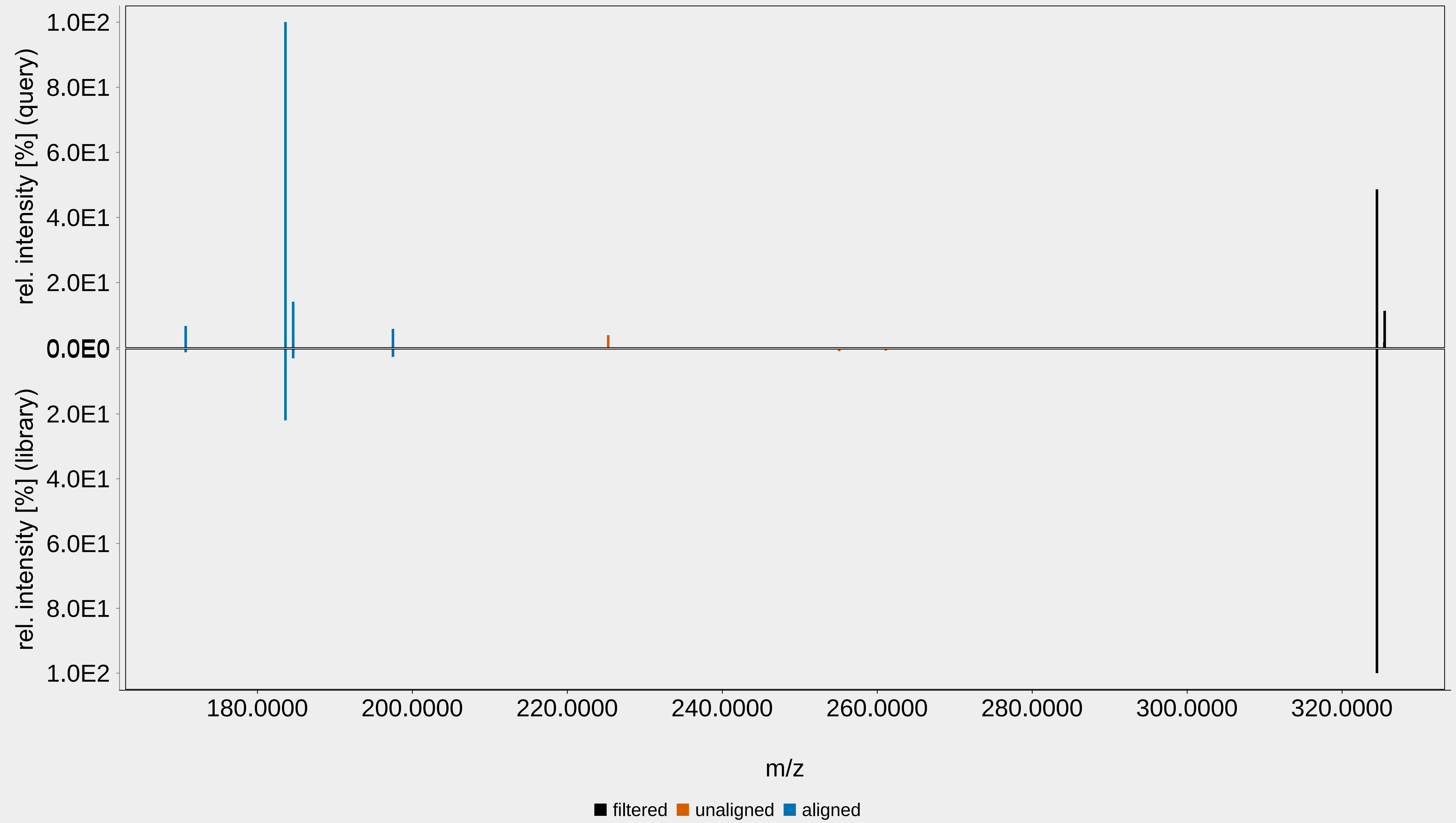

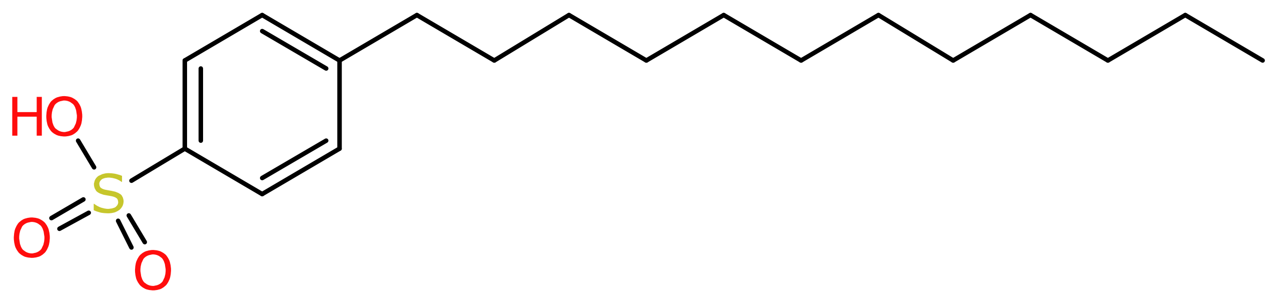

Compound information

Name: Dodecylbenzenesulfonic acid

Formula: C18H30O3S

Mol. weight: 326.19157

Exact mass: 326.19157

Ion type: [M-H]-

Precursor m/z: 325.1843

Polarity: negative

InChI:

InChI key:

SMILES:

Instrument information

Instrument type: LC-ESI-QFT

Instrument: Q Exactive Plus Orbitrap Thermo

Ion source: ESI

Resolution: 35000

Ms level: MS2

Collision energy: 35 (nominal)

Database links

Pubchem: 8485

MoNA ID: SM876251

Chemspider: 8172

CAS: 121-65-3

Other information

Principal investigator: Krauss M, Schymanski

Data collector: Krauss M, Schymanski EL,

Dodecylbenzenesulfonic acid

0.966

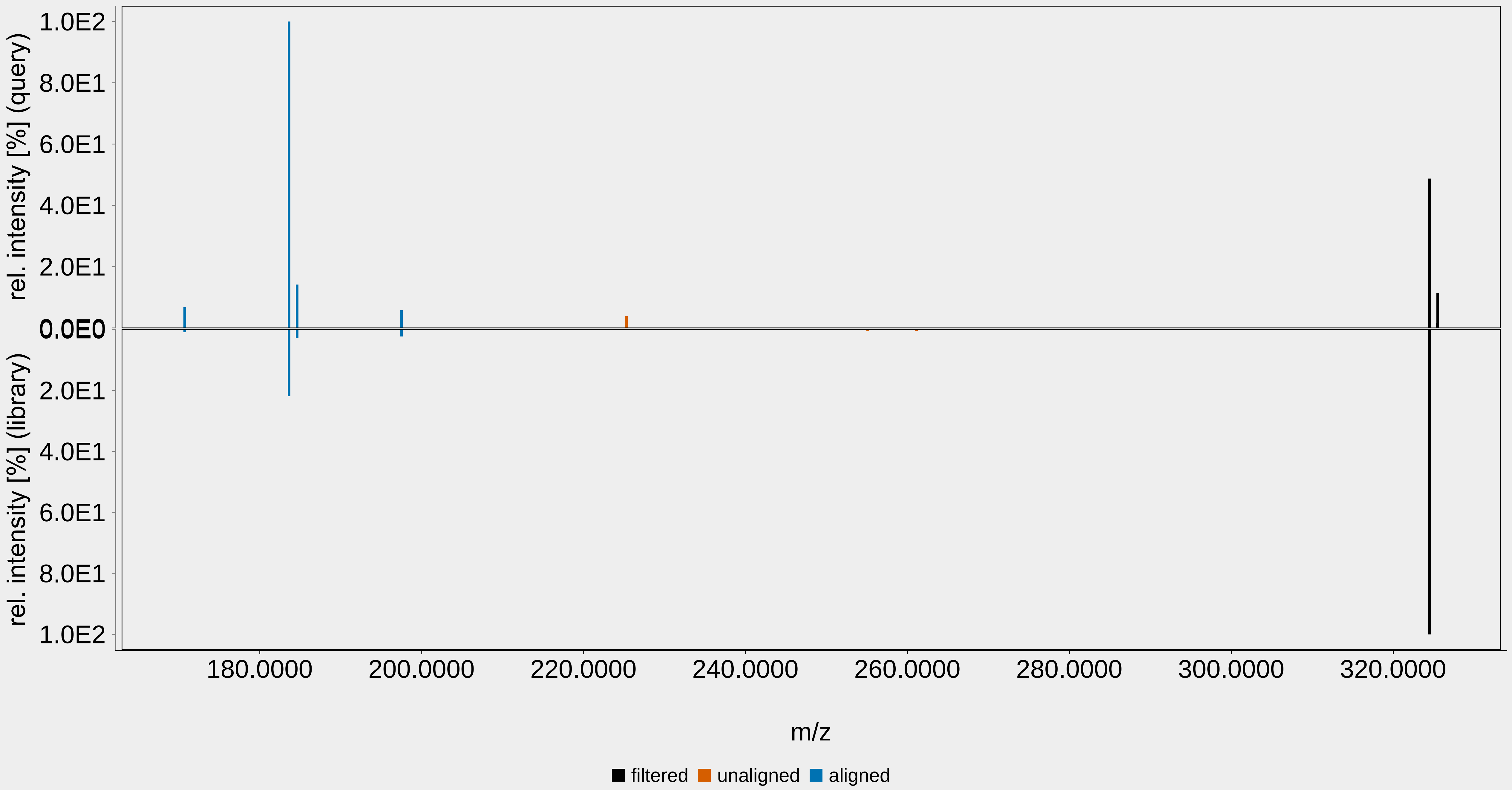

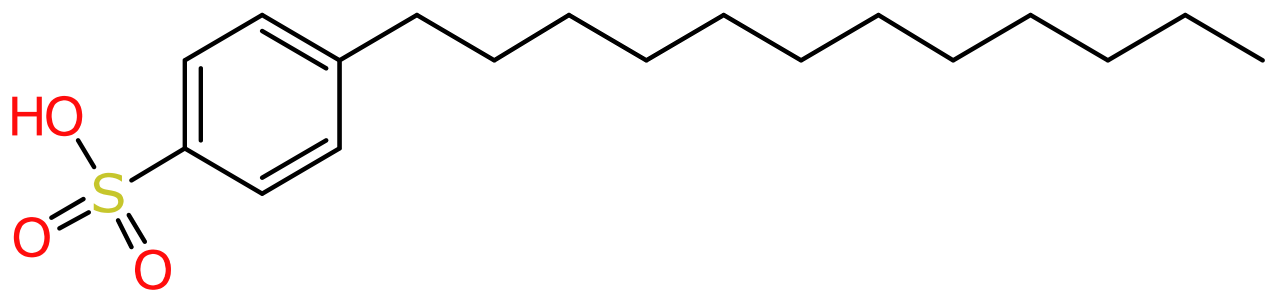

Compound information

Name: Dodecylbenzenesulfonic acid

Formula: C18H30O3S

Exact mass: 326.19157

Ion type: [M-H]-

Precursor m/z: 325.1843

Polarity: NEGATIVE

InChI:

InChI key:

SMILES:

Num peaks: 7

Instrument information

Instrument type: LC-ESI-QFT

Instrument: Q Exactive Plus Orbitrap Thermo

Ms level: MS2

Collision energy: 35 (nominal)

Database links

Other information

Entry ID: MSBNK-CASMI_2016-SM876251

Comment: Parent=325.1843

PS(16:0/18:1); [M-H]- C40H75N1O10P1 **0.938**

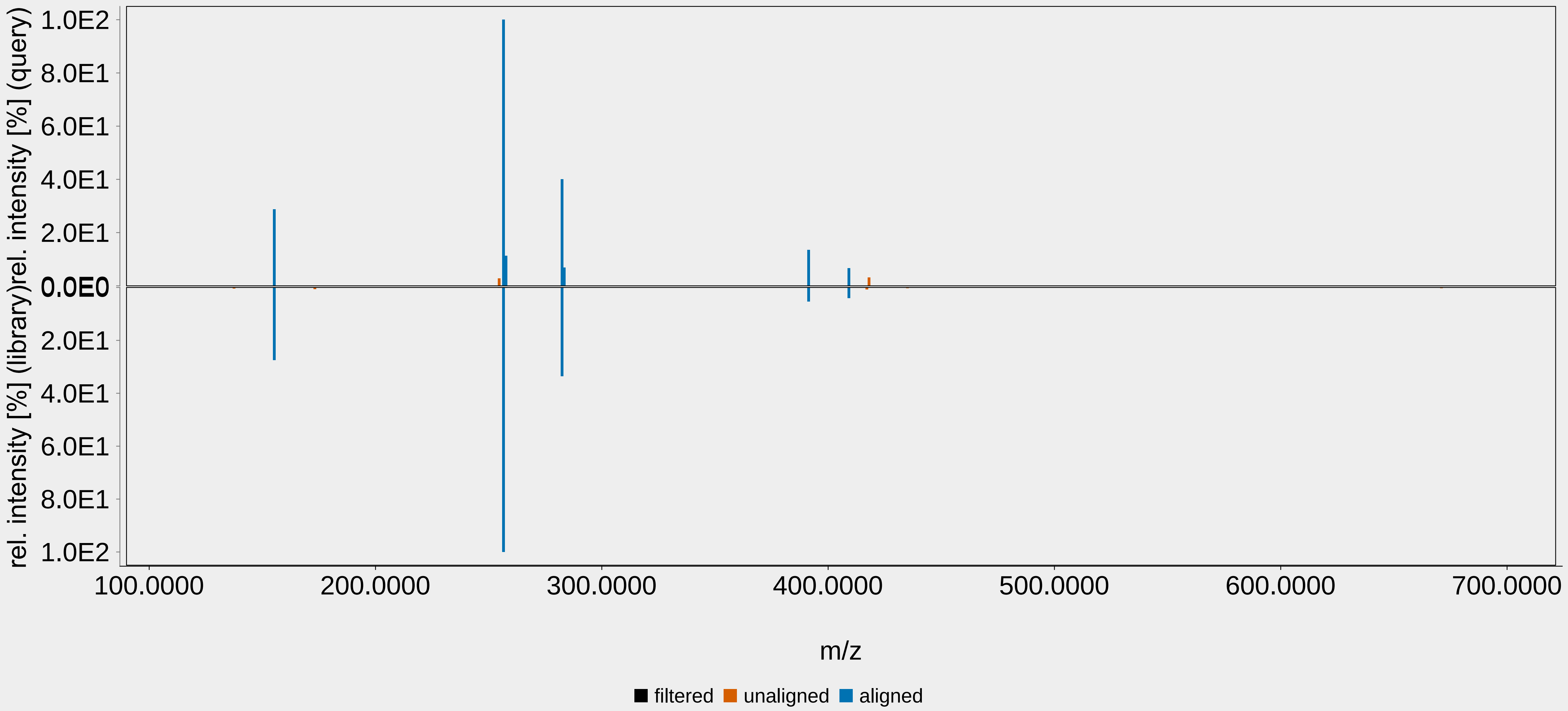

Compound information

Name: PS(16:0/18:1); [M-H]- C40H75N1O10P1

Exact mass: 0.0

Ion type: M-H

Precursor m/z: 760.513

Charge: 1

Polarity: Negative

Instrument information

Instrument type: HCD; Lumos

Ion source: LC-ESI

Ms level: 2

Acquisition: Commercial

Database links

Other information

Principal investigator: Thomas Metz

Data collector: Thomas Metz

Entry ID: CCMSLIB00010059839

PS(16:0/18:1); [M-H]- C40H75N1O10P1 **0.938**

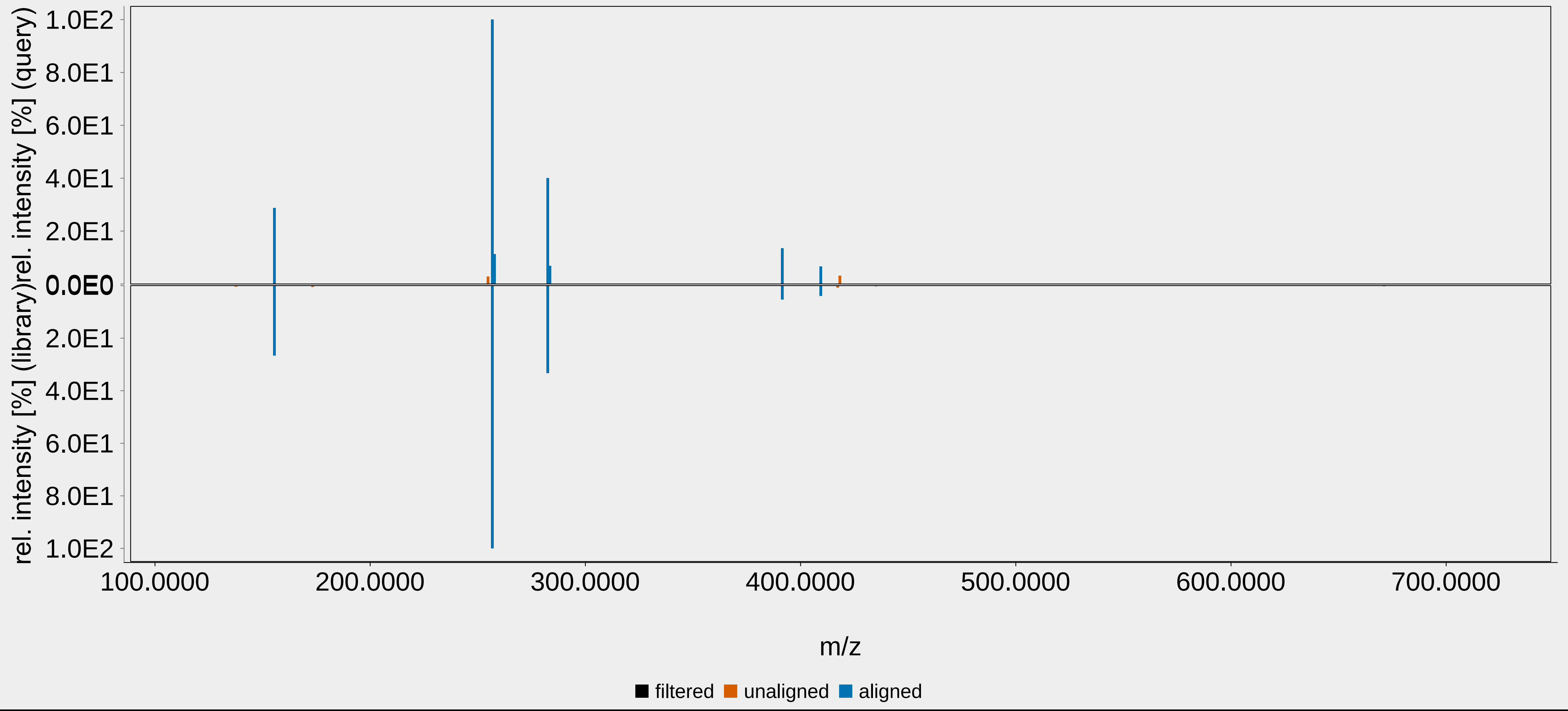

Compound information

Name: PS(16:0/18:1); [M-H]- C40H75N1O10P1

Exact mass: 0.0

Ion type: M-H

Precursor m/z: 760.513

Charge: 1

Polarity: Negative

Instrument information

Instrument type: HCD; Lumos

Ion source: LC-ESI

Ms level: 2

Acquisition: Commercial

Database links

Other information

Principal investigator: Thomas Metz

Data collector: Thomas Metz

Entry ID: CCMSLIB00010059457

PI 36:1

0.730

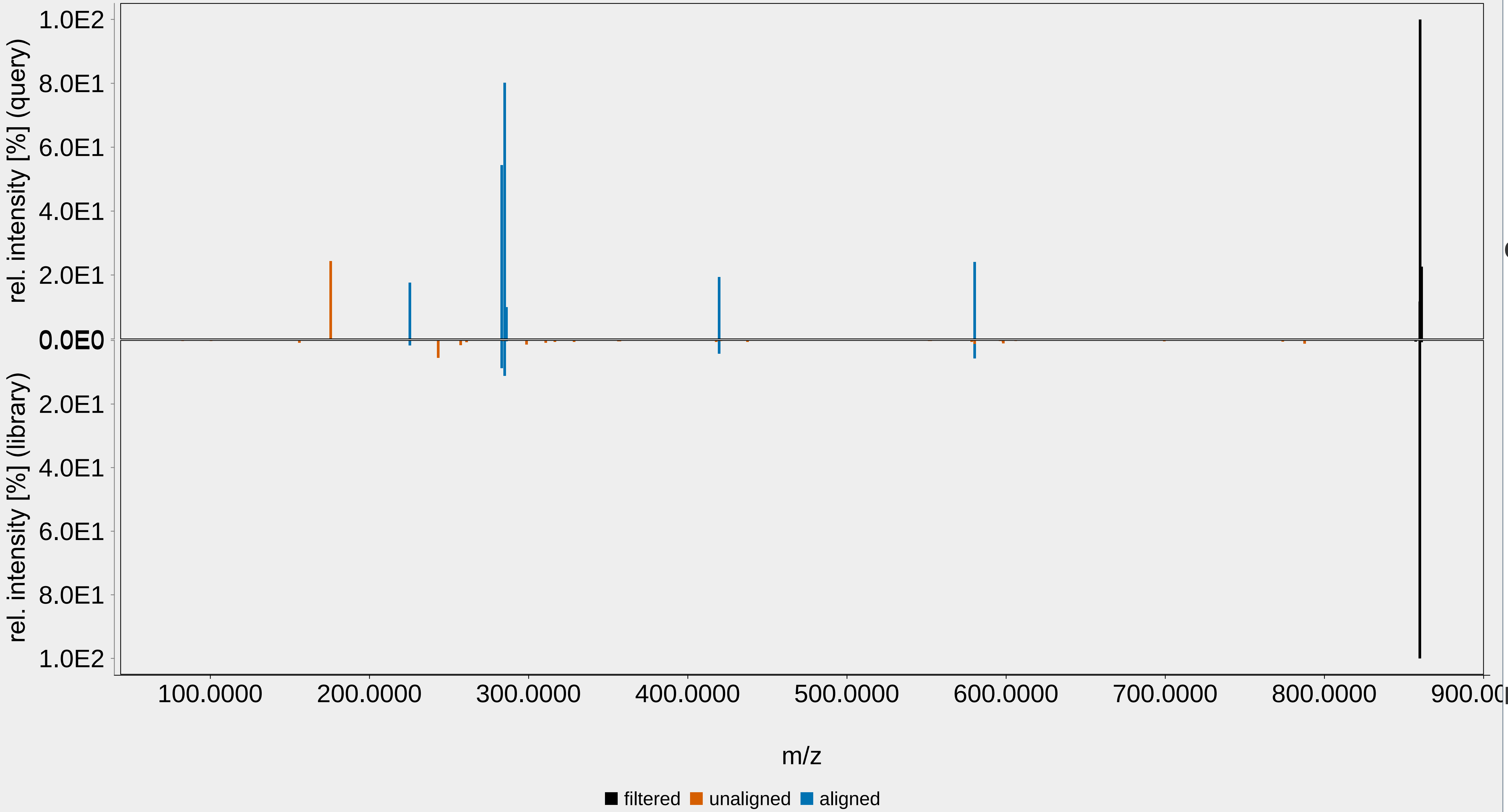

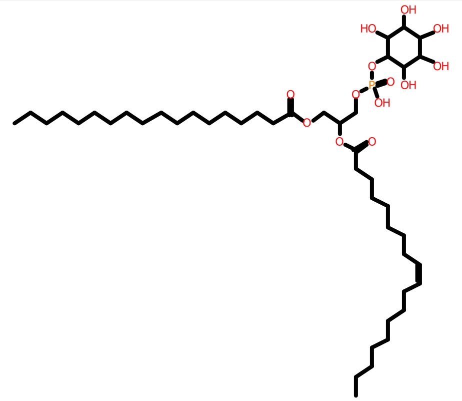

Compound information

Name: PI 36:1

Formula: C45H85O13P

Mol. weight: 865.1379999999999

Exact mass: 865.1379999999999

Ion type: [M-H]-

Precursor m/z: 863.5657

RT: 9.13166

Polarity: negative

InChI:

InChI key: YOBFISPJJWPPTK-ZZEZOPTASA-I

SMILES:

Instrument information

Instrument type: LC-ESI-QTOF

Instrument: AB Sciex TripleTOF 5600+ system

Ms level: MS2

Collision energy: 45V

Database links

Pubchem: 138306742

MoNA ID: LQB00691

Other information

Principal investigator: Kazutaka Ikeda, Hiroshi

Data collector: Kazutaka Ikeda, Hiroshi

PI 36:1 **0.730**

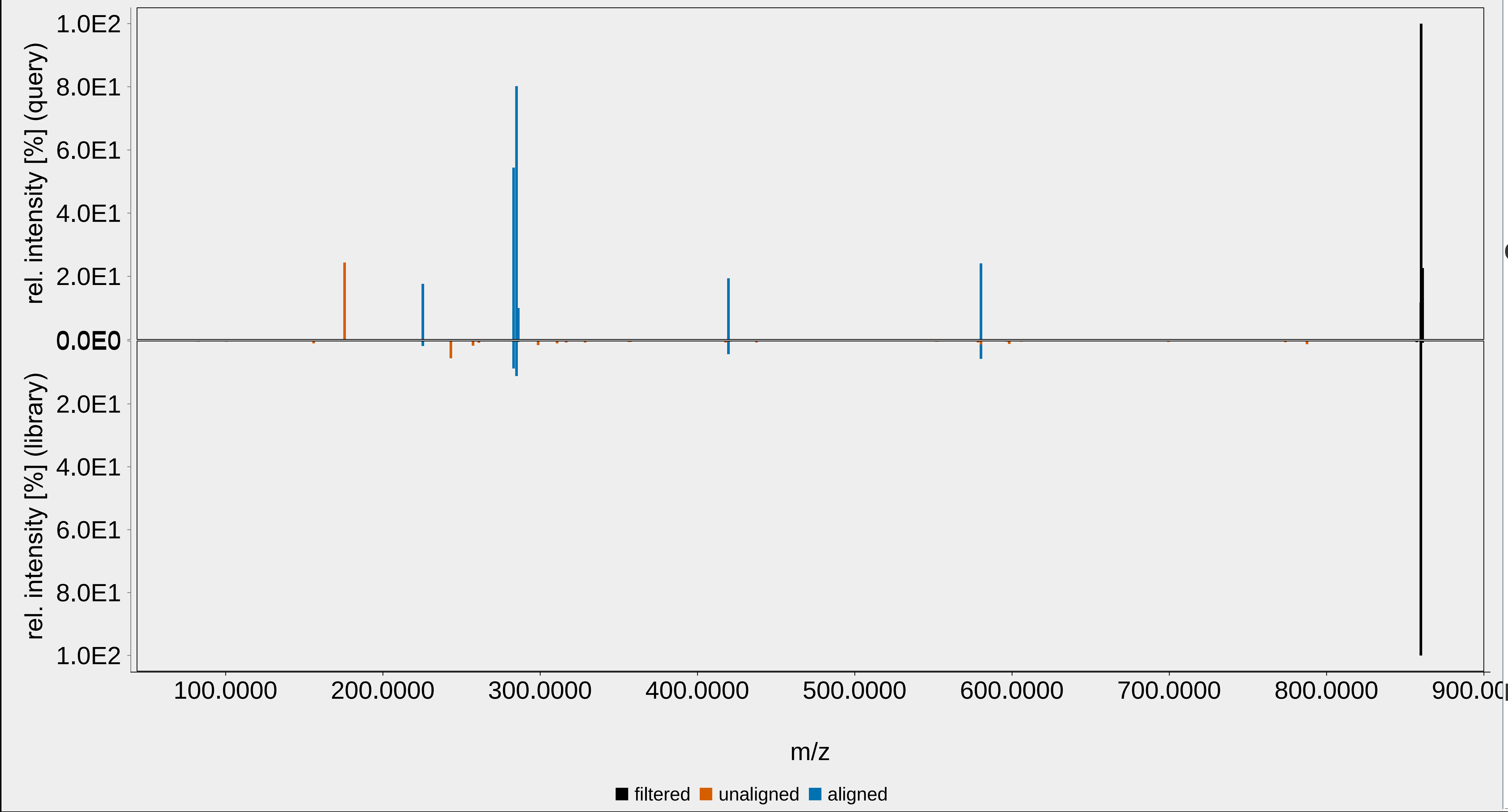

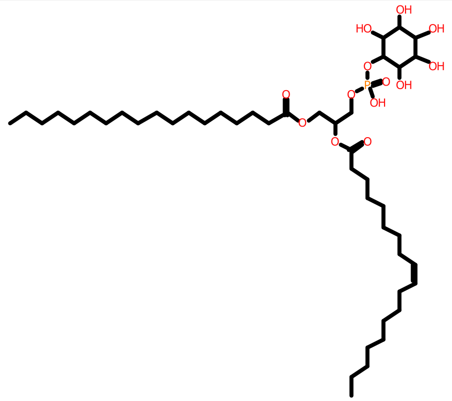

Compound information

Name: PI 36:1

Formula: C45H85O13P

Mol. weight: 865.1379999999999

Exact mass: 865.1379999999999

Ion type: [M-H]-

Precursor m/z: 863.5657

RT: 9.13166

Polarity: negative

InChI:

InChI key: YOBFISPJJWPPTK-ZZEZOPTASA-I

SMILES:

Instrument information

Instrument type: LC-ESI-QTOF

Instrument: AB Sciex TripleTOF 5600+ system

Ms level: MS2

Collision energy: 45V

Database links

Pubchem: 138306742

MoNA ID: LQB00305

Other information

Principal investigator: Kazutaka Ikeda, Hiroshi

Data collector: Kazutaka Ikeda, Hiroshi

PA 16:0_18:0

0.850

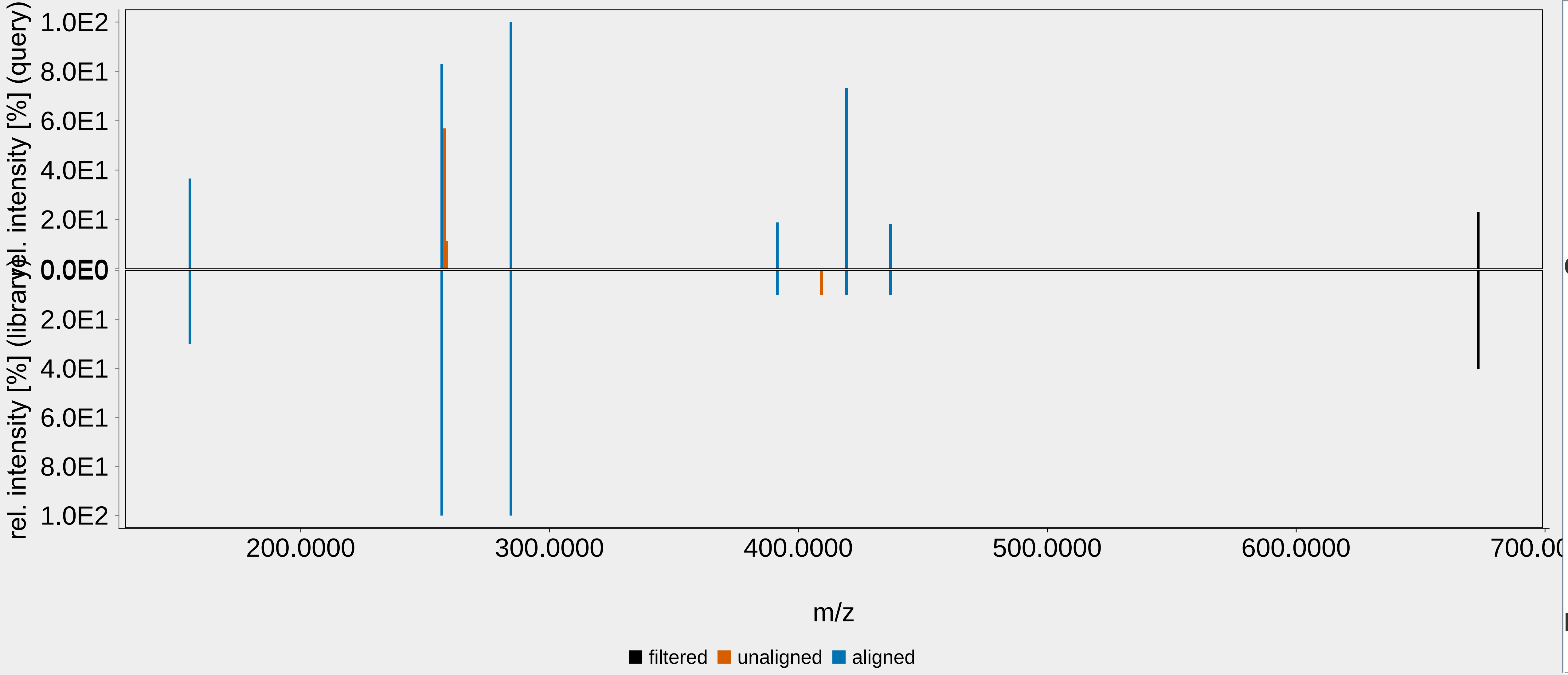

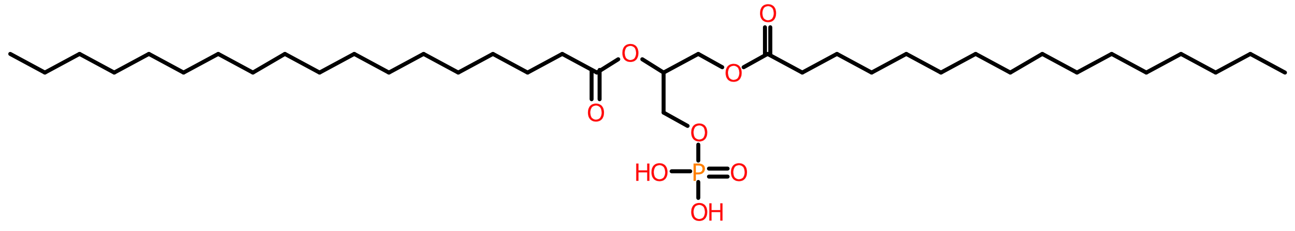

Compound information

Name: PA 16:0_18:0

Formula: C37H73O8P

Ion type: [M-H]-

Precursor m/z: 675.49703

Ccs: 256.25

InChI key: GASPGIDKILJBMM-UHFFFAOYNA-

SMILES:

Num peaks: 8

Instrument information

Database links

Other information

PA(16:1/18:1); [M-H]- C37H68O8P1 **0.788**

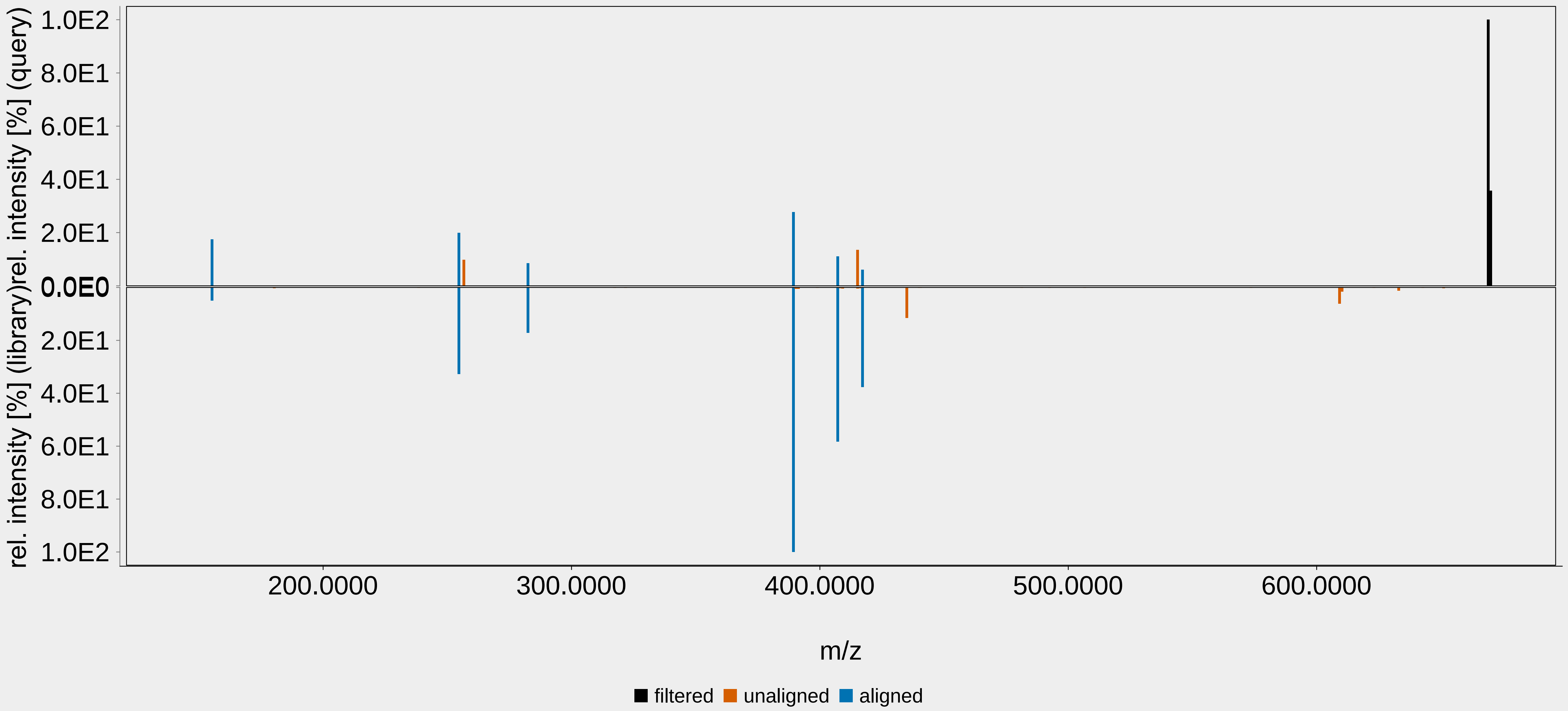

Compound information

Name: PA(16:1/18:1); [M-H]- C37H68O8P1

Exact mass: 0.0

Ion type: M-H

Precursor m/z: 671.465

Charge: 1

Polarity: Negative

Instrument information

Instrument type: CID; Lumos

Ion source: LC-ESI

Ms level: 2

Acquisition: Commercial

Database links

Other information

Principal investigator: Thomas Metz

Data collector: Thomas Metz

Entry ID: CCMSLIB00010059155

PA(16:1/18:1); [M-H]- C37H68O8P1 **0.913**

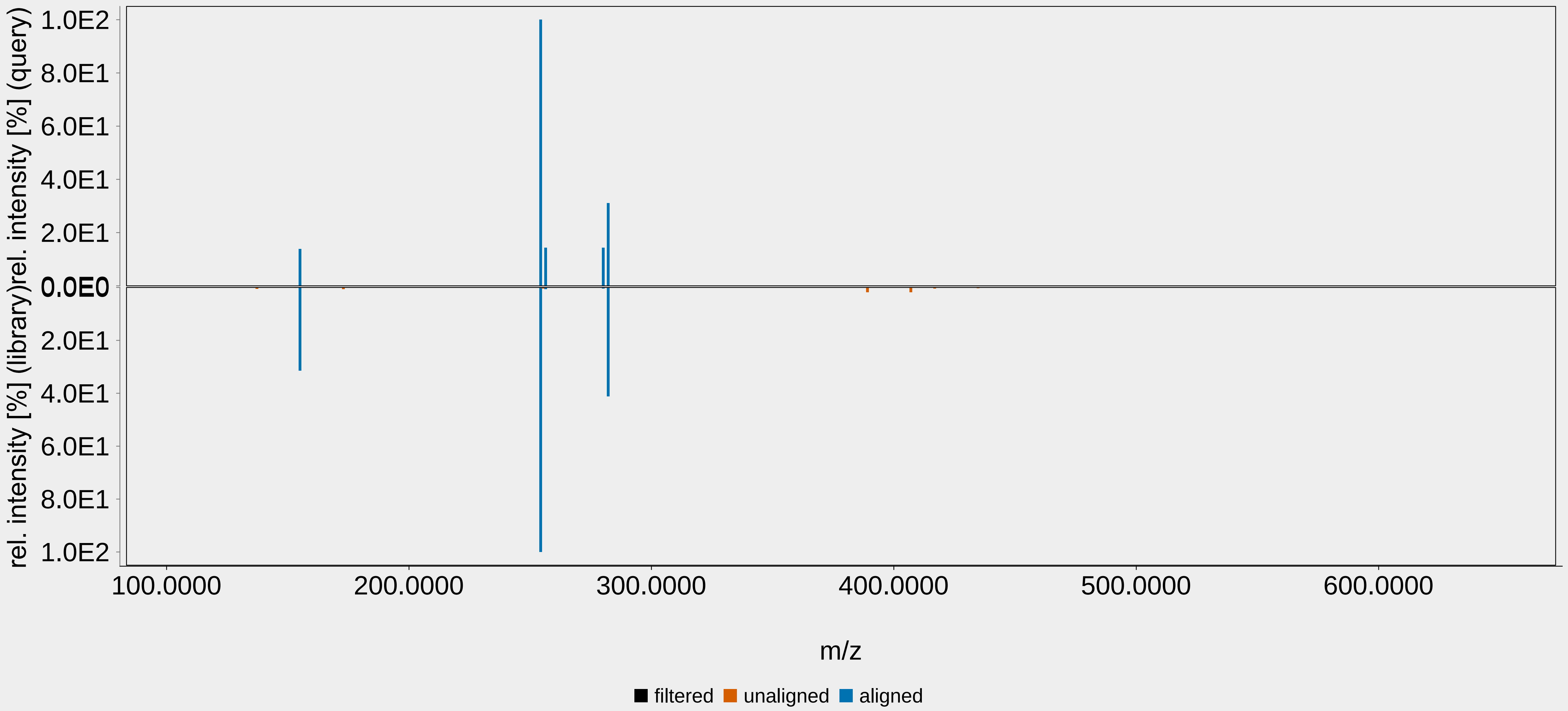

Compound information

Name: PA(16:1/18:1); [M-H]- C37H68O8P1

Exact mass: 0.0

Ion type: M-H

Precursor m/z: 671.465

Charge: 1

Polarity: Negative

Instrument information

Instrument type: HCD; Lumos

Ion source: LC-ESI

Ms level: 2

Acquisition: Commercial

Database links

Other information

Principal investigator: Thomas Metz

Data collector: Thomas Metz

Entry ID: CCMSLIB00010059156

ADENOSINE 5'-TRIPHOSPHATE

0.743

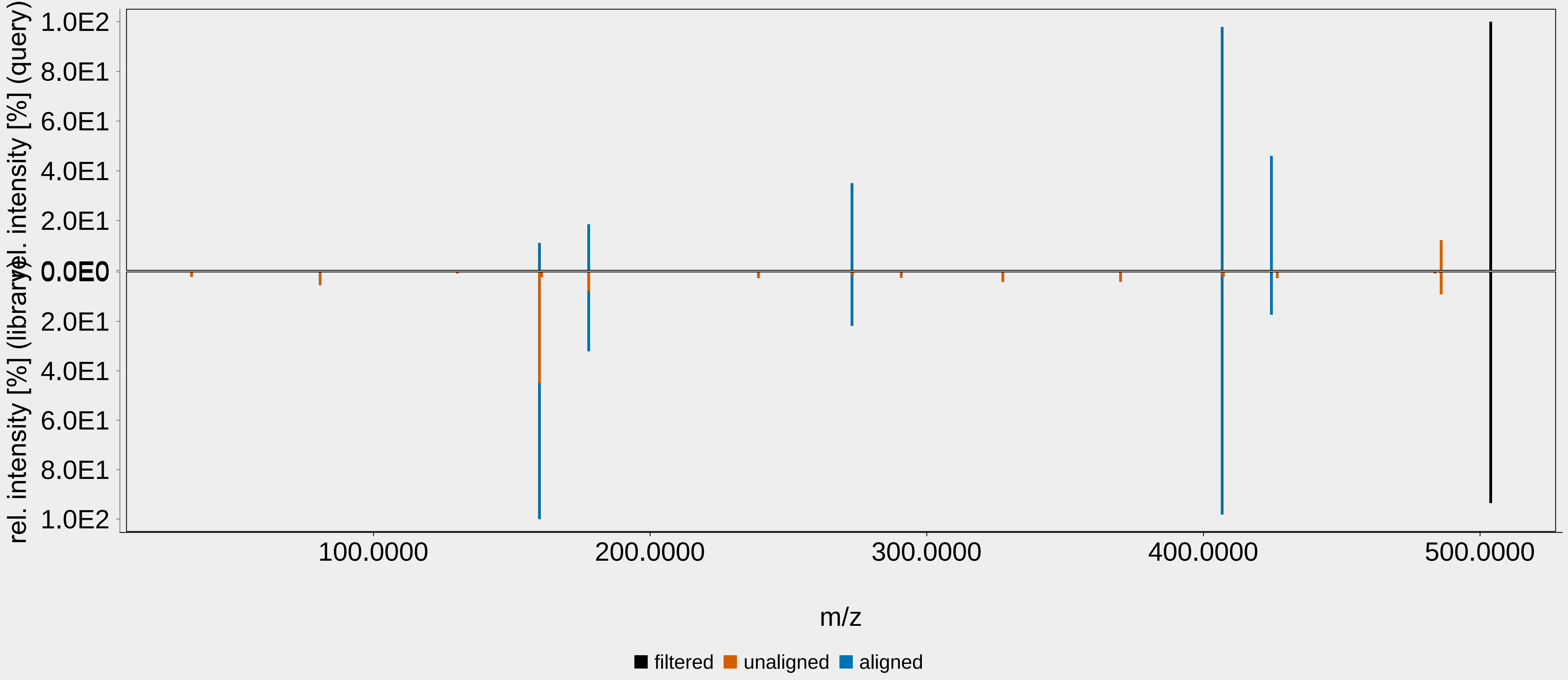

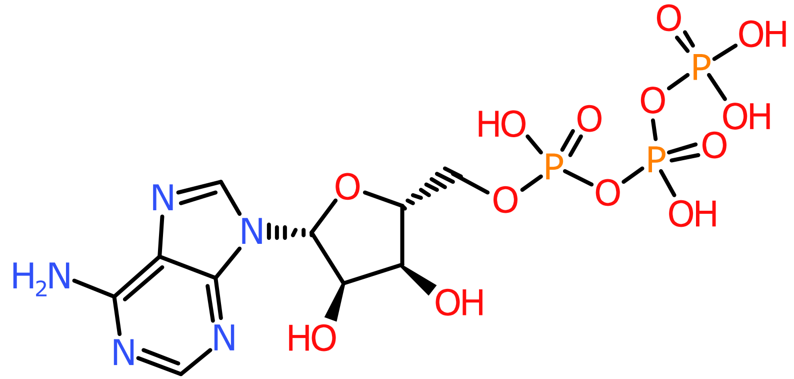

Compound information

Name: ADENOSINE 5'-TRIPHOSPHATE

Formula: C10H16N5O13P3

Ion type: [M-H]-

Precursor m/z: 505.9884695

Polarity: negative

InChI:

InChI key:

SMILES:

Instrument information

Instrument type: LC-ESI-QTOF

Instrument: Agilent qTOF 6545

Ms level: MS2

Collision energy: 20 eV

Database links

Other information

ADENOSINE 5'-TRIPHOSPHATE

0.743

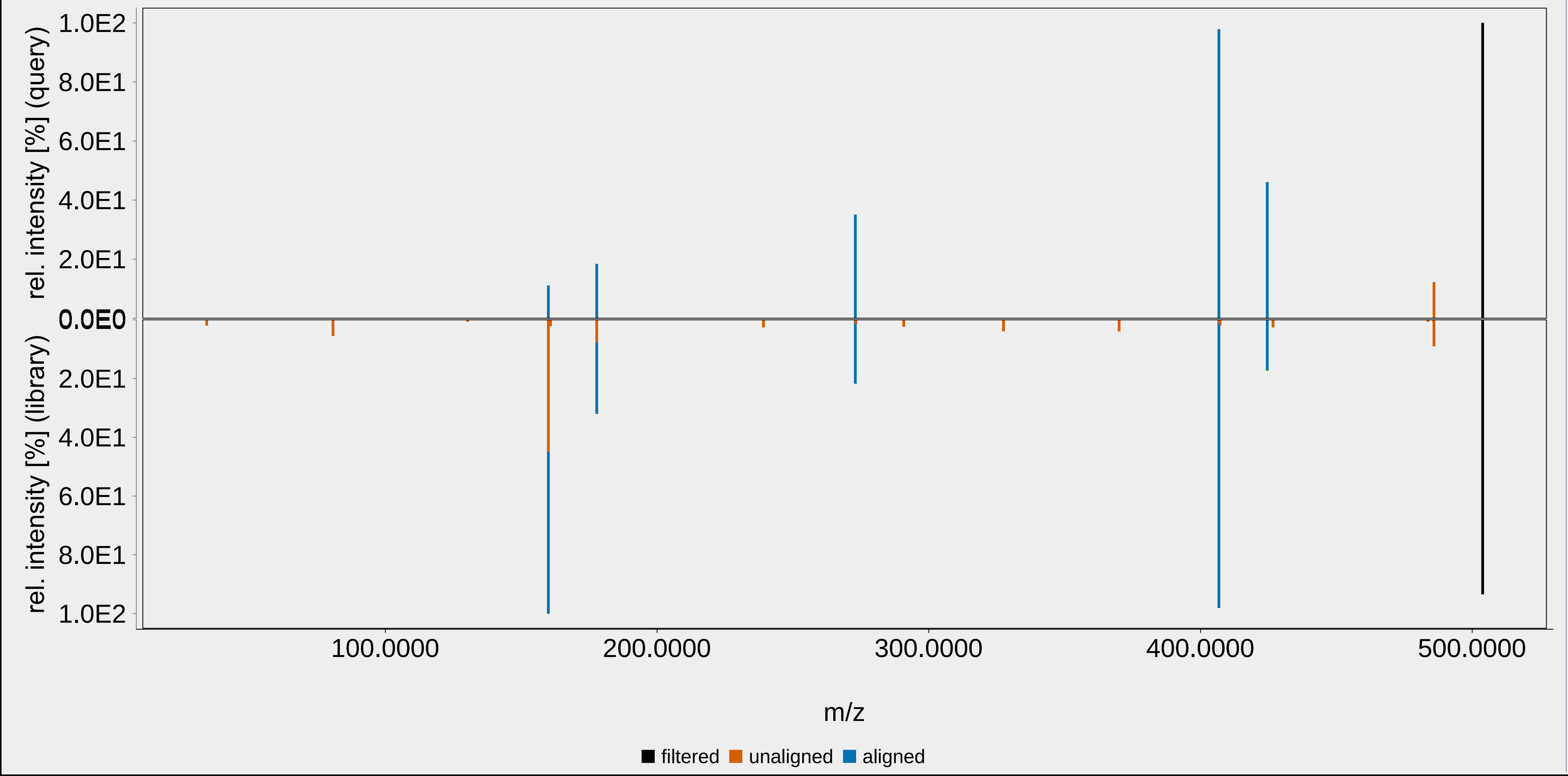

Compound information

Name: ADENOSINE 5'-TRIPHOSPHATE

Exact mass: 0.0

Ion type: M-H

Precursor m/z: 505.988

Charge: 1

Polarity: negative

InChI:

SMILES:

Instrument information

Instrument type: qTof

Ion source: ESI

Ms level: 2

Acquisition: isolated

Database links

Other information

Principal investigator: MoNA

Data collector: MoNA:MoNA034214

Entry ID: CCMSLIB00006685072

PA 18:1_18:2

0.890

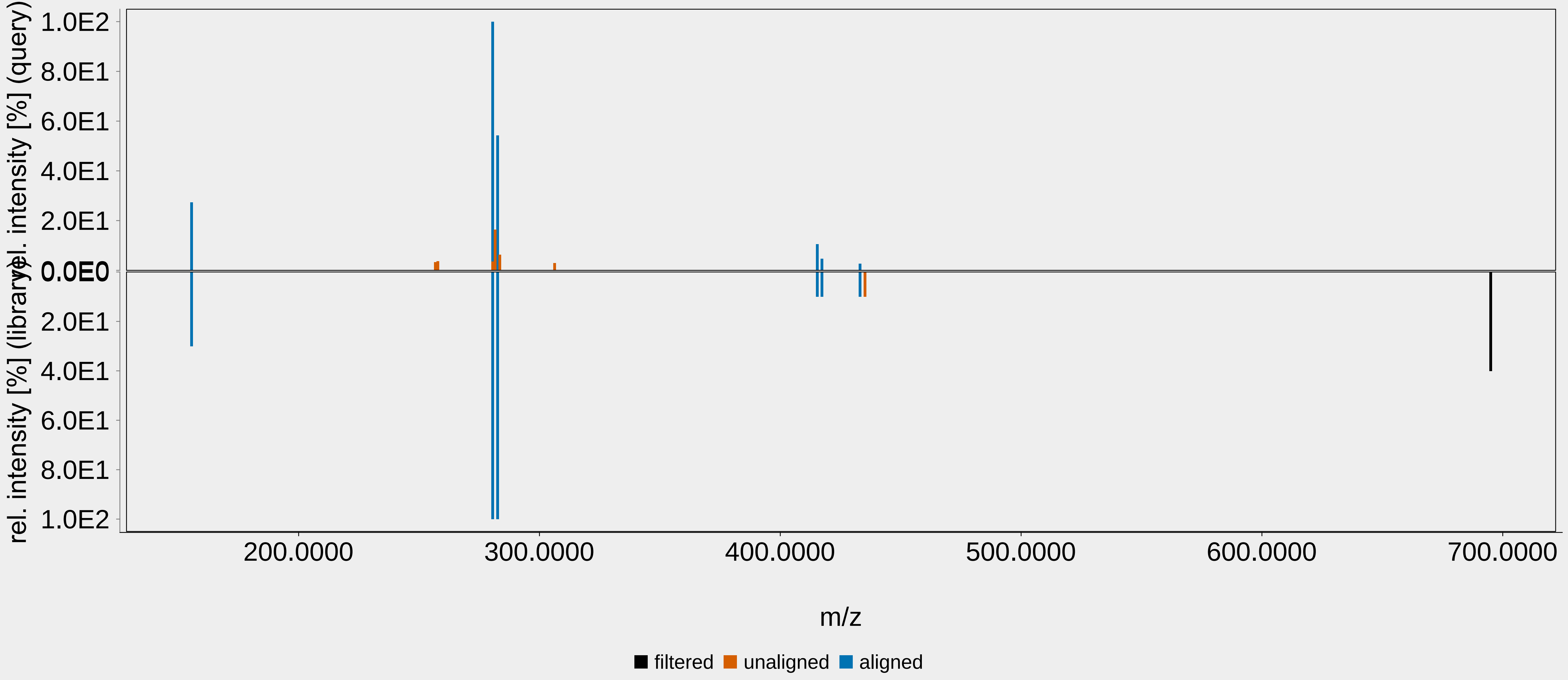

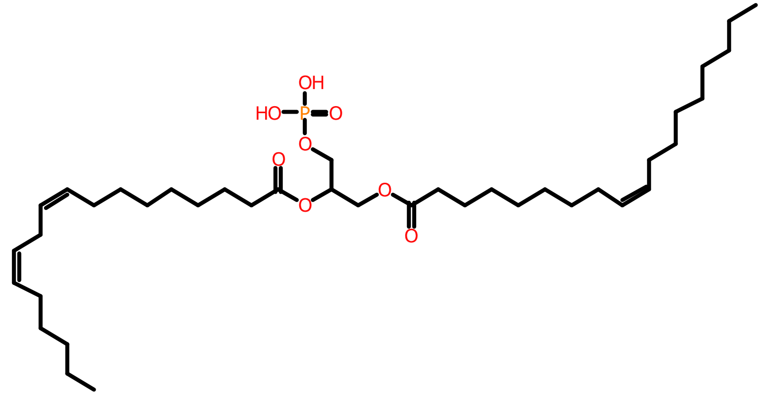

Compound information

Name: PA 18:1_18:2

Formula: C39H71O8P

Ion type: [M-H]-

Precursor m/z: 697.48138

Ccs: 255.37

InChI key:

SMILES:

Num peaks: 8

Instrument information

Database links

Other information

PE(18:1/18:1); [M-H]- C41H77N1O8P1 **0.941**

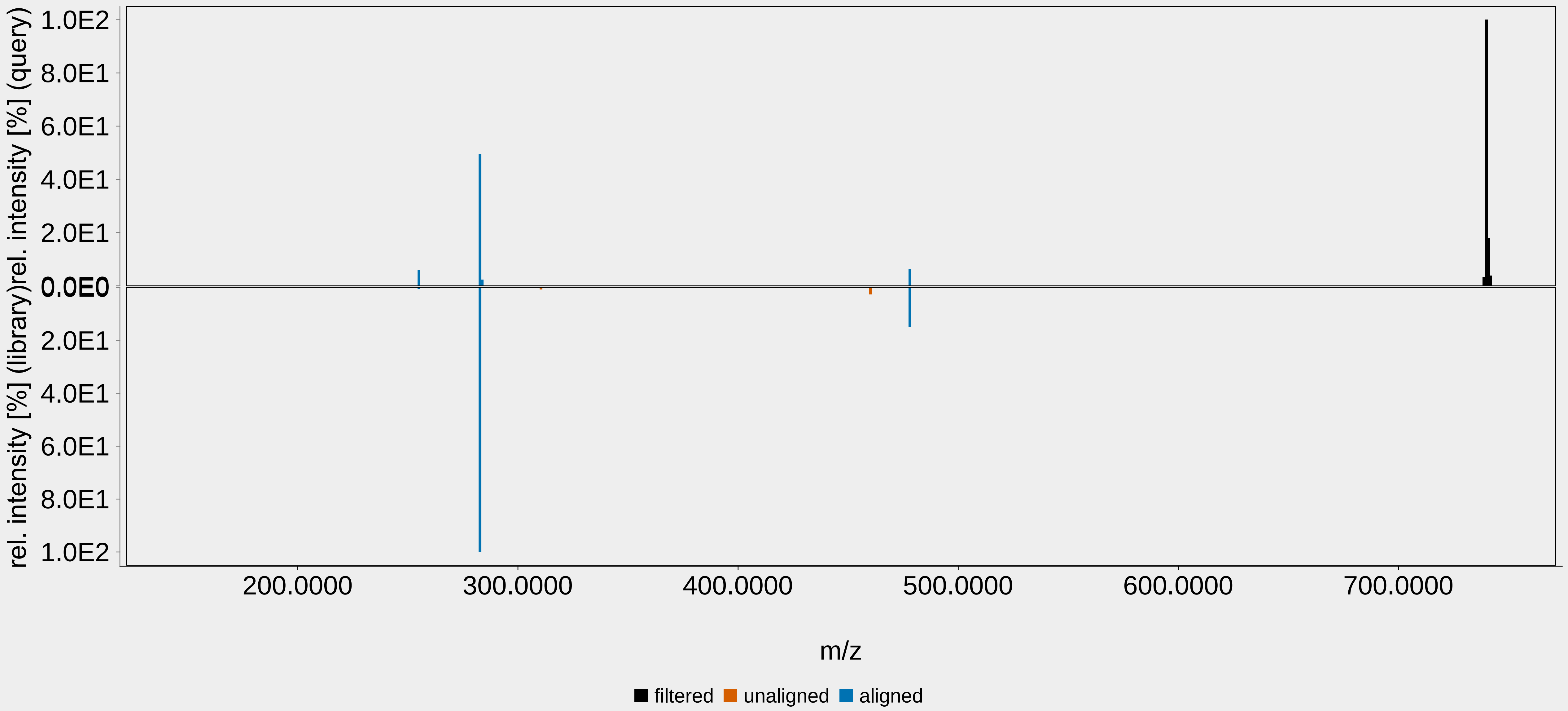

Compound information

Name: PE(18:1/18:1); [M-H]- C41H77N1O8P1

Exact mass: 0.0

Ion type: M-H

Precursor m/z: 742.539

Charge: 1

Polarity: Negative

Instrument information

Instrument type: CID; Lumos

Ion source: LC-ESI

Ms level: 2

Acquisition: Commercial

Database links

Other information

Principal investigator: Thomas Metz

Data collector: Thomas Metz

Entry ID: CCMSLIB00010059182

Spectral Match to 1-Stearoyl-2-linoleoyl-sn-glycero-3-phosphoethanolamine from NIST14

0.957

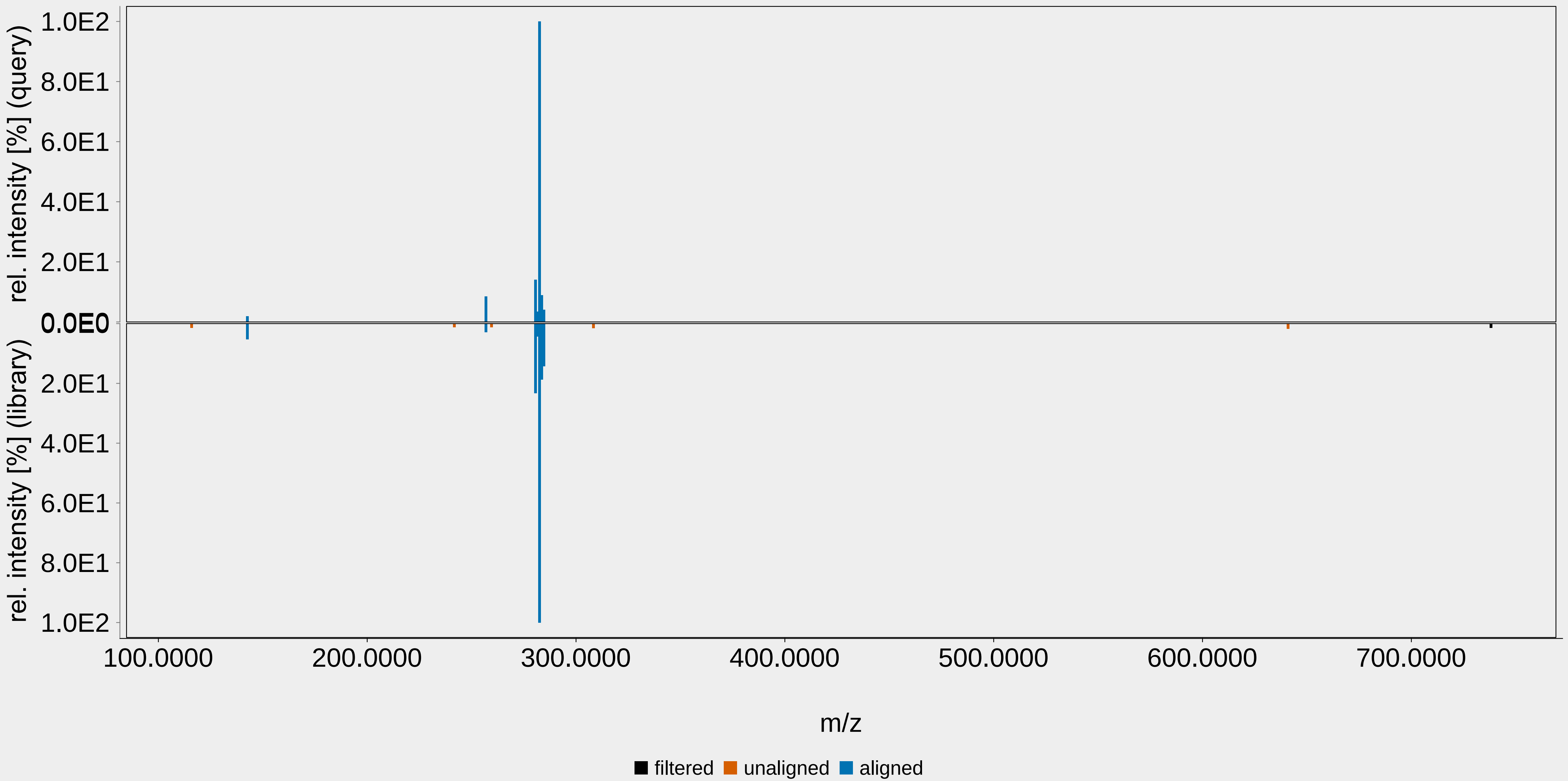

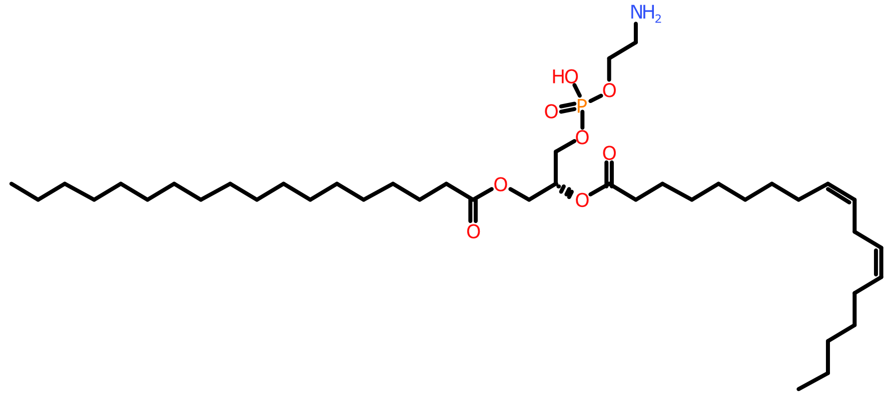

Compound information

Name: Spectral Match to

Exact mass: 743.547

Ion type: M-H

Precursor m/z: 742.541

Charge: 1

Polarity: Negative

InChI:

SMILES:

Instrument information

Instrument type: qTof

Ion source: ESI

Ms level: 2

Acquisition: Isolated

Database links

CAS: 7266537

Other information

Principal investigator: Data from Wiemann

Data collector: Data deposited by pwiemann

Entry ID: CCMSLIB00003136842

PS 18:1_24:1

0.831

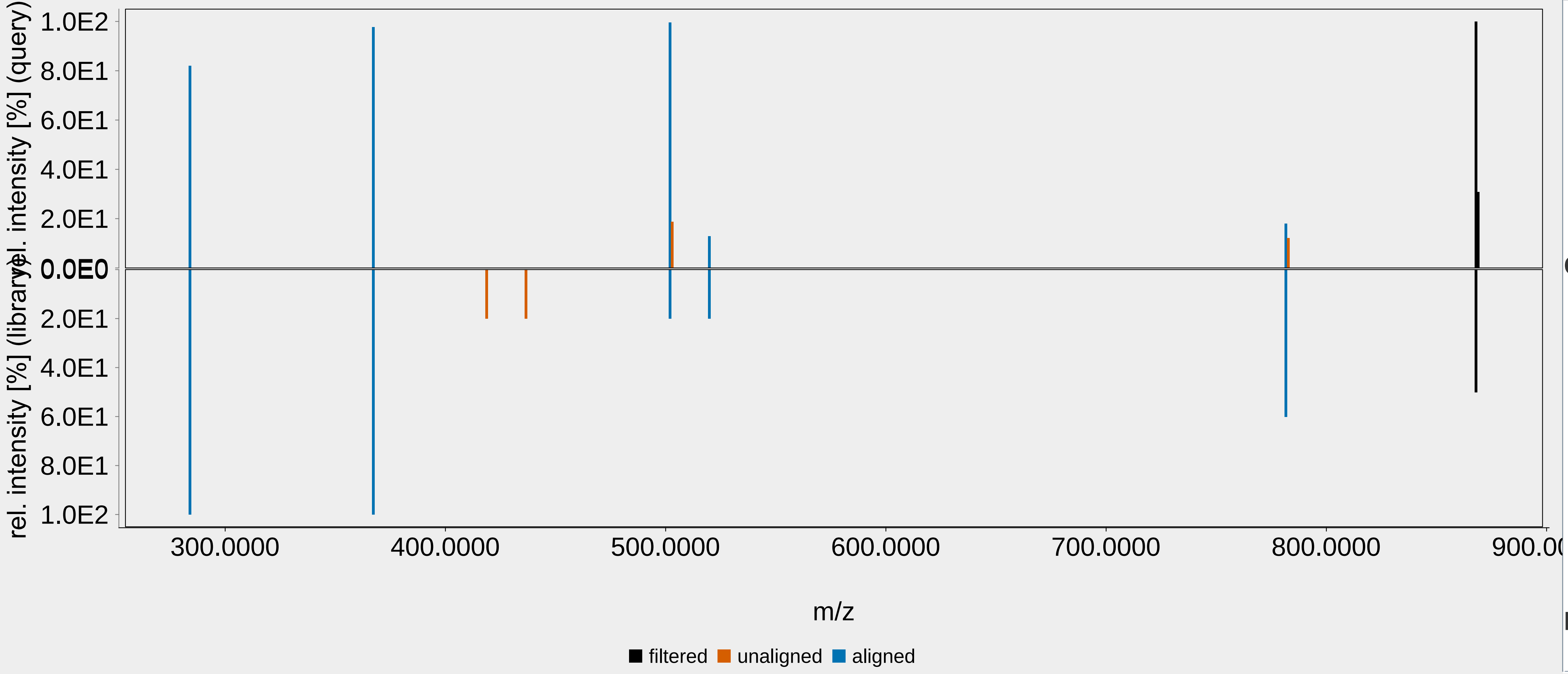

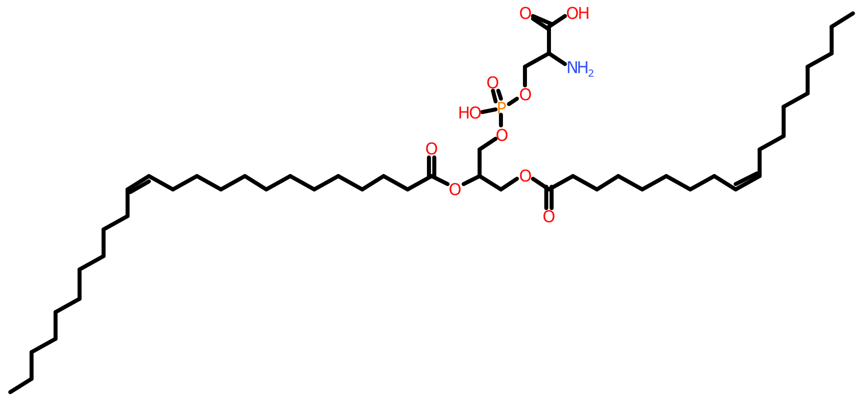

Compound information

Name: PS 18:1_24:1

Formula: C48H90NO10P

Ion type: [M-H]-

Precursor m/z: 870.62296

Ccs: 292.75

InChI key:

SMILES:

Num peaks: 8

Instrument information

Database links

Other information

PE(P-18:0/18:1); [M-H]- C41H79N1O7P1

0.784

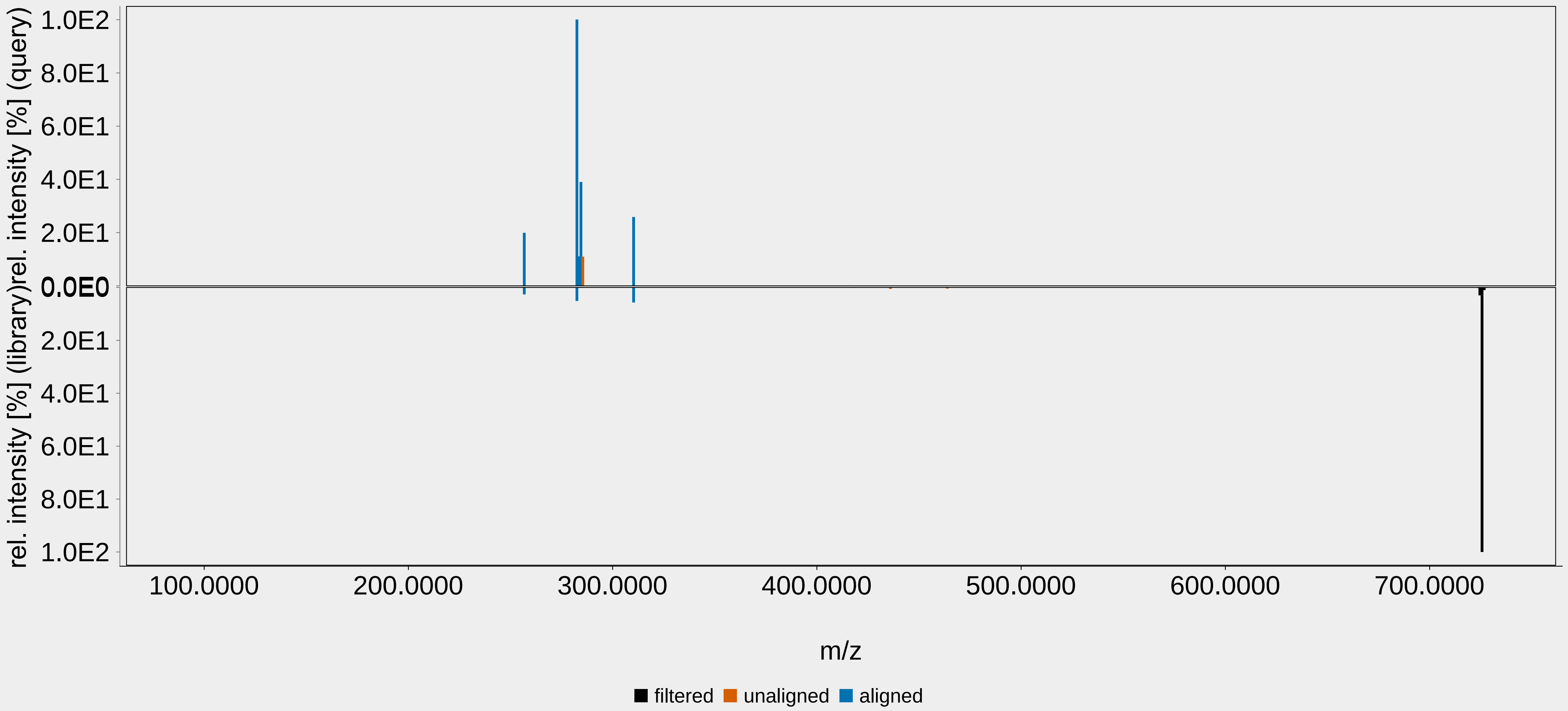

Compound information

Name: PE(P-18:0/18:1); [M-H]-

Exact mass: 0.0

Ion type: M-H

Precursor m/z: 728.559

Charge: 1

Polarity: Negative

Instrument information

Instrument type: HCD; Velos

Ion source: LC-ESI

Ms level: 2

Acquisition: Commercial

Database links

Other information

Principal investigator: Thomas Metz

Data collector: Thomas Metz

Entry ID: CCMSLIB00010072263

PE(P-18:0/18:1); [M-H]- C41H79N1O7P1 **0.780**

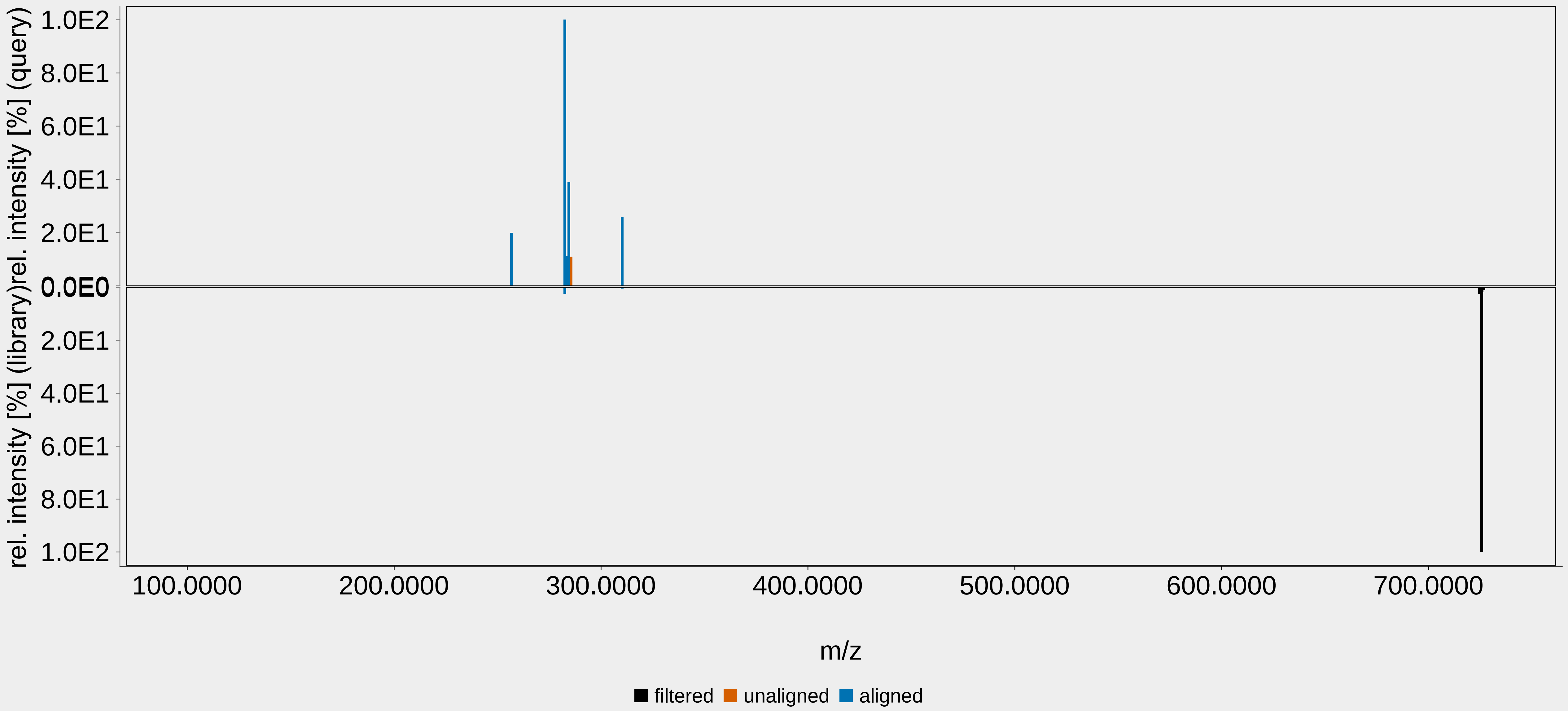

Compound information

Name: PE(P-18:0/18:1); [M-H]-

Exact mass: 0.0

Ion type: M-H

Precursor m/z: 728.559

Charge: 1

Polarity: Negative

Instrument information

Instrument type: HCD; Velos

Ion source: LC-ESI

Ms level: 2

Acquisition: Commercial

Database links

Other information

Principal investigator: Thomas Metz

Data collector: Thomas Metz

Entry ID: CCMSLIB00010068572

Spectral Match to 1-Palmitoyl-2-hydroxy-sn-glycero-3-phosphoethanolamine from NIST14

0.944

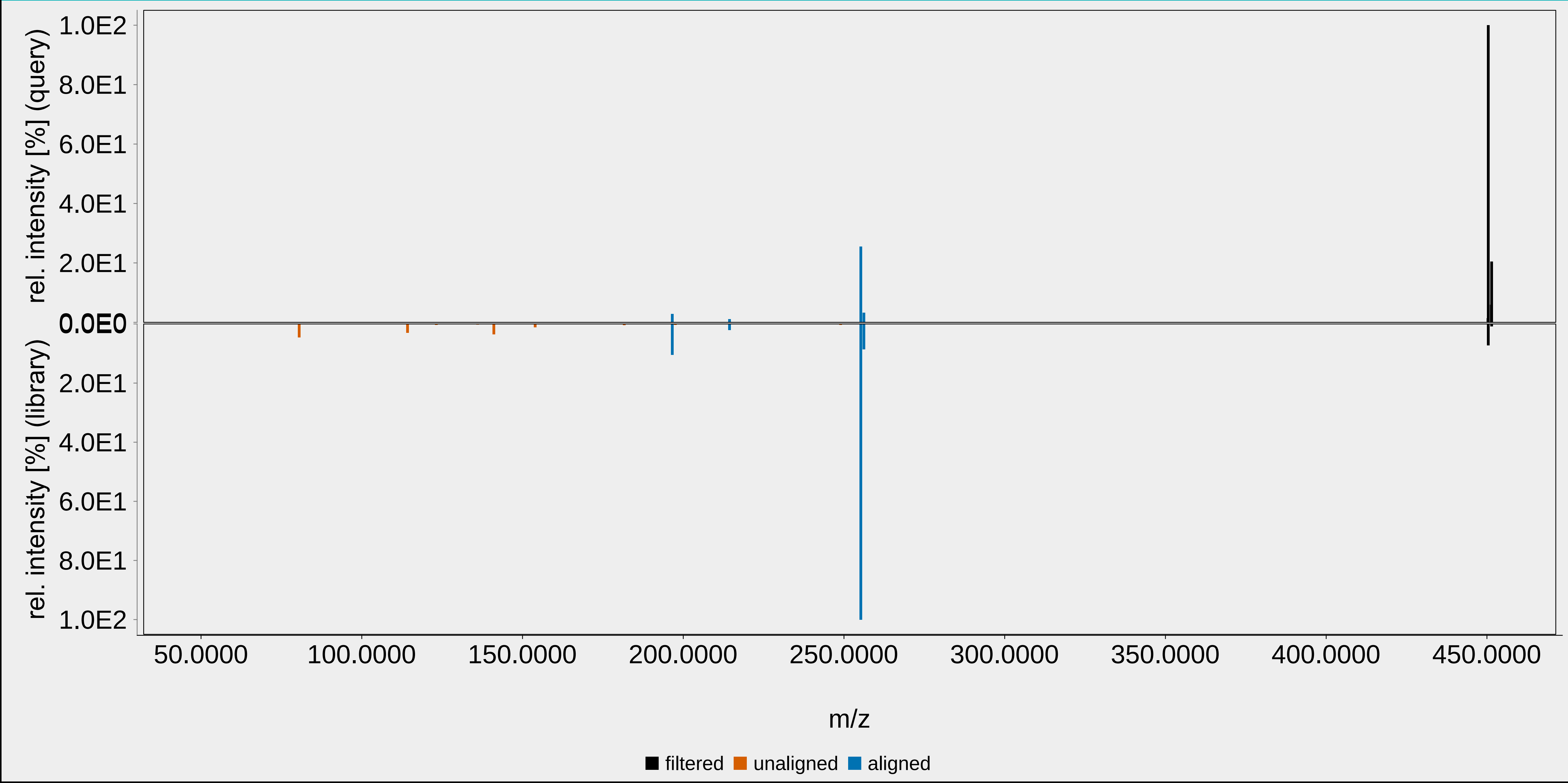

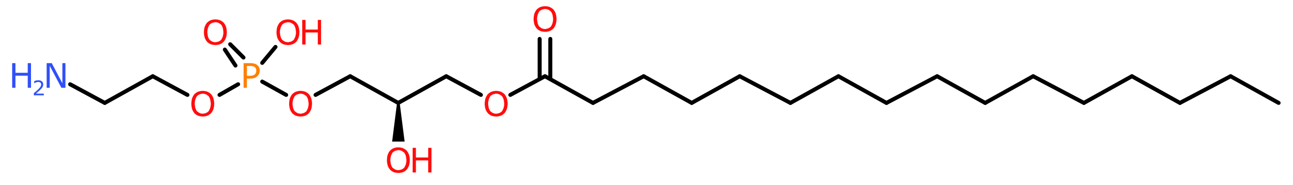

Compound information

Name: Spectral Match to

Exact mass: 453.285

Ion type: M-H

Precursor m/z: 452.279

Charge: 1

Polarity: Negative

InChI:

SMILES:

Instrument information

Instrument type: qTof

Ion source: ESI

Ms level: 2

Acquisition: Isolated

Database links

CAS: 53862354

Other information

Principal investigator: Data from Kevin Bush

Data collector: Data deposited by fevargas

Entry ID: CCMSLIB00003137341

Spectral Match to 1-Palmitoyl-2-hydroxy-sn-glycero-3-phosphoethanolamine from NIST14

0.953

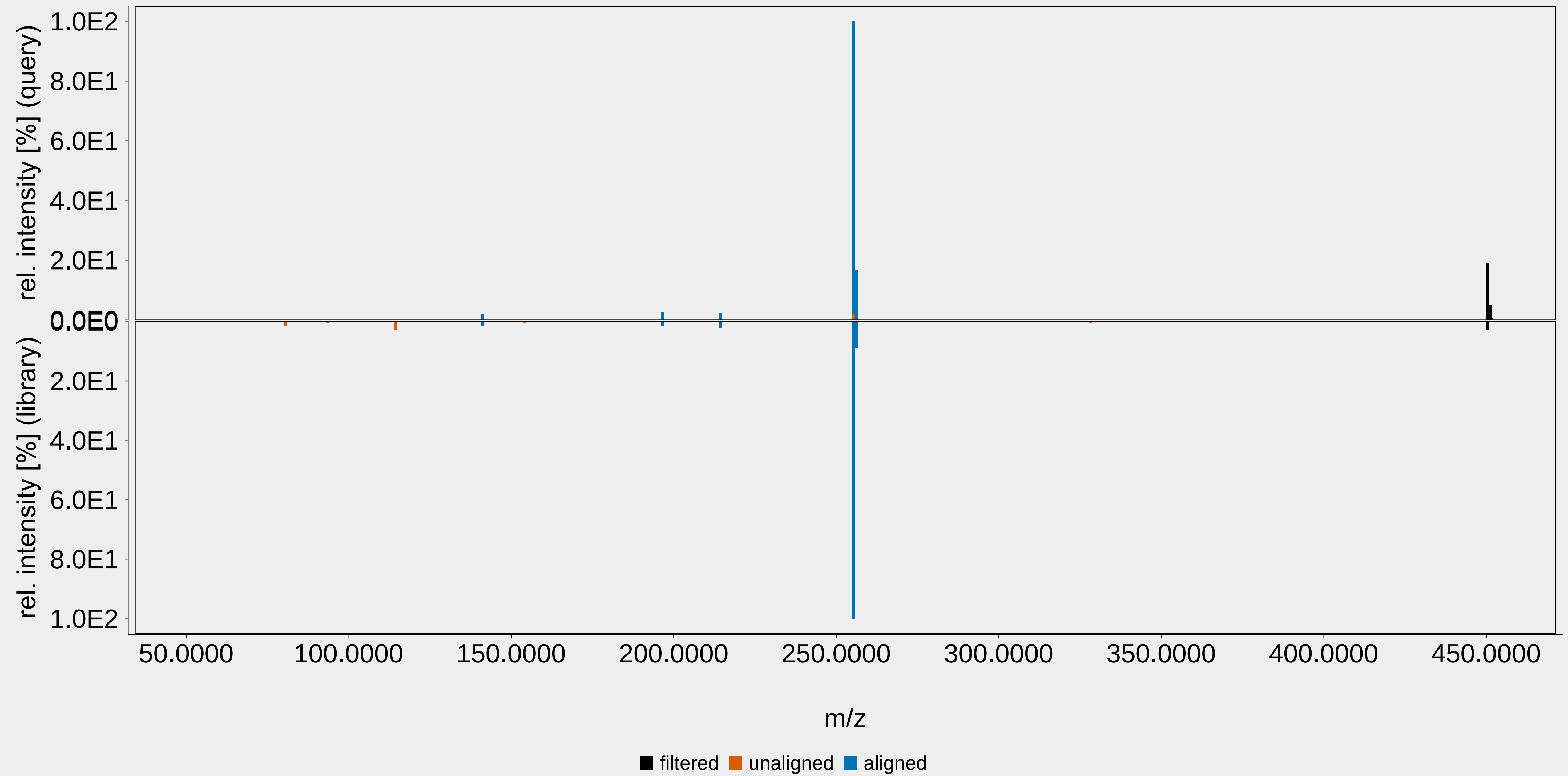

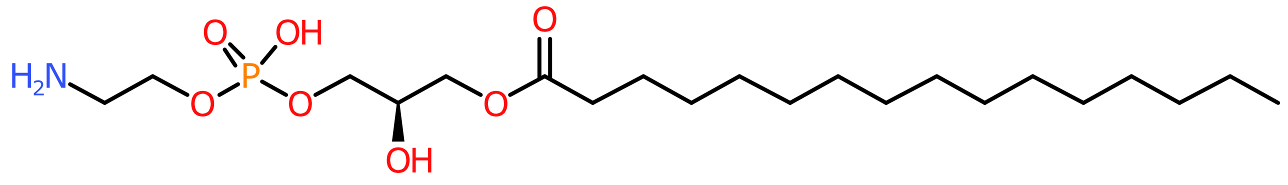

Compound information

Name: Spectral Match to

Exact mass: 453.285

Ion type: M-H

Precursor m/z: 452.279

Charge: 1

Polarity: Negative

InChI:

SMILES:

Instrument information

Instrument type: qTof

Ion source: ESI

Ms level: 2

Acquisition: Isolated

Database links

CAS: 53862354

Other information

Principal investigator: Data from Kevin Bush

Data collector: Data deposited by fevargas

Entry ID: CCMSLIB00003137726

PA 18:1_22:1

0.808

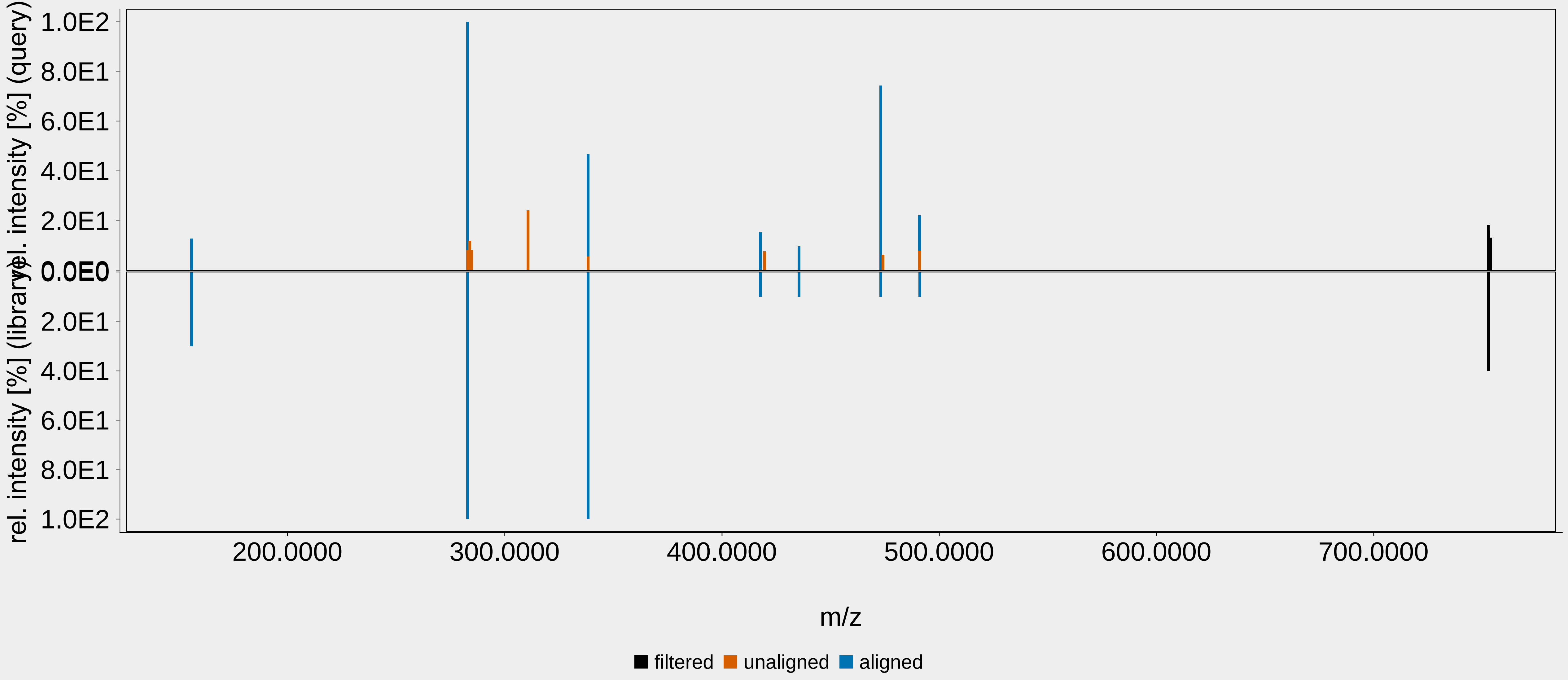

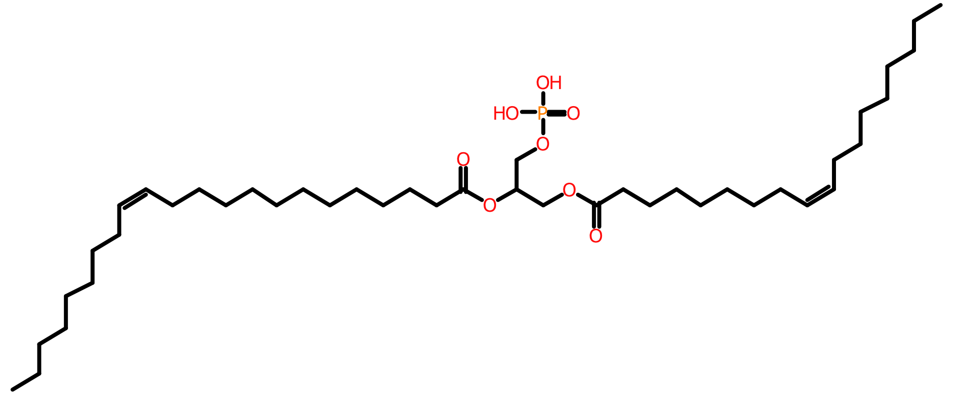

Compound information

Name: PA 18:1_22:1

Formula: C43H81O8P

Ion type: [M-H]-

Precursor m/z: 755.55963

Ccs: 271.45

InChI key:

SMILES:

Num peaks: 8

Instrument information

Database links

Other information

PE(20:1/0:0); [M-H]- C25H49N1O7P1 **0.739**

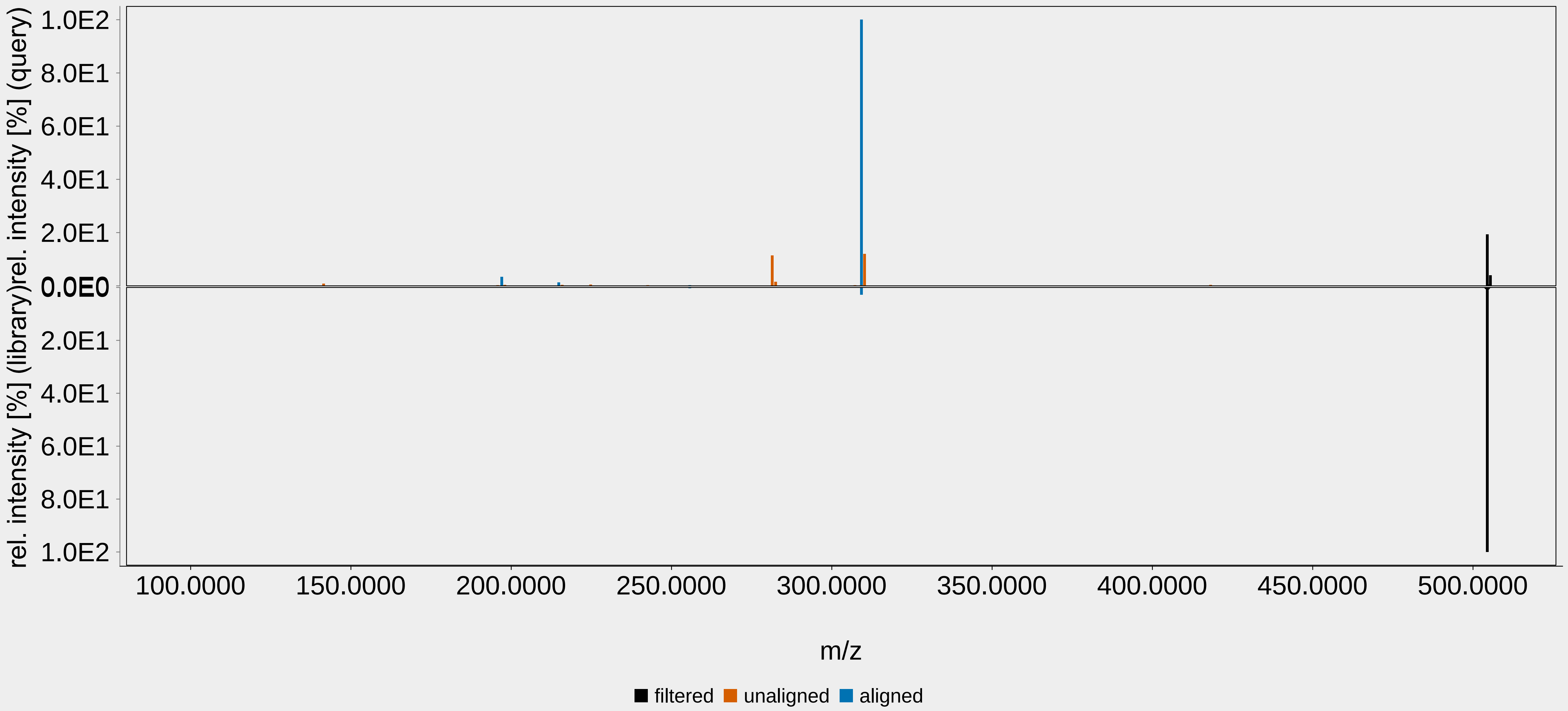

Compound information

Name: PE(20:1/0:0); [M-H]- C25H49N1O7P1

Exact mass: 0.0

Ion type: M-H

Precursor m/z: 506.325

Charge: 1

Polarity: Negative

Instrument information

Instrument type: HCD; Velos

Ion source: LC-ESI

Ms level: 2

Acquisition: Commercial

Database links

Other information

Principal investigator: Thomas Metz

Data collector: Thomas Metz

Entry ID: CCMSLIB00010086765

PE(20:1/0:0); [M-H]- C25H49N1O7P1 **0.757**

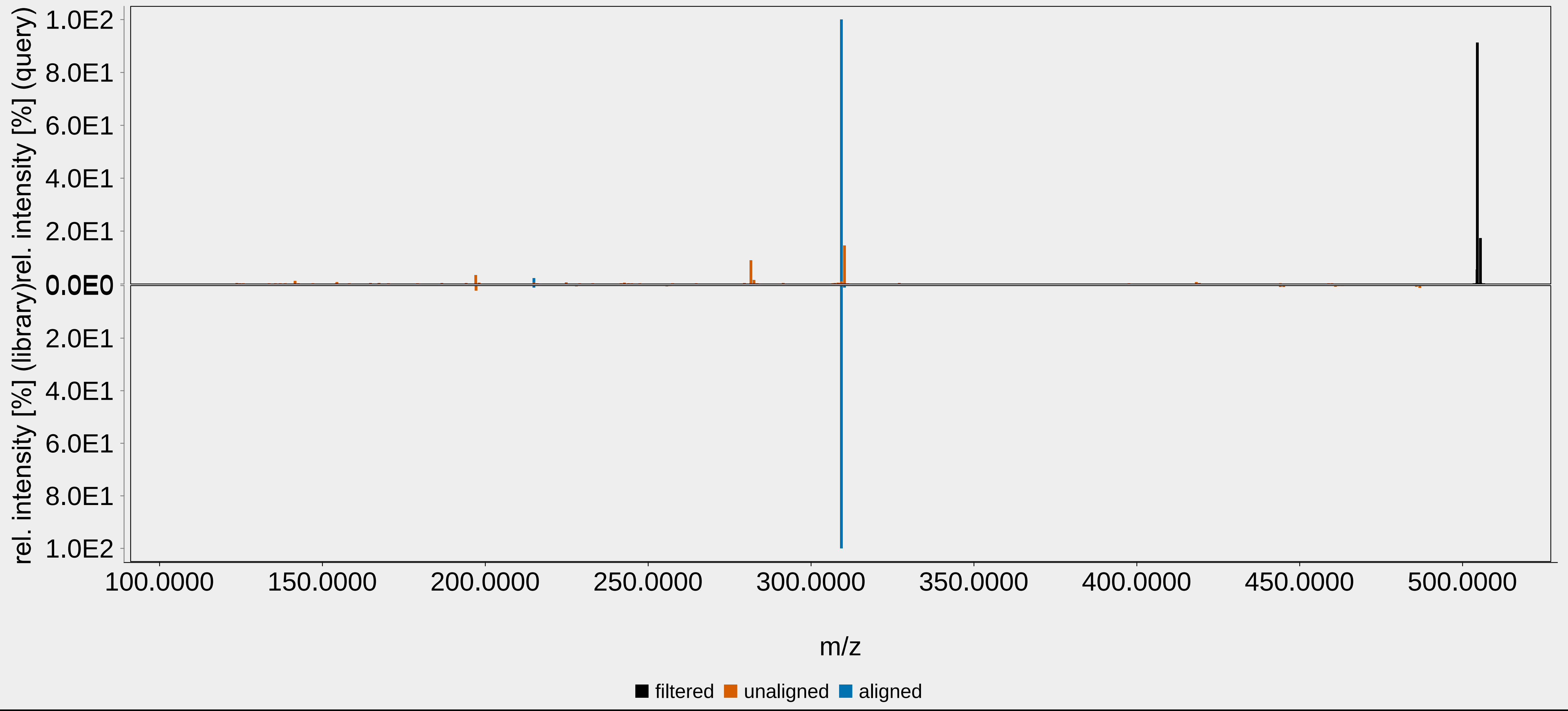

Compound information

Name: PE(20:1/0:0); [M-H]- C25H49N1O7P1

Exact mass: 0.0

Ion type: M-H

Precursor m/z: 506.325

Charge: 1

Polarity: Negative

Instrument information

Instrument type: CID; Velos

Ion source: LC-ESI

Ms level: 2

Acquisition: Commercial

Database links

Other information

Principal investigator: Thomas Metz

Data collector: Thomas Metz

Entry ID: CCMSLIB00010063977

Canrenone

0.939

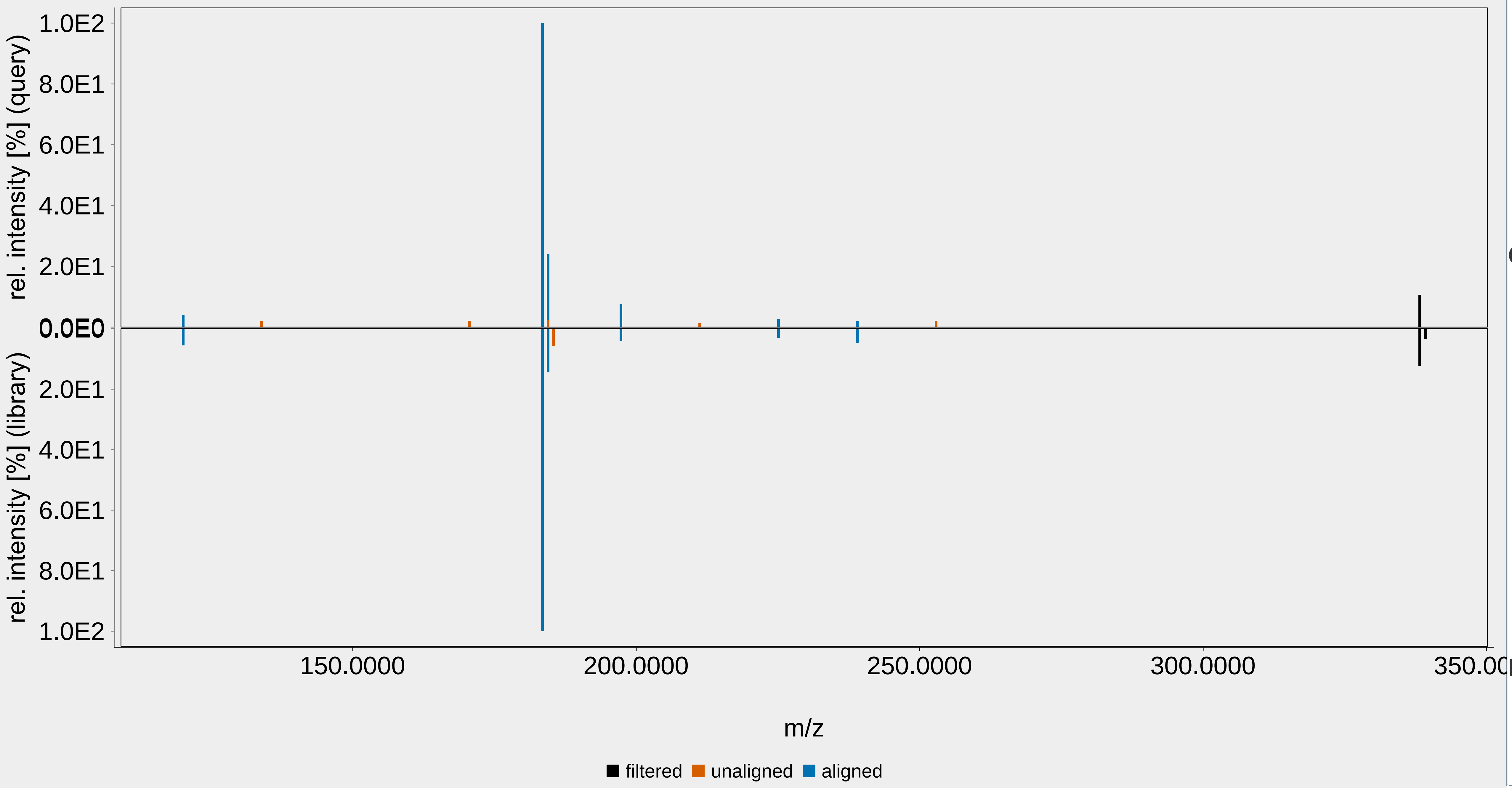

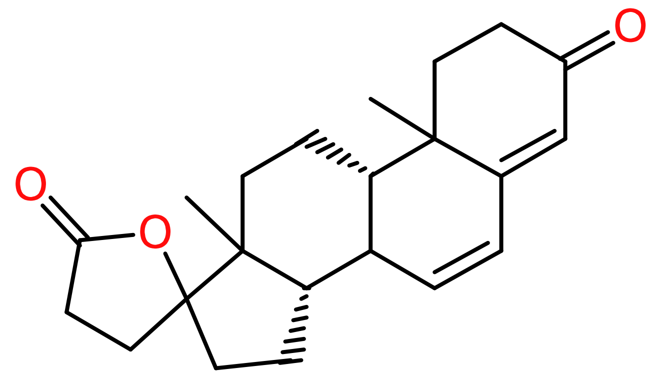

Compound information

Name: Canrenone

Formula: C22H28O3

Exact mass: 340.2038448

Ion type: [M-H]-

Precursor m/z: 339.1966

Polarity: NEGATIVE

InChI:

InChI key:

SMILES:

Num peaks: 9

Instrument information

Instrument type: LC-ESI-QTOF

Instrument: Bruker maXis Impact

Ms level: MS2

Collision energy: 40 eV

Database links

Other information

Entry ID: MSBNK-Athens_Univ-AU239460

Comment: Parent=339.1966

Canrenone, (9S,14S)-10,13-dimethylspiro[2,8,9,11,12,14,15,16-octahydro-1H-cyclopenta[a]phenanthrene-17,5`-oxolane]-2`,3-dione

0.939

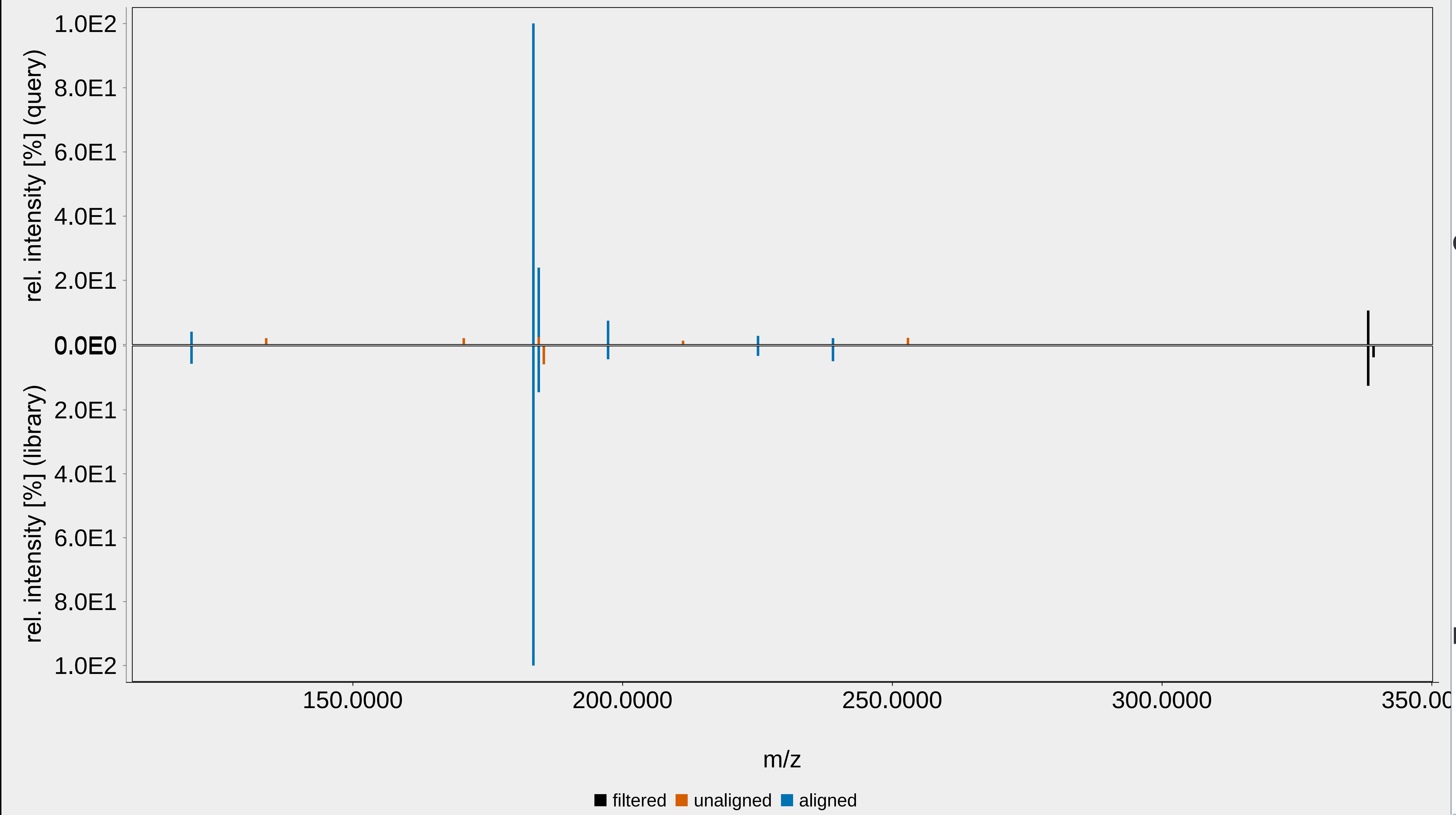

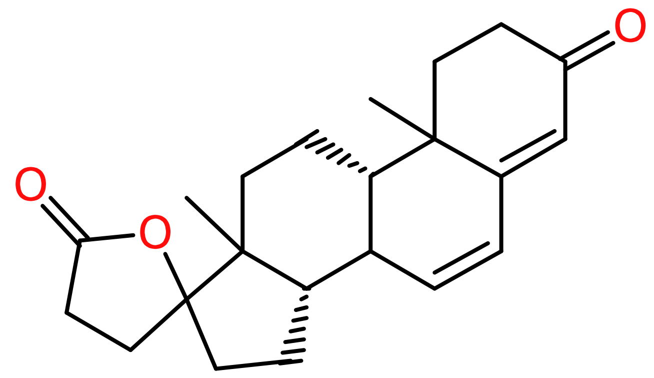

Compound information

Name: Canrenone,

Formula: C22H28O3

Mol. weight: 340.2038448

Exact mass: 340.2038448

Ion type: [M-H]-

Precursor m/z: 339.1966

Polarity: negative

InChI:

InChI key:

SMILES:

Instrument information

Instrument type: LC-ESI-QTOF

Instrument: Bruker maXis Impact

Ion source: ESI

Resolution: 35000

Ms level: MS2

Collision energy: 40 eV

Database links

Pubchem: 24728626

MoNA ID: AU239460

Chemspider: 21112020

CAS: 976-71-6

Other information

Principal investigator: Nikiforos Alygizakis,

Data collector: Nikiforos Alygizakis, Katerina

Guanosine diphosphate - 30.0 eV

0.876

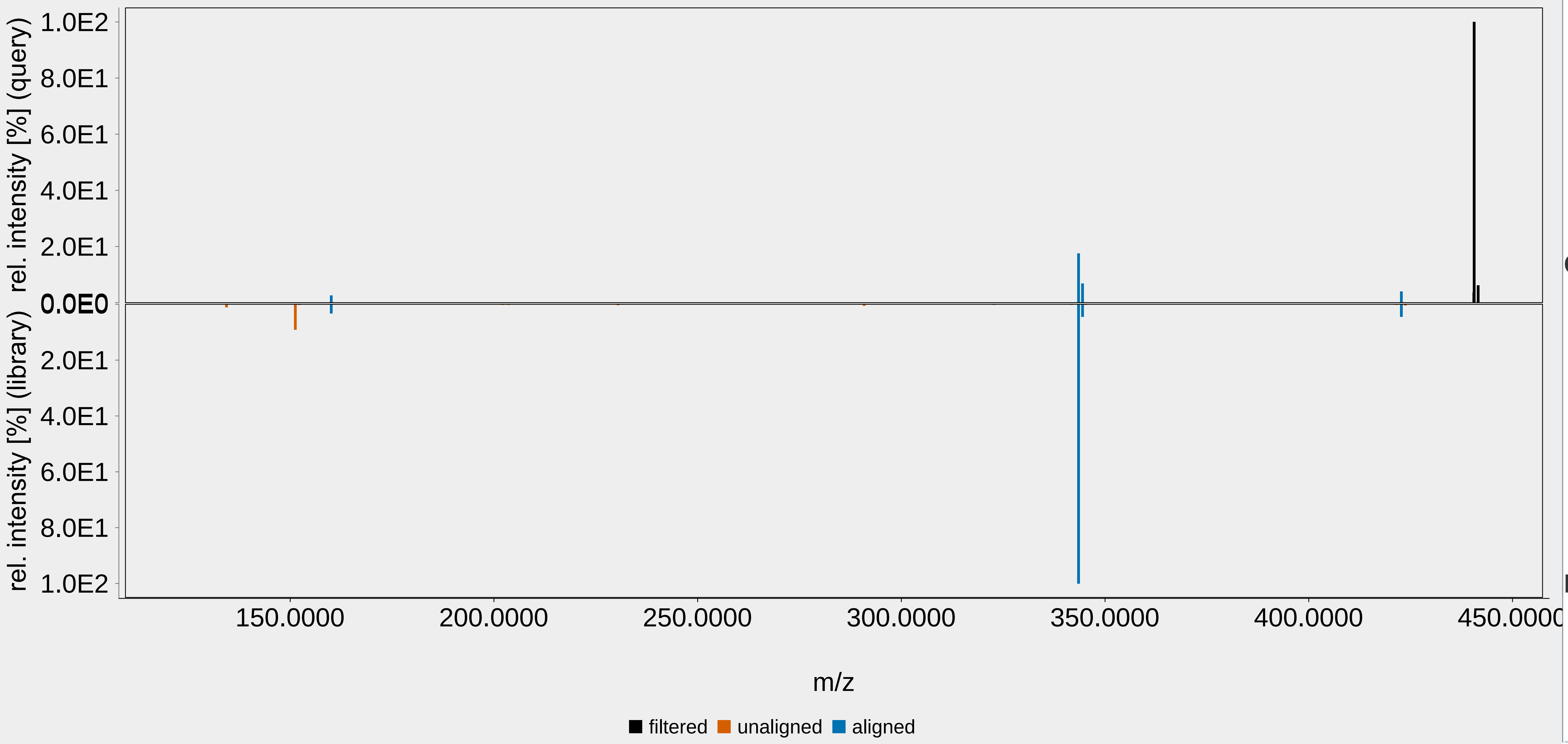

Compound information

Name: Guanosine diphosphate - 30.0 eV

Exact mass: 0.0

Ion type: M-H

Precursor m/z: 442.017

Charge: 1

Polarity: Negative

SMILES:

Instrument information

Instrument type: Orbitrap

Ion source: ESI

Ms level: 2

Acquisition: Isolated

Database links

CAS:

Other information

Principal investigator: PI

Data collector: Data Collector

Entry ID: CCMSLIB00006123856

Guanosine diphosphate - 30.0 eV

0.880

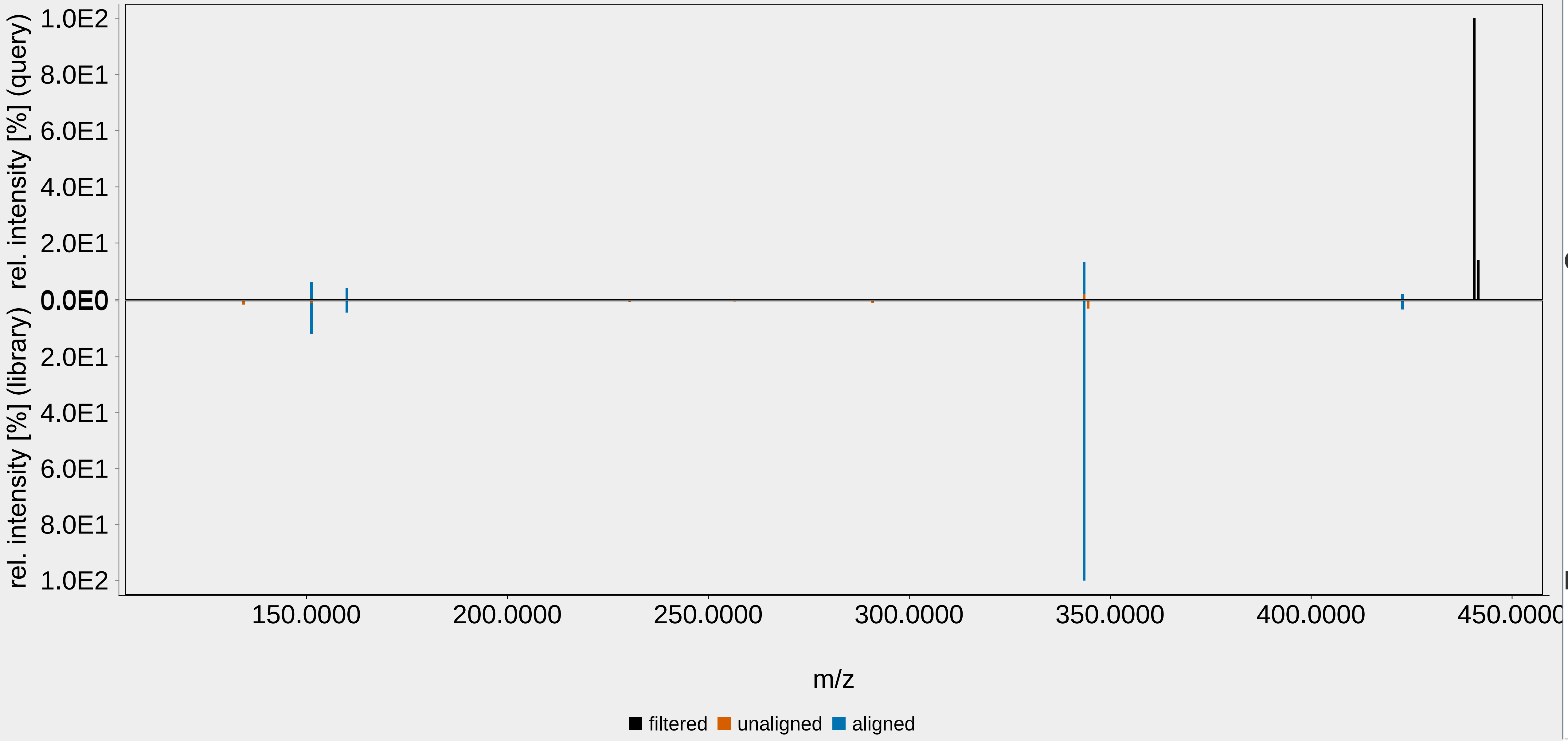

Compound information

Name: Guanosine diphosphate - 30.0 eV

Exact mass: 0.0

Ion type: M-H

Precursor m/z: 442.017

Charge: 1

Polarity: Negative

SMILES:

Instrument information

Instrument type: Orbitrap

Ion source: ESI

Ms level: 2

Acquisition: Isolated

Database links

CAS:

Other information

Principal investigator: PI

Data collector: Data Collector

Entry ID: CCMSLIB00006123838

PS 23:0_18:1

0.841

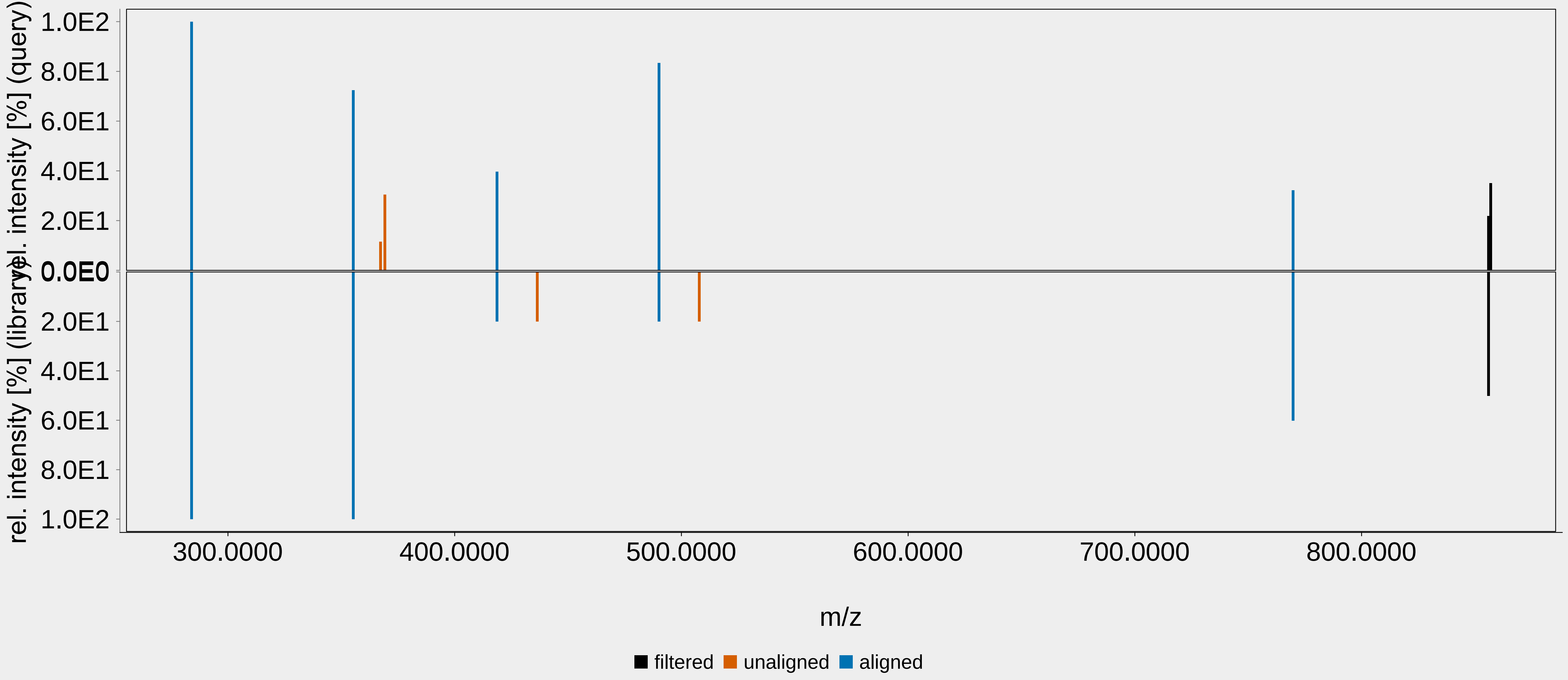

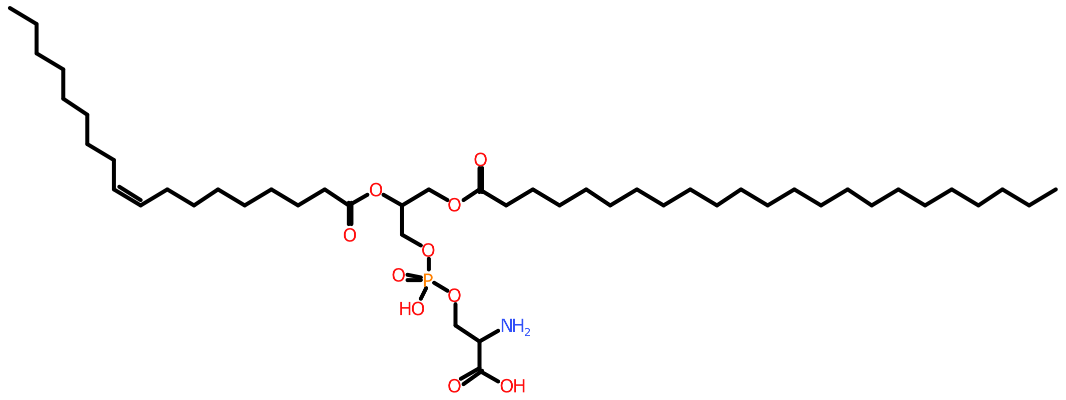

Compound information

Name: PS 23:0_18:1

Formula: C47H90NO10P

Ion type: [M-H]-

Precursor m/z: 858.62296

Ccs: 292.27

InChI key:

SMILES:

Num peaks: 8

Instrument information

Database links

Other information

PA 18:1_18:1 0.876

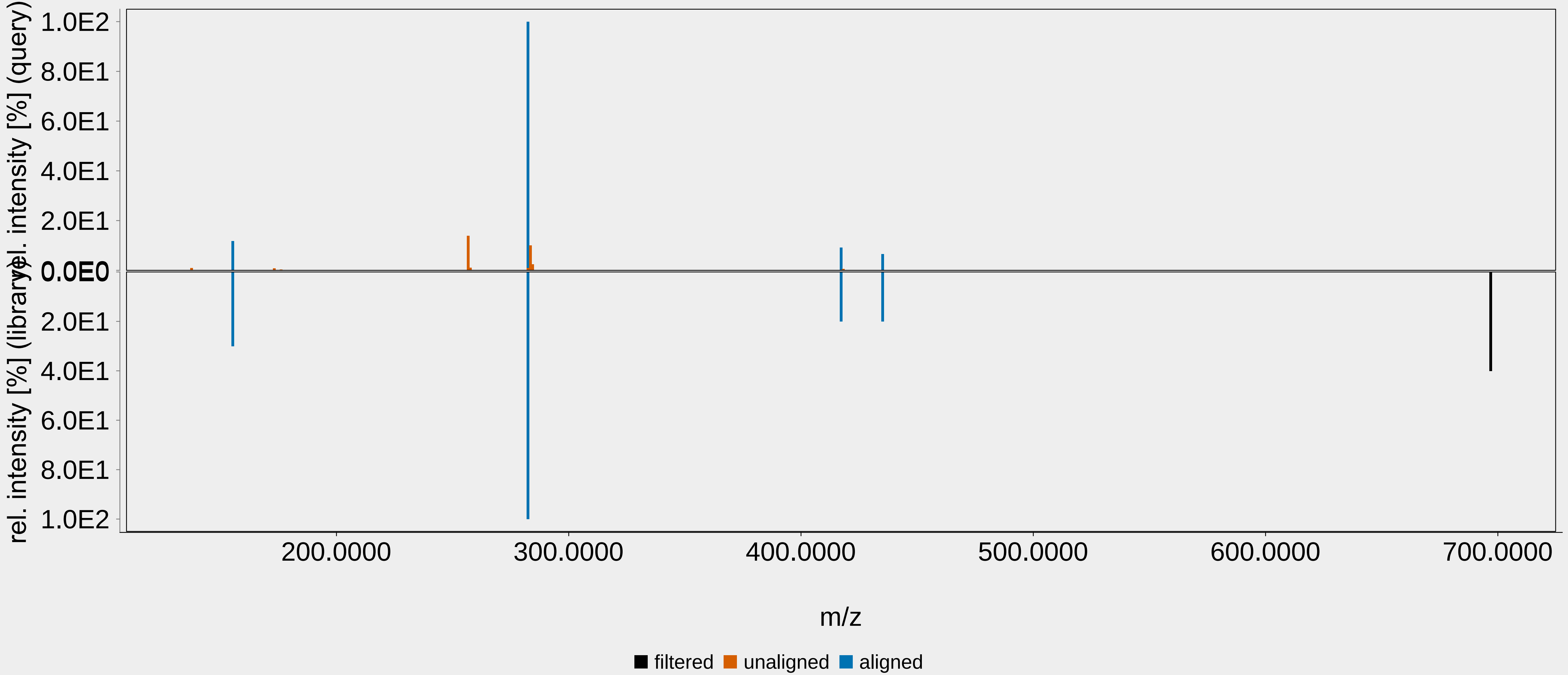

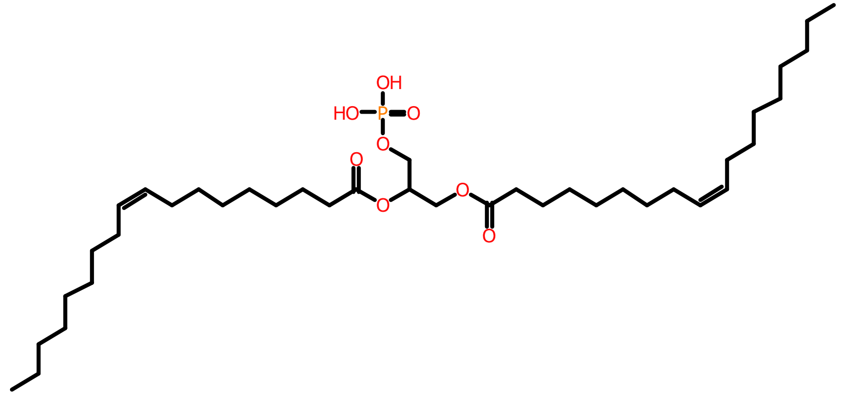

Compound information

Name: PA 18:1_18:1

Formula: C39H73O8P

Ion type: [M-H]-

Precursor m/z: 699.49703

Ccs: 257.54

InChI key: MHUWZNTUIIFHAS-CLFAGFIQNA-I

SMILES:

Num peaks: 5

Instrument information

Database links

Other information

Glutathione - 30.0 eV

0.876

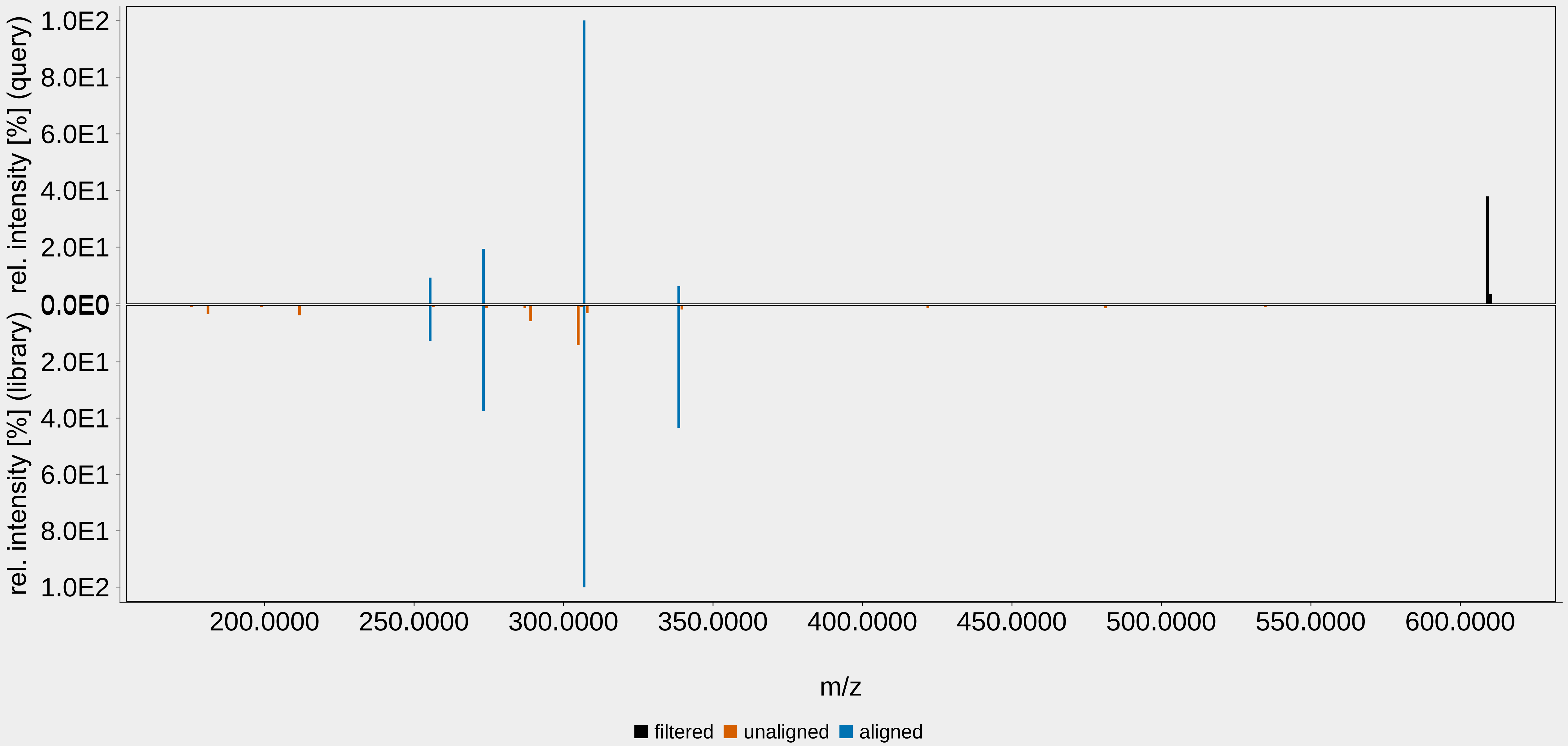

Compound information

Name: Glutathione - 30.0 eV

Exact mass: 0.0

Ion type: M-H

Precursor m/z: 611.145

Charge: 1

Polarity: Negative

SMILES:

Instrument information

Instrument type: Orbitrap

Ion source: ESI

Ms level: 2

Acquisition: Isolated

Database links

CAS:

Other information

Principal investigator: PI

Data collector: Data Collector

Entry ID: CCMSLIB00006123948

Glutathione (oxidized)

0.918

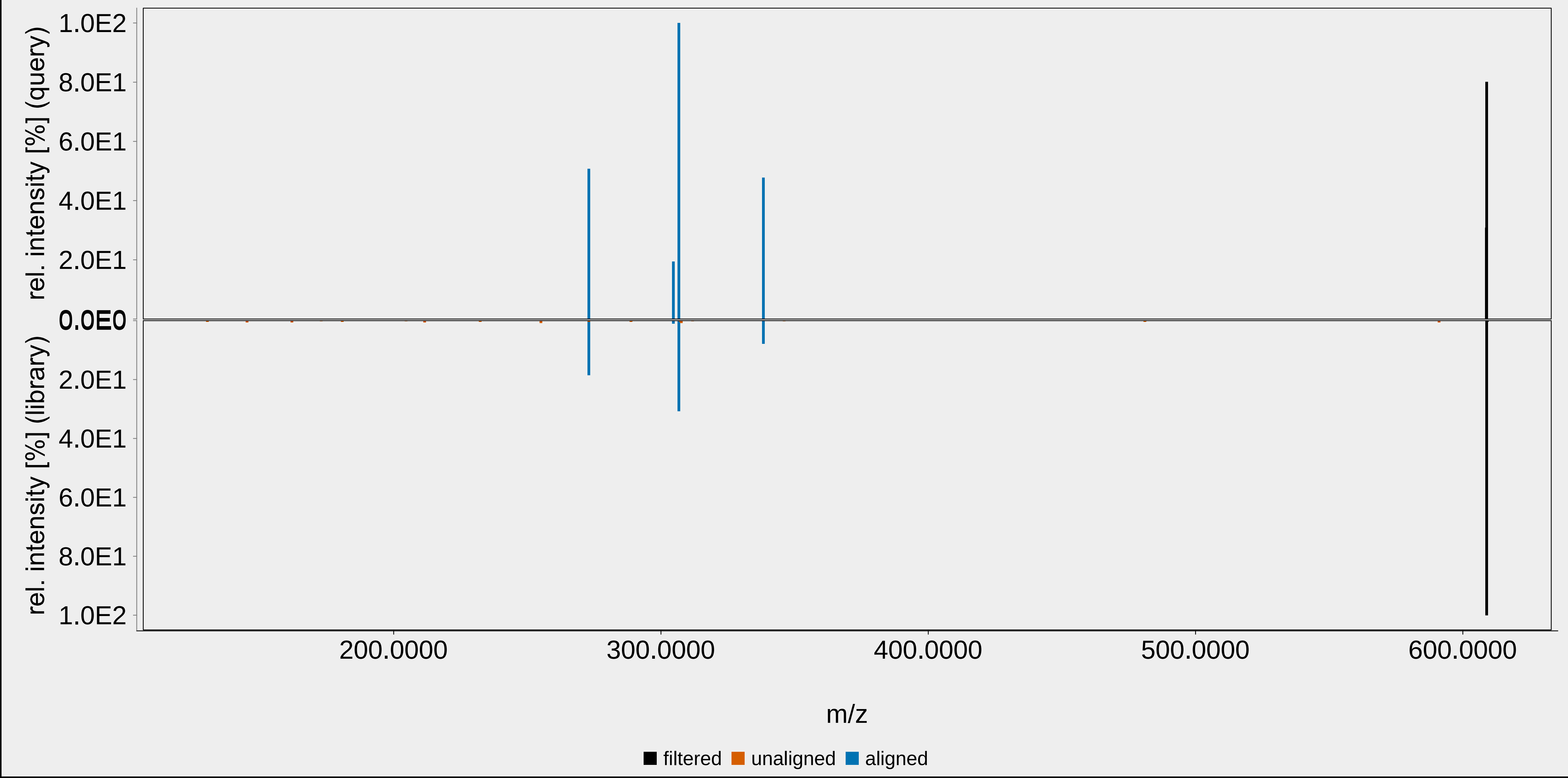

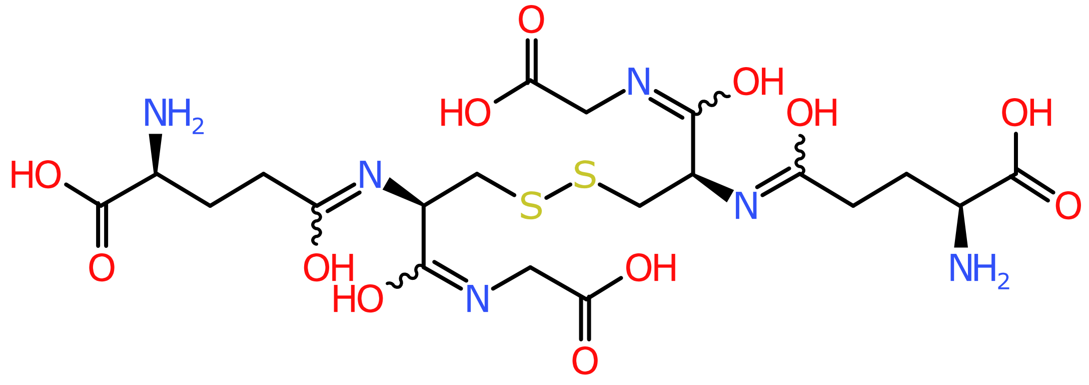

Compound information

Name: Glutathione (oxidized)

Formula: C20H32N6O12S2

Ion type: [M-H]-

Precursor m/z: 611.142

RT: 9.794383

Polarity: negative

InChI:

InChI key:

SMILES:

Instrument information

Instrument type: LC-ESI-QTOF

Instrument: SCIEX TripleTOF 6600

Ms level: MS2

Collision energy: 35 eV

Database links

Other information

Principal investigator: Michael Sa and Megan

Data collector: Michael Sa and Megan

PE(18:1/20:1); [M-H]- C43H81N1O8P1 **0.917**

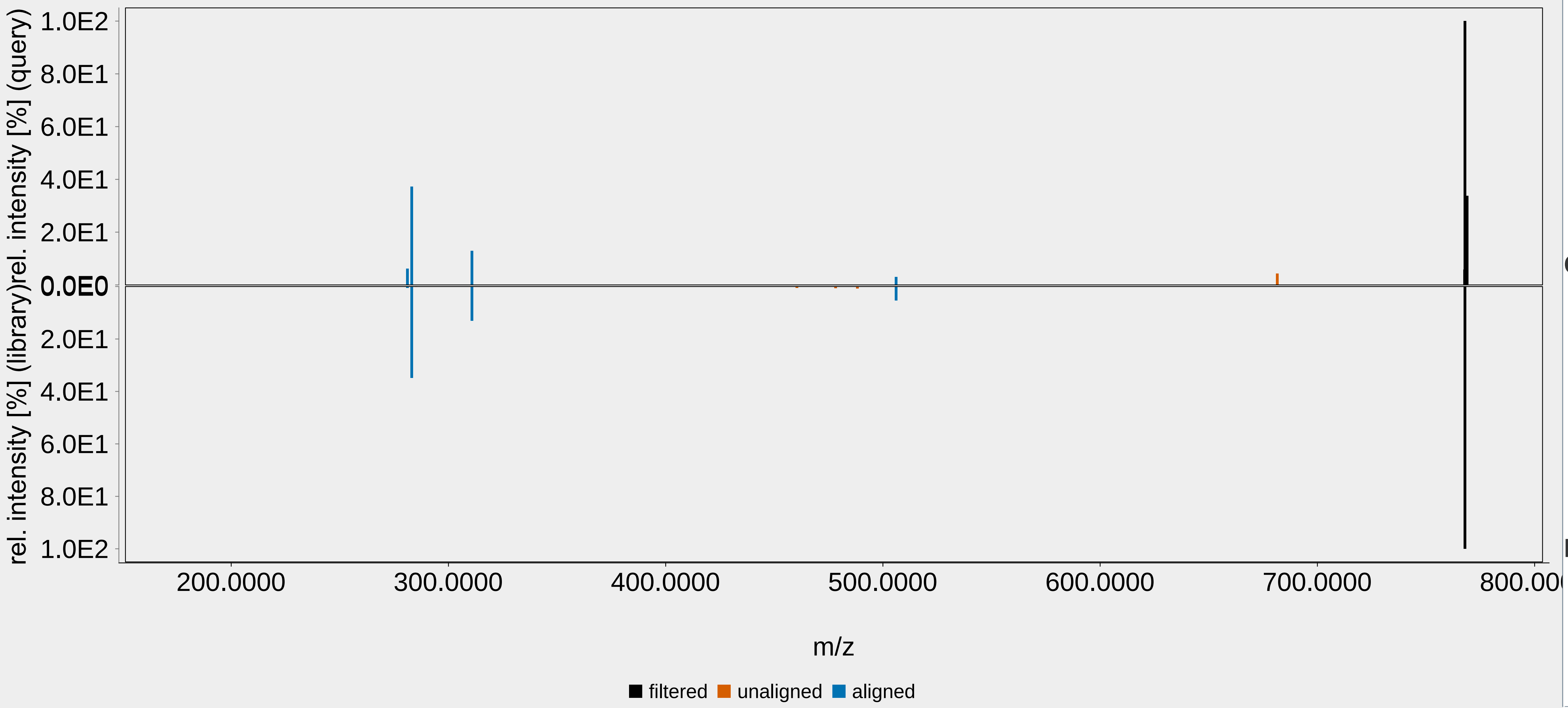

Compound information

Name: PE(18:1/20:1); [M-H]- C43H81N1O8P1

Exact mass: 0.0

Ion type: M-H

Precursor m/z: 770.57

Charge: 1

Polarity: Negative

Instrument information

Instrument type: CID; Lumos

Ion source: LC-ESI

Ms level: 2

Acquisition: Commercial

Database links

Other information

Principal investigator: Thomas Metz

Data collector: Thomas Metz

Entry ID: CCMSLIB00010059697

PE(18:1/20:1); [M-H]- C43H81N1O8P1 0.918

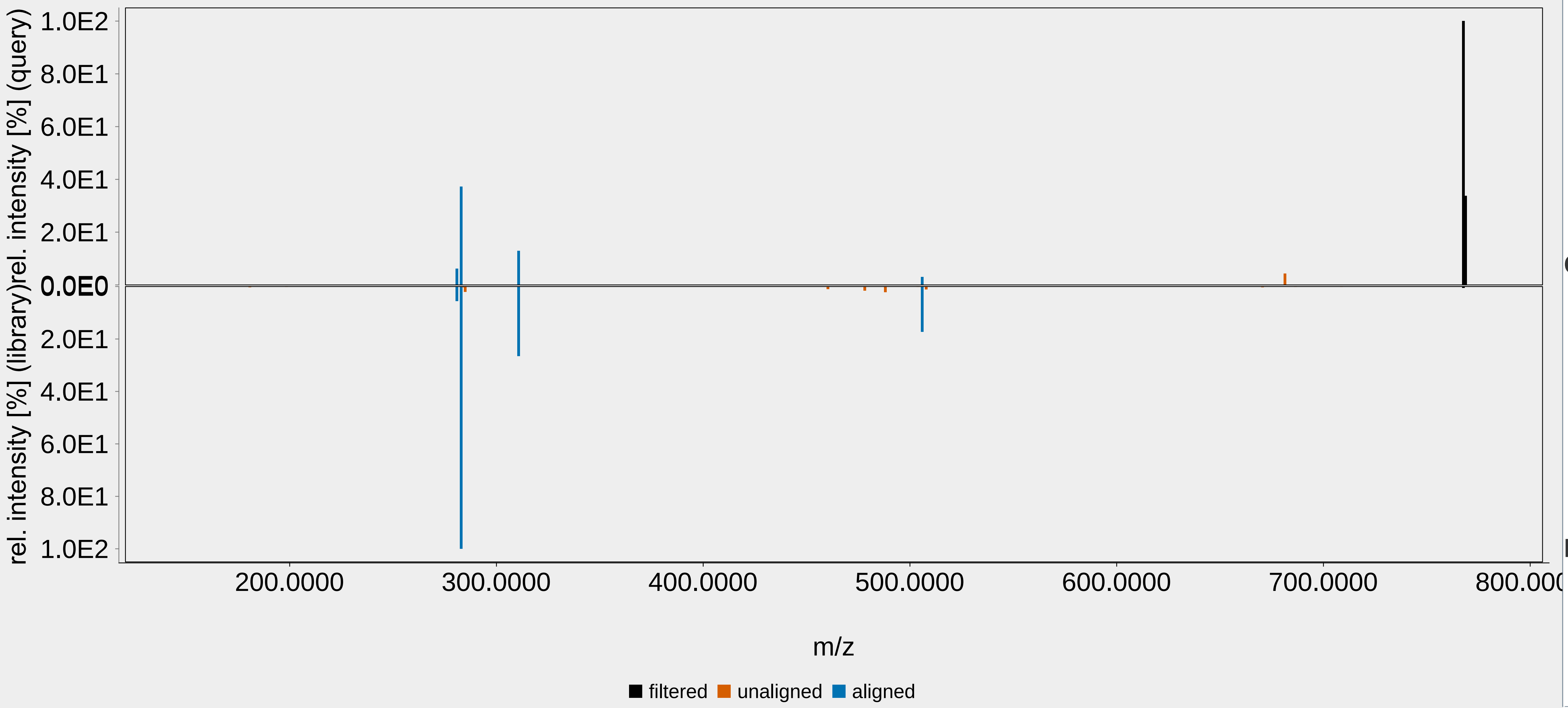

Compound information

Name: PE(18:1/20:1); [M-H]- C43H81N1O8P1

Exact mass: 0.0

Ion type: M-H

Precursor m/z: 770.57

Charge: 1

Polarity: Negative

Instrument information

Instrument type: CID; Lumos

Ion source: LC-ESI

Ms level: 2

Acquisition: Commercial

Database links

Other information

Principal investigator: Thomas Metz

Data collector: Thomas Metz

Entry ID: CCMSLIB00010059418

Docosahexaenoic acid

0.865

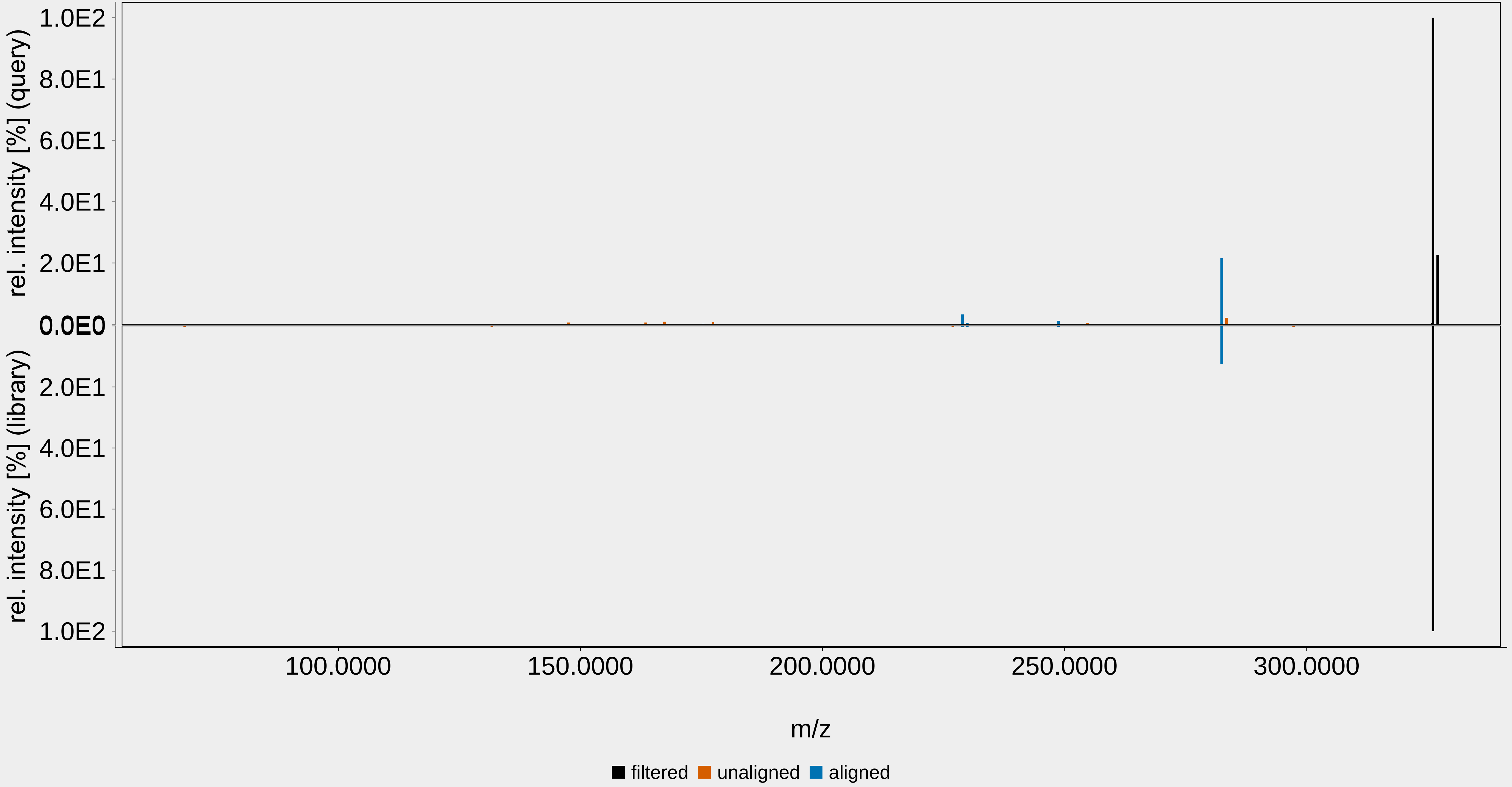

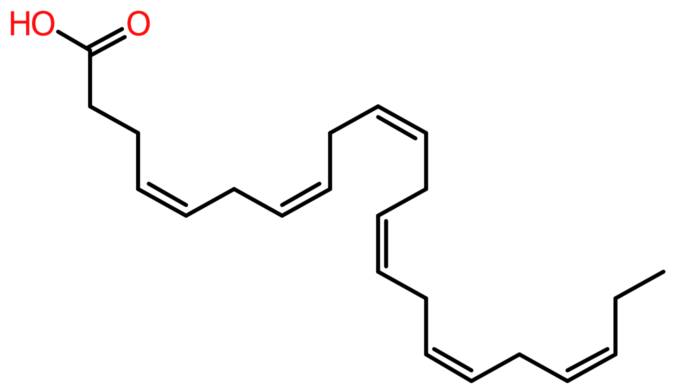

Compound information

Name: Docosahexaenoic acid

Formula: C22H32O2

Exact mass: 328.24023

Ion type: [M-H]-

Precursor m/z: 327.233

Polarity: NEGATIVE

InChI:

InChI key:

SMILES:

Num peaks: 9

Instrument information

Instrument type: LC-ESI-QTOF

Instrument: maXis plus UHR-ToF-MS, Bruker

Ms level: MS2

Collision energy: 10

Database links

Other information

Entry ID: MSBNK-BGC_Munich-RP029611

Comment: Parent=327.233

Spectral Match to cis-4,7,10,13,16,19-Docosahexaenoic acid from NIST14

0.882

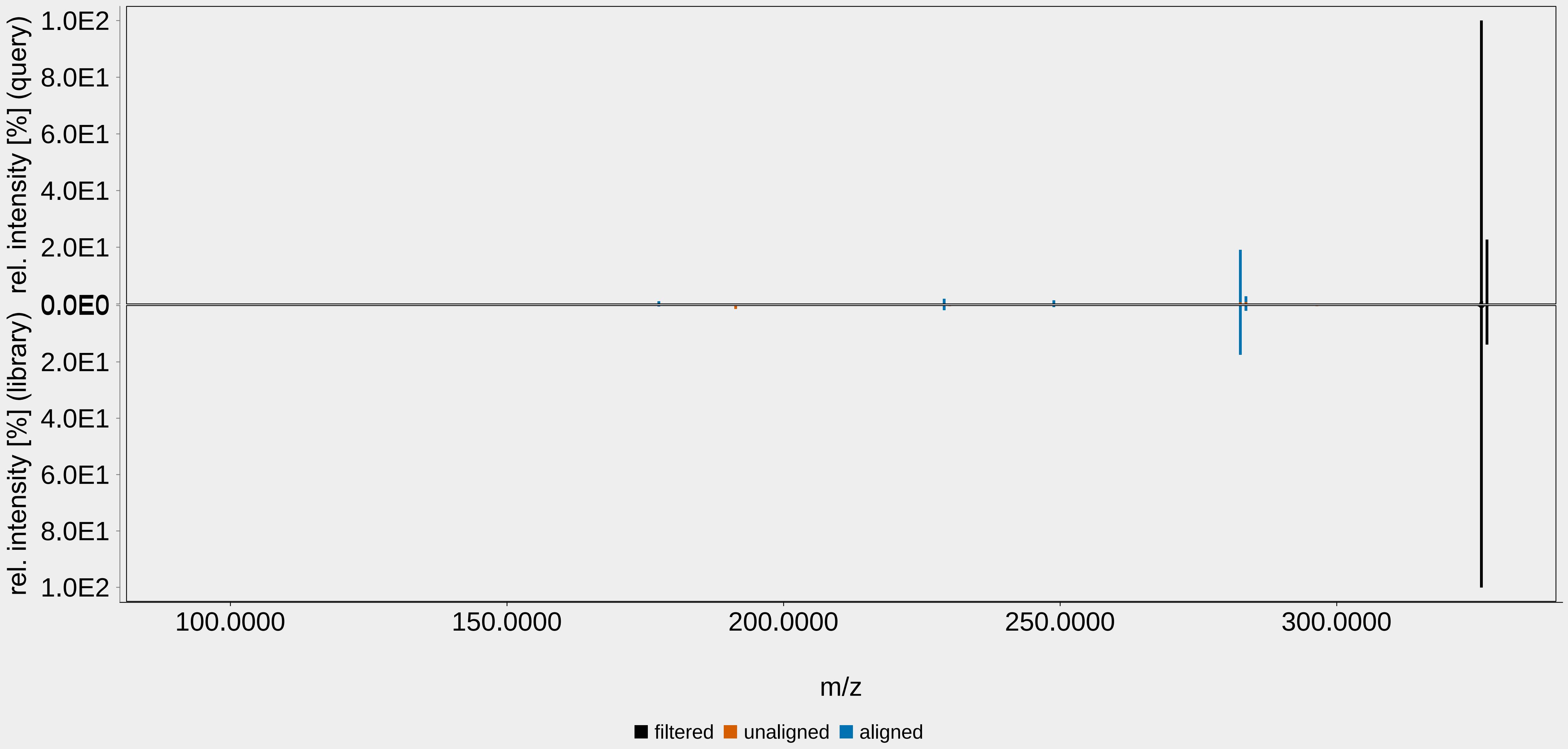

Compound information

Name: Spectral Match to

Exact mass: 0.0

Ion type: M-H

Precursor m/z: 327.232

Charge: 1

Polarity: Negative

SMILES:

Instrument information

Instrument type: qTof

Ion source: LC-ESI

Ms level: 2

Acquisition: Isolated

Database links

CAS: 6217545

Other information

Principal investigator: Data from Charles

Data collector: Data deposited by alchemistmatt

Entry ID: CCMSLIB00003137612

PE(20:0/0:0); [M-H]- C25H51N1O7P1 **0.757**

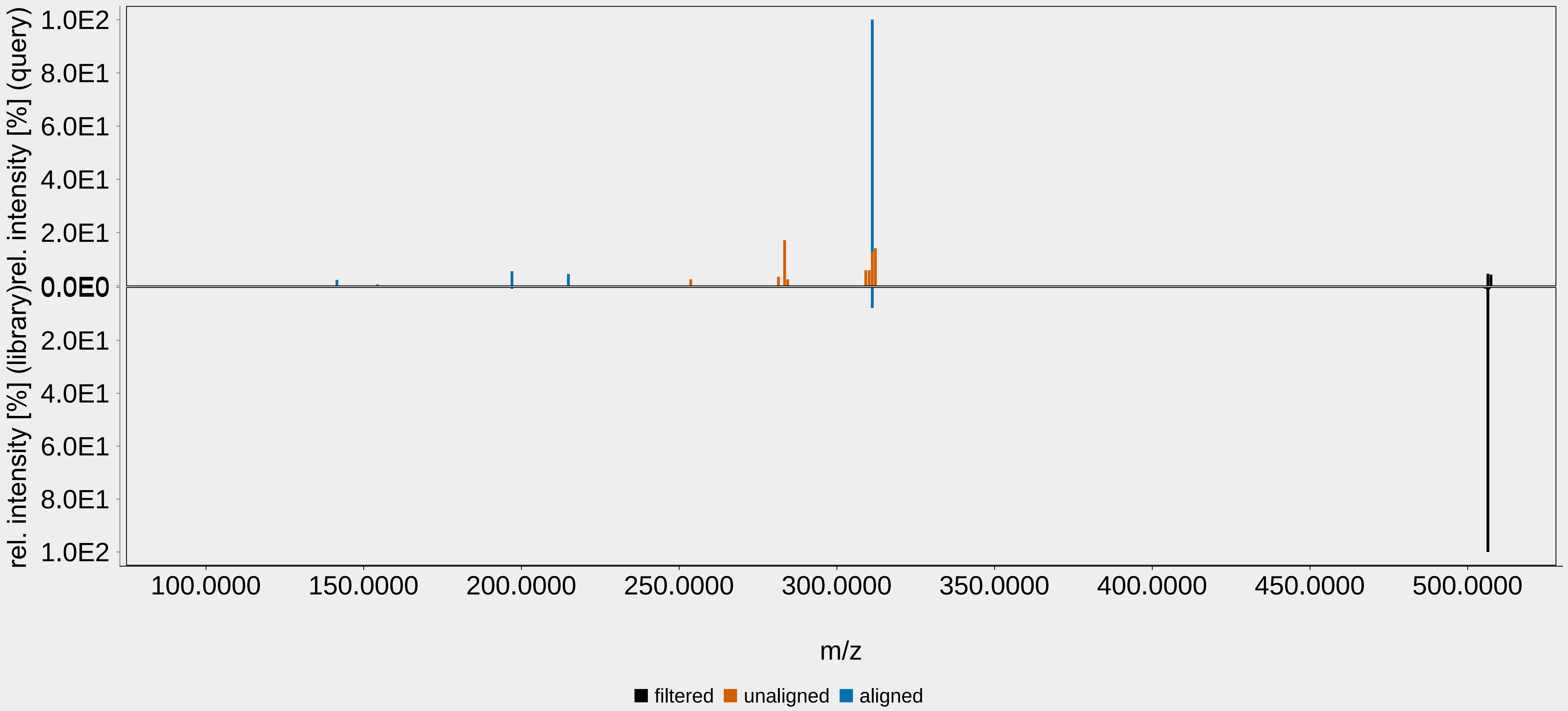

Compound information

Name: PE(20:0/0:0); [M-H]- C25H51N1O7P1

Exact mass: 0.0

Ion type: M-H

Precursor m/z: 508.34

Charge: 1

Polarity: Negative

Instrument information

Instrument type: HCD; Velos

Ion source: LC-ESI

Ms level: 2

Acquisition: Commercial

Database links

Other information

Principal investigator: Thomas Metz

Data collector: Thomas Metz

Entry ID: CCMSLIB00010063231

Adenosine diphosphate - 30.0 eV

0.874

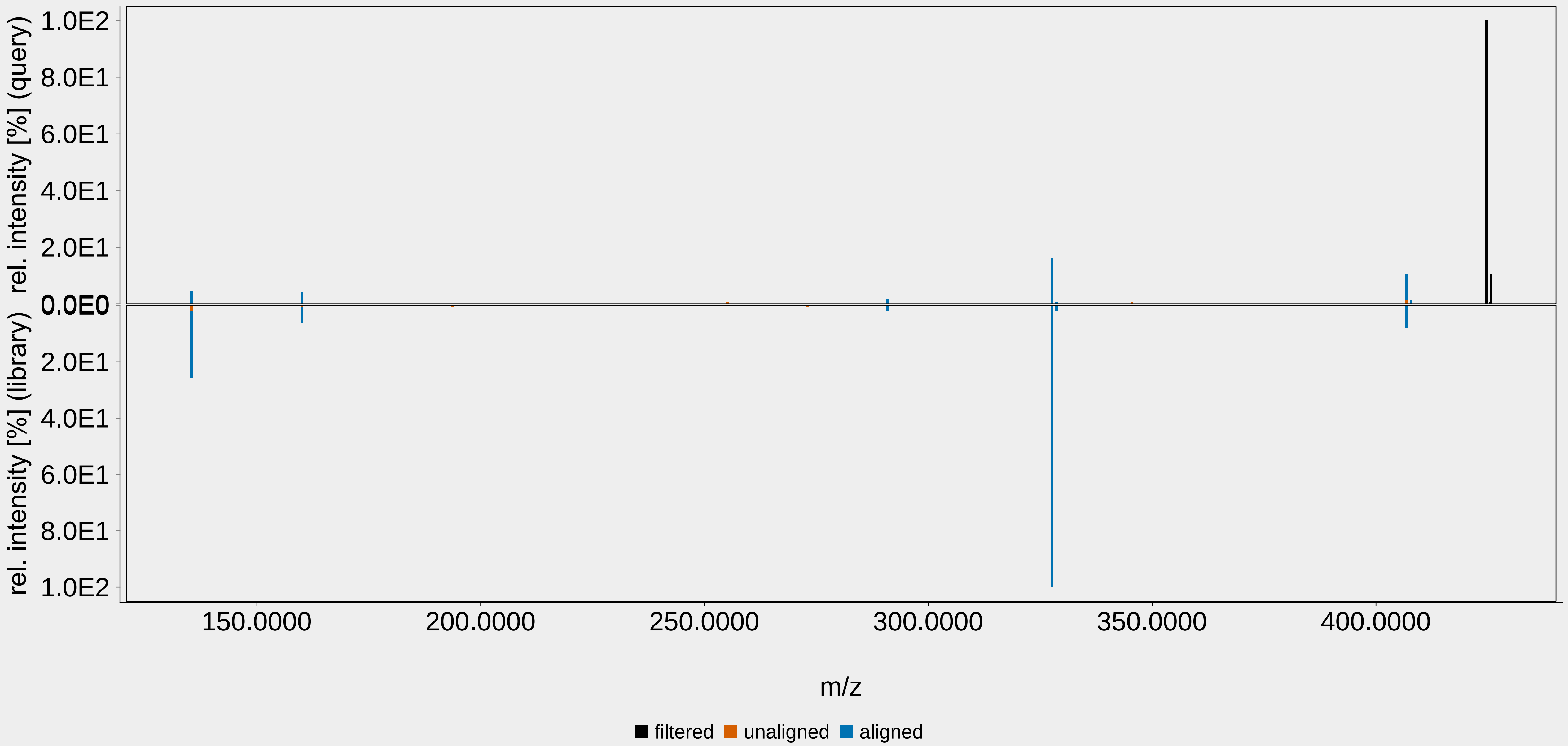

Compound information

Name: Adenosine diphosphate - 30.0 eV

Exact mass: 0.0

Ion type: M-H

Precursor m/z: 426.022

Charge: 1

Polarity: Negative

SMILES:

Instrument information

Instrument type: Orbitrap

Ion source: ESI

Ms level: 2

Acquisition: Isolated

Database links

CAS:

Other information

Principal investigator: PI

Data collector: Data Collector

Entry ID: CCMSLIB00006124443

Adenosine diphosphate - 30.0 eV

0.876

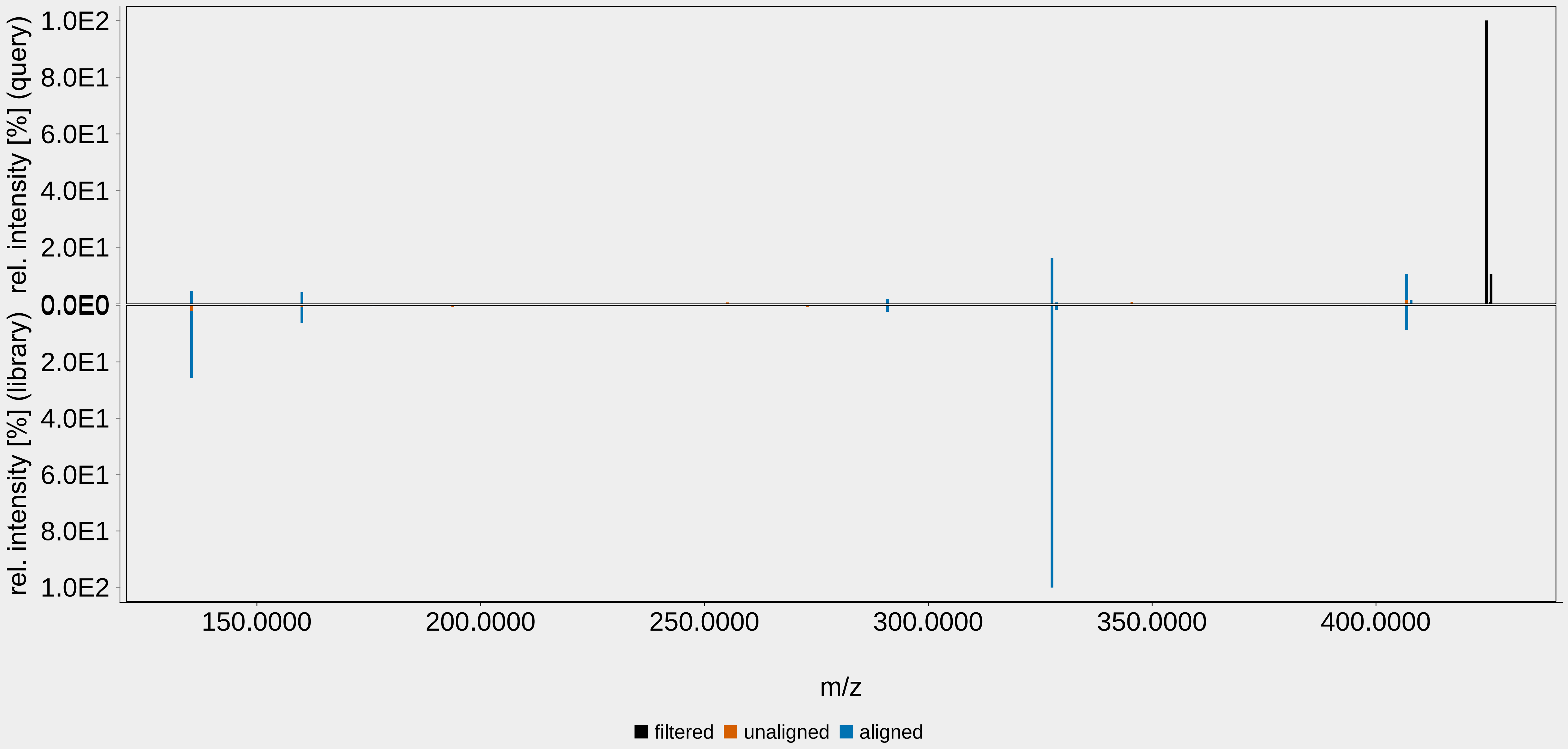

Compound information

Name: Adenosine diphosphate - 30.0 eV

Exact mass: 0.0

Ion type: M-H

Precursor m/z: 426.022

Charge: 1

Polarity: Negative

SMILES:

Instrument information

Instrument type: Orbitrap

Ion source: ESI

Ms level: 2

Acquisition: Isolated

Database links

CAS:

Other information

Principal investigator: PI

Data collector: Data Collector

Entry ID: CCMSLIB00006124450

PG(16:0/18:1); [M-H]- C40H76O10P1 **0.859**

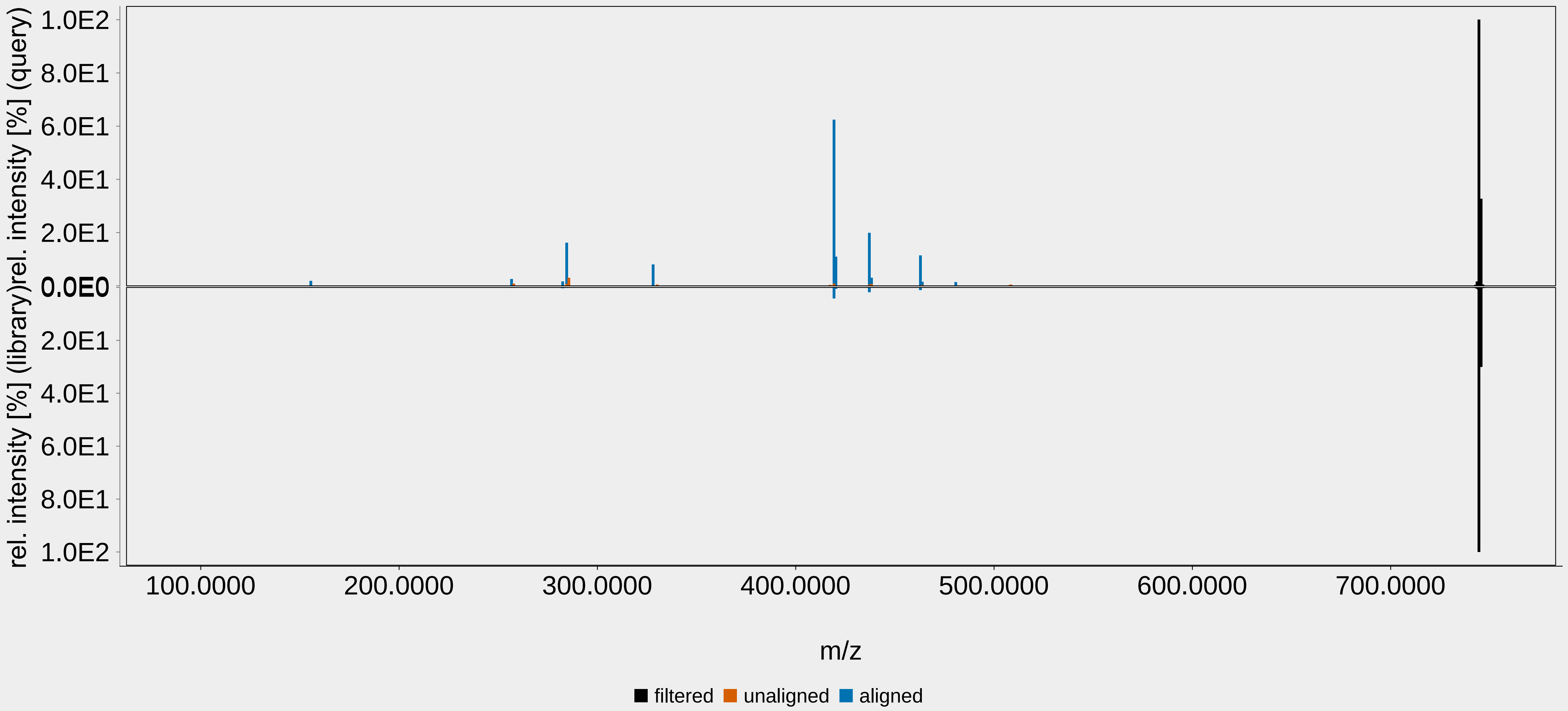

Compound information

Name: PG(16:0/18:1); [M-H]- C40H76O10P1

Exact mass: 0.0

Ion type: M-H

Precursor m/z: 747.518

Charge: 1

Polarity: Negative

Instrument information

Instrument type: HCD; Velos

Ion source: LC-ESI

Ms level: 2

Acquisition: Commercial

Database links

Other information

Principal investigator: Thomas Metz

Data collector: Thomas Metz

Entry ID: CCMSLIB00010063005

PG(16:0/18:1); [M-H]- C40H76O10P1 **0.864**

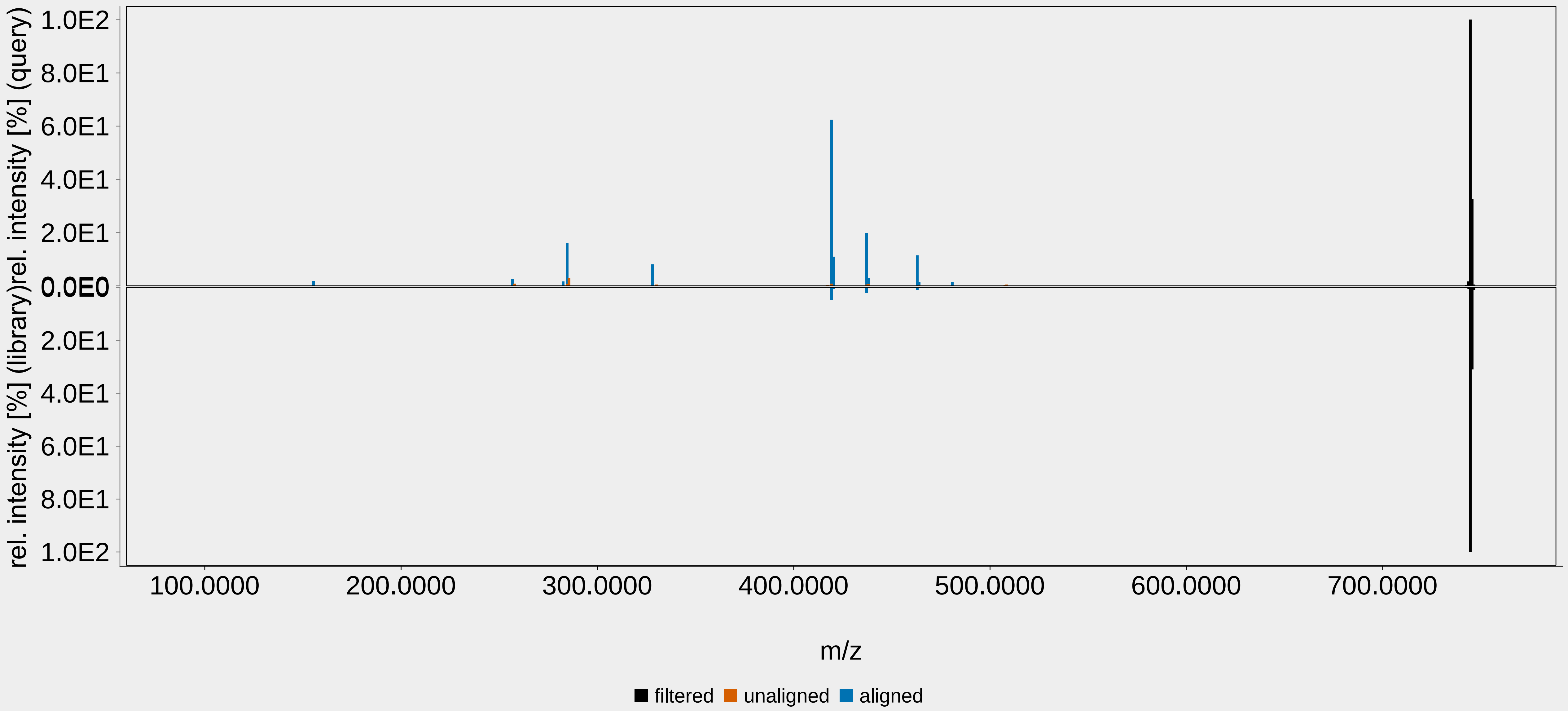

Compound information

Name: PG(16:0/18:1); [M-H]- C40H76O10P1

Exact mass: 0.0

Ion type: M-H

Precursor m/z: 747.518

Charge: 1

Polarity: Negative

Instrument information

Instrument type: HCD; Velos

Ion source: LC-ESI

Ms level: 2

Acquisition: Commercial

Database links

Other information

Principal investigator: Thomas Metz

Data collector: Thomas Metz

Entry ID: CCMSLIB00010063728

C11-LAS (STANDARD MIX), C11-linear alkylbenzyl sulfonate, 4-(undecan-5-yl)benzenesulfonic acid

0.874

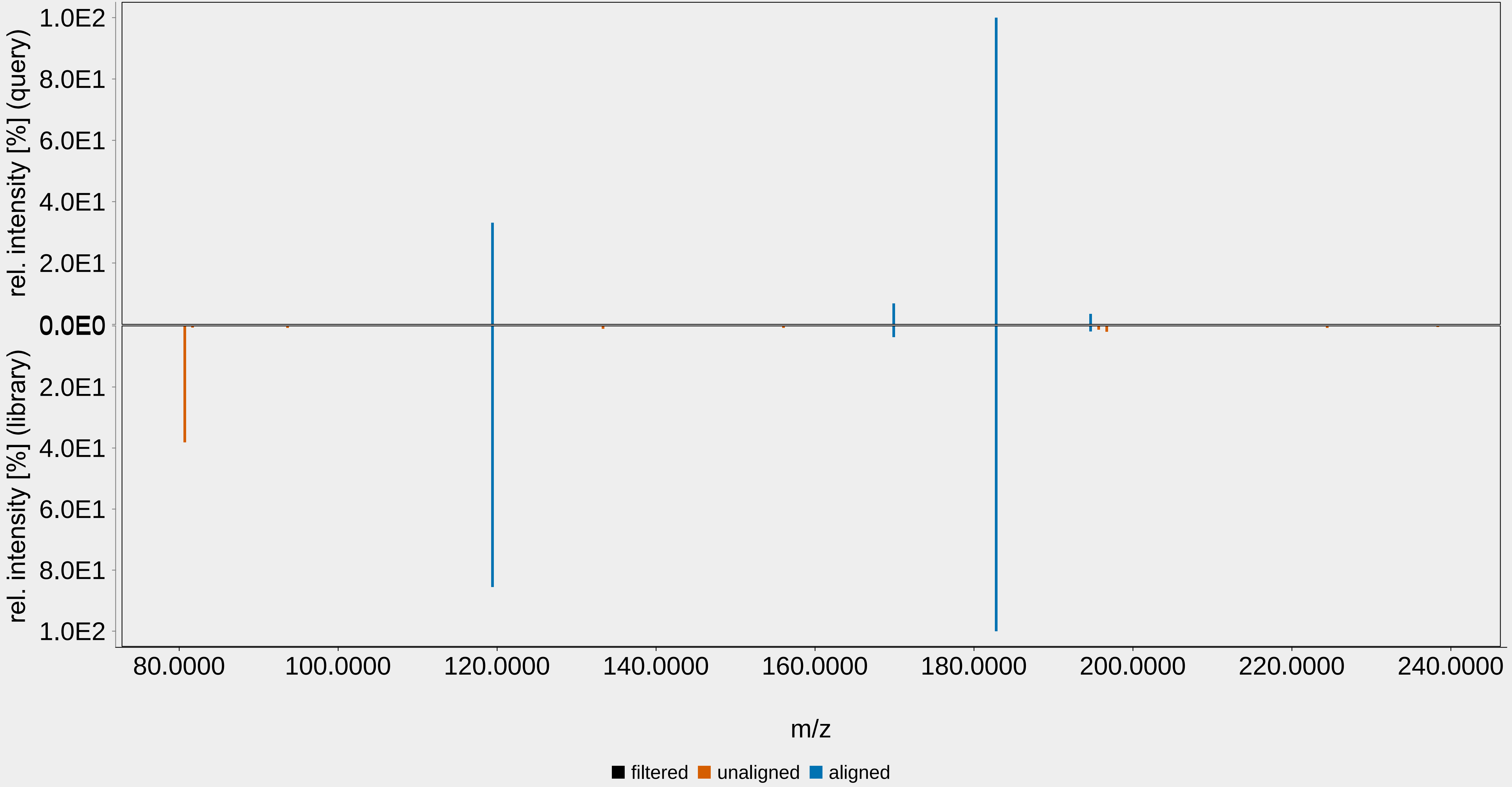

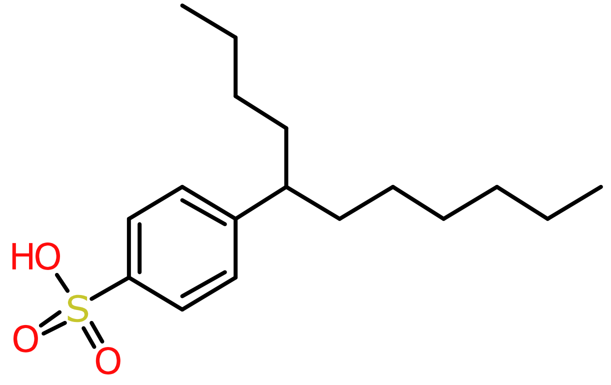

Compound information

Name: C11-LAS (STANDARD MIX), C11-linear

Formula: C17H28O3S

Mol. weight: 312.1759

Exact mass: 312.1759

Ion type: [M-H]-

Precursor m/z: 311.1686

Polarity: negative

InChI:

InChI key:

SMILES:

Instrument information

Instrument type: LC-ESI-QFT

Instrument: Q Exactive Orbitrap Thermo

Ion source: ESI

Ms level: MS2

Collision energy: 90 NCE

Database links

Pubchem: 15593874

MoNA ID: ETS00014

Other information

Principal investigator: E. Schymanski, Dept. of

Data collector: E. Schymanski, Dept. of

C11-LAS (STANDARD MIX)

0.874

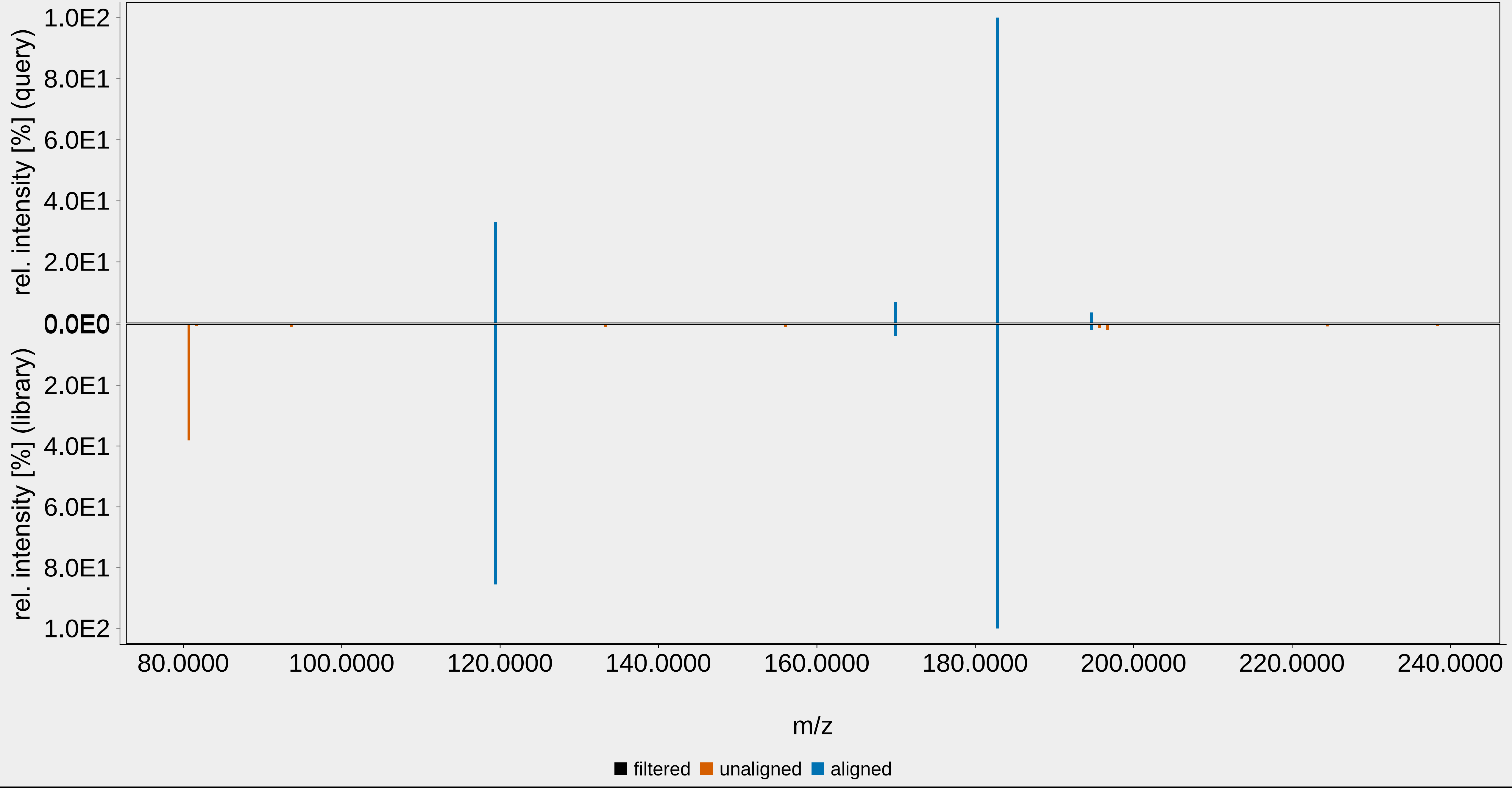

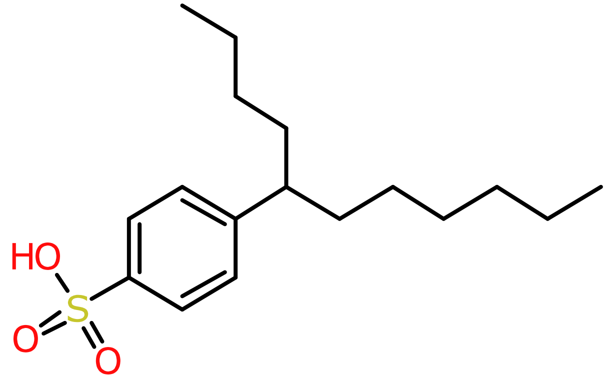

Compound information

Name: C11-LAS (STANDARD MIX)

Formula: C17H28O3S

Exact mass: 312.1759

Ion type: [M-H]-

Precursor m/z: 311.1686

Polarity: NEGATIVE

InChI:

InChI key:

SMILES:

Num peaks: 13

Instrument information

Instrument type: LC-ESI-QFT

Instrument: Q Exactive Orbitrap Thermo

Ms level: MS2

Collision energy: 90 NCE

Database links

Other information

Entry ID:

Comment: Parent=311.1686

PA 18:0_20:4

0.875

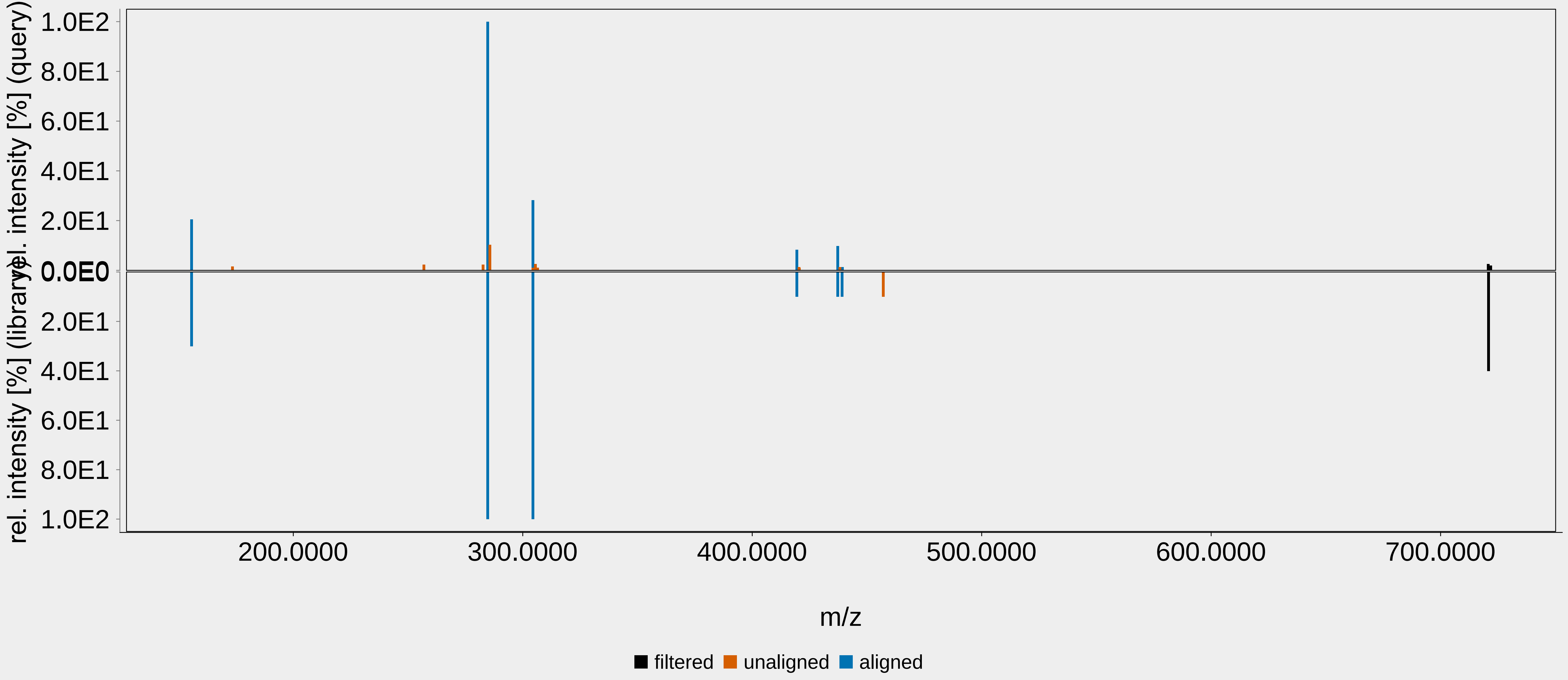

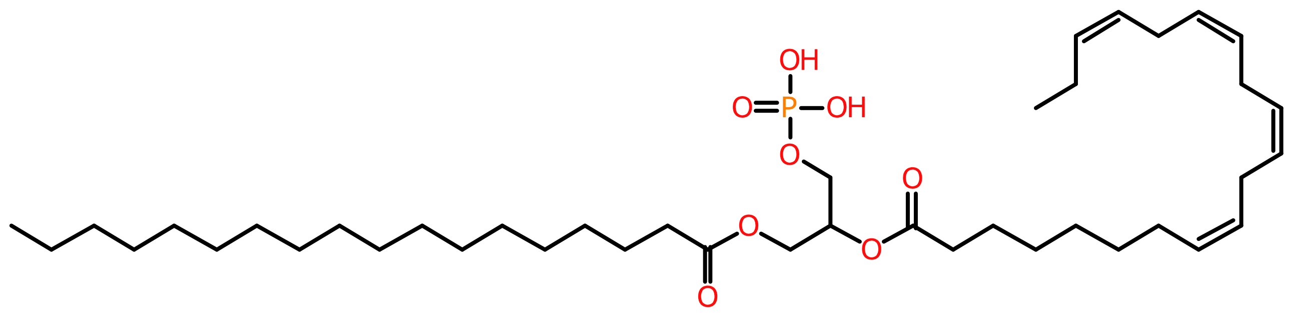

Compound information

Name: PA 18:0_20:4

Formula: C41H73O8P

Ion type: [M-H]-

Precursor m/z: 723.49703

Ccs: 262.1

InChI key:

SMILES:

Num peaks: 8

Instrument information

Database links

Other information

PA(18:0/20:4); [M-H]- C41H72O8P1 0.880

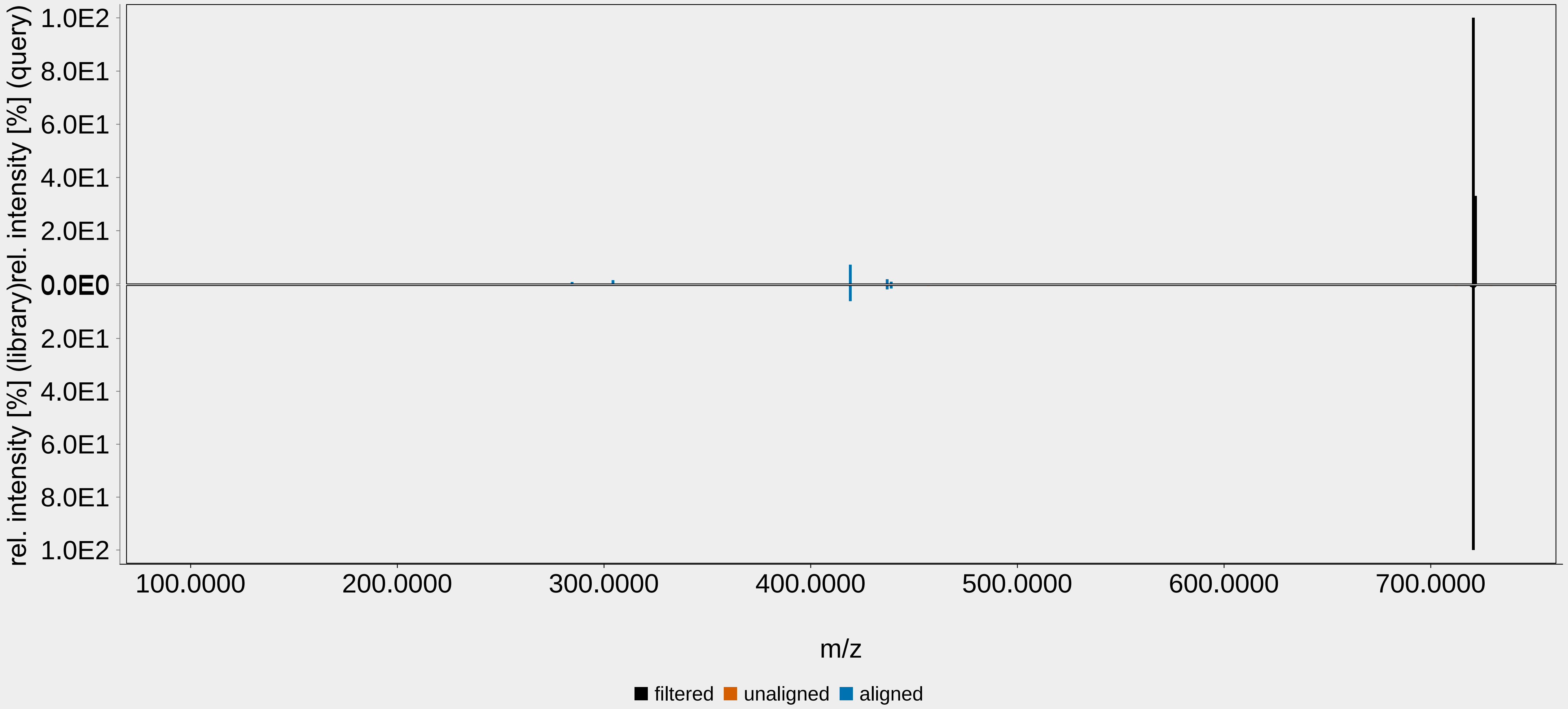

Compound information

Name: PA(18:0/20:4); [M-H]- C41H72O8P1

Exact mass: 0.0

Ion type: M-H

Precursor m/z: 723.496

Charge: 1

Polarity: Negative

Instrument information

Instrument type: HCD; Velos

Ion source: LC-ESI

Ms level: 2

Acquisition: Commercial

Database links

Other information

Principal investigator: Thomas Metz

Data collector: Thomas Metz

Entry ID: CCMSLIB00010065411

[1-hexadecanoyloxy-3-phosphonooxypropan-2-yl]octadec-9-enoate

0.908

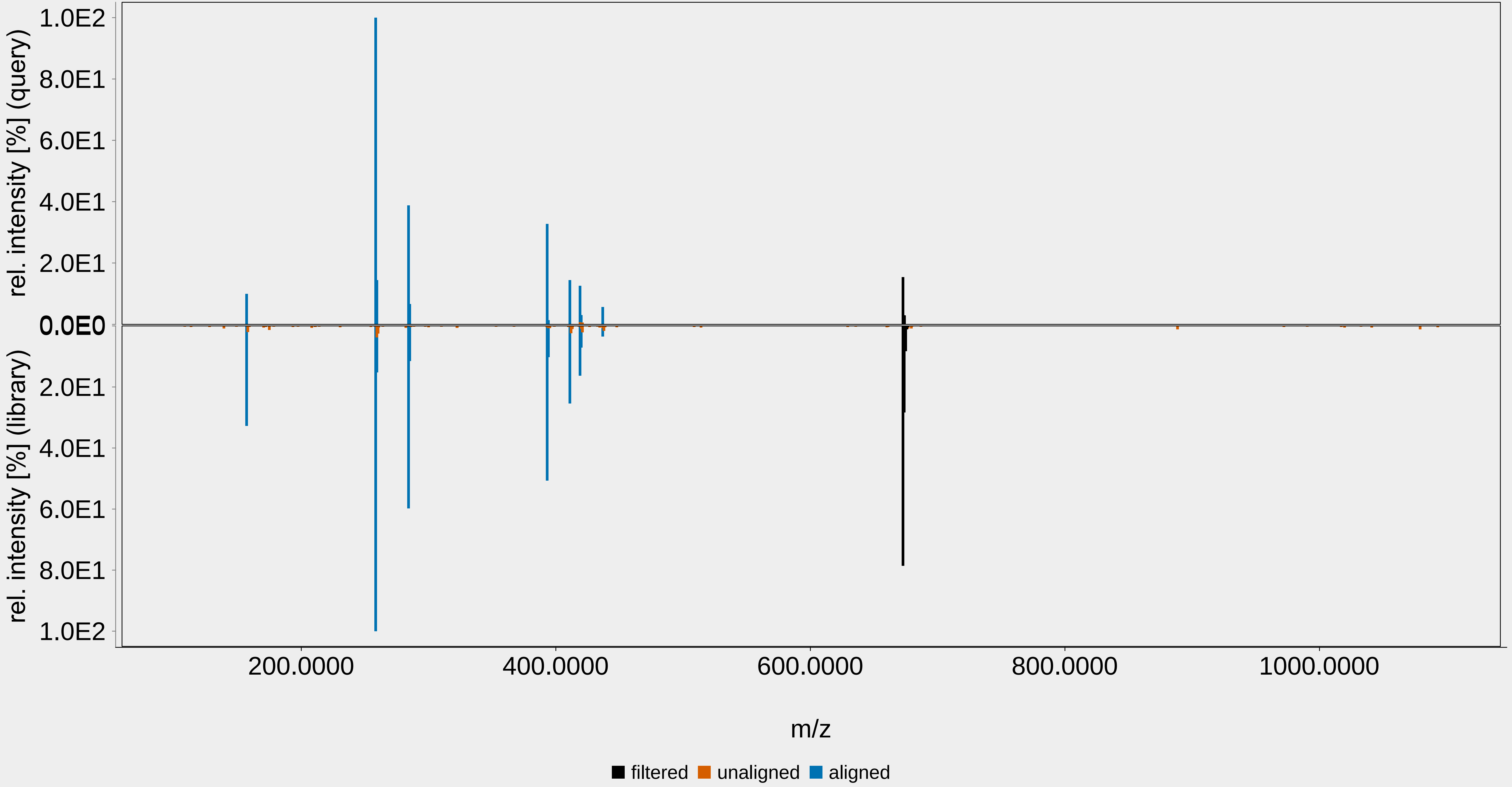

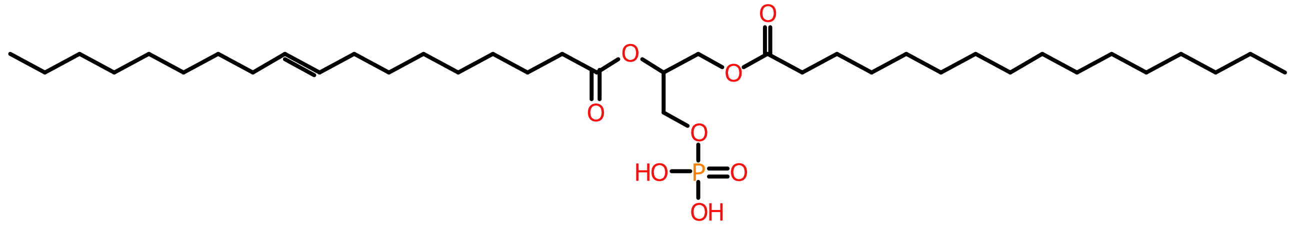

Compound information

Name:

Formula: C37H71O8P

Mol. weight: 674.489

Exact mass: 674.489

Ion type: [M-H]-

Precursor m/z: 673.481

Polarity: negative

InChI:

InChI key:

SMILES:

Instrument information

Instrument: qTof

Ms level: MS2

Database links

Other information

Principal investigator: O Laprevote, UMR 8038

Data collector: O Laprevote, UMR 8038

PA 16:0_18:1

0.935

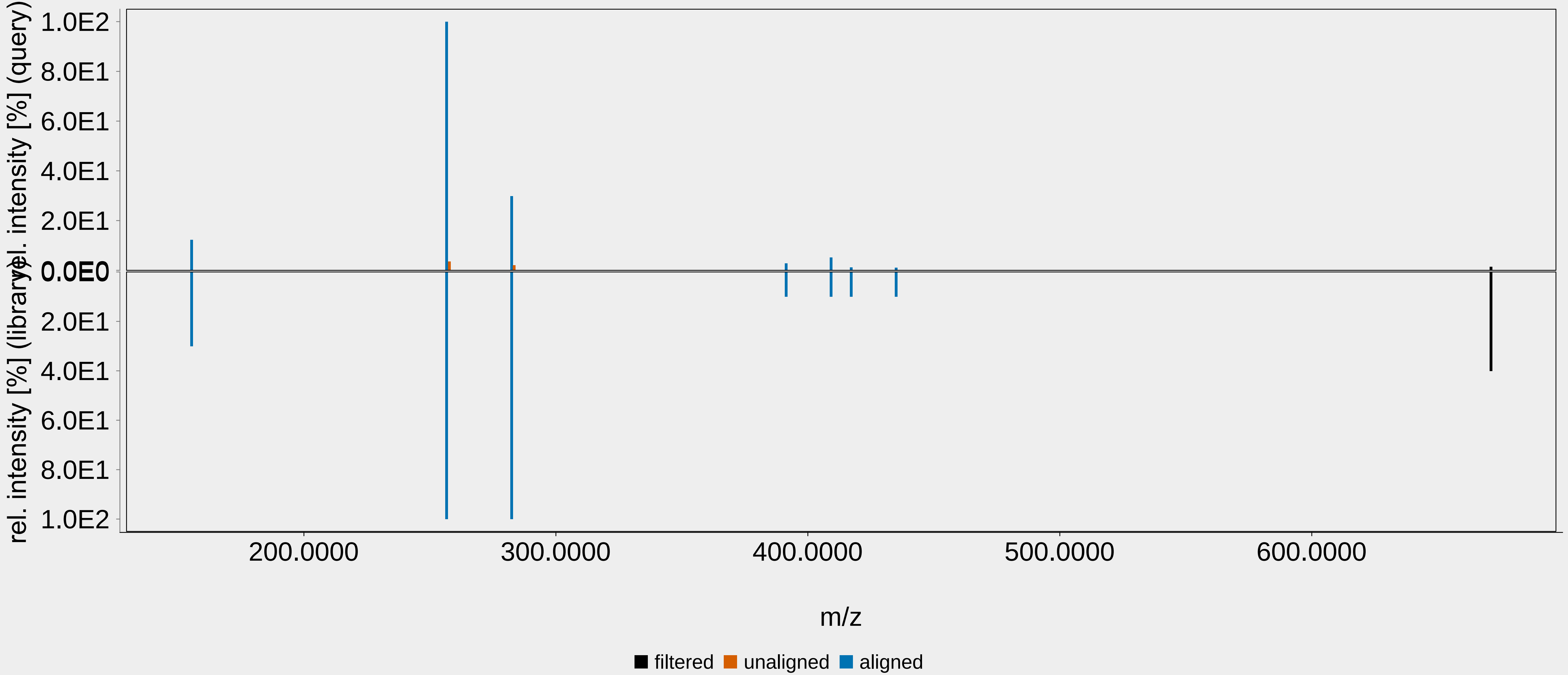

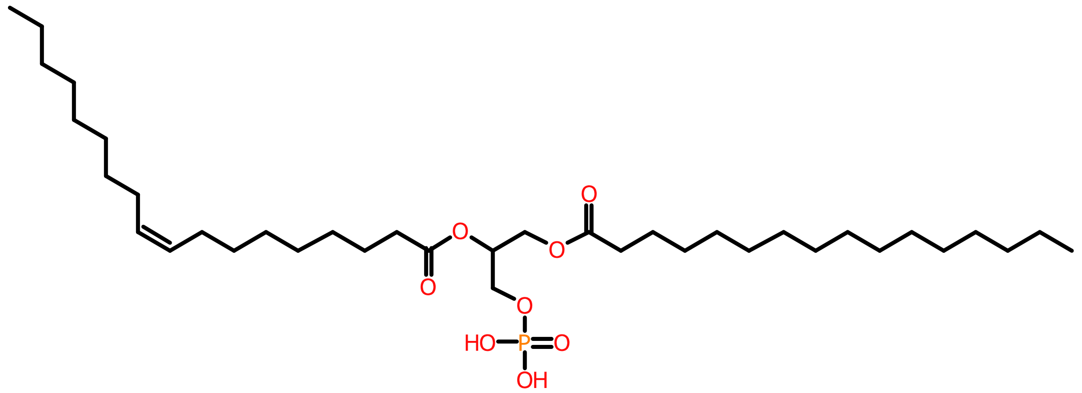

Compound information

Name: PA 16:0_18:1

Formula: C37H71O8P

Ion type: [M-H]-

Precursor m/z: 673.48138

Ccs: 252.18

InChI key:

SMILES:

Num peaks: 8

Instrument information

Database links

Other information

PA 22:4_22:6 **0.872**

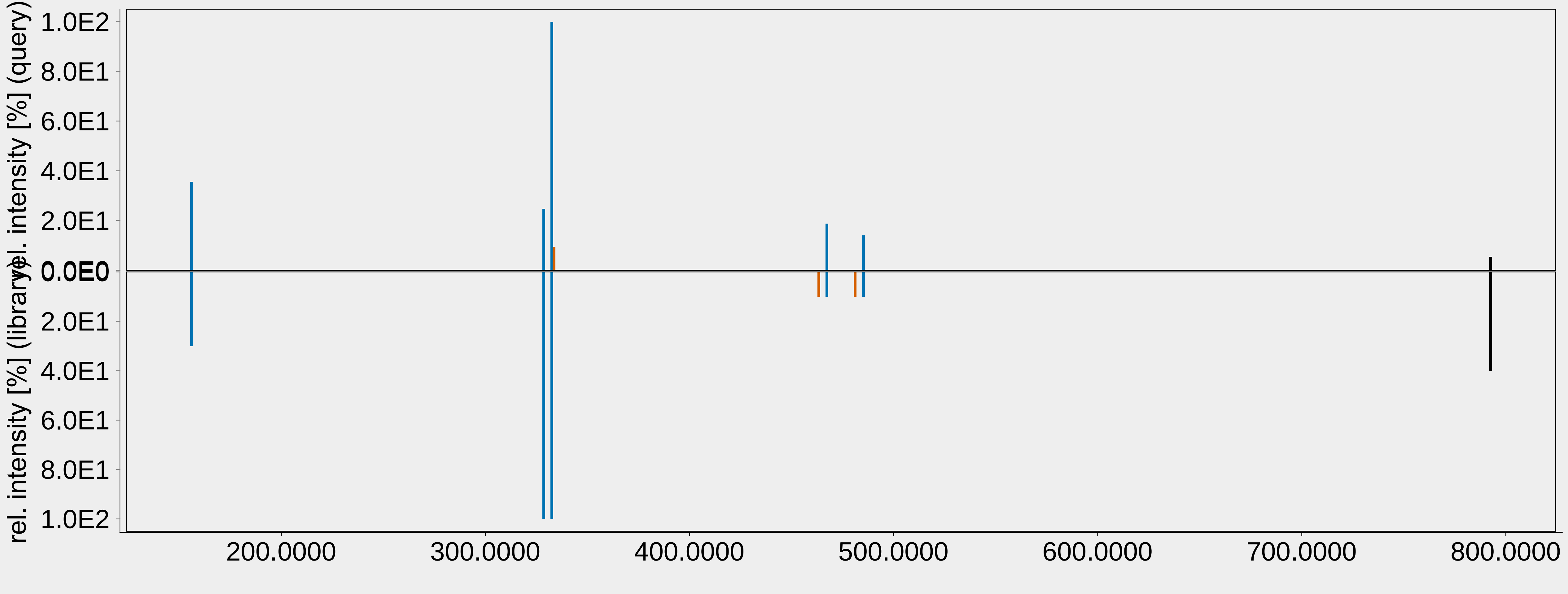

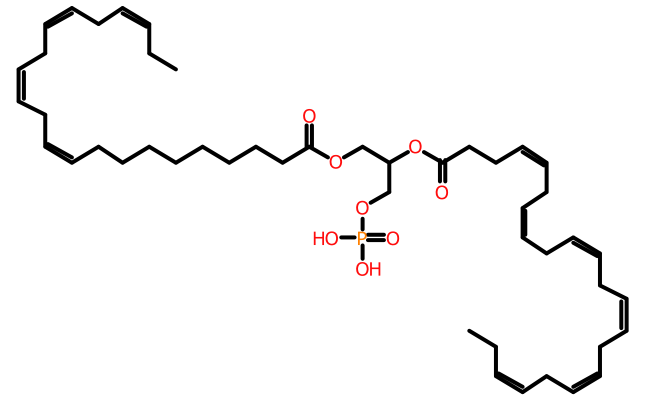

Compound information

Name: PA 22:4_22:6

Formula: C47H73O8P

Ion type: [M-H]-

Precursor m/z: 795.49703

Ccs: 272.68

InChI key:

SMILES:

Num peaks: 8

Instrument information

Database links

Other information

Spectral Match to 1-Oleoyl-L-.alpha.-lysophosphatidic acid from NIST14

0.830

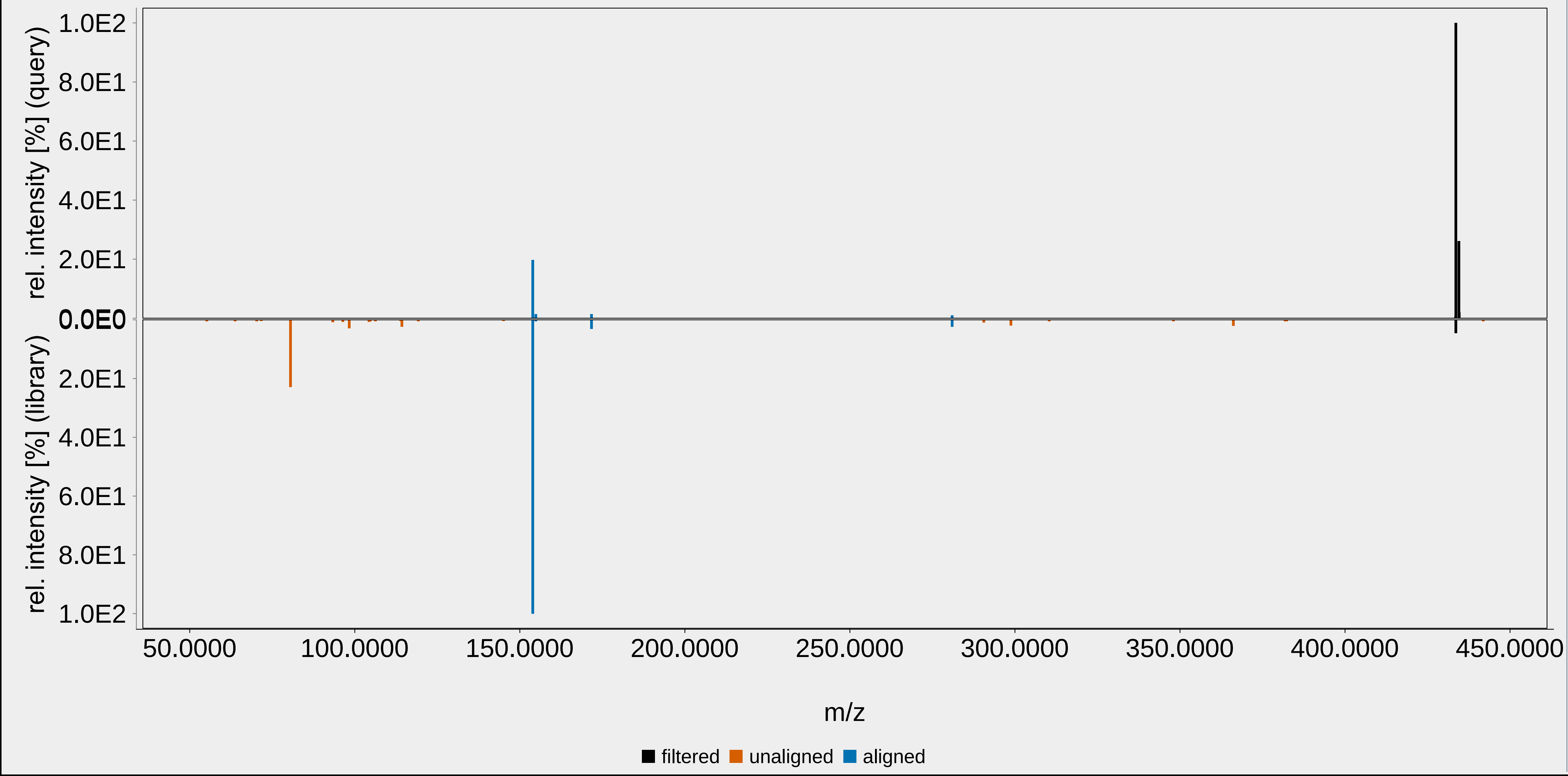

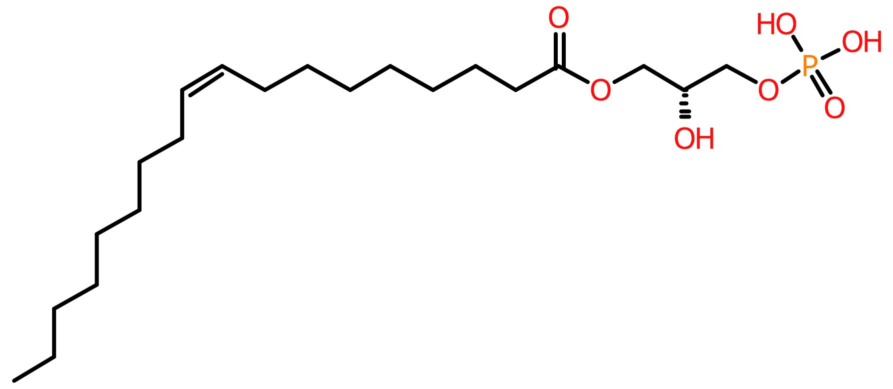

Compound information

Name: Spectral Match to

Exact mass: 436.259

Ion type: M-H

Precursor m/z: 435.252

Charge: 1

Polarity: Negative

InChI:

SMILES:

Instrument information

Instrument type: qTof

Ion source: ESI

Ms level: 2

Acquisition: Isolated

Database links

CAS: 22002875

Other information

Principal investigator: Data from Kevin Bush

Data collector: Data deposited by fevargas

Entry ID: CCMSLIB00003137366

PS 18:1_20:1 **0.733**

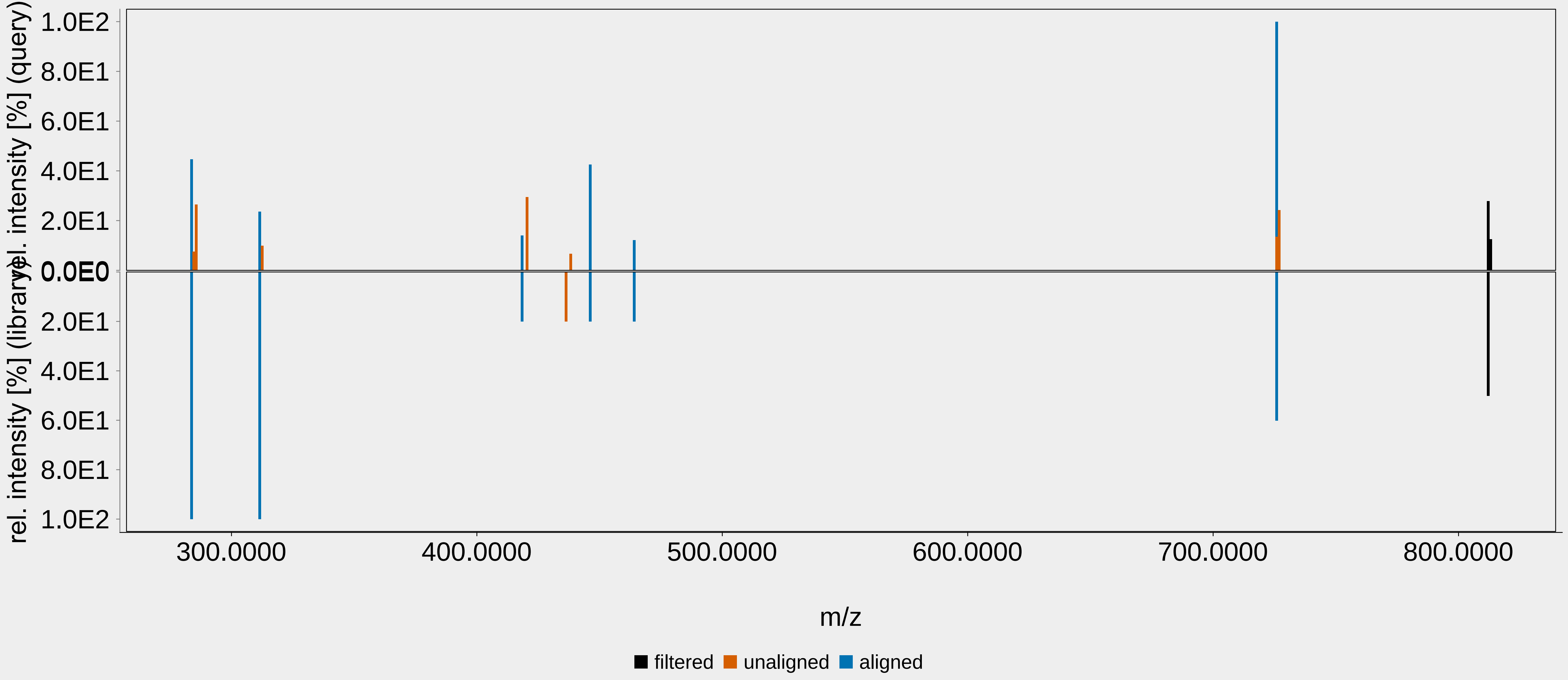

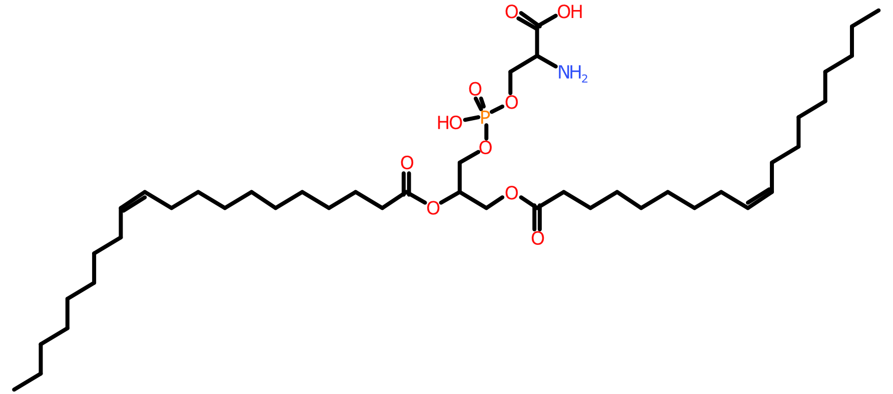

Compound information

Name: PS 18:1_20:1

Formula: C44H82NO10P

Ion type: [M-H]-

Precursor m/z: 814.56036

Ccs: 282.14

InChI key:

SMILES:

Num peaks: 8

Instrument information

Database links

Other information

PS 18:0_20:1 **0.783**

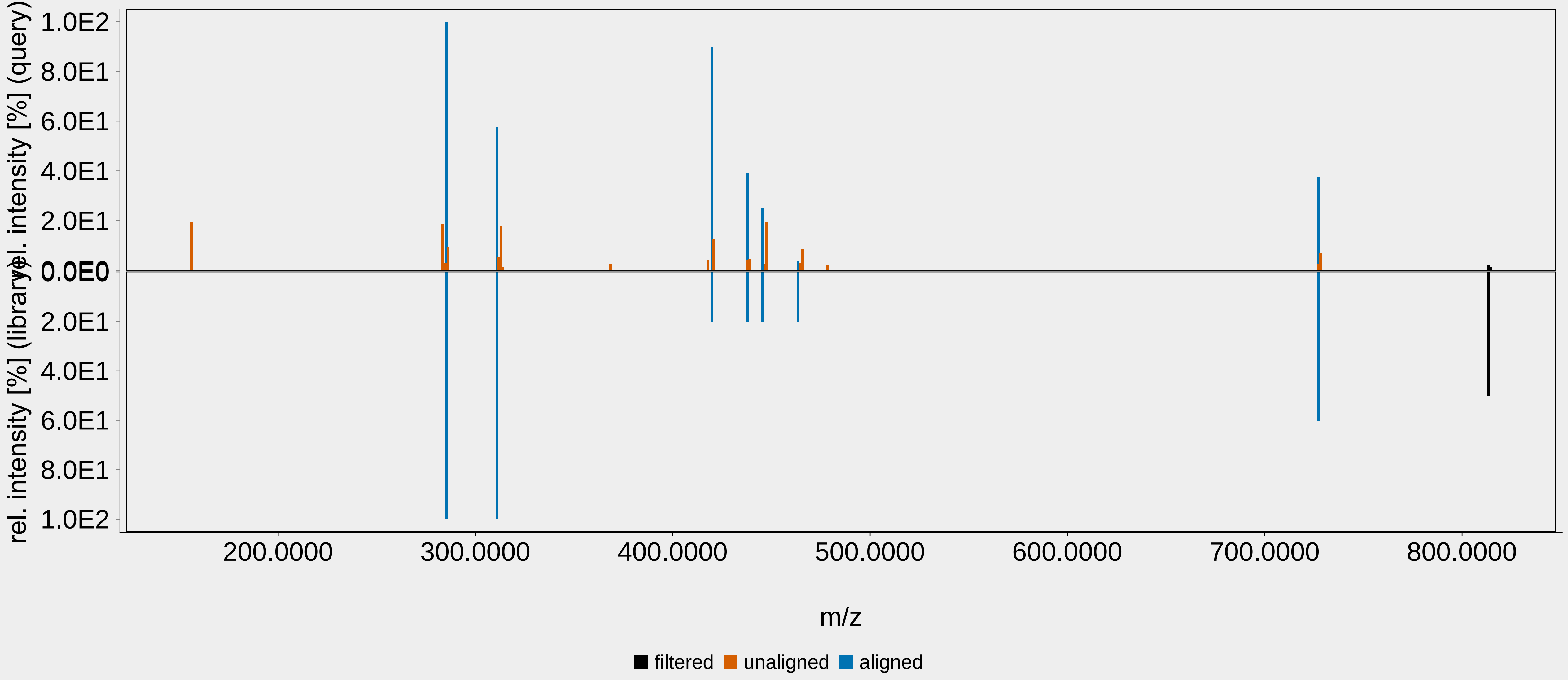

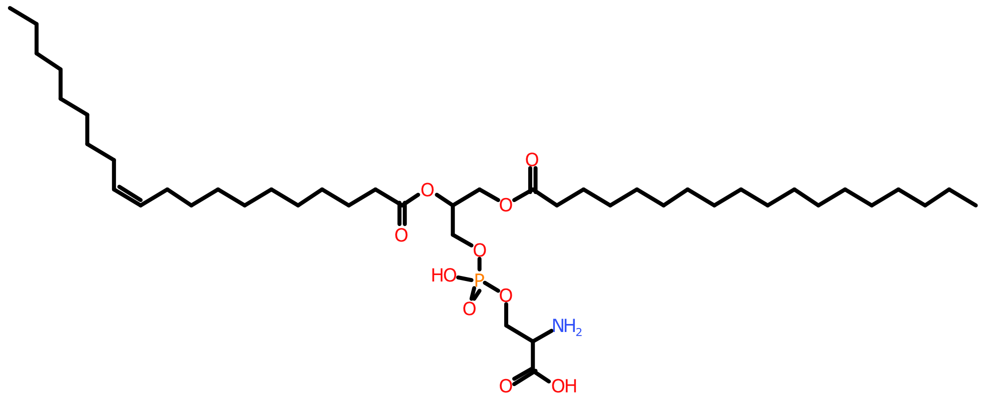

Compound information

Name: PS 18:0_20:1

Formula: C44H84NO10P

Ion type: [M-H]-

Precursor m/z: 816.57601

Ccs: 284.42

InChI key: KVMIIDXBXADXIM-ZPHPHTNENA-I

SMILES:

Num peaks: 8

Instrument information

Database links

Other information

PS(22:6/0:0); [M-H]- C28H43N1O9P1 **0.753**

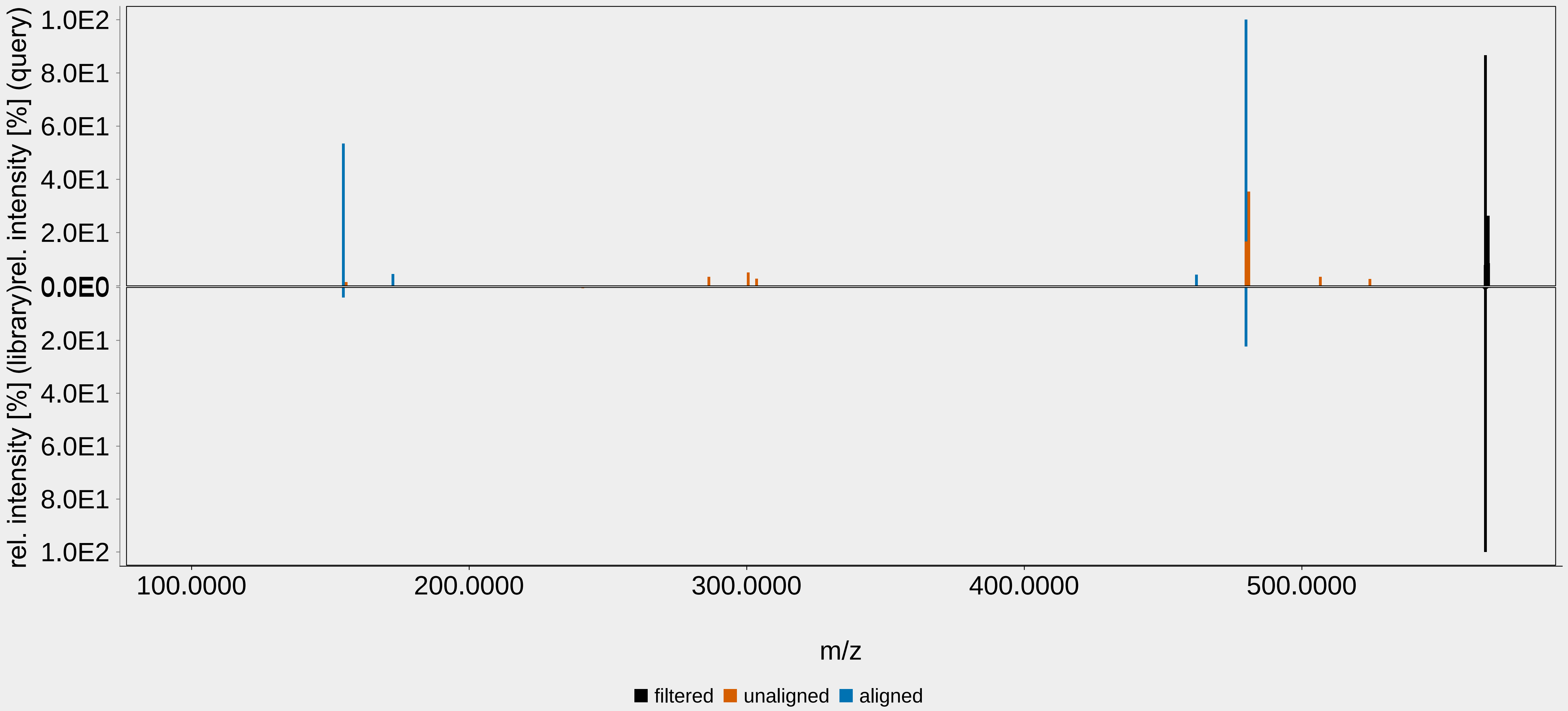

Compound information

Name: PS(22:6/0:0); [M-H]- C28H43N1O9P1

Exact mass: 0.0

Ion type: M-H

Precursor m/z: 568.268

Charge: 1

Polarity: Negative

Instrument information

Instrument type: HCD; Velos

Ion source: LC-ESI

Ms level: 2

Acquisition: Commercial

Database links

Other information

Principal investigator: Thomas Metz

Data collector: Thomas Metz

Entry ID: CCMSLIB00010087165

PE(22:4/22:6); [M-H]- C49H77N1O8P1 **0.908**

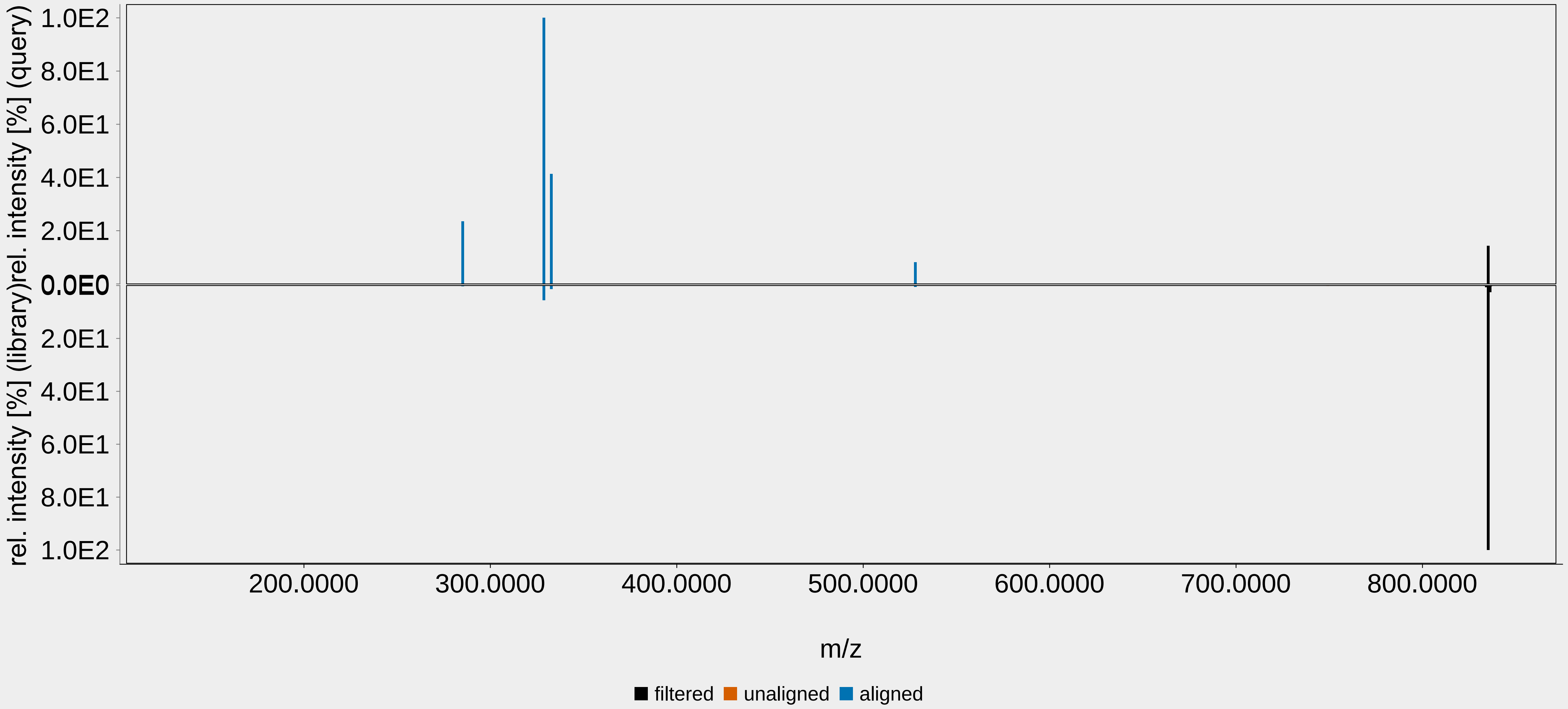

Compound information

Name: PE(22:4/22:6); [M-H]- C49H77N1O8P1

Exact mass: 0.0

Ion type: M-H

Precursor m/z: 838.539

Charge: 1

Polarity: Negative

Instrument information

Instrument type: HCD; Velos

Ion source: LC-ESI

Ms level: 2

Acquisition: Commercial

Database links

Other information

Principal investigator: Thomas Metz

Data collector: Thomas Metz

Entry ID: CCMSLIB00010063765

PE(22:4/22:6); [M-H]- C49H77N1O8P1 **0.900**

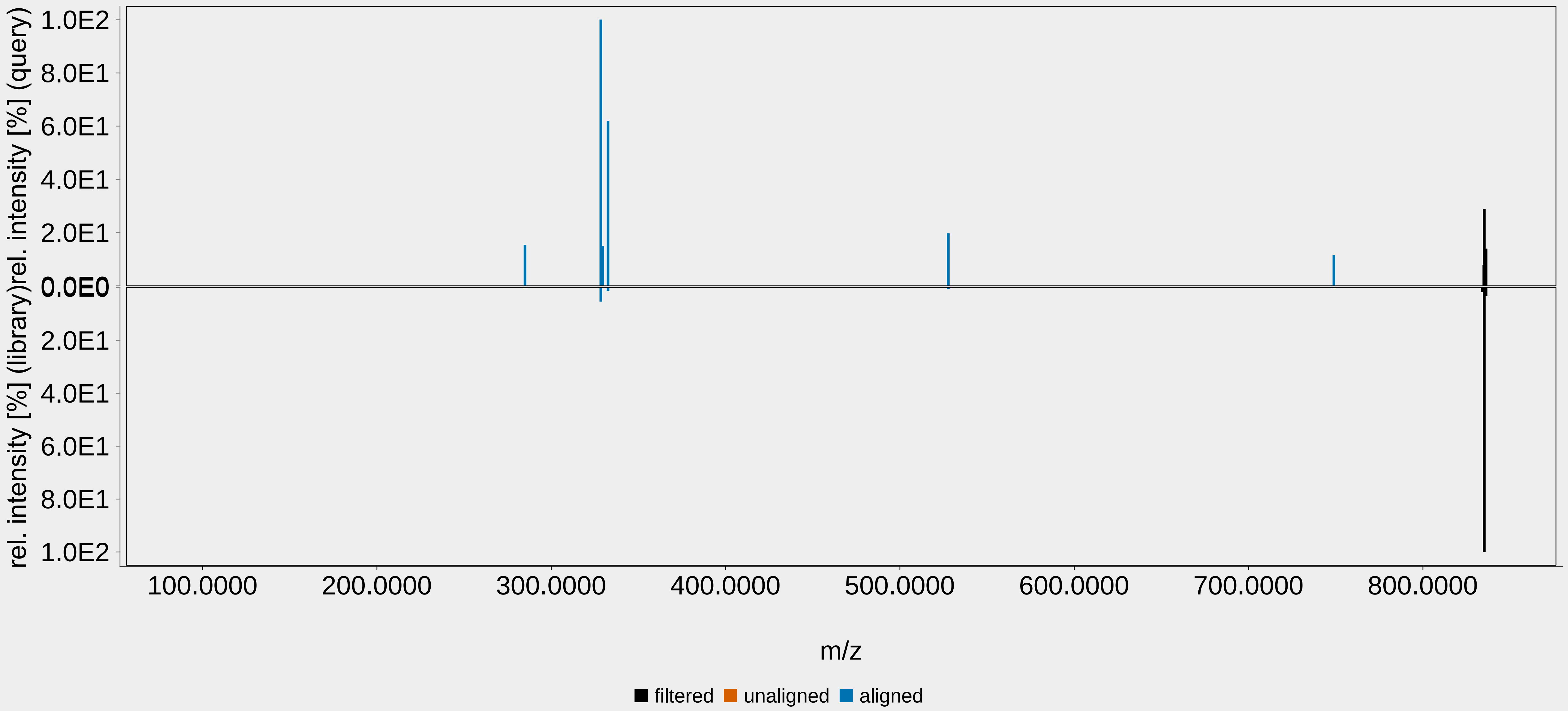

Compound information

Name: PE(22:4/22:6); [M-H]- C49H77N1O8P1

Exact mass: 0.0

Ion type: M-H

Precursor m/z: 838.539

Charge: 1

Polarity: Negative

Instrument information

Instrument type: HCD; Velos

Ion source: LC-ESI

Ms level: 2

Acquisition: Commercial

Database links

Other information

Principal investigator: Thomas Metz

Data collector: Thomas Metz

Entry ID: CCMSLIB00010086991

PI 36:2 **0.716**

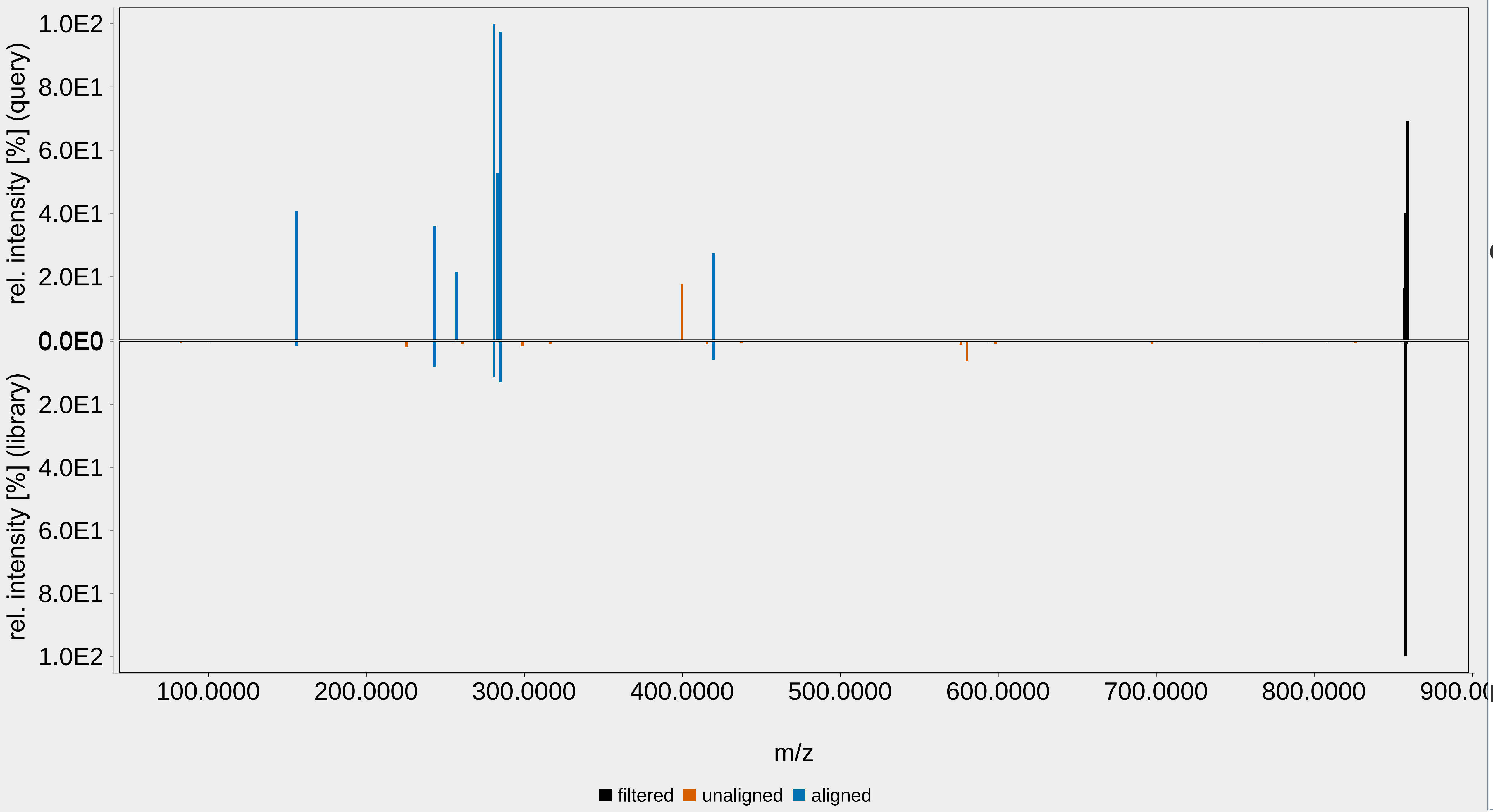

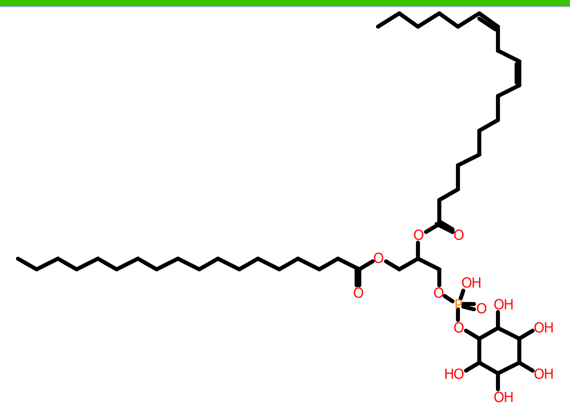

Compound information

Name: PI 36:2

Formula: C45H83O13P

Mol. weight: 863.122

Exact mass: 863.122

Ion type: [M-H]-

Precursor m/z: 861.5504

RT: 8.773114

Polarity: negative

InChI:

InChI key:

SMILES:

Instrument information

Instrument type: LC-ESI-QTOF

Instrument: AB Sciex TripleTOF 5600+ system

Ms level: MS2

Collision energy: 45V

Database links

Pubchem: 138199526

MoNA ID: LQB00692

Other information

Principal investigator: Kazutaka Ikeda, Hiroshi

Data collector: Kazutaka Ikeda, Hiroshi

PI 36:2 0.716

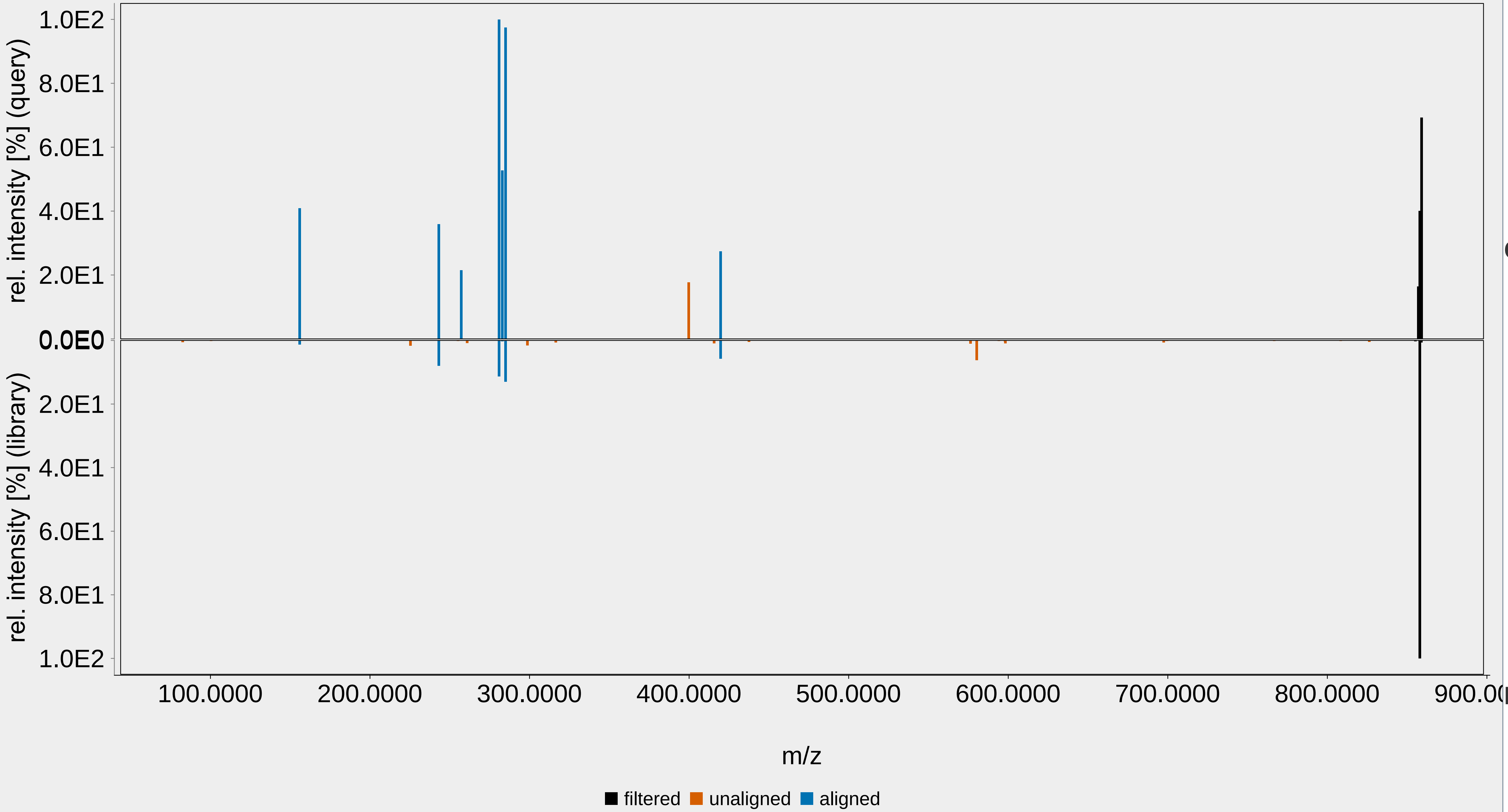

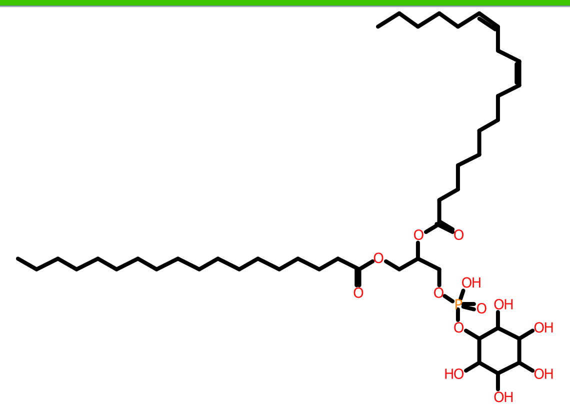

Compound information

Name: PI 36:2

Formula: C45H83O13P

Mol. weight: 863.122

Exact mass: 863.122

Ion type: [M-H]-

Precursor m/z: 861.5504

RT: 8.773114

Polarity: negative

InChI:

InChI key:

SMILES:

Instrument information

Instrument type: LC-ESI-QTOF

Instrument: AB Sciex TripleTOF 5600+ system

Ms level: MS2

Collision energy: 45V

Database links

Pubchem: 138199526

MoNA ID: LQB00306

Other information

Principal investigator: Kazutaka Ikeda, Hiroshi

Data collector: Kazutaka Ikeda, Hiroshi

PI 38:6 **0.821**

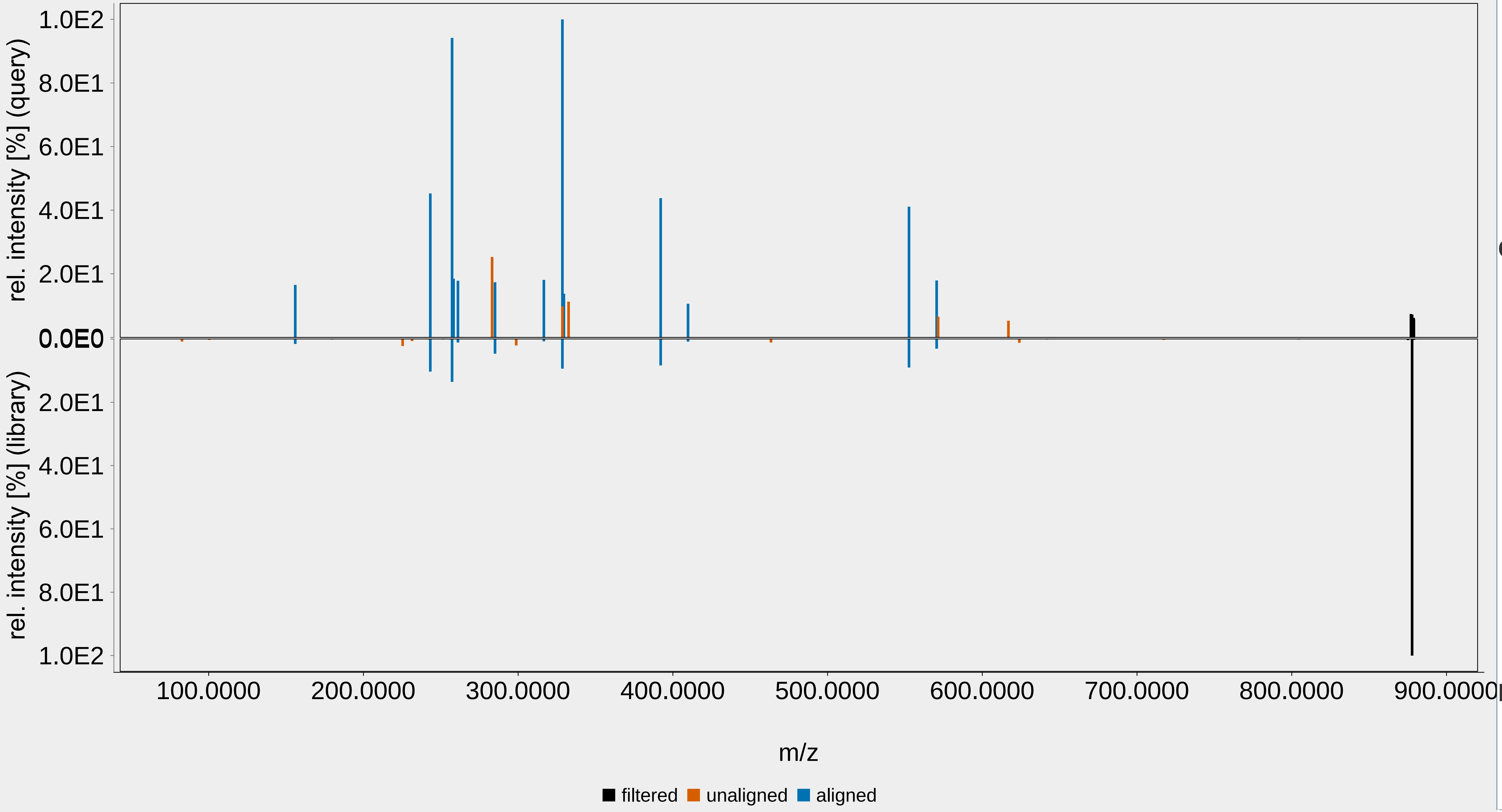

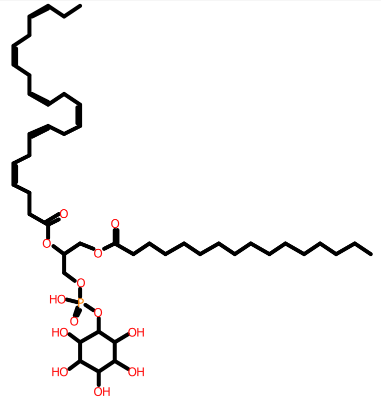

Compound information

Name: PI 38:6

Formula: C47H79O13P

Mol. weight: 883.1119999999999

Exact mass: 883.1119999999999

Ion type: [M-H]-

Precursor m/z: 881.514

RT: 8.246706

Polarity: negative

InChI:

InChI key:

SMILES:

Instrument information

Instrument type: LC-ESI-QTOF

Instrument: AB Sciex TripleTOF 5600+ system

Ms level: MS2

Collision energy: 45V

Database links

Pubchem: 138154339

MoNA ID: LQB00686

Other information

Principal investigator: Kazutaka Ikeda, Hiroshi

Data collector: Kazutaka Ikeda, Hiroshi

PI 38:6 — 0.821

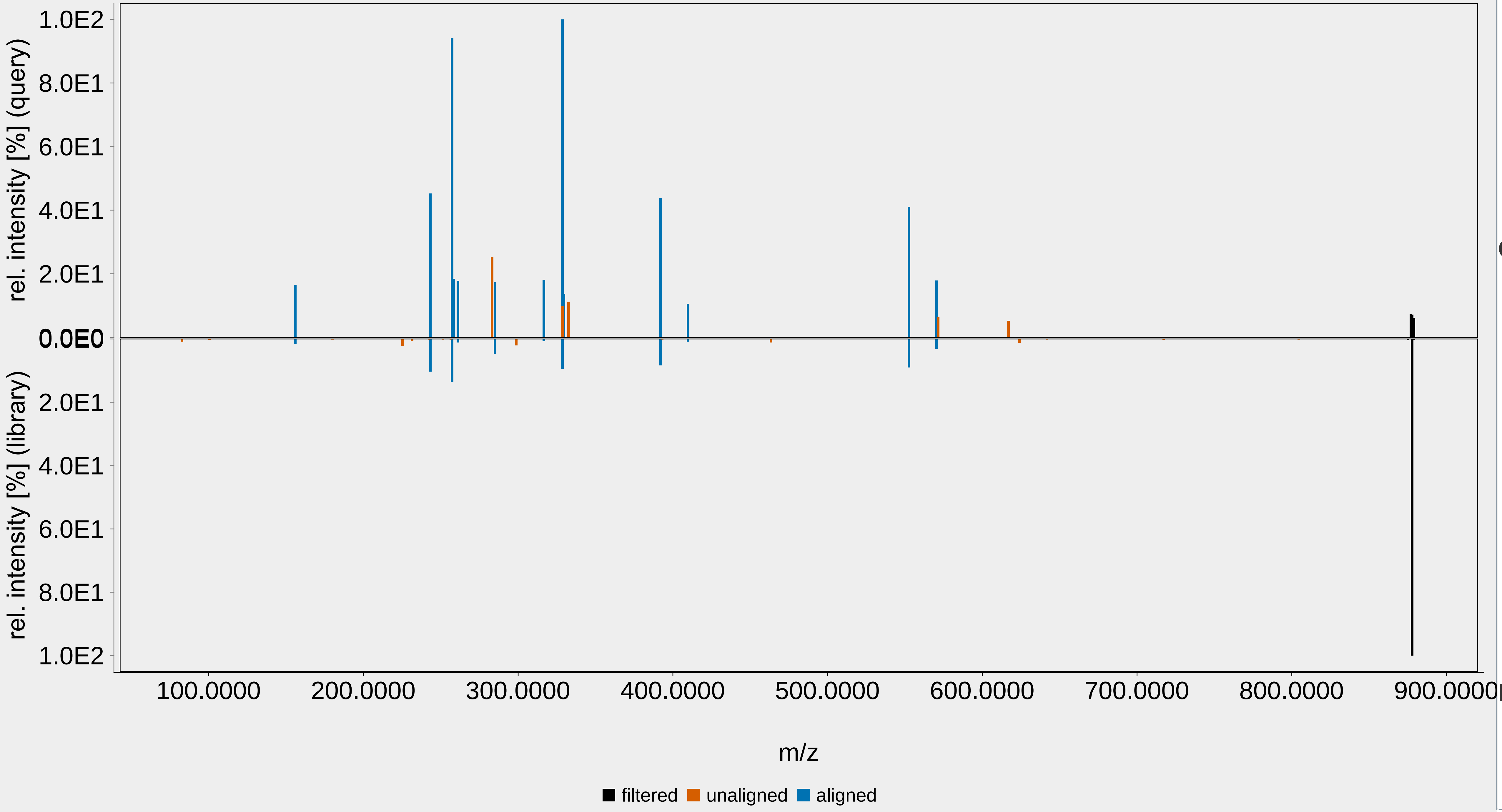

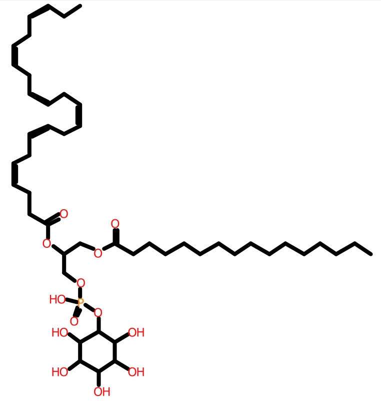

Compound information

Name: PI 38:6

Formula: C47H79O13P

Mol. weight: 883.1119999999999

Exact mass: 883.1119999999999

Ion type: [M-H]-

Precursor m/z: 881.514

RT: 8.246706

Polarity: negative

InChI:

InChI key:

SMILES:

Instrument information

Instrument type: LC-ESI-QTOF

Instrument: AB Sciex TripleTOF 5600+ system

Ms level: MS2

Collision energy: 45V

Database links

Pubchem: 138154339

MoNA ID: LQB00300

Other information

Principal investigator: Kazutaka Ikeda, Hiroshi

Data collector: Kazutaka Ikeda, Hiroshi

PI 36:4 0.864

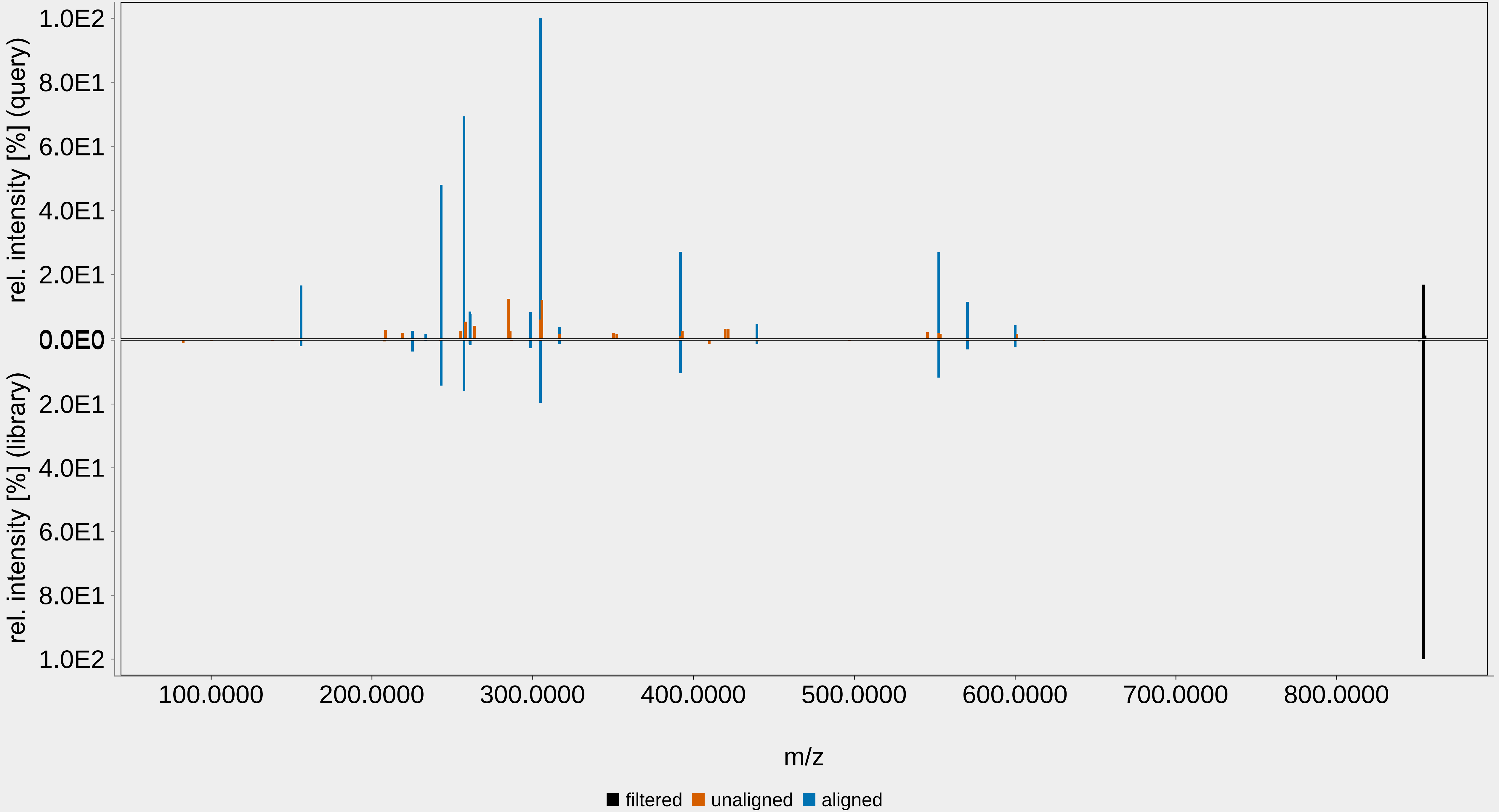

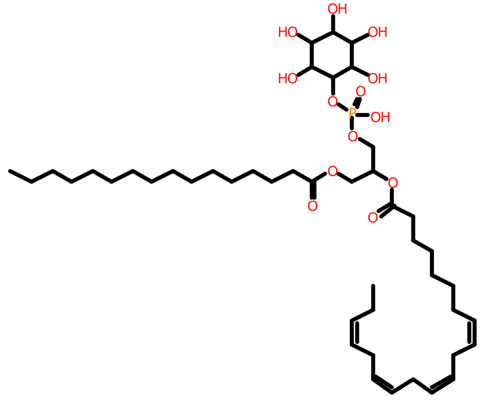

Compound information

Name: PI 36:4

Formula: C45H79O13P

Mol. weight: 859.0899999999999

Exact mass: 859.0899999999999

Ion type: [M-H]-

Precursor m/z: 857.5197

RT: 8.301669

Polarity: negative

InChI:

InChI key:

SMILES:

Instrument information

Instrument type: LC-ESI-QTOF

Instrument: AB Sciex TripleTOF 5600+ system

Ms level: MS2

Collision energy: 45V

Database links

Pubchem: 138284476

MoNA ID: LQB00683

Other information

Principal investigator: Kazutaka Ikeda, Hiroshi

Data collector: Kazutaka Ikeda, Hiroshi

PI 36:4 0.864

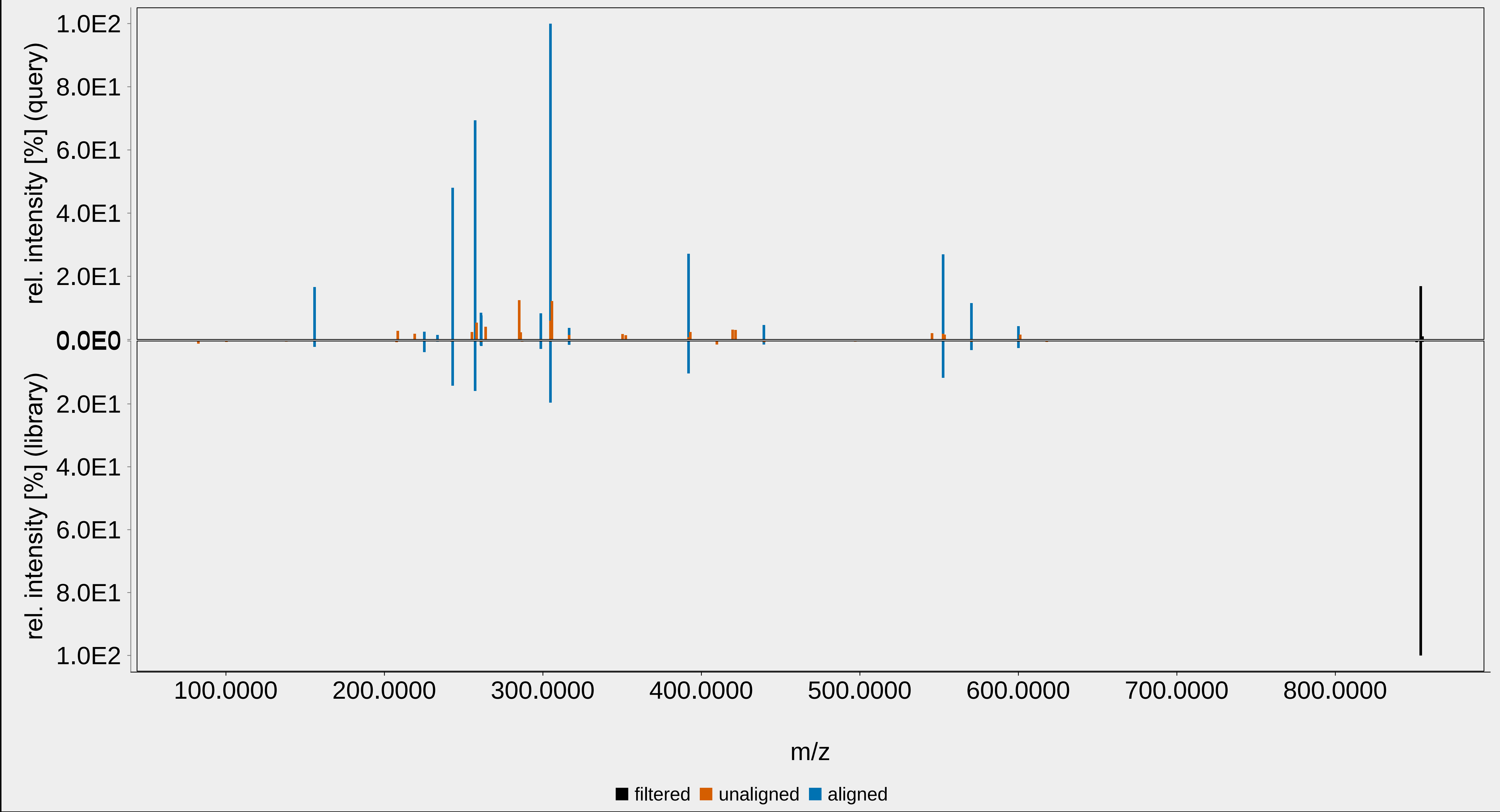

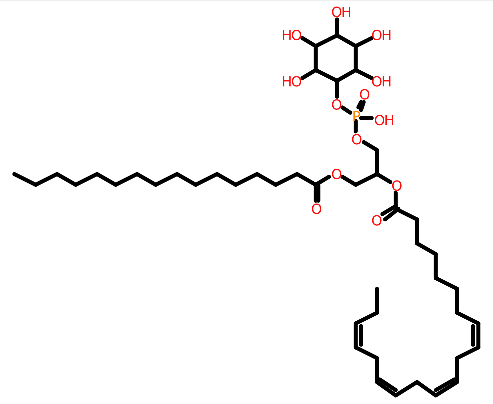

Compound information

Name: PI 36:4

Formula: C45H79O13P

Mol. weight: 859.0899999999999

Exact mass: 859.0899999999999

Ion type: [M-H]-

Precursor m/z: 857.5197

RT: 8.301669

Polarity: negative

InChI:

InChI key:

SMILES:

Instrument information

Instrument type: LC-ESI-QTOF

Instrument: AB Sciex TripleTOF 5600+ system

Ms level: MS2

Collision energy: 45V

Database links

Pubchem: 138284476

MoNA ID: LQB00297

Other information

Principal investigator: Kazutaka Ikeda, Hiroshi

Data collector: Kazutaka Ikeda, Hiroshi

Canrenone

0.939

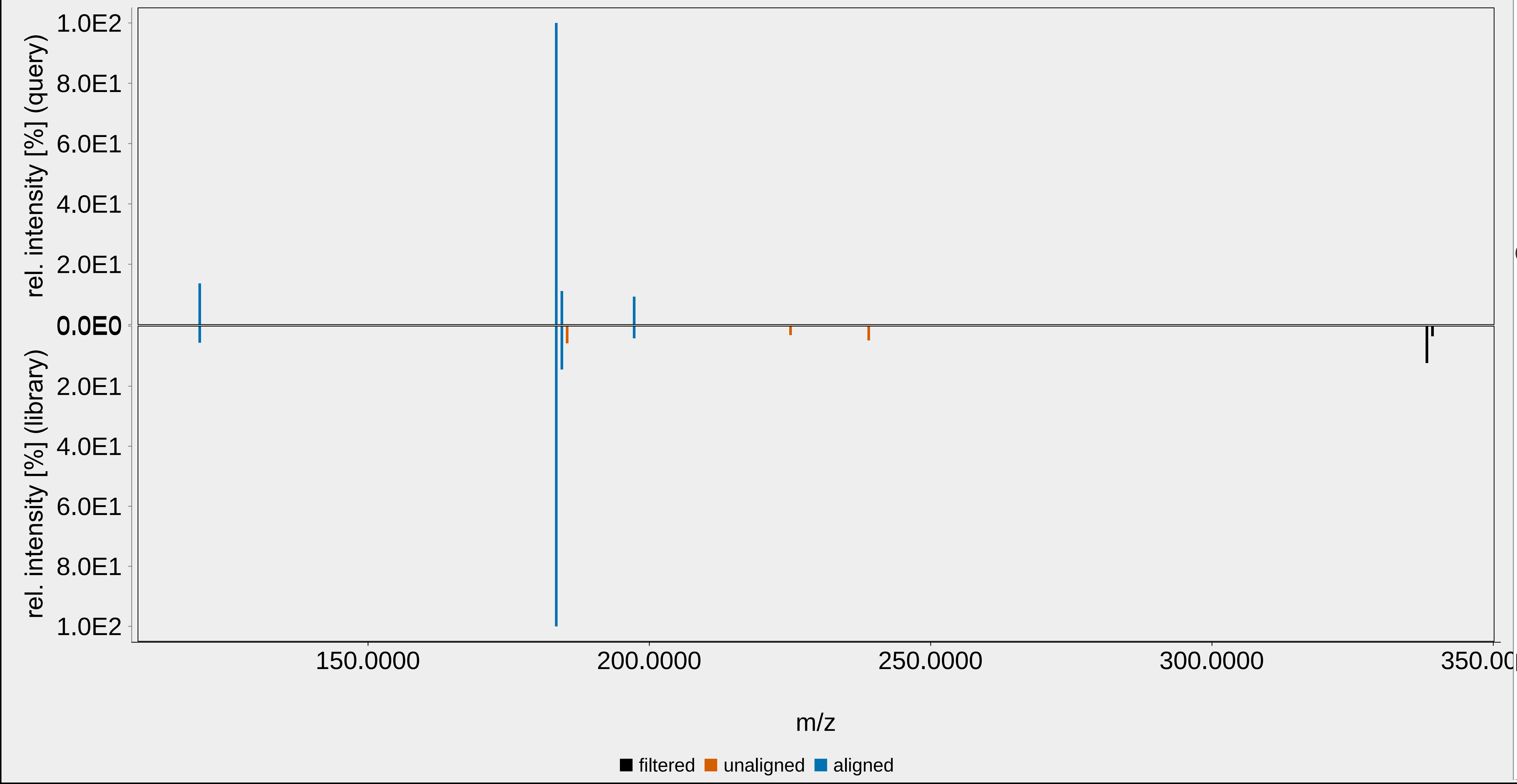

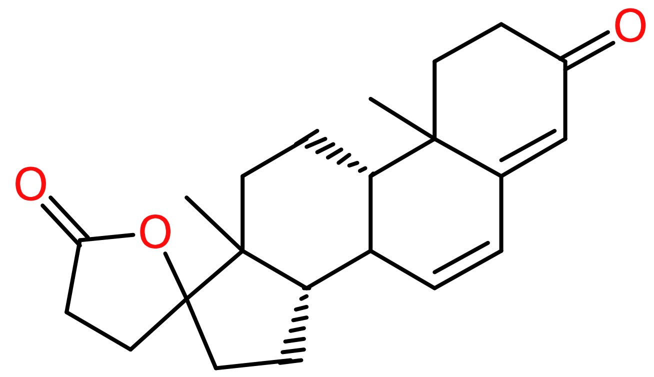

Compound information

Name: Canrenone

Formula: C22H28O3

Exact mass: 340.2038448

Ion type: [M-H]-

Precursor m/z: 339.1966

Polarity: NEGATIVE

InChI:

InChI key:

SMILES:

Num peaks: 9

Instrument information

Instrument type: LC-ESI-QTOF

Instrument: Bruker maXis Impact

Ms level: MS2

Collision energy: 40 eV

Database links

Other information

Entry ID: MSBNK-Athens_Univ-AU239460

Comment: Parent=339.1966

Canrenone, (9S,14S)-10,13-dimethylspiro[2,8,9,11,12,14,15,16-octahydro-1H-cyclopenta[a]phenanthrene-17,5`-oxolane]-2`,3-dione

0.939

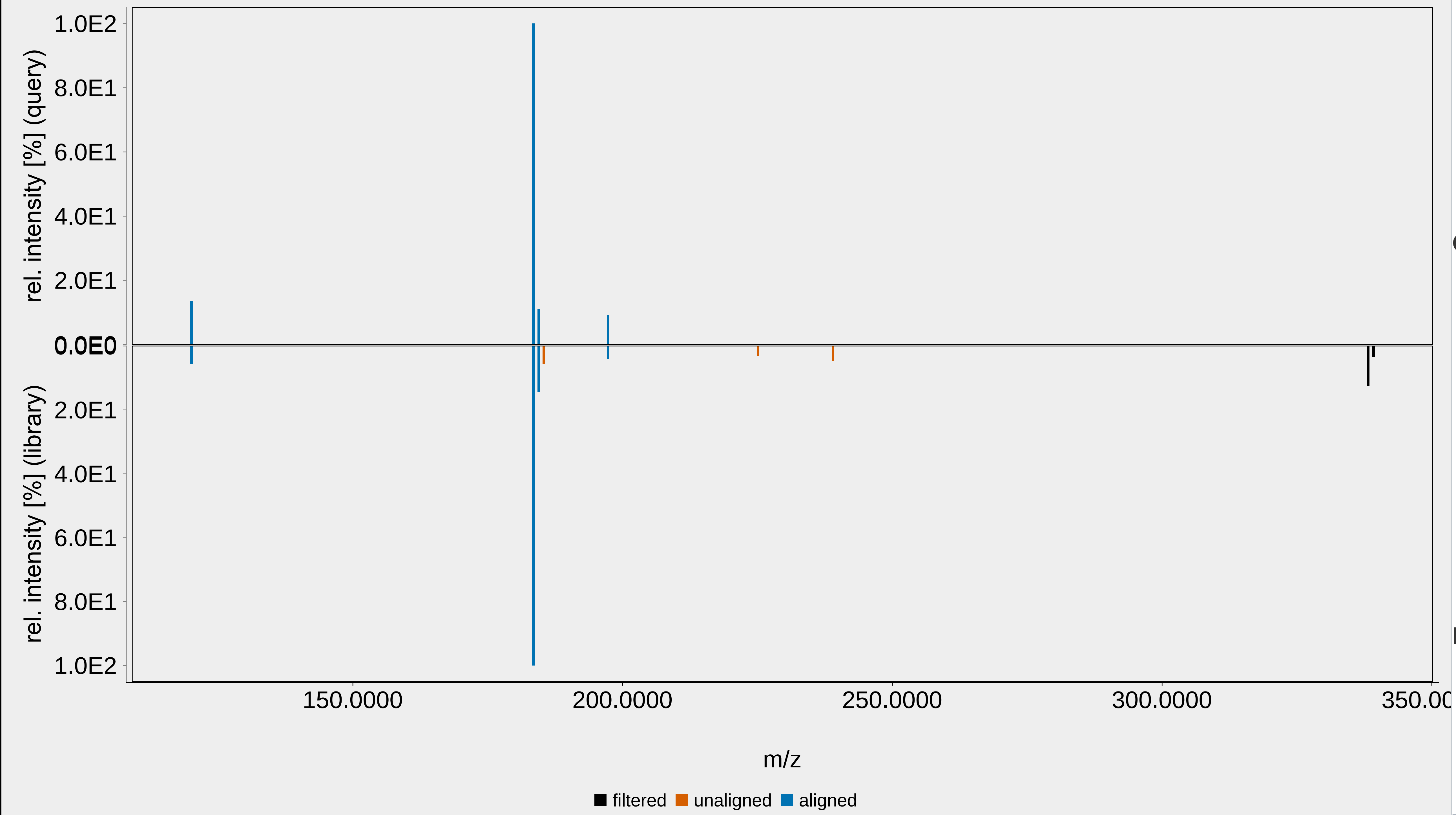

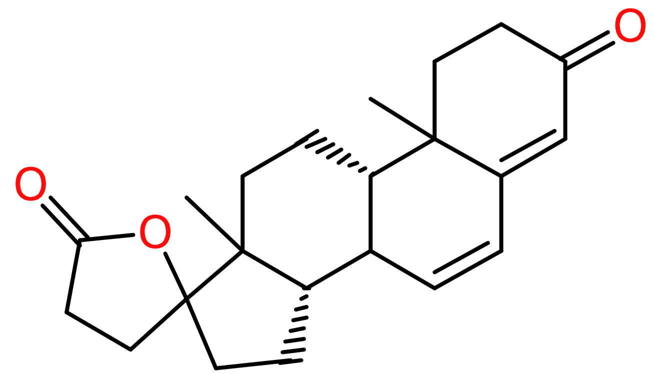

Compound information

Name: Canrenone,

Formula: C22H28O3

Mol. weight: 340.2038448

Exact mass: 340.2038448

Ion type: [M-H]-

Precursor m/z: 339.1966

Polarity: negative

InChI:

InChI key:

SMILES:

Instrument information

Instrument type: LC-ESI-QTOF

Instrument: Bruker maXis Impact

Ion source: ESI

Resolution: 35000

Ms level: MS2

Collision energy: 40 eV

Database links

Pubchem: 24728626

MoNA ID: AU239460

Chemspider: 21112020

CAS: 976-71-6

Other information

Principal investigator: Nikiforos Alygizakis,

Data collector: Nikiforos Alygizakis, Katerina

PA 18:0_22:6

0.827

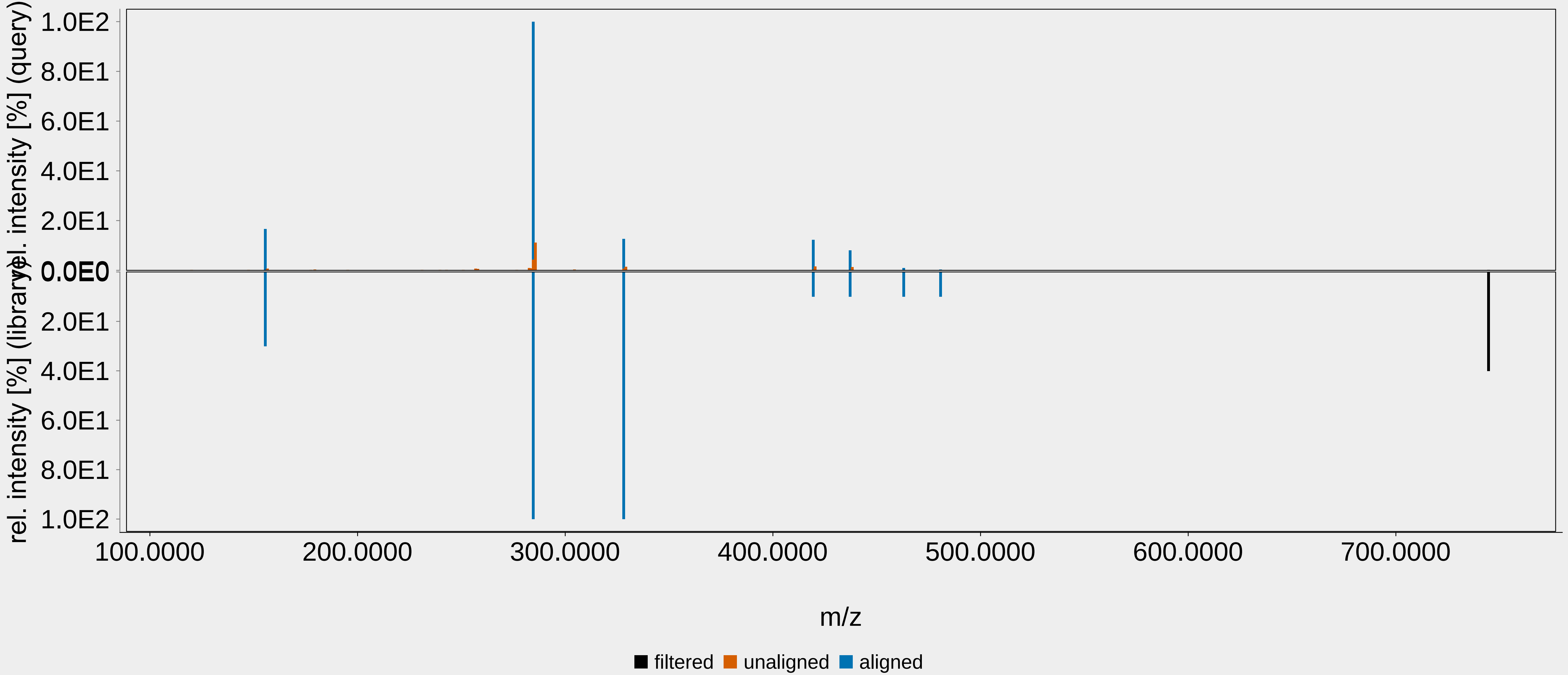

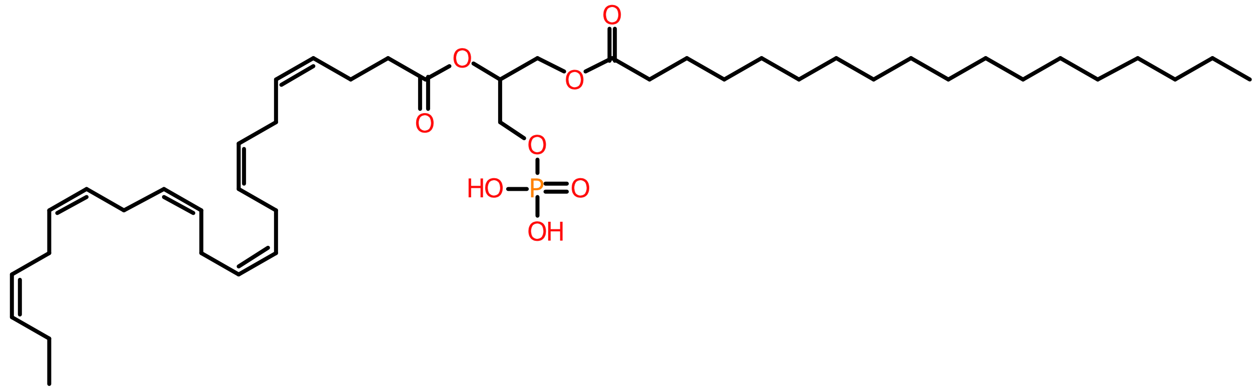

Compound information

Name: PA 18:0_22:6

Formula: C43H73O8P

Ion type: [M-H]-

Precursor m/z: 747.49703

Ccs: 266.76

InChI key:

SMILES:

Num peaks: 8

Instrument information

Database links

Other information

Spectral Match to 1-Octadecanoyl-2-(4Z,7Z,10Z,13Z,16Z,19Z-docosahexaenoyl)-sn-glycero-3-phospho-L-serine from NIST14

0.881

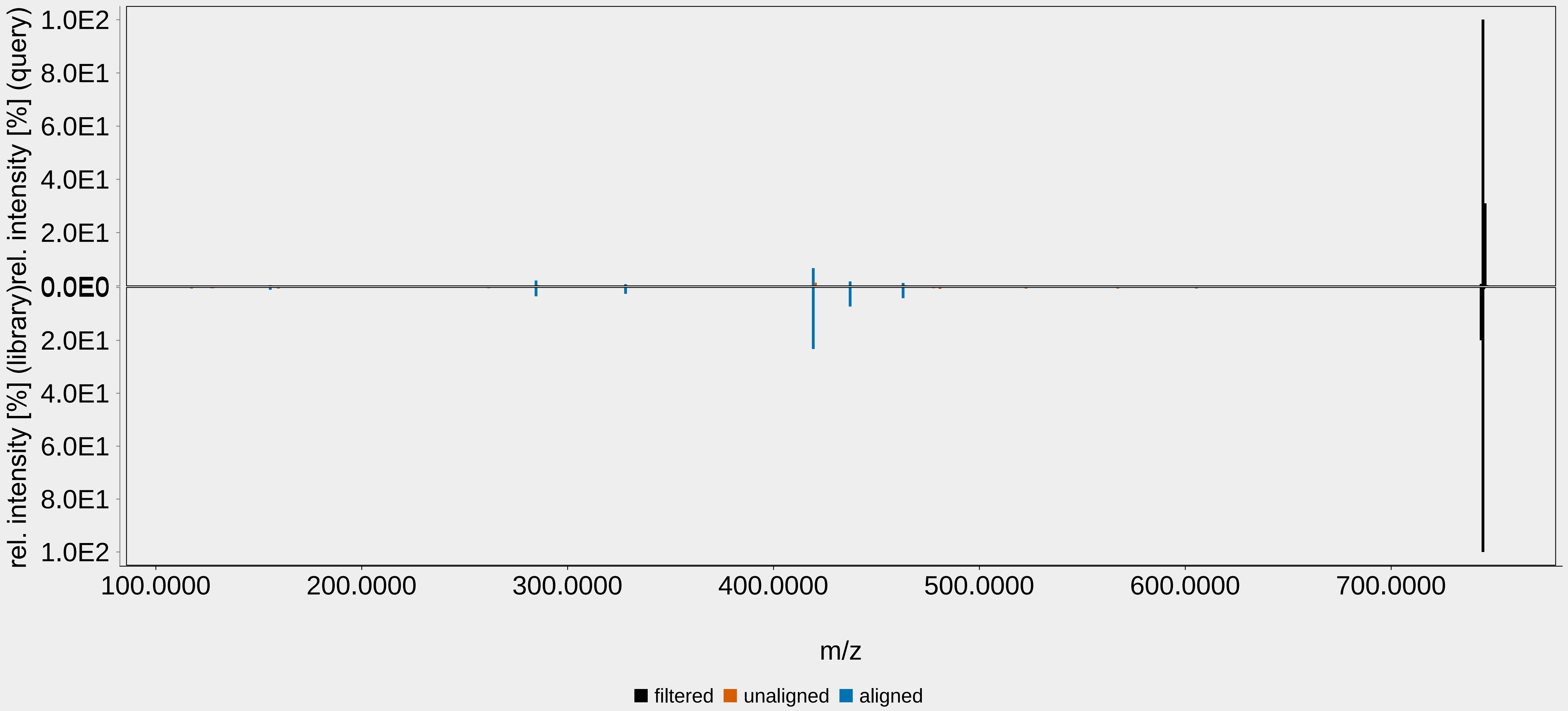

Compound information

Name: Spectral Match to

Exact mass: 0.0

Ion type: M-H-C3H5NO2

Precursor m/z: 747.498

Charge: 1

Polarity: Negative

Instrument information

Instrument type: IT/ion trap

Ion source: ESI

Ms level: 2

Acquisition: Isolated

Database links

CAS: 137173989

Other information

Principal investigator: Data from Katrina

Data collector: Data deposited by alchemistmatt

Entry ID: CCMSLIB00003134591

[1-[[2,3-dihydroxypropoxy]-hydroxyphosphoryl]oxy-3-[octadec-11-enoyl]oxypropan-2-yl] octadecanoate

0.758

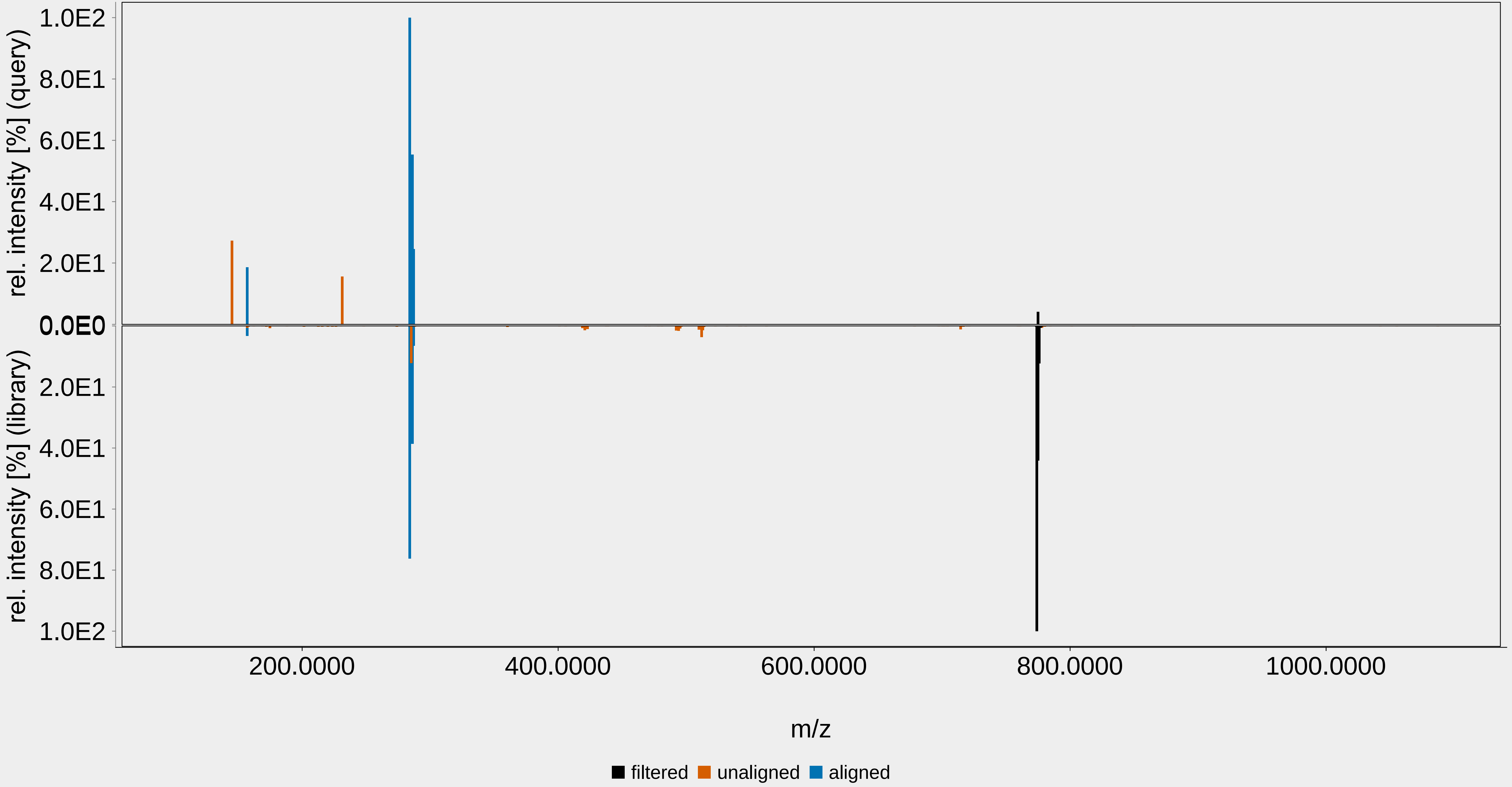

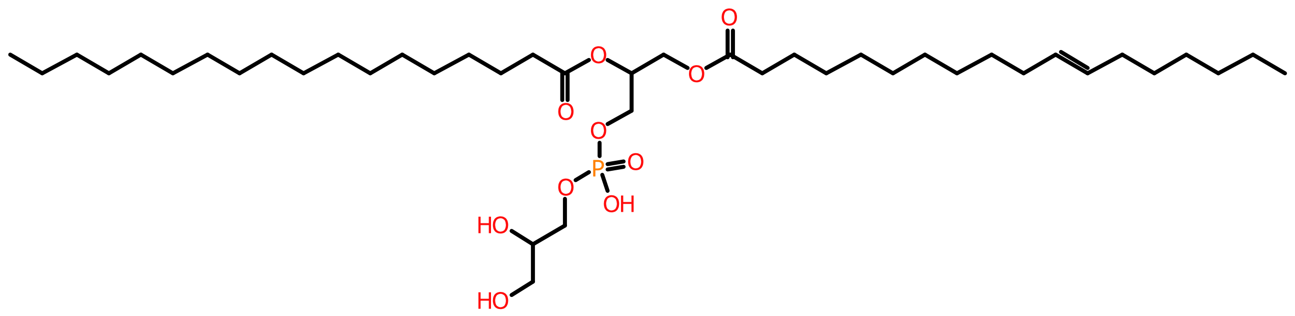

Compound information

Name:

Formula: C42H81O10P

Mol. weight: 776.557

Exact mass: 776.557

Ion type: [M-H]-

Precursor m/z: 775.549

Polarity: negative

InChI:

InChI key:

SMILES:

Instrument information

Instrument: qTof

Ms level: MS2

Database links

Other information

Principal investigator: O Laprevote, UMR 8038

Data collector: O Laprevote, UMR 8038

PG(18:0_18:1) - [2.3-dihydroxypropoxy][2-[octadec-9-enoyloxy]-3-(octadecanoyloxy)propoxy]phosphinic acid

0.775

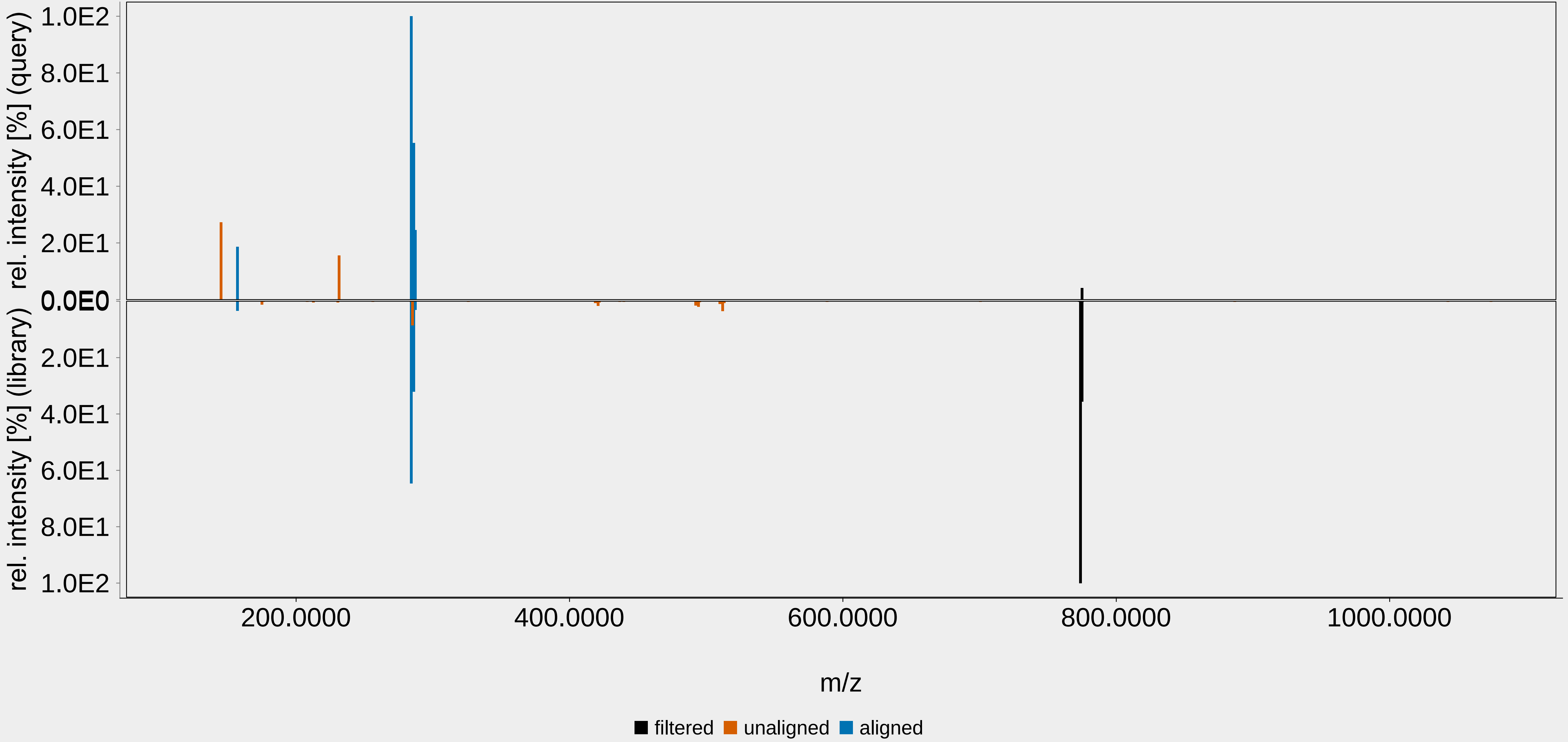

Compound information

Name: PG(18:0_18:1) -

Exact mass: 776.557

Ion type: M-H

Precursor m/z: 775.547

Charge: 1

Polarity: Negative

SMILES:

Instrument information

Instrument type: qTof

Ion source: LC-ESI

Ms level: 2

Acquisition: Lysate

Database links

Other information

Principal investigator: N Auzeil

Data collector: UMR CNRS 8038 Universite de

Entry ID: CCMSLIB00005724243

uridine 5?-diphosphate - 30.0 eV

0.882

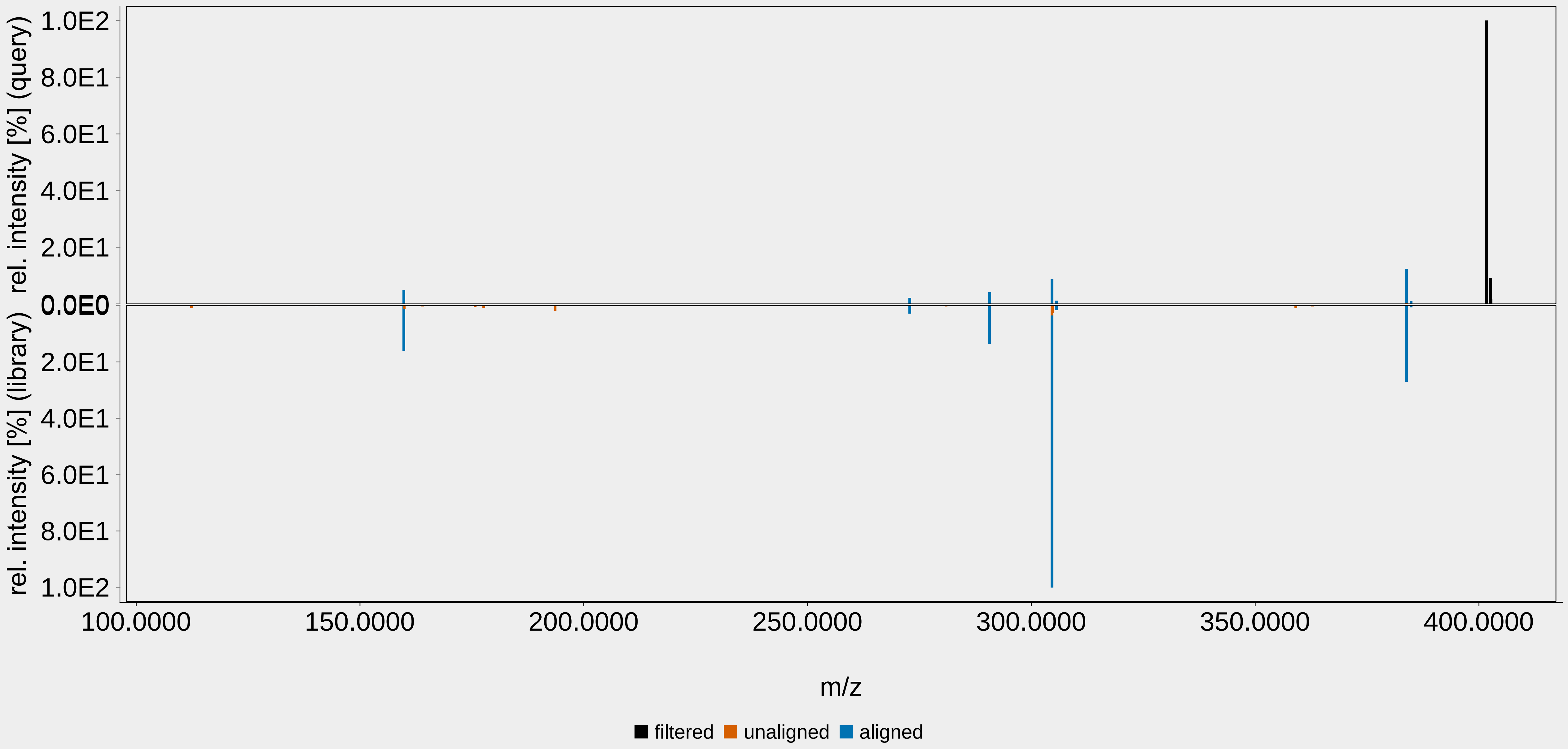

Compound information

Name: uridine 5?-diphosphate - 30.0 eV

Exact mass: 0.0

Ion type: M-H

Precursor m/z: 402.995

Charge: 1

Polarity: Negative

SMILES:

Instrument information

Instrument type: Orbitrap

Ion source: ESI

Ms level: 2

Acquisition: Isolated

Database links

CAS:

Other information

Principal investigator: PI

Data collector: Data Collector

Entry ID: CCMSLIB00006123791

uridine 5?-diphosphate - 30.0 eV

0.899

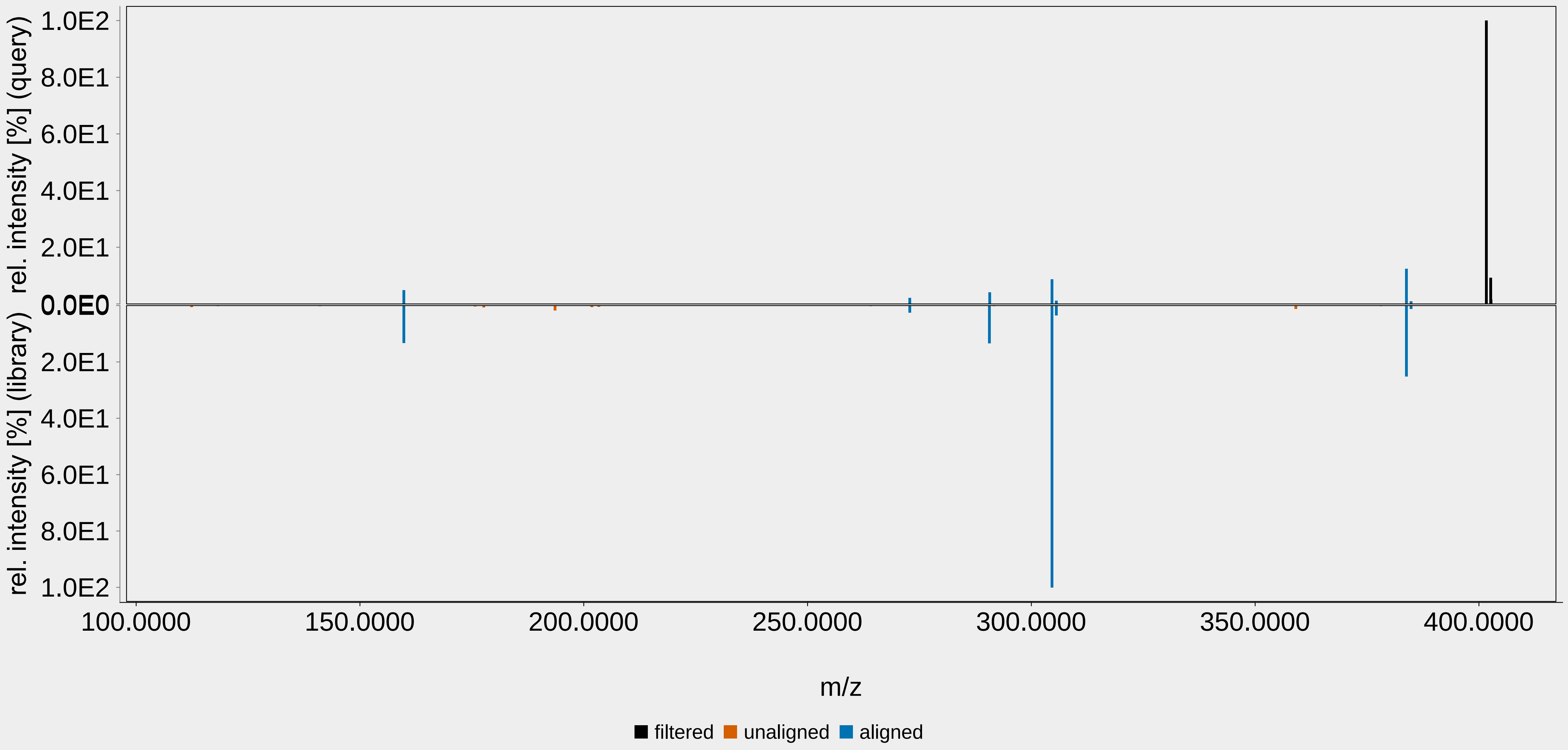

Compound information

Name: uridine 5?-diphosphate - 30.0 eV

Exact mass: 0.0

Ion type: M-H

Precursor m/z: 402.995

Charge: 1

Polarity: Negative

SMILES:

Instrument information

Instrument type: Orbitrap

Ion source: ESI

Ms level: 2

Acquisition: Isolated

Database links

CAS:

Other information

Principal investigator: PI

Data collector: Data Collector

Entry ID: CCMSLIB00006123813

PS(22:6/22:6); [M-H]- C50H73N1O10P1 0.760

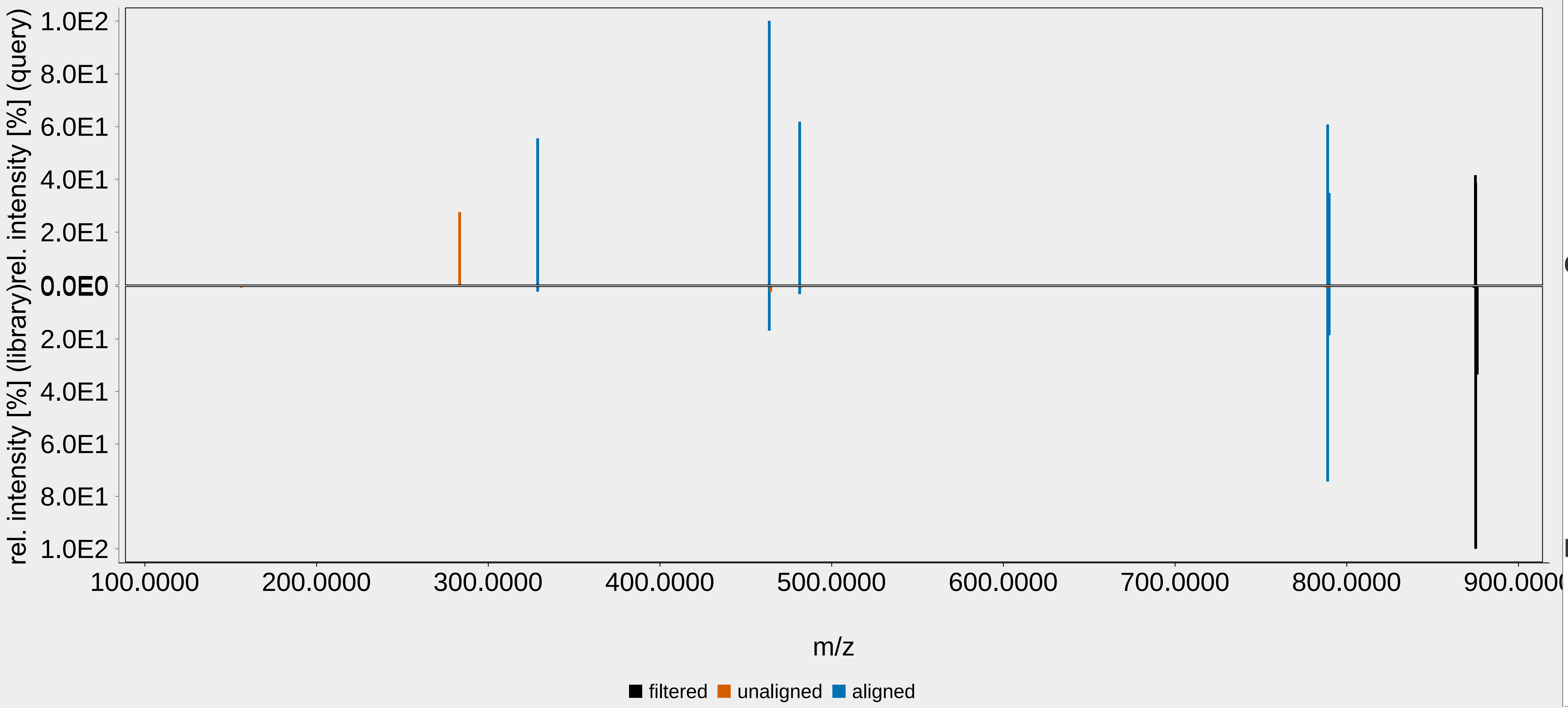

Compound information

Name: PS(22:6/22:6); [M-H]- C50H73N1O10P1

Exact mass: 0.0

Ion type: M-H

Precursor m/z: 878.497

Charge: 1

Polarity: Negative

Instrument information

Instrument type: HCD; Velos

Ion source: LC-ESI

Ms level: 2

Acquisition: Commercial

Database links

Other information

Principal investigator: Thomas Metz

Data collector: Thomas Metz

Entry ID: CCMSLIB00010083009

PS 22:6_22:6 0.940

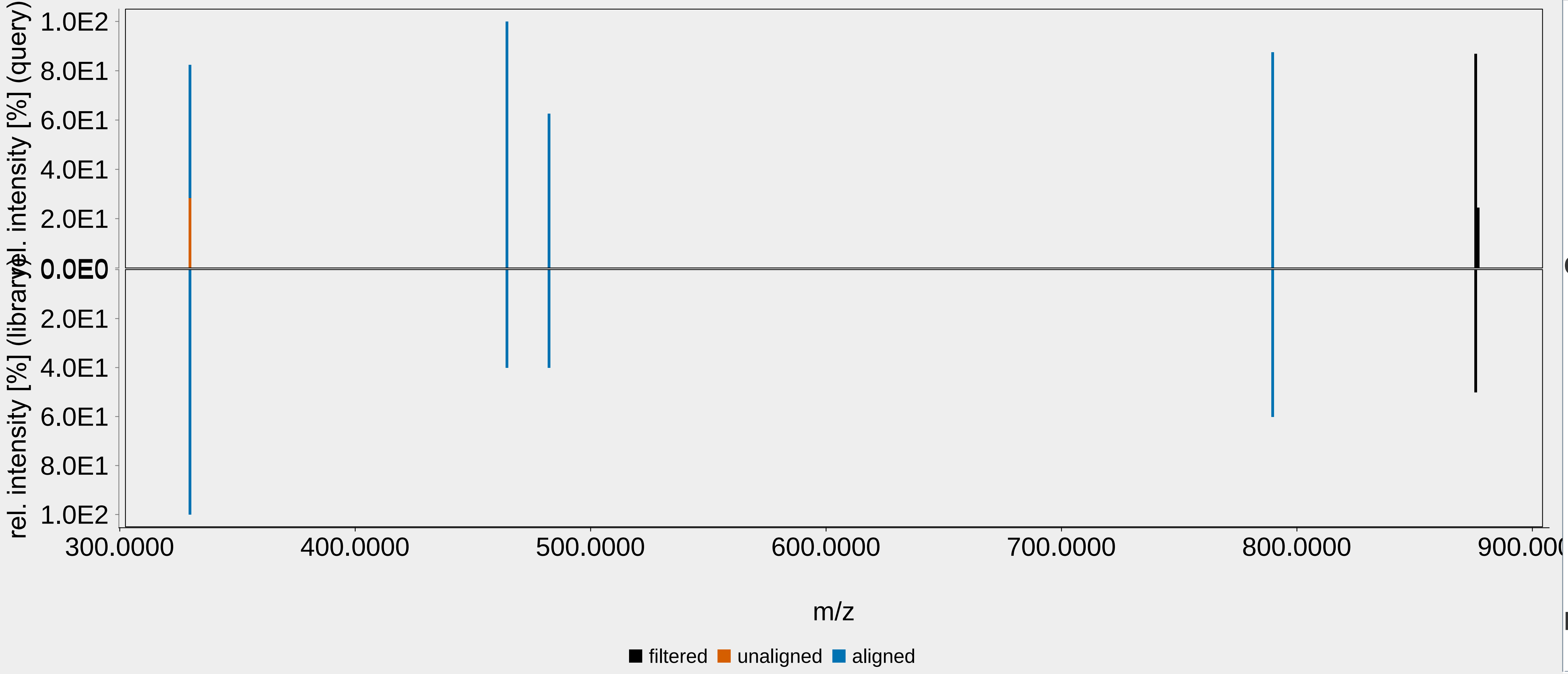

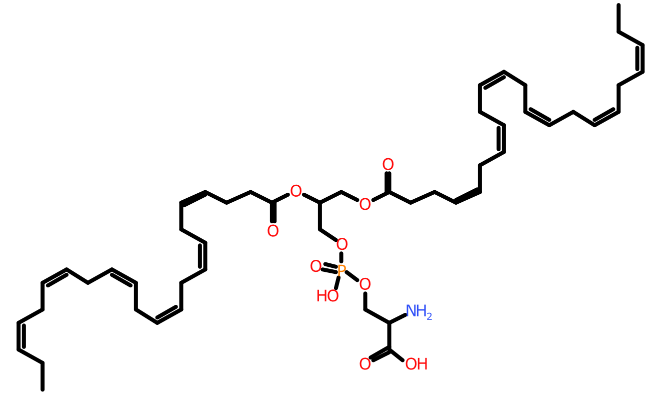

Compound information

Name: PS 22:6_22:6

Formula: C50H74NO10P

Ion type: [M-H]-

Precursor m/z: 878.49776

Ccs: 284.65

InChI key:

SMILES:

Num peaks: 5

Instrument information

Database links

Other information

PS 18:1_22:1 **0.787**

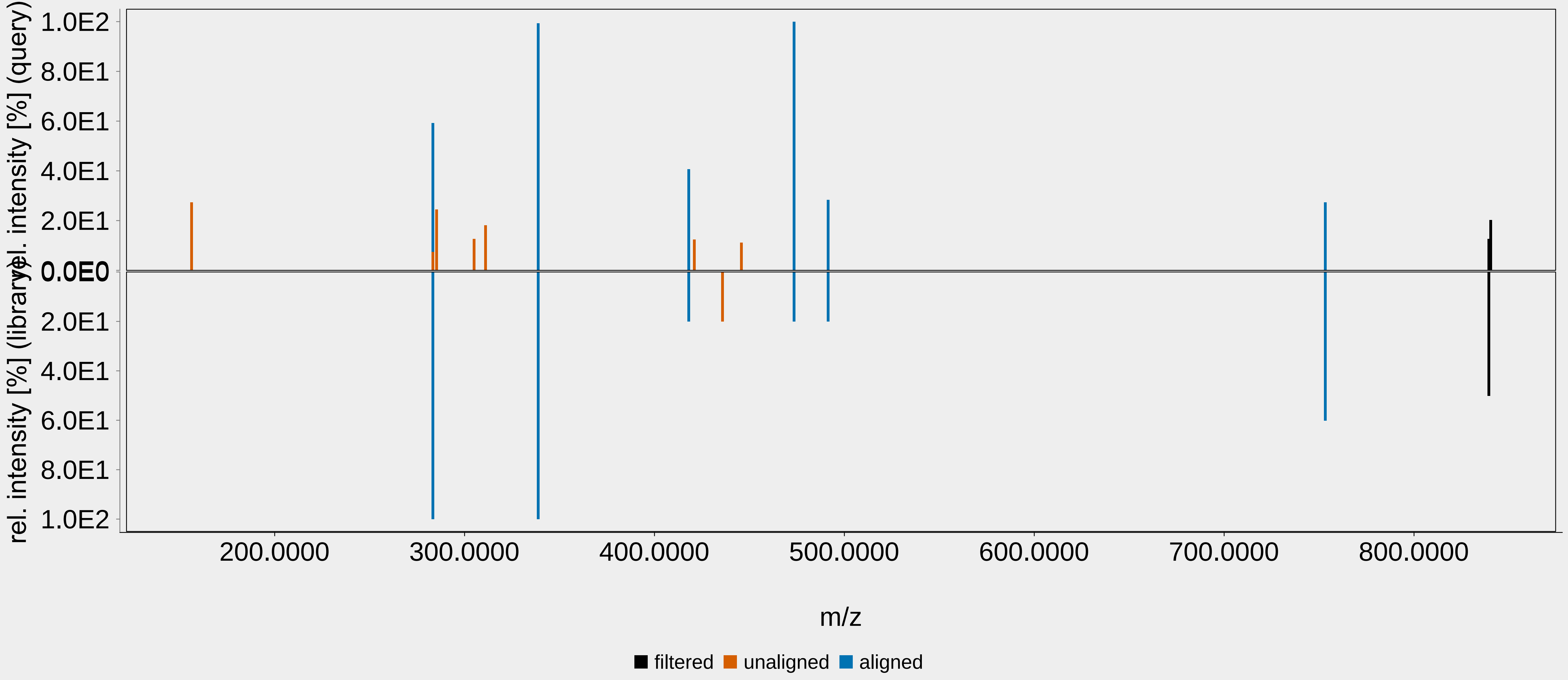

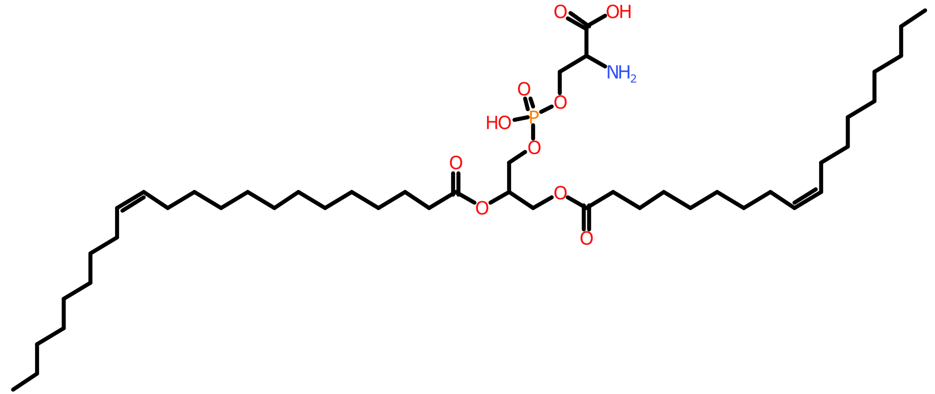

Compound information

Name: PS 18:1_22:1

Formula: C46H86NO10P

Ion type: [M-H]-

Precursor m/z: 842.59166

Ccs: 287.03

InChI key:

SMILES:

Num peaks: 8

Instrument information

Database links

Other information

PA 18:1_20:1 **0.720**

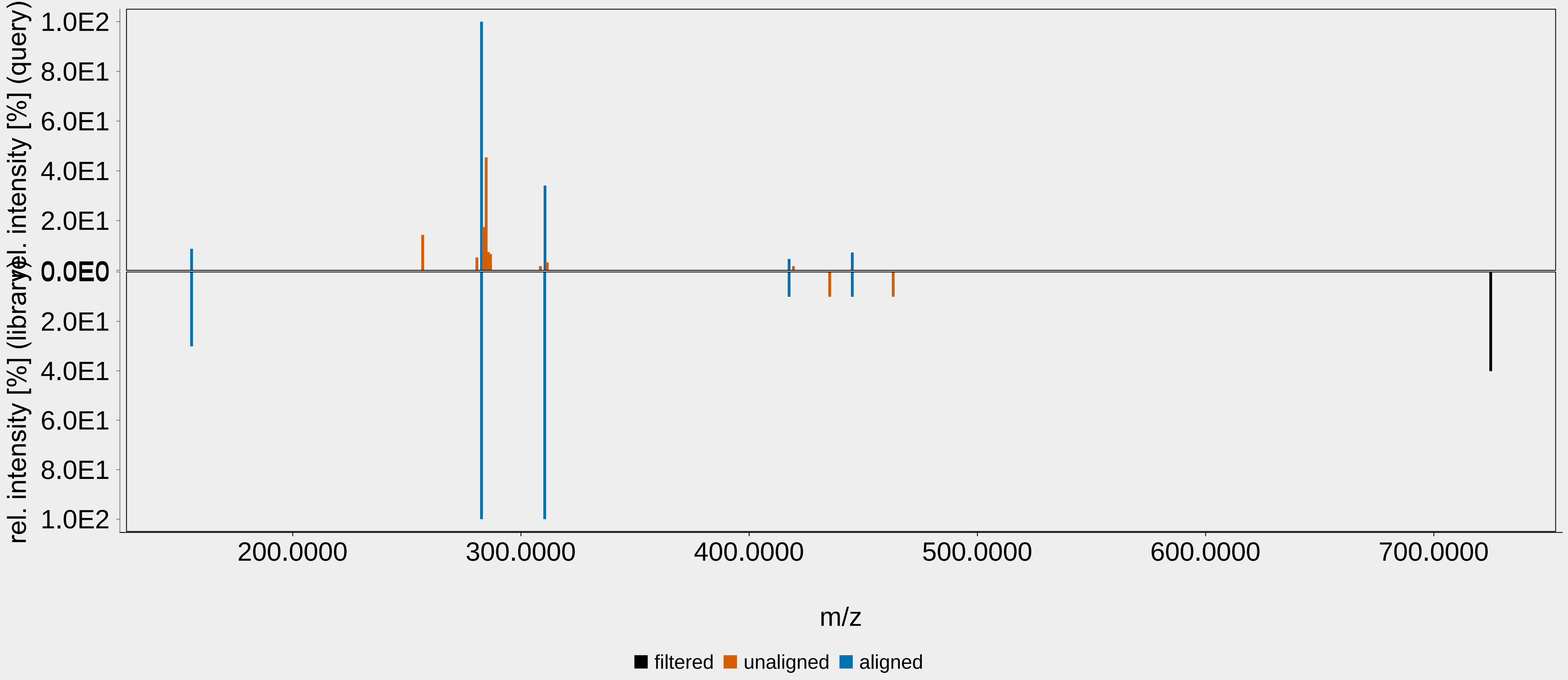

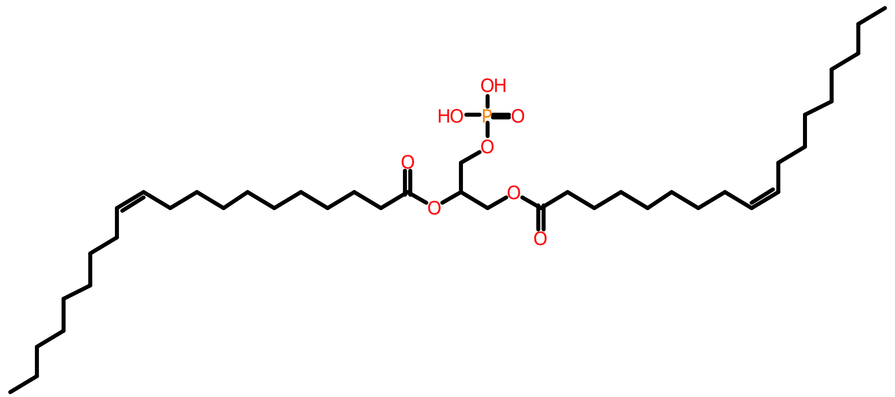

Compound information

Name: PA 18:1_20:1

Formula: C41H77O8P

Ion type: [M-H]-

Precursor m/z: 727.52833

Ccs: 265.22

InChI key:

SMILES:

Num peaks: 8

Instrument information

Database links

Other information

PG(16:1/18:1); [M-H]- C40H74O10P1 **0.779**

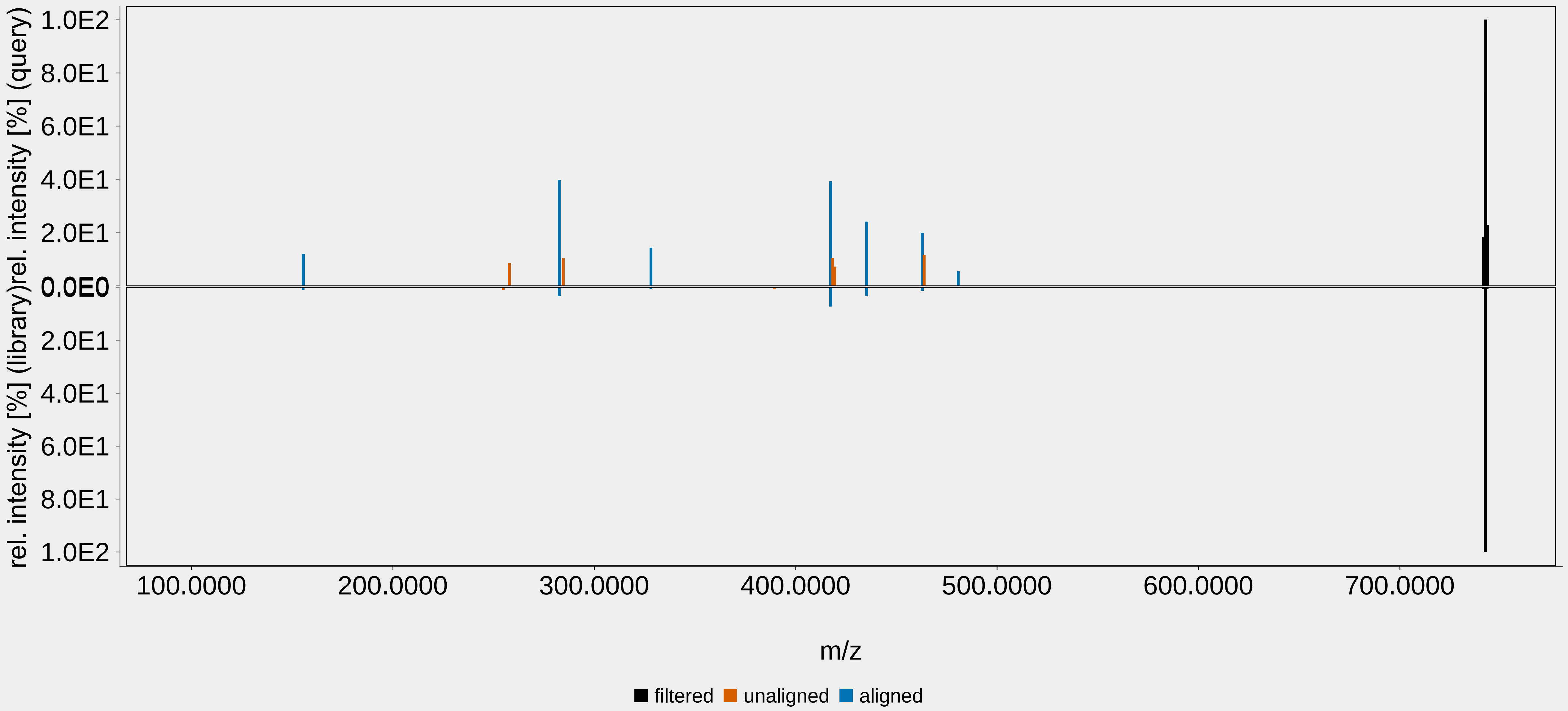

Compound information

Name: PG(16:1/18:1); [M-H]- C40H74O10P1

Exact mass: 0.0

Ion type: M-H

Precursor m/z: 745.502

Charge: 1

Polarity: Negative

Instrument information

Instrument type: HCD; Velos

Ion source: LC-ESI

Ms level: 2

Acquisition: Commercial

Database links

Other information

Principal investigator: Thomas Metz

Data collector: Thomas Metz

Entry ID: CCMSLIB00010087352

PG(16:1/18:1); [M-H]- C40H74O10P1 **0.782**

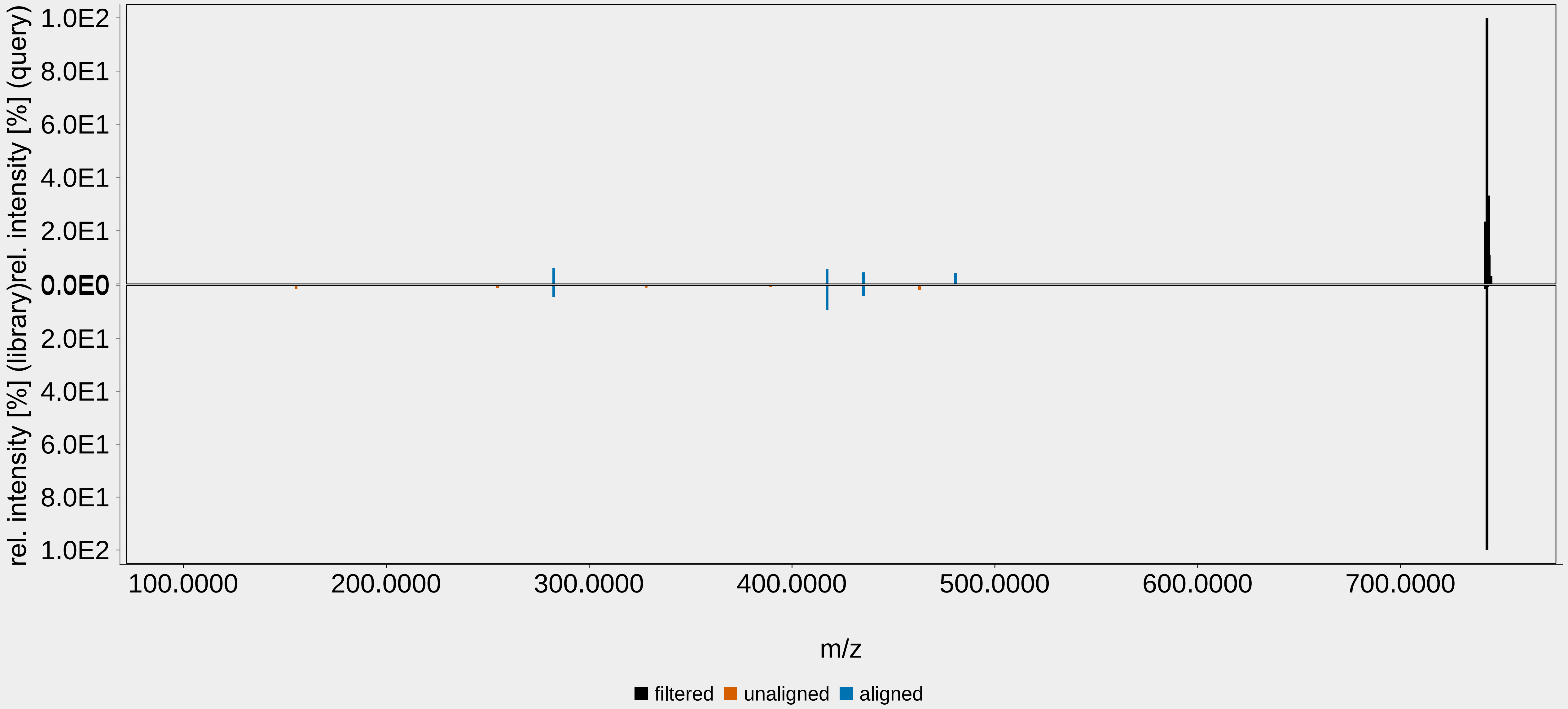

Compound information

Name: PG(16:1/18:1); [M-H]- C40H74O10P1

Exact mass: 0.0

Ion type: M-H

Precursor m/z: 745.502

Charge: 1

Polarity: Negative

Instrument information

Instrument type: HCD; Velos

Ion source: LC-ESI

Ms level: 2

Acquisition: Commercial

Database links

Other information

Principal investigator: Thomas Metz

Data collector: Thomas Metz

Entry ID: CCMSLIB00010073906

PS 40:4

0.883

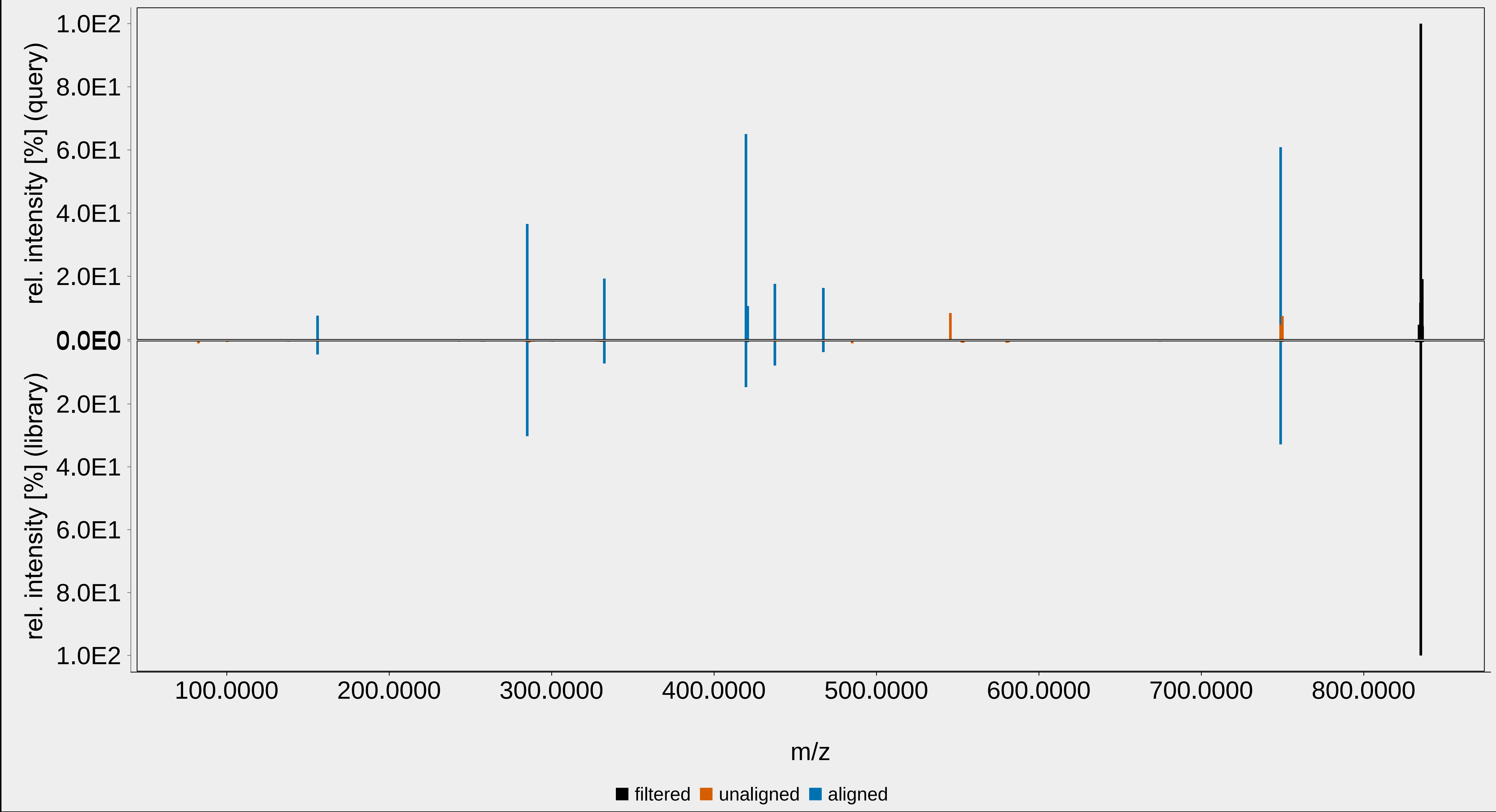

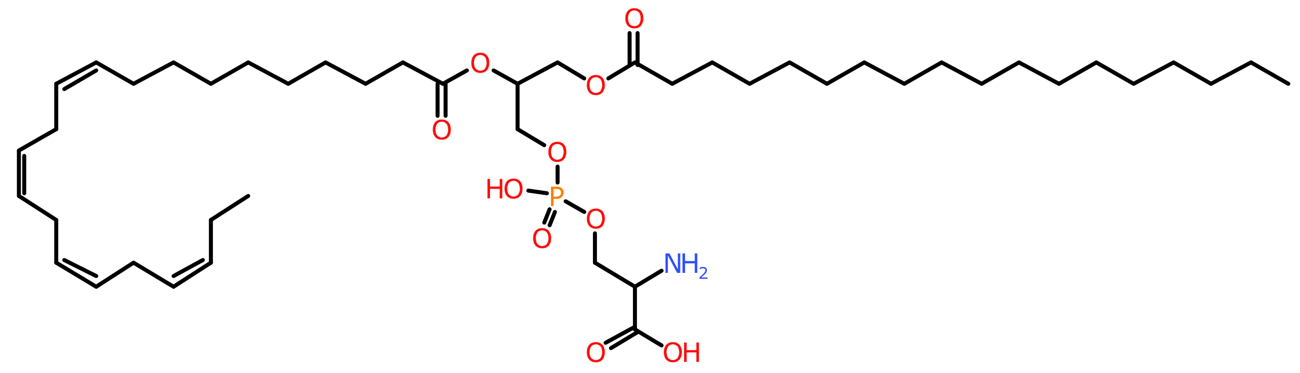

Compound information

Name: PS 40:4

Formula: C46H82NO10P

Mol. weight: 840.135

Exact mass: 840.135

Ion type: [M-H]-

Precursor m/z: 838.5547

RT: 9.189651

Polarity: negative

InChI:

InChI key:

SMILES:

Instrument information

Instrument type: LC-ESI-QTOF

Instrument: AB Sciex TripleTOF 5600+ system

Ms level: MS2

Collision energy: 45V

Database links

Pubchem: 138202734

MoNA ID: LQB00724

Other information

Principal investigator: Kazutaka Ikeda, Hiroshi

Data collector: Kazutaka Ikeda, Hiroshi

PS 40:4

0.883

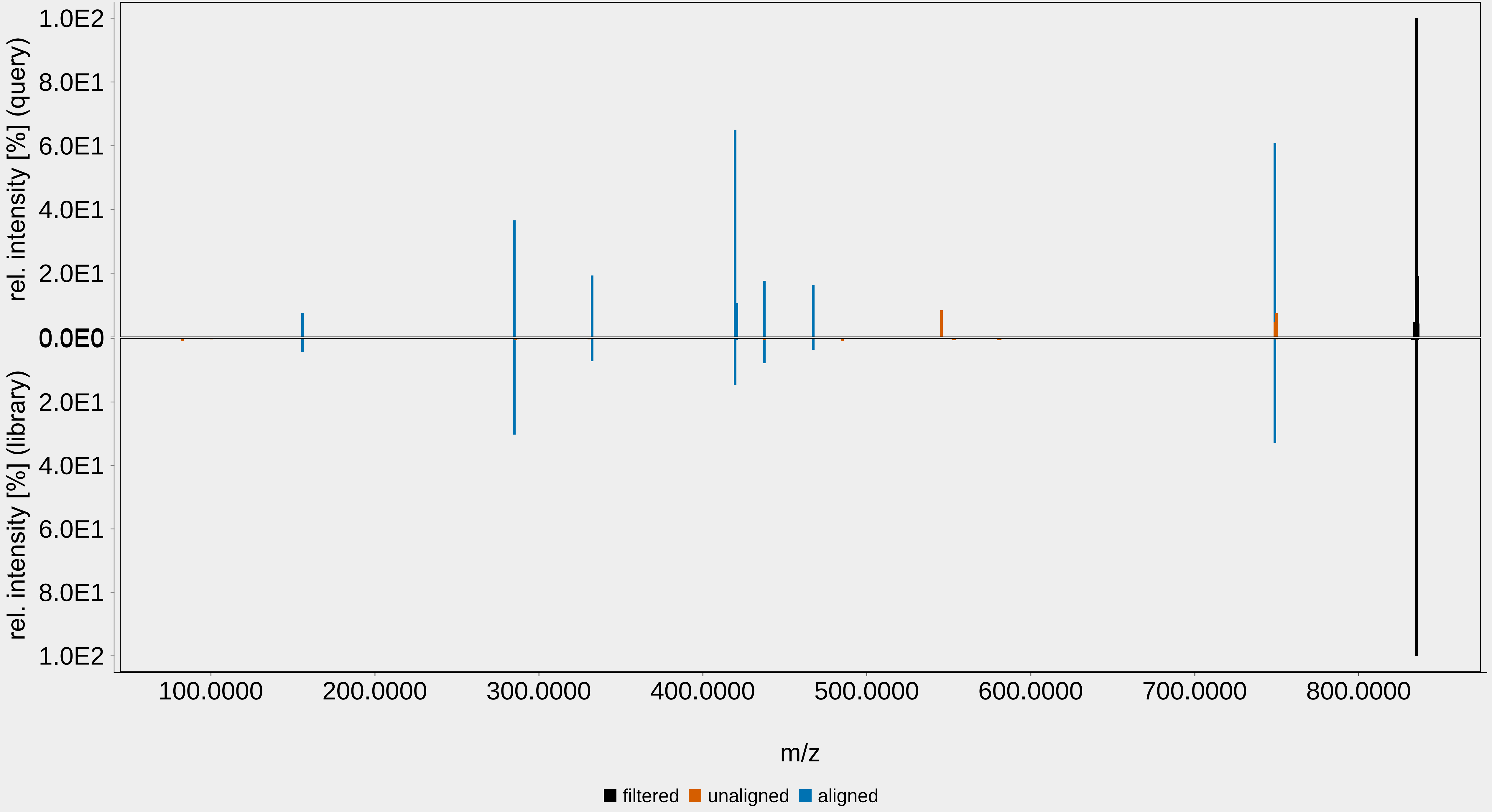

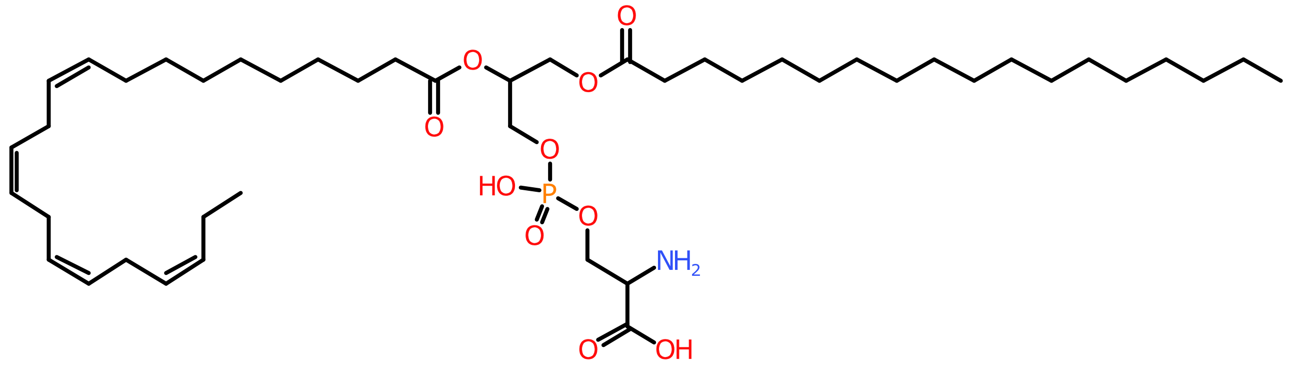

Compound information

Name: PS 40:4

Formula: C46H82NO10P

Mol. weight: 840.135

Exact mass: 840.135

Ion type: [M-H]-

Precursor m/z: 838.5547

RT: 9.189651

Polarity: negative

InChI:

InChI key:

SMILES:

Instrument information

Instrument type: LC-ESI-QTOF

Instrument: AB Sciex TripleTOF 5600+ system

Ms level: MS2

Collision energy: 45V

Database links

Pubchem: 138202734

MoNA ID: LQB00338

Other information

Principal investigator: Kazutaka Ikeda, Hiroshi

Data collector: Kazutaka Ikeda, Hiroshi

PHYTIC ACID

0.904

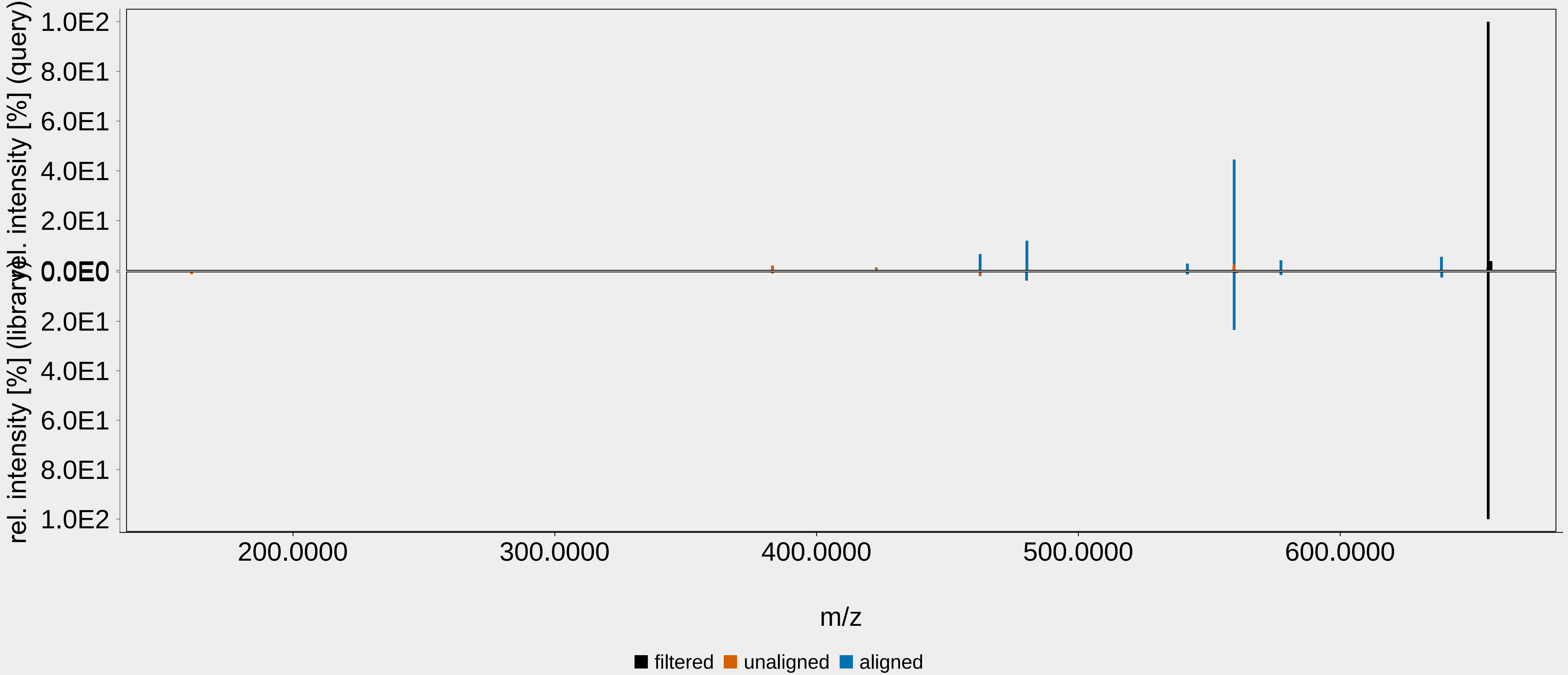

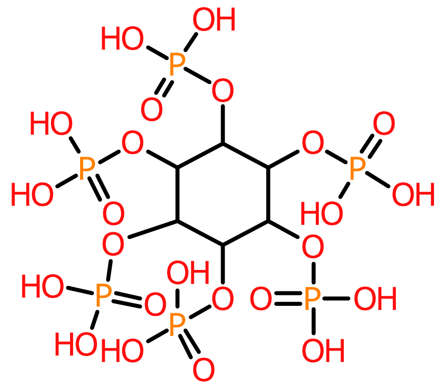

Compound information

Name: PHYTIC ACID

Formula: C6H18O24P6

Ion type: [M-H]-

Precursor m/z: 658.8540952

Polarity: negative

InChI:

InChI key: IMQLKJBTEOYOSI-UHFFFAOYSA-I

SMILES:

Instrument information

Instrument type: LC-ESI-QTOF

Instrument: Agilent qTOF 6545

Ms level: MS2

Collision energy: 20 eV

Database links

Other information

PHYTIC ACID 0.904

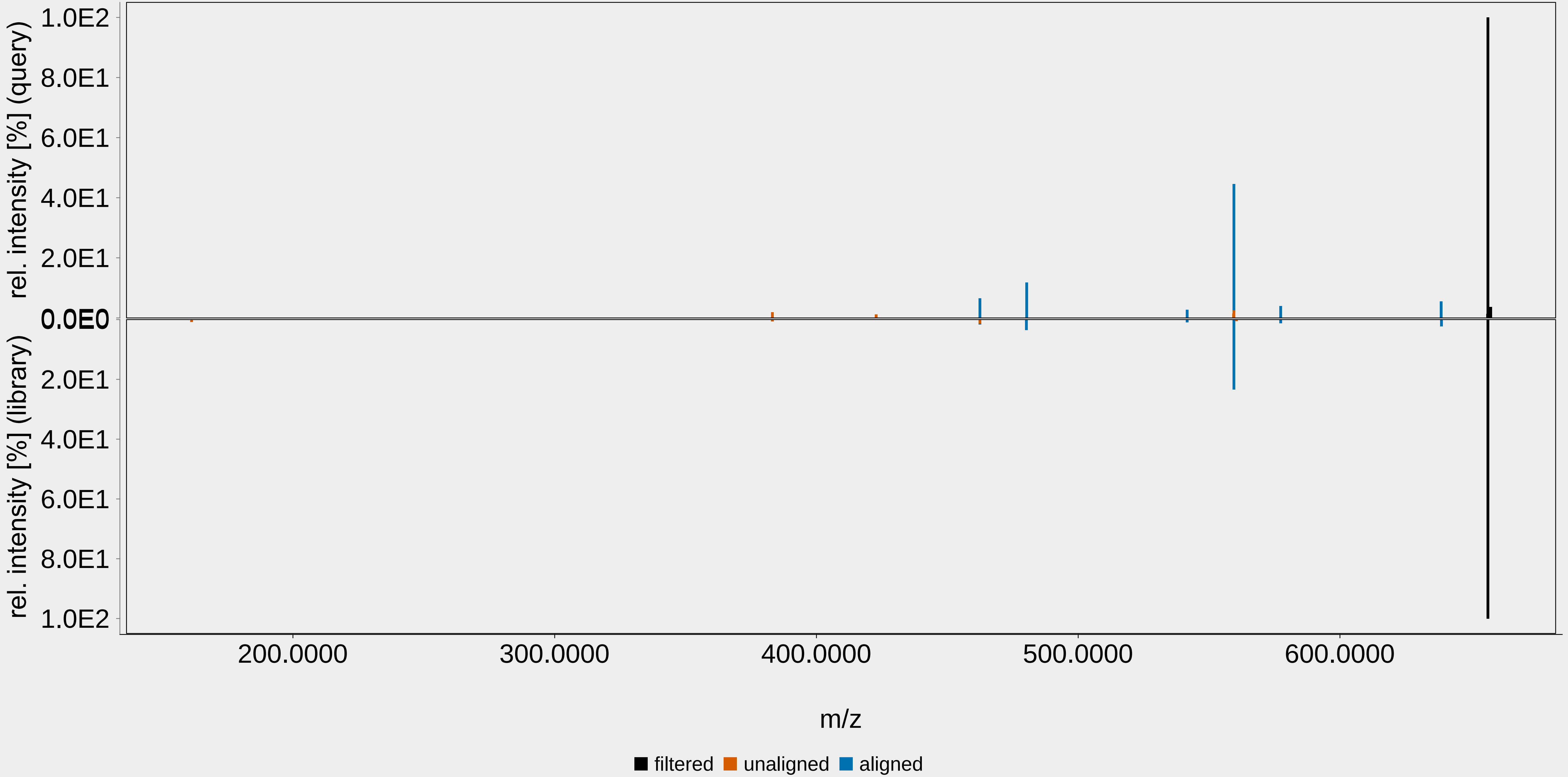

Compound information

Name: PHYTIC ACID

Exact mass: 0.0

Ion type: M-H

Precursor m/z: 658.854

Charge: 1

Polarity: negative

InChI:

SMILES:

Instrument information

Instrument type: qTof

Ion source: ESI

Ms level: 2

Acquisition: isolated

Database links

Other information

Principal investigator: MoNA

Data collector: MoNA:MoNA034158

Entry ID: CCMSLIB00006685256

PA 18:2_22:6 0.859

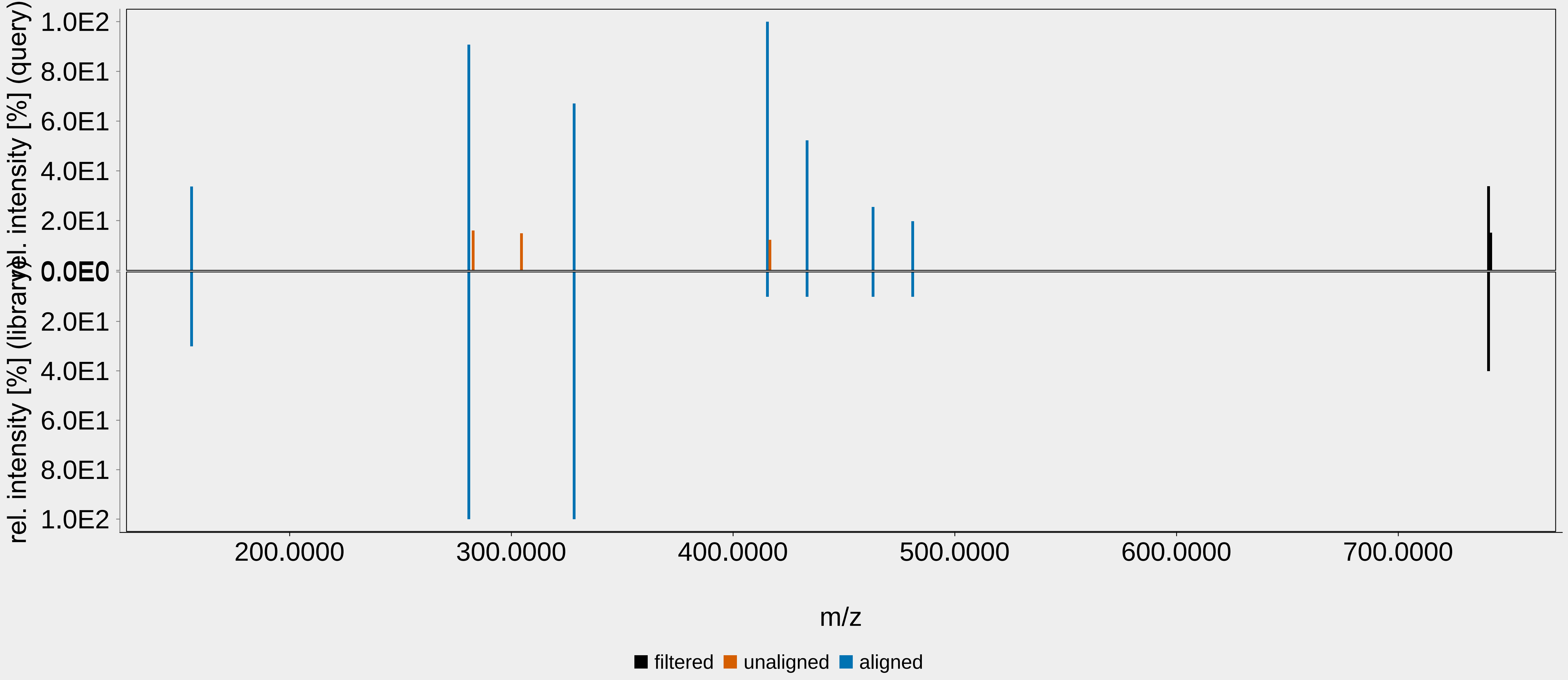

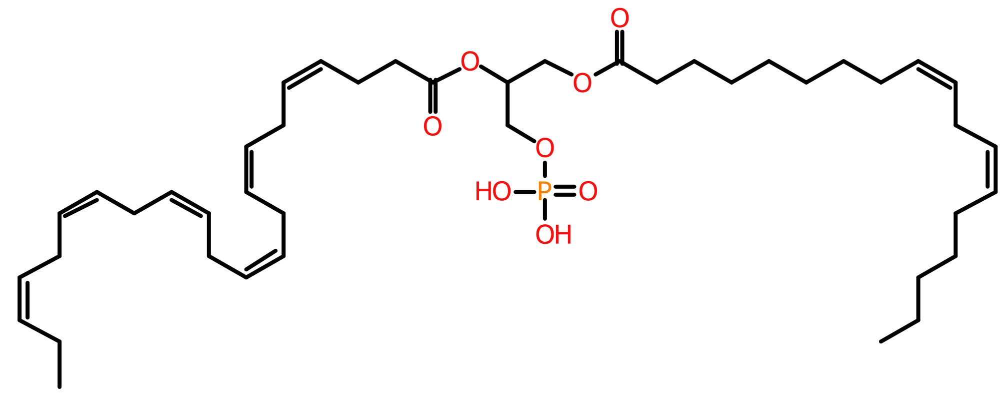

Compound information

Name: PA 18:2_22:6

Formula: C43H69O8P

Ion type: [M-H]-

Precursor m/z: 743.46573

Ccs: 264.57

InChI key:

SMILES:

Num peaks: 8

Instrument information

Database links

Other information

PA 18:1_22:6 **0.796**

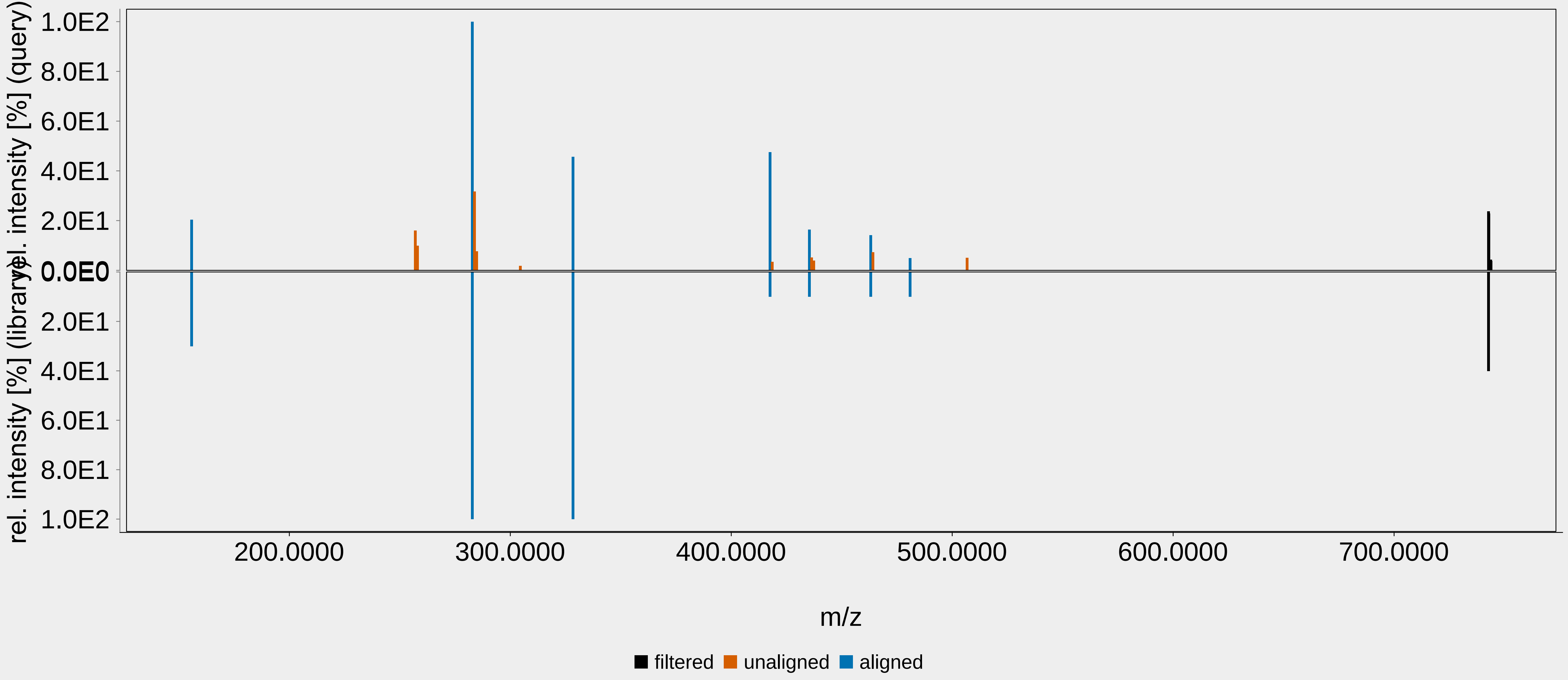

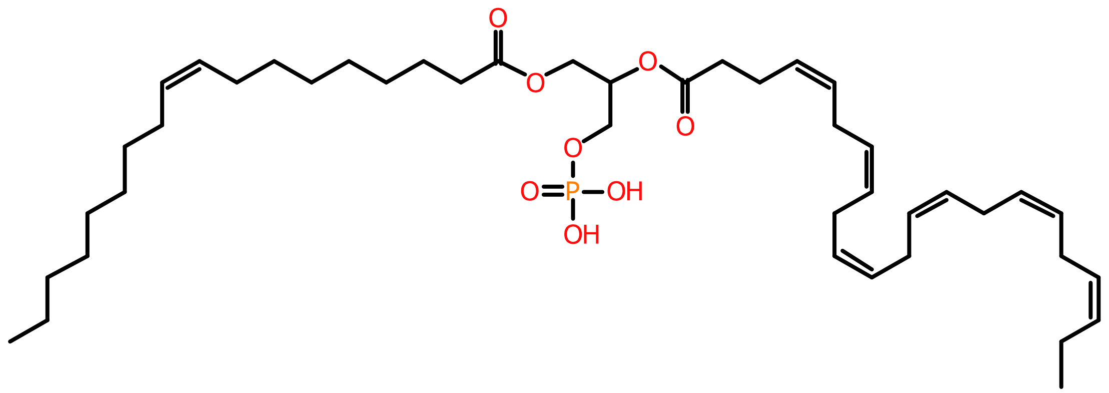

Compound information

Name: PA 18:1_22:6

Formula: C43H71O8P

Ion type: [M-H]-

Precursor m/z: 745.48138

Ccs: 264.25

InChI key:

SMILES:

Num peaks: 8

Instrument information

Database links

Other information

PE(18:0/22:6); [M-H]- C45H77N1O8P1 **0.878**

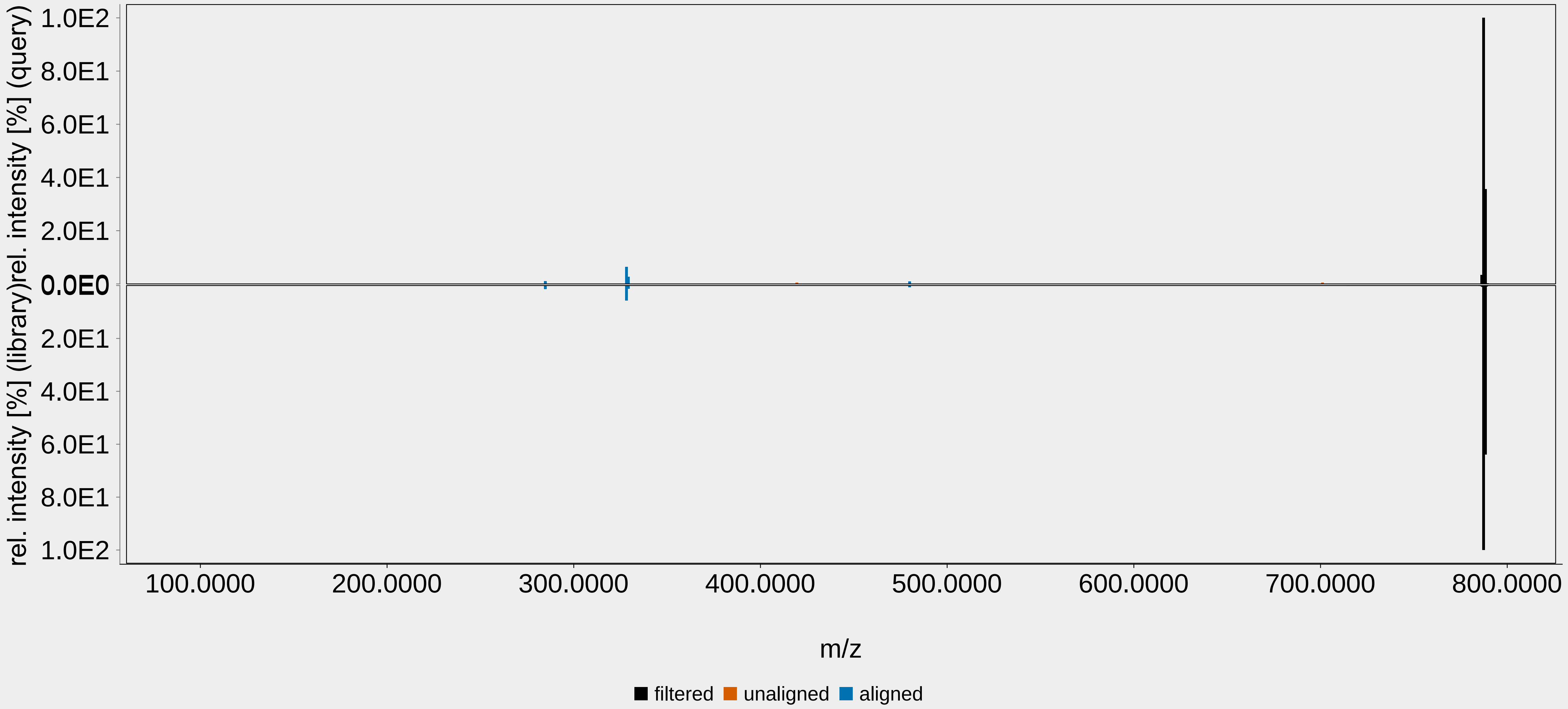

Compound information

Name: PE(18:0/22:6); [M-H]- C45H77N1O8P1

Exact mass: 0.0

Ion type: M-H

Precursor m/z: 790.539

Charge: 1

Polarity: Negative

Instrument information

Instrument type: HCD; Velos

Ion source: LC-ESI

Ms level: 2

Acquisition: Commercial

Database links

Other information

Principal investigator: Thomas Metz

Data collector: Thomas Metz

Entry ID: CCMSLIB00010085809

PE(18:0/22:6); [M-H]- C45H77N1O8P1 **0.880**

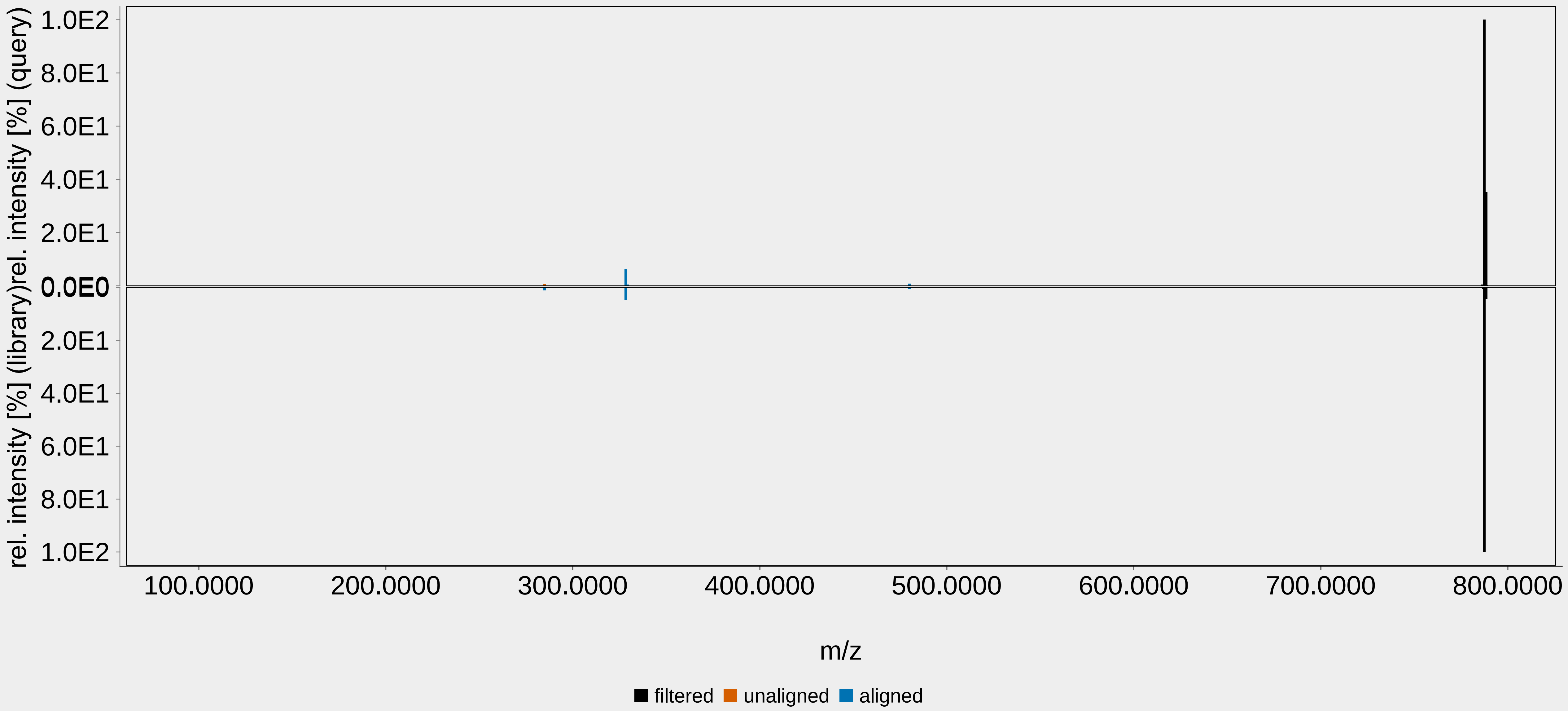

Compound information

Name: PE(18:0/22:6); [M-H]- C45H77N1O8P1

Exact mass: 0.0

Ion type: M-H

Precursor m/z: 790.539

Charge: 1

Polarity: Negative

Instrument information

Instrument type: HCD; Velos

Ion source: LC-ESI

Ms level: 2

Acquisition: Commercial

Database links

Other information

Principal investigator: Thomas Metz

Data collector: Thomas Metz

Entry ID: CCMSLIB00010073326

NADH - 30.0 eV

0.936

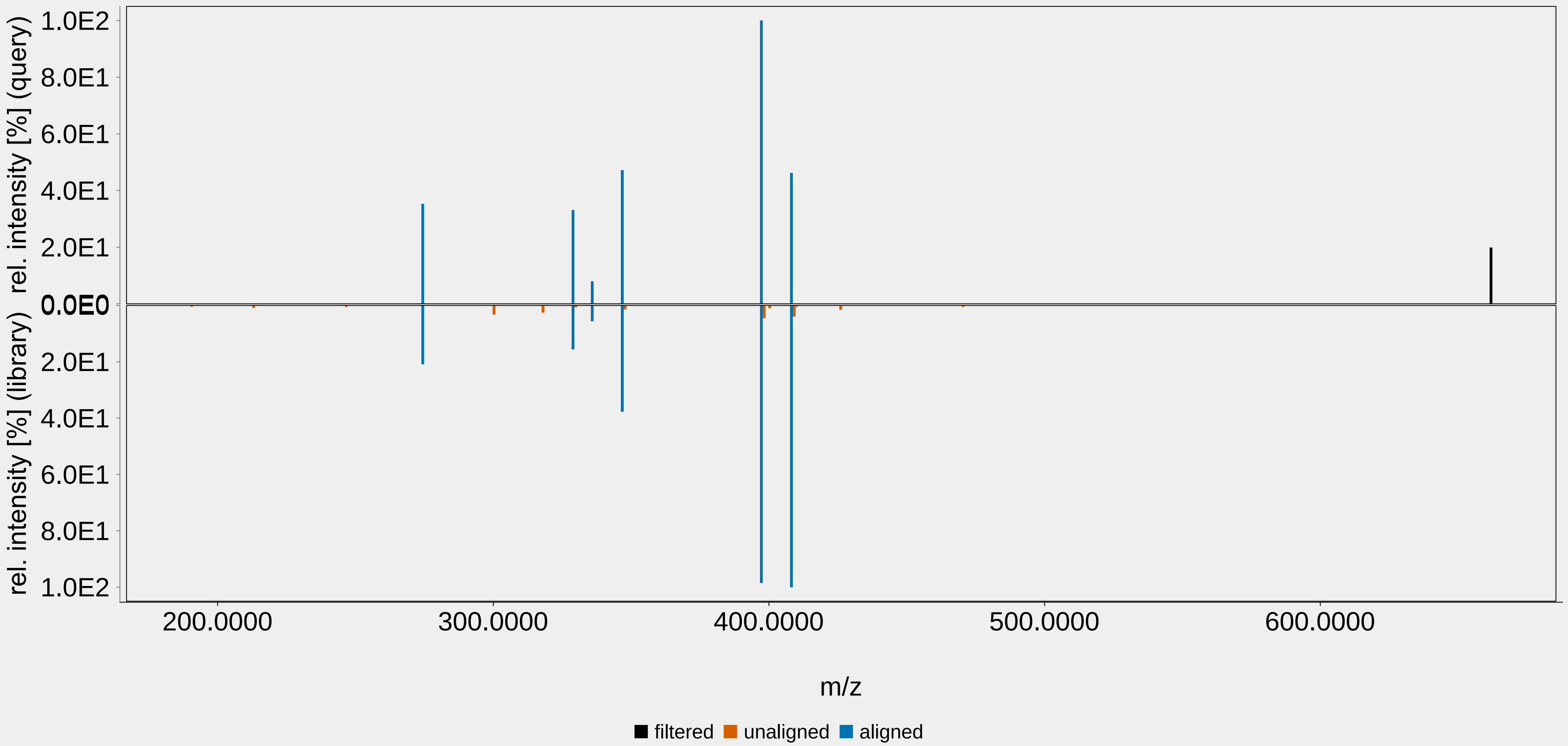

Compound information

Name: NADH - 30.0 eV

Exact mass: 0.0

Ion type: M-H

Precursor m/z: 664.118

Charge: 1

Polarity: Negative

SMILES:

Instrument information

Instrument type: Orbitrap

Ion source: ESI

Ms level: 2

Acquisition: Isolated

Database links

CAS:

Other information

Principal investigator: PI

Data collector: Data Collector

Entry ID: CCMSLIB00006123962

NADH - 30.0 eV 0.944

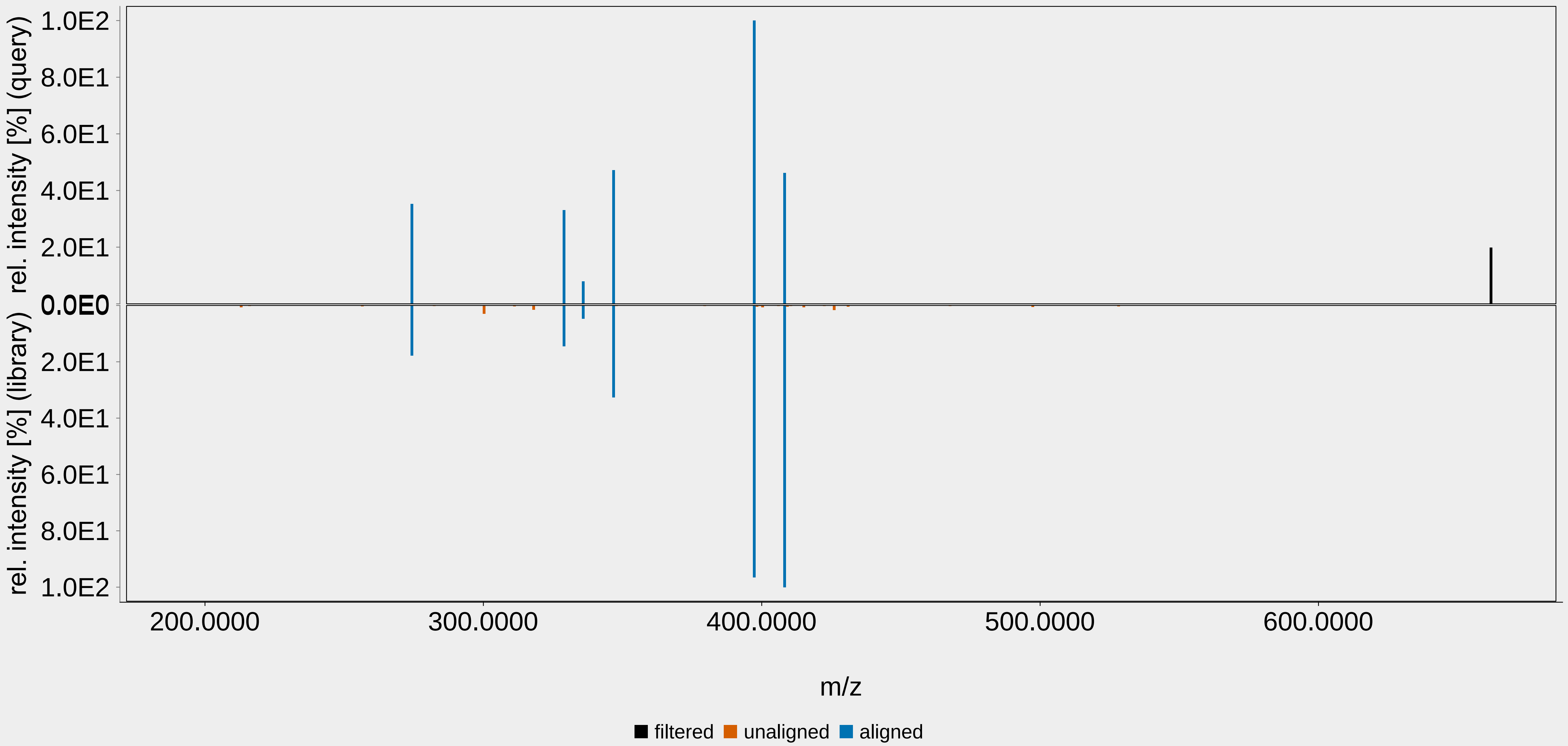

Compound information

Name: NADH - 30.0 eV

Exact mass: 0.0

Ion type: M-H

Precursor m/z: 664.117

Charge: 1

Polarity: Negative

SMILES:

Instrument information

Instrument type: Orbitrap

Ion source: ESI

Ms level: 2

Acquisition: Isolated

Database links

CAS:

Other information

Principal investigator: PI

Data collector: Data Collector

Entry ID: CCMSLIB00006123977

PI 40:6

0.845

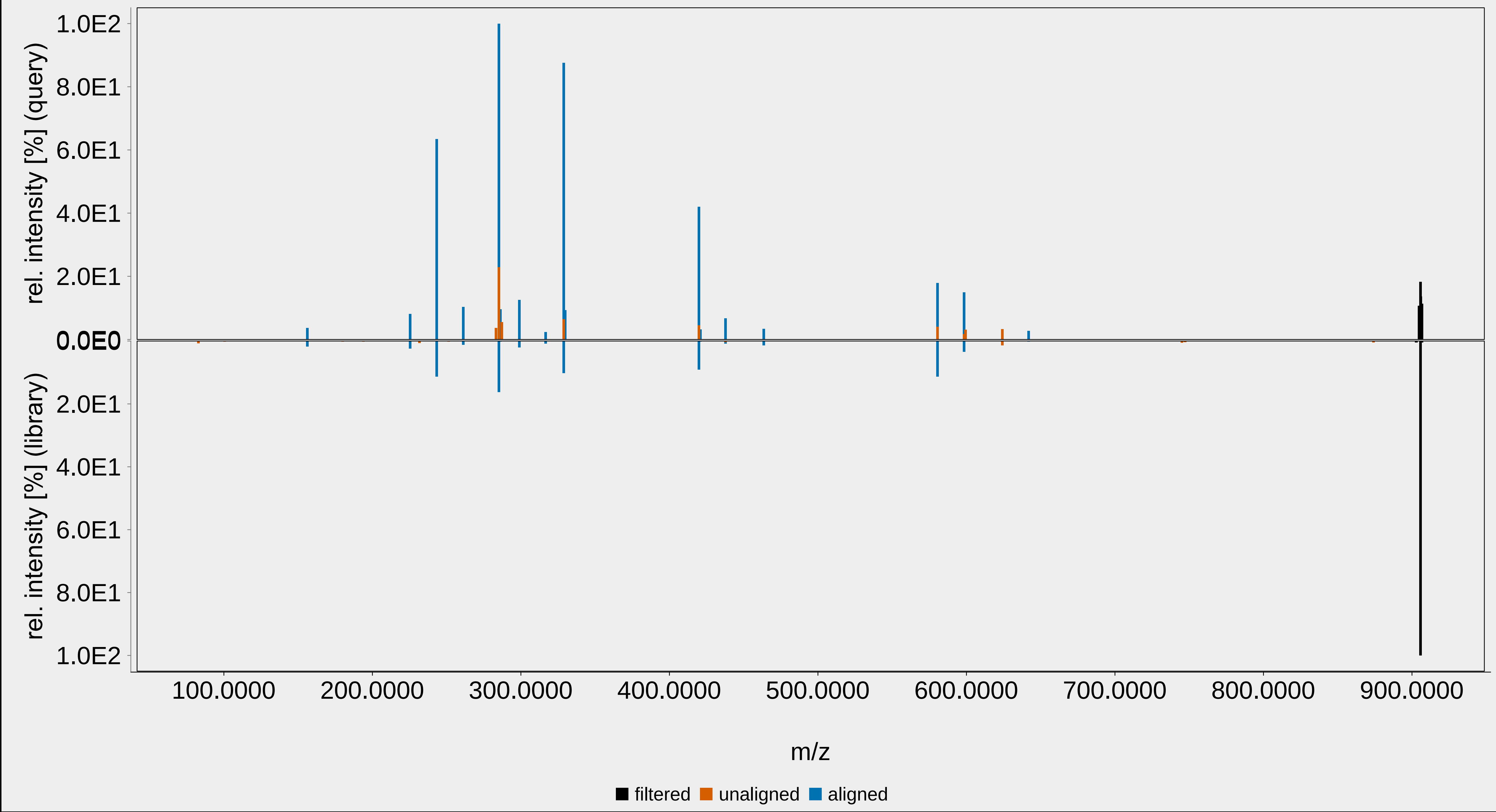

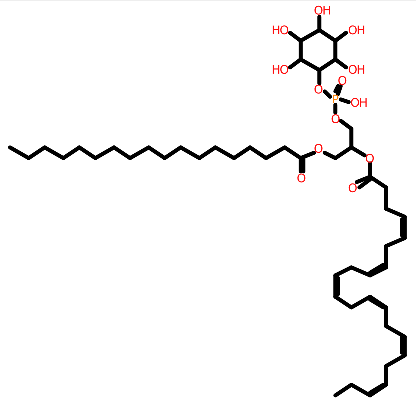

Compound information

Name: PI 40:6

Formula: C49H83O13P

Mol. weight: 911.1659999999999

Exact mass: 911.1659999999999

Ion type: [M-H]-

Precursor m/z: 909.5445

RT: 8.653857

Polarity: negative

InChI:

InChI key:

SMILES:

Instrument information

Instrument type: LC-ESI-QTOF

Instrument: AB Sciex TripleTOF 5600+ system

Ms level: MS2

Collision energy: 45V

Database links

Pubchem: 138144195

MoNA ID: LQB00699

Other information

Principal investigator: Kazutaka Ikeda, Hiroshi

Data collector: Kazutaka Ikeda, Hiroshi

PI 40:6 0.845

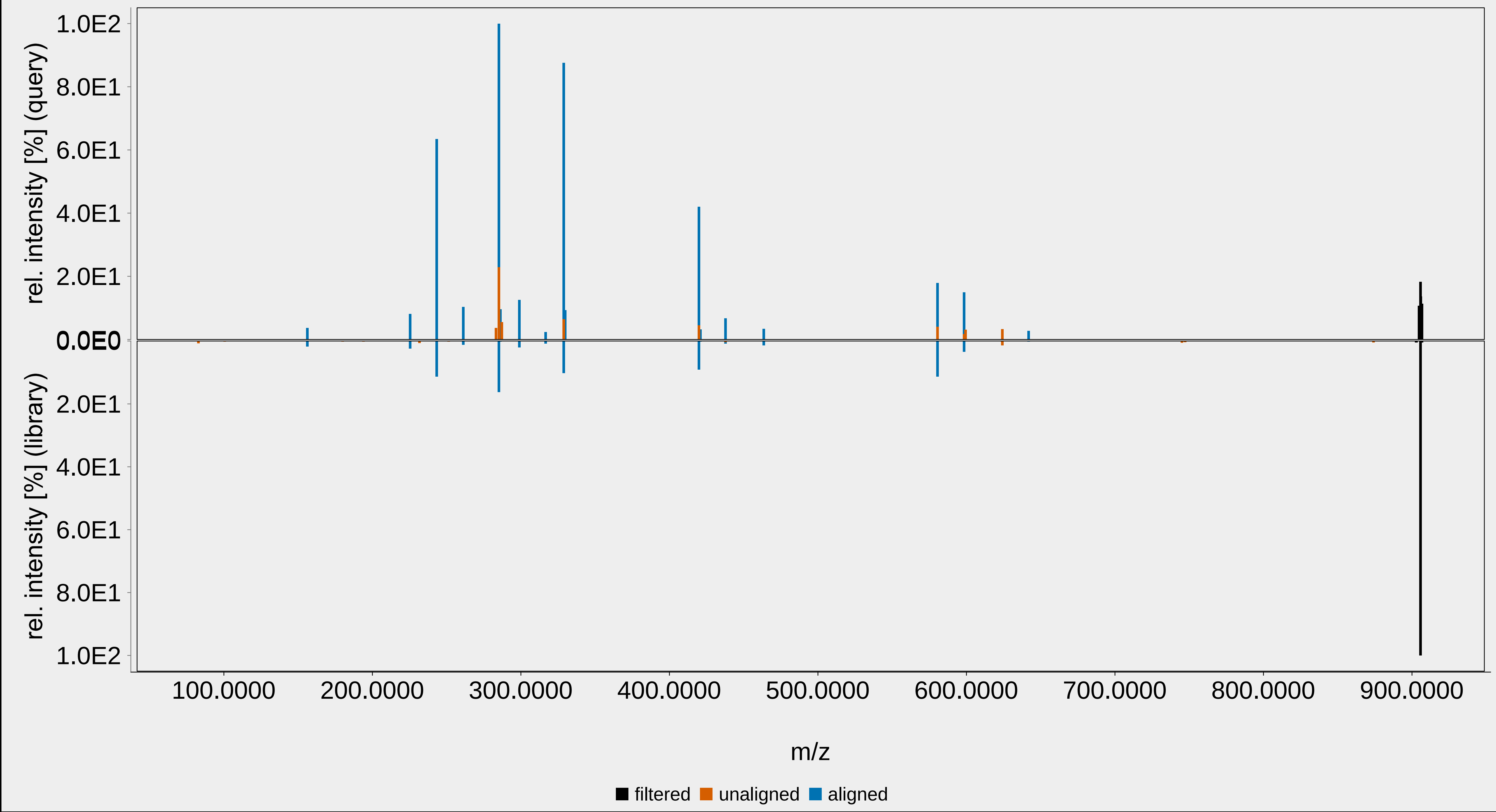

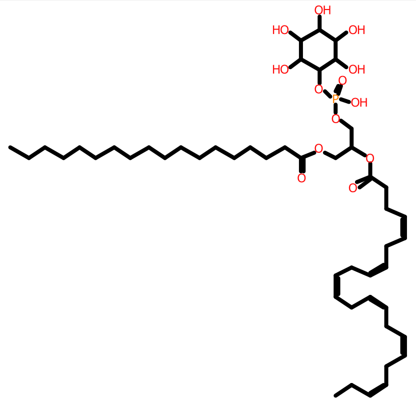

Compound information

Name: PI 40:6

Formula: C49H83O13P

Mol. weight: 911.1659999999999

Exact mass: 911.1659999999999

Ion type: [M-H]-

Precursor m/z: 909.5445

RT: 8.653857

Polarity: negative

InChI:

InChI key:

SMILES:

Instrument information

Instrument type: LC-ESI-QTOF

Instrument: AB Sciex TripleTOF 5600+ system

Ms level: MS2

Collision energy: 45V

Database links

Pubchem: 138144195

MoNA ID: LQB00313

Other information

Principal investigator: Kazutaka Ikeda, Hiroshi

Data collector: Kazutaka Ikeda, Hiroshi

PE(P-18:0/22:6); [M-H]- C45H77N1O7P1 **0.852**

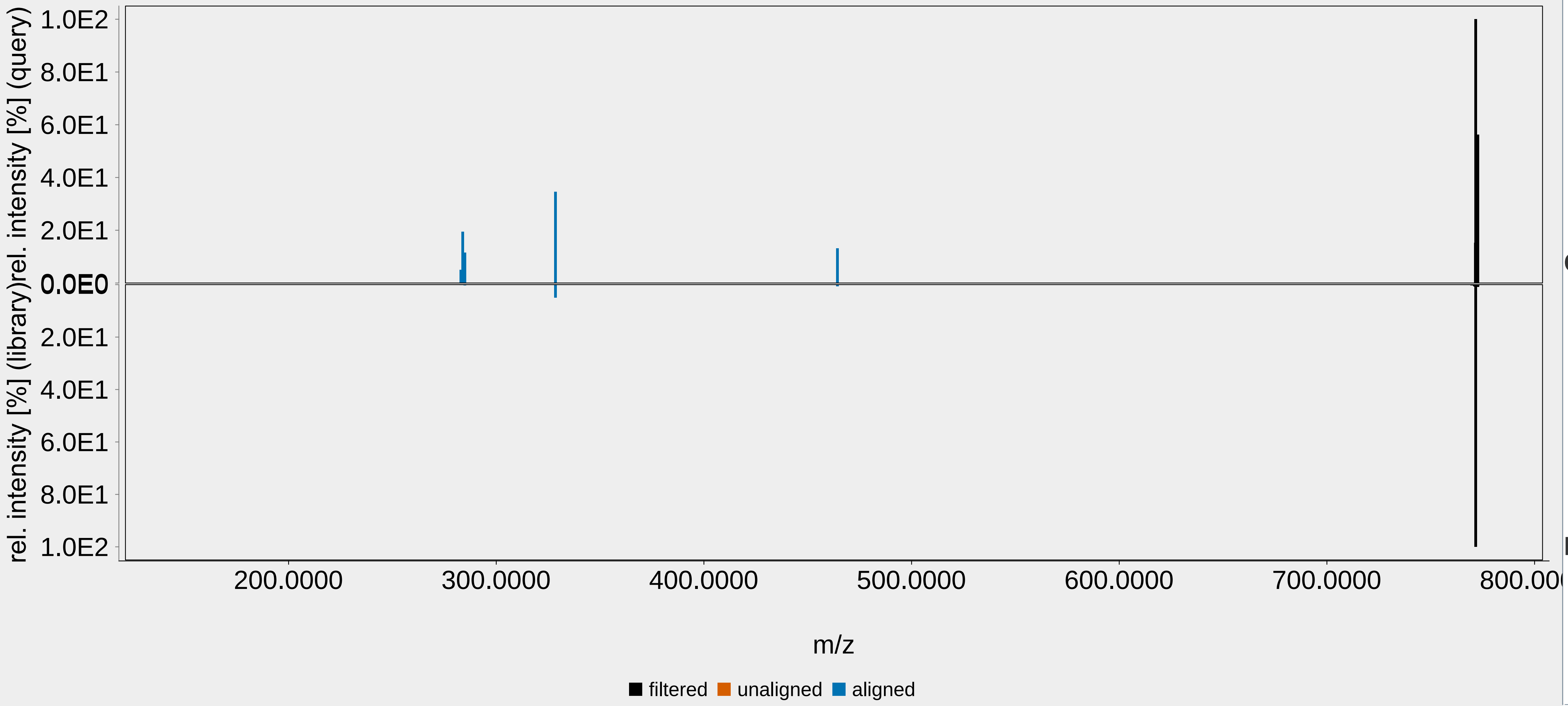

Compound information

Name: PE(P-18:0/22:6); [M-H]-

Exact mass: 0.0

Ion type: M-H

Precursor m/z: 774.544

Charge: 1

Polarity: Negative

Instrument information

Instrument type: HCD; Velos

Ion source: LC-ESI

Ms level: 2

Acquisition: Commercial

Database links

Other information

Principal investigator: Thomas Metz

Data collector: Thomas Metz

Entry ID: CCMSLIB00010087922

PE(P-18:0/22:6); [M-H]- C45H77N1O7P1 **0.860**

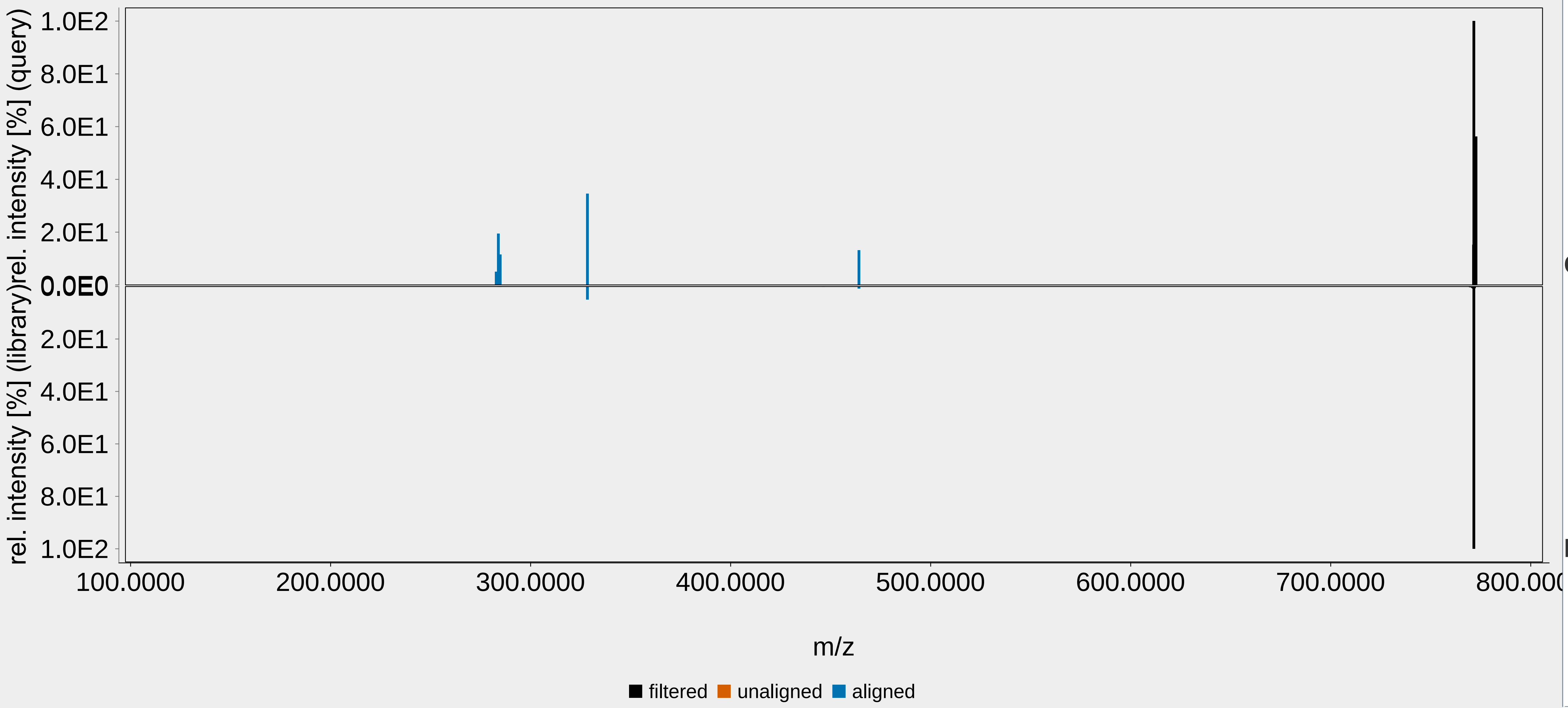

Compound information

Name: PE(P-18:0/22:6); [M-H]-

Exact mass: 0.0

Ion type: M-H

Precursor m/z: 774.544

Charge: 1

Polarity: Negative

Instrument information

Instrument type: HCD; Velos

Ion source: LC-ESI

Ms level: 2

Acquisition: Commercial

Database links

Other information

Principal investigator: Thomas Metz

Data collector: Thomas Metz

Entry ID: CCMSLIB00010088413

PA 16:0_22:6 **0.729**

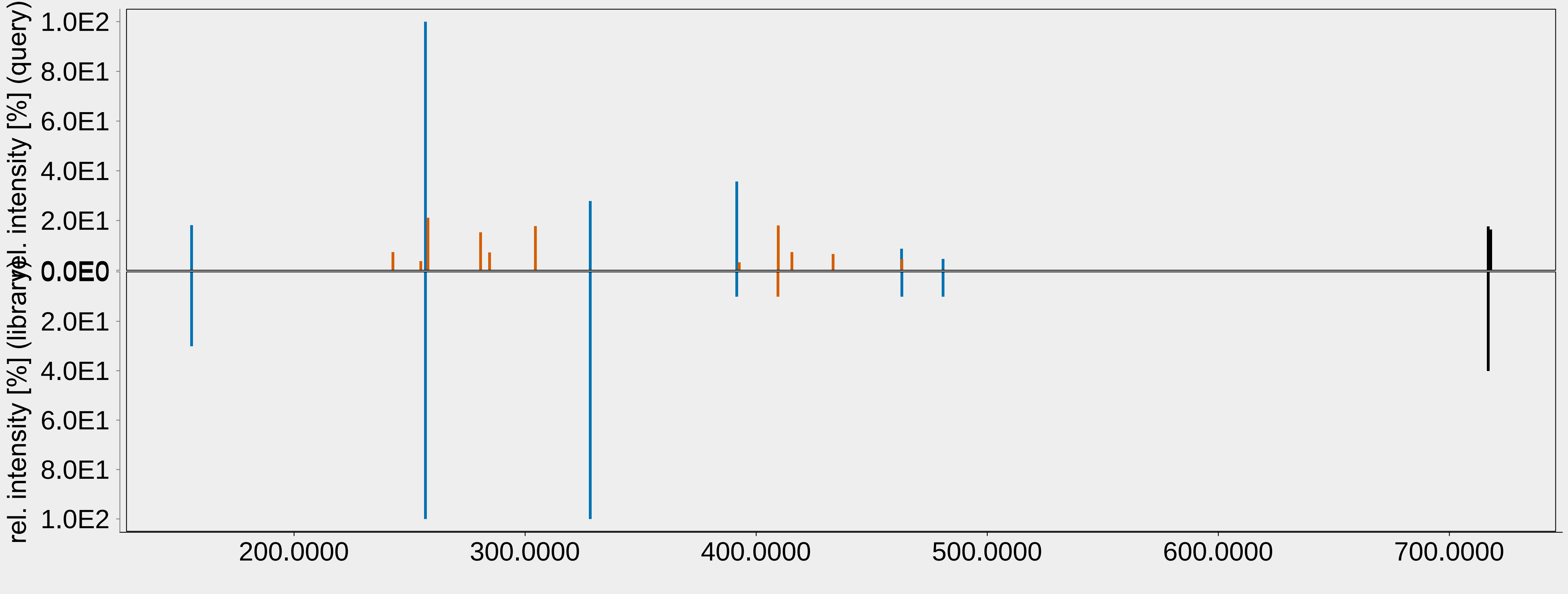

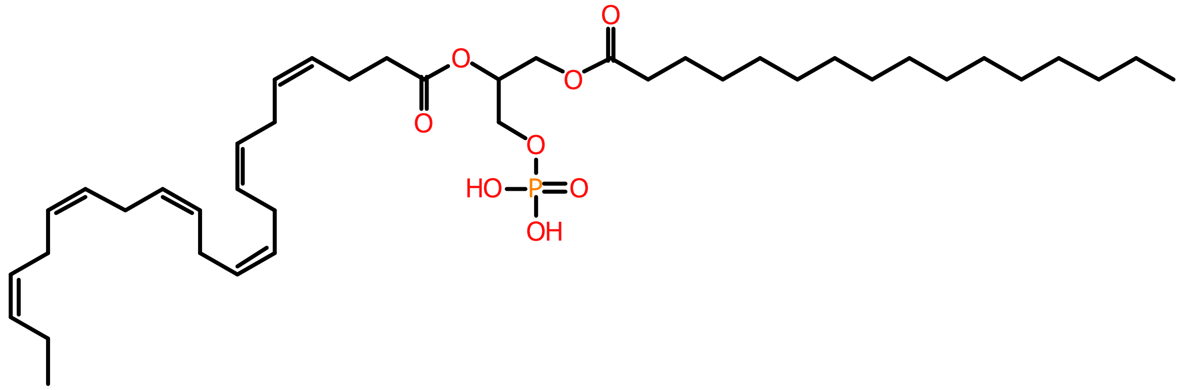

Compound information

Name: PA 16:0_22:6

Formula: C41H69O8P

Ion type: [M-H]-

Precursor m/z: 719.46573

Ccs: 257.26

InChI key:

SMILES:

Num peaks: 8

Instrument information

Database links

Other information

PG(22:6/22:6); [M-H]- C50H74O10P1 0.709

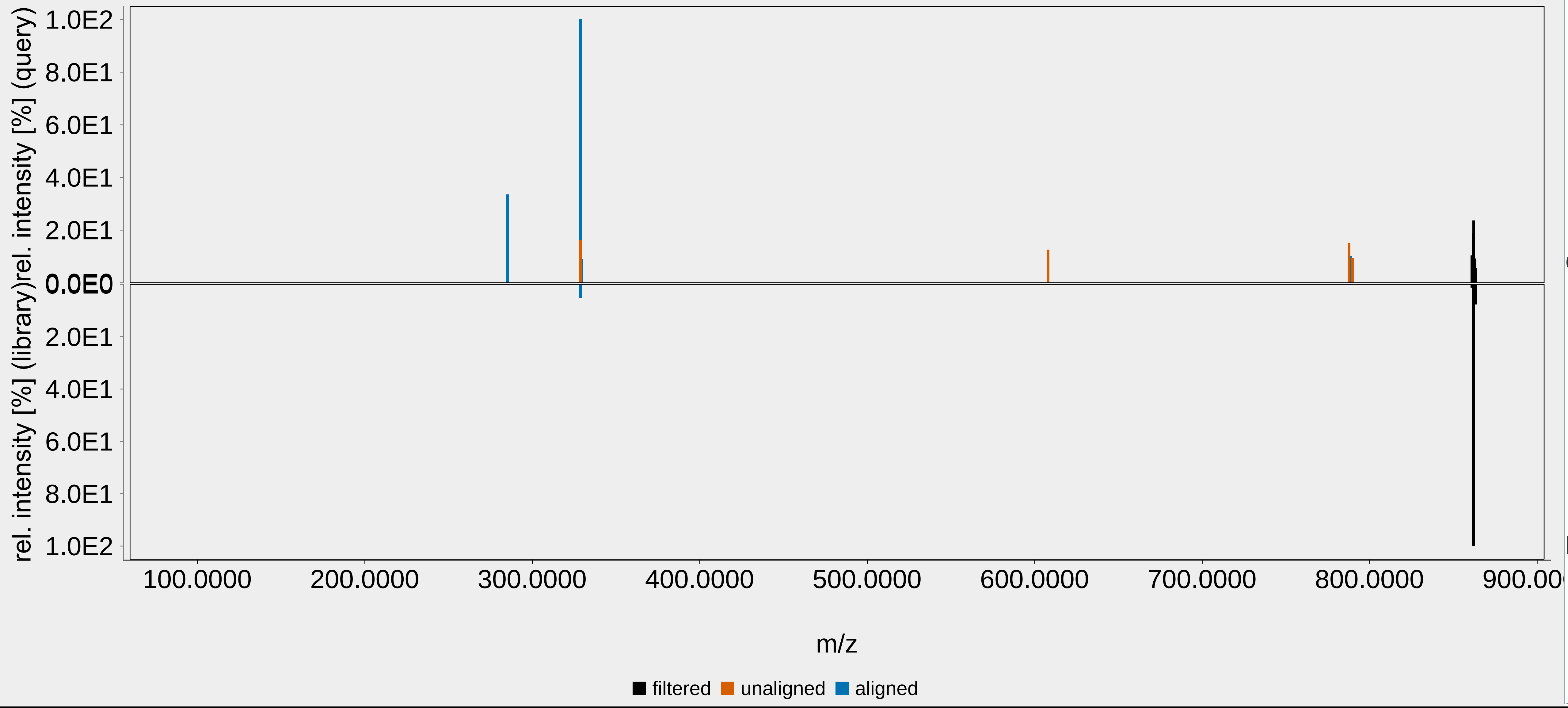

Compound information

Name: PG(22:6/22:6); [M-H]- C50H74O10P1

Exact mass: 0.0

Ion type: M-H

Precursor m/z: 865.502

Charge: 1

Polarity: Negative

Instrument information

Instrument type: HCD; Velos

Ion source: LC-ESI

Ms level: 2

Acquisition: Commercial

Database links

Other information

Principal investigator: Thomas Metz

Data collector: Thomas Metz

Entry ID: CCMSLIB00010083995

PG(22:6/22:6); [M-H]- C50H74O10P1 **0.726**

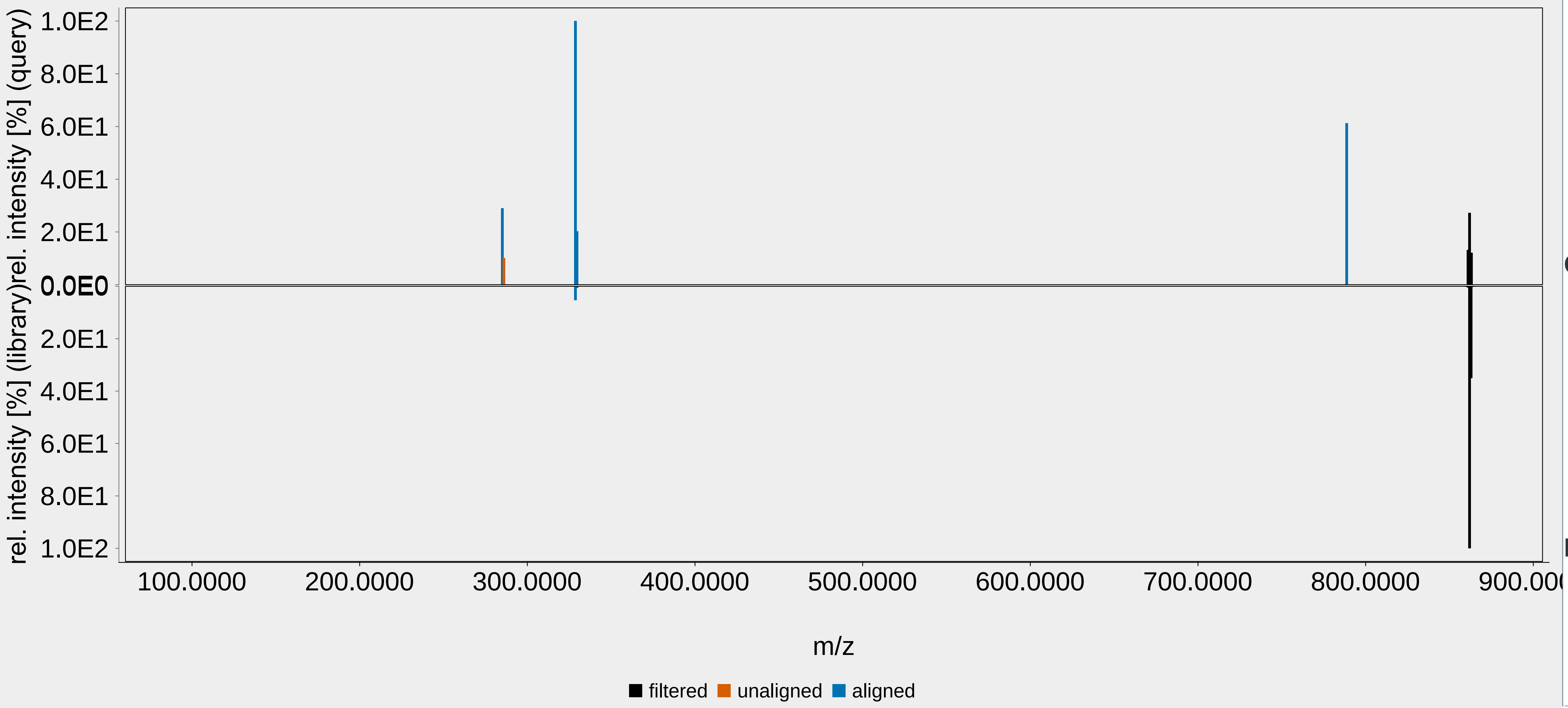

Compound information

Name: PG(22:6/22:6); [M-H]- C50H74O10P1

Exact mass: 0.0

Ion type: M-H

Precursor m/z: 865.502

Charge: 1

Polarity: Negative

Instrument information

Instrument type: HCD; Velos

Ion source: LC-ESI

Ms level: 2

Acquisition: Commercial

Database links

Other information

Principal investigator: Thomas Metz

Data collector: Thomas Metz

Entry ID: CCMSLIB00010082177

PI 40:4 **0.787**

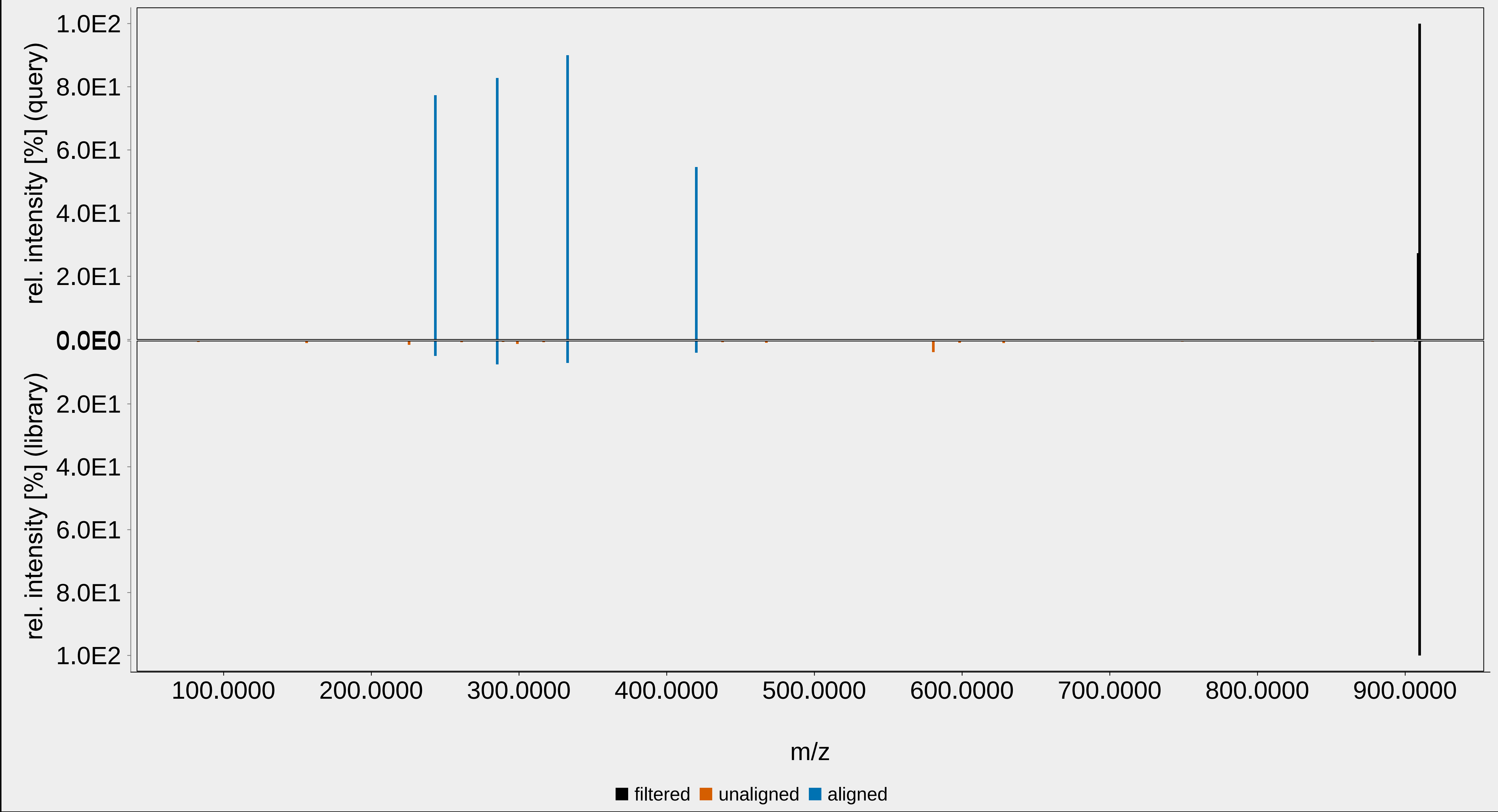

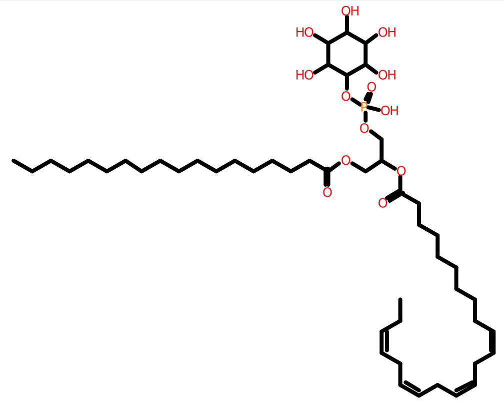

Compound information

Name: PI 40:4

Formula: C49H87O13P

Mol. weight: 915.198

Exact mass: 915.198

Ion type: [M-H]-

Precursor m/z: 913.5835

RT: 9.142293

Polarity: negative

InChI:

InChI key: VTYLJIRGLIZEIC-PYQGTGCGSA-N

SMILES:

Instrument information

Instrument type: LC-ESI-QTOF

Instrument: AB Sciex TripleTOF 5600+ system

Ms level: MS2

Collision energy: 45V

Database links

Pubchem: 138283546

MoNA ID: LQB00697

Other information

Principal investigator: Kazutaka Ikeda, Hiroshi

Data collector: Kazutaka Ikeda, Hiroshi

PI 40:4 **0.787**

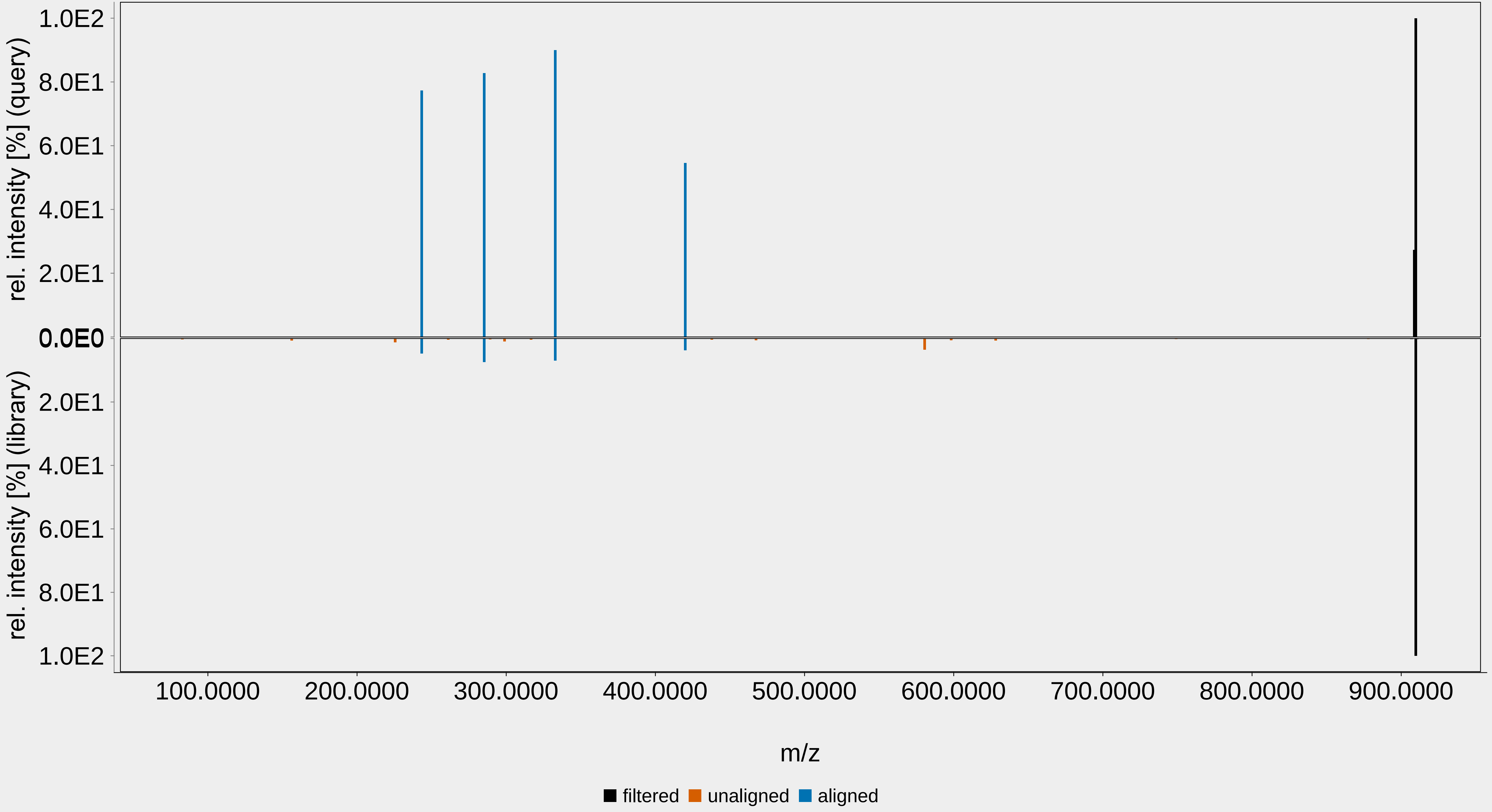

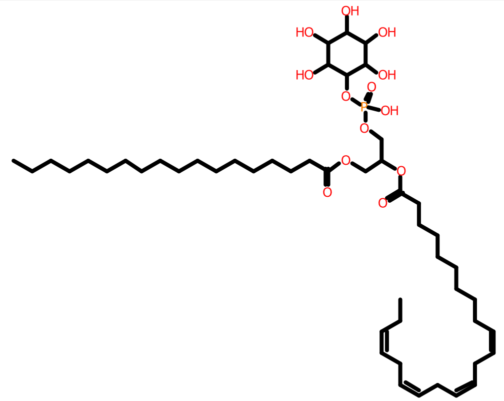

Compound information

Name: PI 40:4

Formula: C49H87O13P

Mol. weight: 915.198

Exact mass: 915.198

Ion type: [M-H]-

Precursor m/z: 913.5835

RT: 9.142293

Polarity: negative

InChI:

InChI key: VTYLJIRGLIZEIC-PYQGTGCGSA-N

SMILES:

Instrument information

Instrument type: LC-ESI-QTOF

Instrument: AB Sciex TripleTOF 5600+ system

Ms level: MS2

Collision energy: 45V

Database links

Pubchem: 138283546

MoNA ID: LQB00311

Other information

Principal investigator: Kazutaka Ikeda, Hiroshi

Data collector: Kazutaka Ikeda, Hiroshi

PE(18:1/20:4); [M-H]- C43H75N1O8P1 **0.872**

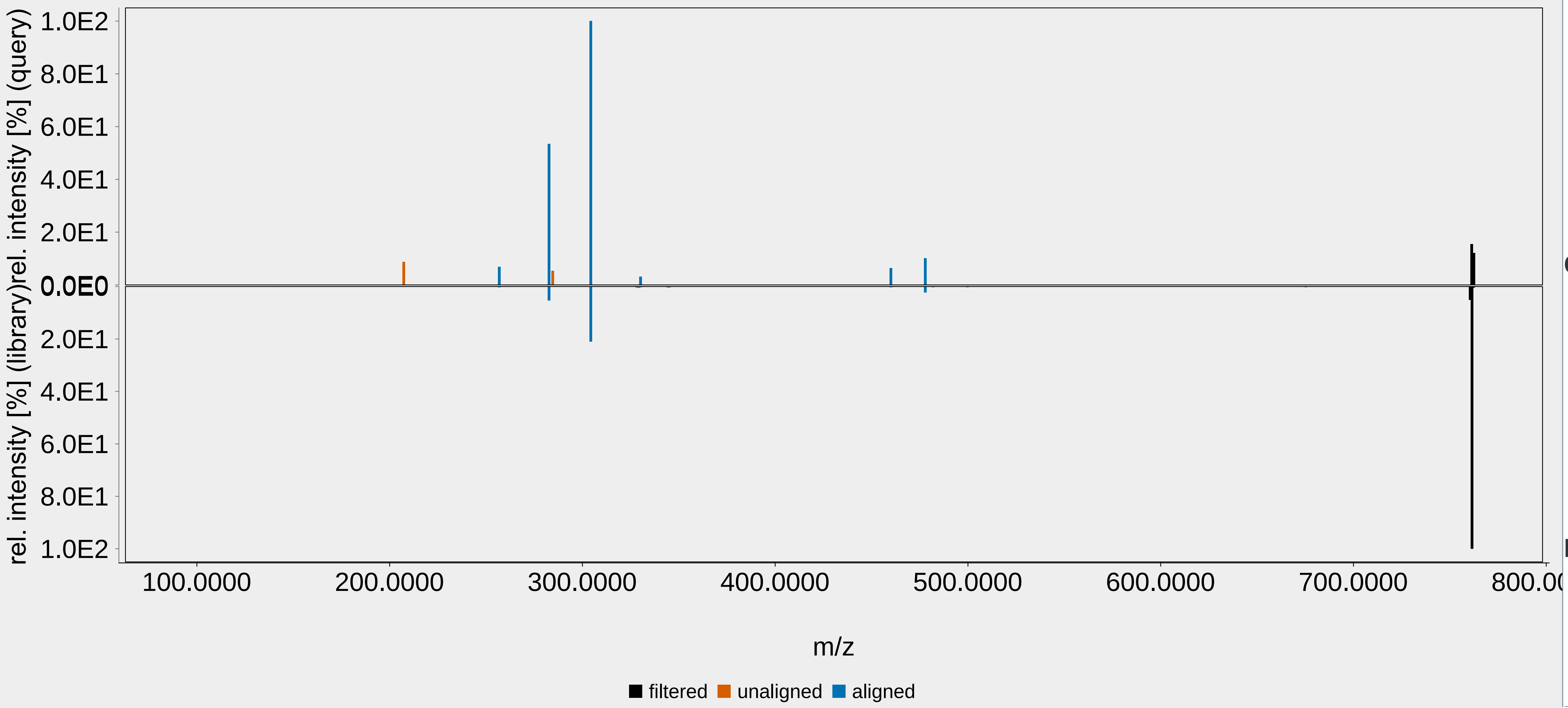

Compound information

Name: PE(18:1/20:4); [M-H]- C43H75N1O8P1

Exact mass: 0.0

Ion type: M-H

Precursor m/z: 764.523

Charge: 1

Polarity: Negative

Instrument information

Instrument type: HCD; Velos

Ion source: LC-ESI

Ms level: 2

Acquisition: Commercial

Database links

Other information

Principal investigator: Thomas Metz

Data collector: Thomas Metz

Entry ID: CCMSLIB00010063428

PE 18:1_20:4

0.880

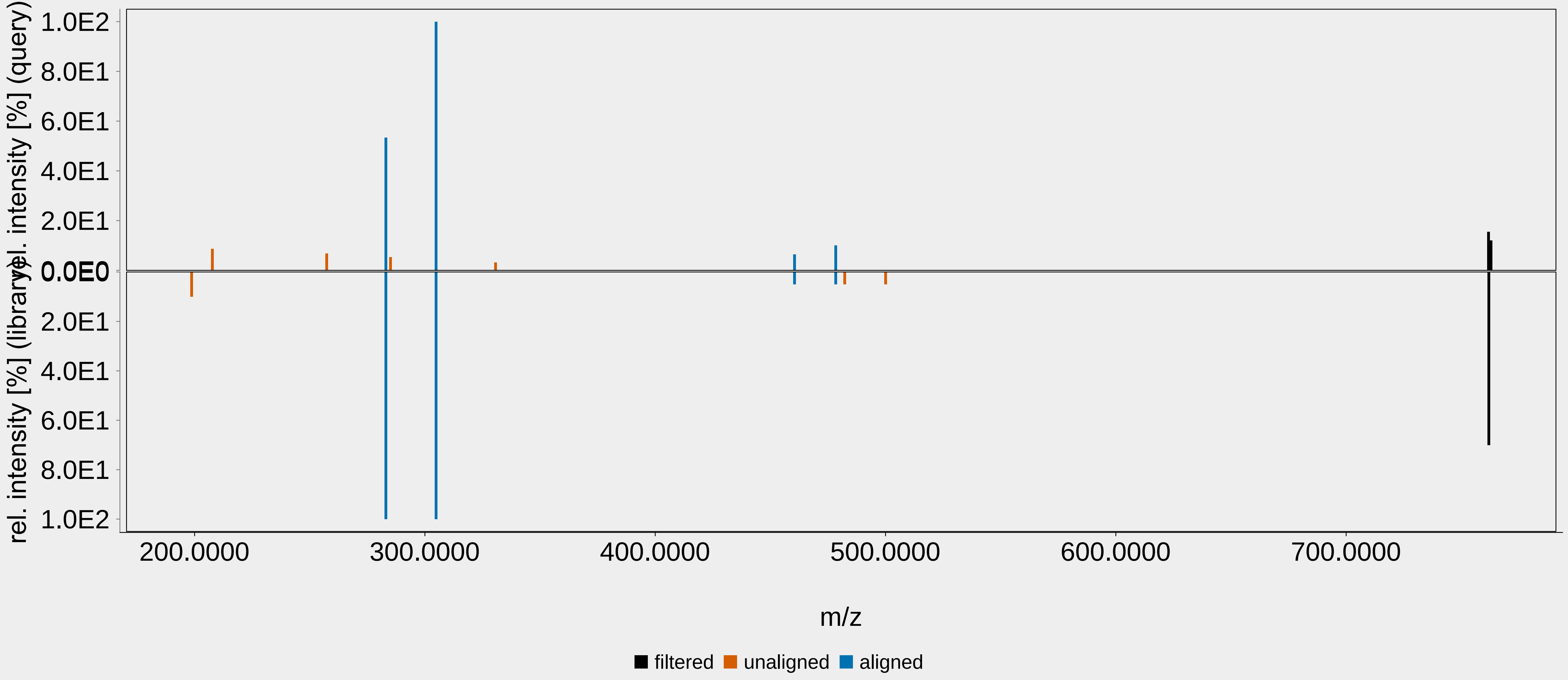

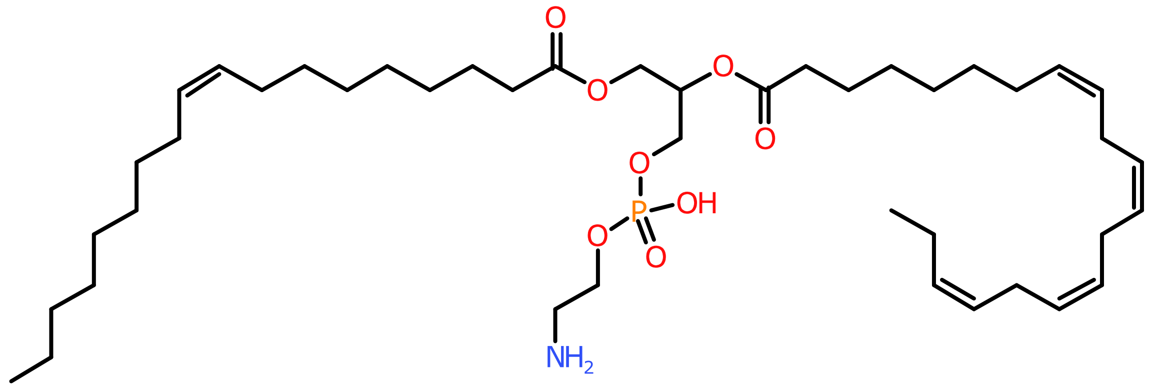

Compound information

Name: PE 18:1_20:4

Formula: C43H76NO8P

Ion type: [M-H]-

Precursor m/z: 764.52358

Ccs: 268.34

InChl key:

SMILES:

Num peaks: 8

Instrument information

Database links

Other information

PA 18:0_22:4

0.878

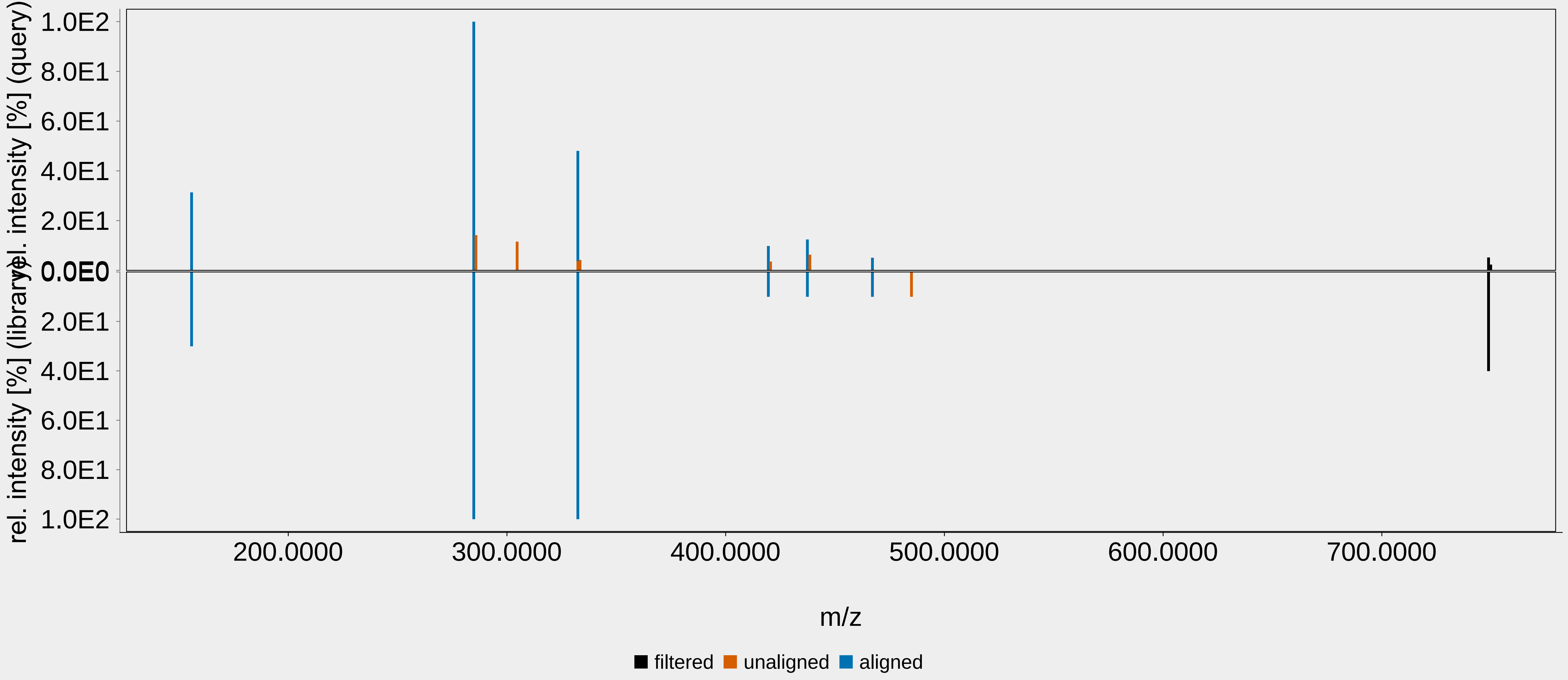

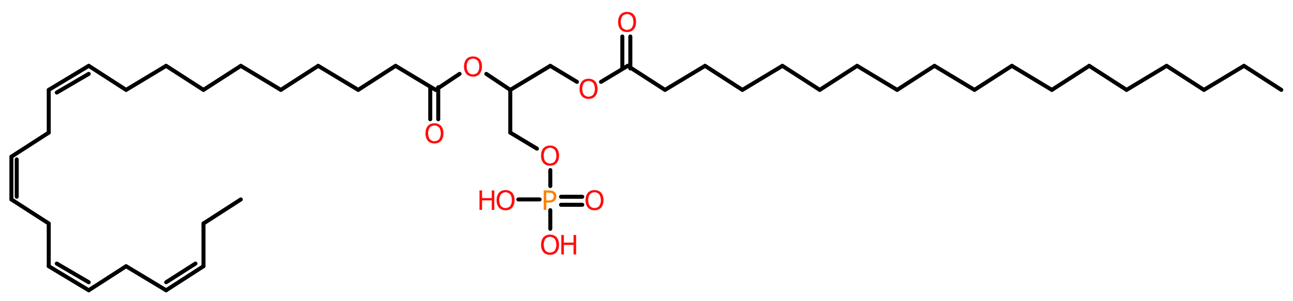

Compound information

Name: PA 18:0_22:4

Formula: C43H77O8P

Ion type: [M-H]-

Precursor m/z: 751.52833

Ccs: 267.82

InChI key:

SMILES:

Num peaks: 8

Instrument information

Database links

Other information

Arachidonic acid

0.714

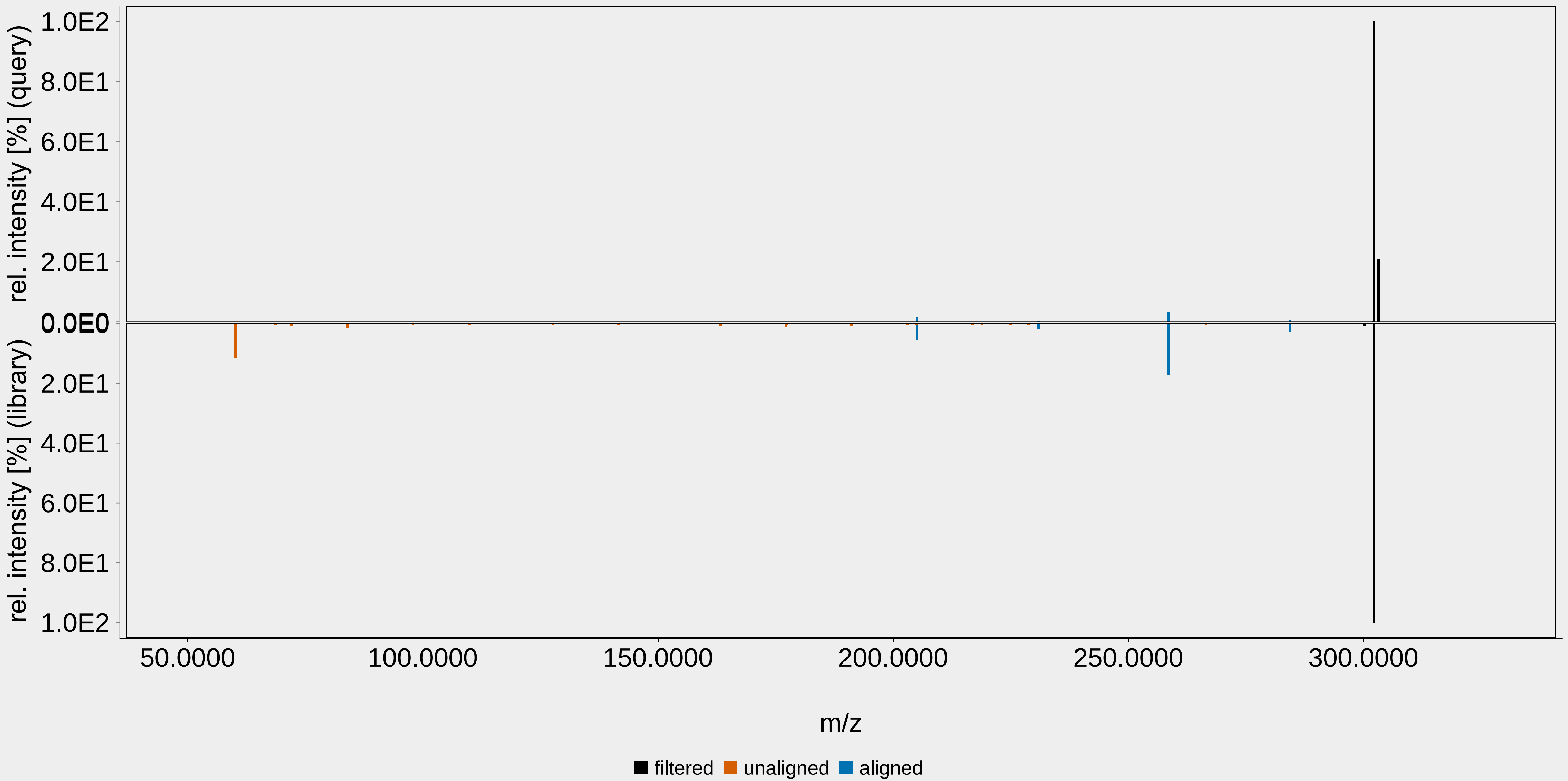

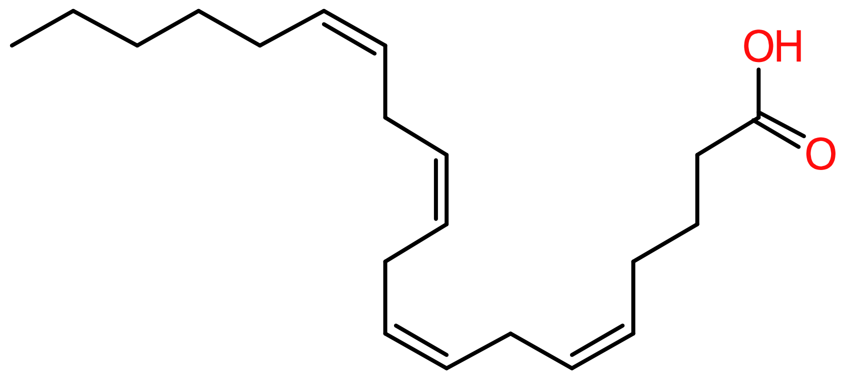

Compound information

Name: Arachidonic acid

Formula: C20H32O2

Ion type: [M-H]-

Precursor m/z: 303.232041974554

RT: 4.158056

Polarity: negative

InChI:

InChI key:

SMILES:

Instrument information

Instrument type: LC-ESI-QFT

Instrument: Thermo Q-Exactive Plus

Ms level: MS2

Collision energy: 30.0 eV

Database links

Other information

Principal investigator: Prasad Phapale

Data collector: Prasad Phapale

EicosatetraenoicAcid_Arachidonic

0.836

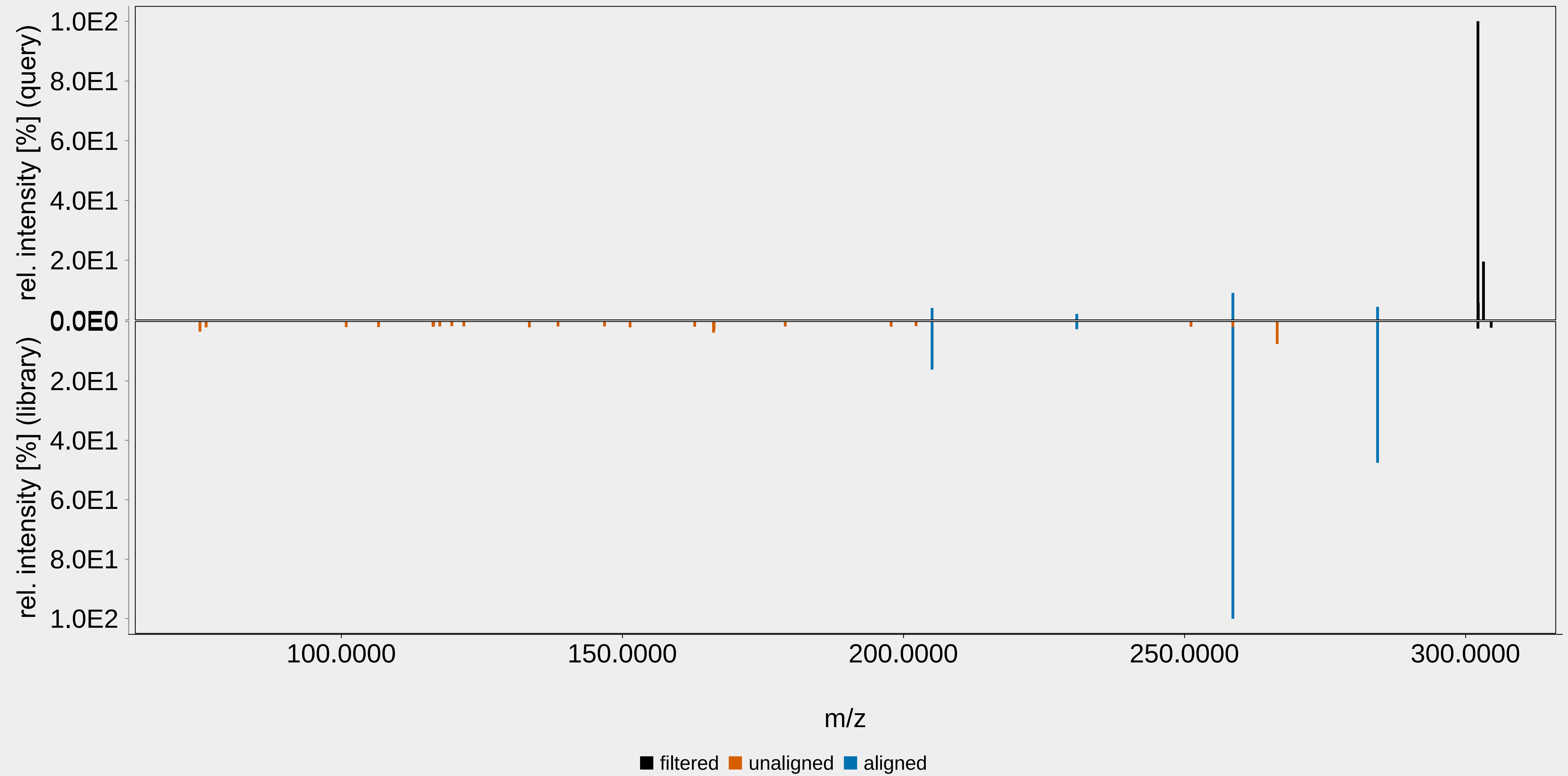

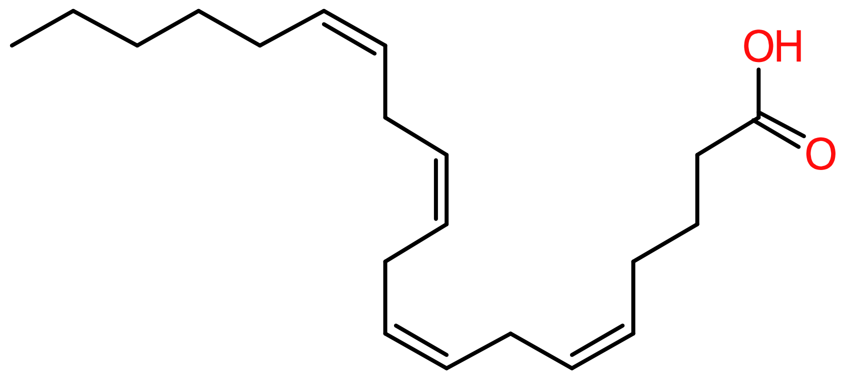

Compound information

Name: EicosatetraenoicAcid_Arachidonic

Exact mass: 304.24

Ion type: M-H

Precursor m/z: 303.233

Charge: -1

Polarity: Negative

InChI:

SMILES:

Instrument information

Instrument type: Orbitrap

Ion source: ESI

Ms level: 2

Acquisition: Commercial

Database links

Pubmed: 444899.0

CAS: 506-32-1

Other information

Principal investigator: Ulaszewska

Data collector: Marynka Ulaszewska

Entry ID: CCMSLIB00006676895

PA 16:1_20:4

0.877

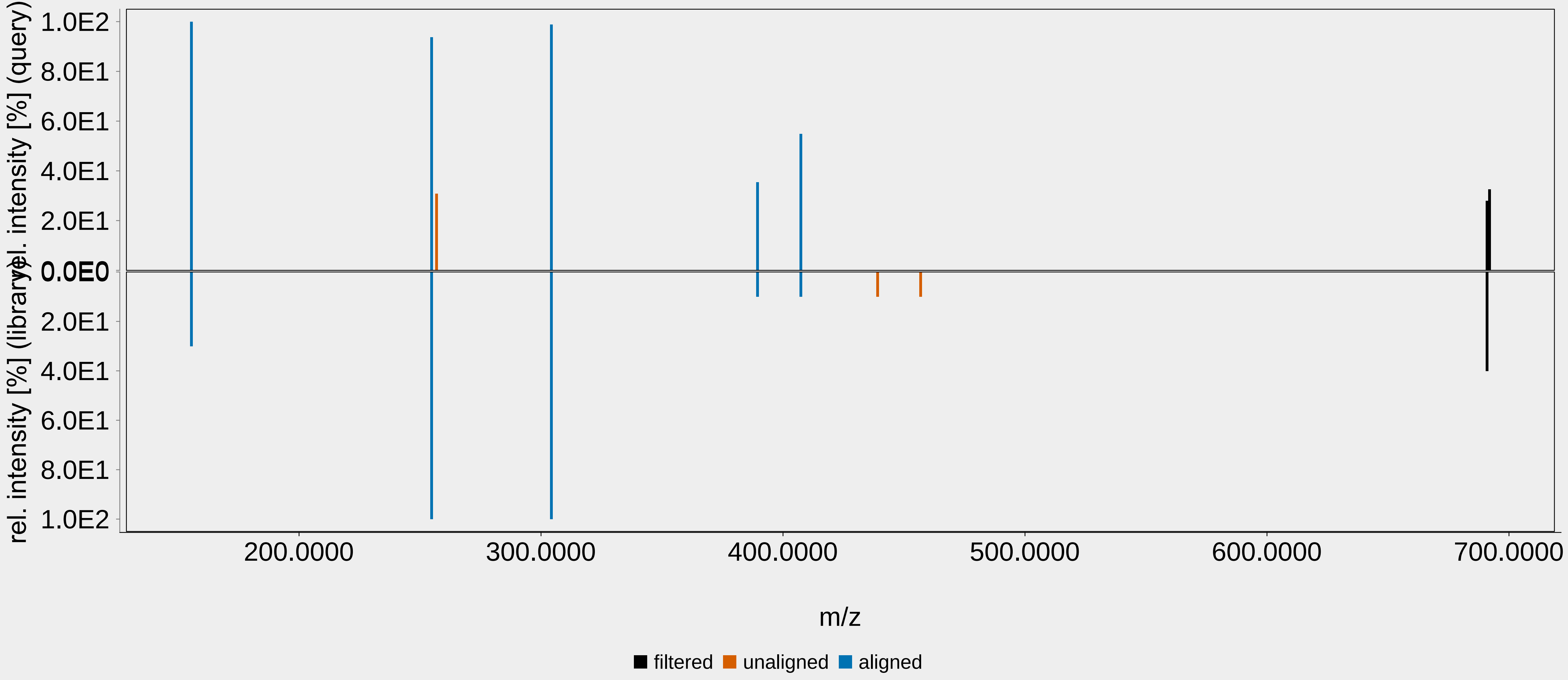

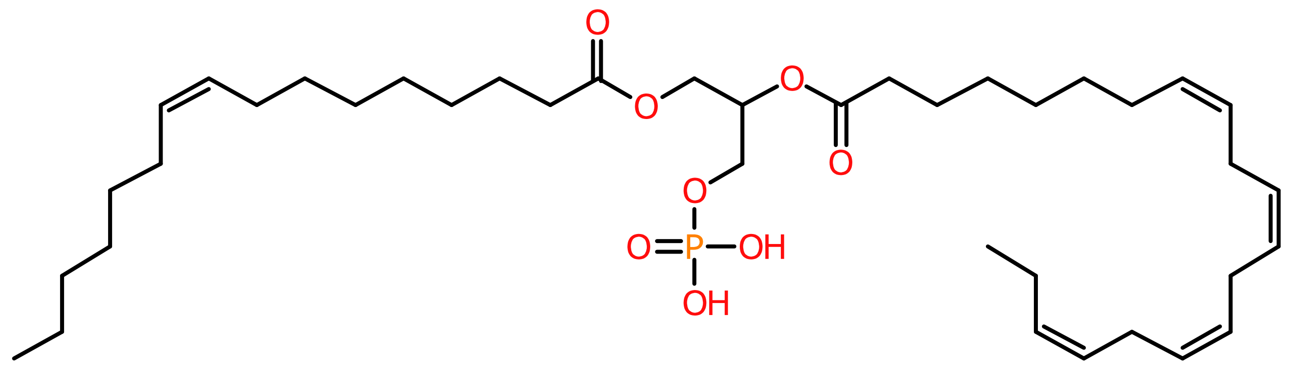

Compound information

Name: PA 16:1_20:4

Formula: C39H67O8P

Ion type: [M-H]-

Precursor m/z: 693.45008

Ccs: 253.91

InChI key:

SMILES:

Num peaks: 8

Instrument information

Database links

Other information

HYDROQUINIDINE

0.884

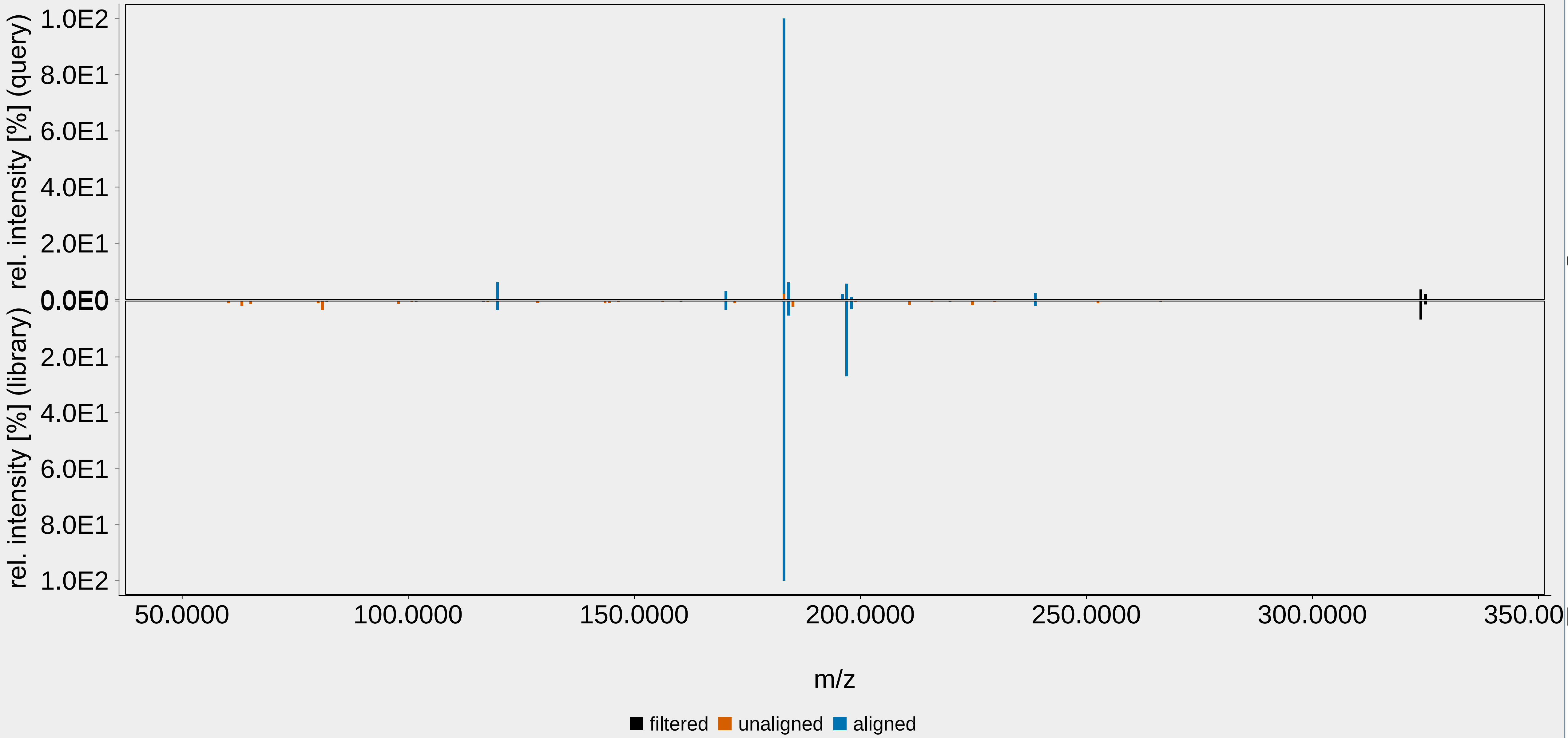

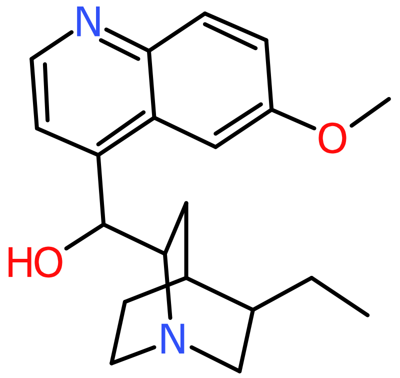

Compound information

Name: HYDROQUINIDINE

Formula: C20H26N2O2

Ion type: [M-H]-

Precursor m/z: 325.192138671875

Polarity: negative

InChI:

InChI key:

SMILES:

Instrument information

Instrument type: ESI-QFT

Instrument: Thermo Q Exactive HF

Ms level: MS2

Collision energy: 65HCD

Database links

Other information

Principal investigator: Arpana Vaniya

Data collector: Arpana Vaniya

HYDROQUINIDINE

0.884

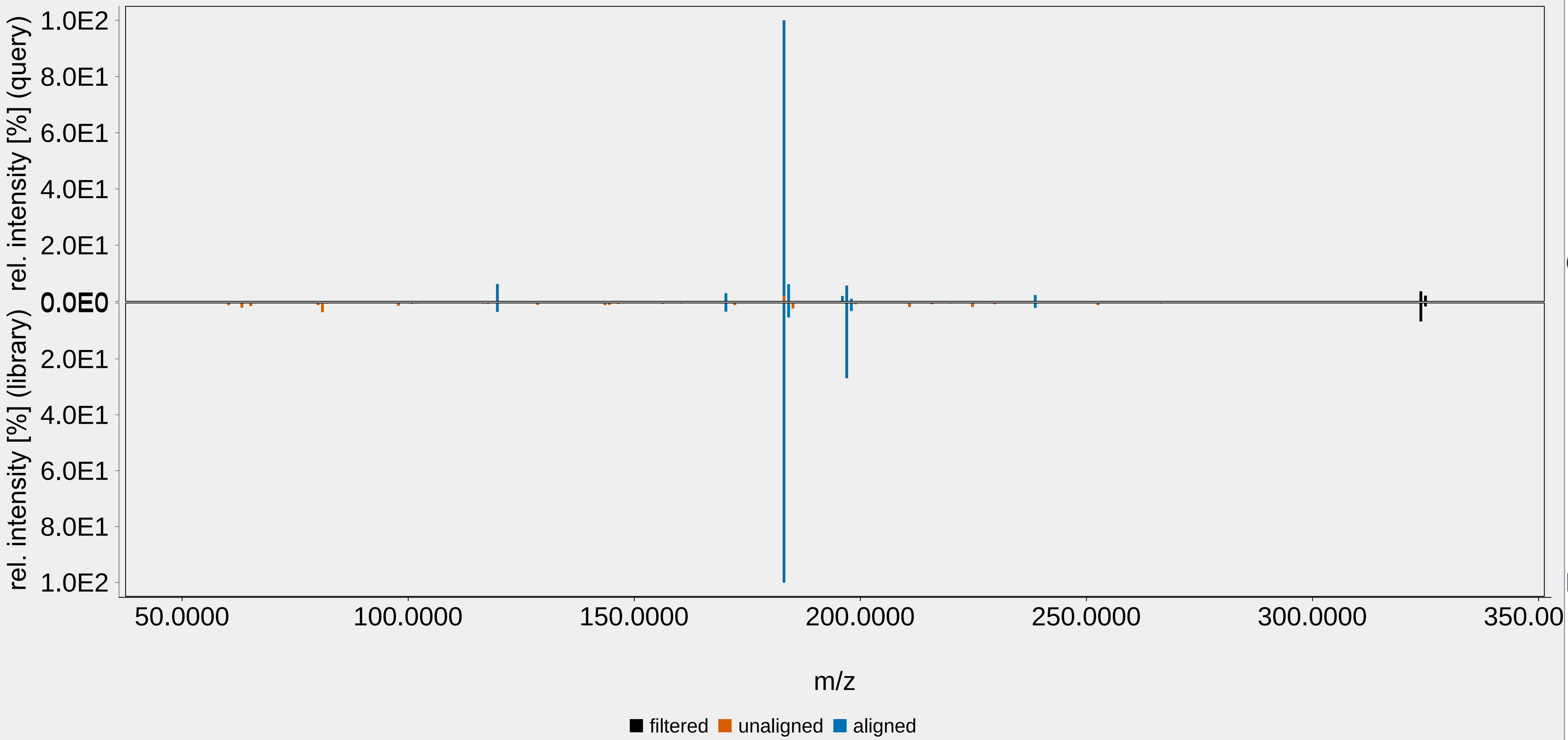

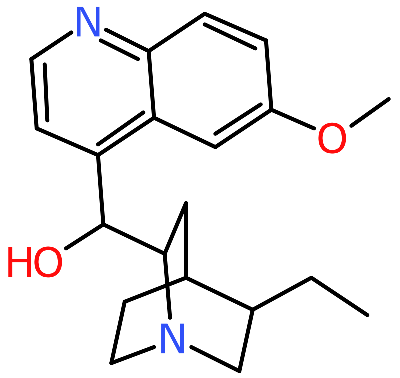

Compound information

Name: HYDROQUINIDINE

Exact mass: 0.0

Ion type: [M-H]-

Precursor m/z: 325.192

Charge: 1

Polarity: negative

InChI:

Instrument information

Instrument type: ESI-QFT

Ms level: 2

Acquisition: isolated

Database links

Other information

Principal investigator: MoNA

Data collector: MoNA:VF-NPL-QEHF003075

Entry ID: CCMSLIB00004694138

PE(18:0/20:3); [M-H]- C43H79N1O8P1 0.730

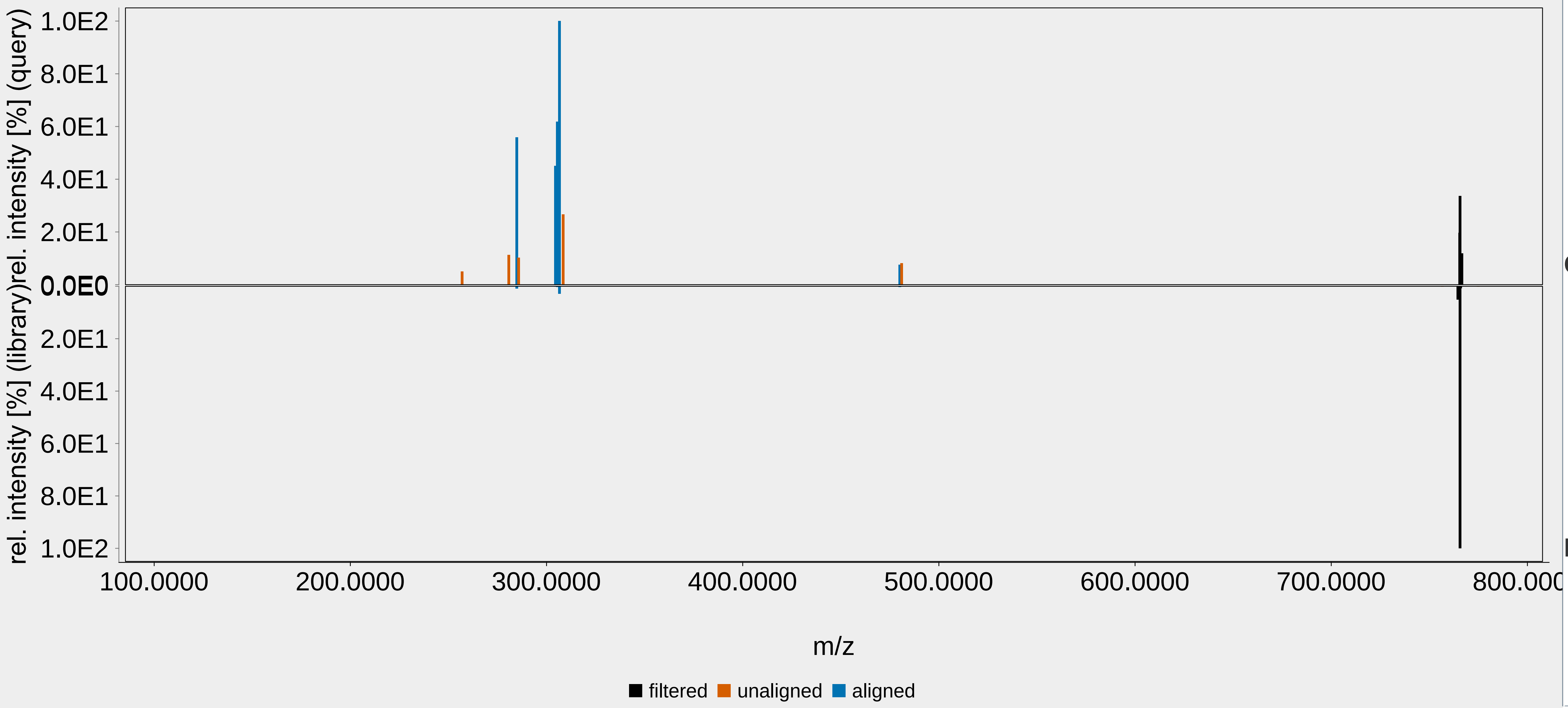

Compound information

Name: PE(18:0/20:3); [M-H]- C43H79N1O8P1

Exact mass: 0.0

Ion type: M-H

Precursor m/z: 768.554

Charge: 1

Polarity: Negative

Instrument information

Instrument type: HCD; Velos

Ion source: LC-ESI

Ms level: 2

Acquisition: Commercial

Database links

Other information

Principal investigator: Thomas Metz

Data collector: Thomas Metz

Entry ID: CCMSLIB00010079224

PE(18:0/20:3); [M-H]- C43H79N1O8P1 **0.759**

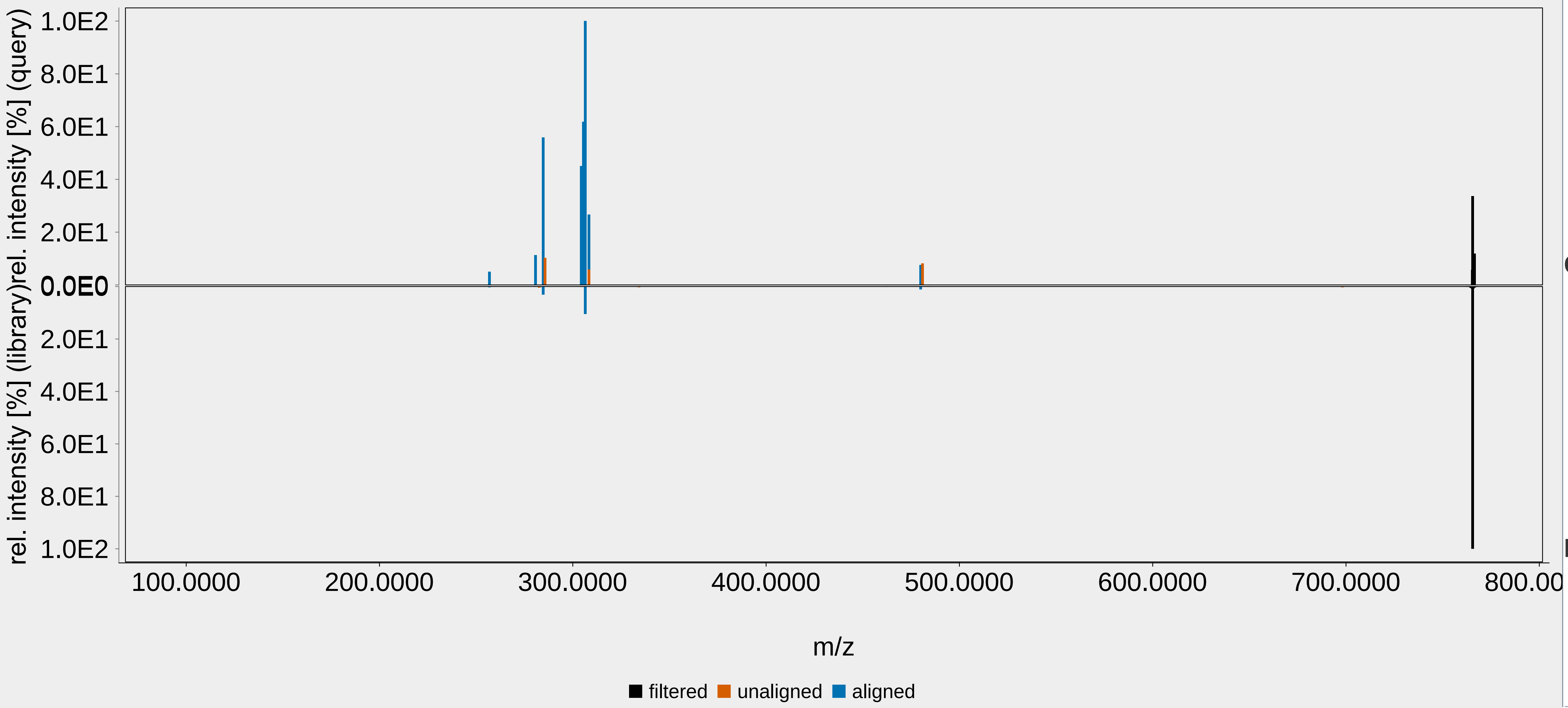

Compound information

Name: PE(18:0/20:3); [M-H]- C43H79N1O8P1

Exact mass: 0.0

Ion type: M-H

Precursor m/z: 768.554

Charge: 1

Polarity: Negative

Instrument information

Instrument type: HCD; Velos

Ion source: LC-ESI

Ms level: 2

Acquisition: Commercial

Database links

Other information

Principal investigator: Thomas Metz

Data collector: Thomas Metz

Entry ID: CCMSLIB00010071376

PA 22:6_22:6 0.946

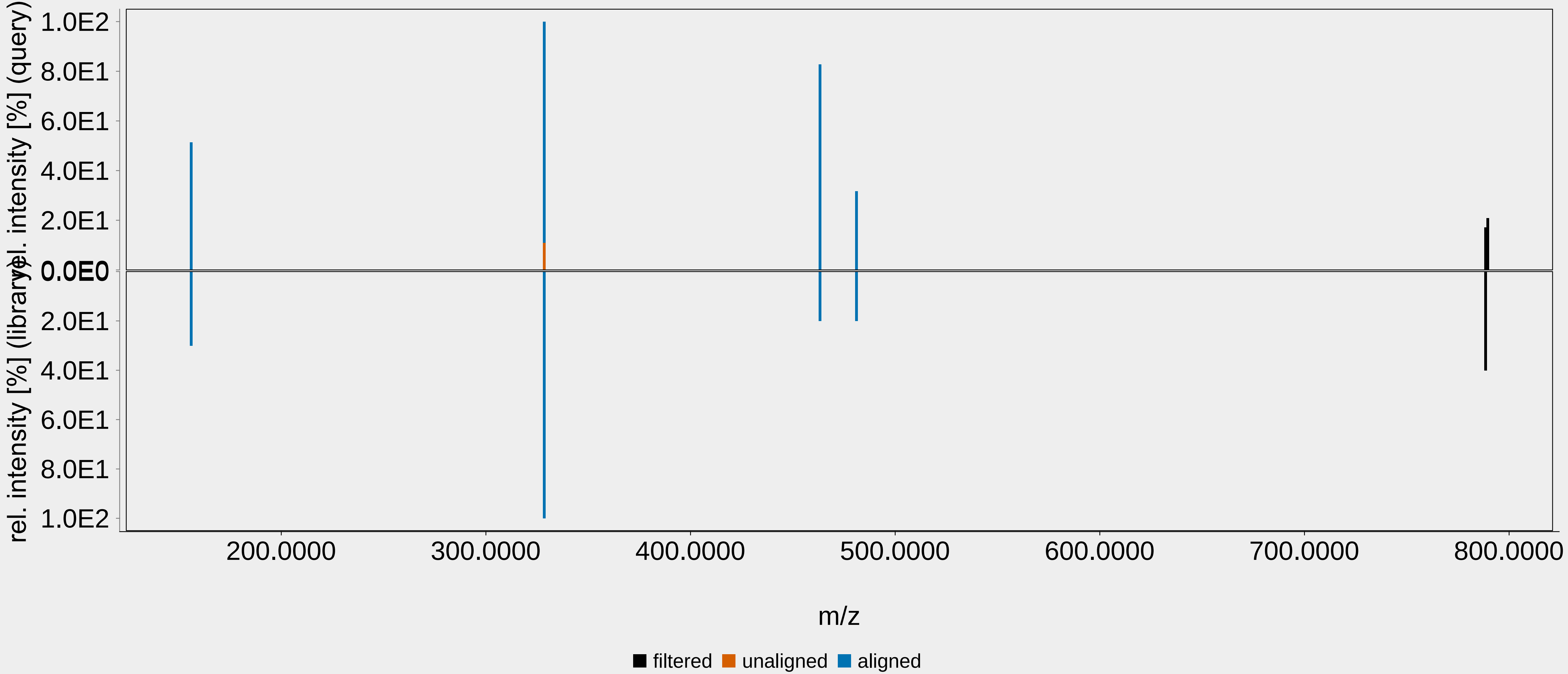

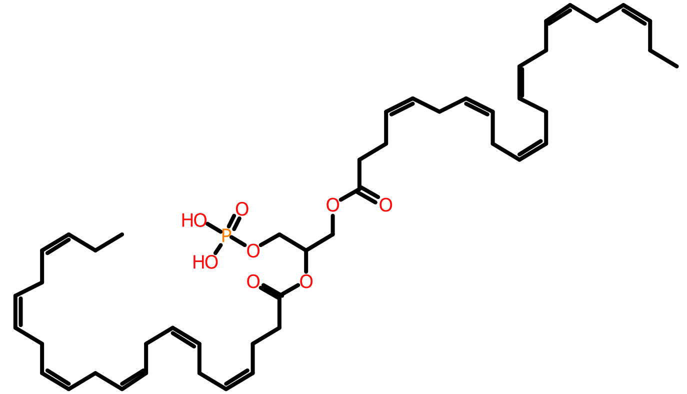

Compound information

Name: PA 22:6_22:6

Formula: C47H69O8P

Ion type: [M-H]-

Precursor m/z: 791.46573

Ccs: 269.85

InChI key: YUDIFVLBAGUYSQ-GZSOIYOPNA-

SMILES:

Num peaks: 5

Instrument information

Database links

Other information

PA(18:0/18:1); [M-H]- C39H74O8P1 **0.862**

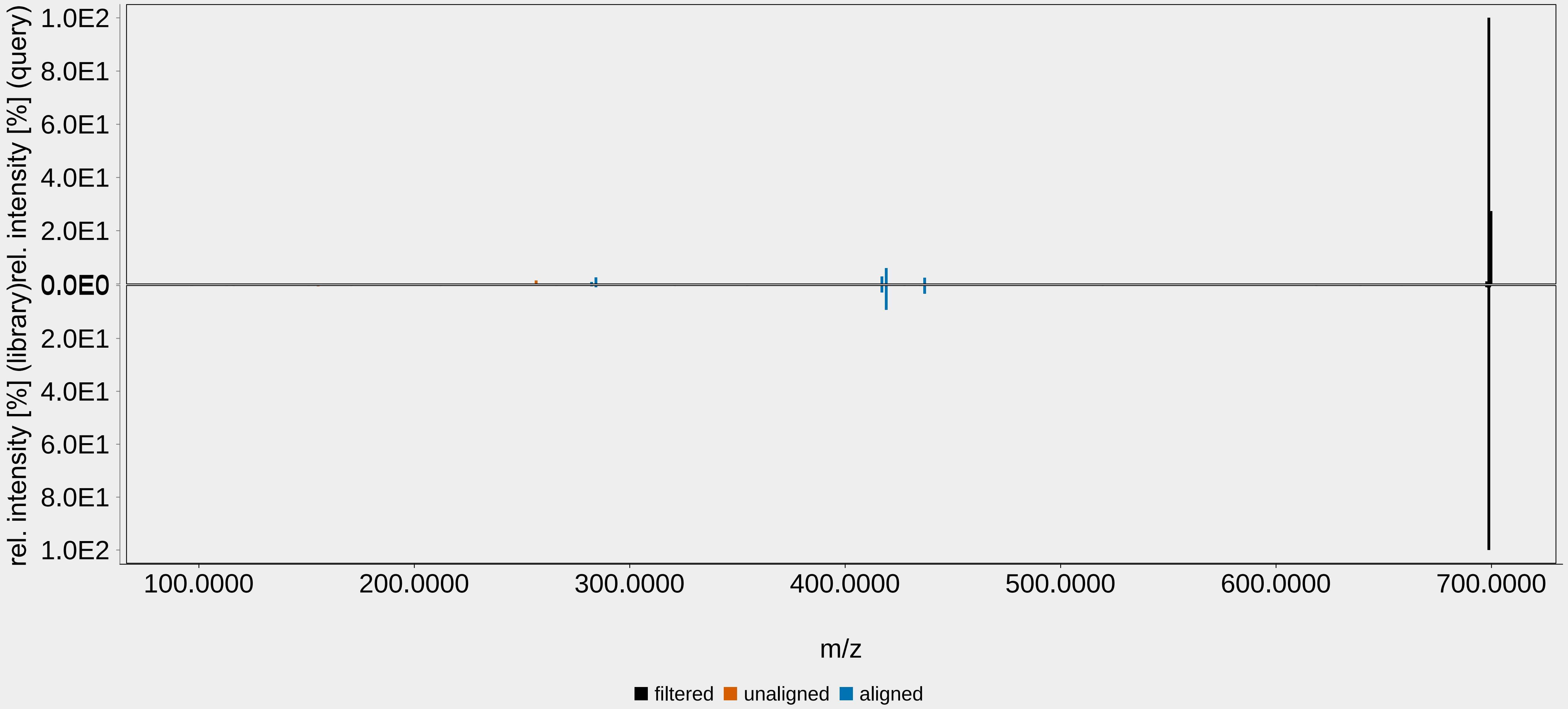

Compound information

Name: PA(18:0/18:1); [M-H]- C39H74O8P1

Exact mass: 0.0

Ion type: M-H

Precursor m/z: 701.512

Charge: 1

Polarity: Negative

Instrument information

Instrument type: HCD; Velos

Ion source: LC-ESI

Ms level: 2

Acquisition: Commercial

Database links

Other information

Principal investigator: Thomas Metz

Data collector: Thomas Metz

Entry ID: CCMSLIB00010076011

PA 18:0_18:1 **0.895**

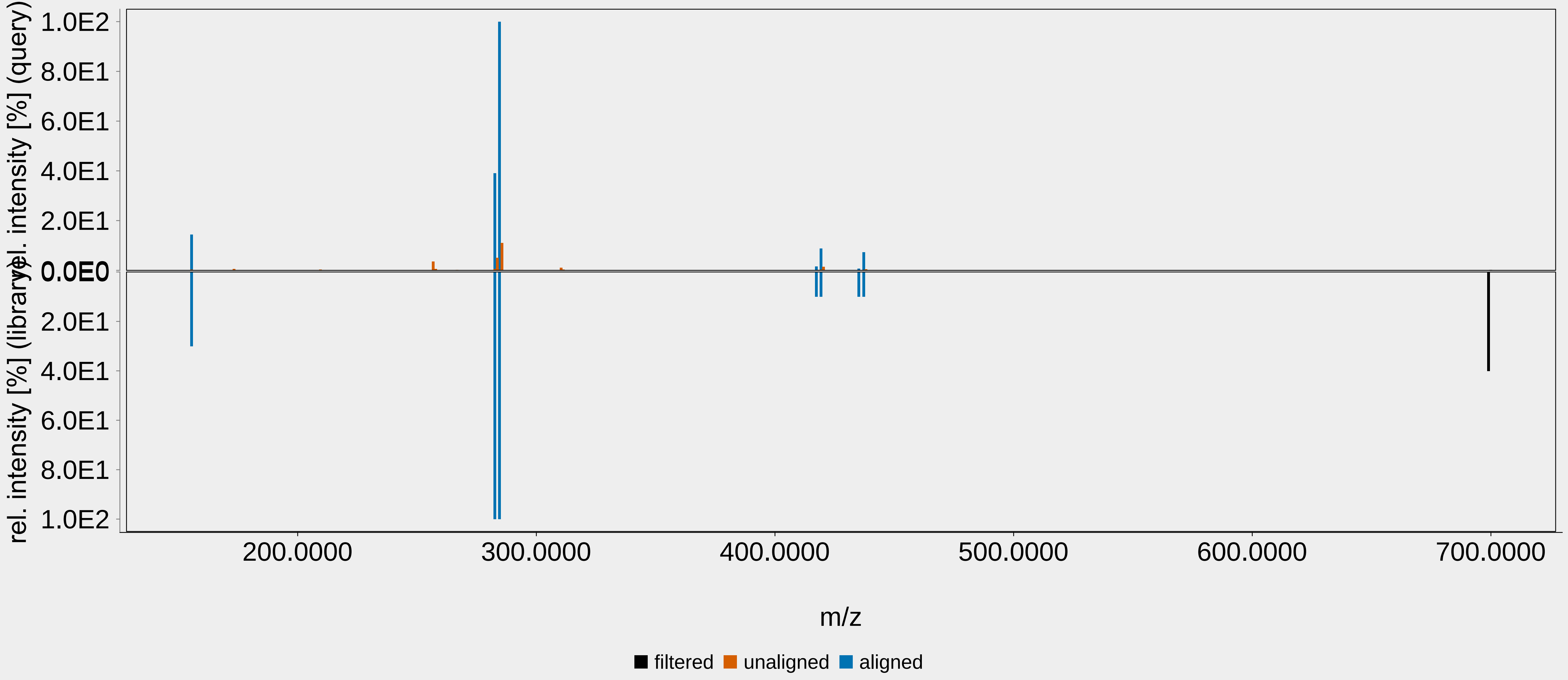

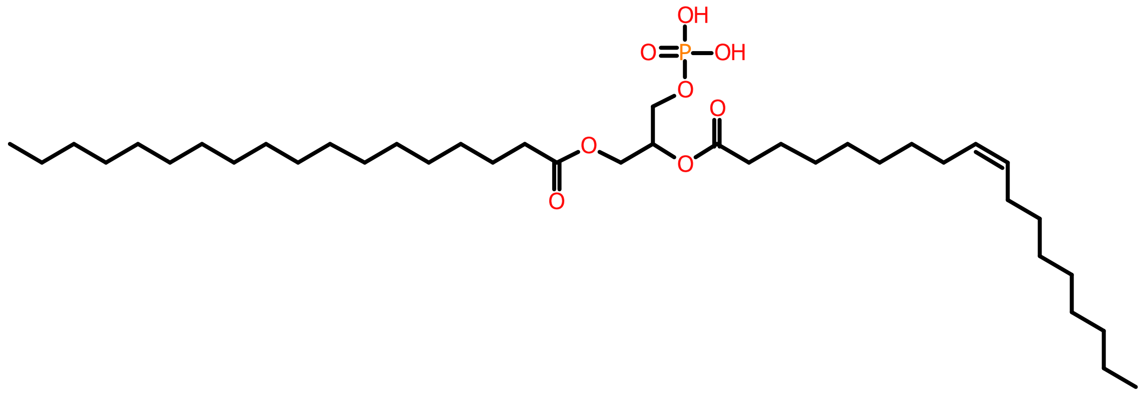

Compound information

Name: PA 18:0_18:1

Formula: C39H75O8P

Ion type: [M-H]-

Precursor m/z: 701.51268

Ccs: 258.98

InChI key:

SMILES:

Num peaks: 8

Instrument information

Database links

Other information

Glutathione - 30.0 eV **0.774**

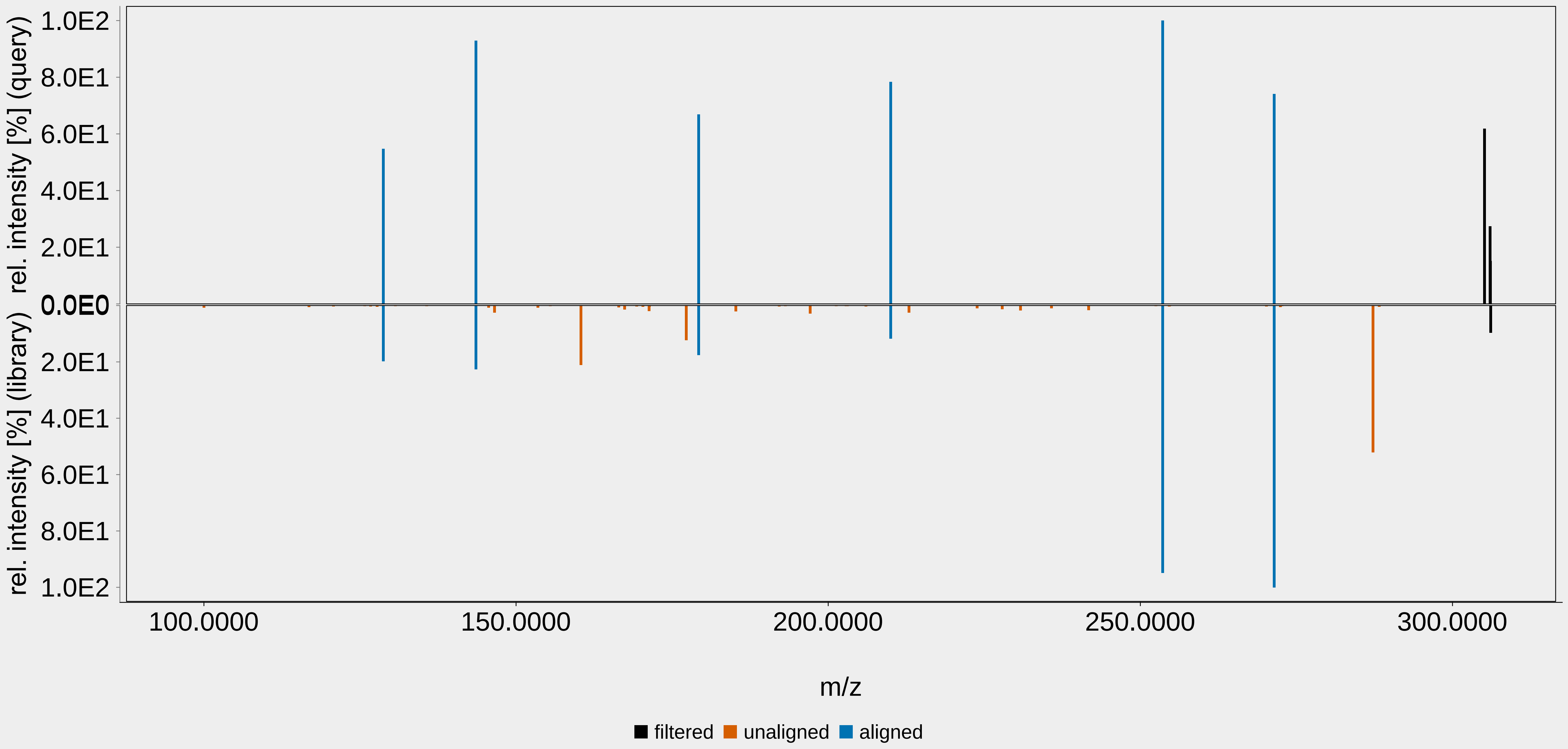

Compound information

Name: Glutathione - 30.0 eV

Exact mass: 0.0

Ion type: M-H

Precursor m/z: 306.077

Charge: 1

Polarity: Negative

SMILES:

Instrument information

Instrument type: Orbitrap

Ion source: ESI

Ms level: 2

Acquisition: Isolated

Database links

CAS:

Other information

Principal investigator: PI

Data collector: Data Collector

Entry ID: CCMSLIB00006121887

Glutathione - 30.0 eV

0.776

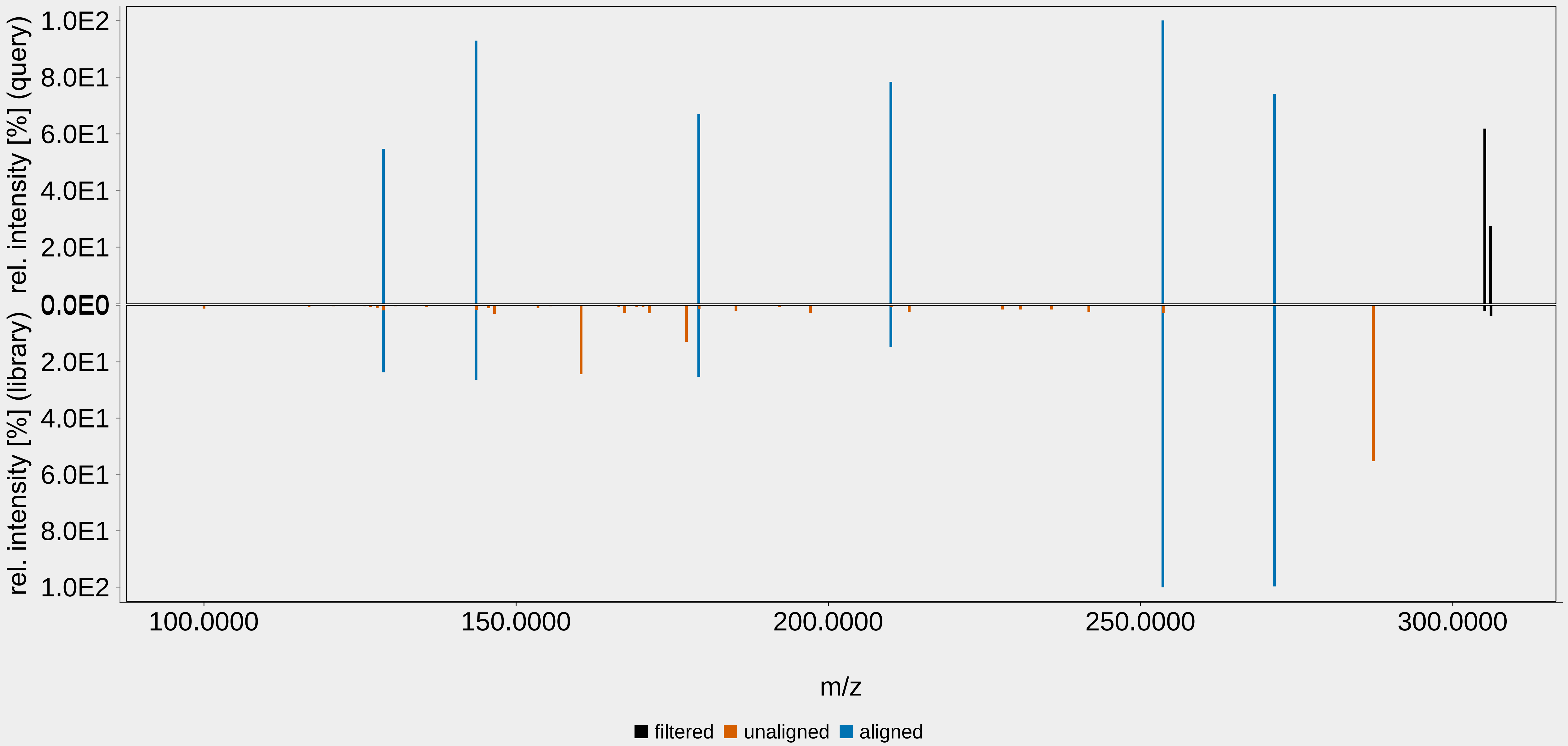

Compound information

Name: Glutathione - 30.0 eV

Exact mass: 0.0

Ion type: M-H

Precursor m/z: 306.077

Charge: 1

Polarity: Negative

SMILES:

Instrument information

Instrument type: Orbitrap

Ion source: ESI

Ms level: 2

Acquisition: Isolated

Database links

CAS:

Other information

Principal investigator: PI

Data collector: Data Collector

Entry ID: CCMSLIB00006121879

PS 24:0_18:1

0.866

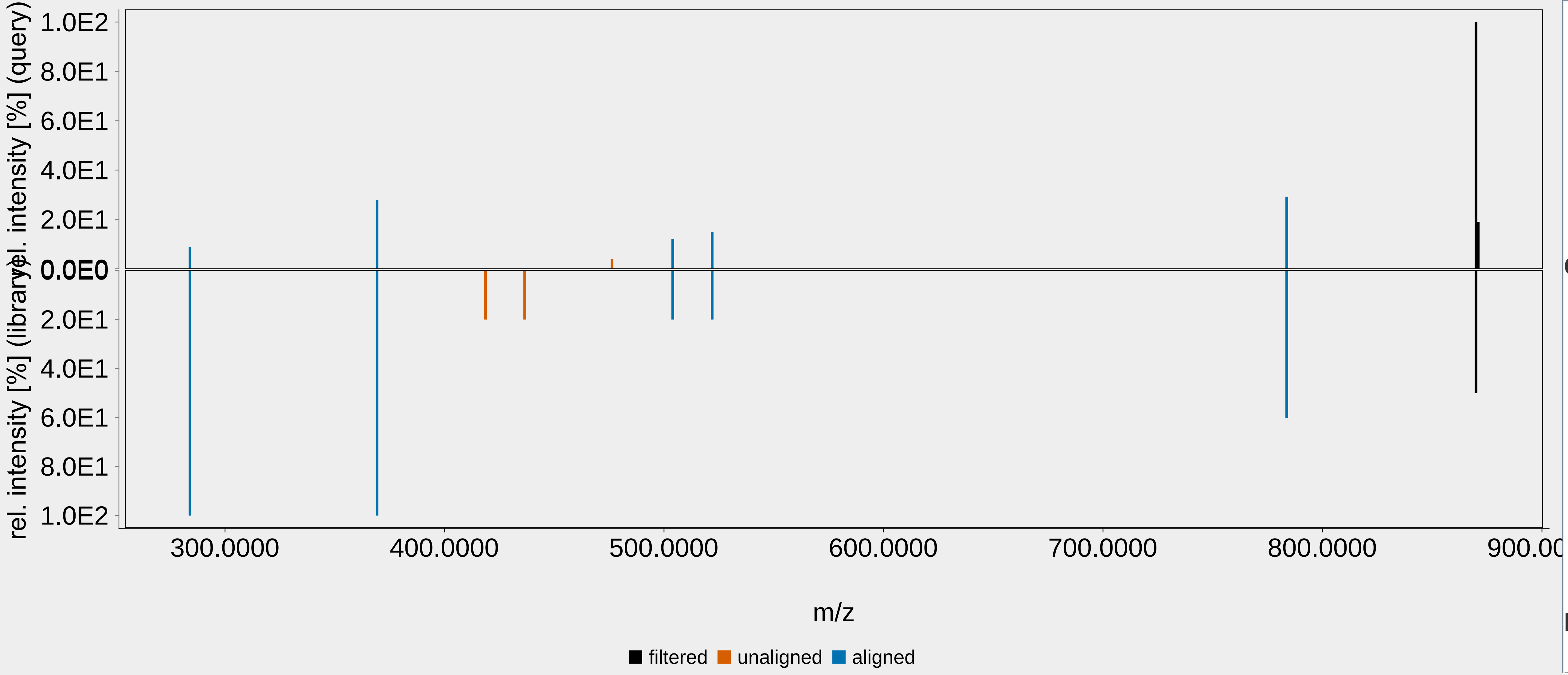

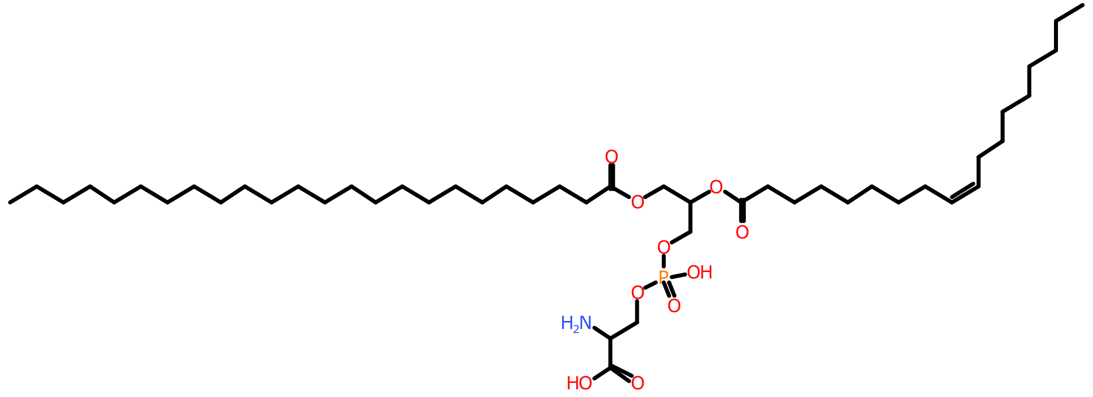

Compound information

Name: PS 24:0_18:1

Formula: C48H92NO10P

Ion type: [M-H]-

Precursor m/z: 872.63861

Ccs: 294.58

InChI key:

SMILES:

Num peaks: 8

Instrument information

Database links

Other information

PE(16:0/22:6); [M-H]- C43H73N1O8P1 **0.944**

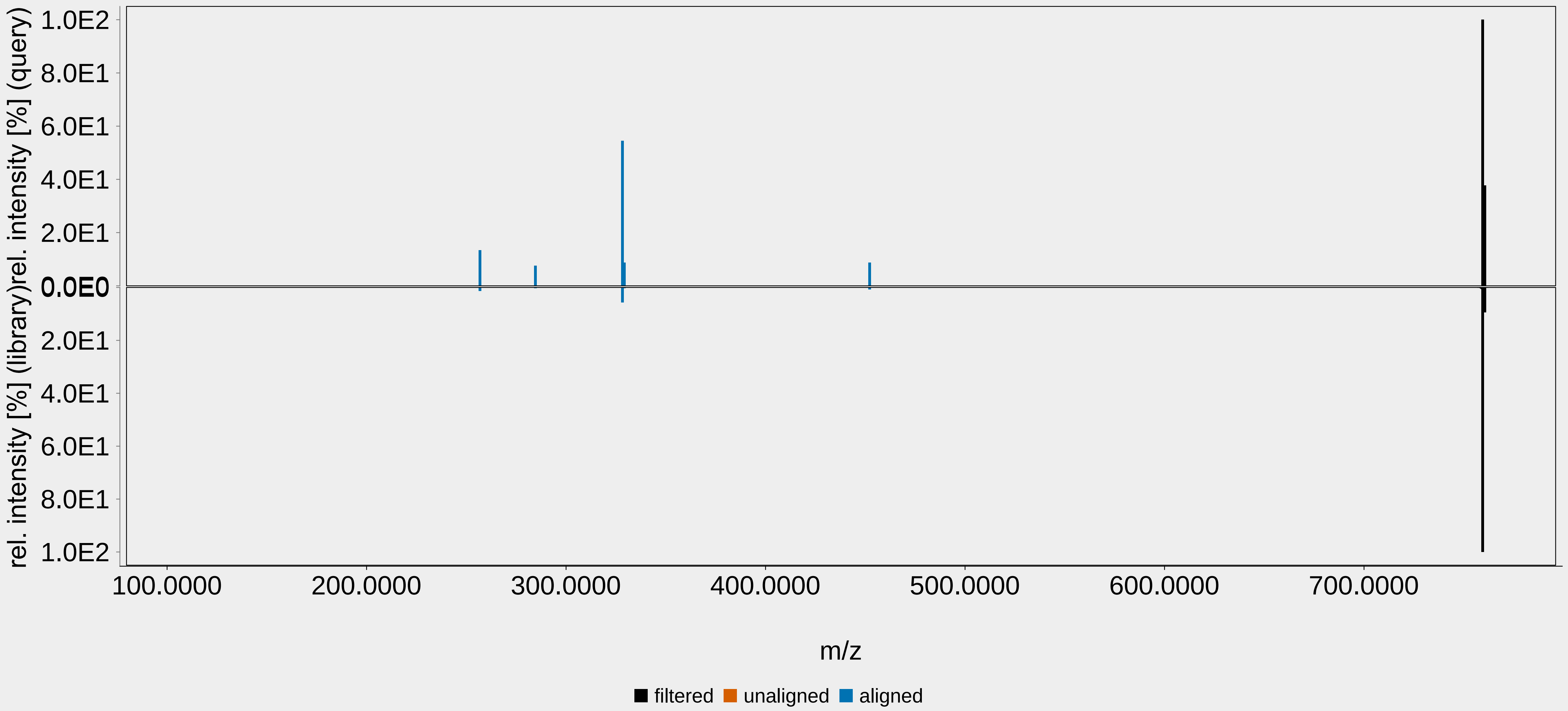

Compound information

Name: PE(16:0/22:6); [M-H]- C43H73N1O8P1

Exact mass: 0.0

Ion type: M-H

Precursor m/z: 762.507

Charge: 1

Polarity: Negative

Instrument information

Instrument type: HCD; Velos

Ion source: LC-ESI

Ms level: 2

Acquisition: Commercial

Database links

Other information

Principal investigator: Thomas Metz

Data collector: Thomas Metz

Entry ID: CCMSLIB00010088316

PE(16:0/22:6); [M-H]- C43H73N1O8P1 0.940

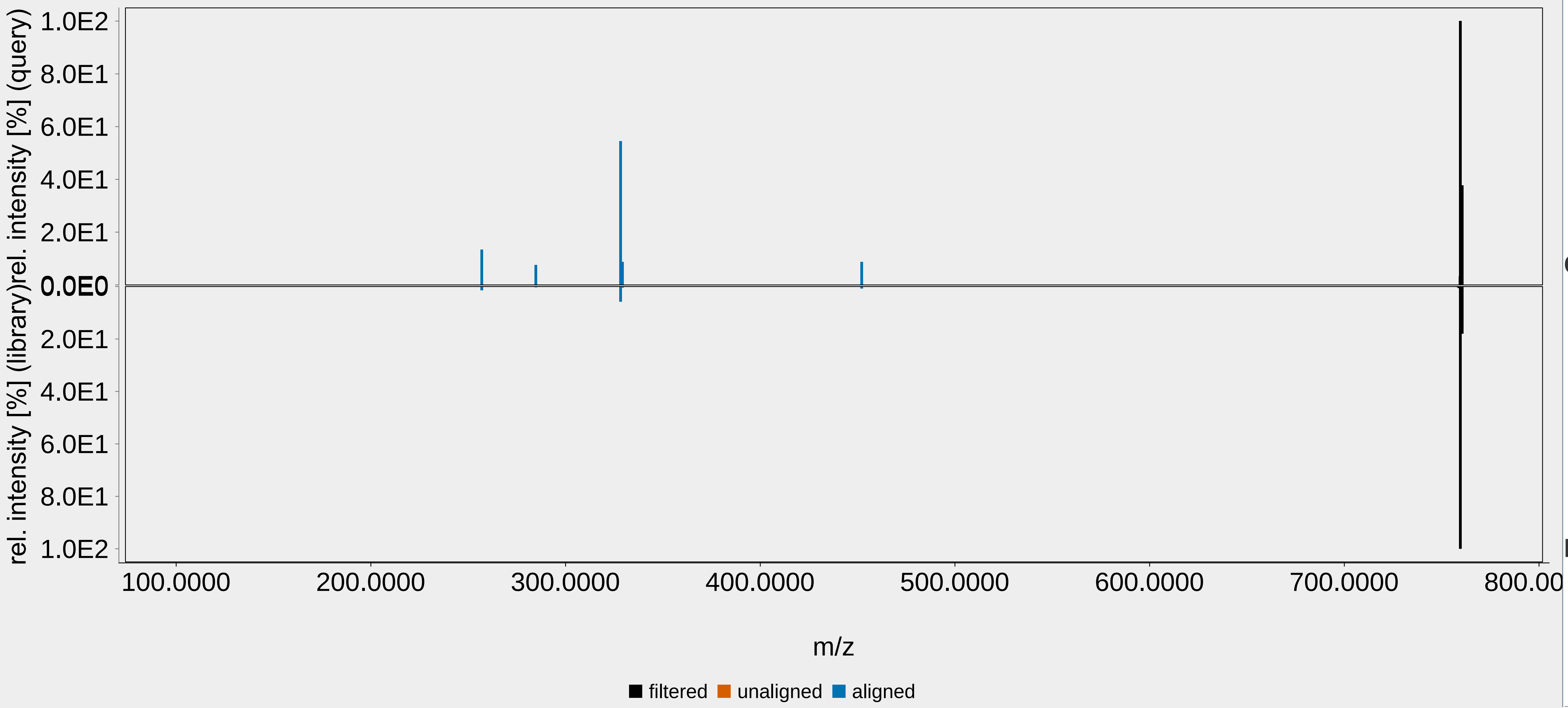

Compound information

Name: PE(16:0/22:6); [M-H]- C43H73N1O8P1

Exact mass: 0.0

Ion type: M-H

Precursor m/z: 762.507

Charge: 1

Polarity: Negative

Instrument information

Instrument type: HCD; Velos

Ion source: LC-ESI

Ms level: 2

Acquisition: Commercial

Database links

Other information

Principal investigator: Thomas Metz

Data collector: Thomas Metz

Entry ID: CCMSLIB00010064176

LPI 22:6

0.830

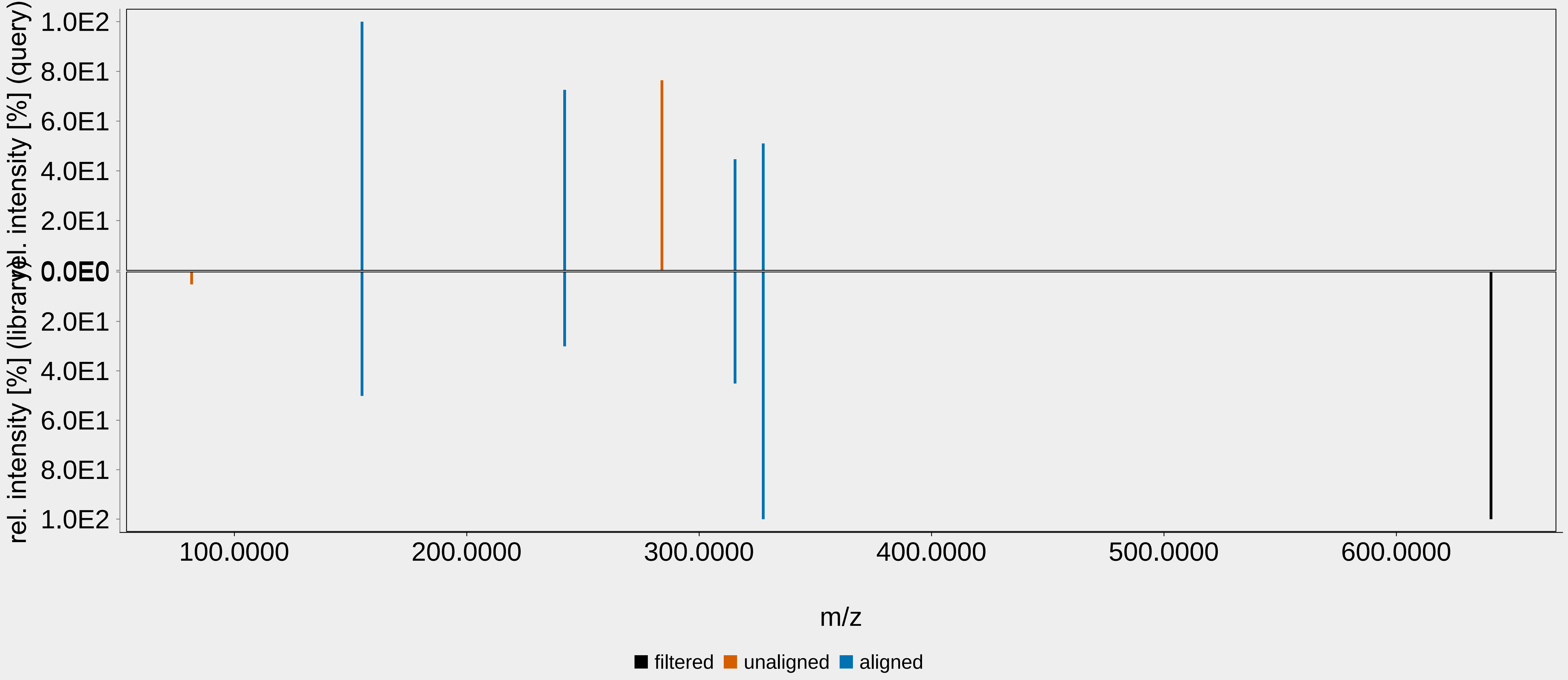

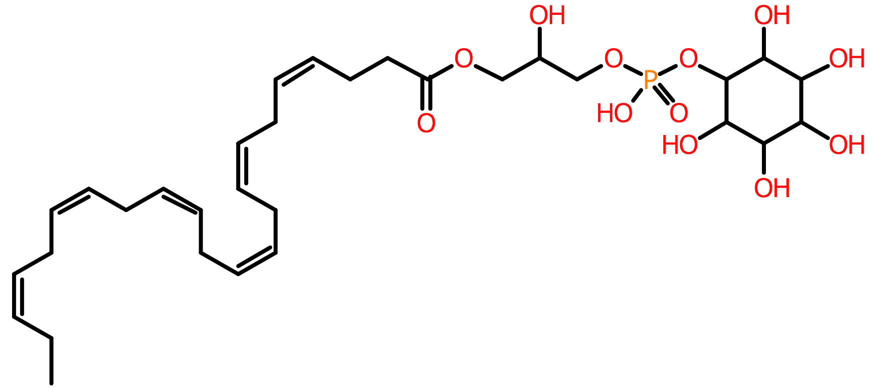

Compound information

Name: LPI 22:6

Formula: C31H49O12P

Ion type: [M-H]-

Precursor m/z: 643.28889

Ccs: 237.14

InChI key:

SMILES:

Num peaks: 6

Instrument information

Database links

Other information

PE(22:4/0:0); [M-H]- C27H47N1O7P1

0.893

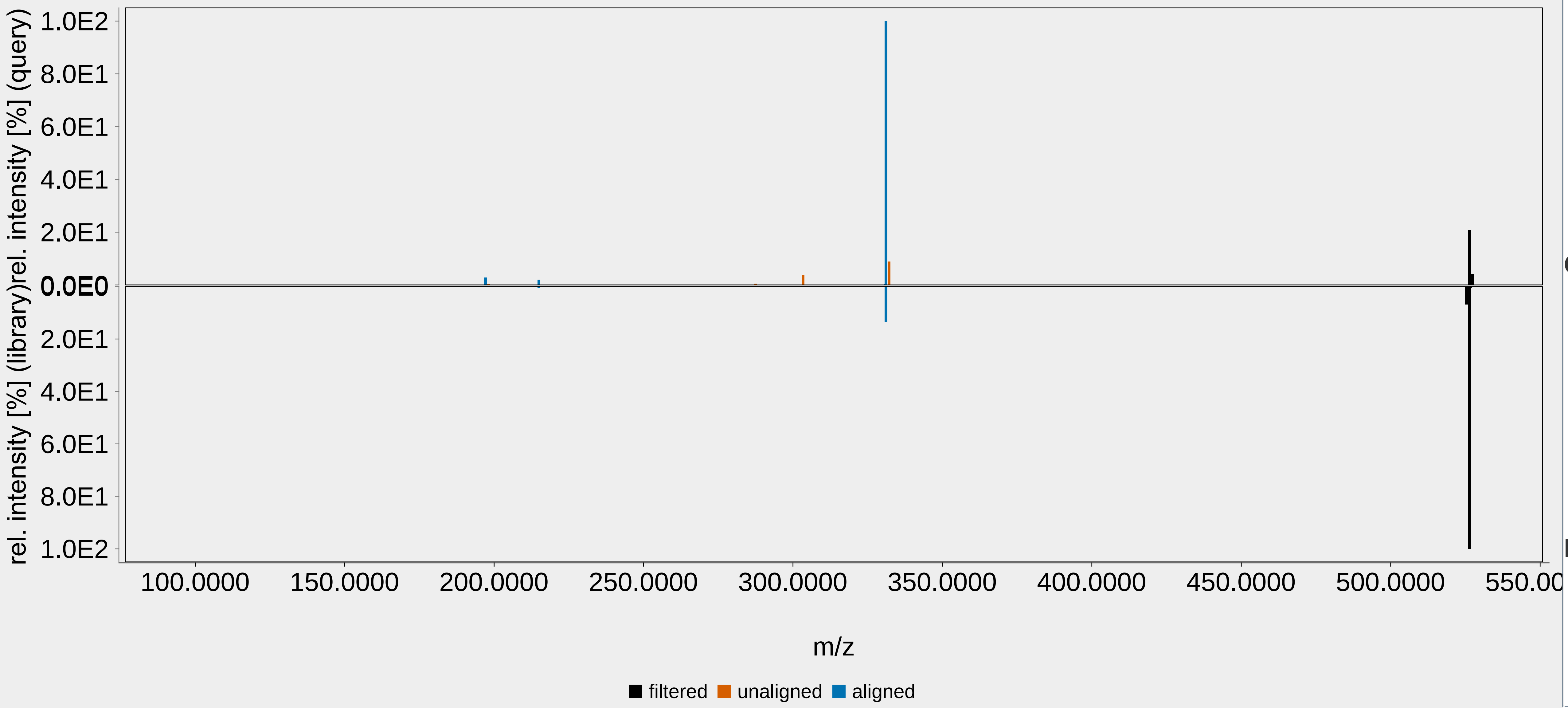

Compound information

Name: PE(22:4/0:0); [M-H]- C27H47N1O7P1

Exact mass: 0.0

Ion type: M-H

Precursor m/z: 528.309

Charge: 1

Polarity: Negative

Instrument information

Instrument type: HCD; Velos

Ion source: LC-ESI

Ms level: 2

Acquisition: Commercial

Database links

Other information

Principal investigator: Thomas Metz

Data collector: Thomas Metz

Entry ID: CCMSLIB00010086739

PE(22:4/0:0); [M-H]- C27H47N1O7P1 **0.897**

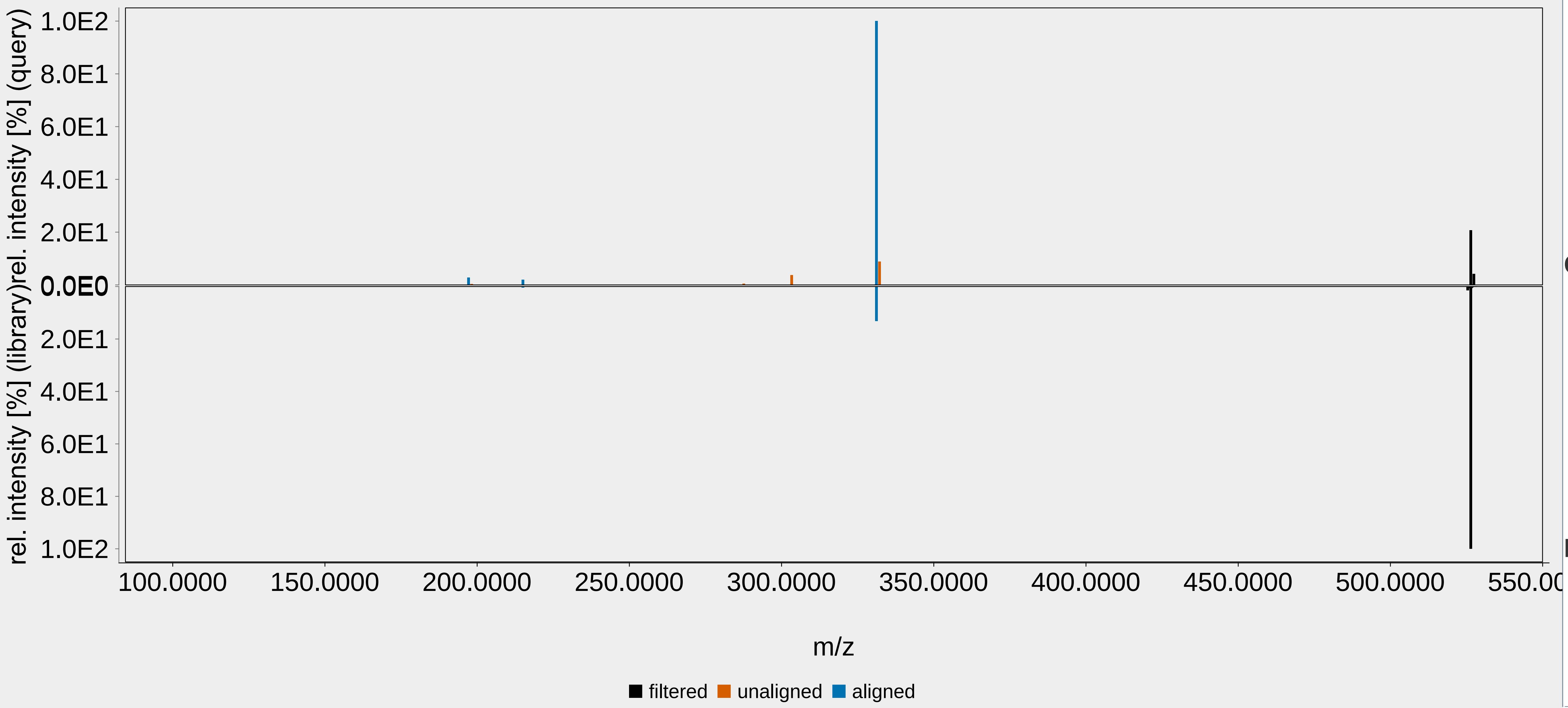

Compound information

Name: PE(22:4/0:0); [M-H]- C27H47N1O7P1

Exact mass: 0.0

Ion type: M-H

Precursor m/z: 528.309

Charge: 1

Polarity: Negative

Instrument information

Instrument type: HCD; Velos

Ion source: LC-ESI

Ms level: 2

Acquisition: Commercial

Database links

Other information

Principal investigator: Thomas Metz

Data collector: Thomas Metz

Entry ID: CCMSLIB00010063574

PC 38:6

0.734

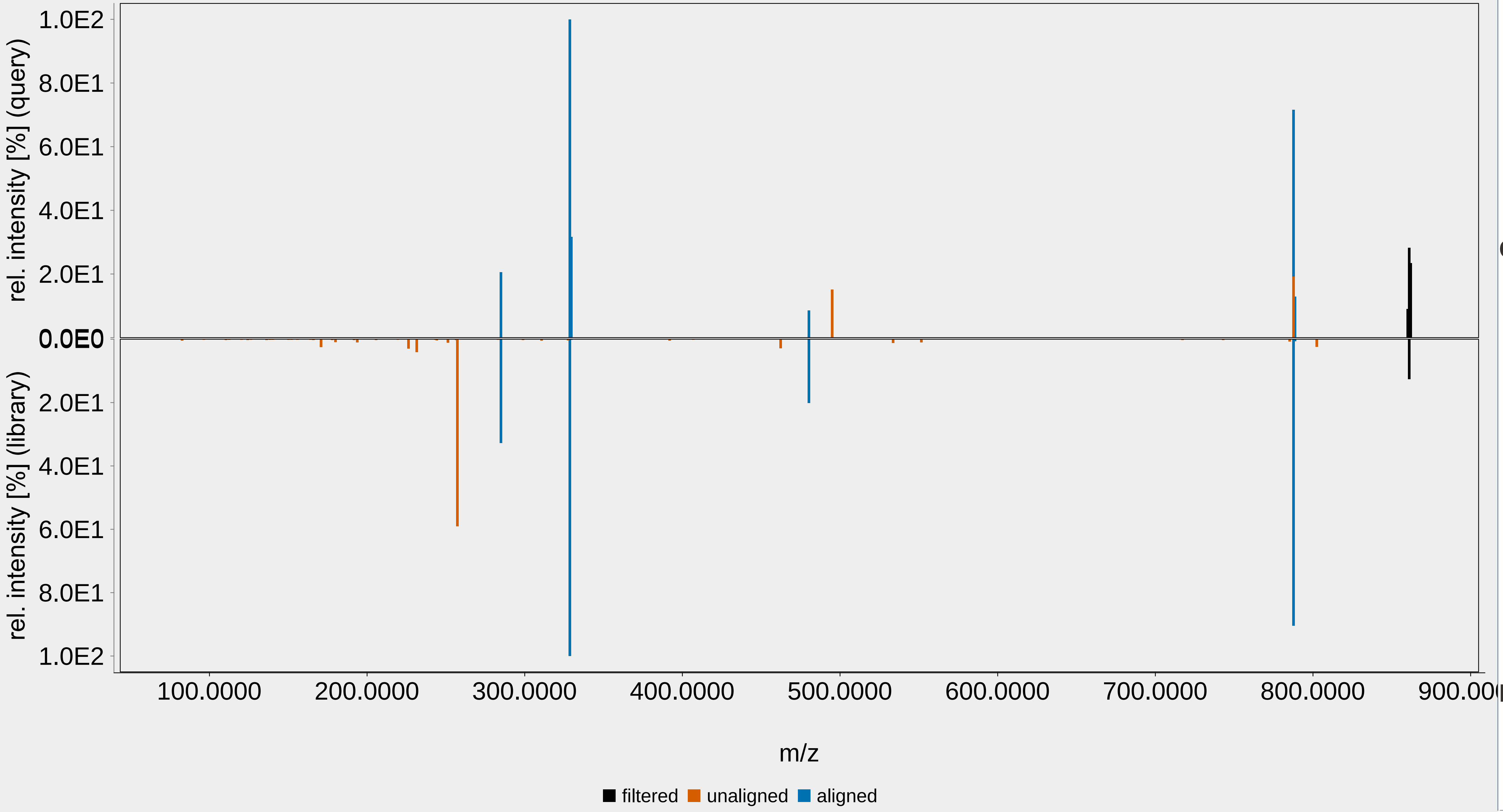

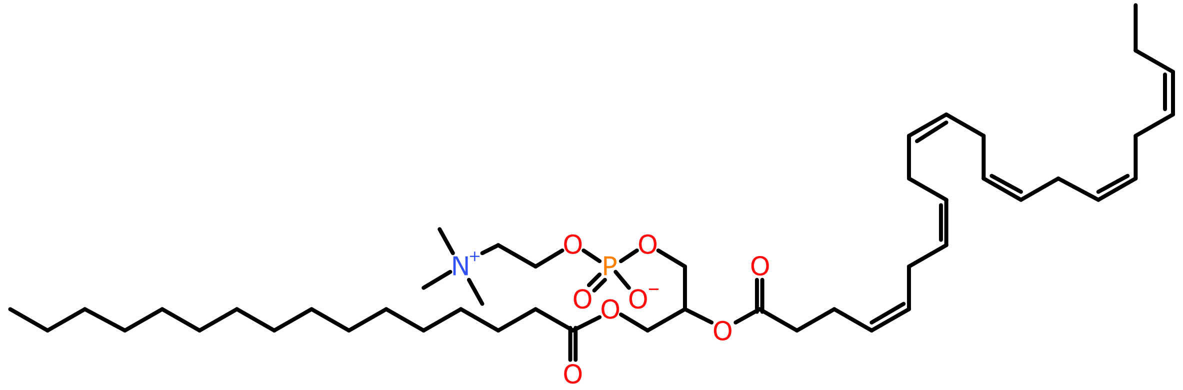

Compound information

Name: PC 38:6

Formula: C46H80NO8P

Mol. weight: 806.1209999999999

Exact mass: 806.1209999999999

Ion type: [M+CH3COOH-H]-

Precursor m/z: 864.5729

RT: 9.202155

Polarity: negative

InChI:

InChI key:

SMILES:

Instrument information

Instrument type: LC-ESI-QTOF

Instrument: AB Sciex TripleTOF 5600+ system

Ms level: MS2

Collision energy: 45V

Database links

Pubchem: 9940652

MoNA ID: LQB00502

Other information

Principal investigator: Kazutaka Ikeda, Hiroshi

Data collector: Kazutaka Ikeda, Hiroshi

PC 38:6

0.734

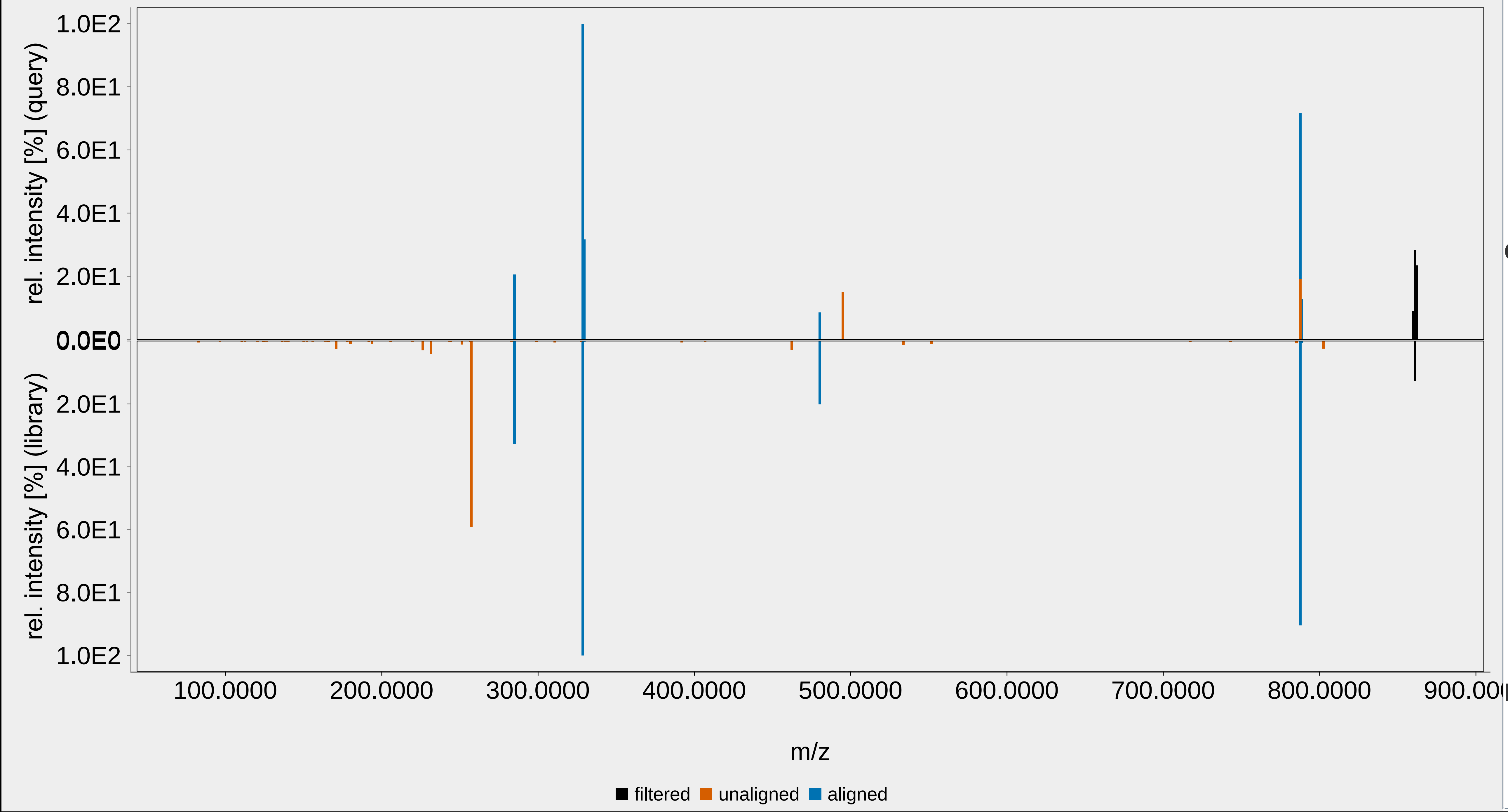

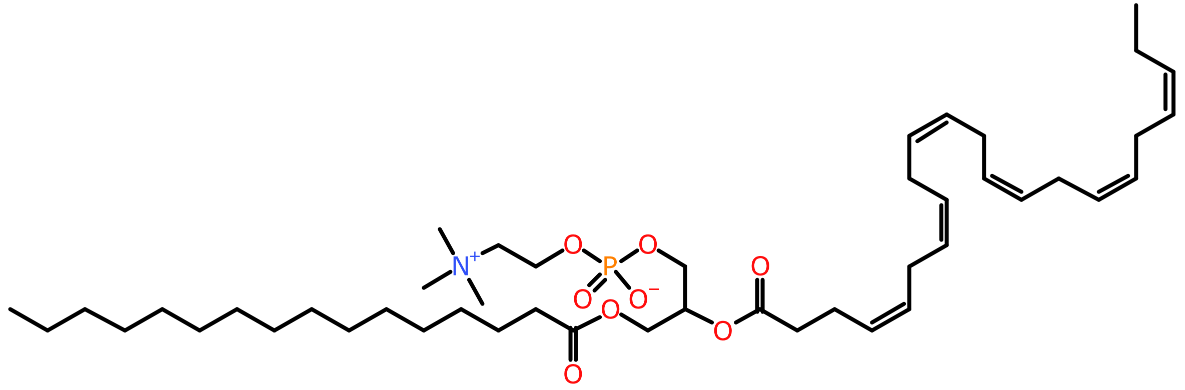

Compound information

Name: PC 38:6

Formula: C46H80NO8P

Mol. weight: 806.1209999999999

Exact mass: 806.1209999999999

Ion type: [M+CH3COOH-H]-

Precursor m/z: 864.5729

RT: 9.202155

Polarity: negative

InChI:

InChI key:

SMILES:

Instrument information

Instrument type: LC-ESI-QTOF

Instrument: AB Sciex TripleTOF 5600+ system

Ms level: MS2

Collision energy: 45V

Database links

Pubchem: 9940652

MoNA ID: LQB00116

Other information

Principal investigator: Kazutaka Ikeda, Hiroshi

Data collector: Kazutaka Ikeda, Hiroshi

PS(18:1_0:0) - 2-amino-3-[hydroxy-[2-hydroxy-3-[octadec-9-enoyl]oxypropoxy]phosphoryl]oxypropanoic acid

0.839

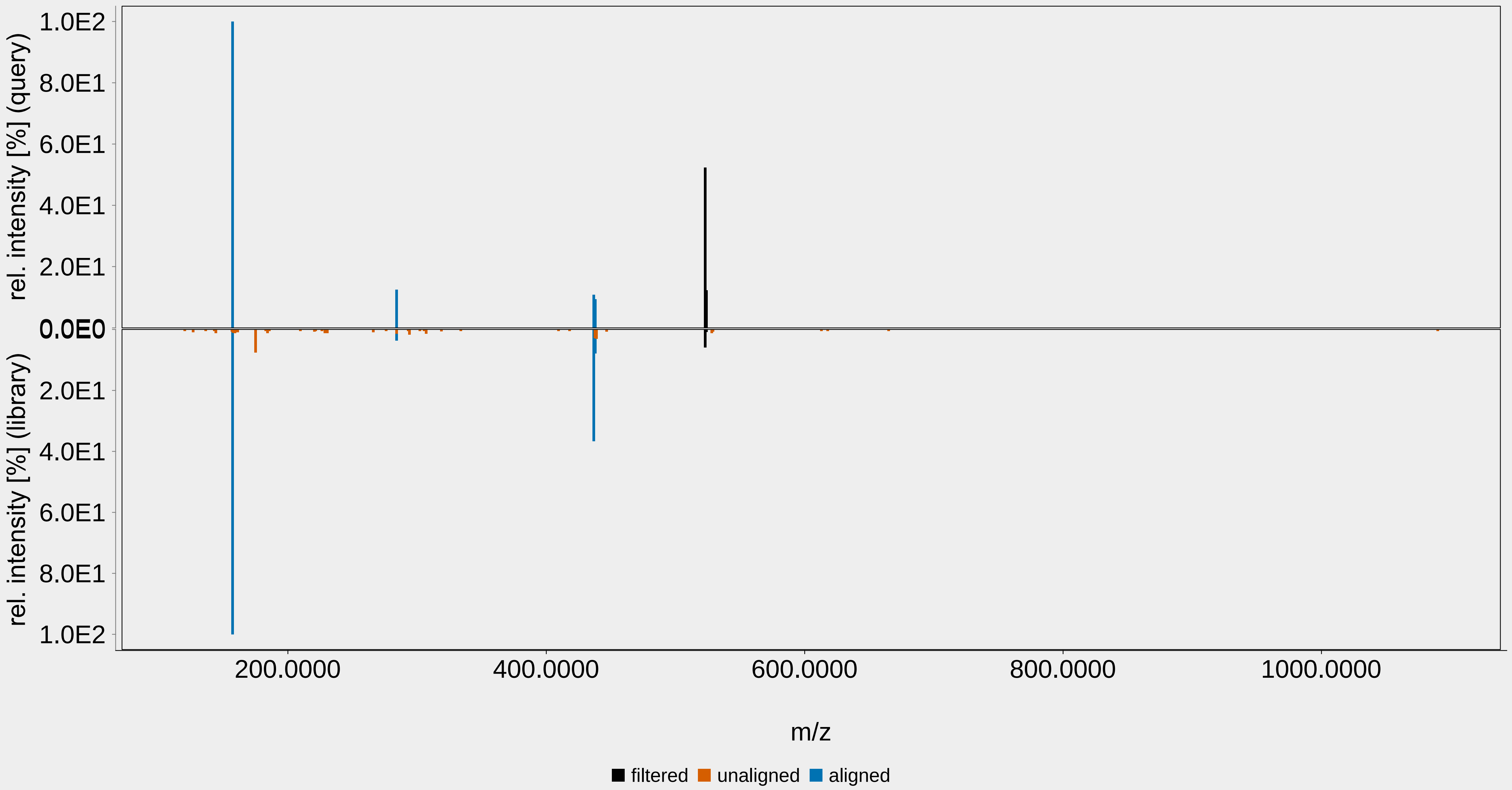

Compound information

Name: PS(18:1_0:0) -

Exact mass: 523.291

Ion type: M-H

Precursor m/z: 522.283

Charge: 1

Polarity: Negative

InChI:

InChI key:

SMILES:

Instrument information

Instrument type: qTof

Ion source: LC-ESI

Ms level: 2

Acquisition: Commercial

Database links

Pubmed:

CAS: 326589-90-6

Other information

Principal investigator: Laprevote

Data collector: UMR 8038 Universite de Paris

Entry ID: CCMSLIB00005436456

2-amino-3-[hydroxy-[2-hydroxy-3-[octadec-9-enoyl]oxypropoxy]phosphoryl]oxypropanoic acid

0.839

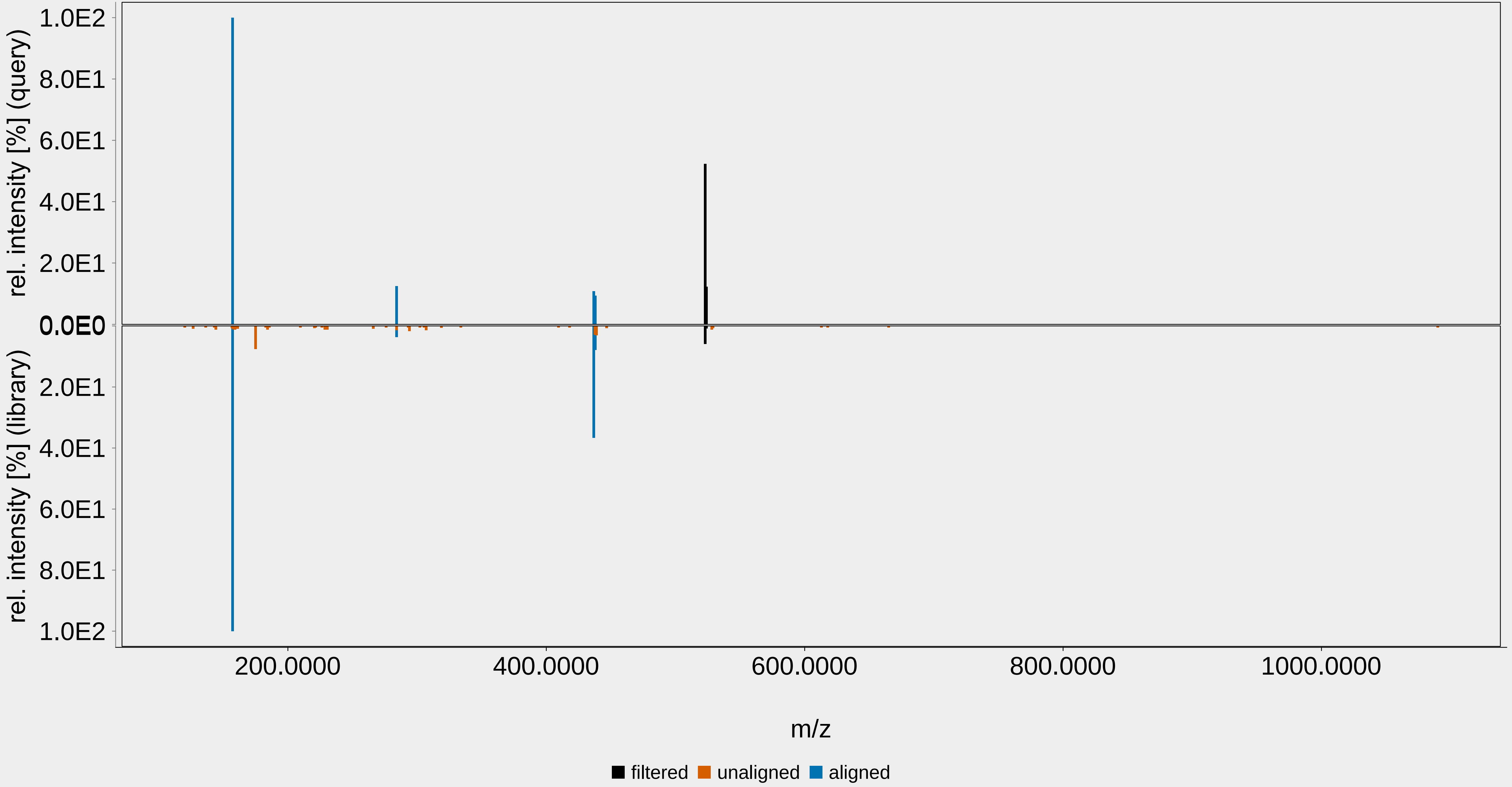

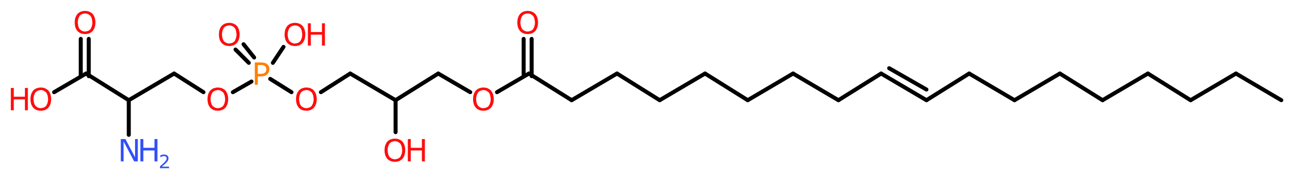

Compound information

Name:

Formula: C24H46NO9P

Mol. weight: 523.291

Exact mass: 523.291

Ion type: [M-H]-

Precursor m/z: 522.283

Polarity: negative

InChI:

InChI key:

SMILES:

Instrument information

Instrument: qTof

Ms level: MS2

Database links

Other information

Principal investigator: Laprevote, UMR 8038

Data collector: Laprevote, UMR 8038 Universite

LNAPE 16:0/N-18:0

0.906

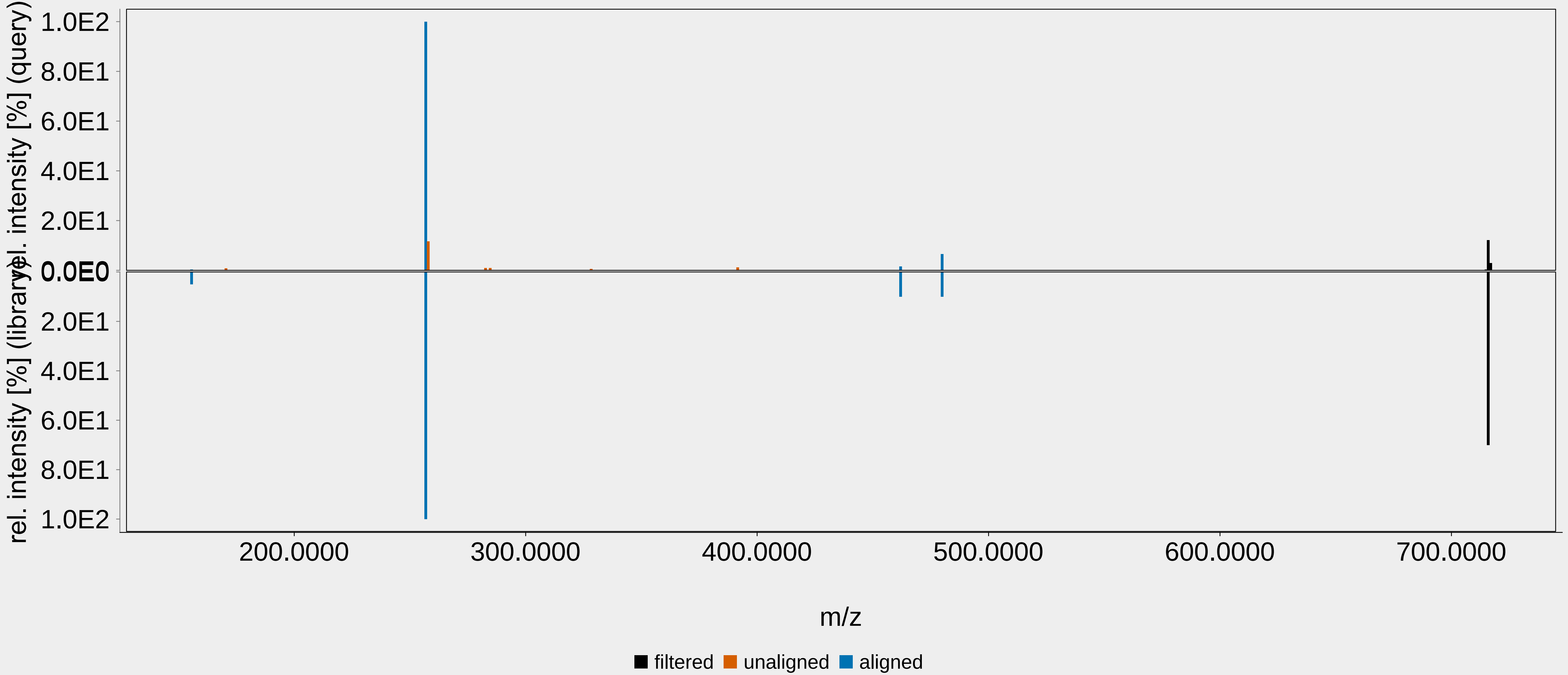

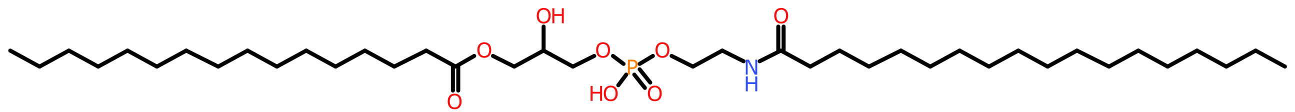

Compound information

Name: LNAPE 16:0/N-18:0

Formula: C39H78NO8P

Ion type: [M-H]-

Precursor m/z: 718.53923

Ccs: 265.73

InChI key:

SMILES:

Num peaks: 5

Instrument information

Database links

Other information

PE(16:0/18:0); [M-H]- C39H77N1O8P1 0.945

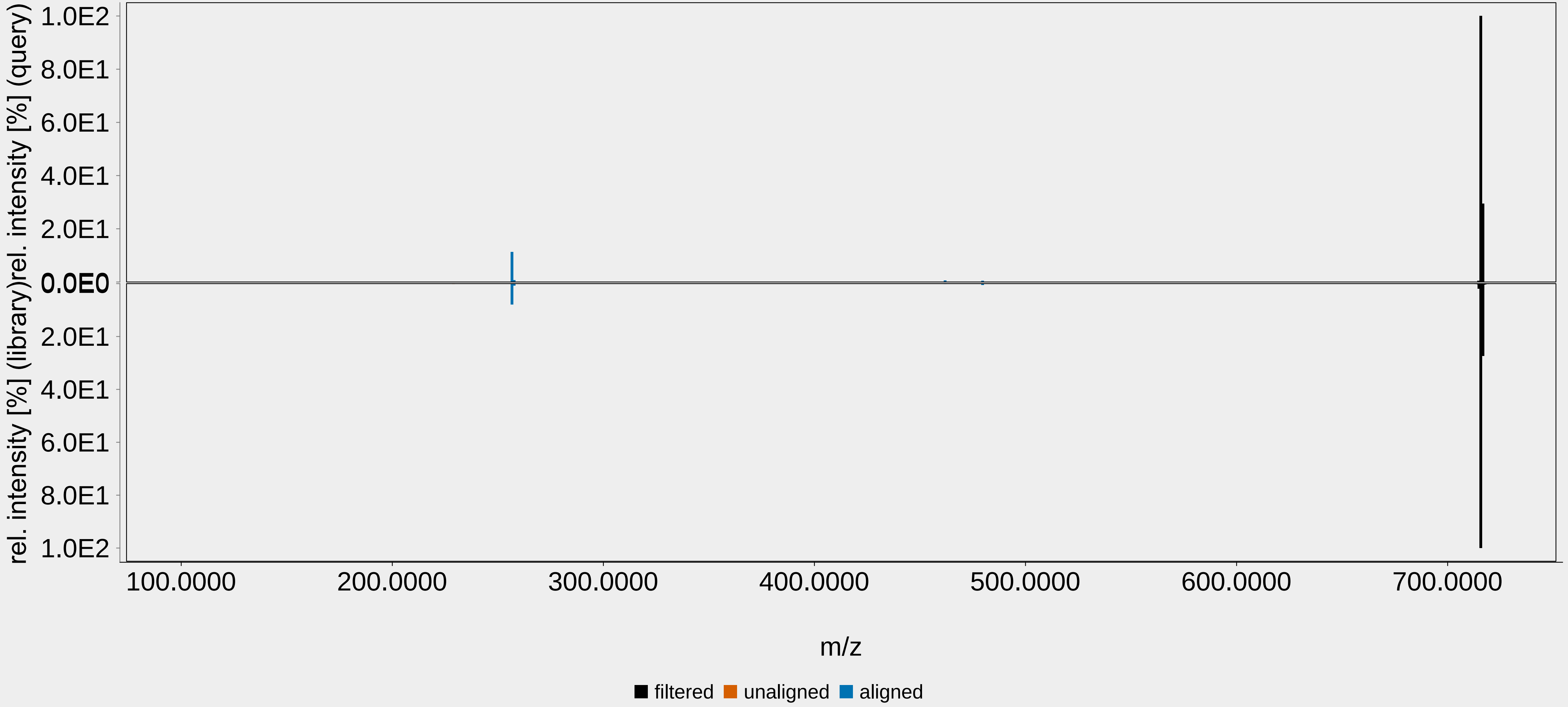

Compound information

Name: PE(16:0/18:0); [M-H]- C39H77N1O8P1

Exact mass: 0.0

Ion type: M-H

Precursor m/z: 718.539

Charge: 1

Polarity: Negative

Instrument information

Instrument type: HCD; Velos

Ion source: LC-ESI

Ms level: 2

Acquisition: Commercial

Database links

Other information

Principal investigator: Thomas Metz

Data collector: Thomas Metz

Entry ID: CCMSLIB00010073693

PE(18:1/22:0); [M-H]- C45H87N1O8P1 **0.729**

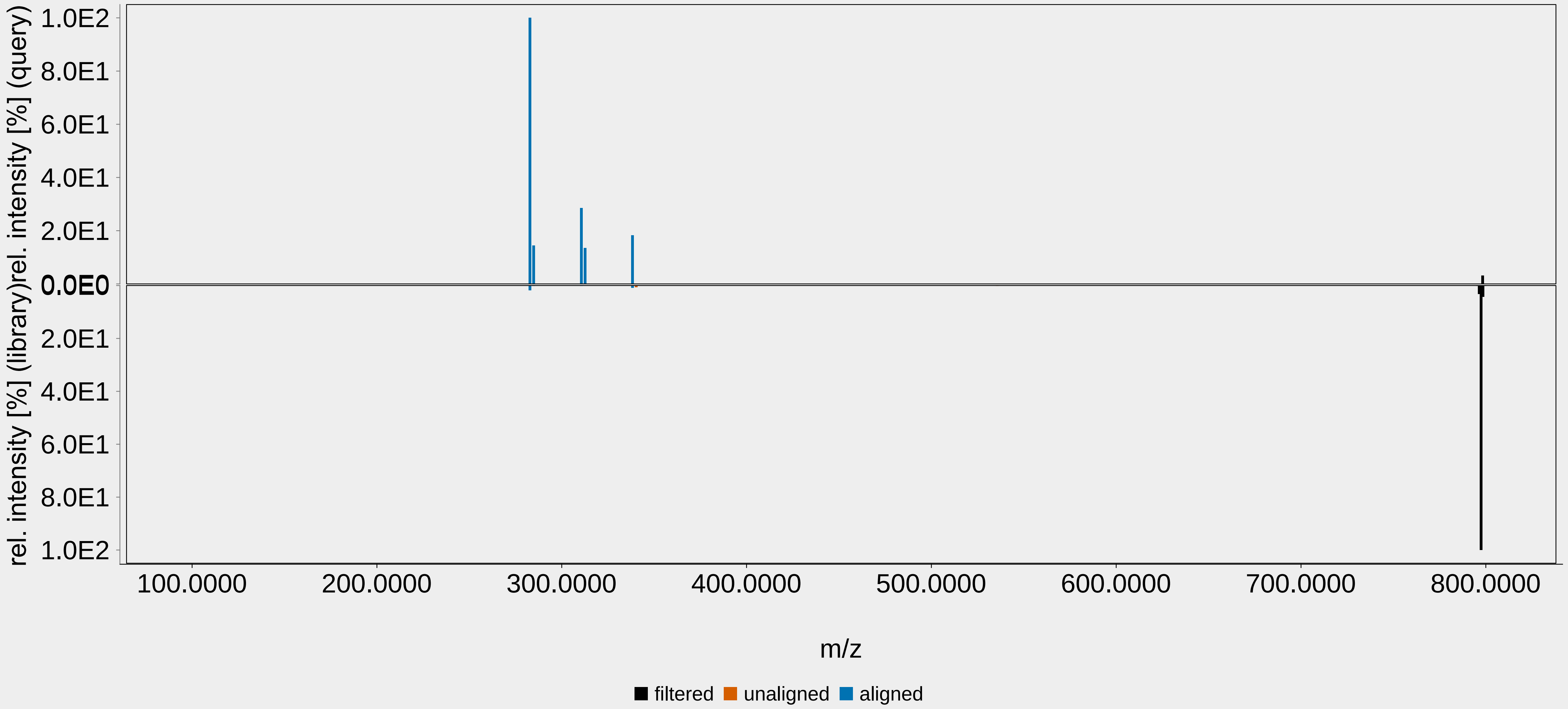

Compound information

Name: PE(18:1/22:0); [M-H]- C45H87N1O8P1

Exact mass: 0.0

Ion type: M-H

Precursor m/z: 800.617

Charge: 1

Polarity: Negative

Instrument information

Instrument type: HCD; Velos

Ion source: LC-ESI

Ms level: 2

Acquisition: Commercial

Database links

Other information

Principal investigator: Thomas Metz

Data collector: Thomas Metz

Entry ID: CCMSLIB00010074098

PE(18:1/22:0); [M-H]- C45H87N1O8P1

0.785

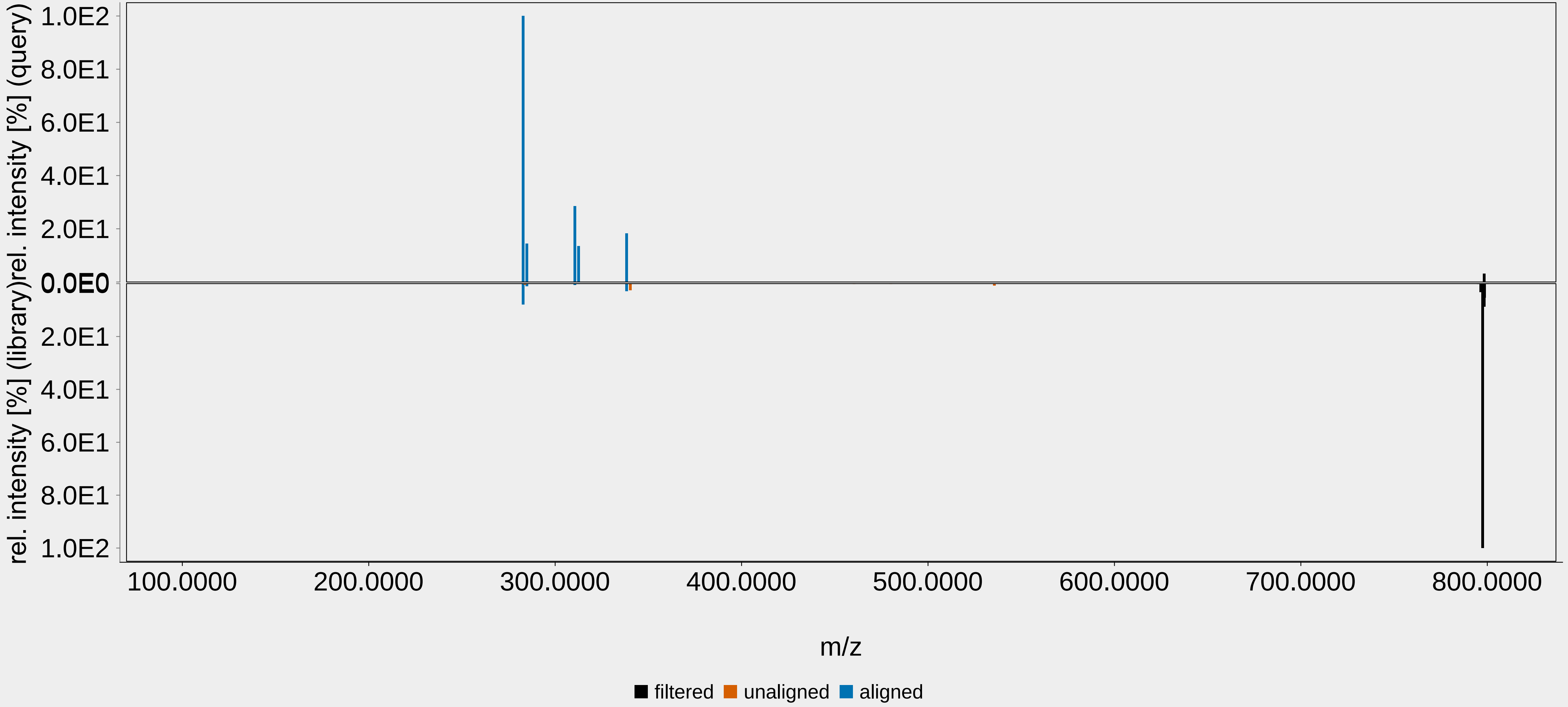

Compound information

Name: PE(18:1/22:0); [M-H]- C45H87N1O8P1

Exact mass: 0.0

Ion type: M-H

Precursor m/z: 800.617

Charge: 1

Polarity: Negative

Instrument information

Instrument type: HCD; Velos

Ion source: LC-ESI

Ms level: 2

Acquisition: Commercial

Database links

Other information

Principal investigator: Thomas Metz

Data collector: Thomas Metz

Entry ID: CCMSLIB00010087976

PE(18:0/22:4); [M-H]- C45H81N1O8P1

0.864

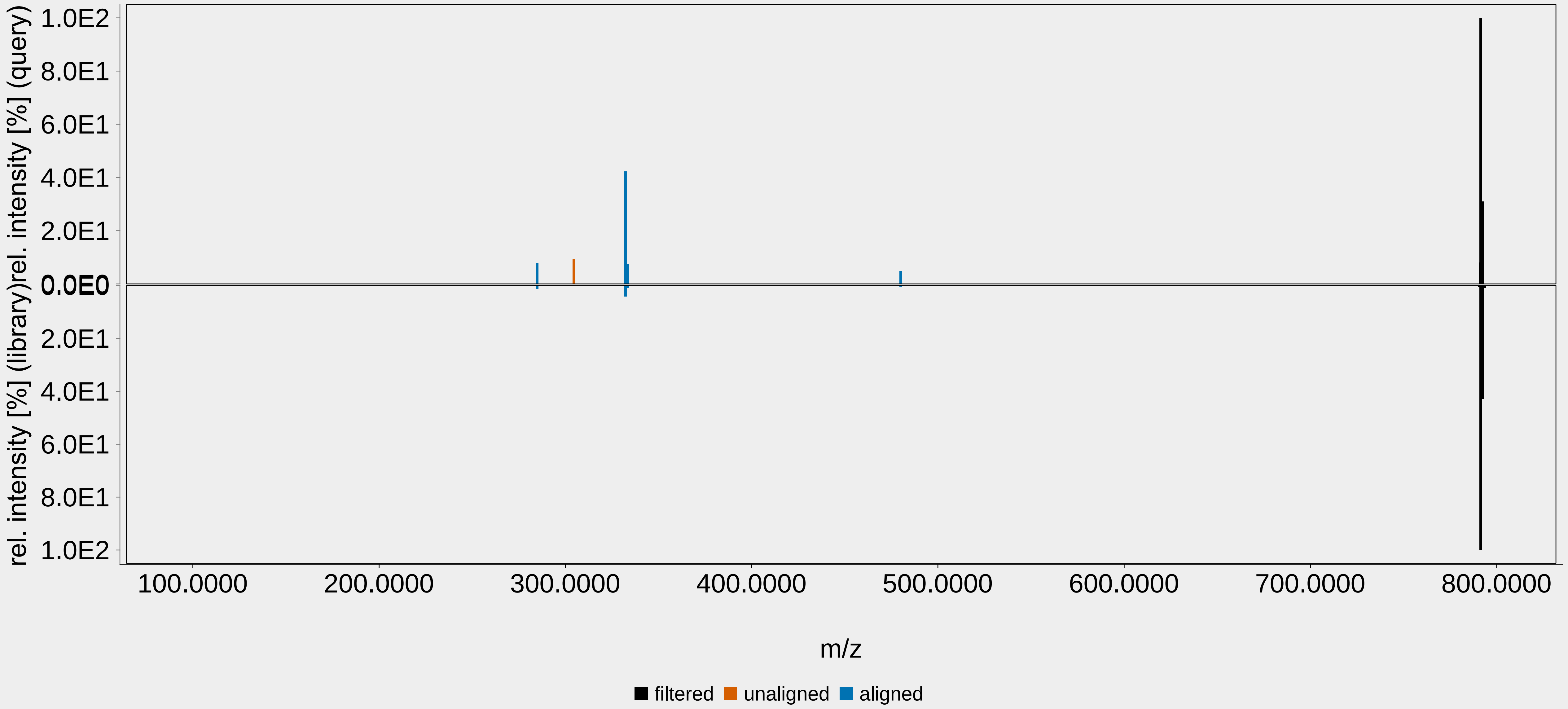

Compound information

Name: PE(18:0/22:4); [M-H]- C45H81N1O8P1

Exact mass: 0.0

Ion type: M-H

Precursor m/z: 794.57

Charge: 1

Polarity: Negative

Instrument information

Instrument type: HCD; Velos

Ion source: LC-ESI

Ms level: 2

Acquisition: Commercial

Database links

Other information

Principal investigator: Thomas Metz

Data collector: Thomas Metz

Entry ID: CCMSLIB00010085505

PE(18:0/22:4); [M-H]- C45H81N1O8P1

0.865

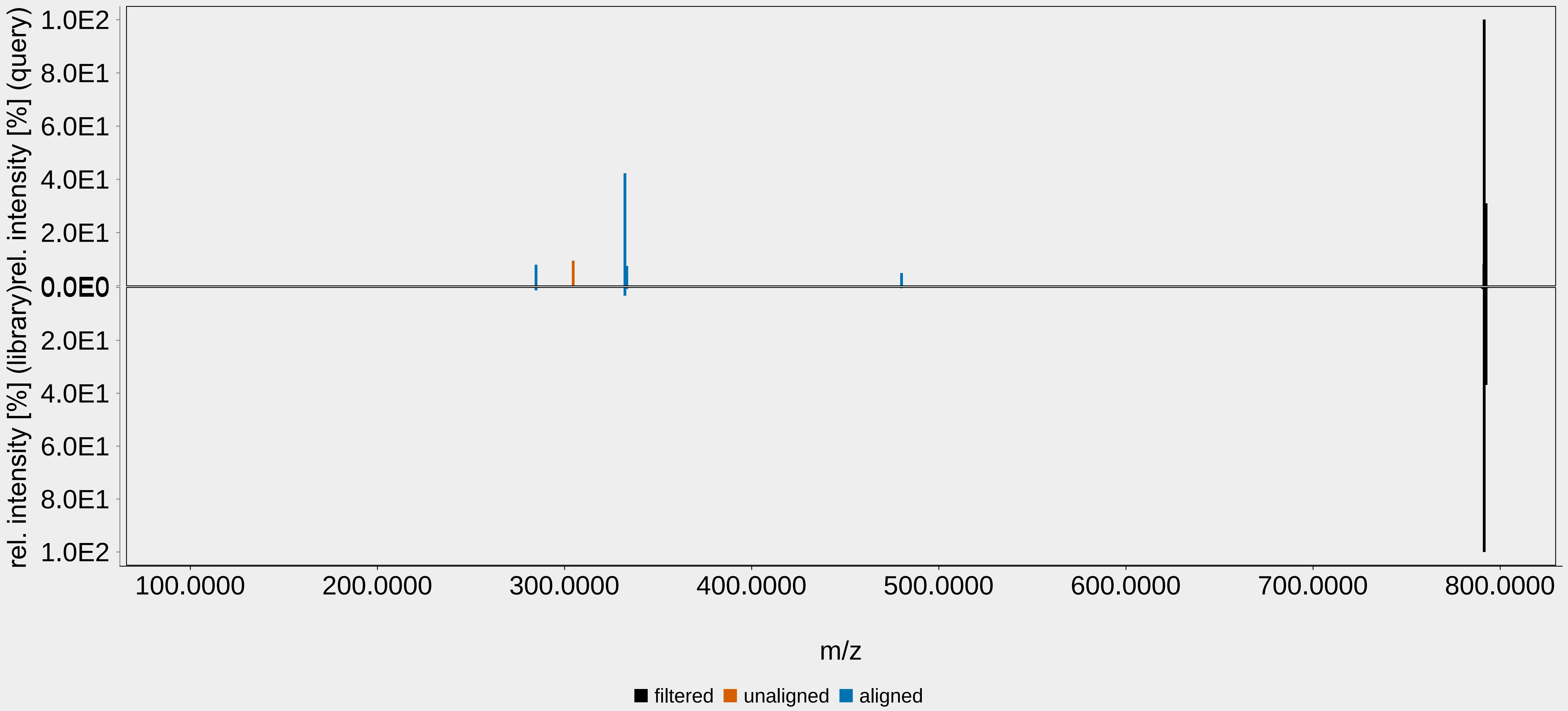

Compound information

Name: PE(18:0/22:4); [M-H]- C45H81N1O8P1

Exact mass: 0.0

Ion type: M-H

Precursor m/z: 794.57

Charge: 1

Polarity: Negative

Instrument information

Instrument type: HCD; Velos

Ion source: LC-ESI

Ms level: 2

Acquisition: Commercial

Database links

Other information

Principal investigator: Thomas Metz

Data collector: Thomas Metz

Entry ID: CCMSLIB00010083860

PS 38:4

0.855

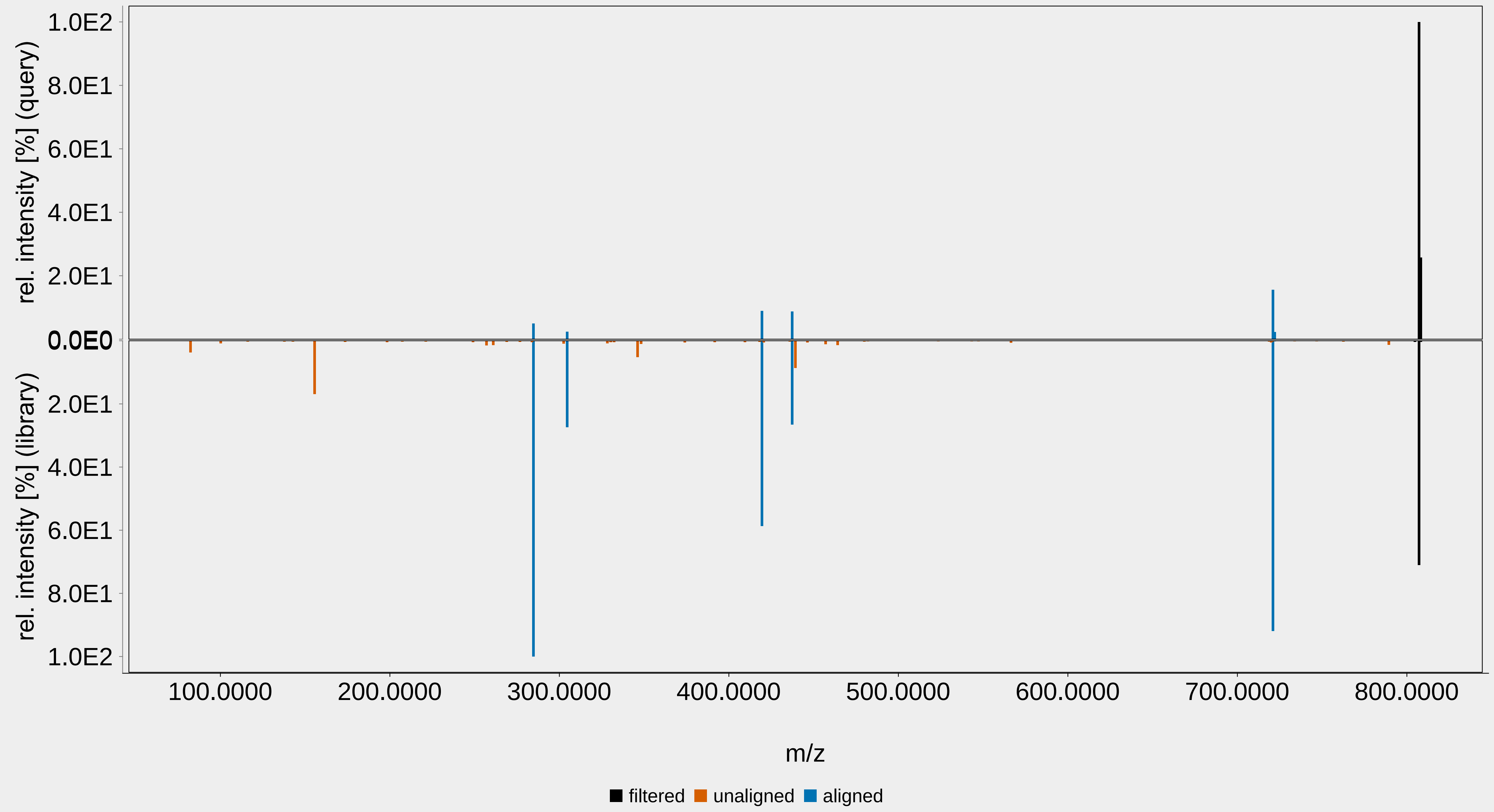

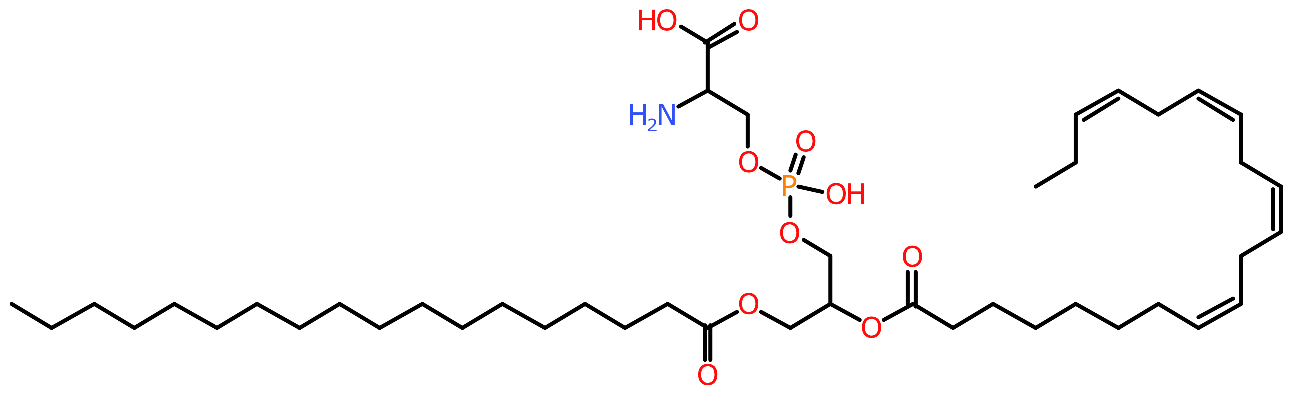

Compound information

Name: PS 38:4

Formula: C44H78NO10P

Mol. weight: 812.0809999999999

Exact mass: 812.0809999999999

Ion type: [M-H]-

Precursor m/z: 810.5303

RT: 8.778674

Polarity: negative

InChI:

InChI key:

SMILES:

Instrument information

Instrument type: LC-ESI-QTOF

Instrument: AB Sciex TripleTOF 5600+ system

Ms level: MS2

Collision energy: 45V

Database links

Pubchem: 138181120

MoNA ID: LQB00722

Other information

Principal investigator: Kazutaka Ikeda, Hiroshi

Data collector: Kazutaka Ikeda, Hiroshi

PS 38:4

0.855

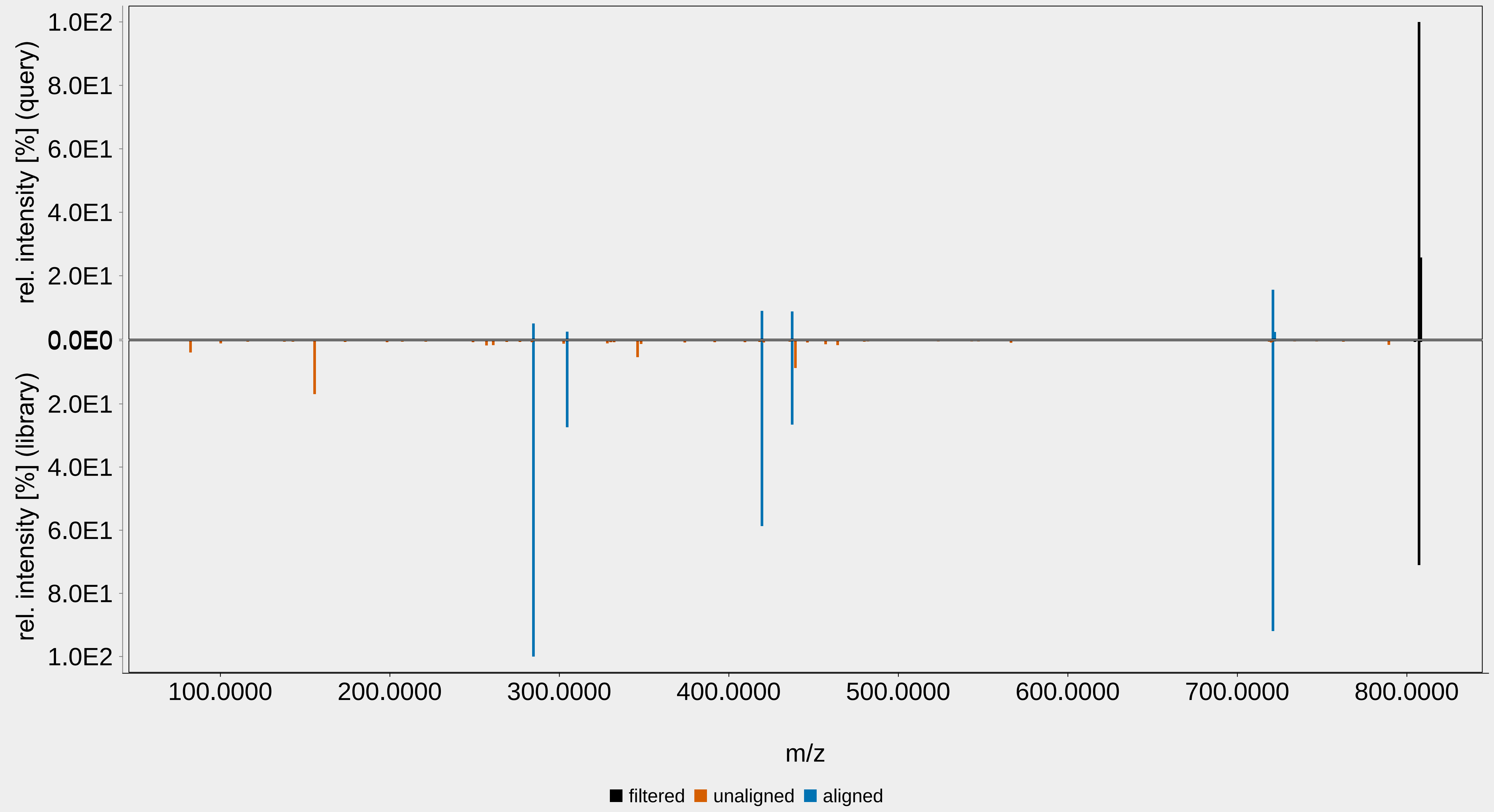

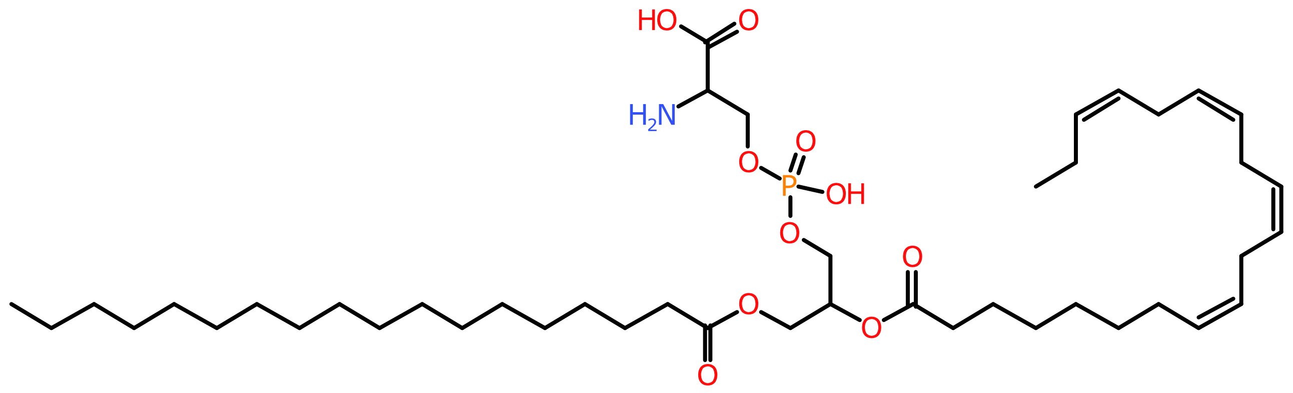

Compound information

Name: PS 38:4

Formula: C44H78NO10P

Mol. weight: 812.0809999999999

Exact mass: 812.0809999999999

Ion type: [M-H]-

Precursor m/z: 810.5303

RT: 8.778674

Polarity: negative

InChI:

InChI key:

SMILES:

Instrument information

Instrument type: LC-ESI-QTOF

Instrument: AB Sciex TripleTOF 5600+ system

Ms level: MS2

Collision energy: 45V

Database links

Pubchem: 138181120

MoNA ID: LQB00336

Other information

Principal investigator: Kazutaka Ikeda, Hiroshi

Data collector: Kazutaka Ikeda, Hiroshi

Spectral Match to cis-4,10,13,16-Docosatetraenoic acid from NIST14

0.707

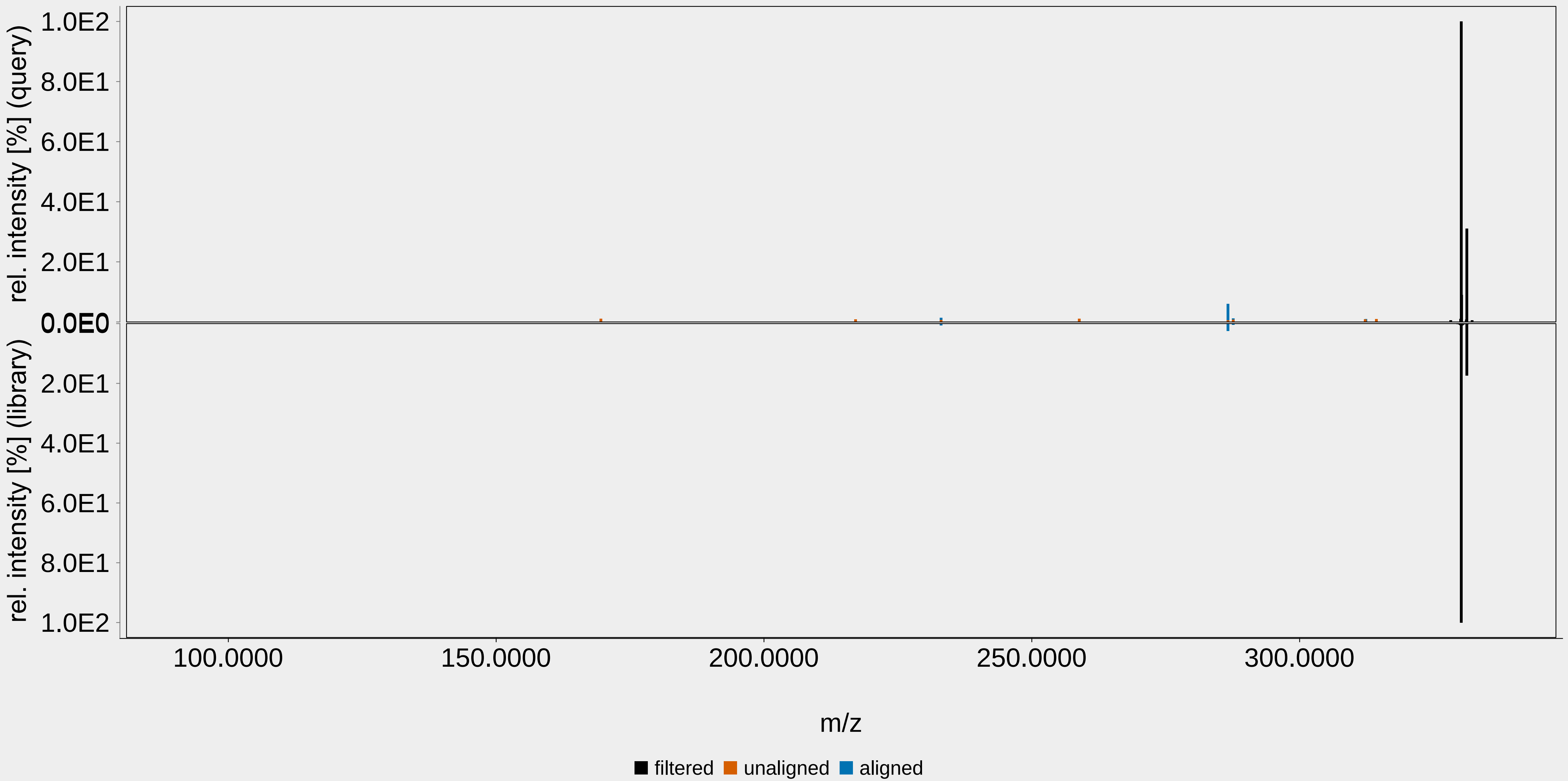

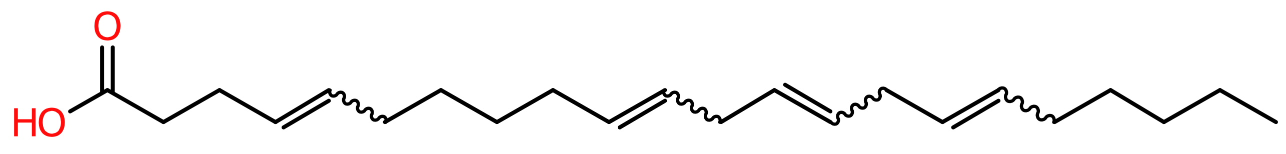

Compound information

Name: Spectral Match to

Exact mass: 332.272

Ion type: M-H

Precursor m/z: 331.263

Charge: 1

Polarity: Negative

InChI:

SMILES:

Instrument information

Instrument type: Ion Trap

Ion source: ESI

Ms level: 2

Acquisition: Isolated

Database links

CAS: 122068080

Other information

Principal investigator: Data from Charles

Data collector: Data deposited by alchemistmatt

Entry ID: CCMSLIB00003138332

PS(18:0_18:1) - 2-amino-3-({hydroxy[2-[octadec-9-enoyloxy]-3-(octadecanoyloxy)propoxy]phosphoryl}oxy)propanoic acid 0.896

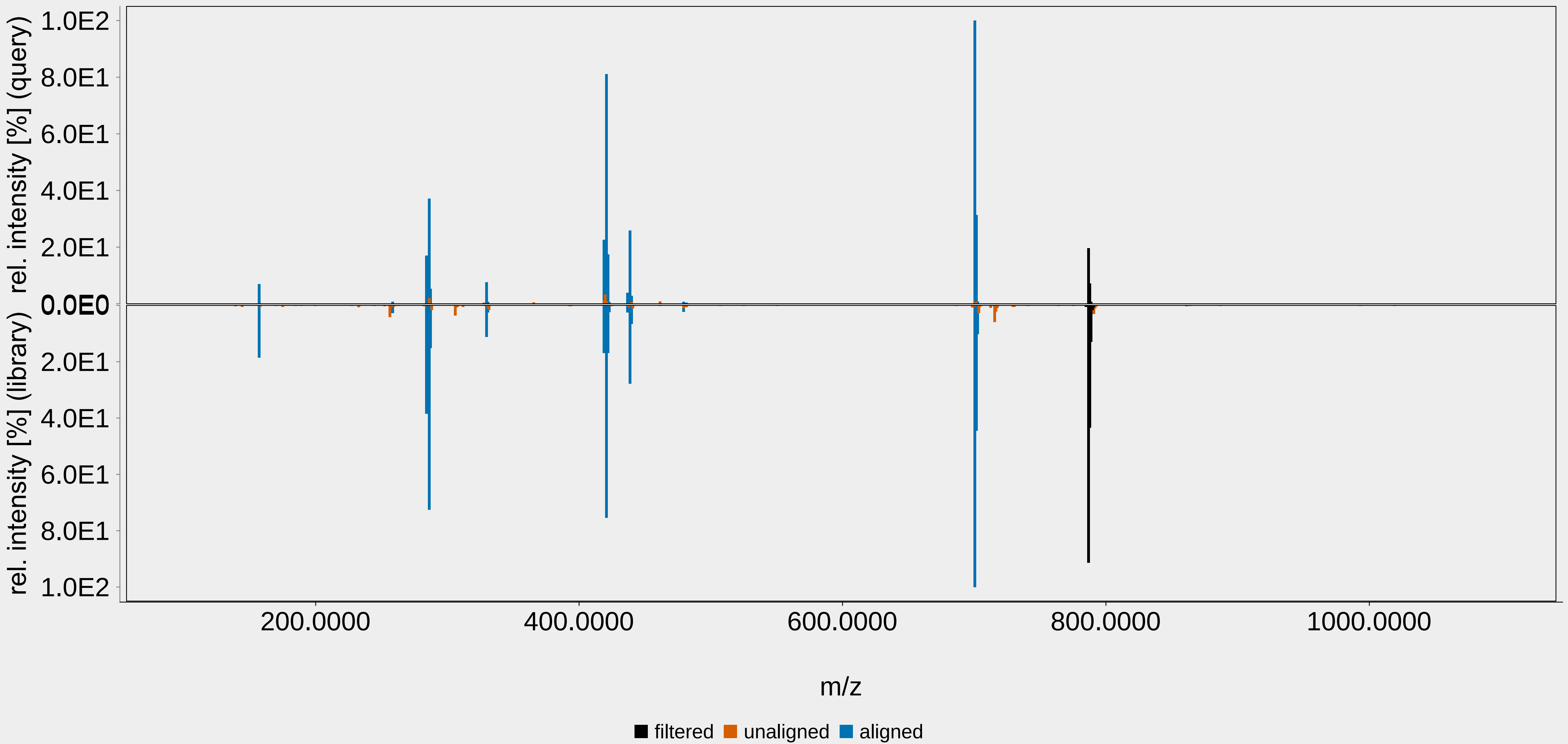

Compound information

Name: PS(18:0_18:1) -

Exact mass: 789.552

Ion type: M-H

Precursor m/z: 788.542

Charge: 1

Polarity: Negative

SMILES:

Instrument information

Instrument type: qTof

Ion source: LC-ESI

Ms level: 2

Acquisition: Lysate

Database links

CAS: 124262-93-7

Other information

Principal investigator: O Laprevote

Data collector: C-TAC

Entry ID: CCMSLIB00005720987

2-amino-3-({hydroxy[2-[octadec-9-enoyloxy]-3-(octadecanoyloxy)propoxy]phosphoryl}oxy)propanoic acid

0.896

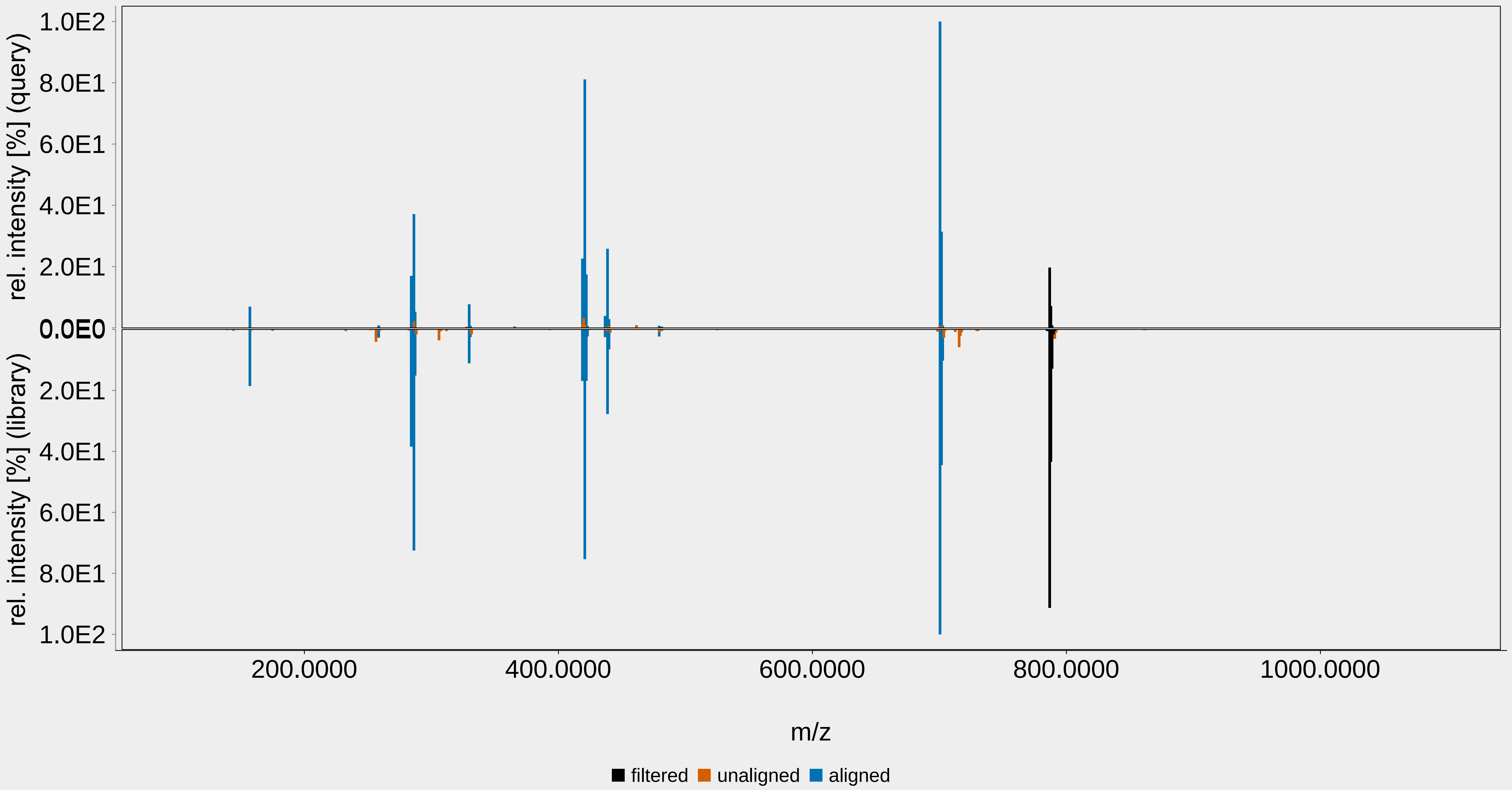

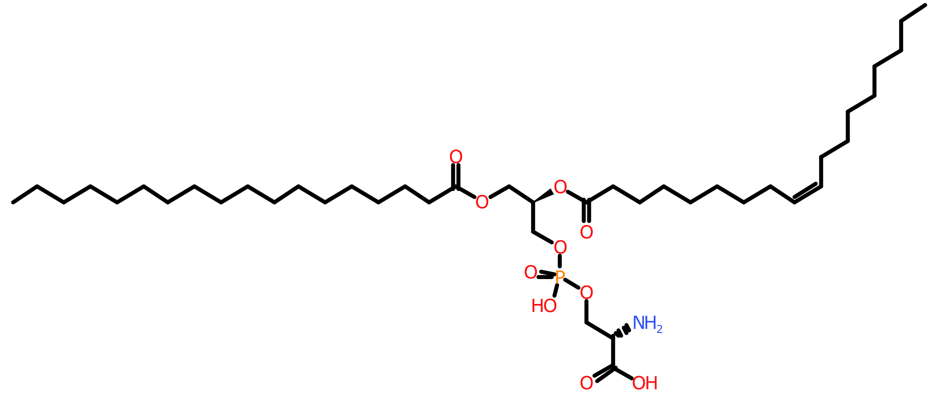

Compound information

Name:

Formula: C42H80NO10P

Mol. weight: 789.552

Exact mass: 789.552

Ion type: [M-H]-

Precursor m/z: 788.542

Polarity: negative

InChI:

InChI key:

SMILES:

Instrument information

Instrument: qTof

Ms level: MS2

Database links

Other information

Principal investigator: O Laprevote,

Data collector: O Laprevote, mpanitchpakdi,

Massbank:LQB00564 PE 36:4

0.947

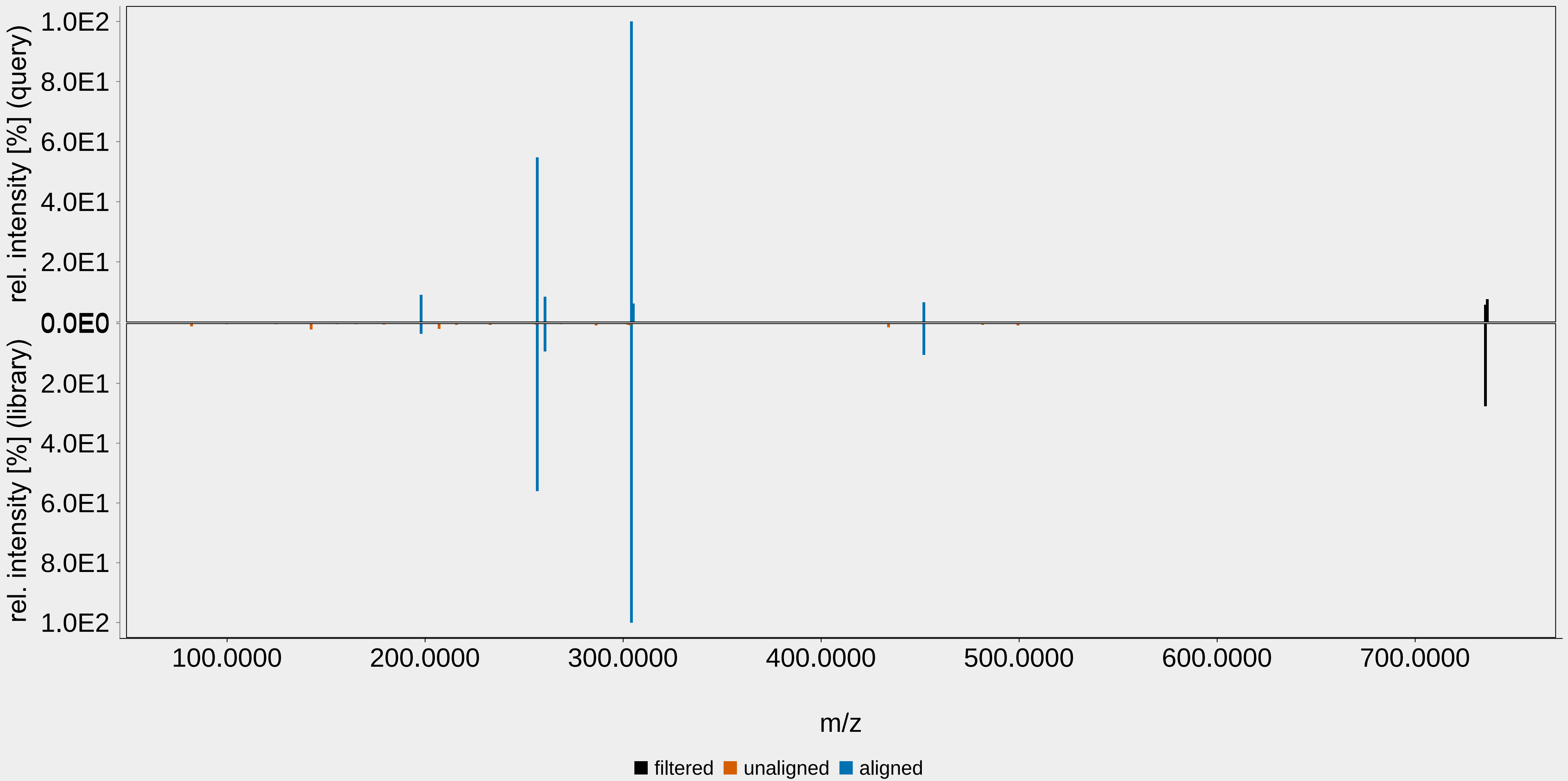

Compound information

Name: Massbank:LQB00564 PE 36:4

Exact mass: 0.0

Ion type: M-H

Precursor m/z: 738.503

Charge: 1

Polarity: Negative

InChI:

SMILES:

Instrument information

Instrument type: qTof

Ion source: ESI

Ms level: 2

Acquisition: Isolated

Database links

Other information

Principal investigator: Massbank

Data collector: Massbank

Entry ID: CCMSLIB00005737665

Massbank:LQB00178 PE 36:4

0.947

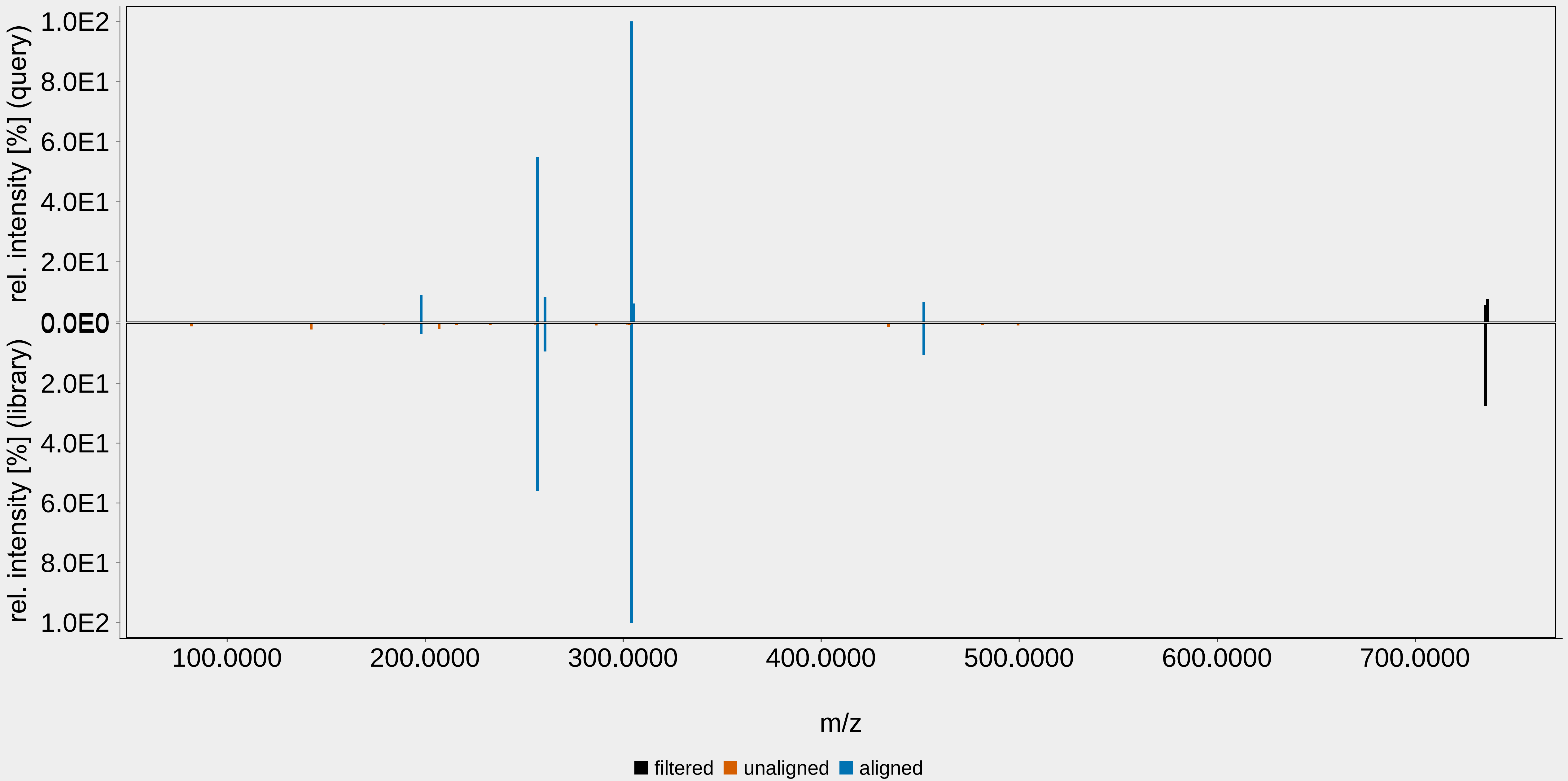

Compound information

Name: Massbank:LQB00178 PE 36:4

Exact mass: 0.0

Ion type: M-H

Precursor m/z: 738.503

Charge: 1

Polarity: Negative

InChI:

SMILES:

Instrument information

Instrument type: qTof

Ion source: ESI

Ms level: 2

Acquisition: Isolated

Database links

Other information

Principal investigator: Massbank

Data collector: Massbank

Entry ID: CCMSLIB00005737734

PE(16:0/18:1); [M-H]- C39H75N1O8P1 **0.930**

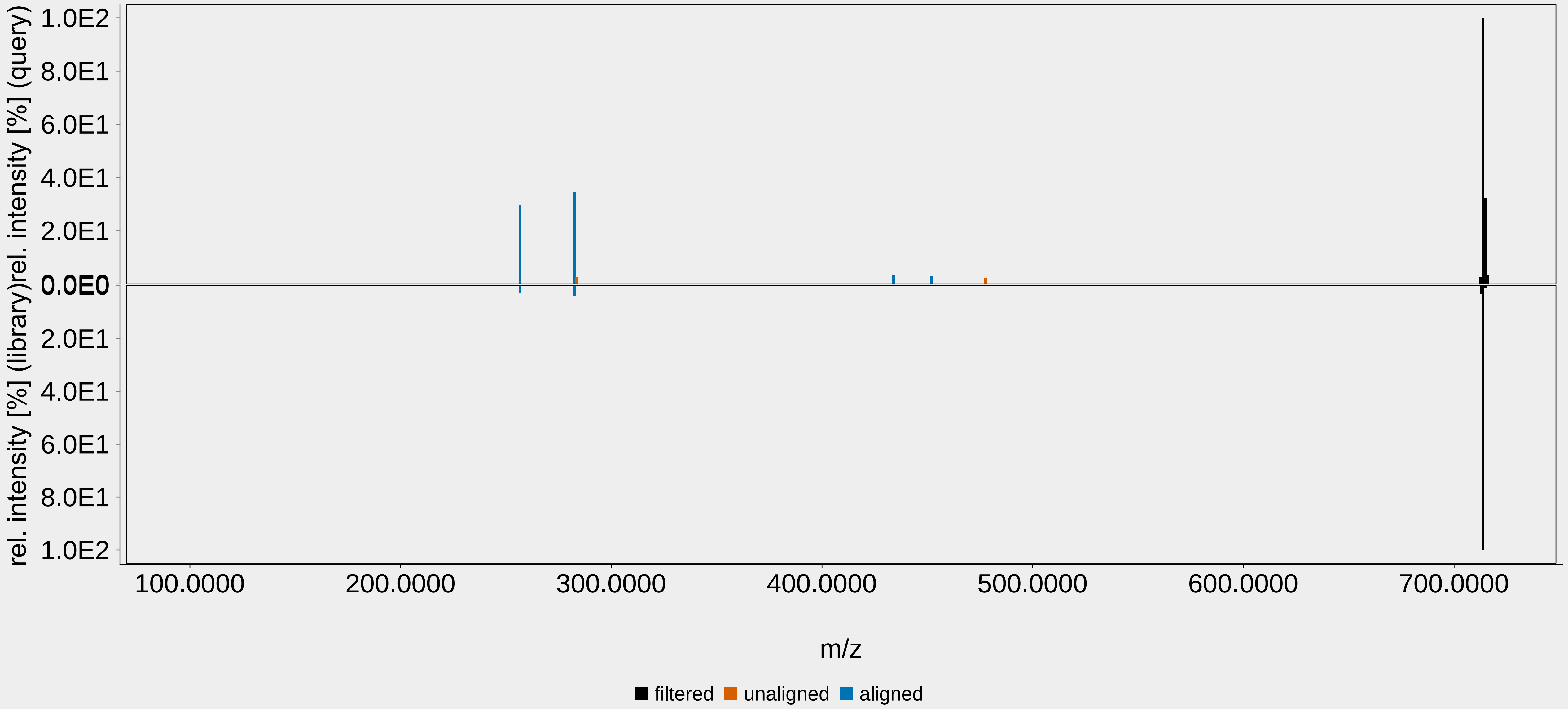

Compound information

Name: PE(16:0/18:1); [M-H]- C39H75N1O8P1

Exact mass: 0.0

Ion type: M-H

Precursor m/z: 716.523

Charge: 1

Polarity: Negative

Instrument information

Instrument type: HCD; Velos

Ion source: LC-ESI

Ms level: 2

Acquisition: Commercial

Database links

Other information

Principal investigator: Thomas Metz

Data collector: Thomas Metz

Entry ID: CCMSLIB00010072728

PE(16:0_18:1) - (2-aminoethoxy)[3-(hexadecanoyloxy)-2-[octadec-9-enoyloxy]propoxy]phosphinic acid

0.942

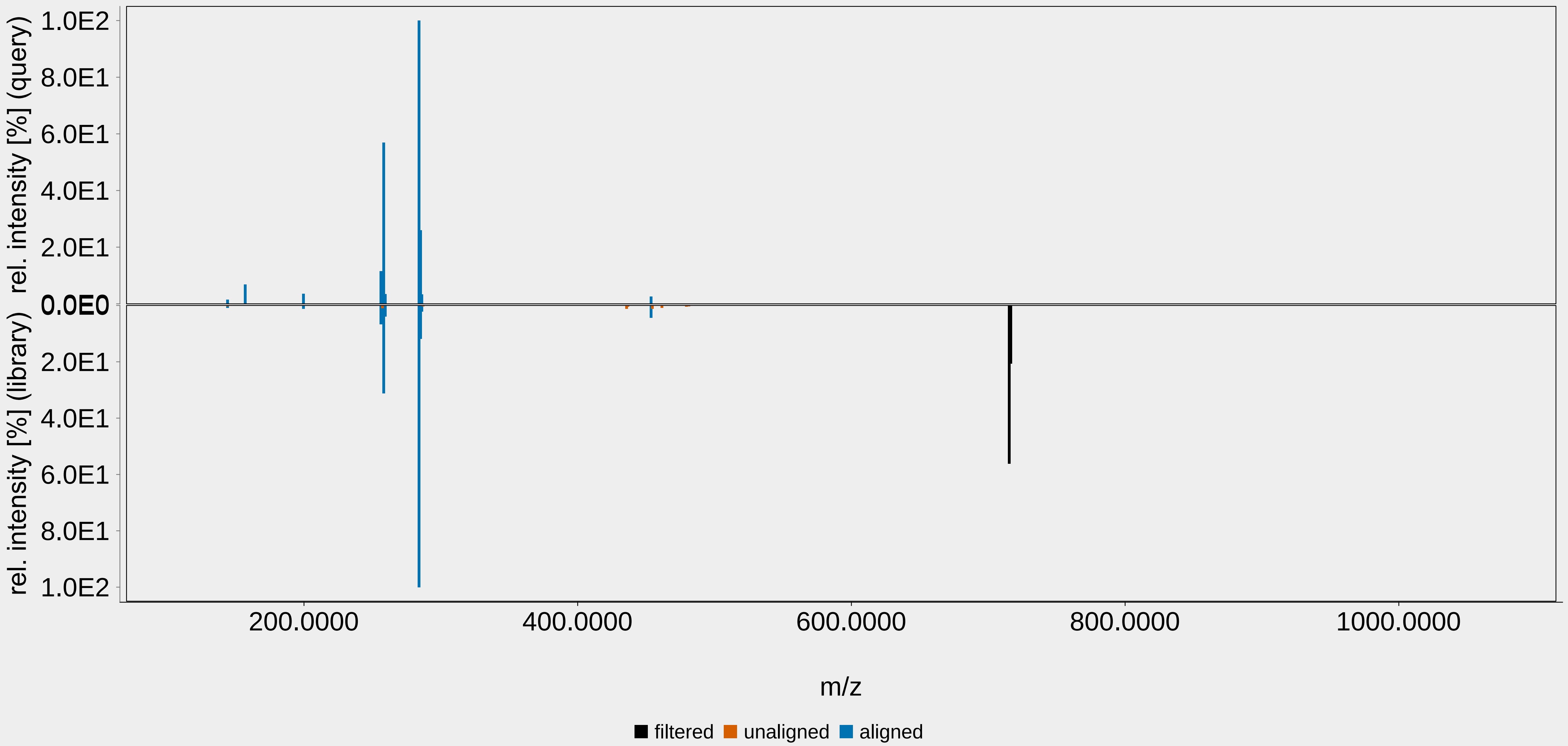

Compound information

Name: PE(16:0_18:1) -

Exact mass: 717.531

Ion type: M-H

Precursor m/z: 716.522

Charge: 1

Polarity: Negative

SMILES:

Instrument information

Instrument type: qTof

Ion source: LC-ESI

Ms level: 2

Acquisition: Lysate

Database links

CAS: 26662-94-2

Other information

Principal investigator: N Auzeil

Data collector: UMR CNRS 8038 Universite de

Entry ID: CCMSLIB00005724205

PA 22:0_18:1

0.786

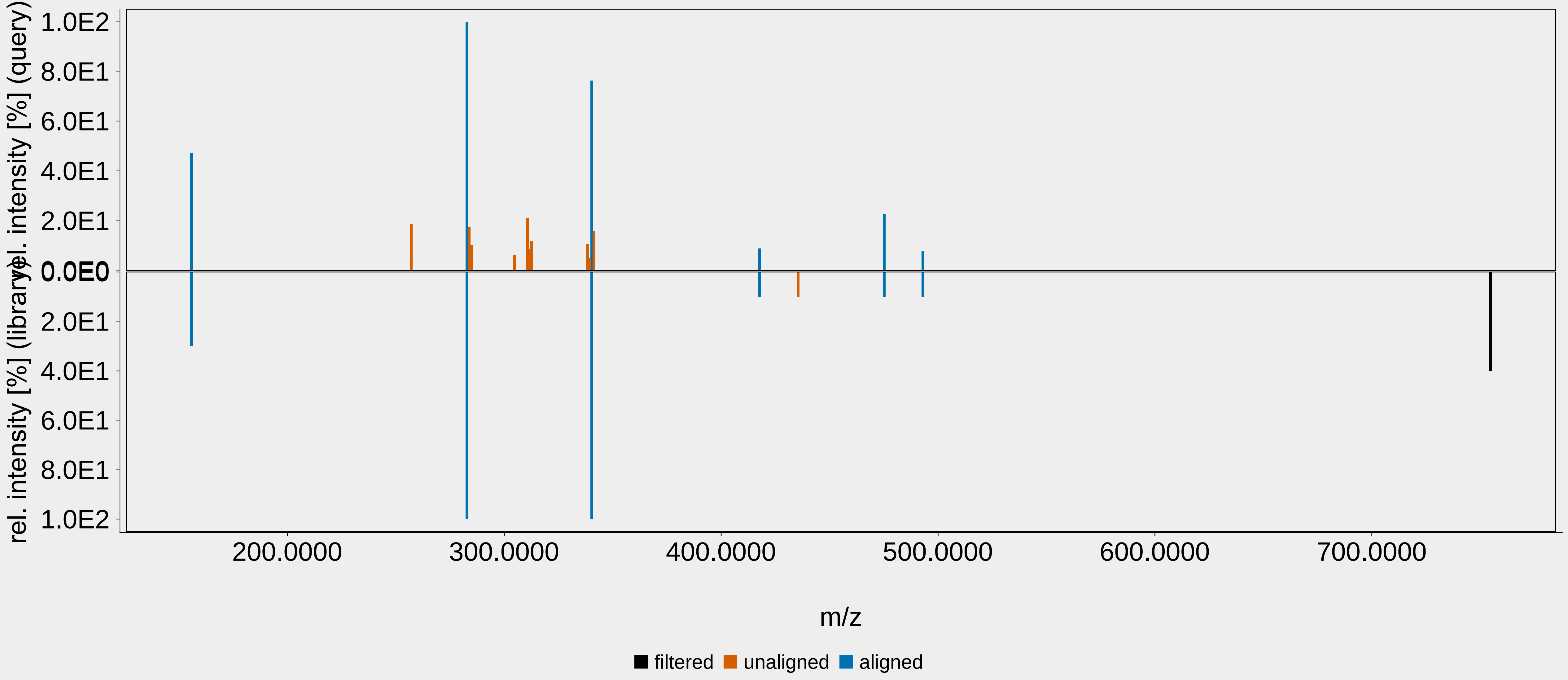

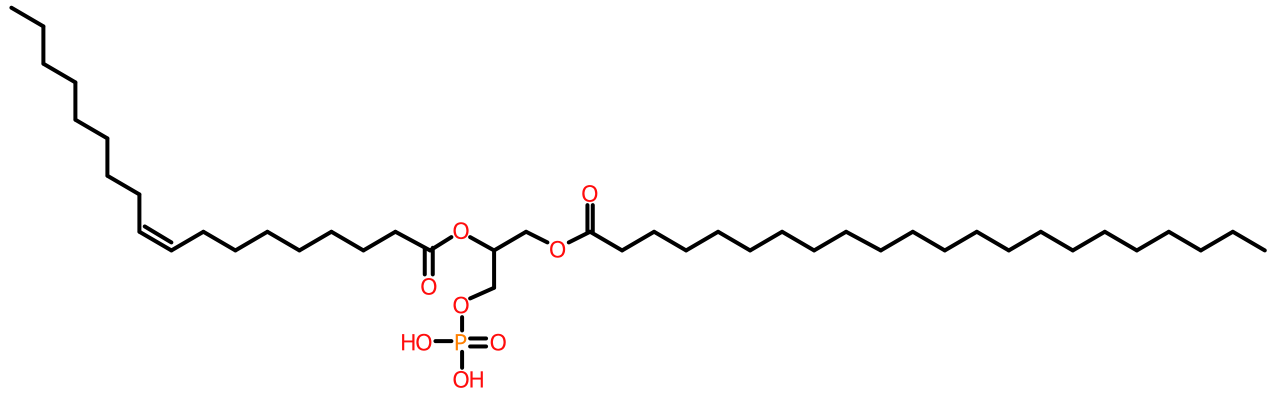

Compound information

Name: PA 22:0_18:1

Formula: C43H83O8P

Ion type: [M-H]-

Precursor m/z: 757.57528

Ccs: 273.22

InChI key:

SMILES:

Num peaks: 8

Instrument information

Database links

Other information

PS 22:4_22:6 **0.706**

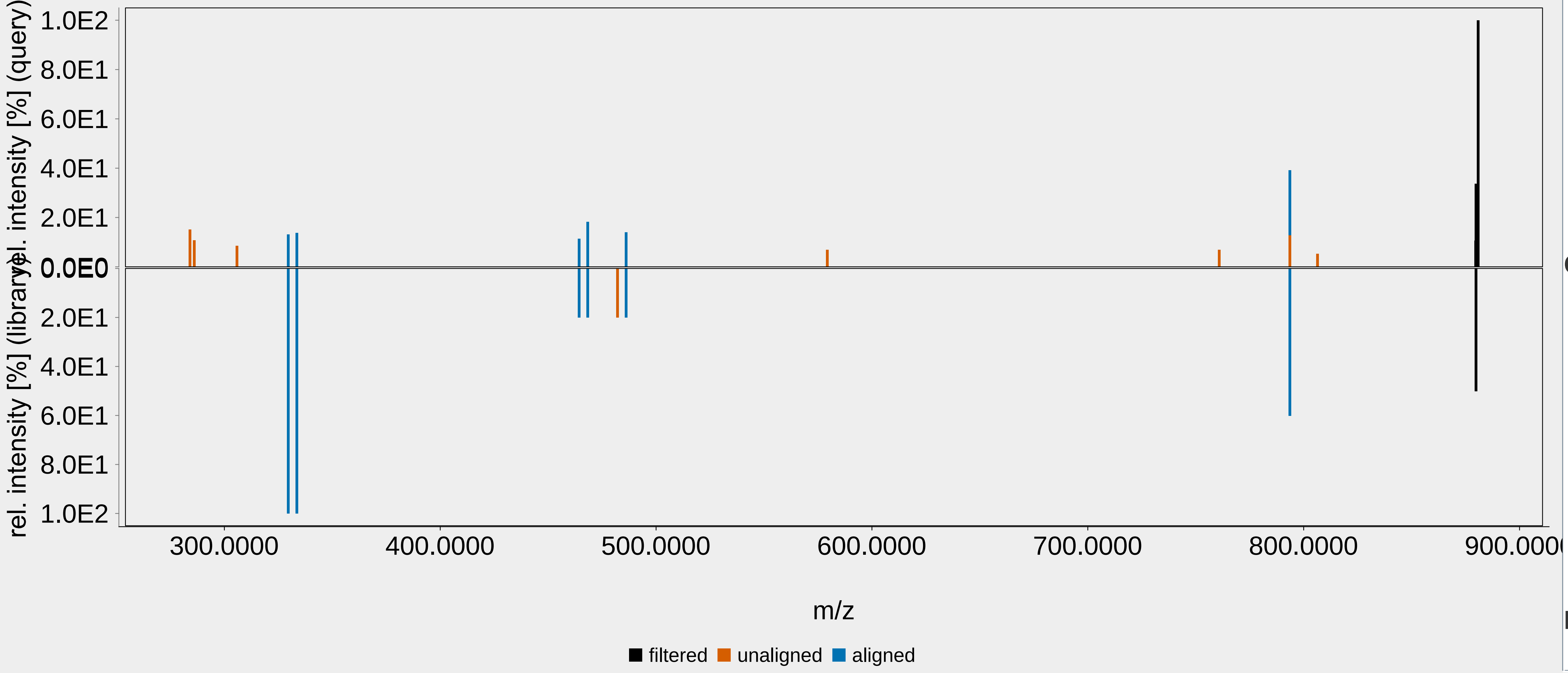

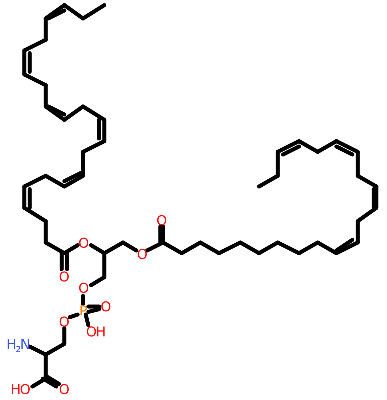

Compound information

Name: PS 22:4_22:6

Formula: C50H78NO10P

Ion type: [M-H]-

Precursor m/z: 882.52906

Ccs: 284.42

InChI key:

SMILES:

Num peaks: 8

Instrument information

Database links

Other information

PI 38:4 **0.925**

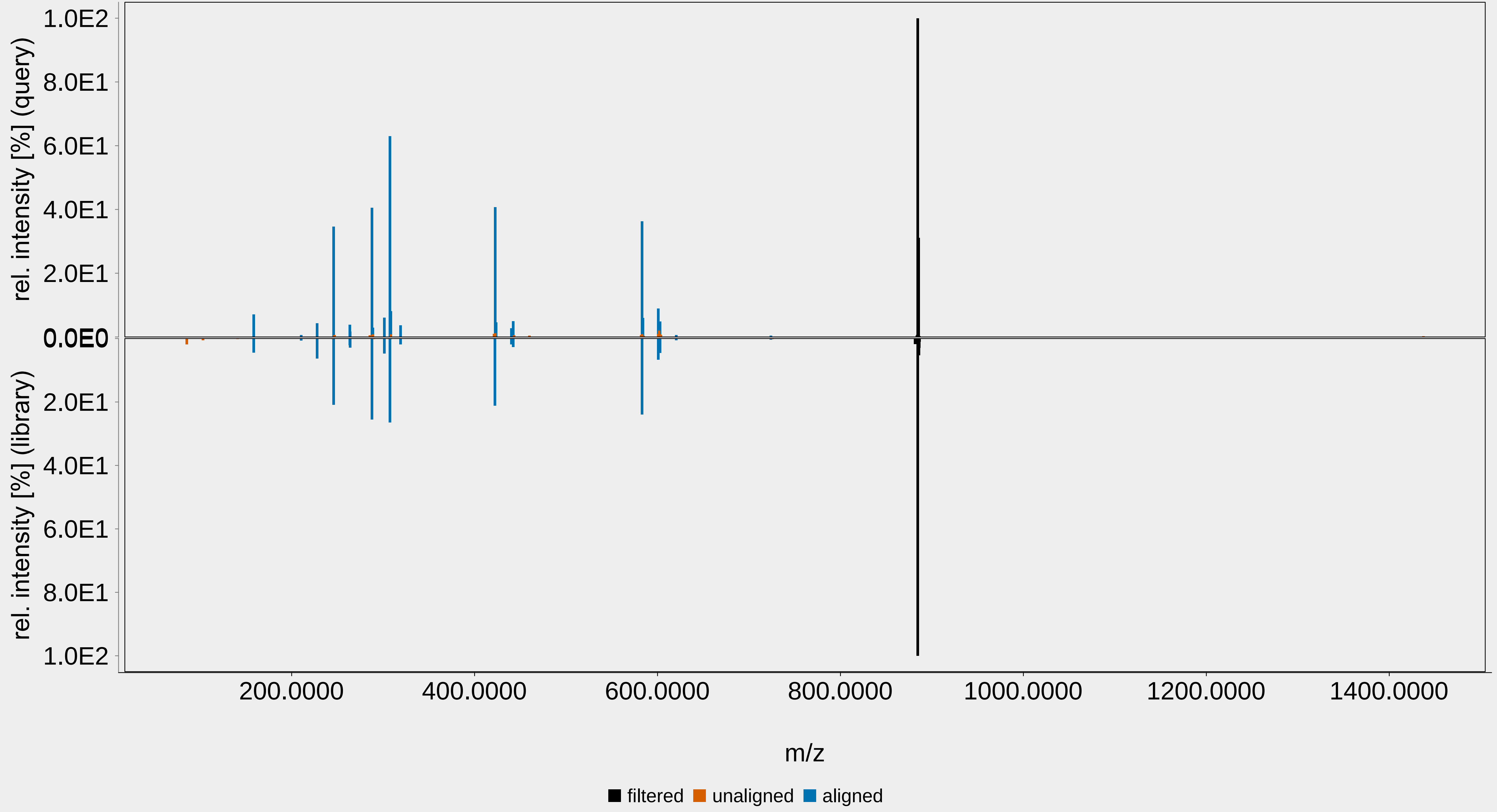

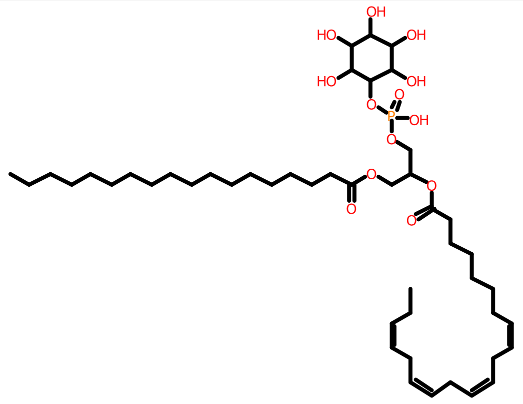

Compound information

Name: PI 38:4

Formula: C47H83O13P

Mol. weight: 887.1439999999999

Exact mass: 887.1439999999999

Ion type: [M-H]-

Precursor m/z: 885.5508

RT: 8.754183

Polarity: negative

InChI:

InChI key:

SMILES:

Instrument information

Instrument type: LC-ESI-QTOF

Instrument: AB Sciex TripleTOF 5600+ system

Ms level: MS2

Collision energy: 45V

Database links

Pubchem: 138227586

MoNA ID: LQB00694

Other information

Principal investigator: Kazutaka Ikeda, Hiroshi

Data collector: Kazutaka Ikeda, Hiroshi

PI 38:4

0.925

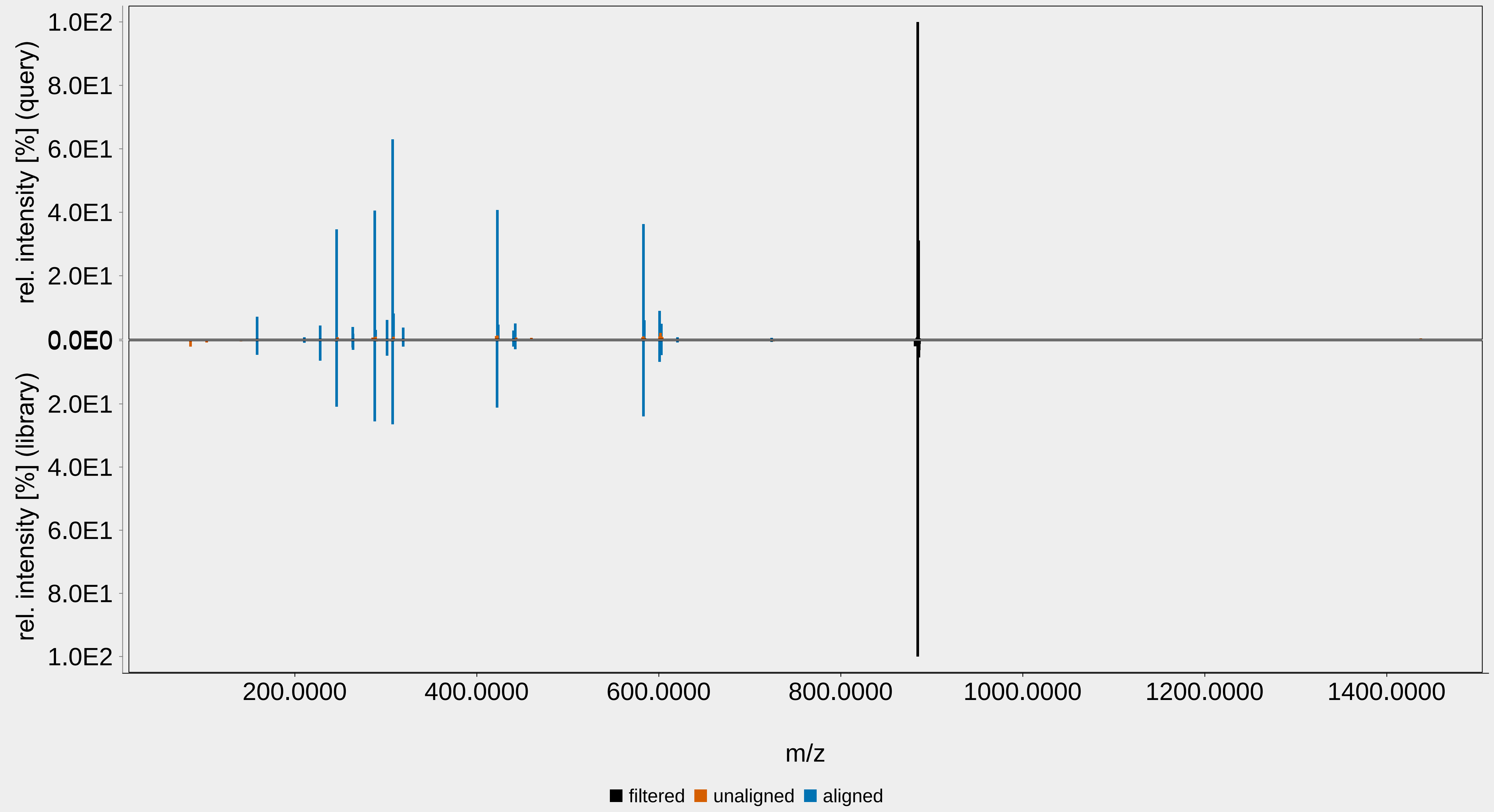

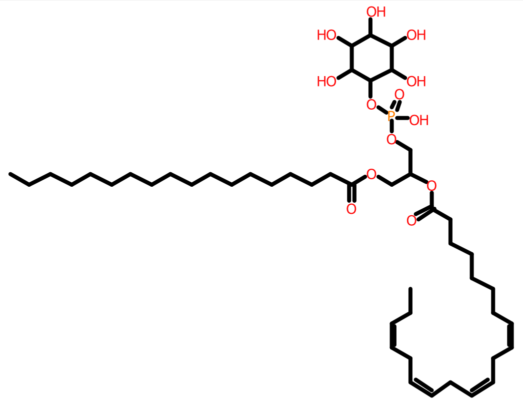

Compound information

Name: PI 38:4

Formula: C47H83O13P

Mol. weight: 887.1439999999999

Exact mass: 887.1439999999999

Ion type: [M-H]-

Precursor m/z: 885.5508

RT: 8.754183

Polarity: negative

InChI:

InChI key:

SMILES:

Instrument information

Instrument type: LC-ESI-QTOF

Instrument: AB Sciex TripleTOF 5600+ system

Ms level: MS2

Collision energy: 45V

Database links

Pubchem: 138227586

MoNA ID: LQB00308

Other information

Principal investigator: Kazutaka Ikeda, Hiroshi

Data collector: Kazutaka Ikeda, Hiroshi

Uridine 5'-diphospho-N-acetylglucosamine

0.785

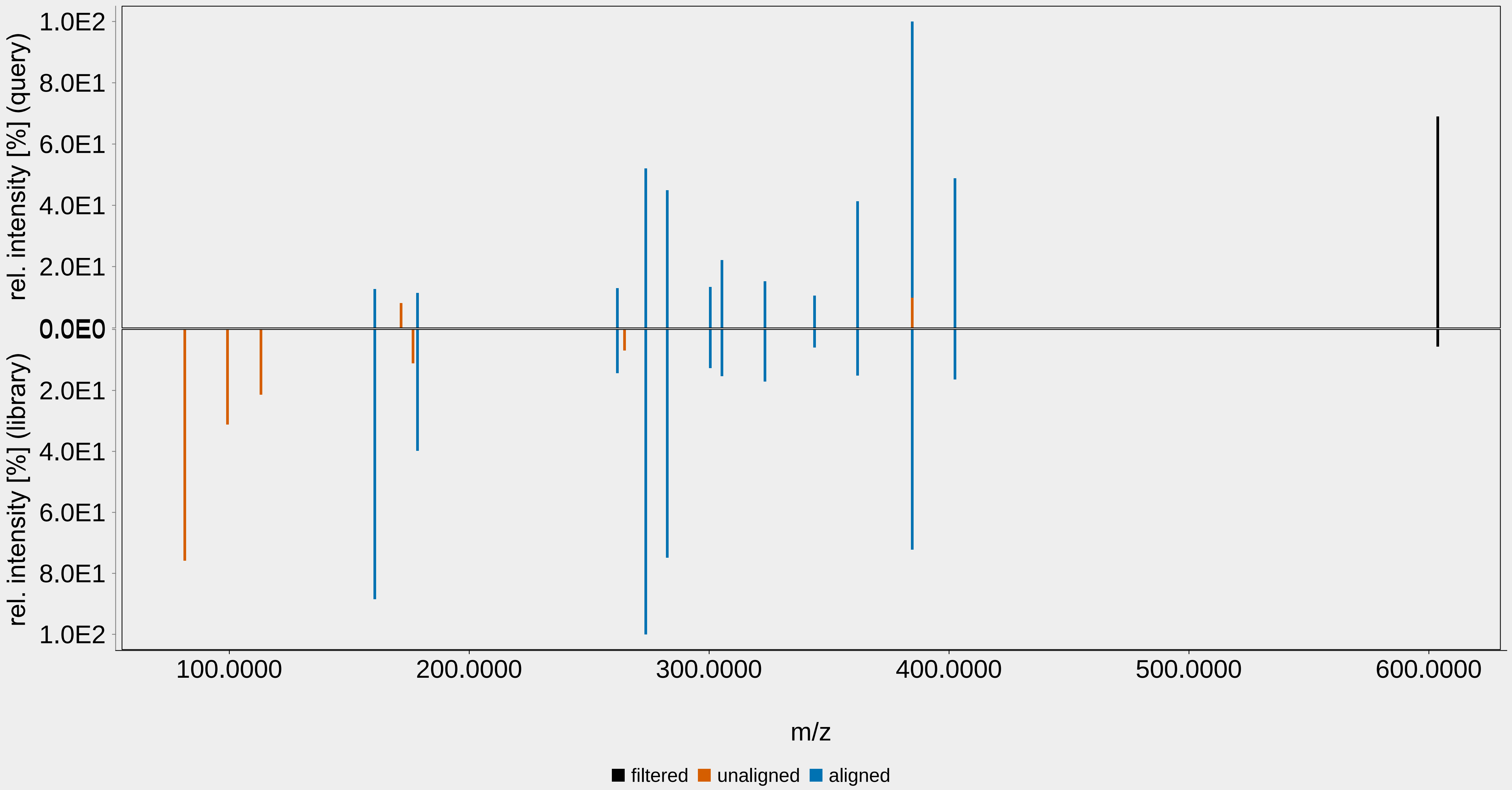

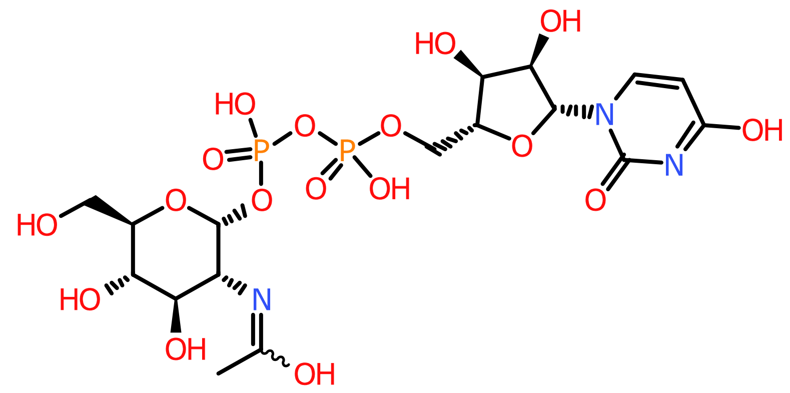

Compound information

Name: Uridine

Formula: C17H27N3O17P2

Exact mass: 607.08157

Ion type: [M-H]-

Precursor m/z: 606.07377

Polarity: NEGATIVE

InChI:

InChI key:

SMILES:

Num peaks: 18

Instrument information

Instrument type: LC-ESI-QTOF

Instrument: UPLC Q-Tof Premier, Waters

Ms level: MS2

Collision energy: 30 V

Database links

Other information

Entry ID: MSBNK-RIKEN-PR100621

Comment: Parent=606.07377

[(2R,3R,4R,5S,6R)-3-acetamido-4,5-dihydroxy-6-(hydroxymethyl)oxan-2-yl] [[(2R,3S,4R,5R)-5-(2,4-dioxopyrimidin-1-yl)-3,4-dihydroxyoxolan-2-yl]methoxy-hydroxyphosphoryl]

0.785

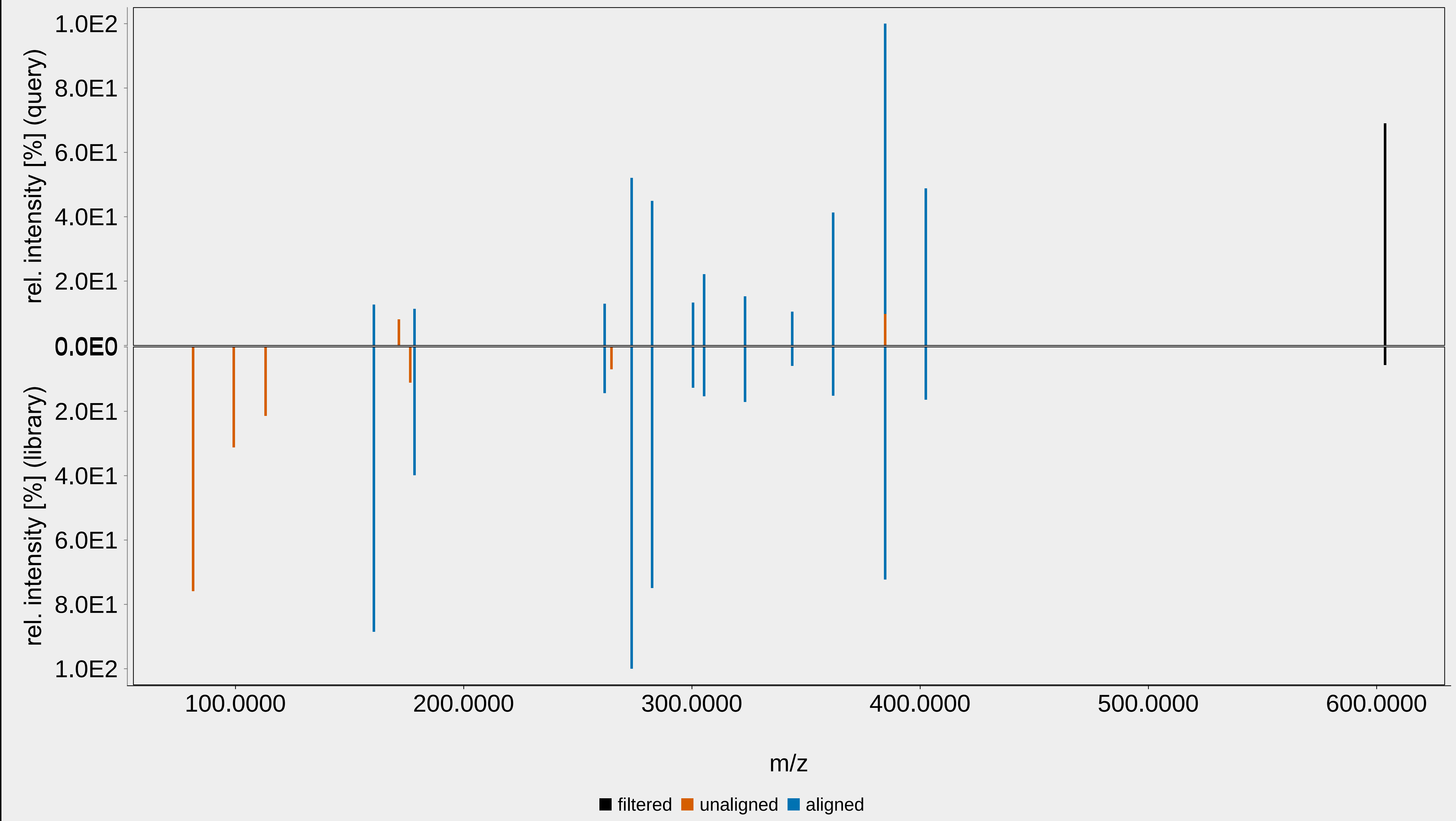

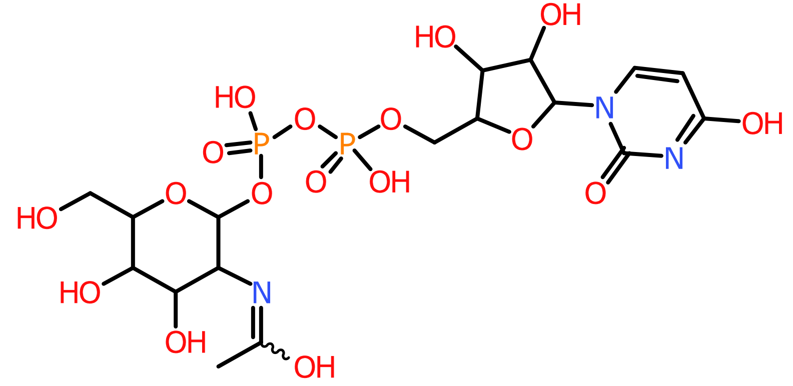

Compound information

Name:

Formula: C17H27N3O17P2

Exact mass: 607.359

Ion type: [M+H]+

Precursor m/z: 606.07377

Polarity: NEGATIVE

InChI:

InChI key:

SMILES:

Num peaks: 18

Instrument information

Instrument type: LC-ESI-QTOF

Instrument: Q-Tof Premier, Waters

Ms level: MS2

Collision energy: 30 V

Database links

Other information

Entry ID: MSBNK-RIKEN_ReSpect-PT203703

Comment: Parent=606.07377

PE(18:0/0:0); [M-H]- C23H47N1O7P1 **0.746**

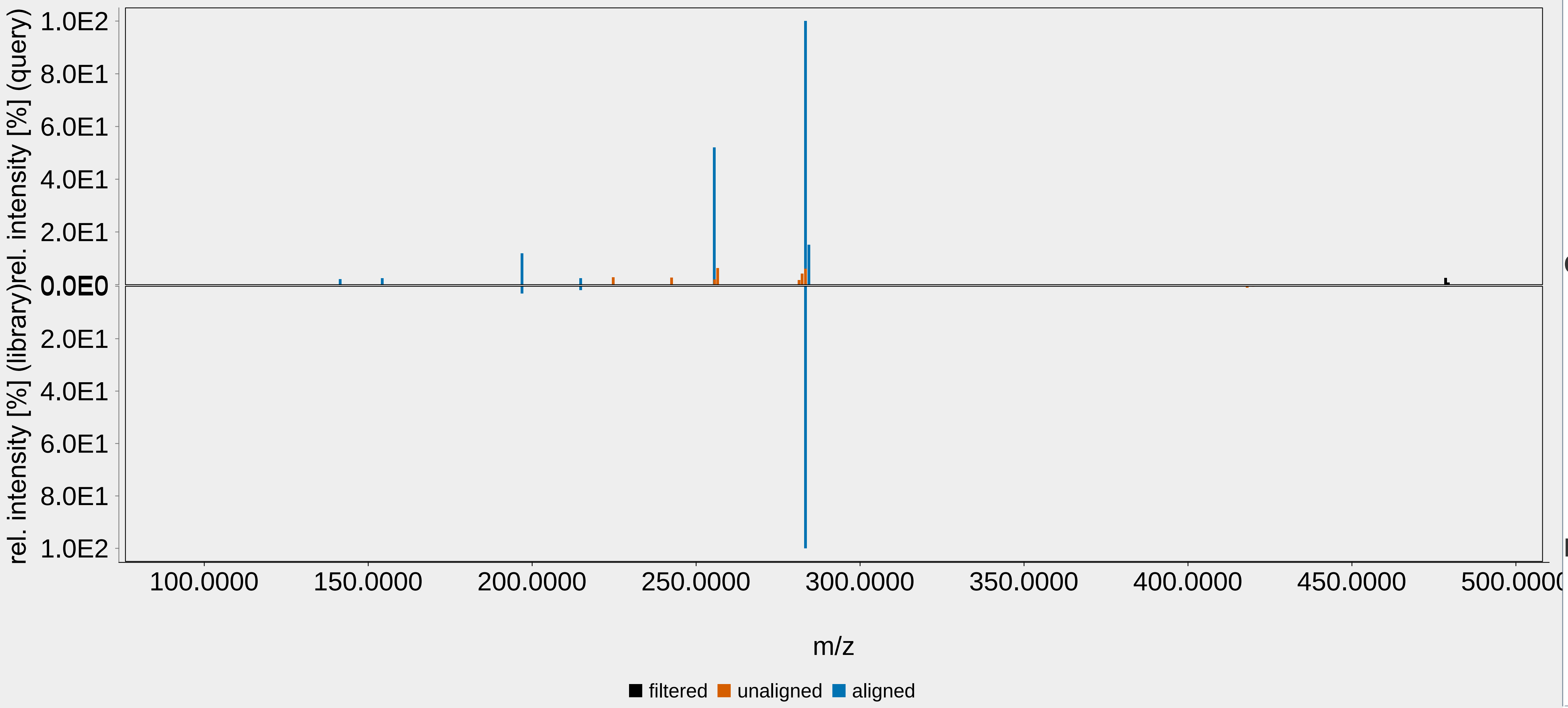

Compound information

Name: PE(18:0/0:0); [M-H]- C23H47N1O7P1

Exact mass: 0.0

Ion type: M-H

Precursor m/z: 480.309

Charge: 1

Polarity: Negative

Instrument information

Instrument type: CID; Lumos

Ion source: LC-ESI

Ms level: 2

Acquisition: Commercial

Database links

Other information

Principal investigator: Thomas Metz

Data collector: Thomas Metz

Entry ID: CCMSLIB00010059720

PE(18:0/0:0); [M-H]- C23H47N1O7P1

0.760

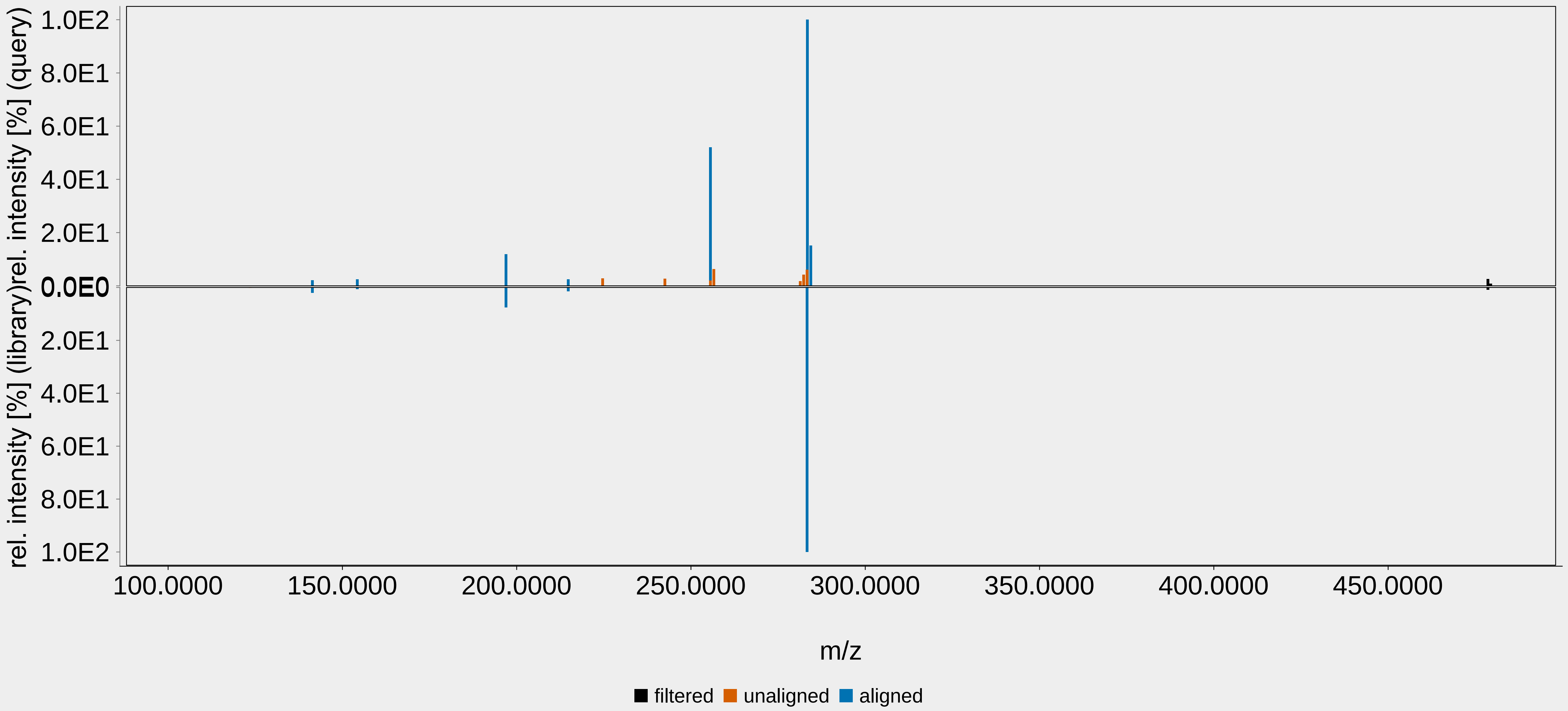

Compound information

Name: PE(18:0/0:0); [M-H]- C23H47N1O7P1

Exact mass: 0.0

Ion type: M-H

Precursor m/z: 480.309

Charge: 1

Polarity: Negative

Instrument information

Instrument type: HCD; Lumos

Ion source: LC-ESI

Ms level: 2

Acquisition: Commercial

Database links

Other information

Principal investigator: Thomas Metz

Data collector: Thomas Metz

Entry ID: CCMSLIB00010059722

FAD - 30.0 eV

0.807

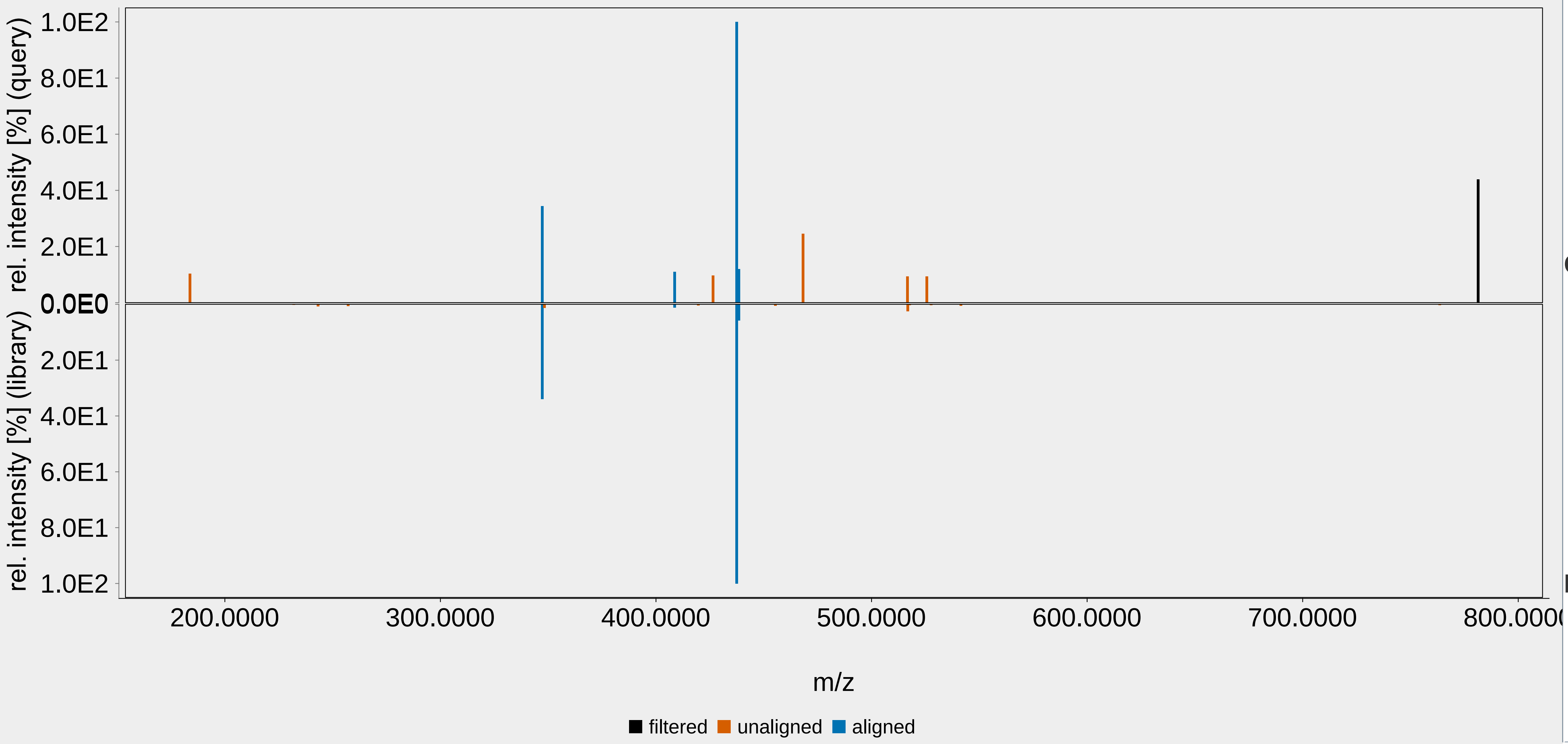

Compound information

Name: FAD - 30.0 eV

Exact mass: 0.0

Ion type: M-H

Precursor m/z: 784.15

Charge: 1

Polarity: Negative

SMILES:

Instrument information

Instrument type: Orbitrap

Ion source: ESI

Ms level: 2

Acquisition: Isolated

Database links

CAS:

Other information

Principal investigator: PI

Data collector: Data Collector

Entry ID: CCMSLIB00006113691

FAD - 30.0 eV

0.822

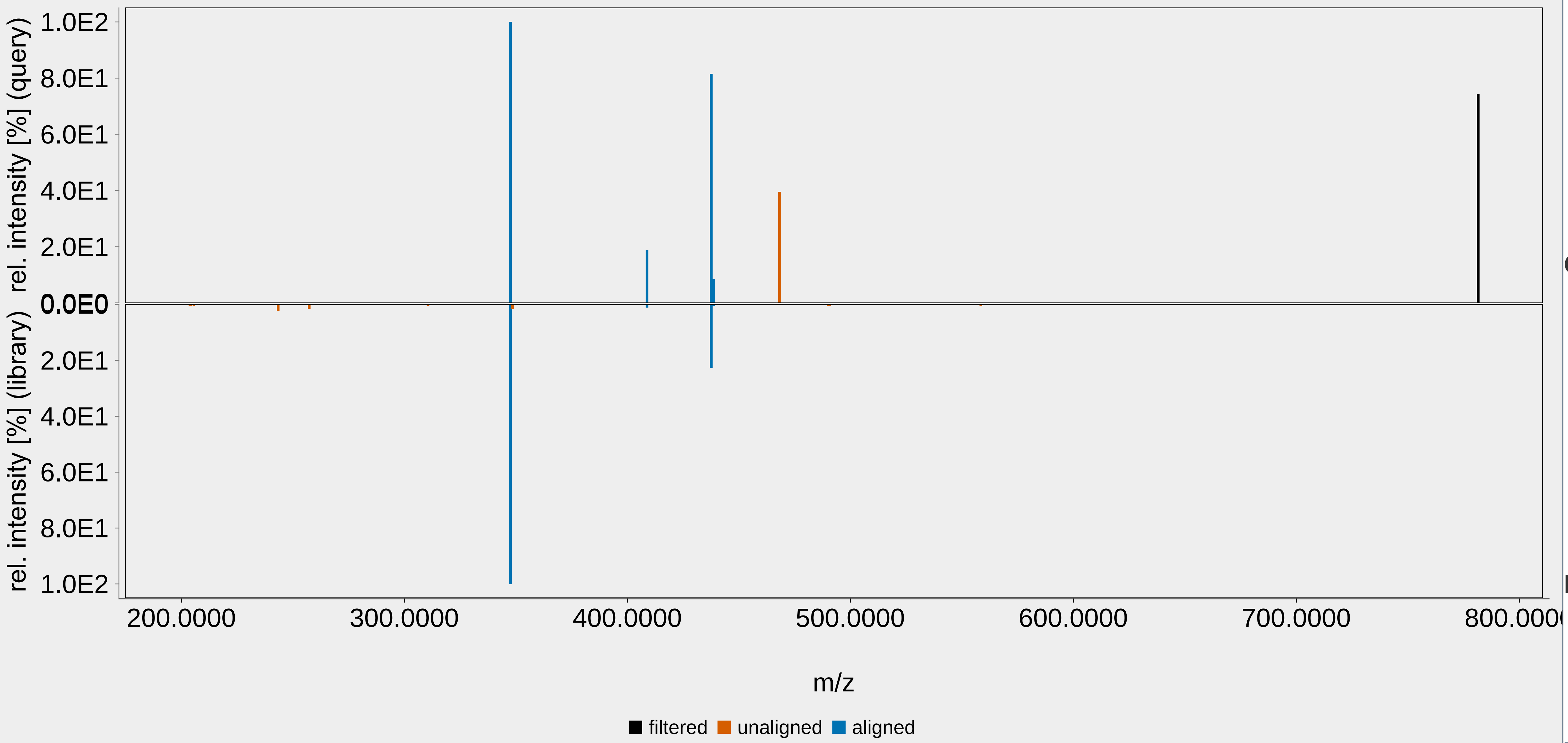

Compound information

Name: FAD - 30.0 eV

Exact mass: 0.0

Ion type: M-H

Precursor m/z: 784.149

Charge: 1

Polarity: Negative

SMILES:

Instrument information

Instrument type: Orbitrap

Ion source: ESI

Ms level: 2

Acquisition: Isolated

Database links

CAS:

Other information

Principal investigator: PI

Data collector: Data Collector

Entry ID: CCMSLIB00006113726

PA 18:1_24:1

0.817

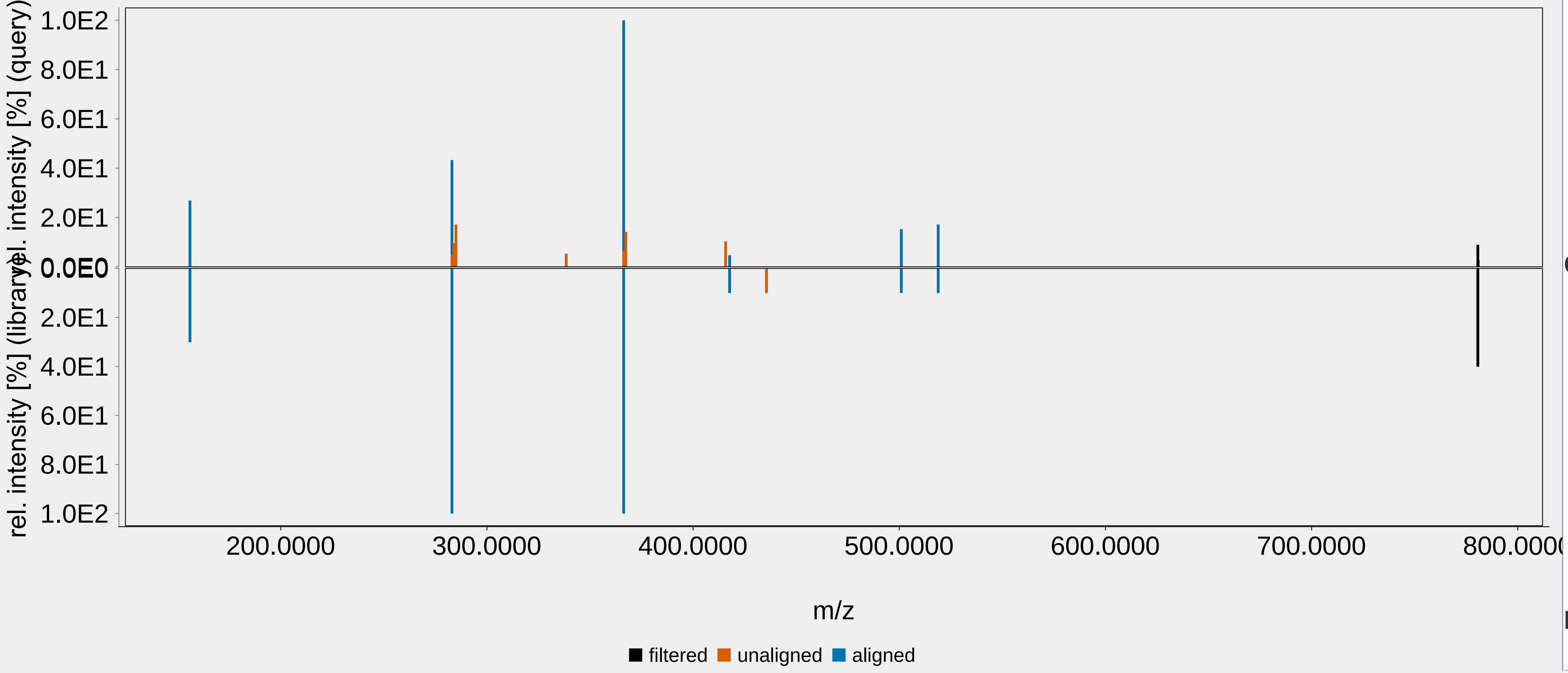

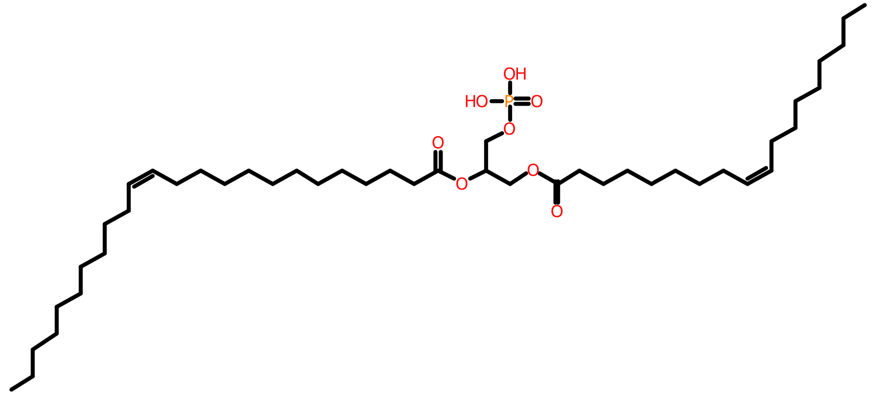

Compound information

Name: PA 18:1_24:1

Formula: C45H85O8P

Ion type: [M-H]-

Precursor m/z: 783.59093

Ccs: 279.35

InChI key:

SMILES:

Num peaks: 8

Instrument information

Database links

Other information

PE(22:6/0:0); [M-H]- C27H43N1O7P1 **0.920**

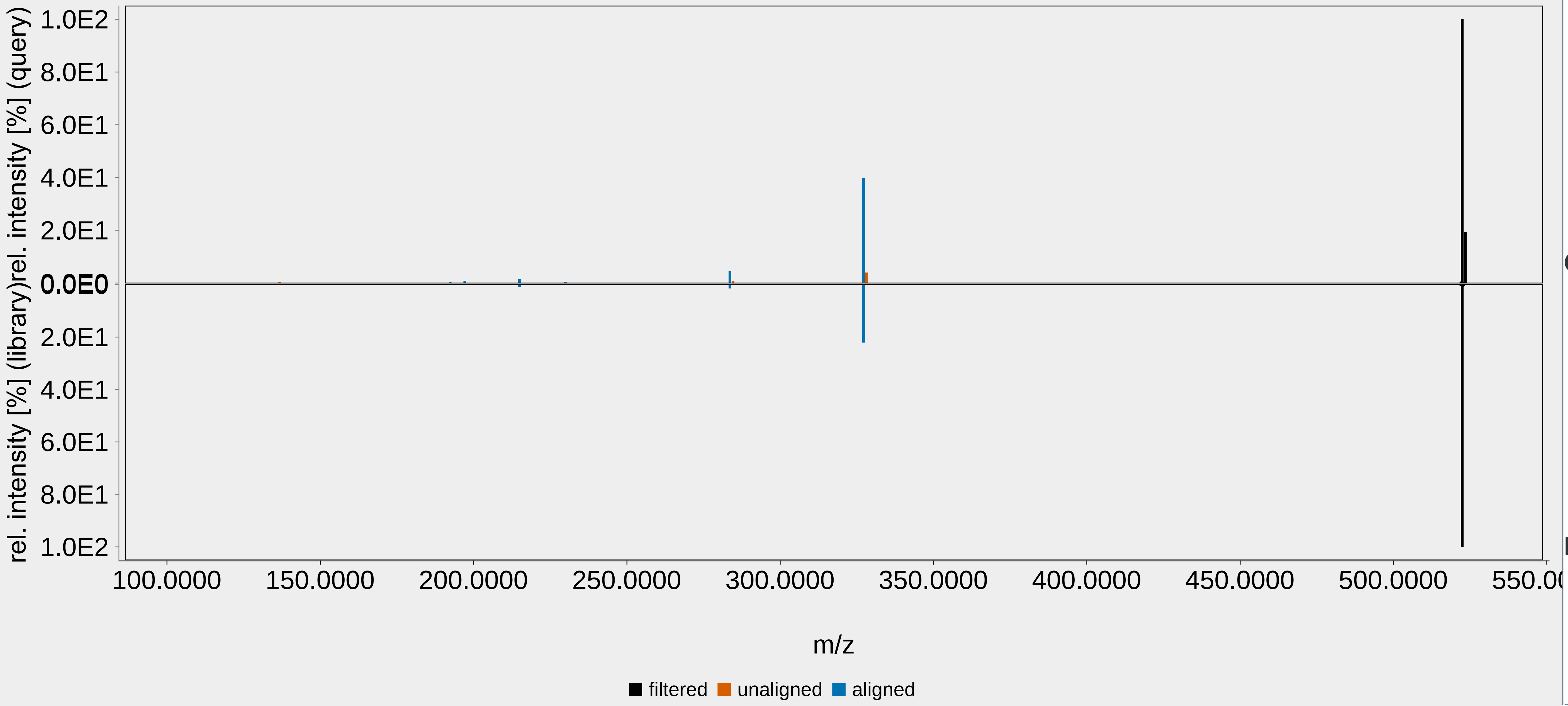

Compound information

Name: PE(22:6/0:0); [M-H]- C27H43N1O7P1

Exact mass: 0.0

Ion type: M-H

Precursor m/z: 524.278

Charge: 1

Polarity: Negative

Instrument information

Instrument type: HCD; Velos

Ion source: LC-ESI

Ms level: 2

Acquisition: Commercial

Database links

Other information

Principal investigator: Thomas Metz

Data collector: Thomas Metz

Entry ID: CCMSLIB00010073025

PE(22:6/0:0); [M-H]- C27H43N1O7P1 **0.932**

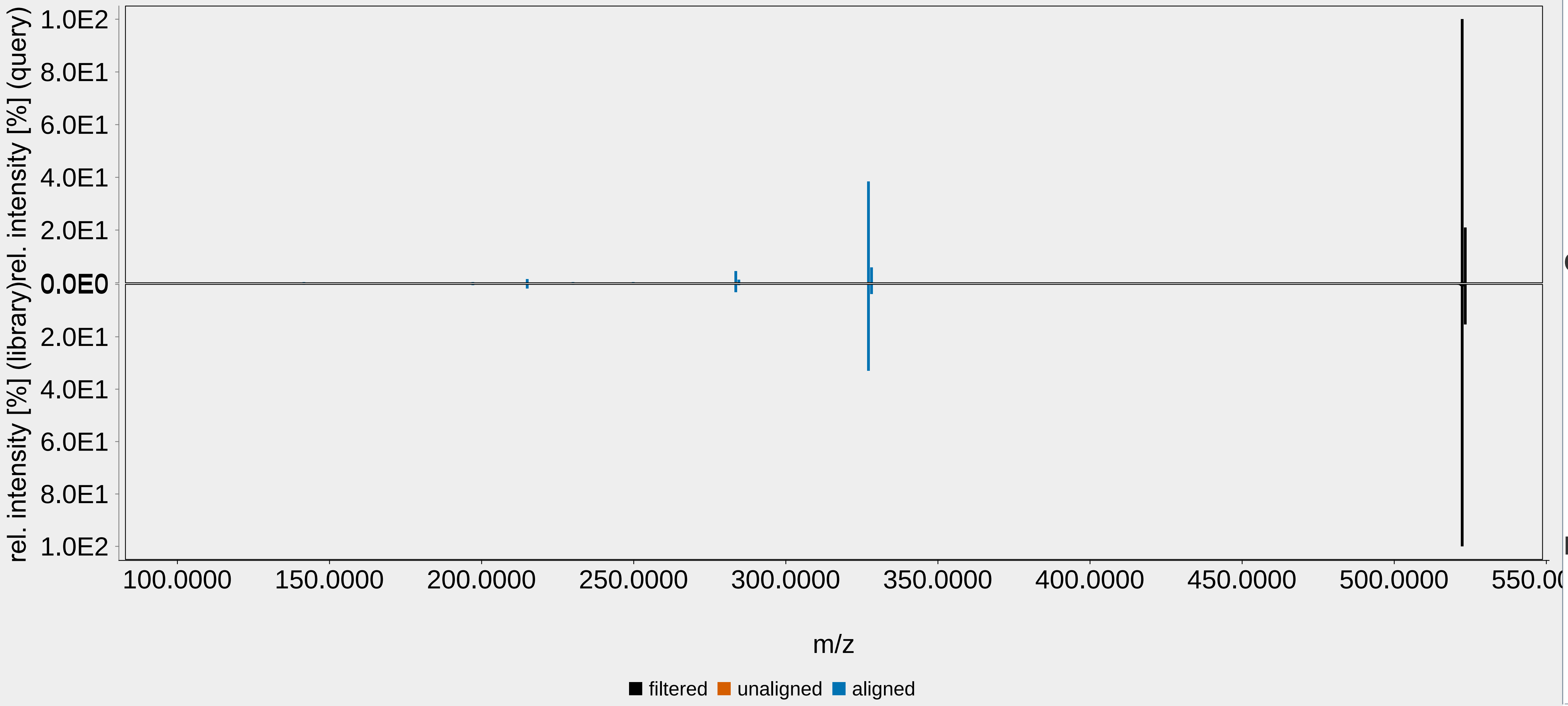

Compound information

Name: PE(22:6/0:0); [M-H]- C27H43N1O7P1

Exact mass: 0.0

Ion type: M-H

Precursor m/z: 524.278

Charge: 1

Polarity: Negative

Instrument information

Instrument type: HCD; Velos

Ion source: LC-ESI

Ms level: 2

Acquisition: Commercial

Database links

Other information

Principal investigator: Thomas Metz

Data collector: Thomas Metz

Entry ID: CCMSLIB00010086705

PE(17:0/22:6); [M-H]- C44H75N1O8P1 0.861

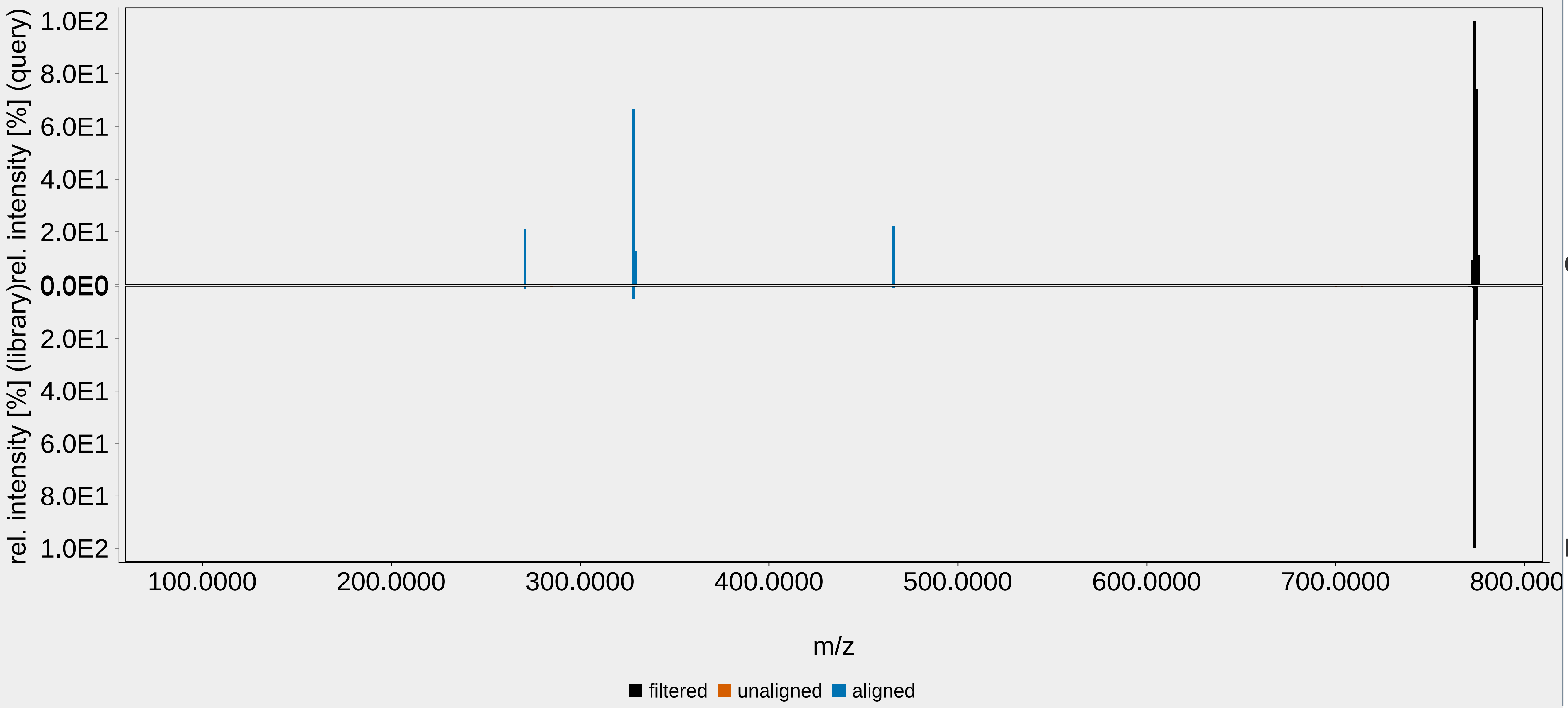

Compound information

Name: PE(17:0/22:6); [M-H]- C44H75N1O8P1

Exact mass: 0.0

Ion type: M-H

Precursor m/z: 776.523

Charge: 1

Polarity: Negative

Instrument information

Instrument type: HCD; Velos

Ion source: LC-ESI

Ms level: 2

Acquisition: Commercial

Database links

Other information

Principal investigator: Thomas Metz

Data collector: Thomas Metz

Entry ID: CCMSLIB00010086568

PE(17:0/22:6); [M-H]- C44H75N1O8P1

0.890

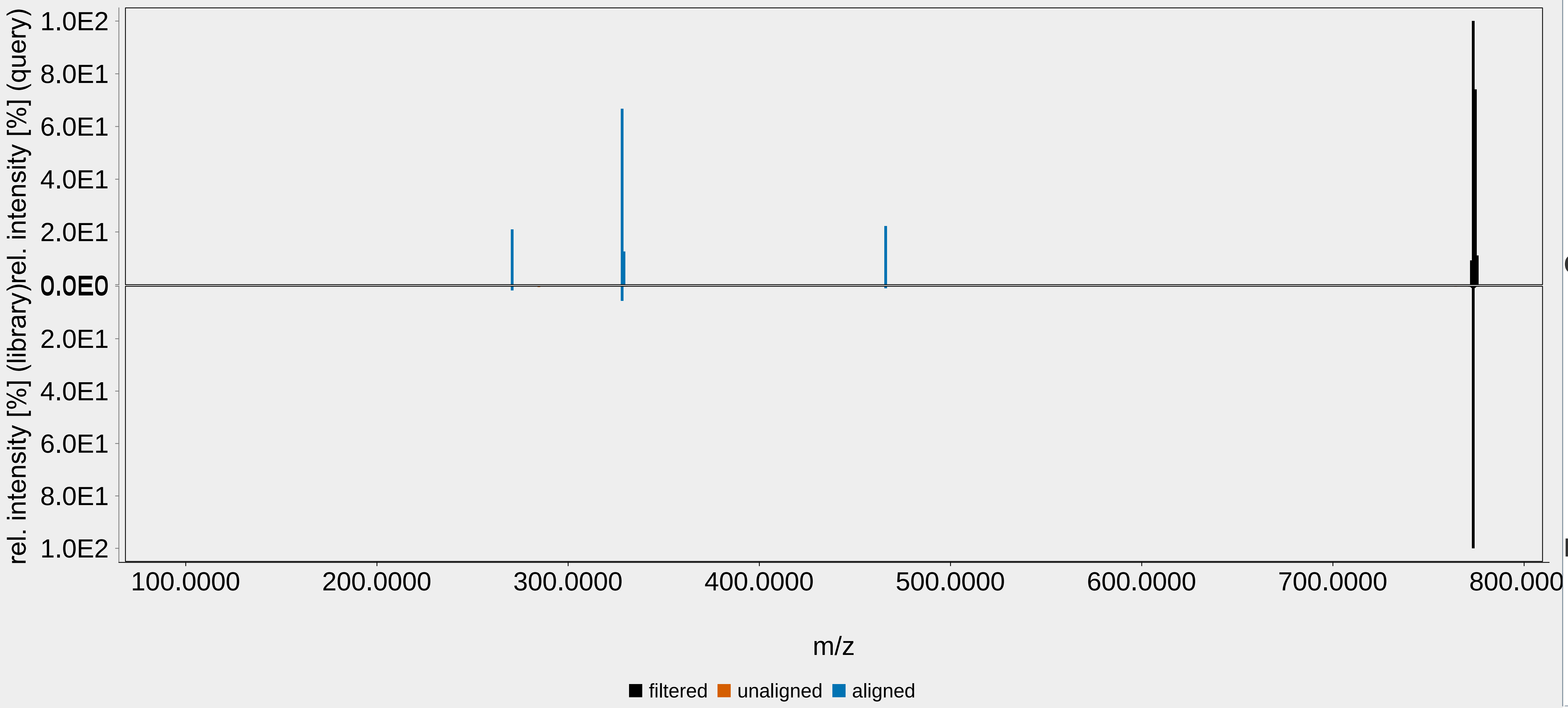

Compound information

Name: PE(17:0/22:6); [M-H]- C44H75N1O8P1

Exact mass: 0.0

Ion type: M-H

Precursor m/z: 776.523

Charge: 1

Polarity: Negative

Instrument information

Instrument type: HCD; Velos

Ion source: LC-ESI

Ms level: 2

Acquisition: Commercial

Database links

Other information

Principal investigator: Thomas Metz

Data collector: Thomas Metz

Entry ID: CCMSLIB00010087500

Spectral Match to 1-Hexadecanoyl-sn-glycero-3-phospho-(1'-myo-inositol) from NIST14

0.892

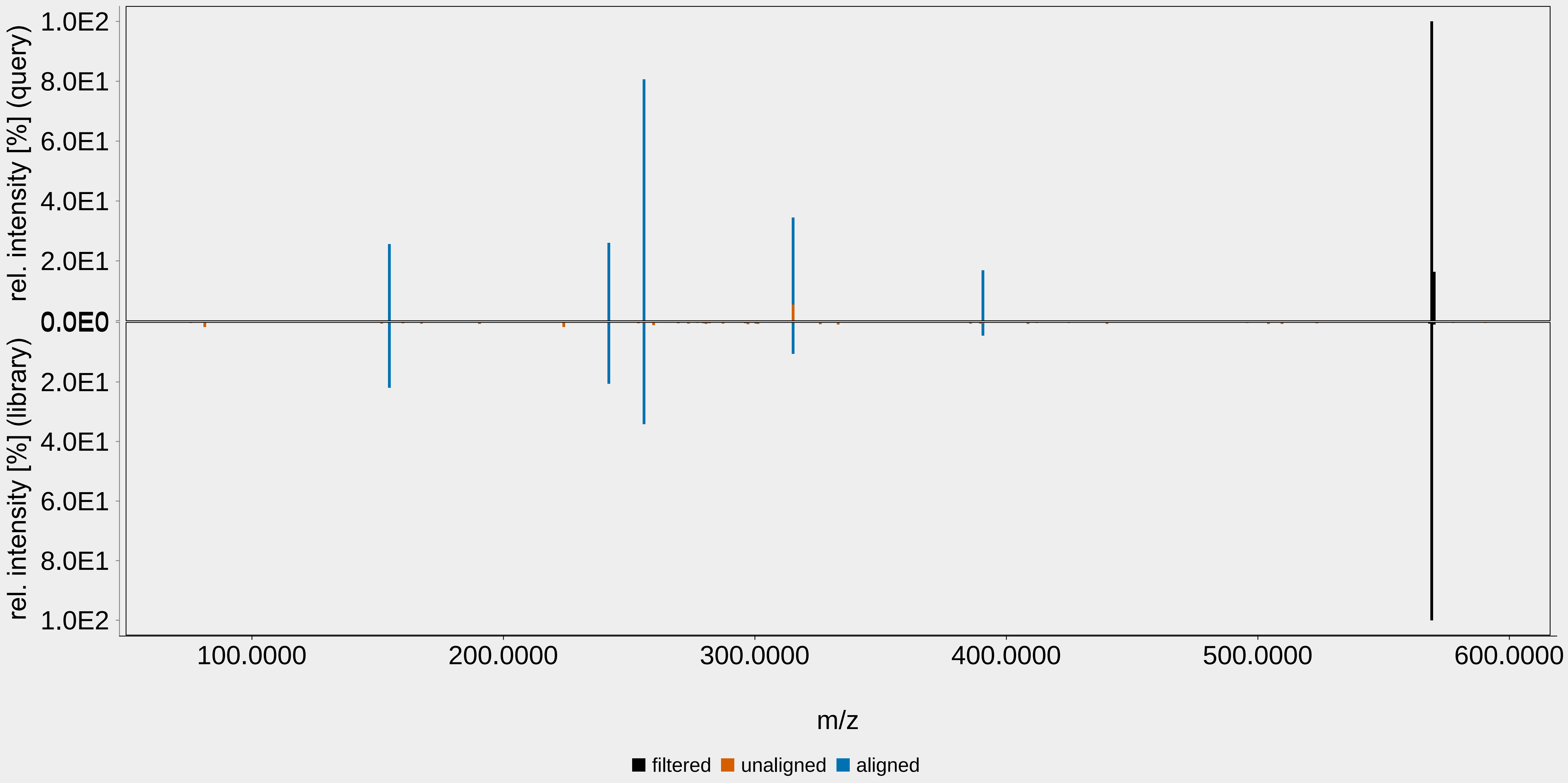

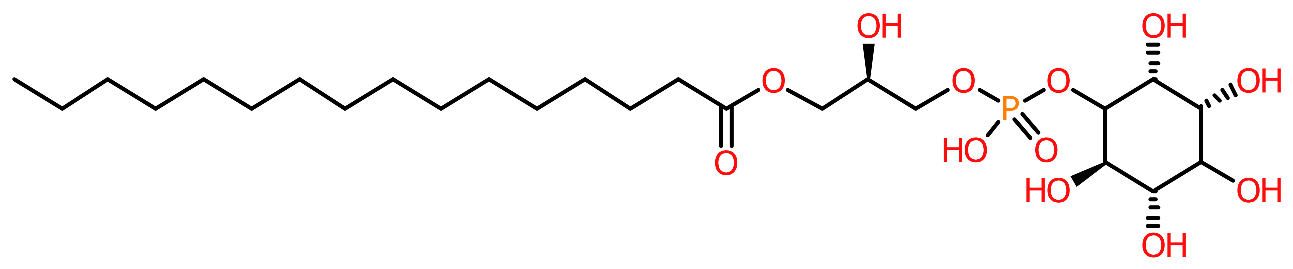

Compound information

Name: Spectral Match to

Exact mass: 572.296

Ion type: M-H

Precursor m/z: 571.294

Charge: 1

Polarity: Negative

InChI:

SMILES:

Instrument information

Instrument type: Ion Trap

Ion source: ESI

Ms level: 2

Acquisition: Isolated

Database links

CAS: 106182201

Other information

Principal investigator: Data from Pieter

Data collector: Data deposited by lfnothias

Entry ID: CCMSLIB00003136330

LPI 16:0 **0.973**

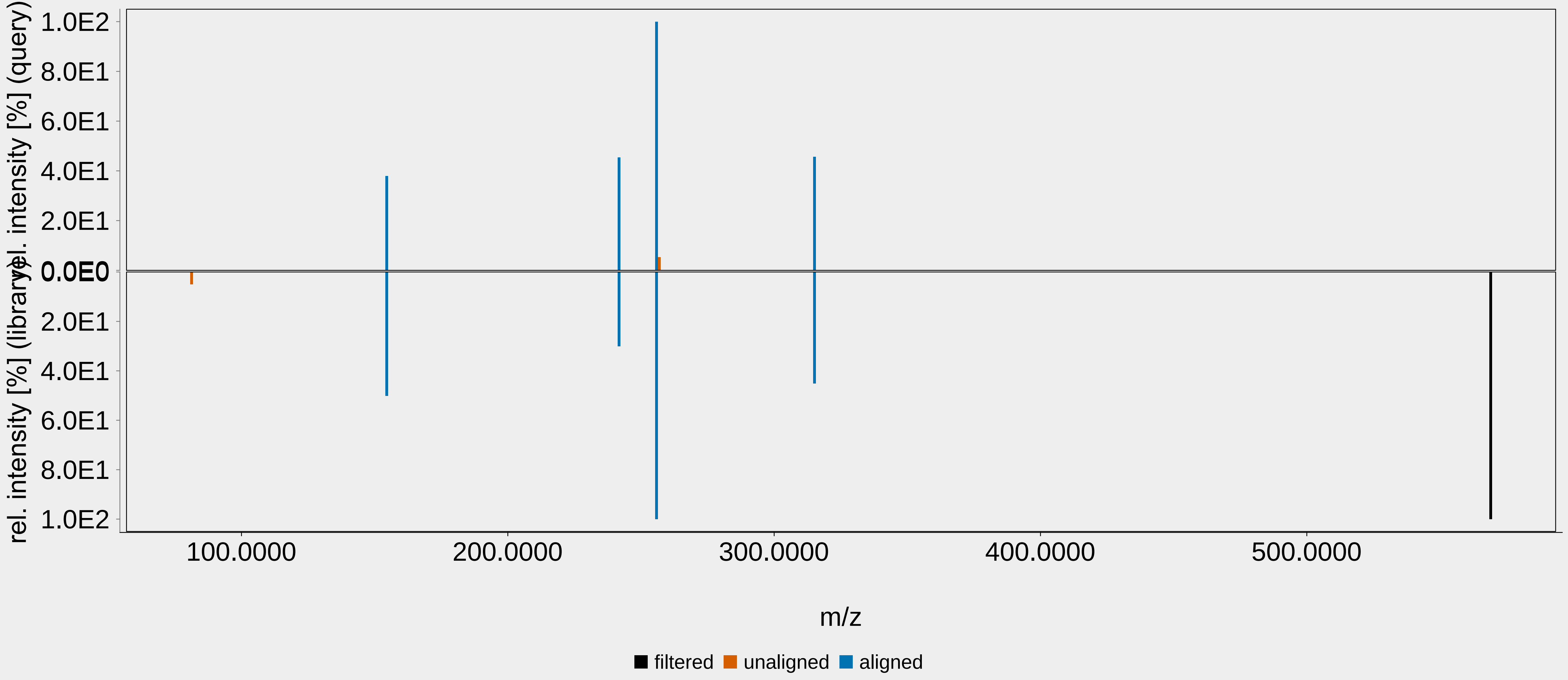

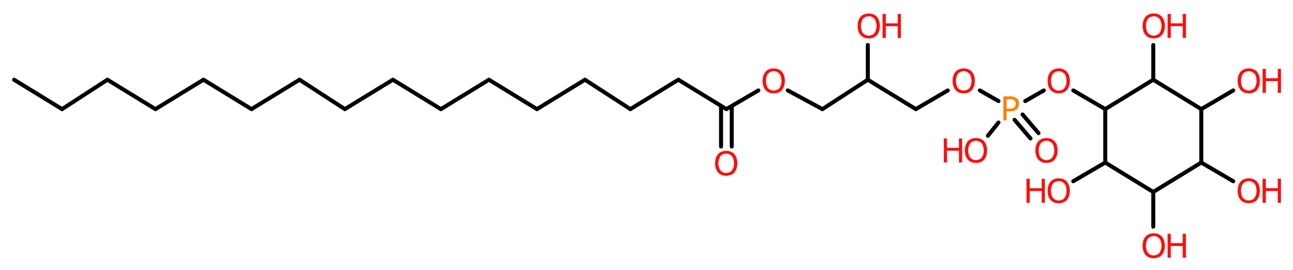

Compound information

Name: LPI 16:0

Formula: C25H49O12P

Ion type: [M-H]-

Precursor m/z: 571.28889

Ccs: 225.12

InChI key:

SMILES:

Num peaks: 6

Instrument information

Database links

Other information

Fosfructose - 30.0 eV

0.722

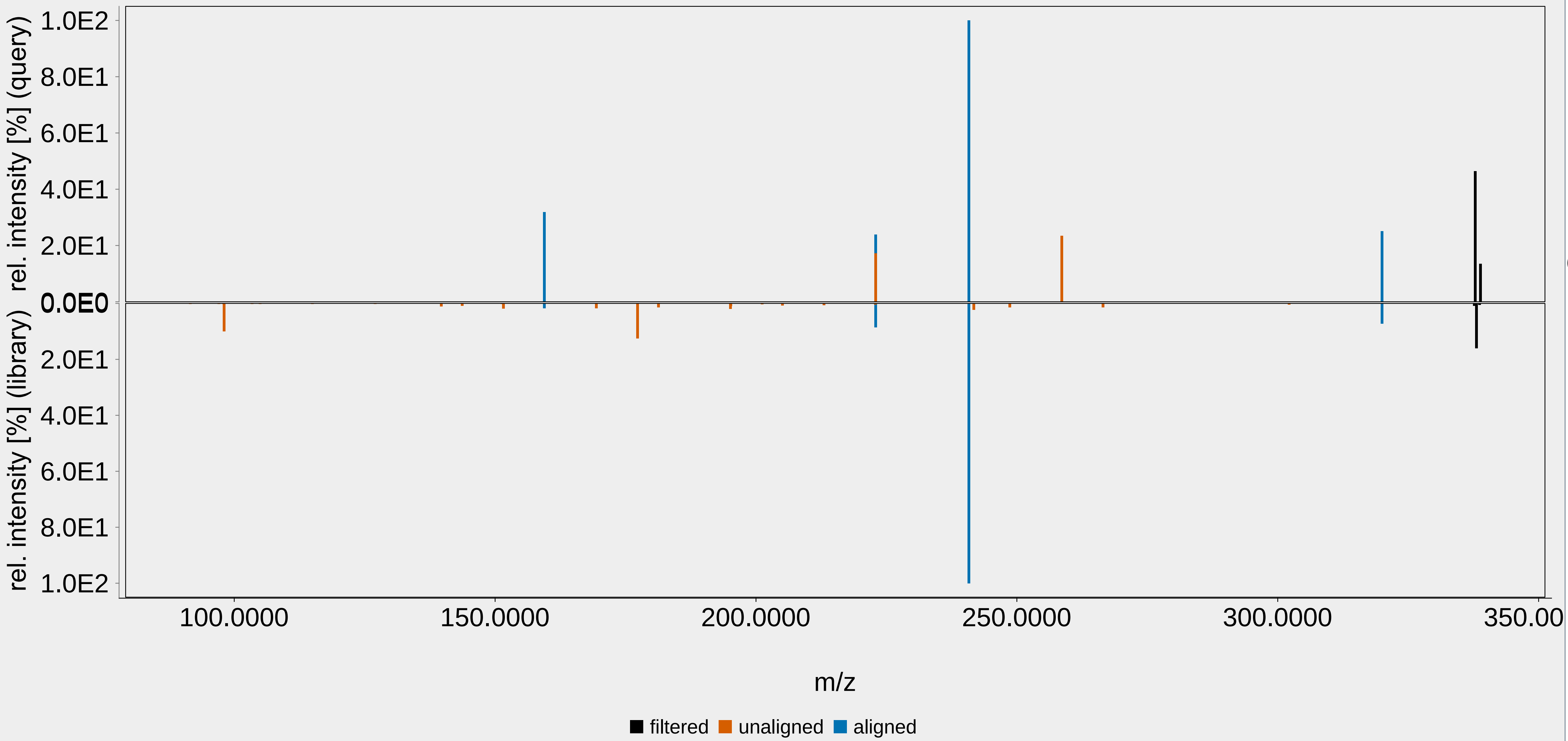

Compound information

Name: Fosfructose - 30.0 eV

Exact mass: 0.0

Ion type: M-H

Precursor m/z: 338.989

Charge: 1

Polarity: Negative

SMILES:

Instrument information

Instrument type: Orbitrap

Ion source: ESI

Ms level: 2

Acquisition: Isolated

Database links

CAS:

Other information

Principal investigator: PI

Data collector: Data Collector

Entry ID: CCMSLIB00006121372

Fosfructose - 30.0 eV

0.720

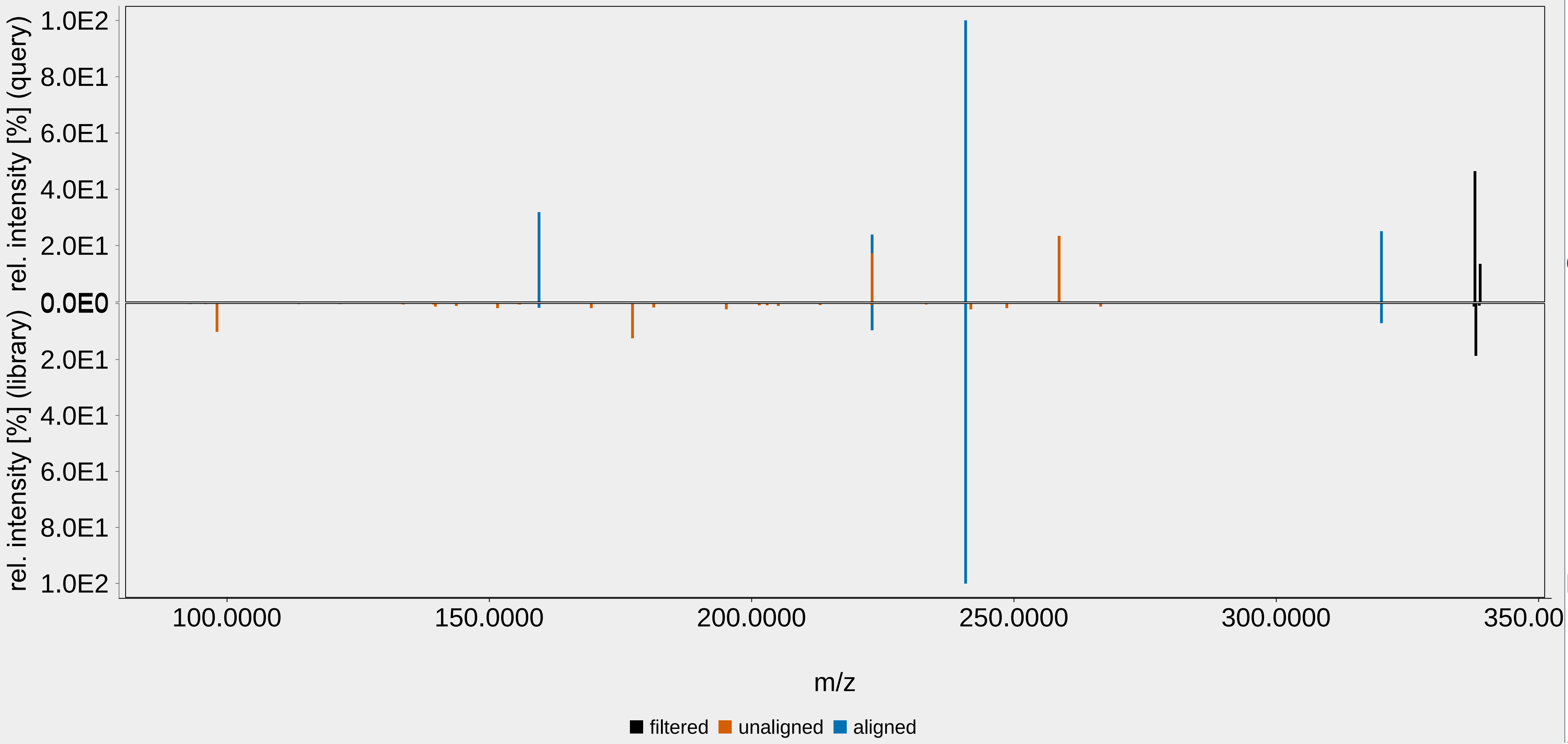

Compound information

Name: Fosfructose - 30.0 eV

Exact mass: 0.0

Ion type: M-H

Precursor m/z: 338.989

Charge: 1

Polarity: Negative

SMILES:

Instrument information

Instrument type: Orbitrap

Ion source: ESI

Ms level: 2

Acquisition: Isolated

Database links

CAS:

Other information

Principal investigator: PI

Data collector: Data Collector

Entry ID: CCMSLIB00006121374

PA 18:1_20:4

0.891

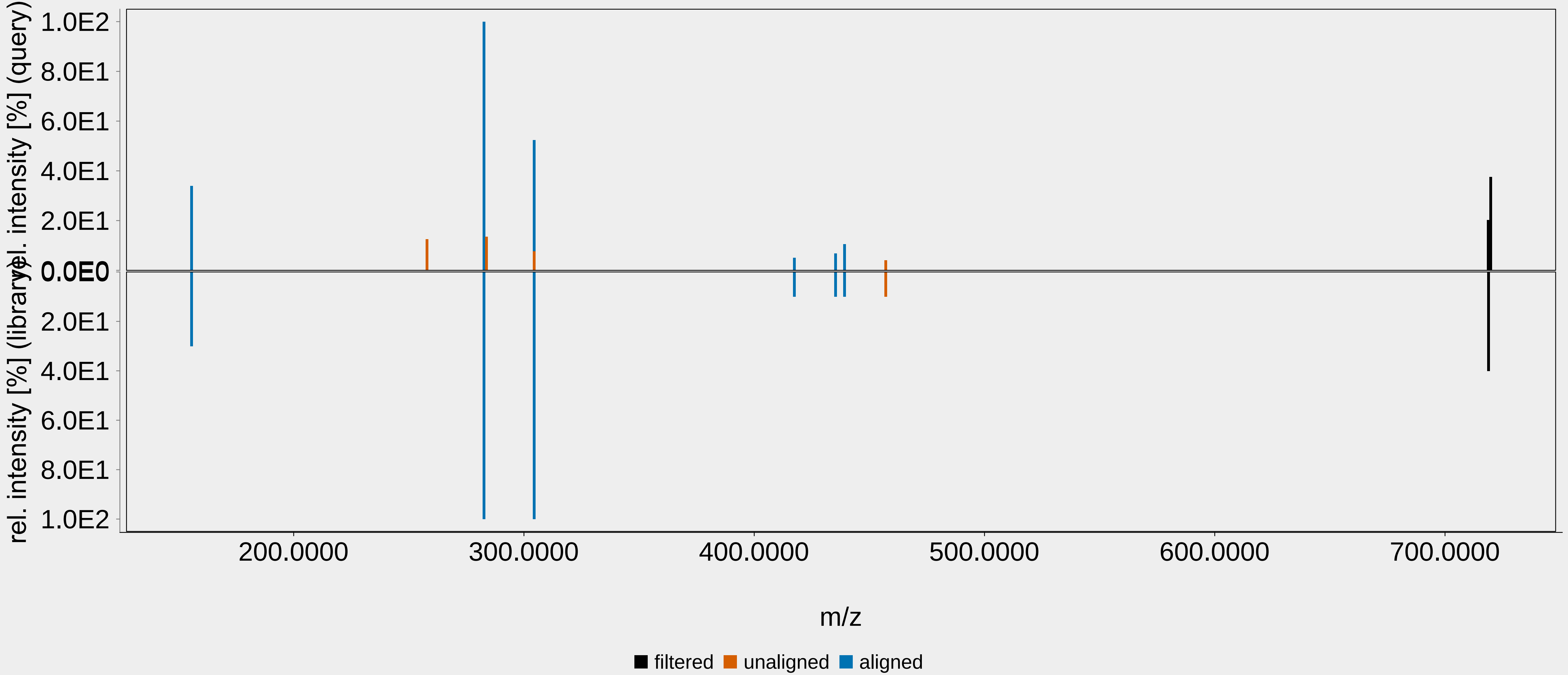

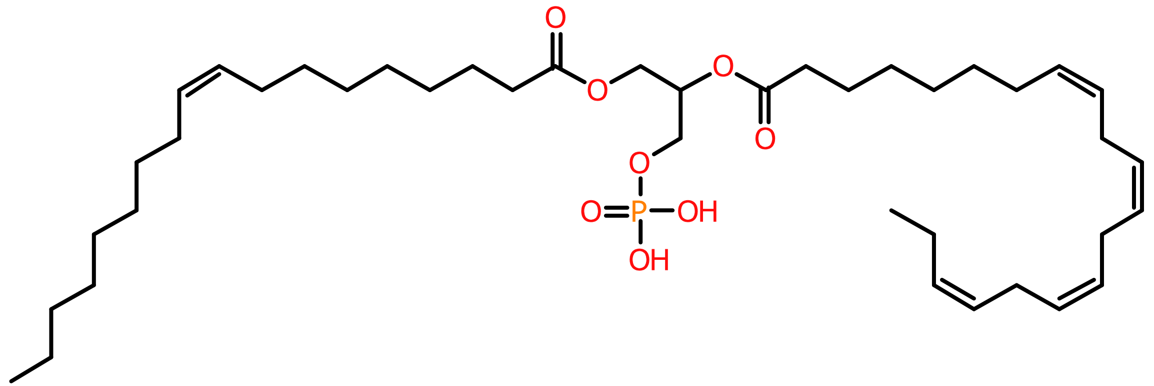

Compound information

Name: PA 18:1_20:4

Formula: C41H71O8P

Ion type: [M-H]-

Precursor m/z: 721.48138

Ccs: 261.27

InChI key:

SMILES:

Num peaks: 8

Instrument information

Database links

Other information

PE(20:1/22:6); [M-H]- C47H79N1O8P1 0.780

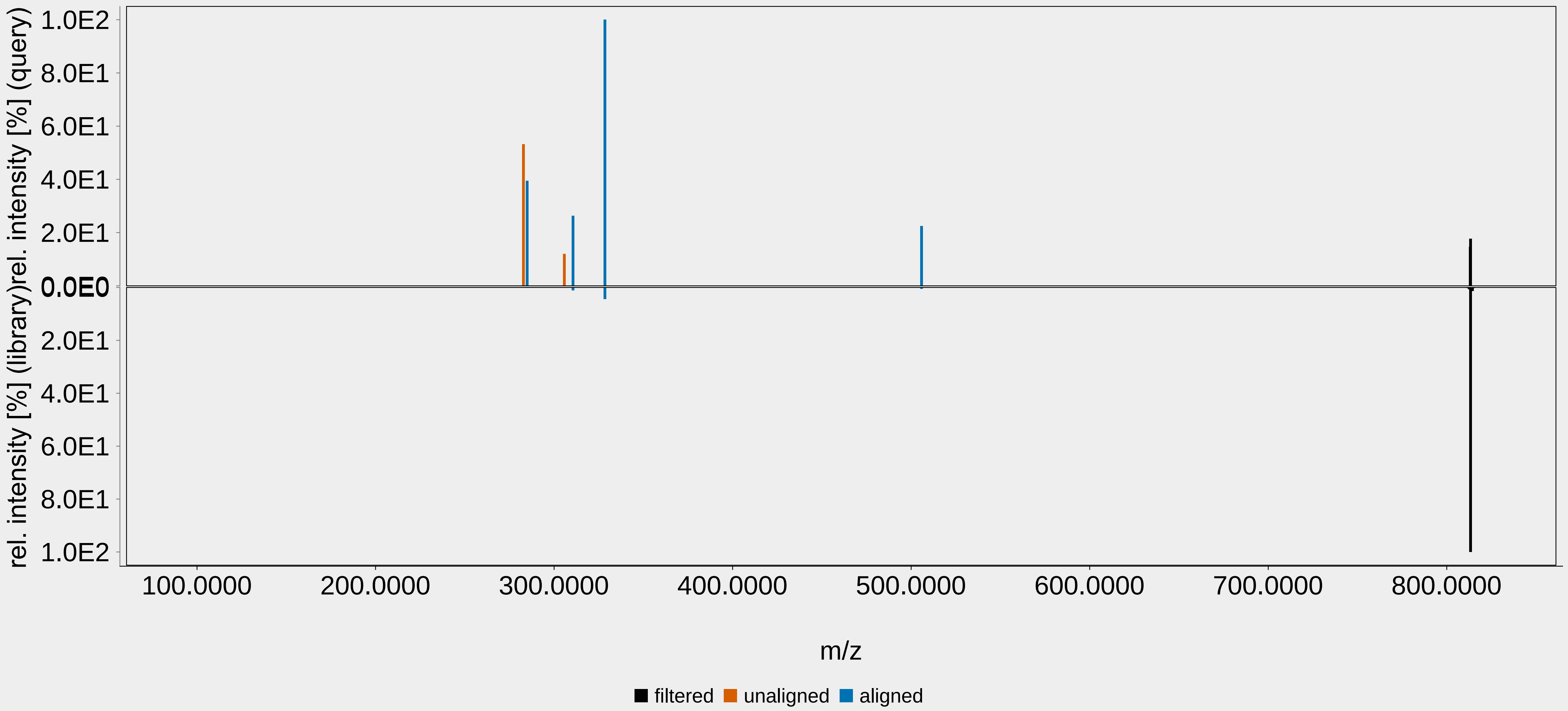

Compound information

Name: PE(20:1/22:6); [M-H]- C47H79N1O8P1

Exact mass: 0.0

Ion type: M-H

Precursor m/z: 816.554

Charge: 1

Polarity: Negative

Instrument information

Instrument type: HCD; Velos

Ion source: LC-ESI

Ms level: 2

Acquisition: Commercial

Database links

Other information

Principal investigator: Thomas Metz

Data collector: Thomas Metz

Entry ID: CCMSLIB00010073329

PS(18:1/18:1); [M-H]- C42H77N1O10P1 **0.830**

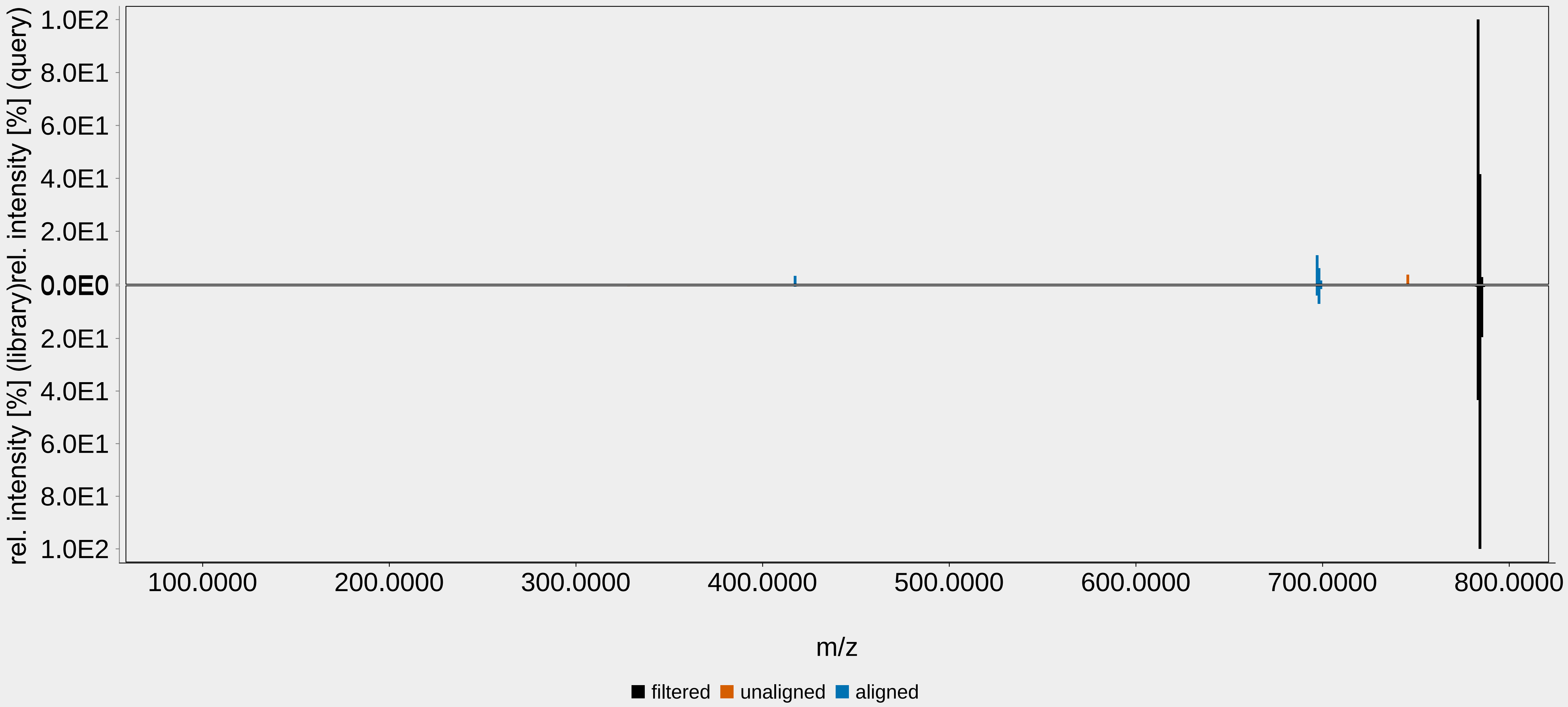

Compound information

Name: PS(18:1/18:1); [M-H]- C42H77N1O10P1

Exact mass: 0.0

Ion type: M-H

Precursor m/z: 786.529

Charge: 1

Polarity: Negative

Instrument information

Instrument type: HCD; Velos

Ion source: LC-ESI

Ms level: 2

Acquisition: Commercial

Database links

Other information

Principal investigator: Thomas Metz

Data collector: Thomas Metz

Entry ID: CCMSLIB00010084418

PS 18:1_18:1

0.869

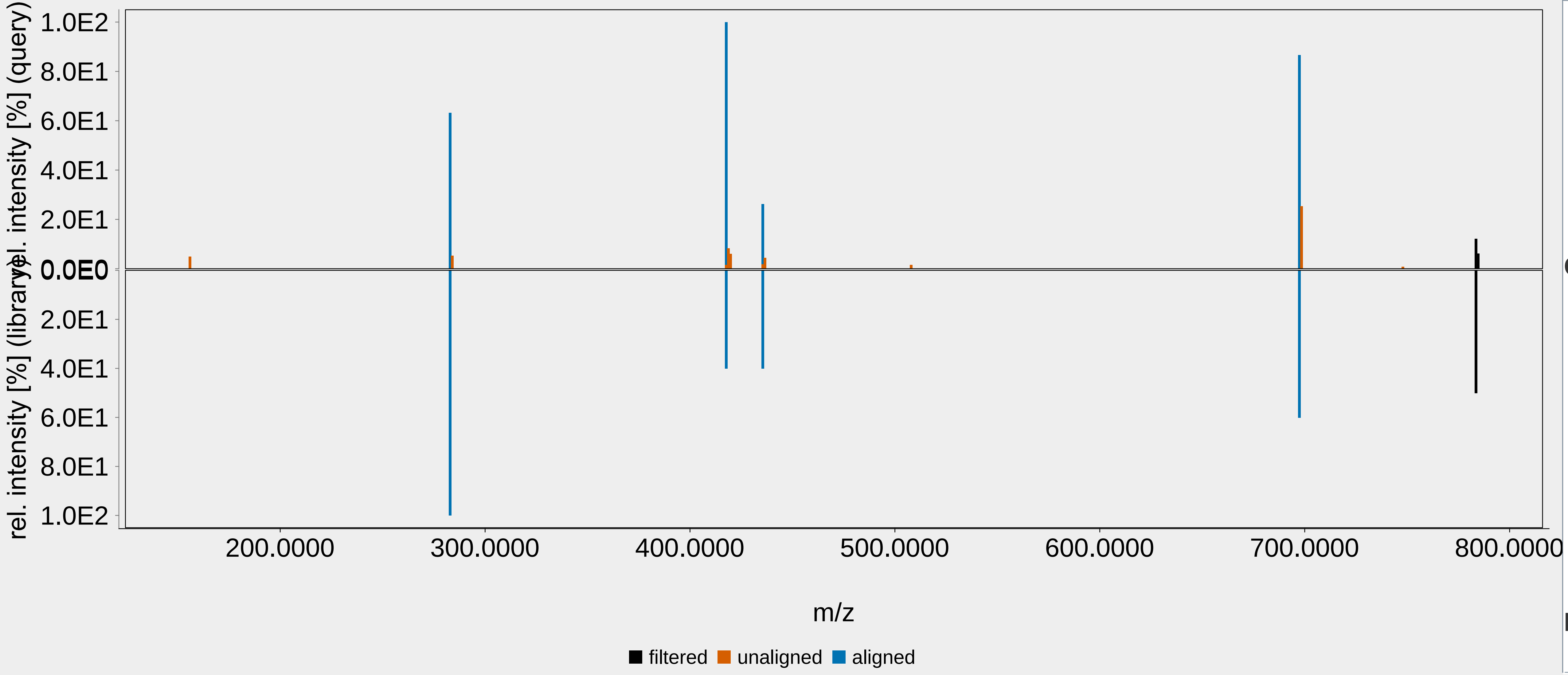

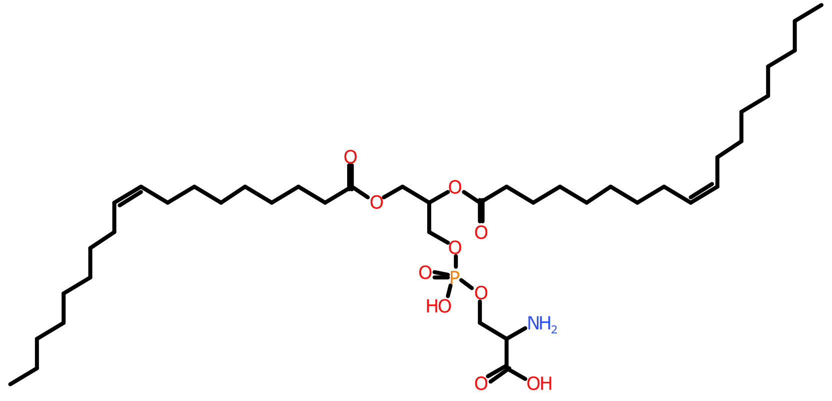

Compound information

Name: PS 18:1_18:1

Formula: C42H78NO10P

Ion type: [M-H]-

Precursor m/z: 786.52906

Ccs: 275.77

InChI key:

SMILES:

Num peaks: 5

Instrument information

Database links

Other information

UPD-alpha-D-Galactose - 30.0 eV

0.752

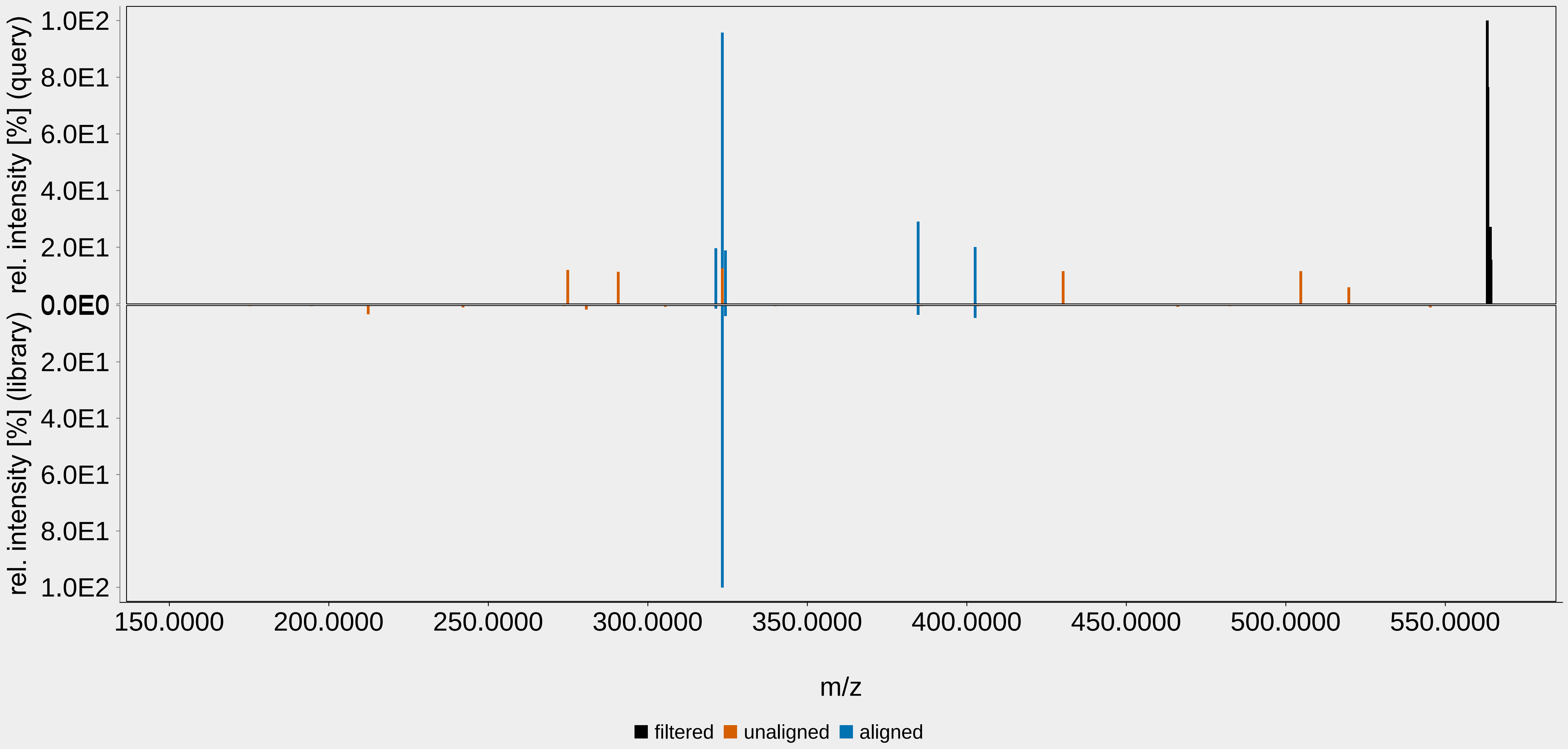

Compound information

Name: UPD-alpha-D-Galactose - 30.0 eV

Exact mass: 0.0

Ion type: Unknown

Precursor m/z: 565.048

Charge: 1

Polarity: Negative

SMILES:

Instrument information

Instrument type: Orbitrap

Ion source: ESI

Ms level: 2

Acquisition: Isolated

Database links

CAS:

Other information

Principal investigator: PI

Data collector: Data Collector

Entry ID: CCMSLIB00006113004

UPD-alpha-D-Galactose - 30.0 eV

0.757

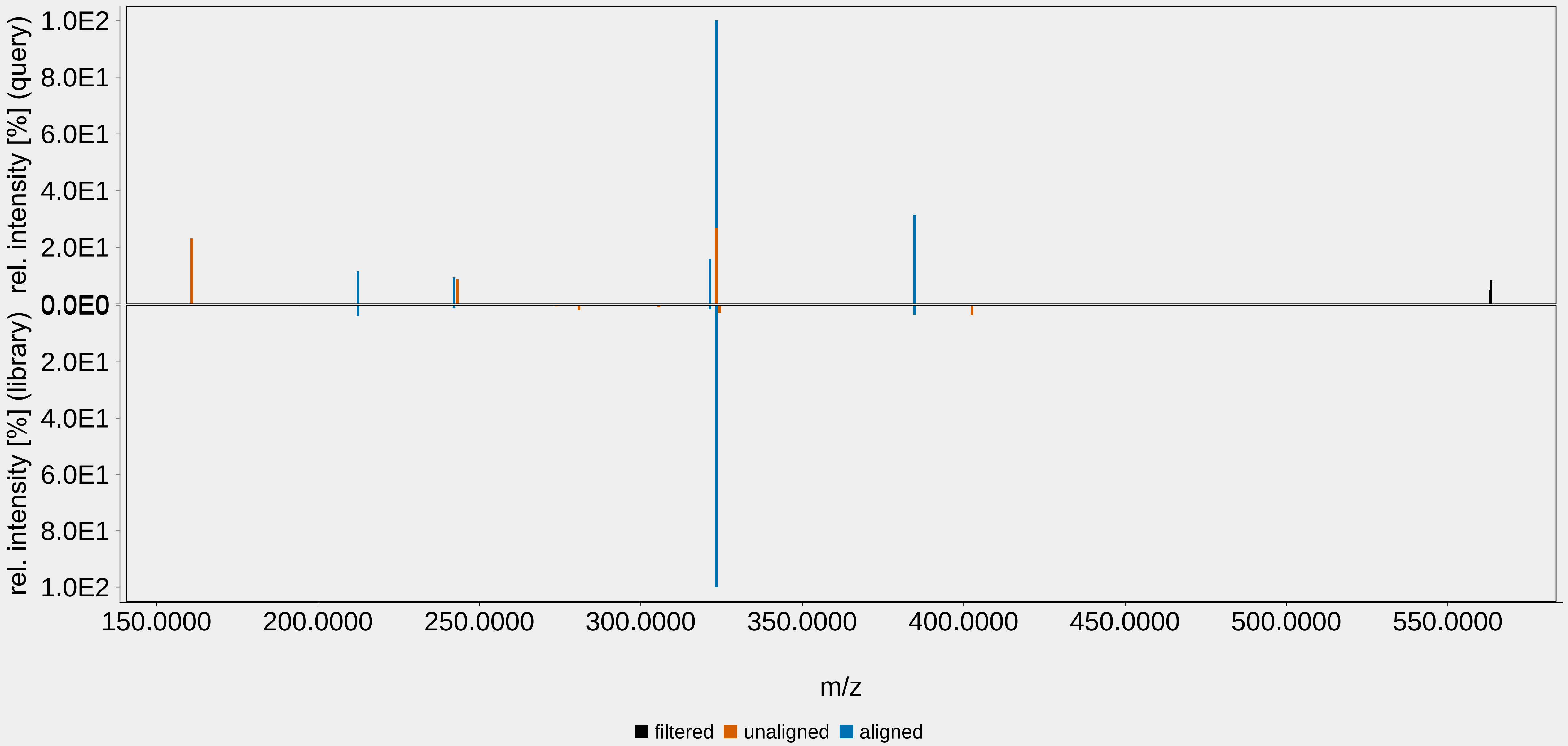

Compound information

Name: UPD-alpha-D-Galactose - 30.0 eV

Exact mass: 0.0

Ion type: Unknown

Precursor m/z: 565.048

Charge: 1

Polarity: Negative

SMILES:

Instrument information

Instrument type: Orbitrap

Ion source: ESI

Ms level: 2

Acquisition: Isolated

Database links

CAS:

Other information

Principal investigator: PI

Data collector: Data Collector

Entry ID: CCMSLIB00006113006

Spectral Match to 1,2-Di(4Z,7Z,10Z,13Z,16Z,19Z-docosahexaenoyl)-sn-glycero-3-phosphoethanolamine from NIST14

0.871

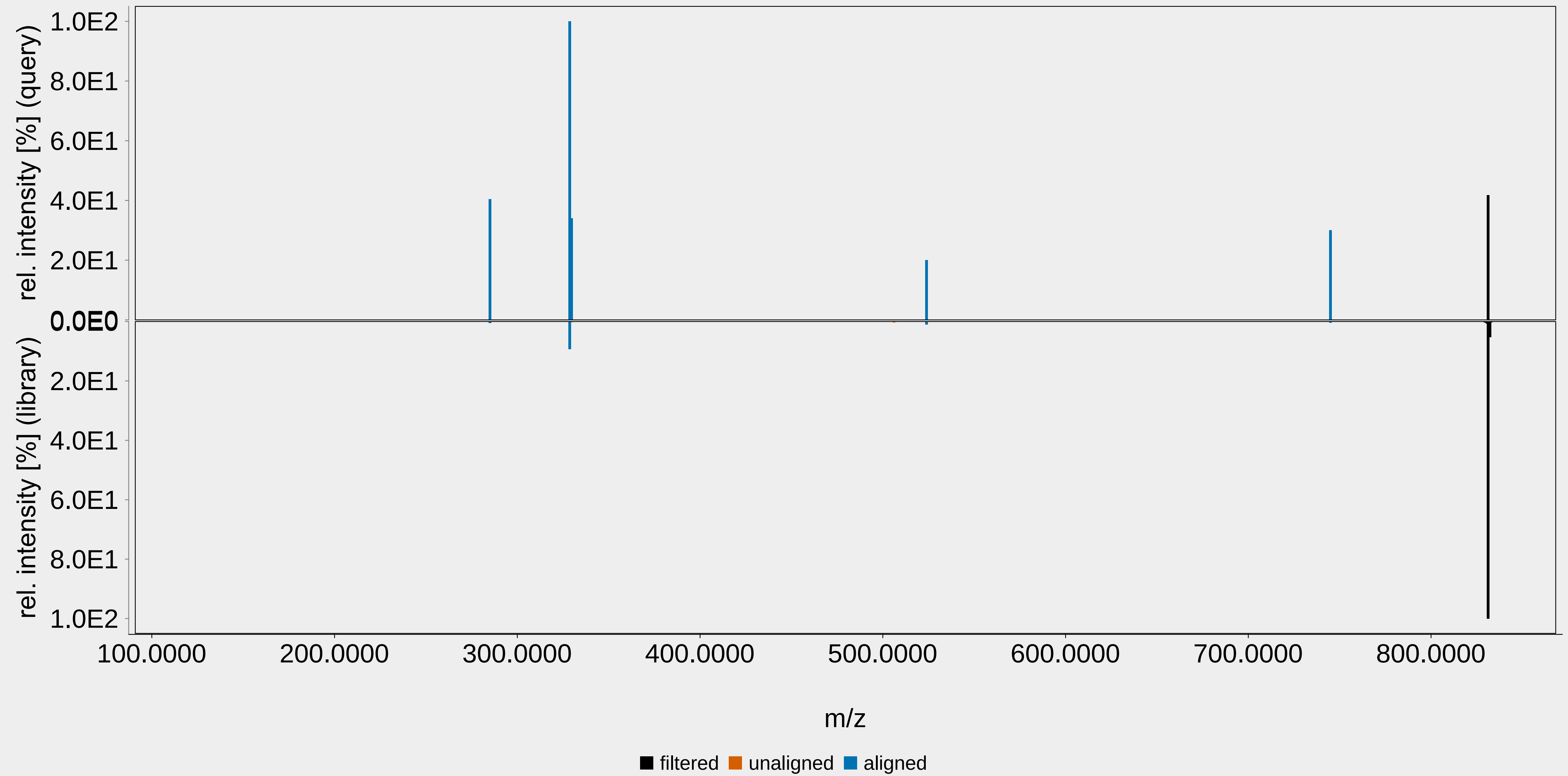

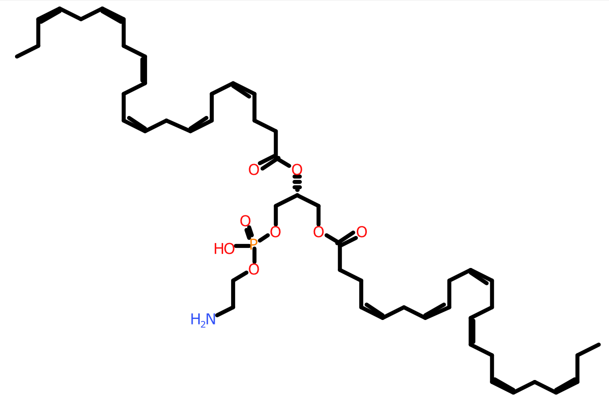

Compound information

Name: Spectral Match to

Exact mass: 835.515

Ion type: M-H

Precursor m/z: 834.512

Charge: 1

Polarity: Negative

InChI:

SMILES:

Instrument information

Instrument type: qTof

Ion source: ESI

Ms level: 2

Acquisition: Isolated

Database links

CAS: 123284811

Other information

Principal investigator: Data from Katrina

Data collector: Data deposited by alchemistmatt

Entry ID: CCMSLIB00003140104

PI(20:4/0:0); [M-H]- C29H48O12P1 **0.742**

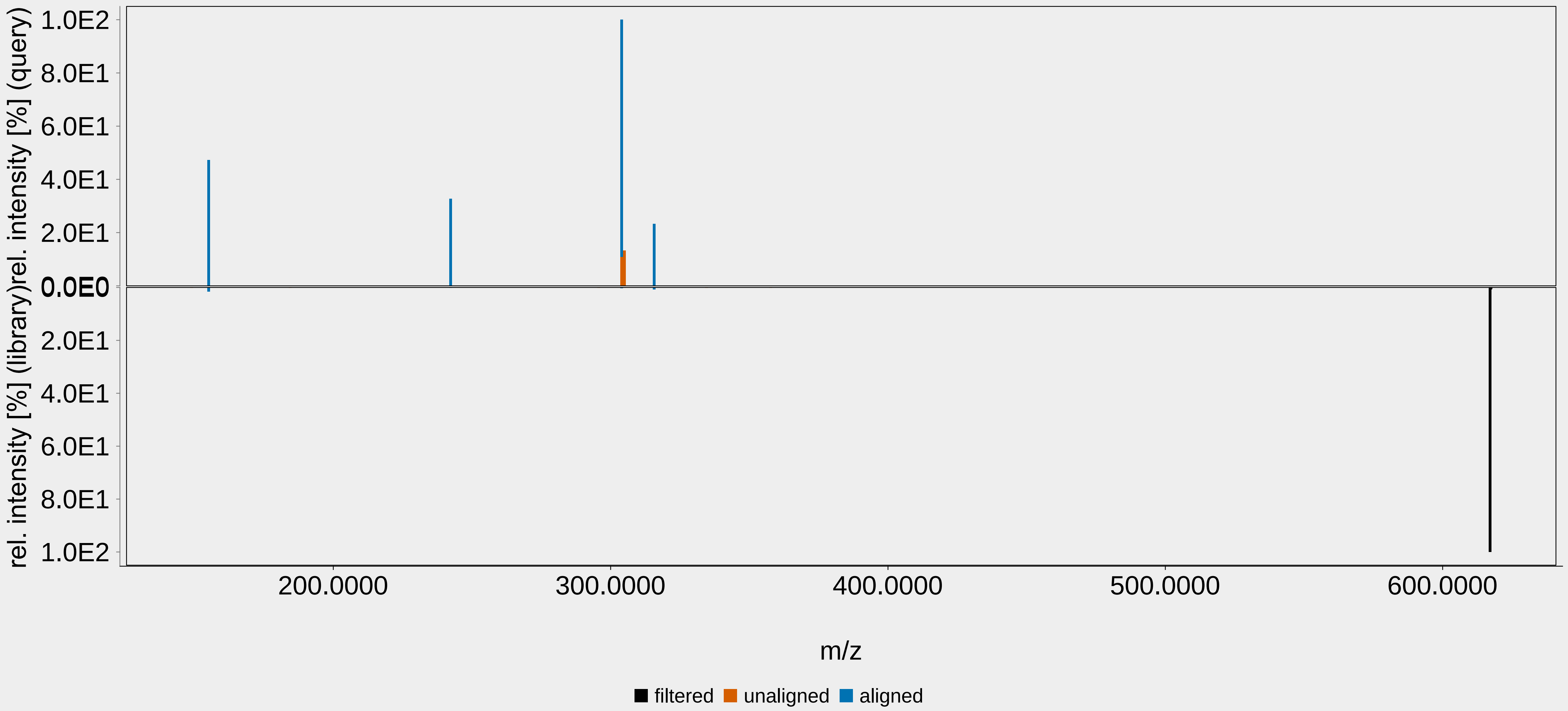

Compound information

Name: PI(20:4/0:0); [M-H]- C29H48O12P1

Exact mass: 0.0

Ion type: M-H

Precursor m/z: 619.288

Charge: 1

Polarity: Negative

Instrument information

Instrument type: HCD; Velos

Ion source: LC-ESI

Ms level: 2

Acquisition: Commercial

Database links

Other information

Principal investigator: Thomas Metz

Data collector: Thomas Metz

Entry ID: CCMSLIB00010084582

LPI 20:4 0.928

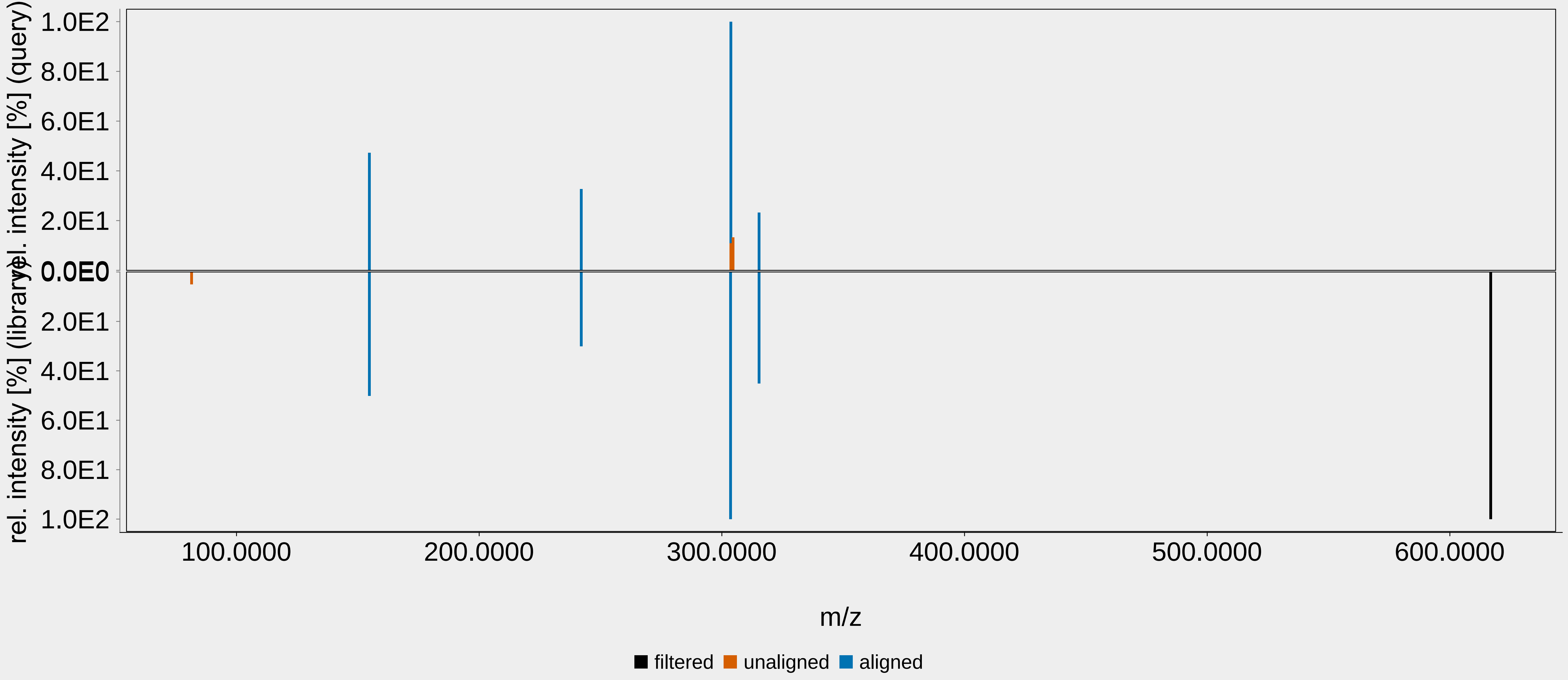

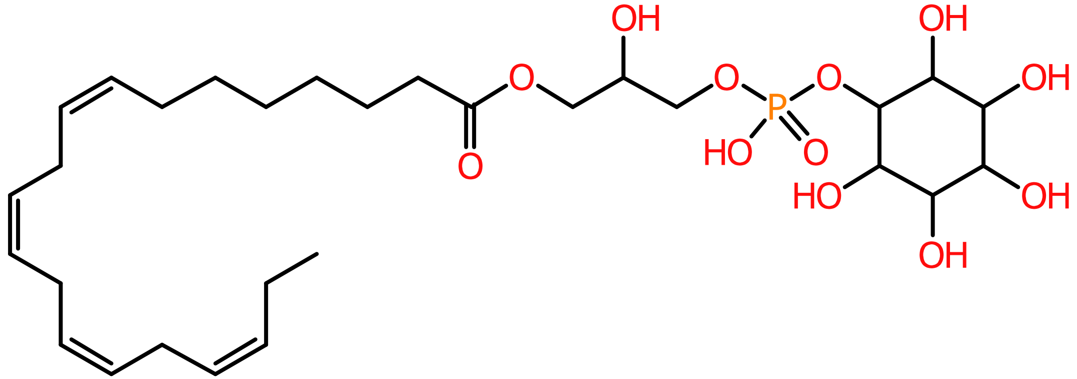

Compound information

Name: LPI 20:4

Formula: C29H49O12P

Ion type: [M-H]-

Precursor m/z: 619.28889

Ccs: 233.07

InChI key:

SMILES:

Num peaks: 6

Instrument information

Database links

Other information

PE(18:2/0:0); [M-H]- C23H43N1O7P1 0.872

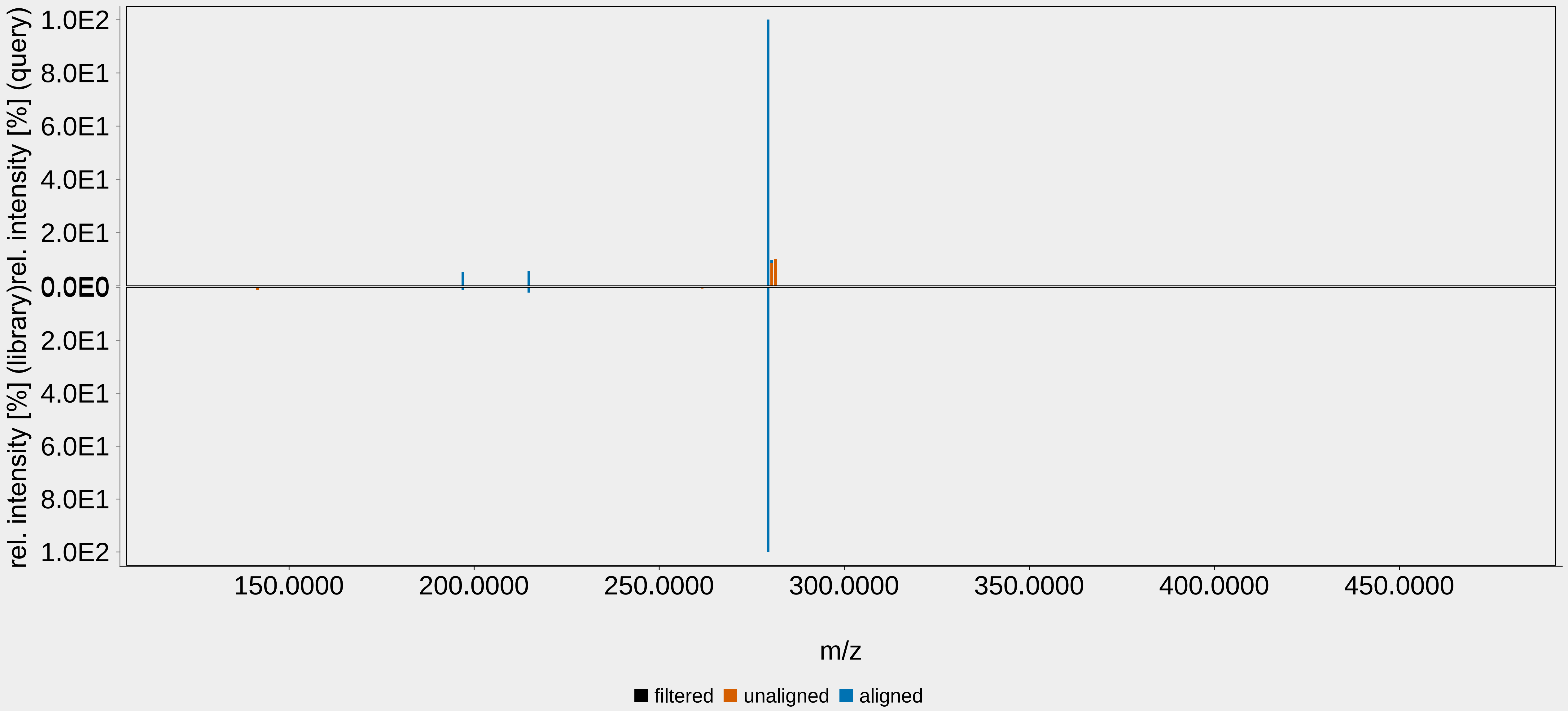

Compound information

Name: PE(18:2/0:0); [M-H]- C23H43N1O7P1

Exact mass: 0.0

Ion type: M-H

Precursor m/z: 476.278

Charge: 1

Polarity: Negative

Instrument information

Instrument type: HCD; Lumos

Ion source: LC-ESI

Ms level: 2

Acquisition: Commercial

Database links

Other information

Principal investigator: Thomas Metz

Data collector: Thomas Metz

Entry ID: CCMSLIB00010059116

PE(18:2/0:0); [M-H]- C23H43N1O7P1 | 0.875

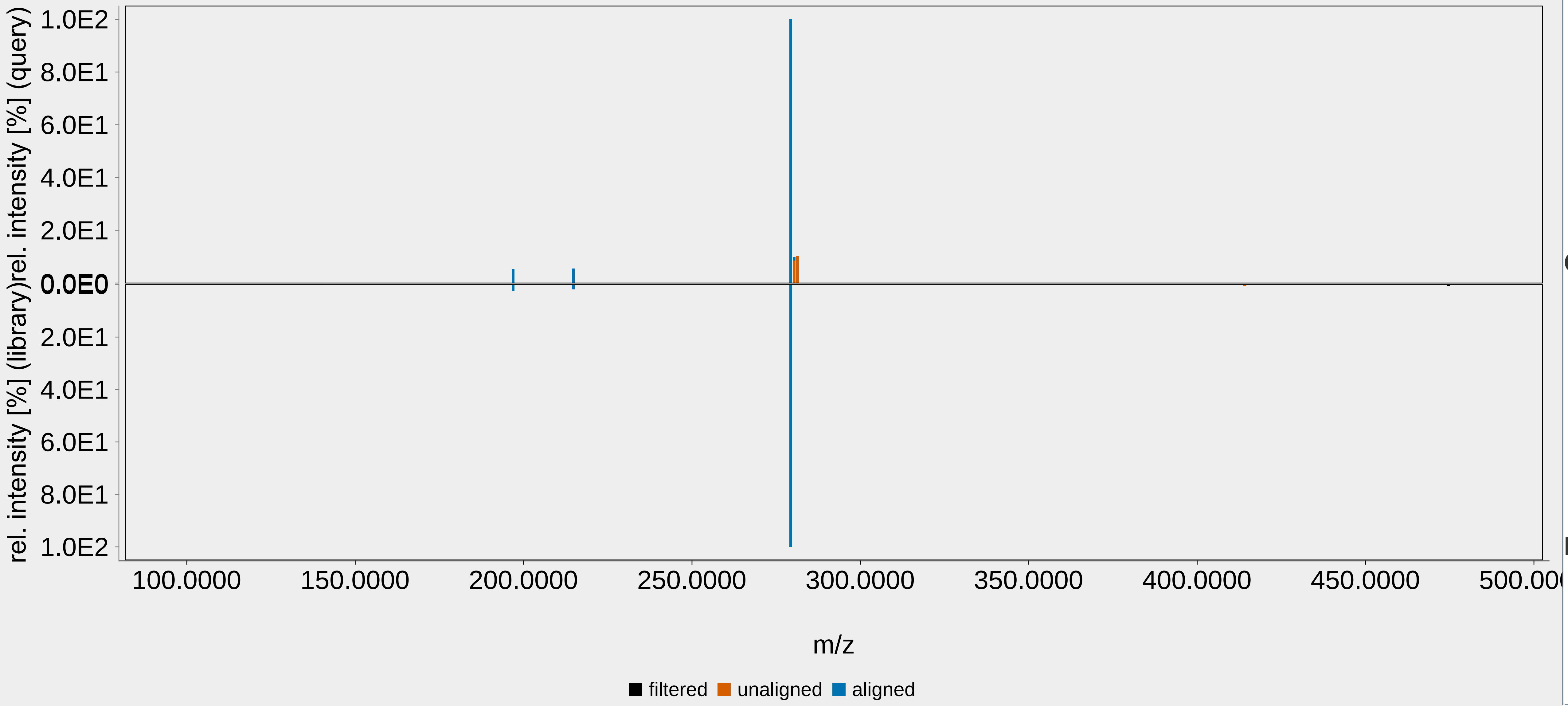

Compound information

Name: PE(18:2/0:0); [M-H]- C23H43N1O7P1

Exact mass: 0.0

Ion type: M-H

Precursor m/z: 476.278

Charge: 1

Polarity: Negative

Instrument information

Instrument type: CID; Lumos

Ion source: LC-ESI

Ms level: 2

Acquisition: Commercial

Database links

Other information

Principal investigator: Thomas Metz

Data collector: Thomas Metz

Entry ID: CCMSLIB00010059801

Guanosine triphosphate

0.779

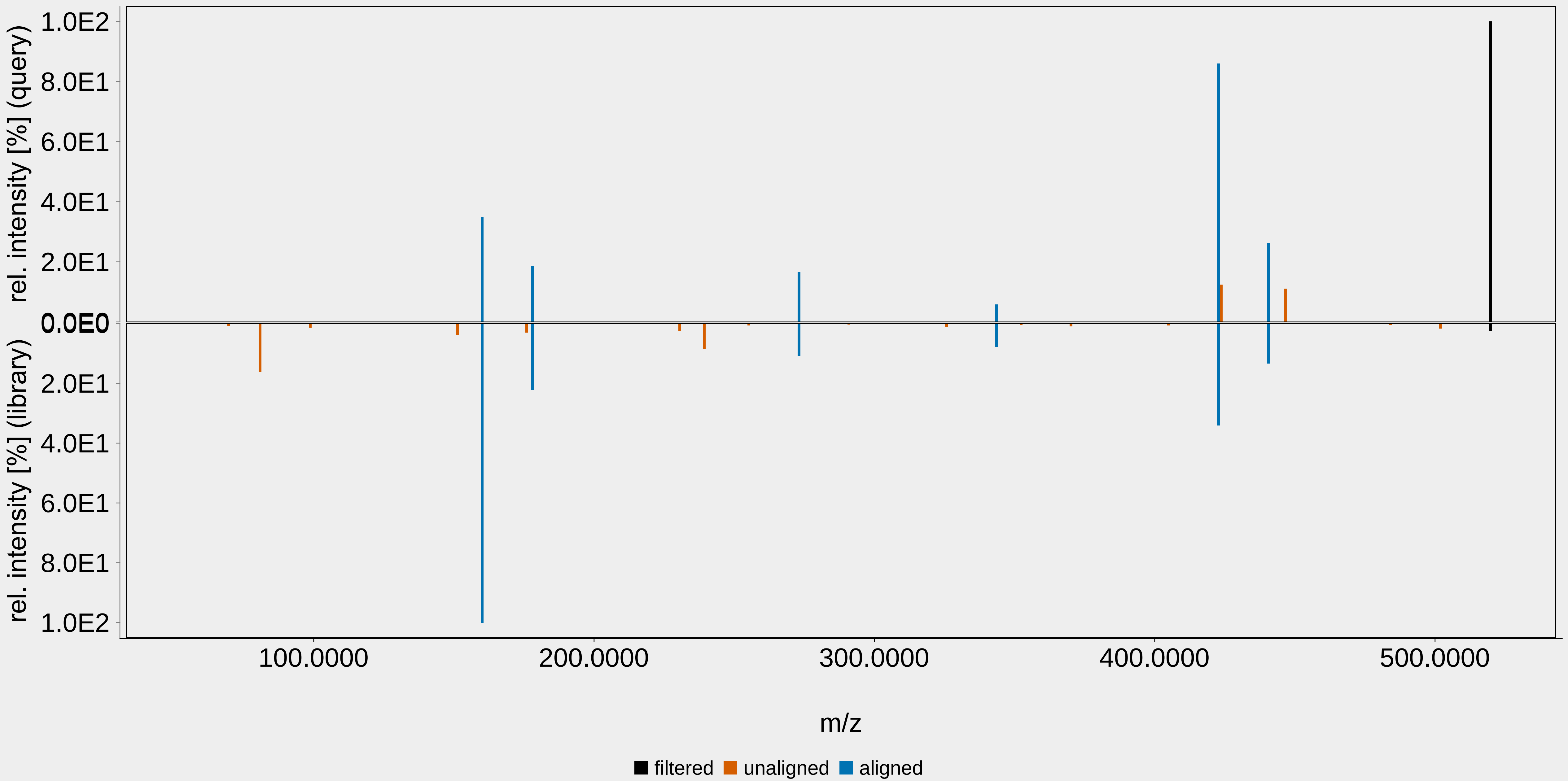

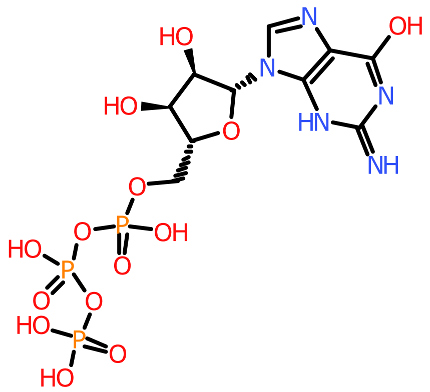

Compound information

Name: Guanosine triphosphate

Formula: C10H16N5O14P3

Ion type: [M-H]-

Precursor m/z: 521.981868542491

RT: 6.6404014

Polarity: negative

InChI:

InChI key:

SMILES:

Instrument information

Instrument type: LC-ESI-QFT

Instrument: Thermo Q-Exactive Plus

Ms level: MS2

Collision energy: 30.0 eV

Database links

Other information

Principal investigator: Prasad Phapale

Data collector: Prasad Phapale

Guanosine triphosphate

0.781

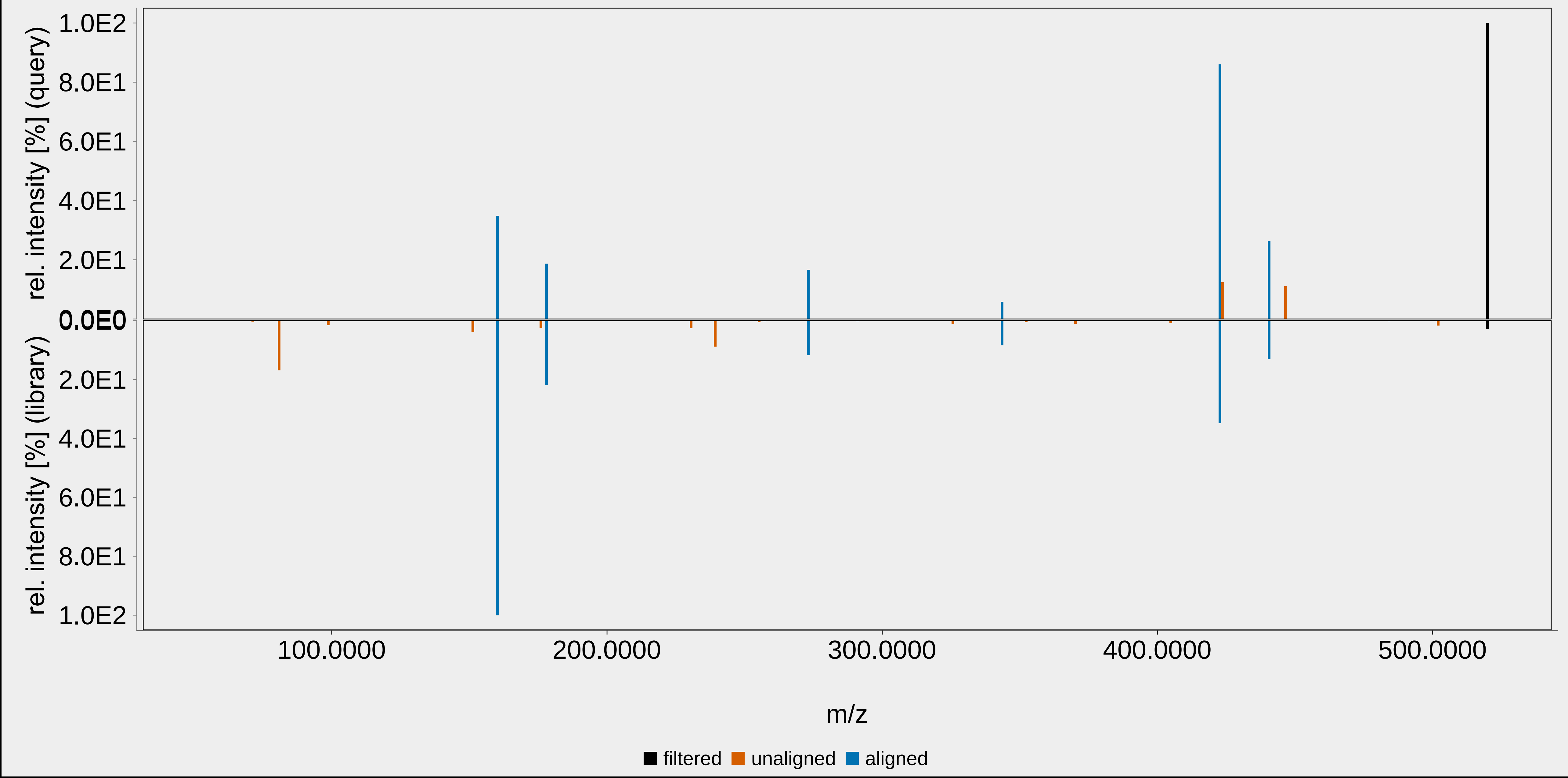

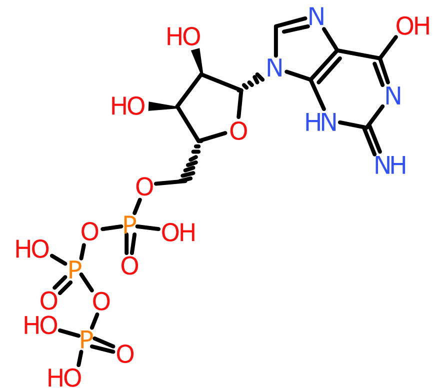

Compound information

Name: Guanosine triphosphate

Formula: C10H16N5O14P3

Ion type: [M-H]-

Precursor m/z: 521.981890480549

RT: 6.6717806

Polarity: negative

InChI:

InChI key:

SMILES:

Instrument information

Instrument type: LC-ESI-QFT

Instrument: Thermo Q-Exactive Plus

Ms level: MS2

Collision energy: 30.0 eV

Database links

Other information

Principal investigator: Prasad Phapale

Data collector: Prasad Phapale

PA 20:4_22:6

0.934

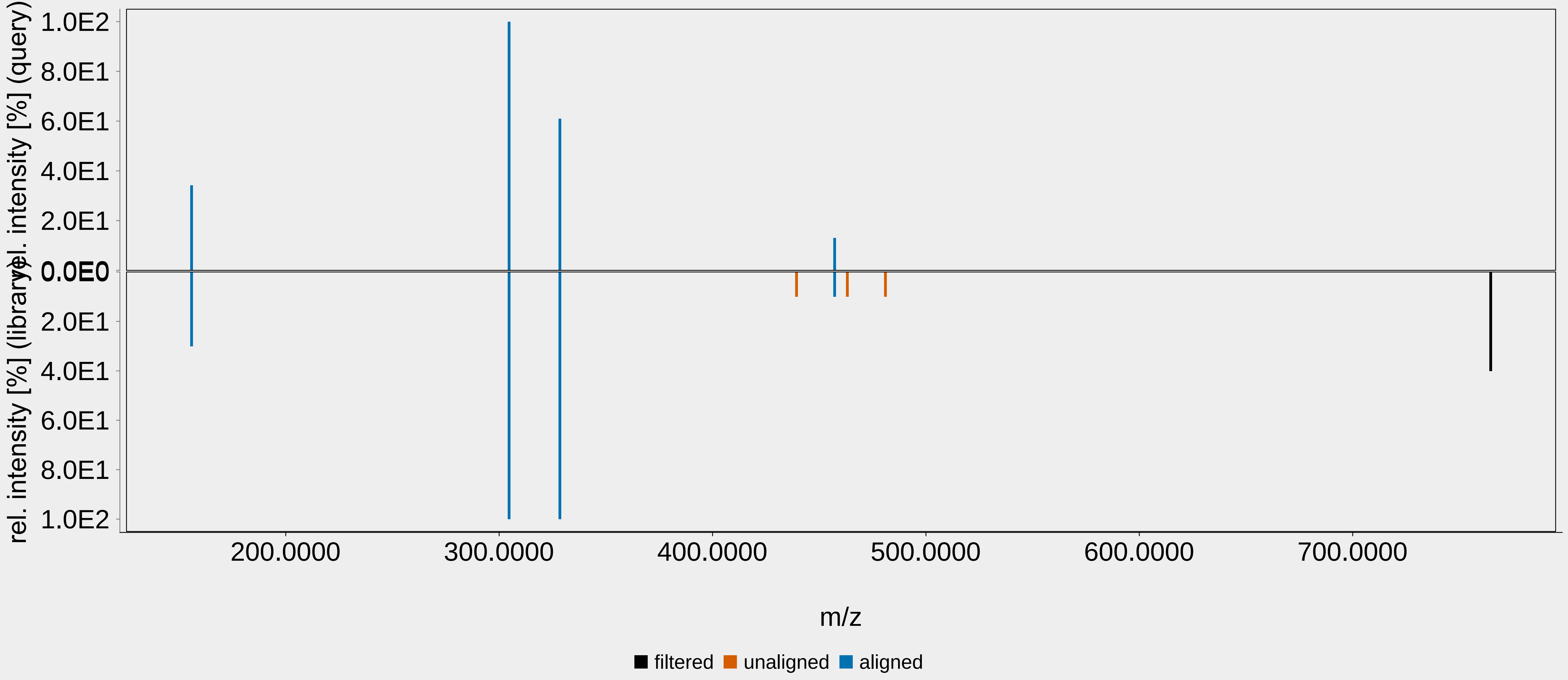

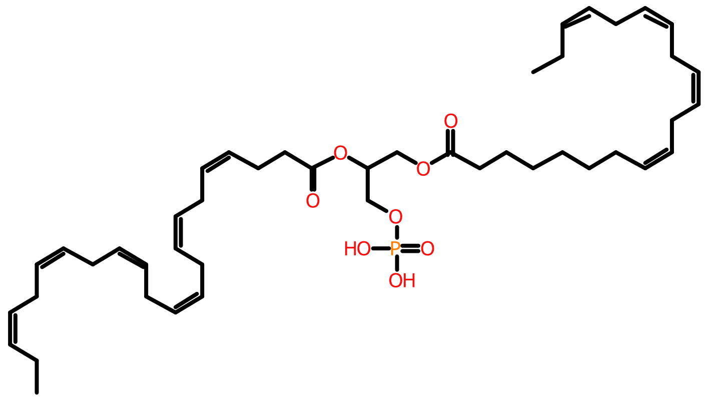

Compound information

Name: PA 20:4_22:6

Formula: C45H69O8P

Ion type: [M-H]-

Precursor m/z: 767.46573

Ccs: 264.96

InChI key:

SMILES:

Num peaks: 8

Instrument information

Database links

Other information

Spectral Match to 1-(9Z-Octadecenoyl)-sn-glycero-3-phospho-(1'-myo-inositol) from NIST14

0.804

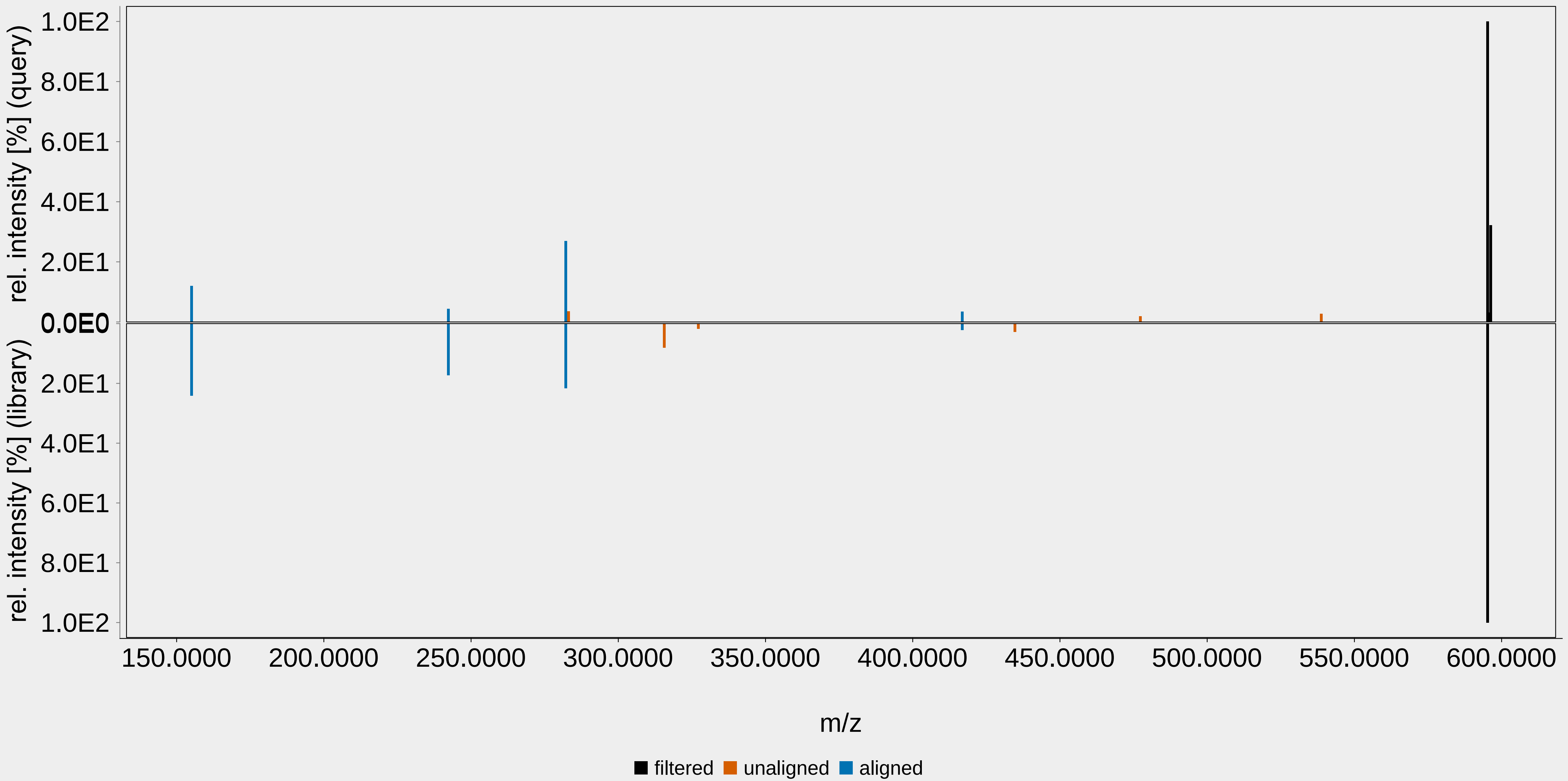

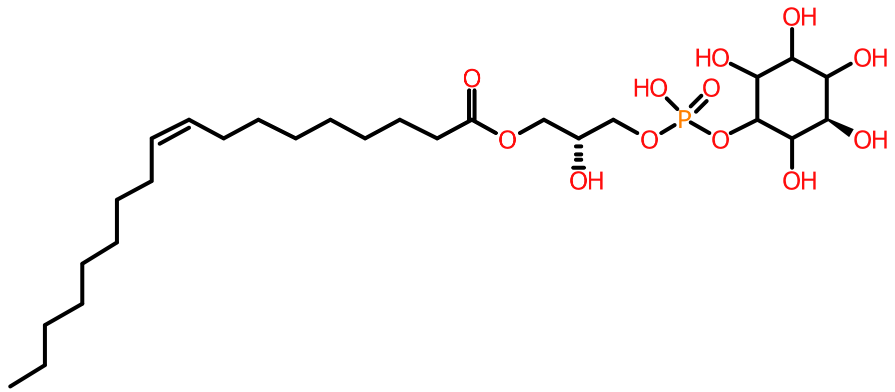

Compound information

Name: Spectral Match to

Exact mass: 598.312

Ion type: M-H

Precursor m/z: 597.3

Charge: 1

Polarity: Negative

InChI:

SMILES:

Instrument information

Instrument type: Ion Trap

Ion source: ESI

Ms level: 2

Acquisition: Isolated

Database links

CAS: 1114770158

Other information

Principal investigator: Data from Pieter

Data collector: Data deposited by lfnothias

Entry ID: CCMSLIB00003134872

LPI 18:1 0.934

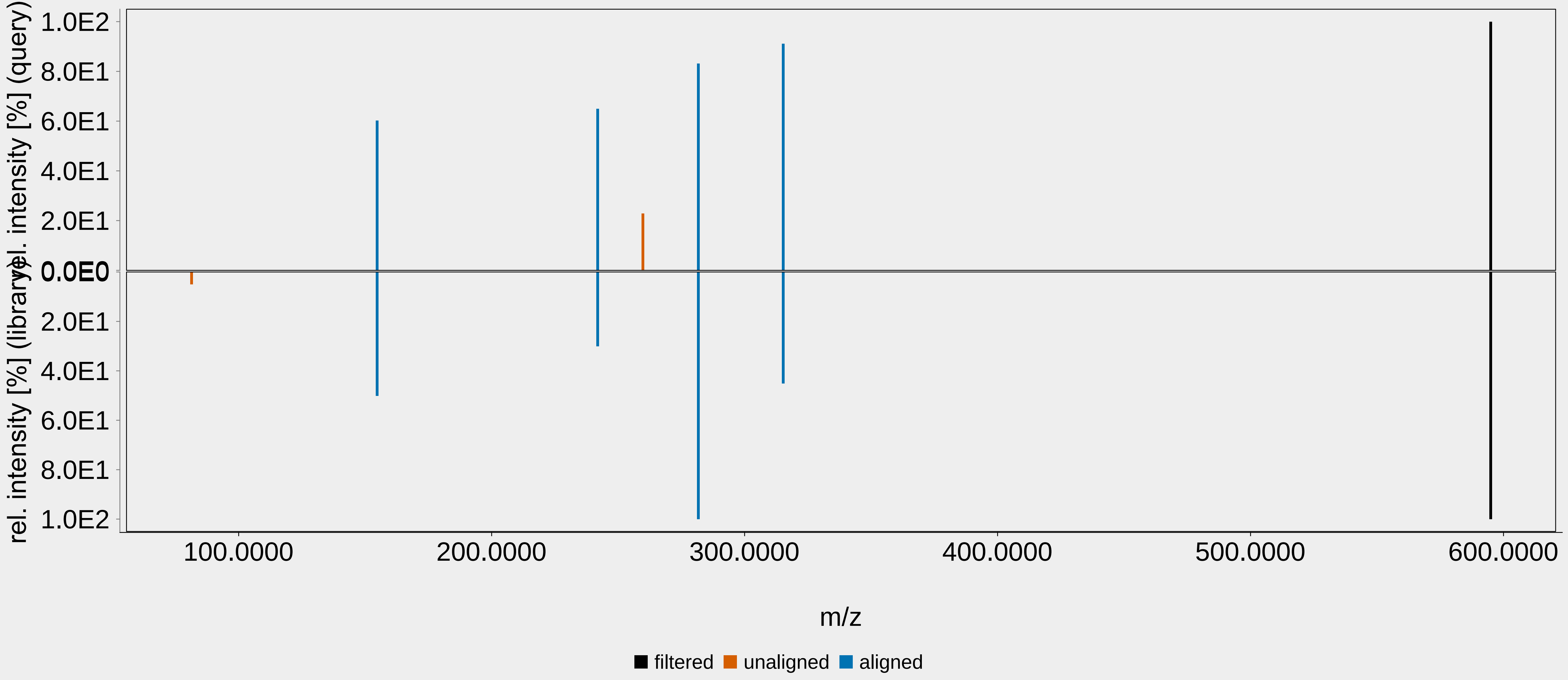

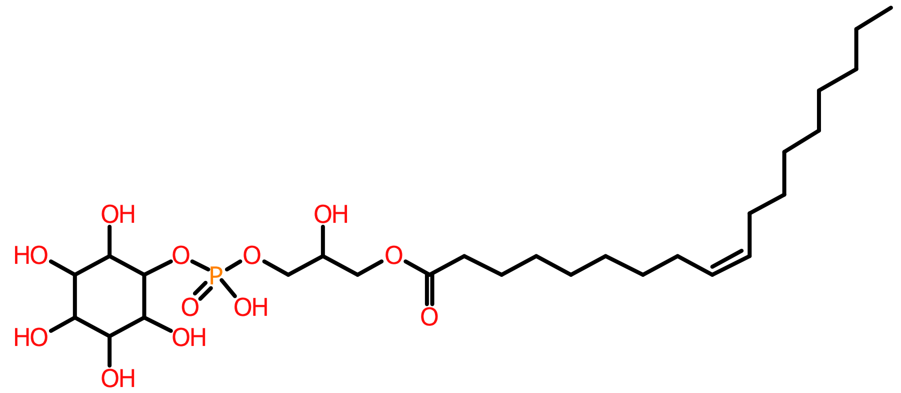

Compound information

Name: LPI 18:1

Formula: C27H51O12P

Ion type: [M-H]-

Precursor m/z: 597.30454

Ccs: 231.61

InChI key:

SMILES:

Num peaks: 6

Instrument information

Database links

Other information

PS 22:0_18:1 0.798

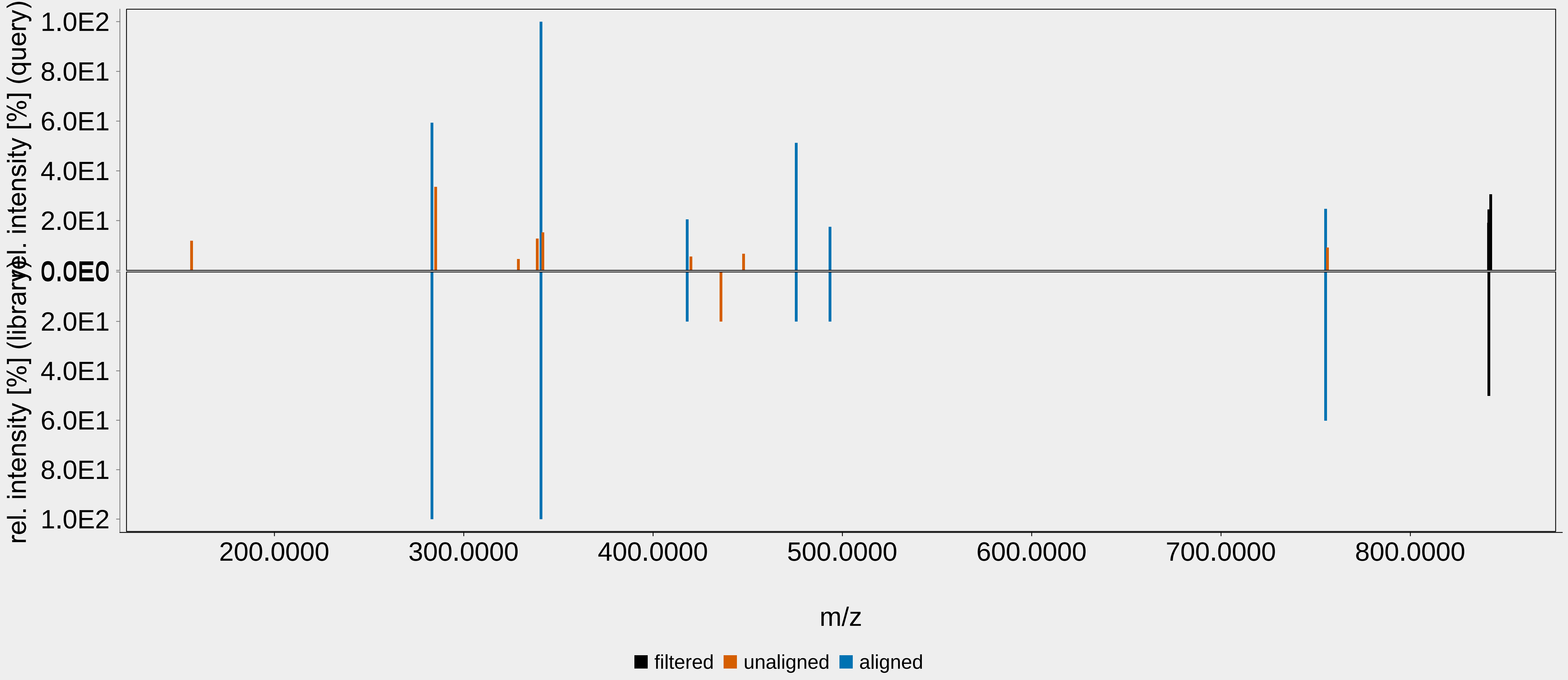

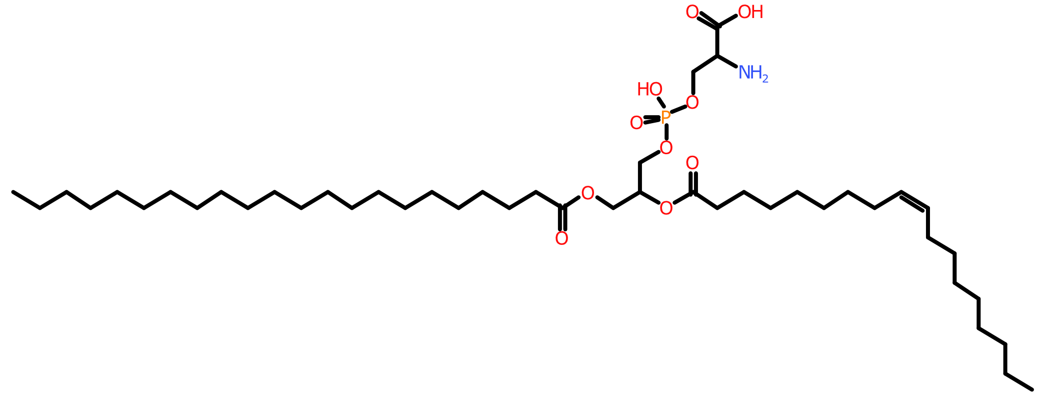

Compound information

Name: PS 22:0_18:1

Formula: C46H88NO10P

Ion type: [M-H]-

Precursor m/z: 844.60731

Ccs: 289.29

InChI key:

SMILES:

Num peaks: 8

Instrument information

Database links

Other information

PE(18:0/20:1); [M-H]- C43H83N1O8P1

0.893

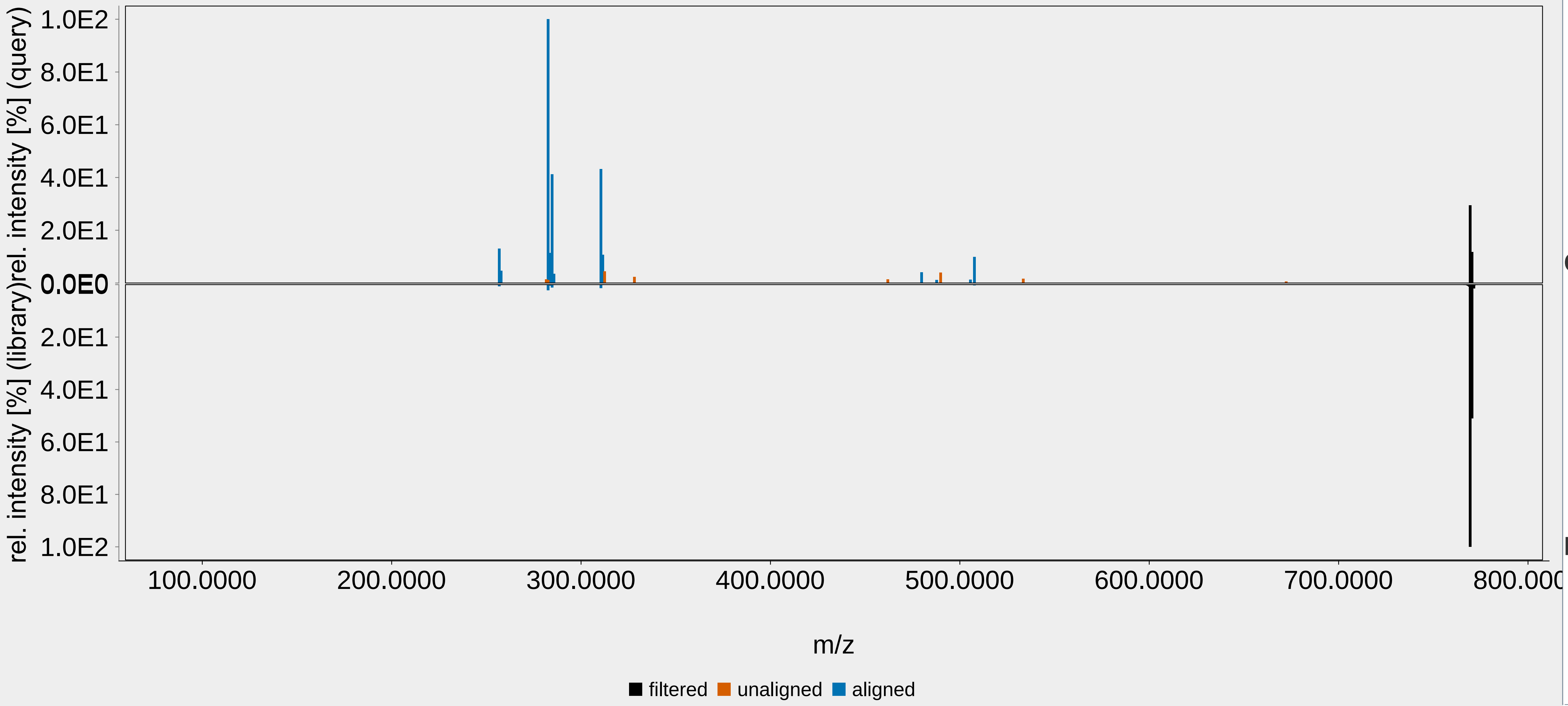

Compound information

Name: PE(18:0/20:1); [M-H]- C43H83N1O8P1

Exact mass: 0.0

Ion type: M-H

Precursor m/z: 772.586

Charge: 1

Polarity: Negative

Instrument information

Instrument type: HCD; Velos

Ion source: LC-ESI

Ms level: 2

Acquisition: Commercial

Database links

Other information

Principal investigator: Thomas Metz

Data collector: Thomas Metz

Entry ID: CCMSLIB00010068369

PE(18:0/20:1); [M-H]- C43H83N1O8P1 0.942

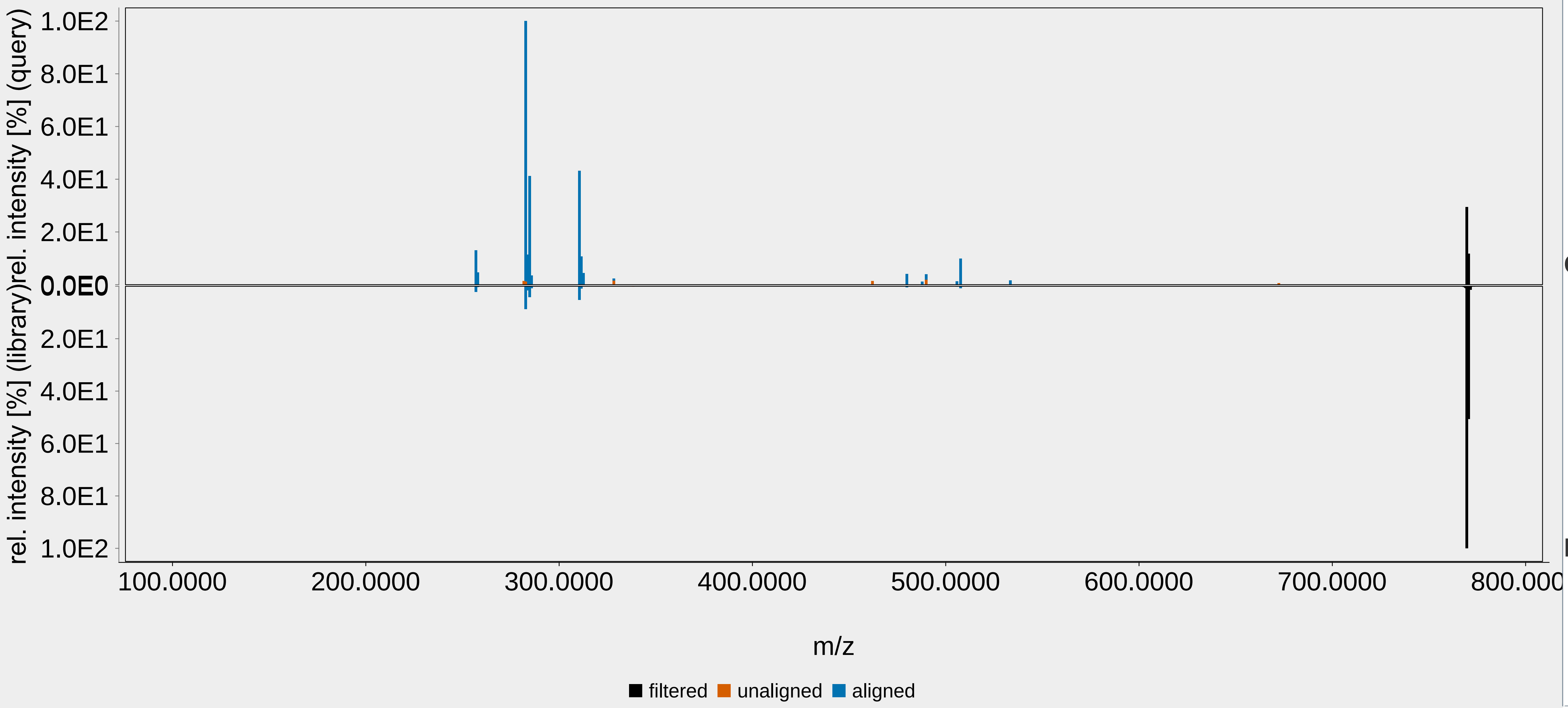

Compound information

Name: PE(18:0/20:1); [M-H]- C43H83N1O8P1

Exact mass: 0.0

Ion type: M-H

Precursor m/z: 772.586

Charge: 1

Polarity: Negative

Instrument information

Instrument type: HCD; Velos

Ion source: LC-ESI

Ms level: 2

Acquisition: Commercial

Database links

Other information

Principal investigator: Thomas Metz

Data collector: Thomas Metz

Entry ID: CCMSLIB00010068147

PI 38:5 0.825

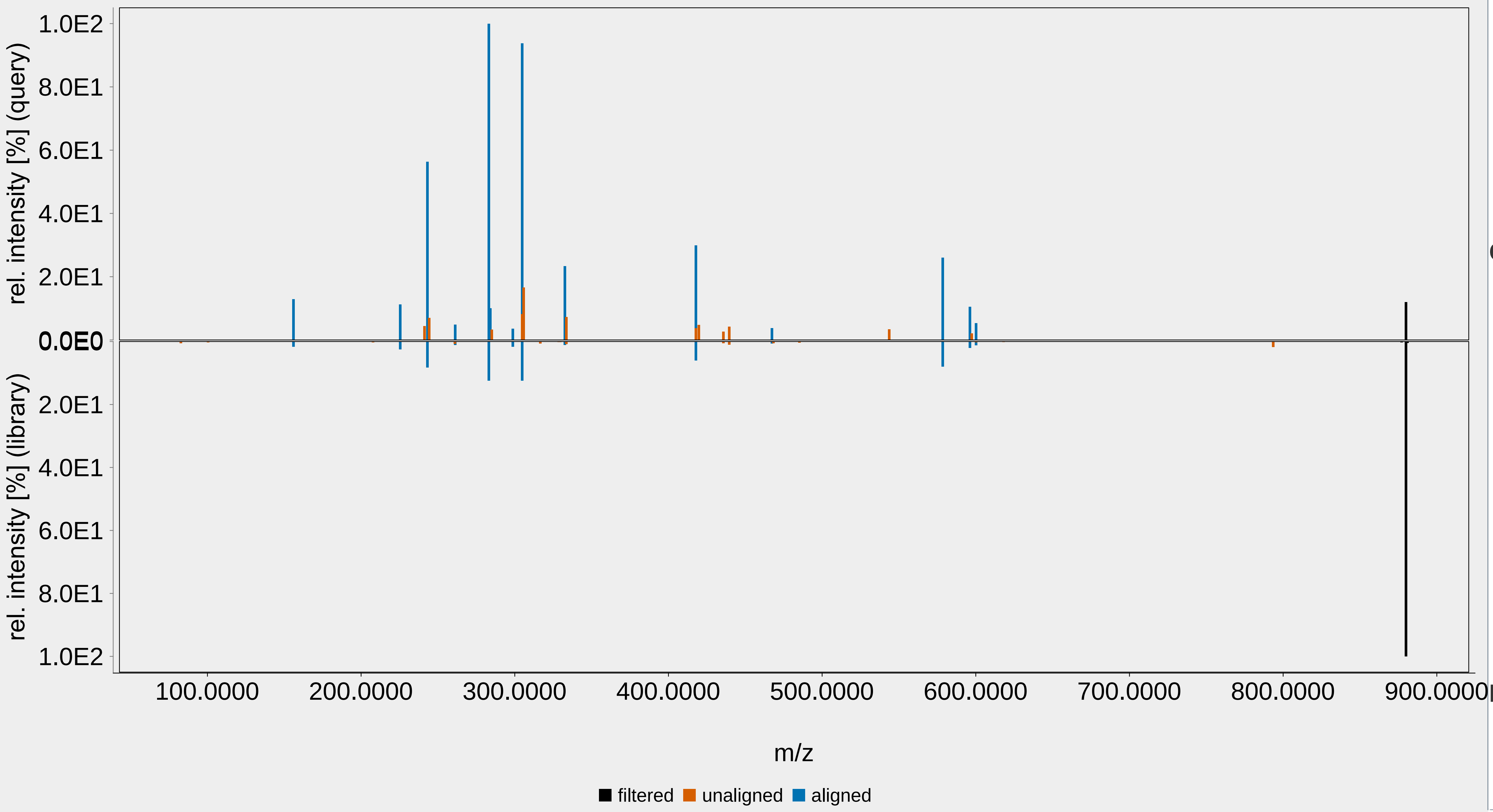

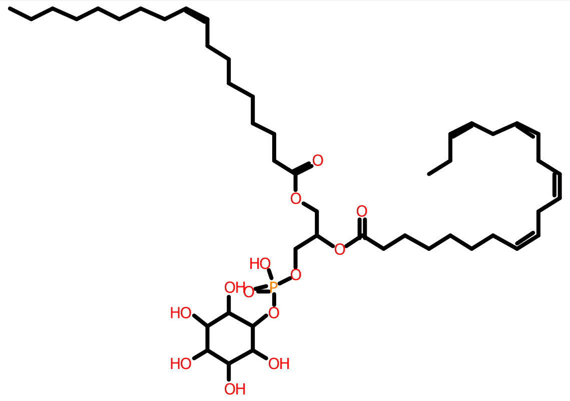

Compound information

Name: PI 38:5

Formula: C47H81O13P

Mol. weight: 885.1279999999999

Exact mass: 885.1279999999999

Ion type: [M-H]-

Precursor m/z: 883.532

RT: 8.353221

Polarity: negative

InChI:

InChI key:

SMILES:

Instrument information

Instrument type: LC-ESI-QTOF

Instrument: AB Sciex TripleTOF 5600+ system

Ms level: MS2

Collision energy: 45V

Database links

Pubchem: 138296016

MoNA ID: LQB00702

Other information

Principal investigator: Kazutaka Ikeda, Hiroshi

Data collector: Kazutaka Ikeda, Hiroshi

PI 38:5 0.825

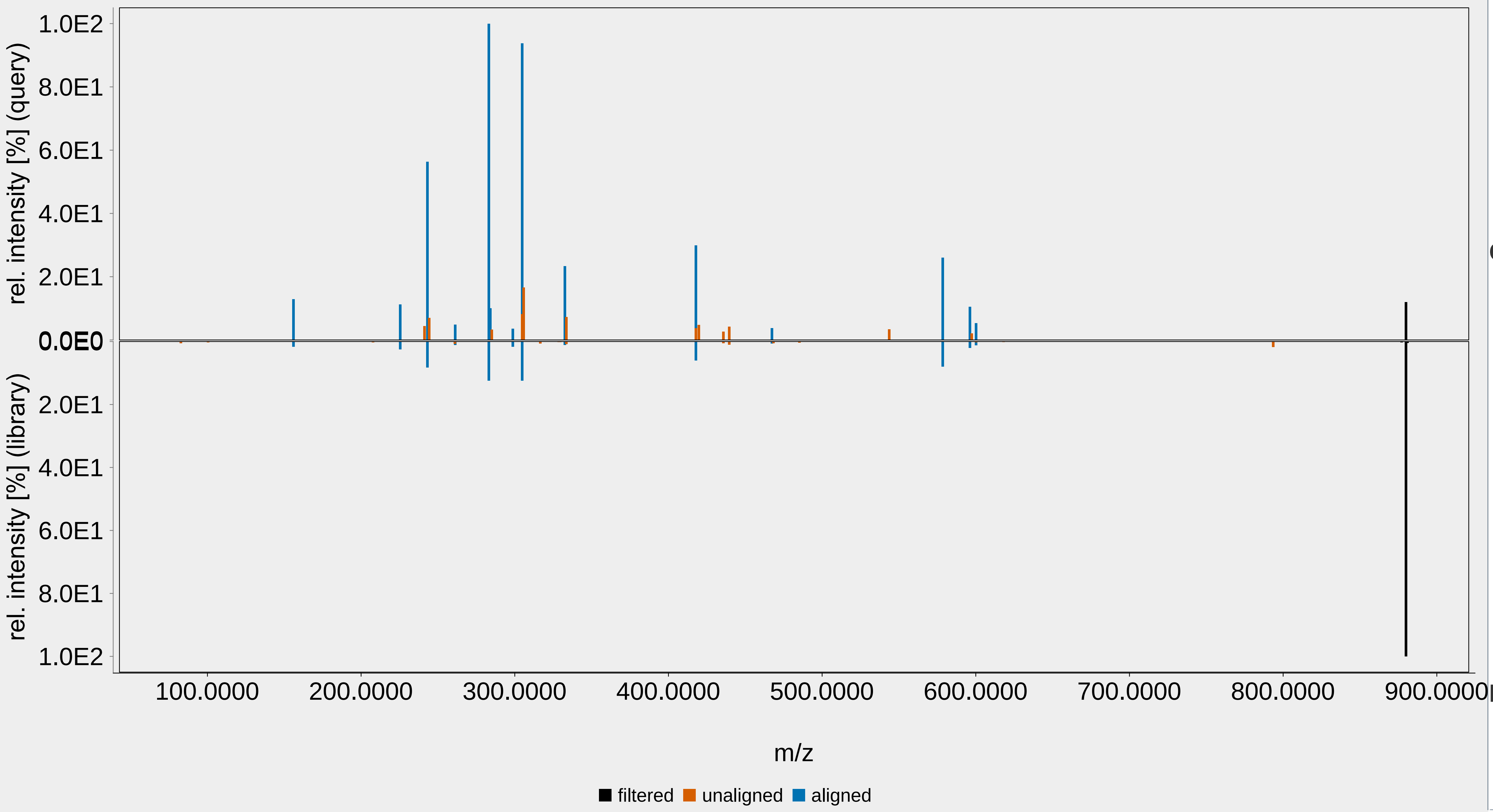

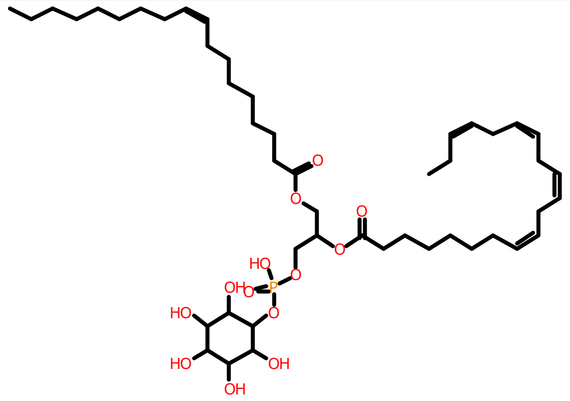

Compound information

Name: PI 38:5

Formula: C47H81O13P

Mol. weight: 885.1279999999999

Exact mass: 885.1279999999999

Ion type: [M-H]-

Precursor m/z: 883.532

RT: 8.353221

Polarity: negative

InChI:

InChI key:

SMILES:

Instrument information

Instrument type: LC-ESI-QTOF

Instrument: AB Sciex TripleTOF 5600+ system

Ms level: MS2

Collision energy: 45V

Database links

Pubchem: 138296016

MoNA ID: LQB00316

Other information

Principal investigator: Kazutaka Ikeda, Hiroshi

Data collector: Kazutaka Ikeda, Hiroshi

PI(18:0_0:0) - [2-hydroxy-3-[hydroxy-[2,3,4,5,6-pentahydroxycyclohexyl]oxyphosphoryl]oxypropyl] octadecanoate

0.778

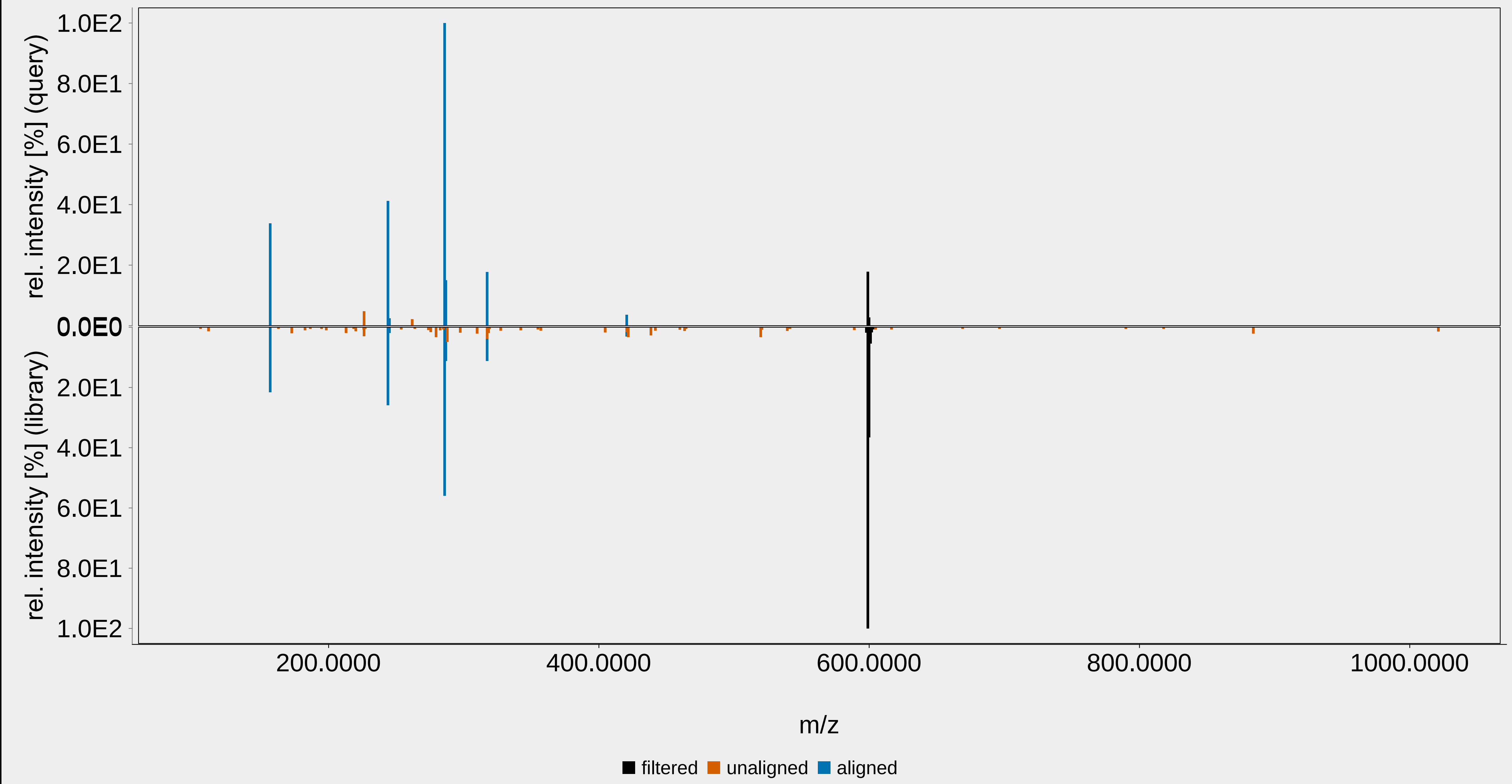

Compound information

Name: PI(18:0_0:0) -

Exact mass: 600.327

Ion type: M-H

Precursor m/z: 599.32

Charge: 1

Polarity: Negative

InChI:

InChI key:

SMILES:

Instrument information

Instrument type: qTof

Ion source: LC-ESI

Ms level: 2

Acquisition: Commercial

Database links

Pubmed:

CAS: 849412-49-3

Other information

Principal investigator: O Laprevote

Data collector: UMR 8038 Universite de Paris

Entry ID: CCMSLIB00005436451

LPI 18:0 **0.948**

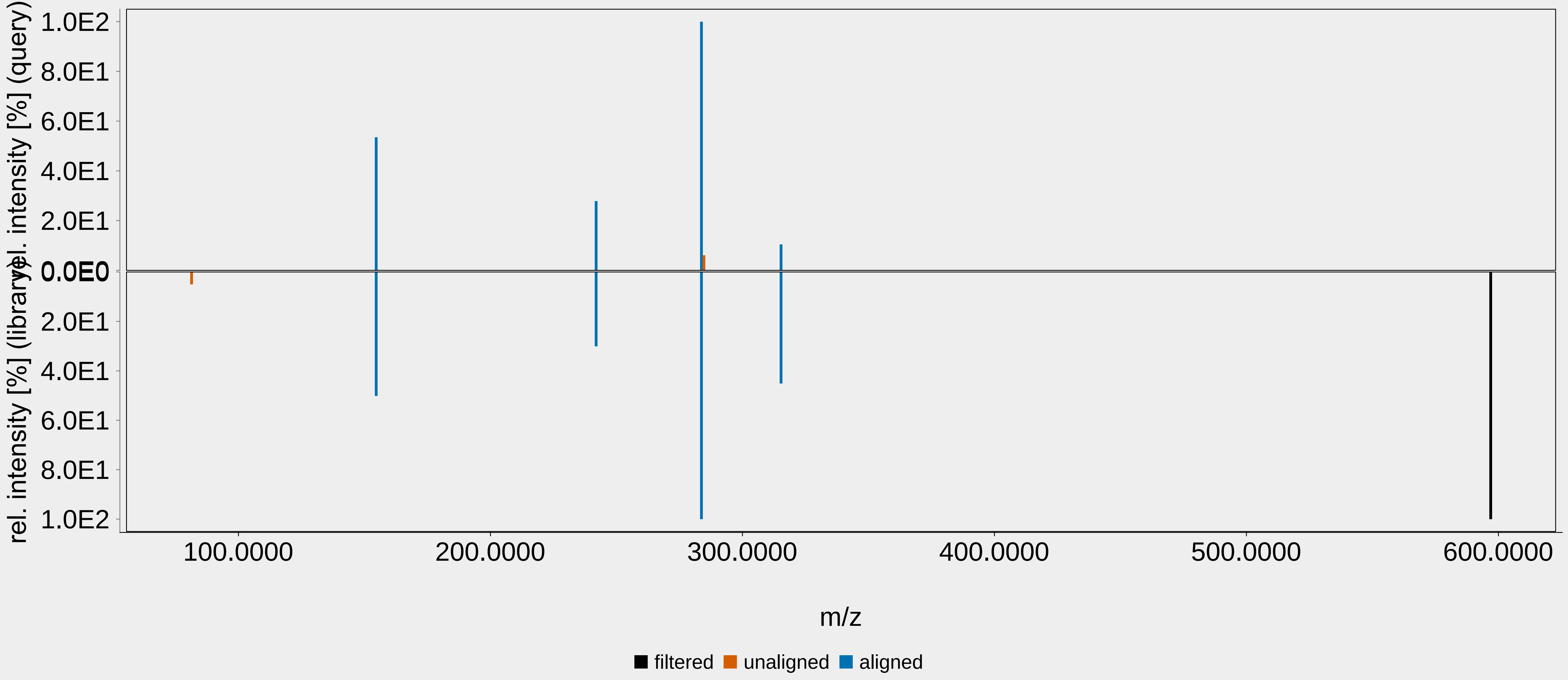

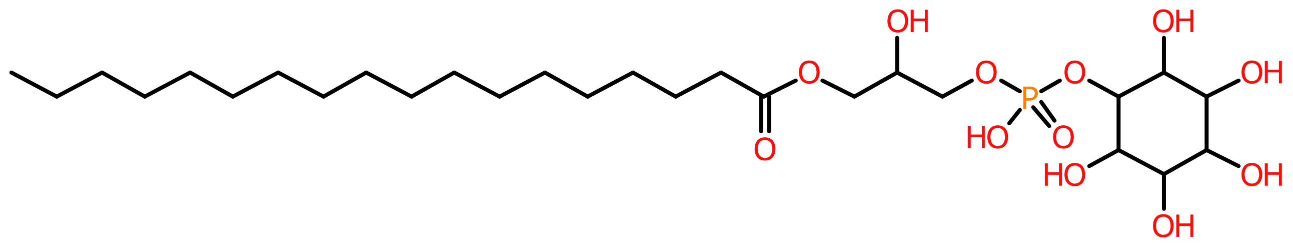

Compound information

Name: LPI 18:0

Formula: C27H53O12P

Ion type: [M-H]-

Precursor m/z: 599.32019

Ccs: 233.47

InChI key: MXAFDFDAIFZFET-UHFFFAOYNA-

SMILES:

Num peaks: 6

Instrument information

Database links

Other information

PE(18:0/18:1); [M-H]- C41H79N1O8P1 0.920

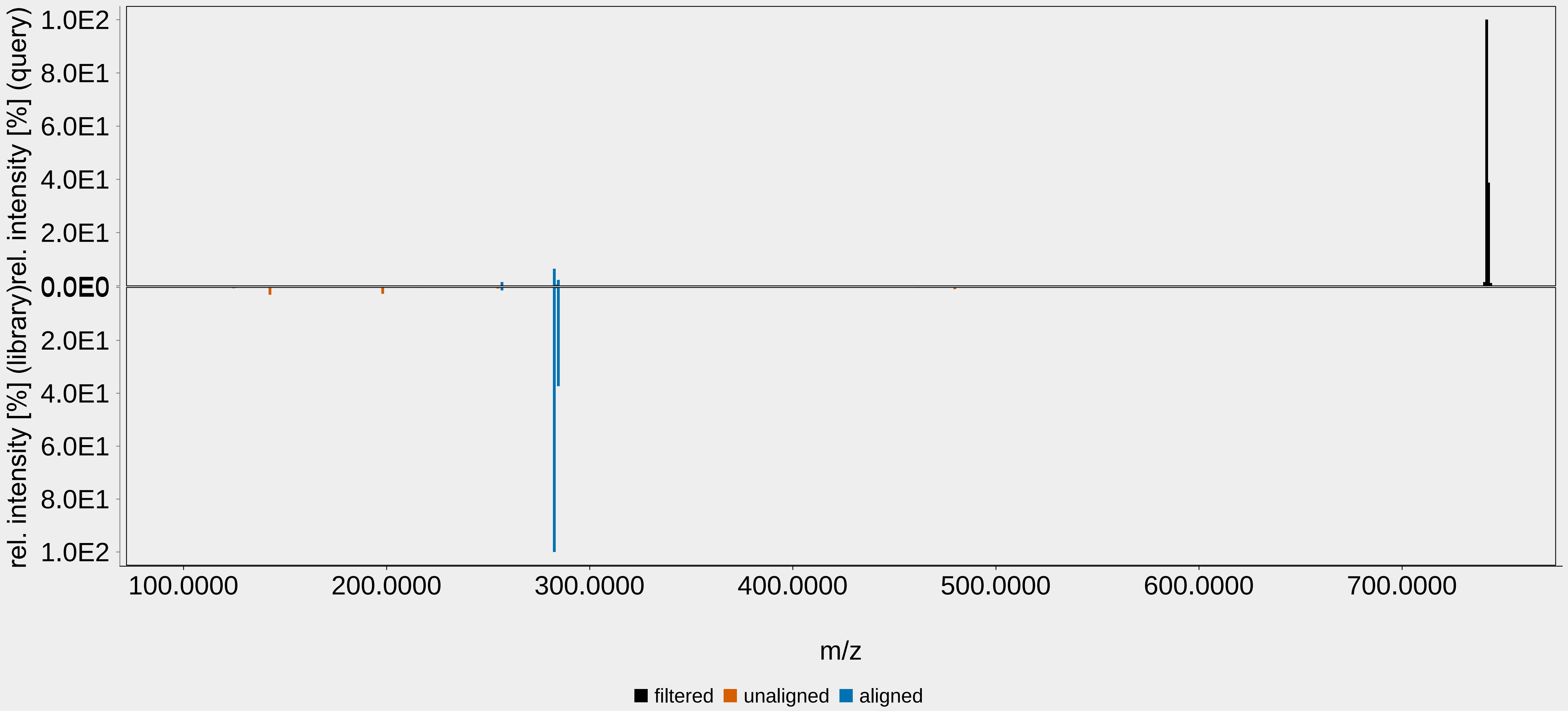

Compound information

Name: PE(18:0/18:1); [M-H]- C41H79N1O8P1

Exact mass: 0.0

Ion type: M-H

Precursor m/z: 744.554

Charge: 1

Polarity: Negative

Instrument information

Instrument type: HCD; Lumos

Ion source: LC-ESI

Ms level: 2

Acquisition: Commercial

Database links

Other information

Principal investigator: Thomas Metz

Data collector: Thomas Metz

Entry ID: CCMSLIB00010059575

PE(18:0/18:1); [M-H]- C41H79N1O8P1

0.930

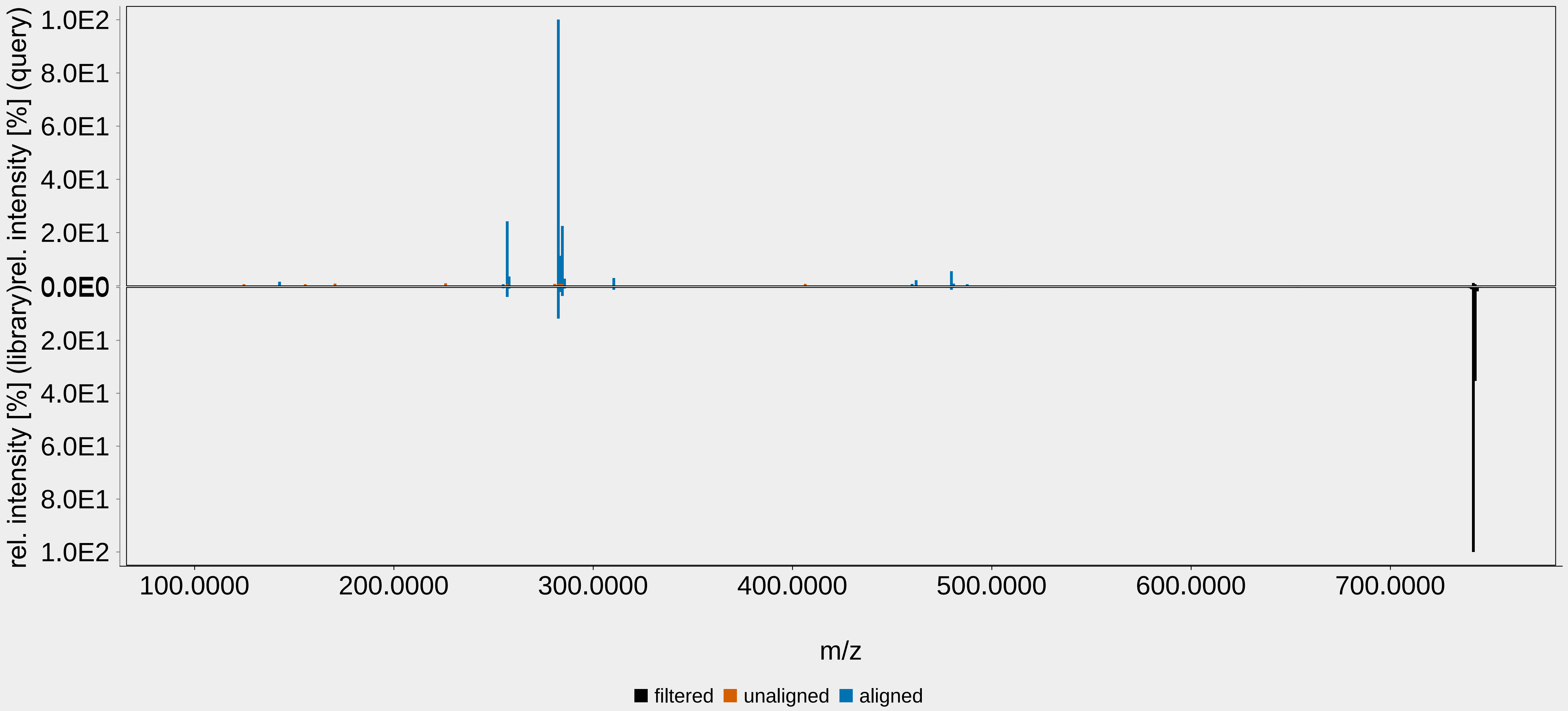

Compound information

Name: PE(18:0/18:1); [M-H]- C41H79N1O8P1

Exact mass: 0.0

Ion type: M-H

Precursor m/z: 744.554

Charge: 1

Polarity: Negative

Instrument information

Instrument type: HCD; Velos

Ion source: LC-ESI

Ms level: 2

Acquisition: Commercial

Database links

Other information

Principal investigator: Thomas Metz

Data collector: Thomas Metz

Entry ID: CCMSLIB00010072259

PE(18:1/22:6); [M-H]- C45H75N1O8P1 **0.910**

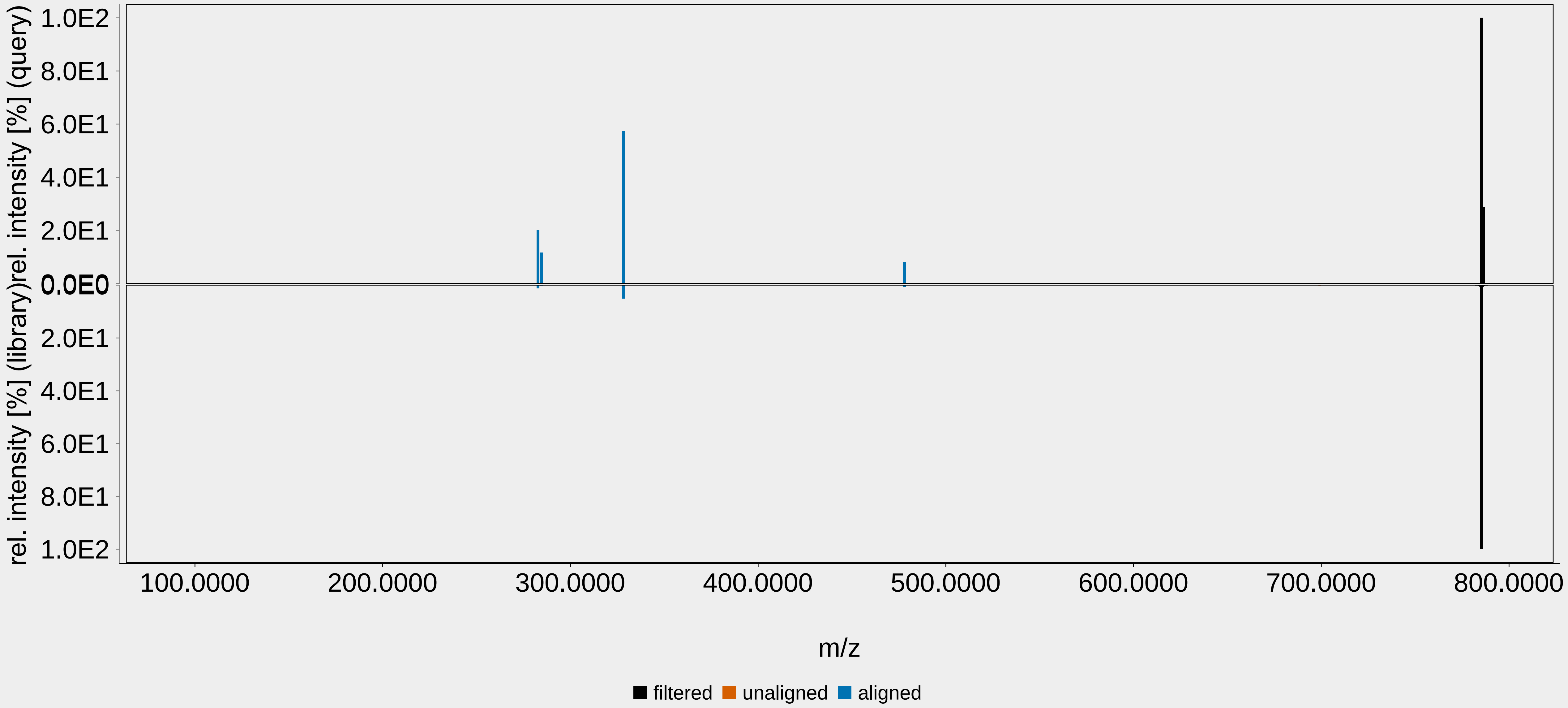

Compound information

Name: PE(18:1/22:6); [M-H]- C45H75N1O8P1

Exact mass: 0.0

Ion type: M-H

Precursor m/z: 788.523

Charge: 1

Polarity: Negative

Instrument information

Instrument type: HCD; Velos

Ion source: LC-ESI

Ms level: 2

Acquisition: Commercial

Database links

Other information

Principal investigator: Thomas Metz

Data collector: Thomas Metz

Entry ID: CCMSLIB00010063779

PE(18:1/22:6); [M-H]- C45H75N1O8P1 0.923

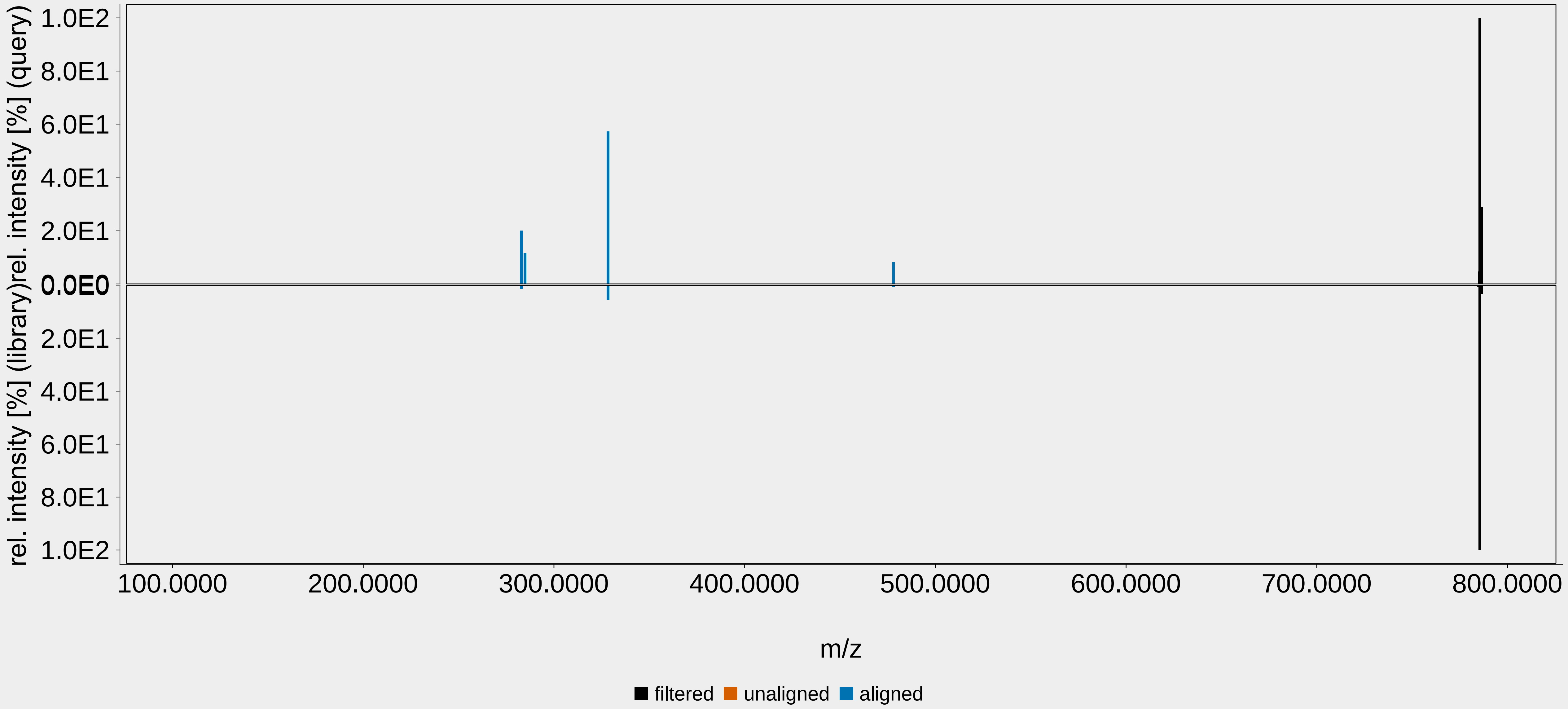

Compound information

Name: PE(18:1/22:6); [M-H]- C45H75N1O8P1

Exact mass: 0.0

Ion type: M-H

Precursor m/z: 788.523

Charge: 1

Polarity: Negative

Instrument information

Instrument type: HCD; Velos

Ion source: LC-ESI

Ms level: 2

Acquisition: Commercial

Database links

Other information

Principal investigator: Thomas Metz

Data collector: Thomas Metz

Entry ID: CCMSLIB00010073275

Adenosine monophosphate - 30.0 eV

0.806

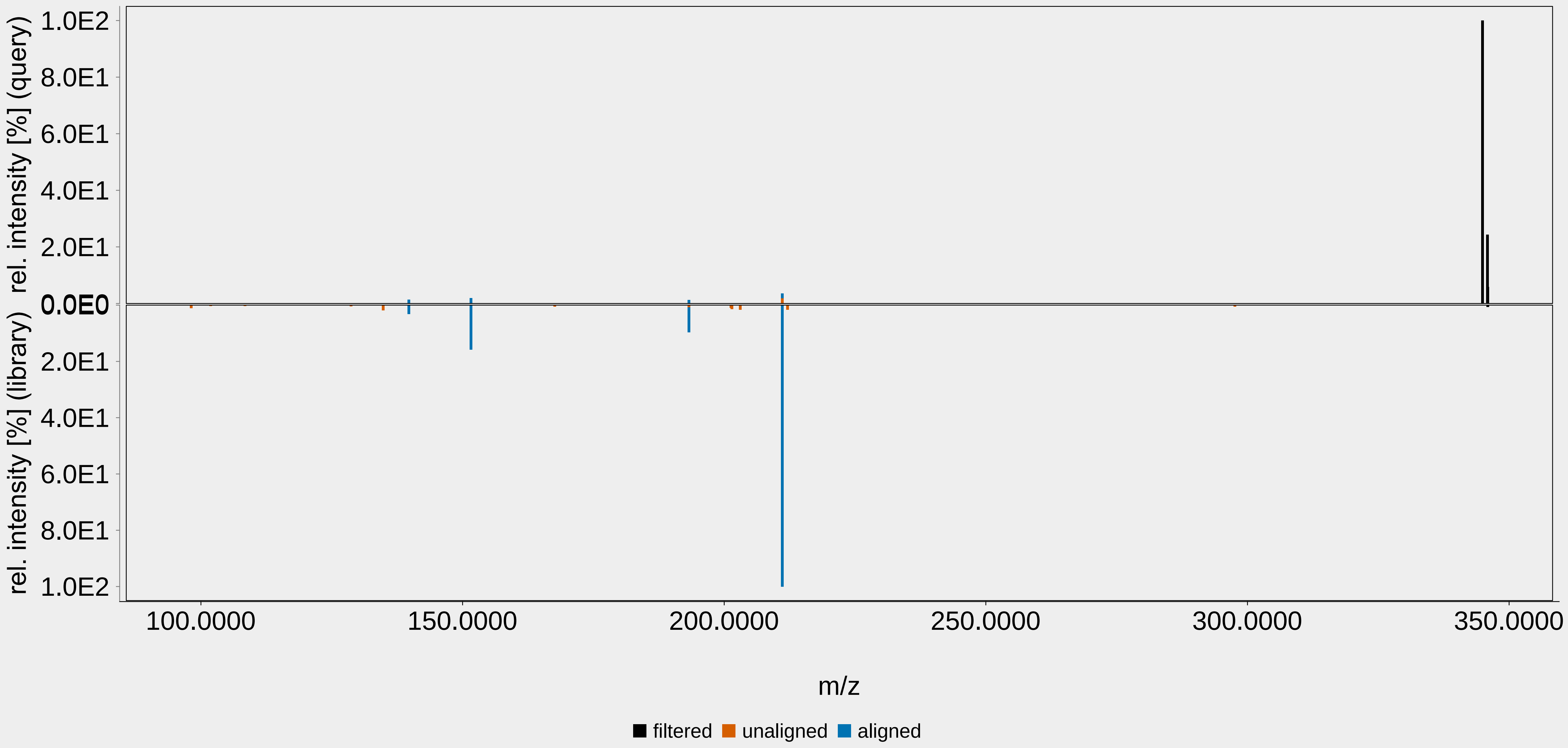

Compound information

Name: Adenosine monophosphate - 30.0 eV

Exact mass: 0.0

Ion type: M-H

Precursor m/z: 346.056

Charge: 1

Polarity: Negative

SMILES:

Instrument information

Instrument type: Orbitrap

Ion source: ESI

Ms level: 2

Acquisition: Isolated

Database links

CAS:

Other information

Principal investigator: PI

Data collector: Data Collector

Entry ID: CCMSLIB00006114160

Adenosine monophosphate - 30.0 eV

0.800

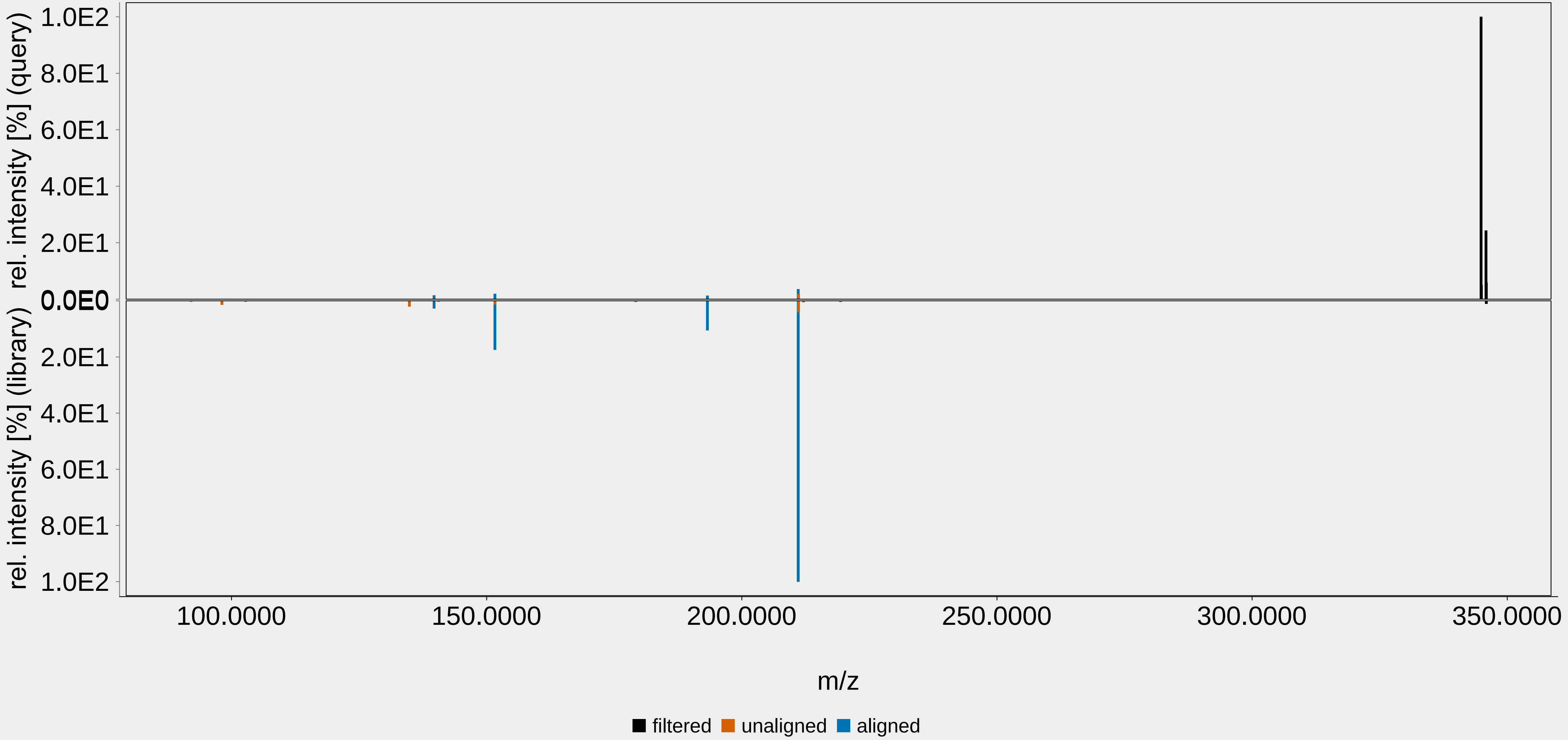

Compound information

Name: Adenosine monophosphate - 30.0 eV

Exact mass: 0.0

Ion type: M-H

Precursor m/z: 346.056

Charge: 1

Polarity: Negative

SMILES:

Instrument information

Instrument type: Orbitrap

Ion source: ESI

Ms level: 2

Acquisition: Isolated

Database links

CAS:

Other information

Principal investigator: PI

Data collector: Data Collector

Entry ID: CCMSLIB00006114141

[5-acetyloxy-3-(hydroxymethyl)-2-oxo-6-propan-2-ylcyclohex-3-en-1-yl] 3-methylbutanoate

0.793

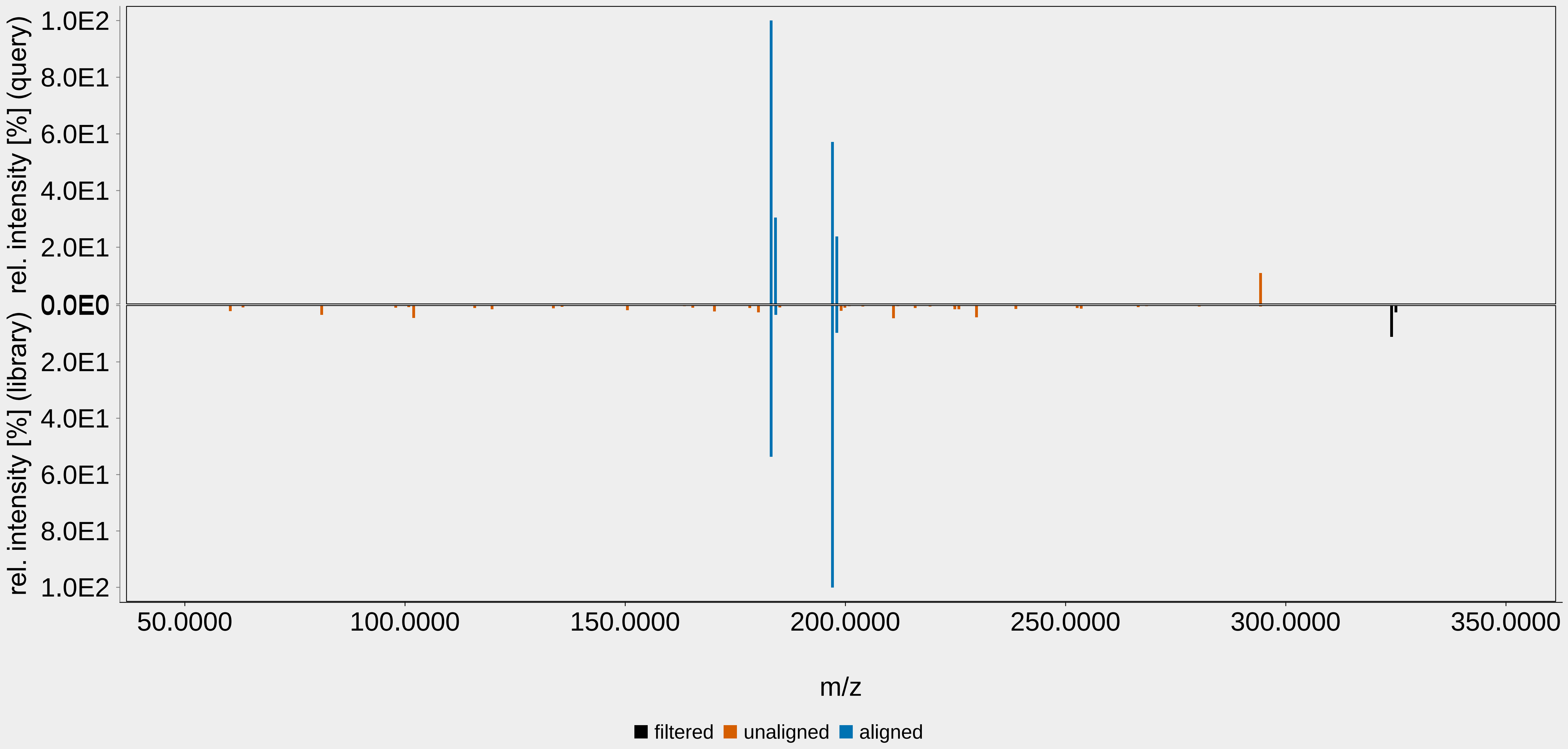

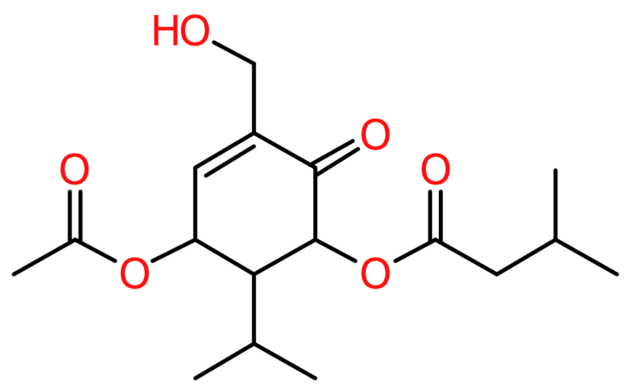

Compound information

Name:

Formula: C17H26O6

Ion type: [M-H]-

Precursor m/z: 325.1656494140625

Polarity: negative

InChI:

InChI key: IVEJMWCGEFICJC-UHFFFAOYSA-

SMILES:

Instrument information

Instrument type: ESI-QFT

Instrument: Thermo Q Exactive HF

Ms level: MS2

Collision energy: 65HCD

Database links

Other information

Principal investigator: Arpana Vaniya

Data collector: Arpana Vaniya

[5-acetyloxy-3-(hydroxymethyl)-2-oxo-6-propan-2-ylcyclohex-3-en-1-yl] 3-methylbutanoate

0.793

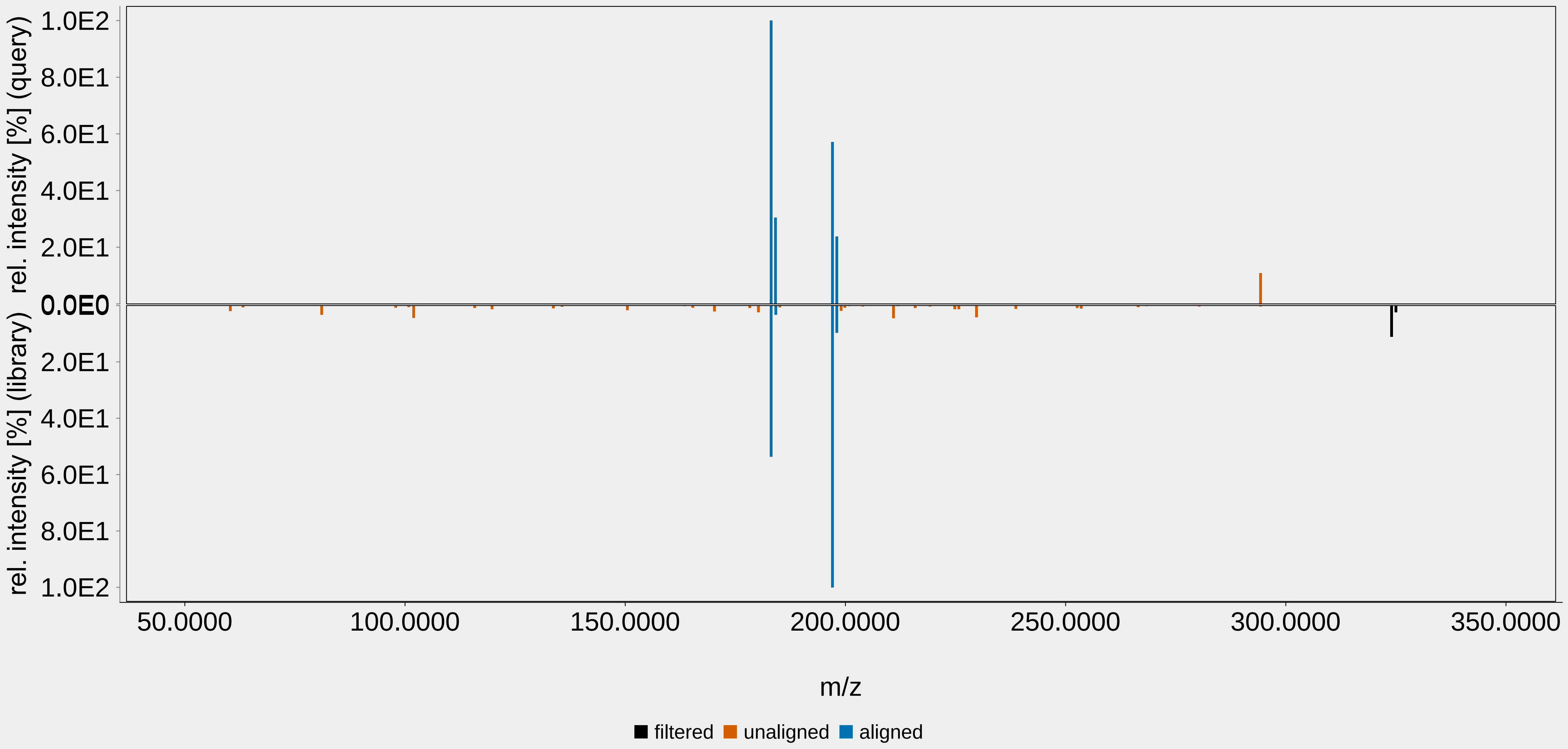

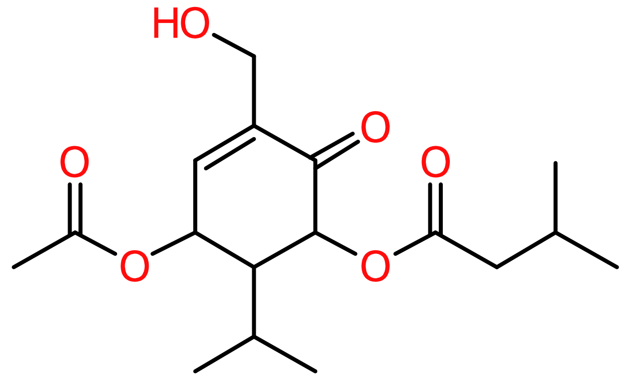

Compound information

Name:

Exact mass: 0.0

Ion type: [M-H]-

Precursor m/z: 325.166

Charge: 1

Polarity: negative

InChI:

Instrument information

Instrument type: ESI-QFT

Ms level: 2

Acquisition: isolated

Database links

Other information

Principal investigator: MoNA

Data collector: MoNA:VF-NPL-QEHF008244

Entry ID: CCMSLIB00004699307

PE(18:1/0:0); [M-H]- C23H45N1O7P1 0.949

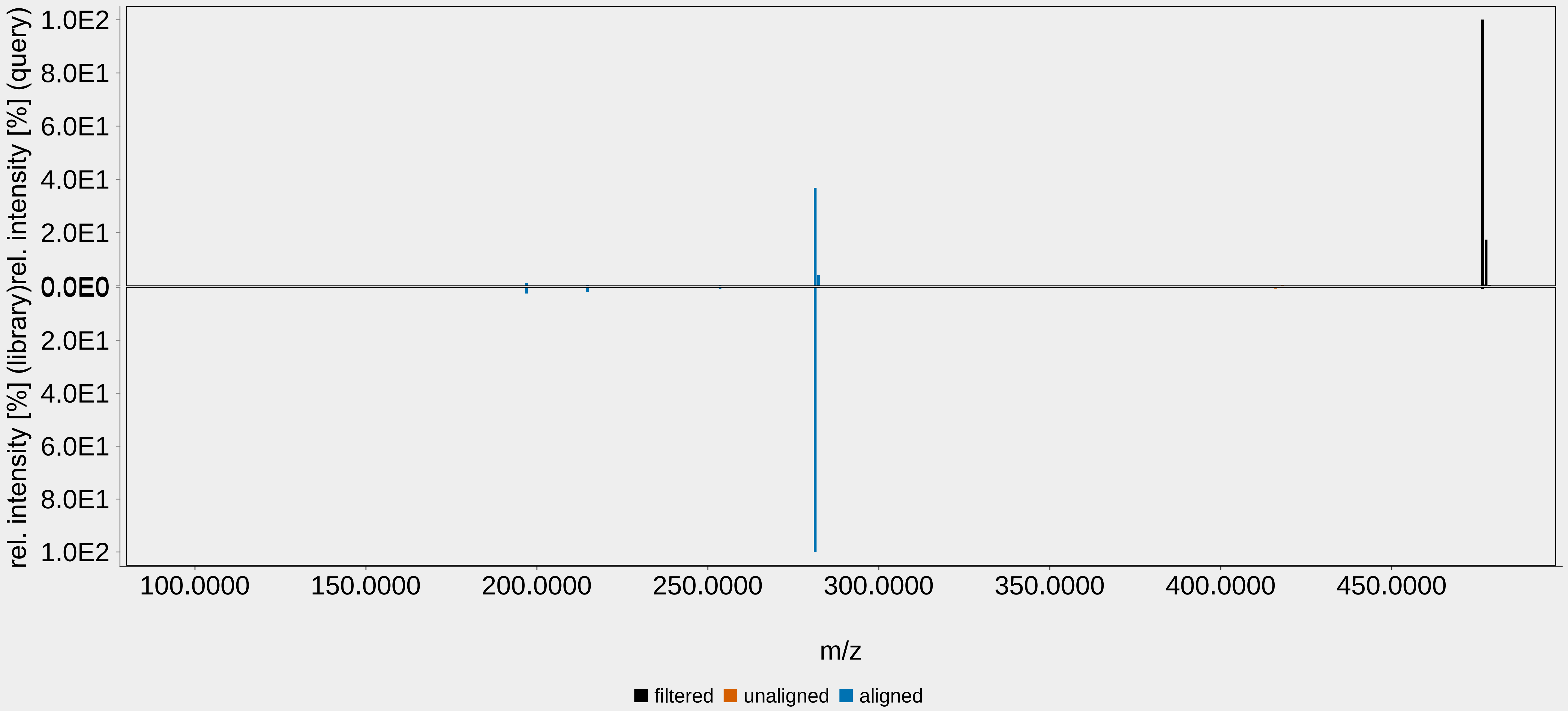

Compound information

Name: PE(18:1/0:0); [M-H]- C23H45N1O7P1

Exact mass: 0.0

Ion type: M-H

Precursor m/z: 478.293

Charge: 1

Polarity: Negative

Instrument information

Instrument type: CID; Lumos

Ion source: LC-ESI

Ms level: 2

Acquisition: Commercial

Database links

Other information

Principal investigator: Thomas Metz

Data collector: Thomas Metz

Entry ID: CCMSLIB00010059278

PE(18:1/0:0); [M-H]- C23H45N1O7P1

0.950

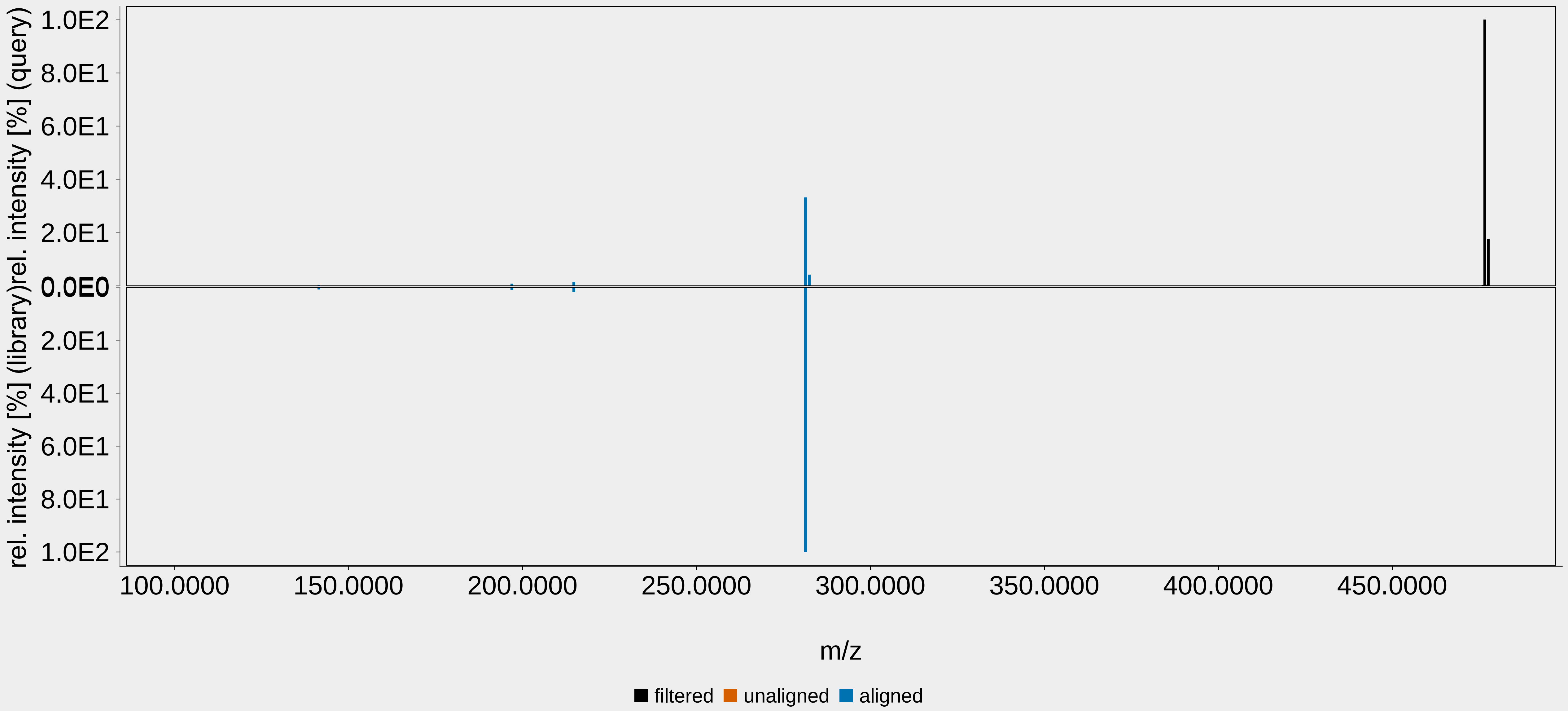

Compound information

Name: PE(18:1/0:0); [M-H]- C23H45N1O7P1

Exact mass: 0.0

Ion type: M-H

Precursor m/z: 478.293

Charge: 1

Polarity: Negative

Instrument information

Instrument type: HCD; Lumos

Ion source: LC-ESI

Ms level: 2

Acquisition: Commercial

Database links

Other information

Principal investigator: Thomas Metz

Data collector: Thomas Metz

Entry ID: CCMSLIB00010059197

C10-LAS (TENTATIVE)

0.899

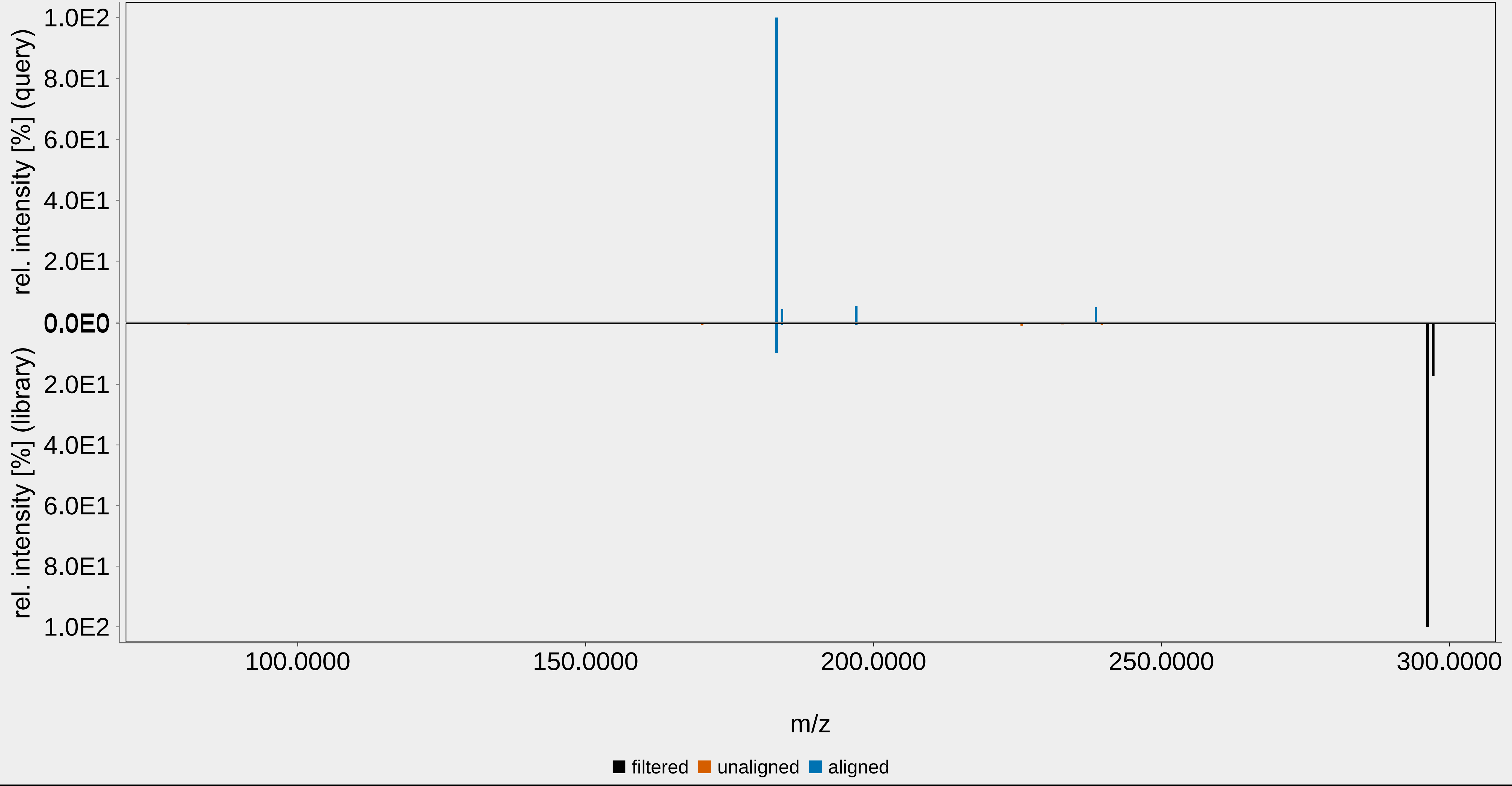

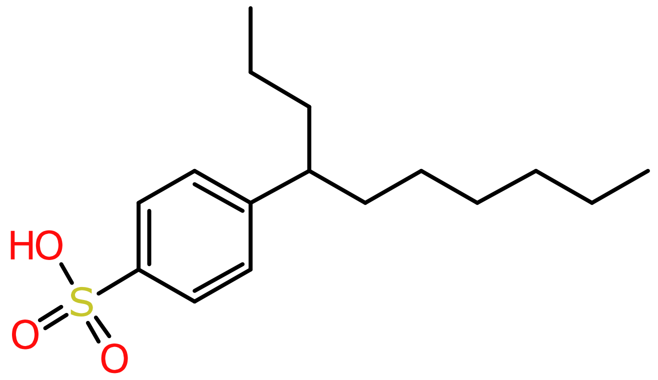

Compound information

Name: C10-LAS (TENTATIVE)

Formula: C16H26O3S

Exact mass: 298.1603

Ion type: [M-H]-

Precursor m/z: 297.1521

Polarity: NEGATIVE

InChI:

InChI key: NANHIUZYPFDGJS-UHFFFAOYSA-

SMILES:

Num peaks: 20

Instrument information

Instrument type: LC-ESI-ITFT

Instrument: LTQ Orbitrap XL Thermo Scientific

Ms level: MS2

Collision energy: 60 NCE

Database links

Other information

Entry ID:

Comment: Parent=297.1521

C10-LAS (TENTATIVE), C10-linear alkylbenzyl sulfonate, 4-(decan-4-yl)benzenesulfonic acid

0.900

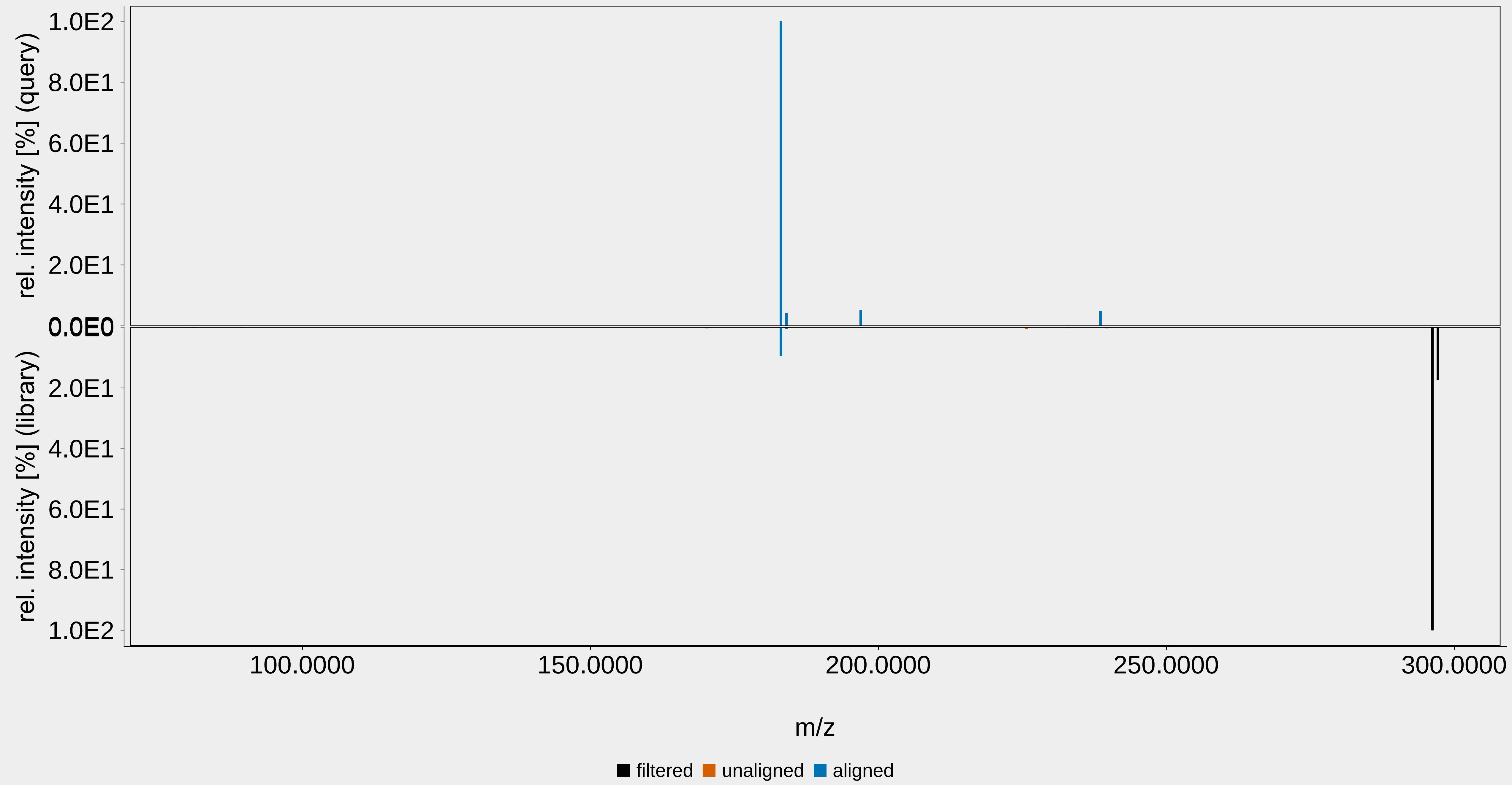

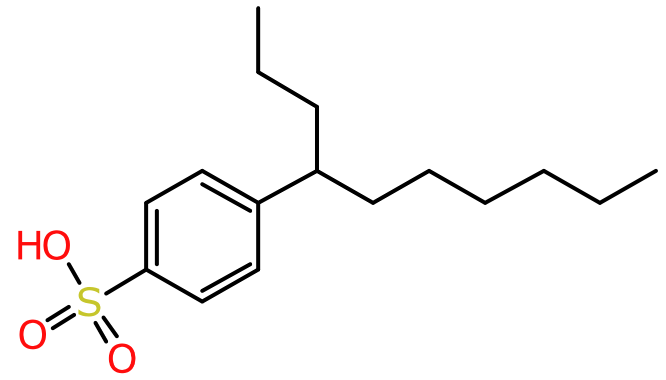

Compound information

Name: C10-LAS (TENTATIVE), C10-linear

Formula: C16H26O3S

Mol. weight: 298.1603

Exact mass: 298.1603

Ion type: [M-H]-

Precursor m/z: 297.1521

Polarity: negative

InChI:

InChI key: NANHIUZYPFDGJS-UHFFFAOYSA-

SMILES:

Instrument information

Instrument type: LC-ESI-ITFT

Instrument: LTQ Orbitrap XL Thermo Scientific

Ion source: ESI

Ms level: MS2

Collision energy: 60 NCE

Database links

Pubchem: 14921167

MoNA ID: ETS00001

Other information

Principal investigator: E. Schymanski, Dept. of

Data collector: E. Schymanski, Dept. of

Crocetin semialdehyde

0.739

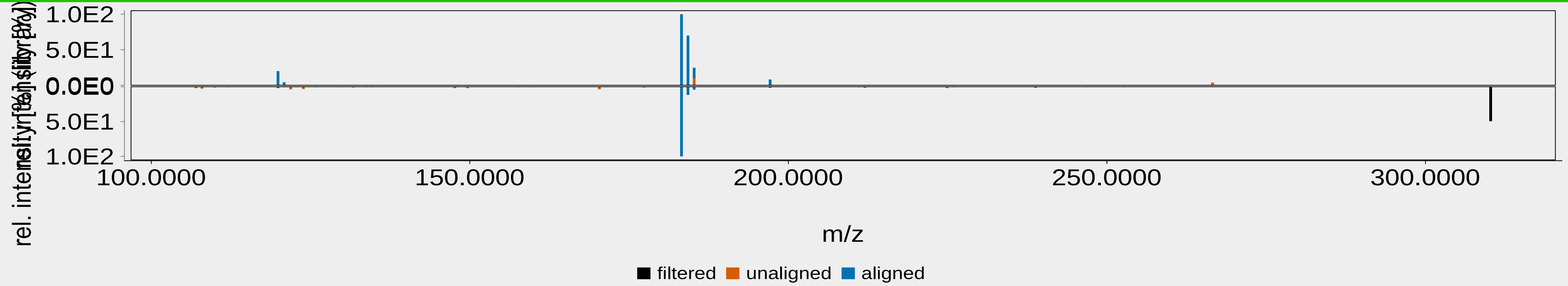

Compound information

Name: Crocetin semialdehyde

Ion type: carotenoid

Precursor m/z: 312.1725

Polarity: negative

Instrument information

Ion source: Electrospray Ionization (ESI)

Ms level: MS2

Database links

Other information

Canrenone

0.912

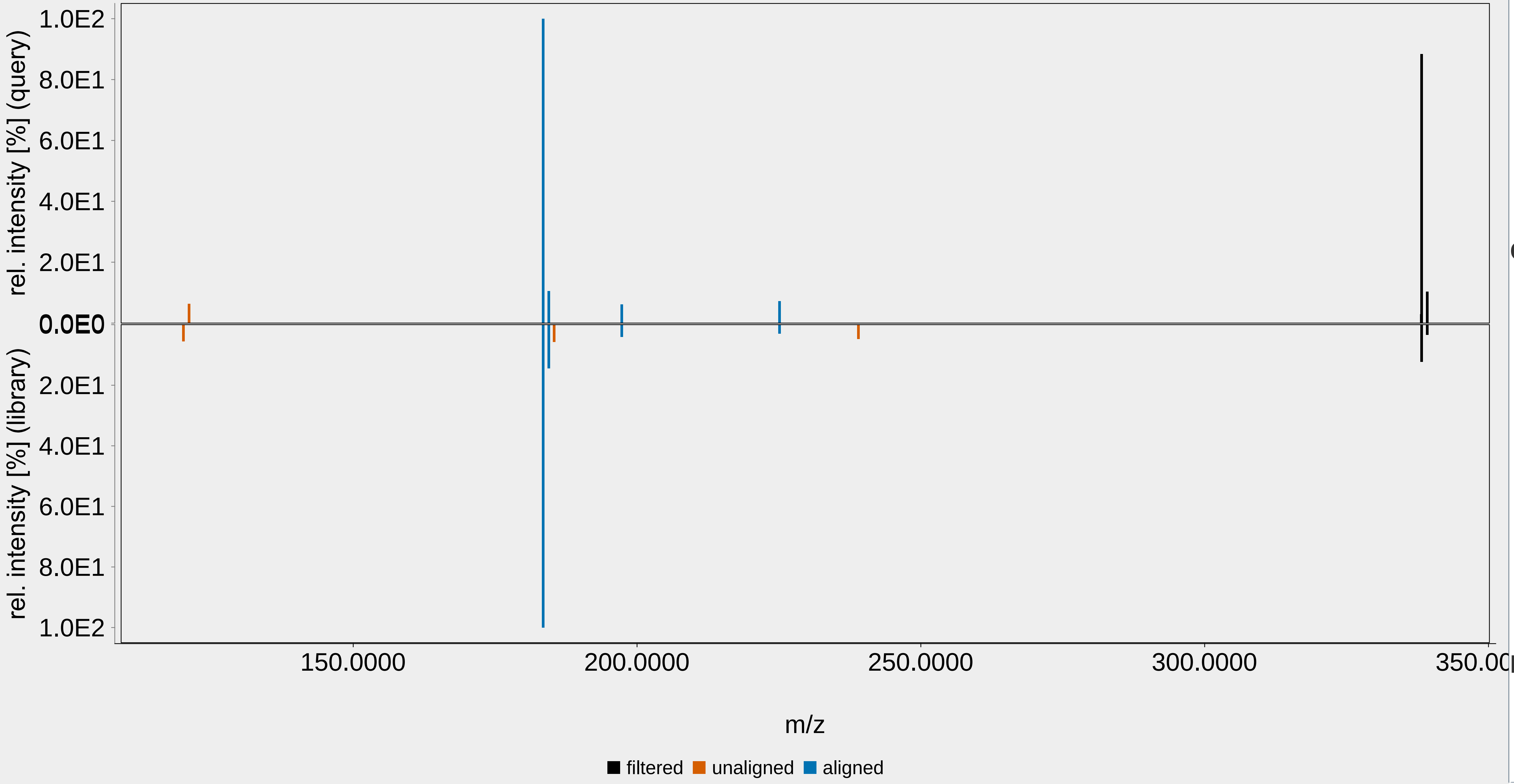

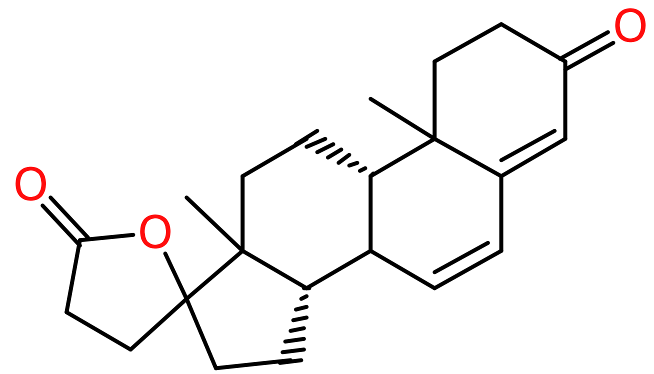

Compound information

Name: Canrenone

Formula: C22H28O3

Exact mass: 340.2038448

Ion type: [M-H]-

Precursor m/z: 339.1966

Polarity: NEGATIVE

InChI:

InChI key:

SMILES:

Num peaks: 9

Instrument information

Instrument type: LC-ESI-QTOF

Instrument: Bruker maXis Impact

Ms level: MS2

Collision energy: 40 eV

Database links

Other information

Entry ID: MSBNK-Athens_Univ-AU239460

Comment: Parent=339.1966

Canrenone, (9S,14S)-10,13-dimethylspiro[2,8,9,11,12,14,15,16-octahydro-1H-cyclopenta[a]phenanthrene-17,5`-oxolane]-2`,3-dione

0.912

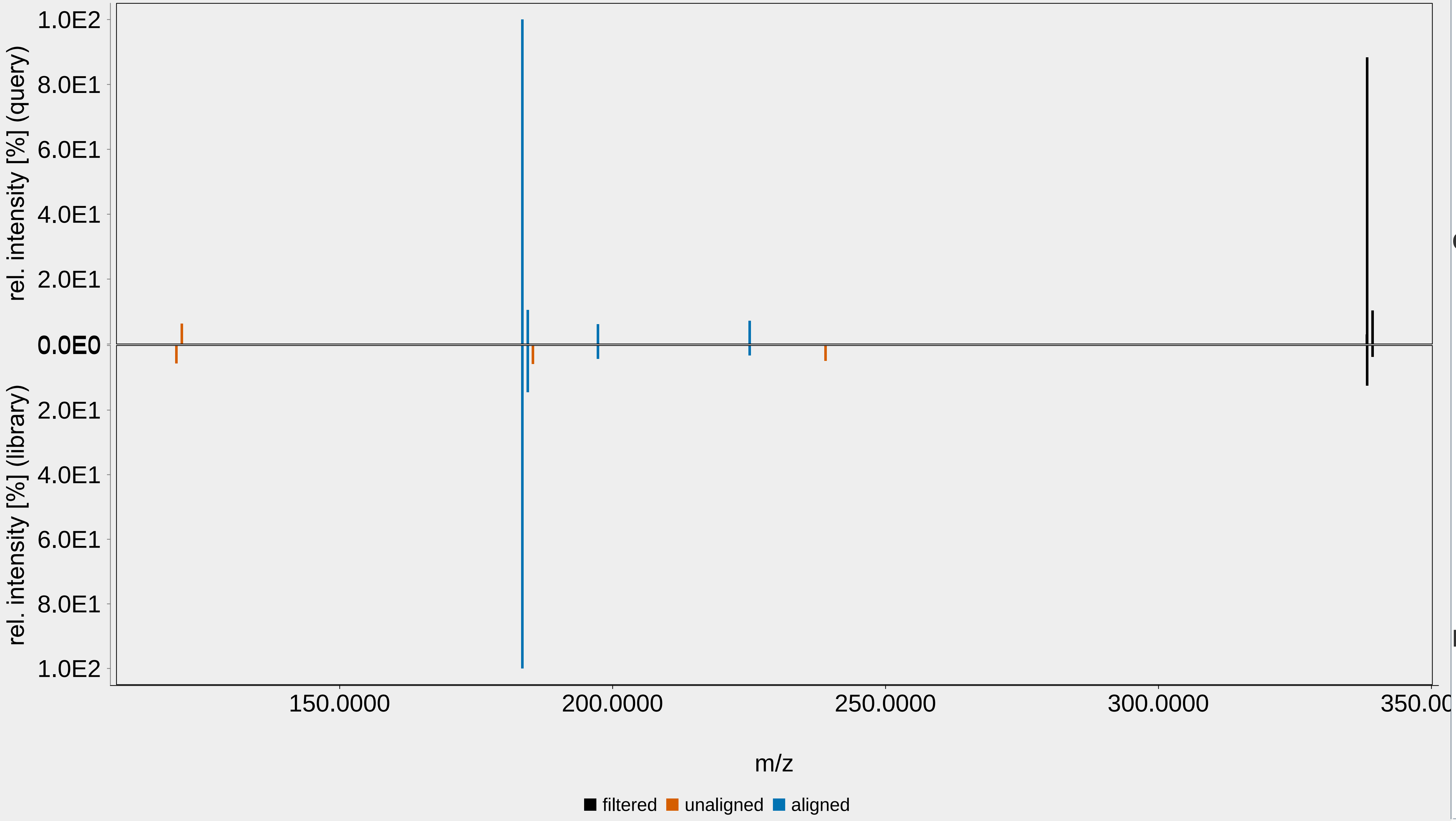

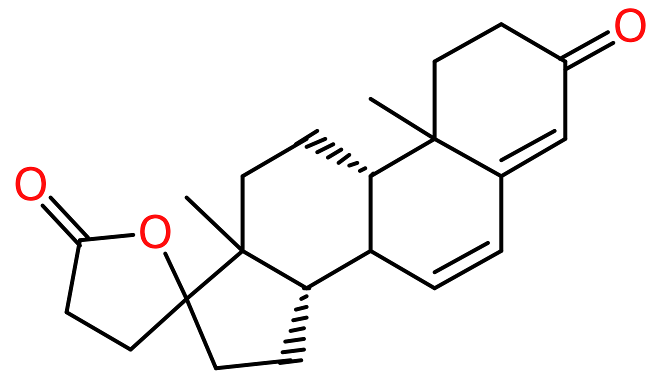

Compound information

Name: Canrenone,

Formula: C22H28O3

Mol. weight: 340.2038448

Exact mass: 340.2038448

Ion type: [M-H]-

Precursor m/z: 339.1966

Polarity: negative

InChI:

InChI key:

SMILES:

Instrument information

Instrument type: LC-ESI-QTOF

Instrument: Bruker maXis Impact

Ion source: ESI

Resolution: 35000

Ms level: MS2

Collision energy: 40 eV

Database links

Pubchem: 24728626

MoNA ID: AU239460

Chemspider: 21112020

CAS: 976-71-6

Other information

Principal investigator: Nikiforos Alygizakis,

Data collector: Nikiforos Alygizakis, Katerina

C10-LAS (TENTATIVE) 0.857

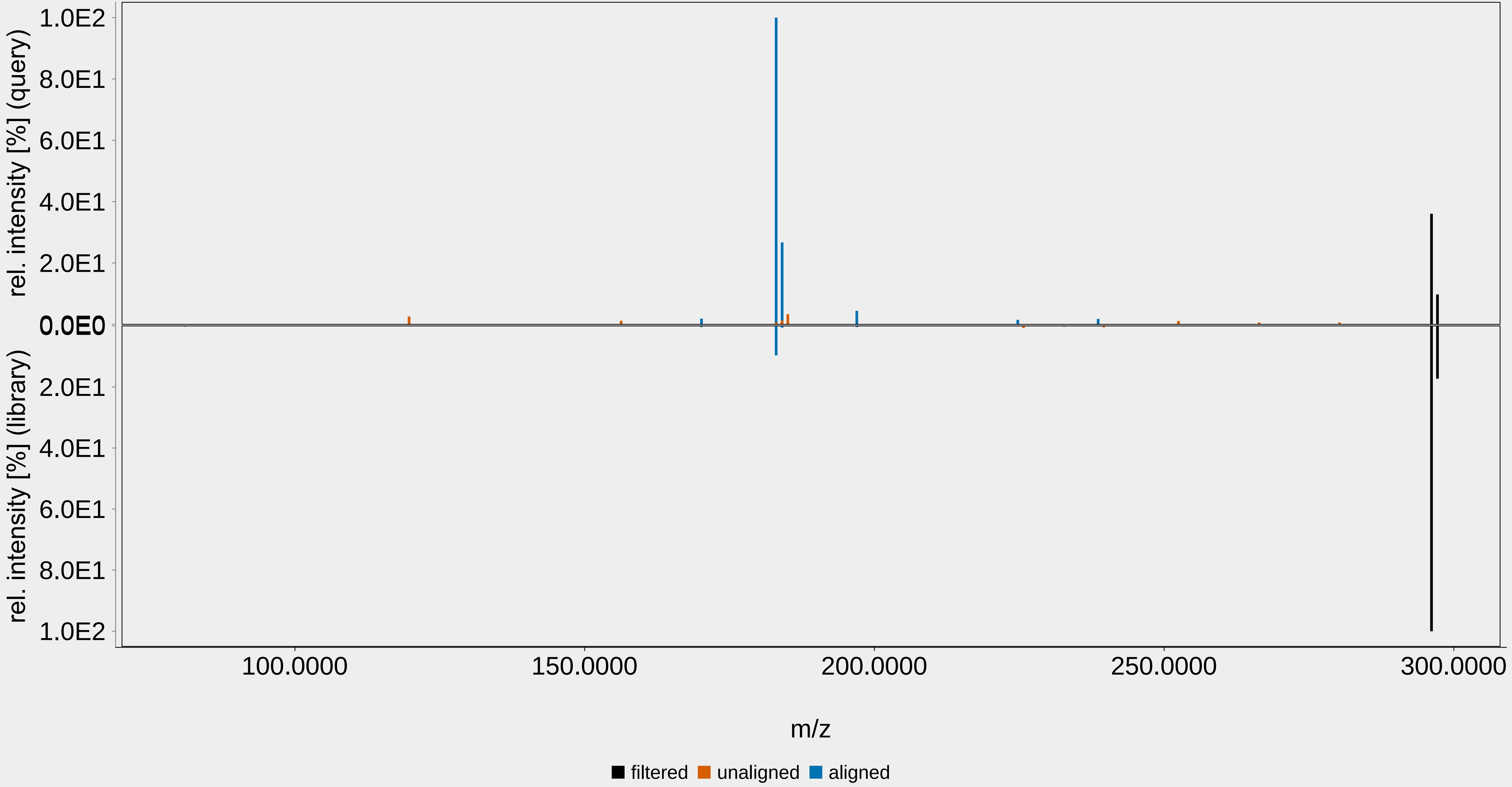

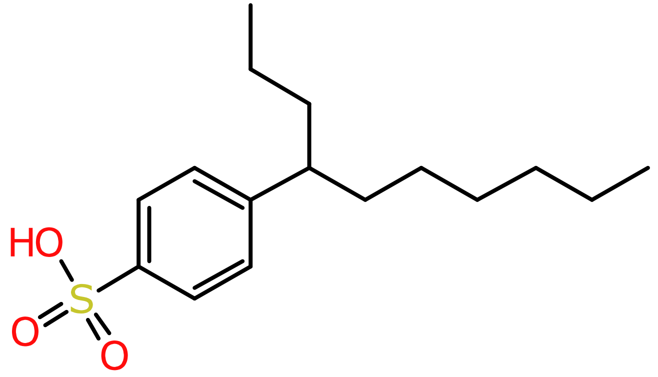

Compound information

Name: C10-LAS (TENTATIVE)

Formula: C16H26O3S

Exact mass: 298.1603

Ion type: [M-H]-

Precursor m/z: 297.1521

Polarity: NEGATIVE

InChI:

InChI key: NANHIUZYPFDGJS-UHFFFAOYSA-

SMILES:

Num peaks: 20

Instrument information

Instrument type: LC-ESI-ITFT

Instrument: LTQ Orbitrap XL Thermo Scientific

Ms level: MS2

Collision energy: 60 NCE

Database links

Other information

Entry ID:

Comment: Parent=297.1521

C10-LAS (TENTATIVE), C10-linear alkylbenzyl sulfonate, 4-(decan-4-yl)benzenesulfonic acid

0.858

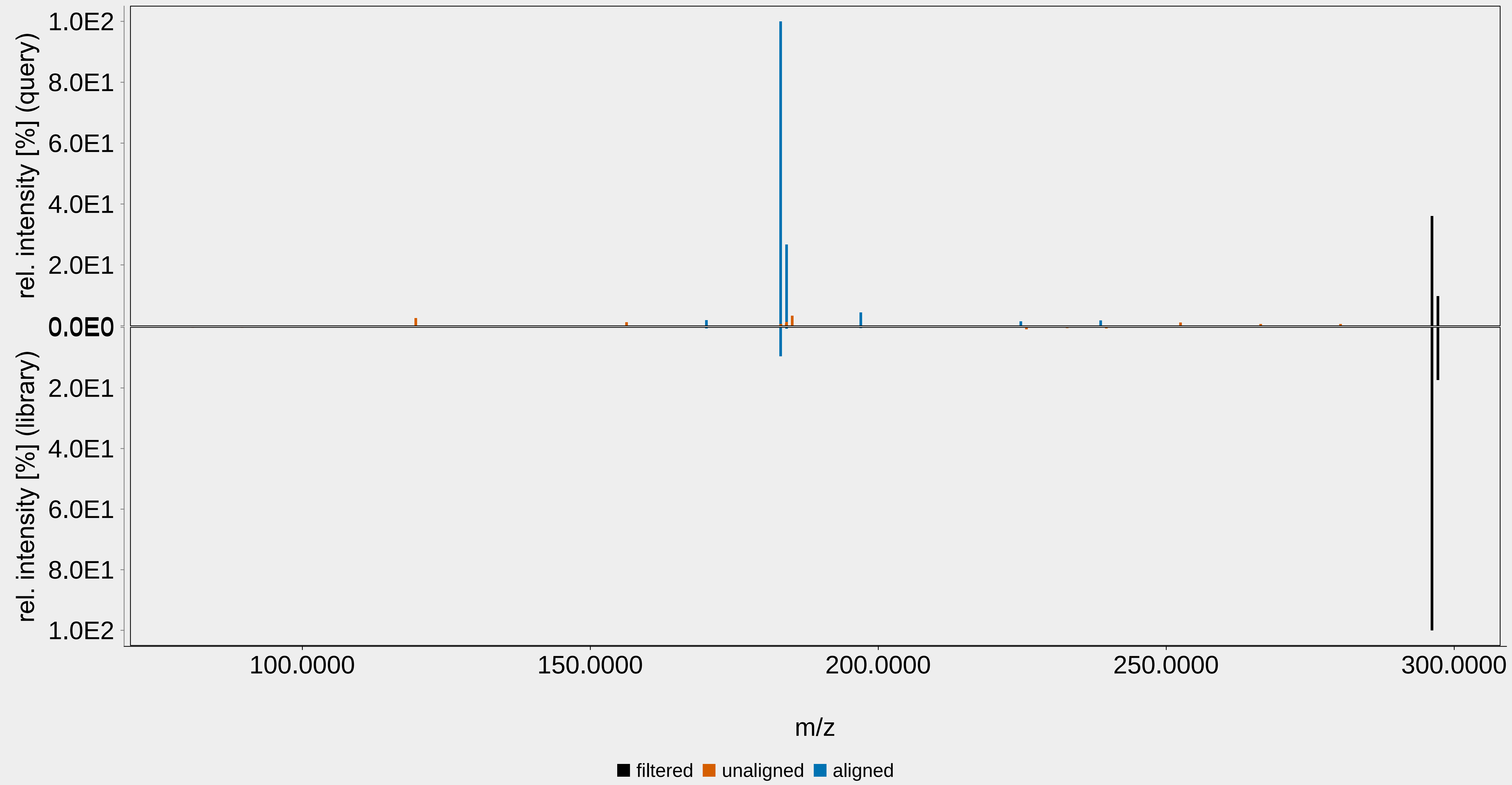

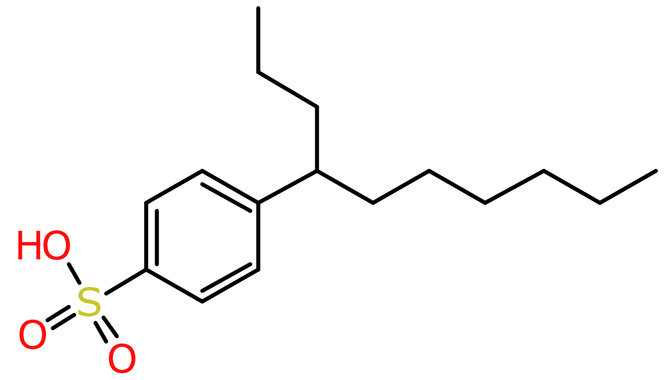

Compound information

Name: C10-LAS (TENTATIVE), C10-linear

Formula: C16H26O3S

Mol. weight: 298.1603

Exact mass: 298.1603

Ion type: [M-H]-

Precursor m/z: 297.1521

Polarity: negative

InChI:

InChI key: NANHIUZYPFDGJS-UHFFFAOYSA-

SMILES:

Instrument information

Instrument type: LC-ESI-ITFT

Instrument: LTQ Orbitrap XL Thermo Scientific

Ion source: ESI

Ms level: MS2

Collision energy: 60 NCE

Database links

Pubchem: 14921167

MoNA ID: ETS00001

Other information

Principal investigator: E. Schymanski, Dept. of

Data collector: E. Schymanski, Dept. of

C11-LAS (STANDARD MIX), C11-linear alkylbenzyl sulfonate, 4-(undecan-5-yl)benzenesulfonic acid

0.862

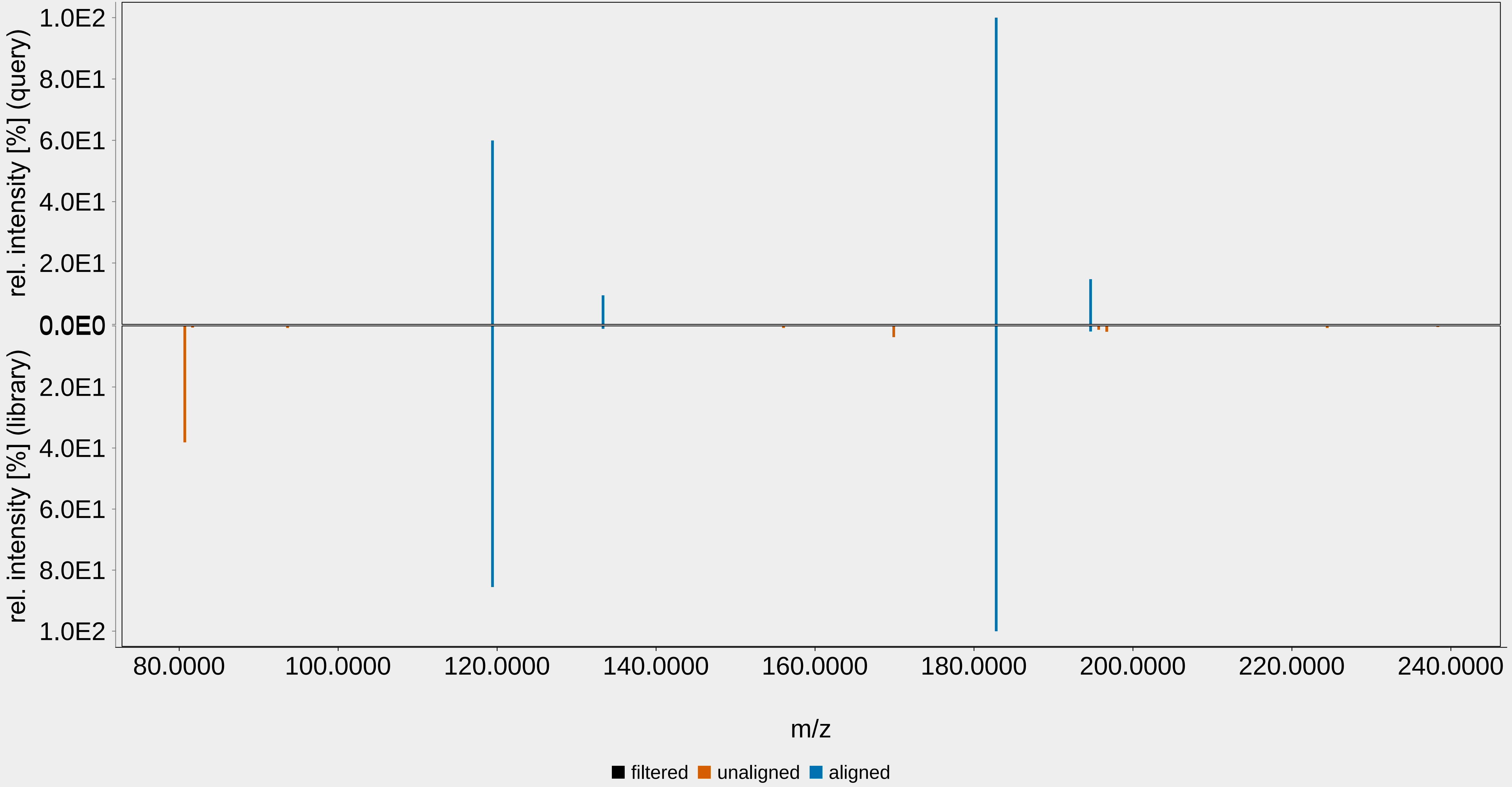

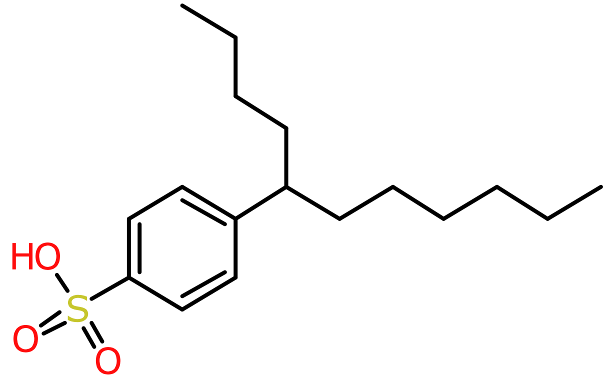

Compound information

Name: C11-LAS (STANDARD MIX), C11-linear

Formula: C17H28O3S

Mol. weight: 312.1759

Exact mass: 312.1759

Ion type: [M-H]-

Precursor m/z: 311.1686

Polarity: negative

InChI:

InChI key:

SMILES:

Instrument information

Instrument type: LC-ESI-QFT

Instrument: Q Exactive Orbitrap Thermo

Ion source: ESI

Ms level: MS2

Collision energy: 90 NCE

Database links

Pubchem: 15593874

MoNA ID: ETS00014

Other information

Principal investigator: E. Schymanski, Dept. of

Data collector: E. Schymanski, Dept. of

C11-LAS (STANDARD MIX)

0.862

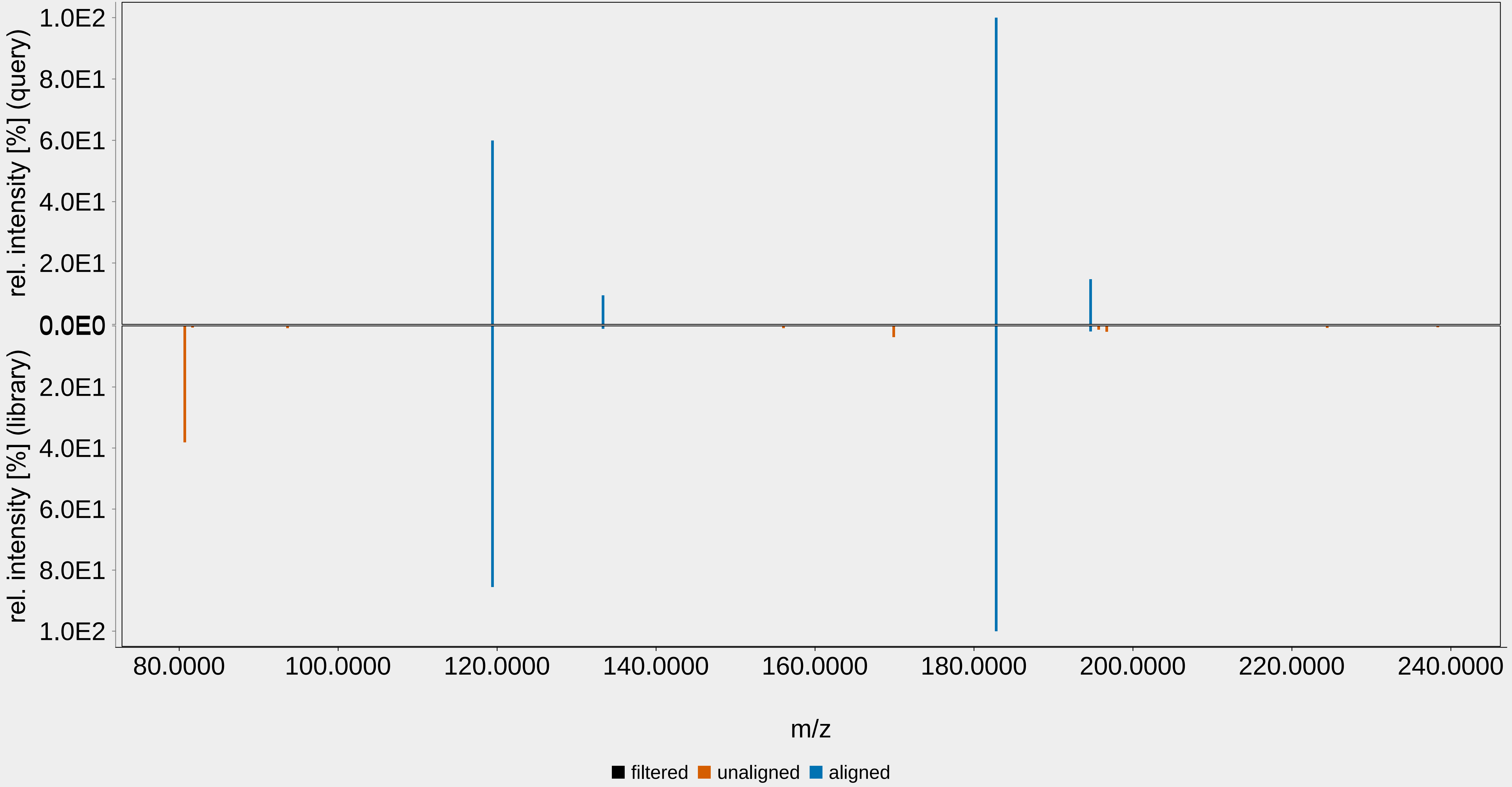

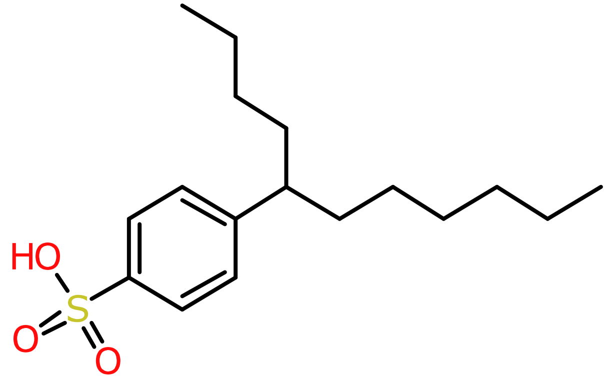

Compound information

Name: C11-LAS (STANDARD MIX)

Formula: C17H28O3S

Exact mass: 312.1759

Ion type: [M-H]-

Precursor m/z: 311.1686

Polarity: NEGATIVE

InChI:

InChI key:

SMILES:

Num peaks: 13

Instrument information

Instrument type: LC-ESI-QFT

Instrument: Q Exactive Orbitrap Thermo

Ms level: MS2

Collision energy: 90 NCE

Database links

Other information

Entry ID:

Comment: Parent=311.1686

Dodecylbenzenesulfonic acid

0.901

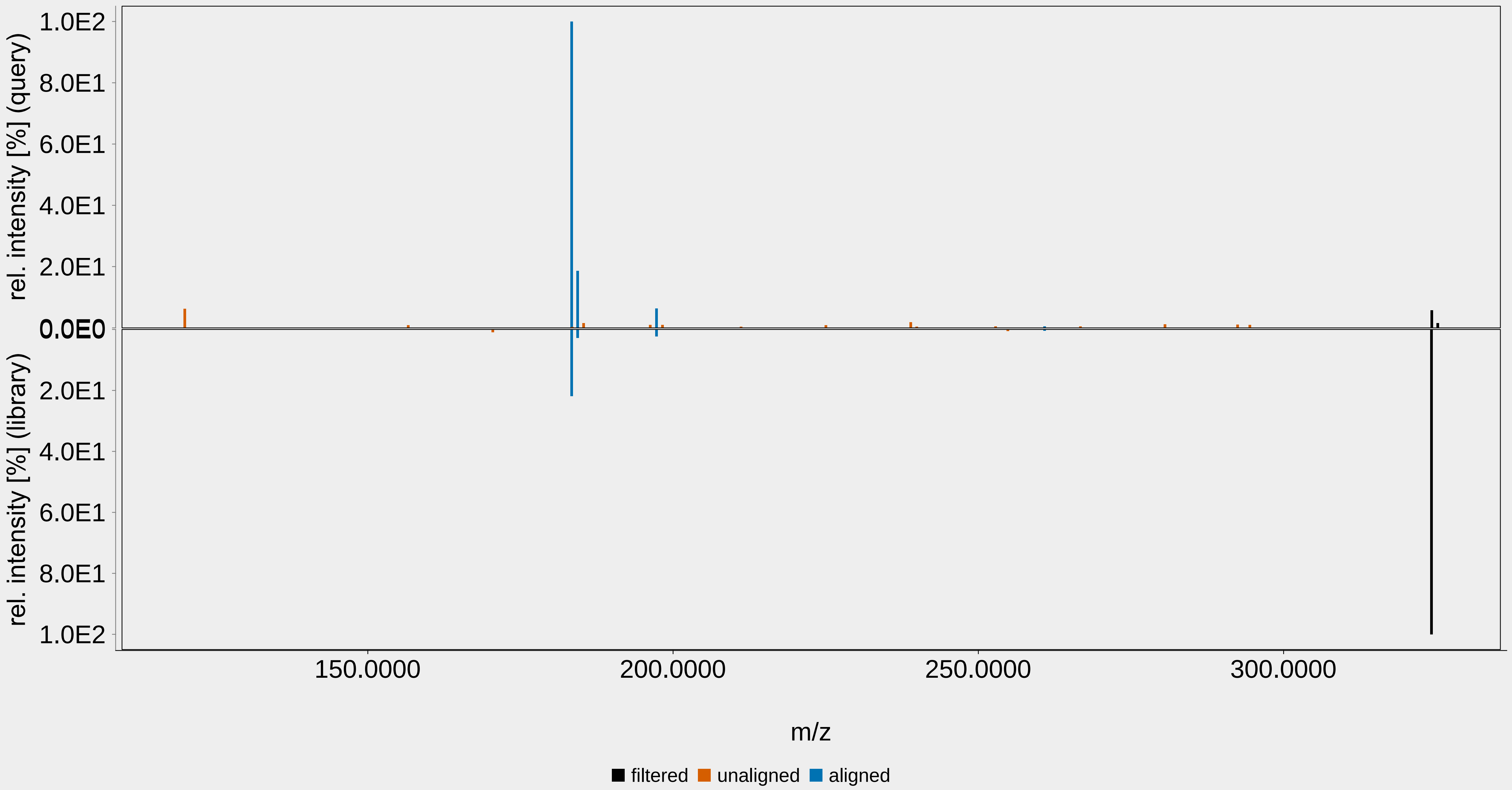

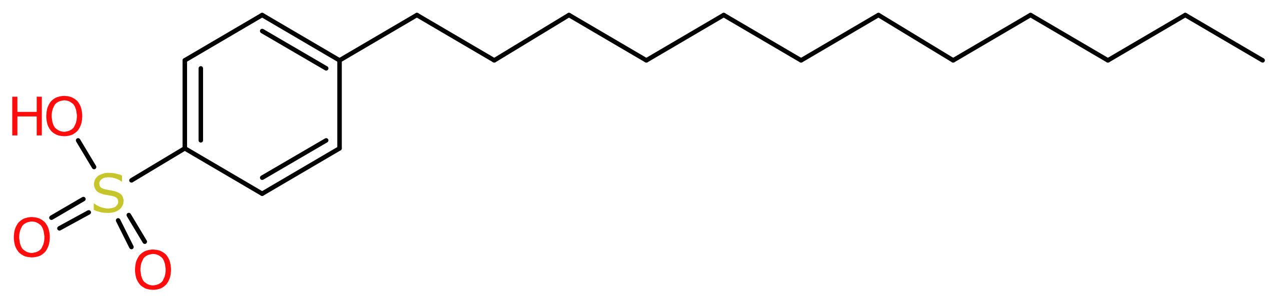

Compound information

Name: Dodecylbenzenesulfonic acid

Formula: C18H30O3S

Exact mass: 326.19157

Ion type: [M-H]-

Precursor m/z: 325.1843

Polarity: NEGATIVE

InChI:

InChI key:

SMILES:

Num peaks: 7

Instrument information

Instrument type: LC-ESI-QFT

Instrument: Q Exactive Plus Orbitrap Thermo

Ms level: MS2

Collision energy: 35 (nominal)

Database links

Other information

Entry ID: MSBNK-CASMI_2016-SM876251

Comment: Parent=325.1843

Dodecylbenzenesulfonic acid

0.900

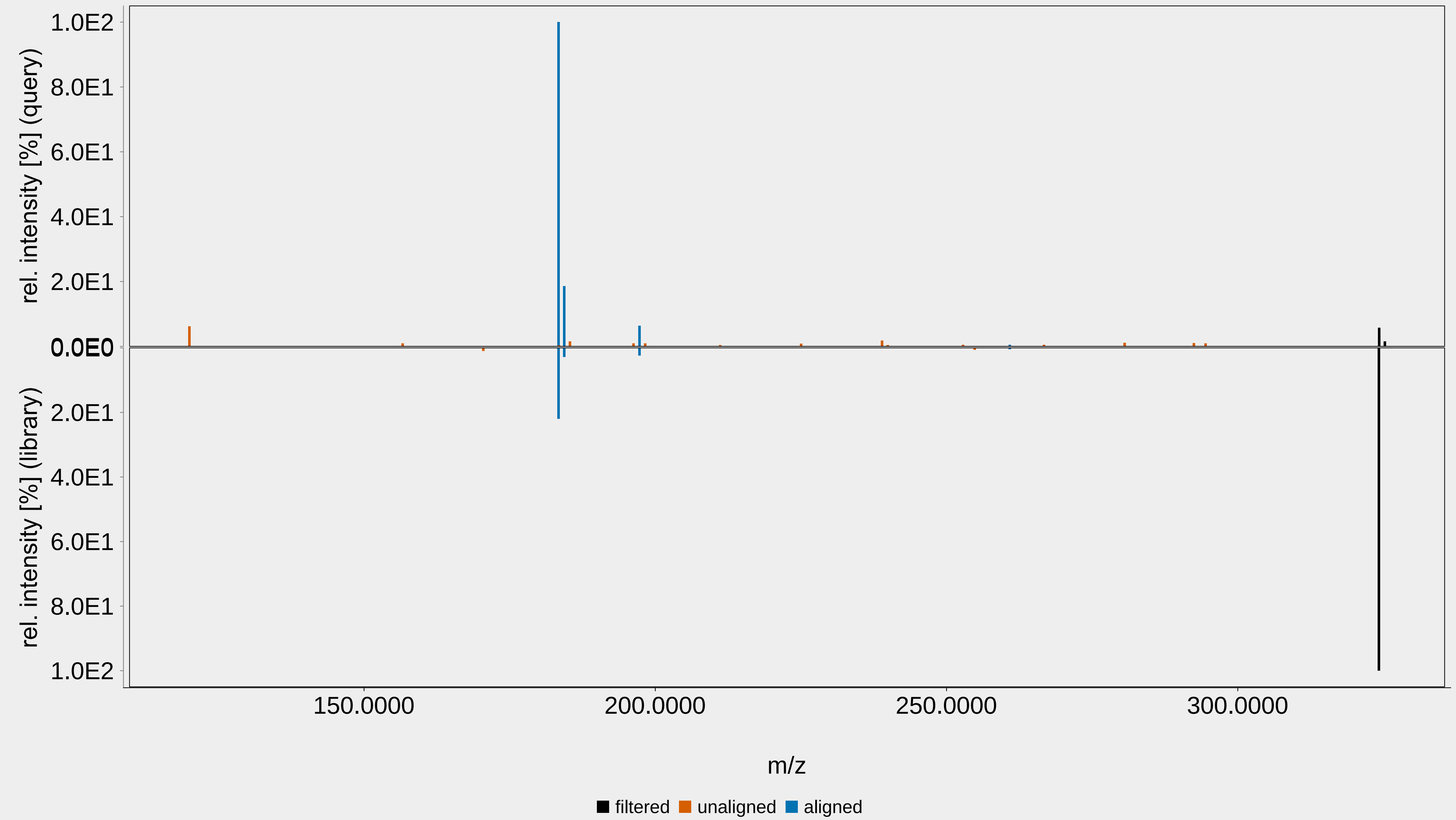

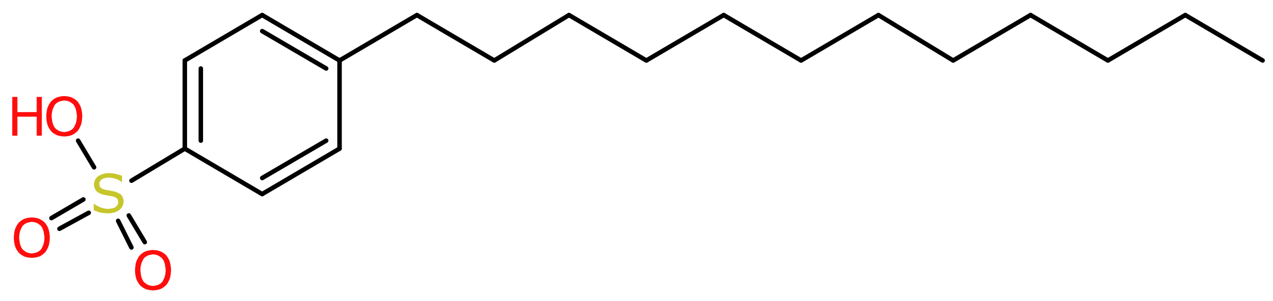

Compound information

Name: Dodecylbenzenesulfonic acid

Formula: C18H30O3S

Mol. weight: 326.19157

Exact mass: 326.19157

Ion type: [M-H]-

Precursor m/z: 325.1843

Polarity: negative

InChI:

InChI key:

SMILES:

Instrument information

Instrument type: LC-ESI-QFT

Instrument: Q Exactive Plus Orbitrap Thermo

Ion source: ESI

Resolution: 35000

Ms level: MS2

Collision energy: 35 (nominal)

Database links

Pubchem: 8485

MoNA ID: SM876251

Chemspider: 8172

CAS: 121-65-3

Other information

Principal investigator: Krauss M, Schymanski

Data collector: Krauss M, Schymanski EL,

Glyceryl phosphate - 30.0 eV

0.985

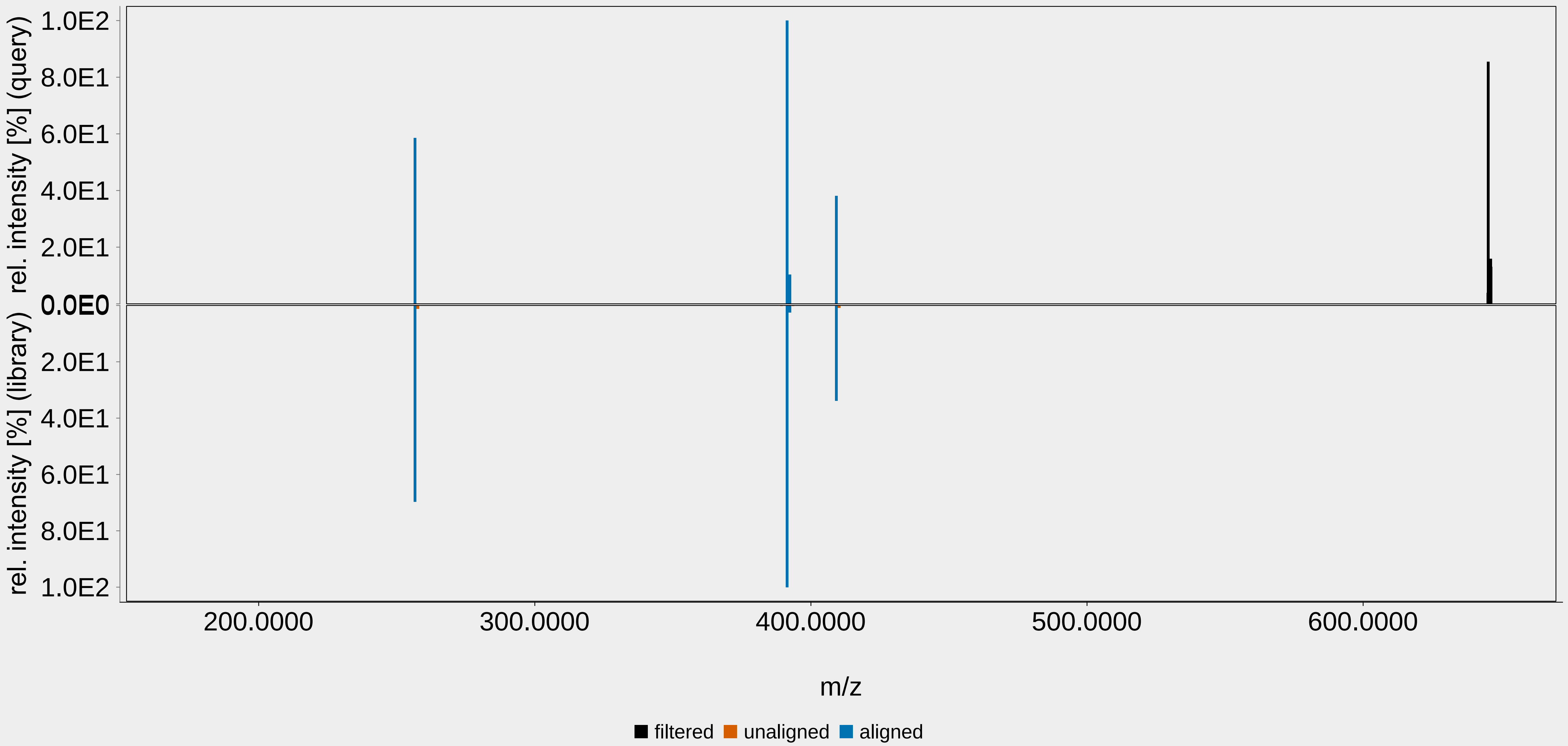

Compound information

Name: Glyceryl phosphate - 30.0 eV

Exact mass: 0.0

Ion type: Unknown

Precursor m/z: 647.466

Charge: 1

Polarity: Negative

SMILES: C([C@H](COP(=O)(O)O)O)O

Instrument information

Instrument type: Orbitrap

Ion source: ESI

Ms level: 2

Acquisition: Isolated

Database links

CAS:

Other information

Principal investigator: PI

Data collector: Data Collector

Entry ID: CCMSLIB00006126789

Glyceryl phosphate - 30.0 eV

0.986

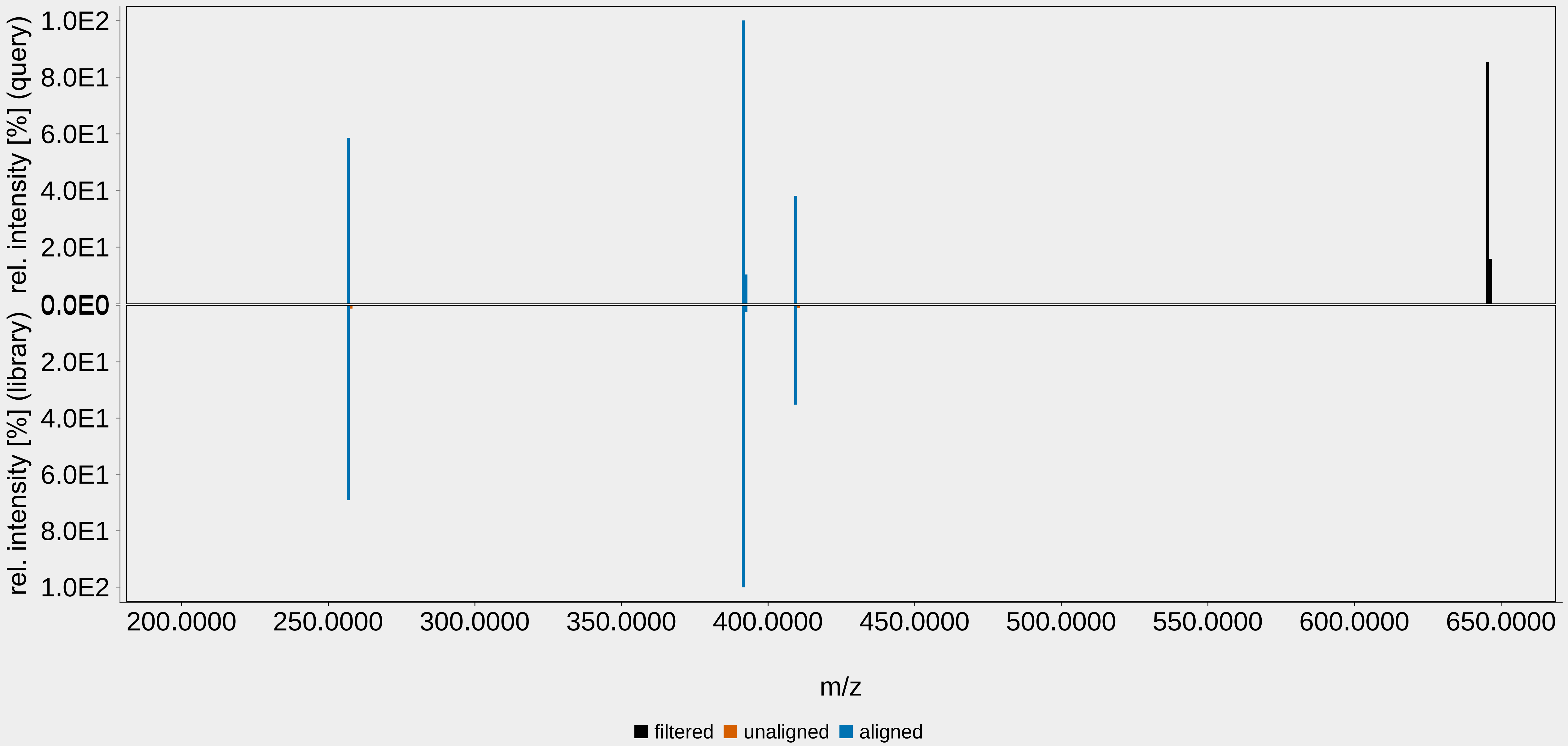

Compound information

Name: Glyceryl phosphate - 30.0 eV

Exact mass: 0.0

Ion type: Unknown

Precursor m/z: 647.466

Charge: 1

Polarity: Negative

SMILES: C([C@H](COP(=O)(O)O)O)O

Instrument information

Instrument type: Orbitrap

Ion source: ESI

Ms level: 2

Acquisition: Isolated

Database links

CAS:

Other information

Principal investigator: PI

Data collector: Data Collector

Entry ID: CCMSLIB00006126790

Dodecylbenzenesulfonic acid

0.939

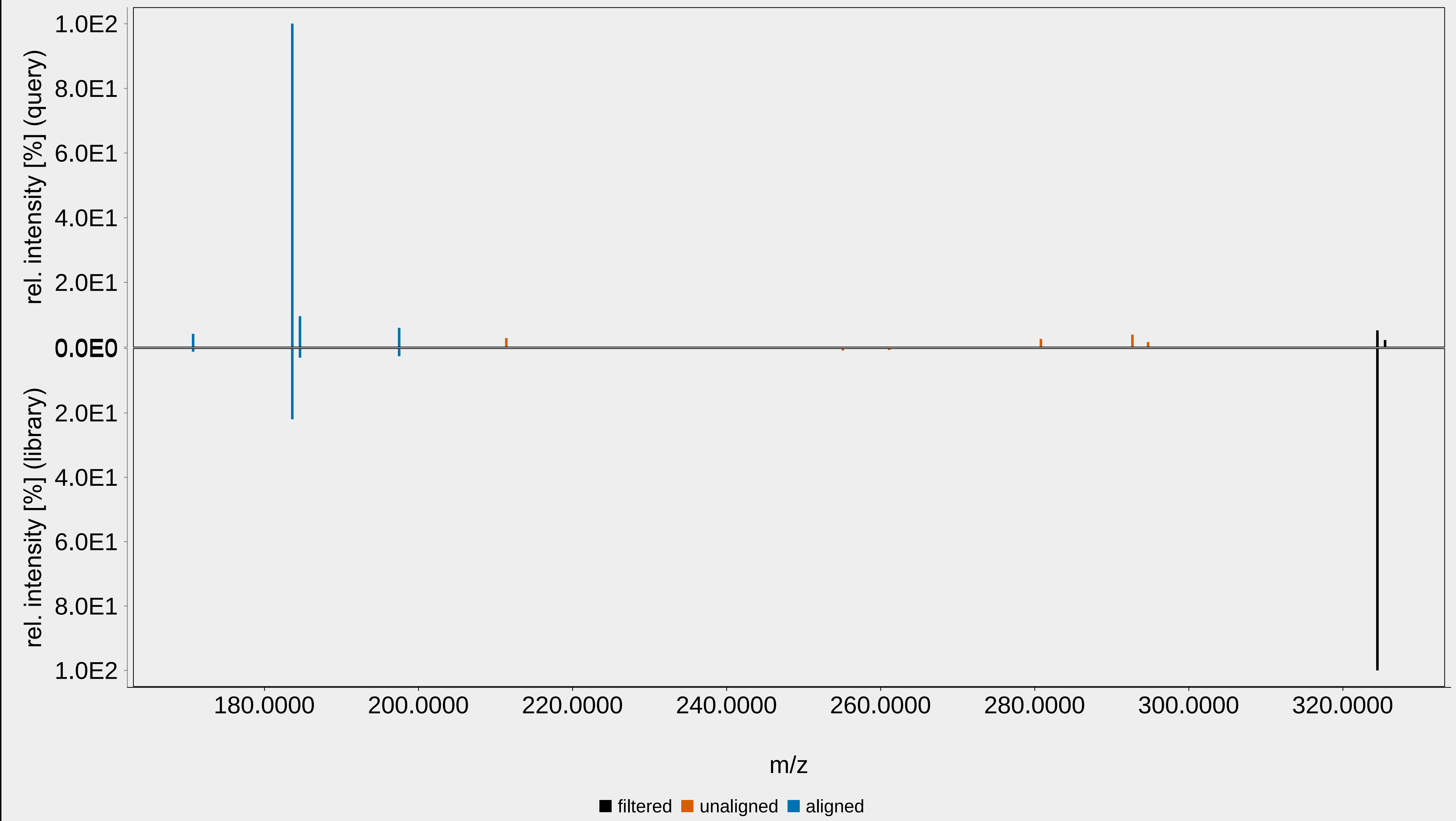

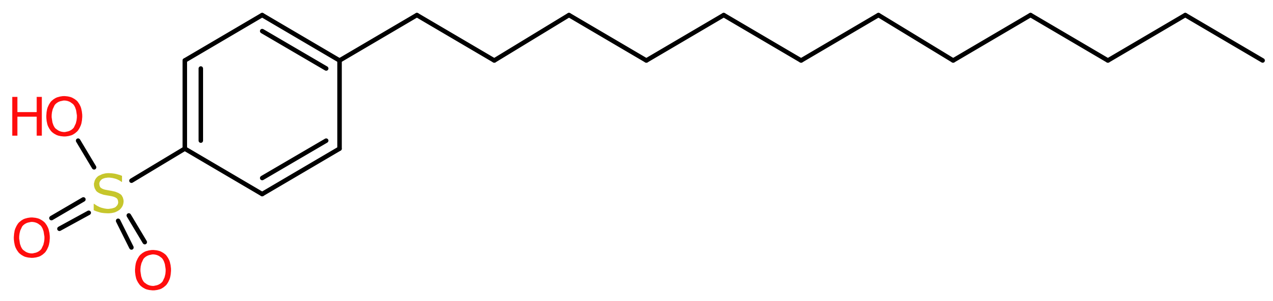

Compound information

Name: Dodecylbenzenesulfonic acid

Formula: C18H30O3S

Mol. weight: 326.19157

Exact mass: 326.19157

Ion type: [M-H]-

Precursor m/z: 325.1843

Polarity: negative

InChI:

InChI key:

SMILES:

Instrument information

Instrument type: LC-ESI-QFT

Instrument: Q Exactive Plus Orbitrap Thermo

Ion source: ESI

Resolution: 35000

Ms level: MS2

Collision energy: 35 (nominal)

Database links

Pubchem: 8485

MoNA ID: SM876251

Chemspider: 8172

CAS: 121-65-3

Other information

Principal investigator: Krauss M, Schymanski

Data collector: Krauss M, Schymanski EL,

Dodecylbenzenesulfonic acid

0.940

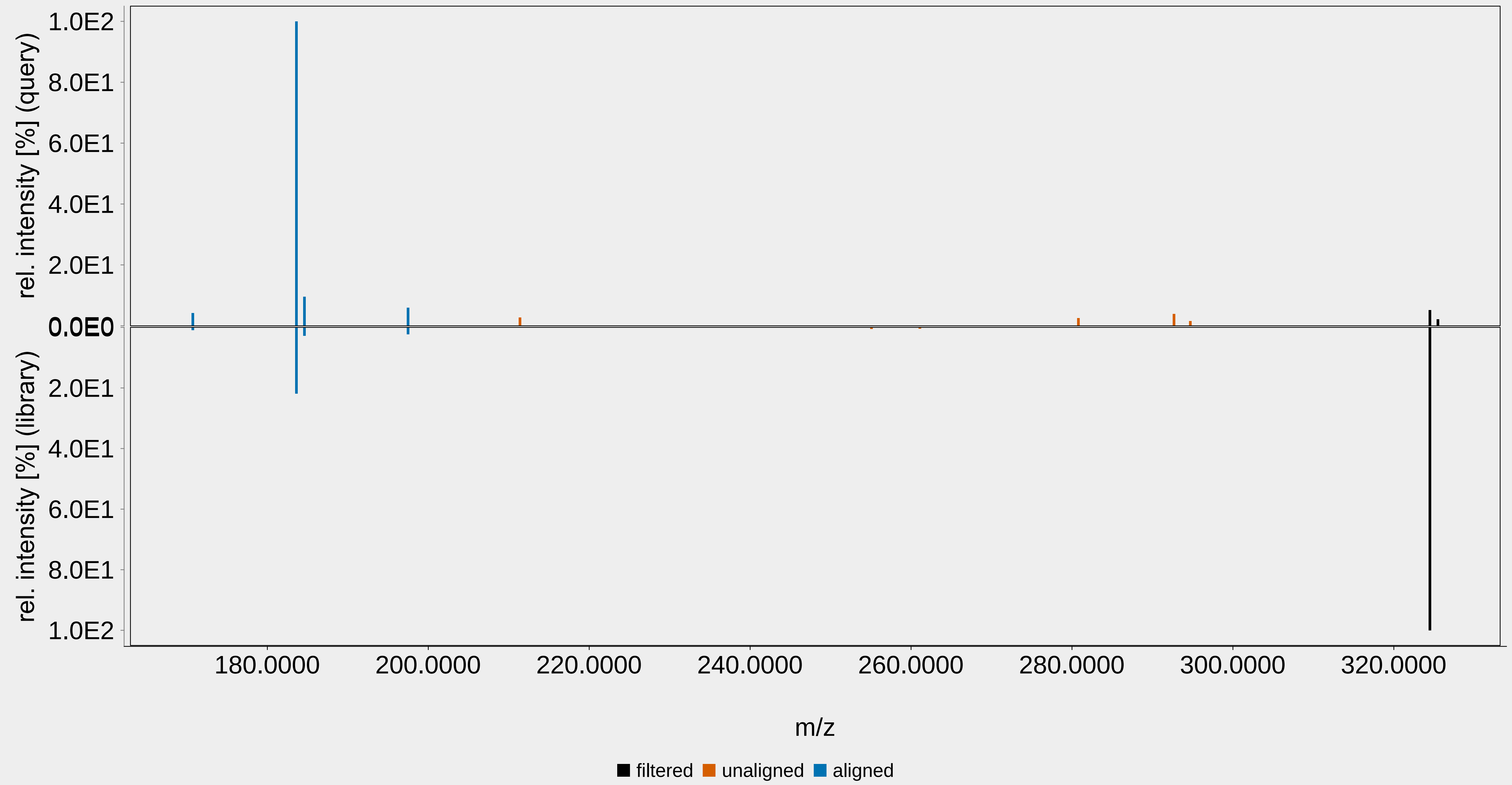

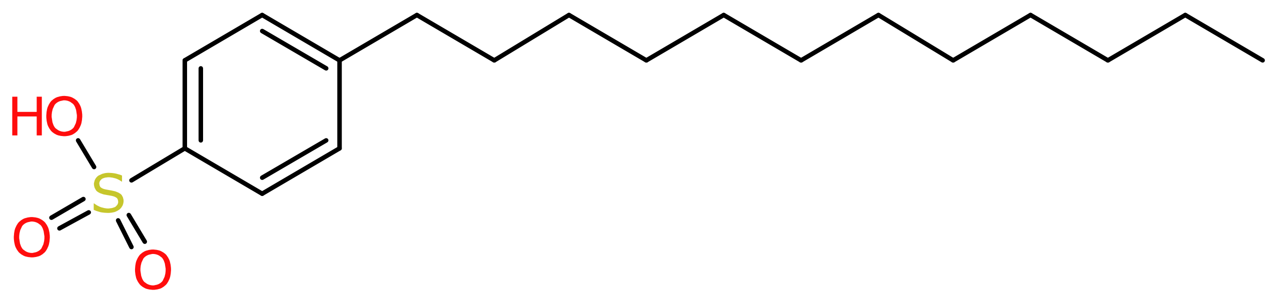

Compound information

Name: Dodecylbenzenesulfonic acid

Formula: C18H30O3S

Exact mass: 326.19157

Ion type: [M-H]-

Precursor m/z: 325.1843

Polarity: NEGATIVE

InChI:

InChI key:

SMILES:

Num peaks: 7

Instrument information

Instrument type: LC-ESI-QFT

Instrument: Q Exactive Plus Orbitrap Thermo

Ms level: MS2

Collision energy: 35 (nominal)

Database links

Other information

Entry ID: MSBNK-CASMI_2016-SM876251

Comment: Parent=325.1843

PS 16:0_22:6

0.854

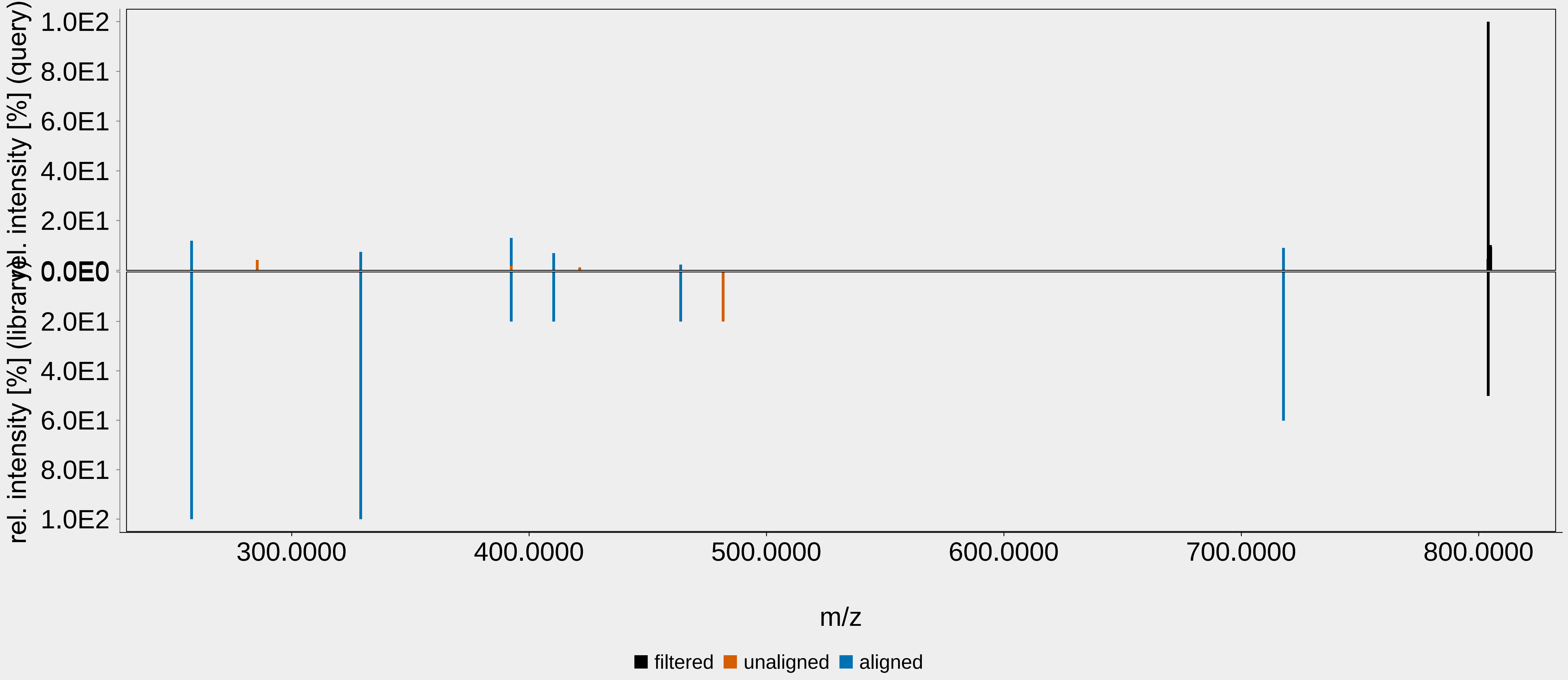

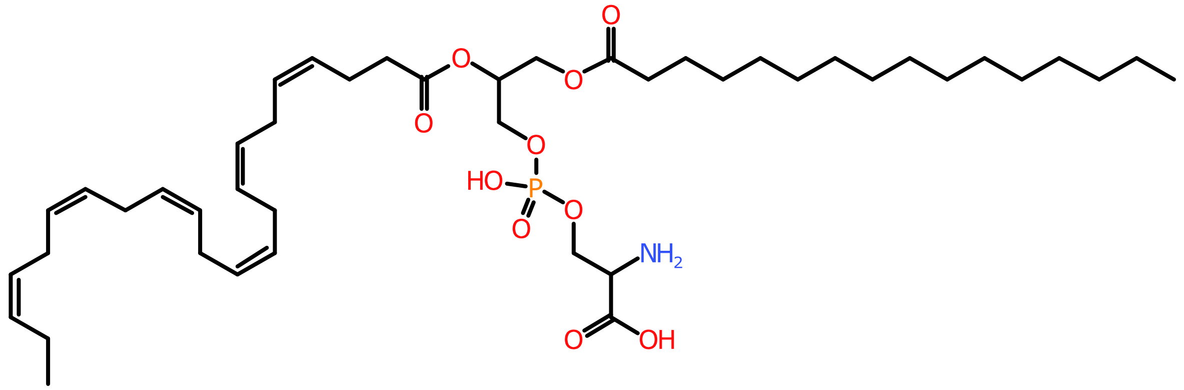

Compound information

Name: PS 16:0_22:6

Formula: C44H74NO10P

Ion type: [M-H]-

Precursor m/z: 806.49776

Ccs: 276.61

InChI key:

SMILES:

Num peaks: 8

Instrument information

Database links

Other information

PS 38:6 **0.850**

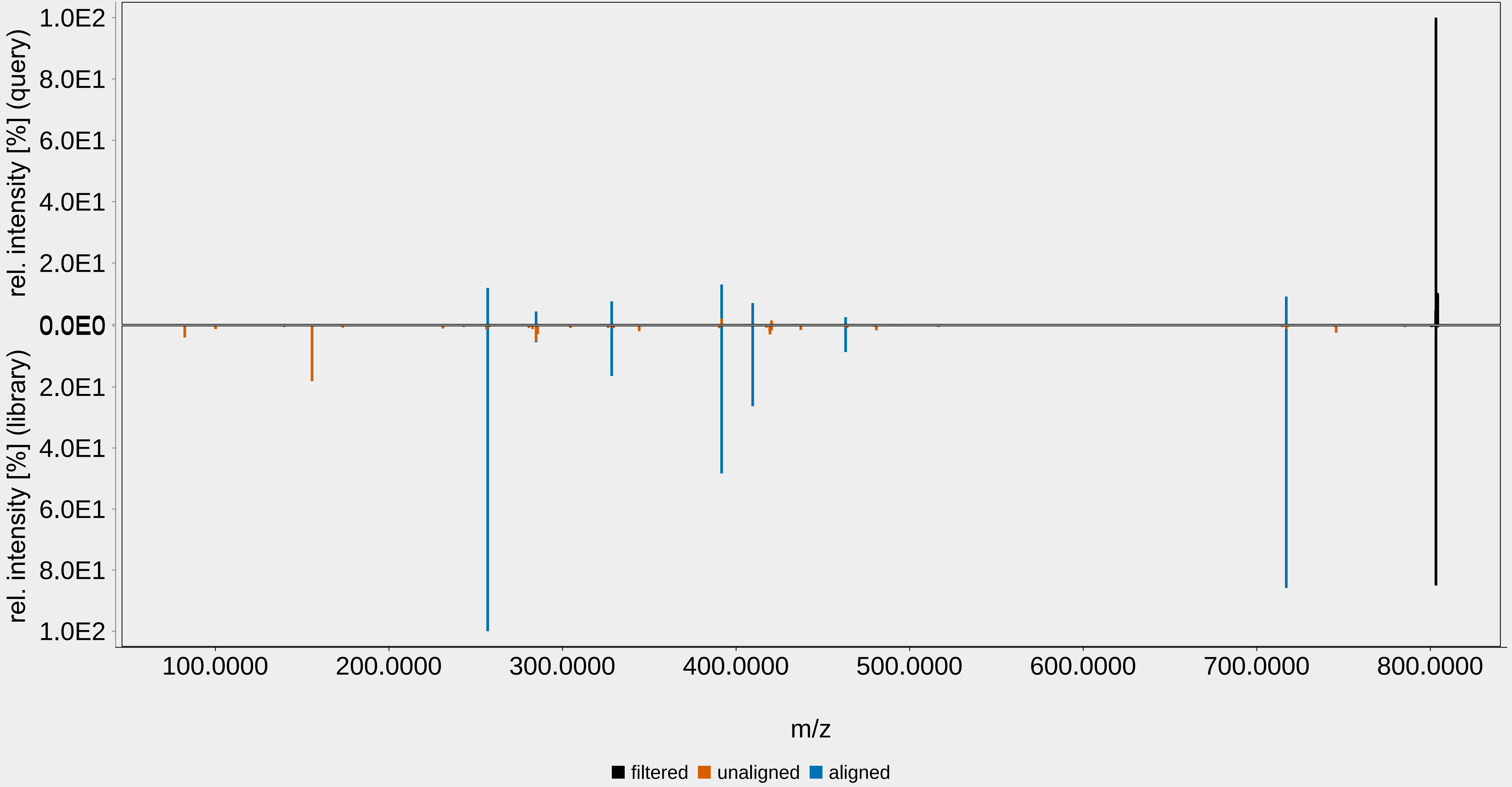

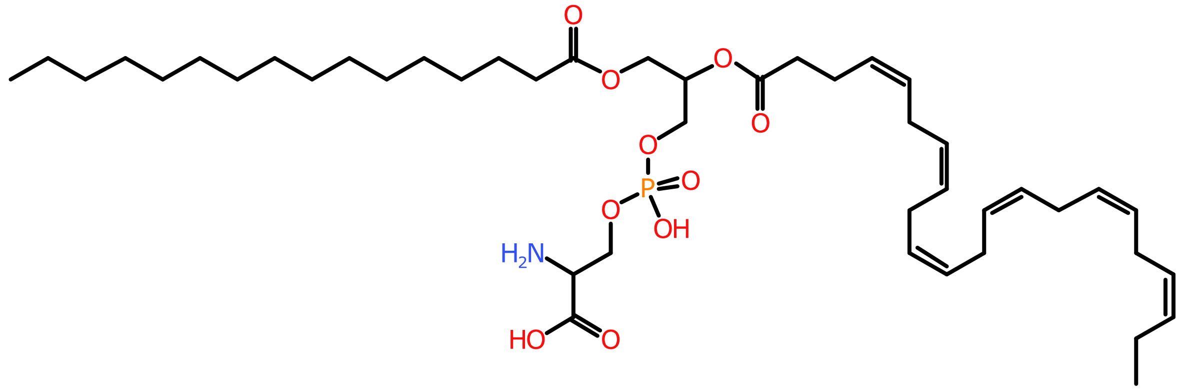

Compound information

Name: PS 38:6

Formula: C44H74NO10P

Exact mass: 808.0489999999999

Ion type: [M-H]-

Precursor m/z: 806.5011

Polarity: NEGATIVE

InChI:

InChI key:

SMILES:

Num peaks: 57

Instrument information

Instrument type: LC-ESI-QTOF

Instrument: AB Sciex TripleTOF 5600+ system

Ms level: MS2

Collision energy: 45V

Database links

Other information

Entry ID: MSBNK-RIKEN_IMS-LQB00330

Comment: Parent=806.5011

PE(16:0/16:0); [M-H]- C37H73N1O8P1 **0.926**

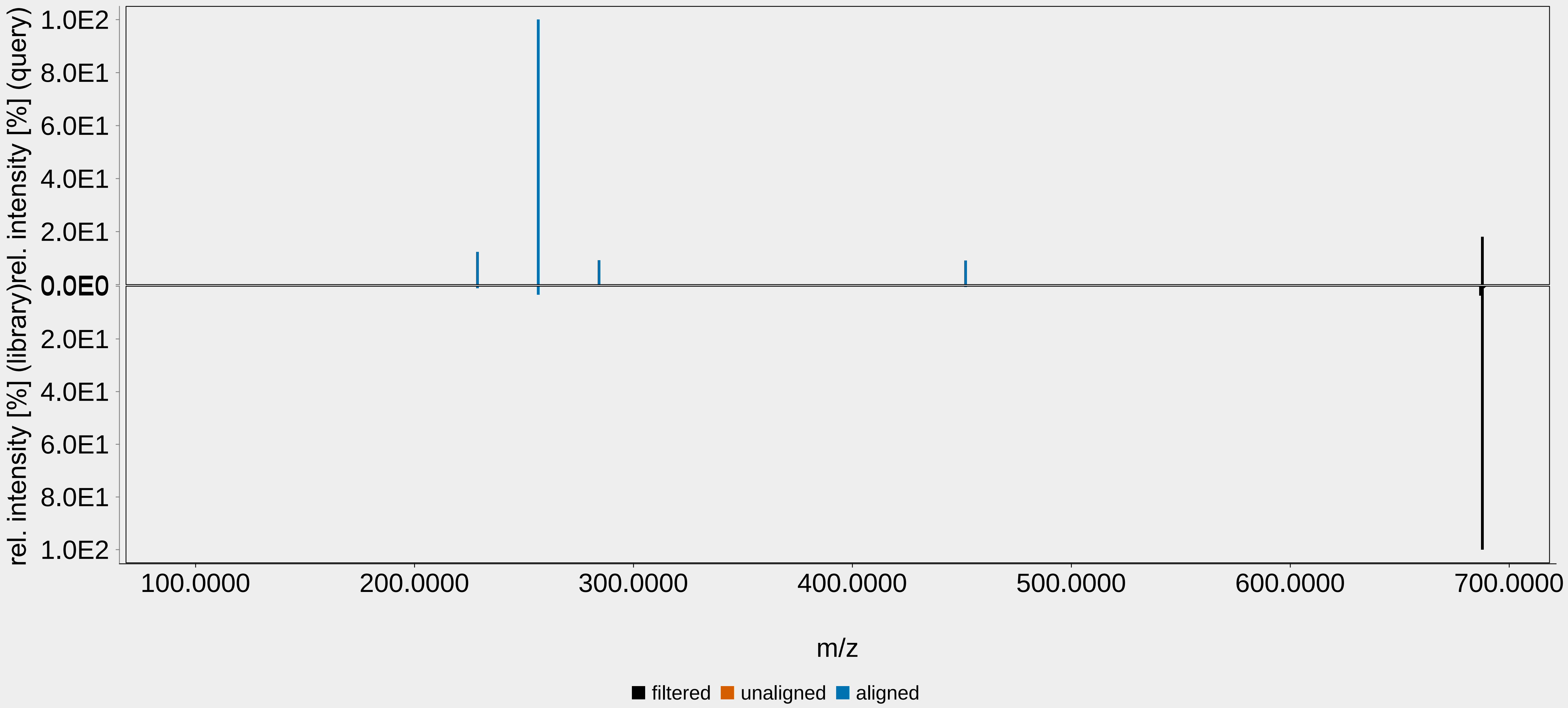

Compound information

Name: PE(16:0/16:0); [M-H]- C37H73N1O8P1

Exact mass: 0.0

Ion type: M-H

Precursor m/z: 690.507

Charge: 1

Polarity: Negative

Instrument information

Instrument type: HCD; Velos

Ion source: LC-ESI

Ms level: 2

Acquisition: Commercial

Database links

Other information

Principal investigator: Thomas Metz

Data collector: Thomas Metz

Entry ID: CCMSLIB00010069794

PE(16:0/16:0); [M-H]- C37H73N1O8P1 0.944

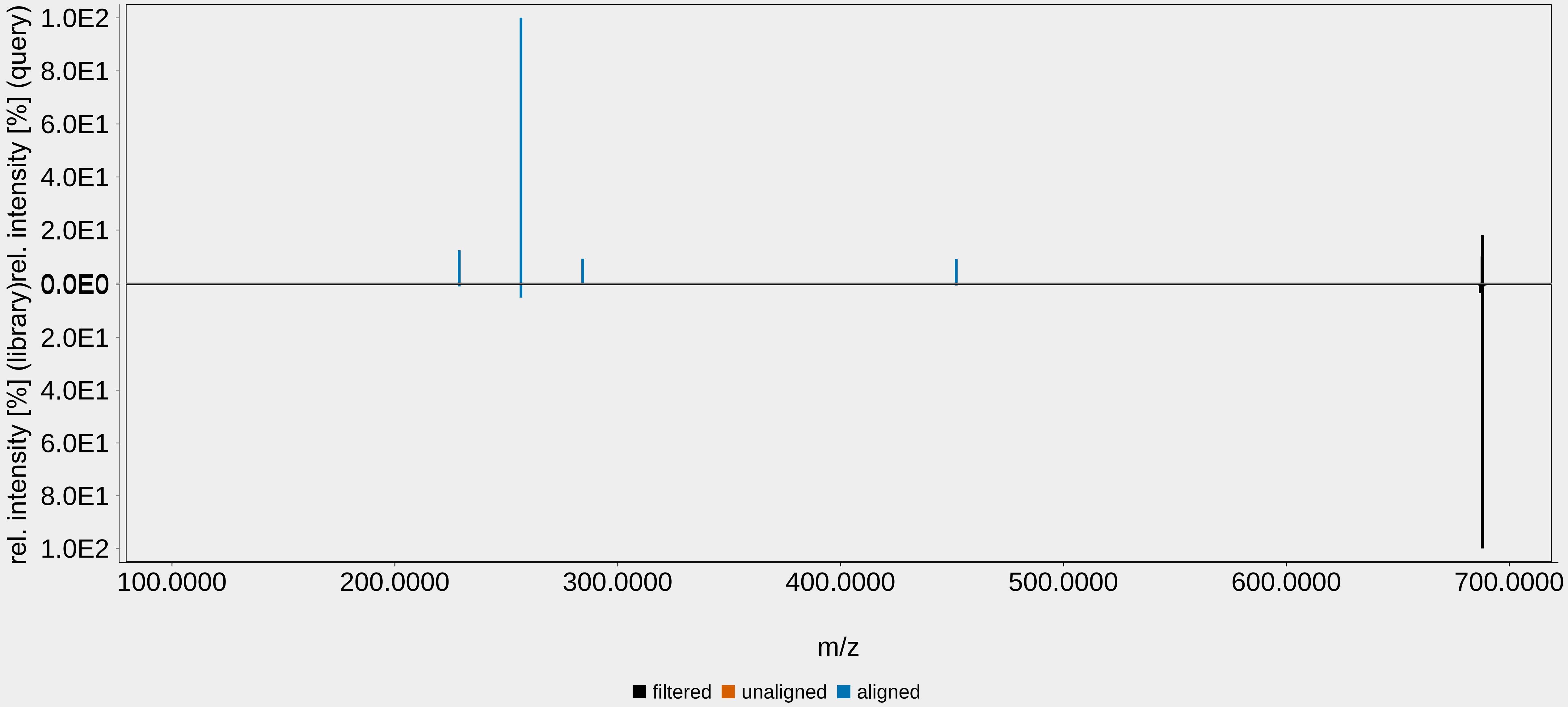

Compound information

Name: PE(16:0/16:0); [M-H]- C37H73N1O8P1

Exact mass: 0.0

Ion type: M-H

Precursor m/z: 690.507

Charge: 1

Polarity: Negative

Instrument information

Instrument type: HCD; Velos

Ion source: LC-ESI

Ms level: 2

Acquisition: Commercial

Database links

Other information

Principal investigator: Thomas Metz

Data collector: Thomas Metz

Entry ID: CCMSLIB00010068325

PA(18:2/18:2); [M-H]- C39H68O8P1 0.721

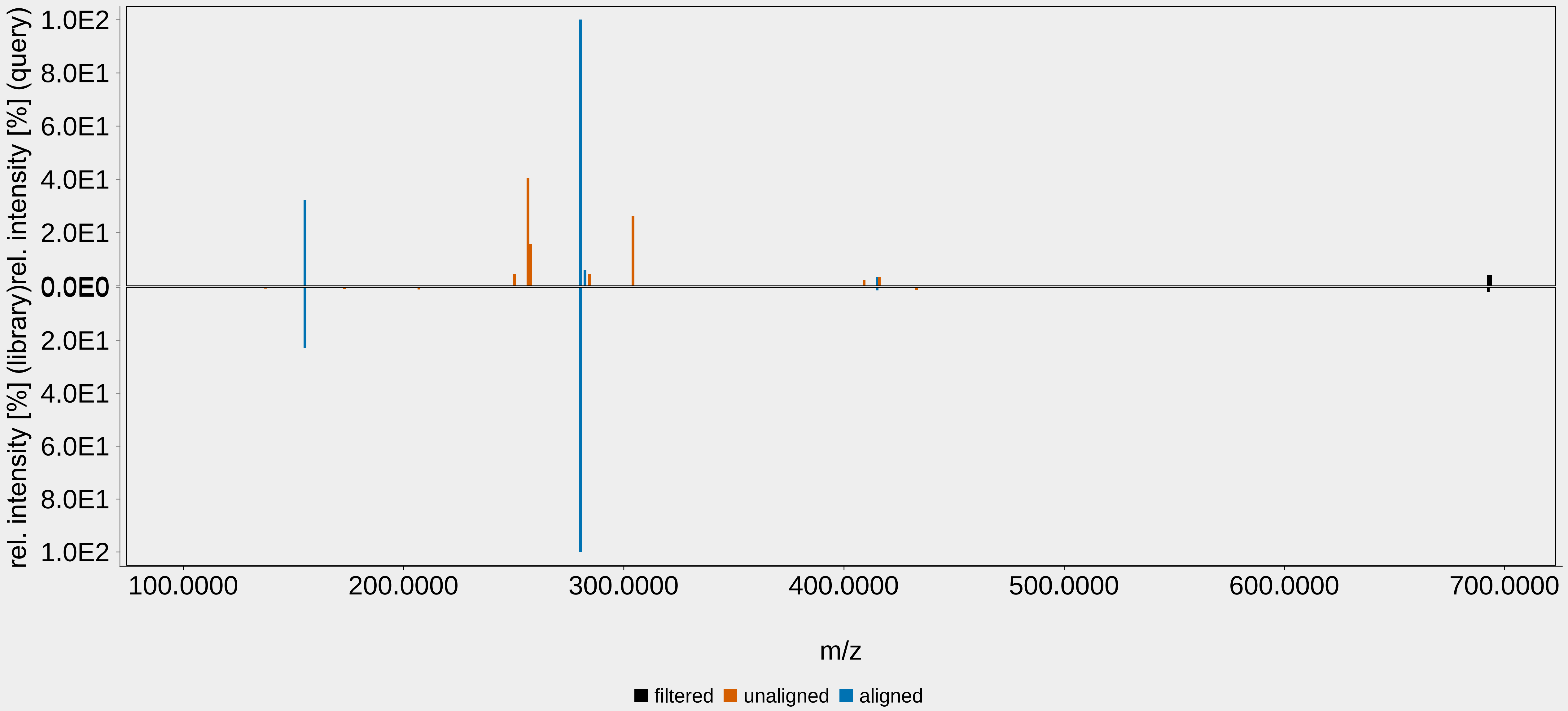

Compound information

Name: PA(18:2/18:2); [M-H]- C39H68O8P1

Exact mass: 0.0

Ion type: M-H

Precursor m/z: 695.465

Charge: 1

Polarity: Negative

Instrument information

Instrument type: HCD; Lumos

Ion source: LC-ESI

Ms level: 2

Acquisition: Commercial

Database links

Other information

Principal investigator: Thomas Metz

Data collector: Thomas Metz

Entry ID: CCMSLIB00010059139

PA 16:0_20:4 **0.903**

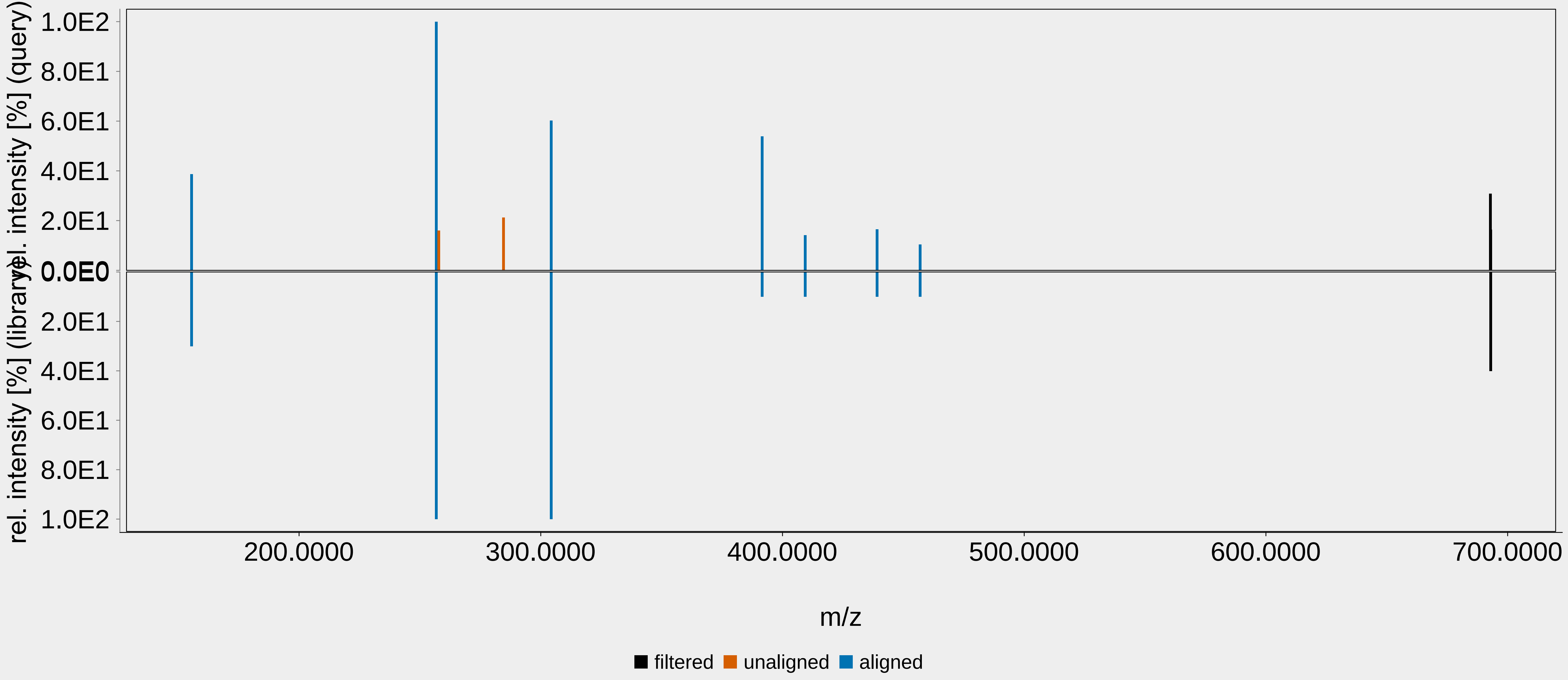

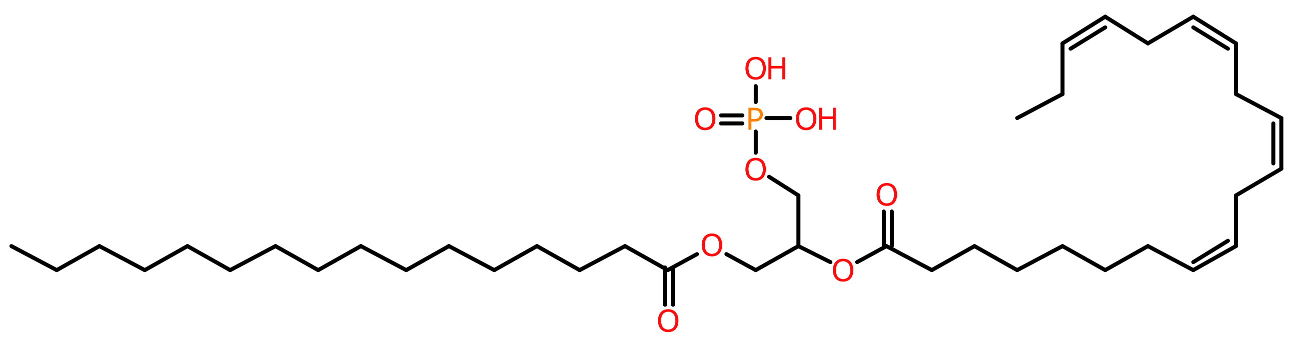

Compound information

Name: PA 16:0_20:4

Formula: C39H69O8P

Ion type: [M-H]-

Precursor m/z: 695.46573

Ccs: 255.1

InChI key:

SMILES:

Num peaks: 8

Instrument information

Database links

Other information

PS(18:1/22:6); [M-H]- C46H75N1O10P1 0.815

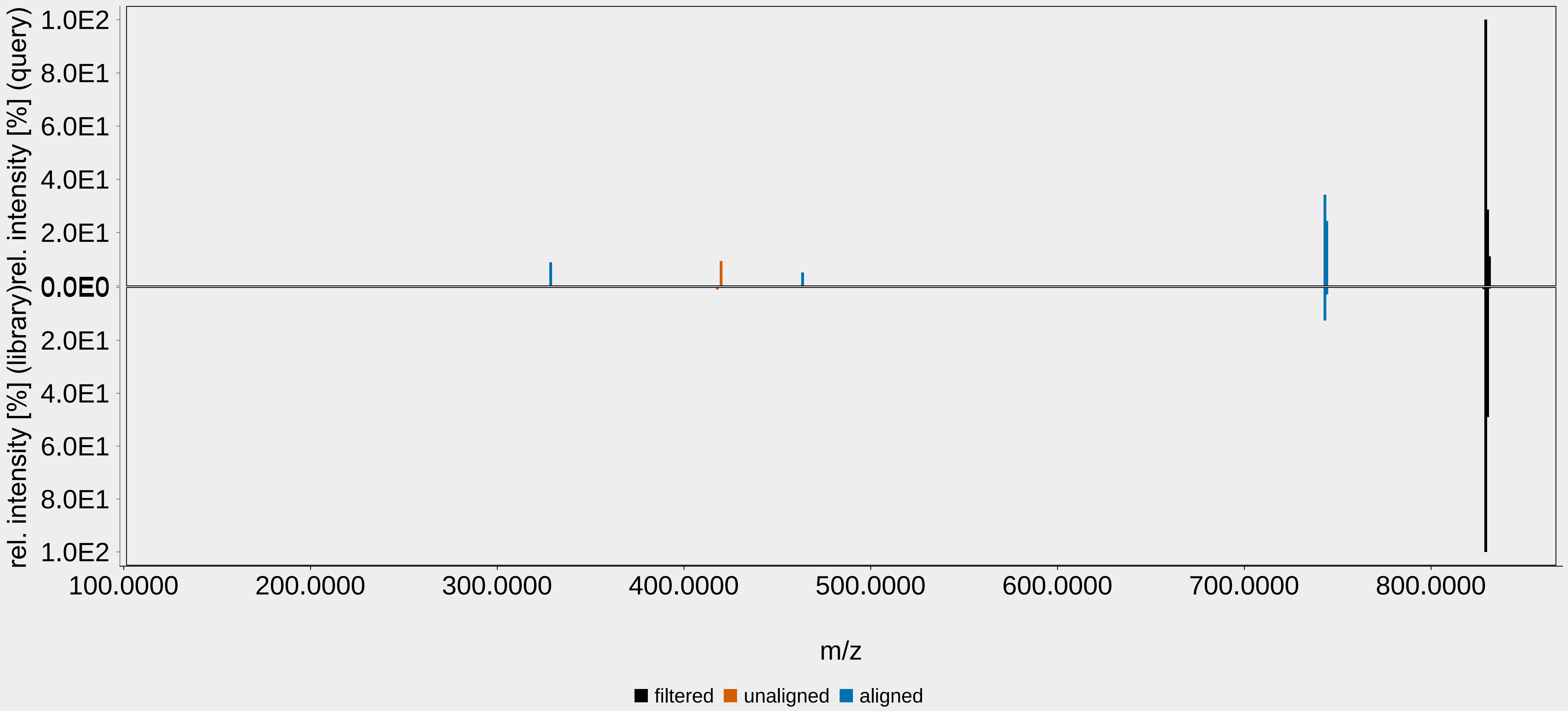

Compound information

Name: PS(18:1/22:6); [M-H]- C46H75N1O10P1

Exact mass: 0.0

Ion type: M-H

Precursor m/z: 832.513

Charge: 1

Polarity: Negative

Instrument information

Instrument type: HCD; Velos

Ion source: LC-ESI

Ms level: 2

Acquisition: Commercial

Database links

Other information

Principal investigator: Thomas Metz

Data collector: Thomas Metz

Entry ID: CCMSLIB00010083738

PS(18:1/22:6); [M-H]- C46H75N1O10P1 0.800

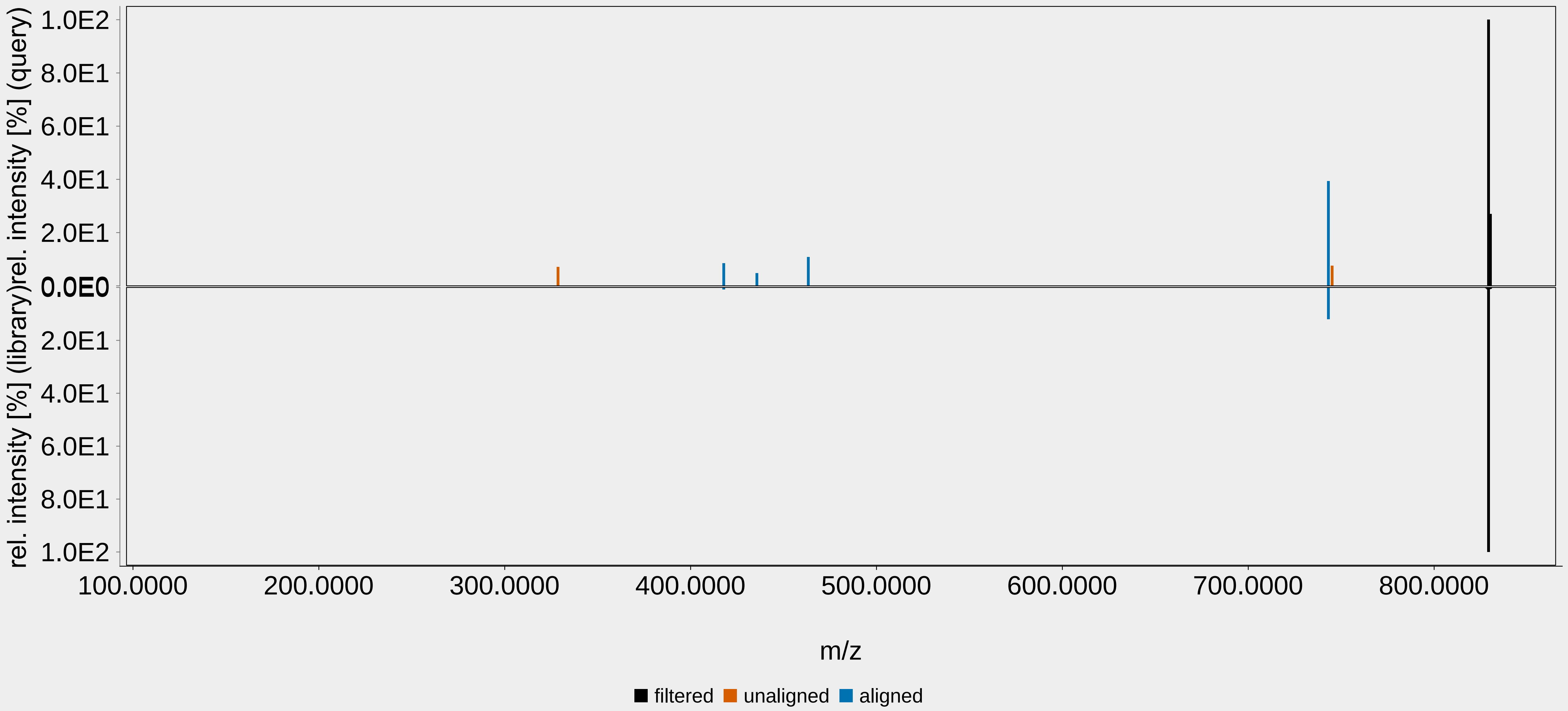

Compound information

Name: PS(18:1/22:6); [M-H]- C46H75N1O10P1

Exact mass: 0.0

Ion type: M-H

Precursor m/z: 832.513

Charge: 1

Polarity: Negative

Instrument information

Instrument type: HCD; Velos

Ion source: LC-ESI

Ms level: 2

Acquisition: Commercial

Database links

Other information

Principal investigator: Thomas Metz

Data collector: Thomas Metz

Entry ID: CCMSLIB00010063314

PS 40:6 **0.892**

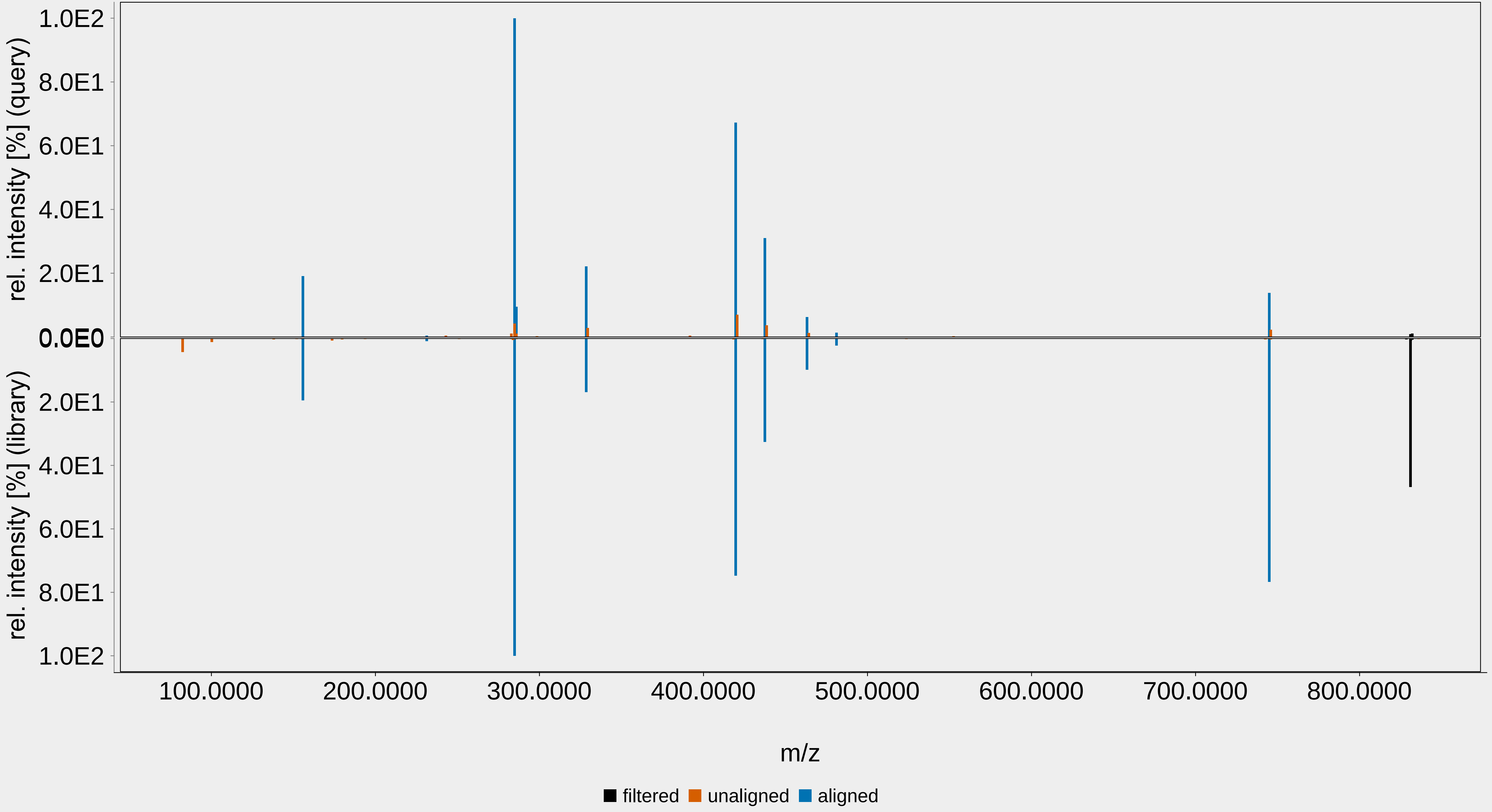

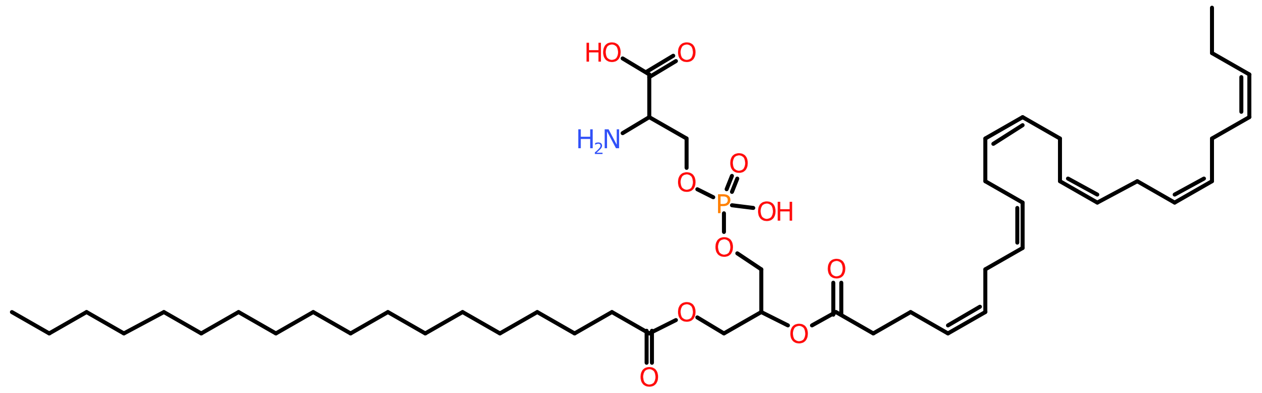

Compound information

Name: PS 40:6

Formula: C46H78NO10P

Mol. weight: 836.103

Exact mass: 836.103

Ion type: [M-H]-

Precursor m/z: 834.5288

RT: 8.68651

Polarity: negative

InChI:

InChI key:

SMILES:

Instrument information

Instrument type: LC-ESI-QTOF

Instrument: AB Sciex TripleTOF 5600+ system

Ms level: MS2

Collision energy: 45V

Database links

Pubchem: 91746251

MoNA ID: LQB00726

Other information

Principal investigator: Kazutaka Ikeda, Hiroshi

Data collector: Kazutaka Ikeda, Hiroshi

PS 40:6

0.892

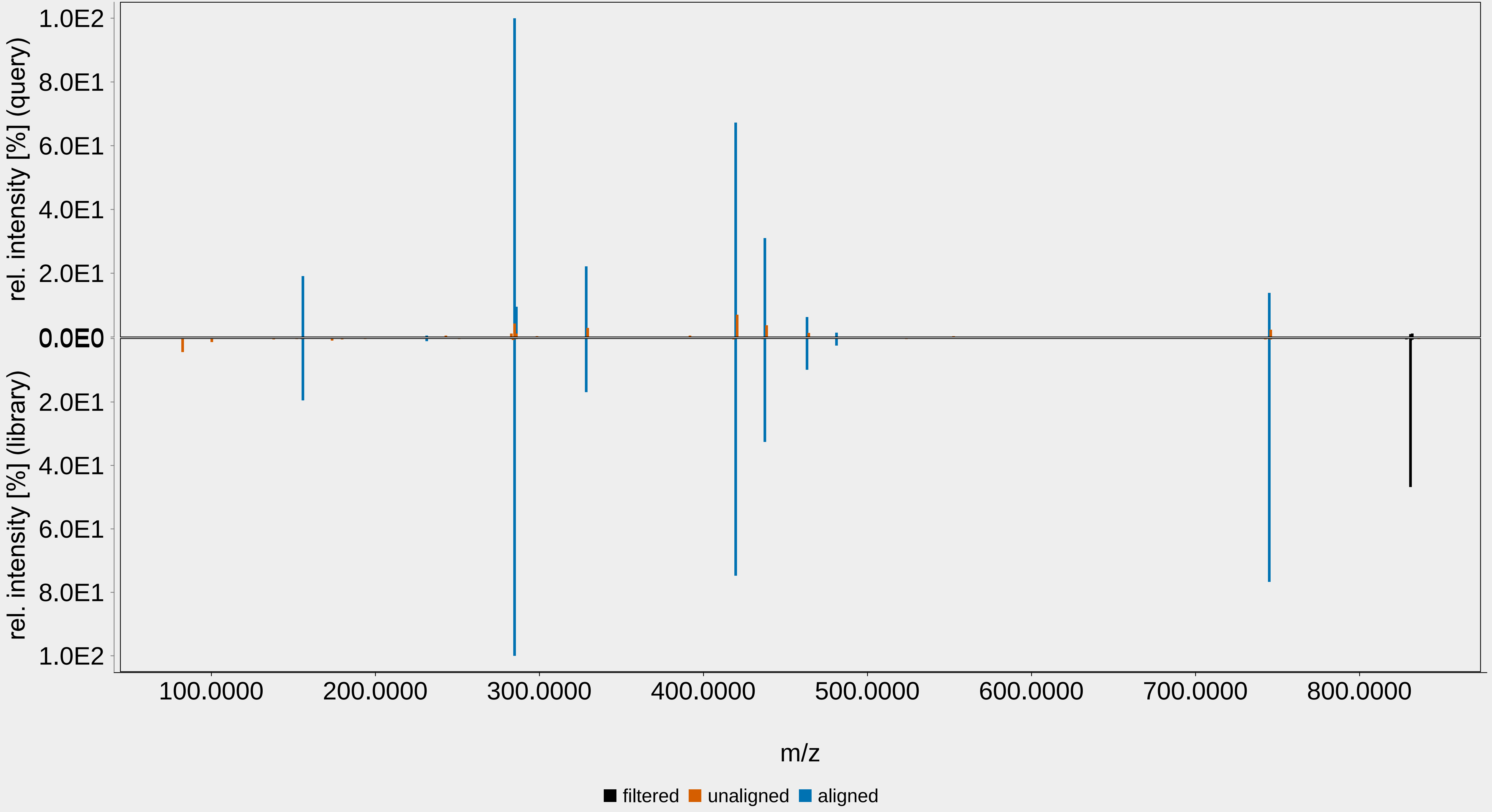

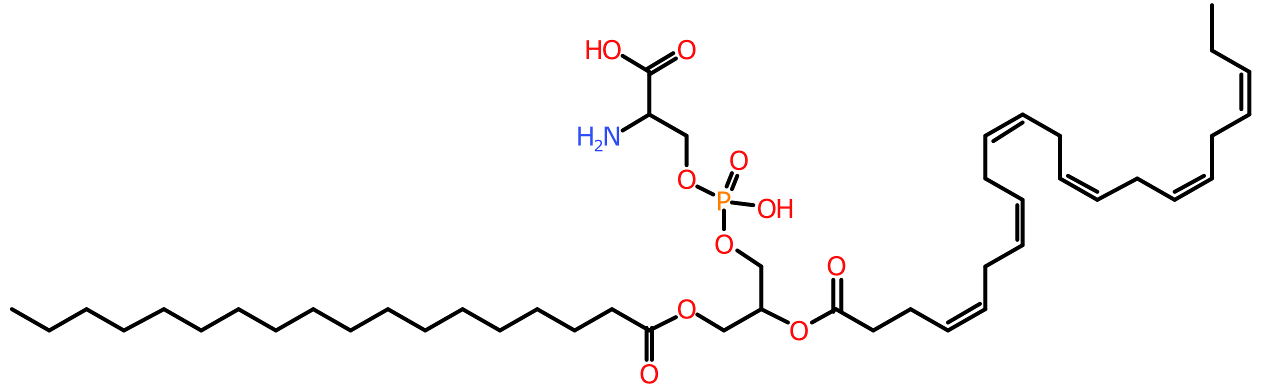

Compound information

Name: PS 40:6

Formula: C46H78NO10P

Mol. weight: 836.103

Exact mass: 836.103

Ion type: [M-H]-

Precursor m/z: 834.5288

RT: 8.68651

Polarity: negative

InChI:

InChI key:

SMILES:

Instrument information

Instrument type: LC-ESI-QTOF

Instrument: AB Sciex TripleTOF 5600+ system

Ms level: MS2

Collision energy: 45V

Database links

Pubchem: 91746251

MoNA ID: LQB00340

Other information

Principal investigator: Kazutaka Ikeda, Hiroshi

Data collector: Kazutaka Ikeda, Hiroshi

PS 18:1_20:4

0.755

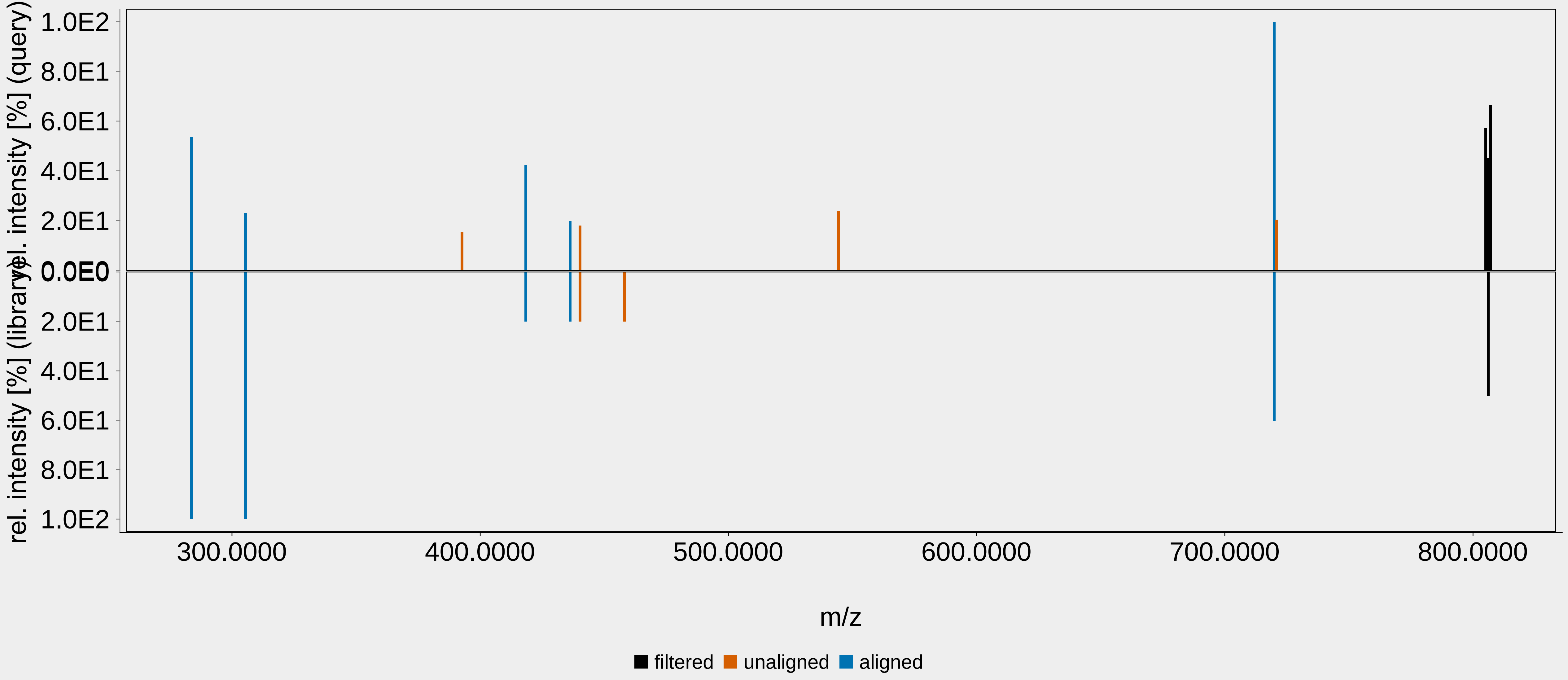

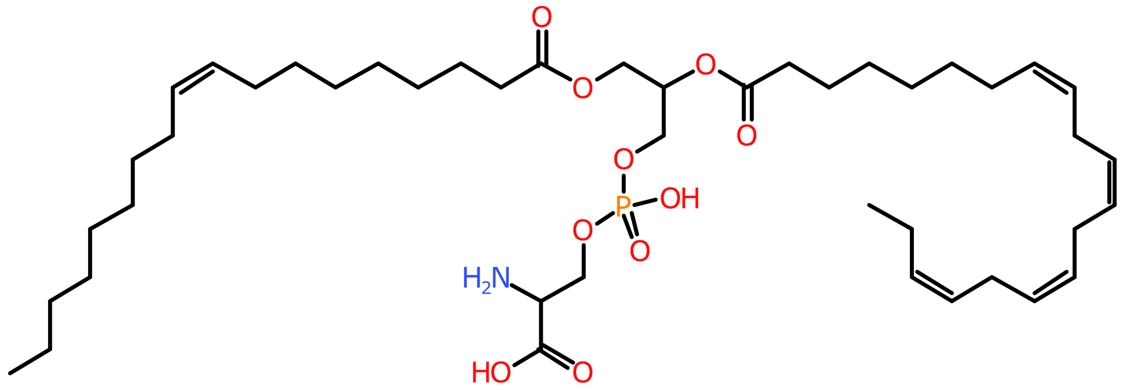

Compound information

Name: PS 18:1_20:4

Formula: C44H76NO10P

Ion type: [M-H]-

Precursor m/z: 808.51341

Ccs: 276.99

InChI key:

SMILES:

Num peaks: 8

Instrument information

Database links

Other information

PE(18:0/20:4); [M-H]- C43H77N1O8P1

0.903

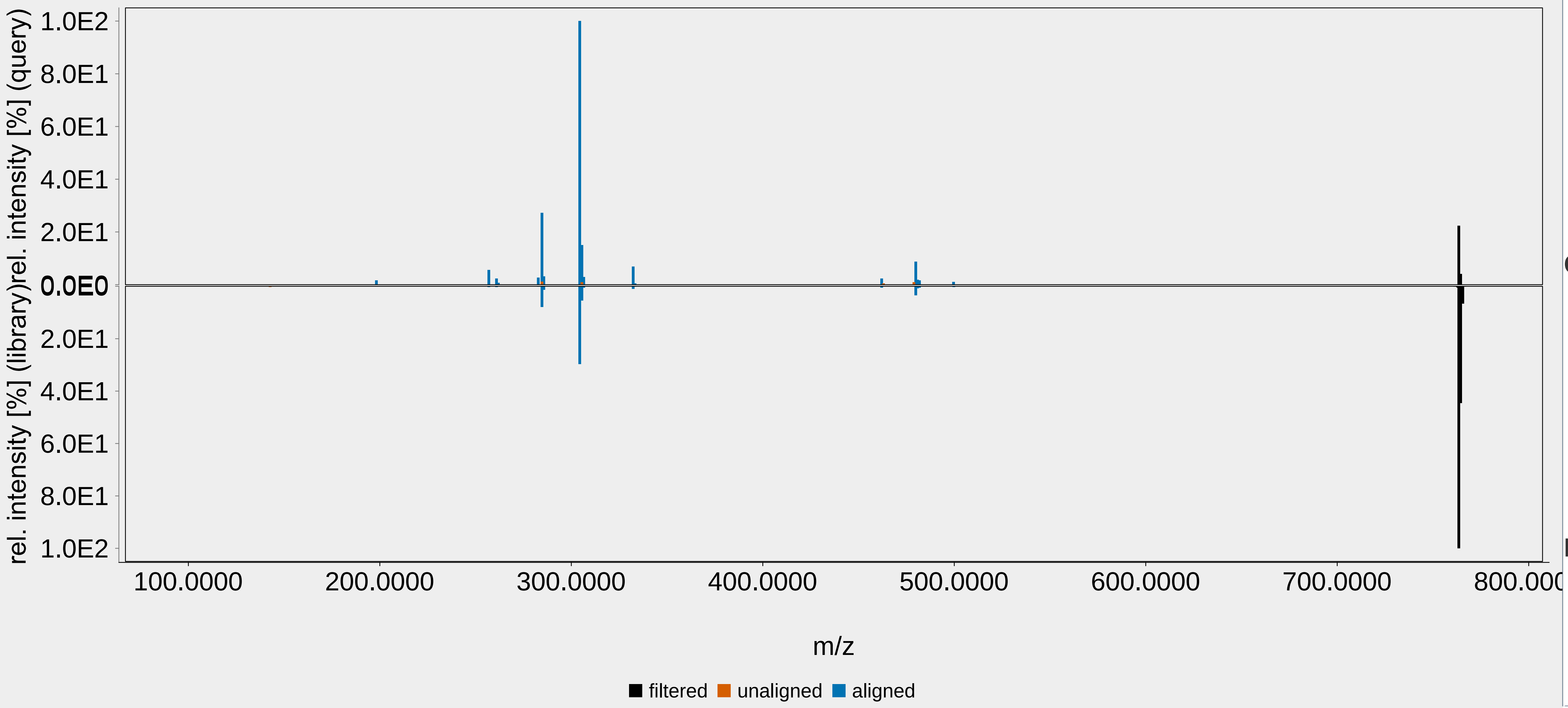

Compound information

Name: PE(18:0/20:4); [M-H]- C43H77N1O8P1

Exact mass: 0.0

Ion type: M-H

Precursor m/z: 766.539

Charge: 1

Polarity: Negative

Instrument information

Instrument type: HCD; Velos

Ion source: LC-ESI

Ms level: 2

Acquisition: Commercial

Database links

Other information

Principal investigator: Thomas Metz

Data collector: Thomas Metz

Entry ID: CCMSLIB00010074407

Spectral Match to 1-Octadecanoyl-2-(5Z,8Z,11Z,14Z-eicosatetraenoyl)-sn-glycero-3-phosphoethanolamine from NIST14

0.904

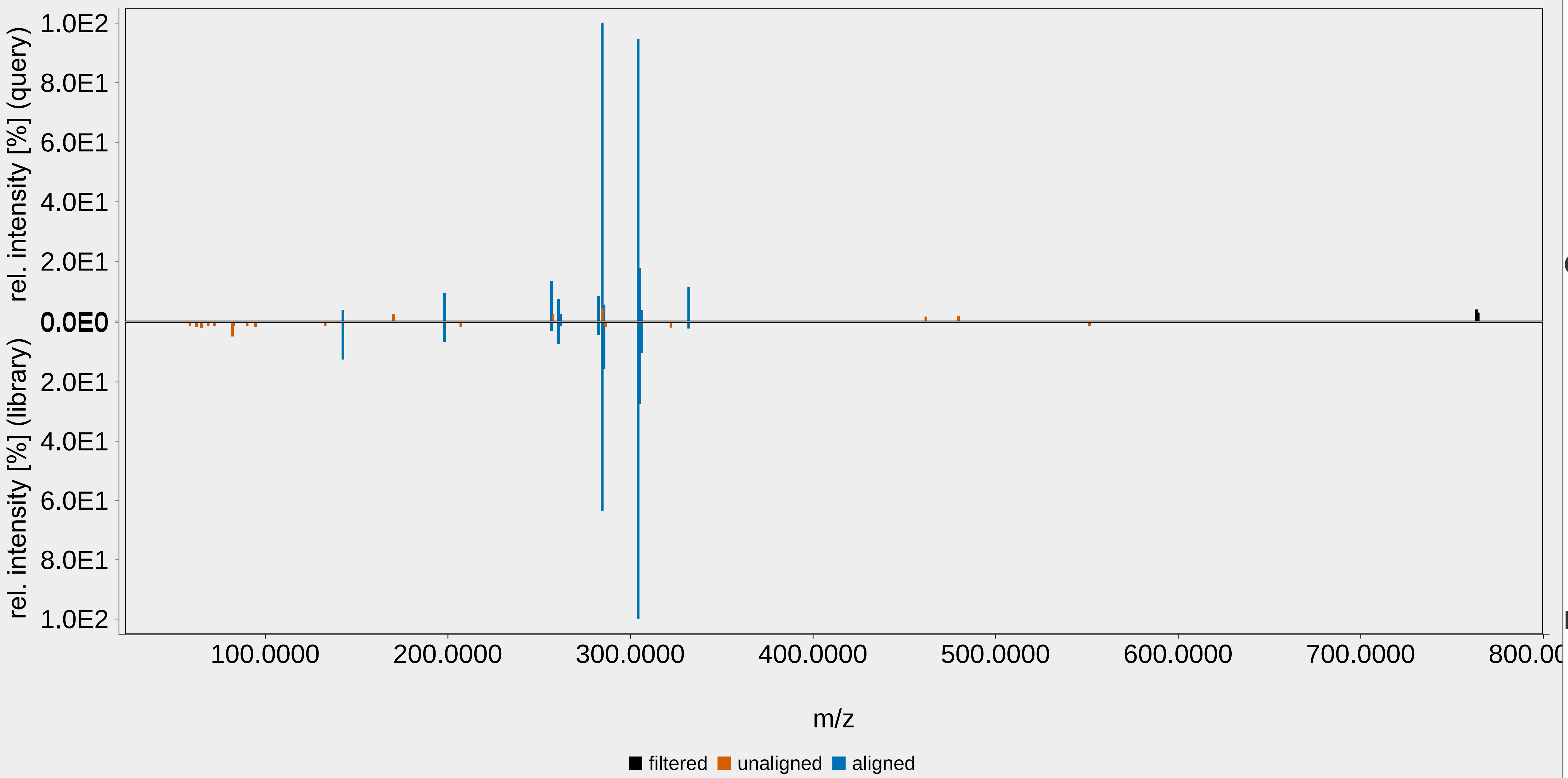

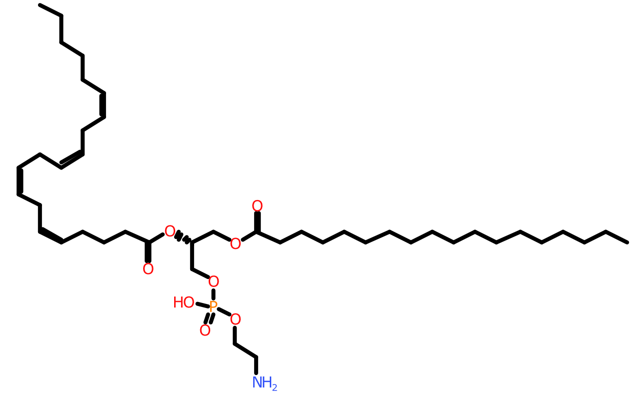

Compound information

Name: Spectral Match to

Exact mass: 767.547

Ion type: M-H

Precursor m/z: 766.541

Charge: 1

Polarity: Negative

InChI:

SMILES:

Instrument information

Instrument type: qTof

Ion source: ESI

Ms level: 2

Acquisition: Isolated

Database links

CAS: 61216624

Other information

Principal investigator: Data from Wiemann

Data collector: Data deposited by pwiemann

Entry ID: CCMSLIB00003139367

PE(16:1/18:1); [M-H]- C39H73N1O8P1 **0.892**

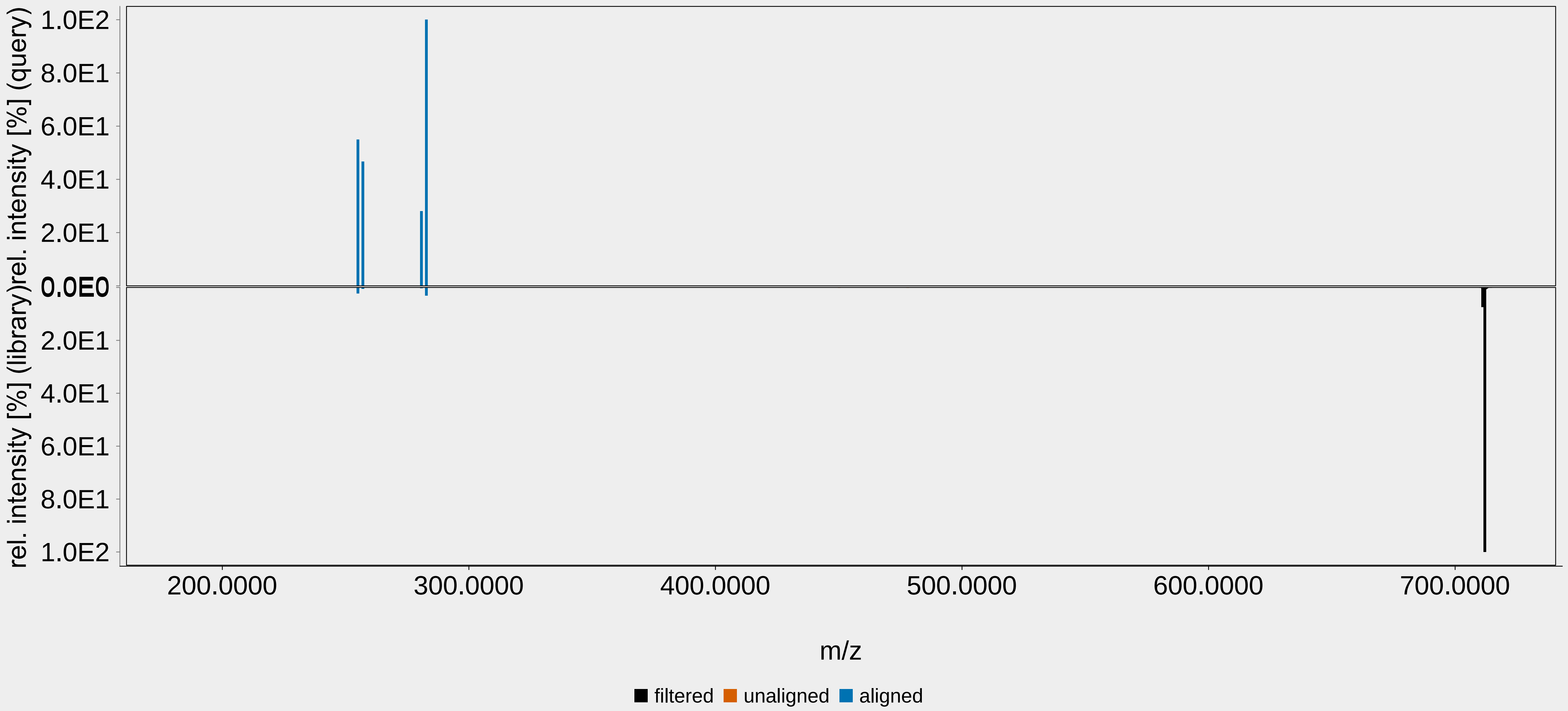

Compound information

Name: PE(16:1/18:1); [M-H]- C39H73N1O8P1

Exact mass: 0.0

Ion type: M-H

Precursor m/z: 714.507

Charge: 1

Polarity: Negative

Instrument information

Instrument type: HCD; Velos

Ion source: LC-ESI

Ms level: 2

Acquisition: Commercial

Database links

Other information

Principal investigator: Thomas Metz

Data collector: Thomas Metz

Entry ID: CCMSLIB00010060068

PE(16:1/18:1); [M-H]- C39H73N1O8P1

0.902

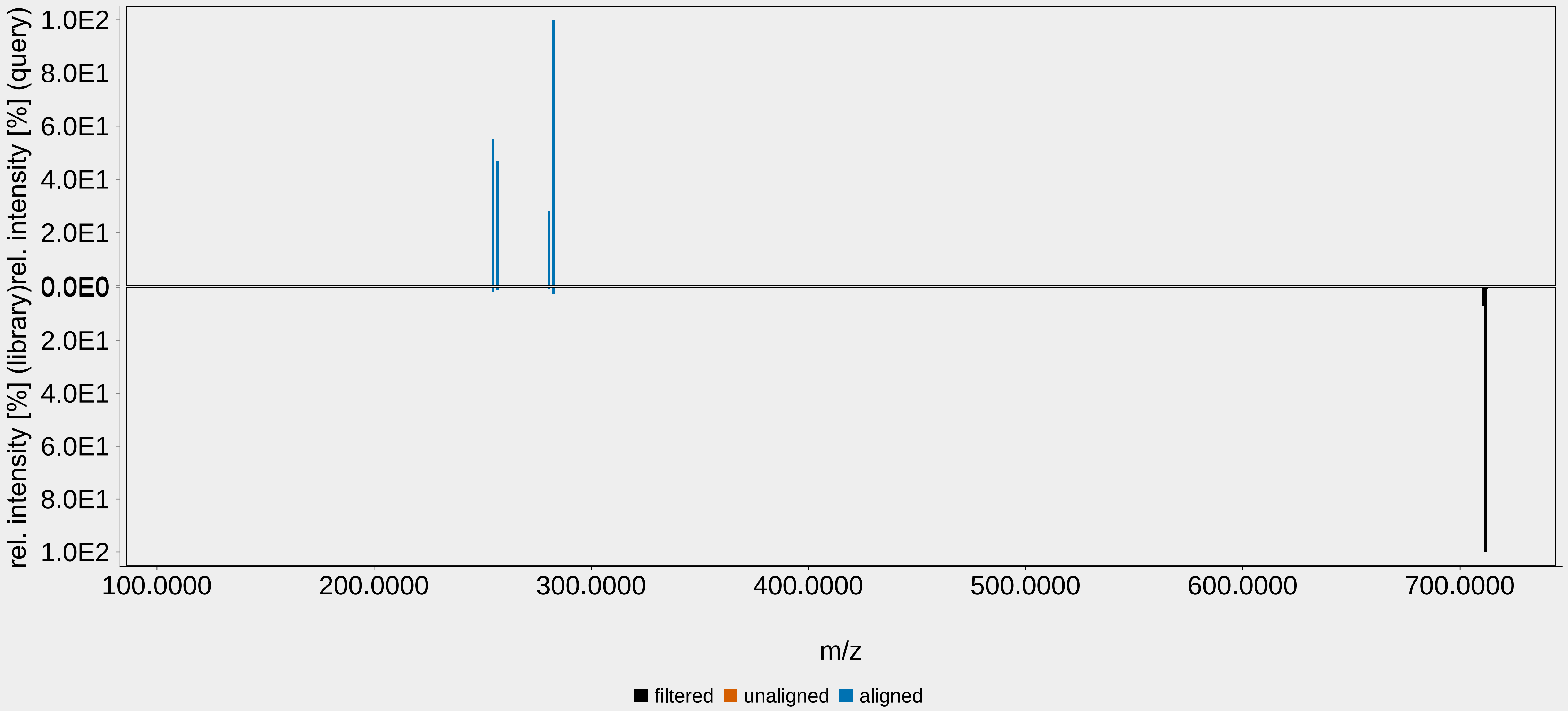

Compound information

Name: PE(16:1/18:1); [M-H]- C39H73N1O8P1

Exact mass: 0.0

Ion type: M-H

Precursor m/z: 714.507

Charge: 1

Polarity: Negative

Instrument information

Instrument type: HCD; Velos

Ion source: LC-ESI

Ms level: 2

Acquisition: Commercial

Database links

Other information

Principal investigator: Thomas Metz

Data collector: Thomas Metz

Entry ID: CCMSLIB00010060185

HYDROQUINIDINE 0.882

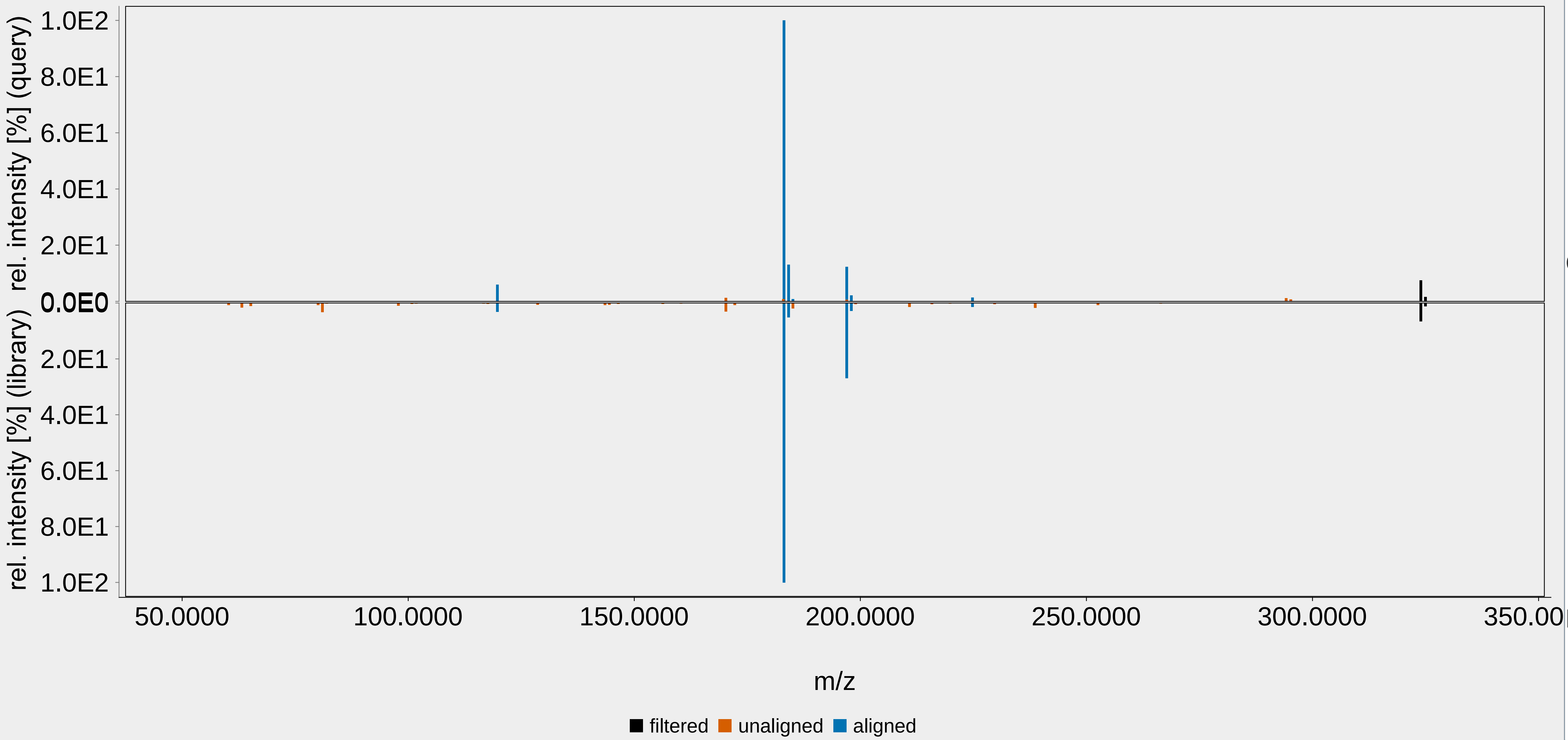

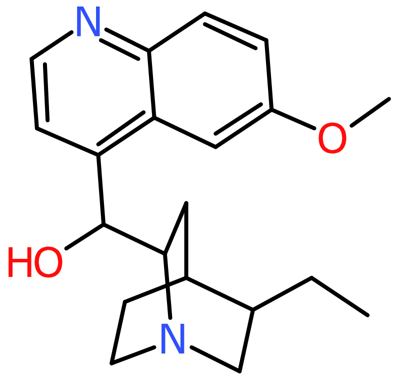

Compound information

Name: HYDROQUINIDINE

Formula: C20H26N2O2

Ion type: [M-H]-

Precursor m/z: 325.192138671875

Polarity: negative

InChI:

InChI key:

SMILES:

Instrument information

Instrument type: ESI-QFT

Instrument: Thermo Q Exactive HF

Ms level: MS2

Collision energy: 65HCD

Database links

Other information

Principal investigator: Arpana Vaniya

Data collector: Arpana Vaniya

HYDROQUINIDINE 0.882

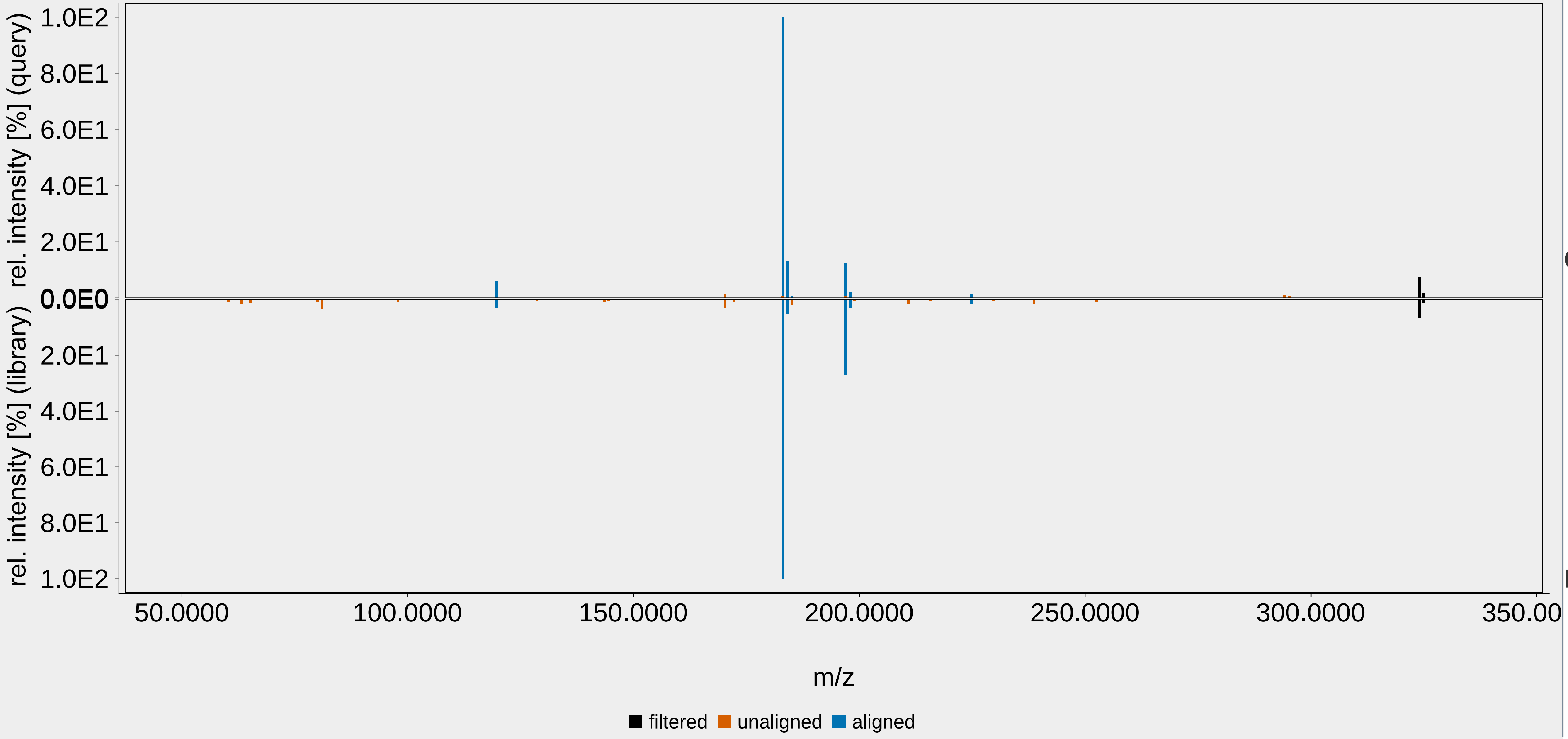

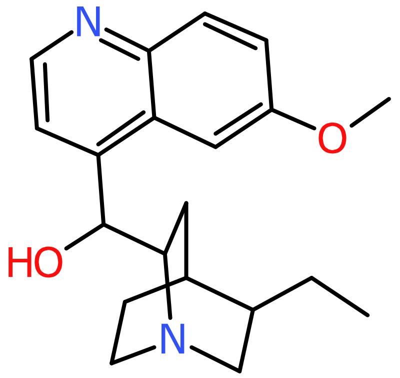

Compound information

Name: HYDROQUINIDINE

Exact mass: 0.0

Ion type: [M-H]-

Precursor m/z: 325.192

Charge: 1

Polarity: negative

InChI:

Instrument information

Instrument type: ESI-QFT

Ms level: 2

Acquisition: isolated

Database links

Other information

Principal investigator: MoNA

Data collector: MoNA:VF-NPL-QEHF003075

Entry ID: CCMSLIB00004694138

Crocetin semialdehyde

0.746

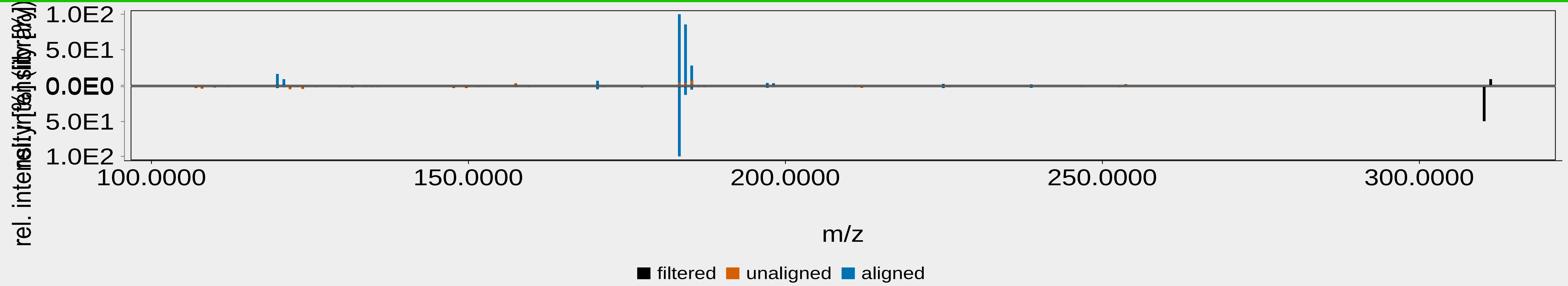

Compound information

Name: Crocetin semialdehyde

Ion type: carotenoid

Precursor m/z: 312.1725

Polarity: negative

Instrument information

Ion source: Electrospray Ionization (ESI)

Ms level: MS2

Database links

Other information

PA 24:0_18:1

0.864

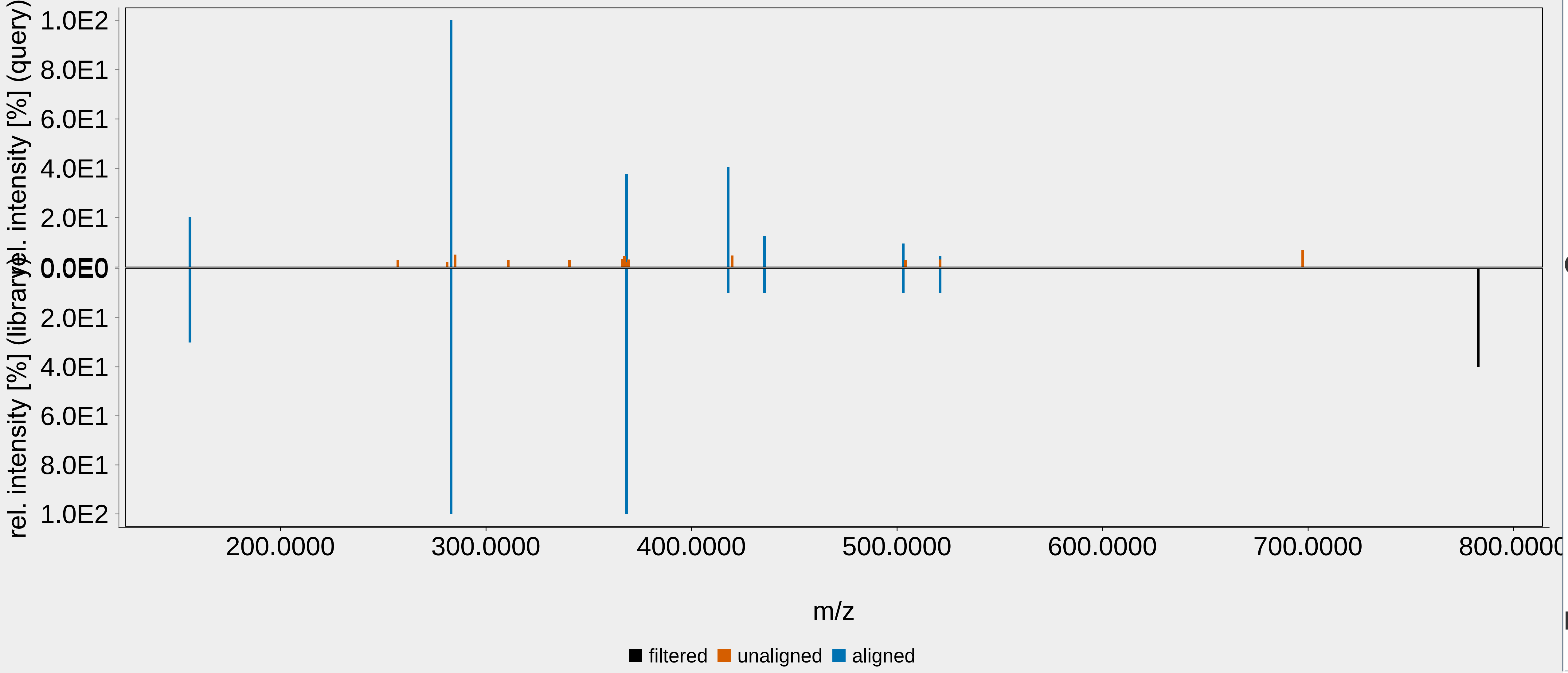

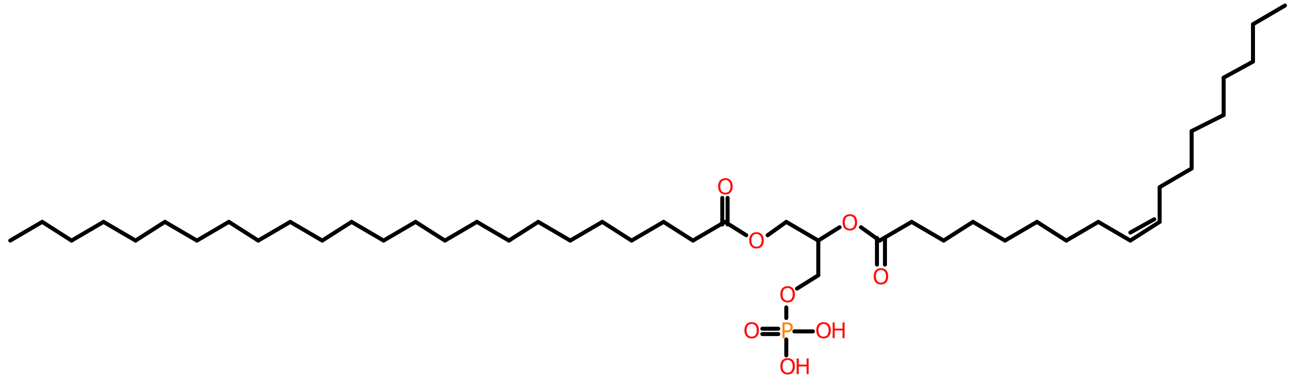

Compound information

Name: PA 24:0_18:1

Formula: C45H87O8P

Ion type: [M-H]-

Precursor m/z: 785.60658

Ccs: 278.64

InChI key:

SMILES:

Num peaks: 8

Instrument information

Database links

Other information

PS 36:1 **0.744**

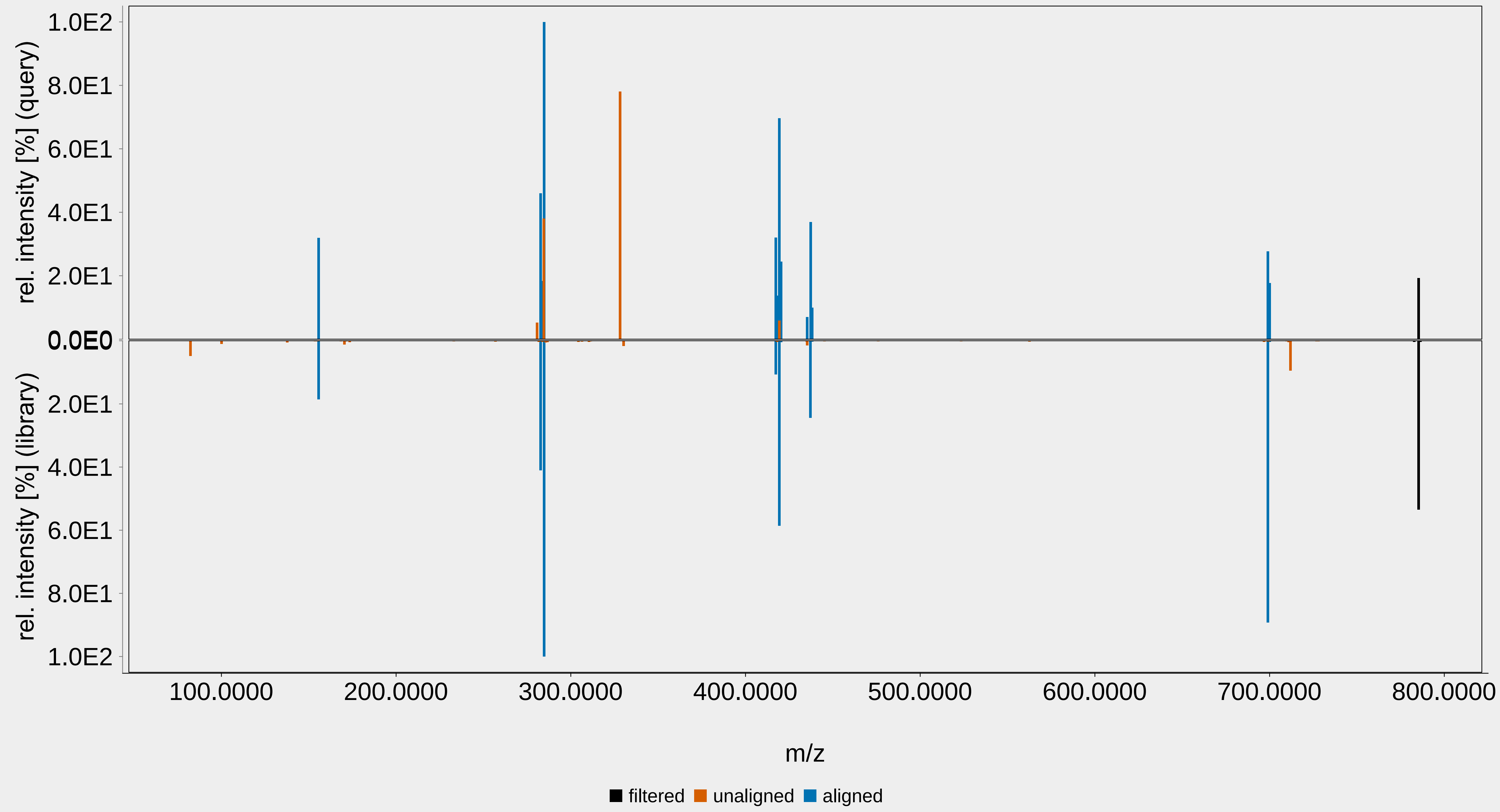

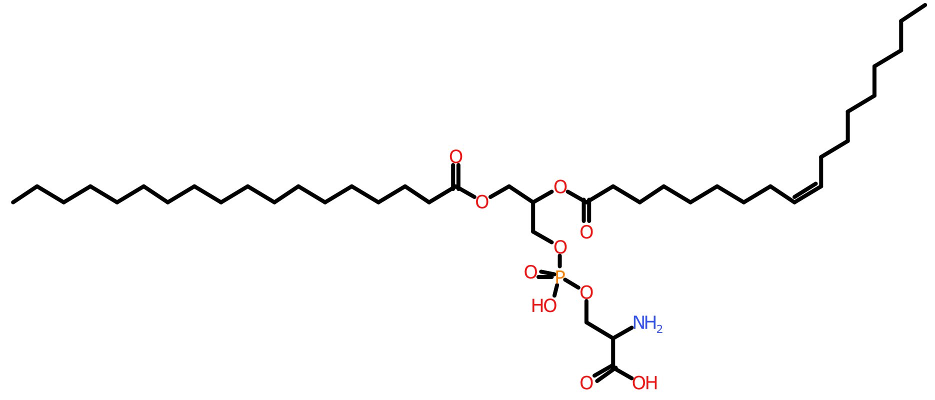

Compound information

Name: PS 36:1

Formula: C42H80NO10P

Mol. weight: 790.0749999999999

Exact mass: 790.0749999999999

Ion type: [M-H]-

Precursor m/z: 788.5428

RT: 9.185796

Polarity: negative

InChI:

InChI key: AJFWREUFUPEYII-ZZEZOPTASA-N

SMILES:

Instrument information

Instrument type: LC-ESI-QTOF

Instrument: AB Sciex TripleTOF 5600+ system

Ms level: MS2

Collision energy: 45V

Database links

Pubchem: 9962404

MoNA ID: LQB00719

Other information

Principal investigator: Kazutaka Ikeda, Hiroshi

Data collector: Kazutaka Ikeda, Hiroshi

PS 36:1 **0.744**

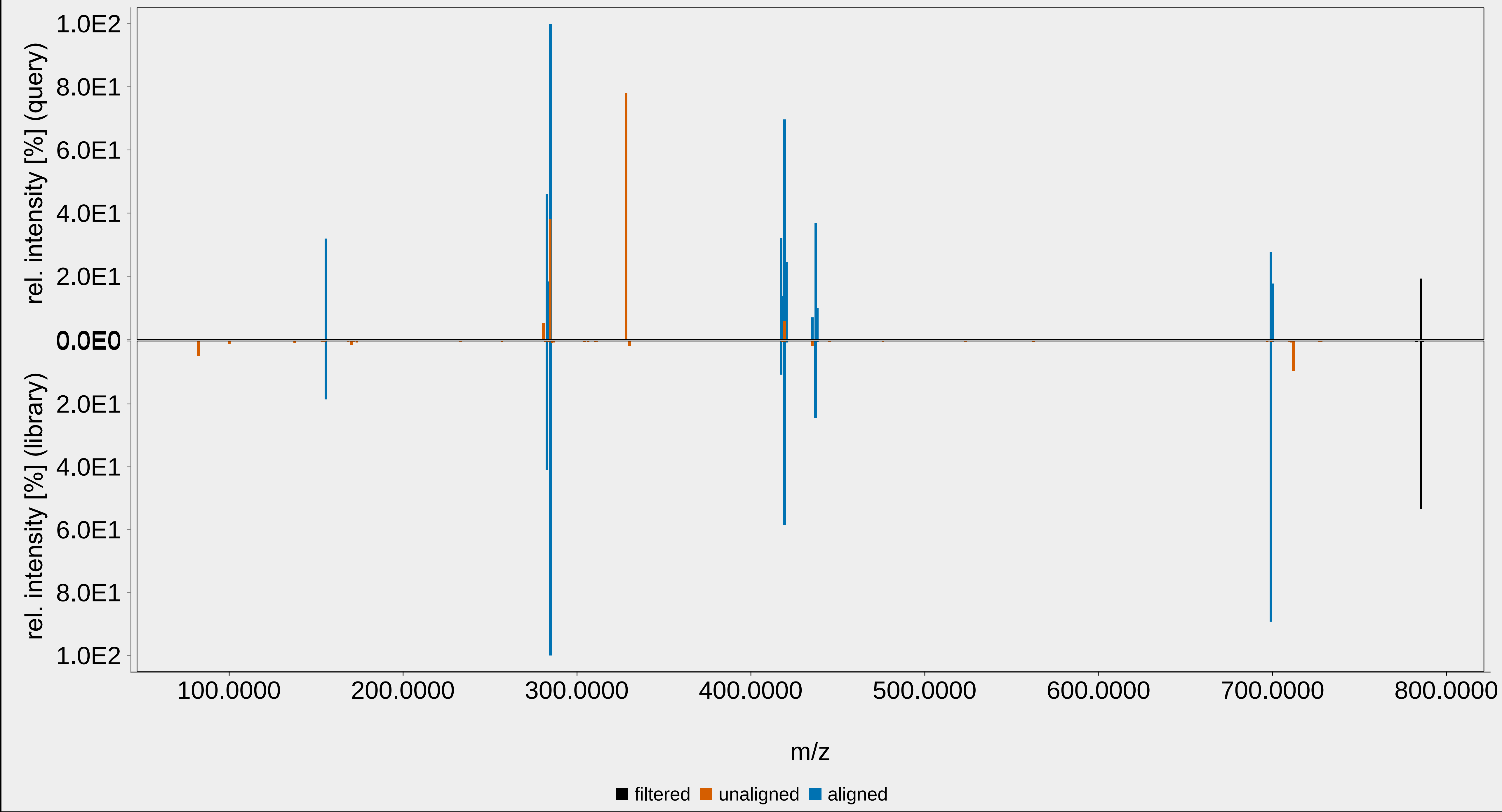

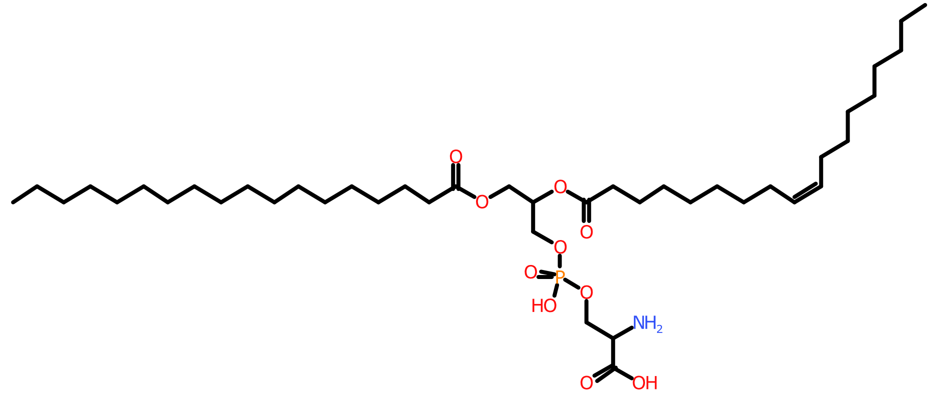

Compound information

Name: PS 36:1

Formula: C42H80NO10P

Mol. weight: 790.0749999999999

Exact mass: 790.0749999999999

Ion type: [M-H]-

Precursor m/z: 788.5428

RT: 9.185796

Polarity: negative

InChI:

InChI key: AJFWREUFUPEYII-ZZEZOPTASA-N

SMILES:

Instrument information

Instrument type: LC-ESI-QTOF

Instrument: AB Sciex TripleTOF 5600+ system

Ms level: MS2

Collision energy: 45V

Database links

Pubchem: 9962404

MoNA ID: LQB00333

Other information

Principal investigator: Kazutaka Ikeda, Hiroshi

Data collector: Kazutaka Ikeda, Hiroshi

PA 17:0_18:1 **0.739**

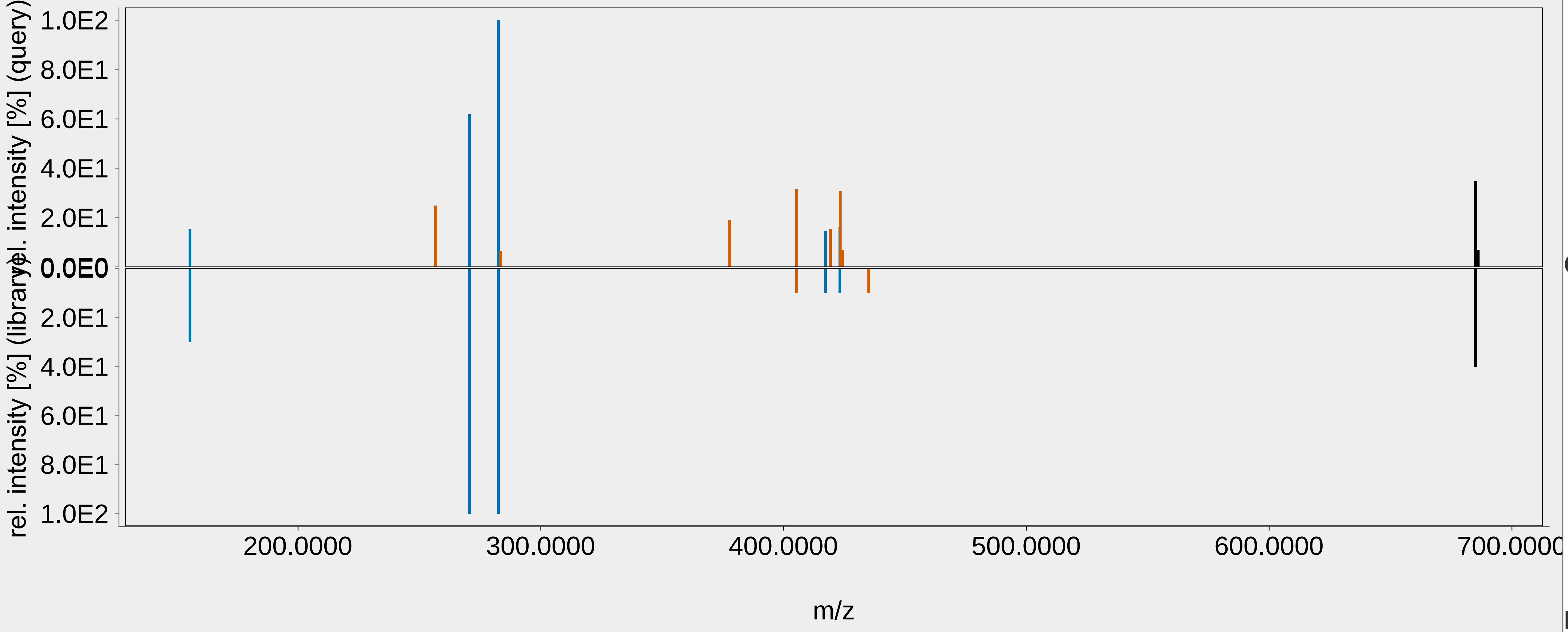

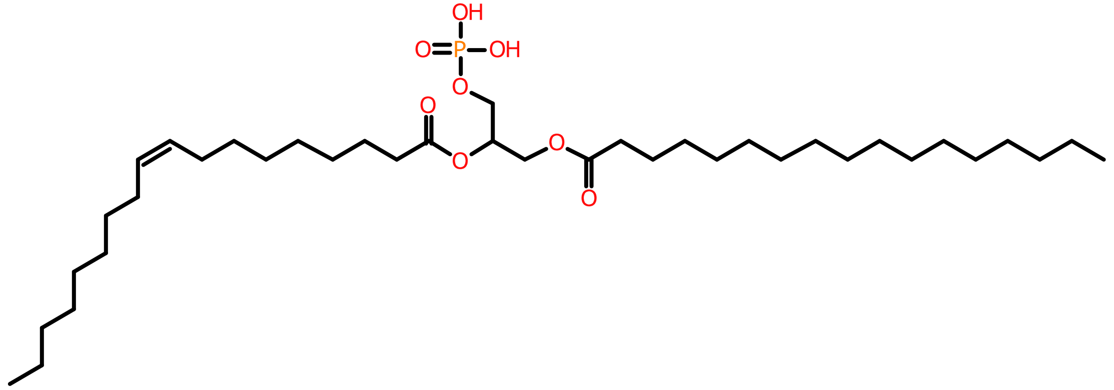

Compound information

Name: PA 17:0_18:1

Formula: C38H73O8P

Ion type: [M-H]-

Precursor m/z: 687.49703

Ccs: 257.18

InChI key:

SMILES:

Num peaks: 8

Instrument information

Database links

Other information

[3-[[2,3-dihydroxypropoxy]-hydroxyphosphoryl]oxy-2-hexadecanoyloxypropyl] hexadecanoate

0.856

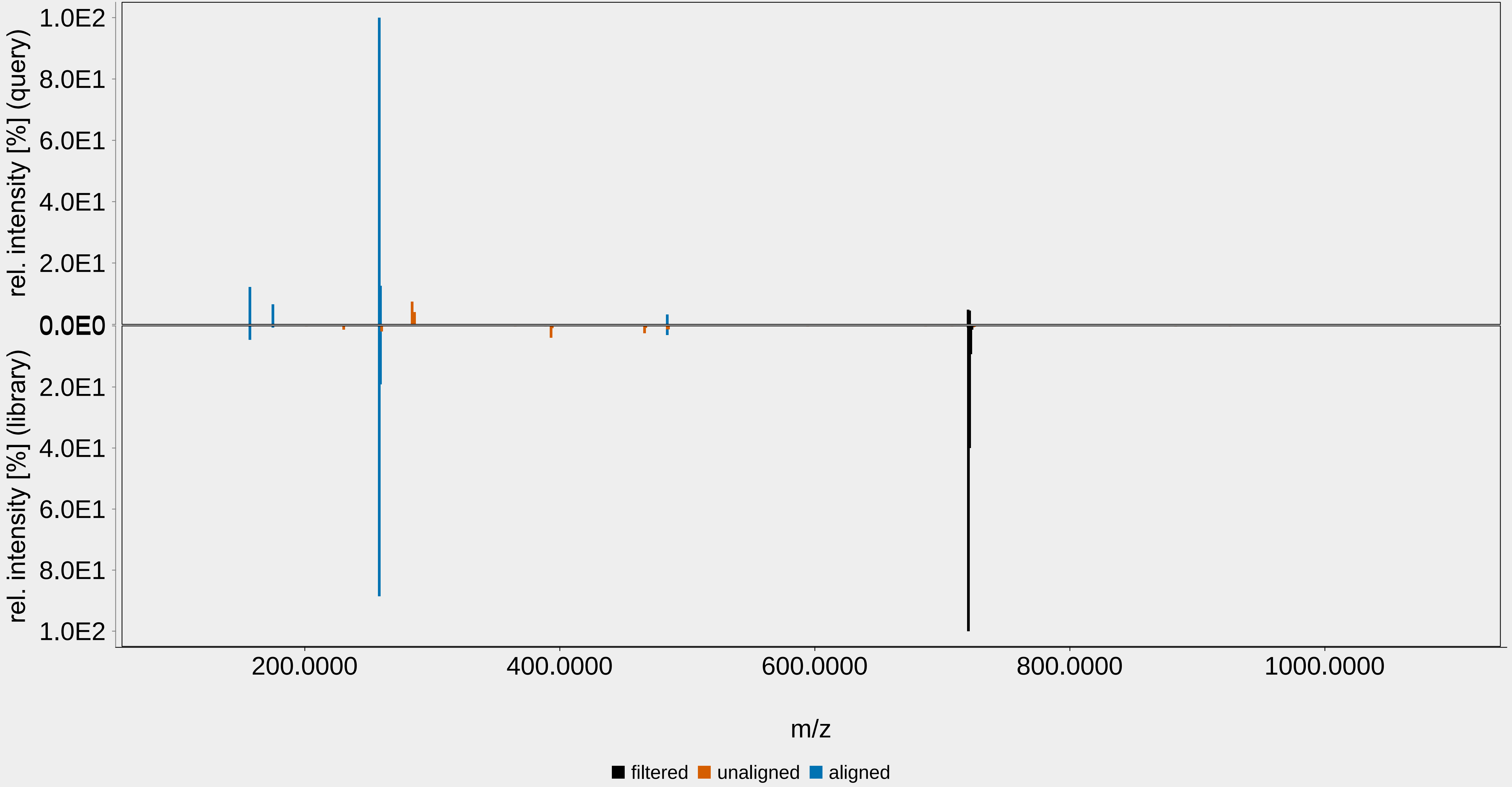

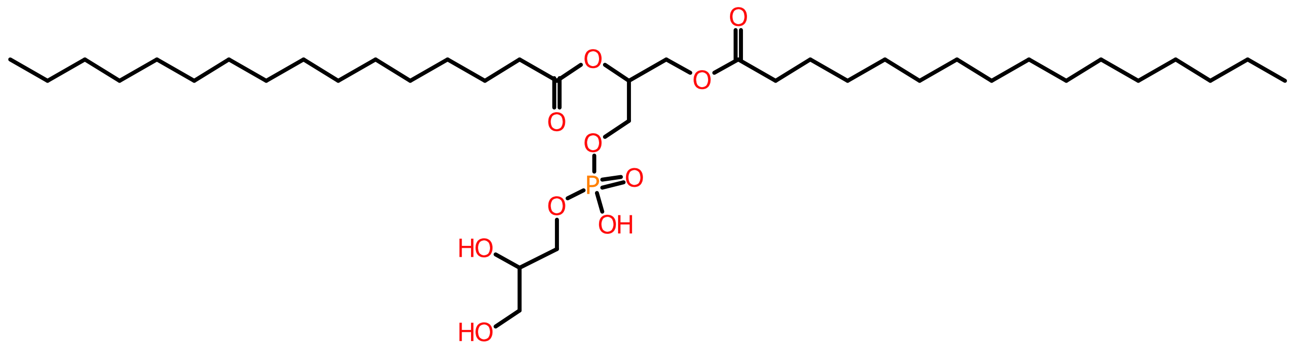

Compound information

Name:

Formula: C38H75O10P

Mol. weight: 722.51

Exact mass: 722.51

Ion type: [M-H]-

Precursor m/z: 721.502

Polarity: negative

InChI:

InChI key:

SMILES:

Instrument information

Instrument: qTof

Ms level: MS2

Database links

Other information

Principal investigator: O Laprevote, UMR 8038

Data collector: O Laprevote, UMR 8038

PG(16:0_16:0) - [3-[[2,3-dihydroxypropoxy]-hydroxyphosphoryl]oxy-2-hexadecanoyloxypropyl] hexadecanoate

0.856

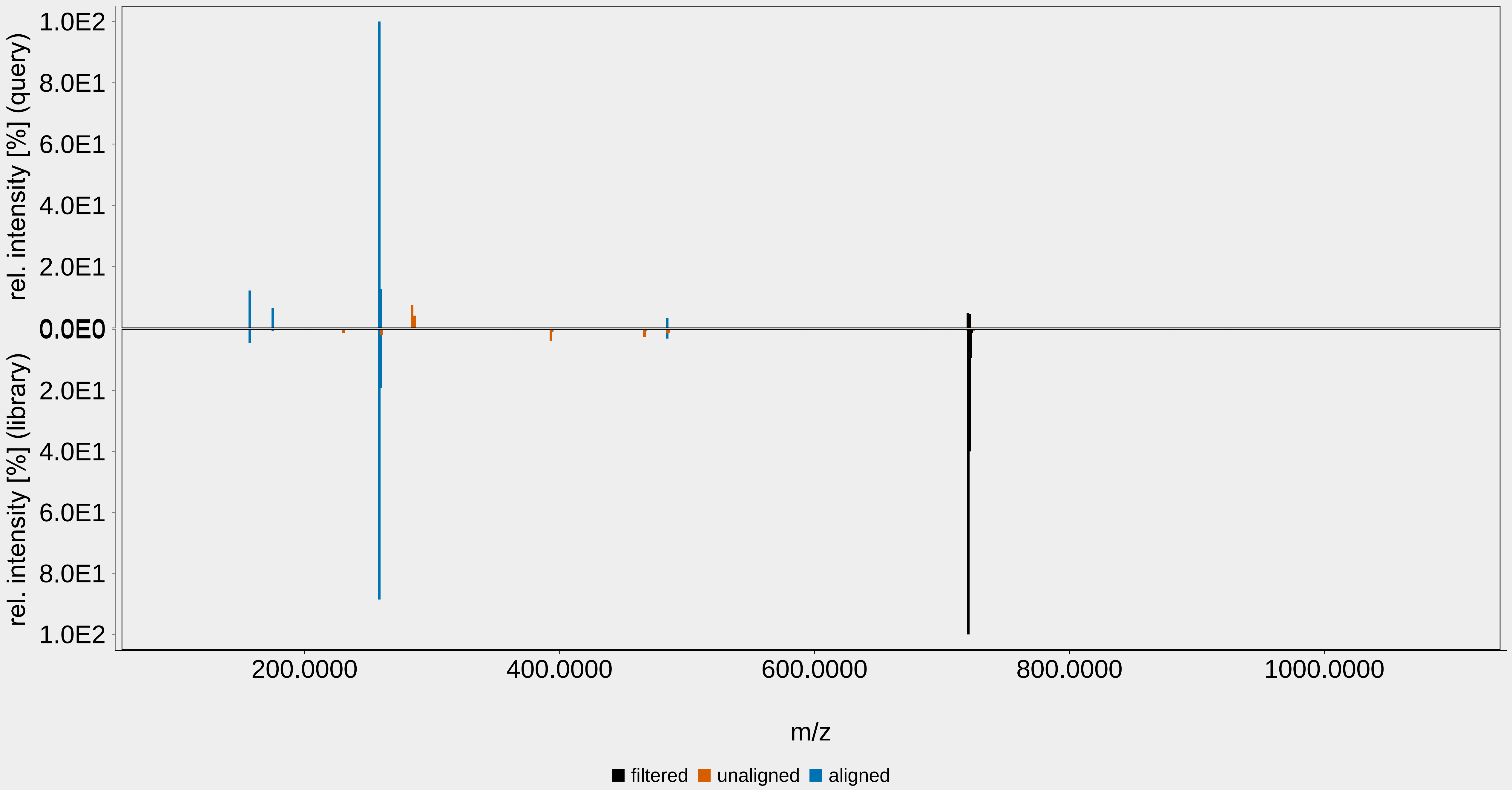

Compound information

Name: PG(16:0_16:0) -

Exact mass: 722.51

Ion type: M-H

Precursor m/z: 721.502

Charge: 1

Polarity: Negative

InChI:

InChI key:

SMILES:

Instrument information

Instrument type: qTof

Ion source: LC-ESI

Ms level: 2

Acquisition: Commercial

Database links

Pubmed:

CAS: 200880-41-7

Other information

Principal investigator: O Laprevote

Data collector: UMR 8038 Universite de Paris

Entry ID: CCMSLIB00005436447

PE(20:3/0:0); [M-H]- C25H45N1O7P1 0.853

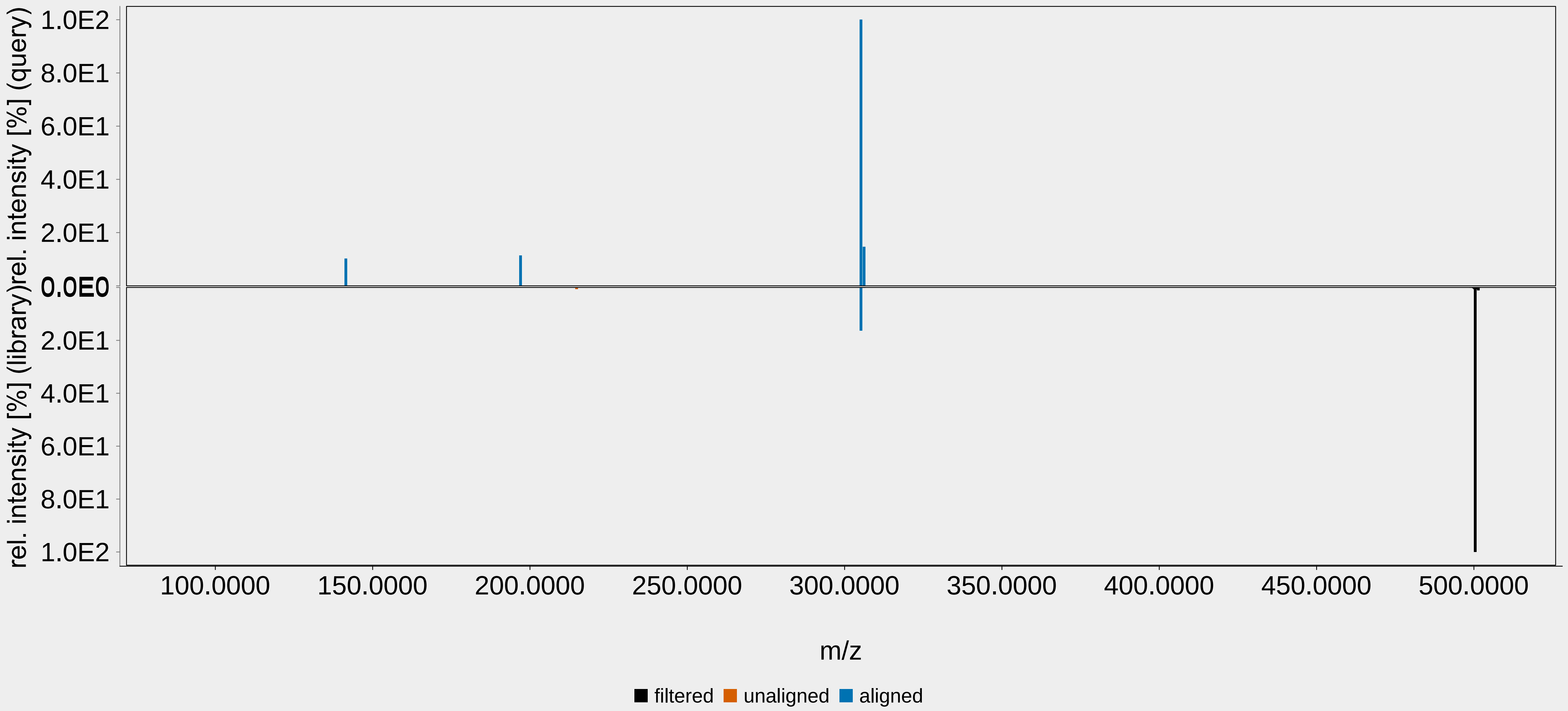

Compound information

Name: PE(20:3/0:0); [M-H]- C25H45N1O7P1

Exact mass: 0.0

Ion type: M-H

Precursor m/z: 502.293

Charge: 1

Polarity: Negative

Instrument information

Instrument type: HCD; Velos

Ion source: LC-ESI

Ms level: 2

Acquisition: Commercial

Database links

Other information

Principal investigator: Thomas Metz

Data collector: Thomas Metz

Entry ID: CCMSLIB00010080527

PE(20:3/0:0); [M-H]- C25H45N1O7P1 **0.868**

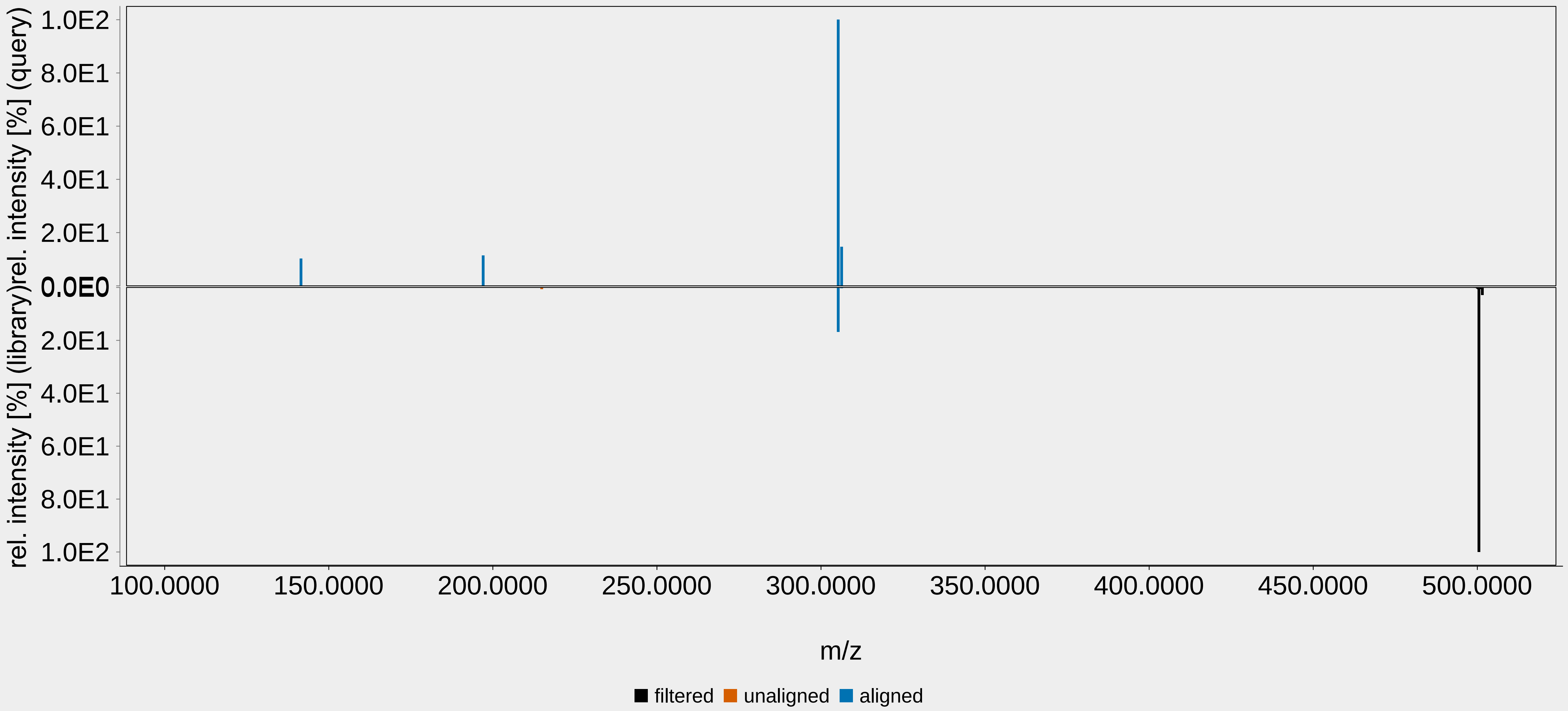

Compound information

Name: PE(20:3/0:0); [M-H]- C25H45N1O7P1

Exact mass: 0.0

Ion type: M-H

Precursor m/z: 502.293

Charge: 1

Polarity: Negative

Instrument information

Instrument type: HCD; Velos

Ion source: LC-ESI

Ms level: 2

Acquisition: Commercial

Database links

Other information

Principal investigator: Thomas Metz

Data collector: Thomas Metz

Entry ID: CCMSLIB00010079788

PE(18:1/18:2); [M-H]- C41H75N1O8P1 0.836

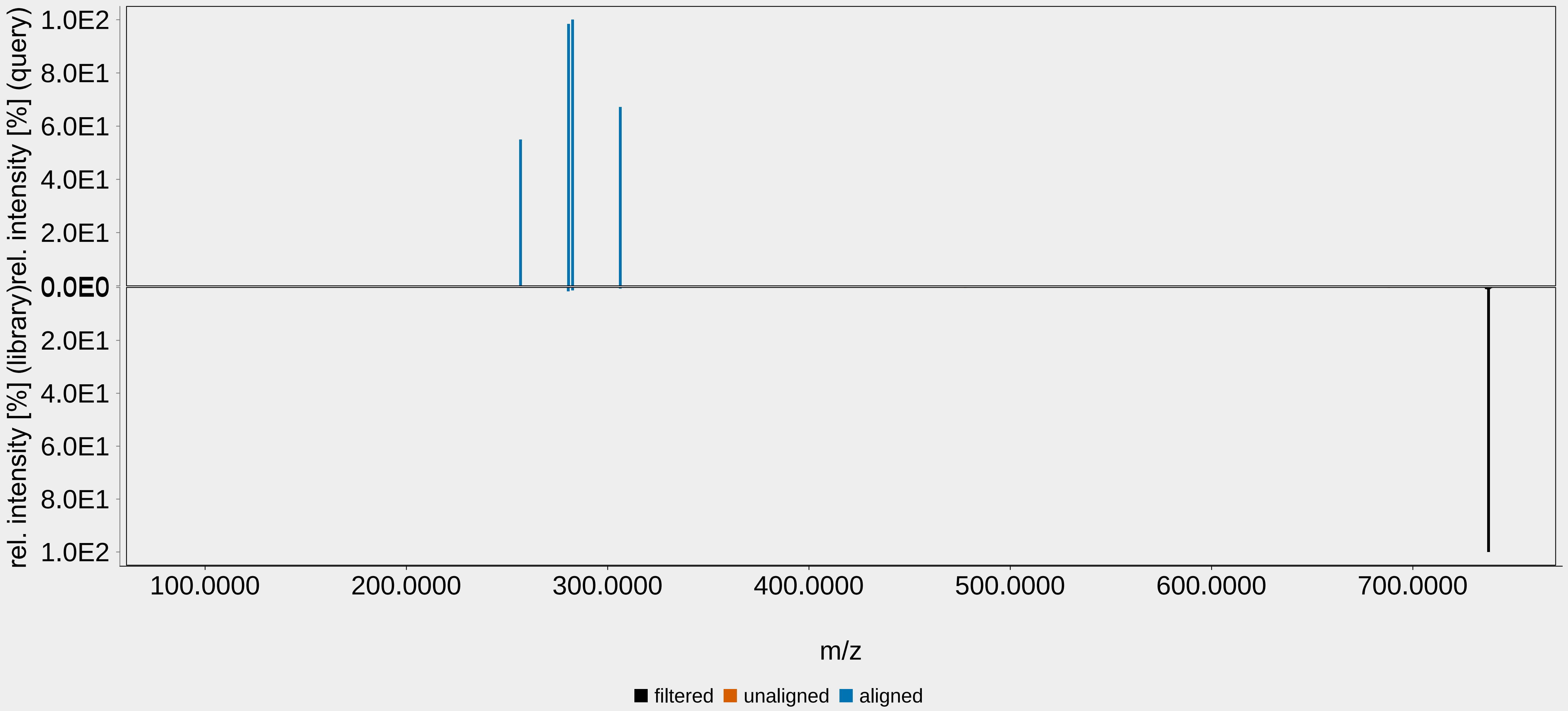

Compound information

Name: PE(18:1/18:2); [M-H]- C41H75N1O8P1

Exact mass: 0.0

Ion type: M-H

Precursor m/z: 740.523

Charge: 1

Polarity: Negative

Instrument information

Instrument type: HCD; Velos

Ion source: LC-ESI

Ms level: 2

Acquisition: Commercial

Database links

Other information

Principal investigator: Thomas Metz

Data collector: Thomas Metz

Entry ID: CCMSLIB00010076756

PE(18:1/18:2); [M-H]- C41H75N1O8P1 **0.880**

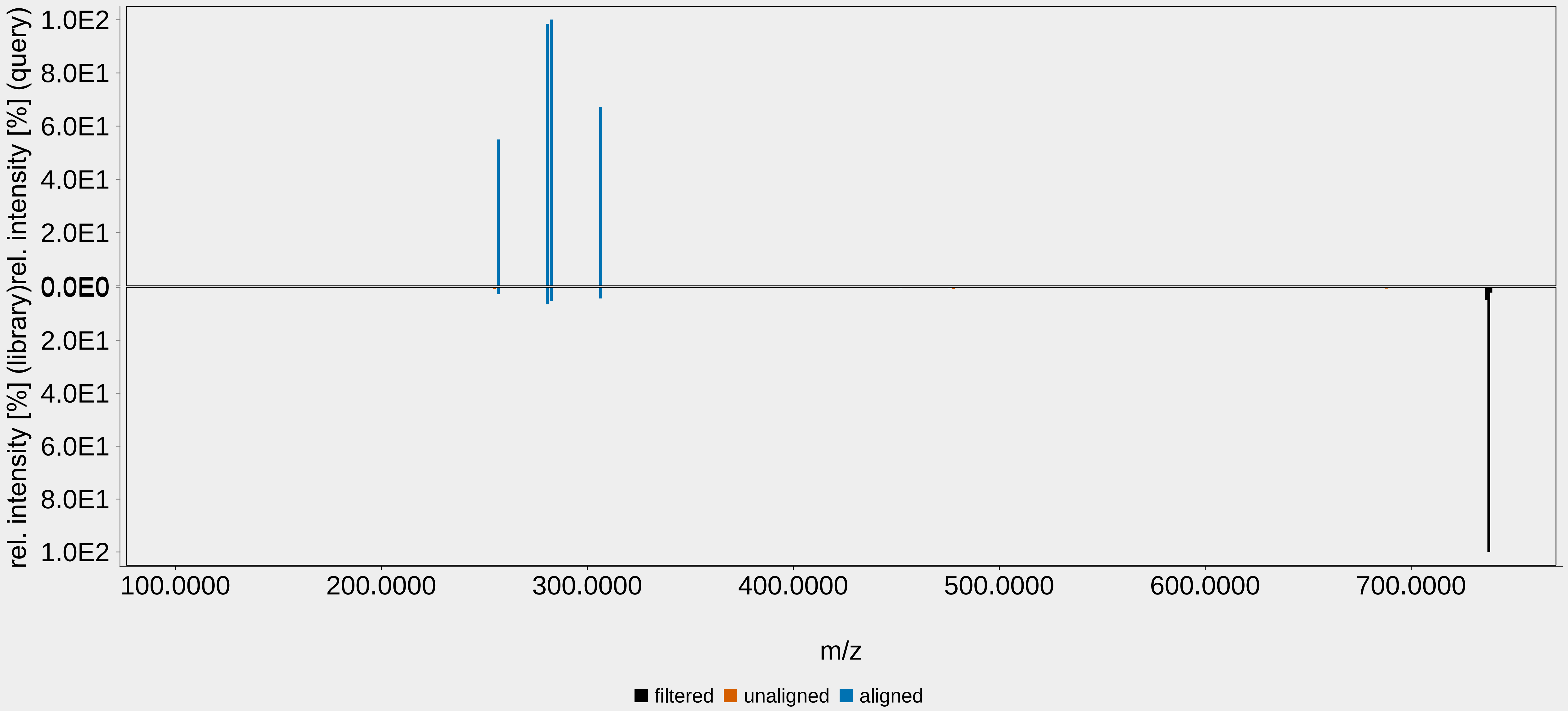

Compound information

Name: PE(18:1/18:2); [M-H]- C41H75N1O8P1

Exact mass: 0.0

Ion type: M-H

Precursor m/z: 740.523

Charge: 1

Polarity: Negative

Instrument information

Instrument type: HCD; Velos

Ion source: LC-ESI

Ms level: 2

Acquisition: Commercial

Database links

Other information

Principal investigator: Thomas Metz

Data collector: Thomas Metz

Entry ID: CCMSLIB00010071000

PS 18:0_20:3 0.753

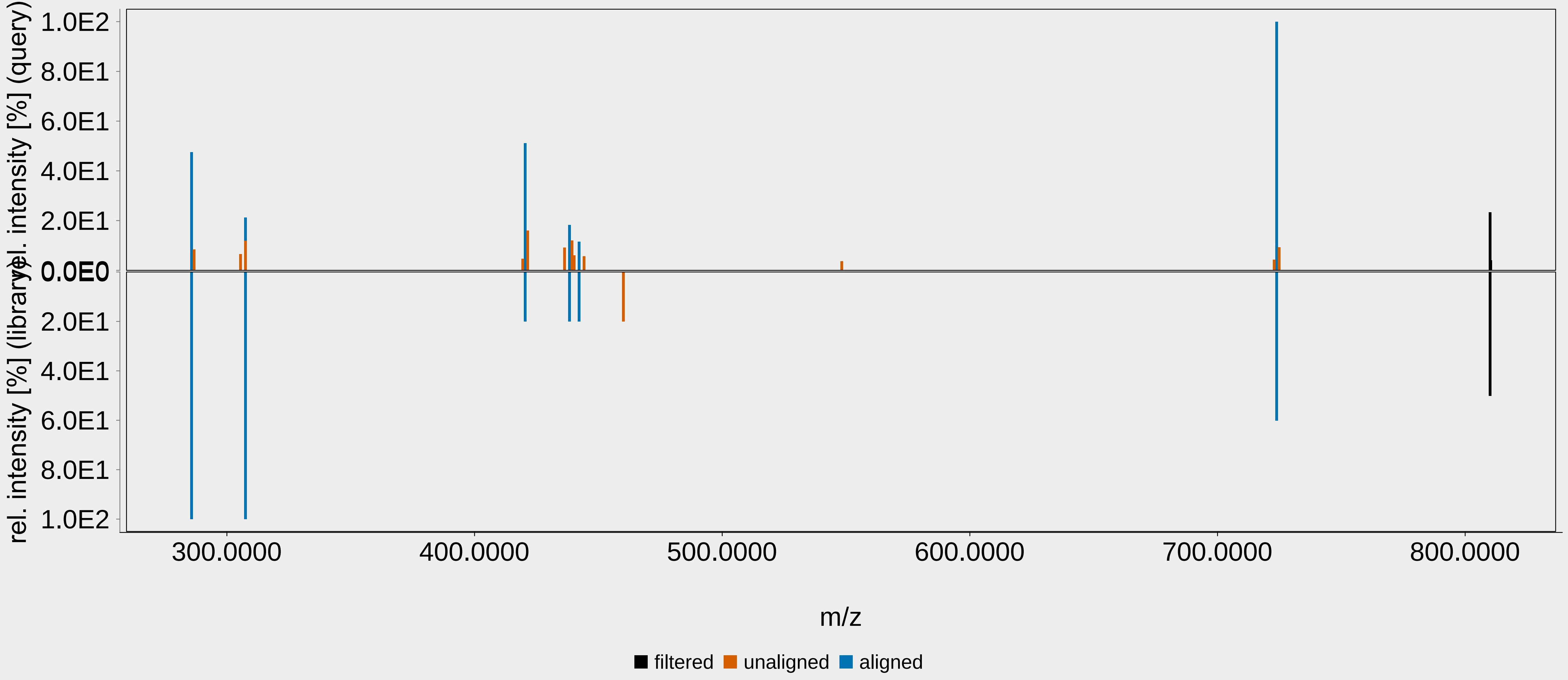

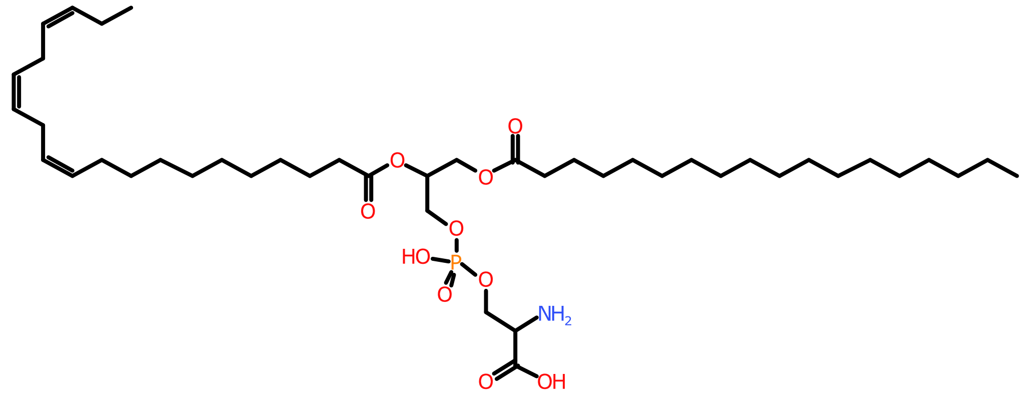

Compound information

Name: PS 18:0_20:3

Formula: C44H80NO10P

Ion type: [M-H]-

Precursor m/z: 812.54471

Ccs: 279.88

InChI key:

SMILES:

Num peaks: 8

Instrument information

Database links

Other information

PS(18:0/20:3); [M-H]- C44H79N1O10P1

0.863

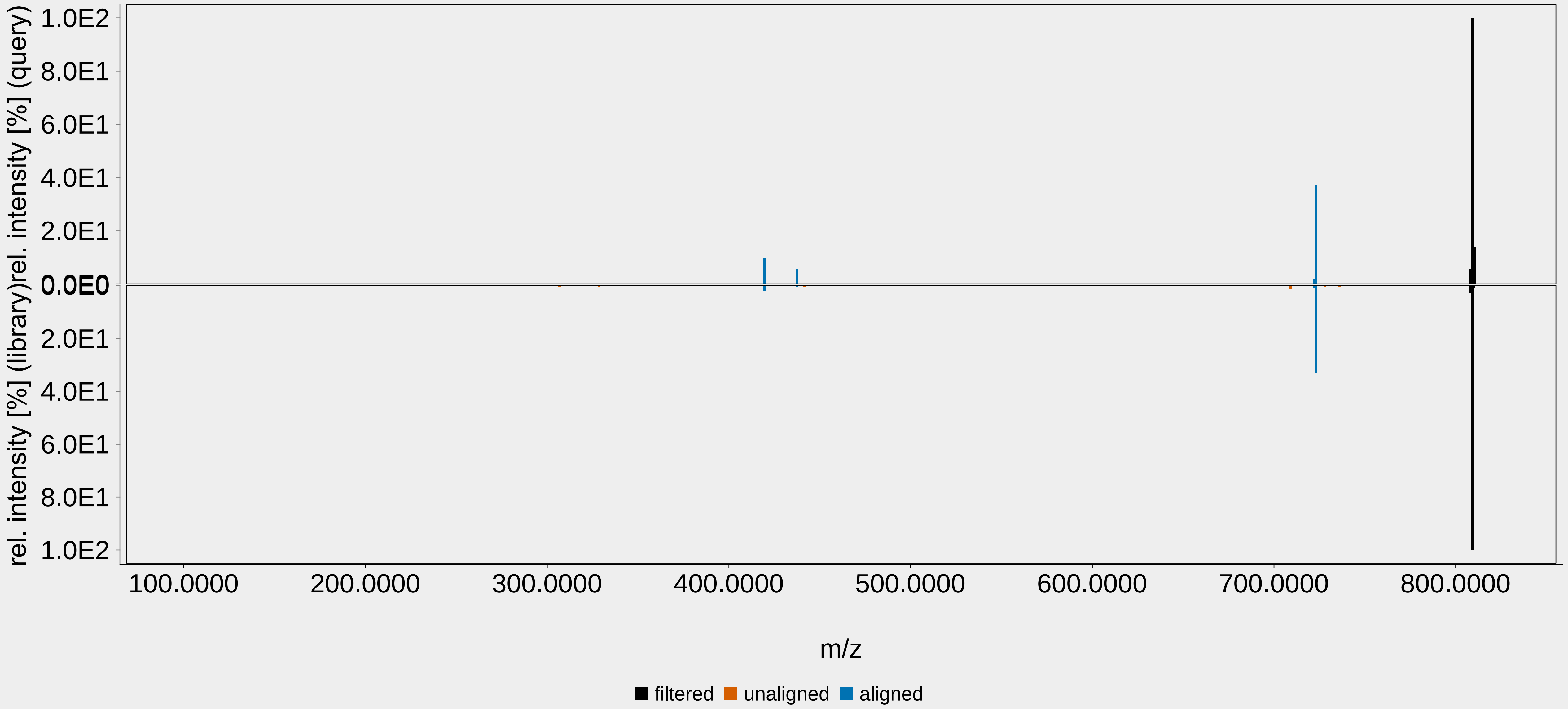

Compound information

Name: PS(18:0/20:3); [M-H]- C44H79N1O10P1

Exact mass: 0.0

Ion type: M-H

Precursor m/z: 812.544

Charge: 1

Polarity: Negative

Instrument information

Instrument type: HCD; Velos

Ion source: LC-ESI

Ms level: 2

Acquisition: Commercial

Database links

Other information

Principal investigator: Thomas Metz

Data collector: Thomas Metz

Entry ID: CCMSLIB00010077483

PE(20:2/0:0); [M-H]- C25H47N1O7P1 **0.925**

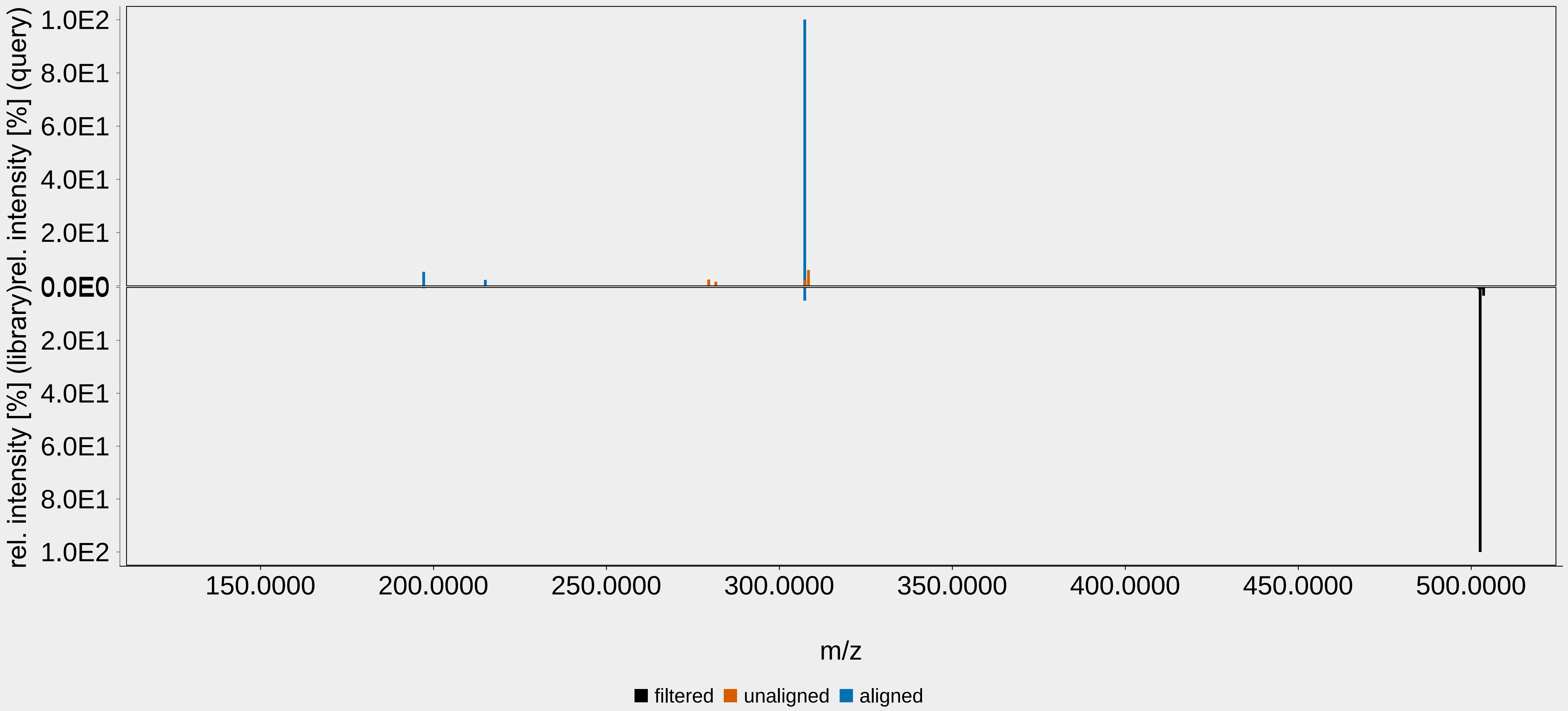

Compound information

Name: PE(20:2/0:0); [M-H]- C25H47N1O7P1

Exact mass: 0.0

Ion type: M-H

Precursor m/z: 504.309

Charge: 1

Polarity: Negative

Instrument information

Instrument type: HCD; Velos

Ion source: LC-ESI

Ms level: 2

Acquisition: Commercial

Database links

Other information

Principal investigator: Thomas Metz

Data collector: Thomas Metz

Entry ID: CCMSLIB00010086312

PS 36:4 **0.759**

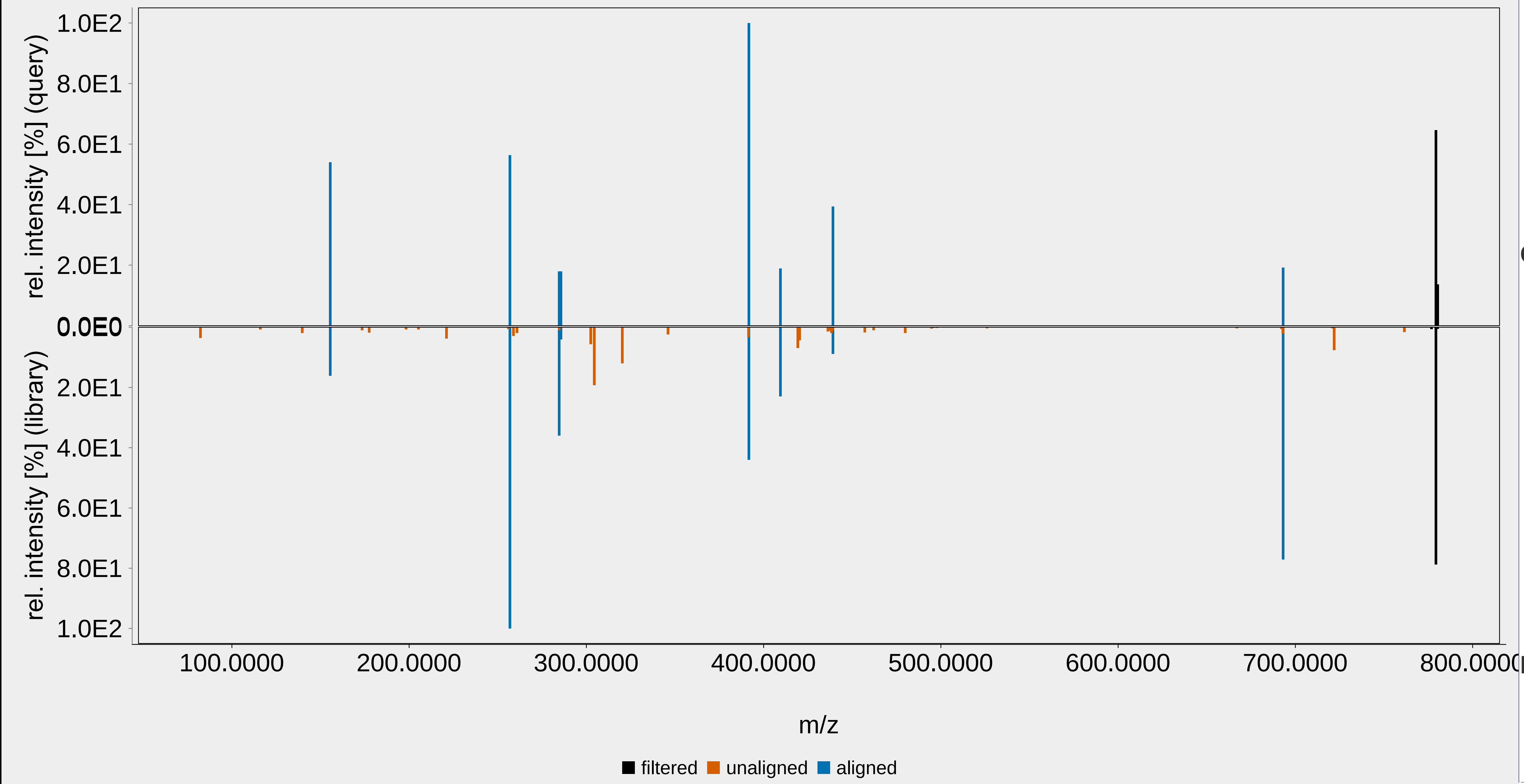

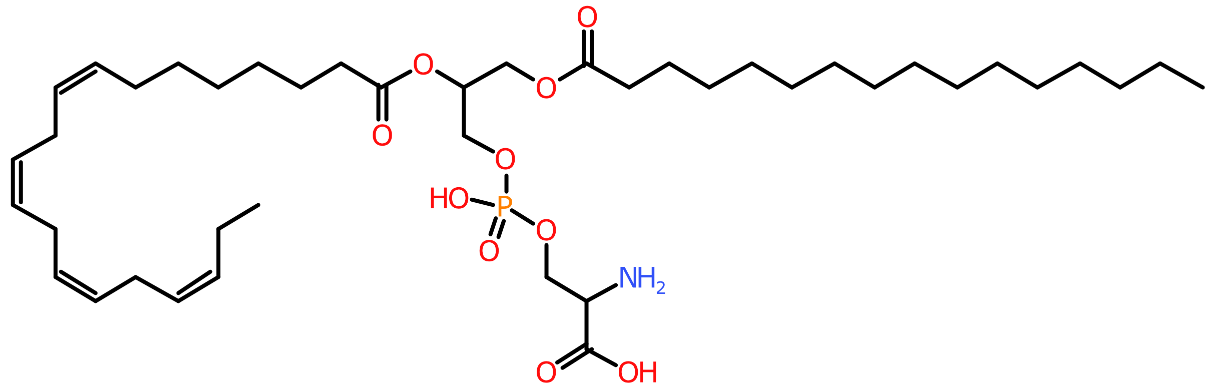

Compound information

Name: PS 36:4

Formula: C42H74NO10P

Exact mass: 784.0269999999999

Ion type: [M-H]-

Precursor m/z: 782.494

Polarity: NEGATIVE

InChI:

InChI key:

SMILES:

Num peaks: 55

Instrument information

Instrument type: LC-ESI-QTOF

Instrument: AB Sciex TripleTOF 5600+ system

Ms level: MS2

Collision energy: 45V

Database links

Other information

Entry ID: MSBNK-RIKEN_IMS-LQB00715

Comment: Parent=782.494

PS 36:4 0.759

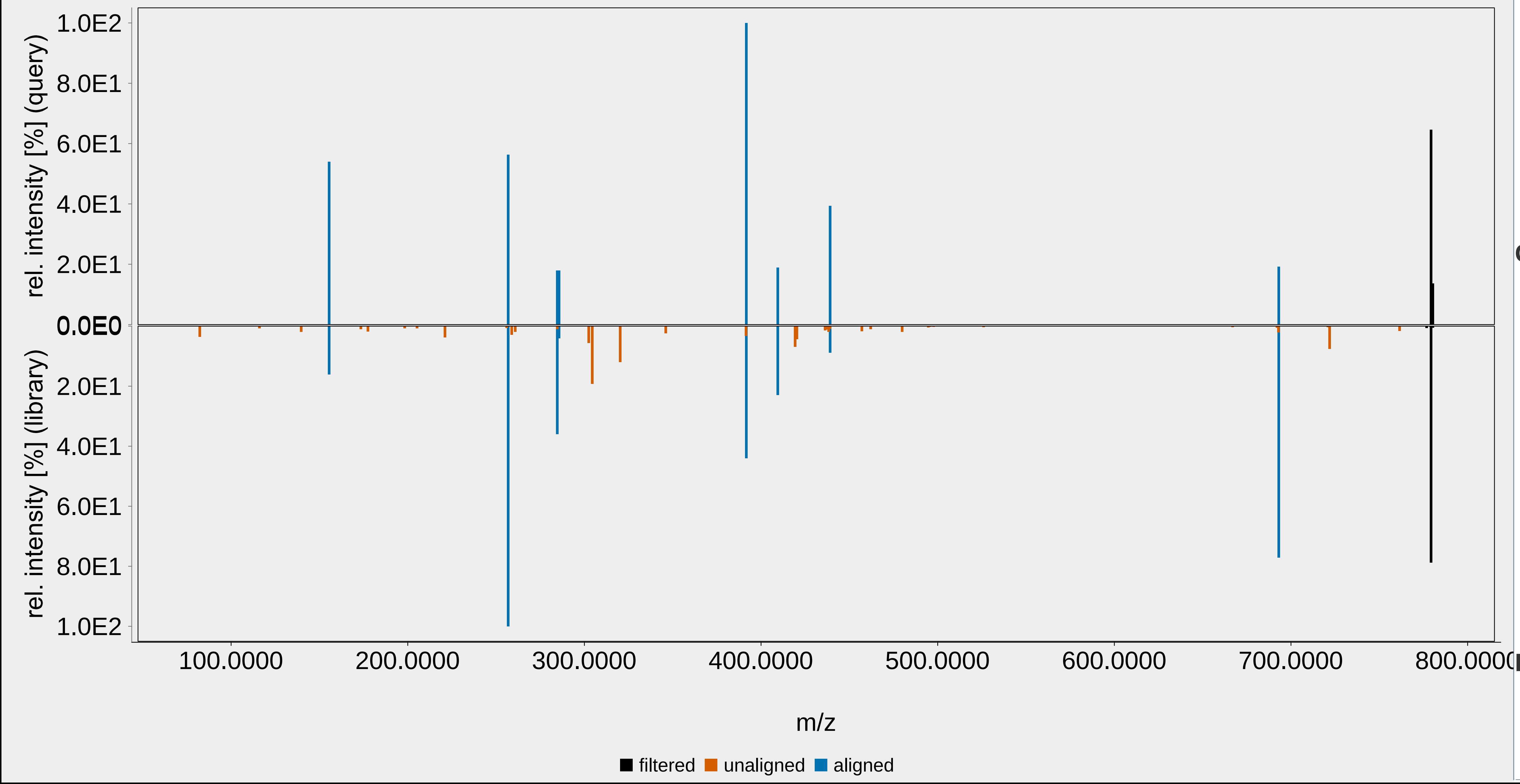

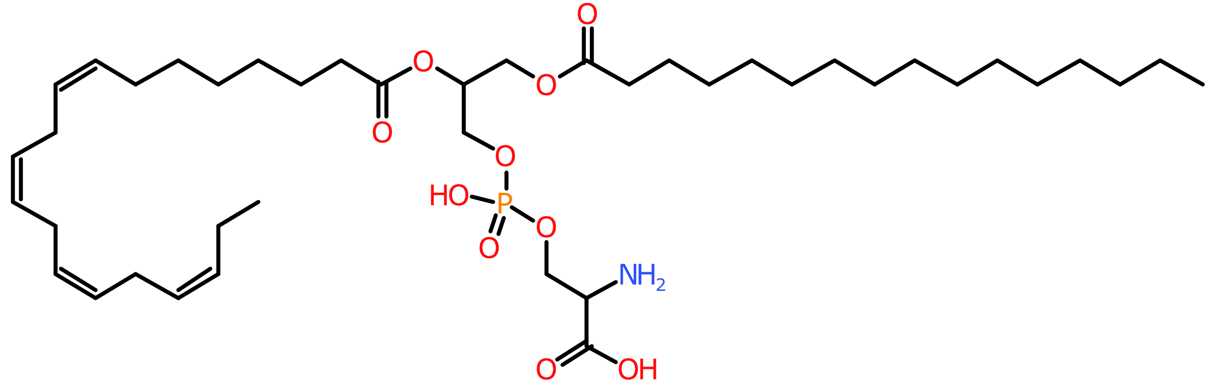

Compound information

Name: PS 36:4

Formula: C42H74NO10P

Exact mass: 784.0269999999999

Ion type: [M-H]-

Precursor m/z: 782.494

Polarity: NEGATIVE

InChI:

InChI key:

SMILES:

Num peaks: 55

Instrument information

Instrument type: LC-ESI-QTOF

Instrument: AB Sciex TripleTOF 5600+ system

Ms level: MS2

Collision energy: 45V

Database links

Other information

Entry ID: MSBNK-RIKEN_IMS-LQB00329

Comment: Parent=782.494

PS(18:0/0:0); [M-H]- C24H47N1O9P1 0.727

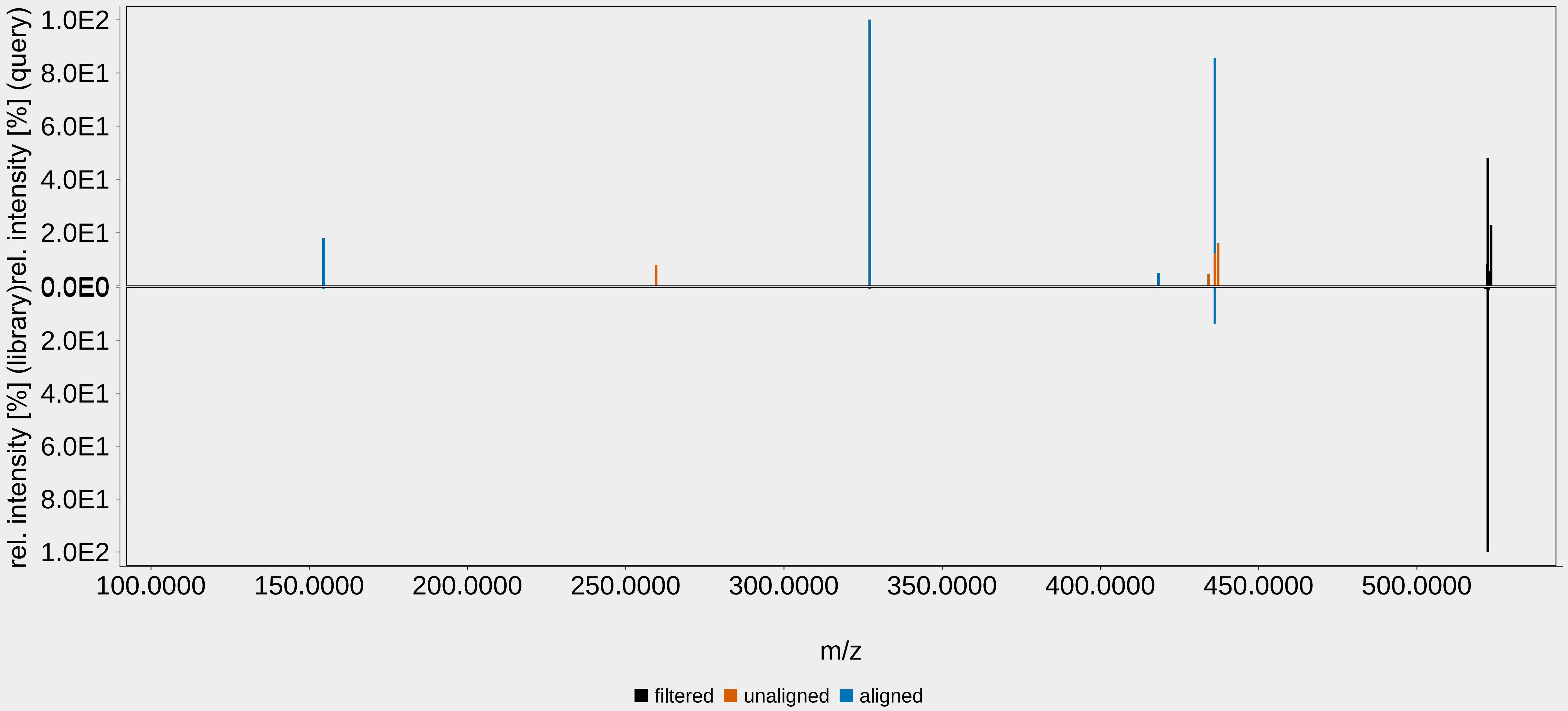

Compound information

Name: PS(18:0/0:0); [M-H]- C24H47N1O9P1

Exact mass: 0.0

Ion type: M-H

Precursor m/z: 524.299

Charge: 1

Polarity: Negative

Instrument information

Instrument type: HCD; Velos

Ion source: LC-ESI

Ms level: 2

Acquisition: Commercial

Database links

Other information

Principal investigator: Thomas Metz

Data collector: Thomas Metz

Entry ID: CCMSLIB00010074162

PE(16:1/0:0); [M-H]- C21H41N1O7P1 **0.825**

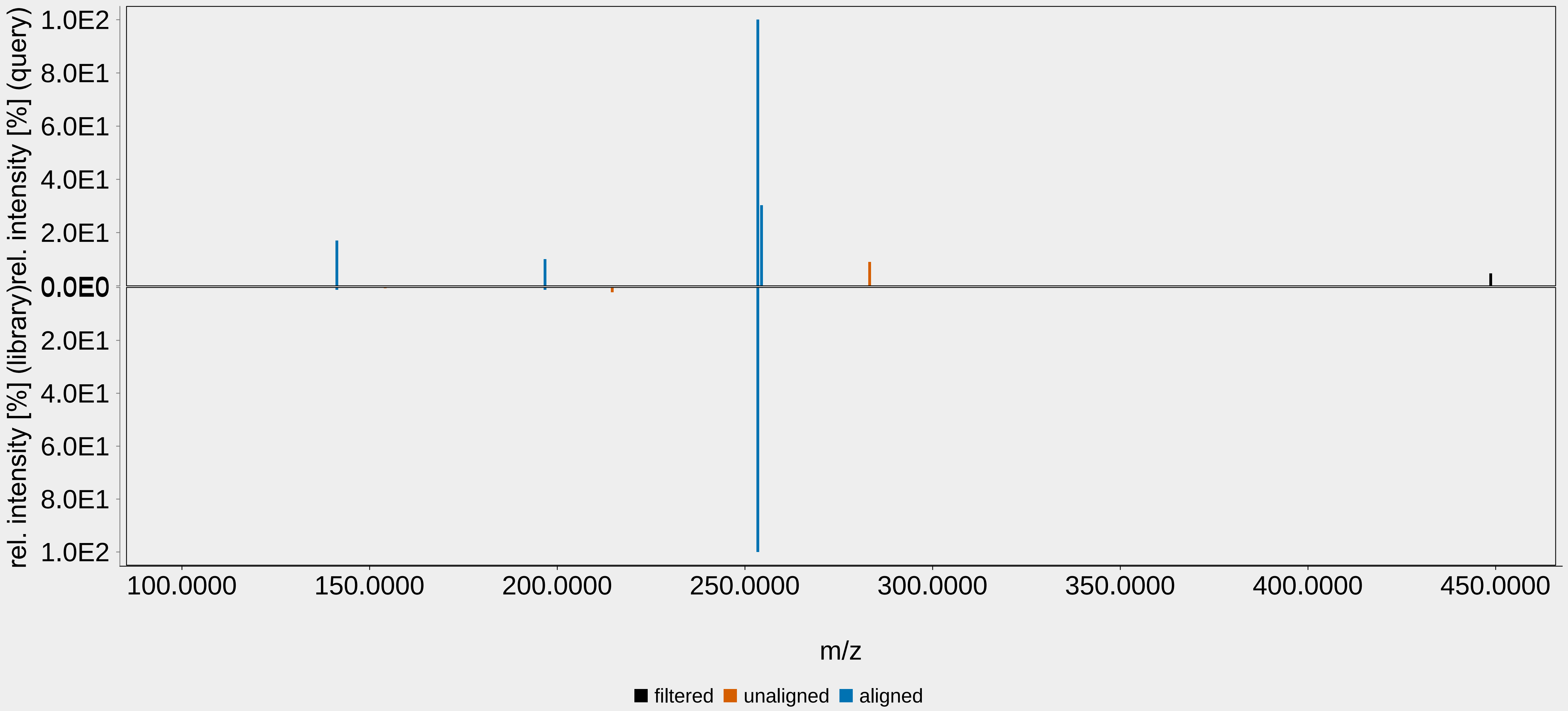

Compound information

Name: PE(16:1/0:0); [M-H]- C21H41N1O7P1

Exact mass: 0.0

Ion type: M-H

Precursor m/z: 450.262

Charge: 1

Polarity: Negative

Instrument information

Instrument type: HCD; Lumos

Ion source: LC-ESI

Ms level: 2

Acquisition: Commercial

Database links

Other information

Principal investigator: Thomas Metz

Data collector: Thomas Metz

Entry ID: CCMSLIB00010059507

PE(16:1/0:0); [M-H]- C21H41N1O7P1 0.848

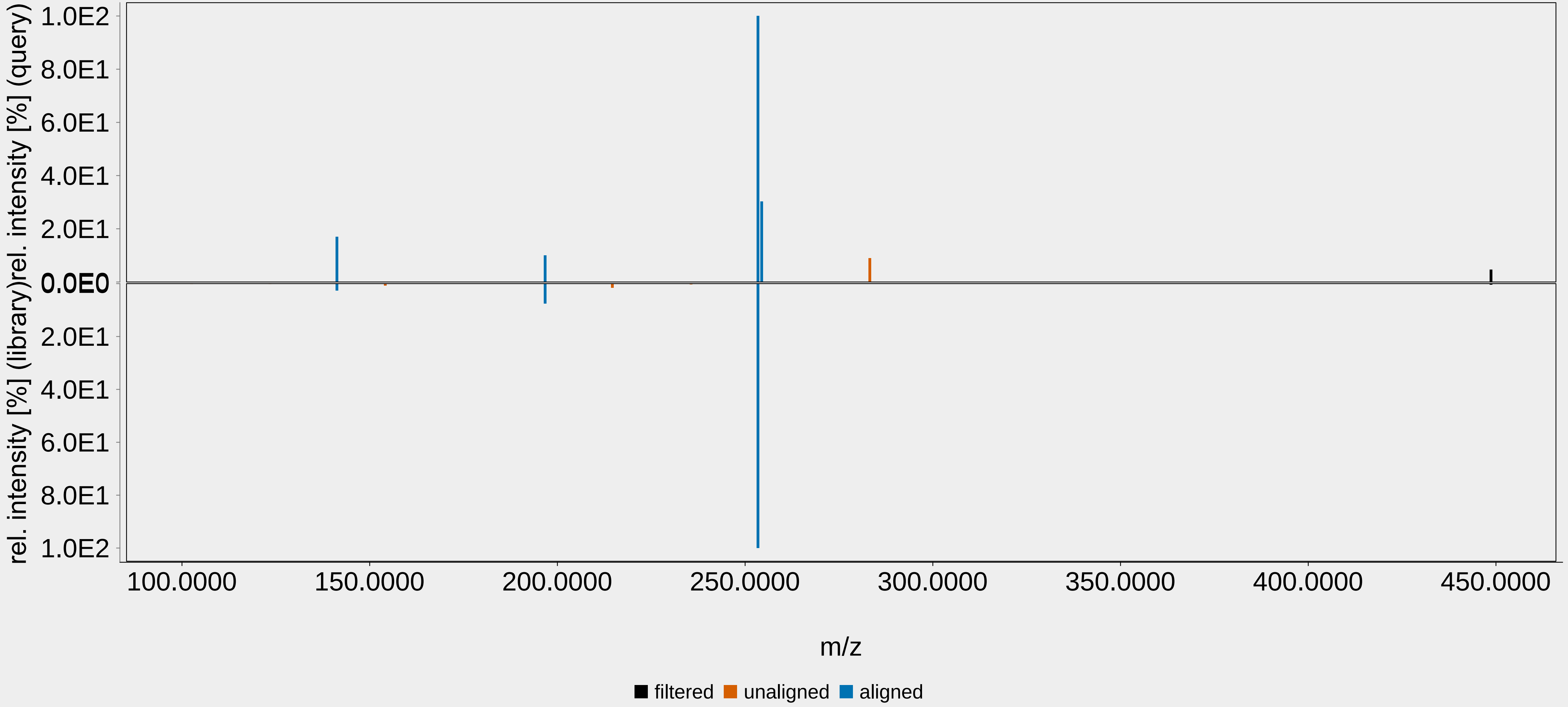

Compound information

Name: PE(16:1/0:0); [M-H]- C21H41N1O7P1

Exact mass: 0.0

Ion type: M-H

Precursor m/z: 450.262

Charge: 1

Polarity: Negative

Instrument information

Instrument type: HCD; Lumos

Ion source: LC-ESI

Ms level: 2

Acquisition: Commercial

Database links

Other information

Principal investigator: Thomas Metz

Data collector: Thomas Metz

Entry ID: CCMSLIB00010059800

PE(20:4/22:4); [M-H]- C47H77N1O8P1 **0.732**

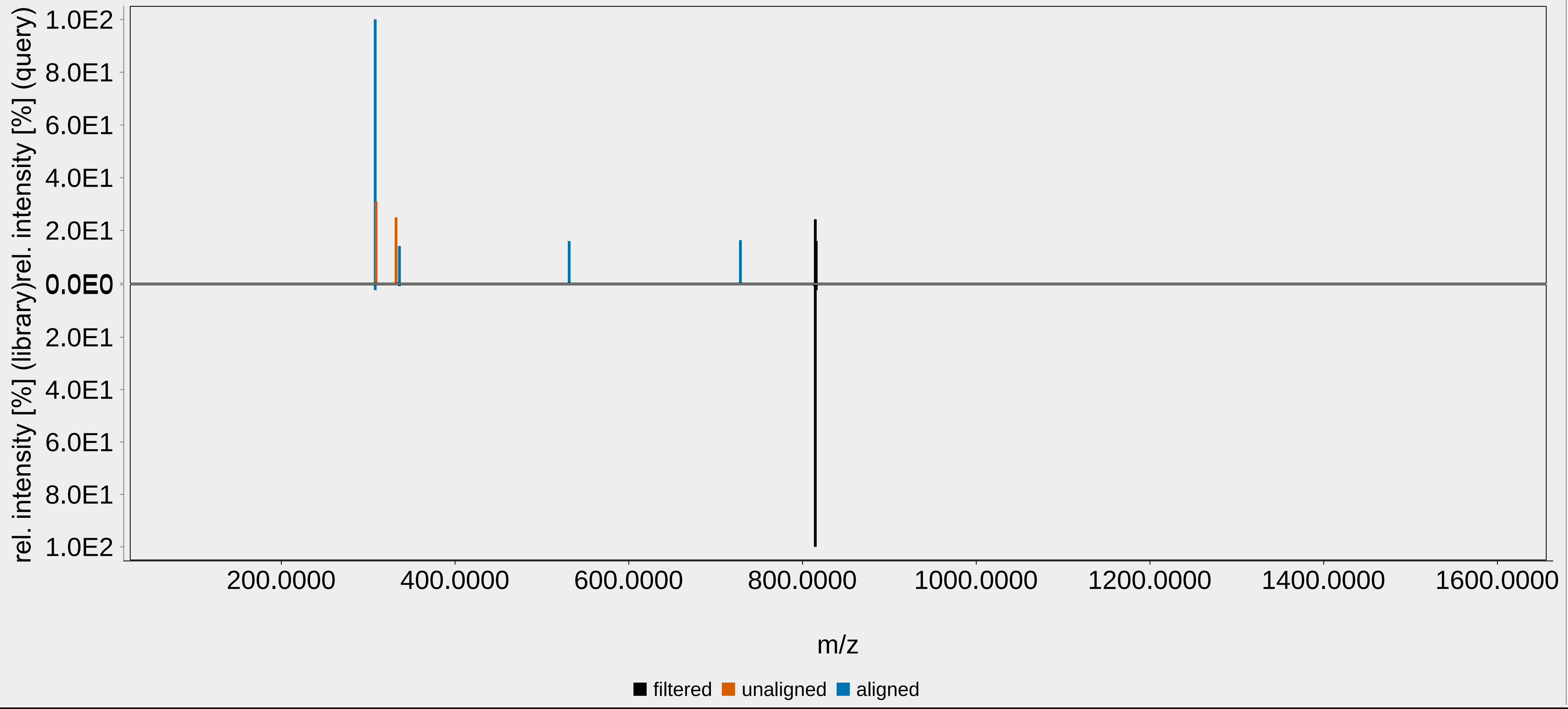

Compound information

Name: PE(20:4/22:4); [M-H]- C47H77N1O8P1

Exact mass: 0.0

Ion type: M-H

Precursor m/z: 814.539

Charge: 1

Polarity: Negative

Instrument information

Instrument type: HCD; Velos

Ion source: LC-ESI

Ms level: 2

Acquisition: Commercial

Database links

Other information

Principal investigator: Thomas Metz

Data collector: Thomas Metz

Entry ID: CCMSLIB00010063417

PE(20:4/22:4); [M-H]- C47H77N1O8P1 **0.860**

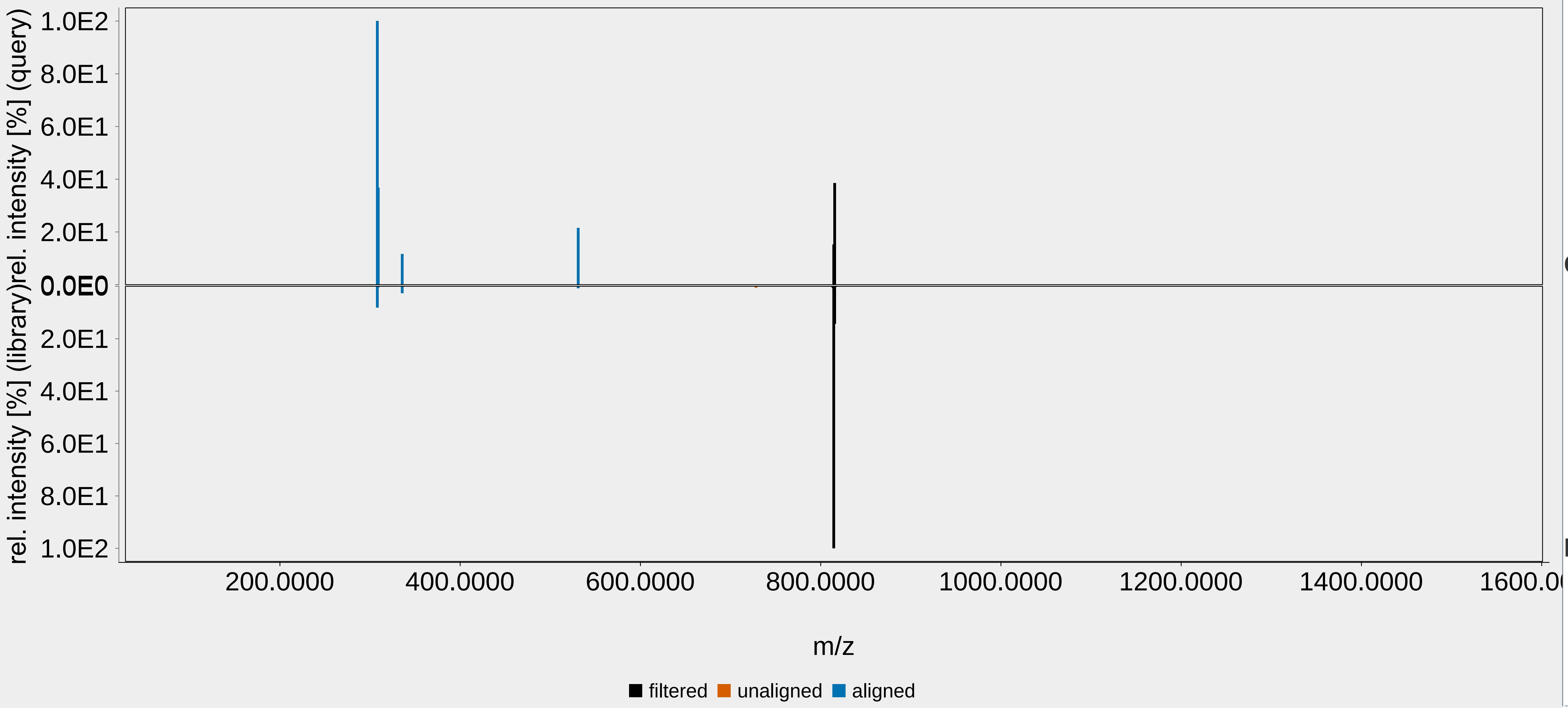

Compound information

Name: PE(20:4/22:4); [M-H]- C47H77N1O8P1

Exact mass: 0.0

Ion type: M-H

Precursor m/z: 814.539

Charge: 1

Polarity: Negative

Instrument information

Instrument type: HCD; Velos

Ion source: LC-ESI

Ms level: 2

Acquisition: Commercial

Database links

Other information

Principal investigator: Thomas Metz

Data collector: Thomas Metz

Entry ID: CCMSLIB00010087014

PA 18:0_20:3 **0.742**

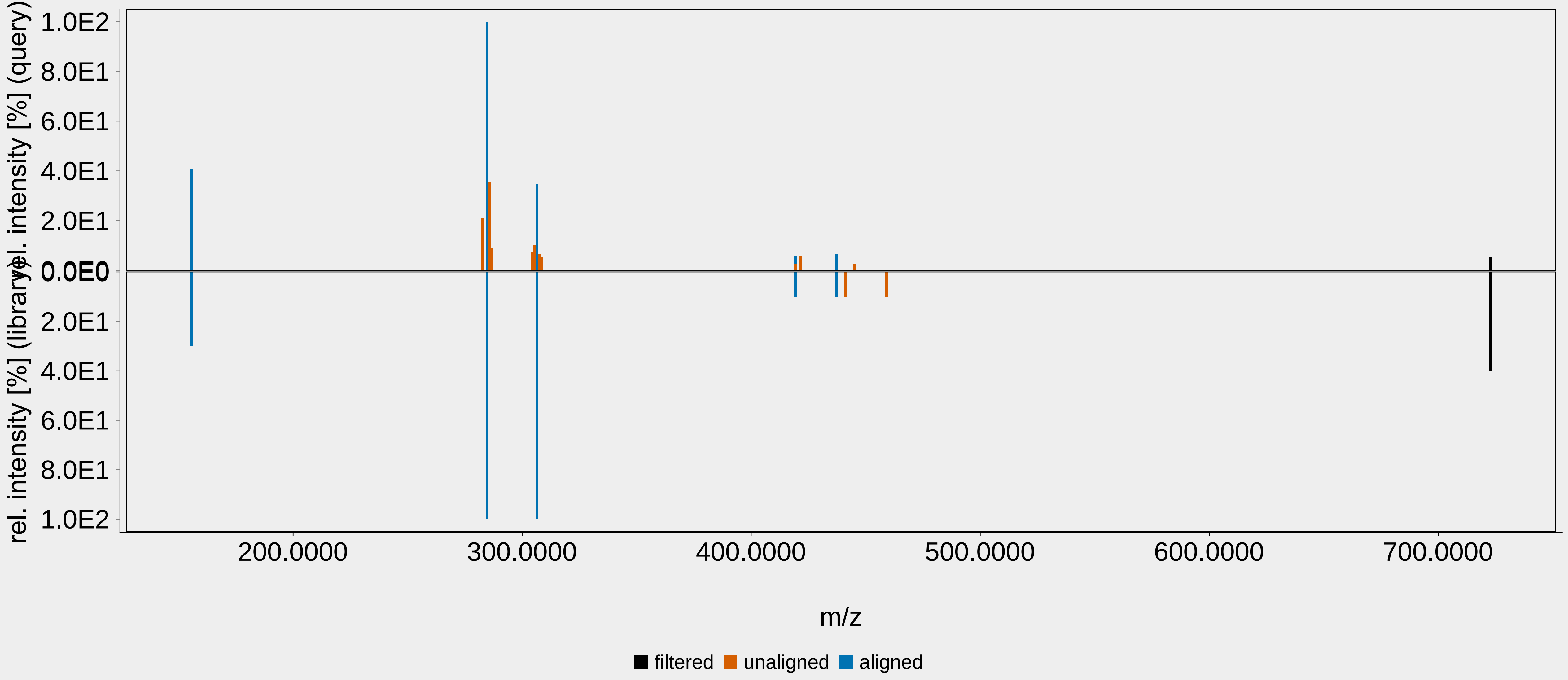

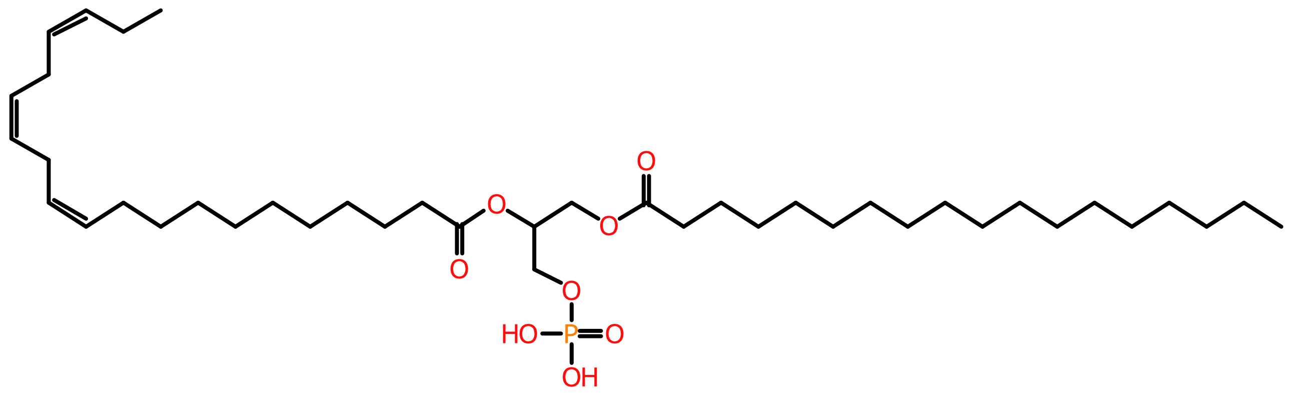

Compound information

Name: PA 18:0_20:3

Formula: C41H75O8P

Ion type: [M-H]-

Precursor m/z: 725.51268

Ccs: 263.04

InChI key: KGDFLTPILJIQMX-JTBMWNAQNA-

SMILES:

Num peaks: 8

Instrument information

Database links

Other information

PE(20:4/0:0); [M-H]- C25H43N1O7P1

0.917

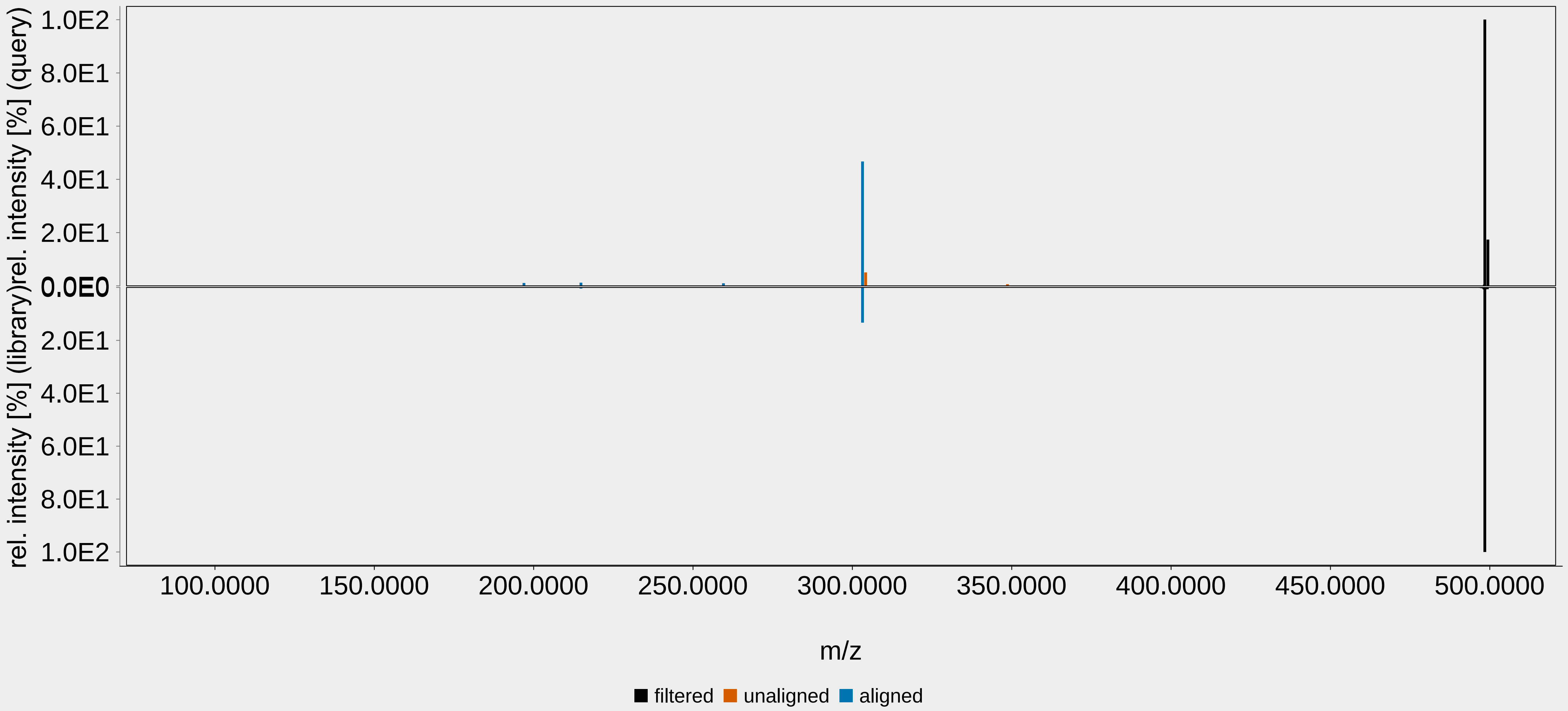

Compound information

Name: PE(20:4/0:0); [M-H]- C25H43N1O7P1

Exact mass: 0.0

Ion type: M-H

Precursor m/z: 500.278

Charge: 1

Polarity: Negative

Instrument information

Instrument type: HCD; Velos

Ion source: LC-ESI

Ms level: 2

Acquisition: Commercial

Database links

Other information

Principal investigator: Thomas Metz

Data collector: Thomas Metz

Entry ID: CCMSLIB00010067367

PE(20:4/0:0); [M-H]- C25H43N1O7P1 **0.921**

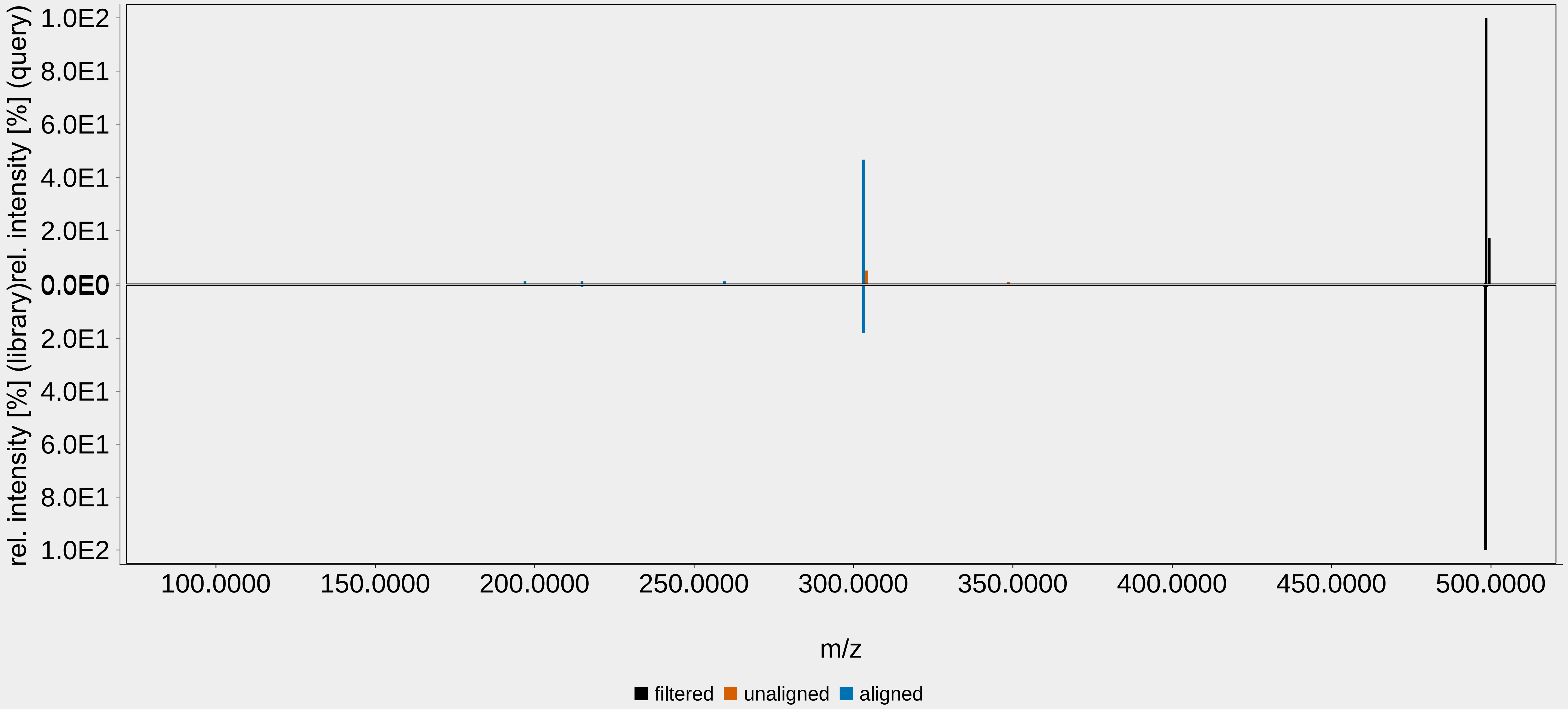

Compound information

Name: PE(20:4/0:0); [M-H]- C25H43N1O7P1

Exact mass: 0.0

Ion type: M-H

Precursor m/z: 500.278

Charge: 1

Polarity: Negative

Instrument information

Instrument type: HCD; Velos

Ion source: LC-ESI

Ms level: 2

Acquisition: Commercial

Database links

Other information

Principal investigator: Thomas Metz

Data collector: Thomas Metz

Entry ID: CCMSLIB00010085243

Dodecylbenzenesulfonic acid

0.965

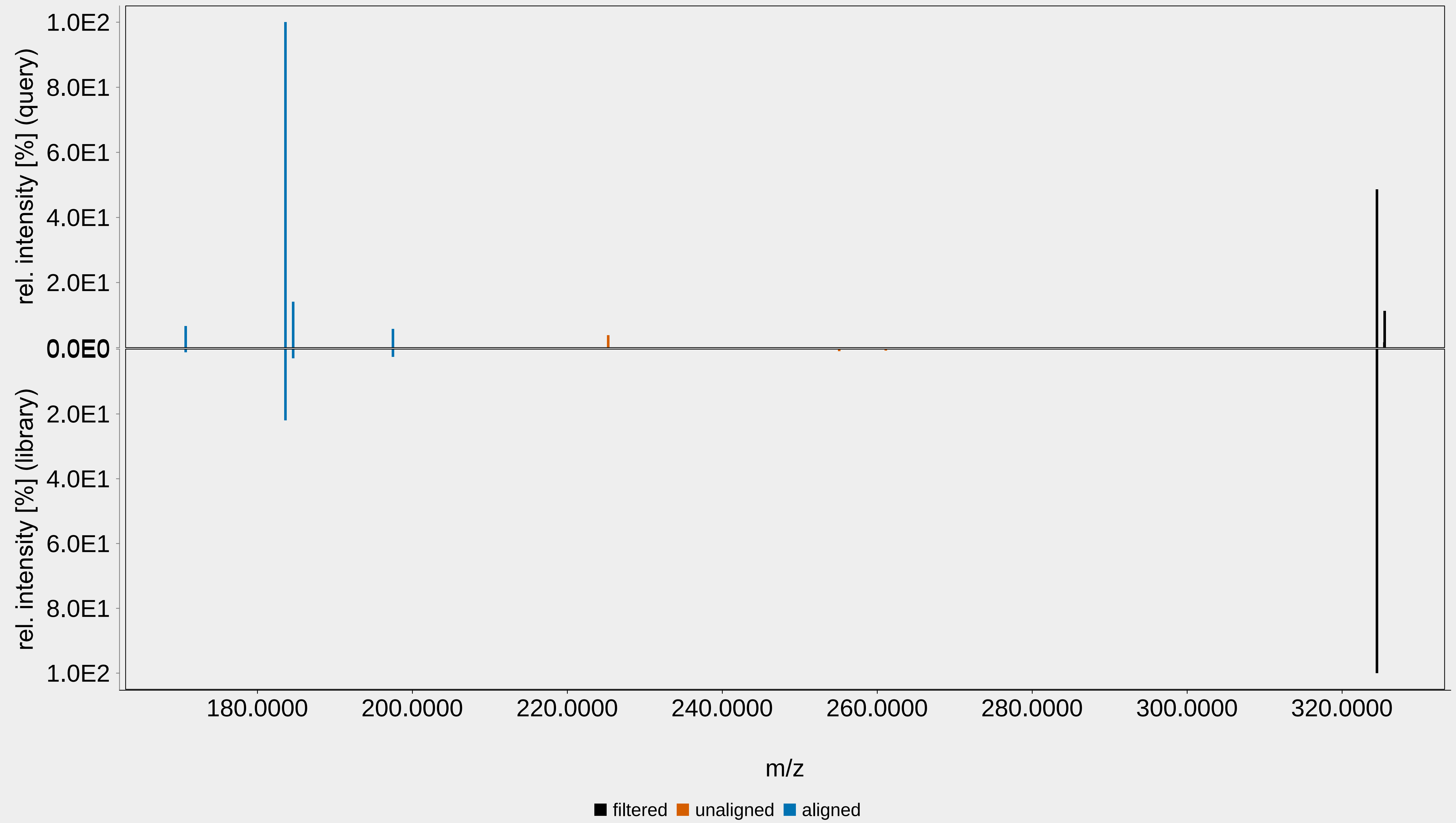

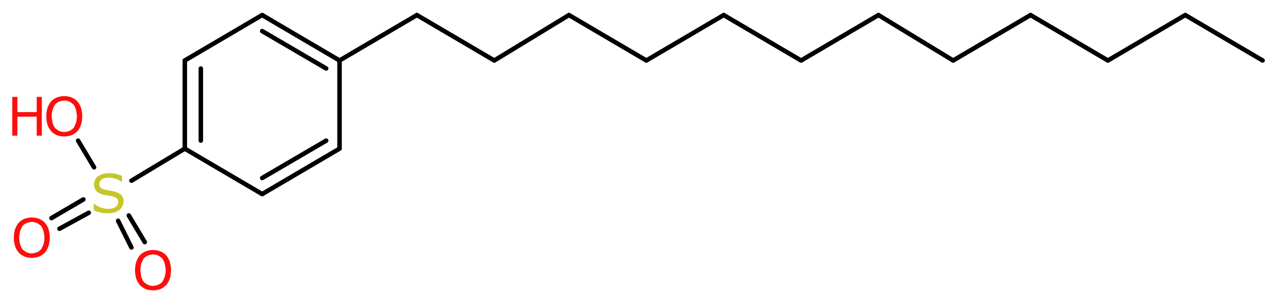

Compound information

Name: Dodecylbenzenesulfonic acid

Formula: C18H30O3S

Mol. weight: 326.19157

Exact mass: 326.19157

Ion type: [M-H]-

Precursor m/z: 325.1843

Polarity: negative

InChI:

InChI key:

SMILES:

Instrument information

Instrument type: LC-ESI-QFT

Instrument: Q Exactive Plus Orbitrap Thermo

Ion source: ESI

Resolution: 35000

Ms level: MS2

Collision energy: 35 (nominal)

Database links

Pubchem: 8485

MoNA ID: SM876251

Chemspider: 8172

CAS: 121-65-3

Other information

Principal investigator: Krauss M, Schymanski

Data collector: Krauss M, Schymanski EL,

Dodecylbenzenesulfonic acid

0.966

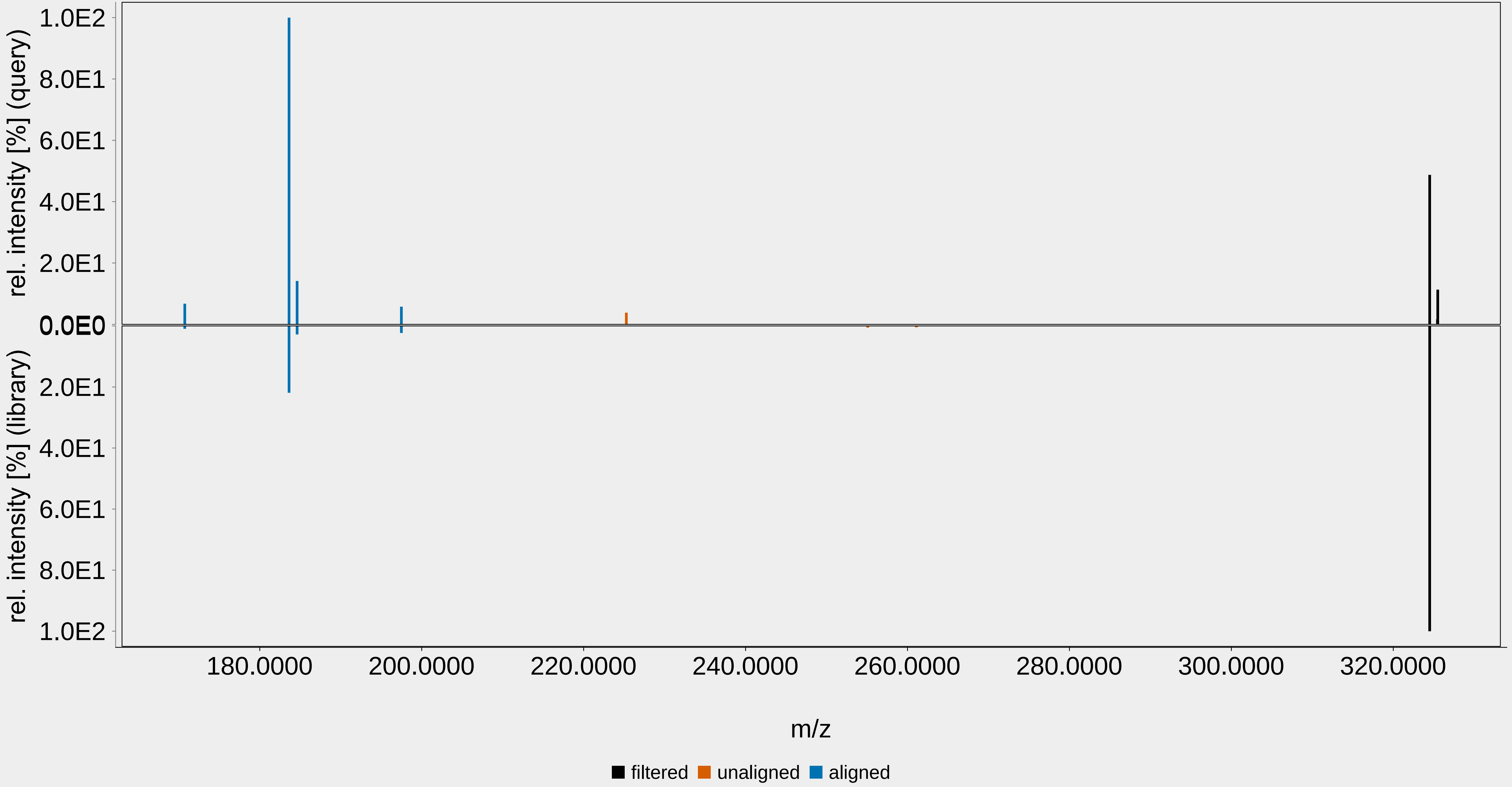

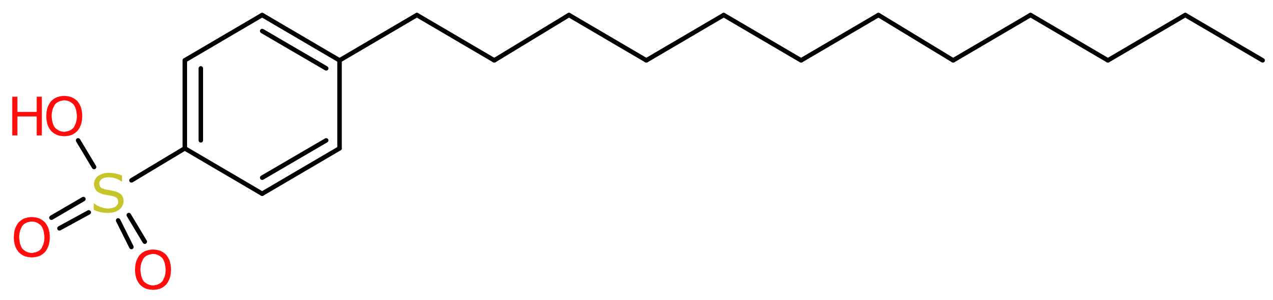

Compound information

Name: Dodecylbenzenesulfonic acid

Formula: C18H30O3S

Exact mass: 326.19157

Ion type: [M-H]-

Precursor m/z: 325.1843

Polarity: NEGATIVE

InChI:

InChI key:

SMILES:

Num peaks: 7

Instrument information

Instrument type: LC-ESI-QFT

Instrument: Q Exactive Plus Orbitrap Thermo

Ms level: MS2

Collision energy: 35 (nominal)

Database links

Other information

Entry ID: MSBNK-CASMI_2016-SM876251

Comment: Parent=325.1843

PS(16:0/18:1); [M-H]- C40H75N1O10P1 0.938

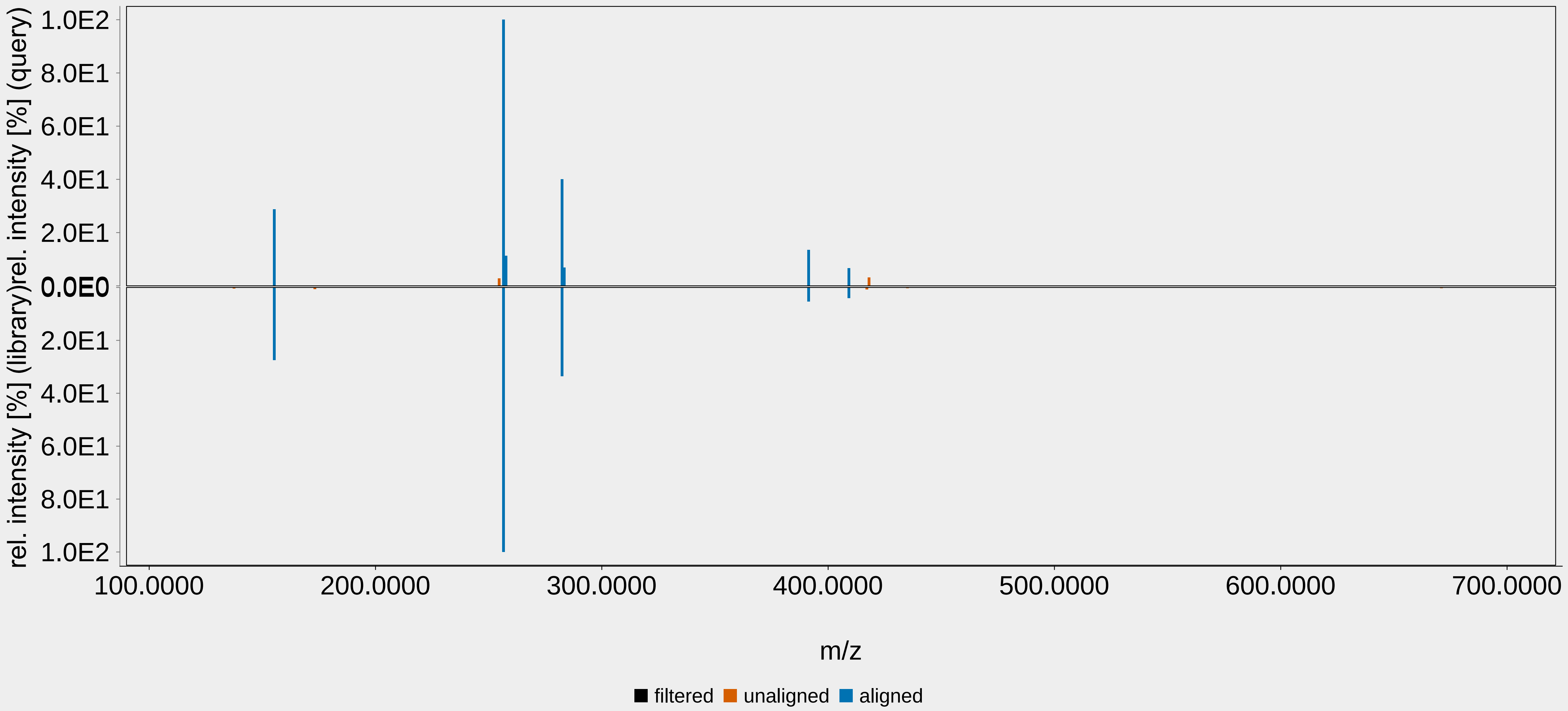

Compound information

Name: PS(16:0/18:1); [M-H]- C40H75N1O10P1

Exact mass: 0.0

Ion type: M-H

Precursor m/z: 760.513

Charge: 1

Polarity: Negative

Instrument information

Instrument type: HCD; Lumos

Ion source: LC-ESI

Ms level: 2

Acquisition: Commercial

Database links

Other information

Principal investigator: Thomas Metz

Data collector: Thomas Metz

Entry ID: CCMSLIB00010059839

PS(16:0/18:1); [M-H]- C40H75N1O10P1

0.938

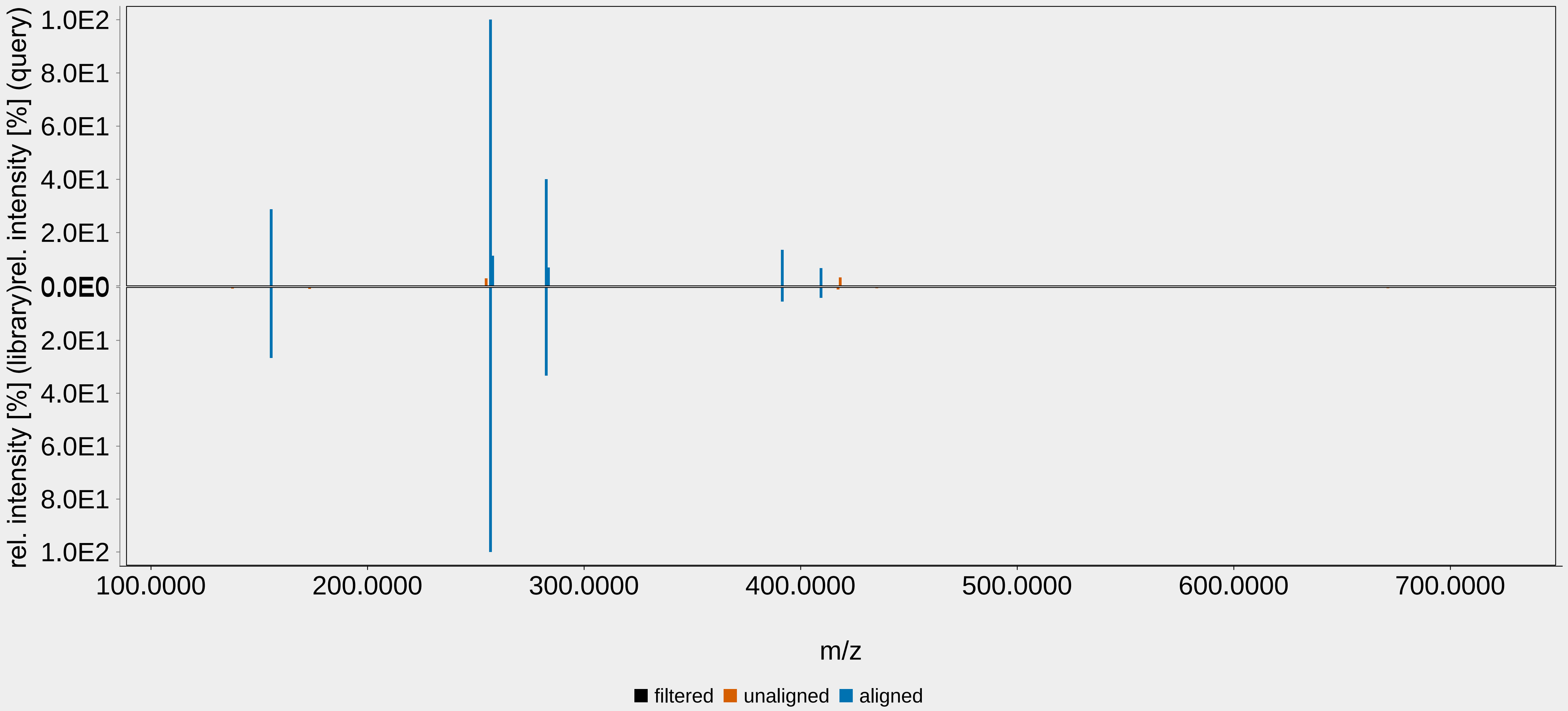

Compound information

Name: PS(16:0/18:1); [M-H]- C40H75N1O10P1

Exact mass: 0.0

Ion type: M-H

Precursor m/z: 760.513

Charge: 1

Polarity: Negative

Instrument information

Instrument type: HCD; Lumos

Ion source: LC-ESI

Ms level: 2

Acquisition: Commercial

Database links

Other information

Principal investigator: Thomas Metz

Data collector: Thomas Metz

Entry ID: CCMSLIB00010059457

PI 36:1

0.730

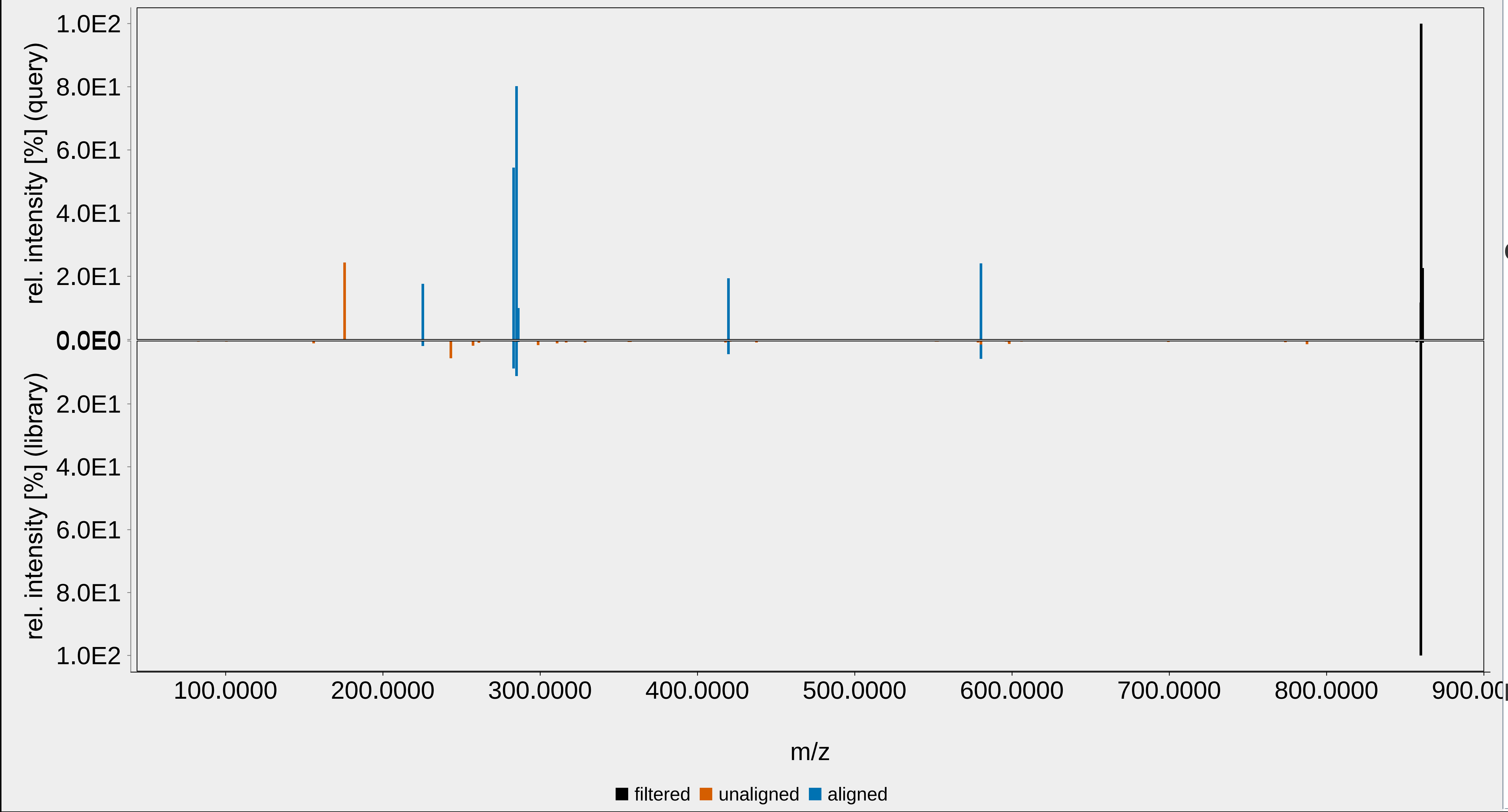

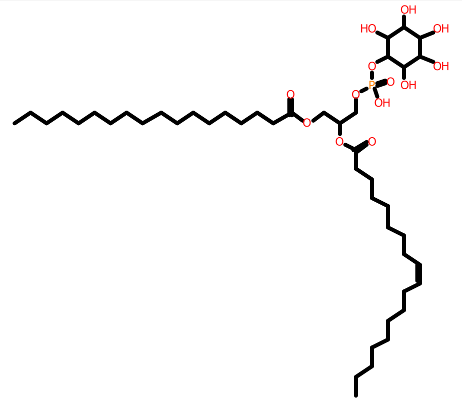

Compound information

Name: PI 36:1

Formula: C45H85O13P

Mol. weight: 865.1379999999999

Exact mass: 865.1379999999999

Ion type: [M-H]-

Precursor m/z: 863.5657

RT: 9.13166

Polarity: negative

InChI:

InChI key: YOBFISPJJWPPTK-ZZEZOPTASA-I

SMILES:

Instrument information

Instrument type: LC-ESI-QTOF

Instrument: AB Sciex TripleTOF 5600+ system

Ms level: MS2

Collision energy: 45V

Database links

Pubchem: 138306742

MoNA ID: LQB00691

Other information

Principal investigator: Kazutaka Ikeda, Hiroshi

Data collector: Kazutaka Ikeda, Hiroshi

PI 36:1 0.730

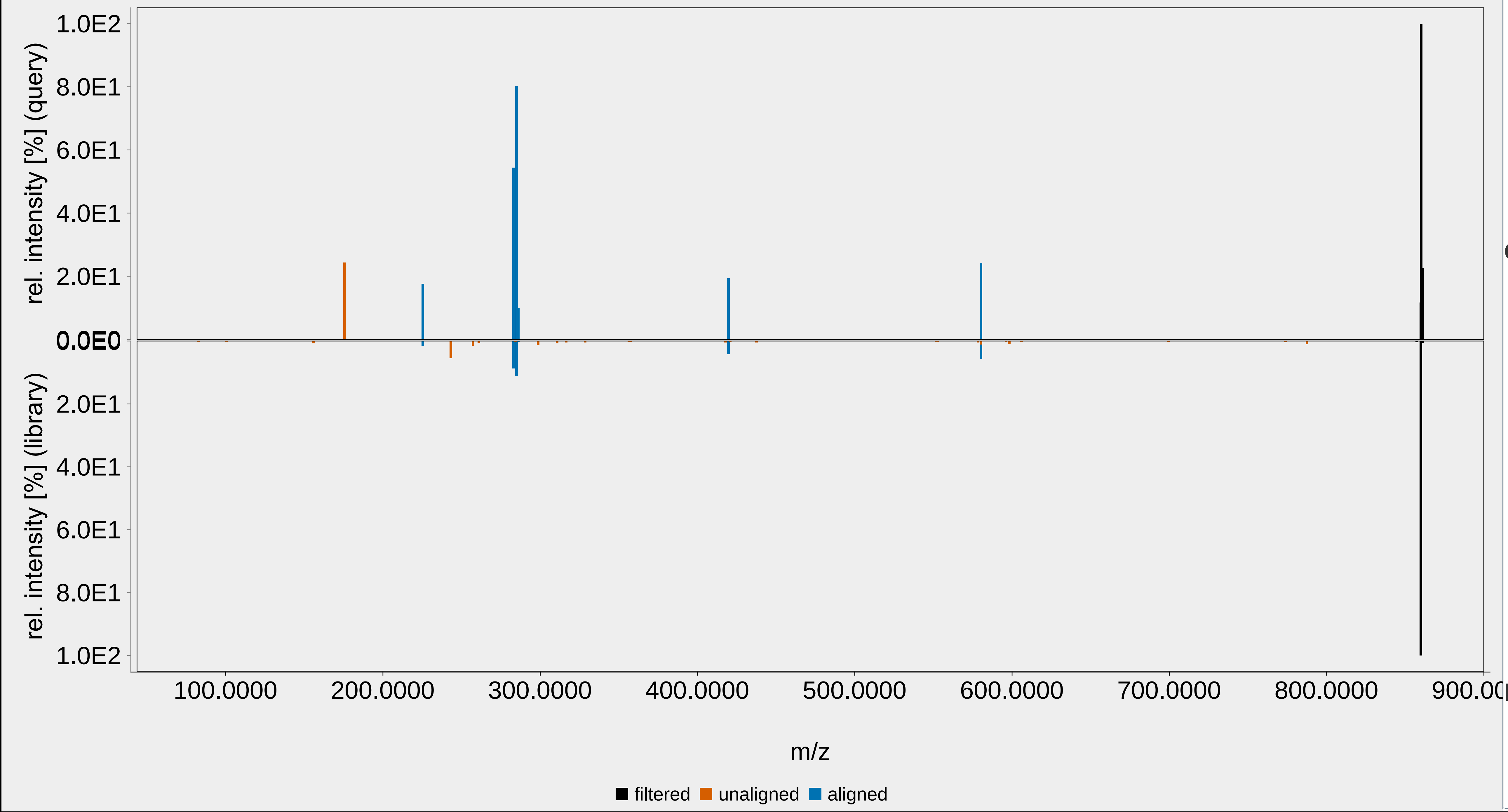

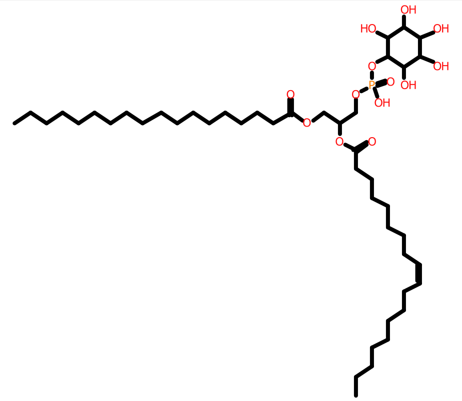

Compound information

Name: PI 36:1

Formula: C45H85O13P

Mol. weight: 865.1379999999999

Exact mass: 865.1379999999999

Ion type: [M-H]-

Precursor m/z: 863.5657

RT: 9.13166

Polarity: negative

InChI:

InChI key: YOBFISPJJWPPTK-ZZEZOPTASA-I

SMILES:

Instrument information

Instrument type: LC-ESI-QTOF

Instrument: AB Sciex TripleTOF 5600+ system

Ms level: MS2

Collision energy: 45V

Database links

Pubchem: 138306742

MoNA ID: LQB00305

Other information

Principal investigator: Kazutaka Ikeda, Hiroshi

Data collector: Kazutaka Ikeda, Hiroshi

PA 16:0_18:0

0.850

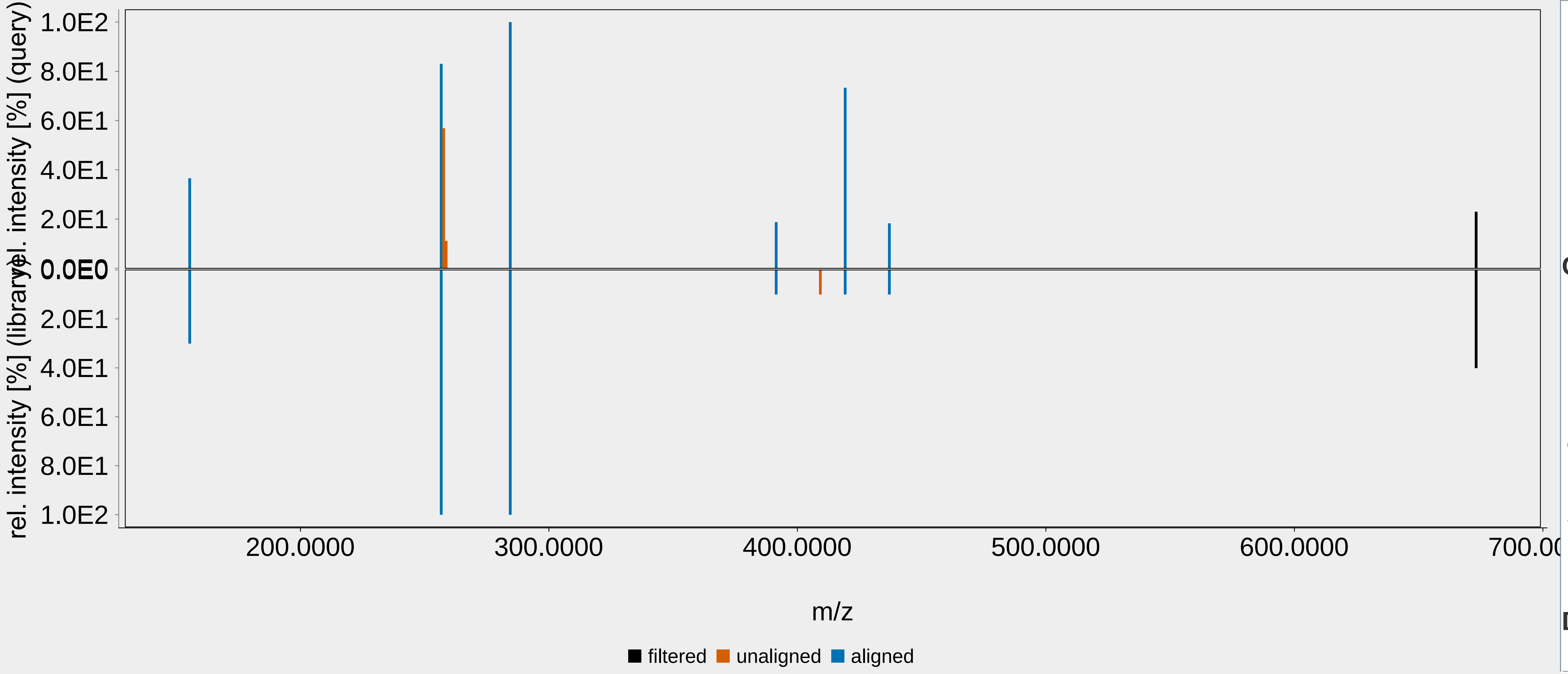

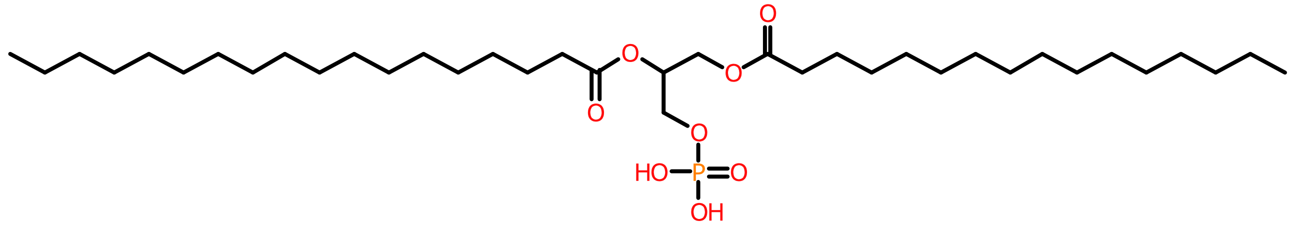

Compound information

Name: PA 16:0_18:0

Formula: C37H73O8P

Ion type: [M-H]-

Precursor m/z: 675.49703

Ccs: 256.25

InChI key: GASPGIDKILJBMM-UHFFFAOYNA-

SMILES:

Num peaks: 8

Instrument information

Database links

Other information

PA(16:1/18:1); [M-H]- C37H68O8P1 **0.788**

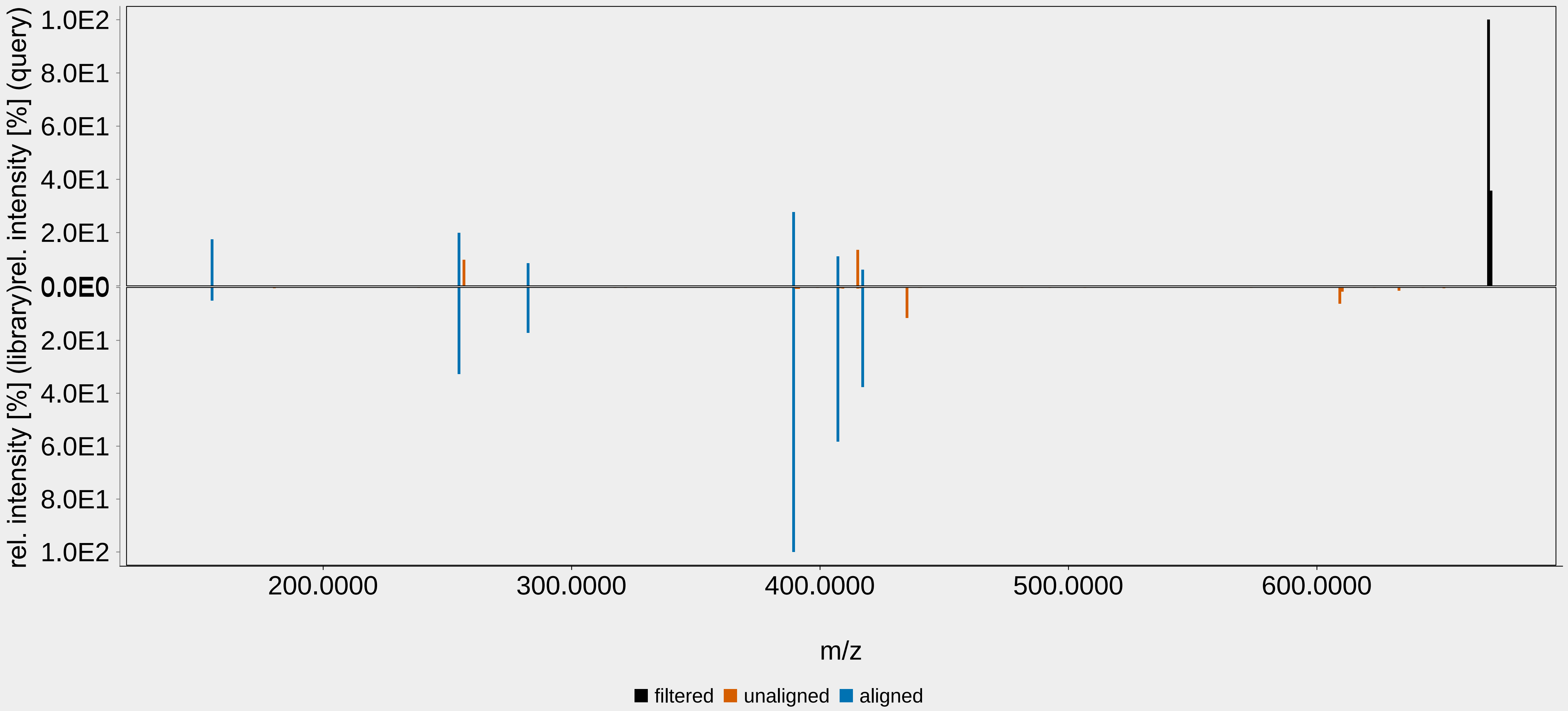

Compound information

Name: PA(16:1/18:1); [M-H]- C37H68O8P1

Exact mass: 0.0

Ion type: M-H

Precursor m/z: 671.465

Charge: 1

Polarity: Negative

Instrument information

Instrument type: CID; Lumos

Ion source: LC-ESI

Ms level: 2

Acquisition: Commercial

Database links

Other information

Principal investigator: Thomas Metz

Data collector: Thomas Metz

Entry ID: CCMSLIB00010059155

PA(16:1/18:1); [M-H]- C37H68O8P1 **0.913**

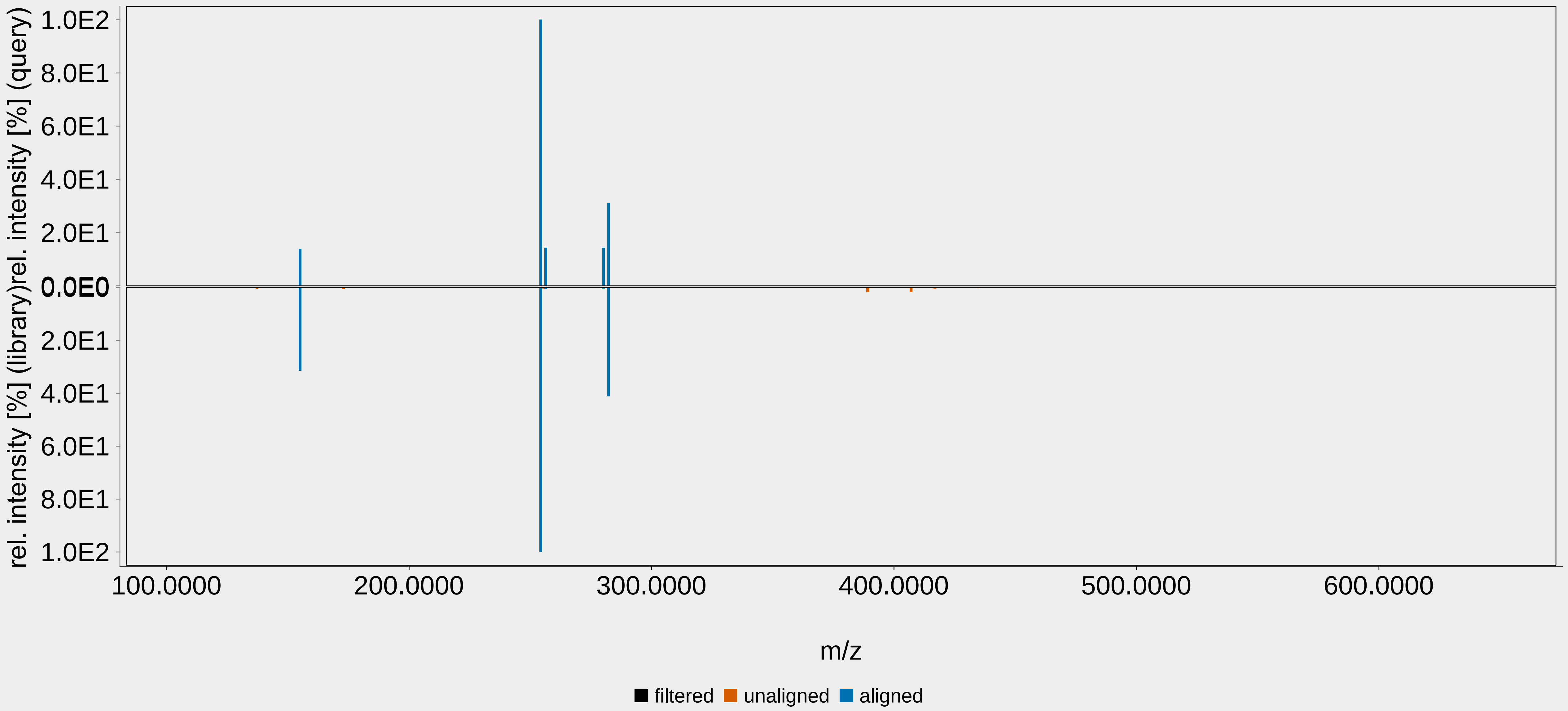

Compound information

Name: PA(16:1/18:1); [M-H]- C37H68O8P1

Exact mass: 0.0

Ion type: M-H

Precursor m/z: 671.465

Charge: 1

Polarity: Negative

Instrument information

Instrument type: HCD; Lumos

Ion source: LC-ESI

Ms level: 2

Acquisition: Commercial

Database links

Other information

Principal investigator: Thomas Metz

Data collector: Thomas Metz

Entry ID: CCMSLIB00010059156

ADENOSINE 5'-TRIPHOSPHATE

0.743

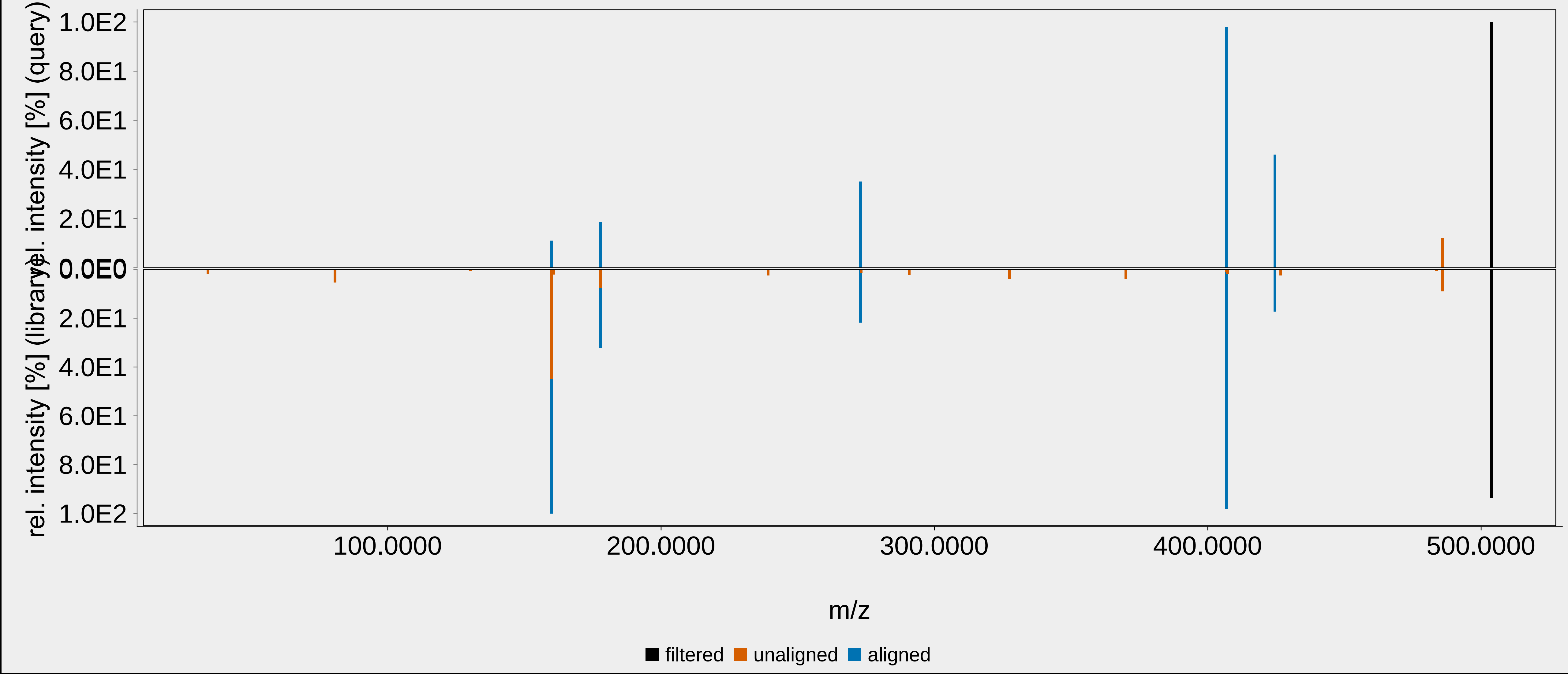

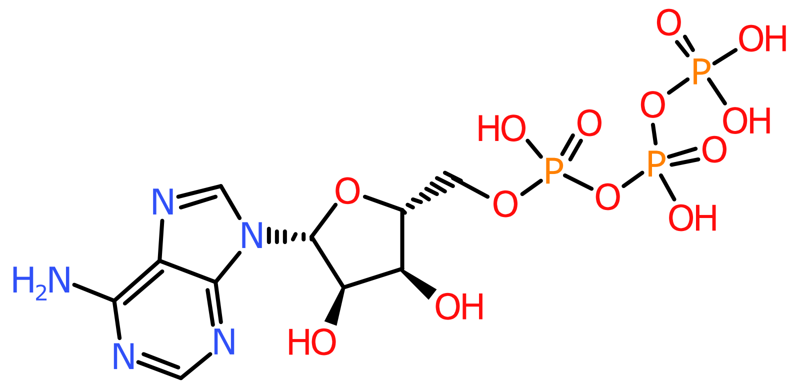

Compound information

Name: ADENOSINE 5'-TRIPHOSPHATE

Formula: C10H16N5O13P3

Ion type: [M-H]-

Precursor m/z: 505.9884695

Polarity: negative

InChI:

InChI key:

SMILES:

Instrument information

Instrument type: LC-ESI-QTOF

Instrument: Agilent qTOF 6545

Ms level: MS2

Collision energy: 20 eV

Database links

Other information

ADENOSINE 5'-TRIPHOSPHATE

0.743

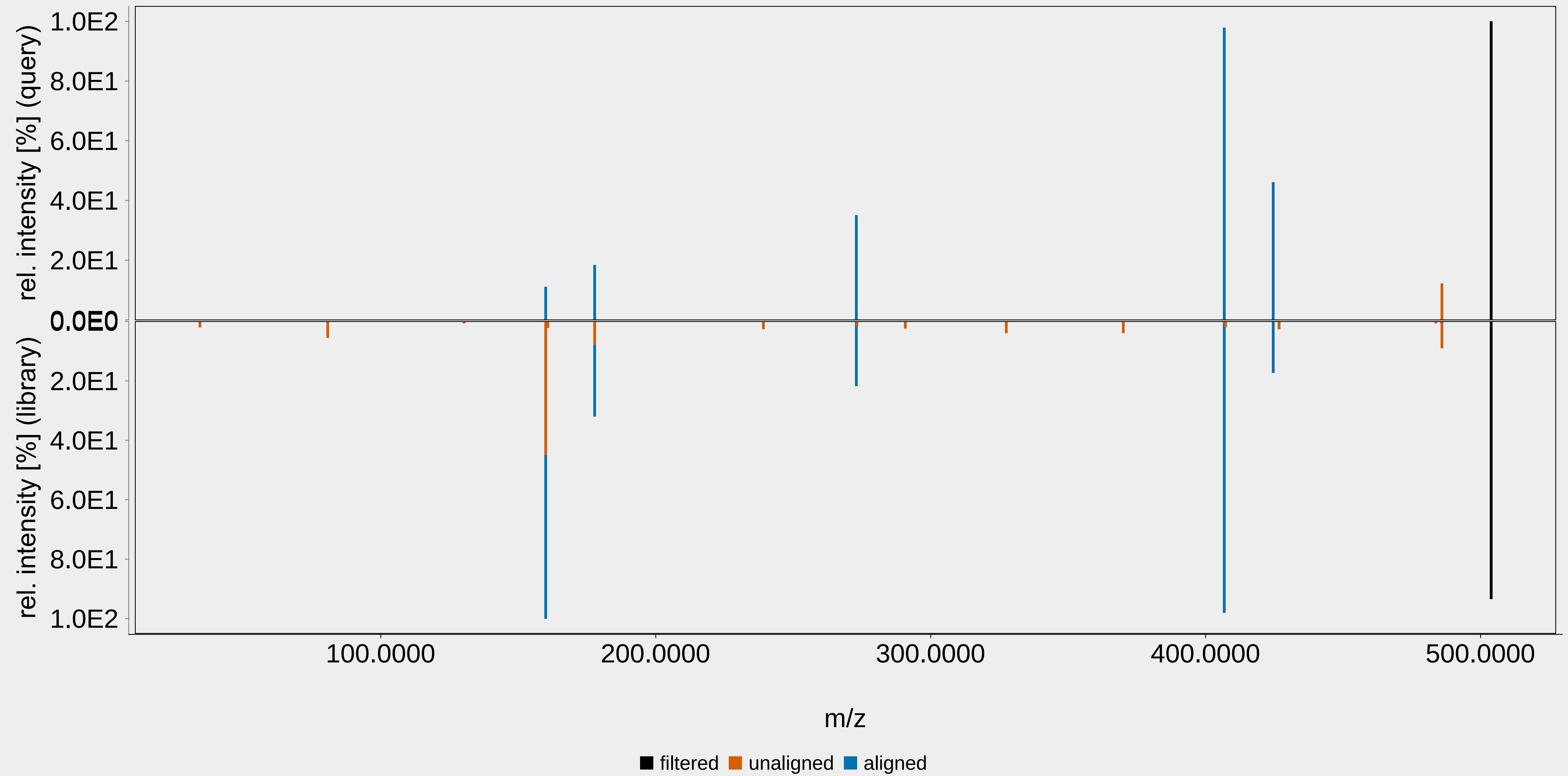

Compound information

Name: ADENOSINE 5'-TRIPHOSPHATE

Exact mass: 0.0

Ion type: M-H

Precursor m/z: 505.988

Charge: 1

Polarity: negative

InChI:

SMILES:

Instrument information

Instrument type: qTof

Ion source: ESI

Ms level: 2

Acquisition: isolated

Database links

Other information

Principal investigator: MoNA

Data collector: MoNA:MoNA034214

Entry ID: CCMSLIB00006685072

PA 18:1_18:2

0.890

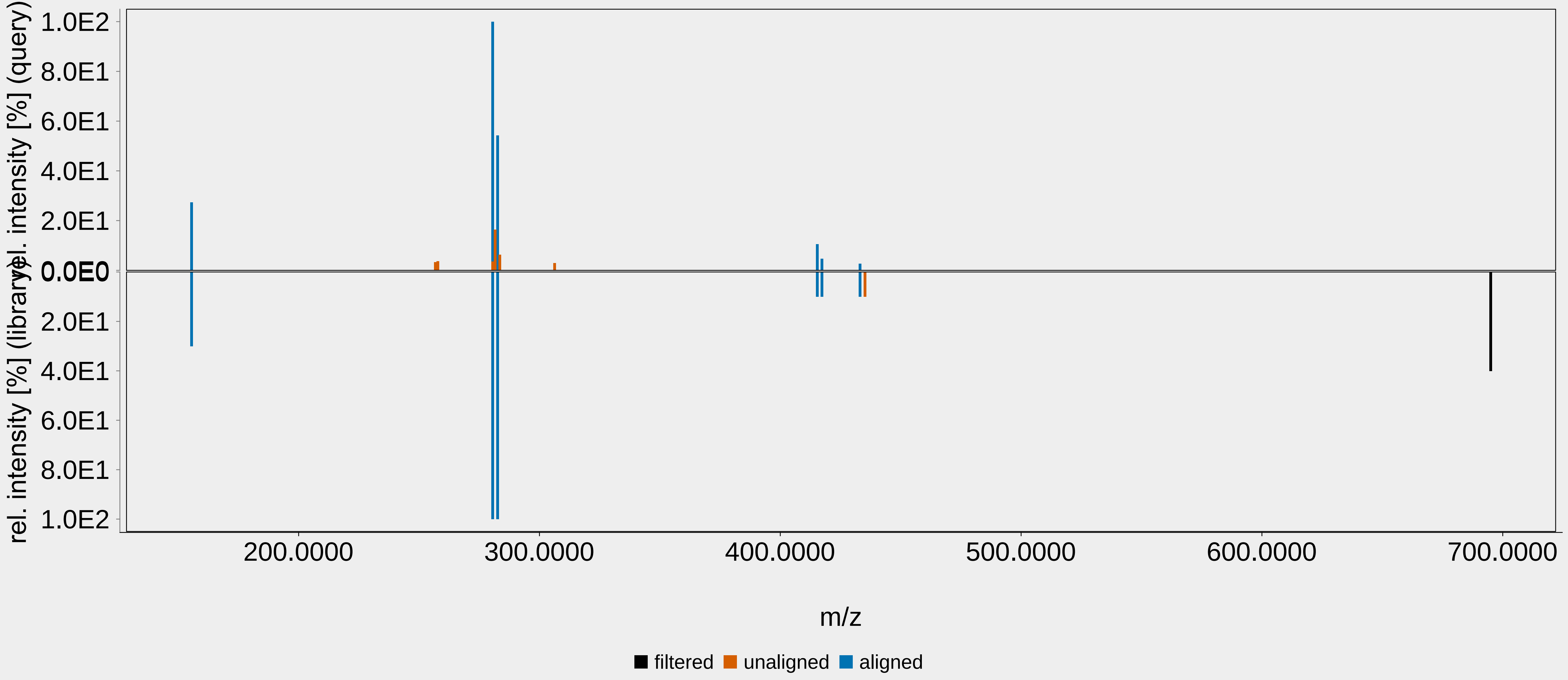

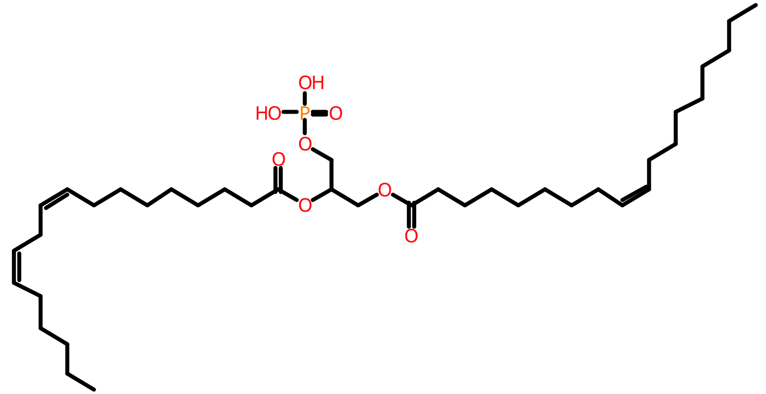

Compound information

Name: PA 18:1_18:2

Formula: C39H71O8P

Ion type: [M-H]-

Precursor m/z: 697.48138

Ccs: 255.37

InChI key:

SMILES:

Num peaks: 8

Instrument information

Database links

Other information

PE(18:1/18:1); [M-H]- C41H77N1O8P1

0.941

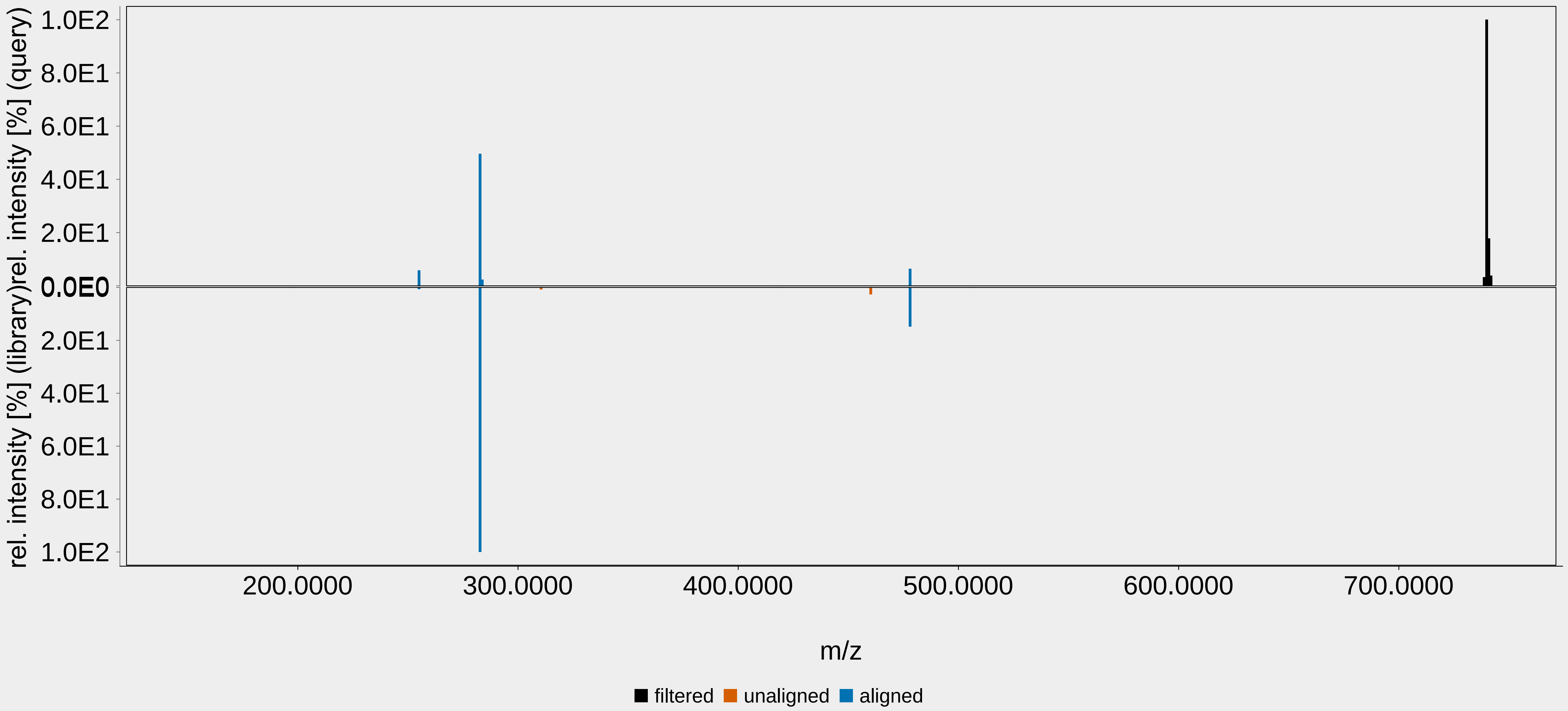

Compound information

Name: PE(18:1/18:1); [M-H]- C41H77N1O8P1

Exact mass: 0.0

Ion type: M-H

Precursor m/z: 742.539

Charge: 1

Polarity: Negative

Instrument information

Instrument type: CID; Lumos

Ion source: LC-ESI

Ms level: 2

Acquisition: Commercial

Database links

Other information

Principal investigator: Thomas Metz

Data collector: Thomas Metz

Entry ID: CCMSLIB00010059182

Spectral Match to 1-Stearoyl-2-linoleoyl-sn-glycero-3-phosphoethanolamine from NIST14

0.957

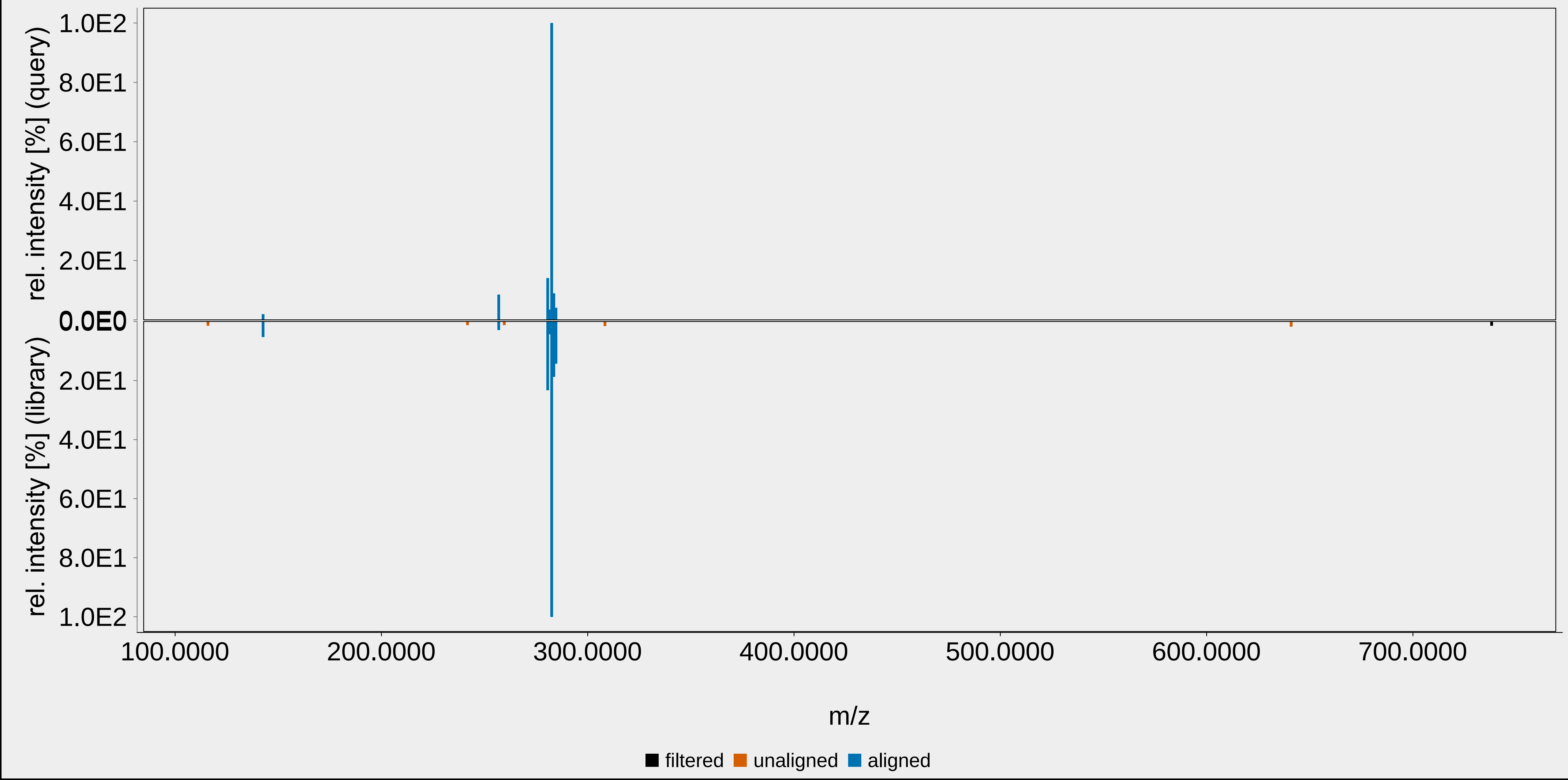

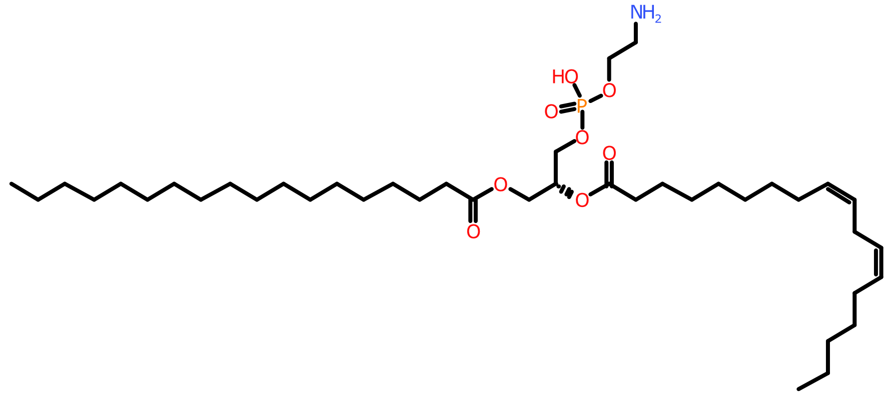

Compound information

Name: Spectral Match to

Exact mass: 743.547

Ion type: M-H

Precursor m/z: 742.541

Charge: 1

Polarity: Negative

InChI:

SMILES:

Instrument information

Instrument type: qTof

Ion source: ESI

Ms level: 2

Acquisition: Isolated

Database links

CAS: 7266537

Other information

Principal investigator: Data from Wiemann

Data collector: Data deposited by pwiemann

Entry ID: CCMSLIB00003136842

PS 18:1_24:1 **0.831**

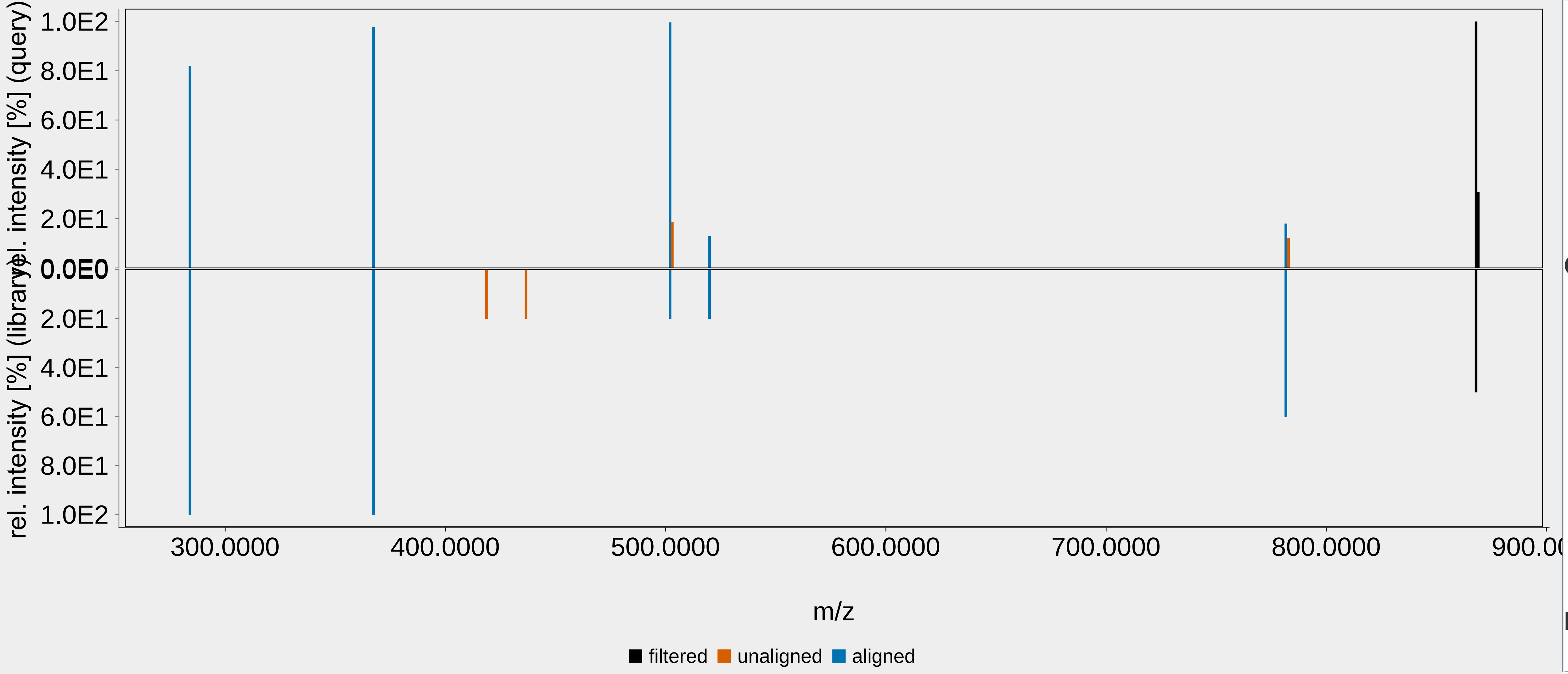

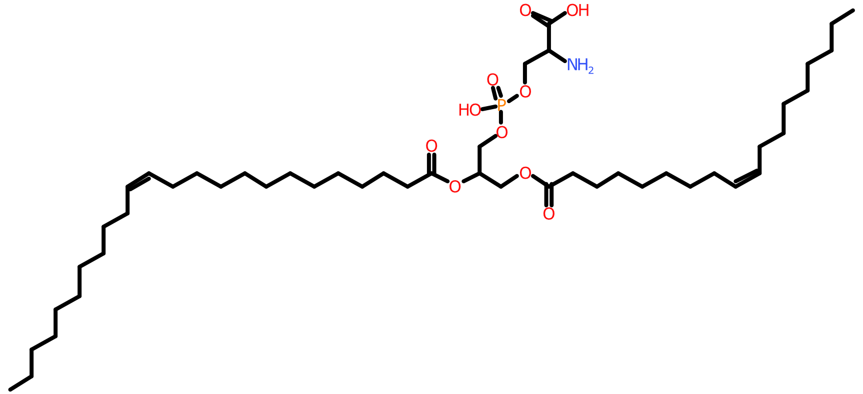

Compound information

Name: PS 18:1_24:1

Formula: C48H90NO10P

Ion type: [M-H]-

Precursor m/z: 870.62296

Ccs: 292.75

InChI key:

SMILES:

Num peaks: 8

Instrument information

Database links

Other information

PE(P-18:0/18:1); [M-H]- C41H79N1O7P1 0.784

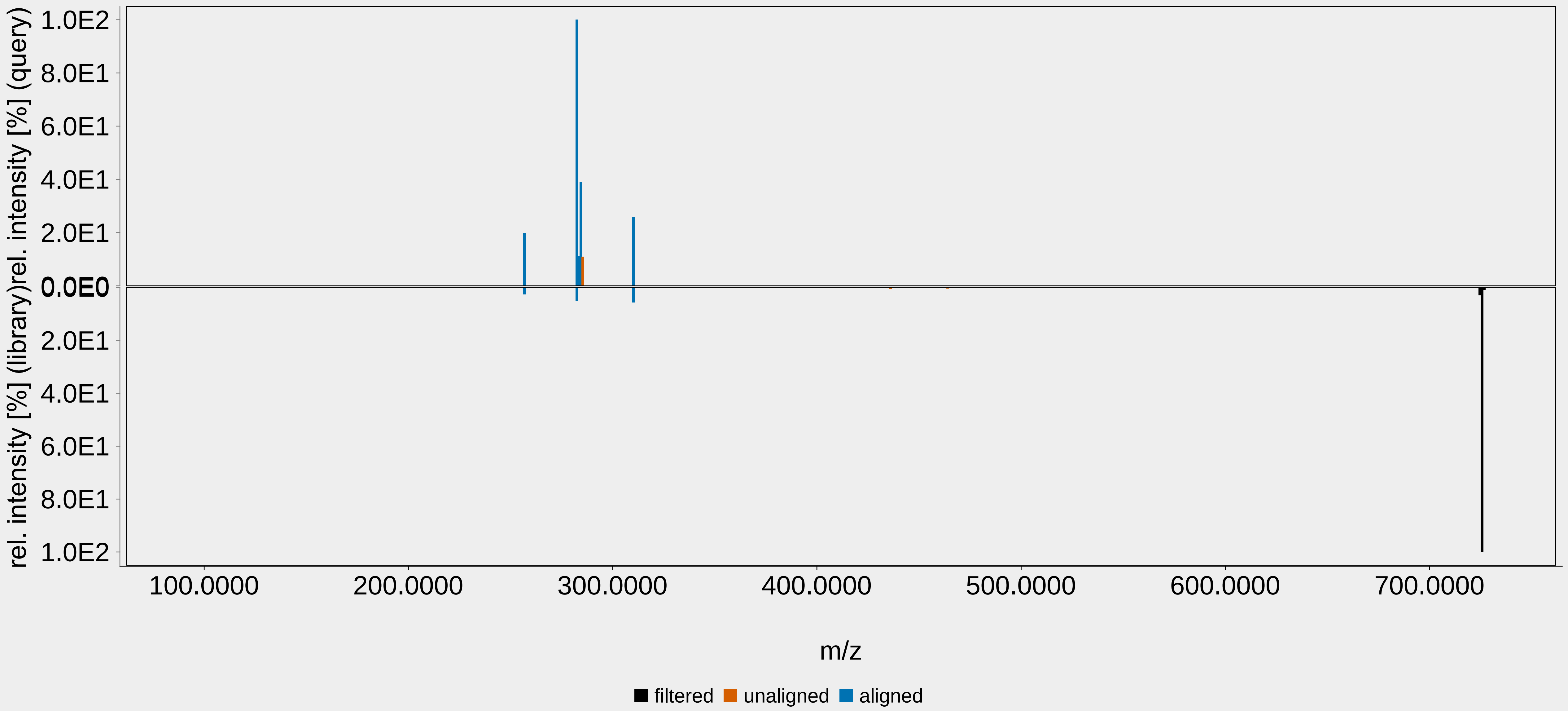

Compound information

Name: PE(P-18:0/18:1); [M-H]-

Exact mass: 0.0

Ion type: M-H

Precursor m/z: 728.559

Charge: 1

Polarity: Negative

Instrument information

Instrument type: HCD; Velos

Ion source: LC-ESI

Ms level: 2

Acquisition: Commercial

Database links

Other information

Principal investigator: Thomas Metz

Data collector: Thomas Metz

Entry ID: CCMSLIB00010072263

PE(P-18:0/18:1); [M-H]- C41H79N1O7P1 0.780

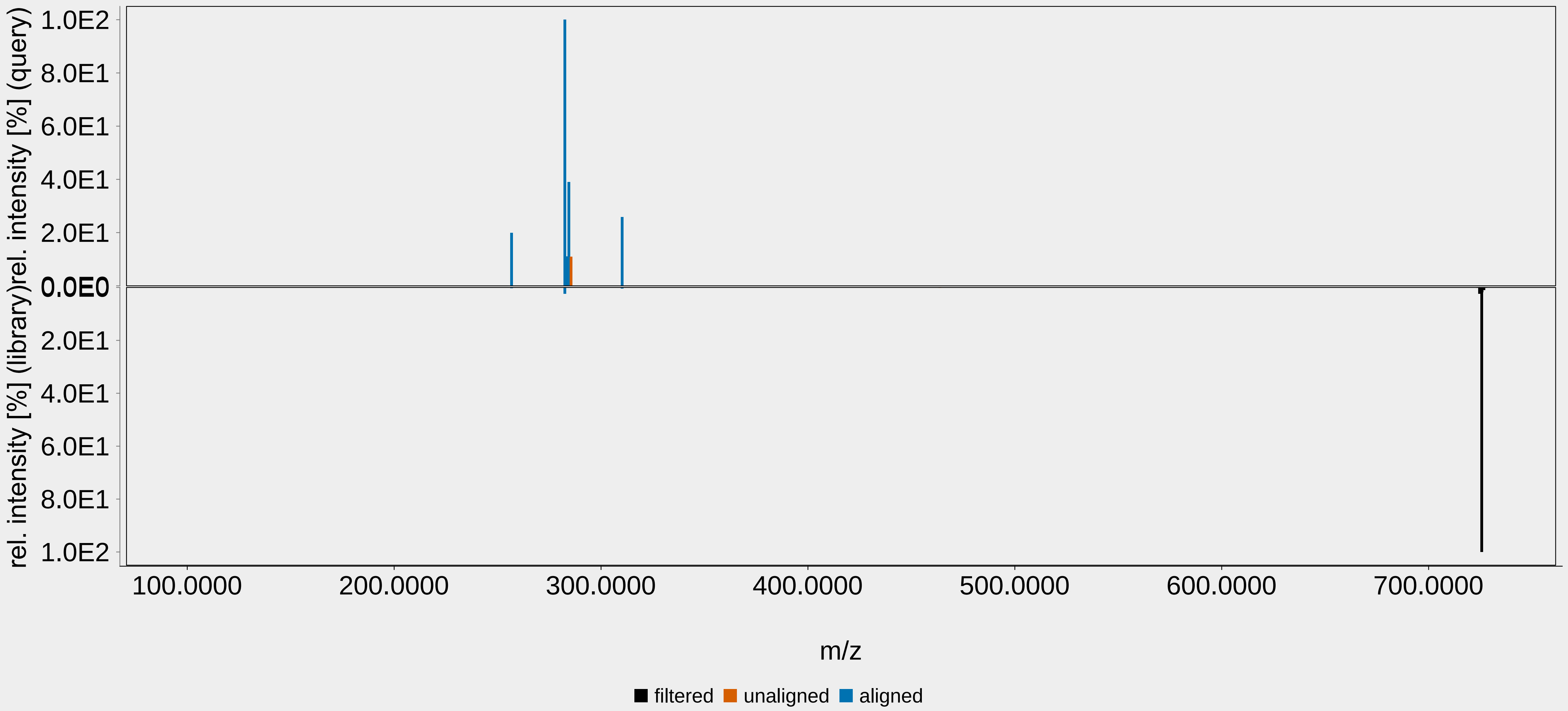

Compound information

Name: PE(P-18:0/18:1); [M-H]-

Exact mass: 0.0

Ion type: M-H

Precursor m/z: 728.559

Charge: 1

Polarity: Negative

Instrument information

Instrument type: HCD; Velos

Ion source: LC-ESI

Ms level: 2

Acquisition: Commercial

Database links

Other information

Principal investigator: Thomas Metz

Data collector: Thomas Metz

Entry ID: CCMSLIB00010068572

Spectral Match to 1-Palmitoyl-2-hydroxy-sn-glycero-3-phosphoethanolamine from NIST14

0.944

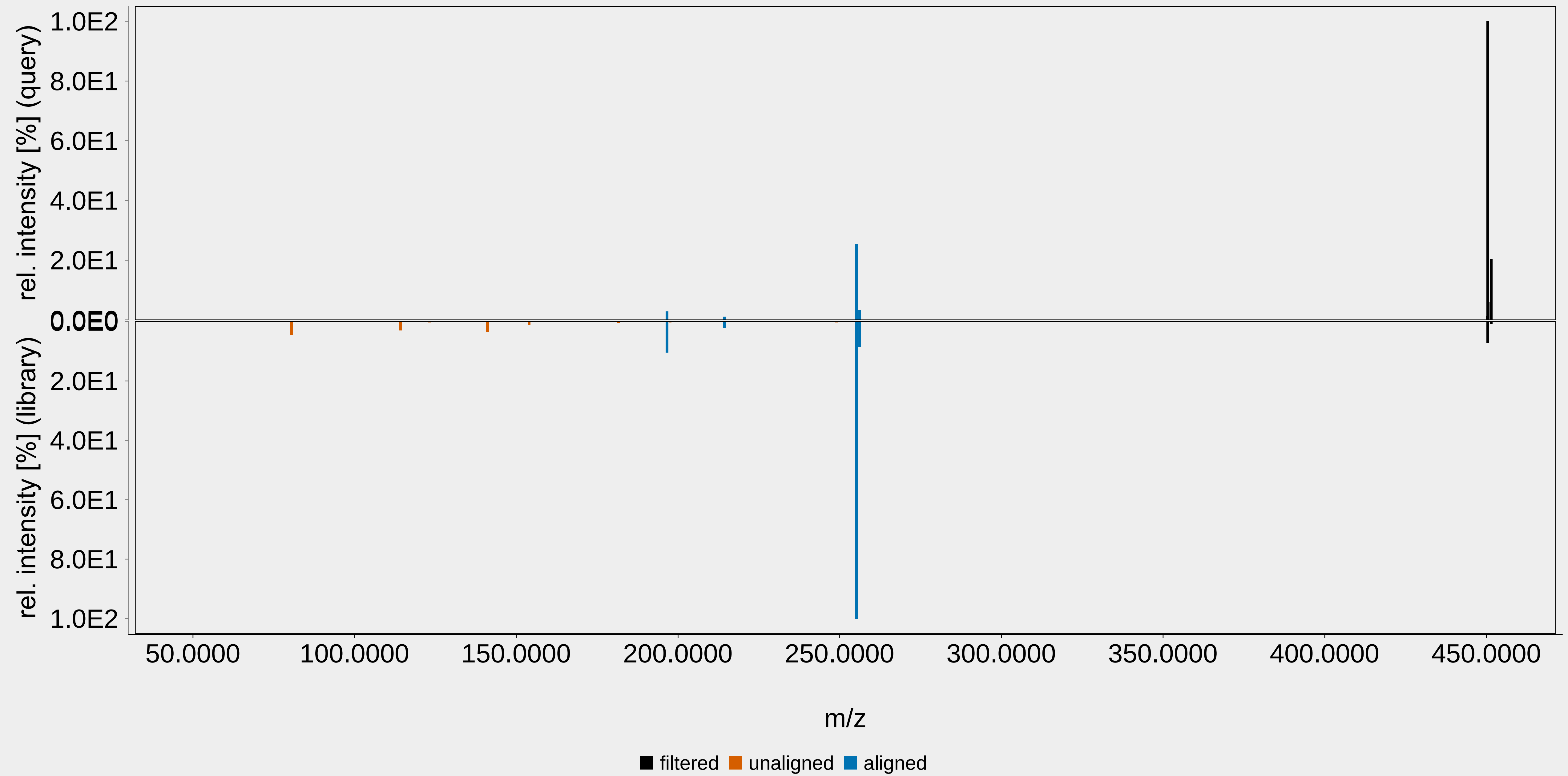

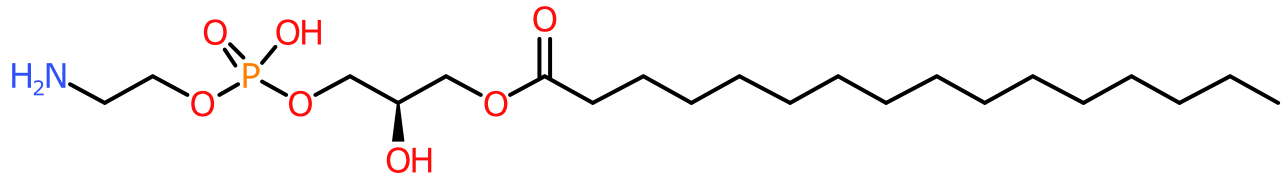

Compound information

Name: Spectral Match to

Exact mass: 453.285

Ion type: M-H

Precursor m/z: 452.279

Charge: 1

Polarity: Negative

InChI:

SMILES:

Instrument information

Instrument type: qTof

Ion source: ESI

Ms level: 2

Acquisition: Isolated

Database links

CAS: 53862354

Other information

Principal investigator: Data from Kevin Bush

Data collector: Data deposited by fevargas

Entry ID: CCMSLIB00003137341

Spectral Match to 1-Palmitoyl-2-hydroxy-sn-glycero-3-phosphoethanolamine from NIST14

0.953

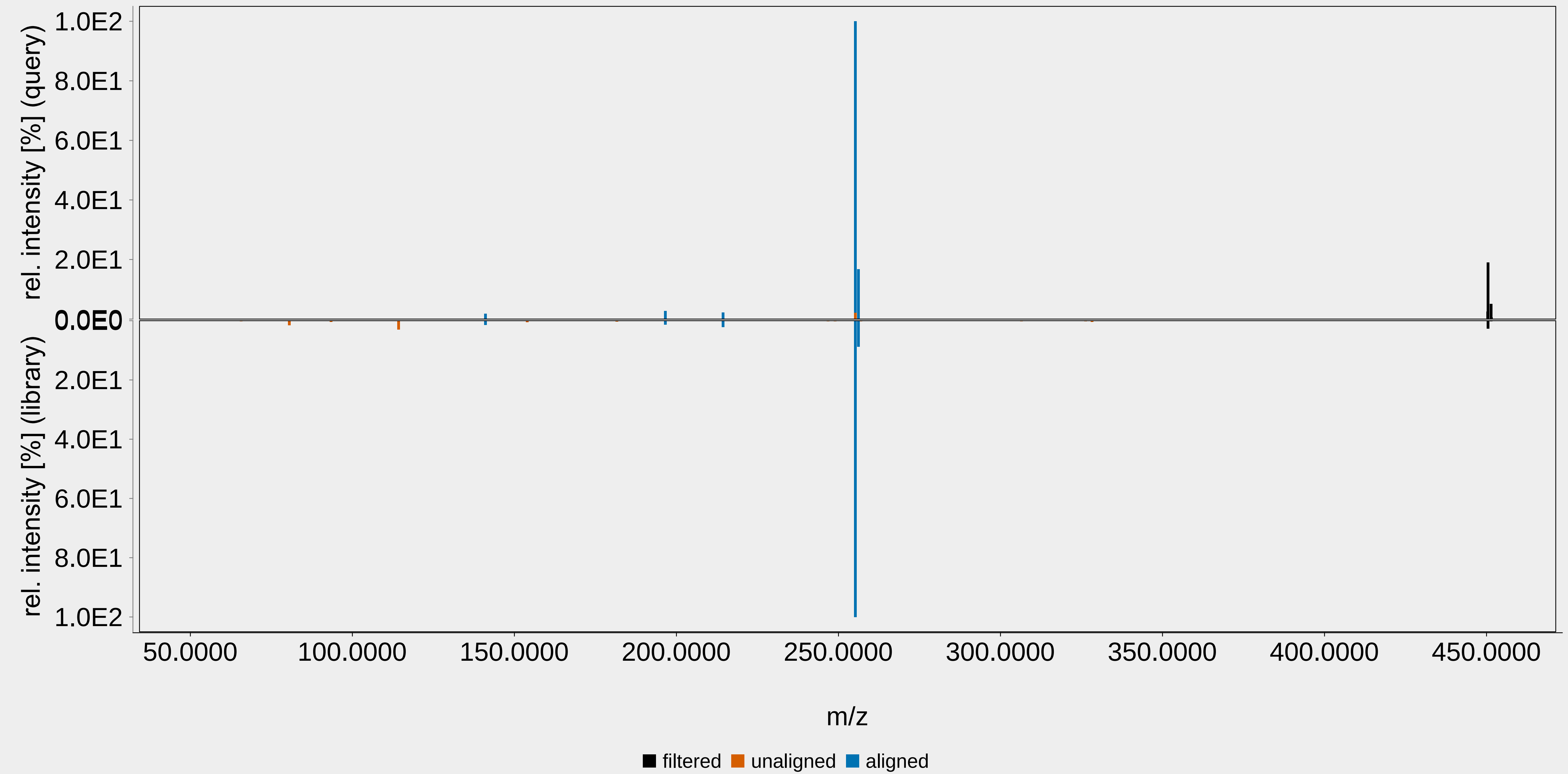

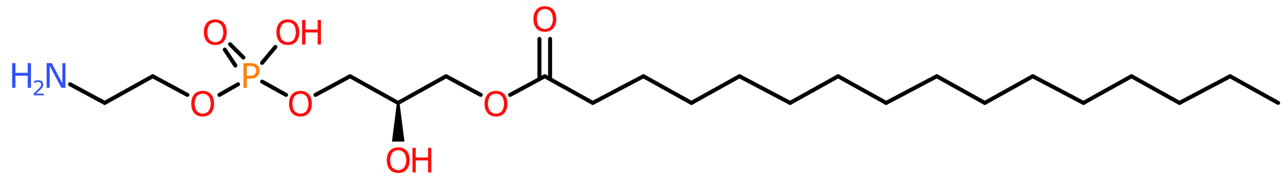

Compound information

Name: Spectral Match to

Exact mass: 453.285

Ion type: M-H

Precursor m/z: 452.279

Charge: 1

Polarity: Negative

InChI:

SMILES:

Instrument information

Instrument type: qTof

Ion source: ESI

Ms level: 2

Acquisition: Isolated

Database links

CAS: 53862354

Other information

Principal investigator: Data from Kevin Bush

Data collector: Data deposited by fevargas

Entry ID: CCMSLIB00003137726

PA 18:1_22:1

0.808

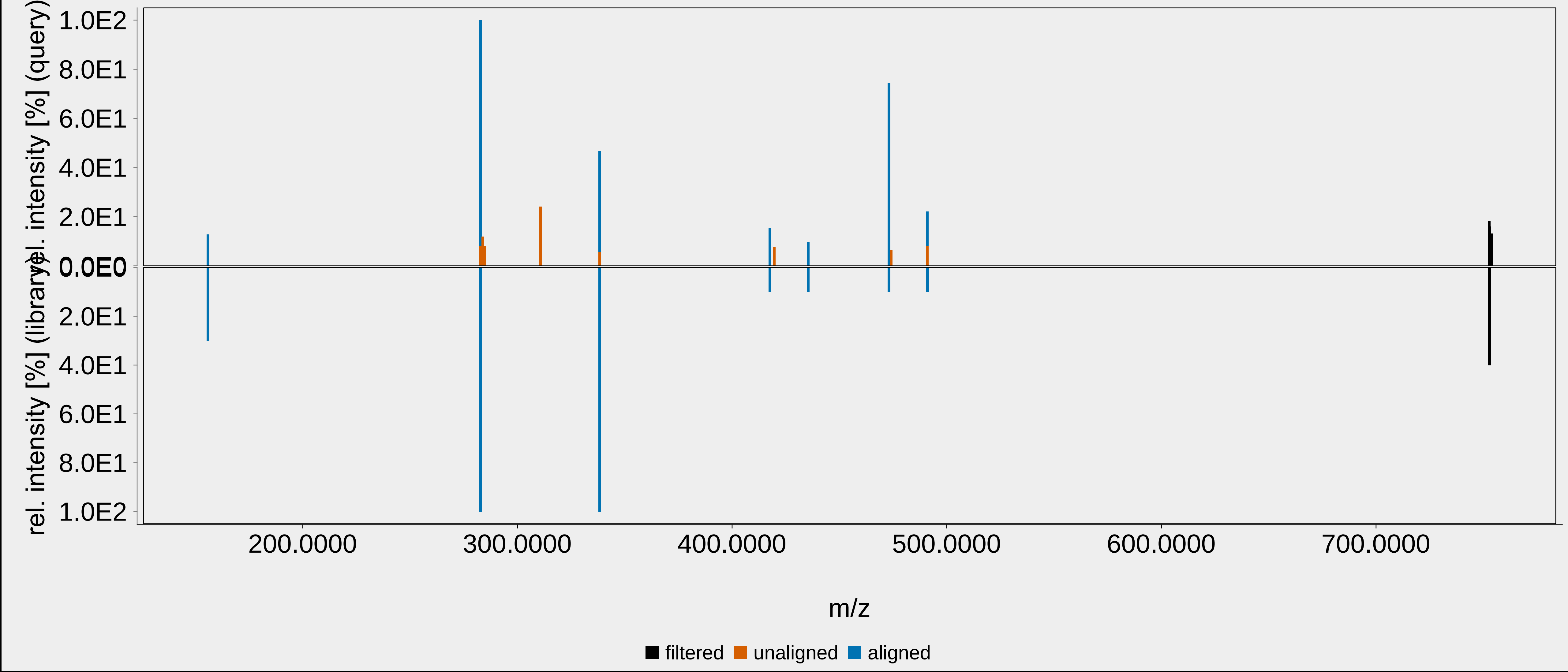

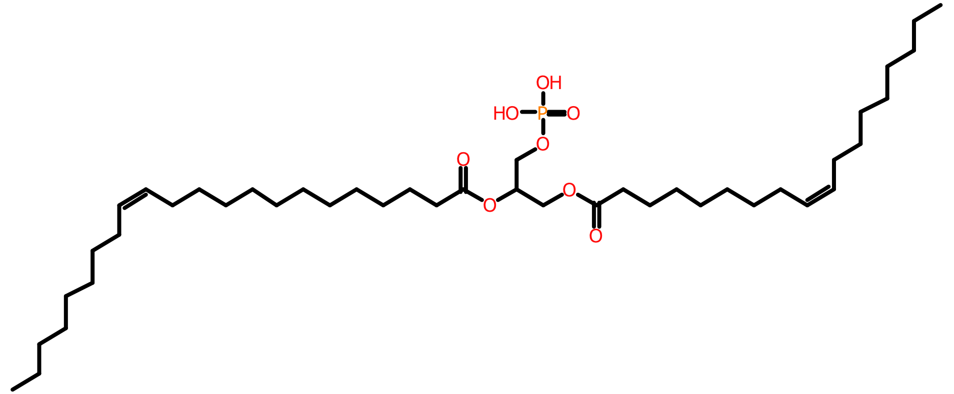

Compound information

Name: PA 18:1_22:1

Formula: C43H81O8P

Ion type: [M-H]-

Precursor m/z: 755.55963

Ccs: 271.45

InChI key:

SMILES:

Num peaks: 8

Instrument information

Database links

Other information

PE(20:1/0:0); [M-H]- C25H49N1O7P1

0.739

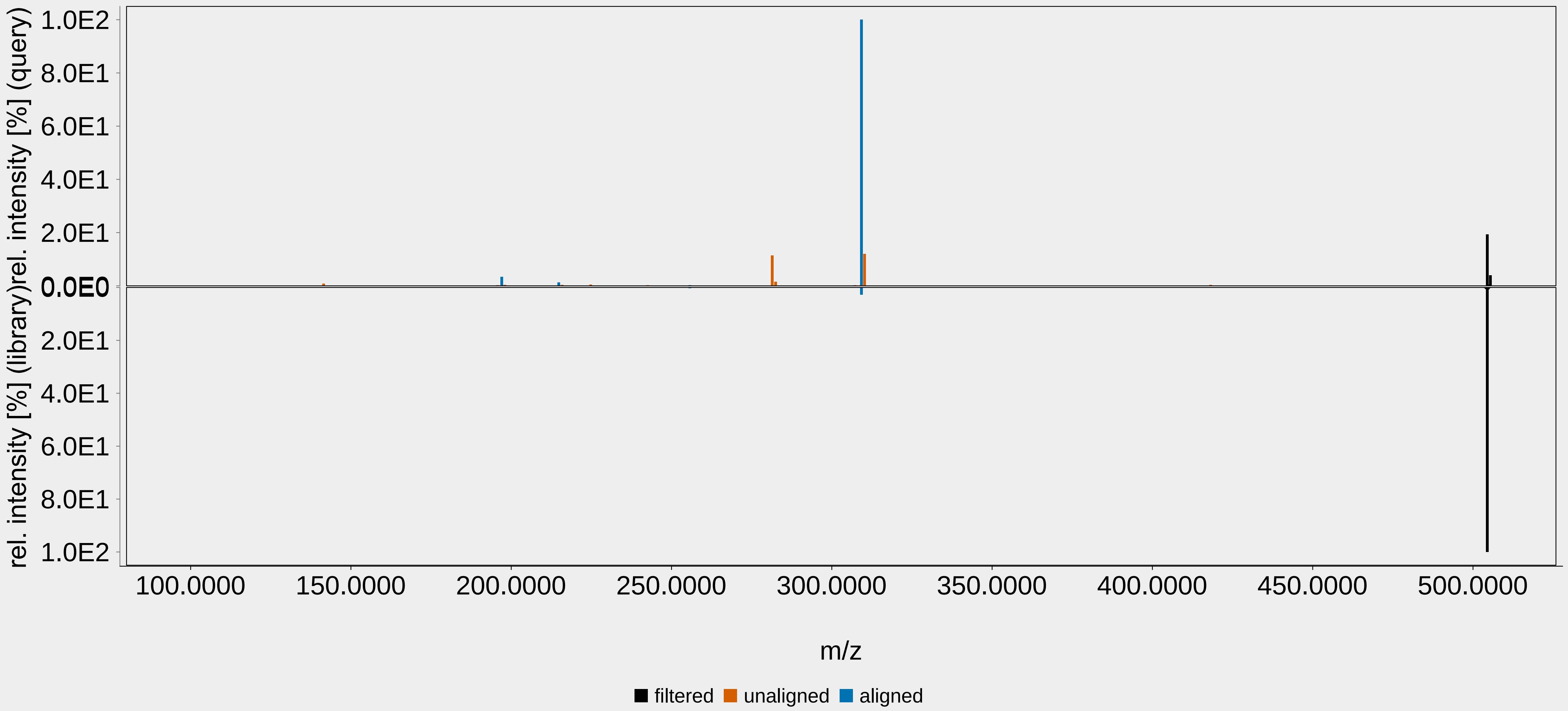

Compound information

Name: PE(20:1/0:0); [M-H]- C25H49N1O7P1

Exact mass: 0.0

Ion type: M-H

Precursor m/z: 506.325

Charge: 1

Polarity: Negative

Instrument information

Instrument type: HCD; Velos

Ion source: LC-ESI

Ms level: 2

Acquisition: Commercial

Database links

Other information

Principal investigator: Thomas Metz

Data collector: Thomas Metz

Entry ID: CCMSLIB00010086765

PE(20:1/0:0); [M-H]- C25H49N1O7P1 **0.757**

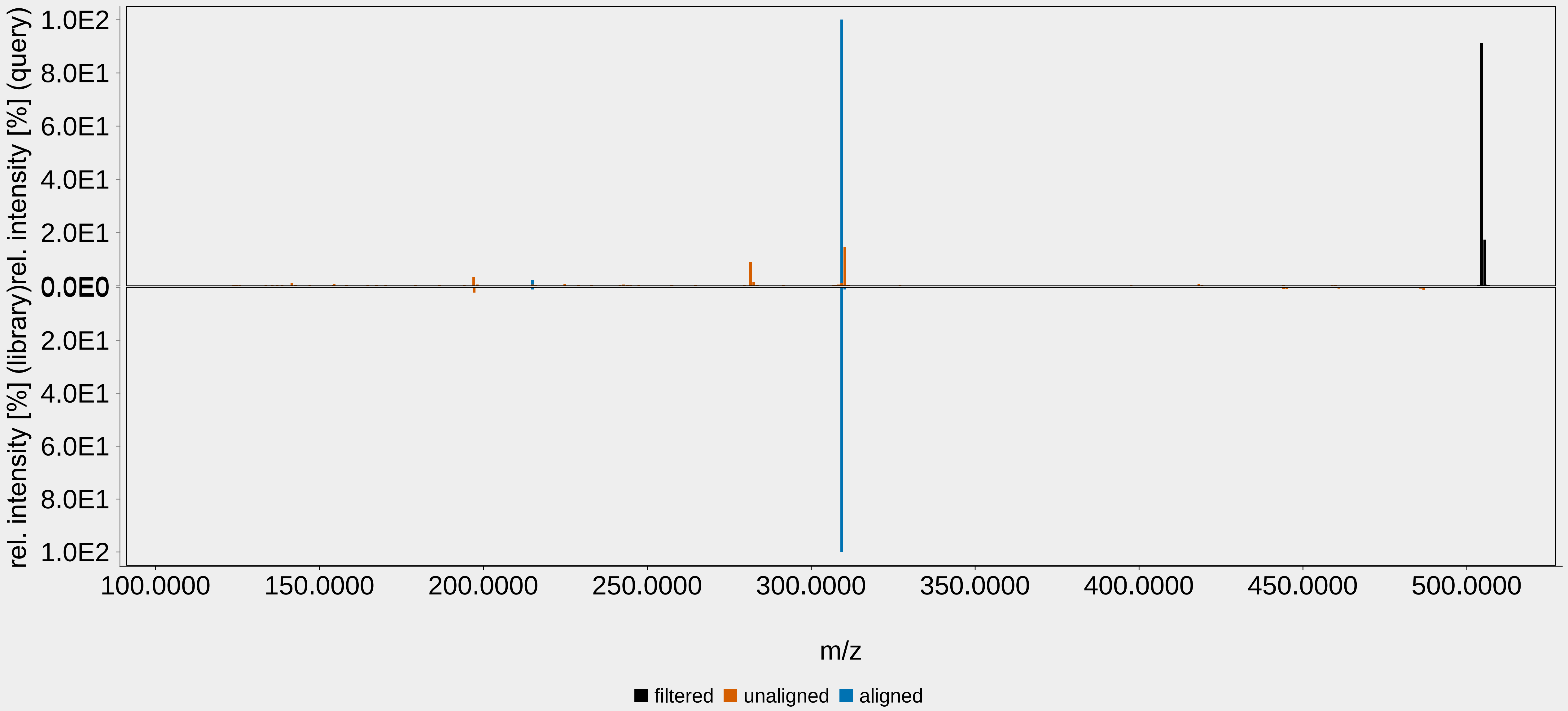

Compound information

Name: PE(20:1/0:0); [M-H]- C25H49N1O7P1

Exact mass: 0.0

Ion type: M-H

Precursor m/z: 506.325

Charge: 1

Polarity: Negative

Instrument information

Instrument type: CID; Velos

Ion source: LC-ESI

Ms level: 2

Acquisition: Commercial

Database links

Other information

Principal investigator: Thomas Metz

Data collector: Thomas Metz

Entry ID: CCMSLIB00010063977

Canrenone

0.939

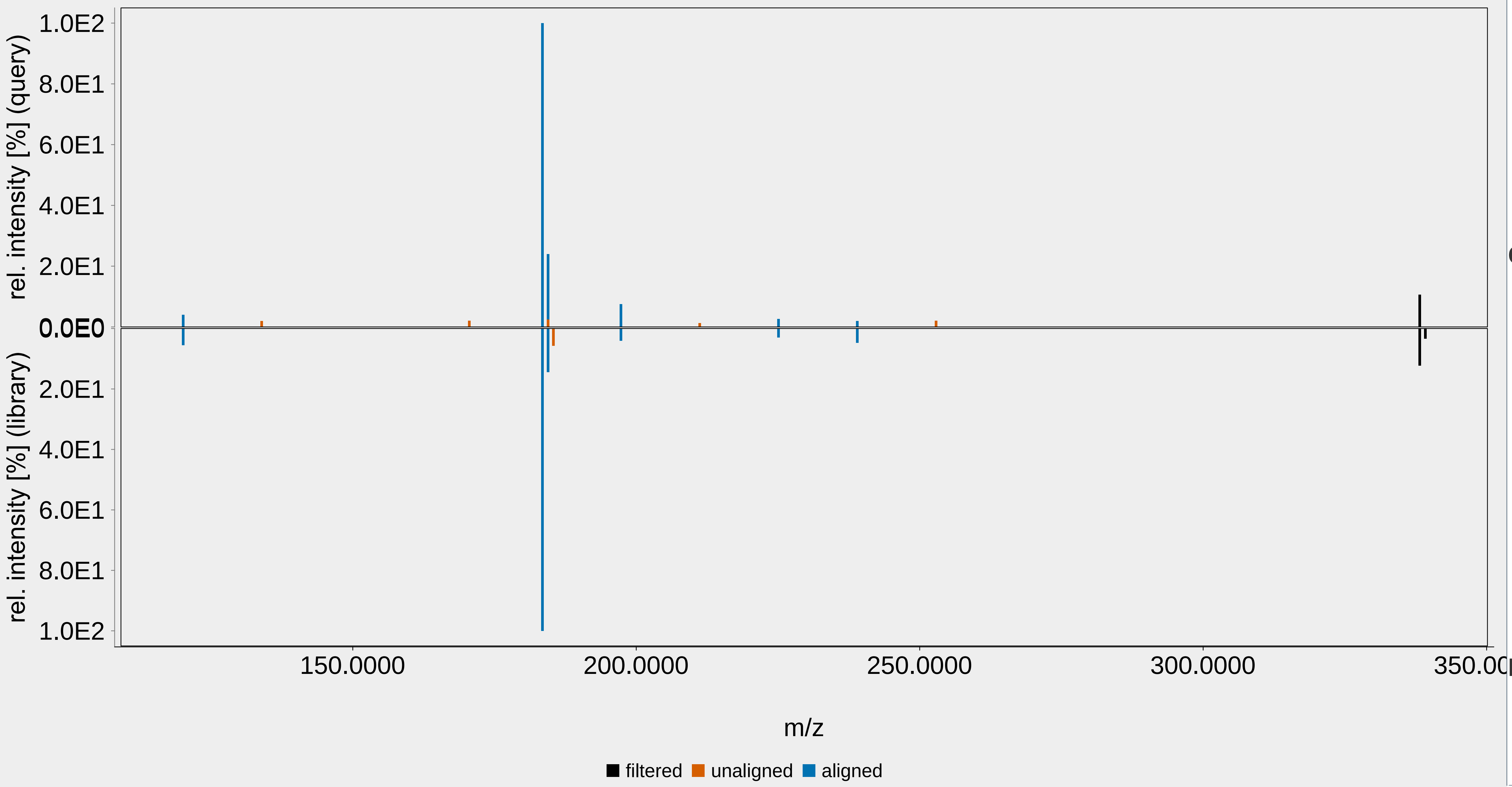

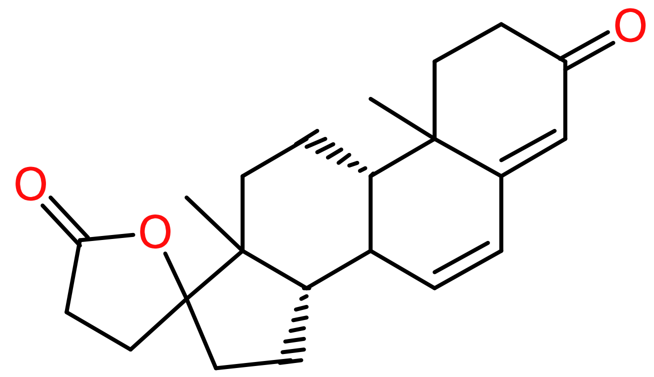

Compound information

Name: Canrenone

Formula: C22H28O3

Exact mass: 340.2038448

Ion type: [M-H]-

Precursor m/z: 339.1966

Polarity: NEGATIVE

InChI:

InChI key:

SMILES:

Num peaks: 9

Instrument information

Instrument type: LC-ESI-QTOF

Instrument: Bruker maXis Impact

Ms level: MS2

Collision energy: 40 eV

Database links

Other information

Entry ID: MSBNK-Athens_Univ-AU239460

Comment: Parent=339.1966

Canrenone, (9S,14S)-10,13-dimethylspiro[2,8,9,11,12,14,15,16-octahydro-1H-cyclopenta[a]phenanthrene-17,5`-oxolane]-2`,3-dione

0.939

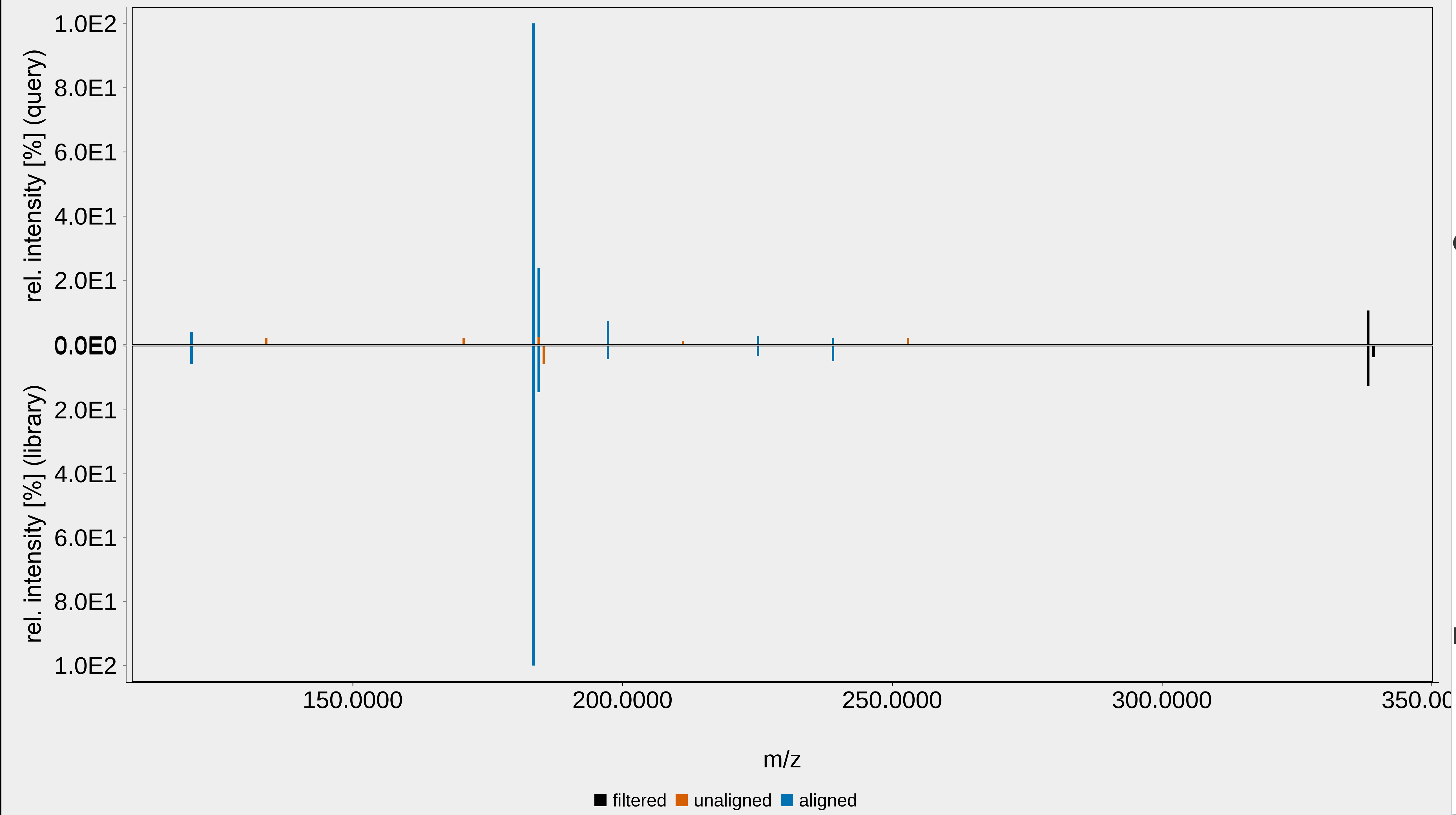

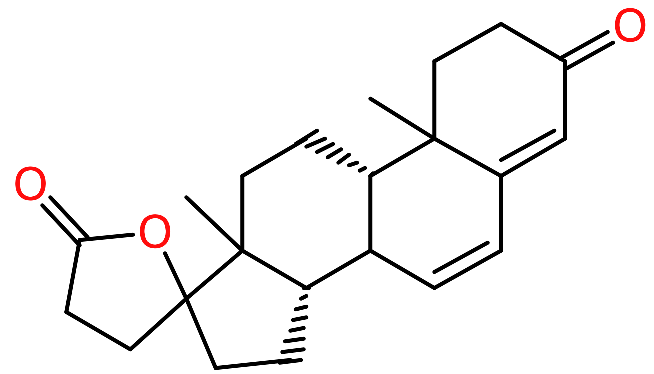

Compound information

Name: Canrenone,

Formula: C22H28O3

Mol. weight: 340.2038448

Exact mass: 340.2038448

Ion type: [M-H]-

Precursor m/z: 339.1966

Polarity: negative

InChI:

InChI key:

SMILES:

Instrument information

Instrument type: LC-ESI-QTOF

Instrument: Bruker maXis Impact

Ion source: ESI

Resolution: 35000

Ms level: MS2

Collision energy: 40 eV

Database links

Pubchem: 24728626

MoNA ID: AU239460

Chemspider: 21112020

CAS: 976-71-6

Other information

Principal investigator: Nikiforos Alygizakis,

Data collector: Nikiforos Alygizakis, Katerina

Guanosine diphosphate - 30.0 eV

0.876

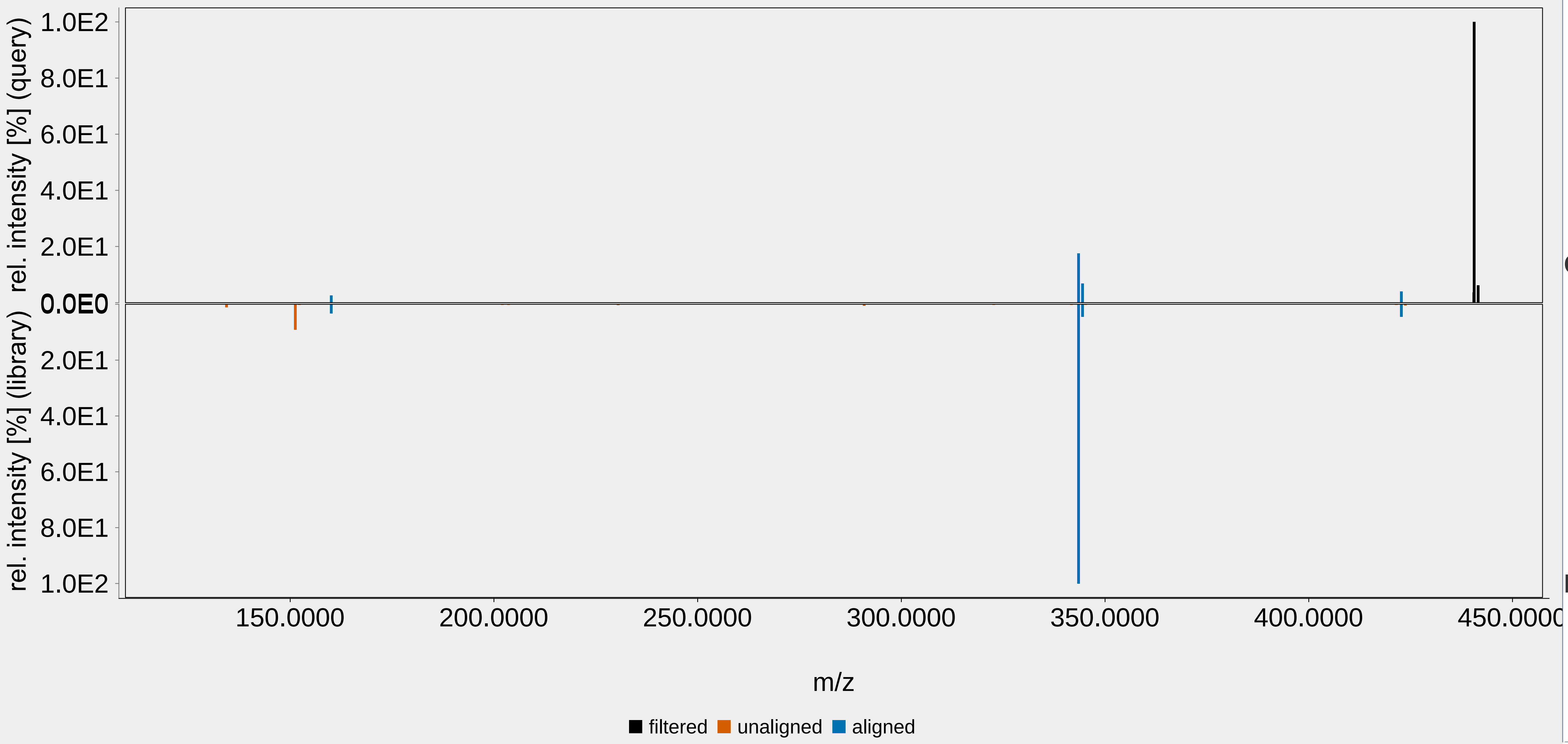

Compound information

Name: Guanosine diphosphate - 30.0 eV

Exact mass: 0.0

Ion type: M-H

Precursor m/z: 442.017

Charge: 1

Polarity: Negative

SMILES:

Instrument information

Instrument type: Orbitrap

Ion source: ESI

Ms level: 2

Acquisition: Isolated

Database links

CAS:

Other information

Principal investigator: PI

Data collector: Data Collector

Entry ID: CCMSLIB00006123856

Guanosine diphosphate - 30.0 eV

0.880

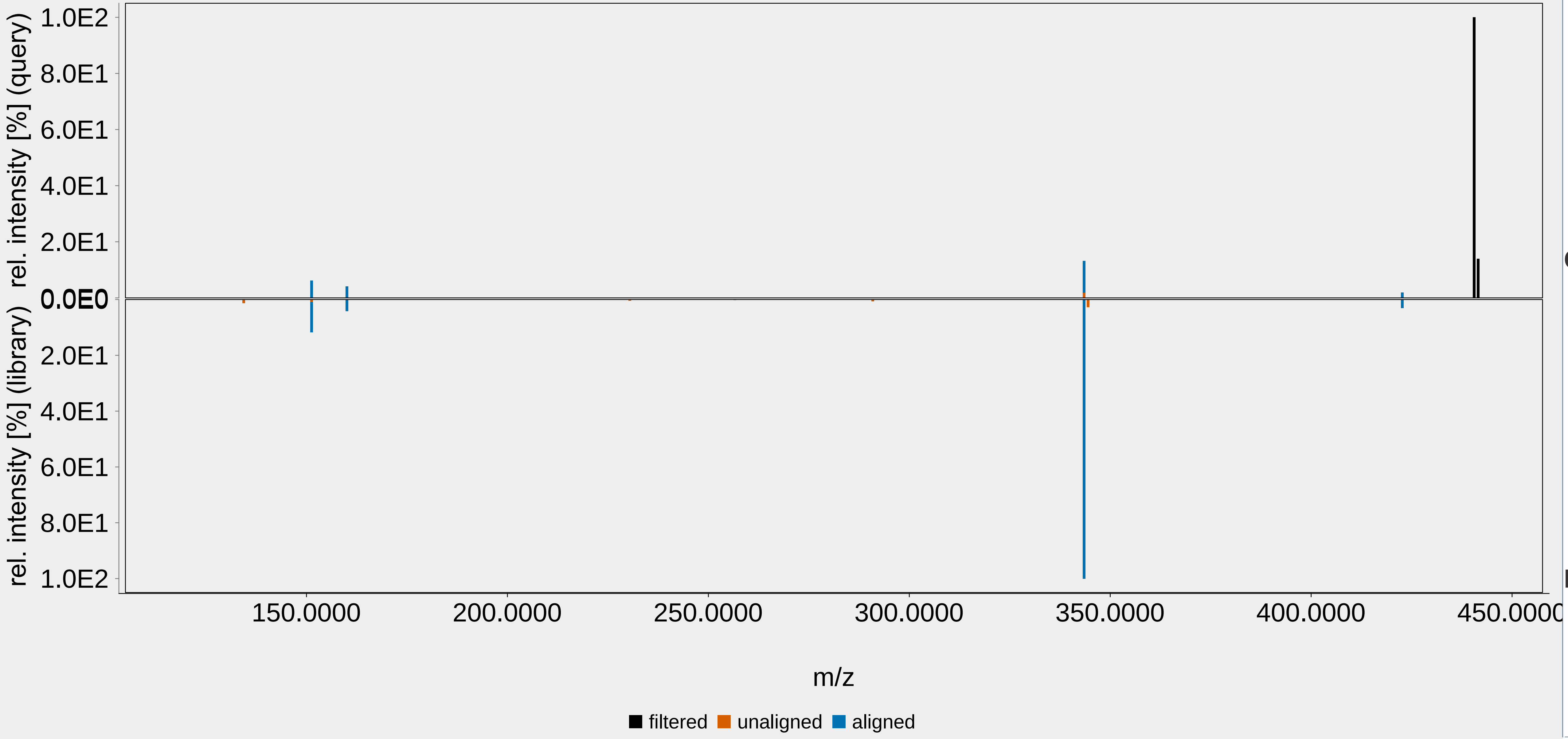

Compound information

Name: Guanosine diphosphate - 30.0 eV

Exact mass: 0.0

Ion type: M-H

Precursor m/z: 442.017

Charge: 1

Polarity: Negative

SMILES:

Instrument information

Instrument type: Orbitrap

Ion source: ESI

Ms level: 2

Acquisition: Isolated

Database links

CAS:

Other information

Principal investigator: PI

Data collector: Data Collector

Entry ID: CCMSLIB00006123838

PS 23:0_18:1

0.841

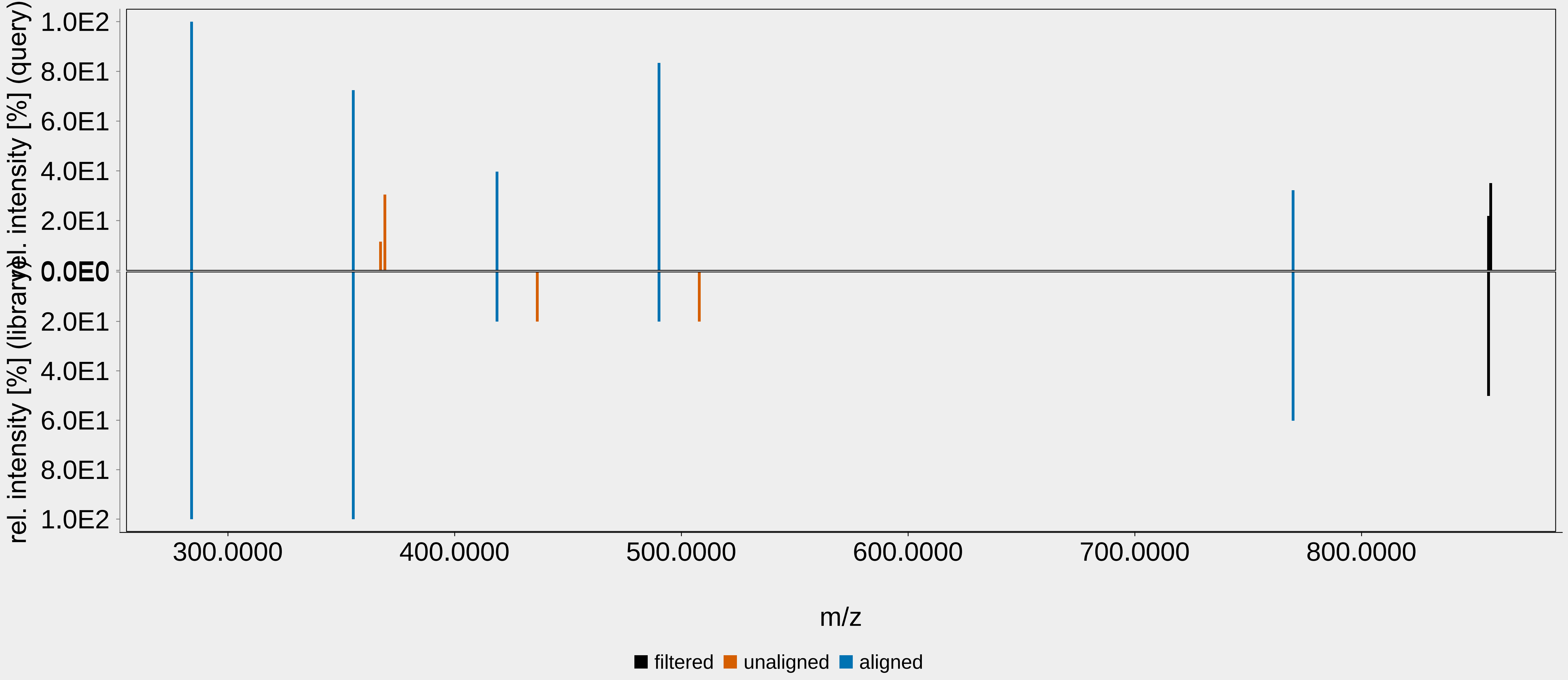

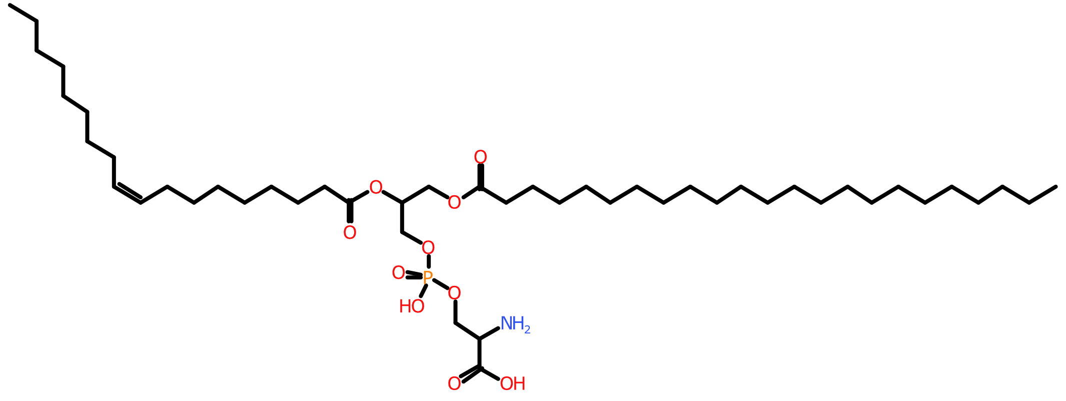

Compound information

Name: PS 23:0_18:1

Formula: C47H90NO10P

Ion type: [M-H]-

Precursor m/z: 858.62296

Ccs: 292.27

InChI key:

SMILES:

Num peaks: 8

Instrument information

Database links

Other information

PA 18:1_18:1

0.876

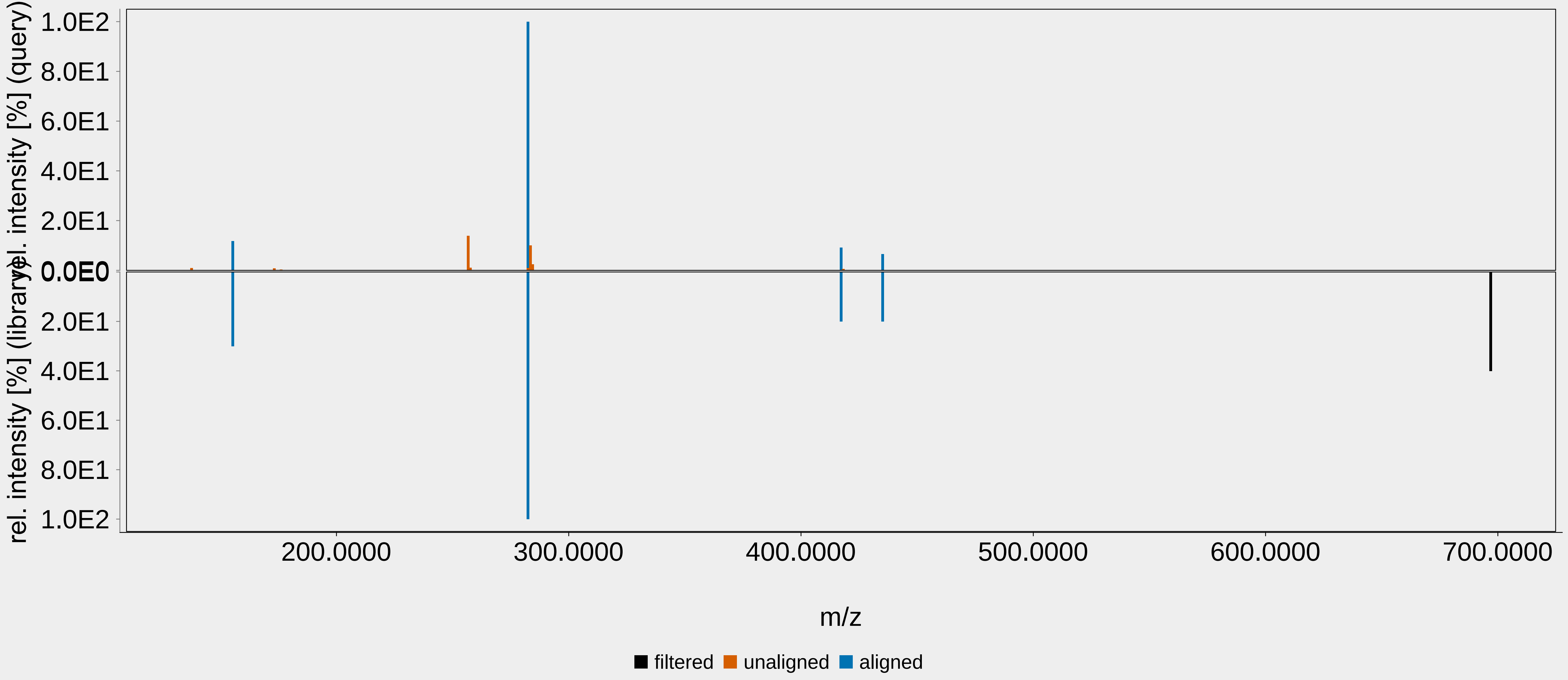

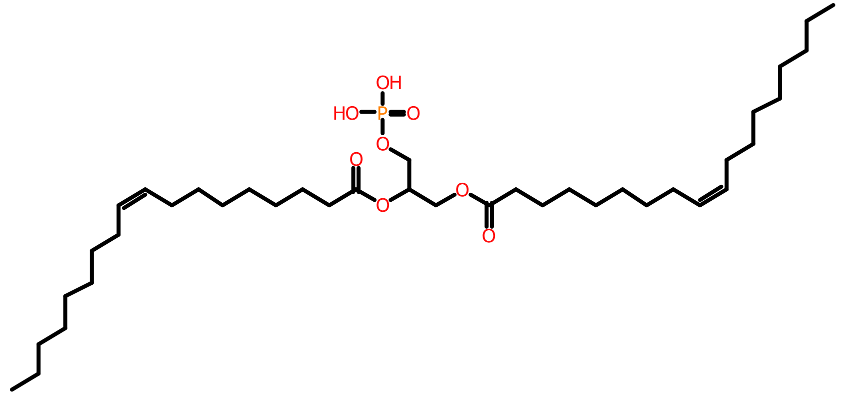

Compound information

Name: PA 18:1_18:1

Formula: C39H73O8P

Ion type: [M-H]-

Precursor m/z: 699.49703

Ccs: 257.54

InChI key: MHUWZNTUIIFHAS-CLFAGFIQNA-I

SMILES:

Num peaks: 5

Instrument information

Database links

Other information

Glutathione - 30.0 eV

0.876

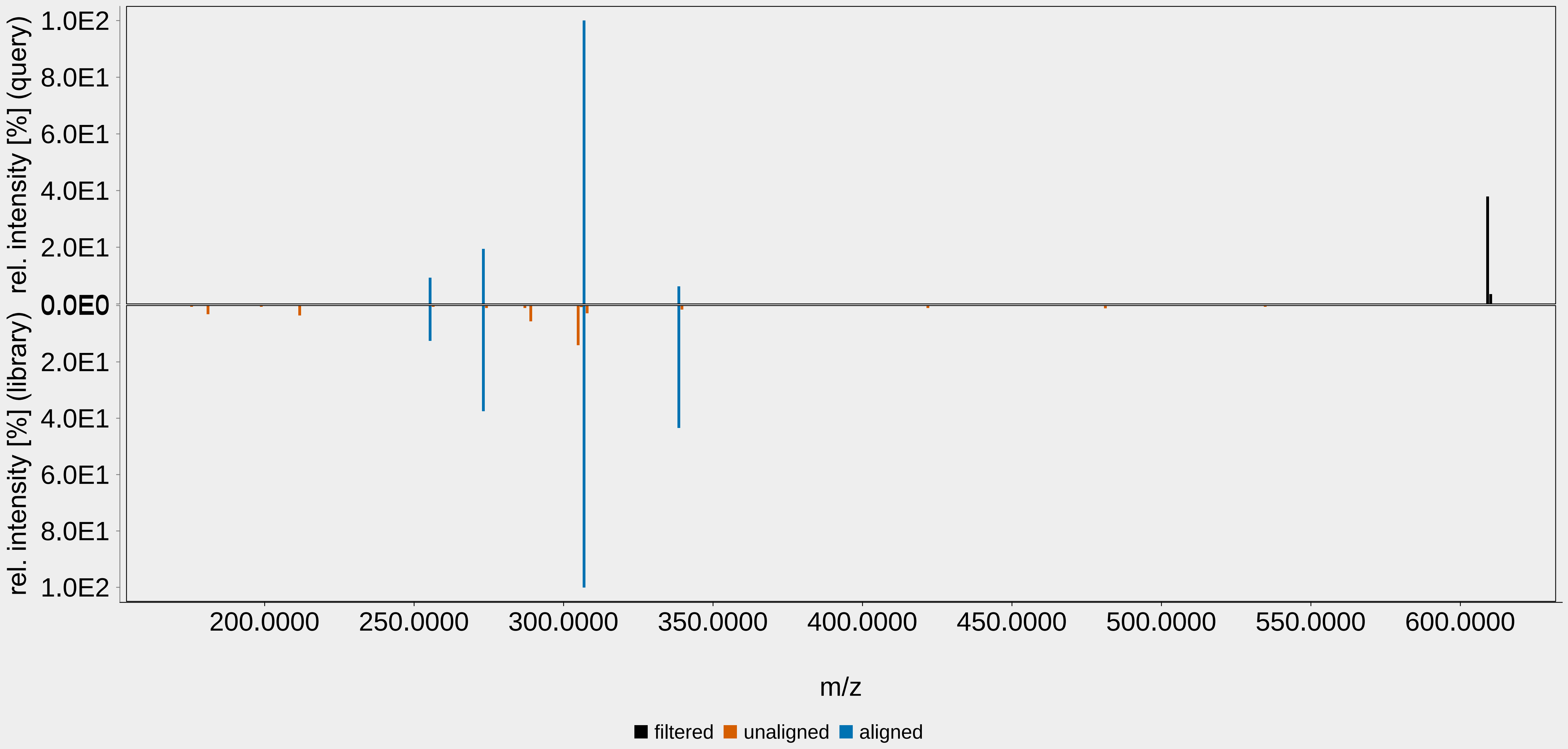

Compound information

Name: Glutathione - 30.0 eV

Exact mass: 0.0

Ion type: M-H

Precursor m/z: 611.145

Charge: 1

Polarity: Negative

SMILES:

Instrument information

Instrument type: Orbitrap

Ion source: ESI

Ms level: 2

Acquisition: Isolated

Database links

CAS:

Other information

Principal investigator: PI

Data collector: Data Collector

Entry ID: CCMSLIB00006123948

Glutathione (oxidized)

0.918

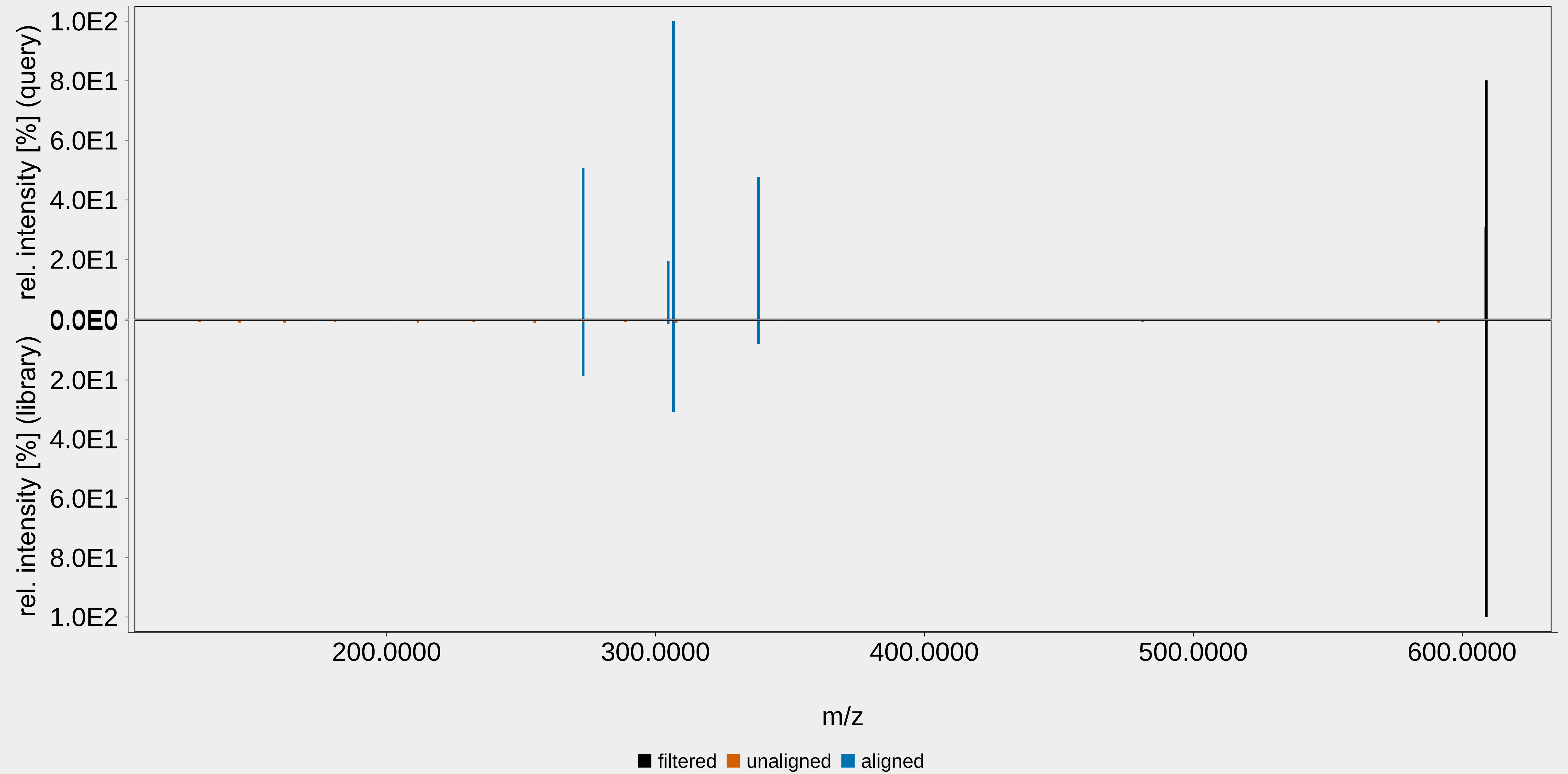

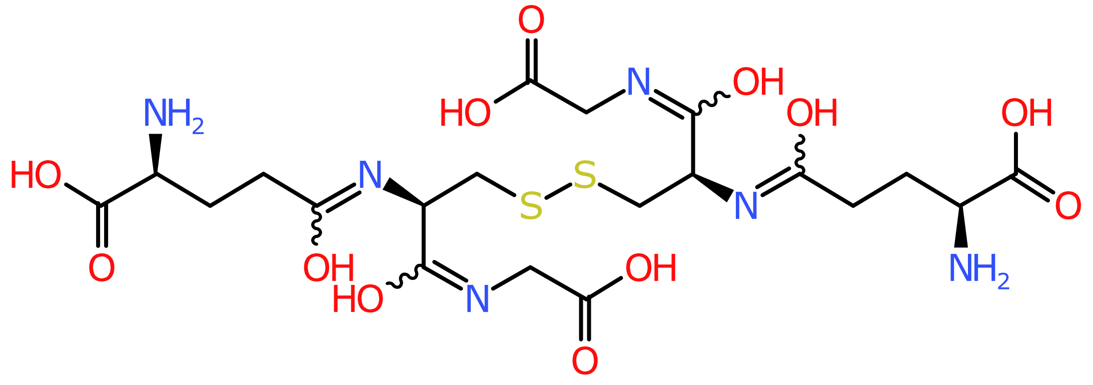

Compound information

Name: Glutathione (oxidized)

Formula: C20H32N6O12S2

Ion type: [M-H]-

Precursor m/z: 611.142

RT: 9.794383

Polarity: negative

InChI:

InChI key:

SMILES:

Instrument information

Instrument type: LC-ESI-QTOF

Instrument: SCIEX TripleTOF 6600

Ms level: MS2

Collision energy: 35 eV

Database links

Other information

Principal investigator: Michael Sa and Megan

Data collector: Michael Sa and Megan

PE(18:1/20:1); [M-H]- C43H81N1O8P1 **0.917**

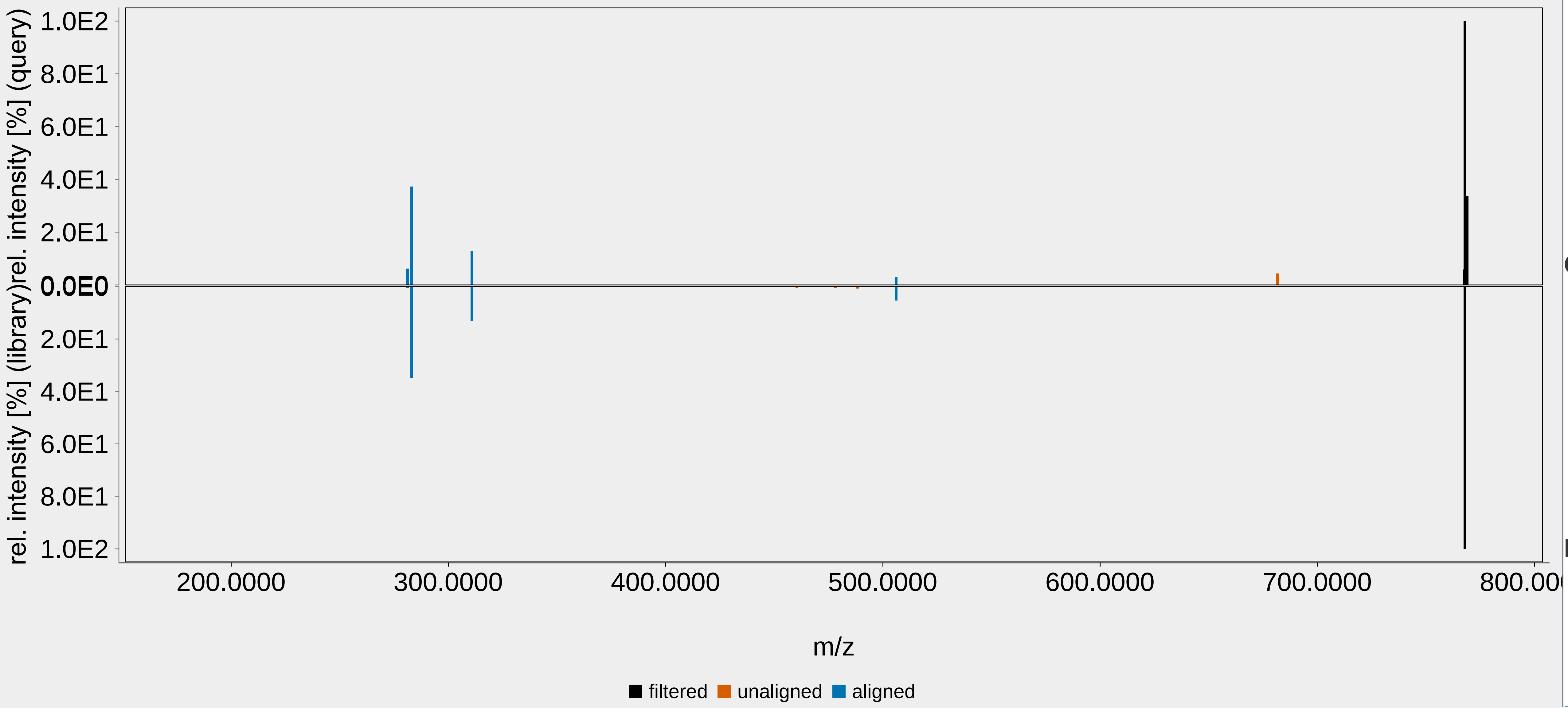

Compound information

Name: PE(18:1/20:1); [M-H]- C43H81N1O8P1

Exact mass: 0.0

Ion type: M-H

Precursor m/z: 770.57

Charge: 1

Polarity: Negative

Instrument information

Instrument type: CID; Lumos

Ion source: LC-ESI

Ms level: 2

Acquisition: Commercial

Database links

Other information

Principal investigator: Thomas Metz

Data collector: Thomas Metz

Entry ID: CCMSLIB00010059697

PE(18:1/20:1); [M-H]- C43H81N1O8P1 **0.918**

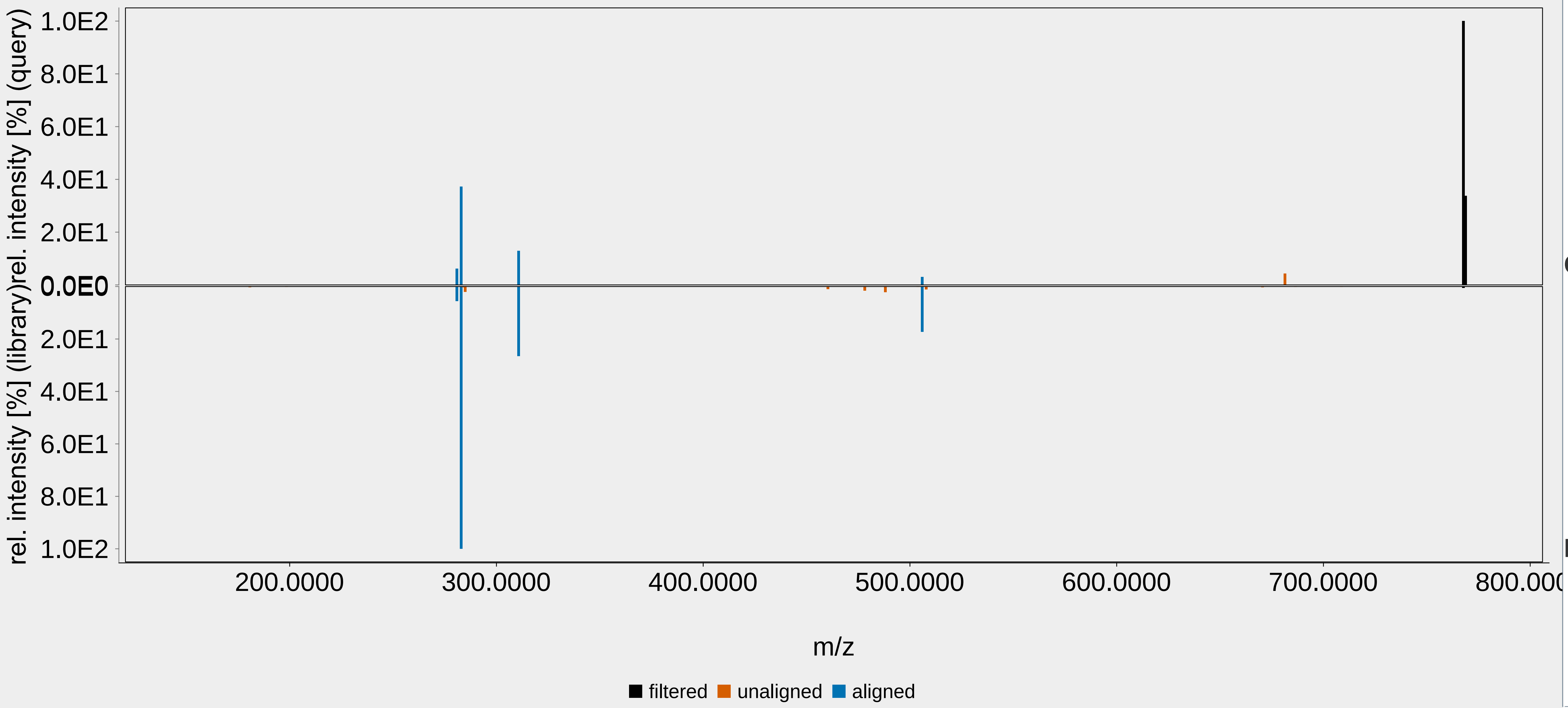

Compound information

Name: PE(18:1/20:1); [M-H]- C43H81N1O8P1

Exact mass: 0.0

Ion type: M-H

Precursor m/z: 770.57

Charge: 1

Polarity: Negative

Instrument information

Instrument type: CID; Lumos

Ion source: LC-ESI

Ms level: 2

Acquisition: Commercial

Database links

Other information

Principal investigator: Thomas Metz

Data collector: Thomas Metz

Entry ID: CCMSLIB00010059418

Docosahexaenoic acid

0.865

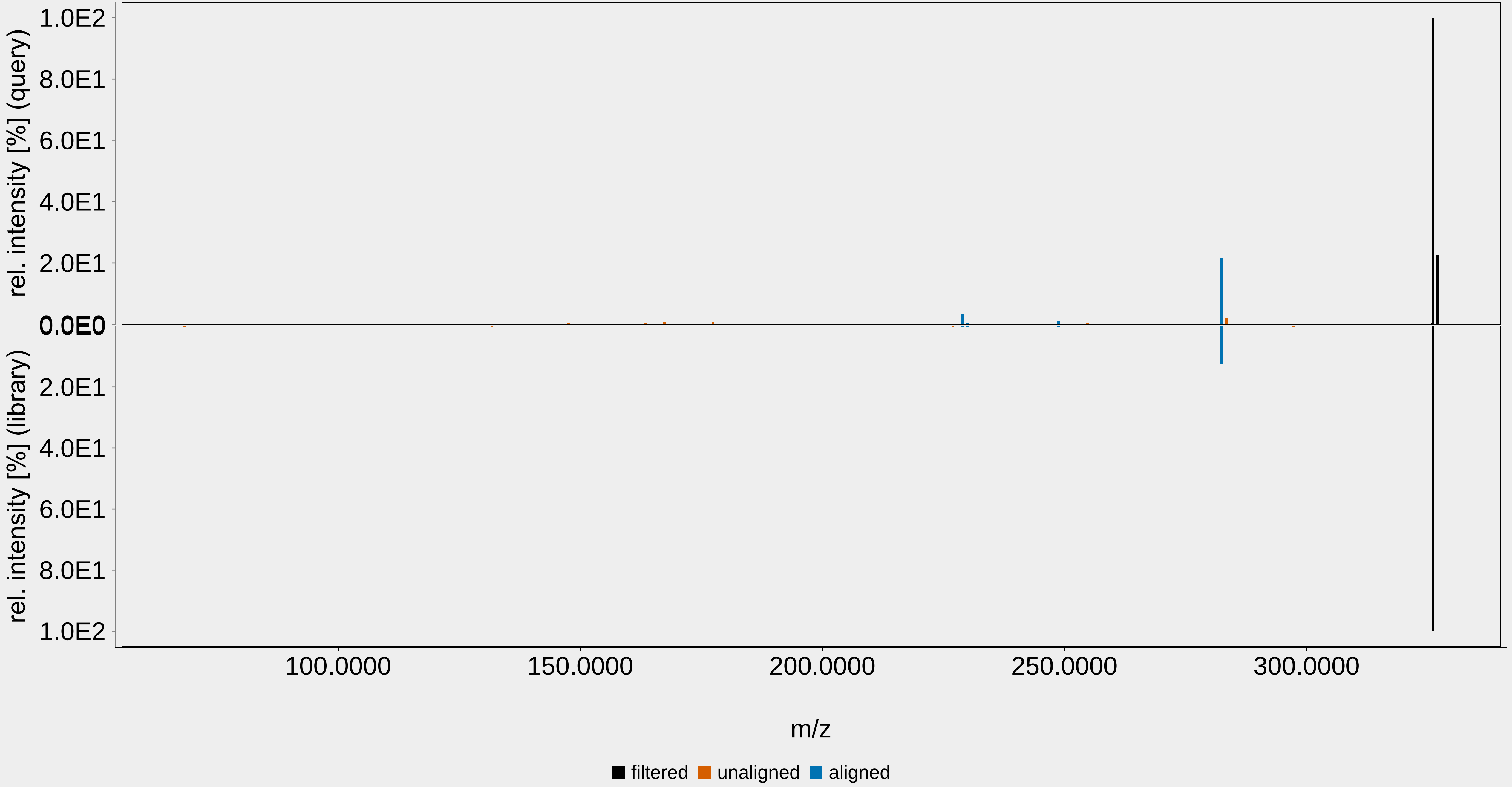

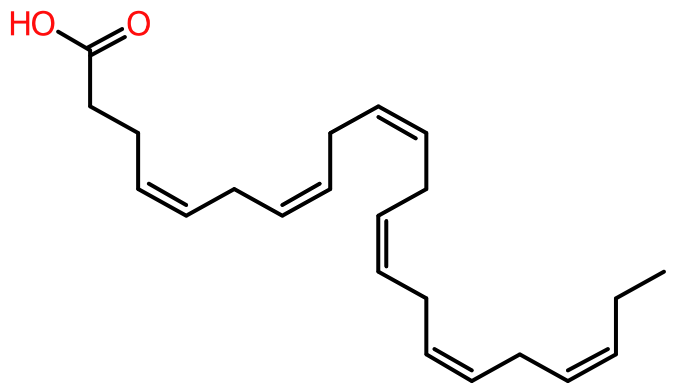

Compound information

Name: Docosahexaenoic acid

Formula: C22H32O2

Exact mass: 328.24023

Ion type: [M-H]-

Precursor m/z: 327.233

Polarity: NEGATIVE

InChI:

InChI key:

SMILES:

Num peaks: 9

Instrument information

Instrument type: LC-ESI-QTOF

Instrument: maXis plus UHR-ToF-MS, Bruker

Ms level: MS2

Collision energy: 10

Database links

Other information

Entry ID: MSBNK-BGC_Munich-RP029611

Comment: Parent=327.233

Spectral Match to cis-4,7,10,13,16,19-Docosahexaenoic acid from NIST14

0.882

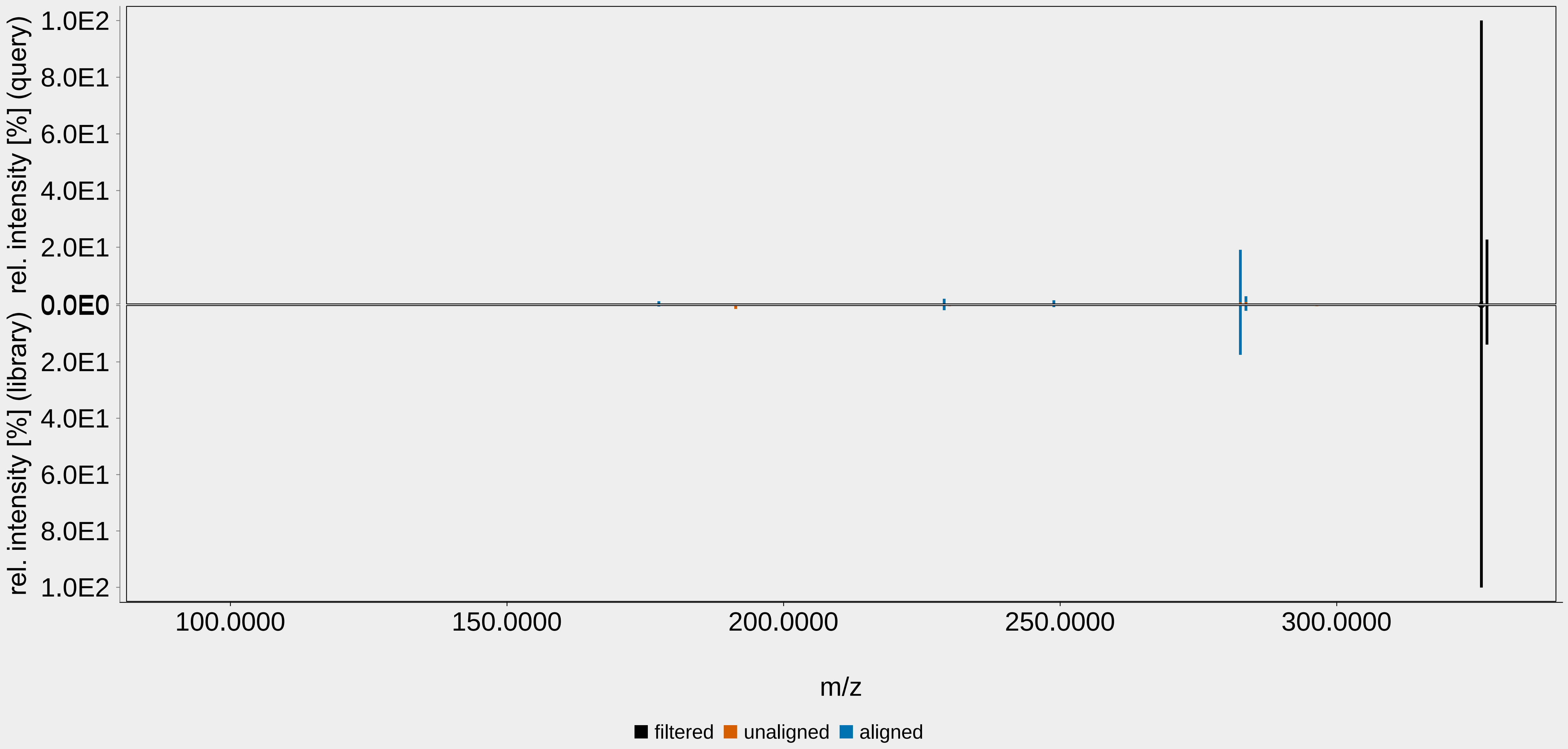

Compound information

Name: Spectral Match to

Exact mass: 0.0

Ion type: M-H

Precursor m/z: 327.232

Charge: 1

Polarity: Negative

SMILES:

Instrument information

Instrument type: qTof

Ion source: LC-ESI

Ms level: 2

Acquisition: Isolated

Database links

CAS: 6217545

Other information

Principal investigator: Data from Charles

Data collector: Data deposited by alchemistmatt

Entry ID: CCMSLIB00003137612

PE(20:0/0:0); [M-H]- C25H51N1O7P1 **0.757**

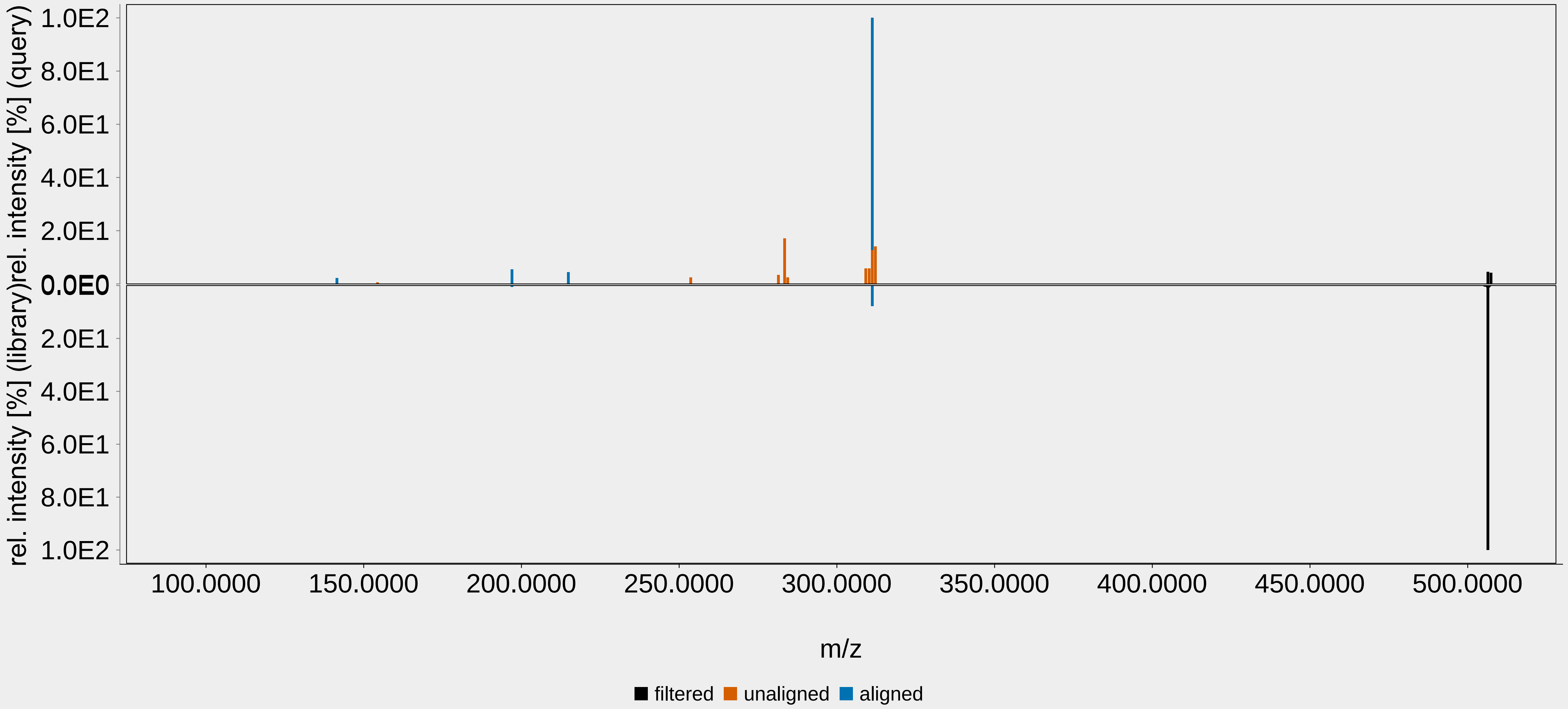

Compound information

Name: PE(20:0/0:0); [M-H]- C25H51N1O7P1

Exact mass: 0.0

Ion type: M-H

Precursor m/z: 508.34

Charge: 1

Polarity: Negative

Instrument information

Instrument type: HCD; Velos

Ion source: LC-ESI

Ms level: 2

Acquisition: Commercial

Database links

Other information

Principal investigator: Thomas Metz

Data collector: Thomas Metz

Entry ID: CCMSLIB00010063231

Adenosine diphosphate - 30.0 eV

0.874

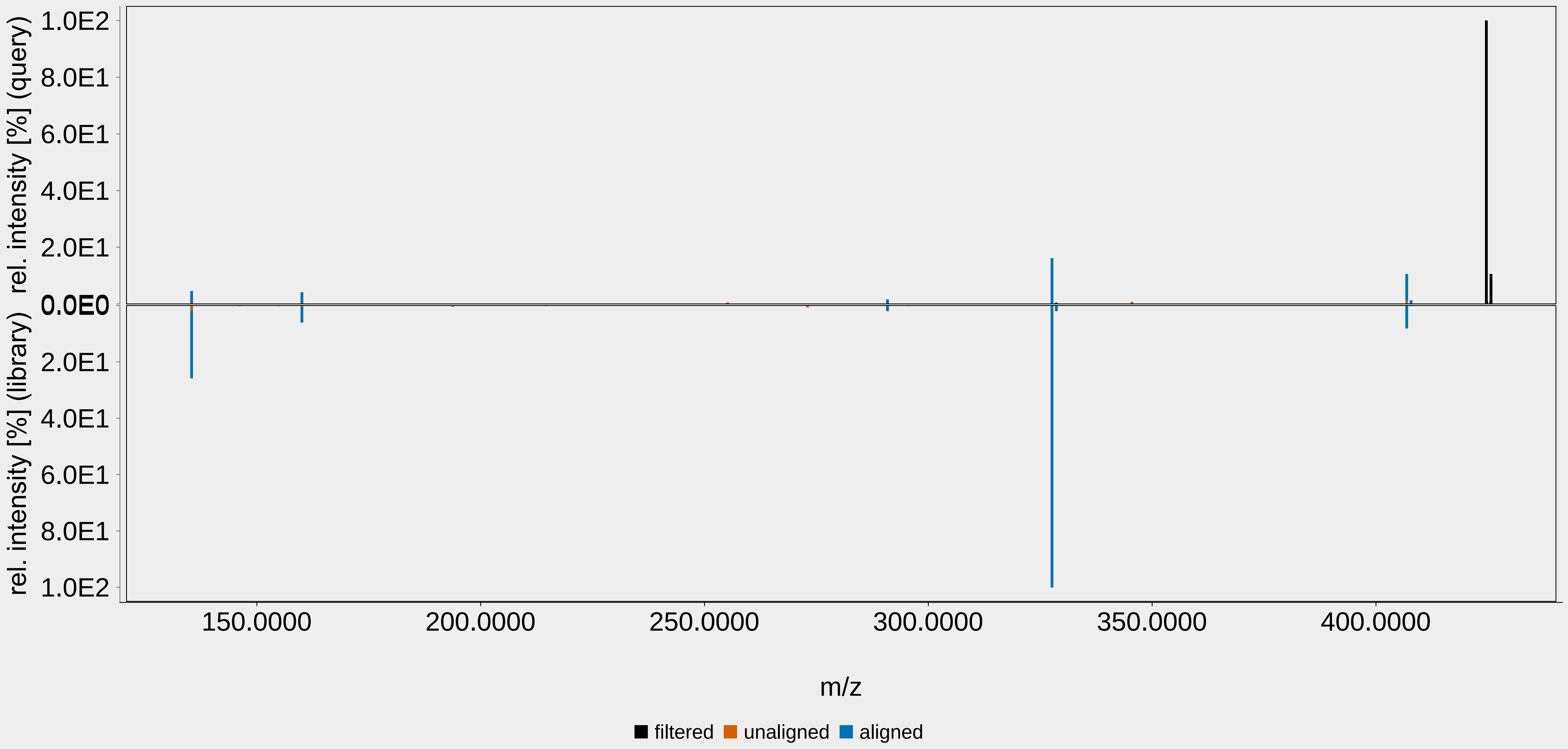

Compound information

Name: Adenosine diphosphate - 30.0 eV

Exact mass: 0.0

Ion type: M-H

Precursor m/z: 426.022

Charge: 1

Polarity: Negative

SMILES:

Instrument information

Instrument type: Orbitrap

Ion source: ESI

Ms level: 2

Acquisition: Isolated

Database links

CAS:

Other information

Principal investigator: PI

Data collector: Data Collector

Entry ID: CCMSLIB00006124443

Adenosine diphosphate - 30.0 eV

0.876

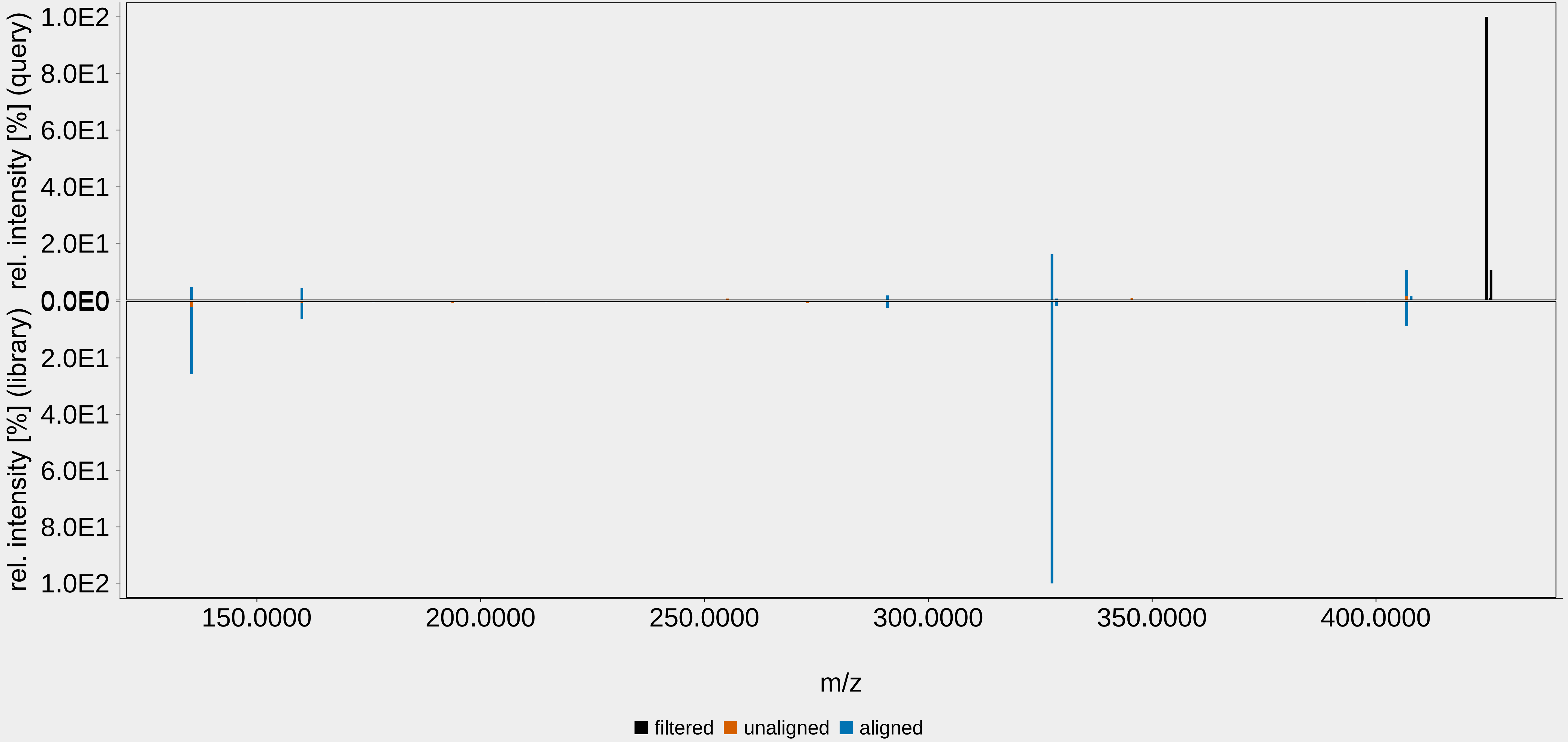

Compound information

Name: Adenosine diphosphate - 30.0 eV

Exact mass: 0.0

Ion type: M-H

Precursor m/z: 426.022

Charge: 1

Polarity: Negative

SMILES:

Instrument information

Instrument type: Orbitrap

Ion source: ESI

Ms level: 2

Acquisition: Isolated

Database links

CAS:

Other information

Principal investigator: PI

Data collector: Data Collector

Entry ID: CCMSLIB00006124450

PG(16:0/18:1); [M-H]- C40H76O10P1 **0.859**

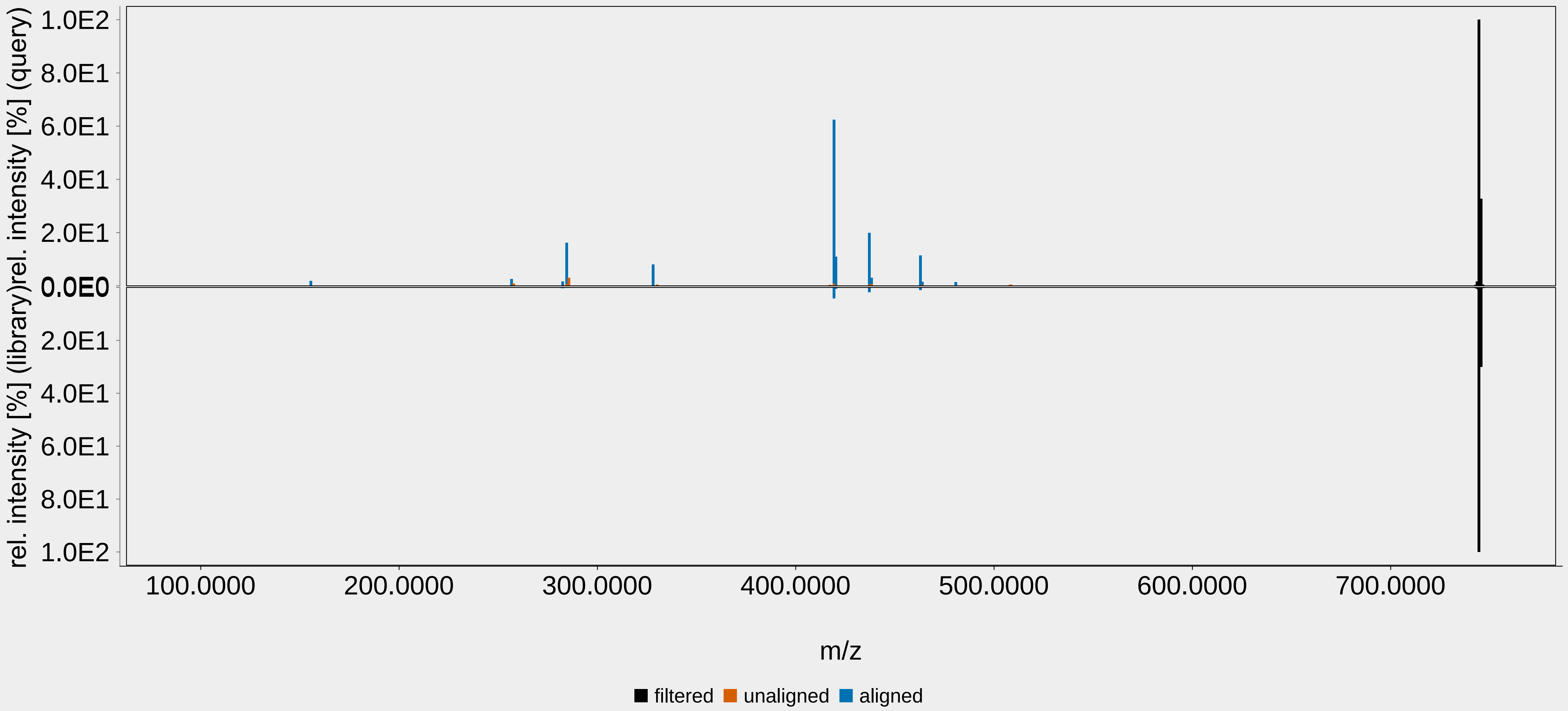

Compound information

Name: PG(16:0/18:1); [M-H]- C40H76O10P1

Exact mass: 0.0

Ion type: M-H

Precursor m/z: 747.518

Charge: 1

Polarity: Negative

Instrument information

Instrument type: HCD; Velos

Ion source: LC-ESI

Ms level: 2

Acquisition: Commercial

Database links

Other information

Principal investigator: Thomas Metz

Data collector: Thomas Metz

Entry ID: CCMSLIB00010063005

PG(16:0/18:1); [M-H]- C40H76O10P1 **0.864**

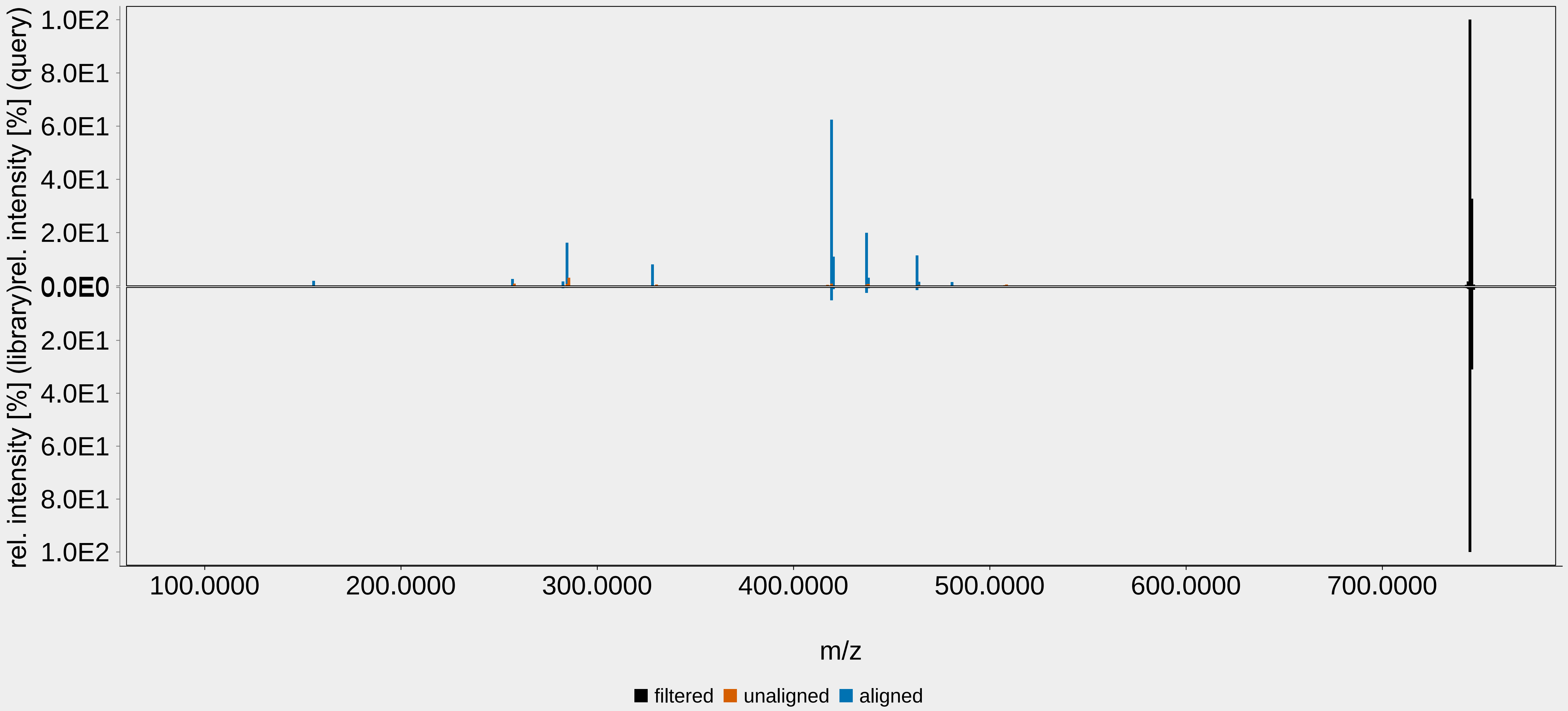

Compound information

Name: PG(16:0/18:1); [M-H]- C40H76O10P1

Exact mass: 0.0

Ion type: M-H

Precursor m/z: 747.518

Charge: 1

Polarity: Negative

Instrument information

Instrument type: HCD; Velos

Ion source: LC-ESI

Ms level: 2

Acquisition: Commercial

Database links

Other information

Principal investigator: Thomas Metz

Data collector: Thomas Metz

Entry ID: CCMSLIB00010063728

C11-LAS (STANDARD MIX), C11-linear alkylbenzyl sulfonate, 4-(undecan-5-yl)benzenesulfonic acid

0.874

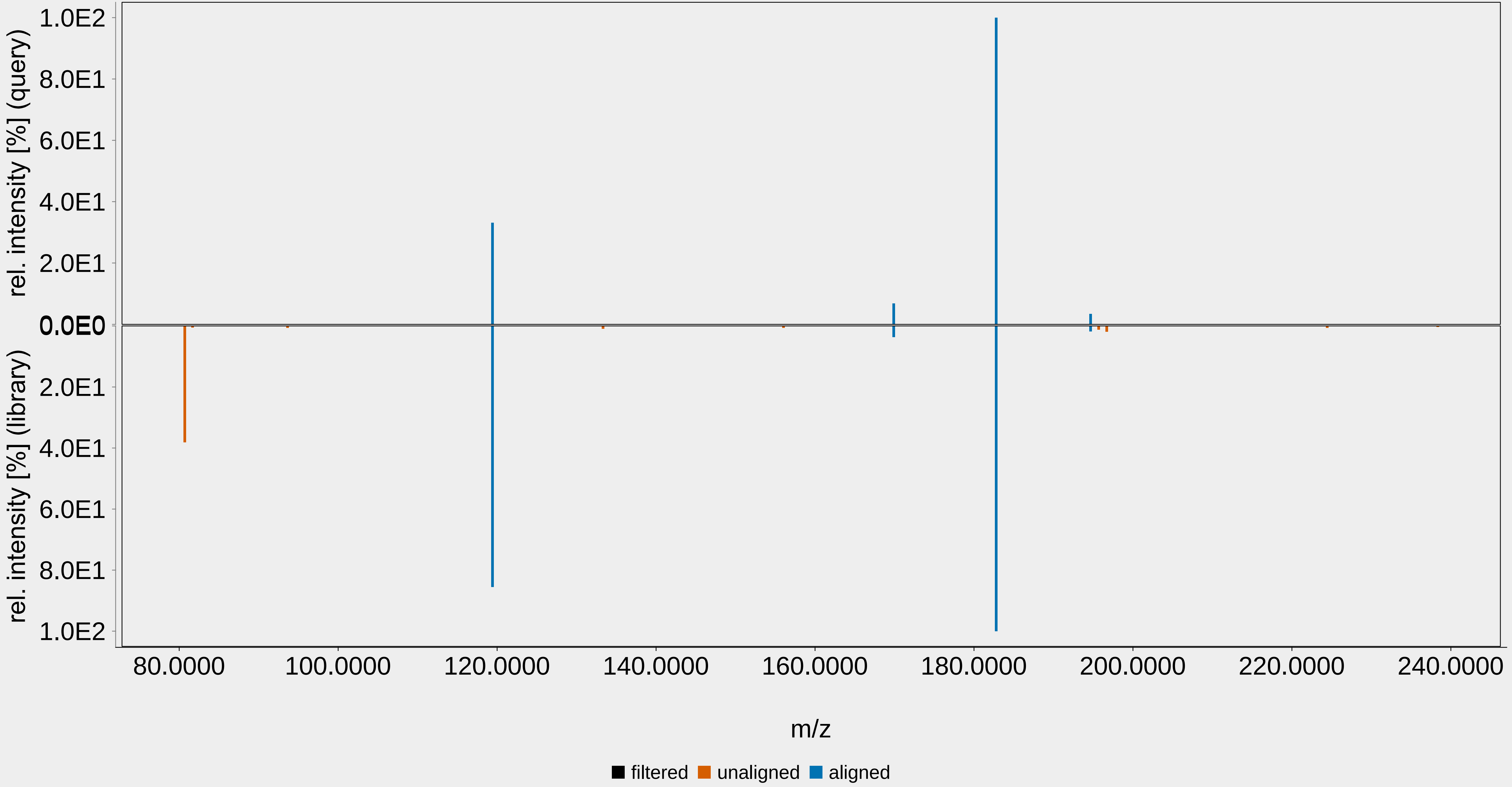

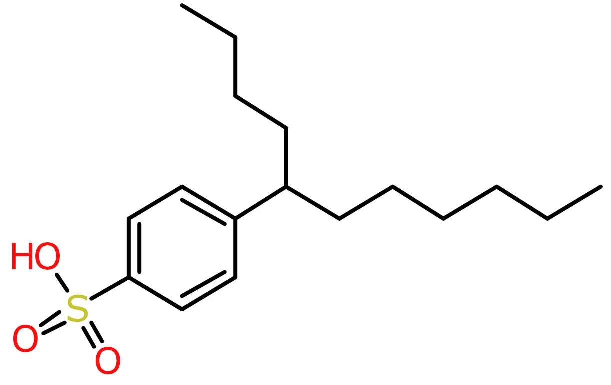

Compound information

Name: C11-LAS (STANDARD MIX), C11-linear

Formula: C17H28O3S

Mol. weight: 312.1759

Exact mass: 312.1759

Ion type: [M-H]-

Precursor m/z: 311.1686

Polarity: negative

InChI:

InChI key:

SMILES:

Instrument information

Instrument type: LC-ESI-QFT

Instrument: Q Exactive Orbitrap Thermo

Ion source: ESI

Ms level: MS2

Collision energy: 90 NCE

Database links

Pubchem: 15593874

MoNA ID: ETS00014

Other information

Principal investigator: E. Schymanski, Dept. of

Data collector: E. Schymanski, Dept. of

C11-LAS (STANDARD MIX) 0.874

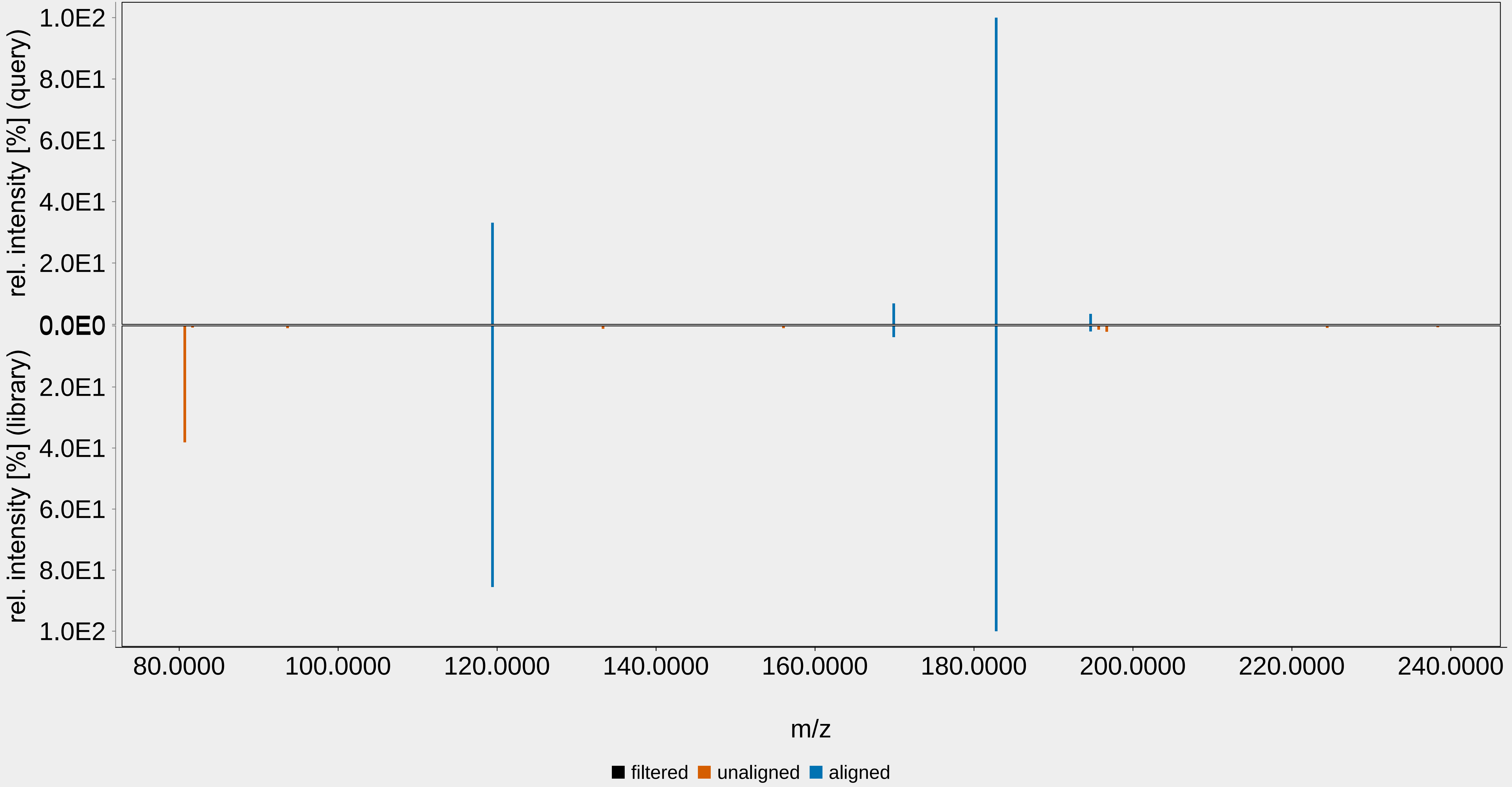

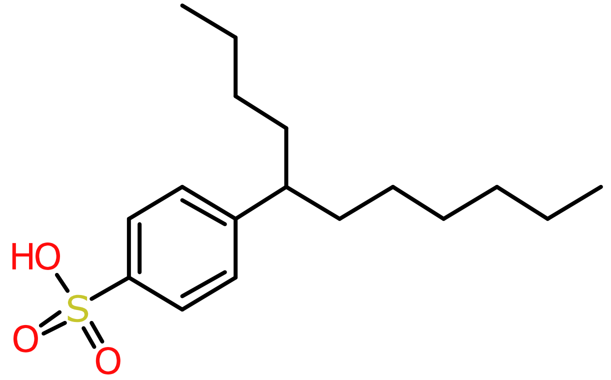

Compound information

Name: C11-LAS (STANDARD MIX)

Formula: C17H28O3S

Exact mass: 312.1759

Ion type: [M-H]-

Precursor m/z: 311.1686

Polarity: NEGATIVE

InChI:

InChI key:

SMILES:

Num peaks: 13

Instrument information

Instrument type: LC-ESI-QFT

Instrument: Q Exactive Orbitrap Thermo

Ms level: MS2

Collision energy: 90 NCE

Database links

Other information

Entry ID:

Comment: Parent=311.1686

PA 18:0_20:4

0.875

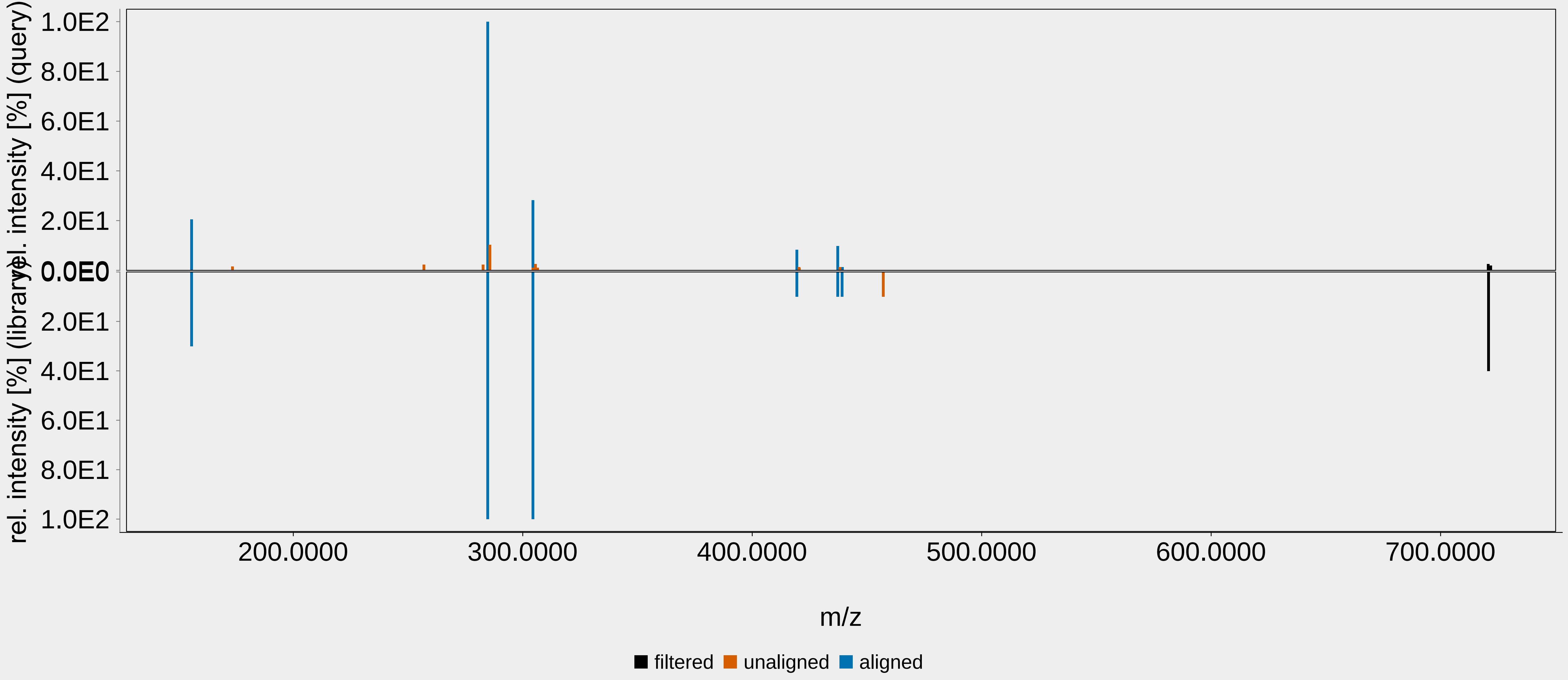

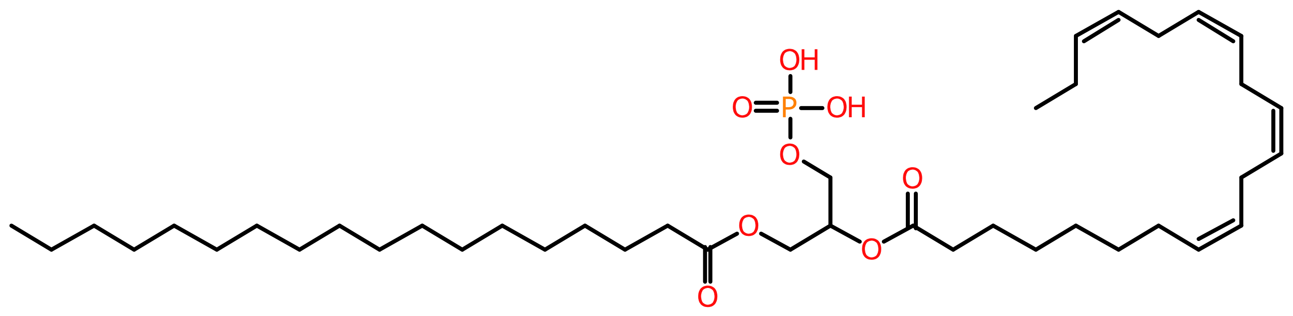

Compound information

Name: PA 18:0_20:4

Formula: C41H73O8P

Ion type: [M-H]-

Precursor m/z: 723.49703

Ccs: 262.1

InChI key:

SMILES:

Num peaks: 8

Instrument information

Database links

Other information

PA(18:0/20:4); [M-H]- C41H72O8P1

0.880

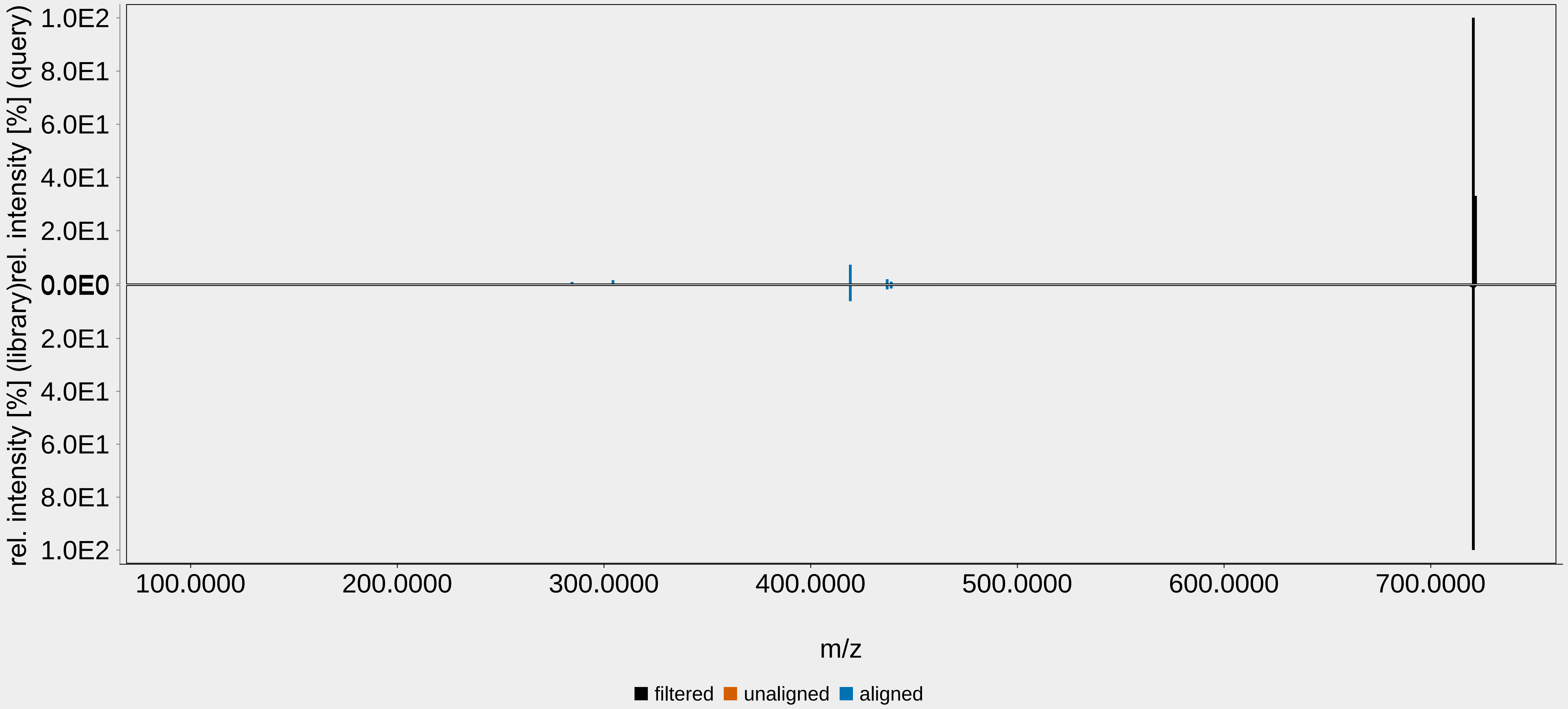

Compound information

Name: PA(18:0/20:4); [M-H]- C41H72O8P1

Exact mass: 0.0

Ion type: M-H

Precursor m/z: 723.496

Charge: 1

Polarity: Negative

Instrument information

Instrument type: HCD; Velos

Ion source: LC-ESI

Ms level: 2

Acquisition: Commercial

Database links

Other information

Principal investigator: Thomas Metz

Data collector: Thomas Metz

Entry ID: CCMSLIB00010065411

[1-hexadecanoyloxy-3-phosphonooxypropan-2-yl]octadec-9-enoate

0.908

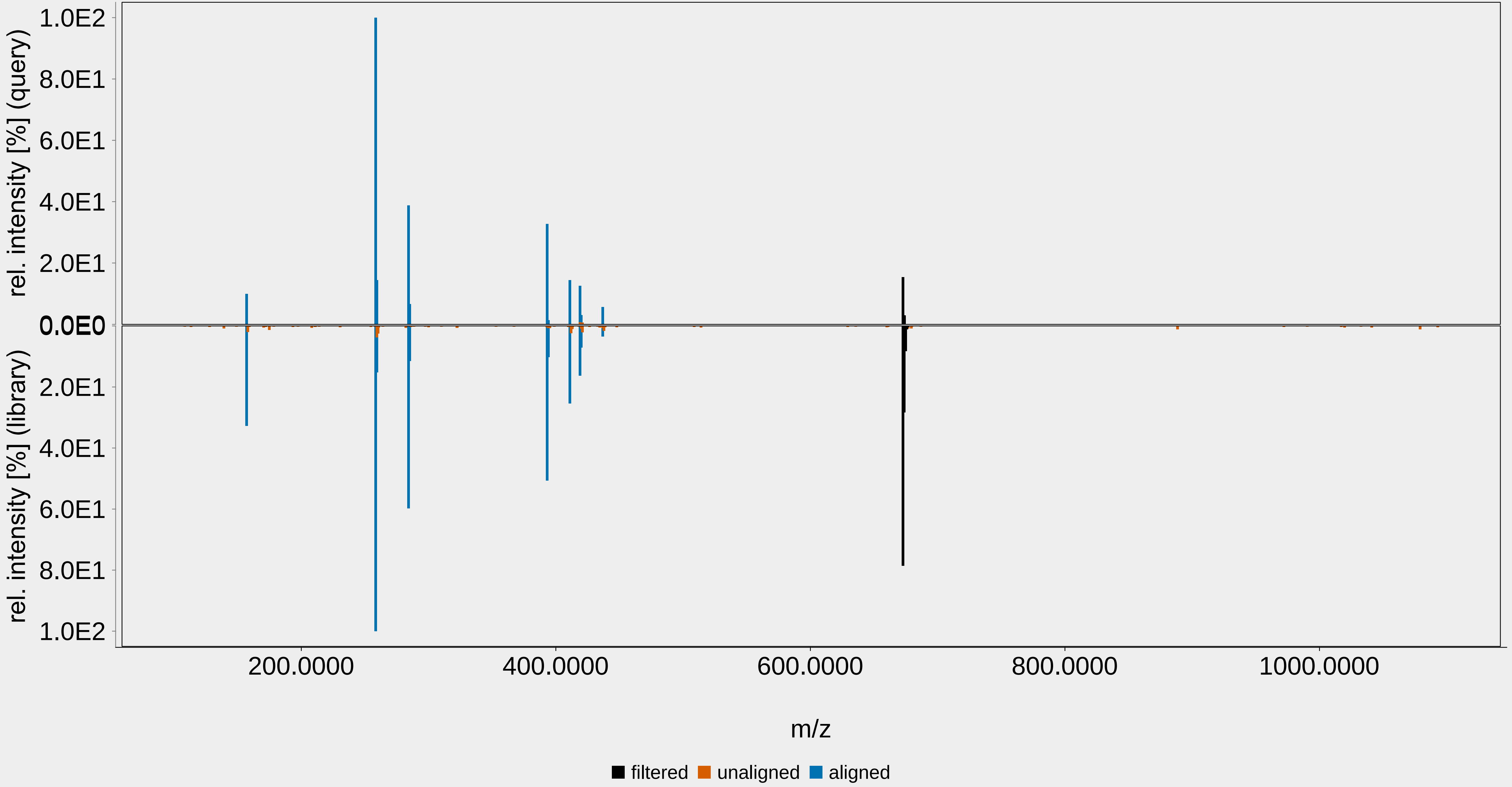

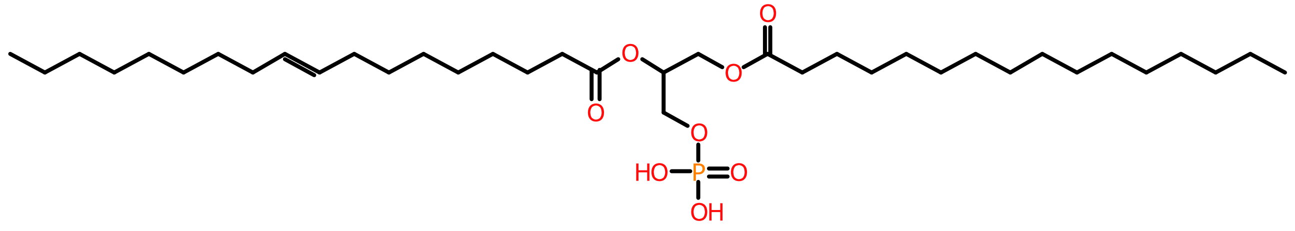

Compound information

Name:

Formula: C37H71O8P

Mol. weight: 674.489

Exact mass: 674.489

Ion type: [M-H]-

Precursor m/z: 673.481

Polarity: negative

InChI:

InChI key:

SMILES:

Instrument information

Instrument: qTof

Ms level: MS2

Database links

Other information

Principal investigator: O Laprevote, UMR 8038

Data collector: O Laprevote, UMR 8038

PA 16:0_18:1

0.935

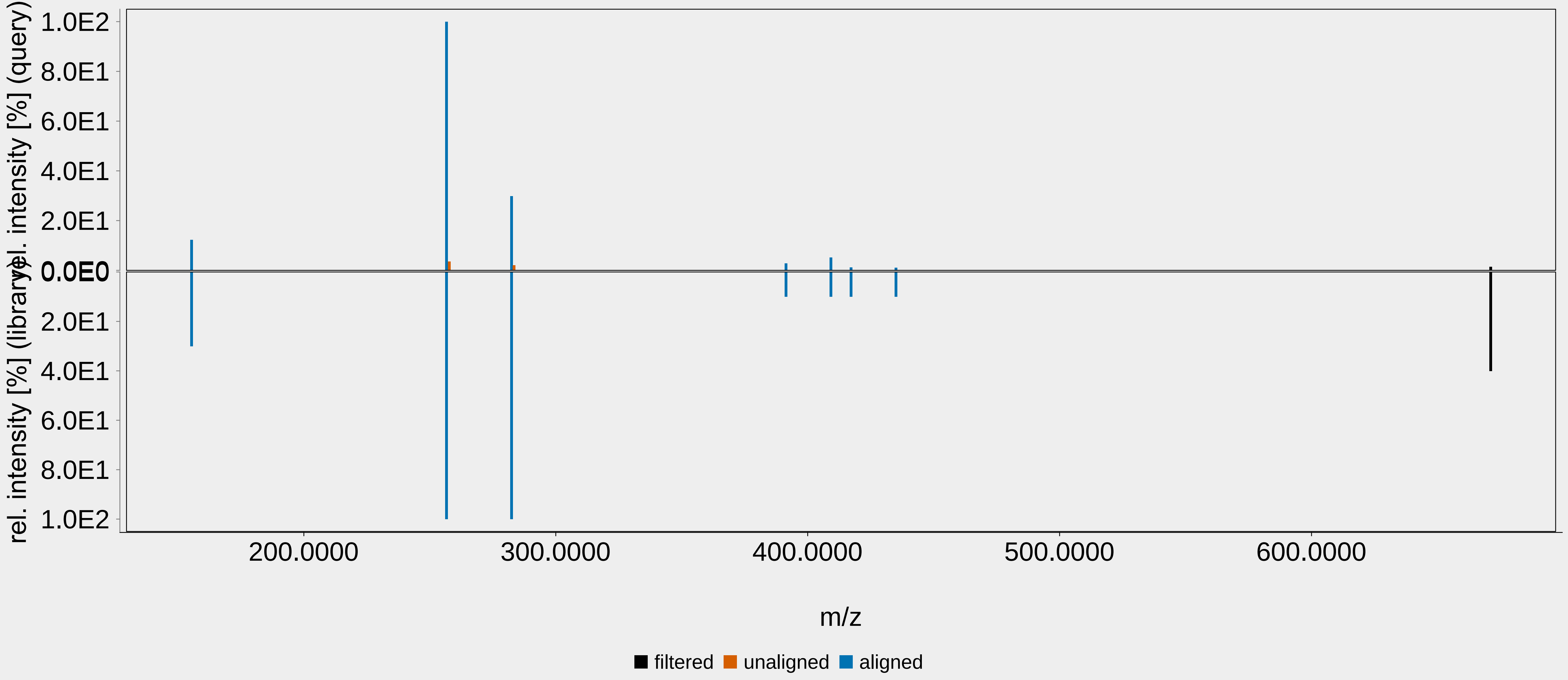

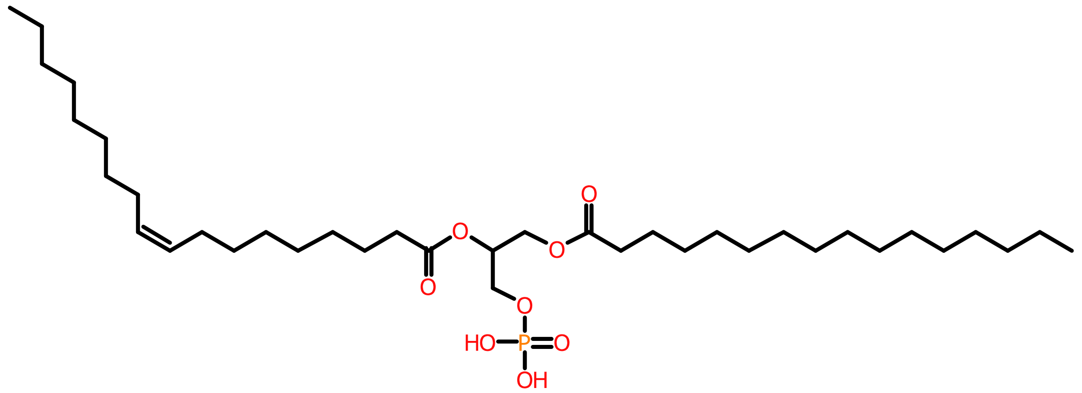

Compound information

Name: PA 16:0_18:1

Formula: C37H71O8P

Ion type: [M-H]-

Precursor m/z: 673.48138

Ccs: 252.18

InChI key:

SMILES:

Num peaks: 8

Instrument information

Database links

Other information

PA 22:4_22:6 **0.872**

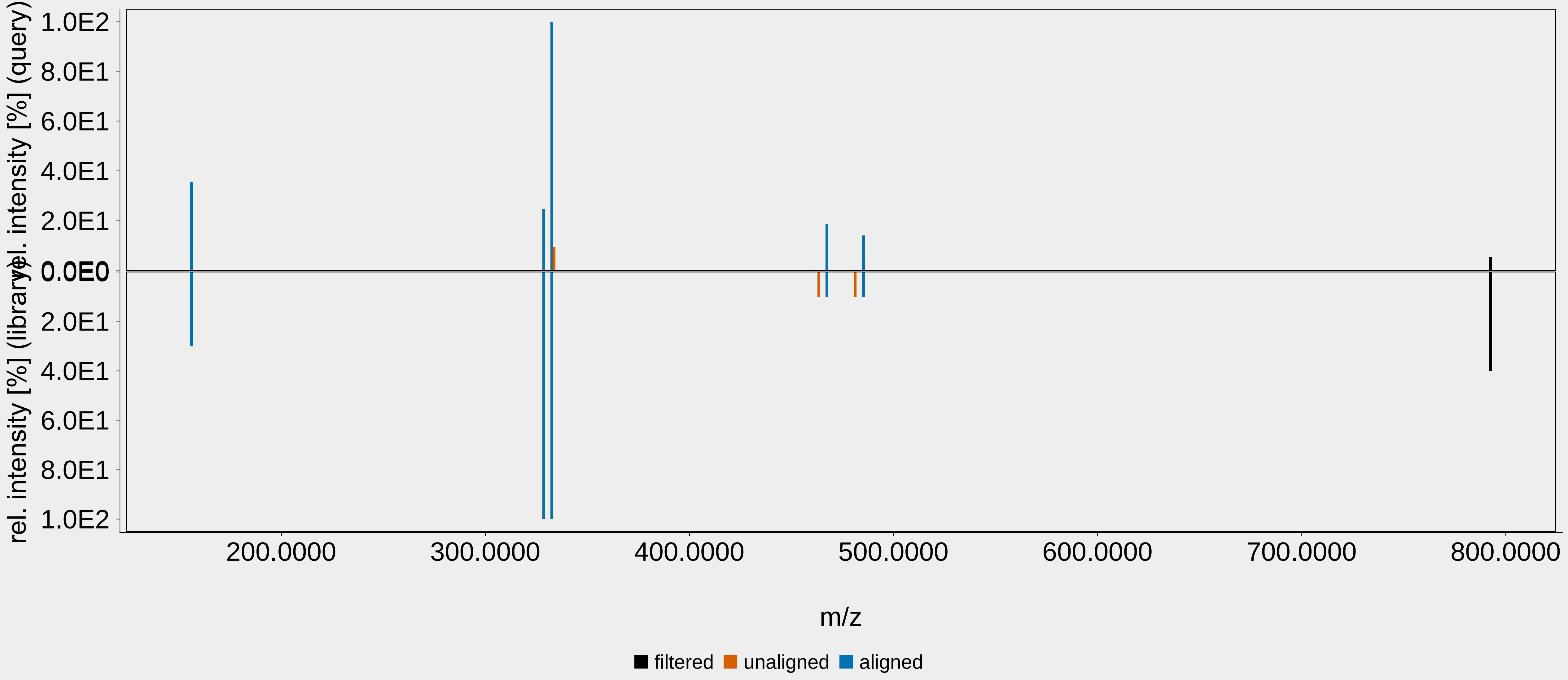

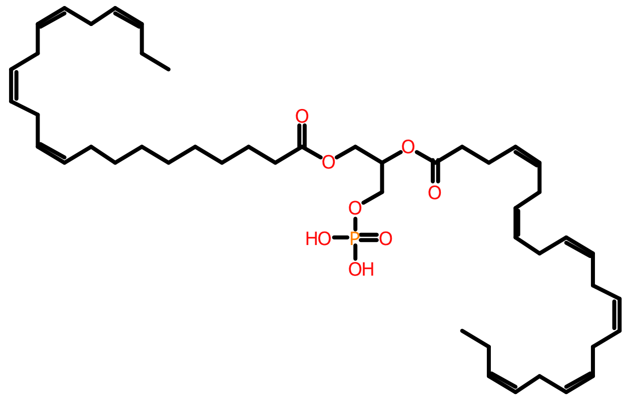

Compound information

Name: PA 22:4_22:6

Formula: C47H73O8P

Ion type: [M-H]-

Precursor m/z: 795.49703

Ccs: 272.68

InChI key:

SMILES:

Num peaks: 8

Instrument information

Database links

Other information

Spectral Match to 1-Oleoyl-L-.alpha.-lysophosphatidic acid from NIST14

0.830

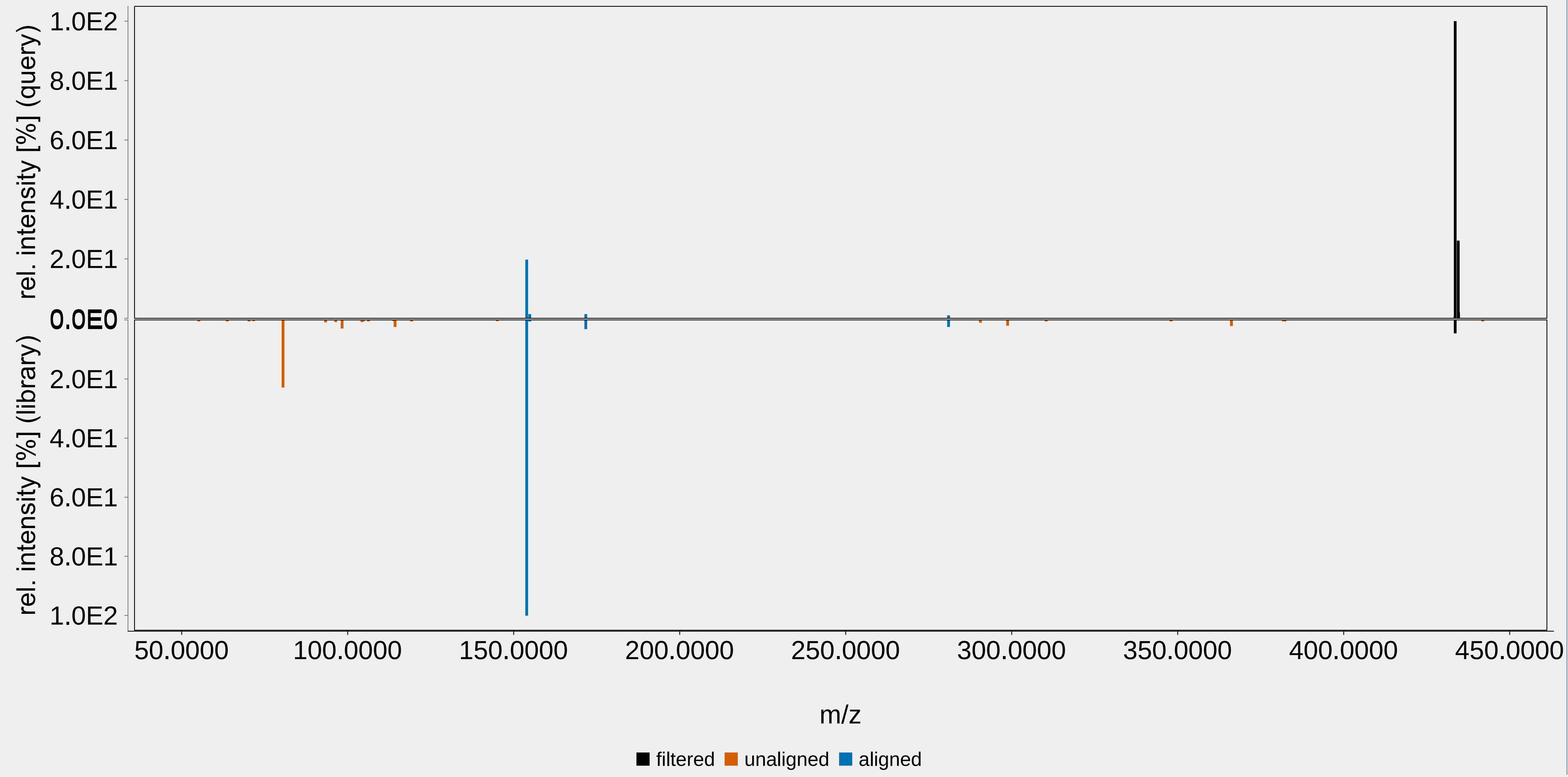

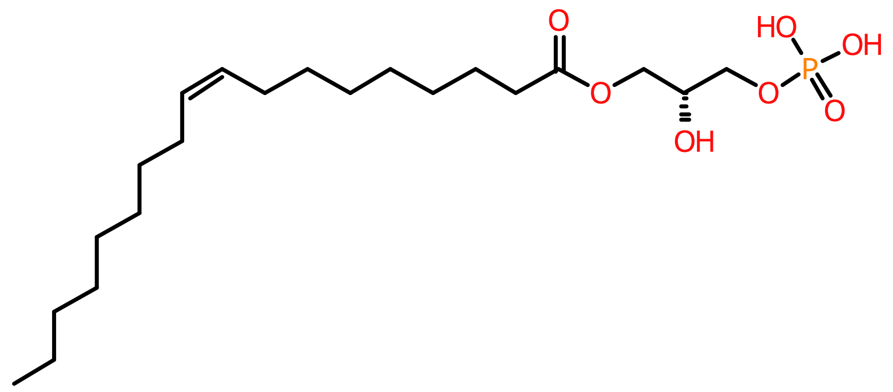

Compound information

Name: Spectral Match to

Exact mass: 436.259

Ion type: M-H

Precursor m/z: 435.252

Charge: 1

Polarity: Negative

InChI:

SMILES:

Instrument information

Instrument type: qTof

Ion source: ESI

Ms level: 2

Acquisition: Isolated

Database links

CAS: 22002875

Other information

Principal investigator: Data from Kevin Bush

Data collector: Data deposited by fevargas

Entry ID: CCMSLIB00003137366

PS 18:1_20:1

0.733

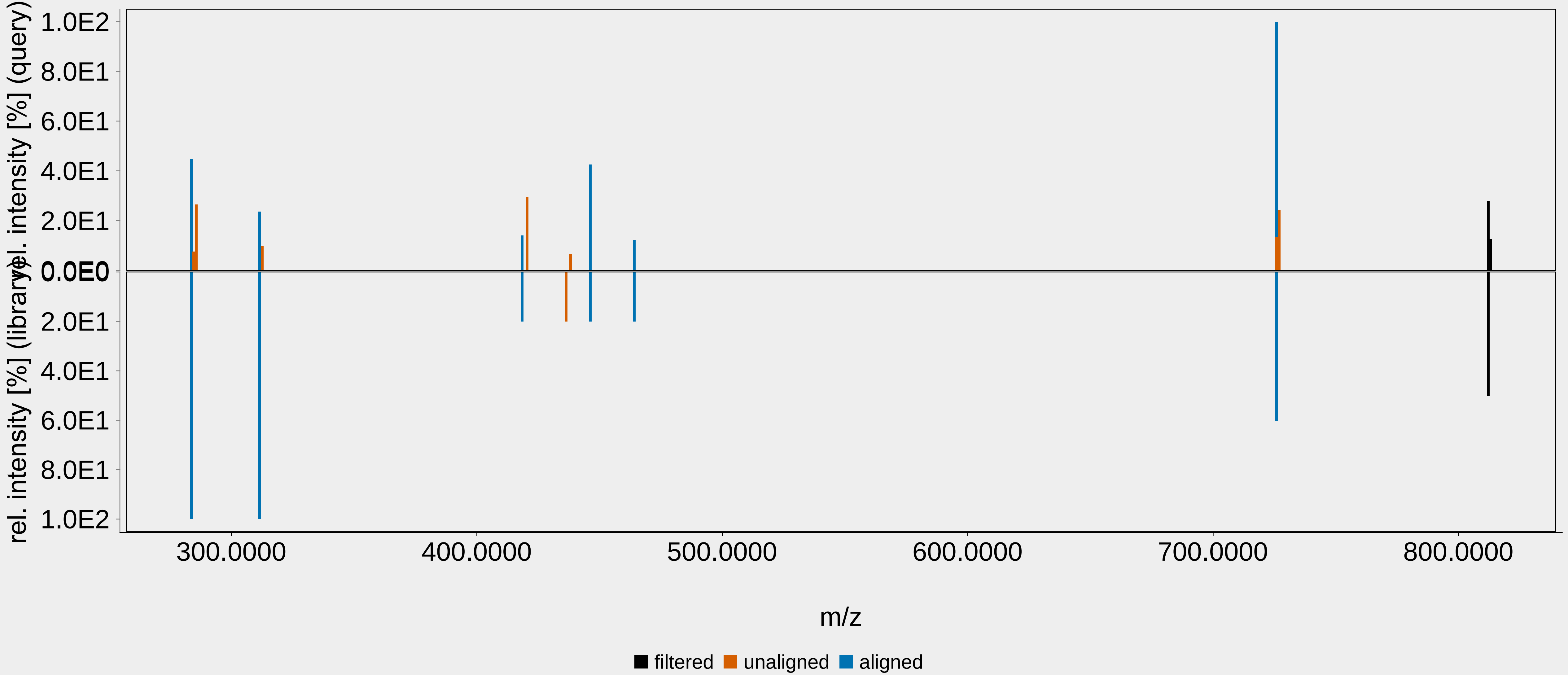

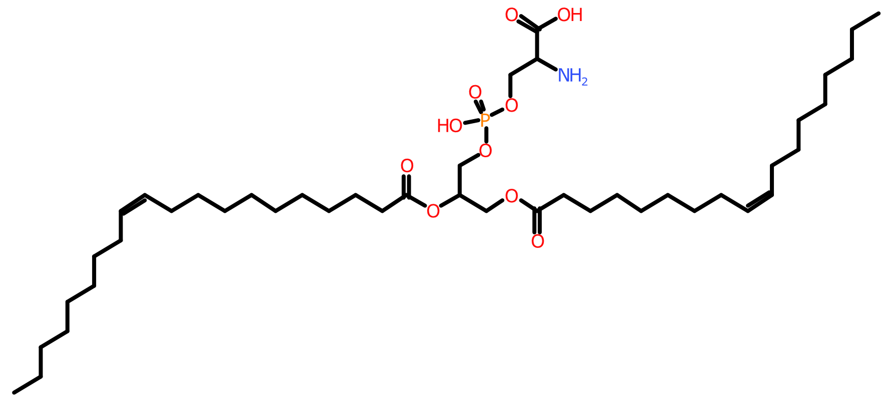

Compound information

Name: PS 18:1_20:1

Formula: C44H82NO10P

Ion type: [M-H]-

Precursor m/z: 814.56036

Ccs: 282.14

InChI key:

SMILES:

Num peaks: 8

Instrument information

Database links

Other information

PS 18:0_20:1

0.783

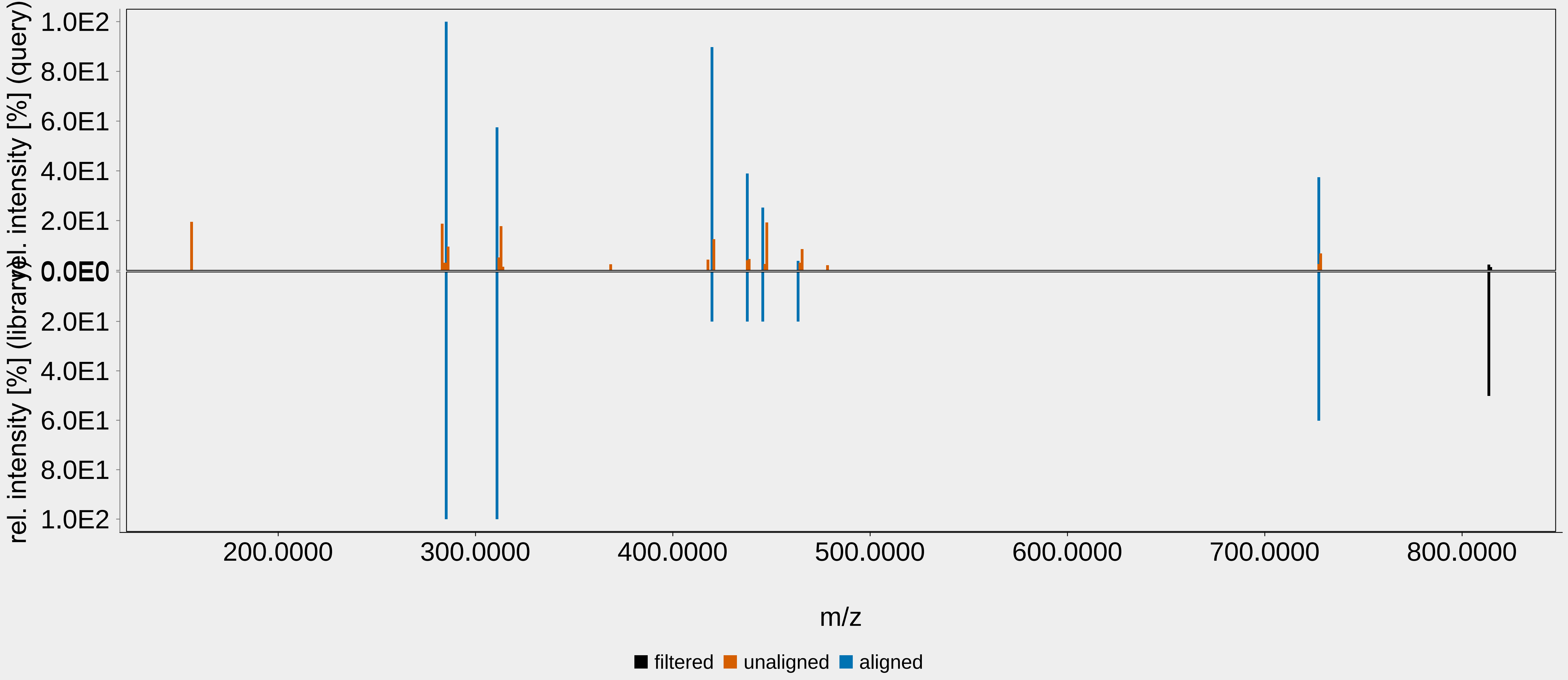

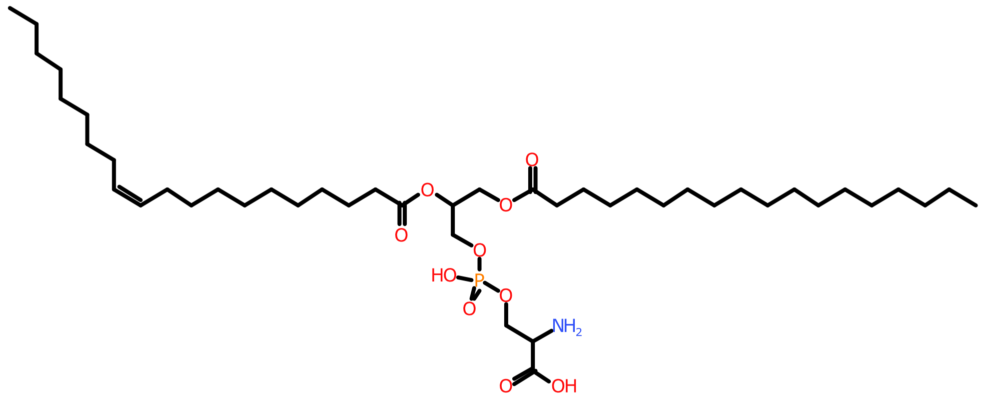

Compound information

Name: PS 18:0_20:1

Formula: C44H84NO10P

Ion type: [M-H]-

Precursor m/z: 816.57601

Ccs: 284.42

InChI key: KVMIIDXBXADXIM-ZPHPHTNENA-I

SMILES:

Num peaks: 8

Instrument information

Database links

Other information

PS(22:6/0:0); [M-H]- C28H43N1O9P1 0.753

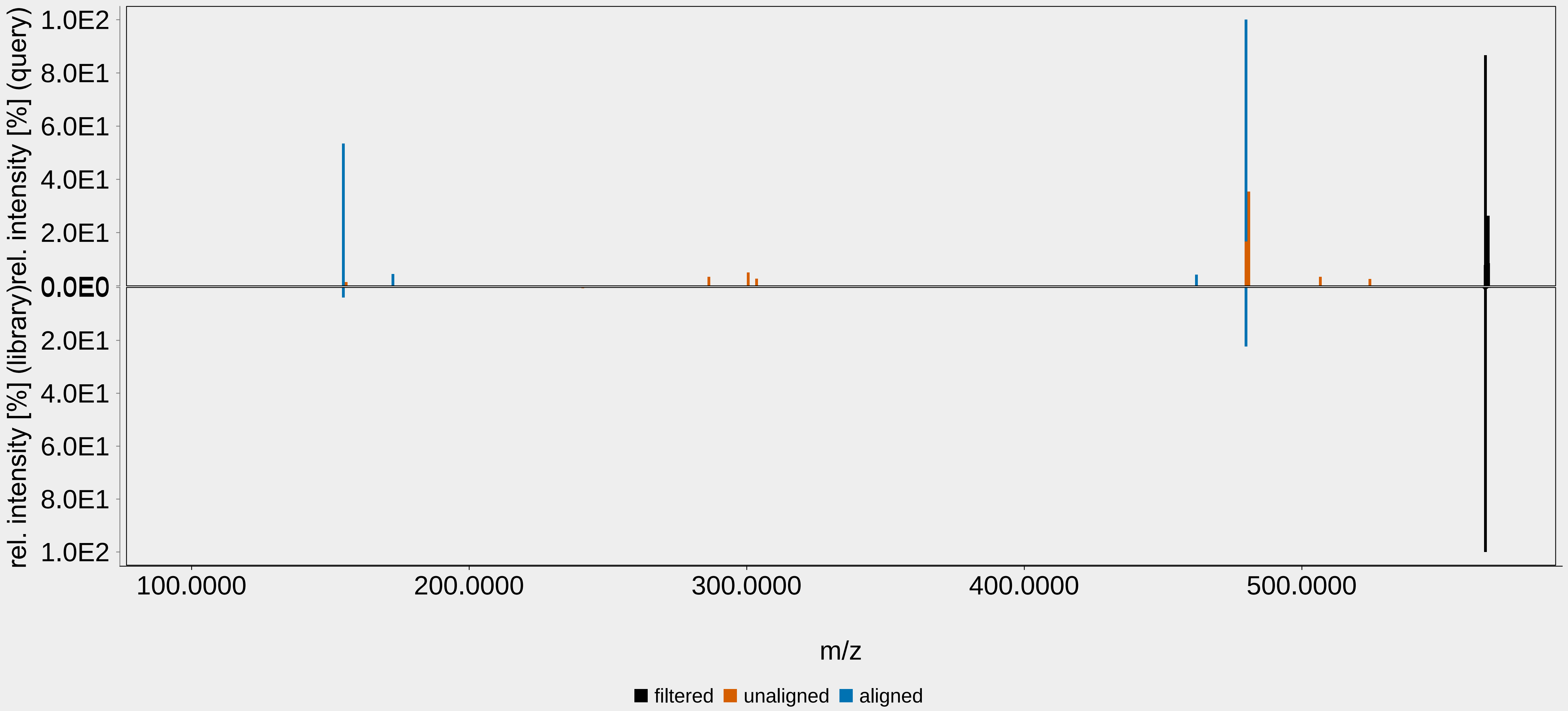

Compound information

Name: PS(22:6/0:0); [M-H]- C28H43N1O9P1

Exact mass: 0.0

Ion type: M-H

Precursor m/z: 568.268

Charge: 1

Polarity: Negative

Instrument information

Instrument type: HCD; Velos

Ion source: LC-ESI

Ms level: 2

Acquisition: Commercial

Database links

Other information

Principal investigator: Thomas Metz

Data collector: Thomas Metz

Entry ID: CCMSLIB00010087165

PE(22:4/22:6); [M-H]- C49H77N1O8P1 **0.908**

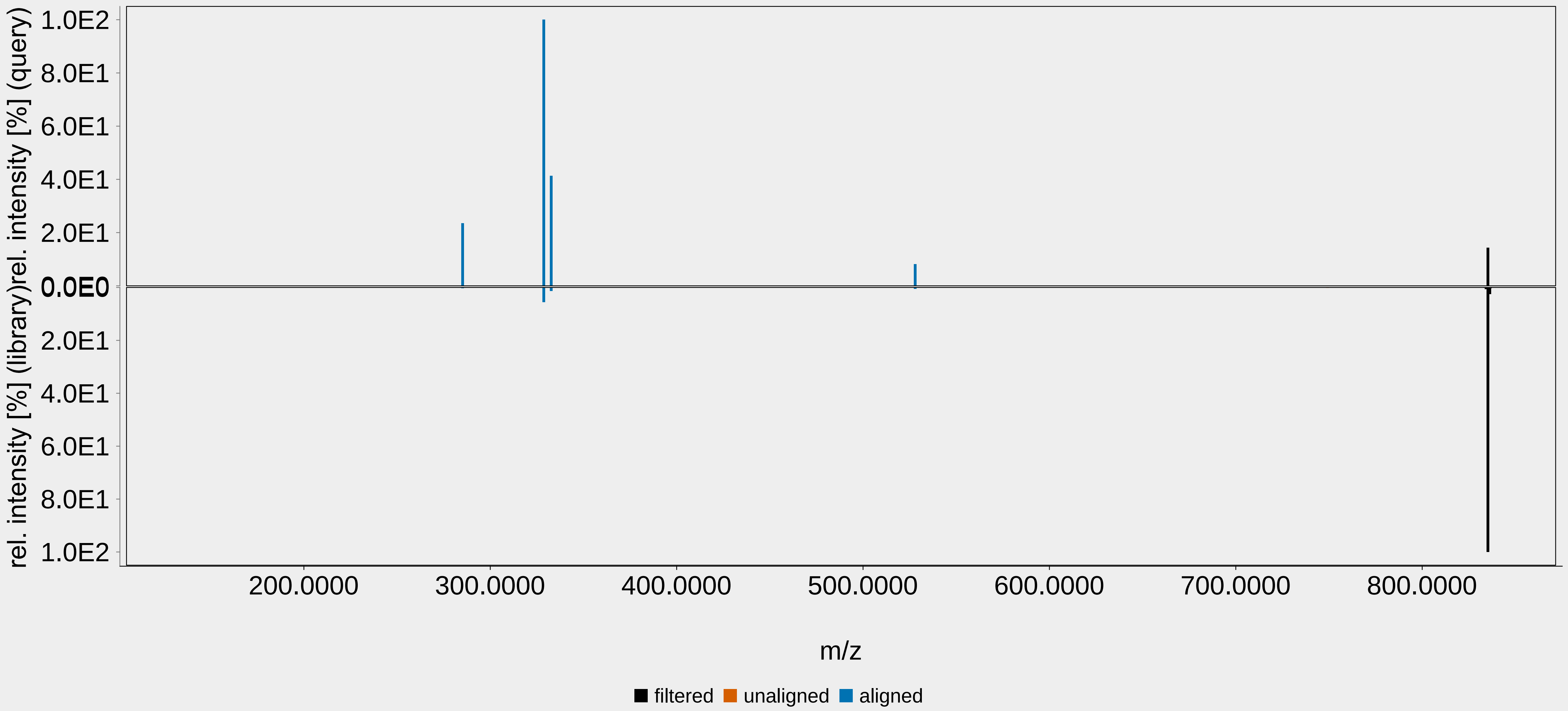

Compound information

Name: PE(22:4/22:6); [M-H]- C49H77N1O8P1

Exact mass: 0.0

Ion type: M-H

Precursor m/z: 838.539

Charge: 1

Polarity: Negative

Instrument information

Instrument type: HCD; Velos

Ion source: LC-ESI

Ms level: 2

Acquisition: Commercial

Database links

Other information

Principal investigator: Thomas Metz

Data collector: Thomas Metz

Entry ID: CCMSLIB00010063765

PE(22:4/22:6); [M-H]- C49H77N1O8P1 **0.900**

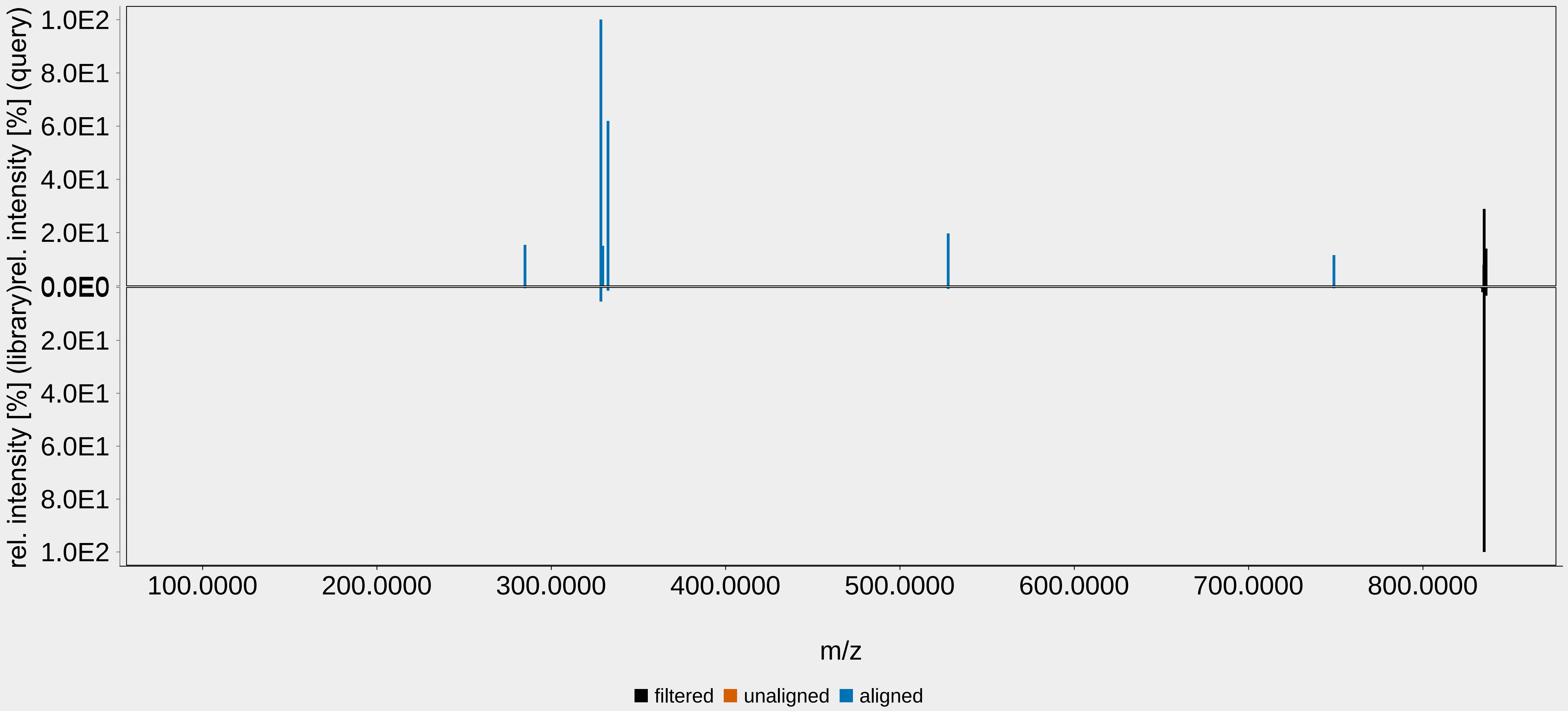

Compound information

Name: PE(22:4/22:6); [M-H]- C49H77N1O8P1

Exact mass: 0.0

Ion type: M-H

Precursor m/z: 838.539

Charge: 1

Polarity: Negative

Instrument information

Instrument type: HCD; Velos

Ion source: LC-ESI

Ms level: 2

Acquisition: Commercial

Database links

Other information

Principal investigator: Thomas Metz

Data collector: Thomas Metz

Entry ID: CCMSLIB00010086991

PI 36:2 0.716

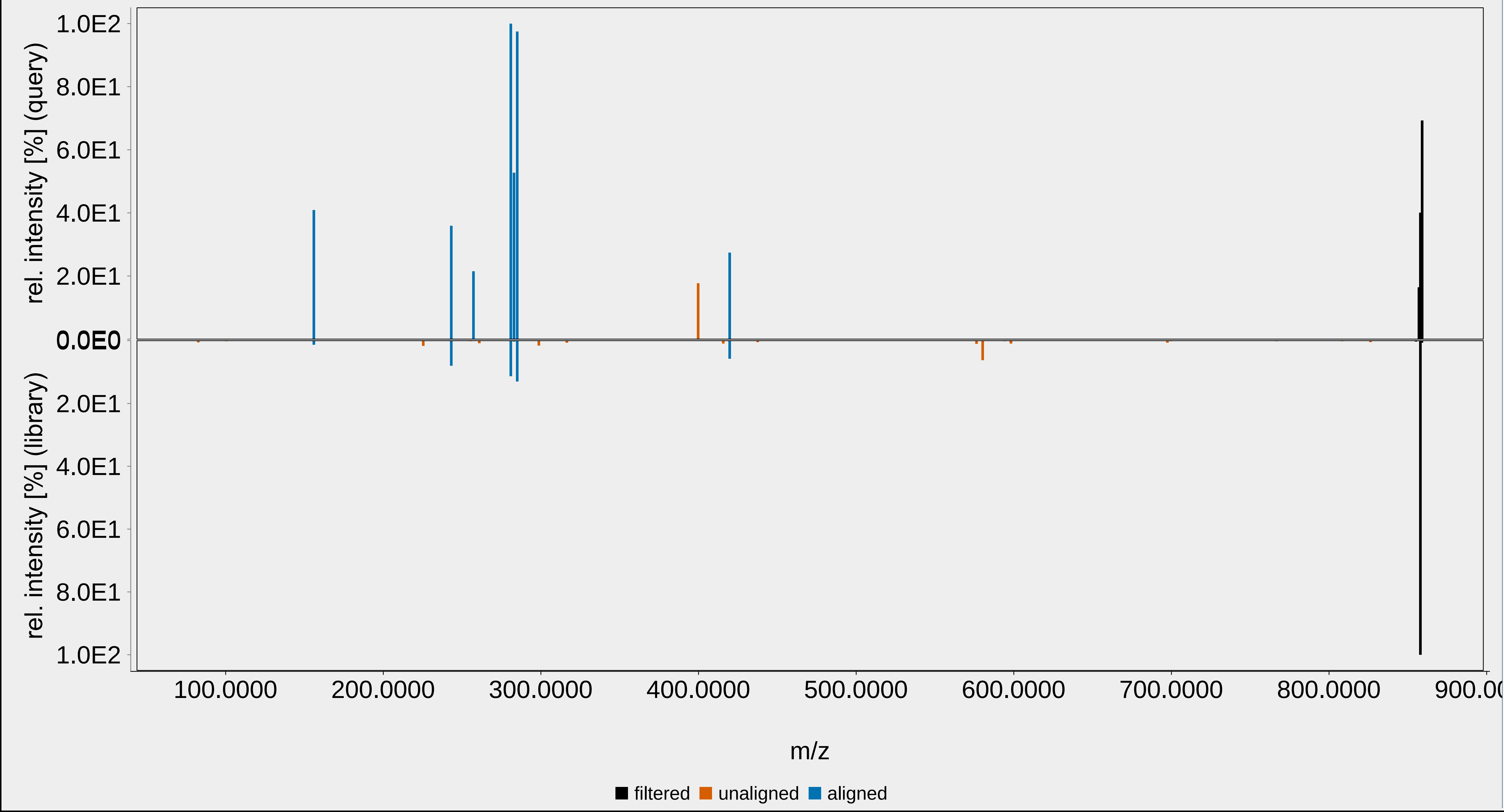

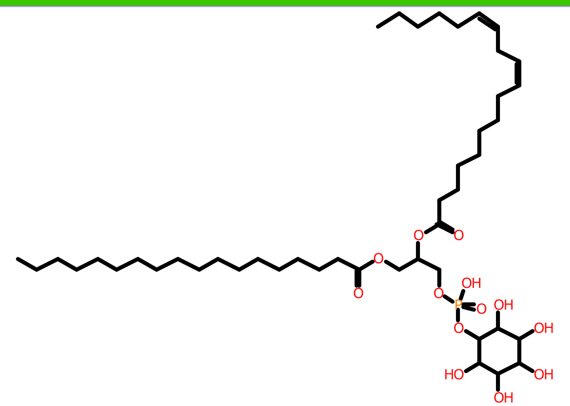

Compound information

Name: PI 36:2

Formula: C45H83O13P

Mol. weight: 863.122

Exact mass: 863.122

Ion type: [M-H]-

Precursor m/z: 861.5504

RT: 8.773114

Polarity: negative

InChI:

InChI key:

SMILES:

Instrument information

Instrument type: LC-ESI-QTOF

Instrument: AB Sciex TripleTOF 5600+ system

Ms level: MS2

Collision energy: 45V

Database links

Pubchem: 138199526

MoNA ID: LQB00692

Other information

Principal investigator: Kazutaka Ikeda, Hiroshi

Data collector: Kazutaka Ikeda, Hiroshi

PI 36:2

0.716

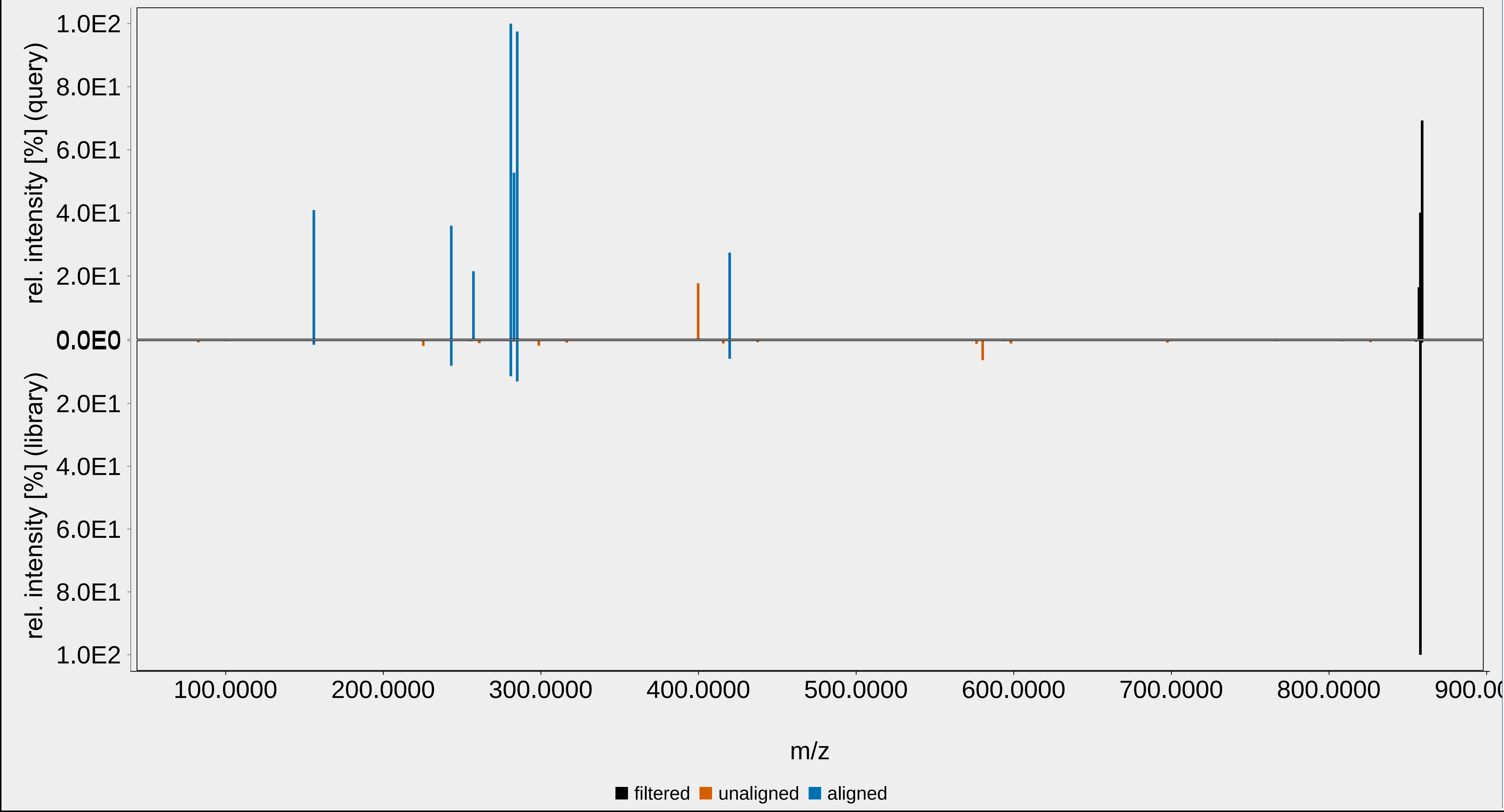

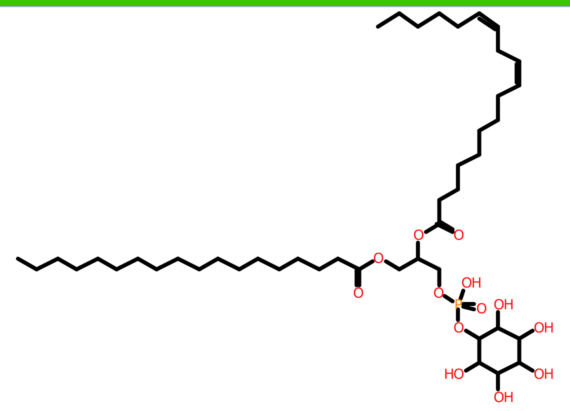

Compound information

Name: PI 36:2

Formula: C45H83O13P

Mol. weight: 863.122

Exact mass: 863.122

Ion type: [M-H]-

Precursor m/z: 861.5504

RT: 8.773114

Polarity: negative

InChI:

InChI key:

SMILES:

Instrument information

Instrument type: LC-ESI-QTOF

Instrument: AB Sciex TripleTOF 5600+ system

Ms level: MS2

Collision energy: 45V

Database links

Pubchem: 138199526

MoNA ID: LQB00306

Other information

Principal investigator: Kazutaka Ikeda, Hiroshi

Data collector: Kazutaka Ikeda, Hiroshi

PI 38:6

0.821

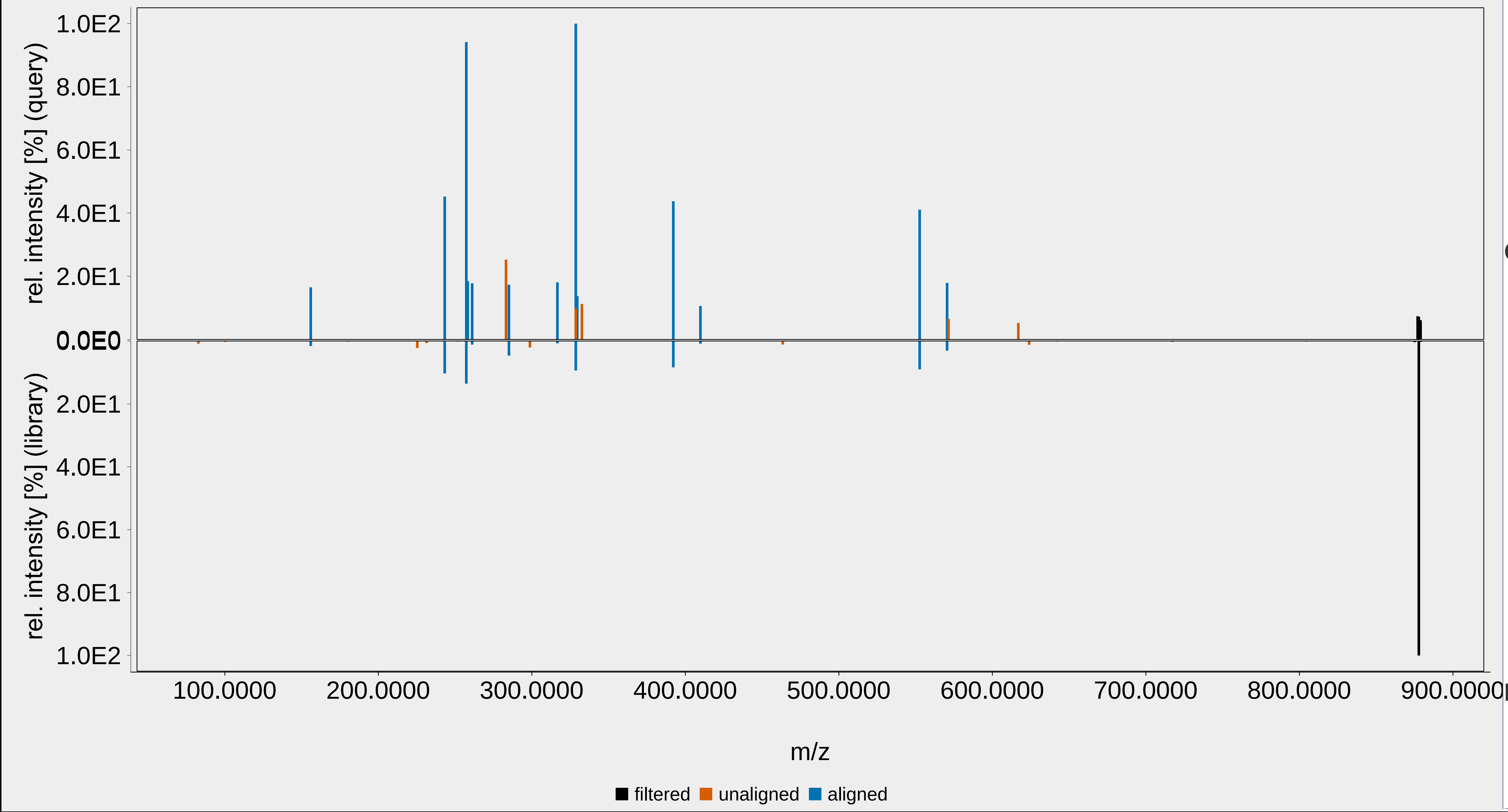

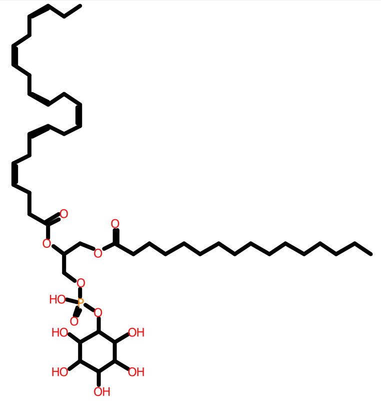

Compound information

Name: PI 38:6

Formula: C47H79O13P

Mol. weight: 883.1119999999999

Exact mass: 883.1119999999999

Ion type: [M-H]-

Precursor m/z: 881.514

RT: 8.246706

Polarity: negative

InChI:

InChI key:

SMILES:

Instrument information

Instrument type: LC-ESI-QTOF

Instrument: AB Sciex TripleTOF 5600+ system

Ms level: MS2

Collision energy: 45V

Database links

Pubchem: 138154339

MoNA ID: LQB00686

Other information

Principal investigator: Kazutaka Ikeda, Hiroshi

Data collector: Kazutaka Ikeda, Hiroshi

PI 38:6

0.821

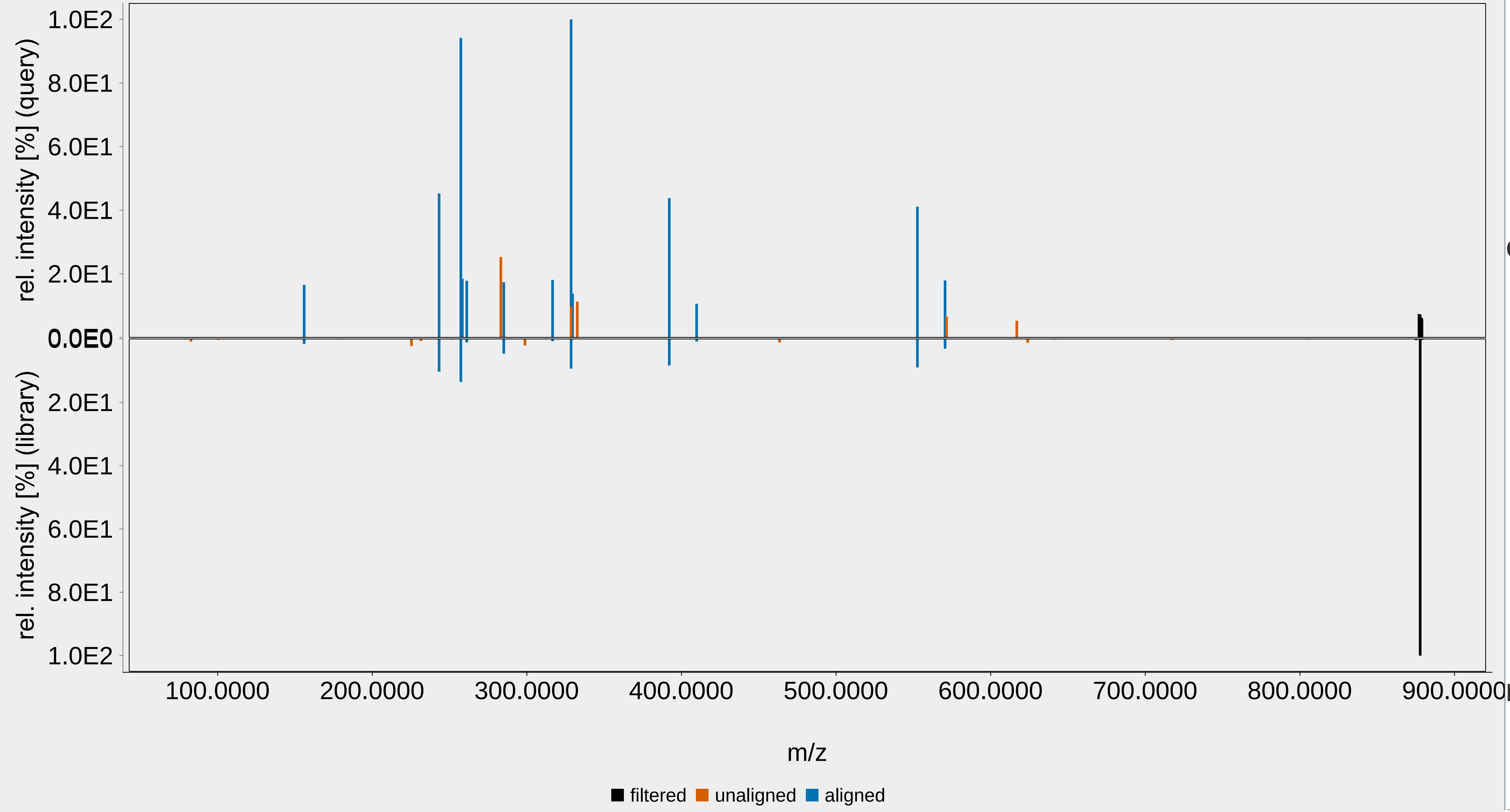

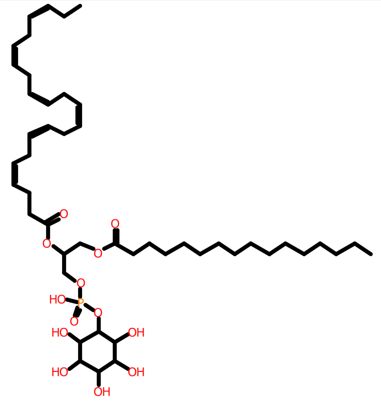

Compound information

Name: PI 38:6

Formula: C47H79O13P

Mol. weight: 883.1119999999999

Exact mass: 883.1119999999999

Ion type: [M-H]-

Precursor m/z: 881.514

RT: 8.246706

Polarity: negative

InChI:

InChI key:

SMILES:

Instrument information

Instrument type: LC-ESI-QTOF

Instrument: AB Sciex TripleTOF 5600+ system

Ms level: MS2

Collision energy: 45V

Database links

Pubchem: 138154339

MoNA ID: LQB00300

Other information

Principal investigator: Kazutaka Ikeda, Hiroshi

Data collector: Kazutaka Ikeda, Hiroshi

PI 36:4

0.864

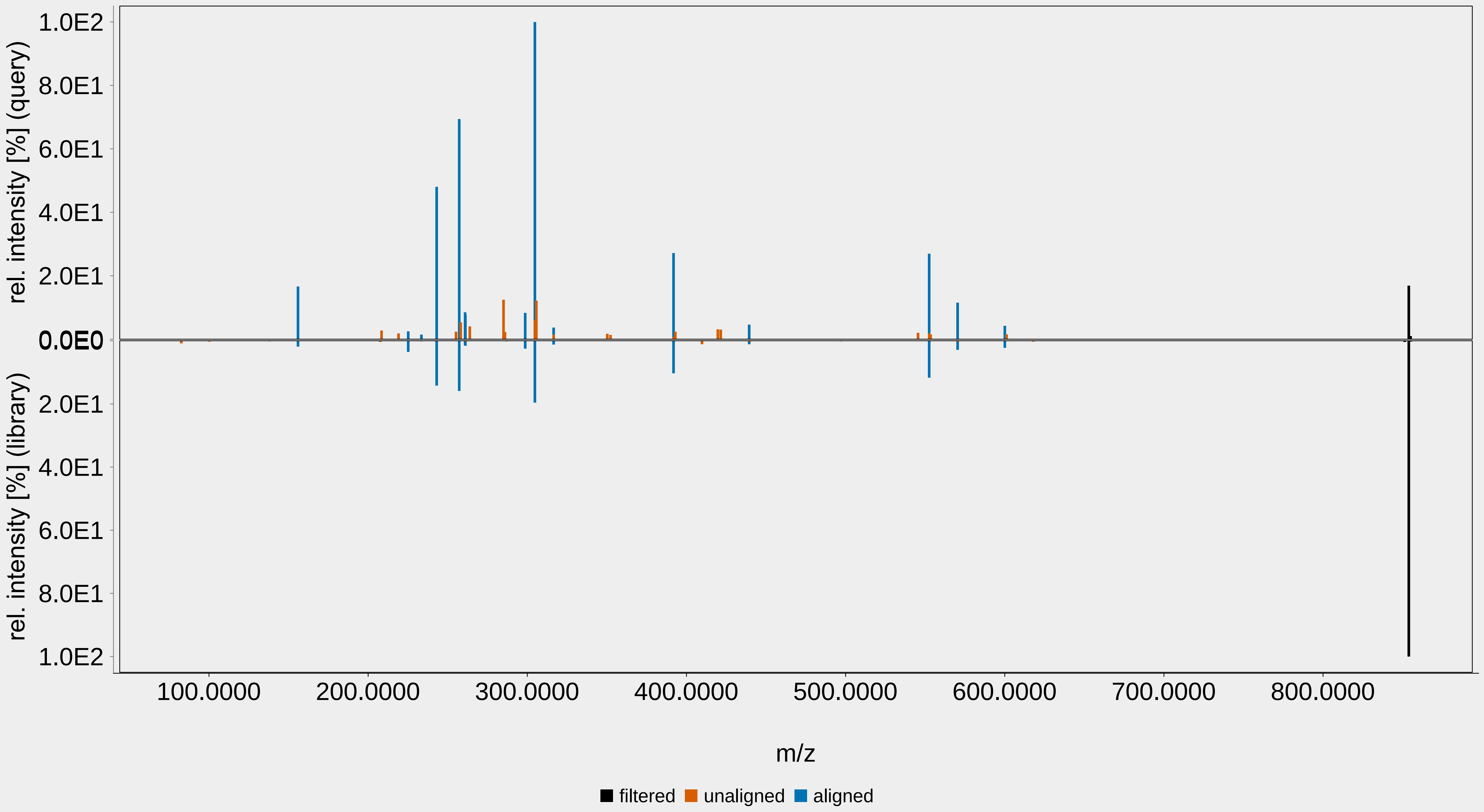

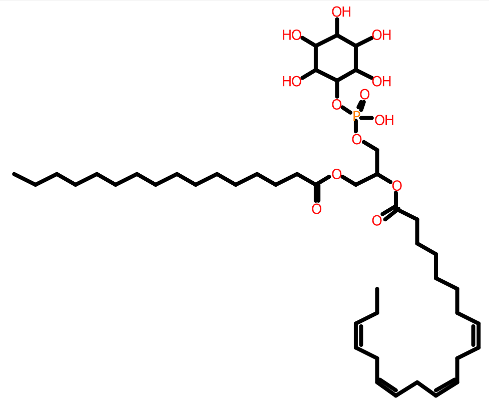

Compound information

Name: PI 36:4

Formula: C45H79O13P

Mol. weight: 859.0899999999999

Exact mass: 859.0899999999999

Ion type: [M-H]-

Precursor m/z: 857.5197

RT: 8.301669

Polarity: negative

InChI:

InChI key:

SMILES:

Instrument information

Instrument type: LC-ESI-QTOF

Instrument: AB Sciex TripleTOF 5600+ system

Ms level: MS2

Collision energy: 45V

Database links

Pubchem: 138284476

MoNA ID: LQB00683

Other information

Principal investigator: Kazutaka Ikeda, Hiroshi

Data collector: Kazutaka Ikeda, Hiroshi

PI 36:4

0.864

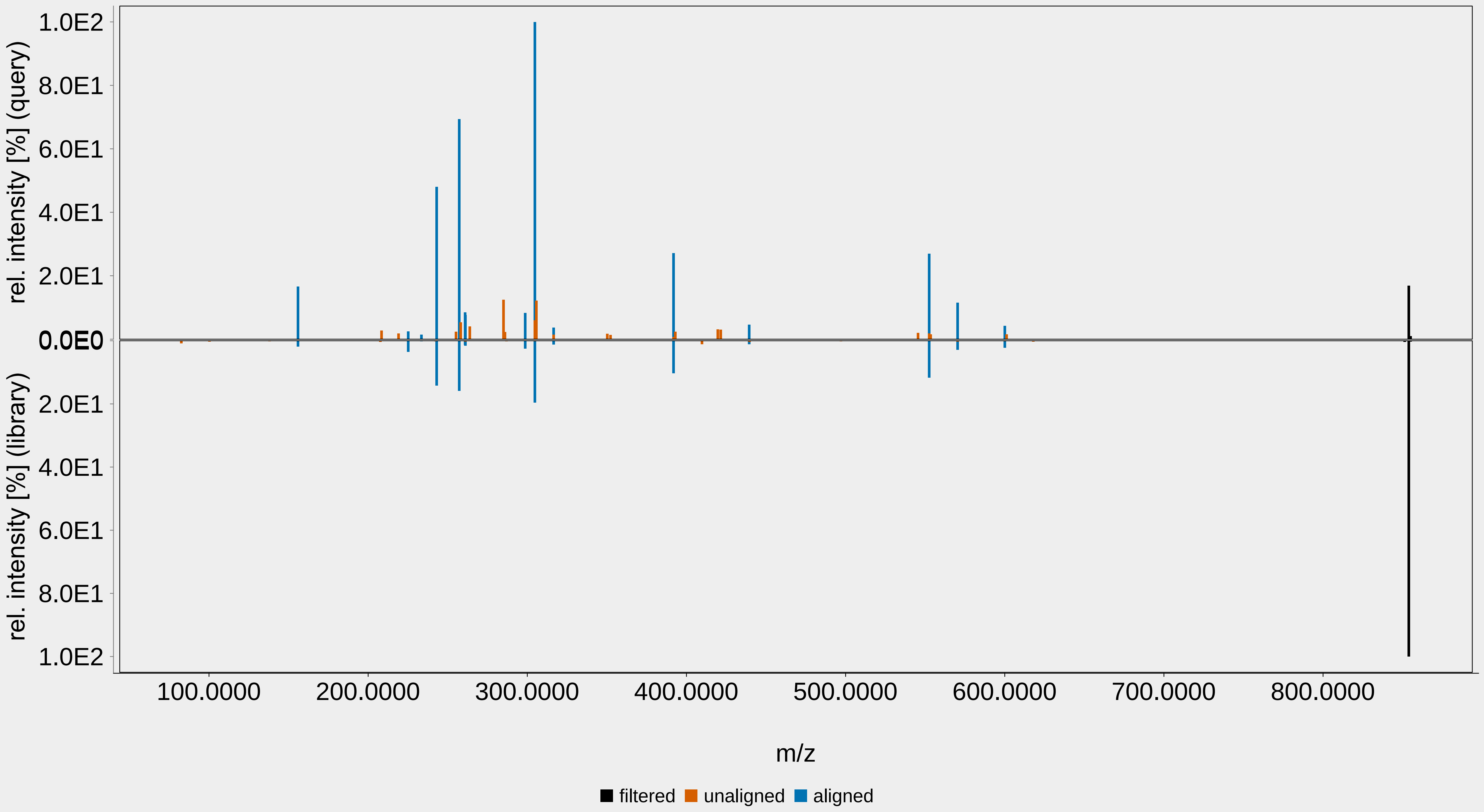

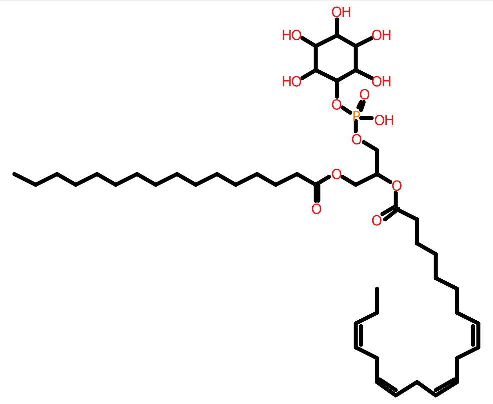

Compound information

Name: PI 36:4

Formula: C45H79O13P

Mol. weight: 859.0899999999999

Exact mass: 859.0899999999999

Ion type: [M-H]-

Precursor m/z: 857.5197

RT: 8.301669

Polarity: negative

InChI:

InChI key:

SMILES:

Instrument information

Instrument type: LC-ESI-QTOF

Instrument: AB Sciex TripleTOF 5600+ system

Ms level: MS2

Collision energy: 45V

Database links

Pubchem: 138284476

MoNA ID: LQB00297

Other information

Principal investigator: Kazutaka Ikeda, Hiroshi

Data collector: Kazutaka Ikeda, Hiroshi

Canrenone

0.939

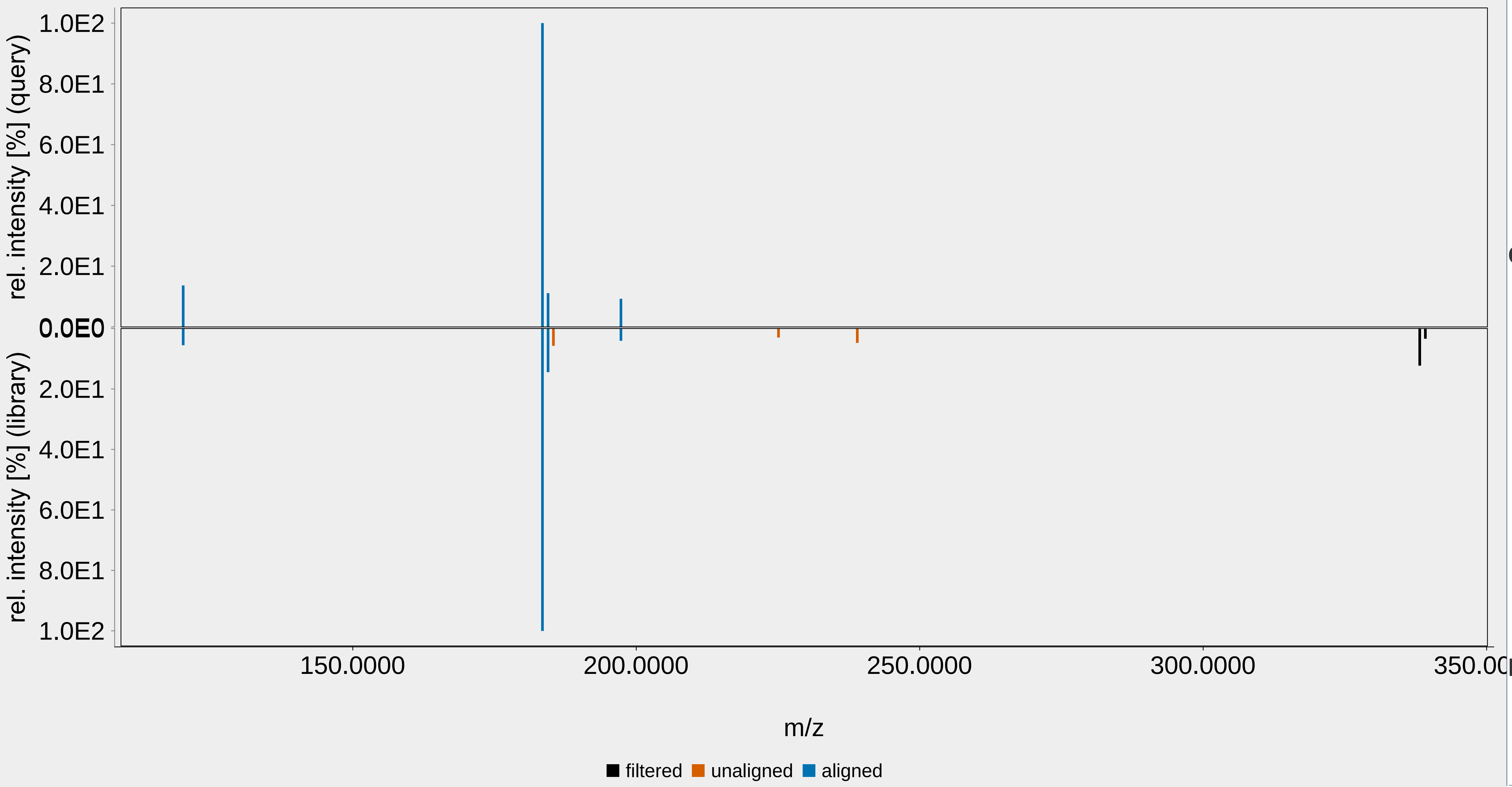

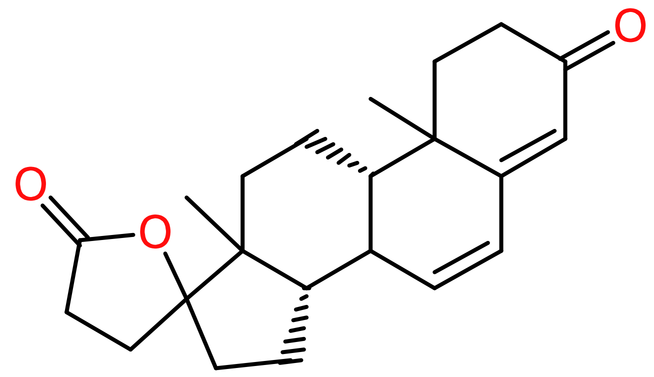

Compound information

Name: Canrenone

Formula: C22H28O3

Exact mass: 340.2038448

Ion type: [M-H]-

Precursor m/z: 339.1966

Polarity: NEGATIVE

InChI:

InChI key:

SMILES:

Num peaks: 9

Instrument information

Instrument type: LC-ESI-QTOF

Instrument: Bruker maXis Impact

Ms level: MS2

Collision energy: 40 eV

Database links

Other information

Entry ID: MSBNK-Athens_Univ-AU239460

Comment: Parent=339.1966

Canrenone, (9S,14S)-10,13-dimethylspiro[2,8,9,11,12,14,15,16-octahydro-1H-cyclopenta[a]phenanthrene-17,5`-oxolane]-2`,3-dione

0.939

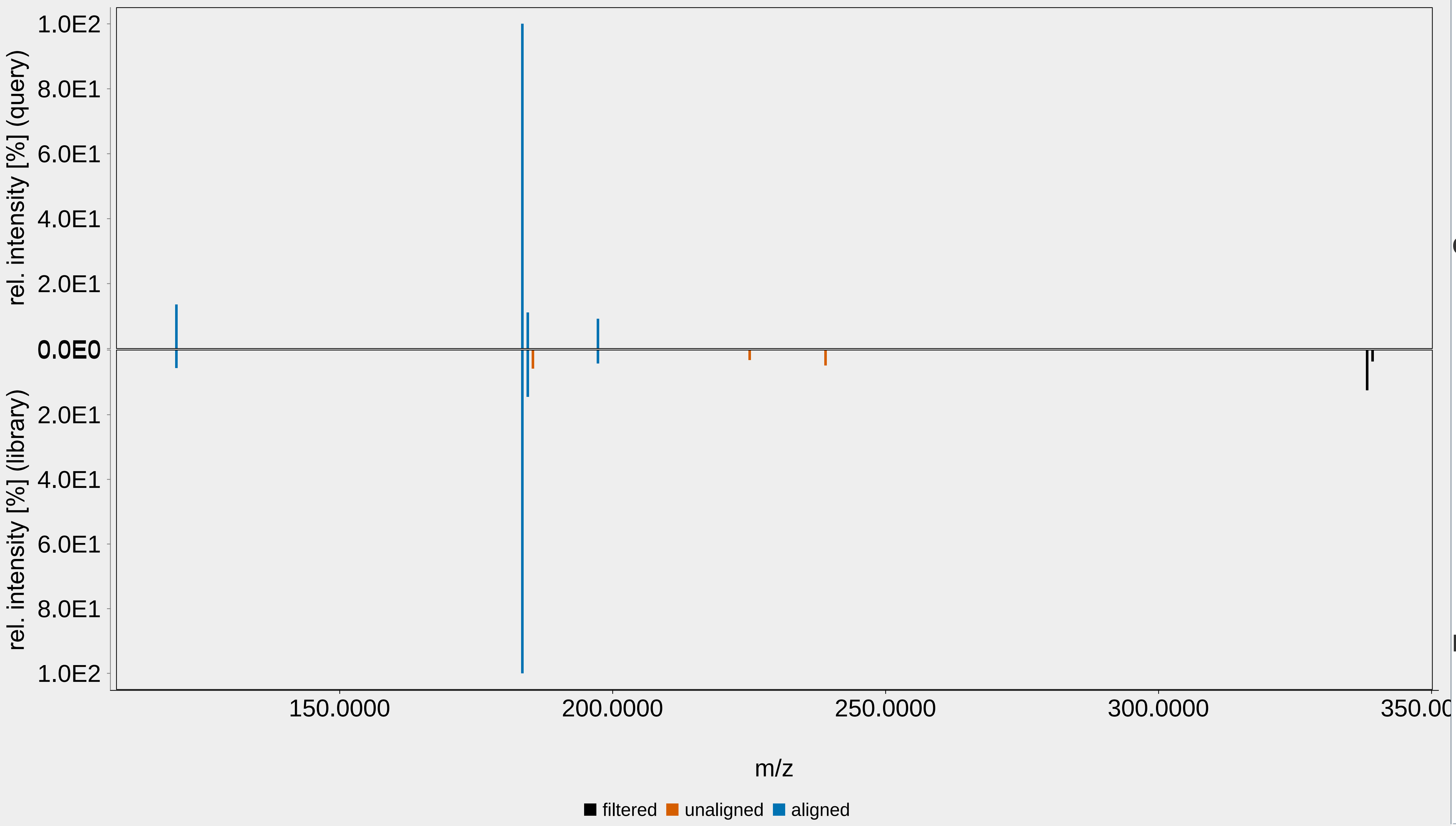

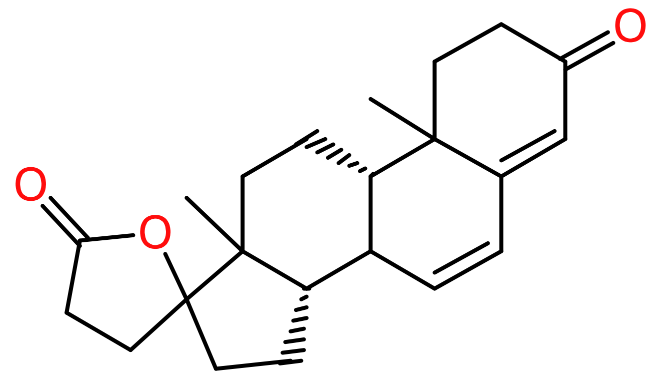

Compound information

Name: Canrenone,

Formula: $C_{22}H_{28}O_3$

Mol. weight: 340.2038448

Exact mass: 340.2038448

Ion type: [M-H]-

Precursor m/z: 339.1966

Polarity: negative

InChI:

InChI key:

SMILES:

Instrument information

Instrument type: LC-ESI-QTOF

Instrument: Bruker maXis Impact

Ion source: ESI

Resolution: 35000

Ms level: MS2

Collision energy: 40 eV

Database links

Pubchem: 24728626

MoNA ID: AU239460

Chemspider: 21112020

CAS: 976-71-6

Other information

Principal investigator: Nikiforos Alygizakis,

Data collector: Nikiforos Alygizakis, Katerina

PA 18:0_22:6

0.827

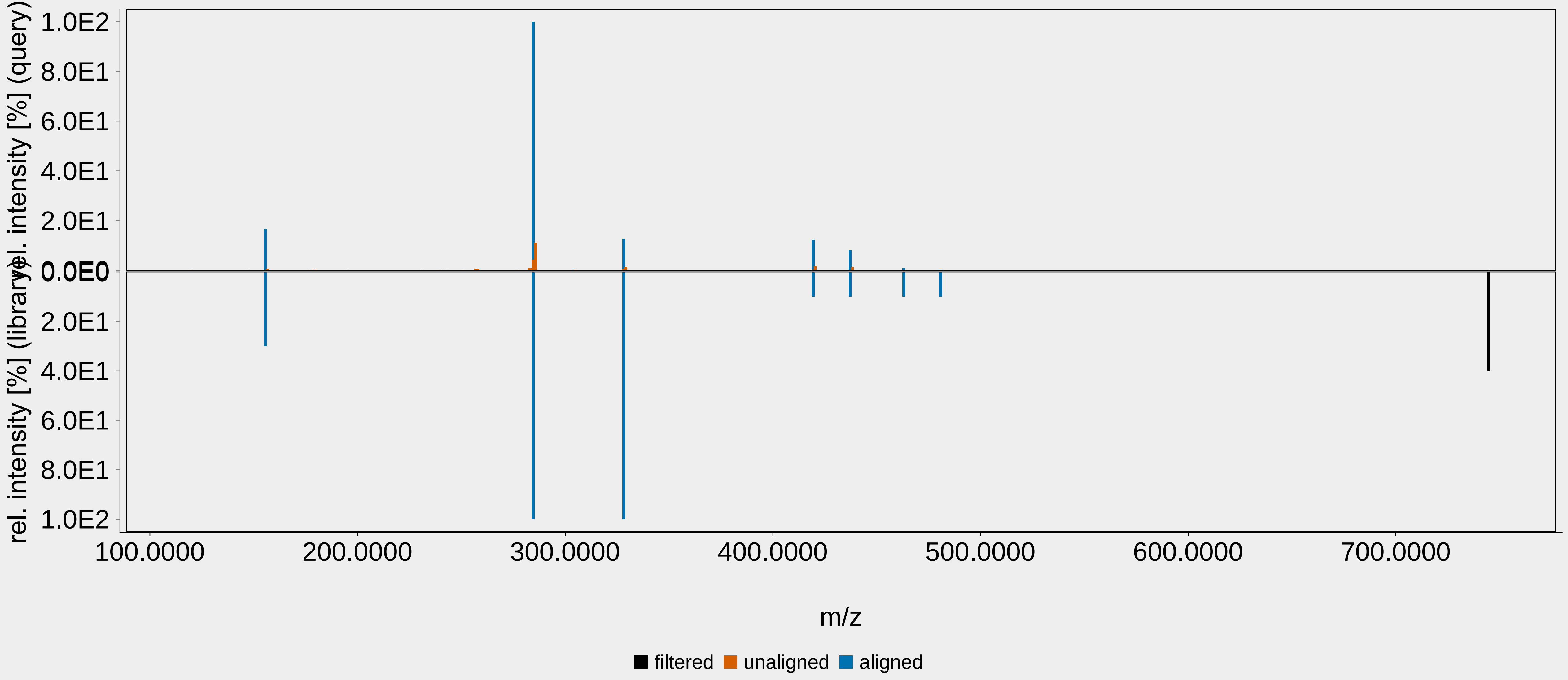

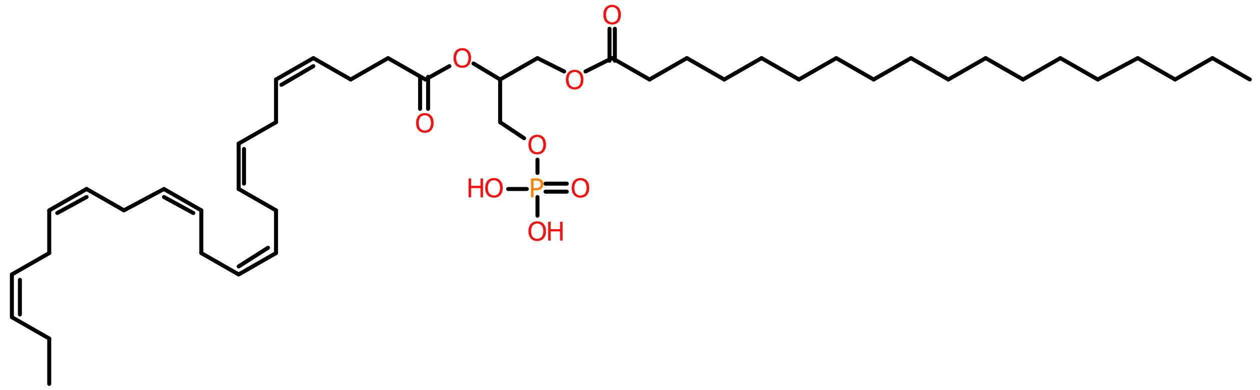

Compound information

Name: PA 18:0_22:6

Formula: C43H73O8P

Ion type: [M-H]-

Precursor m/z: 747.49703

Ccs: 266.76

InChI key:

SMILES:

Num peaks: 8

Instrument information

Database links

Other information

Spectral Match to 1-Octadecanoyl-2-(4Z,7Z,10Z,13Z,16Z,19Z-docosahexaenoyl)-sn-glycero-3-phospho-L-serine from NIST14

0.881

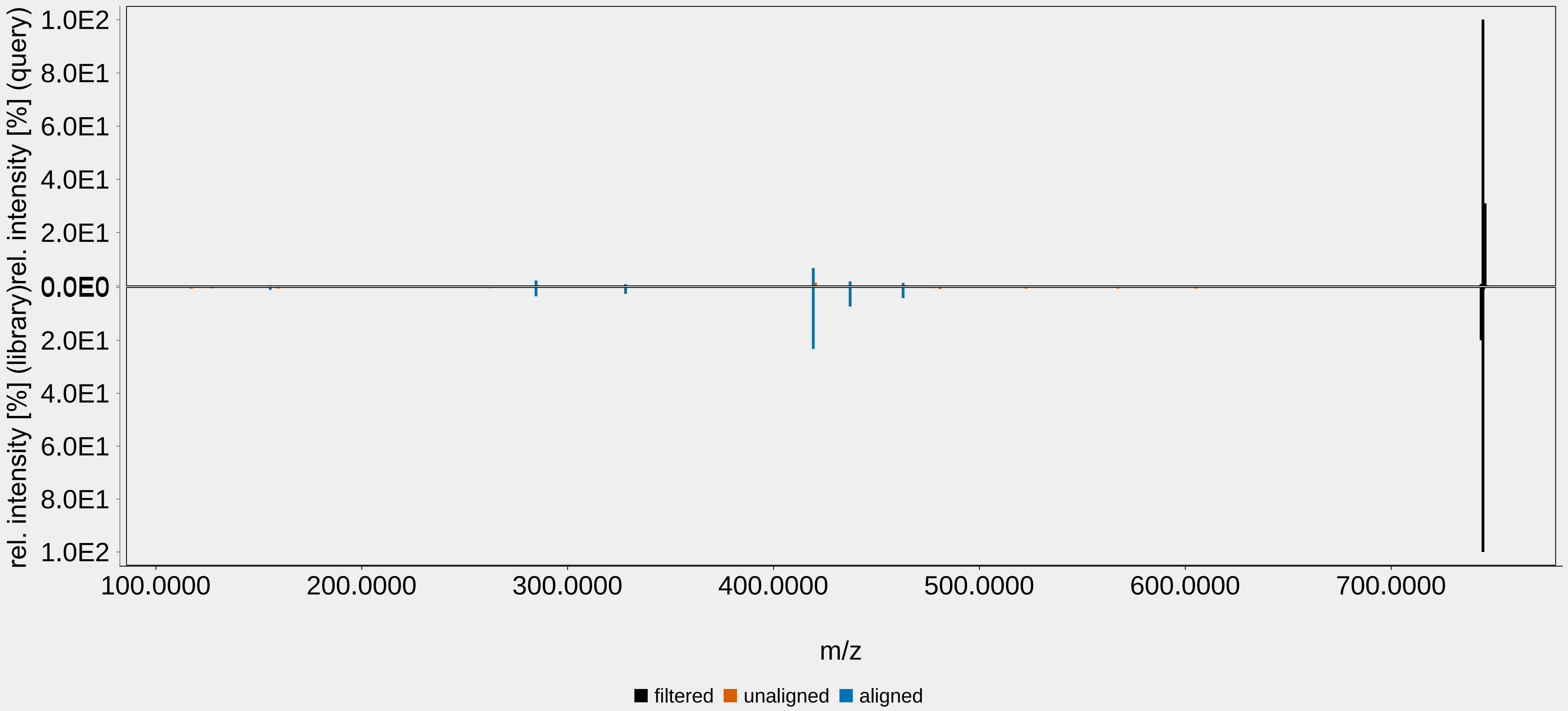

Compound information

Name: Spectral Match to

Exact mass: 0.0

Ion type: M-H-C3H5NO2

Precursor m/z: 747.498

Charge: 1

Polarity: Negative

Instrument information

Instrument type: IT/ion trap

Ion source: ESI

Ms level: 2

Acquisition: Isolated

Database links

CAS: 137173989

Other information

Principal investigator: Data from Katrina

Data collector: Data deposited by alchemistmatt

Entry ID: CCMSLIB00003134591

[1-[[2,3-dihydroxypropoxy]-hydroxyphosphoryl]oxy-3-[octadec-11-enoyl]oxypropan-2-yl] octadecanoate

0.758

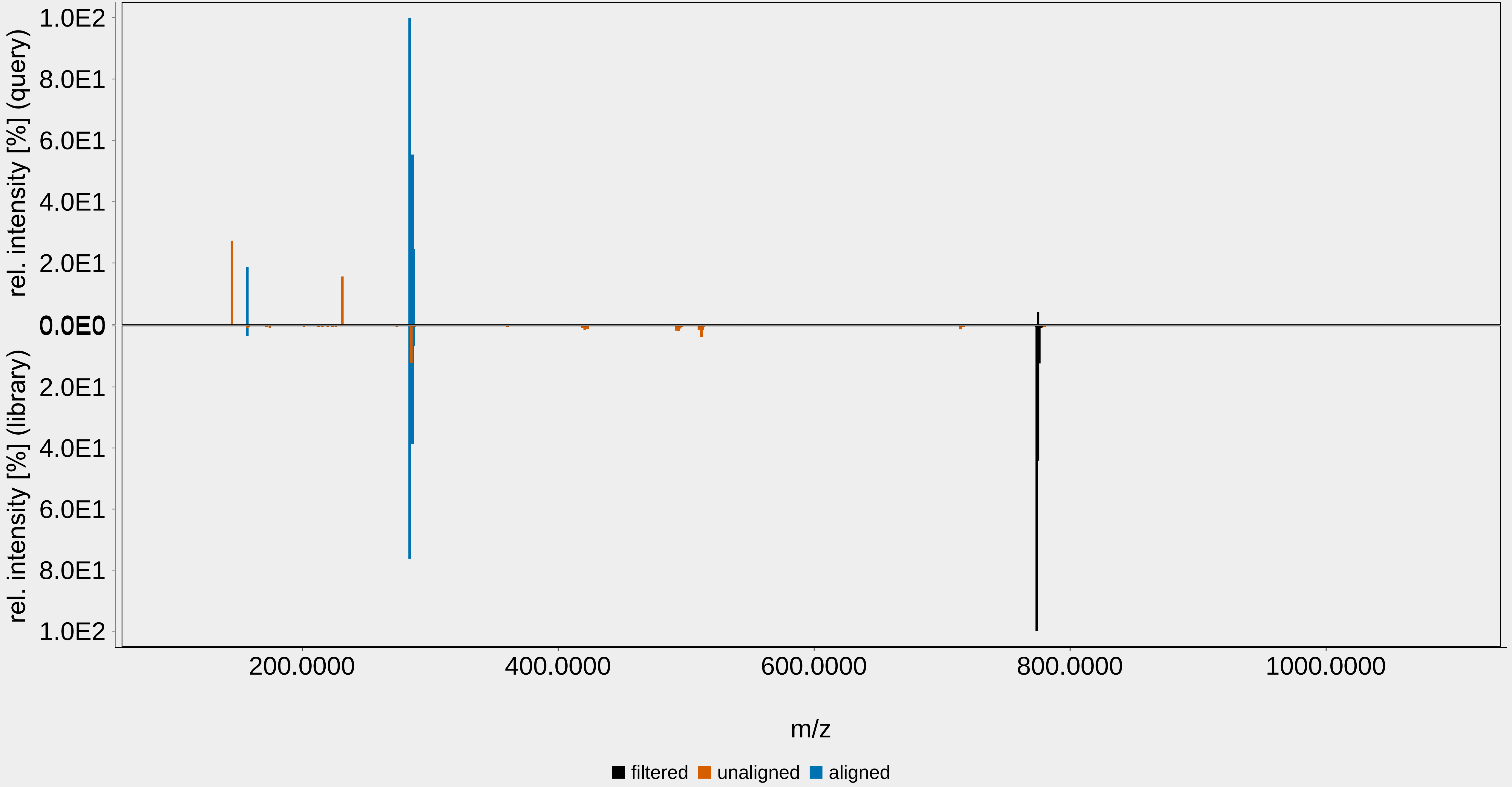

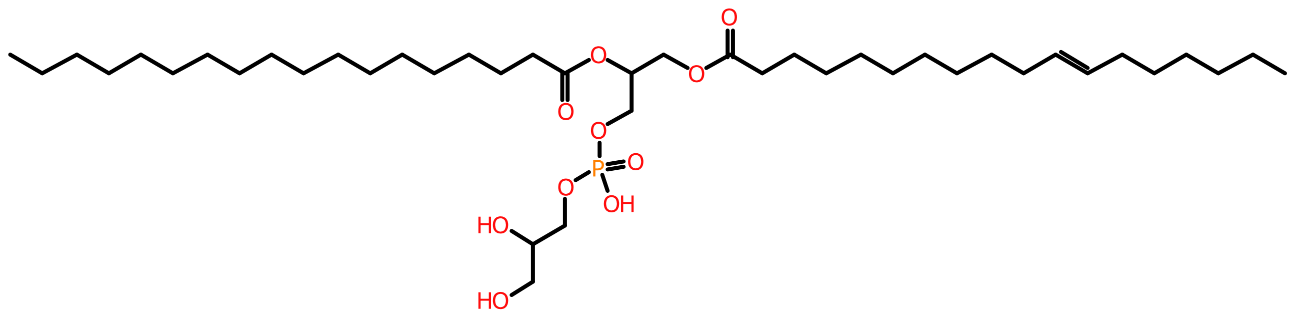

Compound information

Name:

Formula: C42H81O10P

Mol. weight: 776.557

Exact mass: 776.557

Ion type: [M-H]-

Precursor m/z: 775.549

Polarity: negative

InChI:

InChI key:

SMILES:

Instrument information

Instrument: qTof

Ms level: MS2

Database links

Other information

Principal investigator: O Laprevote, UMR 8038

Data collector: O Laprevote, UMR 8038

PG(18:0_18:1) - [2.3-dihydroxypropoxy][2-[octadec-9-enoyloxy]-3-(octadecanoyloxy)propoxy]phosphinic acid 0.775

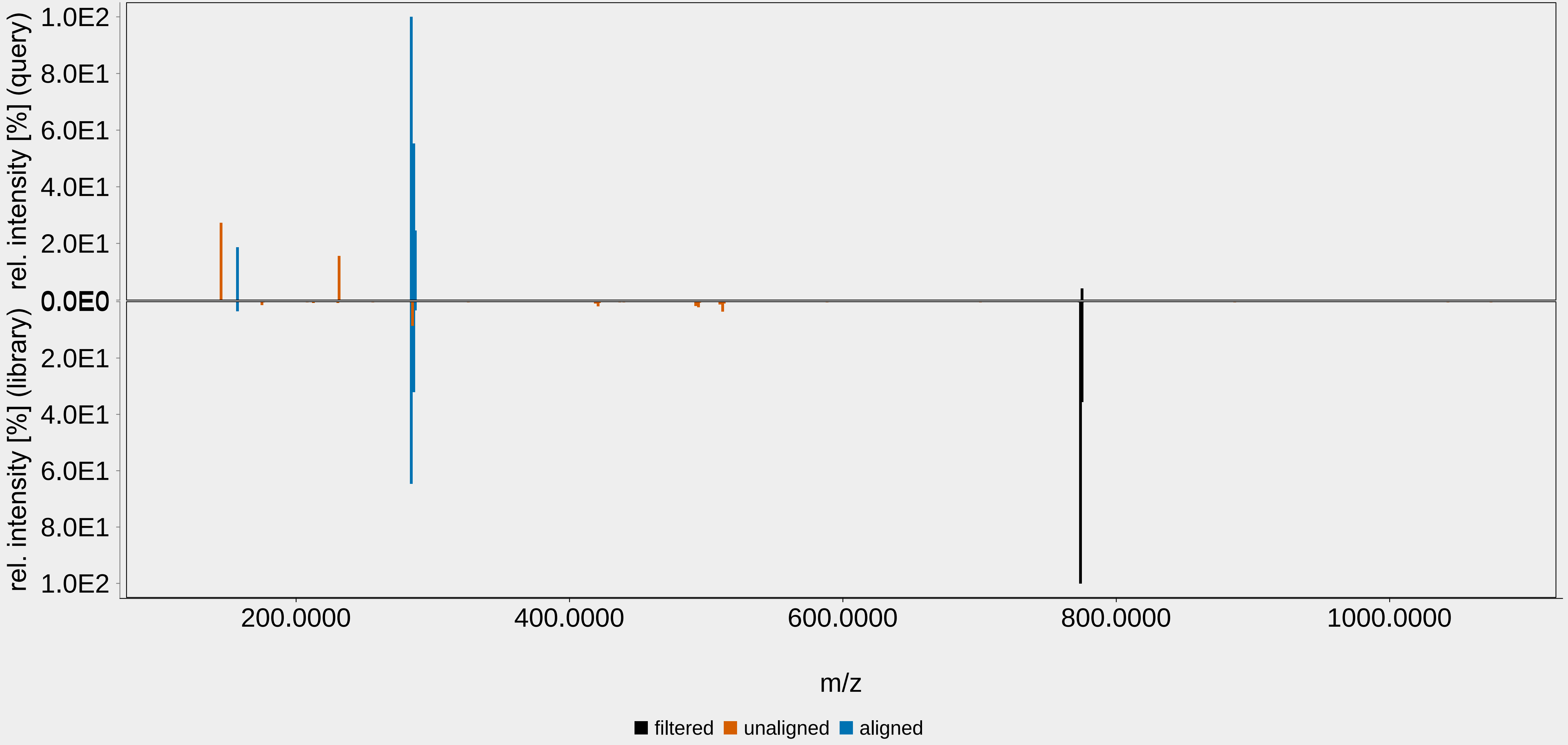

Compound information

Name: PG(18:0_18:1) -

Exact mass: 776.557

Ion type: M-H

Precursor m/z: 775.547

Charge: 1

Polarity: Negative

SMILES:

Instrument information

Instrument type: qTof

Ion source: LC-ESI

Ms level: 2

Acquisition: Lysate

Database links

Other information

Principal investigator: N Auzeil

Data collector: UMR CNRS 8038 Universite de

Entry ID: CCMSLIB00005724243

uridine 5?-diphosphate - 30.0 eV

0.882

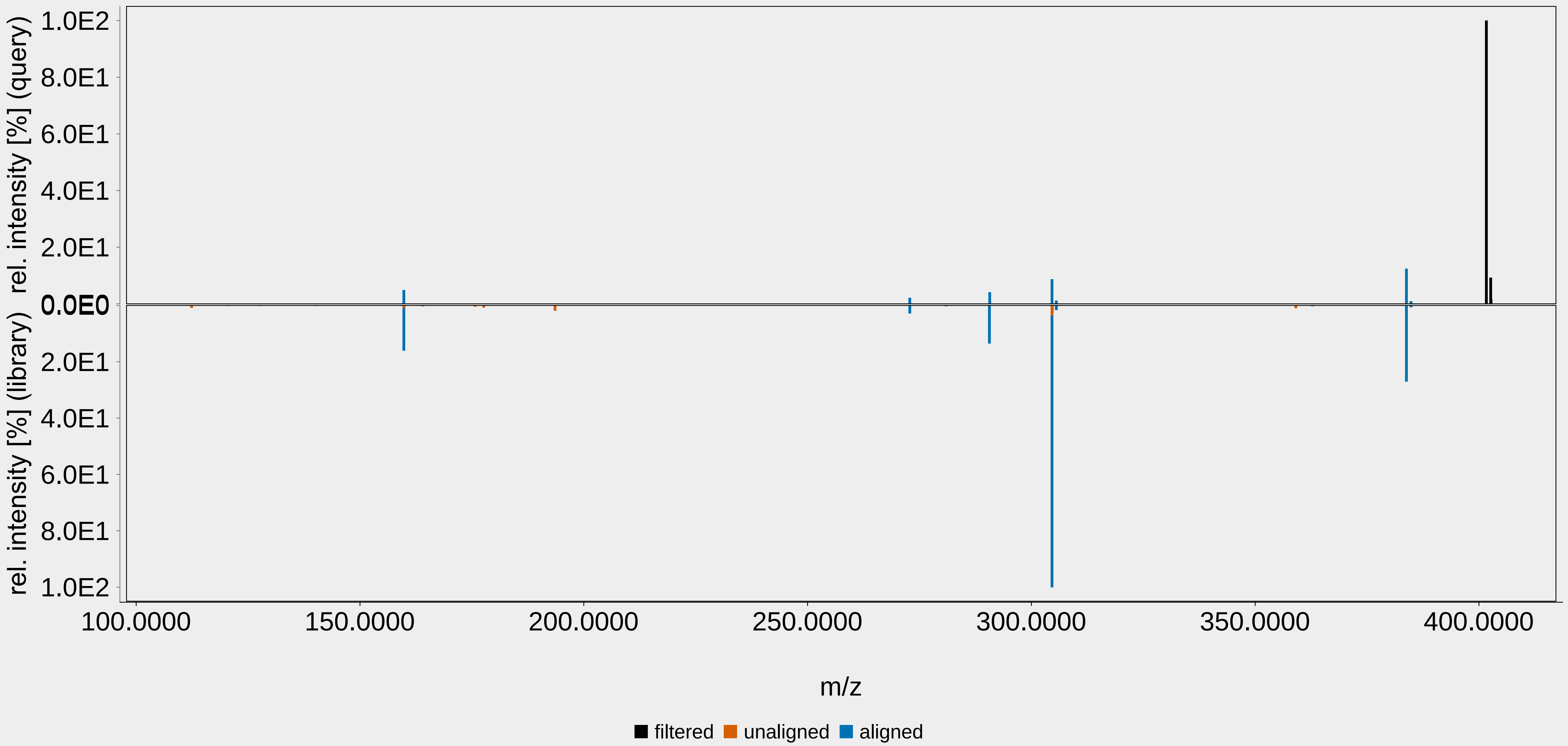

Compound information

Name: uridine 5?-diphosphate - 30.0 eV

Exact mass: 0.0

Ion type: M-H

Precursor m/z: 402.995

Charge: 1

Polarity: Negative

SMILES:

Instrument information

Instrument type: Orbitrap

Ion source: ESI

Ms level: 2

Acquisition: Isolated

Database links

CAS:

Other information

Principal investigator: PI

Data collector: Data Collector

Entry ID: CCMSLIB00006123791

uridine 5?-diphosphate - 30.0 eV

0.899

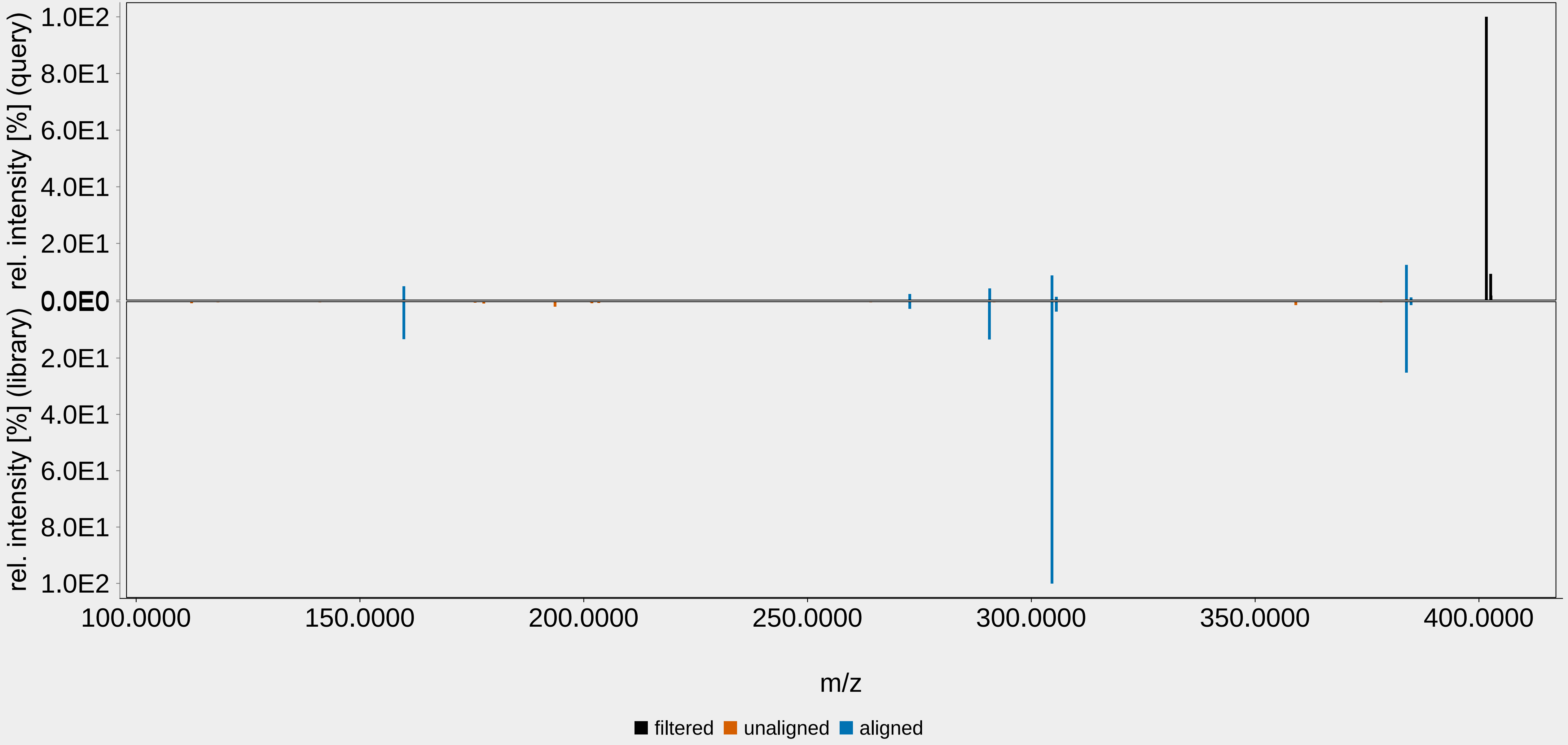

Compound information

Name: uridine 5?-diphosphate - 30.0 eV

Exact mass: 0.0

Ion type: M-H

Precursor m/z: 402.995

Charge: 1

Polarity: Negative

SMILES:

Instrument information

Instrument type: Orbitrap

Ion source: ESI

Ms level: 2

Acquisition: Isolated

Database links

CAS:

Other information

Principal investigator: PI

Data collector: Data Collector

Entry ID: CCMSLIB00006123813

PS(22:6/22:6); [M-H]- C50H73N1O10P1

0.760

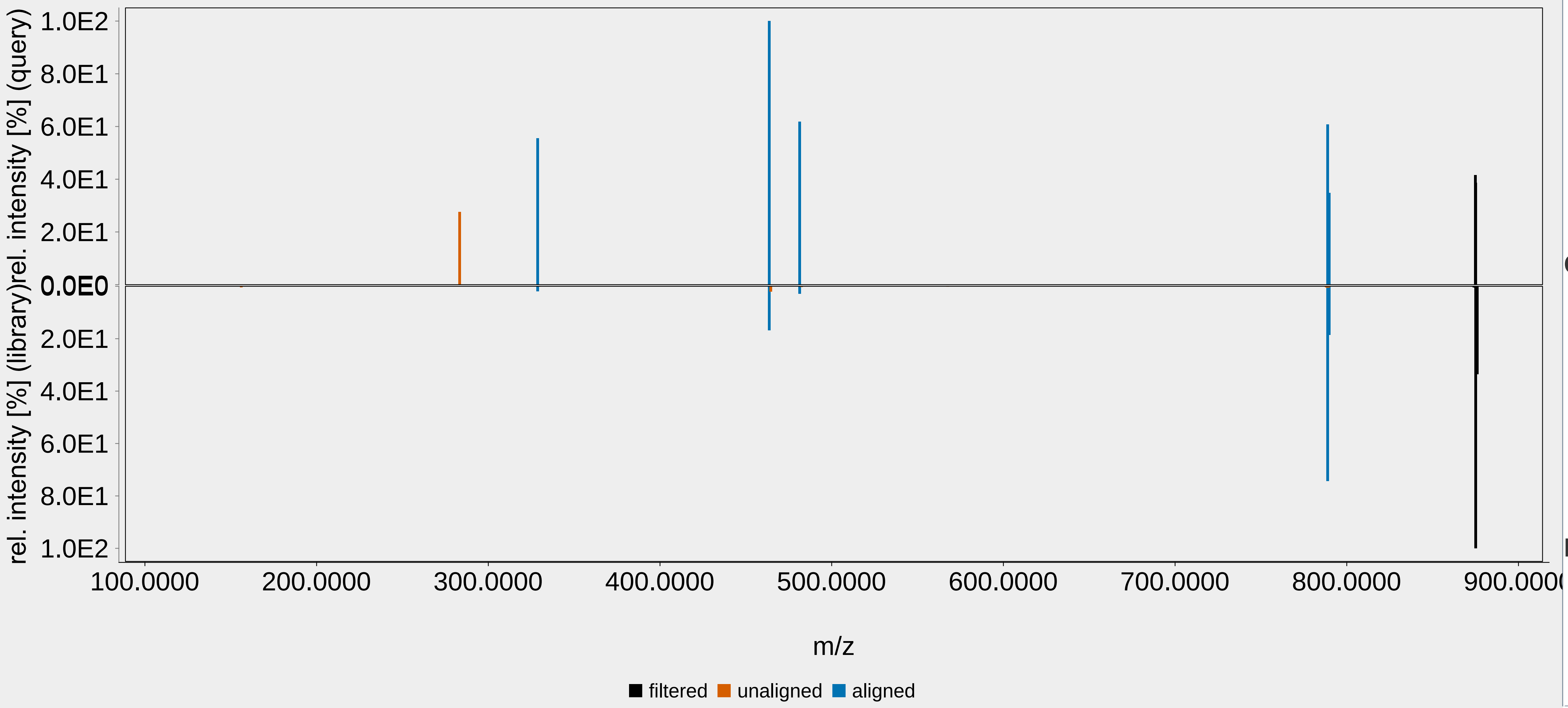

Compound information

Name: PS(22:6/22:6); [M-H]- C50H73N1O10P1

Exact mass: 0.0

Ion type: M-H

Precursor m/z: 878.497

Charge: 1

Polarity: Negative

Instrument information

Instrument type: HCD; Velos

Ion source: LC-ESI

Ms level: 2

Acquisition: Commercial

Database links

Other information

Principal investigator: Thomas Metz

Data collector: Thomas Metz

Entry ID: CCMSLIB00010083009

PS 22:6_22:6

0.940

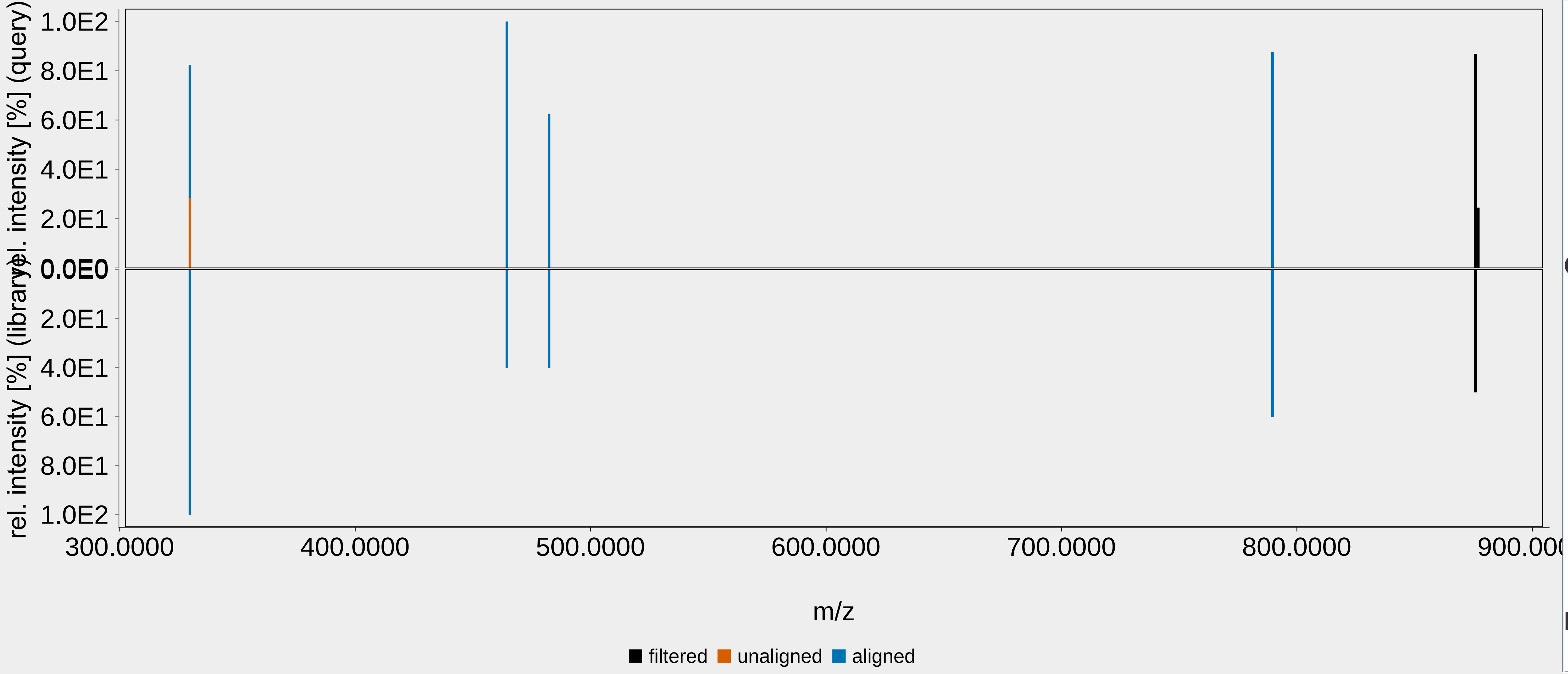

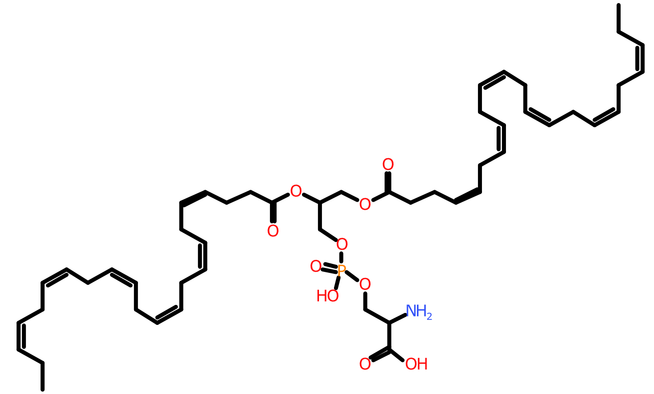

Compound information

Name: PS 22:6_22:6

Formula: C50H74NO10P

Ion type: [M-H]-

Precursor m/z: 878.49776

Ccs: 284.65

InChI key:

SMILES:

Num peaks: 5

Instrument information

Database links

Other information

PS 18:1_22:1 **0.787**

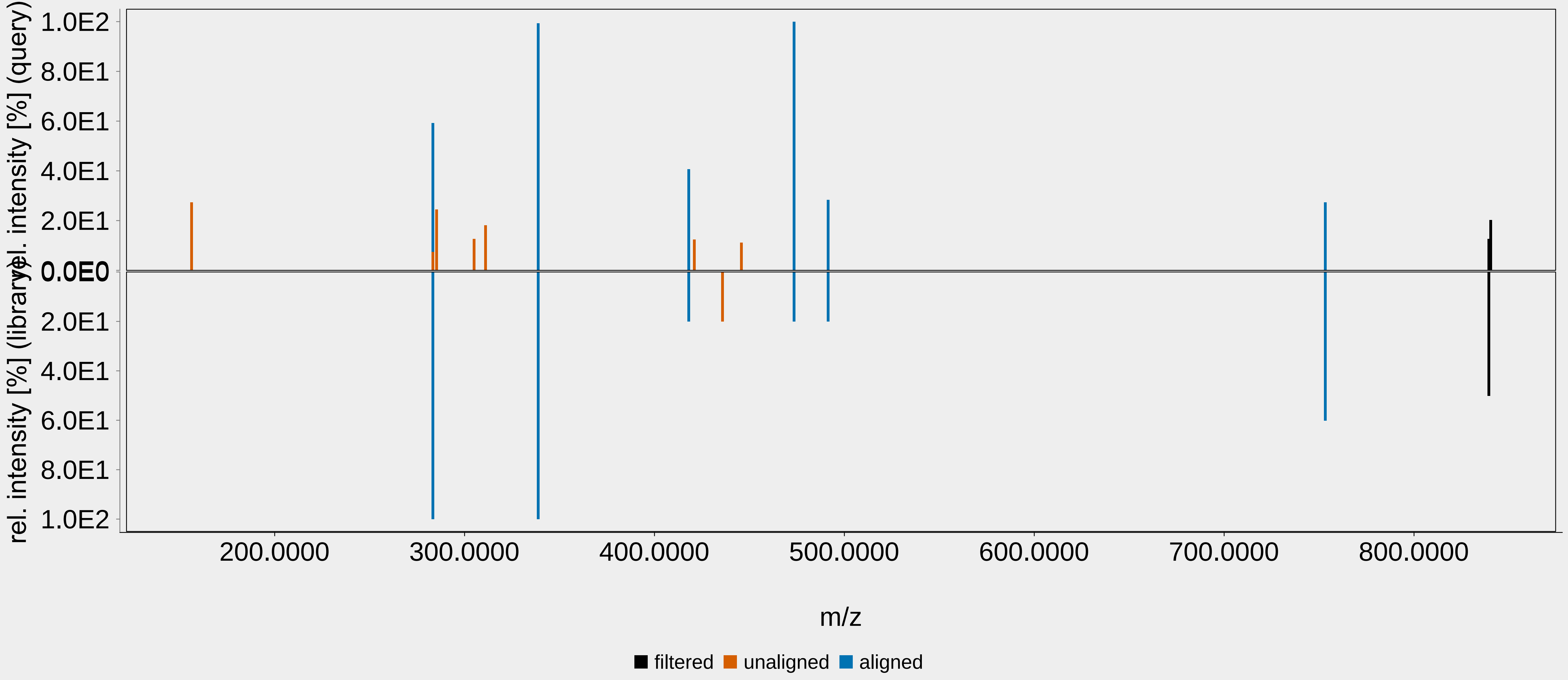

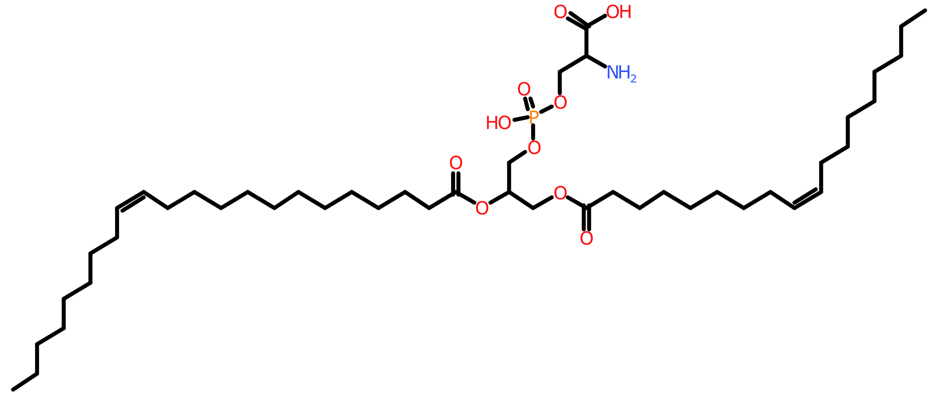

Compound information

Name: PS 18:1_22:1

Formula: C46H86NO10P

Ion type: [M-H]-

Precursor m/z: 842.59166

Ccs: 287.03

InChI key:

SMILES:

Num peaks: 8

Instrument information

Database links

Other information

PA 18:1_20:1 **0.720**

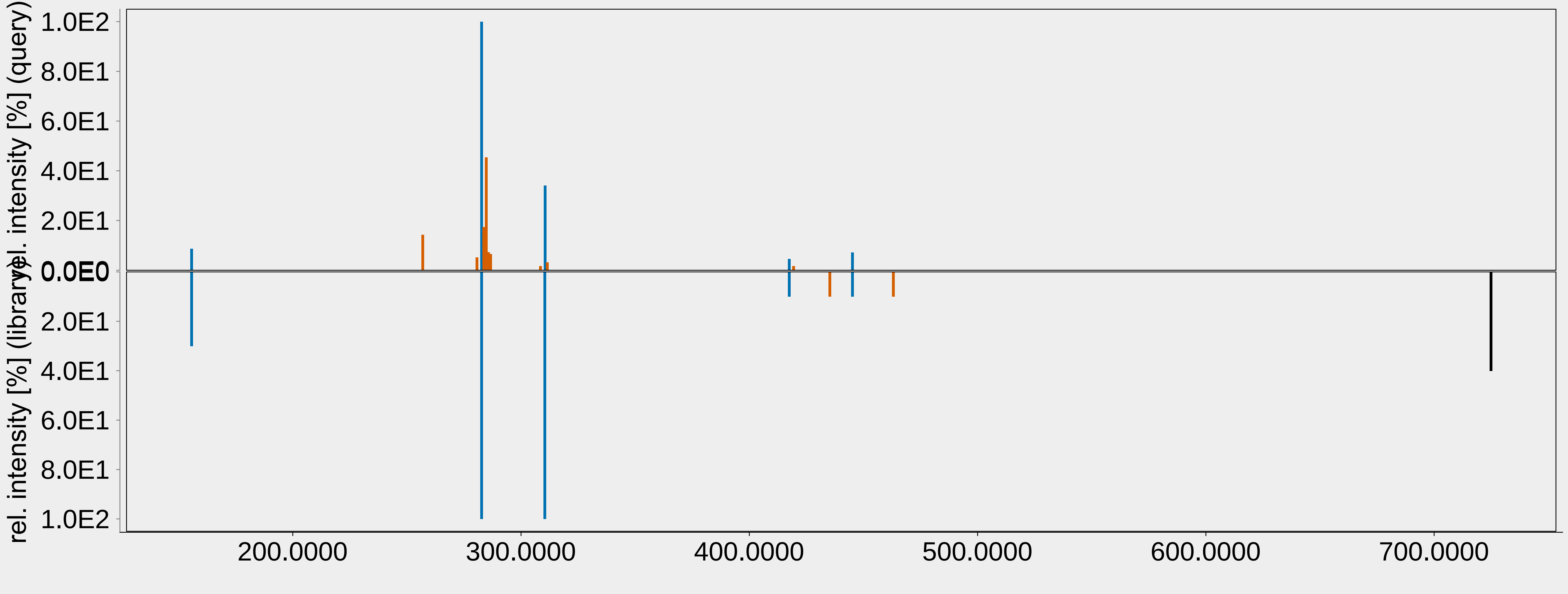

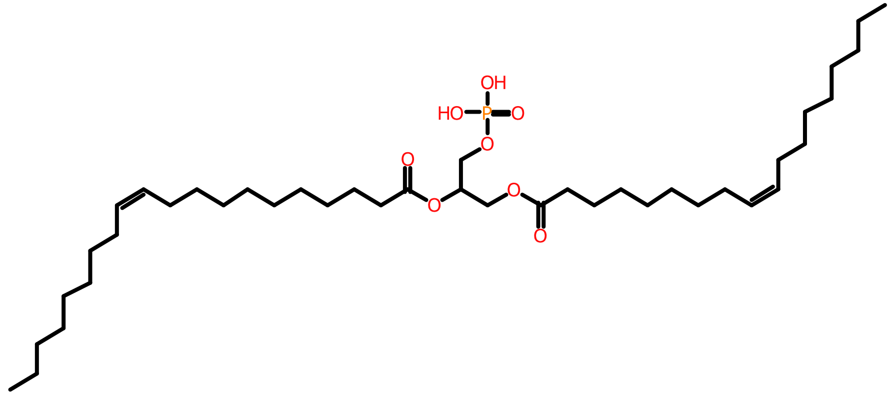

Compound information

Name: PA 18:1_20:1

Formula: C41H77O8P

Ion type: [M-H]-

Precursor m/z: 727.52833

Ccs: 265.22

InChI key:

SMILES:

Num peaks: 8

Instrument information

Database links

Other information

PG(16:1/18:1); [M-H]- C40H74O10P1 **0.779**

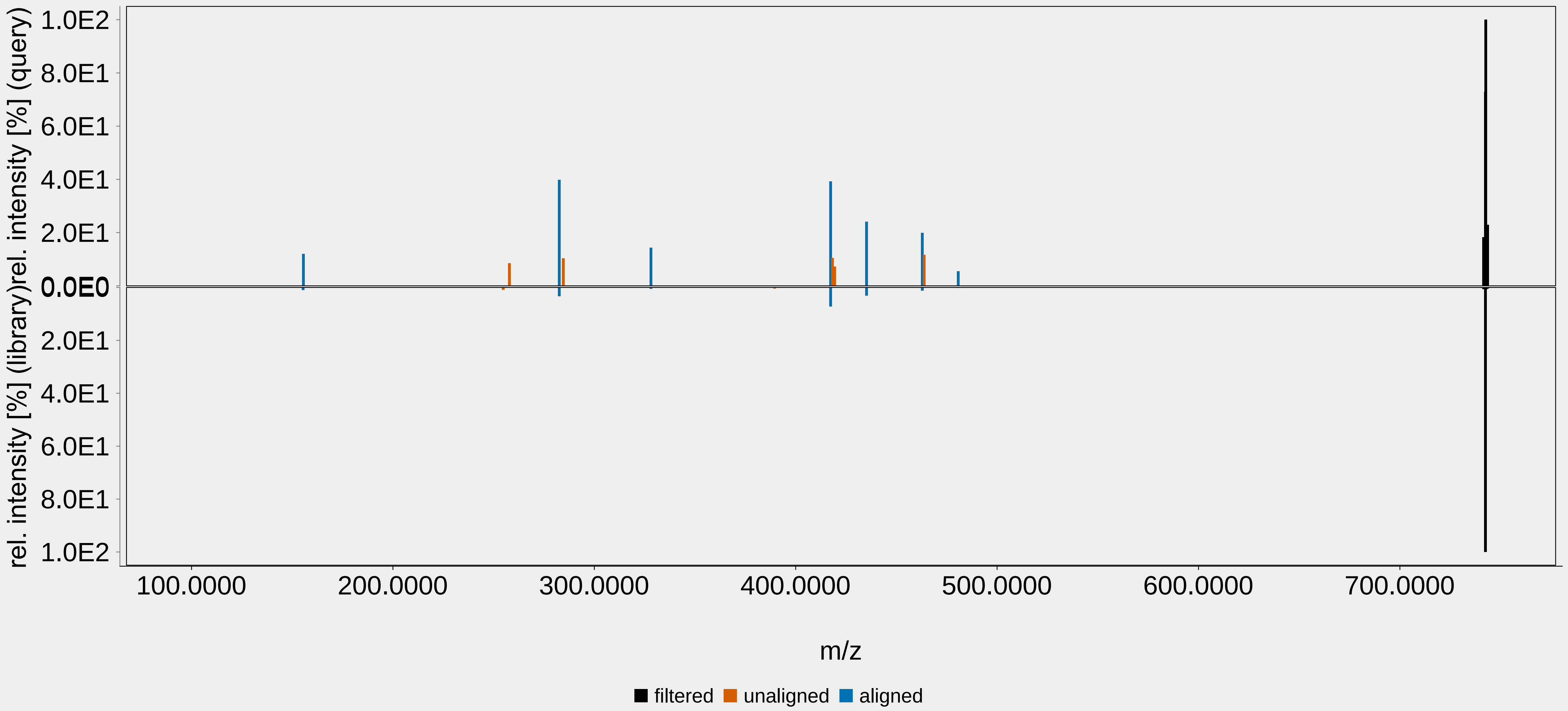

Compound information

Name: PG(16:1/18:1); [M-H]- C40H74O10P1

Exact mass: 0.0

Ion type: M-H

Precursor m/z: 745.502

Charge: 1

Polarity: Negative

Instrument information

Instrument type: HCD; Velos

Ion source: LC-ESI

Ms level: 2

Acquisition: Commercial

Database links

Other information

Principal investigator: Thomas Metz

Data collector: Thomas Metz

Entry ID: CCMSLIB00010087352

PG(16:1/18:1); [M-H]- C40H74O10P1 **0.782**

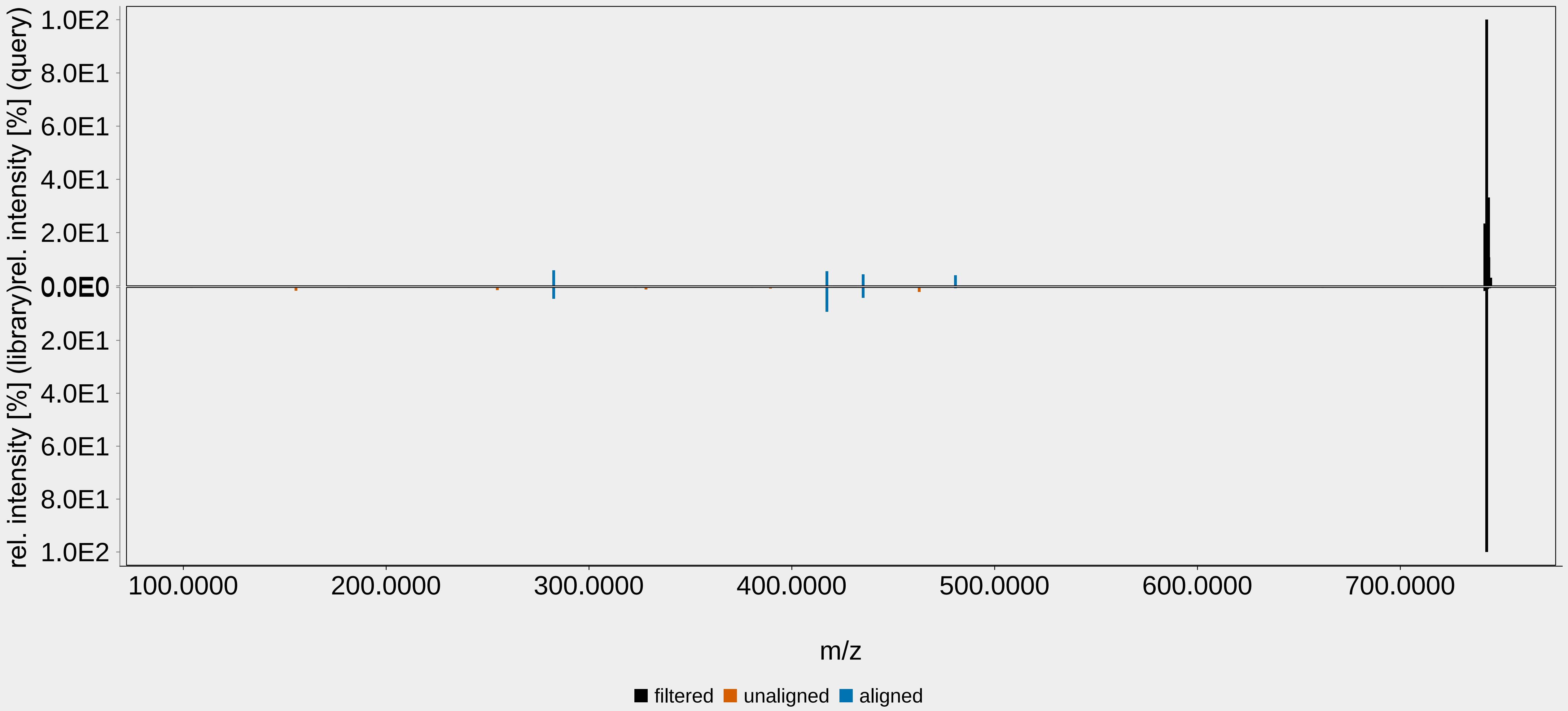

Compound information

Name: PG(16:1/18:1); [M-H]- C40H74O10P1

Exact mass: 0.0

Ion type: M-H

Precursor m/z: 745.502

Charge: 1

Polarity: Negative

Instrument information

Instrument type: HCD; Velos

Ion source: LC-ESI

Ms level: 2

Acquisition: Commercial

Database links

Other information

Principal investigator: Thomas Metz

Data collector: Thomas Metz

Entry ID: CCMSLIB00010073906

PS 40:4 **0.883**

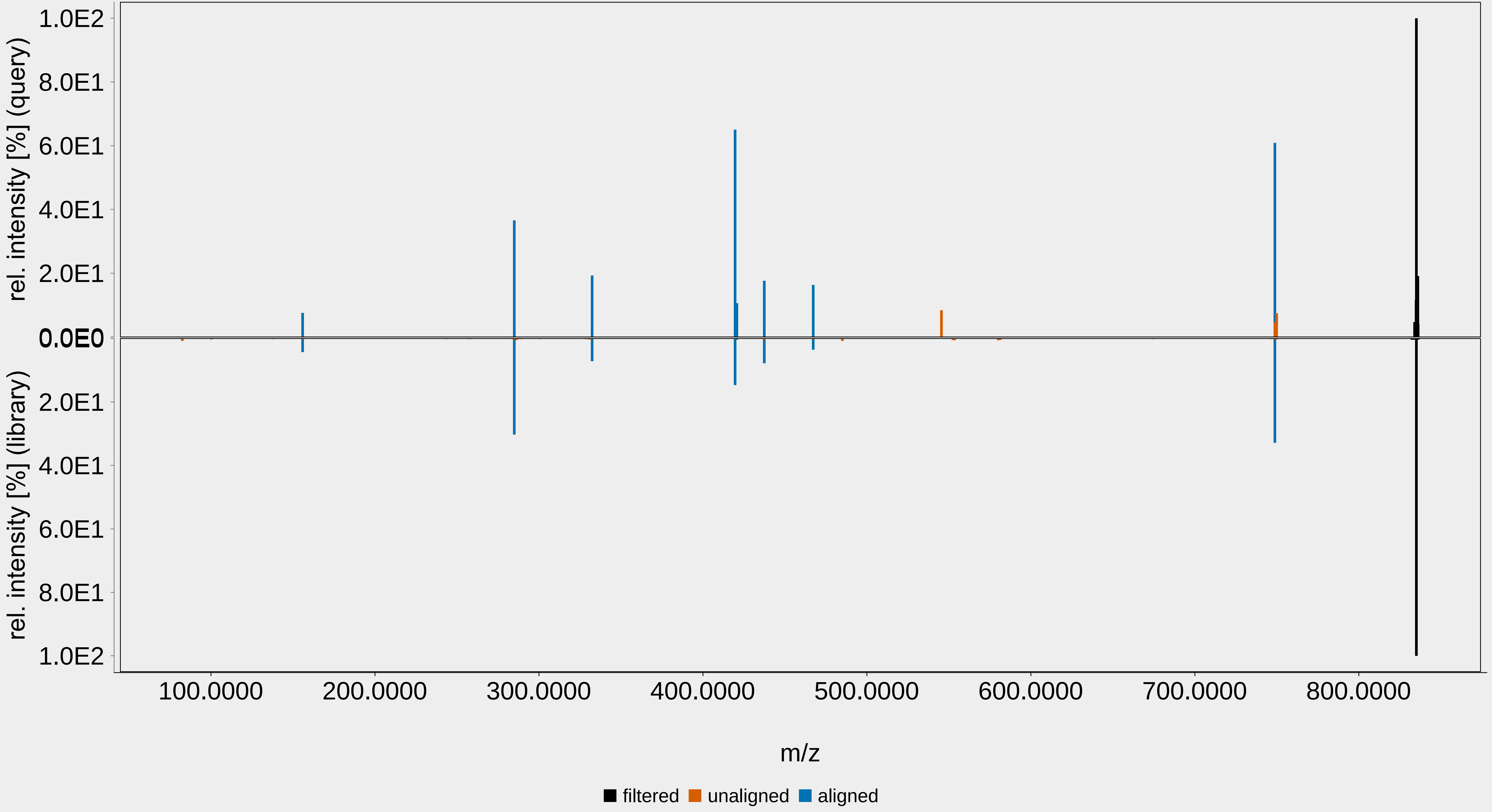

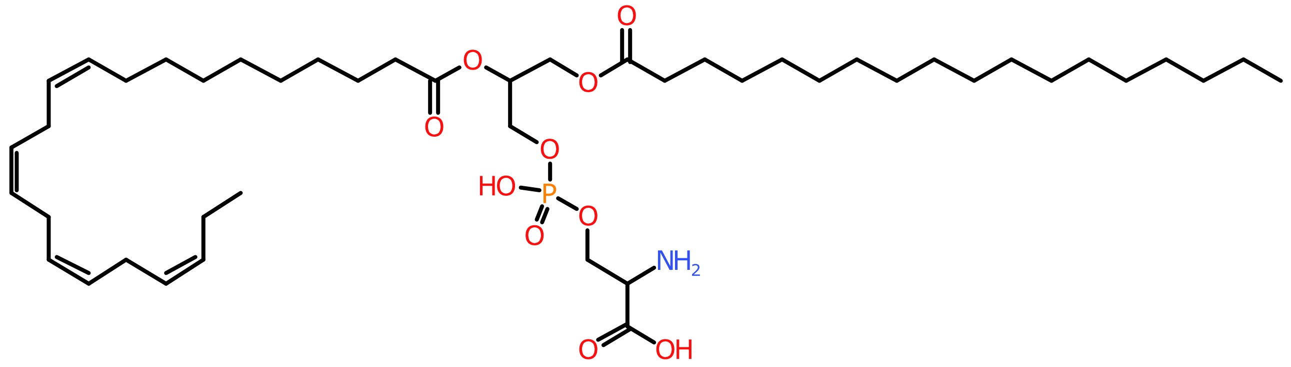

Compound information

Name: PS 40:4

Formula: C46H82NO10P

Mol. weight: 840.135

Exact mass: 840.135

Ion type: [M-H]-

Precursor m/z: 838.5547

RT: 9.189651

Polarity: negative

InChI:

InChI key:

SMILES:

Instrument information

Instrument type: LC-ESI-QTOF

Instrument: AB Sciex TripleTOF 5600+ system

Ms level: MS2

Collision energy: 45V

Database links

Pubchem: 138202734

MoNA ID: LQB00724

Other information

Principal investigator: Kazutaka Ikeda, Hiroshi

Data collector: Kazutaka Ikeda, Hiroshi

PS 40:4

0.883

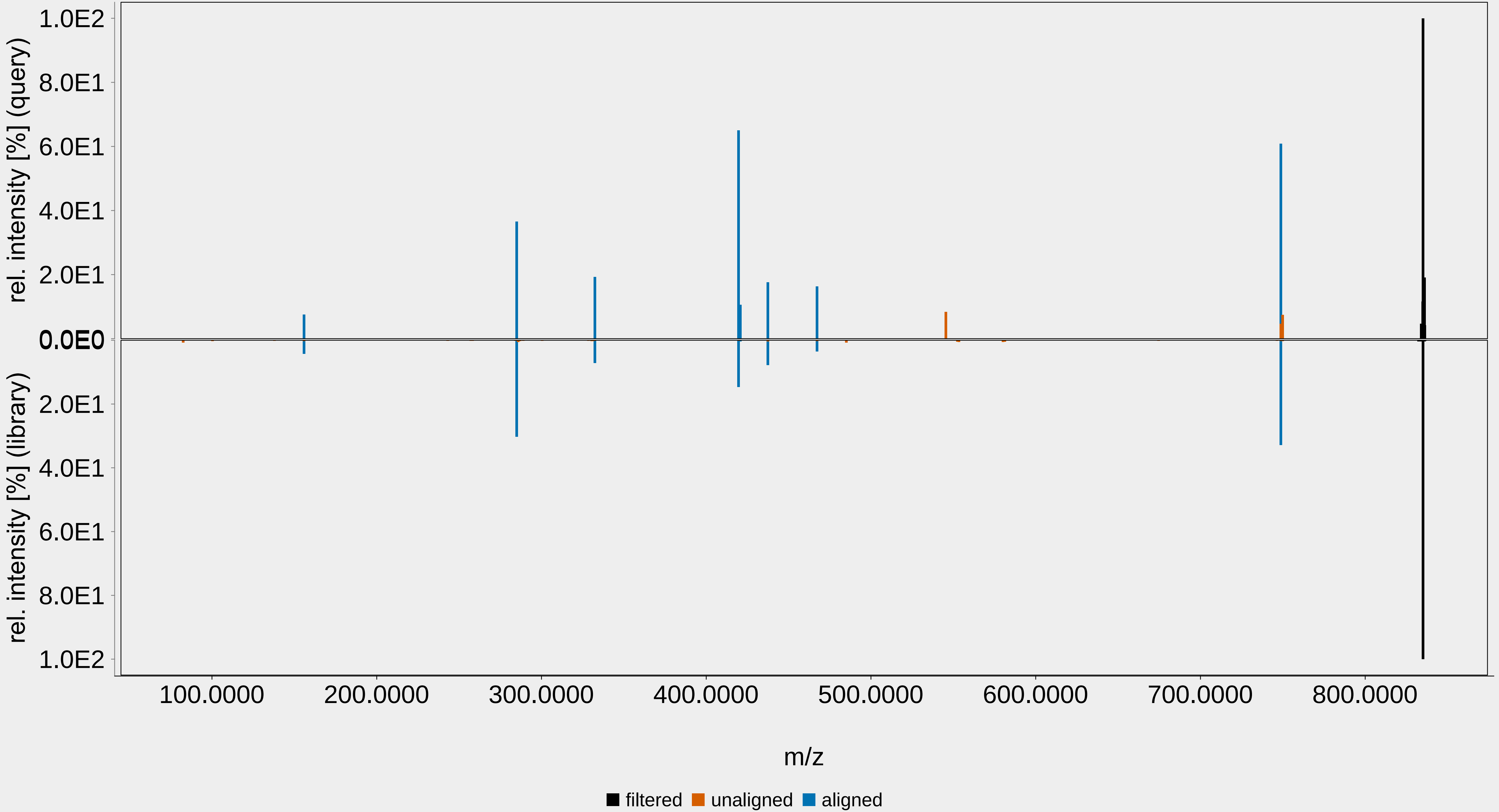

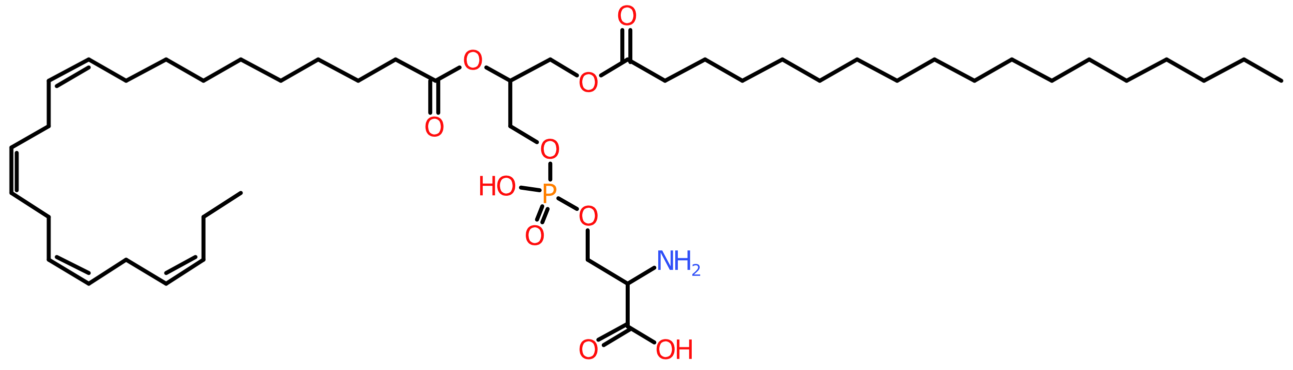

Compound information

Name: PS 40:4

Formula: C46H82NO10P

Mol. weight: 840.135

Exact mass: 840.135

Ion type: [M-H]-

Precursor m/z: 838.5547

RT: 9.189651

Polarity: negative

InChI:

InChI key:

SMILES:

Instrument information

Instrument type: LC-ESI-QTOF

Instrument: AB Sciex TripleTOF 5600+ system

Ms level: MS2

Collision energy: 45V

Database links

Pubchem: 138202734

MoNA ID: LQB00338

Other information

Principal investigator: Kazutaka Ikeda, Hiroshi

Data collector: Kazutaka Ikeda, Hiroshi

PHYTIC ACID

0.904

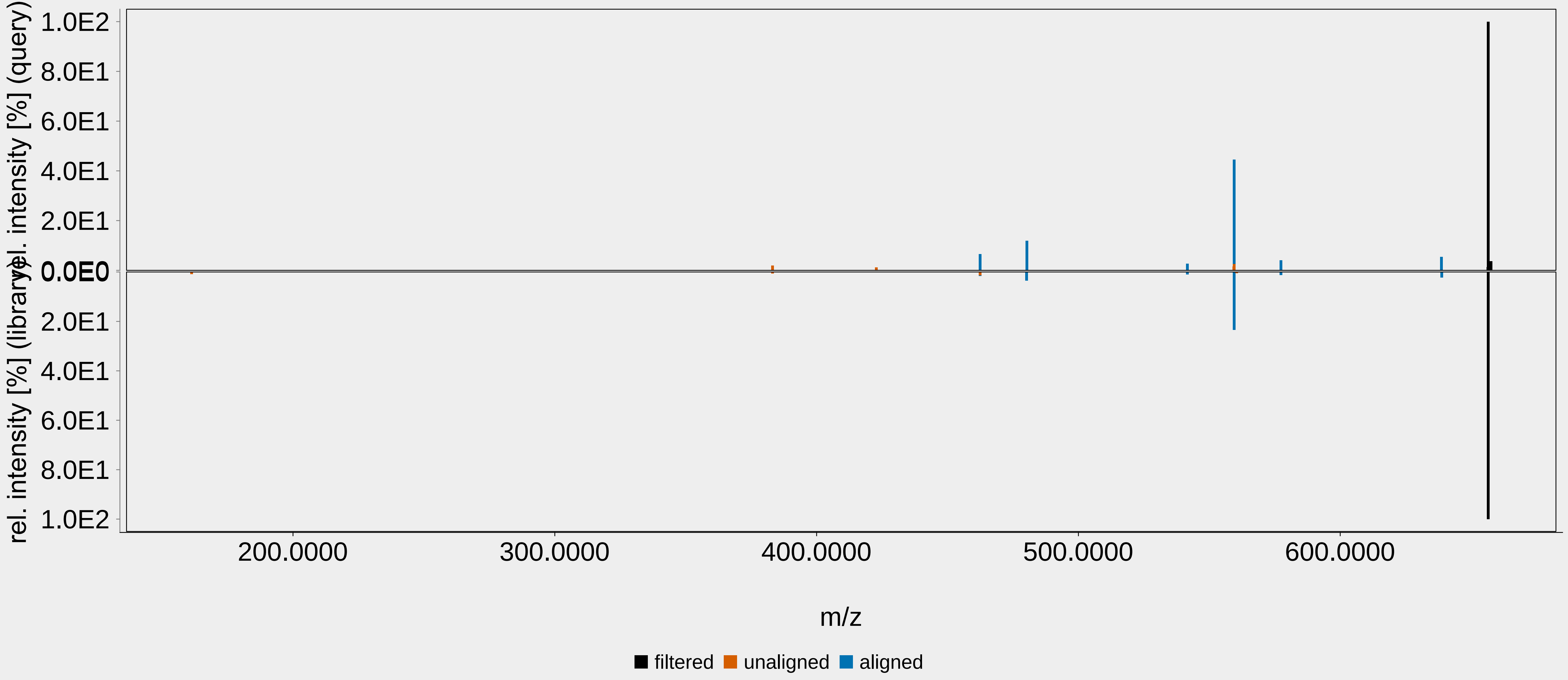

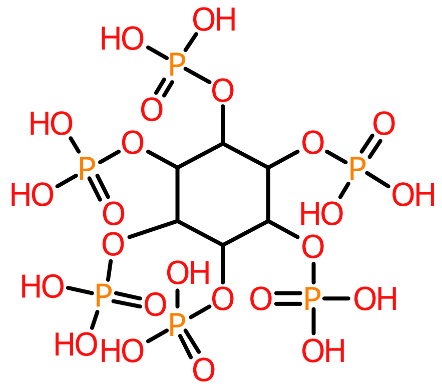

Compound information

Name: PHYTIC ACID

Formula: C6H18O24P6

Ion type: [M-H]-

Precursor m/z: 658.8540952

Polarity: negative

InChI:

InChI key: IMQLKJBTEOYOSI-UHFFFAOYSA-I

SMILES:

Instrument information

Instrument type: LC-ESI-QTOF

Instrument: Agilent qTOF 6545

Ms level: MS2

Collision energy: 20 eV

Database links

Other information

PHYTIC ACID

0.904

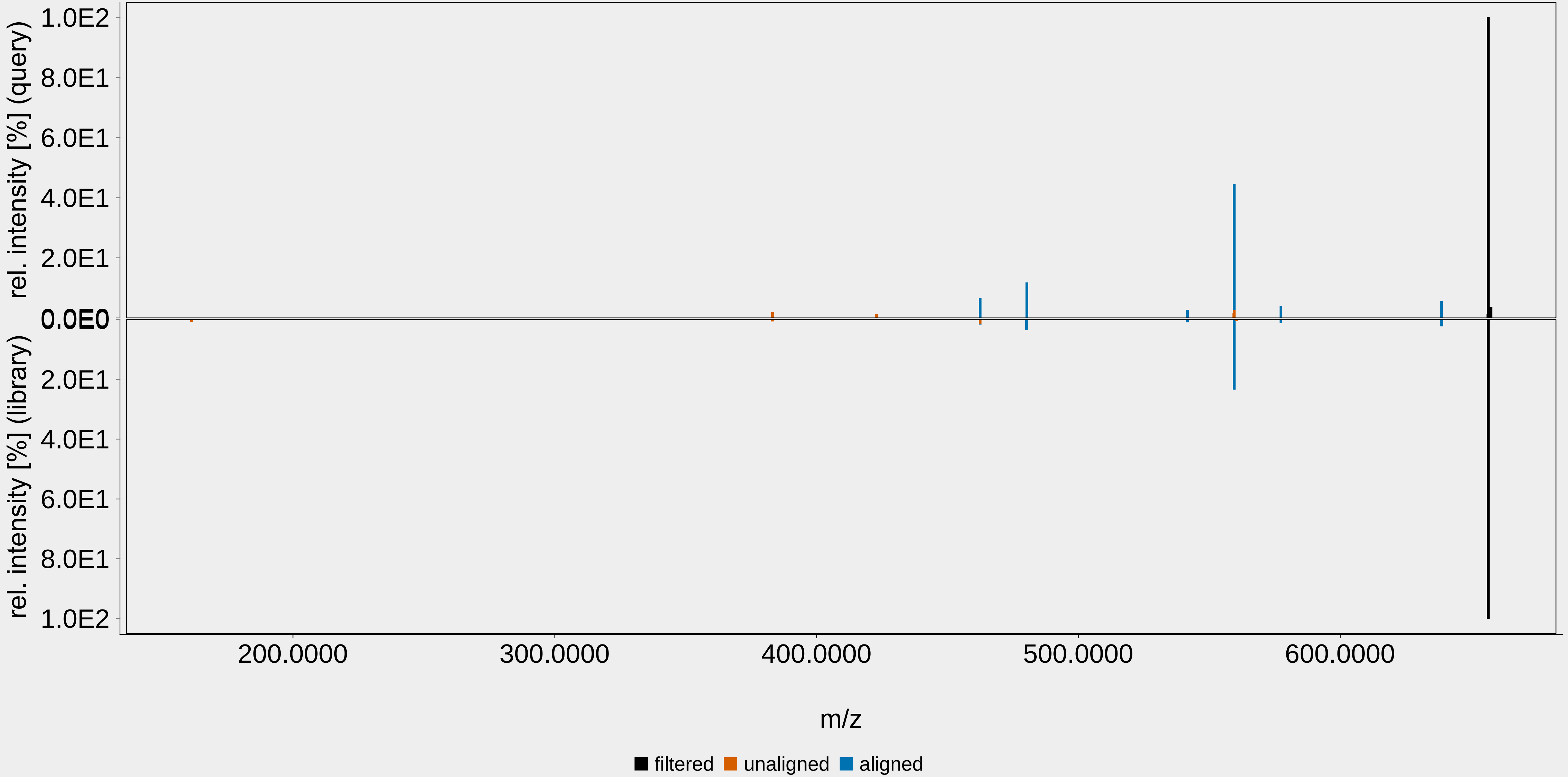

Compound information

Name: PHYTIC ACID

Exact mass: 0.0

Ion type: M-H

Precursor m/z: 658.854

Charge: 1

Polarity: negative

InChI:

SMILES:

Instrument information

Instrument type: qTof

Ion source: ESI

Ms level: 2

Acquisition: isolated

Database links

Other information

Principal investigator: MoNA

Data collector: MoNA:MoNA034158

Entry ID: CCMSLIB00006685256

PA 18:2_22:6 **0.859**

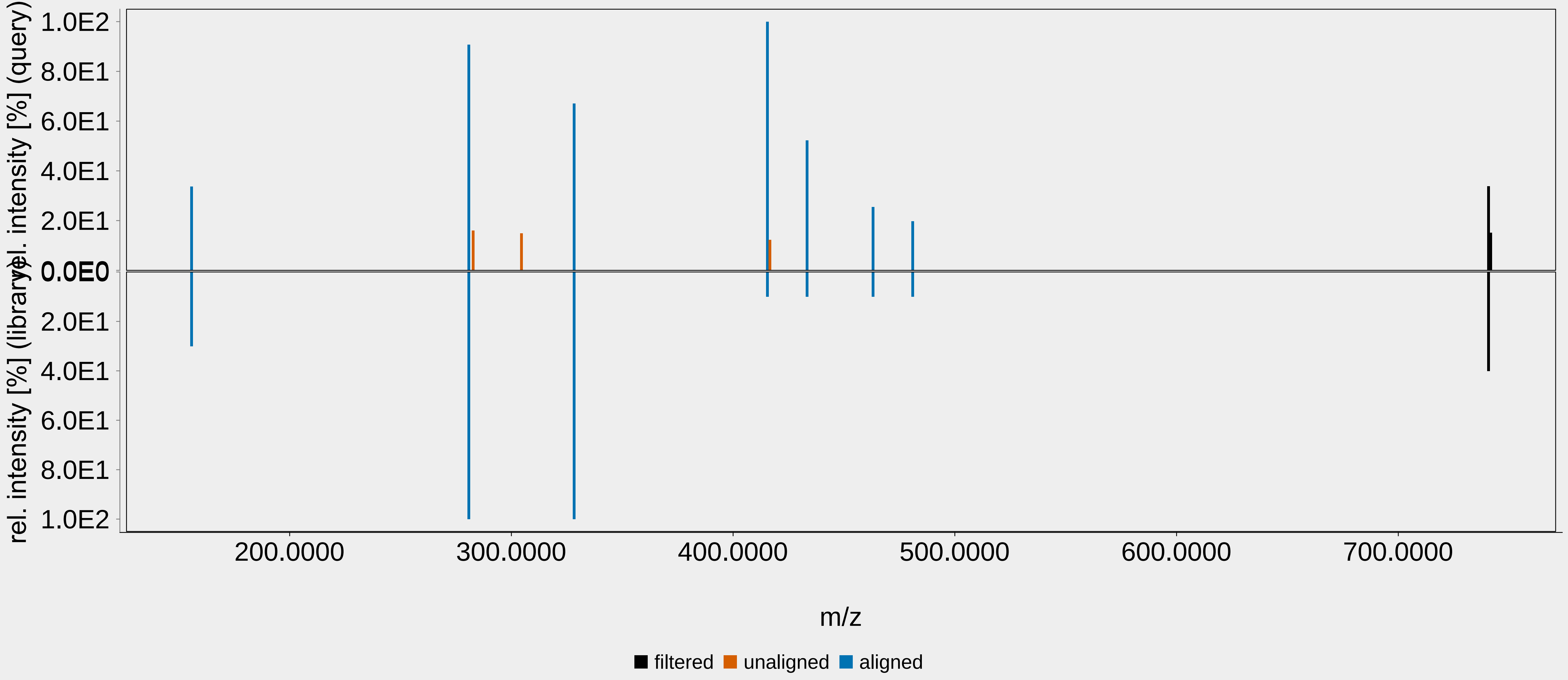

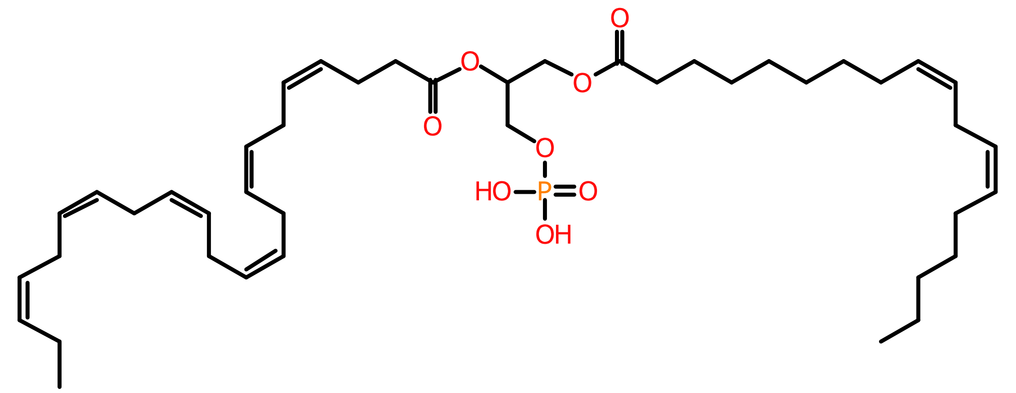

Compound information

Name: PA 18:2_22:6

Formula: C43H69O8P

Ion type: [M-H]-

Precursor m/z: 743.46573

Ccs: 264.57

InChI key:

SMILES:

Num peaks: 8

Instrument information

Database links

Other information

PA 18:1_22:6 **0.796**

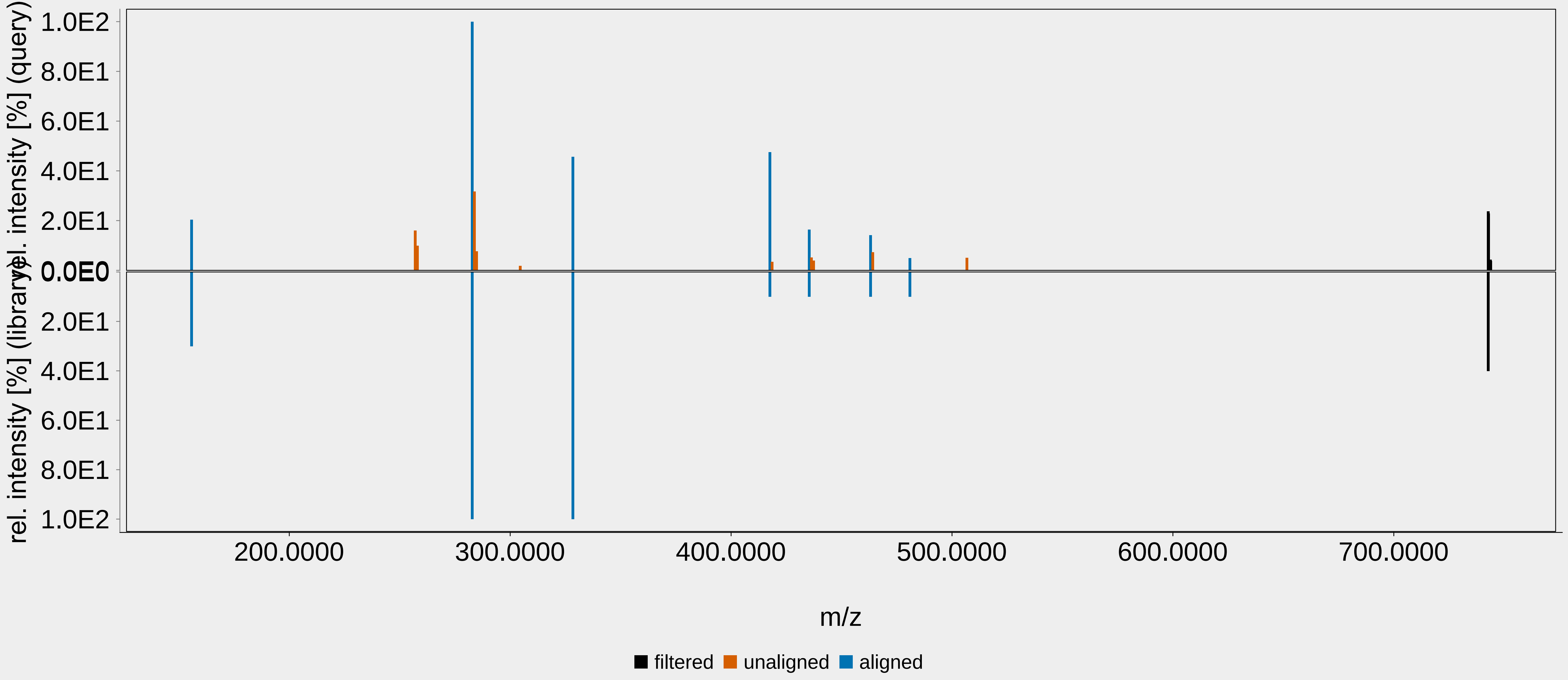

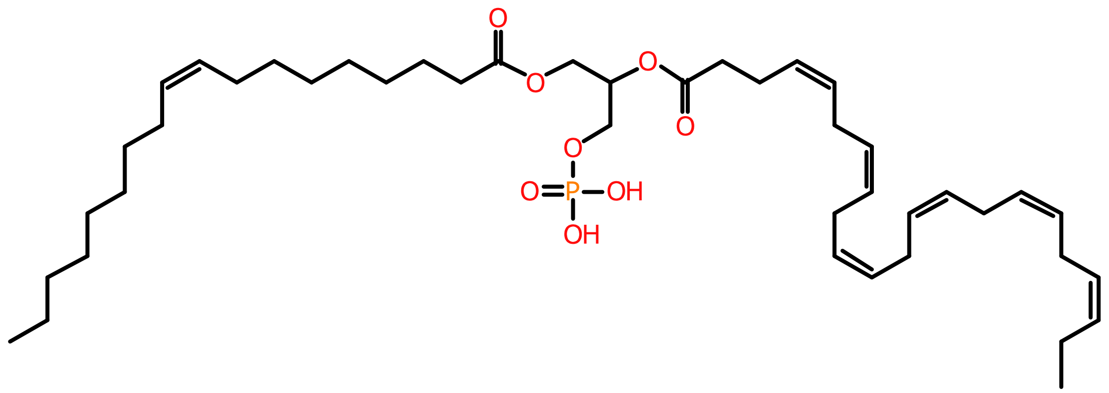

Compound information

Name: PA 18:1_22:6

Formula: C43H71O8P

Ion type: [M-H]-

Precursor m/z: 745.48138

Ccs: 264.25

InChI key:

SMILES:

Num peaks: 8

Instrument information

Database links

Other information

PE(18:0/22:6); [M-H]- C45H77N1O8P1

0.878

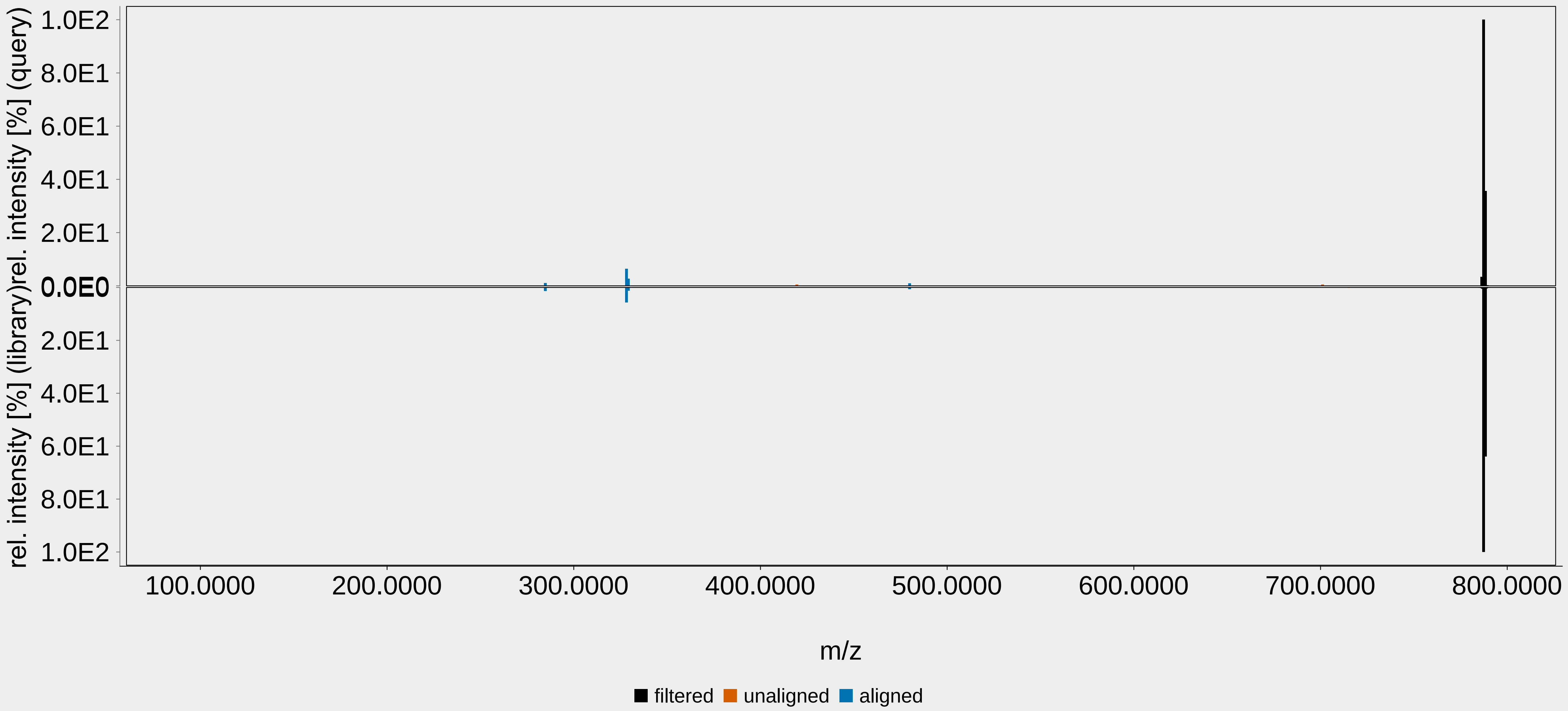

Compound information

Name: PE(18:0/22:6); [M-H]- C45H77N1O8P1

Exact mass: 0.0

Ion type: M-H

Precursor m/z: 790.539

Charge: 1

Polarity: Negative

Instrument information

Instrument type: HCD; Velos

Ion source: LC-ESI

Ms level: 2

Acquisition: Commercial

Database links

Other information

Principal investigator: Thomas Metz

Data collector: Thomas Metz

Entry ID: CCMSLIB00010085809

PE(18:0/22:6); [M-H]- C45H77N1O8P1 0.880

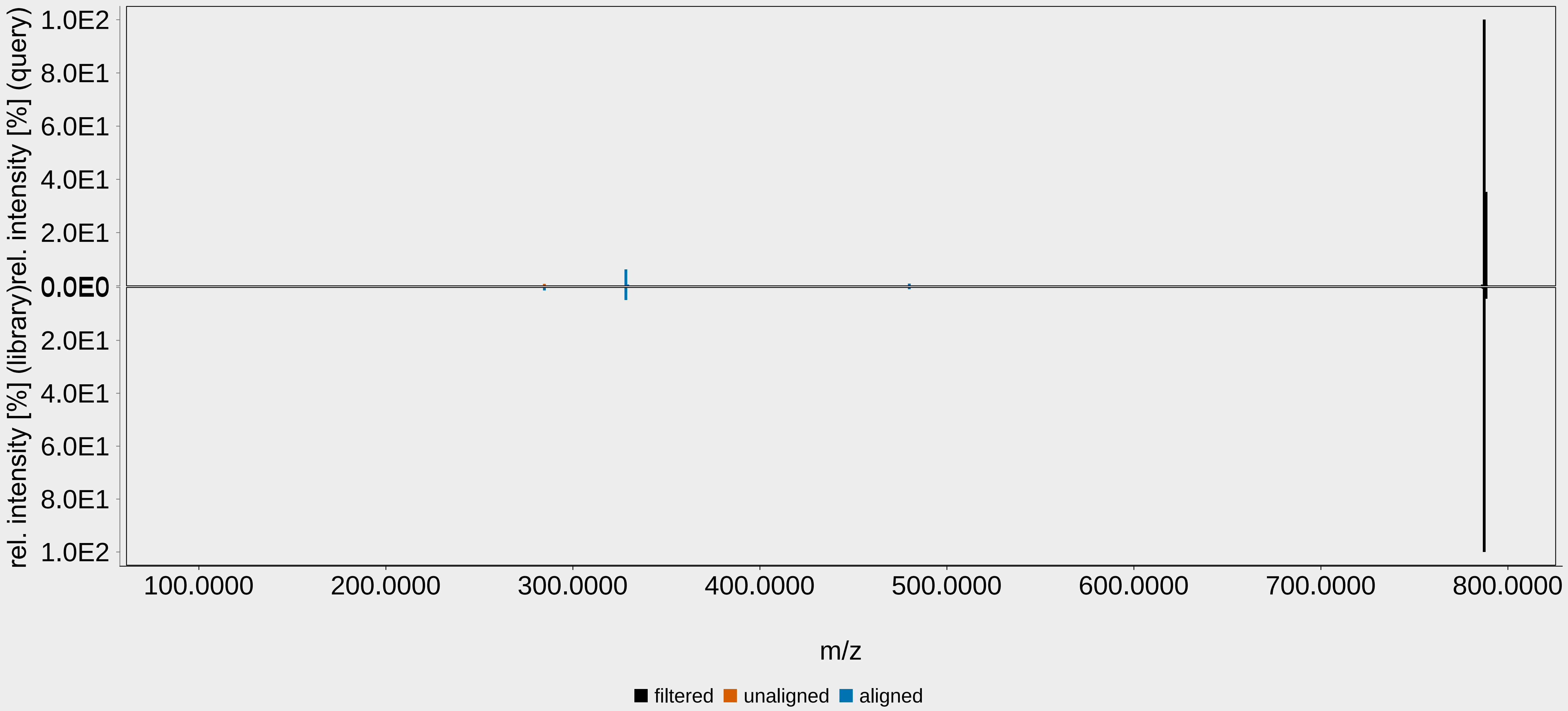

Compound information

Name: PE(18:0/22:6); [M-H]- C45H77N1O8P1

Exact mass: 0.0

Ion type: M-H

Precursor m/z: 790.539

Charge: 1

Polarity: Negative

Instrument information

Instrument type: HCD; Velos

Ion source: LC-ESI

Ms level: 2

Acquisition: Commercial

Database links

Other information

Principal investigator: Thomas Metz

Data collector: Thomas Metz

Entry ID: CCMSLIB00010073326

NADH - 30.0 eV

0.936

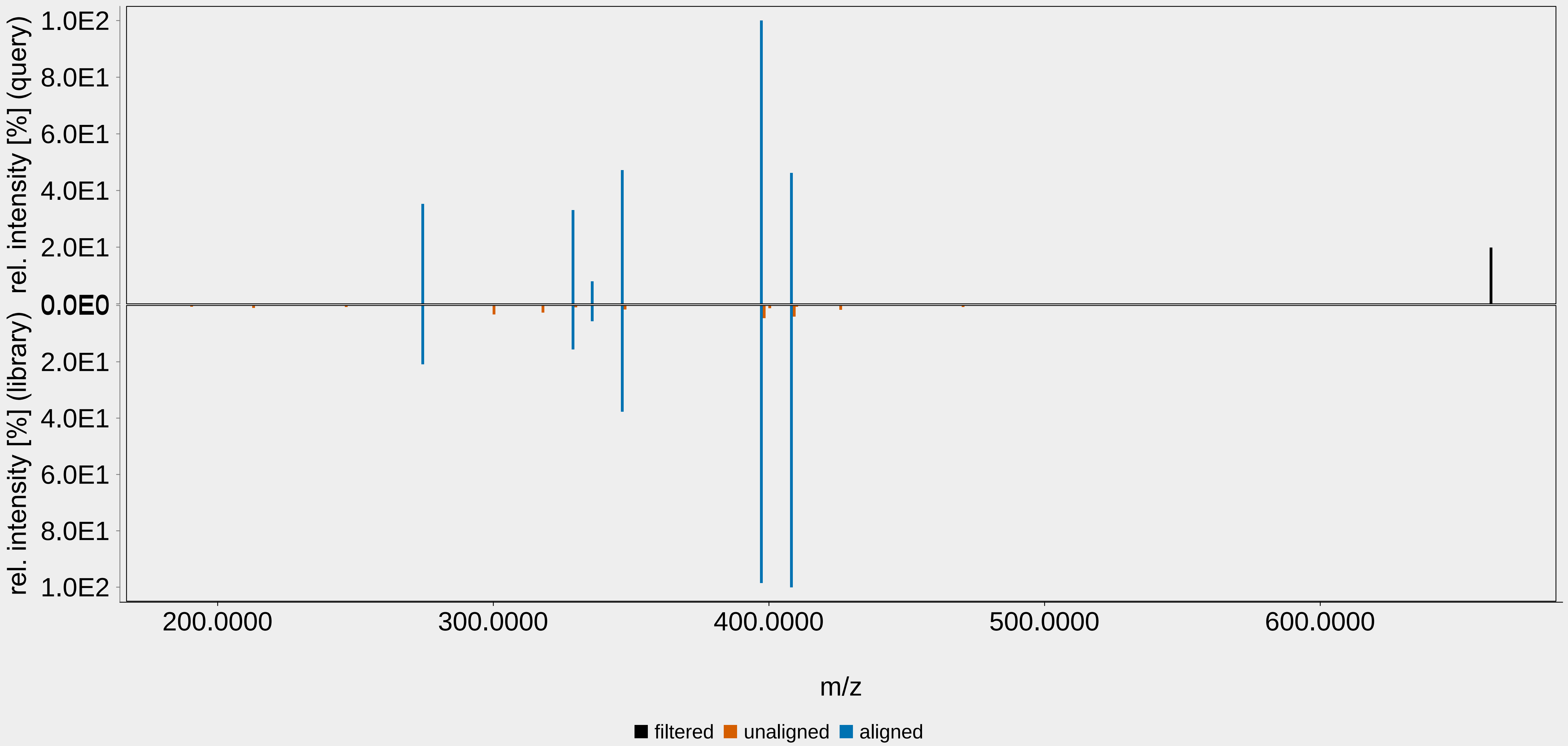

Compound information

Name: NADH - 30.0 eV

Exact mass: 0.0

Ion type: M-H

Precursor m/z: 664.118

Charge: 1

Polarity: Negative

SMILES:

Instrument information

Instrument type: Orbitrap

Ion source: ESI

Ms level: 2

Acquisition: Isolated

Database links

CAS:

Other information

Principal investigator: PI

Data collector: Data Collector

Entry ID: CCMSLIB00006123962

NADH - 30.0 eV

0.944

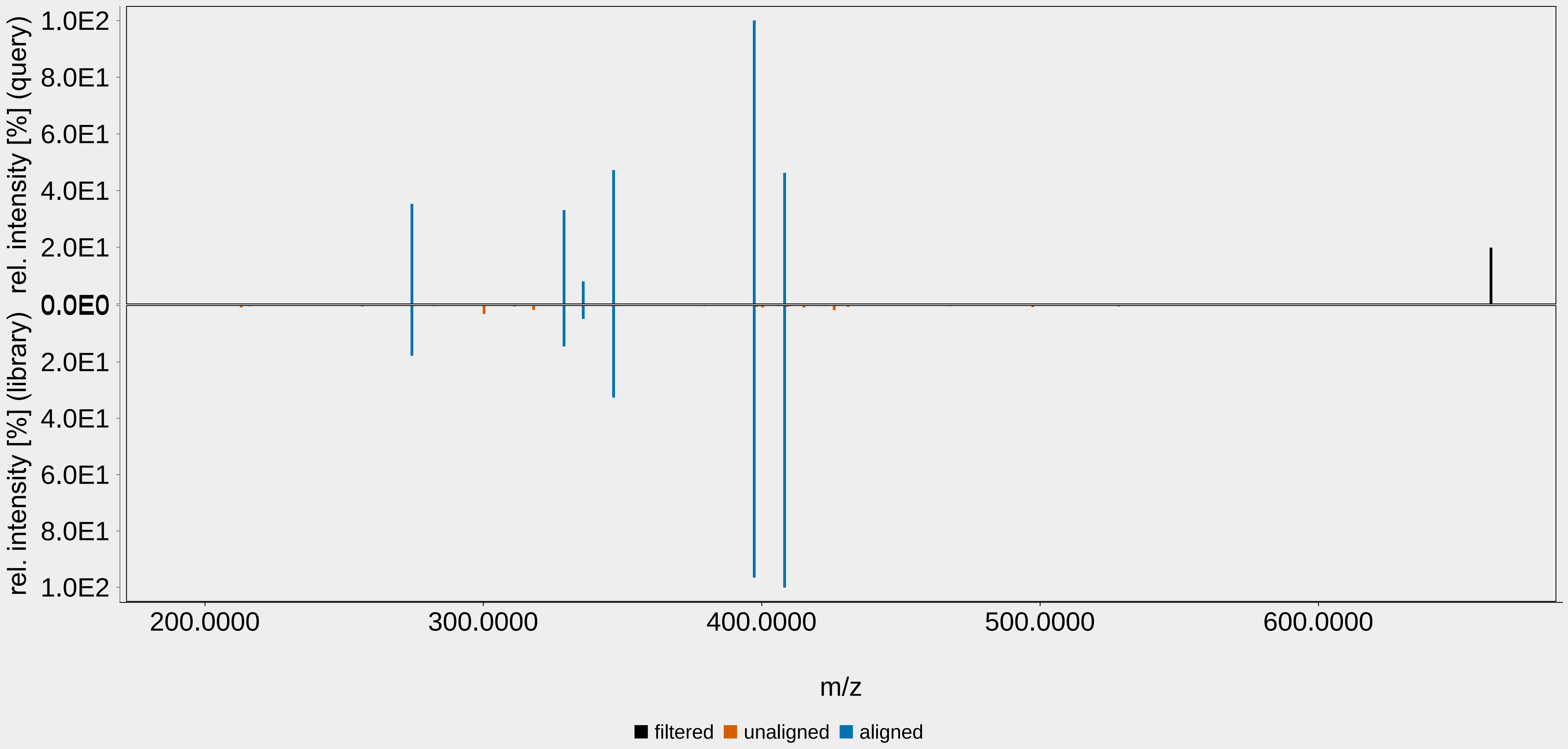

Compound information

Name: NADH - 30.0 eV

Exact mass: 0.0

Ion type: M-H

Precursor m/z: 664.117

Charge: 1

Polarity: Negative

SMILES:

Instrument information

Instrument type: Orbitrap

Ion source: ESI

Ms level: 2

Acquisition: Isolated

Database links

CAS:

Other information

Principal investigator: PI

Data collector: Data Collector

Entry ID: CCMSLIB00006123977

PI 40:6

0.845

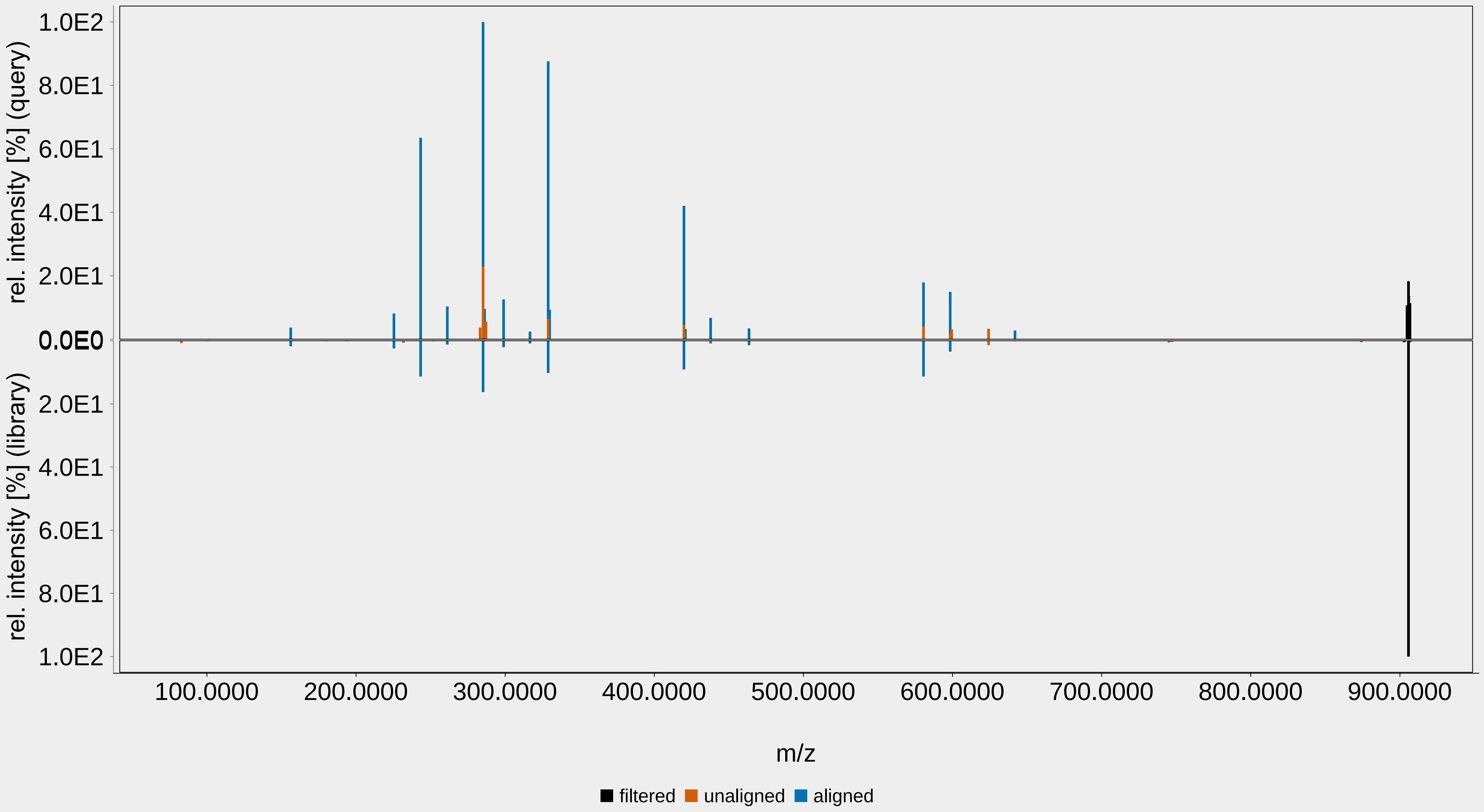

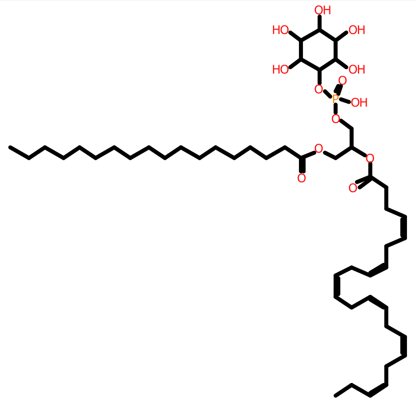

Compound information

Name: PI 40:6

Formula: C49H83O13P

Mol. weight: 911.1659999999999

Exact mass: 911.1659999999999

Ion type: [M-H]-

Precursor m/z: 909.5445

RT: 8.653857

Polarity: negative

InChI:

InChI key:

SMILES:

Instrument information

Instrument type: LC-ESI-QTOF

Instrument: AB Sciex TripleTOF 5600+ system

Ms level: MS2

Collision energy: 45V

Database links

Pubchem: 138144195

MoNA ID: LQB00699

Other information

Principal investigator: Kazutaka Ikeda, Hiroshi

Data collector: Kazutaka Ikeda, Hiroshi

PI 40:6

0.845

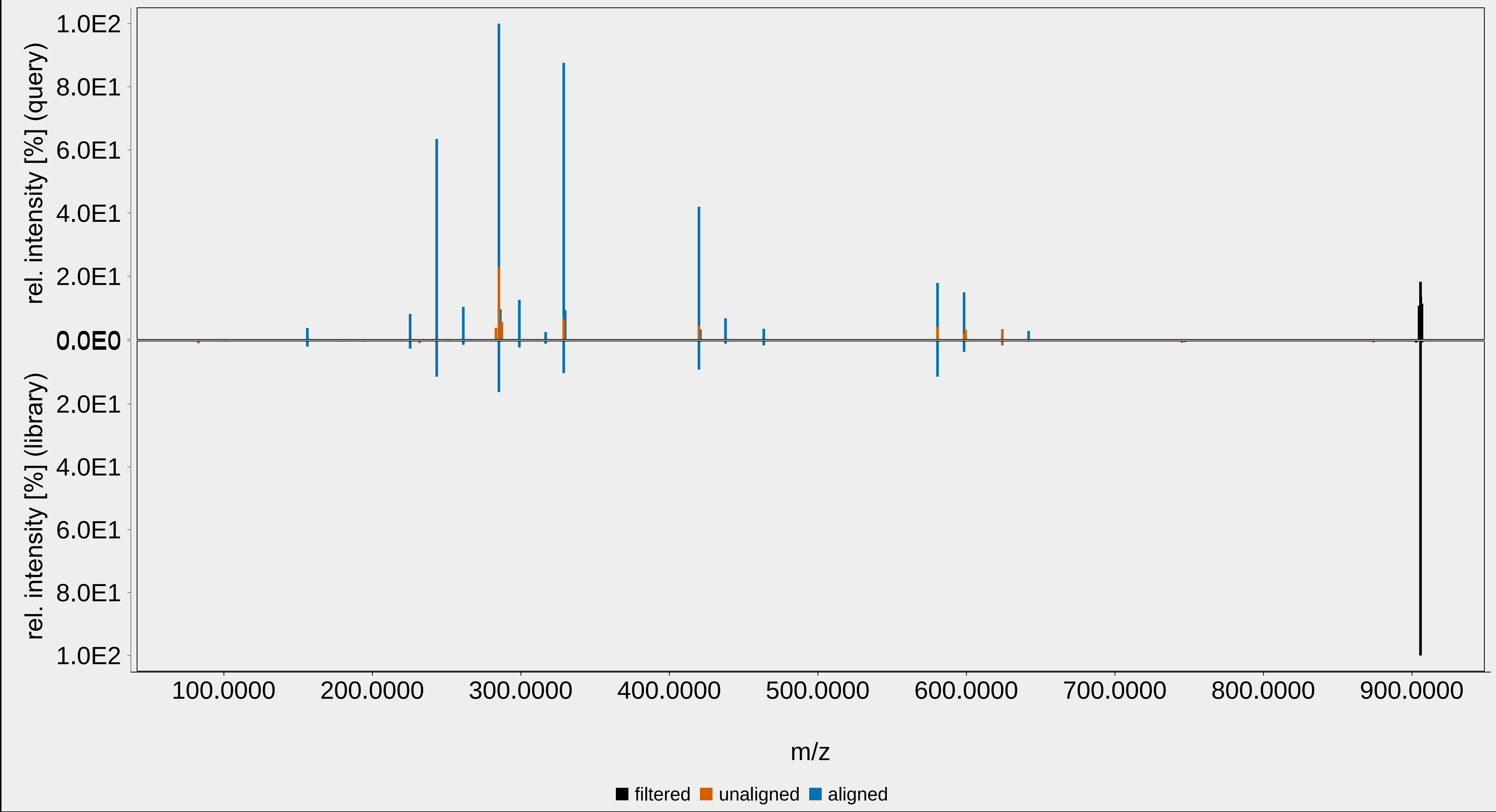

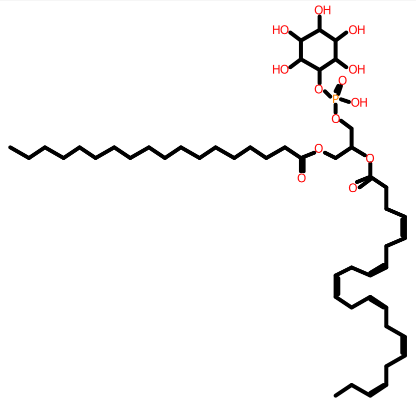

Compound information

Name: PI 40:6

Formula: C49H83O13P

Mol. weight: 911.1659999999999

Exact mass: 911.1659999999999

Ion type: [M-H]-

Precursor m/z: 909.5445

RT: 8.653857

Polarity: negative

InChI:

InChI key:

SMILES:

Instrument information

Instrument type: LC-ESI-QTOF

Instrument: AB Sciex TripleTOF 5600+ system

Ms level: MS2

Collision energy: 45V

Database links

Pubchem: 138144195

MoNA ID: LQB00313

Other information

Principal investigator: Kazutaka Ikeda, Hiroshi

Data collector: Kazutaka Ikeda, Hiroshi

PE(P-18:0/22:6); [M-H]- C45H77N1O7P1

0.852

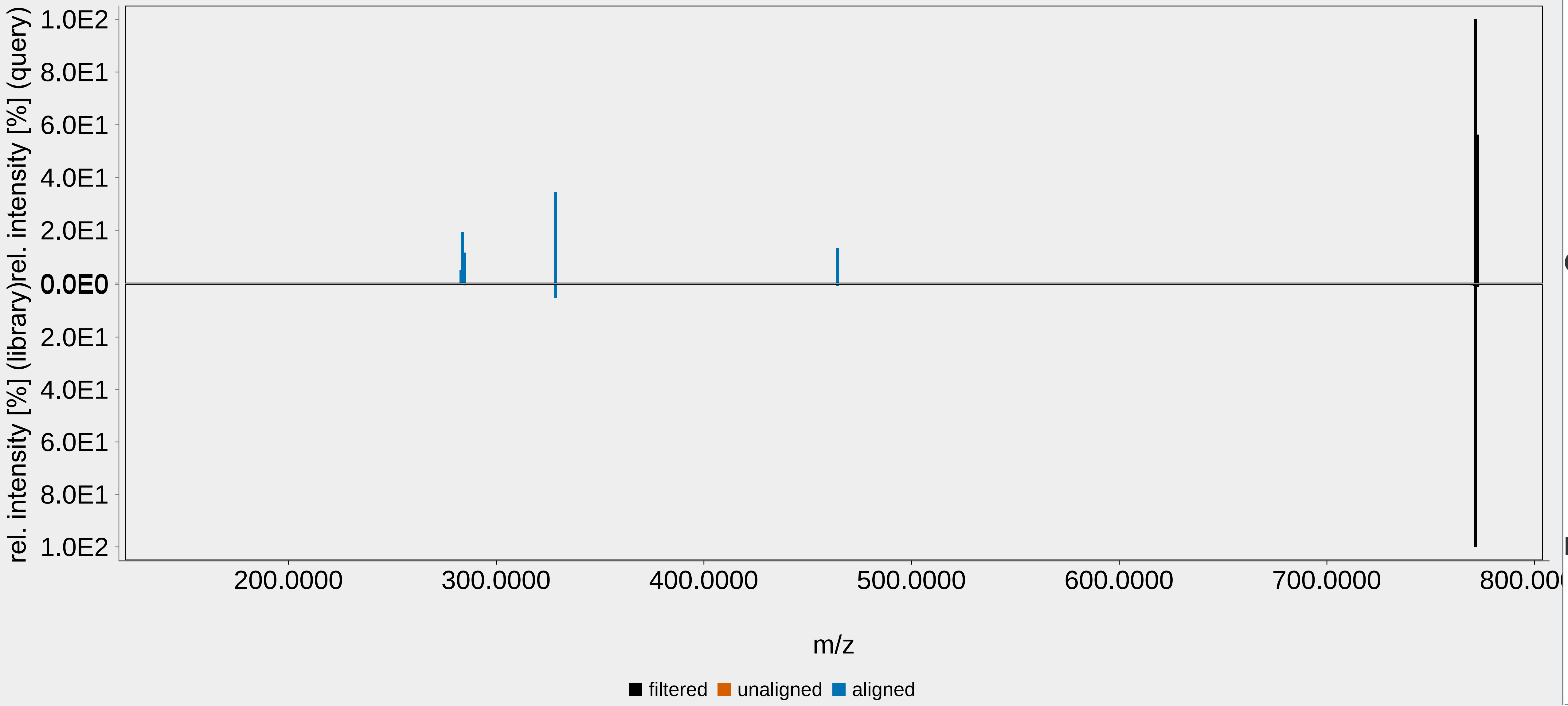

Compound information

Name: PE(P-18:0/22:6); [M-H]-

Exact mass: 0.0

Ion type: M-H

Precursor m/z: 774.544

Charge: 1

Polarity: Negative

Instrument information

Instrument type: HCD; Velos

Ion source: LC-ESI

Ms level: 2

Acquisition: Commercial

Database links

Other information

Principal investigator: Thomas Metz

Data collector: Thomas Metz

Entry ID: CCMSLIB00010087922

PE(P-18:0/22:6); [M-H]- C45H77N1O7P1

0.860

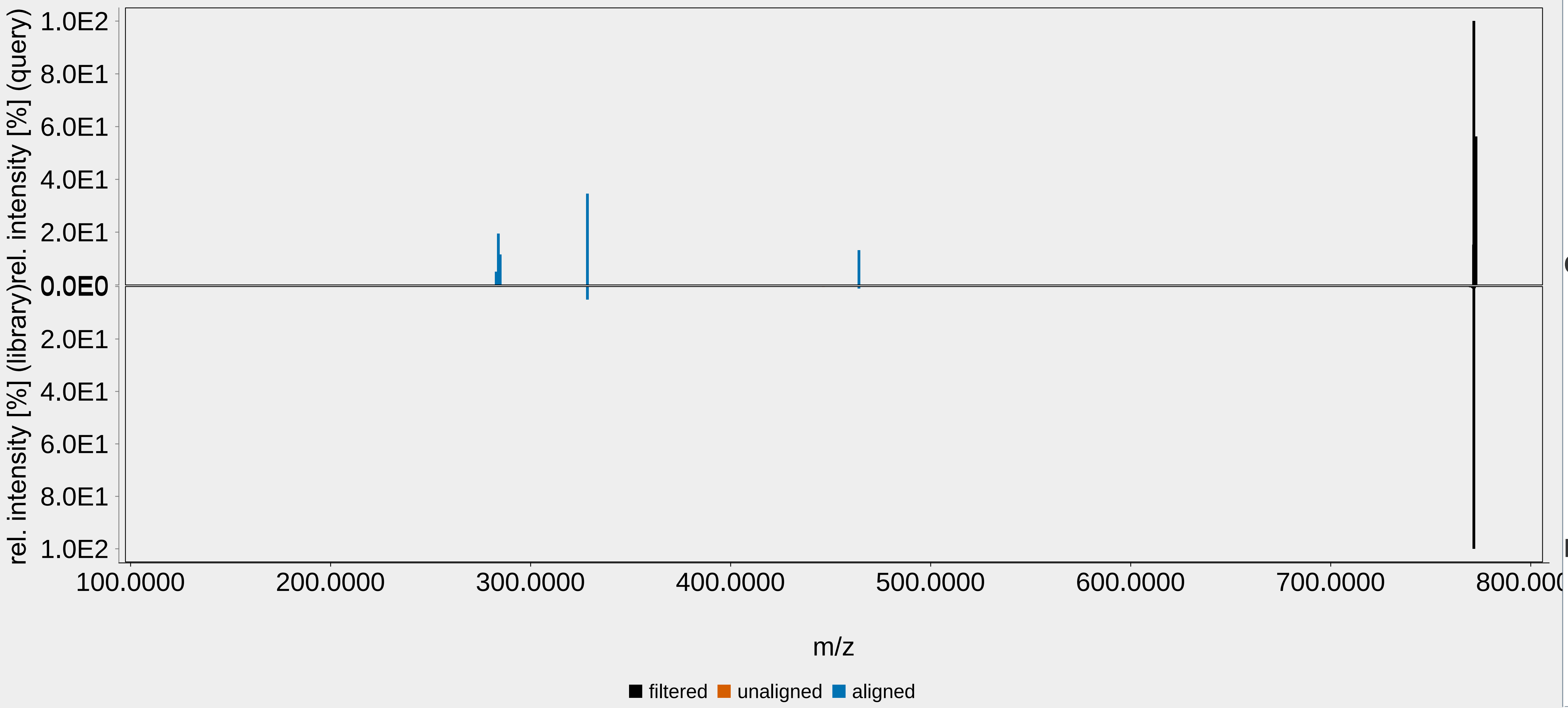

Compound information

Name: PE(P-18:0/22:6); [M-H]-

Exact mass: 0.0

Ion type: M-H

Precursor m/z: 774.544

Charge: 1

Polarity: Negative

Instrument information

Instrument type: HCD; Velos

Ion source: LC-ESI

Ms level: 2

Acquisition: Commercial

Database links

Other information

Principal investigator: Thomas Metz

Data collector: Thomas Metz

Entry ID: CCMSLIB00010088413

PA 16:0_22:6

0.729

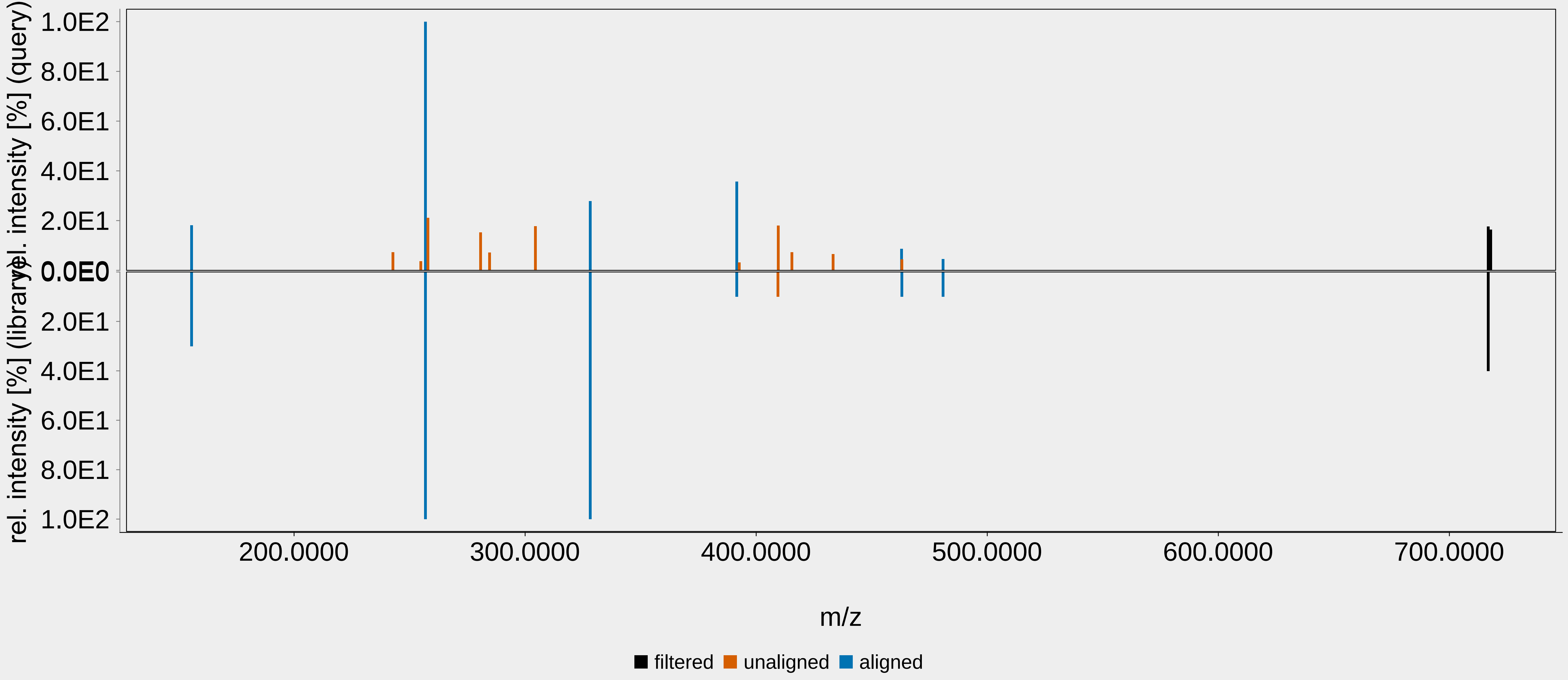

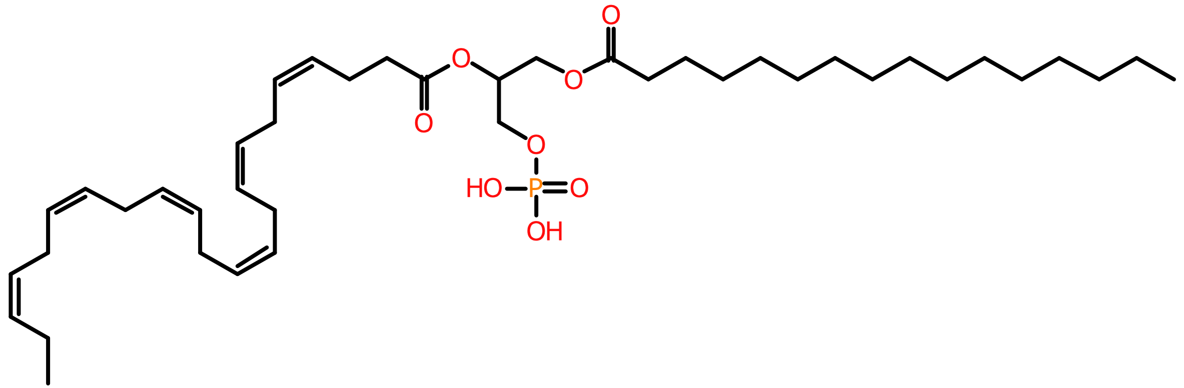

Compound information

Name: PA 16:0_22:6

Formula: C41H69O8P

Ion type: [M-H]-

Precursor m/z: 719.46573

Ccs: 257.26

InChI key:

SMILES:

Num peaks: 8

Instrument information

Database links

Other information

PG(22:6/22:6); [M-H]- C50H74O10P1 **0.709**

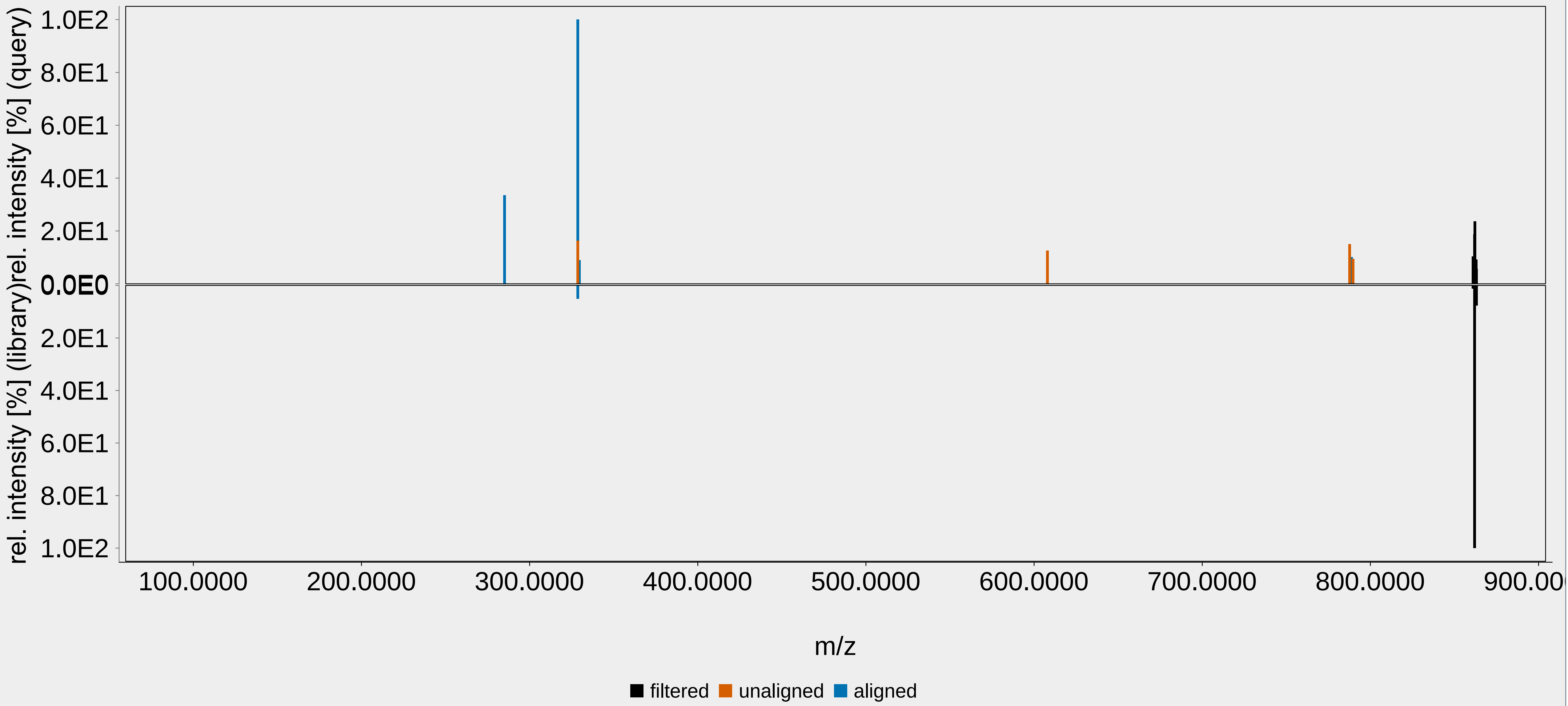

Compound information

Name: PG(22:6/22:6); [M-H]- C50H74O10P1

Exact mass: 0.0

Ion type: M-H

Precursor m/z: 865.502

Charge: 1

Polarity: Negative

Instrument information

Instrument type: HCD; Velos

Ion source: LC-ESI

Ms level: 2

Acquisition: Commercial

Database links

Other information

Principal investigator: Thomas Metz

Data collector: Thomas Metz

Entry ID: CCMSLIB00010083995

PG(22:6/22:6); [M-H]- C50H74O10P1 **0.726**

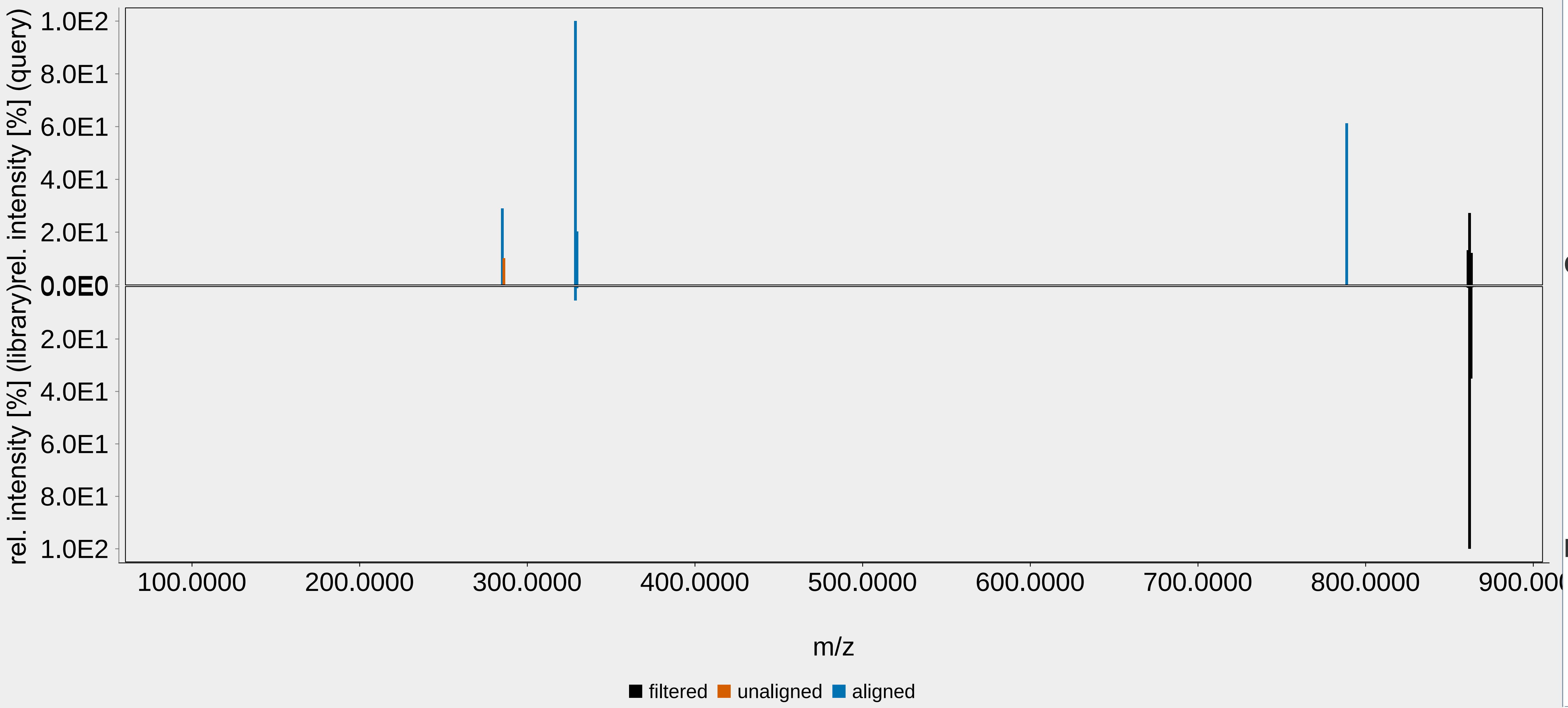

Compound information

Name: PG(22:6/22:6); [M-H]- C50H74O10P1

Exact mass: 0.0

Ion type: M-H

Precursor m/z: 865.502

Charge: 1

Polarity: Negative

Instrument information

Instrument type: HCD; Velos

Ion source: LC-ESI

Ms level: 2

Acquisition: Commercial

Database links

Other information

Principal investigator: Thomas Metz

Data collector: Thomas Metz

Entry ID: CCMSLIB00010082177

PI 40:4

0.787

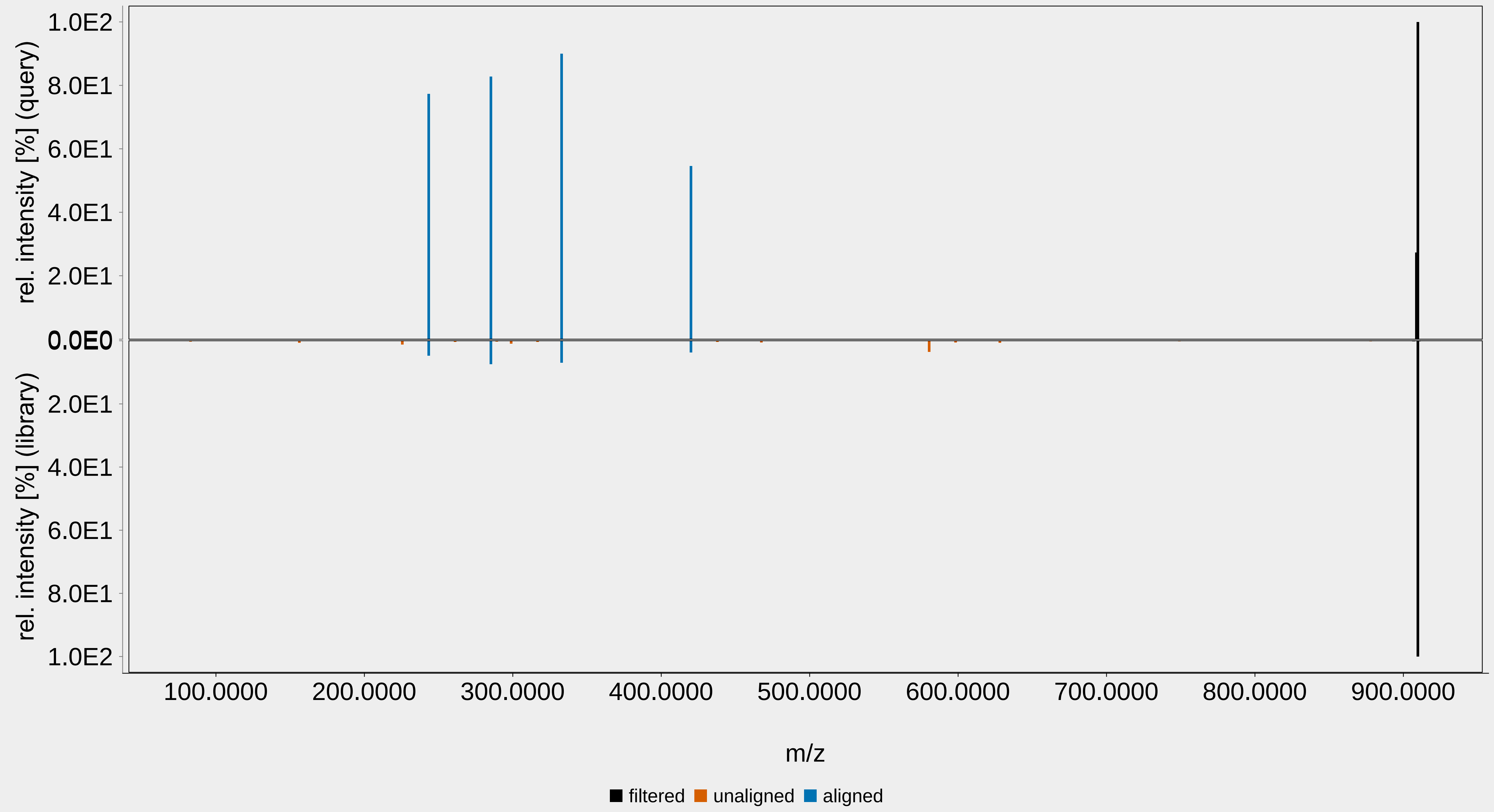

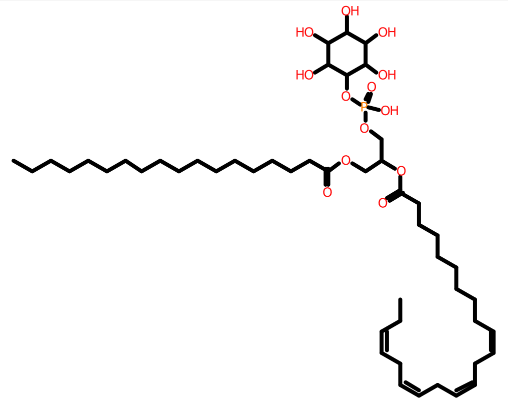

Compound information

Name: PI 40:4

Formula: C49H87O13P

Mol. weight: 915.198

Exact mass: 915.198

Ion type: [M-H]-

Precursor m/z: 913.5835

RT: 9.142293

Polarity: negative

InChI:

InChI key: VTYLJIRGLIZEIC-PYQGTGCGSA-N

SMILES:

Instrument information

Instrument type: LC-ESI-QTOF

Instrument: AB Sciex TripleTOF 5600+ system

Ms level: MS2

Collision energy: 45V

Database links

Pubchem: 138283546

MoNA ID: LQB00697

Other information

Principal investigator: Kazutaka Ikeda, Hiroshi

Data collector: Kazutaka Ikeda, Hiroshi

PI 40:4 **0.787**

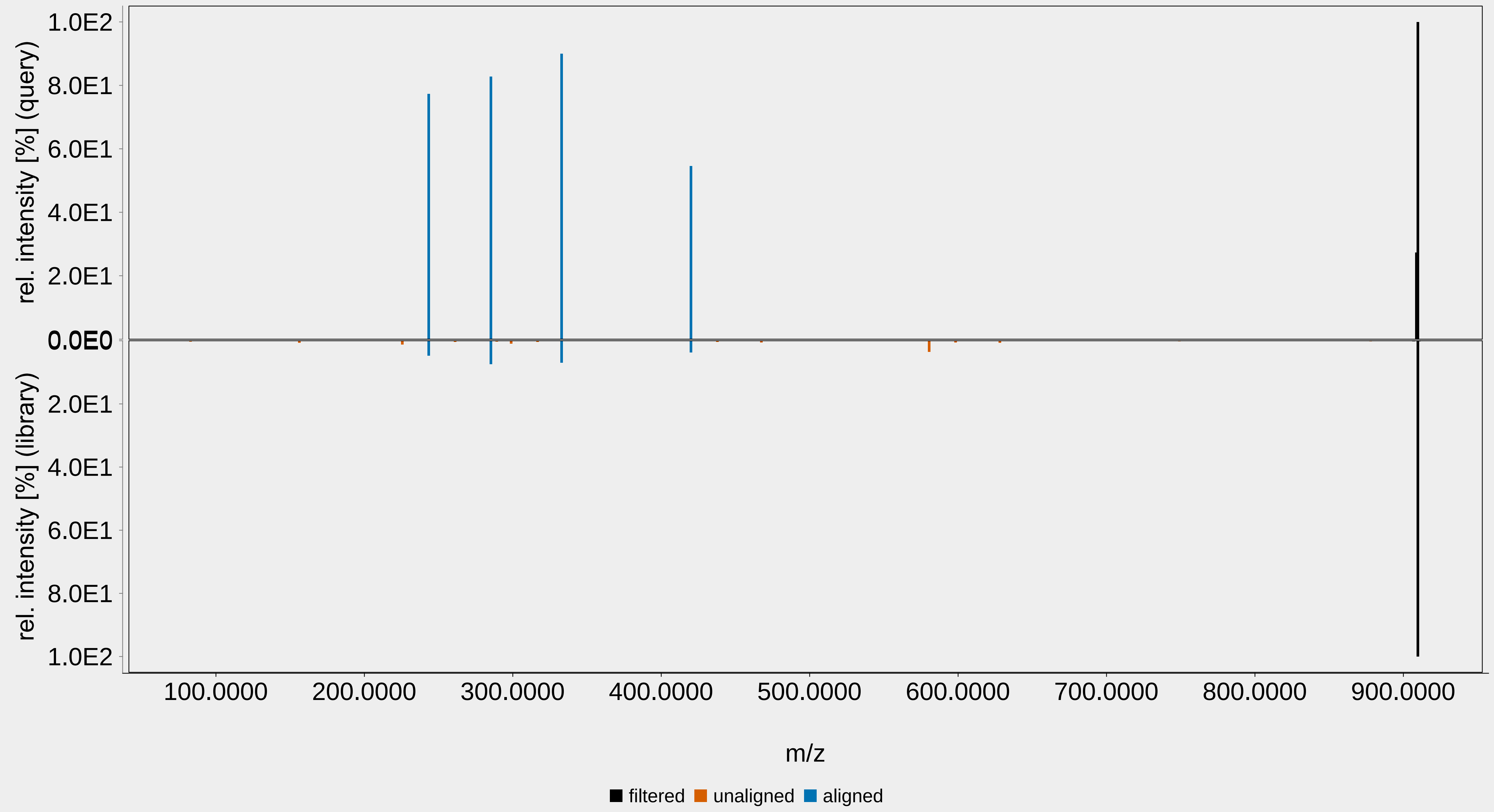

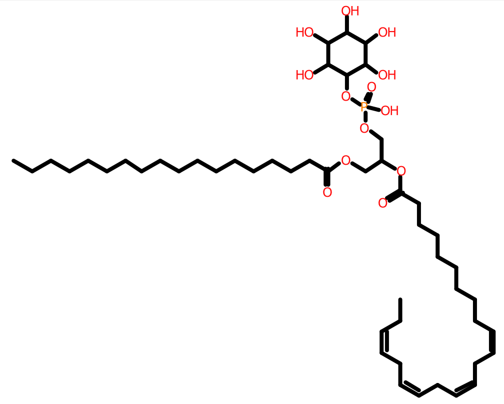

Compound information

Name: PI 40:4

Formula: C49H87O13P

Mol. weight: 915.198

Exact mass: 915.198

Ion type: [M-H]-

Precursor m/z: 913.5835

RT: 9.142293

Polarity: negative

InChI:

InChI key: VTYLJIRGLIZEIC-PYQGTGCGSA-N

SMILES:

Instrument information

Instrument type: LC-ESI-QTOF

Instrument: AB Sciex TripleTOF 5600+ system

Ms level: MS2

Collision energy: 45V

Database links

Pubchem: 138283546

MoNA ID: LQB00311

Other information

Principal investigator: Kazutaka Ikeda, Hiroshi

Data collector: Kazutaka Ikeda, Hiroshi

PE(18:1/20:4); [M-H]- C43H75N1O8P1

0.872

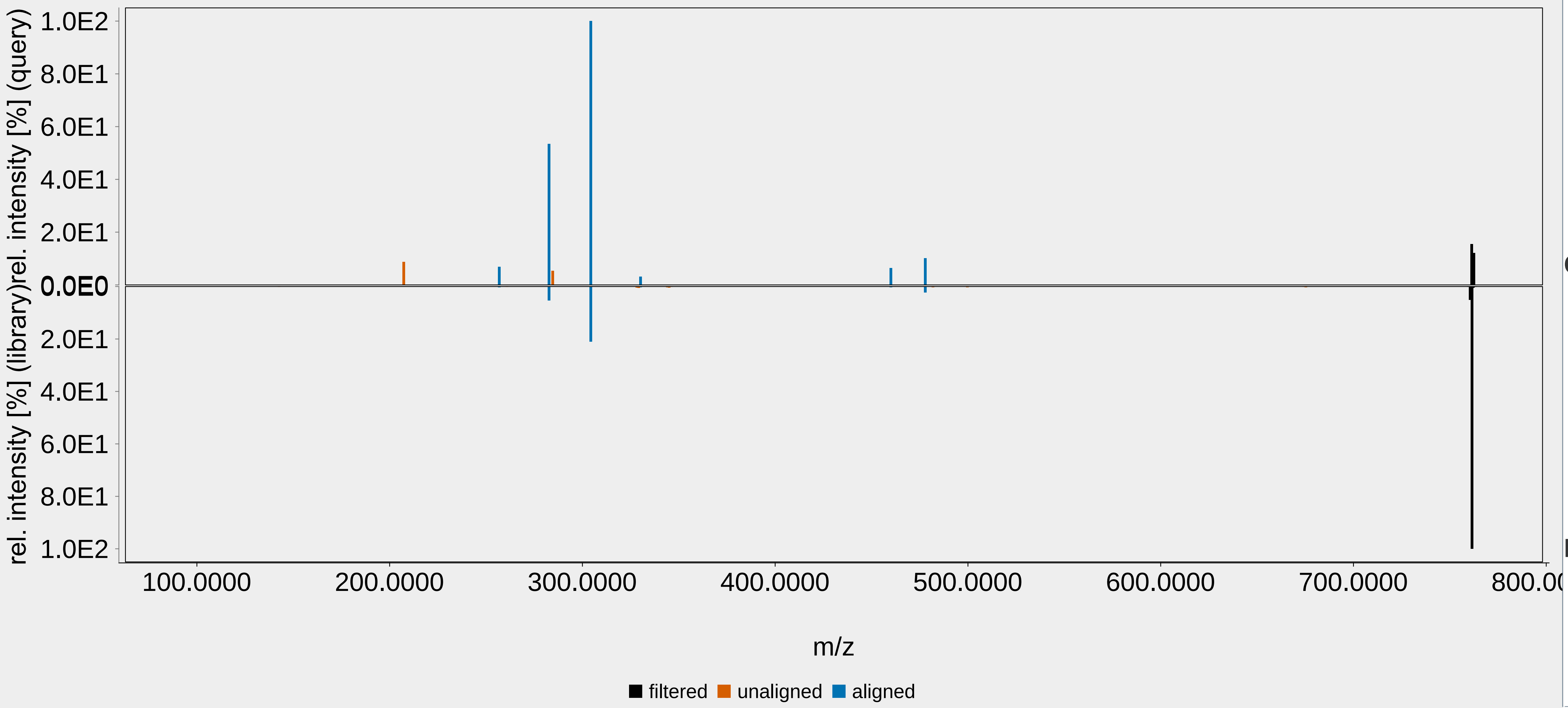

Compound information

Name: PE(18:1/20:4); [M-H]- C43H75N1O8P1

Exact mass: 0.0

Ion type: M-H

Precursor m/z: 764.523

Charge: 1

Polarity: Negative

Instrument information

Instrument type: HCD; Velos

Ion source: LC-ESI

Ms level: 2

Acquisition: Commercial

Database links

Other information

Principal investigator: Thomas Metz

Data collector: Thomas Metz

Entry ID: CCMSLIB00010063428

PE 18:1_20:4

0.880

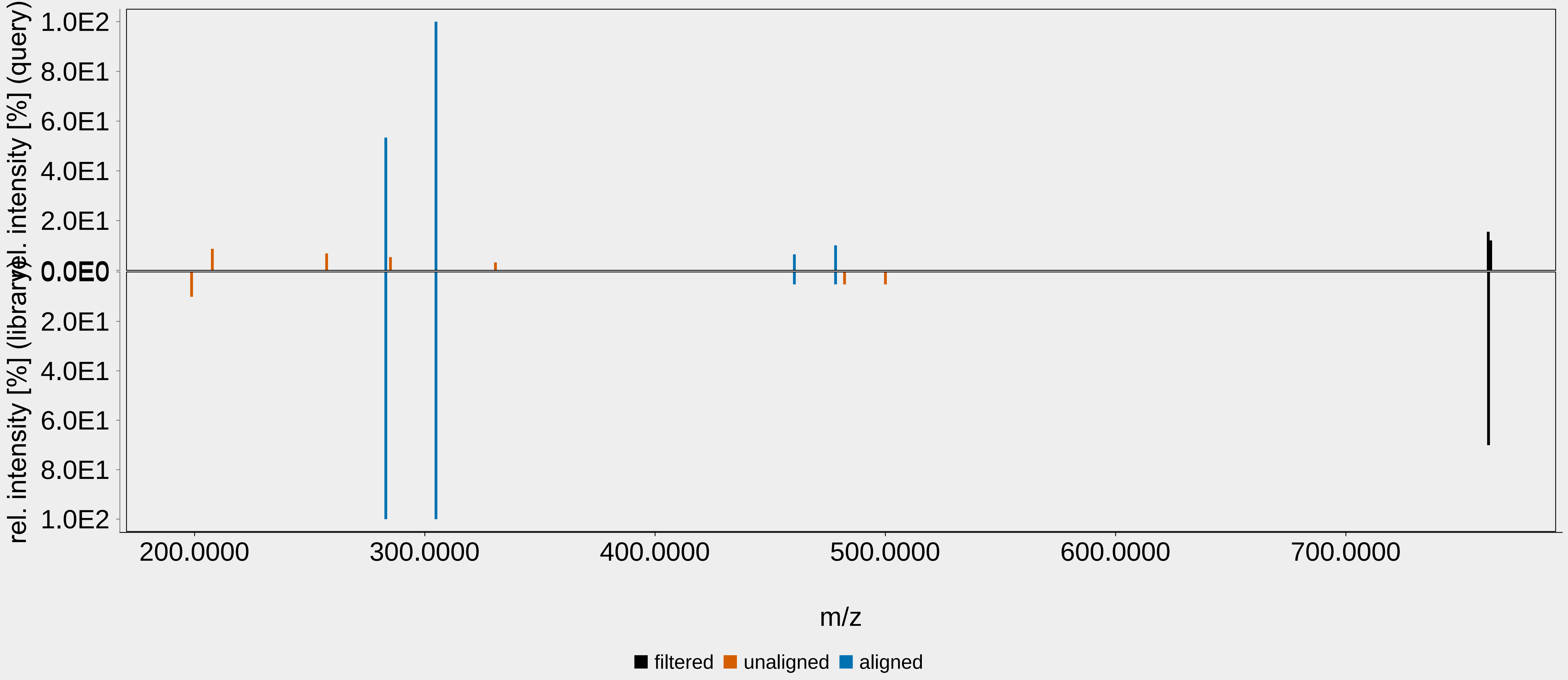

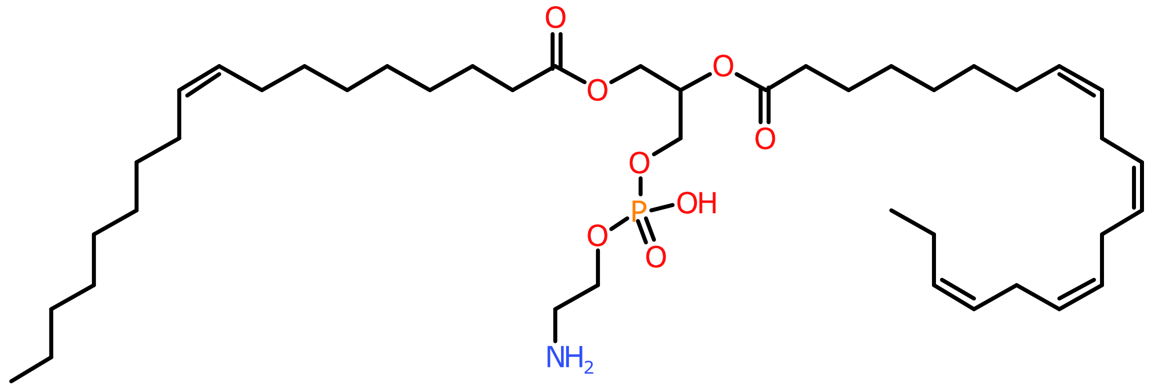

Compound information

Name: PE 18:1_20:4

Formula: C43H76NO8P

Ion type: [M-H]-

Precursor m/z: 764.52358

Ccs: 268.34

InChI key:

SMILES:

Num peaks: 8

Instrument information

Database links

Other information

PA 18:0_22:4

0.878

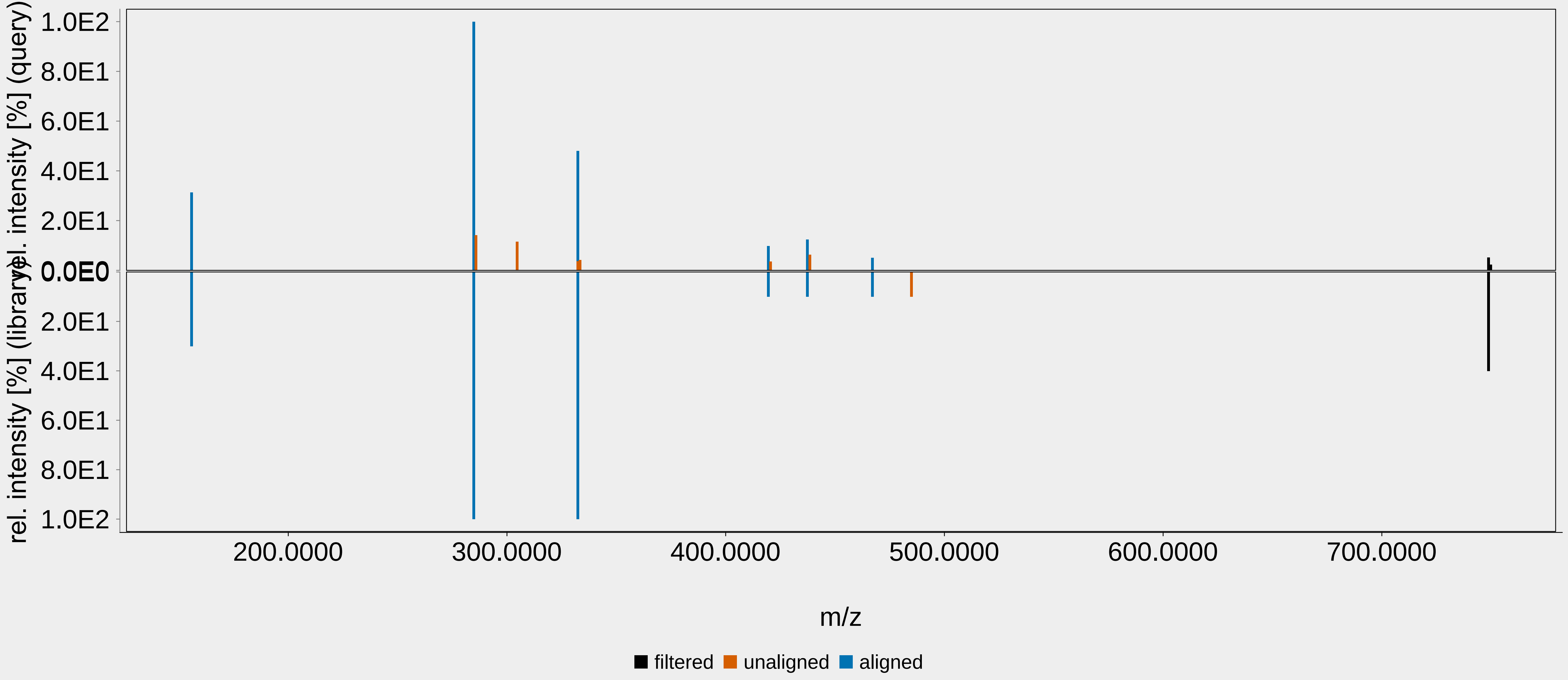

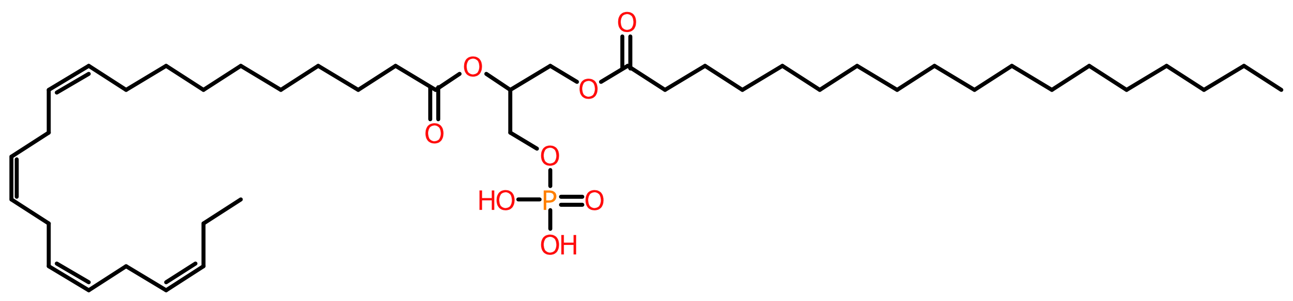

Compound information

Name: PA 18:0_22:4

Formula: C43H77O8P

Ion type: [M-H]-

Precursor m/z: 751.52833

Ccs: 267.82

InChI key:

SMILES:

Num peaks: 8

Instrument information

Database links

Other information

Arachidonic acid

0.714

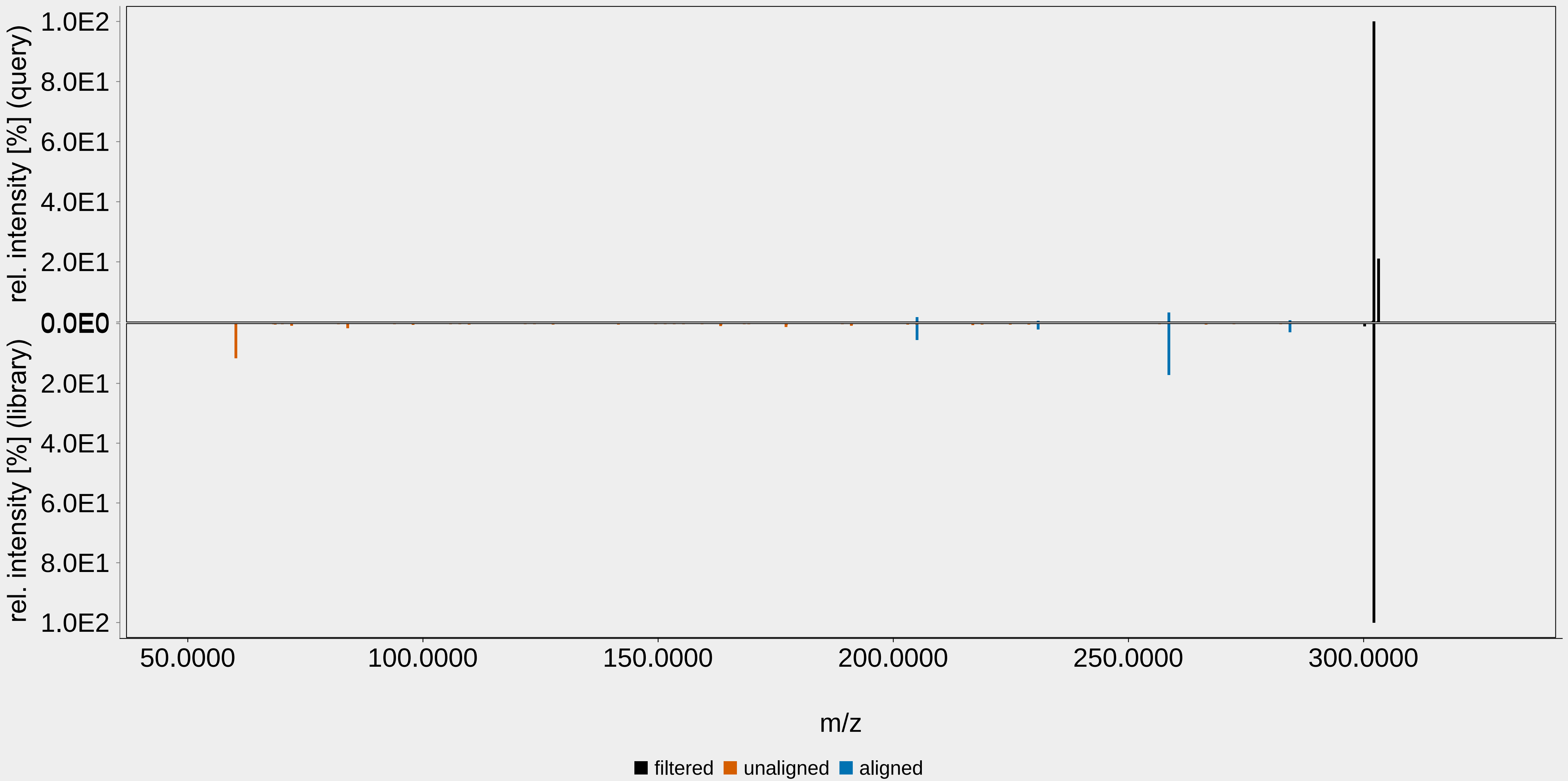

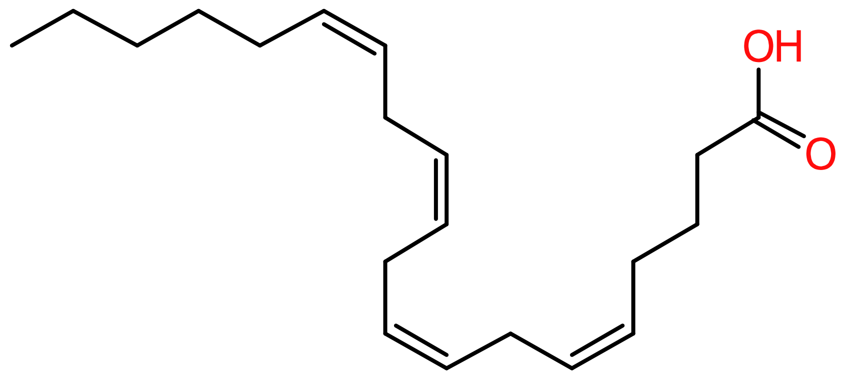

Compound information

Name: Arachidonic acid

Formula: C20H32O2

Ion type: [M-H]-

Precursor m/z: 303.232041974554

RT: 4.158056

Polarity: negative

InChI:

InChI key:

SMILES:

Instrument information

Instrument type: LC-ESI-QFT

Instrument: Thermo Q-Exactive Plus

Ms level: MS2

Collision energy: 30.0 eV

Database links

Other information

Principal investigator: Prasad Phapale

Data collector: Prasad Phapale

EicosatetraenoicAcid_Arachidonic

0.836

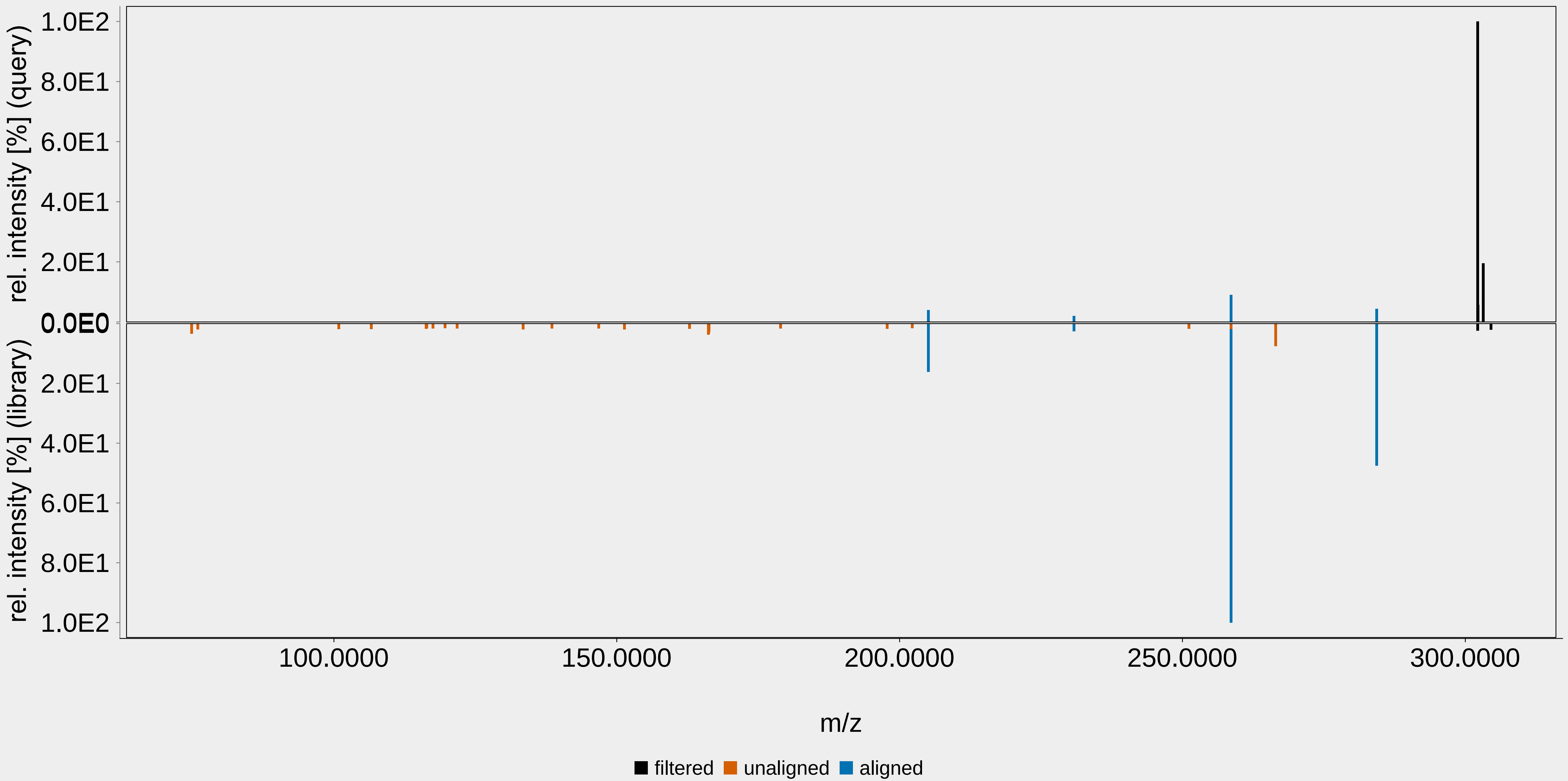

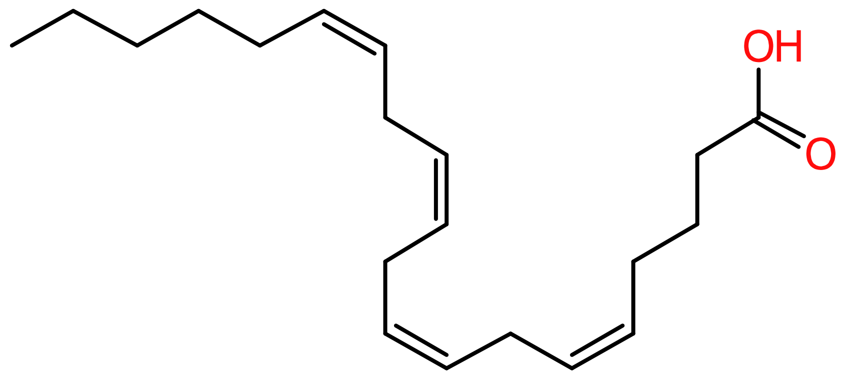

Compound information

Name: EicosatetraenoicAcid_Arachidonic

Exact mass: 304.24

Ion type: M-H

Precursor m/z: 303.233

Charge: -1

Polarity: Negative

InChI:

SMILES:

Instrument information

Instrument type: Orbitrap

Ion source: ESI

Ms level: 2

Acquisition: Commercial

Database links

Pubmed: 444899.0

CAS: 506-32-1

Other information

Principal investigator: Ulaszewska

Data collector: Marynka Ulaszewska

Entry ID: CCMSLIB00006676895

PA 16:1_20:4

0.877

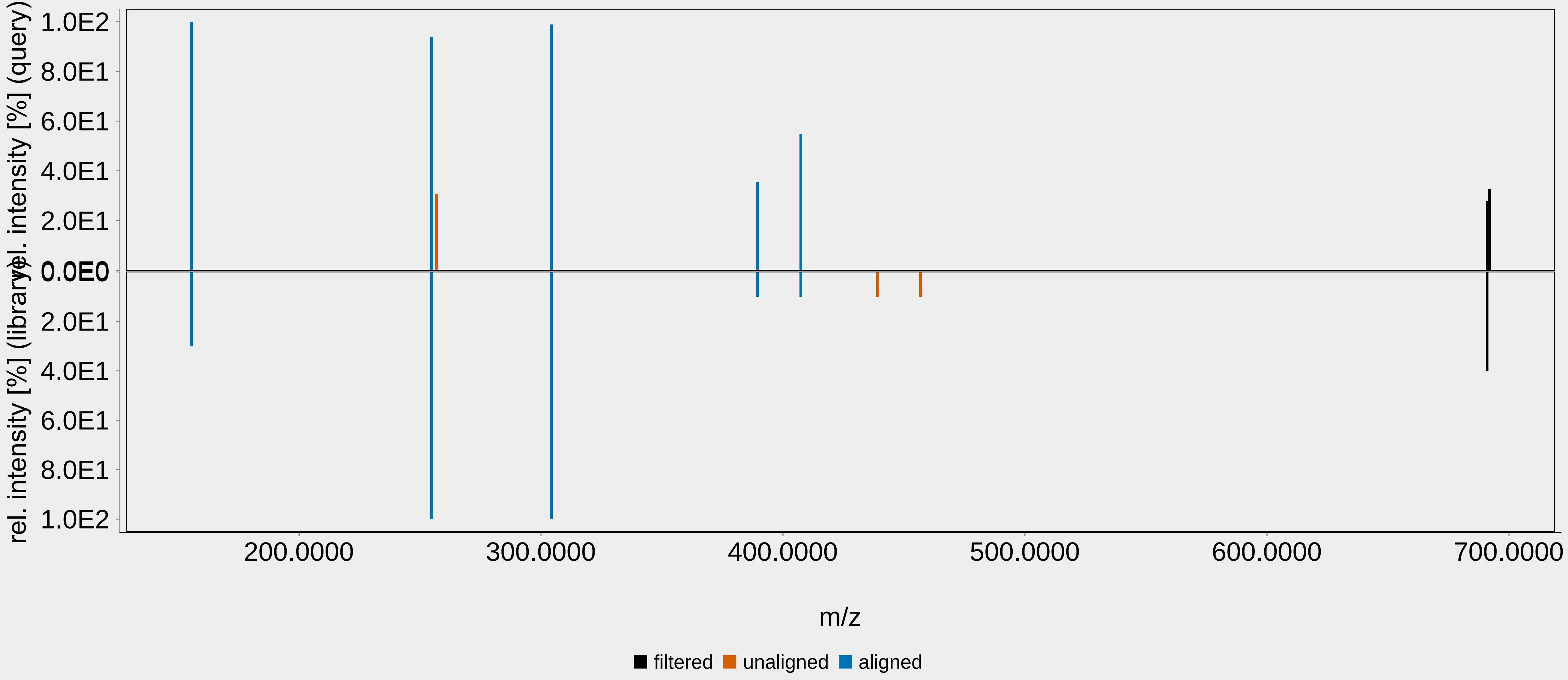

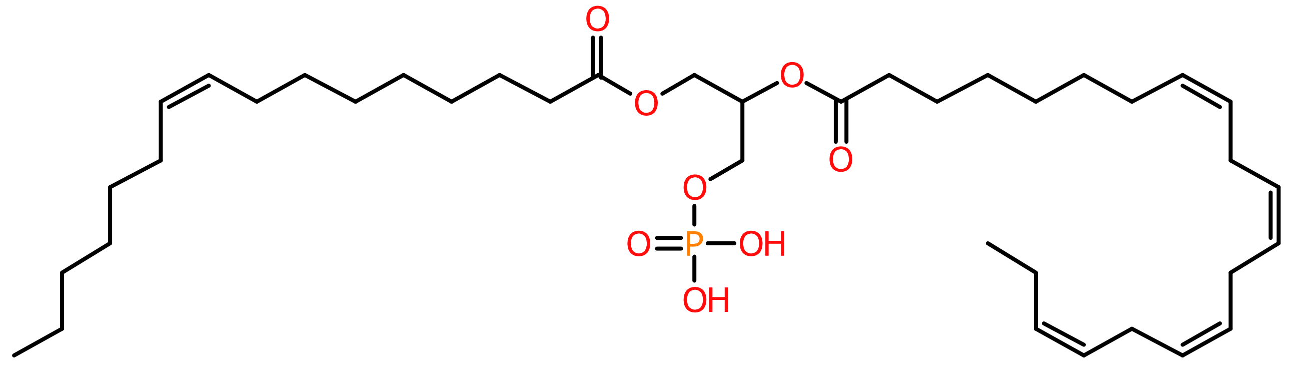

Compound information

Name: PA 16:1_20:4

Formula: C39H67O8P

Ion type: [M-H]-

Precursor m/z: 693.45008

Ccs: 253.91

InChI key:

SMILES:

Num peaks: 8

Instrument information

Database links

Other information

HYDROQUINIDINE

0.884

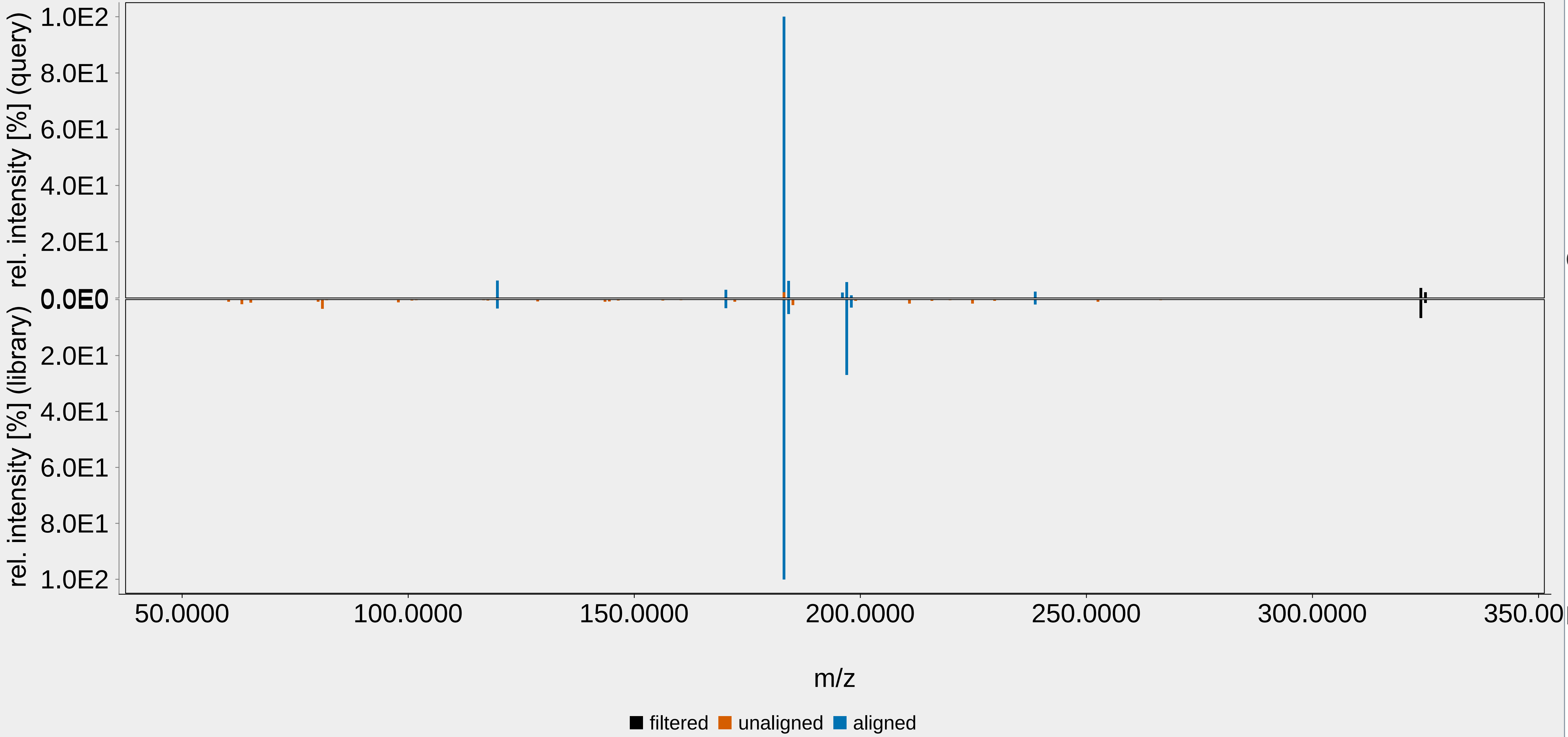

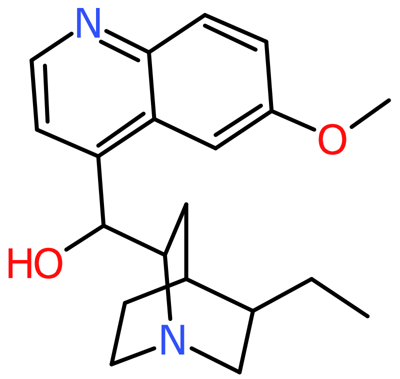

Compound information

Name: HYDROQUINIDINE

Formula: C20H26N2O2

Ion type: [M-H]-

Precursor m/z: 325.192138671875

Polarity: negative

InChI:

InChI key:

SMILES:

Instrument information

Instrument type: ESI-QFT

Instrument: Thermo Q Exactive HF

Ms level: MS2

Collision energy: 65HCD

Database links

Other information

Principal investigator: Arpana Vaniya

Data collector: Arpana Vaniya

HYDROQUINIDINE

0.884

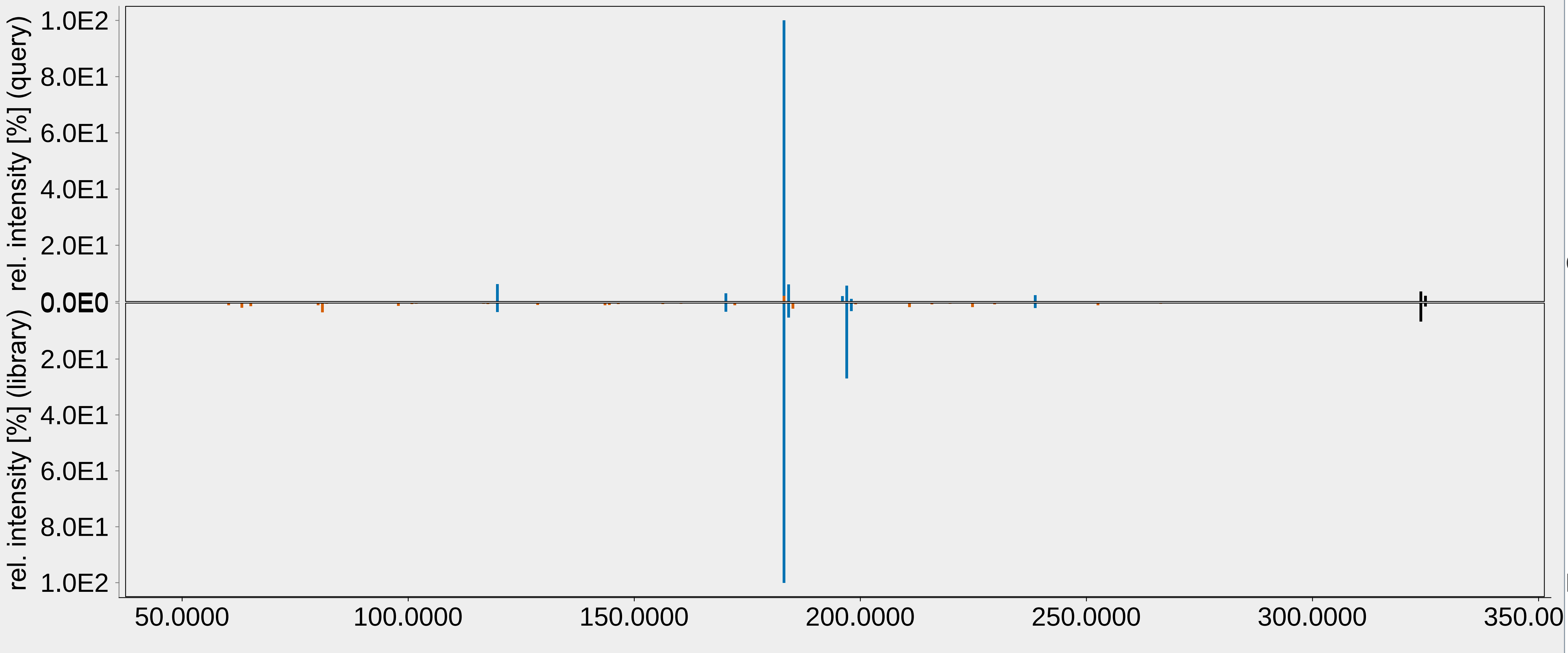

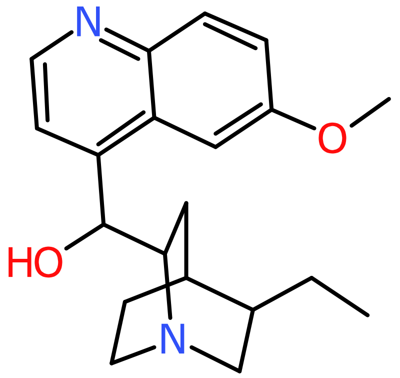

Compound information

Name: HYDROQUINIDINE

Exact mass: 0.0

Ion type: [M-H]-

Precursor m/z: 325.192

Charge: 1

Polarity: negative

InChI:

Instrument information

Instrument type: ESI-QFT

Ms level: 2

Acquisition: isolated

Database links

Other information

Principal investigator: MoNA

Data collector: MoNA:VF-NPL-QEHF003075

Entry ID: CCMSLIB00004694138

PE(18:0/20:3); [M-H]- C43H79N1O8P1 0.730

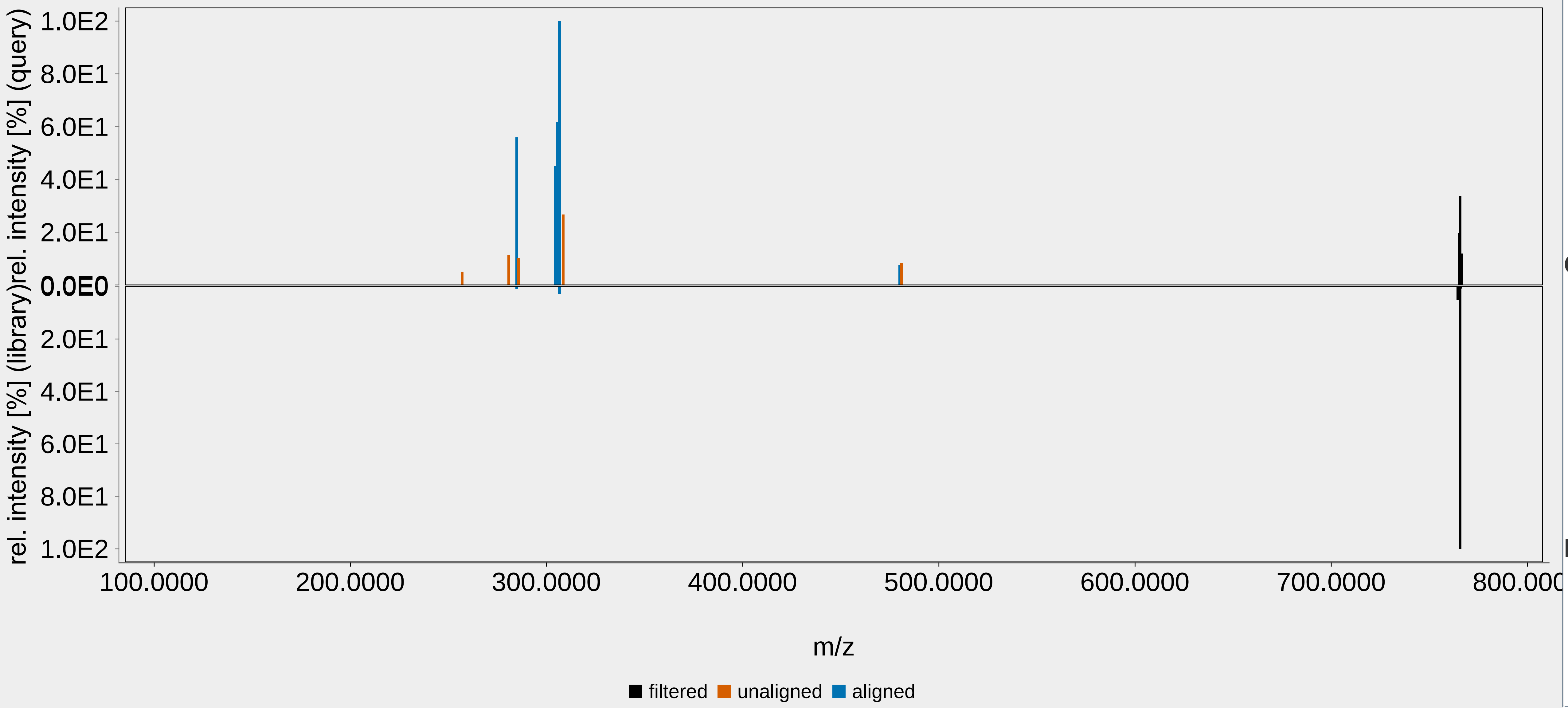

Compound information

Name: PE(18:0/20:3); [M-H]- C43H79N1O8P1

Exact mass: 0.0

Ion type: M-H

Precursor m/z: 768.554

Charge: 1

Polarity: Negative

Instrument information

Instrument type: HCD; Velos

Ion source: LC-ESI

Ms level: 2

Acquisition: Commercial

Database links

Other information

Principal investigator: Thomas Metz

Data collector: Thomas Metz

Entry ID: CCMSLIB00010079224

PE(18:0/20:3); [M-H]- C43H79N1O8P1 **0.759**

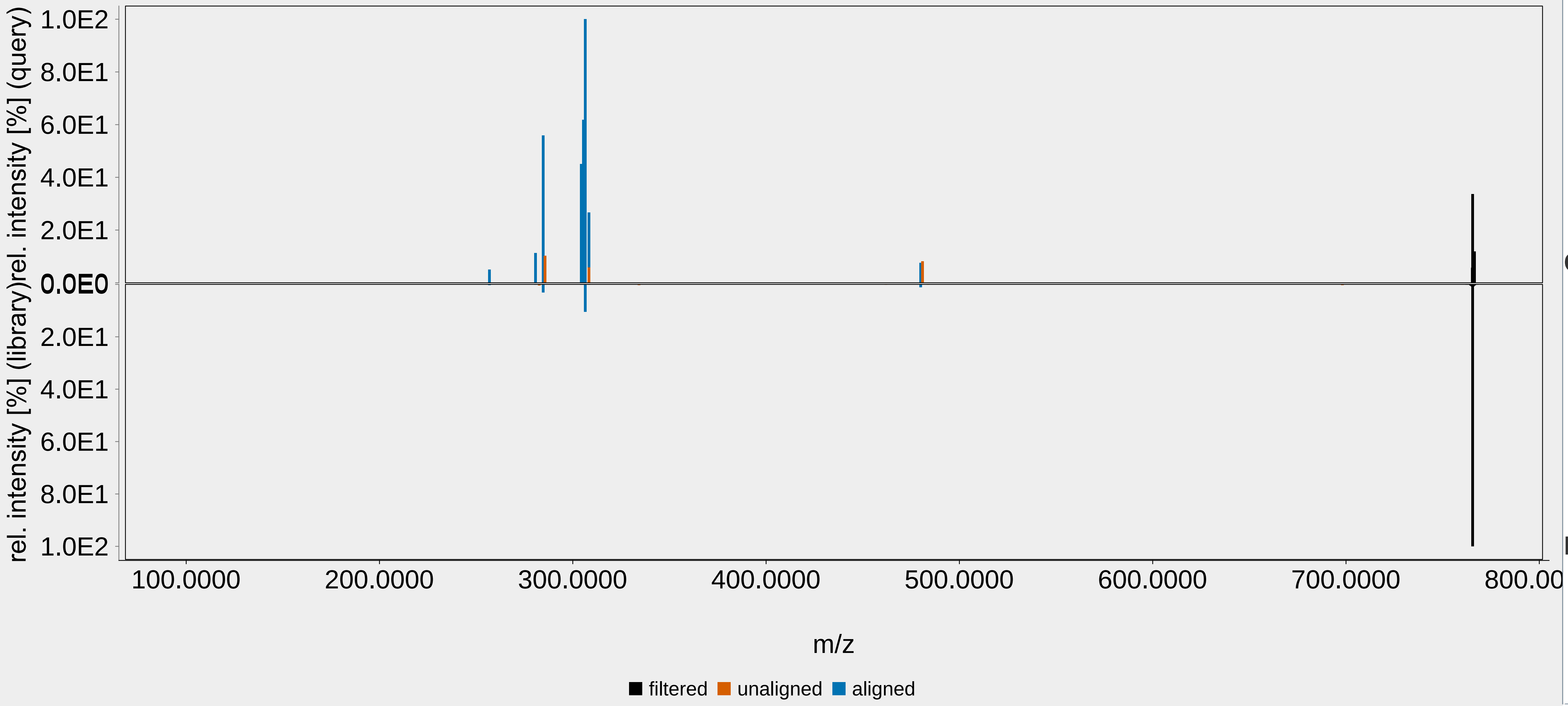

Compound information

Name: PE(18:0/20:3); [M-H]- C43H79N1O8P1

Exact mass: 0.0

Ion type: M-H

Precursor m/z: 768.554

Charge: 1

Polarity: Negative

Instrument information

Instrument type: HCD; Velos

Ion source: LC-ESI

Ms level: 2

Acquisition: Commercial

Database links

Other information

Principal investigator: Thomas Metz

Data collector: Thomas Metz

Entry ID: CCMSLIB00010071376

PA 22:6_22:6

0.946

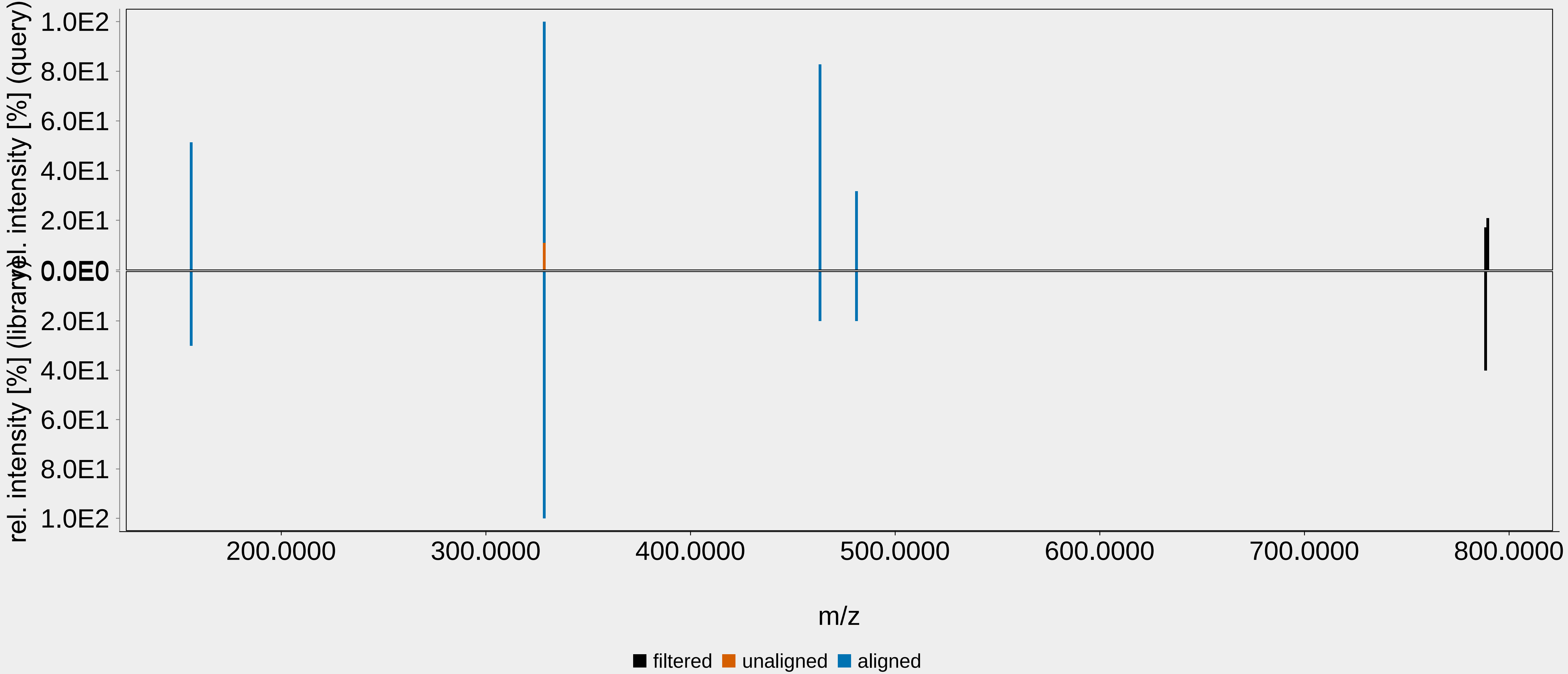

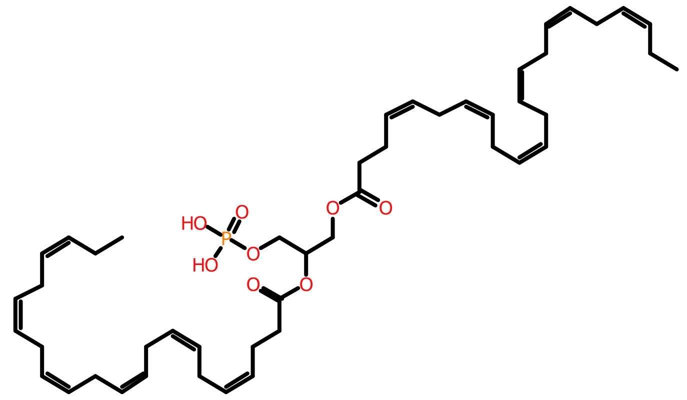

Compound information

Name: PA 22:6_22:6

Formula: C47H69O8P

Ion type: [M-H]-

Precursor m/z: 791.46573

Ccs: 269.85

InChI key: YUDIFVLBAGUYSQ-GZSOIYOPNA-

SMILES:

Num peaks: 5

Instrument information

Database links

Other information

PA(18:0/18:1); [M-H]- C39H74O8P1 0.862

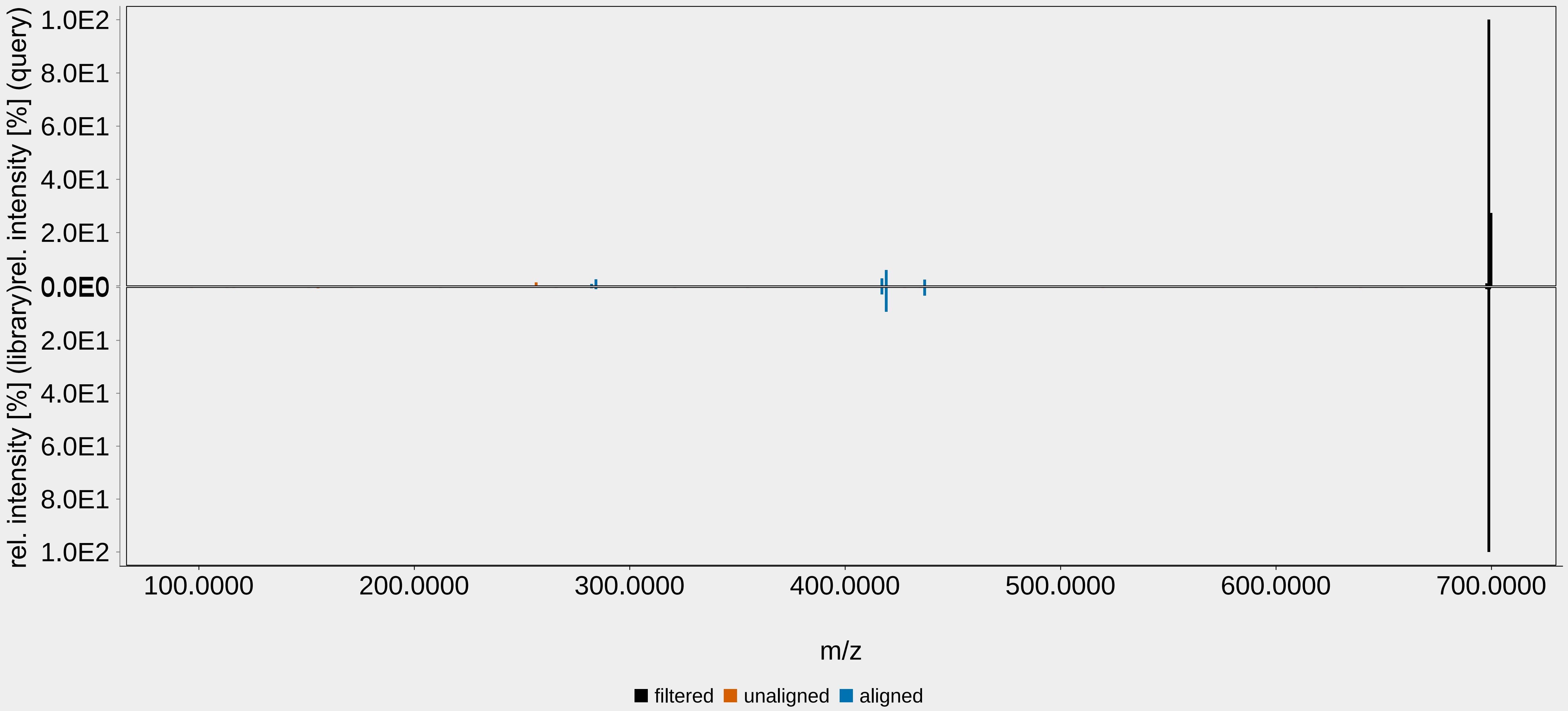

Compound information

Name: PA(18:0/18:1); [M-H]- C39H74O8P1

Exact mass: 0.0

Ion type: M-H

Precursor m/z: 701.512

Charge: 1

Polarity: Negative

Instrument information

Instrument type: HCD; Velos

Ion source: LC-ESI

Ms level: 2

Acquisition: Commercial

Database links

Other information

Principal investigator: Thomas Metz

Data collector: Thomas Metz

Entry ID: CCMSLIB00010076011

PA 18:0_18:1

0.895

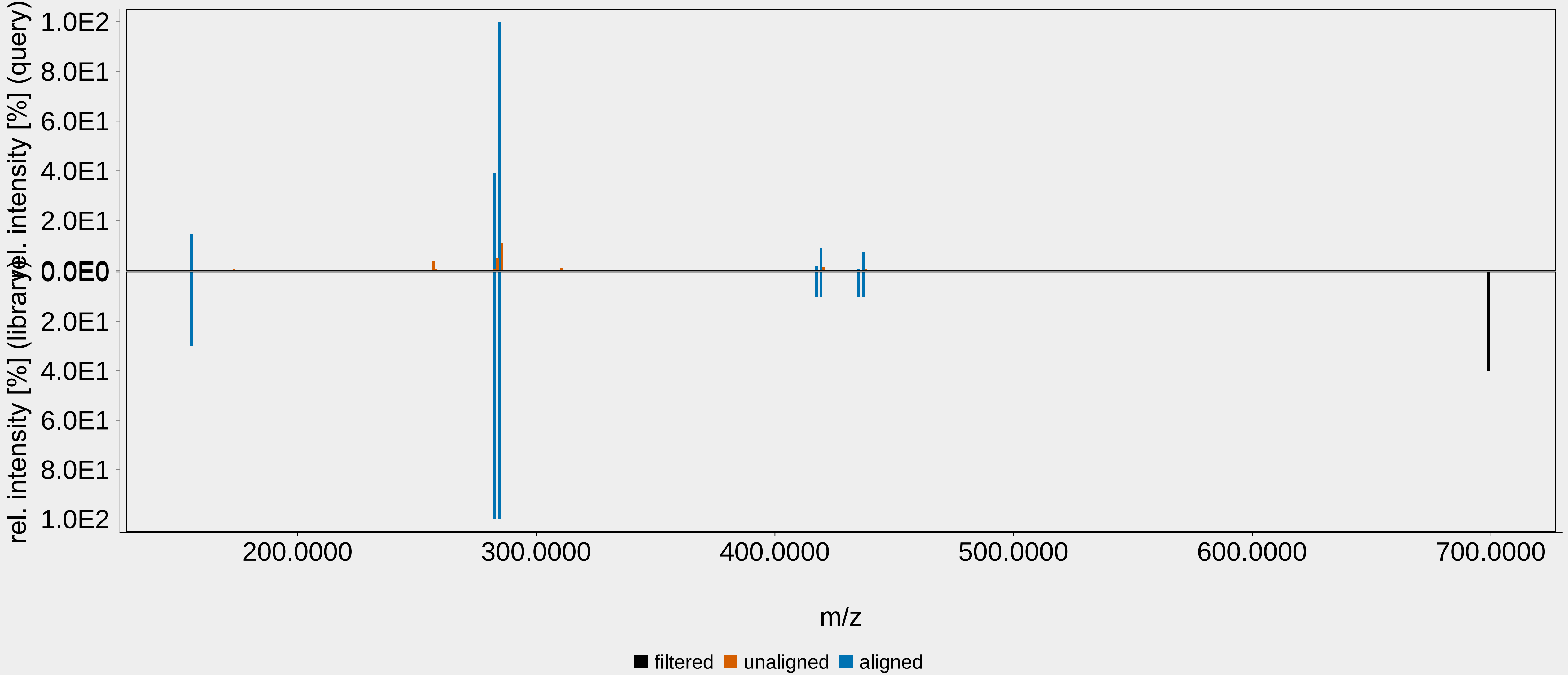

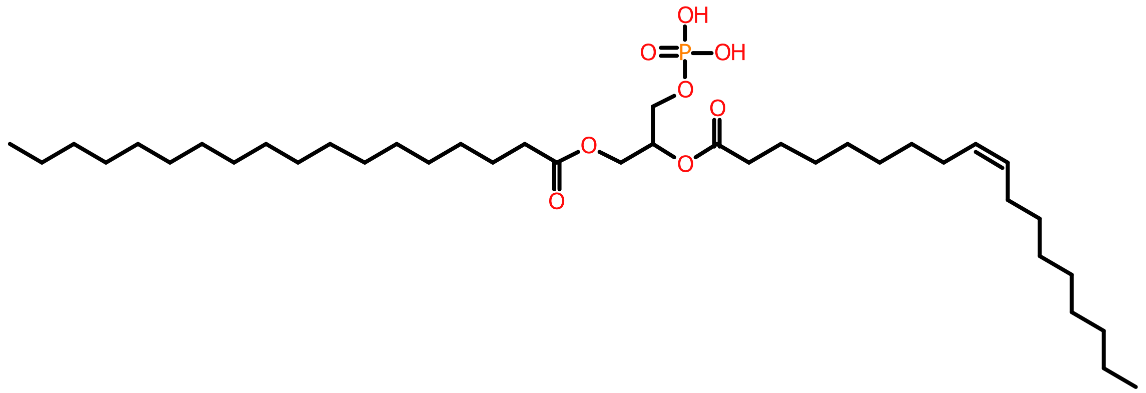

Compound information

Name: PA 18:0_18:1

Formula: C39H75O8P

Ion type: [M-H]-

Precursor m/z: 701.51268

Ccs: 258.98

InChI key:

SMILES:

Num peaks: 8

Instrument information

Database links

Other information

Glutathione - 30.0 eV

0.774

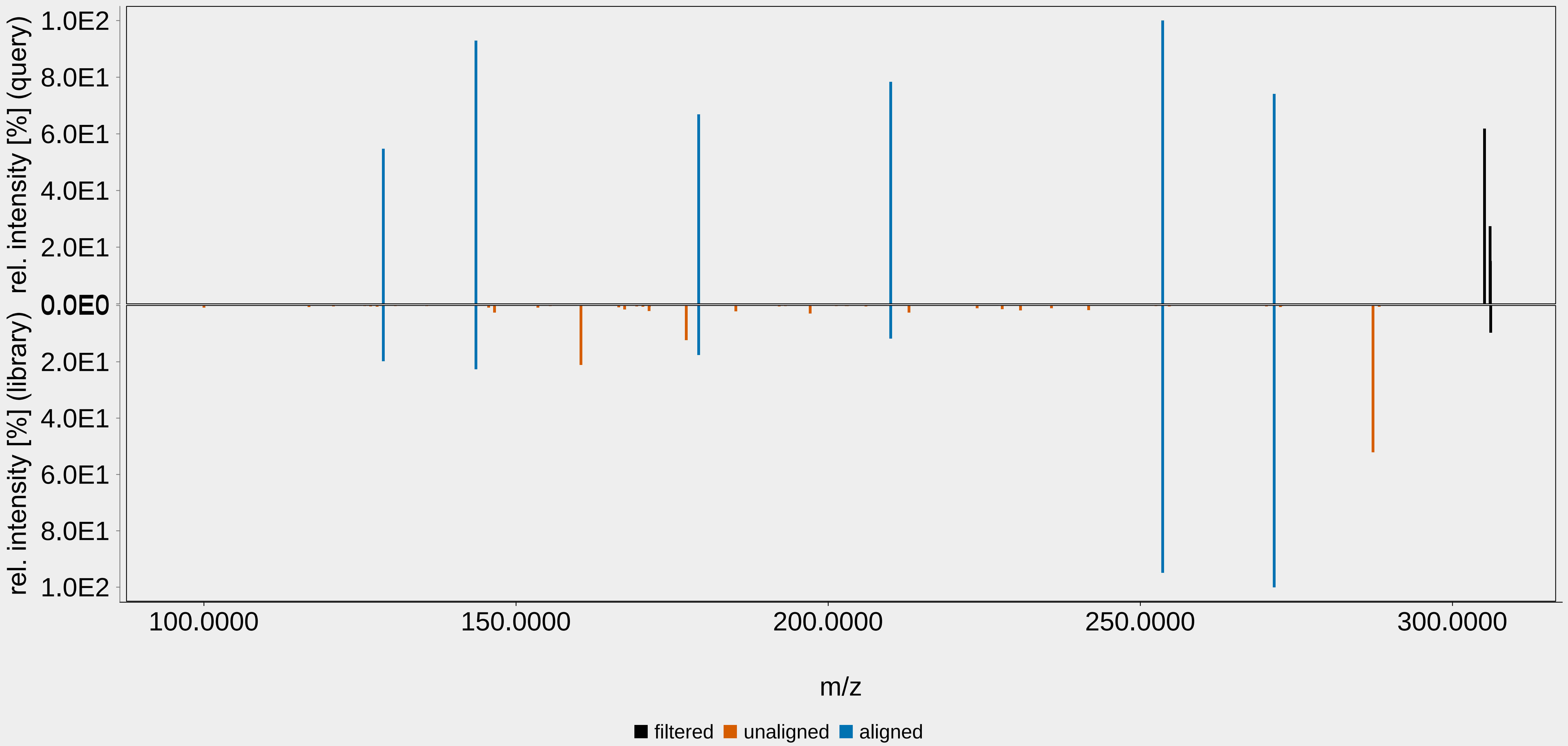

Compound information

Name: Glutathione - 30.0 eV

Exact mass: 0.0

Ion type: M-H

Precursor m/z: 306.077

Charge: 1

Polarity: Negative

SMILES:

Instrument information

Instrument type: Orbitrap

Ion source: ESI

Ms level: 2

Acquisition: Isolated

Database links

CAS:

Other information

Principal investigator: PI

Data collector: Data Collector

Entry ID: CCMSLIB00006121887

Glutathione - 30.0 eV 0.776

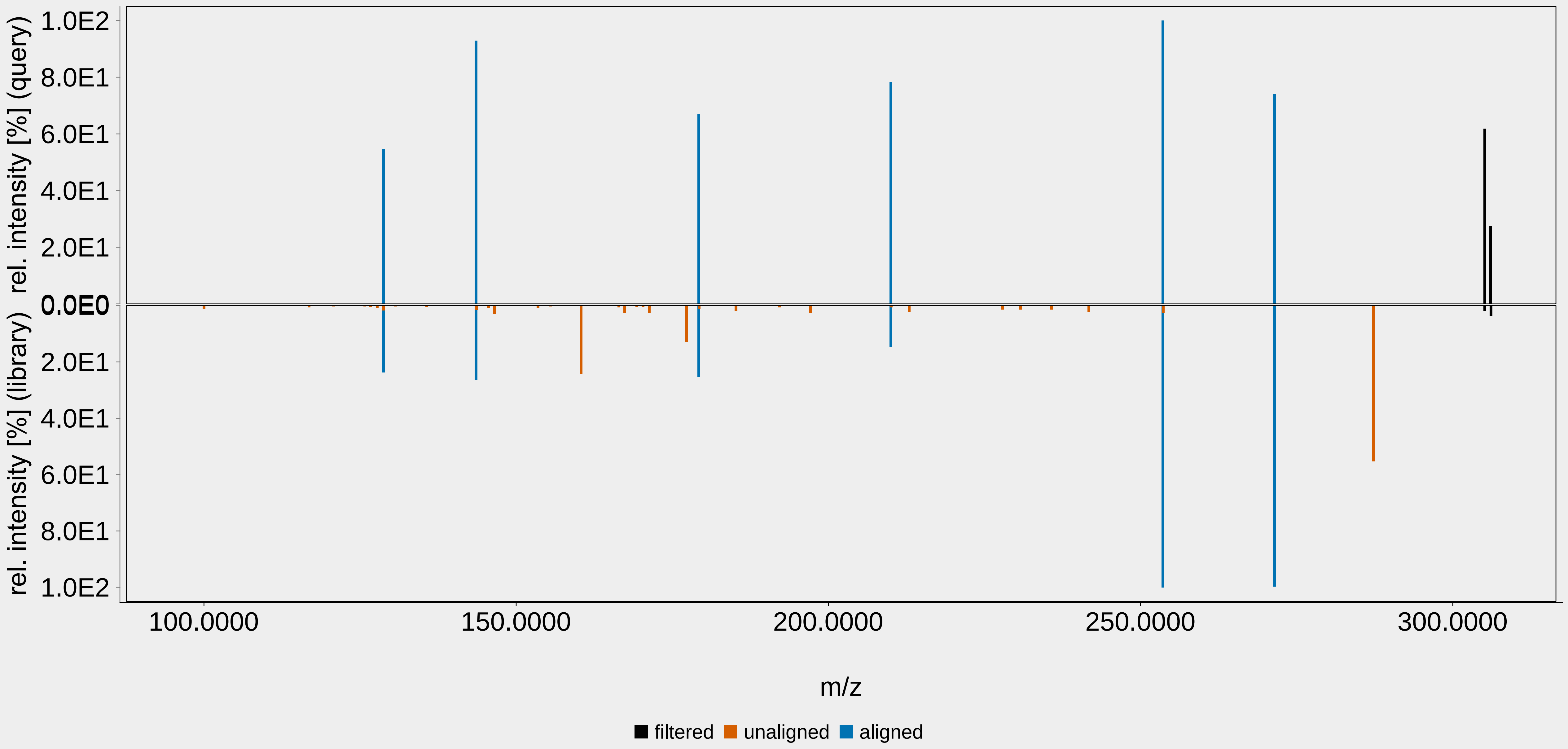

Compound information

Name: Glutathione - 30.0 eV

Exact mass: 0.0

Ion type: M-H

Precursor m/z: 306.077

Charge: 1

Polarity: Negative

SMILES:

Instrument information

Instrument type: Orbitrap

Ion source: ESI

Ms level: 2

Acquisition: Isolated

Database links

CAS:

Other information

Principal investigator: PI

Data collector: Data Collector

Entry ID: CCMSLIB00006121879

PS 24:0_18:1

0.866

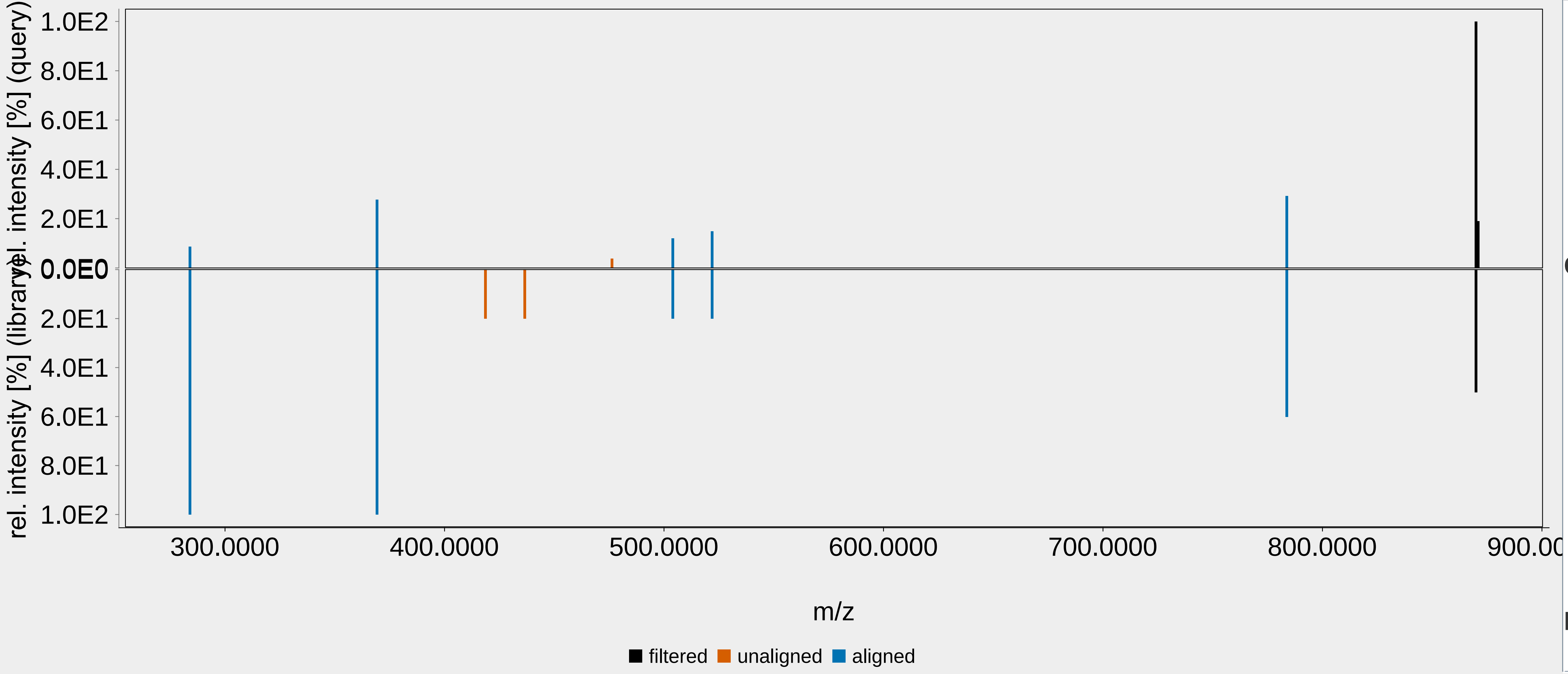

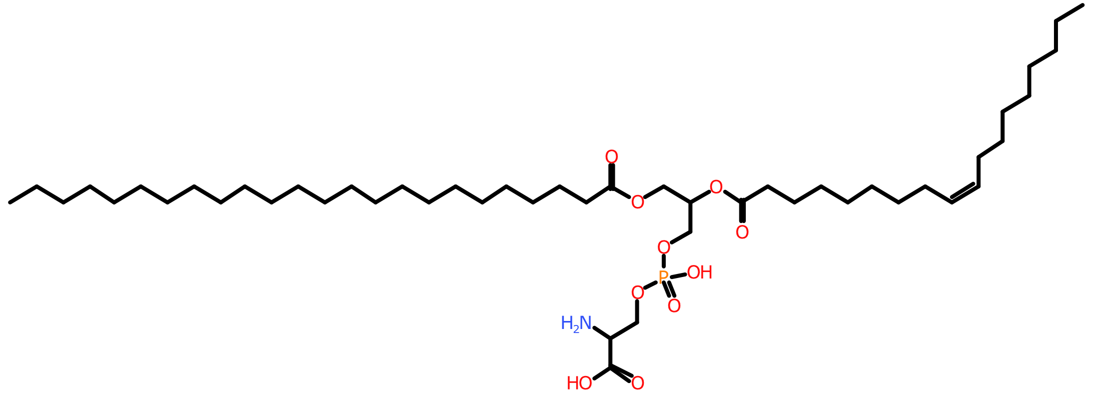

Compound information

Name: PS 24:0_18:1

Formula: C48H92NO10P

Ion type: [M-H]-

Precursor m/z: 872.63861

Ccs: 294.58

InChI key:

SMILES:

Num peaks: 8

Instrument information

Database links

Other information

PE(16:0/22:6); [M-H]- C43H73N1O8P1 **0.944**

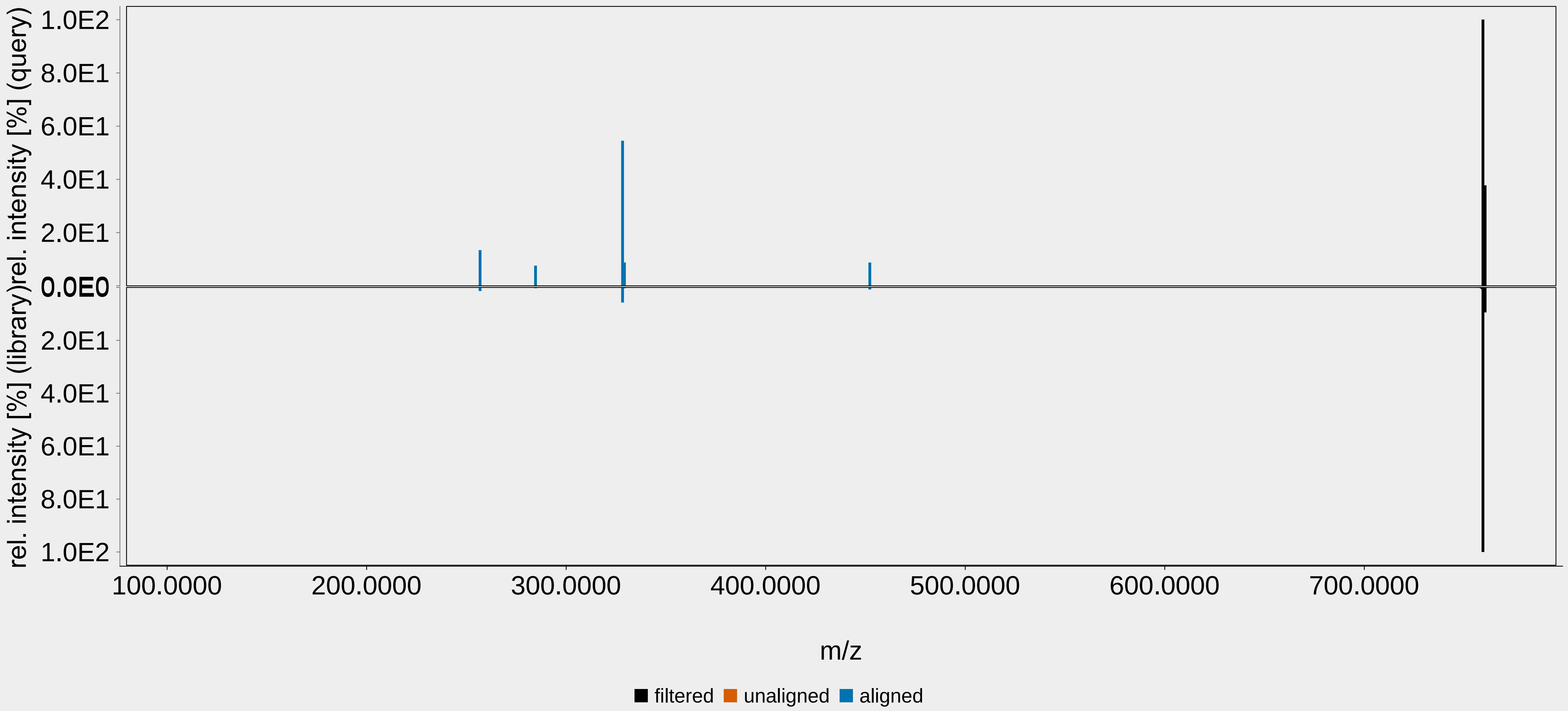

Compound information

Name: PE(16:0/22:6); [M-H]- C43H73N1O8P1

Exact mass: 0.0

Ion type: M-H

Precursor m/z: 762.507

Charge: 1

Polarity: Negative

Instrument information

Instrument type: HCD; Velos

Ion source: LC-ESI

Ms level: 2

Acquisition: Commercial

Database links

Other information

Principal investigator: Thomas Metz

Data collector: Thomas Metz

Entry ID: CCMSLIB00010088316

PE(16:0/22:6); [M-H]- C43H73N1O8P1 0.940

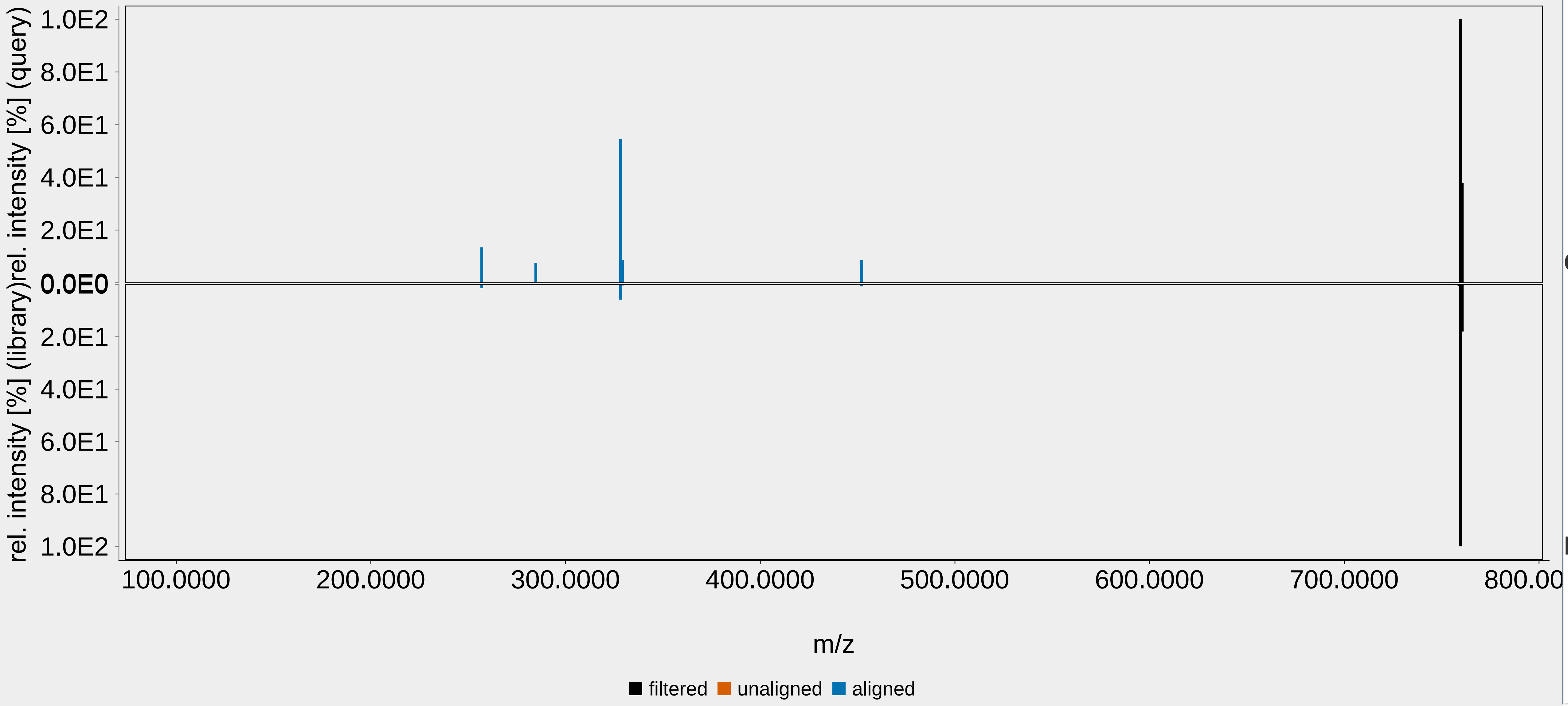

Compound information

Name: PE(16:0/22:6); [M-H]- C43H73N1O8P1

Exact mass: 0.0

Ion type: M-H

Precursor m/z: 762.507

Charge: 1

Polarity: Negative

Instrument information

Instrument type: HCD; Velos

Ion source: LC-ESI

Ms level: 2

Acquisition: Commercial

Database links

Other information

Principal investigator: Thomas Metz

Data collector: Thomas Metz

Entry ID: CCMSLIB00010064176

LPI 22:6

0.830

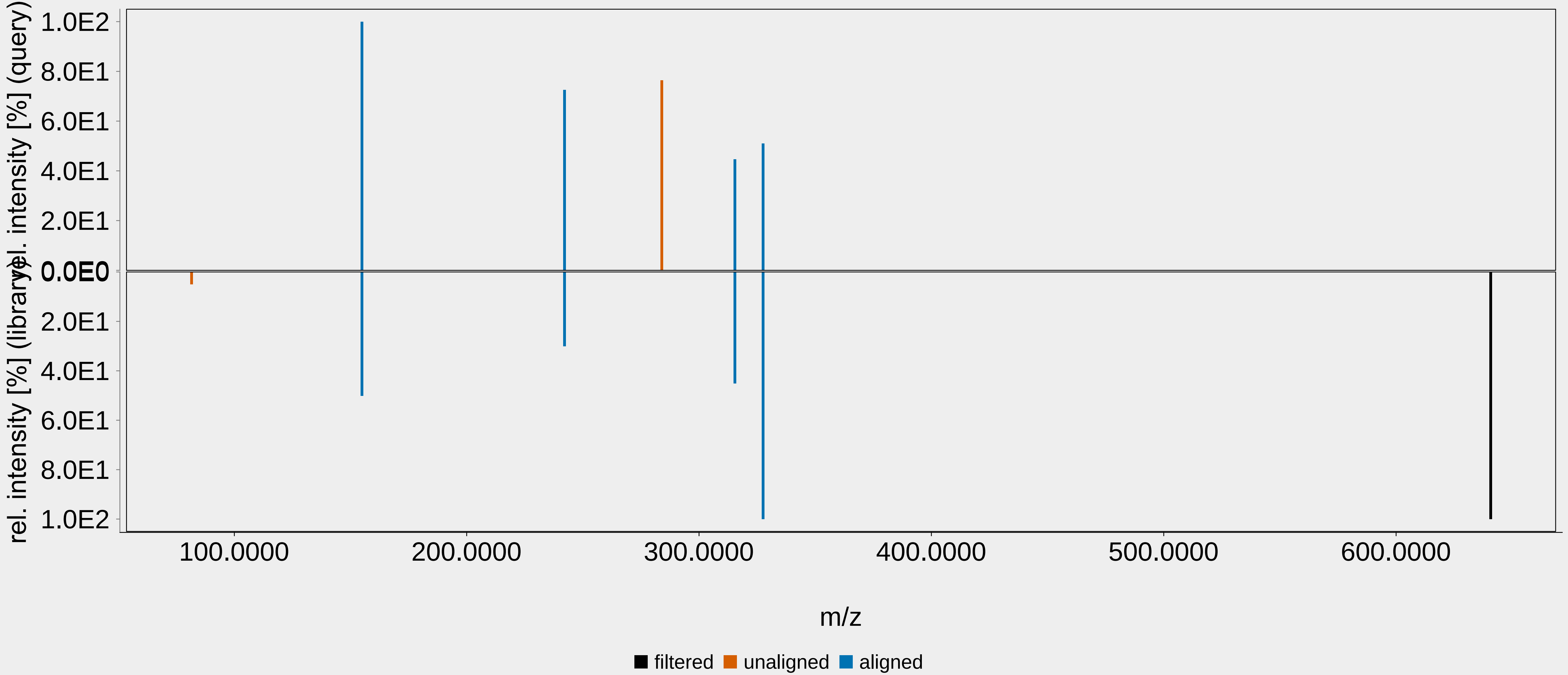

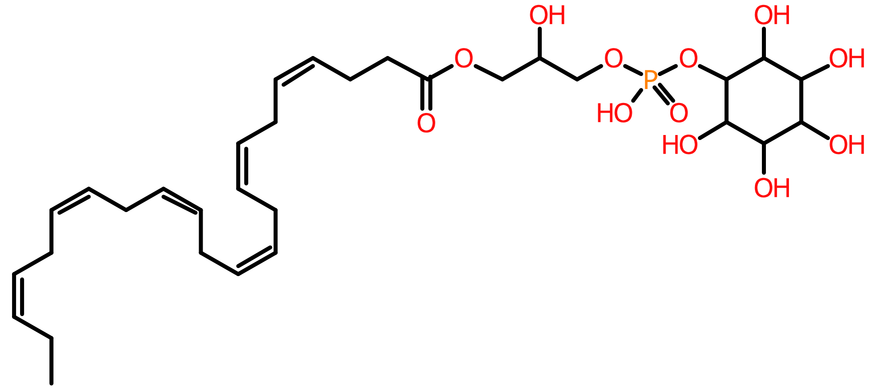

Compound information

Name: LPI 22:6

Formula: C31H49O12P

Ion type: [M-H]-

Precursor m/z: 643.28889

Ccs: 237.14

InChI key:

SMILES:

Num peaks: 6

Instrument information

Database links

Other information

PE(22:4/0:0); [M-H]- C27H47N1O7P1

0.893

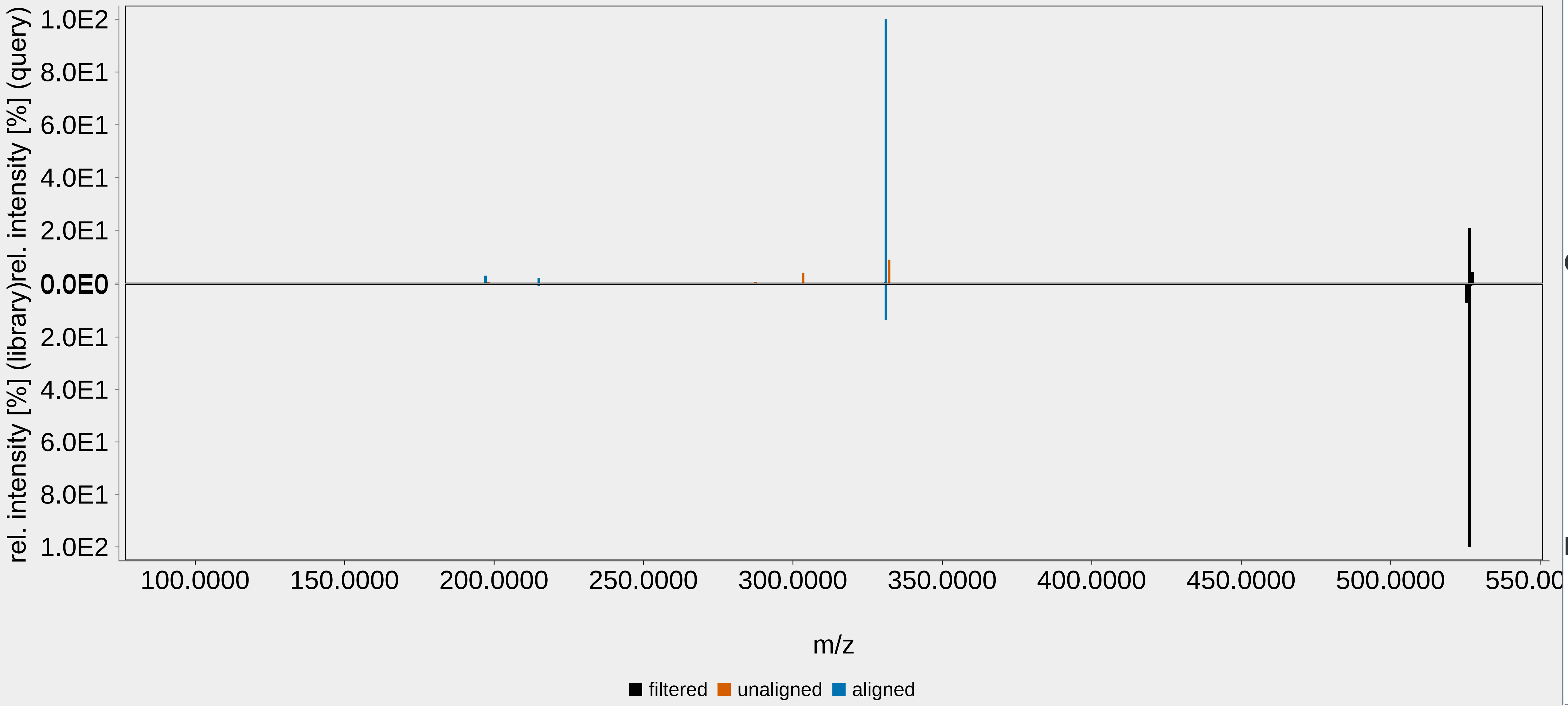

Compound information

Name: PE(22:4/0:0); [M-H]- C27H47N1O7P1

Exact mass: 0.0

Ion type: M-H

Precursor m/z: 528.309

Charge: 1

Polarity: Negative

Instrument information

Instrument type: HCD; Velos

Ion source: LC-ESI

Ms level: 2

Acquisition: Commercial

Database links

Other information

Principal investigator: Thomas Metz

Data collector: Thomas Metz

Entry ID: CCMSLIB00010086739

PE(22:4/0:0); [M-H]- C27H47N1O7P1

0.897

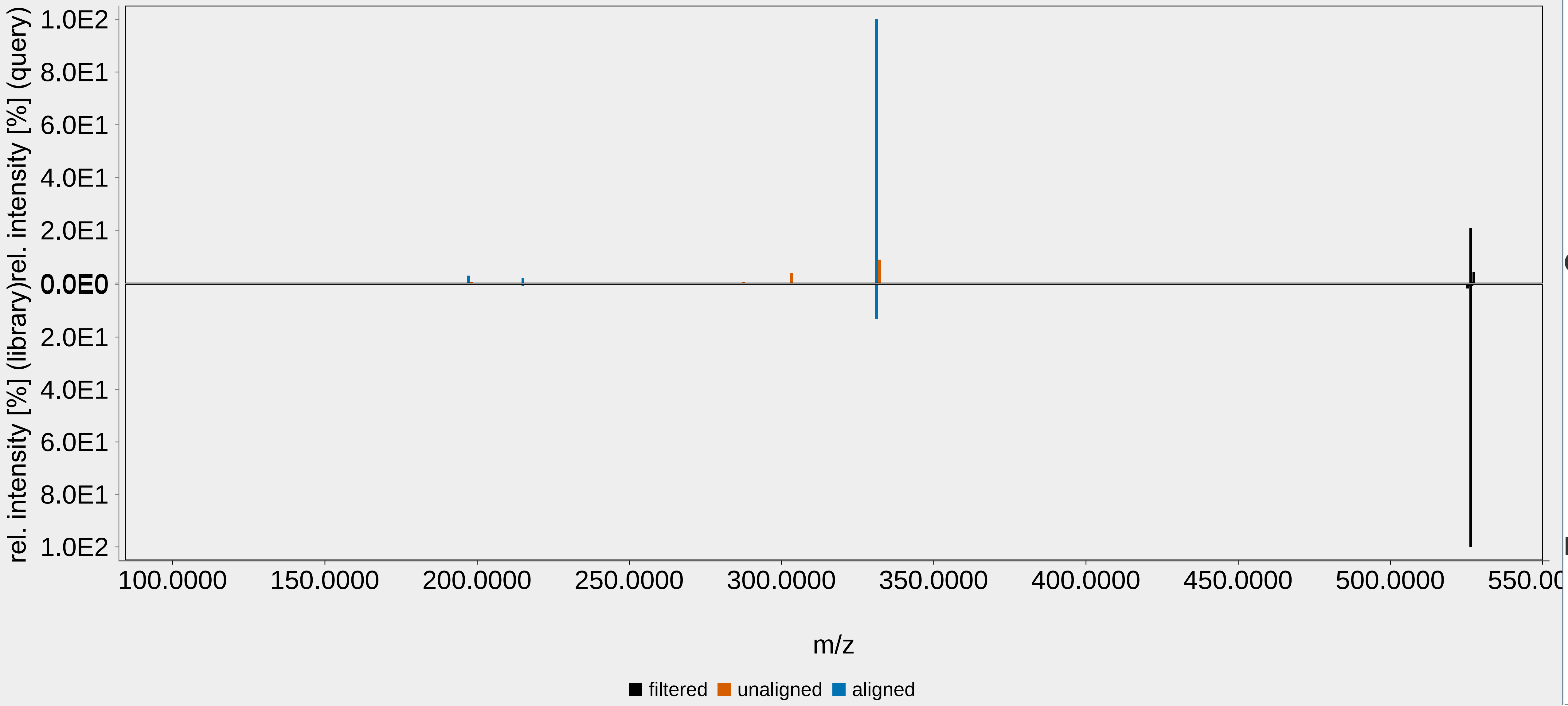

Compound information

Name: PE(22:4/0:0); [M-H]- C27H47N1O7P1

Exact mass: 0.0

Ion type: M-H

Precursor m/z: 528.309

Charge: 1

Polarity: Negative

Instrument information

Instrument type: HCD; Velos

Ion source: LC-ESI

Ms level: 2

Acquisition: Commercial

Database links

Other information

Principal investigator: Thomas Metz

Data collector: Thomas Metz

Entry ID: CCMSLIB00010063574

PC 38:6

0.734

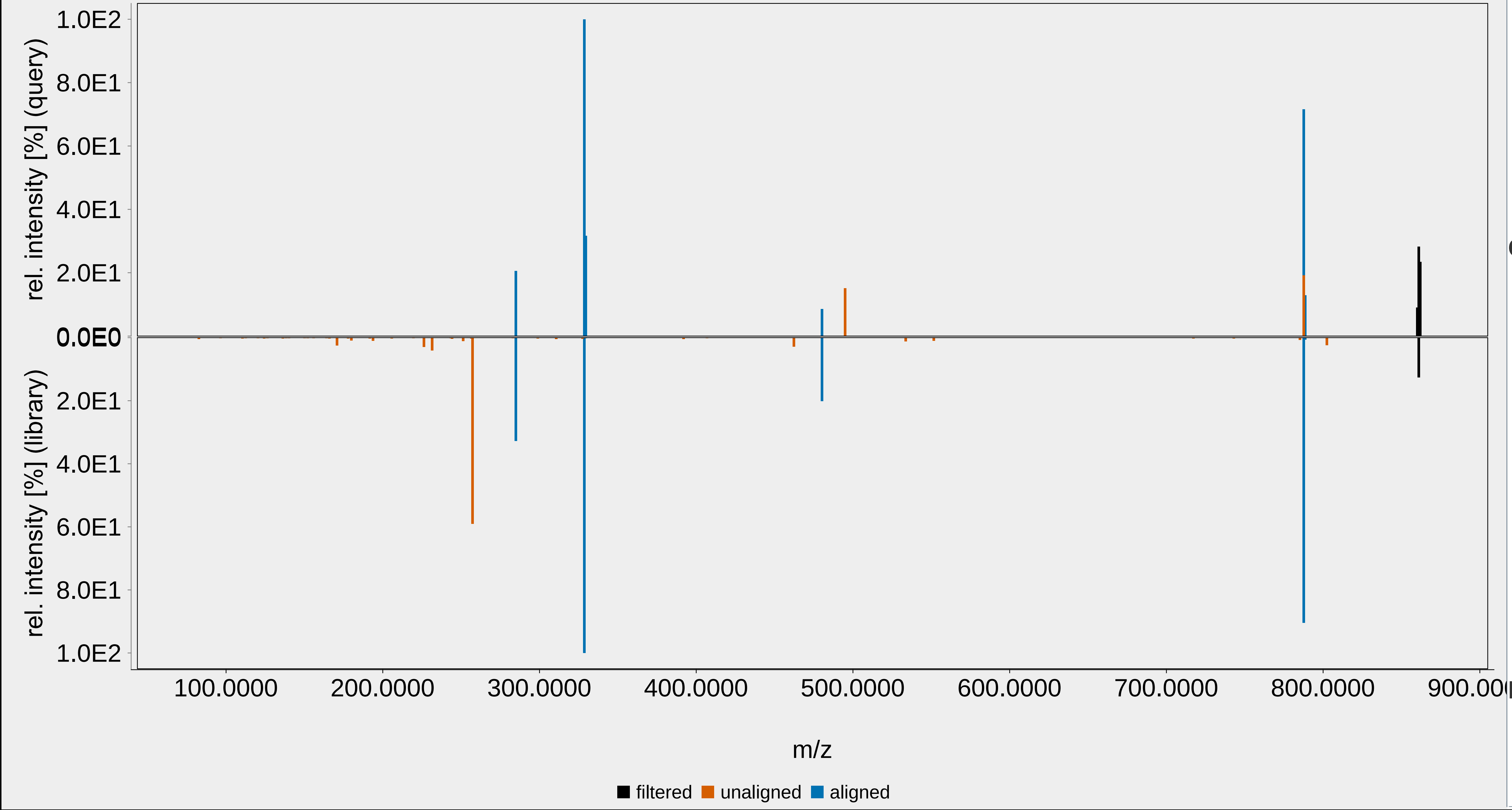

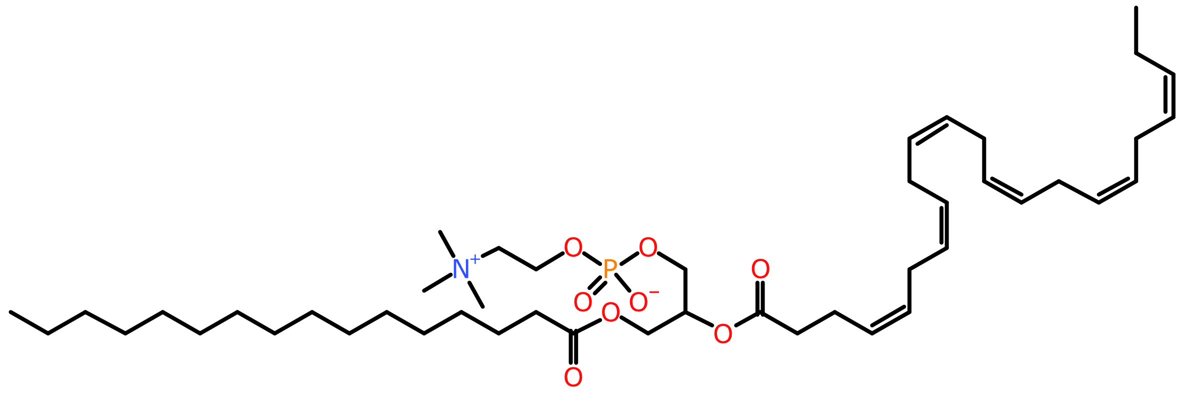

Compound information

Name: PC 38:6

Formula: C46H80NO8P

Mol. weight: 806.1209999999999

Exact mass: 806.1209999999999

Ion type: [M+CH3COOH-H]-

Precursor m/z: 864.5729

RT: 9.202155

Polarity: negative

InChI:

InChI key:

SMILES:

Instrument information

Instrument type: LC-ESI-QTOF

Instrument: AB Sciex TripleTOF 5600+ system

Ms level: MS2

Collision energy: 45V

Database links

Pubchem: 9940652

MoNA ID: LQB00502

Other information

Principal investigator: Kazutaka Ikeda, Hiroshi

Data collector: Kazutaka Ikeda, Hiroshi

PC 38:6 **0.734**

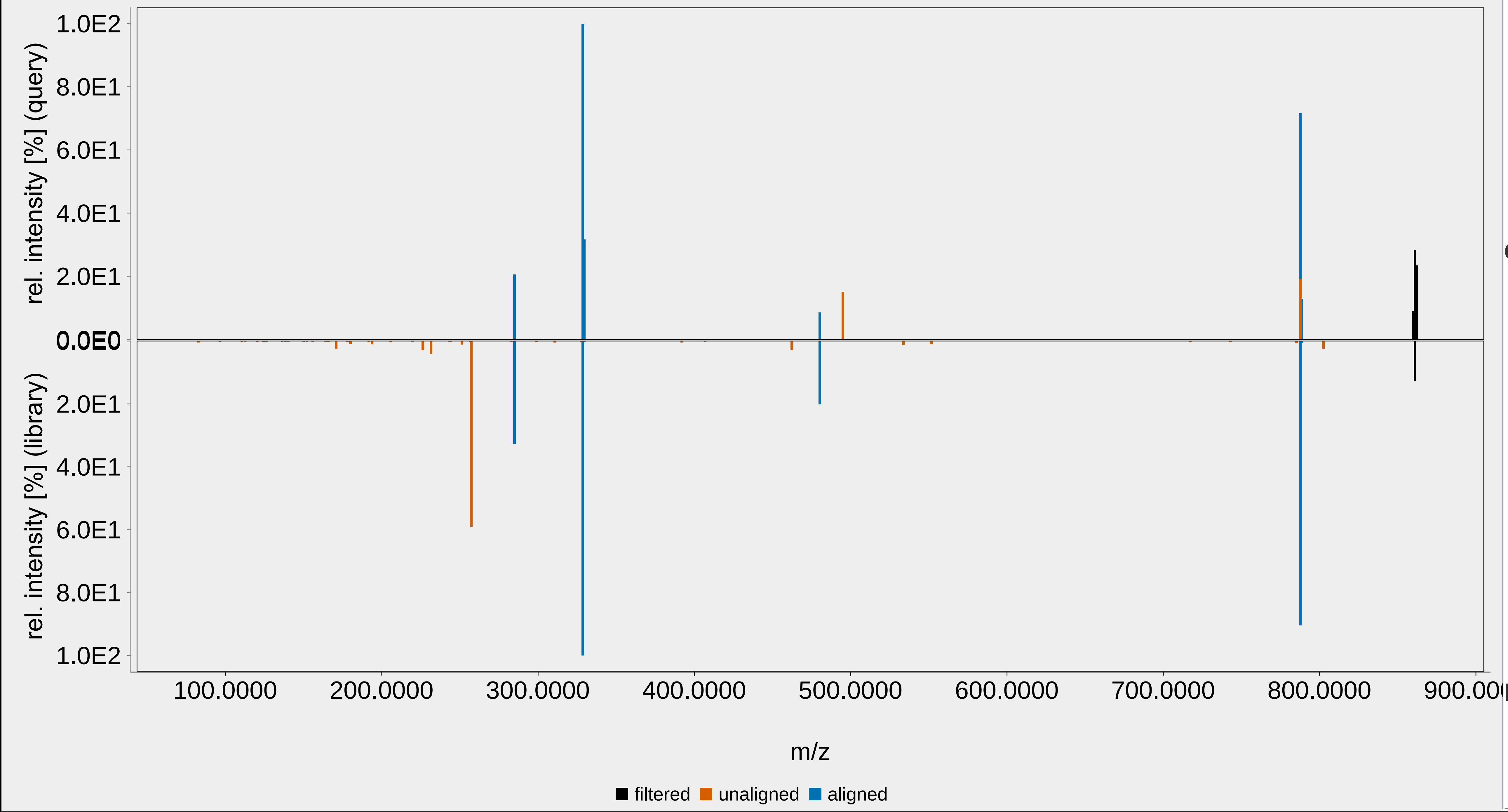

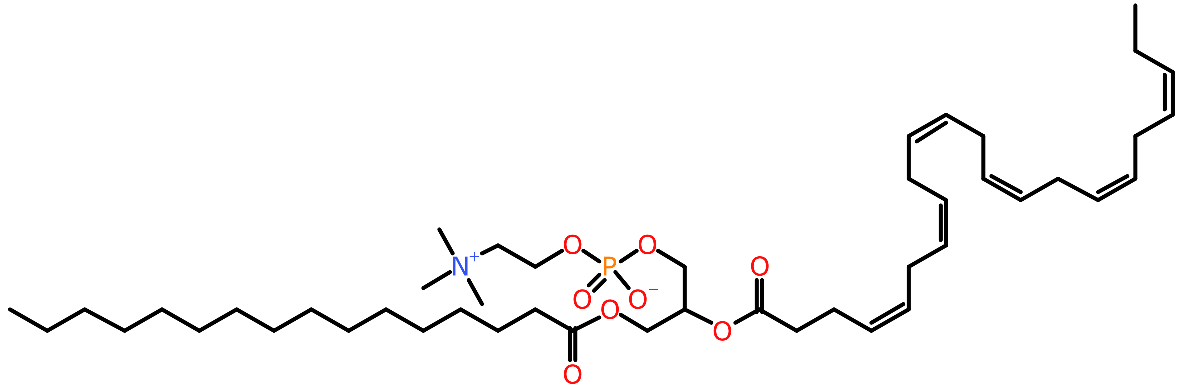

Compound information

Name: PC 38:6

Formula: C46H80NO8P

Mol. weight: 806.1209999999999

Exact mass: 806.1209999999999

Ion type: [M+CH3COOH-H]-

Precursor m/z: 864.5729

RT: 9.202155

Polarity: negative

InChI:

InChI key:

SMILES:

Instrument information

Instrument type: LC-ESI-QTOF

Instrument: AB Sciex TripleTOF 5600+ system

Ms level: MS2

Collision energy: 45V

Database links

Pubchem: 9940652

MoNA ID: LQB00116

Other information

Principal investigator: Kazutaka Ikeda, Hiroshi

Data collector: Kazutaka Ikeda, Hiroshi

PS(18:1_0:0) - 2-amino-3-[hydroxy-[2-hydroxy-3-[octadec-9-enoyl]oxypropoxy]phosphoryl]oxypropanoic acid

0.839

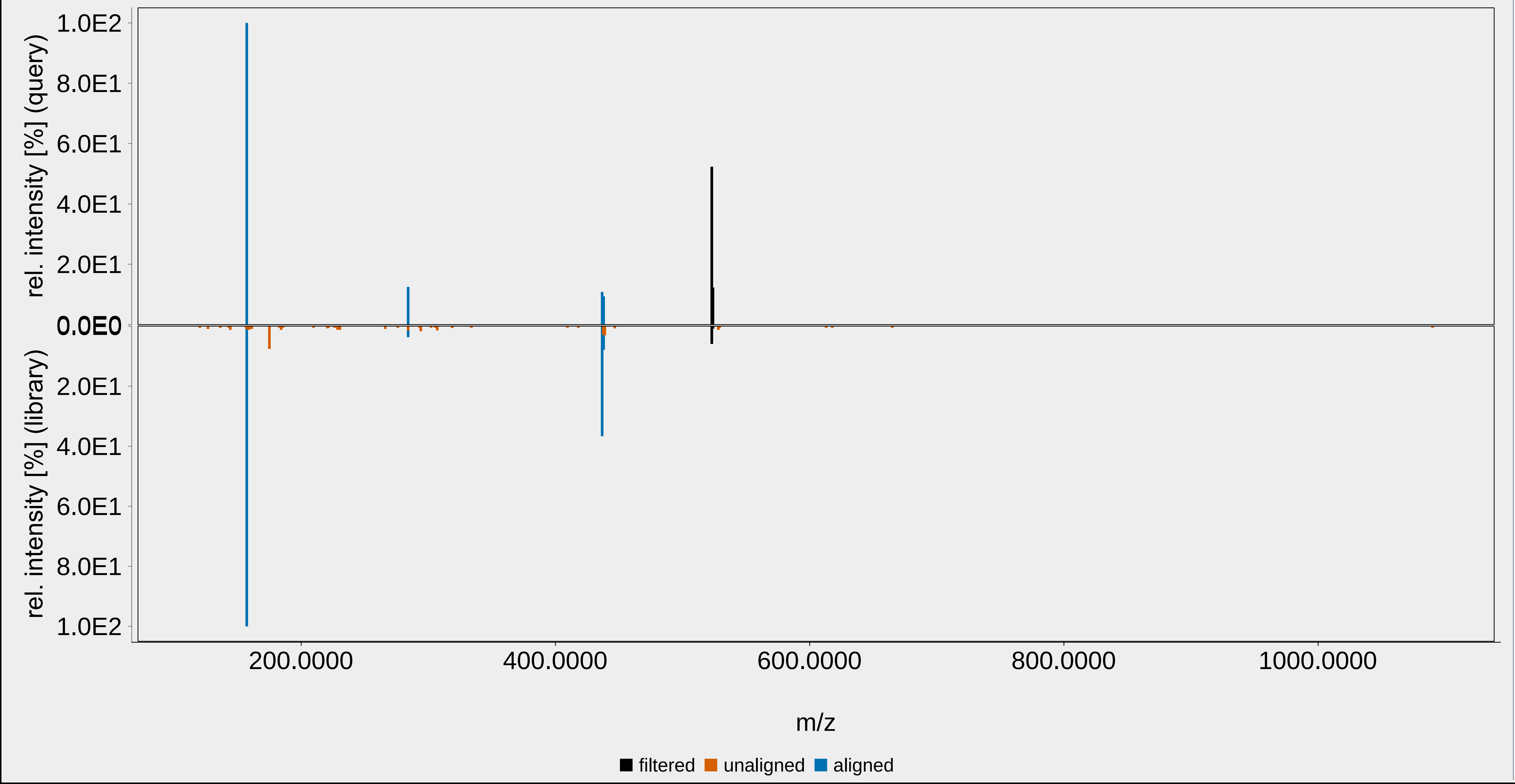

Compound information

Name: PS(18:1_0:0) -

Exact mass: 523.291

Ion type: M-H

Precursor m/z: 522.283

Charge: 1

Polarity: Negative

InChI:

InChI key:

SMILES:

Instrument information

Instrument type: qTof

Ion source: LC-ESI

Ms level: 2

Acquisition: Commercial

Database links

Pubmed:

CAS: 326589-90-6

Other information

Principal investigator: Laprevote

Data collector: UMR 8038 Universite de Paris

Entry ID: CCMSLIB00005436456

2-amino-3-[hydroxy-[2-hydroxy-3-[octadec-9-enoyl]oxypropoxy]phosphoryl]oxypropanoic acid

0.839

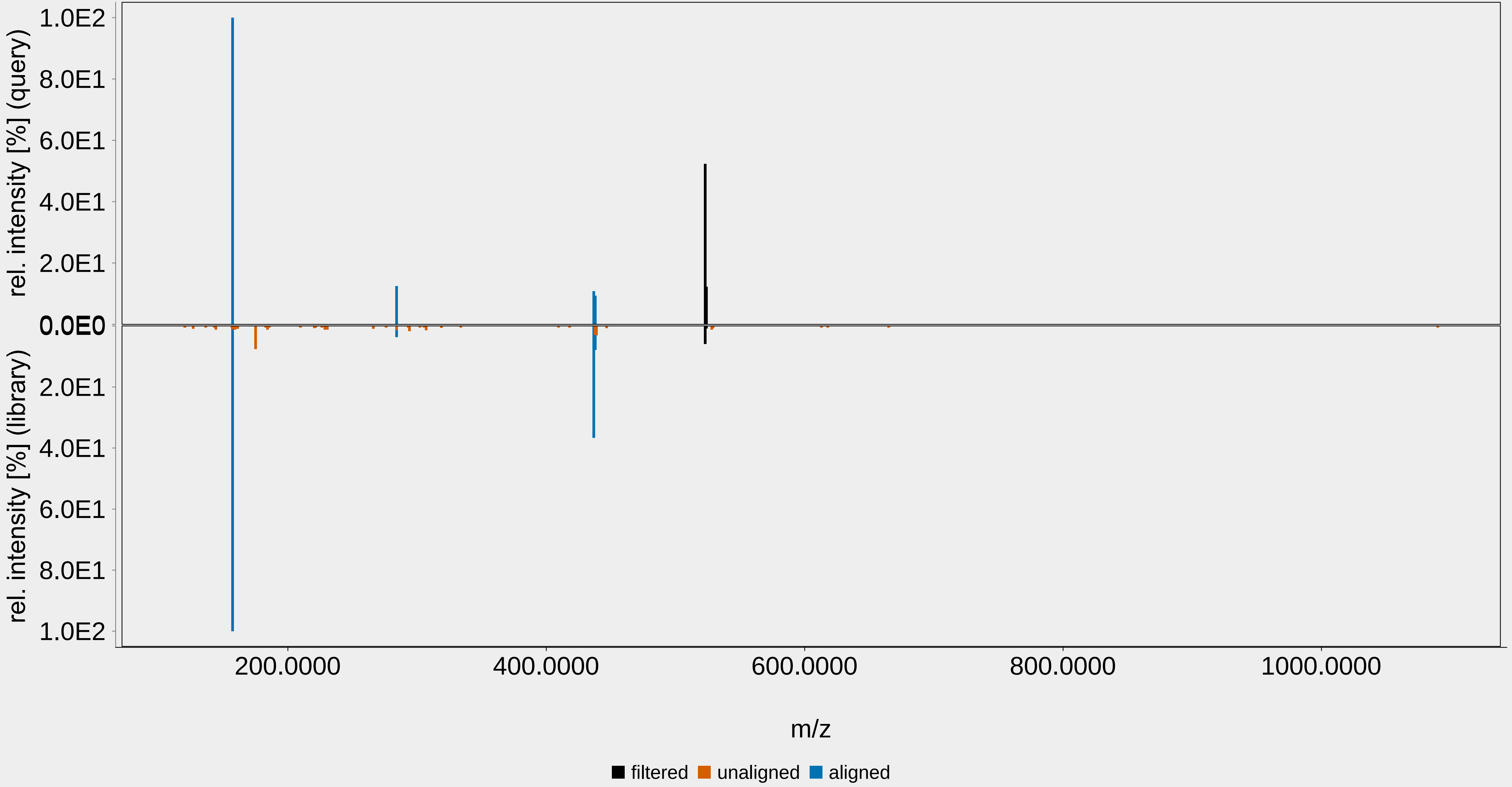

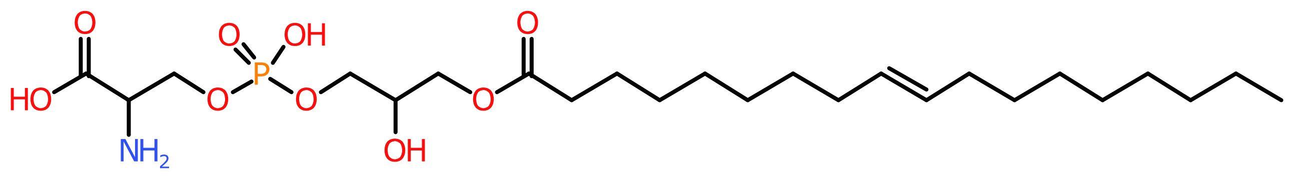

Compound information

Name:

Formula: C24H46NO9P

Mol. weight: 523.291

Exact mass: 523.291

Ion type: [M-H]-

Precursor m/z: 522.283

Polarity: negative

InChI:

InChI key:

SMILES:

Instrument information

Instrument: qTof

Ms level: MS2

Database links

Other information

Principal investigator: Laprevote, UMR 8038

Data collector: Laprevote, UMR 8038 Universite

LNAPE 16:0/N-18:0

0.906

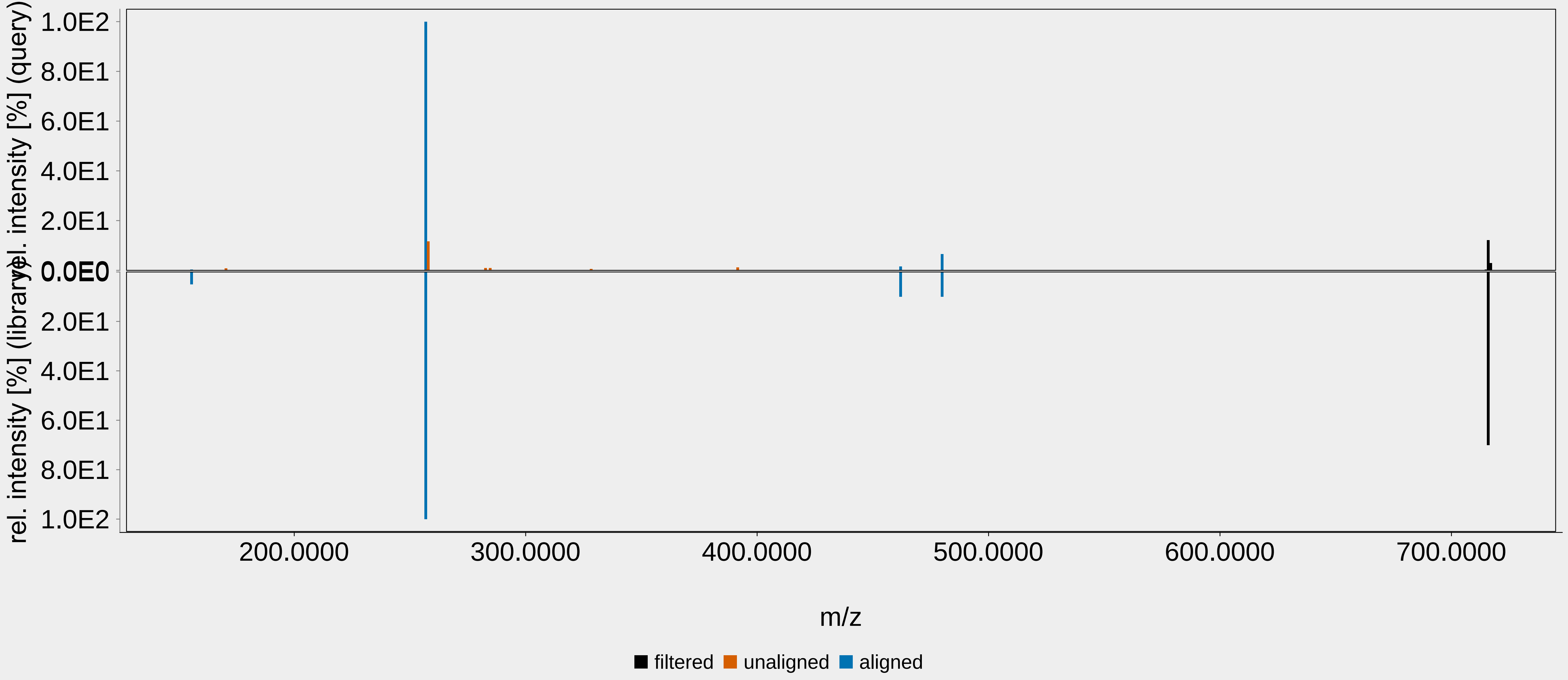

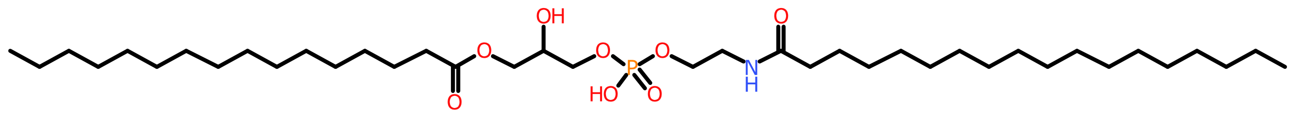

Compound information

Name: LNAPE 16:0/N-18:0

Formula: C39H78NO8P

Ion type: [M-H]-

Precursor m/z: 718.53923

Ccs: 265.73

InChI key:

SMILES:

Num peaks: 5

Instrument information

Database links

Other information

PE(16:0/18:0); [M-H]- C39H77N1O8P1 0.945

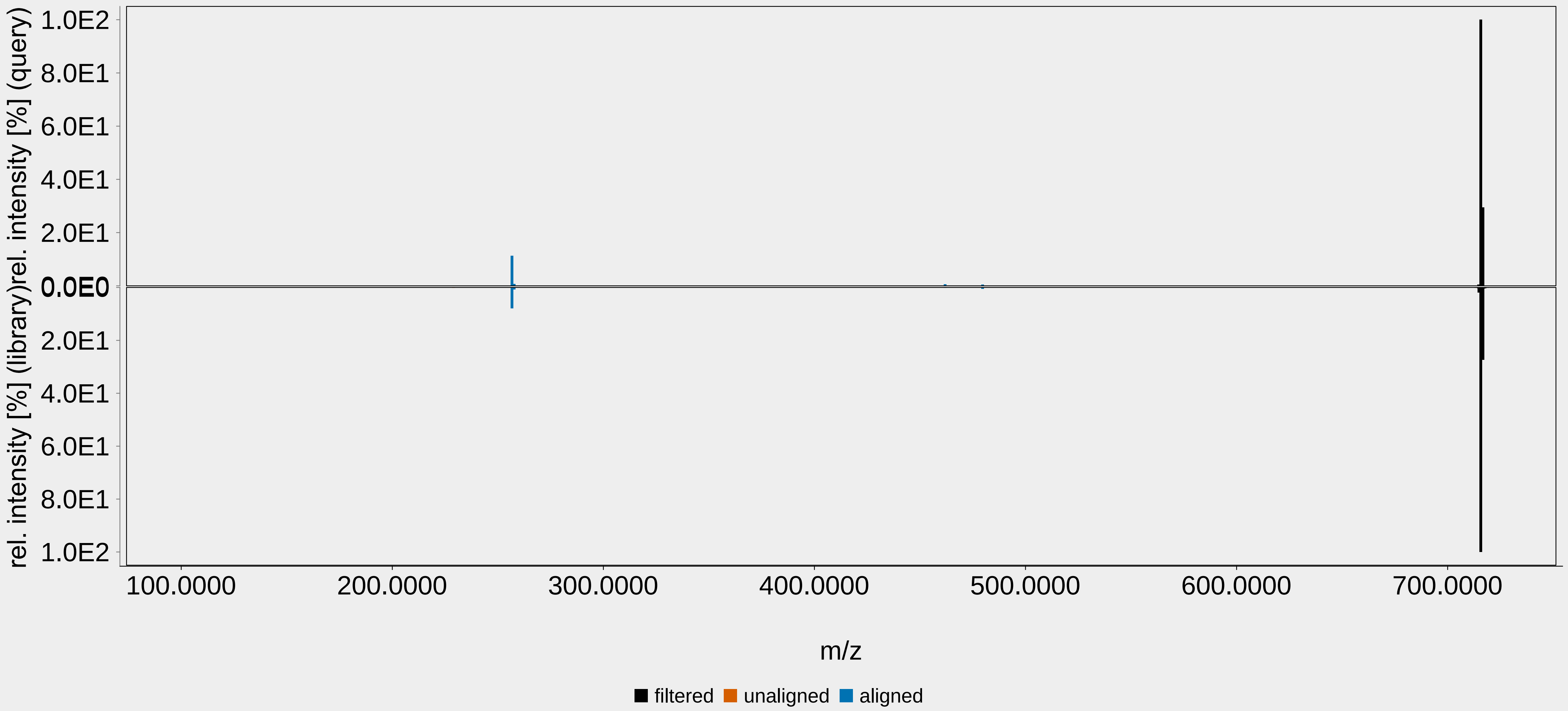

Compound information

Name: PE(16:0/18:0); [M-H]- C39H77N1O8P1

Exact mass: 0.0

Ion type: M-H

Precursor m/z: 718.539

Charge: 1

Polarity: Negative

Instrument information

Instrument type: HCD; Velos

Ion source: LC-ESI

Ms level: 2

Acquisition: Commercial

Database links

Other information

Principal investigator: Thomas Metz

Data collector: Thomas Metz

Entry ID: CCMSLIB00010073693

PE(18:1/22:0); [M-H]- C45H87N1O8P1 **0.729**

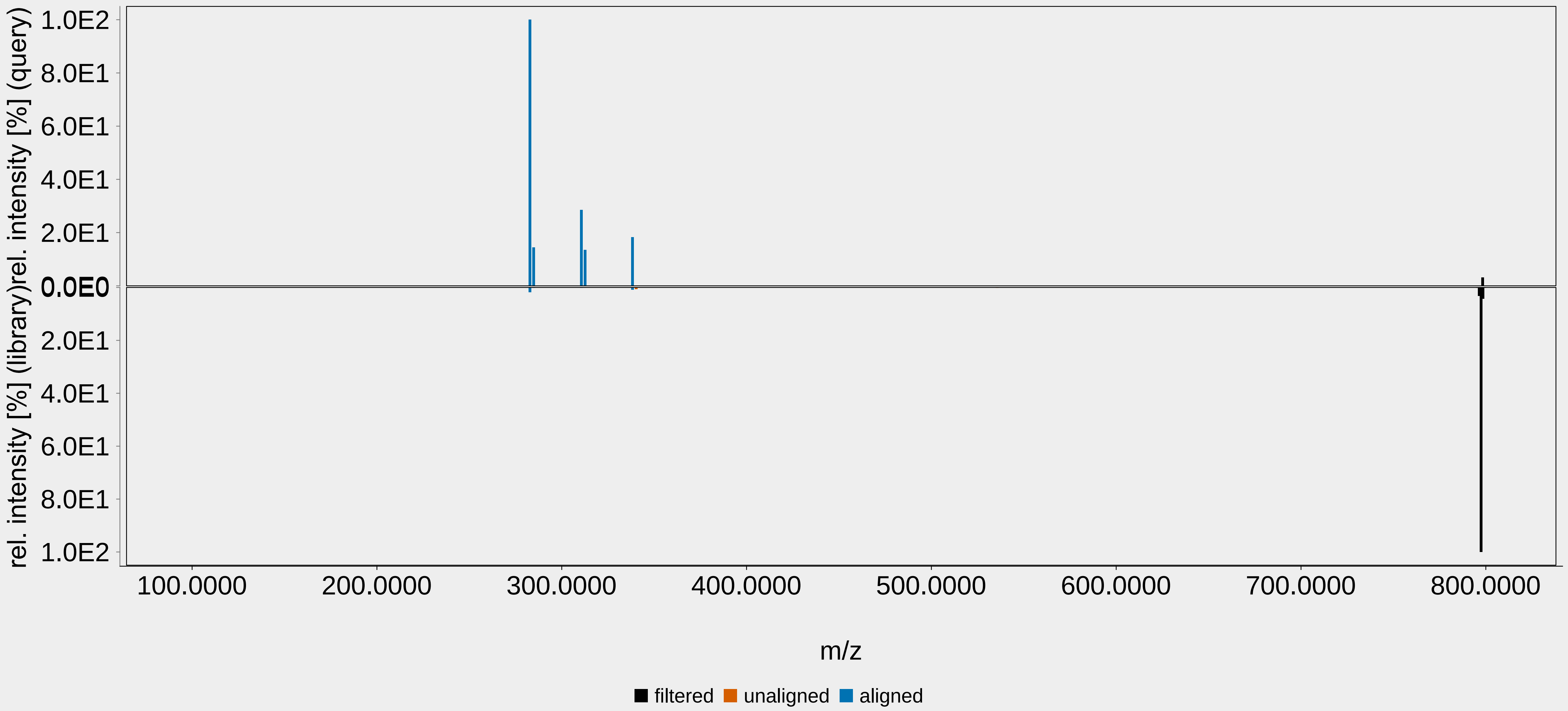

Compound information

Name: PE(18:1/22:0); [M-H]- C45H87N1O8P1

Exact mass: 0.0

Ion type: M-H

Precursor m/z: 800.617

Charge: 1

Polarity: Negative

Instrument information

Instrument type: HCD; Velos

Ion source: LC-ESI

Ms level: 2

Acquisition: Commercial

Database links

Other information

Principal investigator: Thomas Metz

Data collector: Thomas Metz

Entry ID: CCMSLIB00010074098

PE(18:1/22:0); [M-H]- C45H87N1O8P1 **0.785**

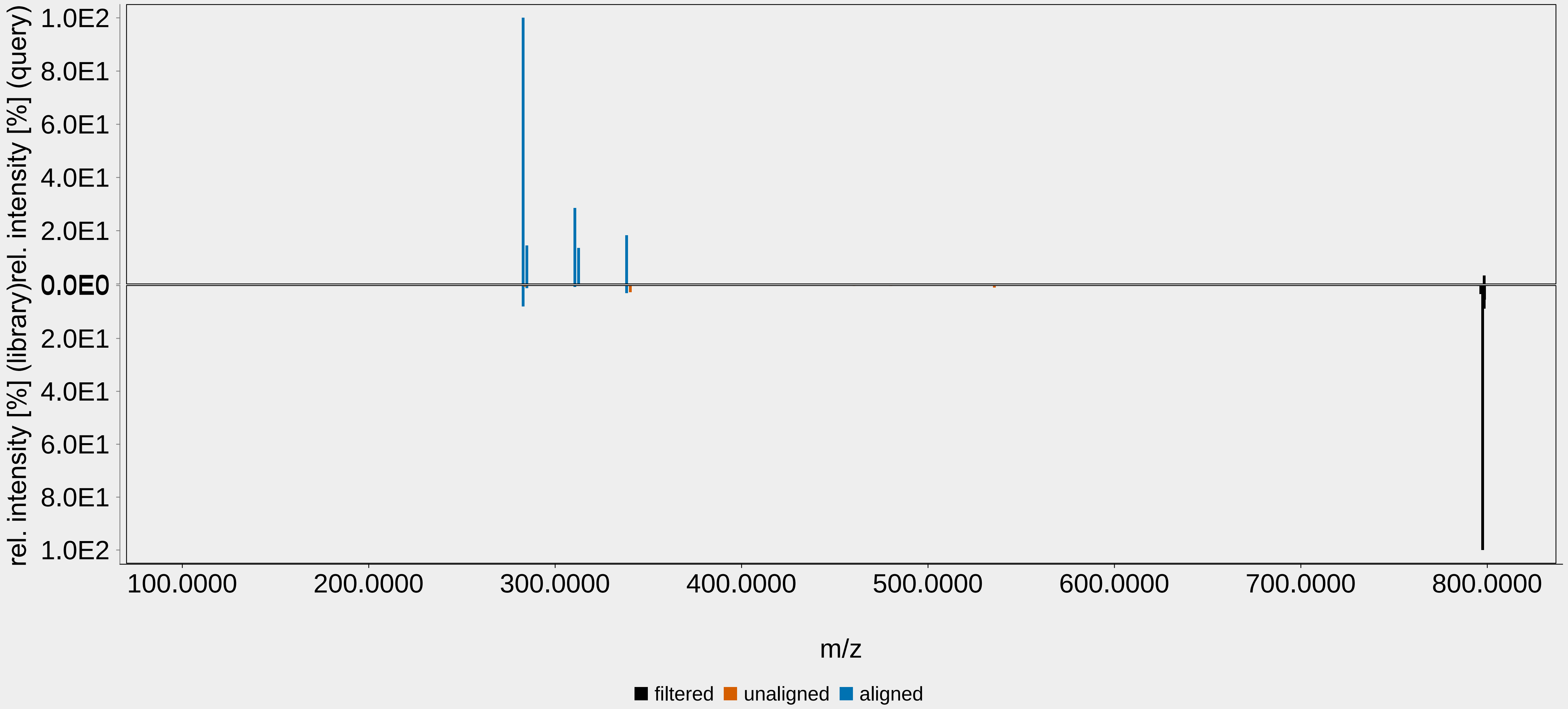

Compound information

Name: PE(18:1/22:0); [M-H]- C45H87N1O8P1

Exact mass: 0.0

Ion type: M-H

Precursor m/z: 800.617

Charge: 1

Polarity: Negative

Instrument information

Instrument type: HCD; Velos

Ion source: LC-ESI

Ms level: 2

Acquisition: Commercial

Database links

Other information

Principal investigator: Thomas Metz

Data collector: Thomas Metz

Entry ID: CCMSLIB00010087976

PE(18:0/22:4); [M-H]- C45H81N1O8P1 **0.864**

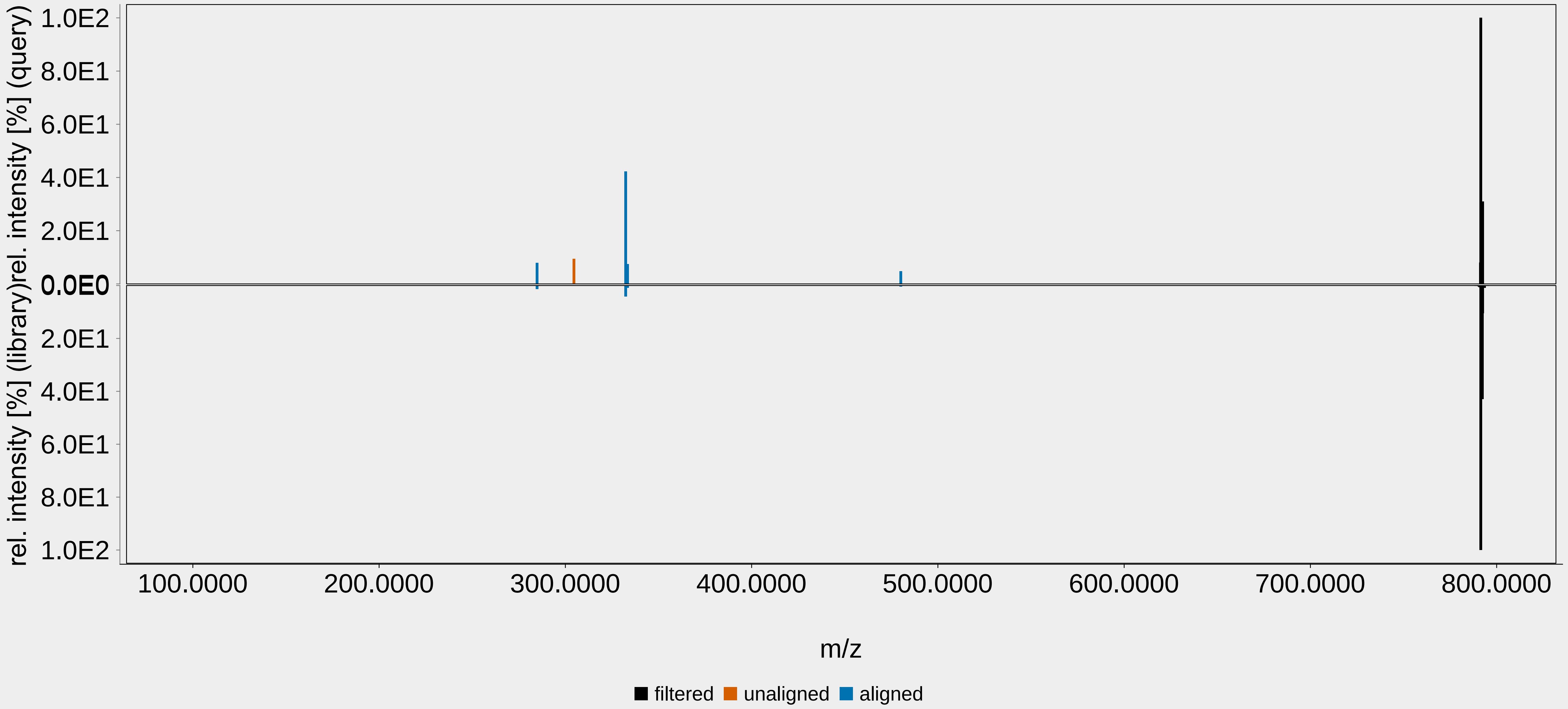

Compound information

Name: PE(18:0/22:4); [M-H]- C45H81N1O8P1

Exact mass: 0.0

Ion type: M-H

Precursor m/z: 794.57

Charge: 1

Polarity: Negative

Instrument information

Instrument type: HCD; Velos

Ion source: LC-ESI

Ms level: 2

Acquisition: Commercial

Database links

Other information

Principal investigator: Thomas Metz

Data collector: Thomas Metz

Entry ID: CCMSLIB00010085505

PE(18:0/22:4); [M-H]- C45H81N1O8P1 0.865

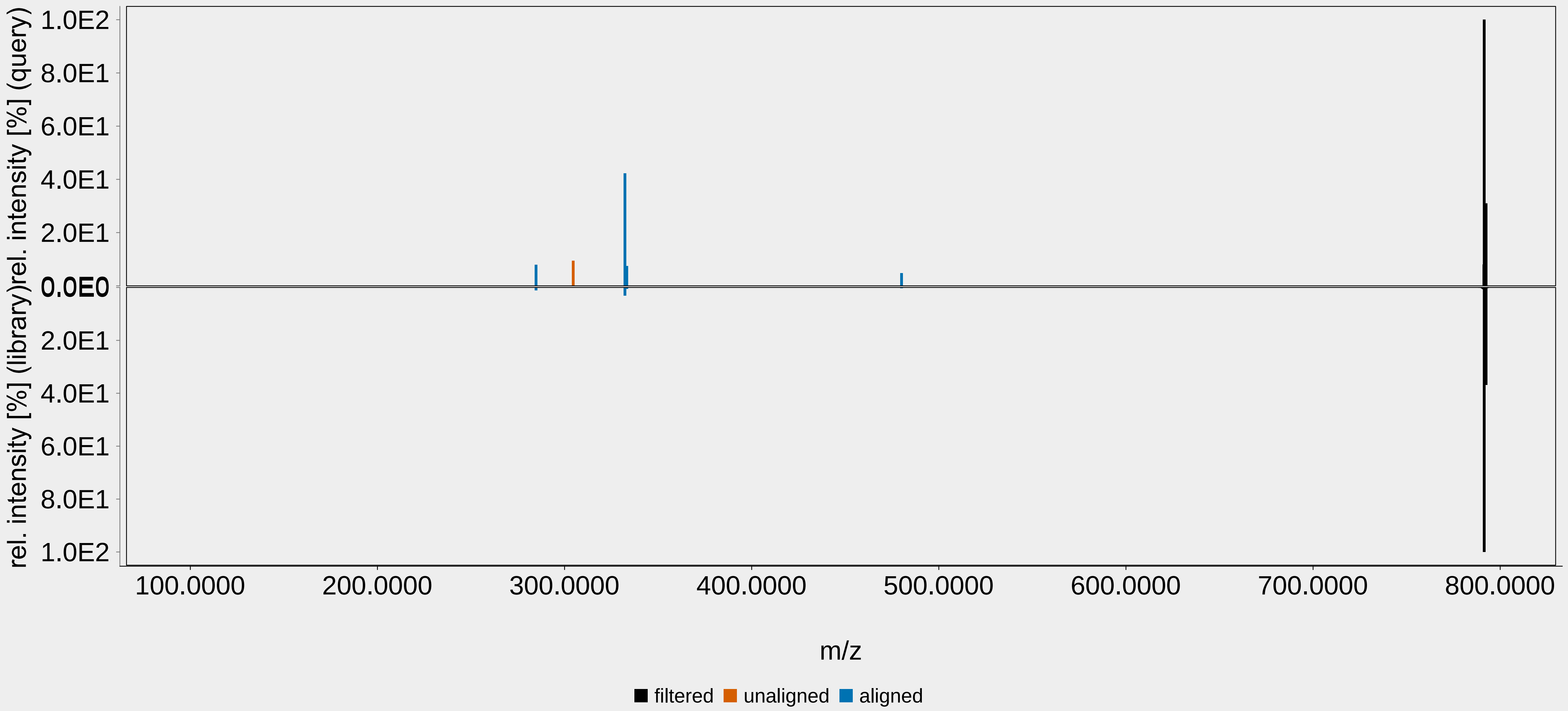

Compound information

Name: PE(18:0/22:4); [M-H]- C45H81N1O8P1

Exact mass: 0.0

Ion type: M-H

Precursor m/z: 794.57

Charge: 1

Polarity: Negative

Instrument information

Instrument type: HCD; Velos

Ion source: LC-ESI

Ms level: 2

Acquisition: Commercial

Database links

Other information

Principal investigator: Thomas Metz

Data collector: Thomas Metz

Entry ID: CCMSLIB00010083860

PS 38:4

0.855

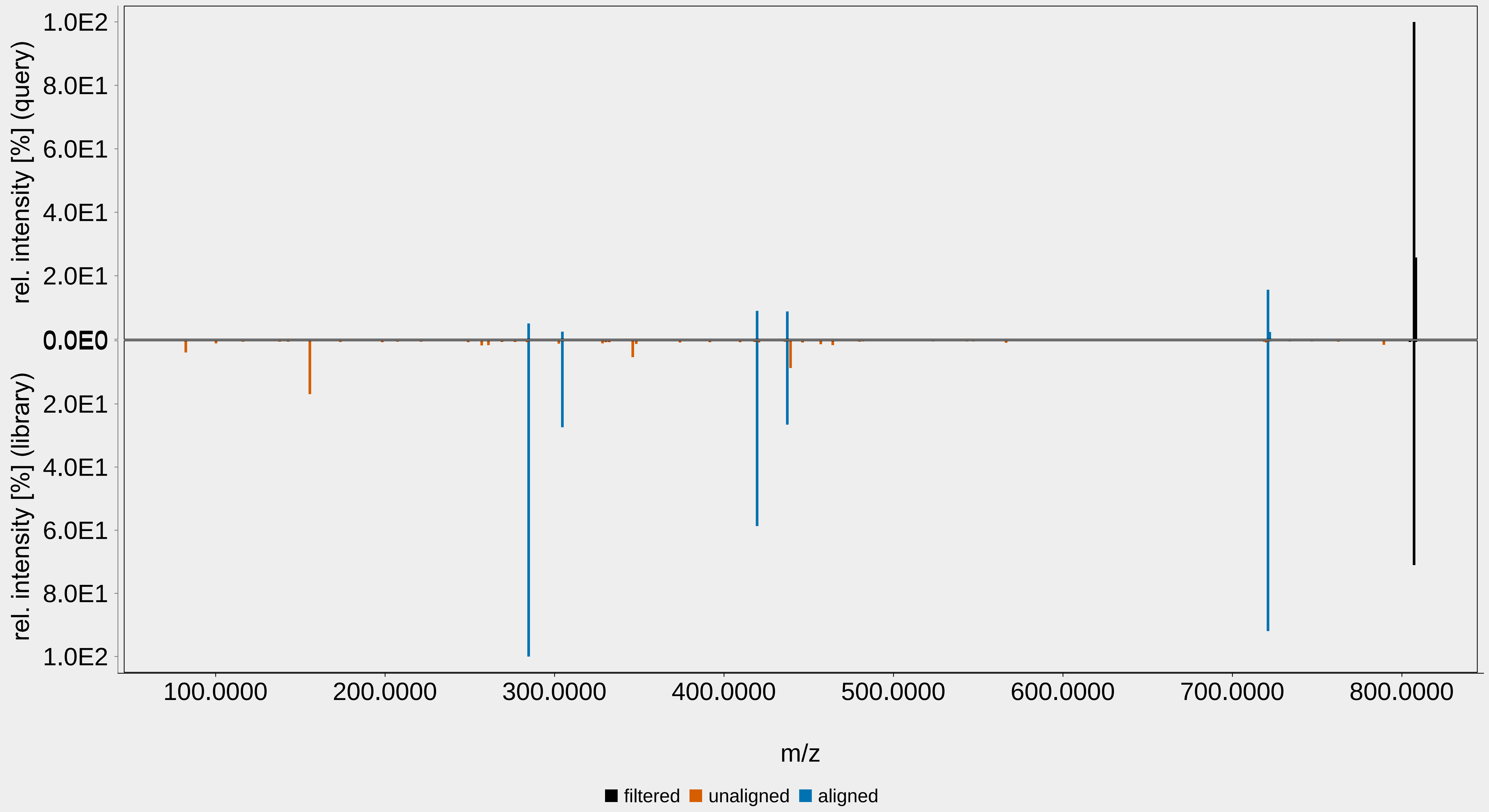

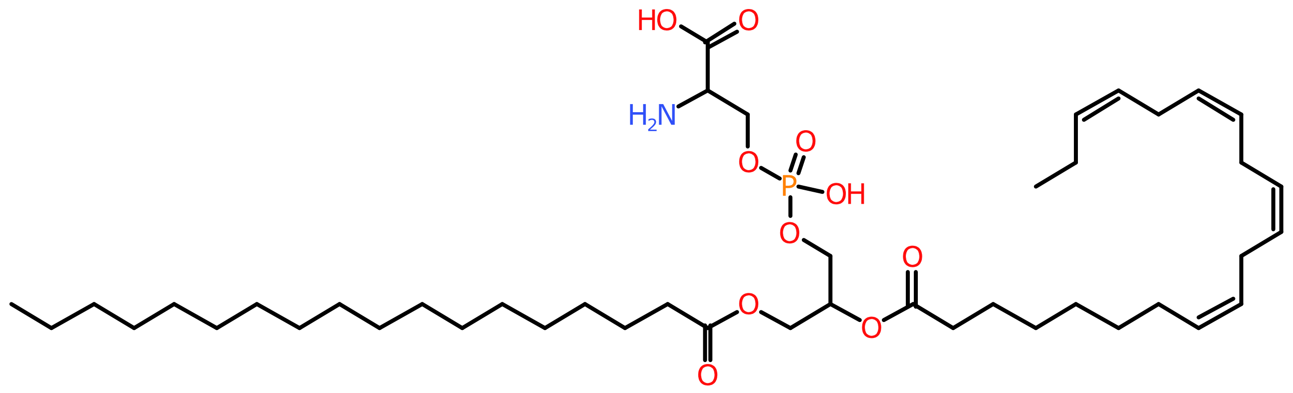

Compound information

Name: PS 38:4

Formula: C44H78NO10P

Mol. weight: 812.0809999999999

Exact mass: 812.0809999999999

Ion type: [M-H]-

Precursor m/z: 810.5303

RT: 8.778674

Polarity: negative

InChI:

InChI key:

SMILES:

Instrument information

Instrument type: LC-ESI-QTOF

Instrument: AB Sciex TripleTOF 5600+ system

Ms level: MS2

Collision energy: 45V

Database links

Pubchem: 138181120

MoNA ID: LQB00722

Other information

Principal investigator: Kazutaka Ikeda, Hiroshi

Data collector: Kazutaka Ikeda, Hiroshi

PS 38:4

0.855

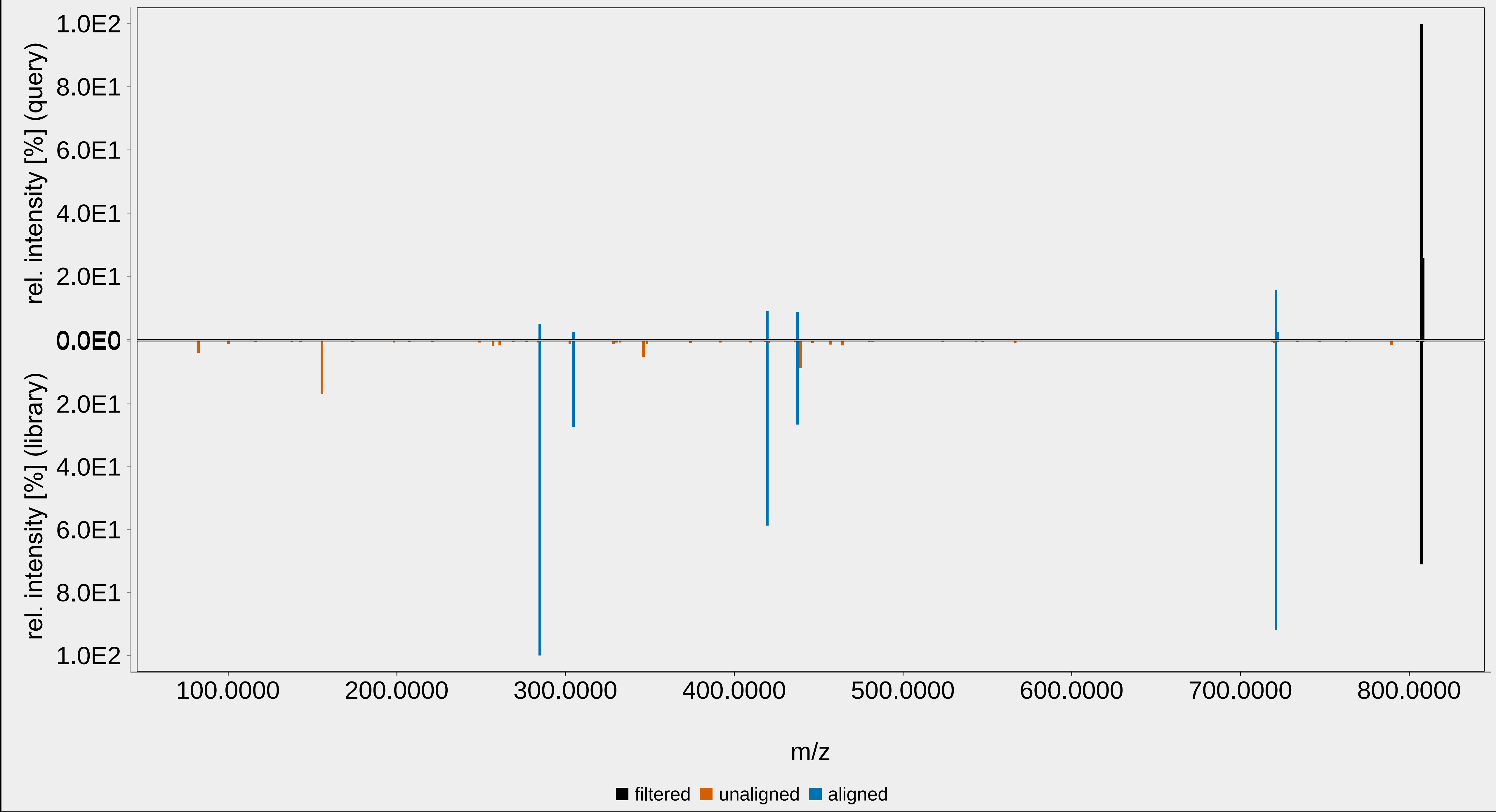

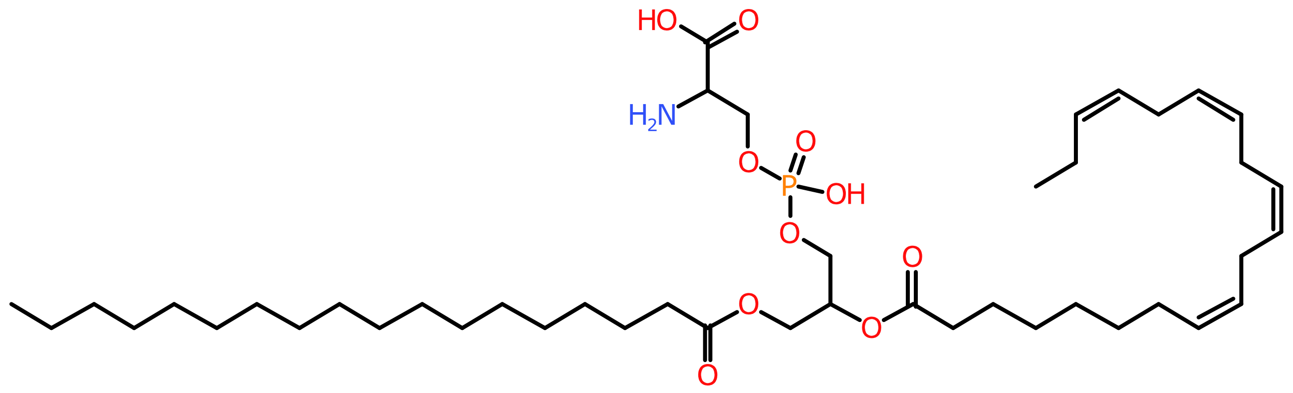

Compound information

Name: PS 38:4

Formula: C44H78NO10P

Mol. weight: 812.0809999999999

Exact mass: 812.0809999999999

Ion type: [M-H]-

Precursor m/z: 810.5303

RT: 8.778674

Polarity: negative

InChI:

InChI key:

SMILES:

Instrument information

Instrument type: LC-ESI-QTOF

Instrument: AB Sciex TripleTOF 5600+ system

Ms level: MS2

Collision energy: 45V

Database links

Pubchem: 138181120

MoNA ID: LQB00336

Other information

Principal investigator: Kazutaka Ikeda, Hiroshi

Data collector: Kazutaka Ikeda, Hiroshi

Spectral Match to cis-4,10,13,16-Docosatetraenoic acid from NIST14

0.707

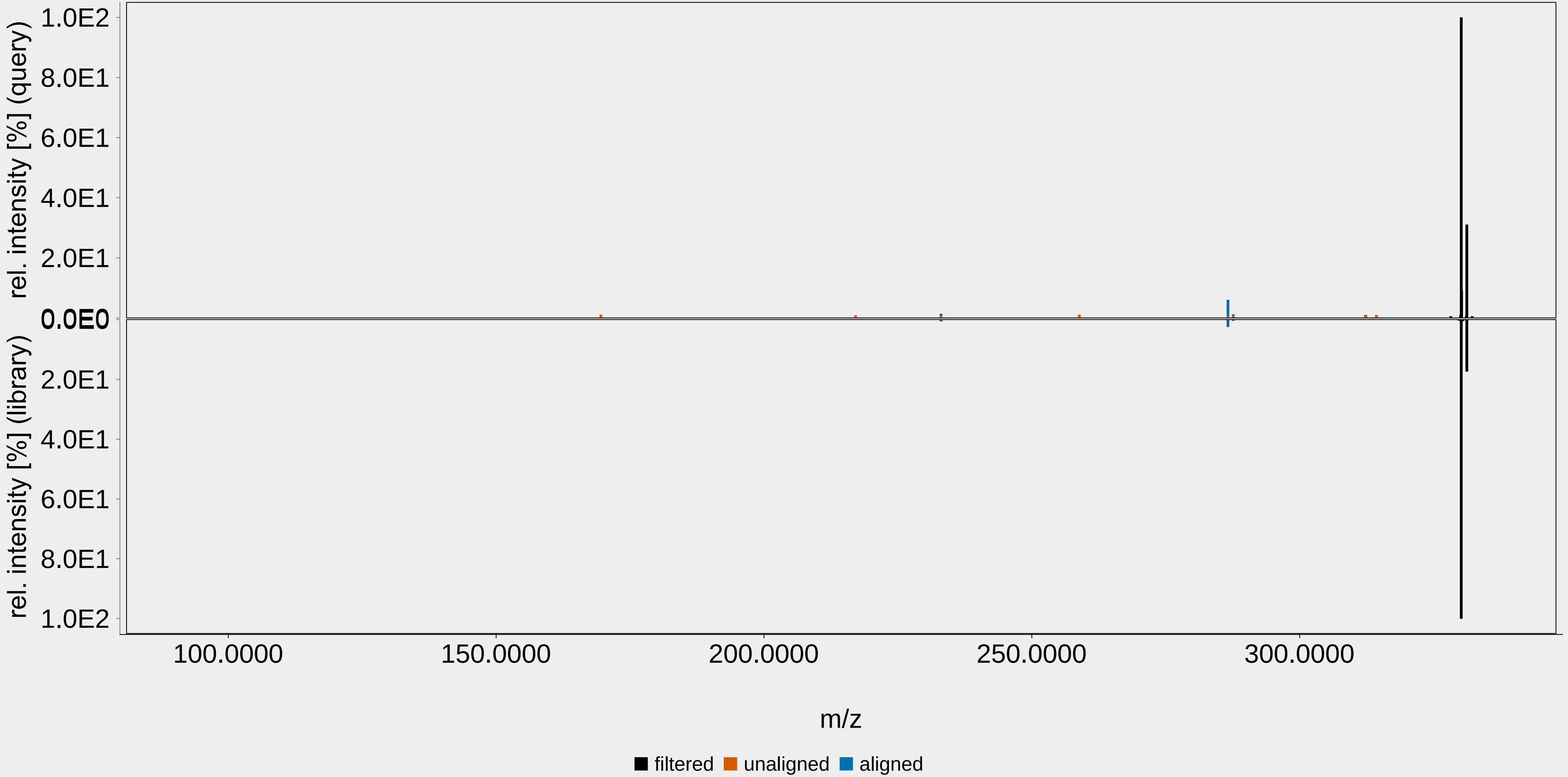

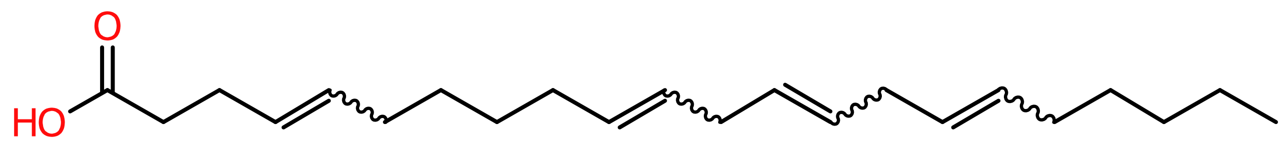

Compound information

Name: Spectral Match to

Exact mass: 332.272

Ion type: M-H

Precursor m/z: 331.263

Charge: 1

Polarity: Negative

InChI:

SMILES:

Instrument information

Instrument type: Ion Trap

Ion source: ESI

Ms level: 2

Acquisition: Isolated

Database links

CAS: 122068080

Other information

Principal investigator: Data from Charles

Data collector: Data deposited by alchemistmatt

Entry ID: CCMSLIB00003138332

PS(18:0_18:1) - 2-amino-3-({hydroxy[2-[octadec-9-enoyloxy]-3-(octadecanoyloxy)propoxy]phosphoryl}oxy)propanoic acid

0.896

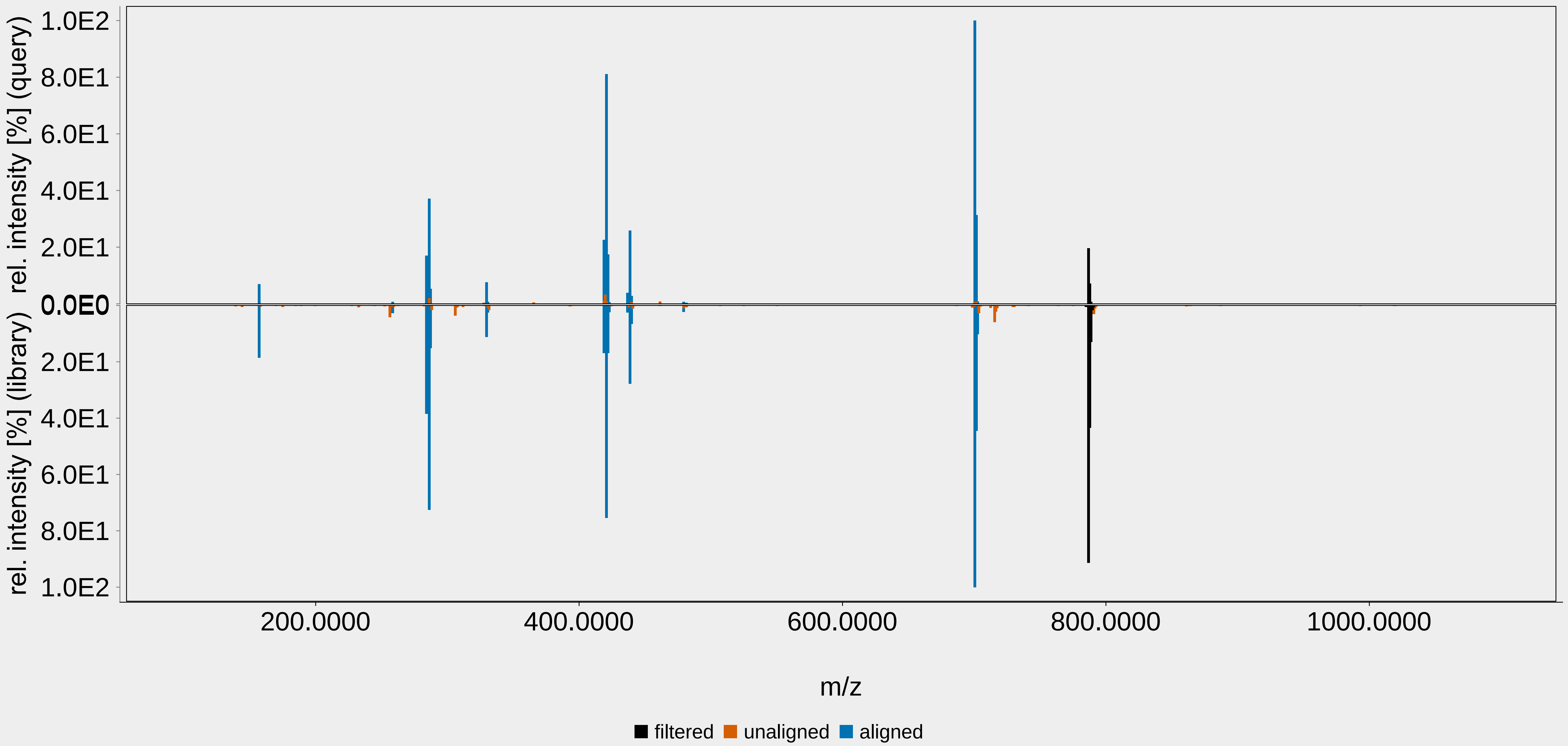

Compound information

Name: PS(18:0_18:1) -

Exact mass: 789.552

Ion type: M-H

Precursor m/z: 788.542

Charge: 1

Polarity: Negative

SMILES:

Instrument information

Instrument type: qTof

Ion source: LC-ESI

Ms level: 2

Acquisition: Lysate

Database links

CAS: 124262-93-7

Other information

Principal investigator: O Laprevote

Data collector: C-TAC

Entry ID: CCMSLIB00005720987

2-amino-3-({hydroxy[2-[octadec-9-enoyloxy]-3-(octadecanoyloxy)propoxy]phosphoryl}oxy)propanoic acid

0.896

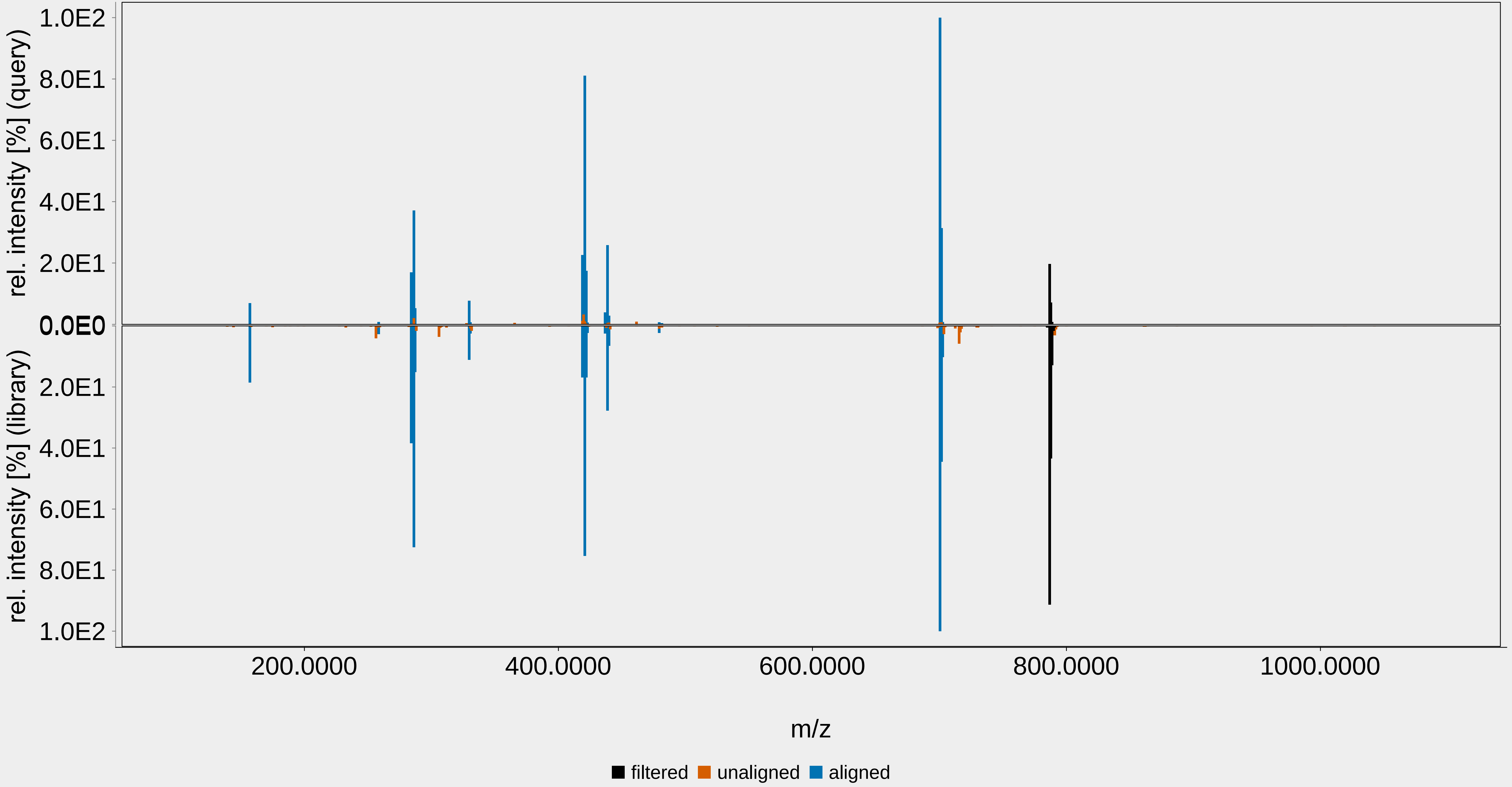

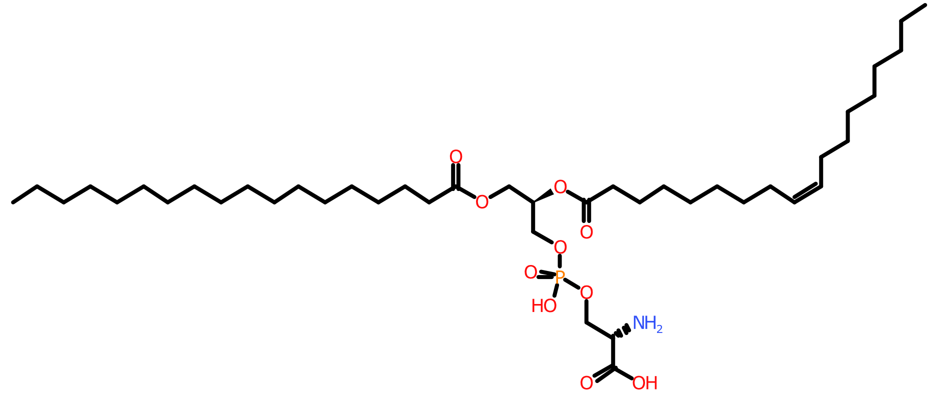

Compound information

Name:

Formula: C42H80NO10P

Mol. weight: 789.552

Exact mass: 789.552

Ion type: [M-H]-

Precursor m/z: 788.542

Polarity: negative

InChI:

InChI key:

SMILES:

Instrument information

Instrument: qTof

Ms level: MS2

Database links

Other information

Principal investigator: O Laprevote,

Data collector: O Laprevote, mpanitchpakdi,

Massbank:LQB00564 PE 36:4

0.947

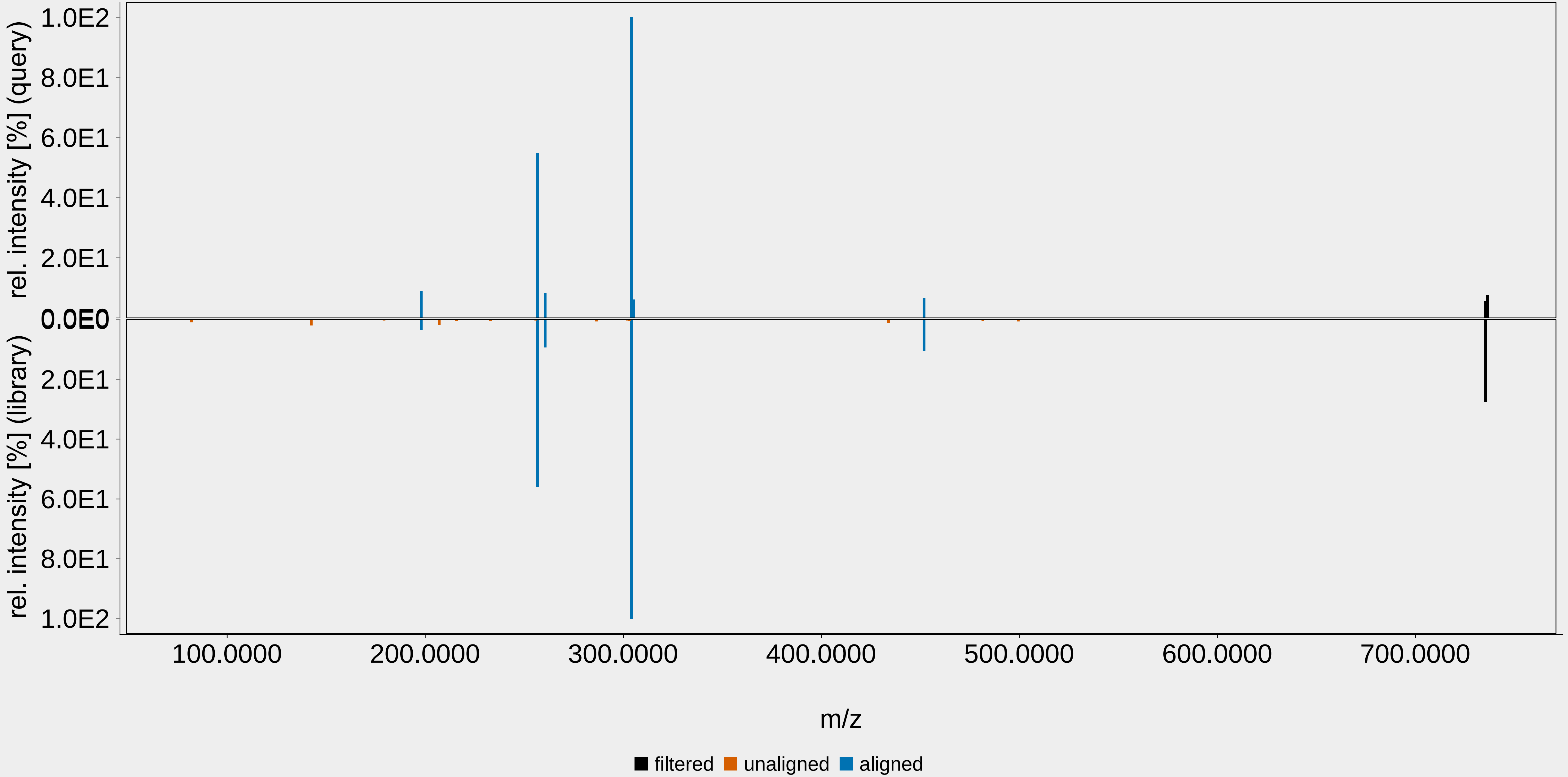

Compound information

Name: Massbank:LQB00564 PE 36:4

Exact mass: 0.0

Ion type: M-H

Precursor m/z: 738.503

Charge: 1

Polarity: Negative

InChI:

SMILES:

Instrument information

Instrument type: qTof

Ion source: ESI

Ms level: 2

Acquisition: Isolated

Database links

Other information

Principal investigator: Massbank

Data collector: Massbank

Entry ID: CCMSLIB00005737665

Massbank:LQB00178 PE 36:4

0.947

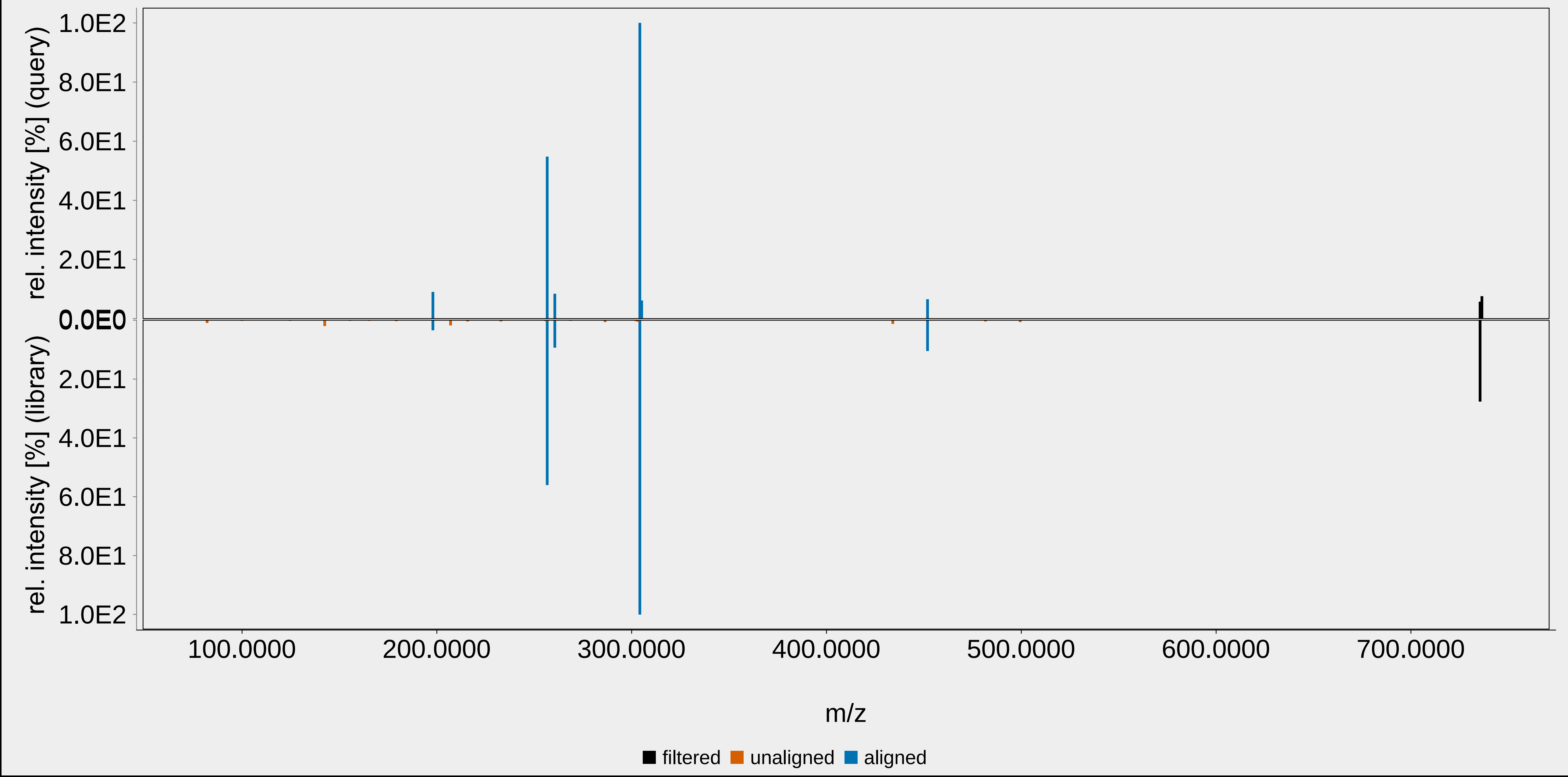

Compound information

Name: Massbank:LQB00178 PE 36:4

Exact mass: 0.0

Ion type: M-H

Precursor m/z: 738.503

Charge: 1

Polarity: Negative

InChI:

SMILES:

Instrument information

Instrument type: qTof

Ion source: ESI

Ms level: 2

Acquisition: Isolated

Database links

Other information

Principal investigator: Massbank

Data collector: Massbank

Entry ID: CCMSLIB00005737734

PE(16:0/18:1); [M-H]- C39H75N1O8P1 **0.930**

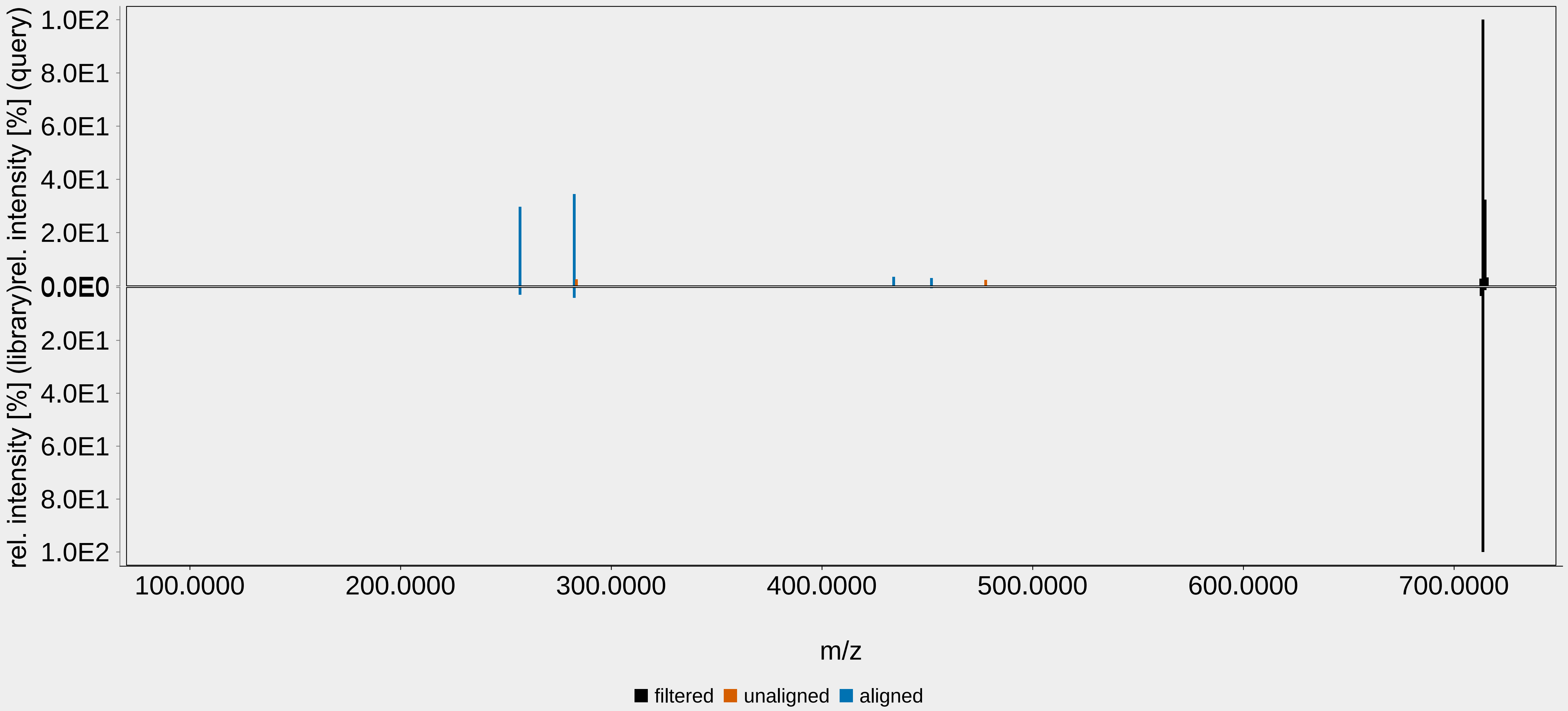

Compound information

Name: PE(16:0/18:1); [M-H]- C39H75N1O8P1

Exact mass: 0.0

Ion type: M-H

Precursor m/z: 716.523

Charge: 1

Polarity: Negative

Instrument information

Instrument type: HCD; Velos

Ion source: LC-ESI

Ms level: 2

Acquisition: Commercial

Database links

Other information

Principal investigator: Thomas Metz

Data collector: Thomas Metz

Entry ID: CCMSLIB00010072728

PE(16:0_18:1) - (2-aminoethoxy)[3-(hexadecanoyloxy)-2-[octadec-9-enoyloxy]propoxy]phosphinic acid

0.942

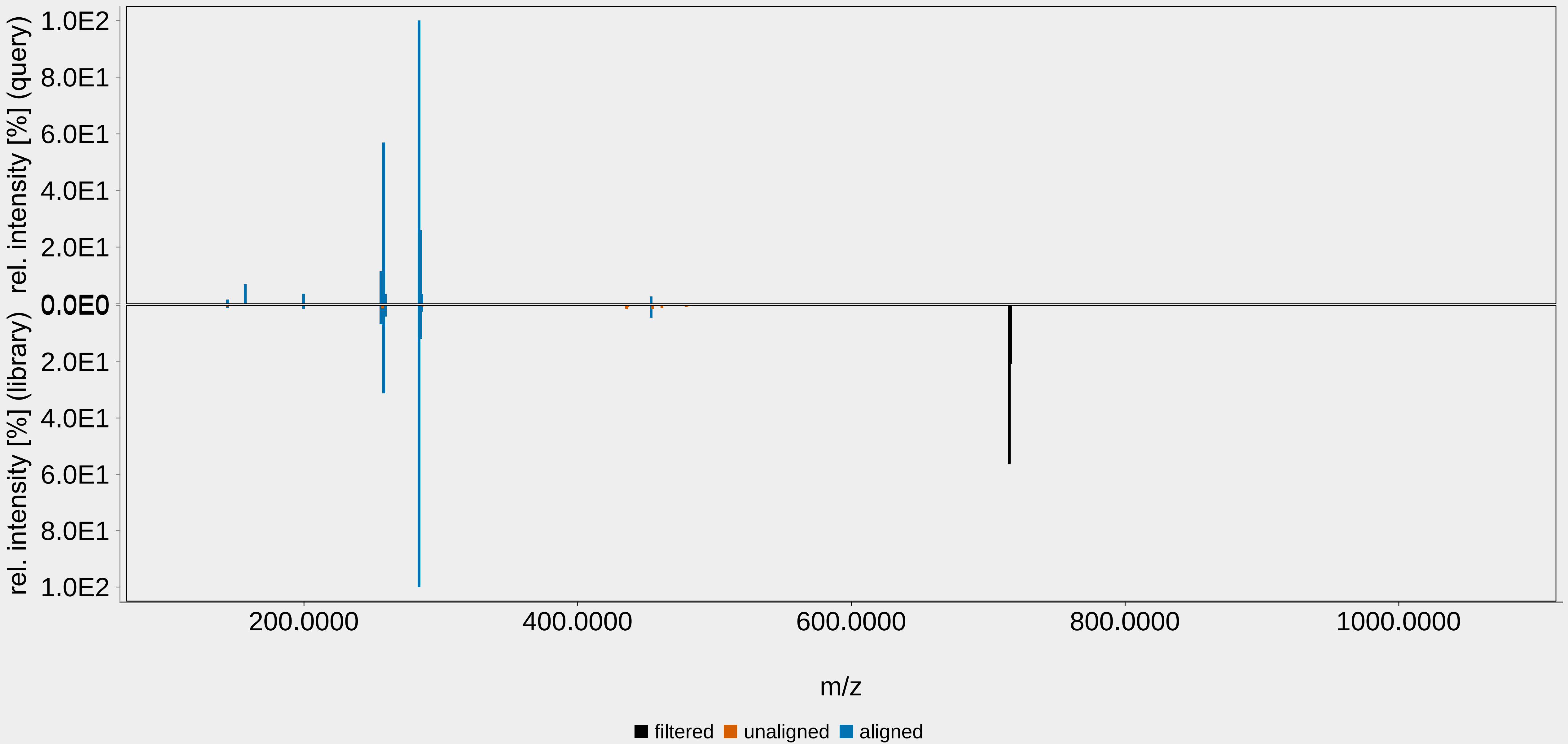

Compound information

Name: PE(16:0_18:1) -

Exact mass: 717.531

Ion type: M-H

Precursor m/z: 716.522

Charge: 1

Polarity: Negative

SMILES:

Instrument information

Instrument type: qTof

Ion source: LC-ESI

Ms level: 2

Acquisition: Lysate

Database links

CAS: 26662-94-2

Other information

Principal investigator: N Auzeil

Data collector: UMR CNRS 8038 Universite de

Entry ID: CCMSLIB00005724205

PA 22:0_18:1 **0.786**

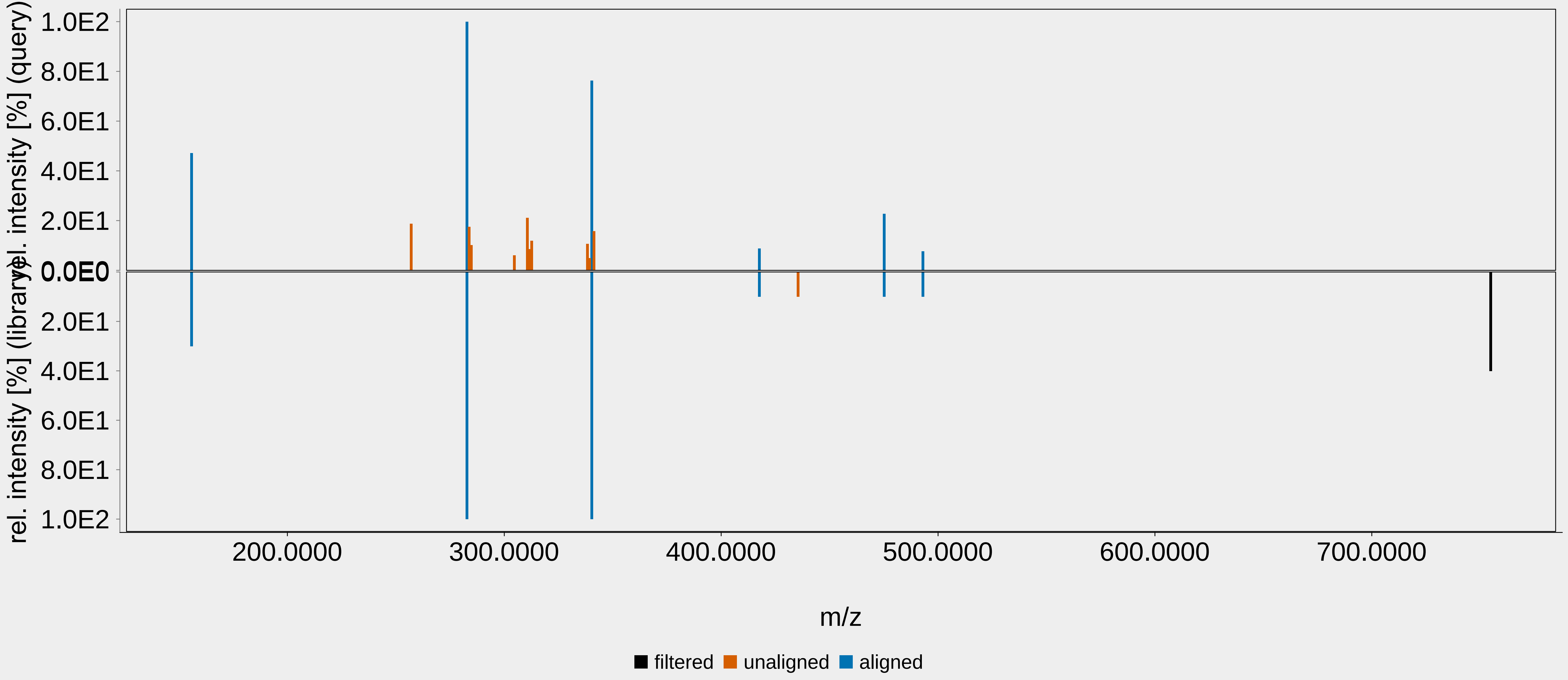

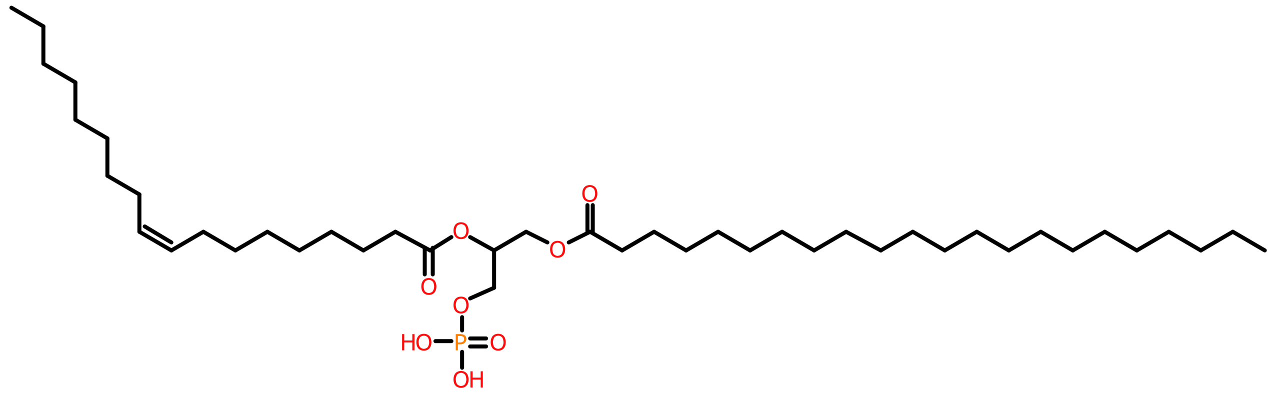

Compound information

Name: PA 22:0_18:1

Formula: C43H83O8P

Ion type: [M-H]-

Precursor m/z: 757.57528

Ccs: 273.22

InChI key:

SMILES:

Num peaks: 8

Instrument information

Database links

Other information

PS 22:4_22:6 **0.706**

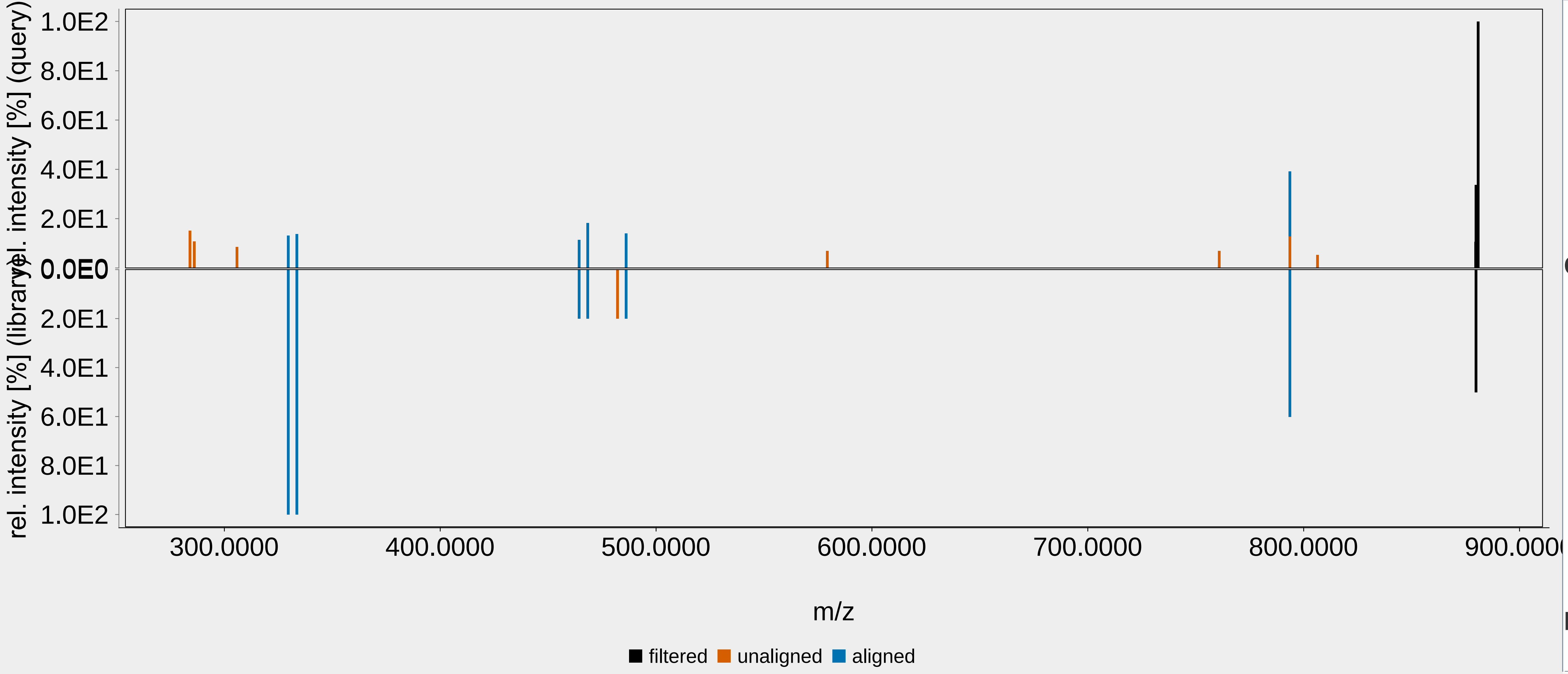

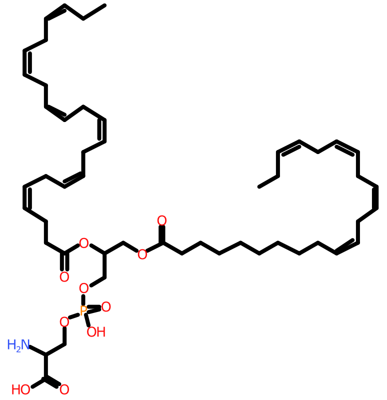

Compound information

Name: PS 22:4_22:6

Formula: C50H78NO10P

Ion type: [M-H]-

Precursor m/z: 882.52906

Ccs: 284.42

InChI key:

SMILES:

Num peaks: 8

Instrument information

Database links

Other information

PI 38:4

0.925

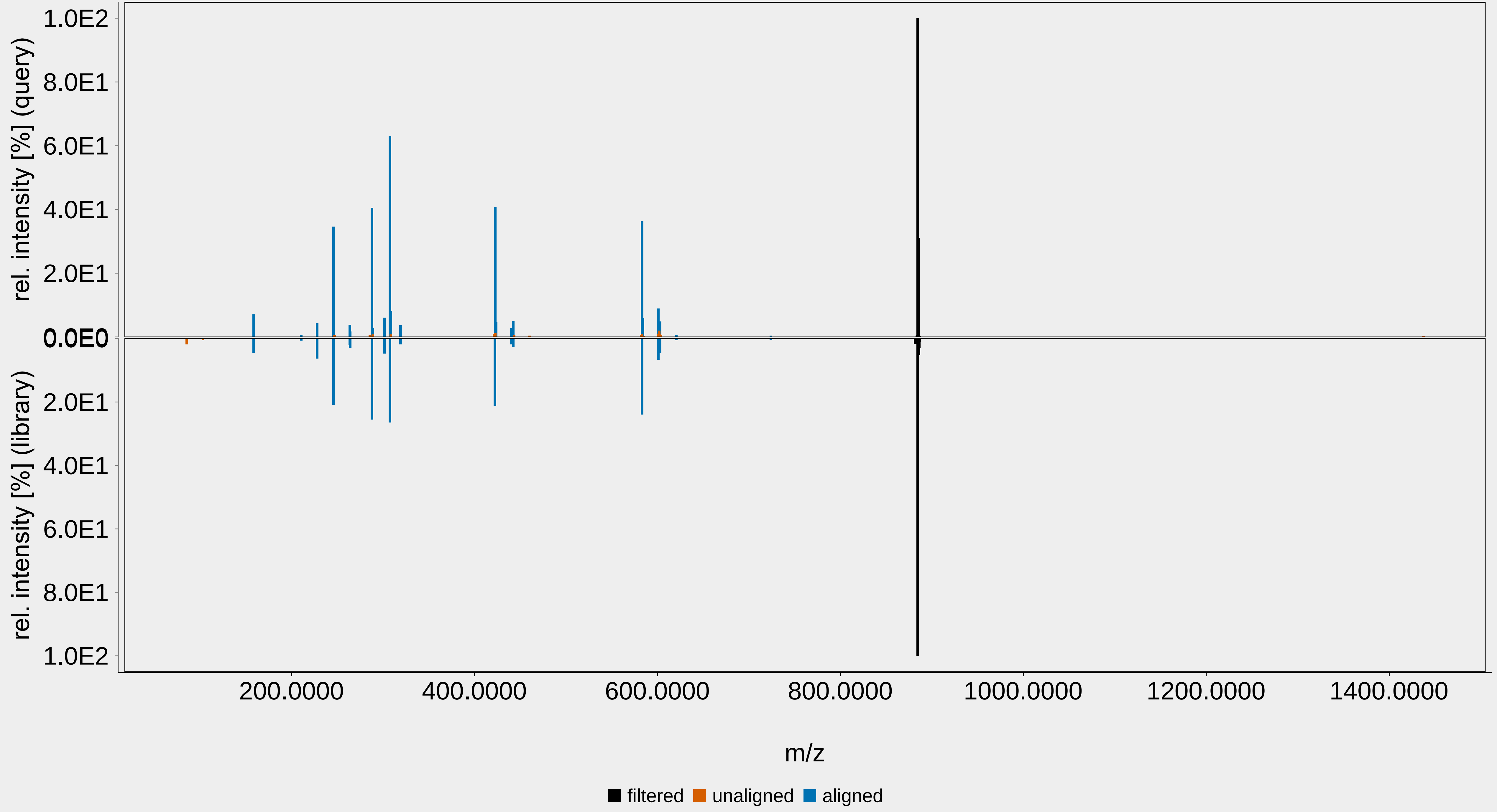

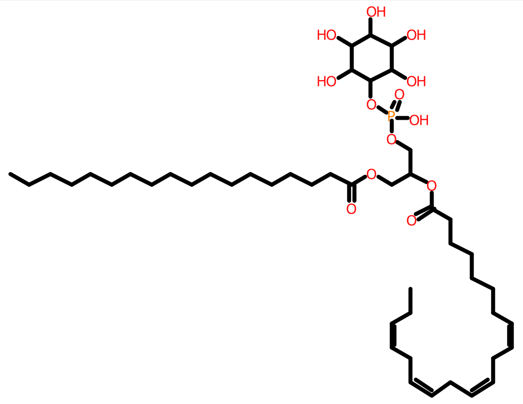

Compound information

Name: PI 38:4

Formula: C47H83O13P

Mol. weight: 887.1439999999999

Exact mass: 887.1439999999999

Ion type: [M-H]-

Precursor m/z: 885.5508

RT: 8.754183

Polarity: negative

InChI:

InChI key:

SMILES:

Instrument information

Instrument type: LC-ESI-QTOF

Instrument: AB Sciex TripleTOF 5600+ system

Ms level: MS2

Collision energy: 45V

Database links

Pubchem: 138227586

MoNA ID: LQB00694

Other information

Principal investigator: Kazutaka Ikeda, Hiroshi

Data collector: Kazutaka Ikeda, Hiroshi

PI 38:4

0.925

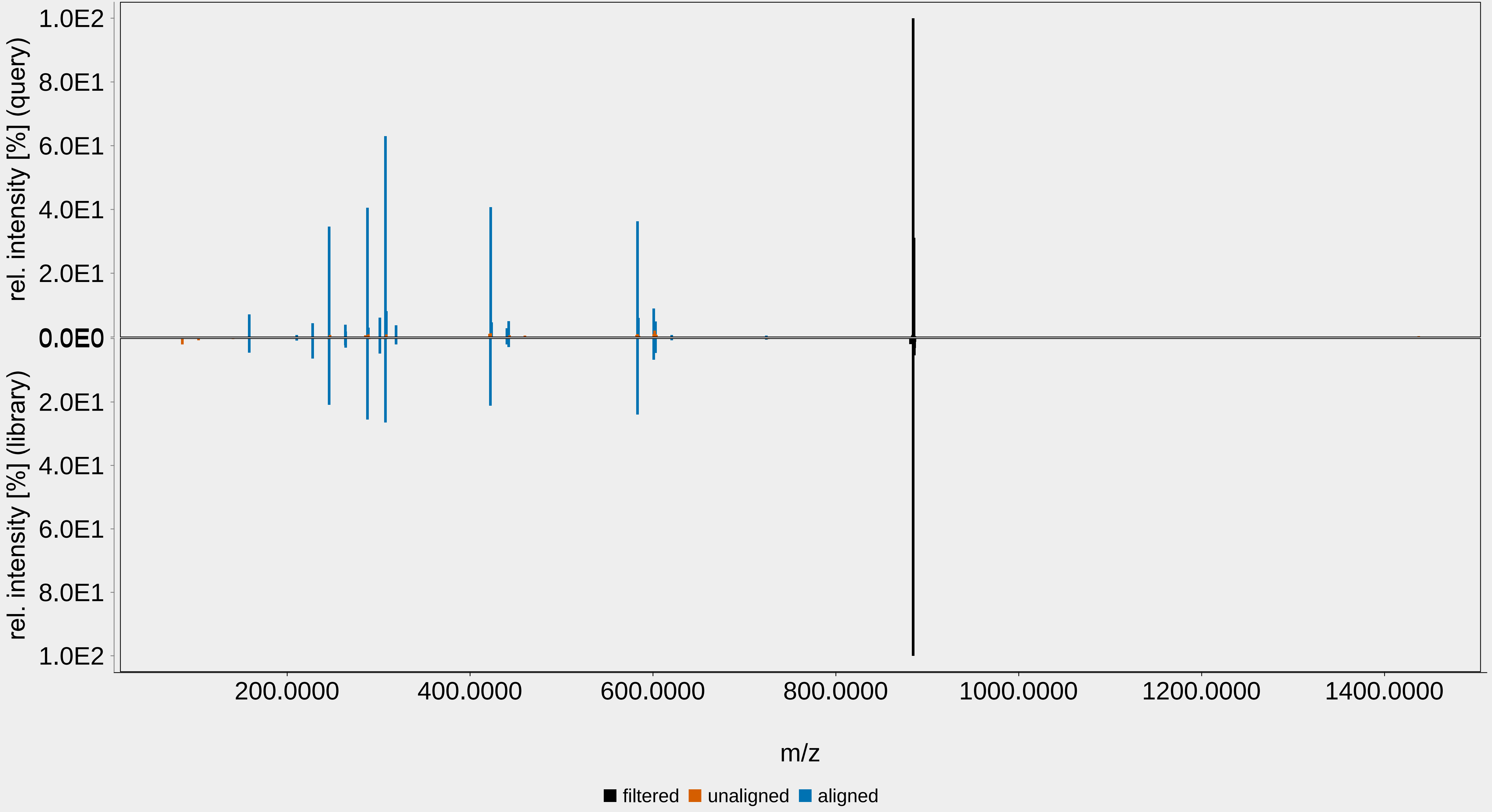

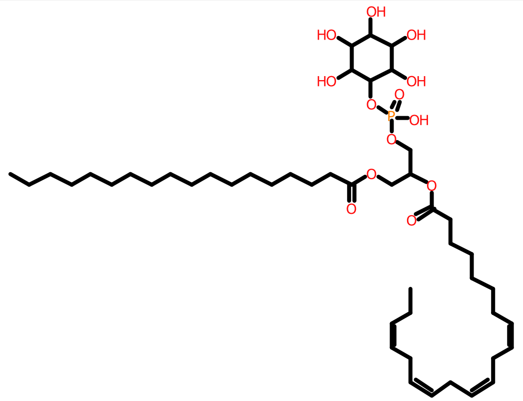

Compound information

Name: PI 38:4

Formula: C47H83O13P

Mol. weight: 887.1439999999999

Exact mass: 887.1439999999999

Ion type: [M-H]-

Precursor m/z: 885.5508

RT: 8.754183

Polarity: negative

InChI:

InChI key:

SMILES:

Instrument information

Instrument type: LC-ESI-QTOF

Instrument: AB Sciex TripleTOF 5600+ system

Ms level: MS2

Collision energy: 45V

Database links

Pubchem: 138227586

MoNA ID: LQB00308

Other information

Principal investigator: Kazutaka Ikeda, Hiroshi

Data collector: Kazutaka Ikeda, Hiroshi

Uridine 5'-diphospho-N-acetylglucosamine

0.785

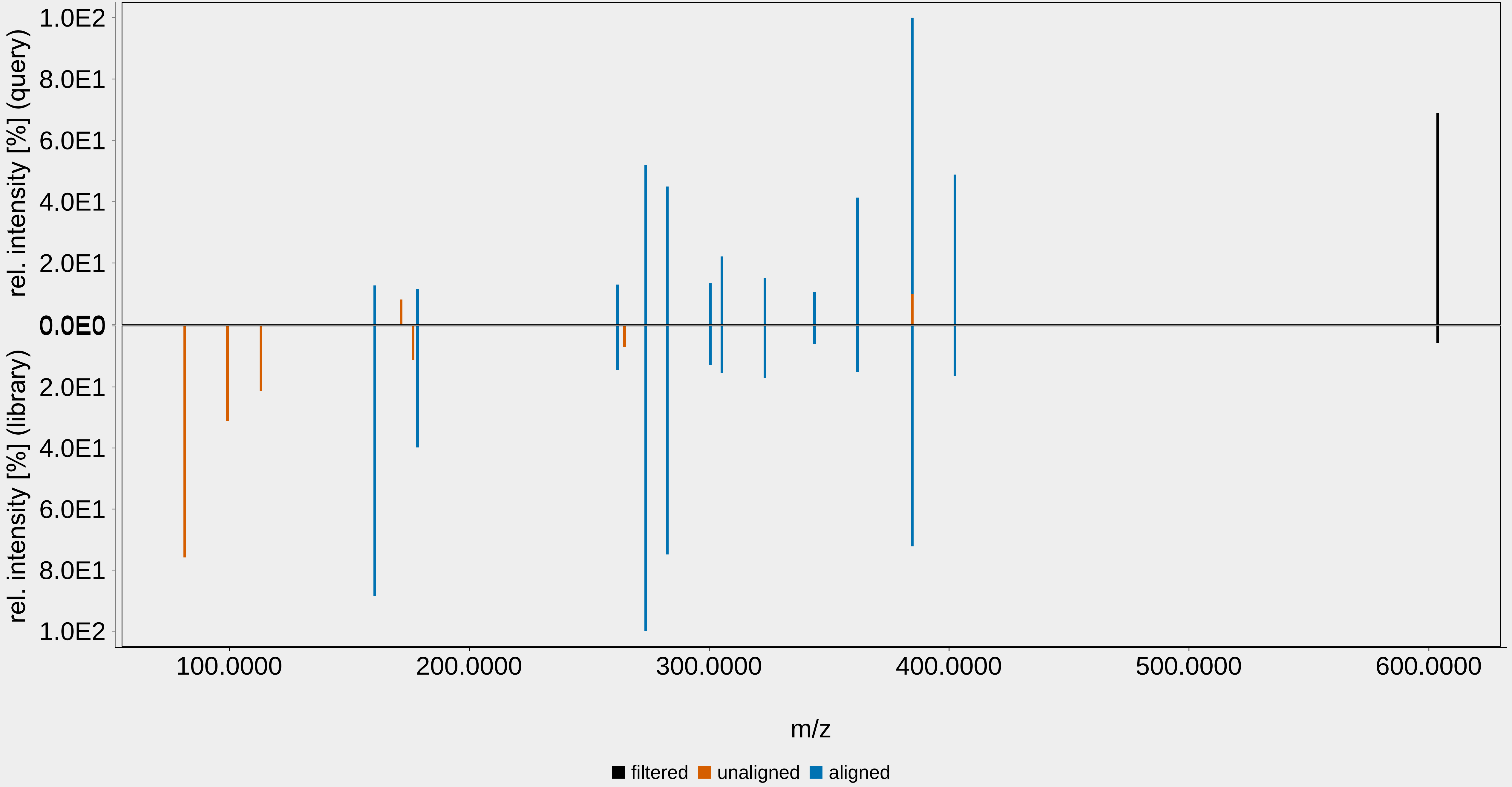

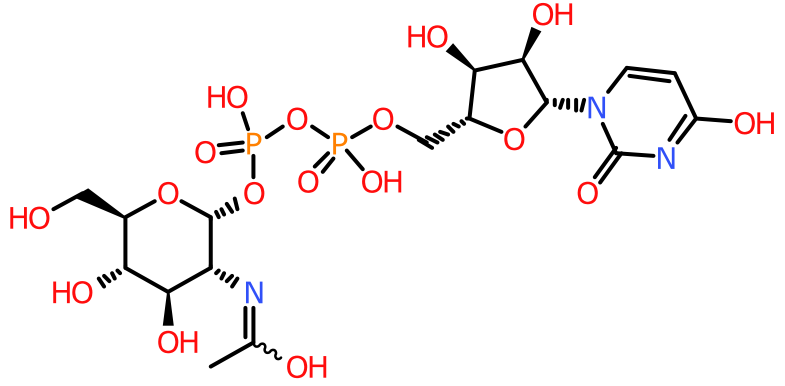

Compound information

Name: Uridine

Formula: C17H27N3O17P2

Exact mass: 607.08157

Ion type: [M-H]-

Precursor m/z: 606.07377

Polarity: NEGATIVE

InChI:

InChI key:

SMILES:

Num peaks: 18

Instrument information

Instrument type: LC-ESI-QTOF

Instrument: UPLC Q-Tof Premier, Waters

Ms level: MS2

Collision energy: 30 V

Database links

Other information

Entry ID: MSBNK-RIKEN-PR100621

Comment: Parent=606.07377

[(2R,3R,4R,5S,6R)-3-acetamido-4,5-dihydroxy-6-(hydroxymethyl)oxan-2-yl] [[(2R,3S,4R,5R)-5-(2,4-dioxopyrimidin-1-yl)-3,4-dihydroxyoxolan-2-yl]methoxy-hydroxyphosphoryl]

0.785

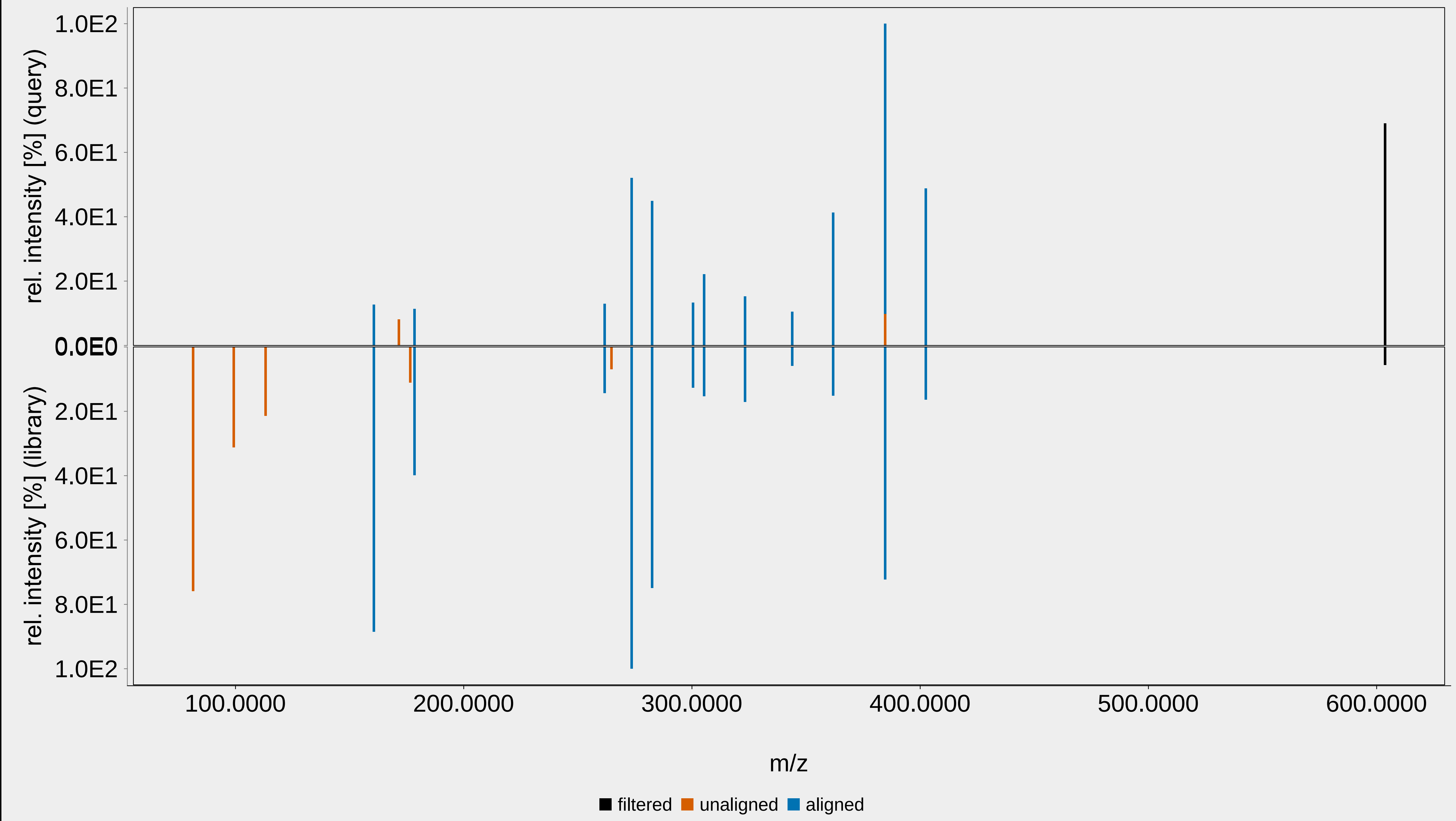

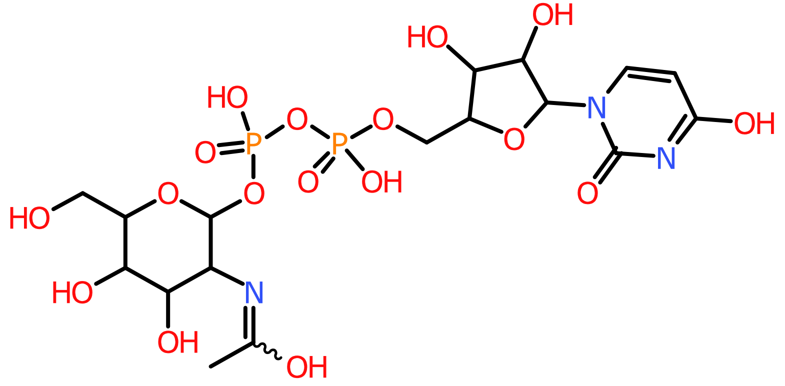

Compound information

Name:

Formula: C17H27N3O17P2

Exact mass: 607.359

Ion type: [M+H]+

Precursor m/z: 606.07377

Polarity: NEGATIVE

InChI:

InChI key:

SMILES:

Num peaks: 18

Instrument information

Instrument type: LC-ESI-QTOF

Instrument: Q-Tof Premier, Waters

Ms level: MS2

Collision energy: 30 V

Database links

Other information

Entry ID: MSBNK-RIKEN_ReSpect-PT203703

Comment: Parent=606.07377

PE(18:0/0:0); [M-H]- C23H47N1O7P1 **0.746**

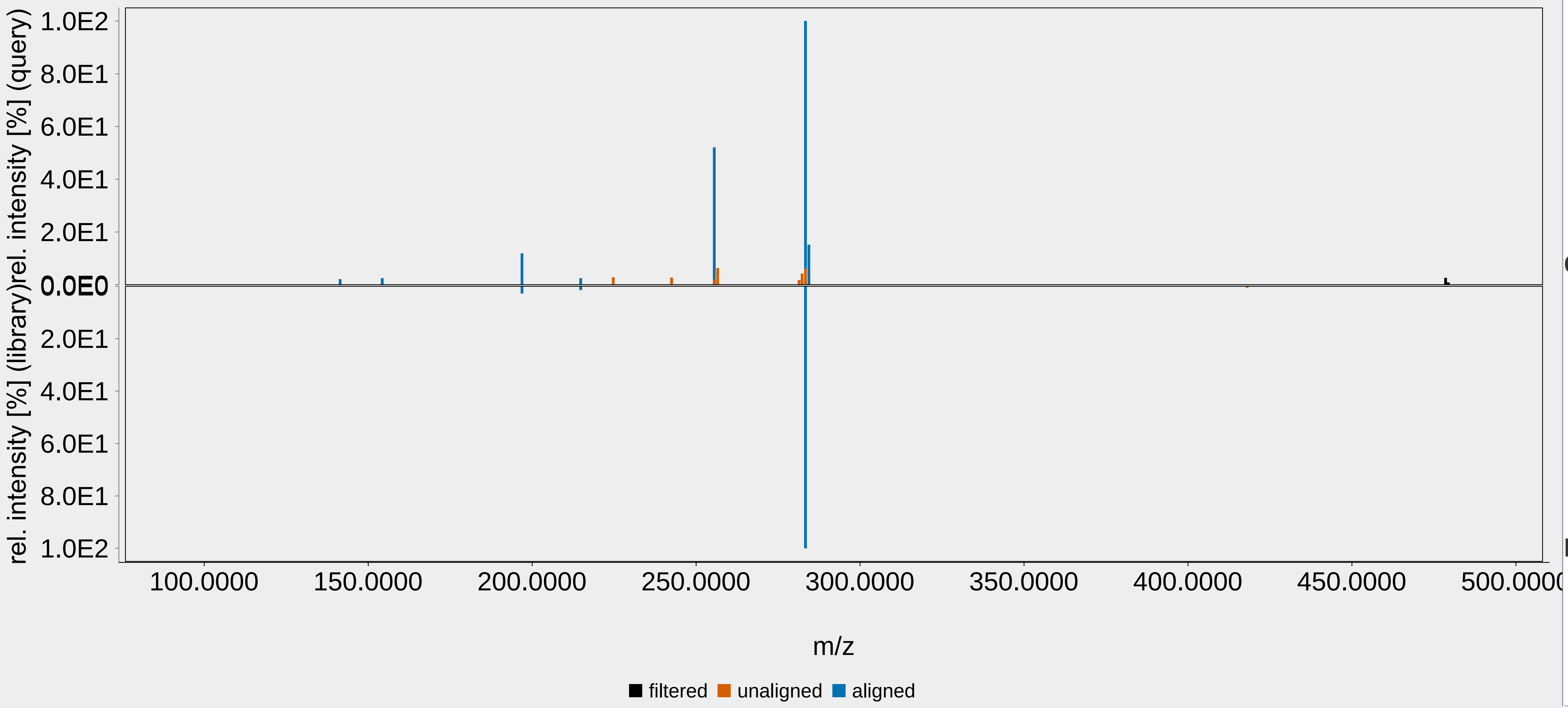

Compound information

Name: PE(18:0/0:0); [M-H]- C23H47N1O7P1

Exact mass: 0.0

Ion type: M-H

Precursor m/z: 480.309

Charge: 1

Polarity: Negative

Instrument information

Instrument type: CID; Lumos

Ion source: LC-ESI

Ms level: 2

Acquisition: Commercial

Database links

Other information

Principal investigator: Thomas Metz

Data collector: Thomas Metz

Entry ID: CCMSLIB00010059720

PE(18:0/0:0); [M-H]- C23H47N1O7P1 **0.760**

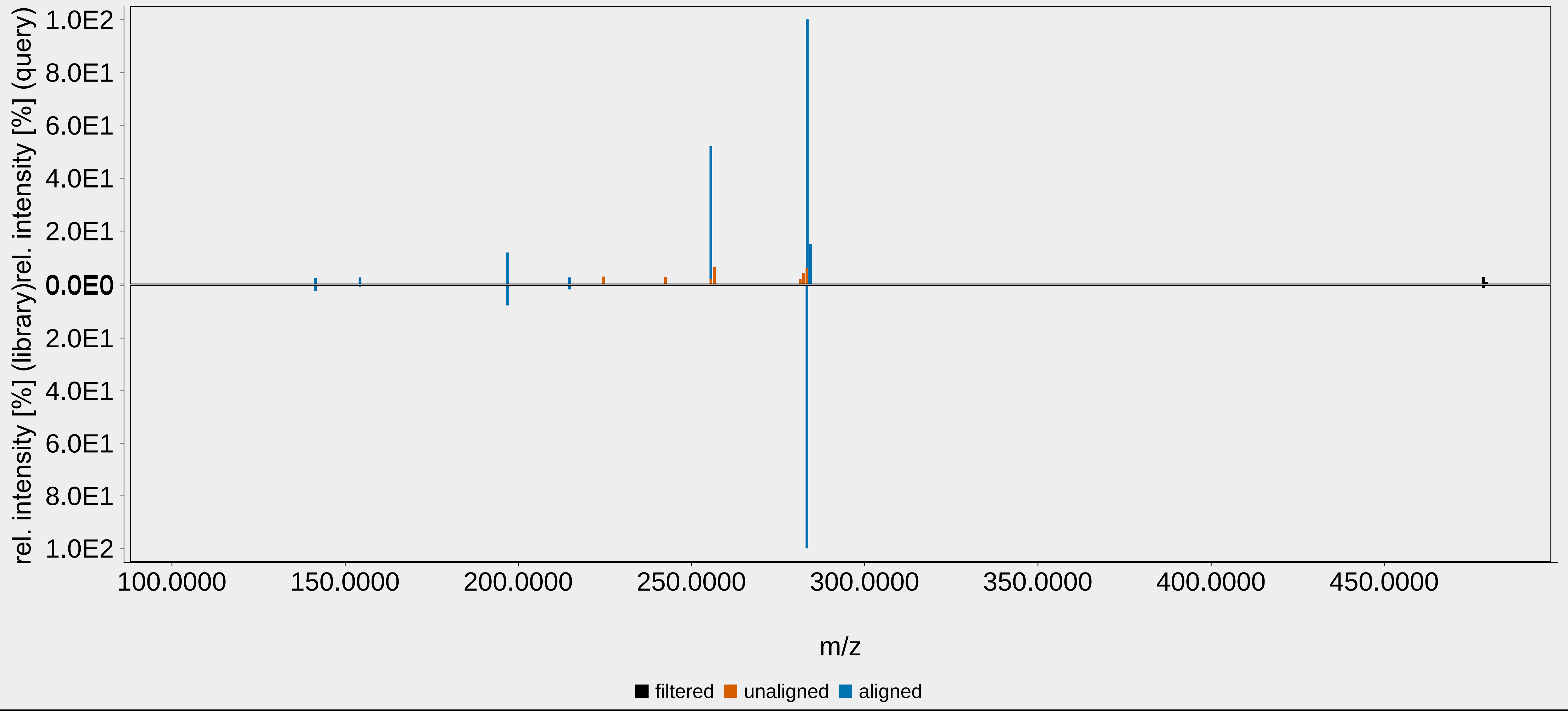

Compound information

Name: PE(18:0/0:0); [M-H]- C23H47N1O7P1

Exact mass: 0.0

Ion type: M-H

Precursor m/z: 480.309

Charge: 1

Polarity: Negative

Instrument information

Instrument type: HCD; Lumos

Ion source: LC-ESI

Ms level: 2

Acquisition: Commercial

Database links

Other information

Principal investigator: Thomas Metz

Data collector: Thomas Metz

Entry ID: CCMSLIB00010059722

FAD - 30.0 eV

0.807

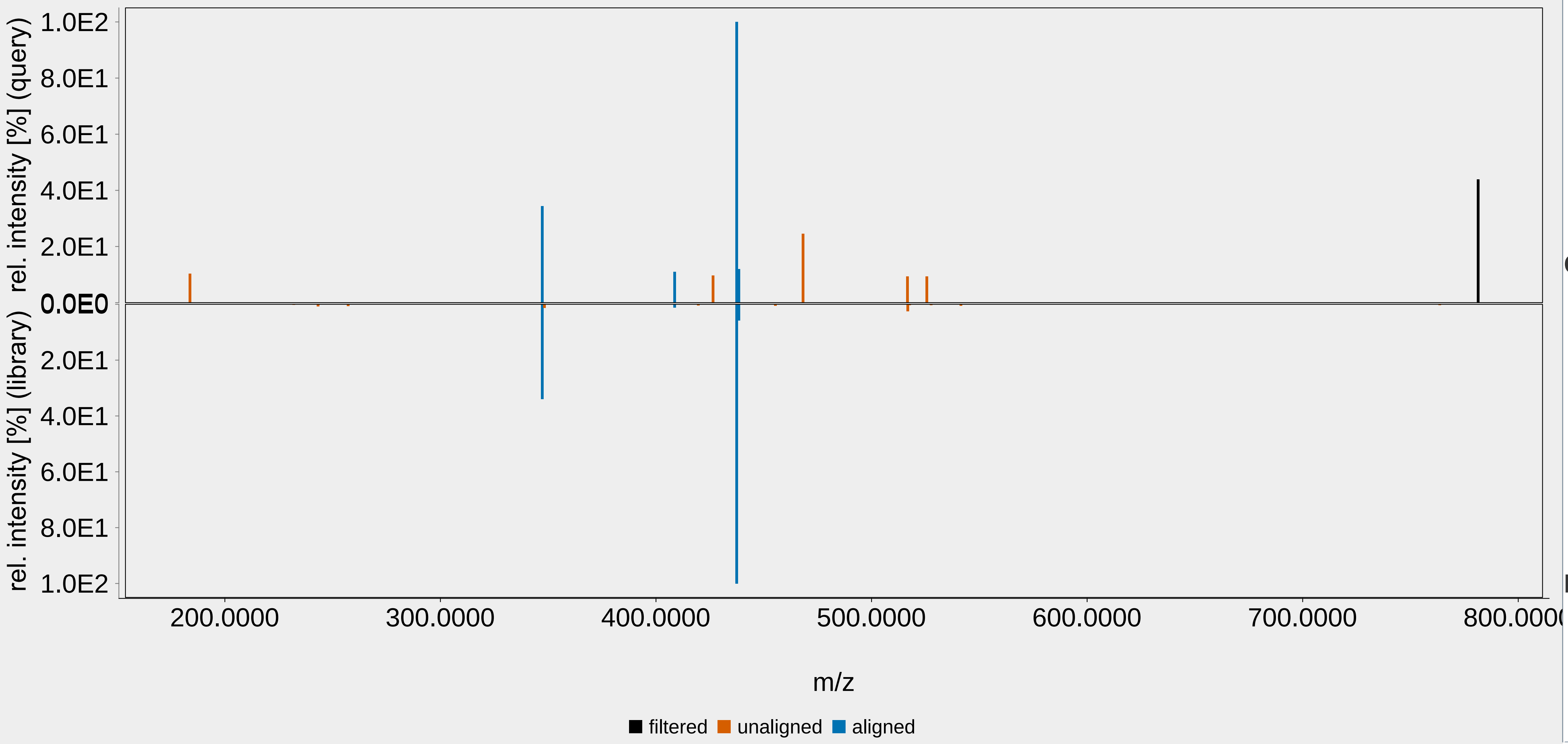

Compound information

Name: FAD - 30.0 eV

Exact mass: 0.0

Ion type: M-H

Precursor m/z: 784.15

Charge: 1

Polarity: Negative

SMILES:

Instrument information

Instrument type: Orbitrap

Ion source: ESI

Ms level: 2

Acquisition: Isolated

Database links

CAS:

Other information

Principal investigator: PI

Data collector: Data Collector

Entry ID: CCMSLIB00006113691

FAD - 30.0 eV

0.822

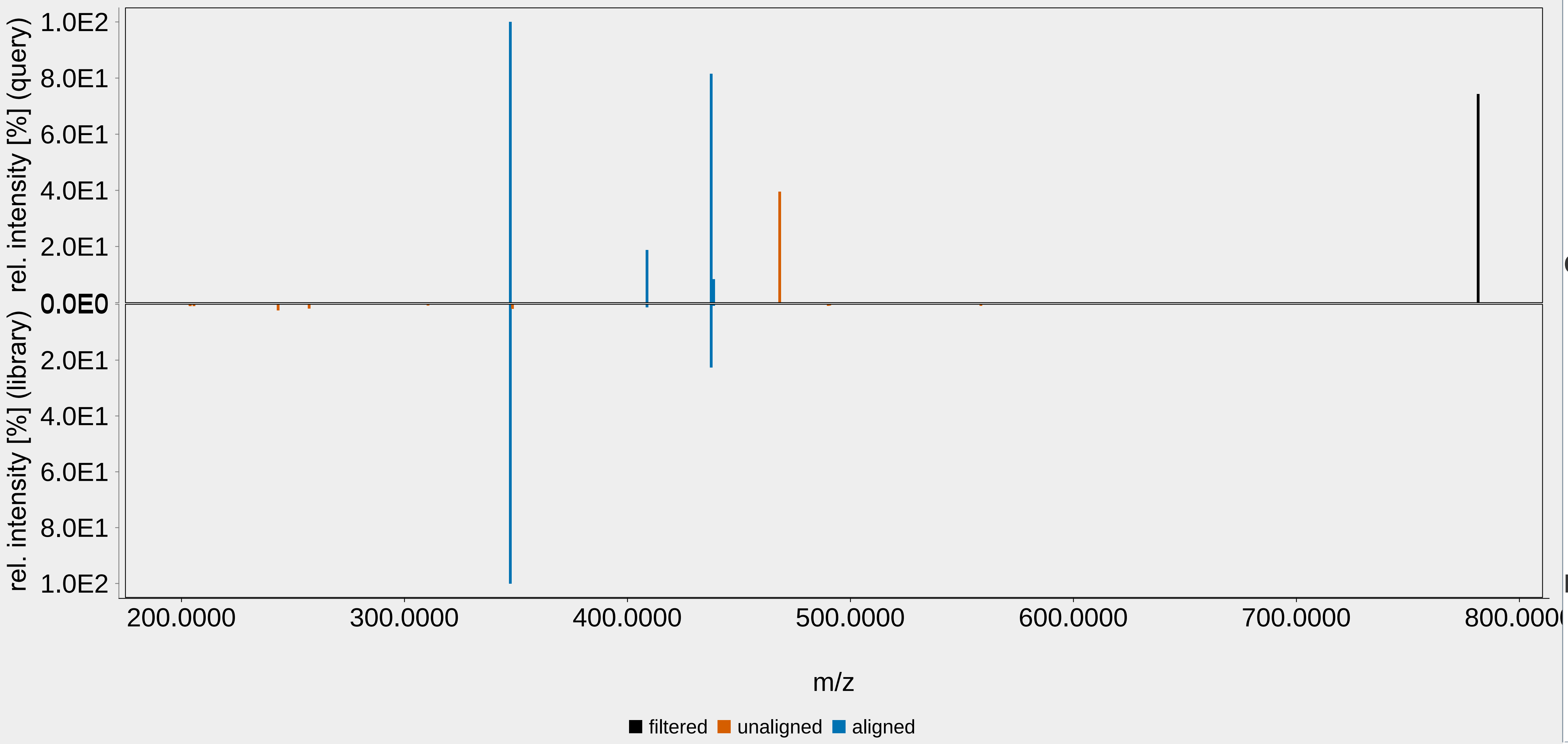

Compound information

Name: FAD - 30.0 eV

Exact mass: 0.0

Ion type: M-H

Precursor m/z: 784.149

Charge: 1

Polarity: Negative

SMILES:

Instrument information

Instrument type: Orbitrap

Ion source: ESI

Ms level: 2

Acquisition: Isolated

Database links

CAS:

Other information

Principal investigator: PI

Data collector: Data Collector

Entry ID: CCMSLIB00006113726

PA 18:1_24:1

0.817

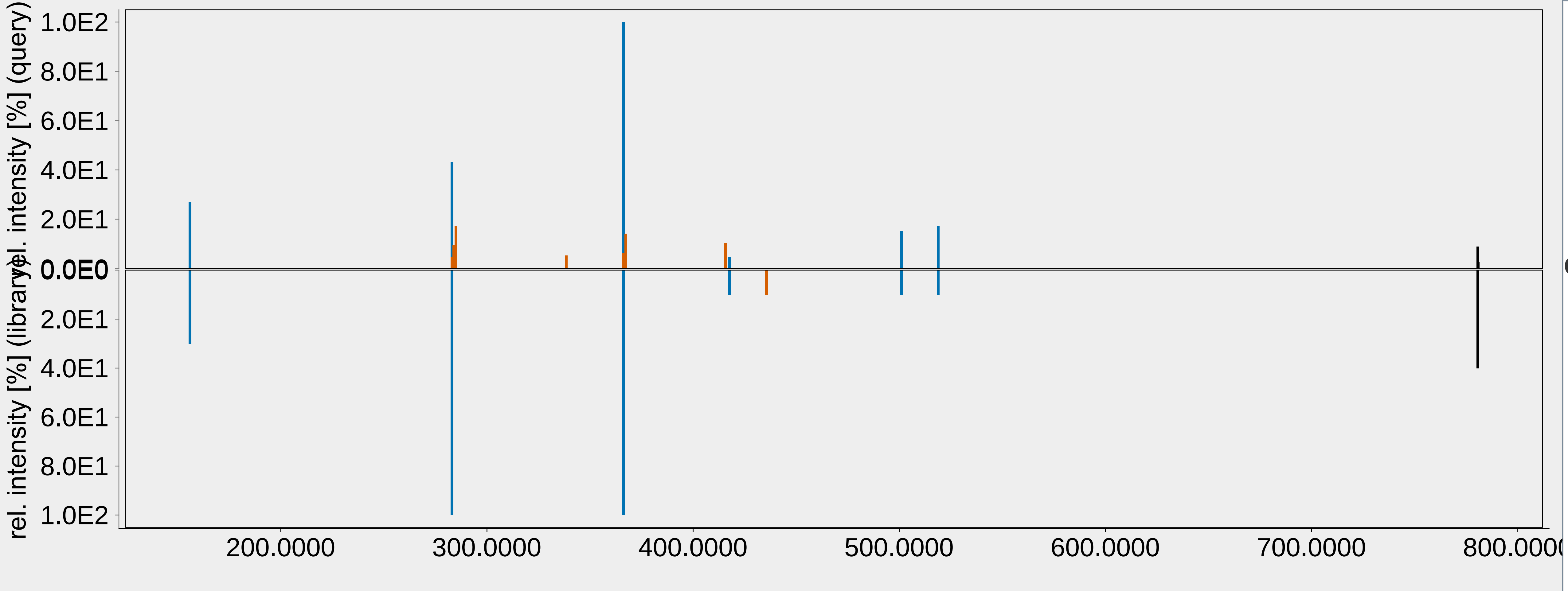

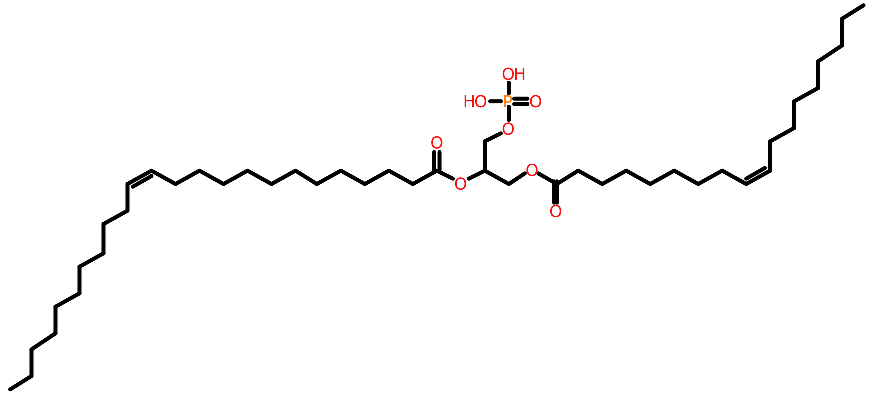

Compound information

Name: PA 18:1_24:1

Formula: C45H85O8P

Ion type: [M-H]-

Precursor m/z: 783.59093

Ccs: 279.35

InChI key:

SMILES:

Num peaks: 8

Instrument information

Database links

Other information

PE(22:6/0:0); [M-H]- C27H43N1O7P1 0.920

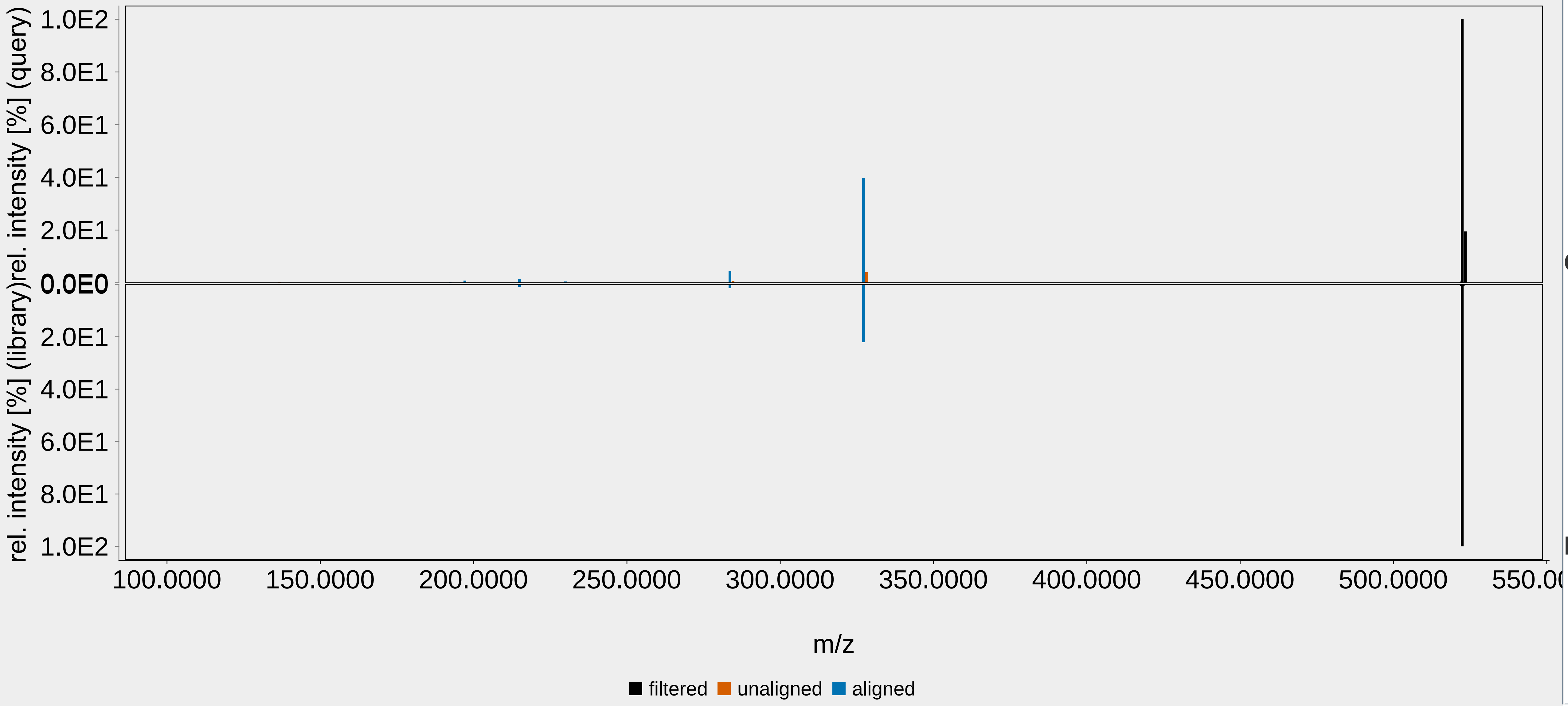

Compound information

Name: PE(22:6/0:0); [M-H]- C27H43N1O7P1

Exact mass: 0.0

Ion type: M-H

Precursor m/z: 524.278

Charge: 1

Polarity: Negative

Instrument information

Instrument type: HCD; Velos

Ion source: LC-ESI

Ms level: 2

Acquisition: Commercial

Database links

Other information

Principal investigator: Thomas Metz

Data collector: Thomas Metz

Entry ID: CCMSLIB00010073025

PE(22:6/0:0); [M-H]- C27H43N1O7P1 0.932

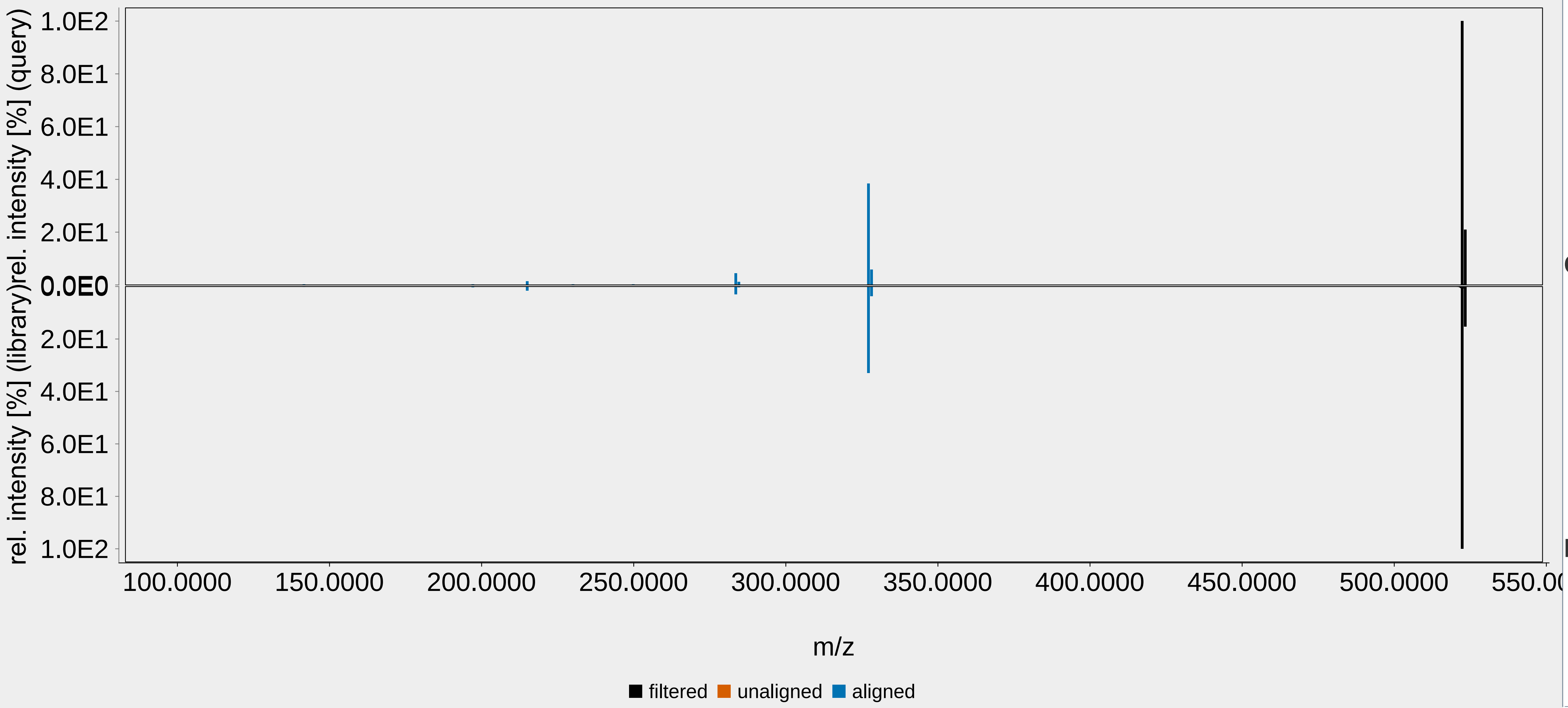

Compound information

Name: PE(22:6/0:0); [M-H]- C27H43N1O7P1

Exact mass: 0.0

Ion type: M-H

Precursor m/z: 524.278

Charge: 1

Polarity: Negative

Instrument information

Instrument type: HCD; Velos

Ion source: LC-ESI

Ms level: 2

Acquisition: Commercial

Database links

Other information

Principal investigator: Thomas Metz

Data collector: Thomas Metz

Entry ID: CCMSLIB00010086705

PE(17:0/22:6); [M-H]- C44H75N1O8P1 **0.861**

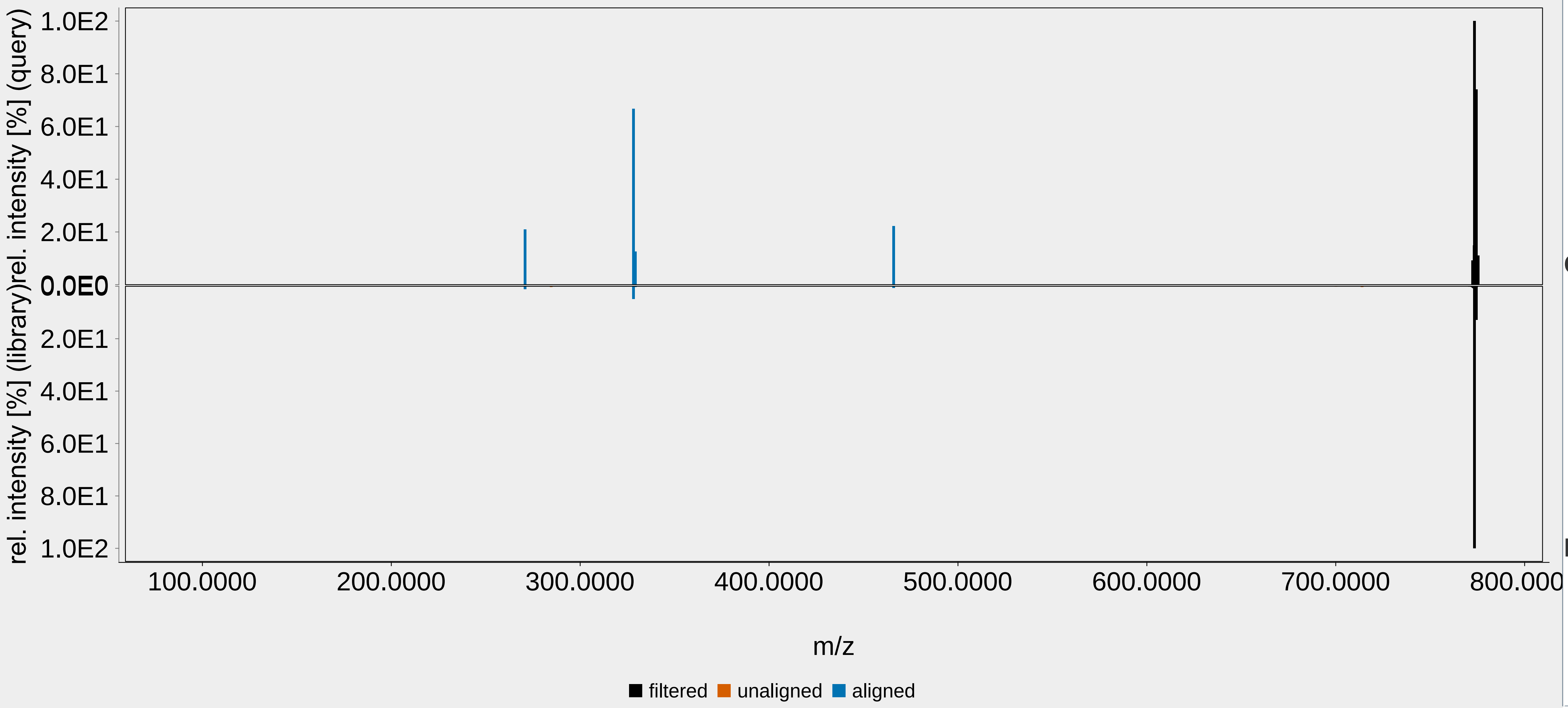

Compound information

Name: PE(17:0/22:6); [M-H]- C44H75N1O8P1

Exact mass: 0.0

Ion type: M-H

Precursor m/z: 776.523

Charge: 1

Polarity: Negative

Instrument information

Instrument type: HCD; Velos

Ion source: LC-ESI

Ms level: 2

Acquisition: Commercial

Database links

Other information

Principal investigator: Thomas Metz

Data collector: Thomas Metz

Entry ID: CCMSLIB00010086568

PE(17:0/22:6); [M-H]- C44H75N1O8P1 **0.890**

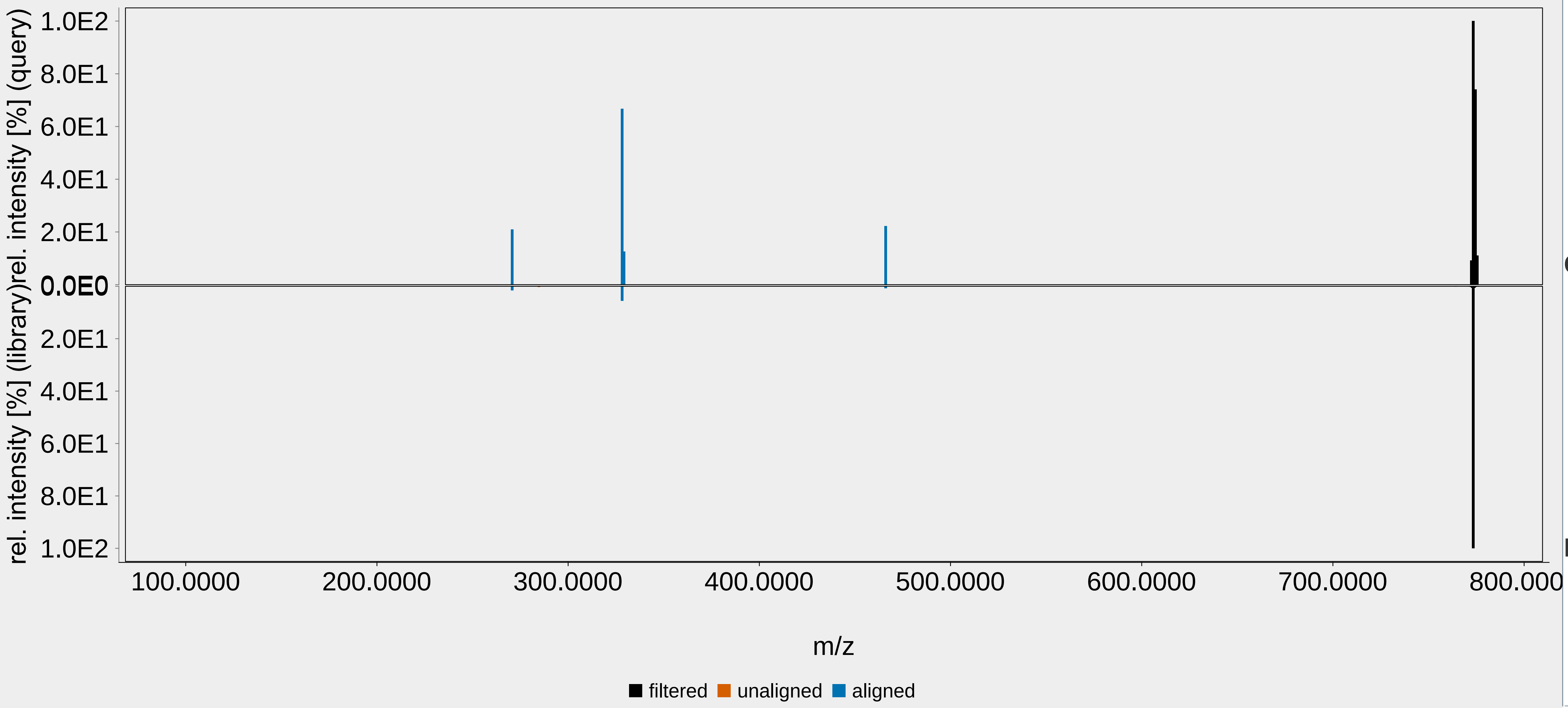

Compound information

Name: PE(17:0/22:6); [M-H]- C44H75N1O8P1

Exact mass: 0.0

Ion type: M-H

Precursor m/z: 776.523

Charge: 1

Polarity: Negative

Instrument information

Instrument type: HCD; Velos

Ion source: LC-ESI

Ms level: 2

Acquisition: Commercial

Database links

Other information

Principal investigator: Thomas Metz

Data collector: Thomas Metz

Entry ID: CCMSLIB00010087500

Spectral Match to 1-Hexadecanoyl-sn-glycero-3-phospho-(1'-myo-inositol) from NIST14

0.892

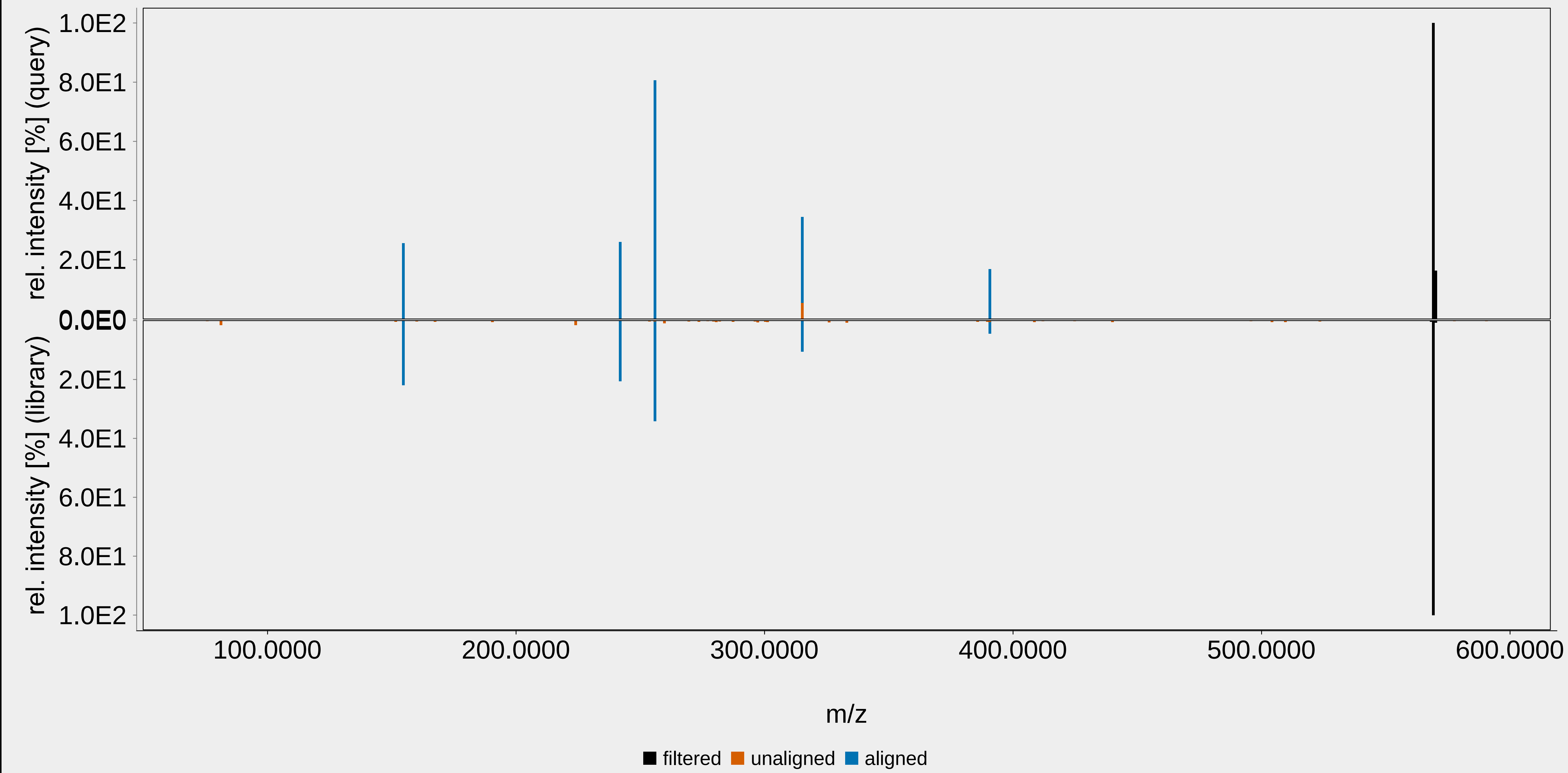

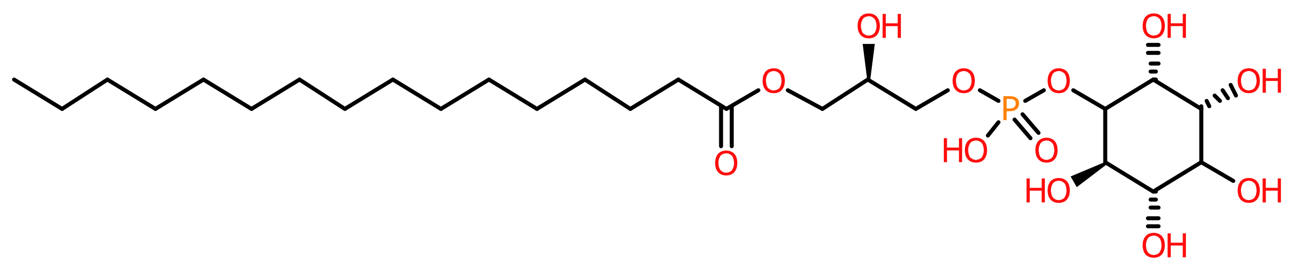

Compound information

Name: Spectral Match to

Exact mass: 572.296

Ion type: M-H

Precursor m/z: 571.294

Charge: 1

Polarity: Negative

InChI:

SMILES:

Instrument information

Instrument type: Ion Trap

Ion source: ESI

Ms level: 2

Acquisition: Isolated

Database links

CAS: 106182201

Other information

Principal investigator: Data from Pieter

Data collector: Data deposited by lfnothias

Entry ID: CCMSLIB00003136330

LPI 16:0 **0.973**

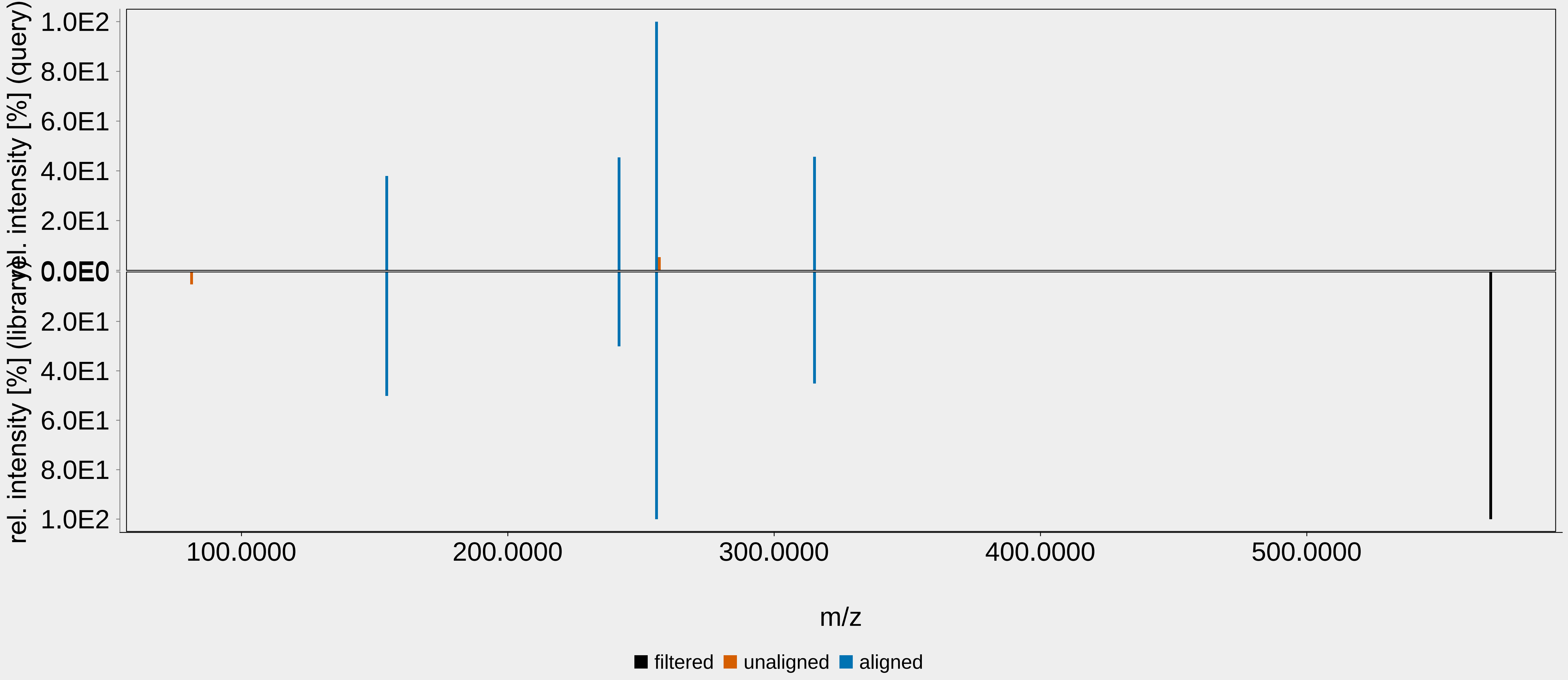

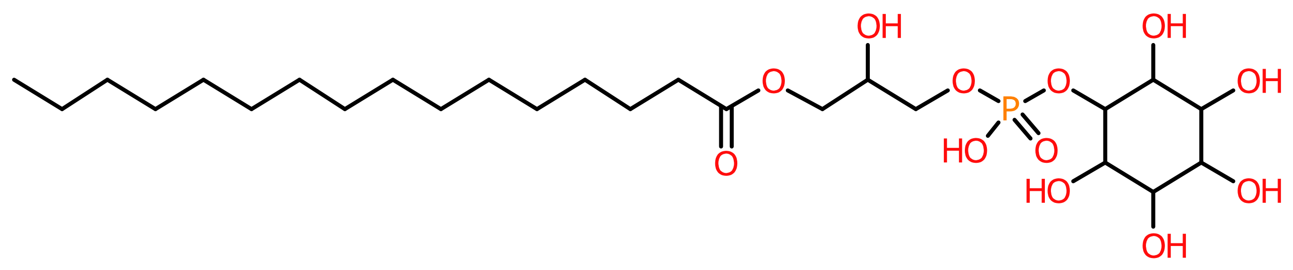

Compound information

Name: LPI 16:0

Formula: C25H49O12P

Ion type: [M-H]-

Precursor m/z: 571.28889

Ccs: 225.12

InChI key:

SMILES:

Num peaks: 6

Instrument information

Database links

Other information

Fosfructose - 30.0 eV

0.722

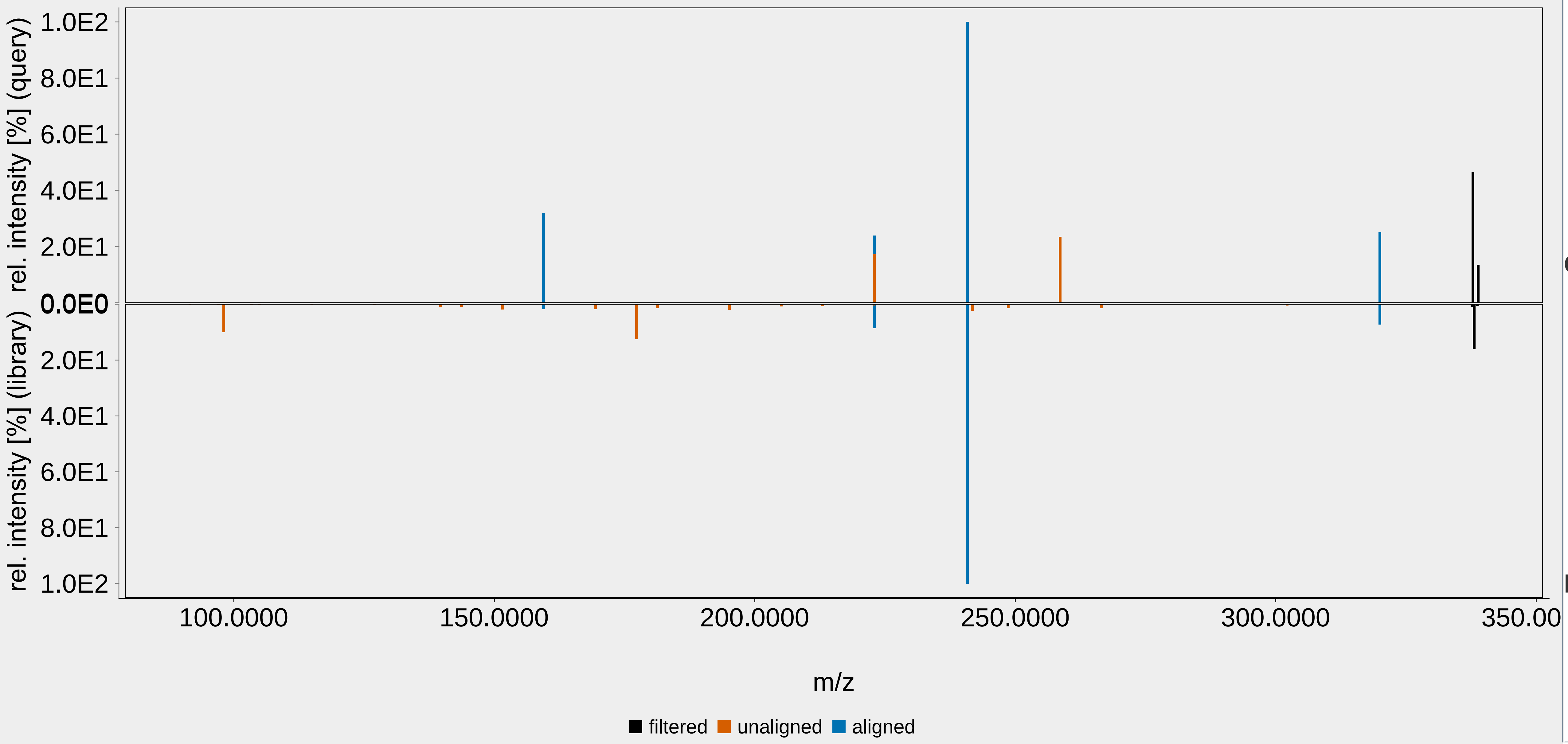

Compound information

Name: Fosfructose - 30.0 eV

Exact mass: 0.0

Ion type: M-H

Precursor m/z: 338.989

Charge: 1

Polarity: Negative

SMILES:

Instrument information

Instrument type: Orbitrap

Ion source: ESI

Ms level: 2

Acquisition: Isolated

Database links

CAS:

Other information

Principal investigator: PI

Data collector: Data Collector

Entry ID: CCMSLIB00006121372

Fosfructose - 30.0 eV

0.720

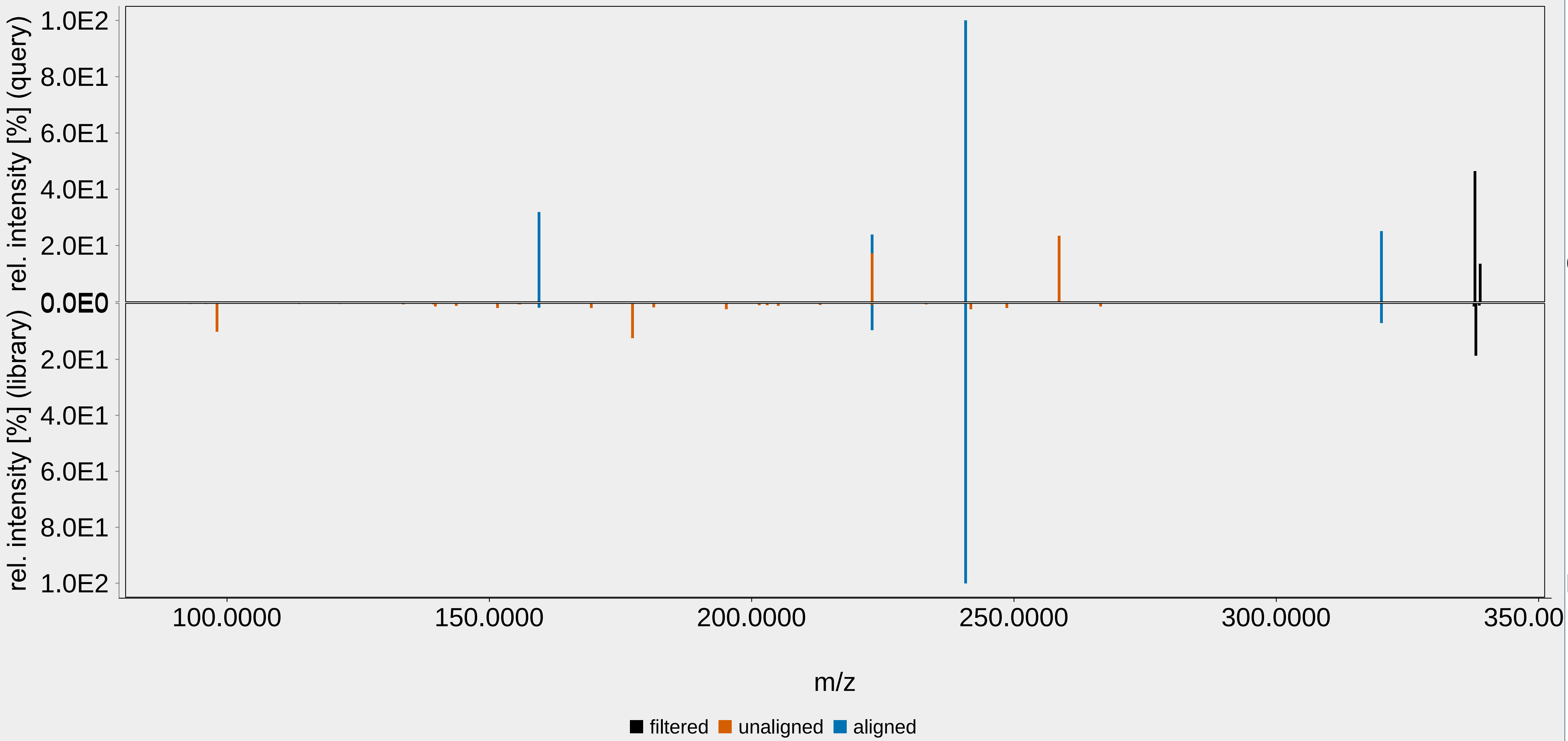

Compound information

Name: Fosfructose - 30.0 eV

Exact mass: 0.0

Ion type: M-H

Precursor m/z: 338.989

Charge: 1

Polarity: Negative

SMILES:

Instrument information

Instrument type: Orbitrap

Ion source: ESI

Ms level: 2

Acquisition: Isolated

Database links

CAS:

Other information

Principal investigator: PI

Data collector: Data Collector

Entry ID: CCMSLIB00006121374

PA 18:1_20:4 **0.891**

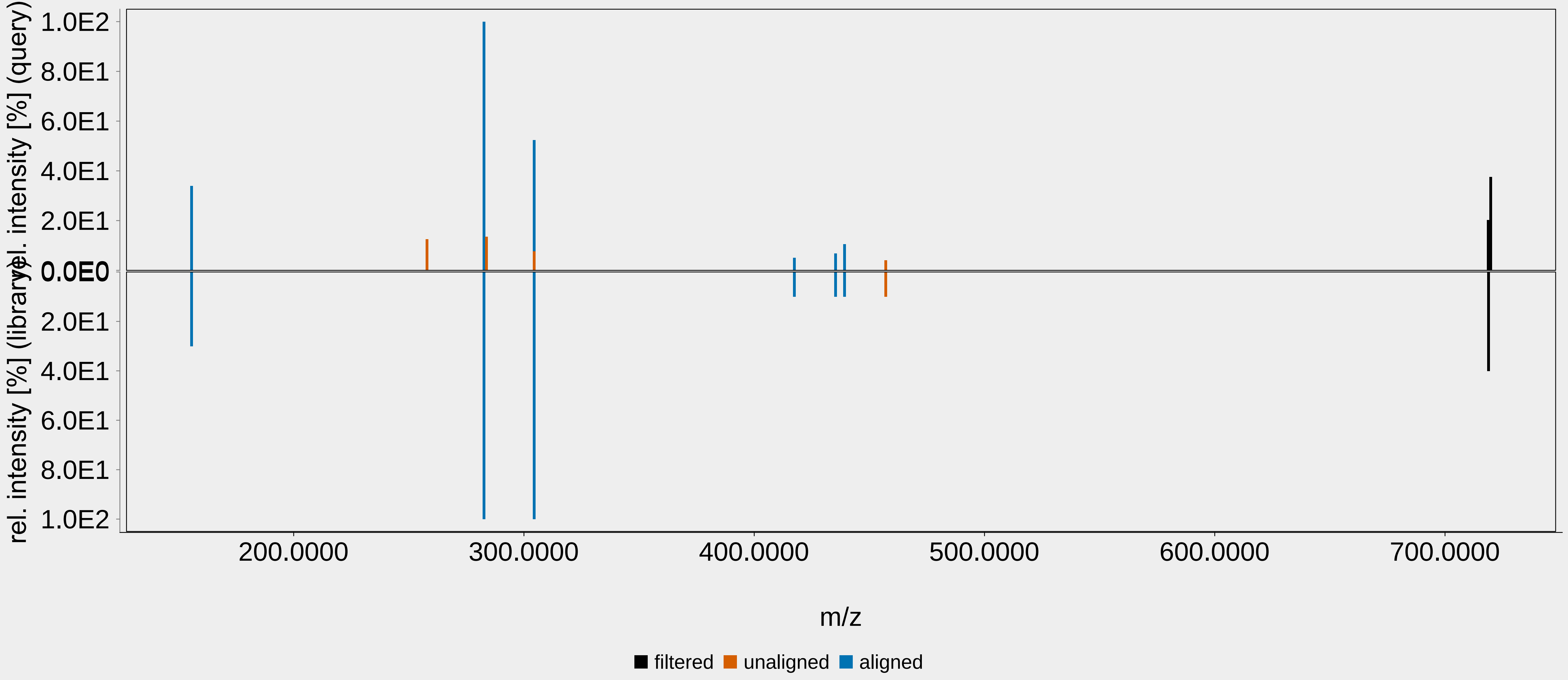

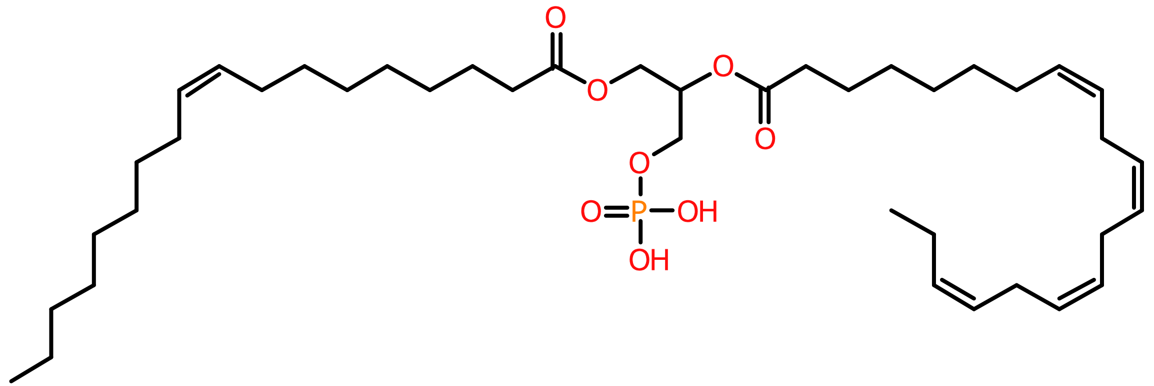

Compound information

Name: PA 18:1_20:4

Formula: C41H71O8P

Ion type: [M-H]-

Precursor m/z: 721.48138

Ccs: 261.27

InChI key:

SMILES:

Num peaks: 8

Instrument information

Database links

Other information

PE(20:1/22:6); [M-H]- C47H79N1O8P1 **0.780**

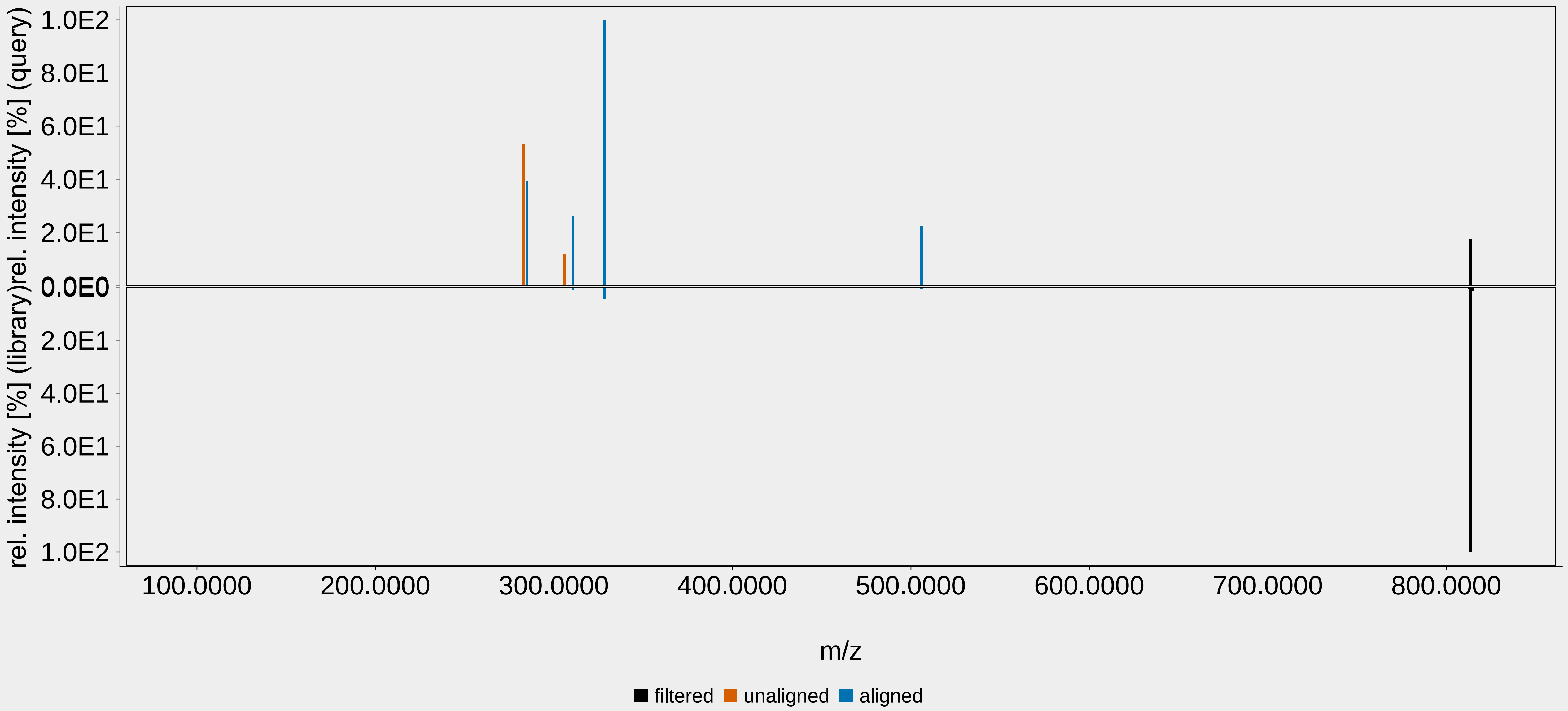

Compound information

Name: PE(20:1/22:6); [M-H]- C47H79N1O8P1

Exact mass: 0.0

Ion type: M-H

Precursor m/z: 816.554

Charge: 1

Polarity: Negative

Instrument information

Instrument type: HCD; Velos

Ion source: LC-ESI

Ms level: 2

Acquisition: Commercial

Database links

Other information

Principal investigator: Thomas Metz

Data collector: Thomas Metz

Entry ID: CCMSLIB00010073329

PS(18:1/18:1); [M-H]- C42H77N1O10P1

0.830

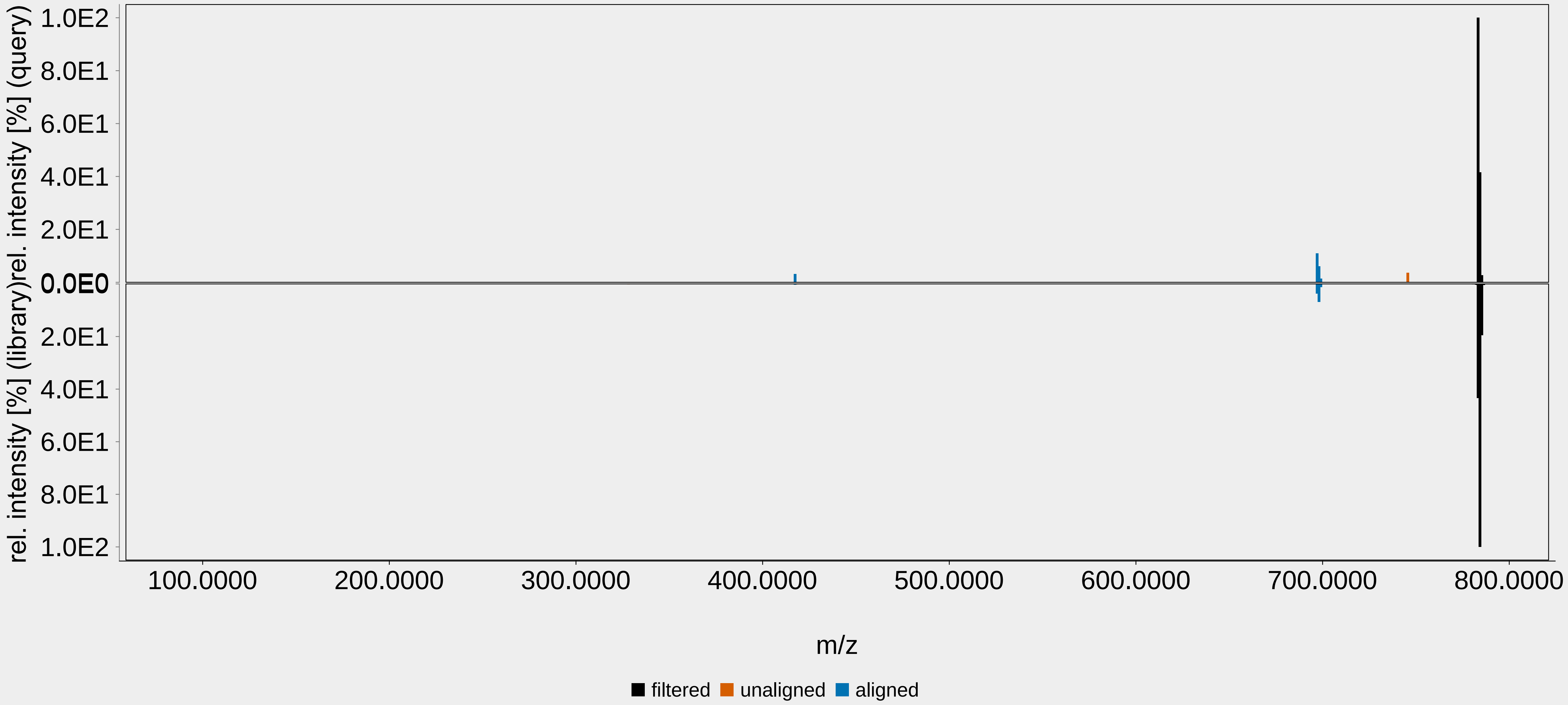

Compound information

Name: PS(18:1/18:1); [M-H]- C42H77N1O10P1

Exact mass: 0.0

Ion type: M-H

Precursor m/z: 786.529

Charge: 1

Polarity: Negative

Instrument information

Instrument type: HCD; Velos

Ion source: LC-ESI

Ms level: 2

Acquisition: Commercial

Database links

Other information

Principal investigator: Thomas Metz

Data collector: Thomas Metz

Entry ID: CCMSLIB00010084418

PS 18:1_18:1

0.869

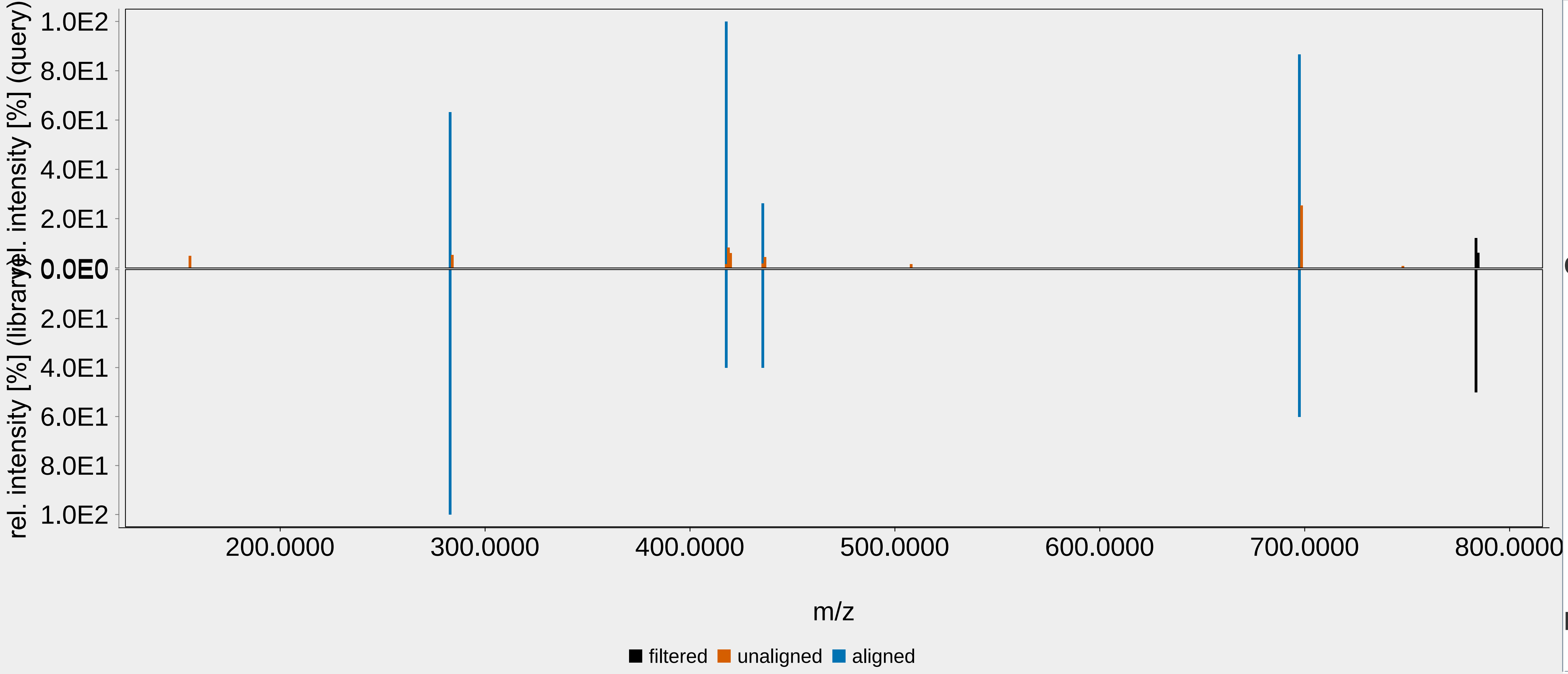

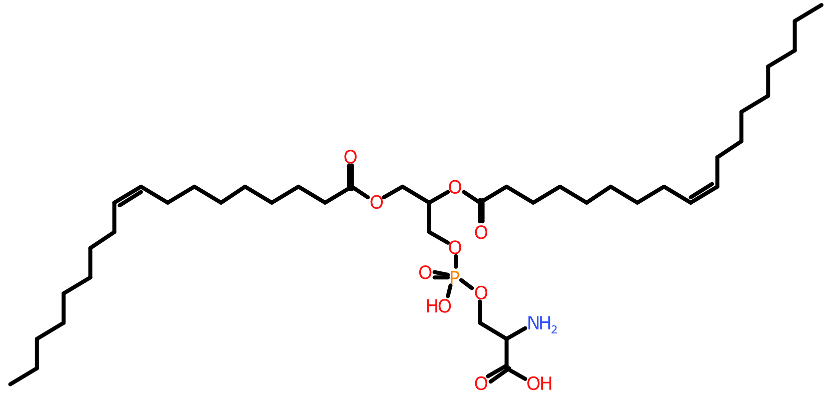

Compound information

Name: PS 18:1_18:1

Formula: C42H78NO10P

Ion type: [M-H]-

Precursor m/z: 786.52906

Ccs: 275.77

InChI key:

SMILES:

Num peaks: 5

Instrument information

Database links

Other information

UPD-alpha-D-Galactose - 30.0 eV

0.752

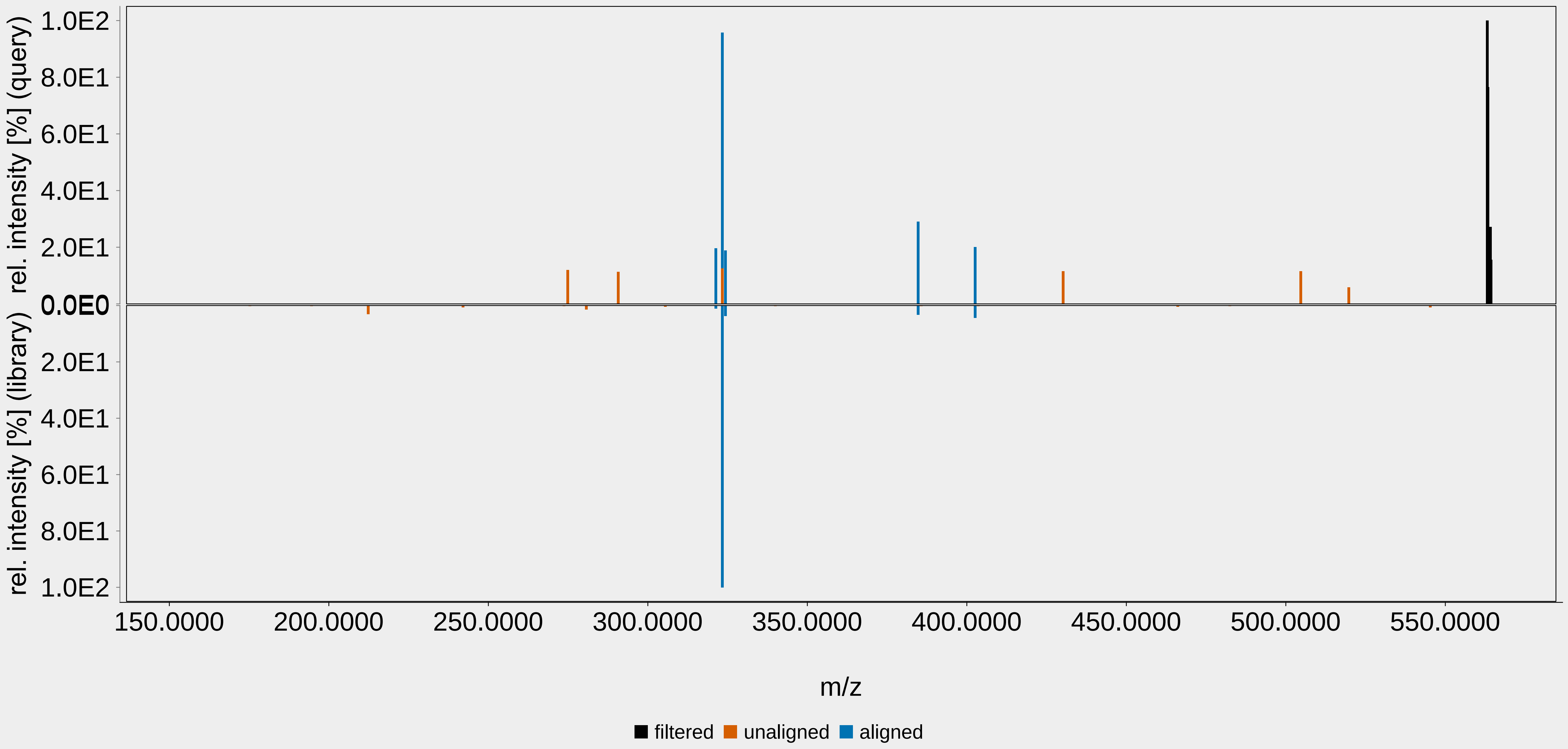

Compound information

Name: UPD-alpha-D-Galactose - 30.0 eV

Exact mass: 0.0

Ion type: Unknown

Precursor m/z: 565.048

Charge: 1

Polarity: Negative

SMILES:

Instrument information

Instrument type: Orbitrap

Ion source: ESI

Ms level: 2

Acquisition: Isolated

Database links

CAS:

Other information

Principal investigator: PI

Data collector: Data Collector

Entry ID: CCMSLIB00006113004

UPD-alpha-D-Galactose - 30.0 eV

0.757

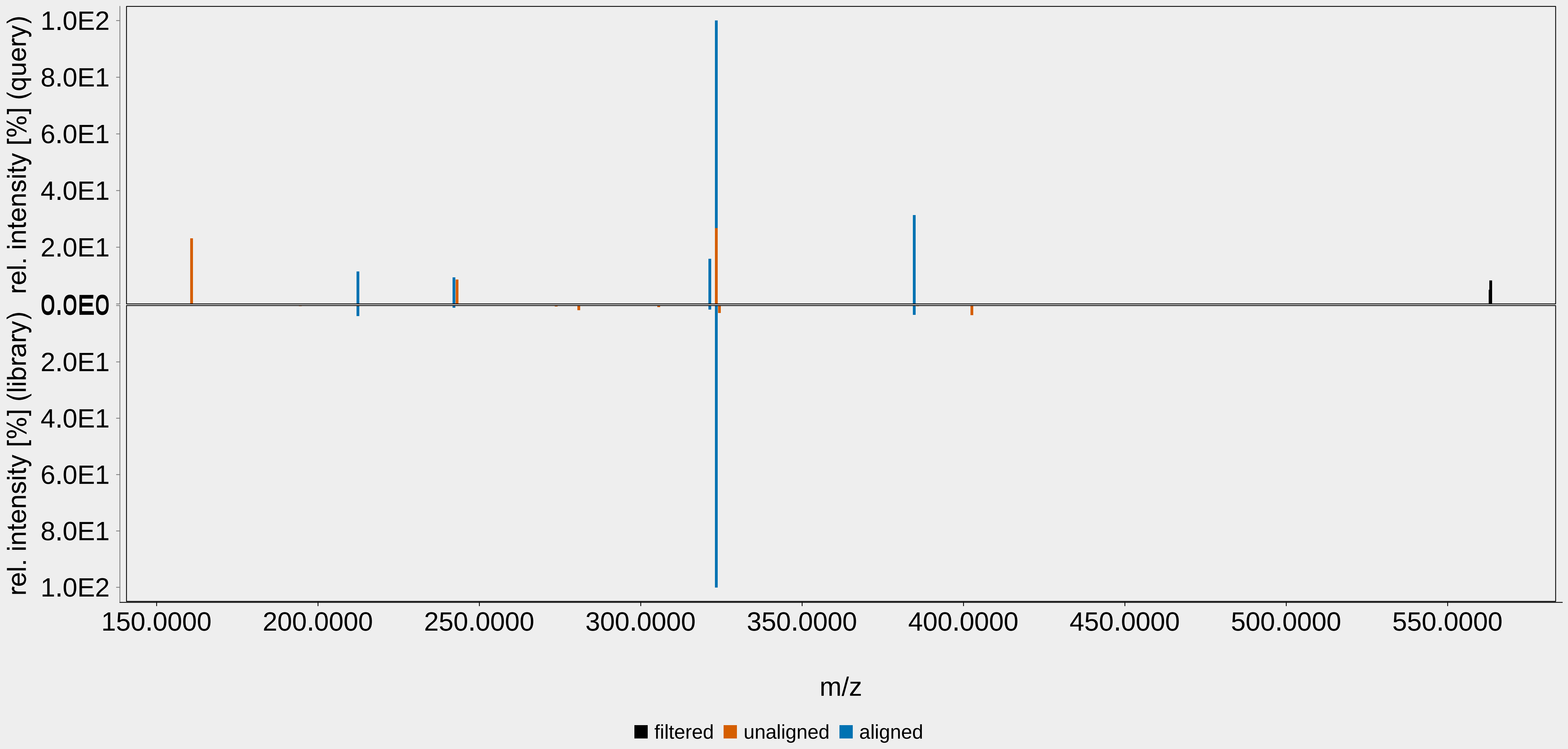

Compound information

Name: UPD-alpha-D-Galactose - 30.0 eV

Exact mass: 0.0

Ion type: Unknown

Precursor m/z: 565.048

Charge: 1

Polarity: Negative

SMILES:

Instrument information

Instrument type: Orbitrap

Ion source: ESI

Ms level: 2

Acquisition: Isolated

Database links

CAS:

Other information

Principal investigator: PI

Data collector: Data Collector

Entry ID: CCMSLIB00006113006

Spectral Match to 1,2-Di(4Z,7Z,10Z,13Z,16Z,19Z-docosahexaenoyl)-sn-glycero-3-phosphoethanolamine from NIST14

0.871

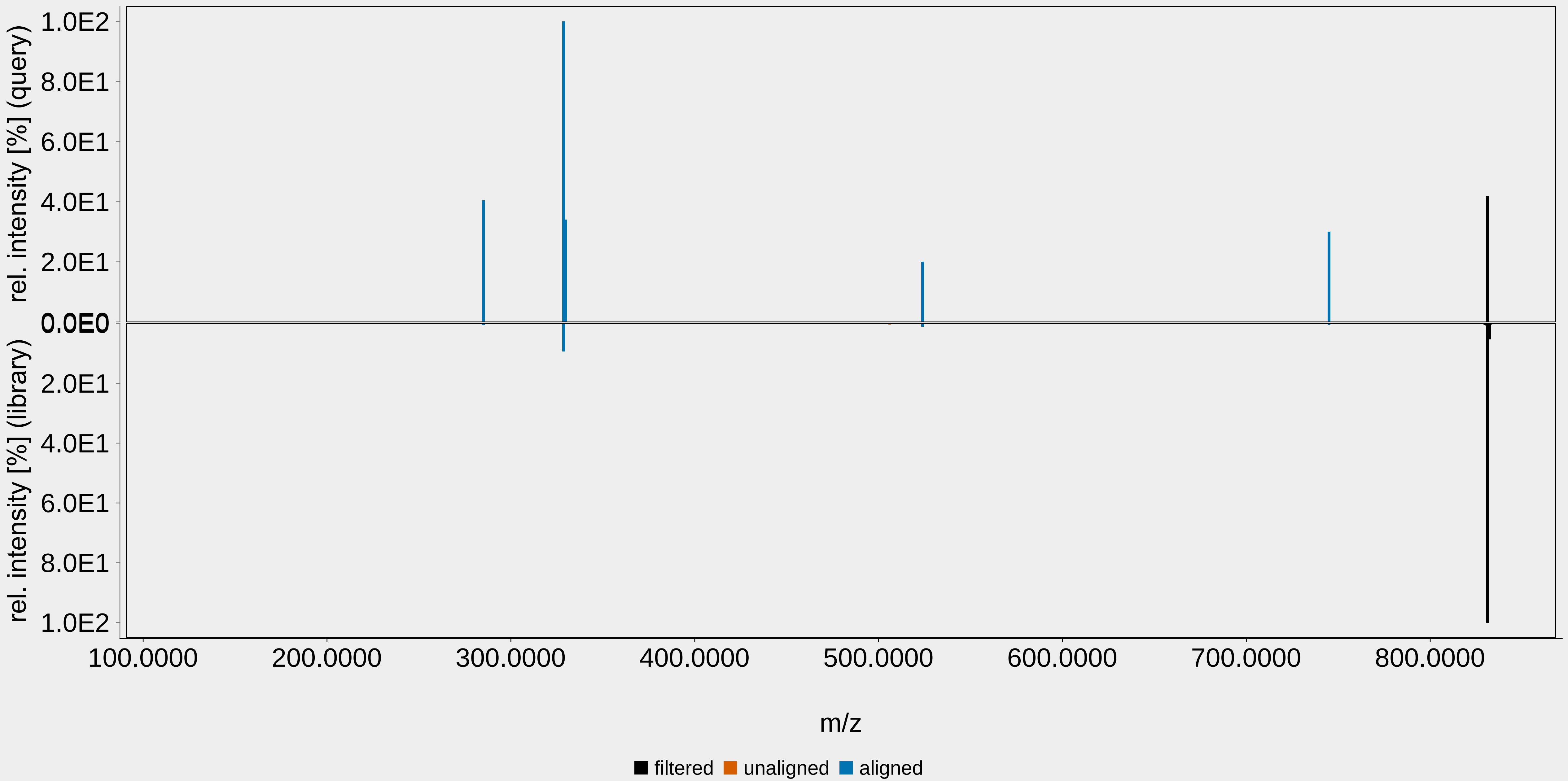

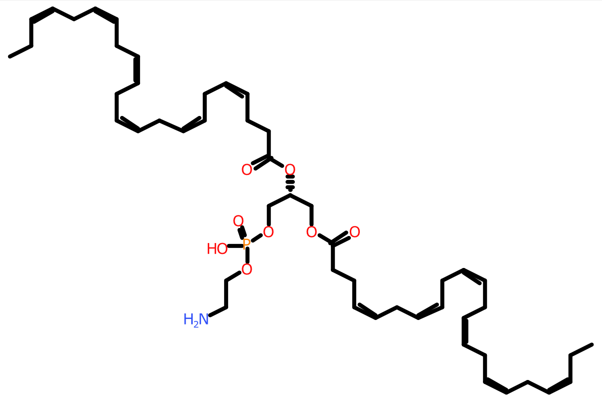

Compound information

Name: Spectral Match to

Exact mass: 835.515

Ion type: M-H

Precursor m/z: 834.512

Charge: 1

Polarity: Negative

InChI:

SMILES:

Instrument information

Instrument type: qTof

Ion source: ESI

Ms level: 2

Acquisition: Isolated

Database links

CAS: 123284811

Other information

Principal investigator: Data from Katrina

Data collector: Data deposited by alchemistmatt

Entry ID: CCMSLIB00003140104

PI(20:4/0:0); [M-H]- C29H48O12P1 **0.742**

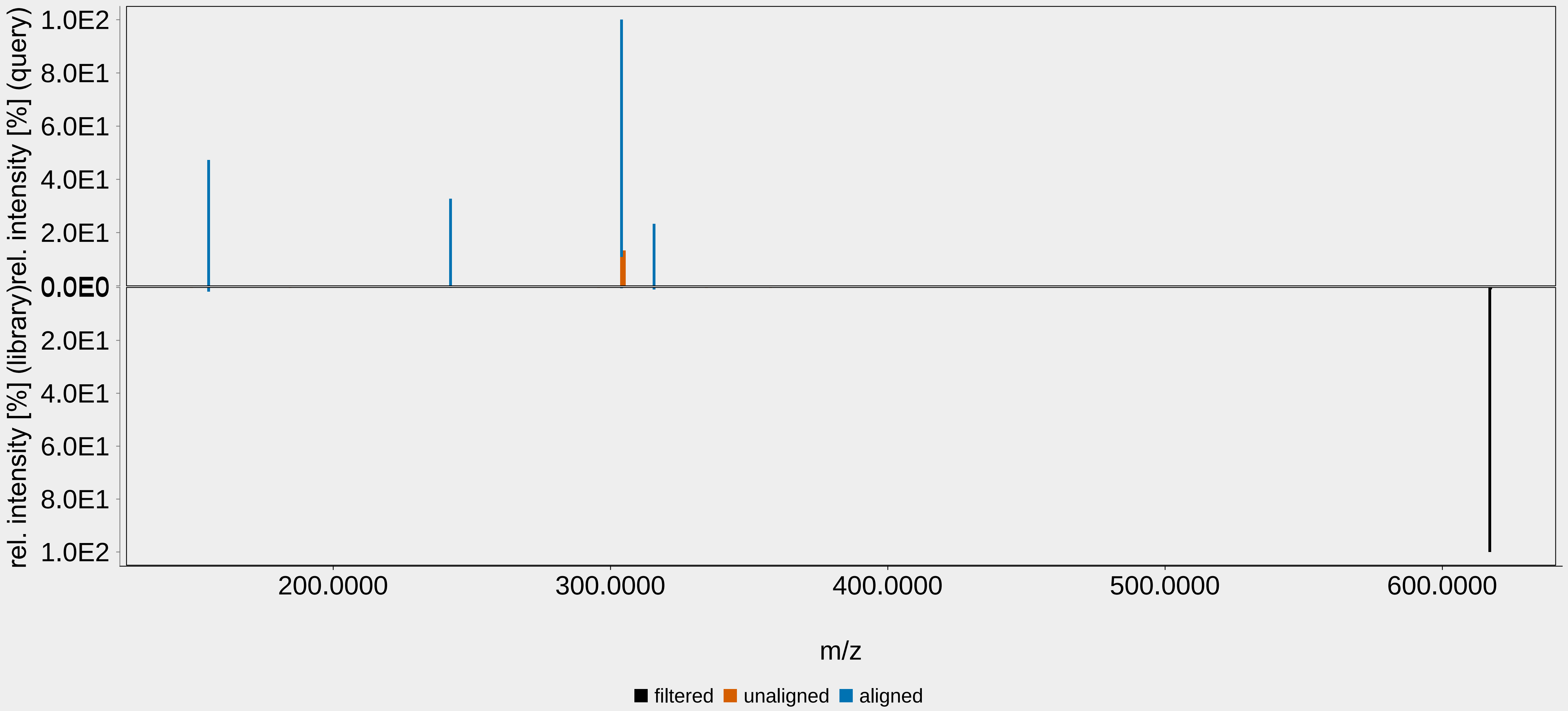

Compound information

Name: PI(20:4/0:0); [M-H]- C29H48O12P1

Exact mass: 0.0

Ion type: M-H

Precursor m/z: 619.288

Charge: 1

Polarity: Negative

Instrument information

Instrument type: HCD; Velos

Ion source: LC-ESI

Ms level: 2

Acquisition: Commercial

Database links

Other information

Principal investigator: Thomas Metz

Data collector: Thomas Metz

Entry ID: CCMSLIB00010084582

LPI 20:4

0.928

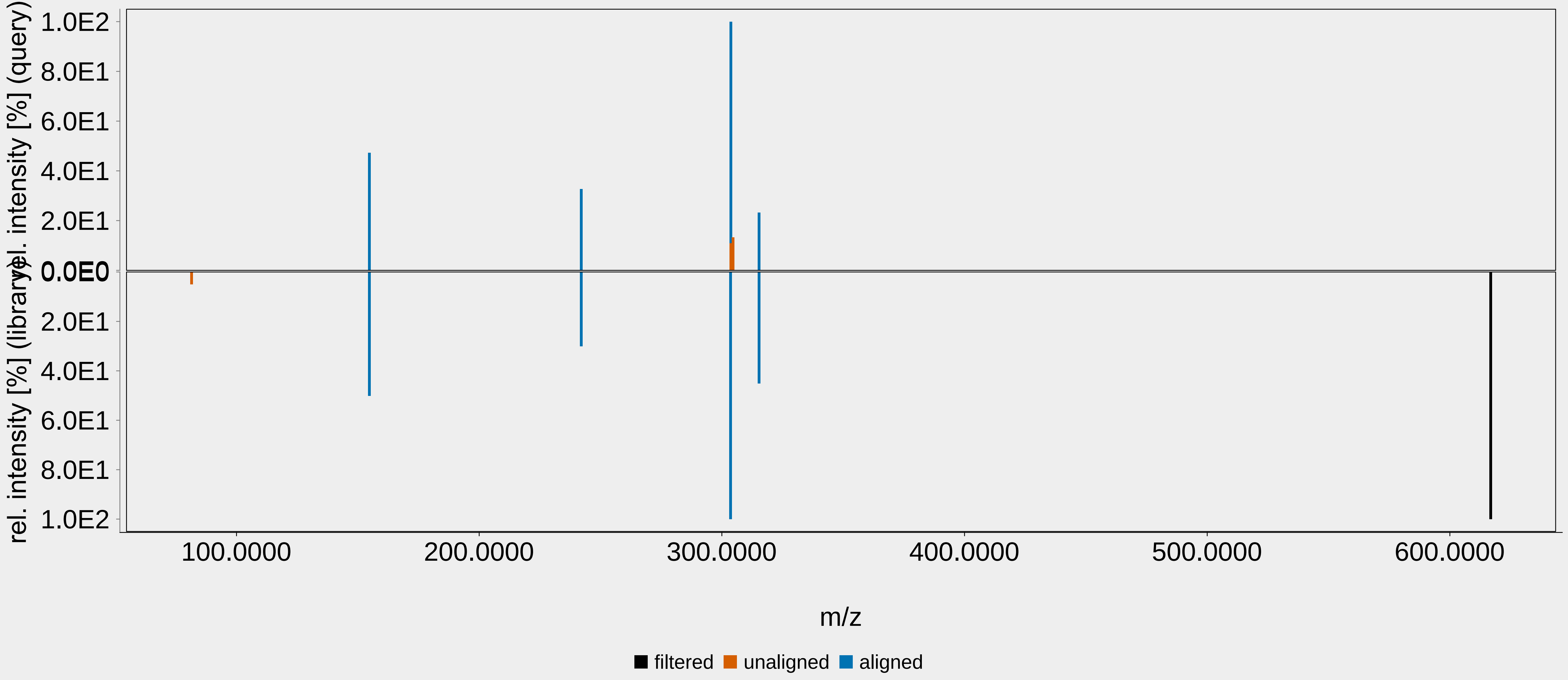

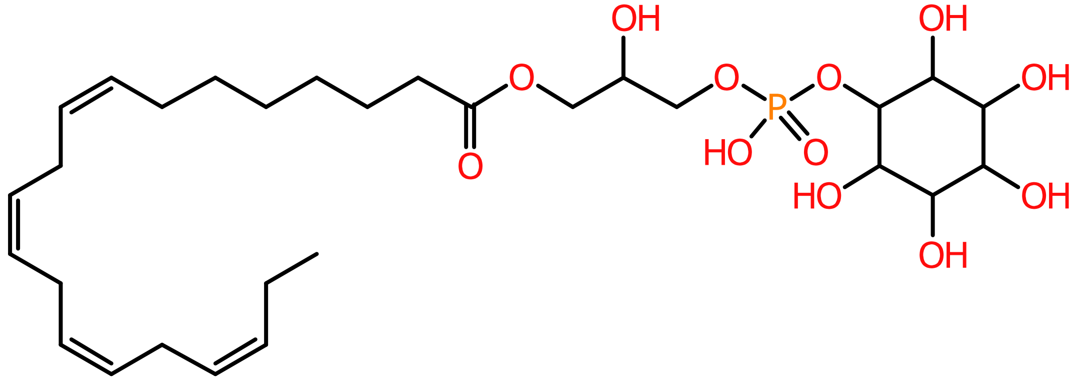

Compound information

Name: LPI 20:4

Formula: C29H49O12P

Ion type: [M-H]-

Precursor m/z: 619.28889

Ccs: 233.07

InChI key:

SMILES:

Num peaks: 6

Instrument information

Database links

Other information

PE(18:2/0:0); [M-H]- C23H43N1O7P1 **0.872**

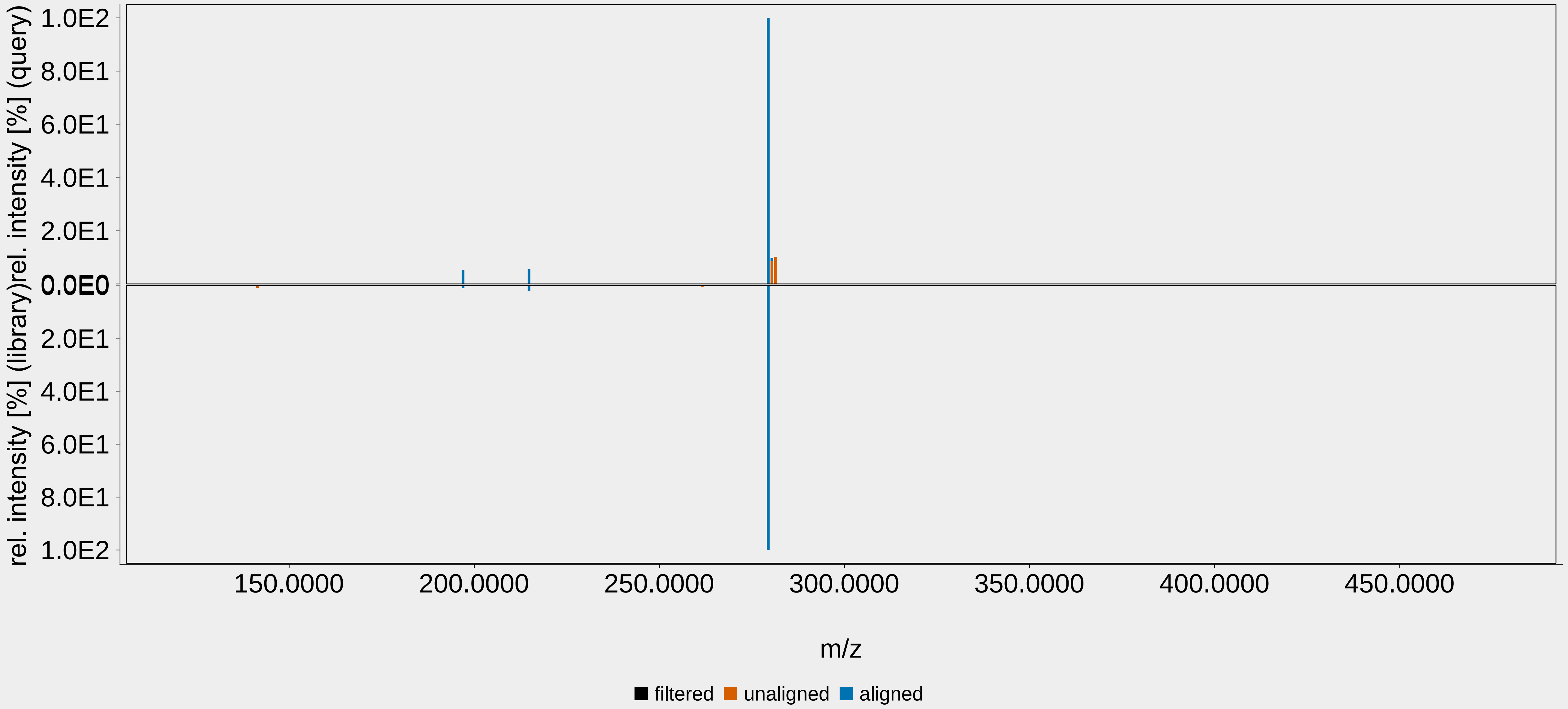

Compound information

Name: PE(18:2/0:0); [M-H]- C23H43N1O7P1

Exact mass: 0.0

Ion type: M-H

Precursor m/z: 476.278

Charge: 1

Polarity: Negative

Instrument information

Instrument type: HCD; Lumos

Ion source: LC-ESI

Ms level: 2

Acquisition: Commercial

Database links

Other information

Principal investigator: Thomas Metz

Data collector: Thomas Metz

Entry ID: CCMSLIB00010059116

PE(18:2/0:0); [M-H]- C23H43N1O7P1 **0.875**

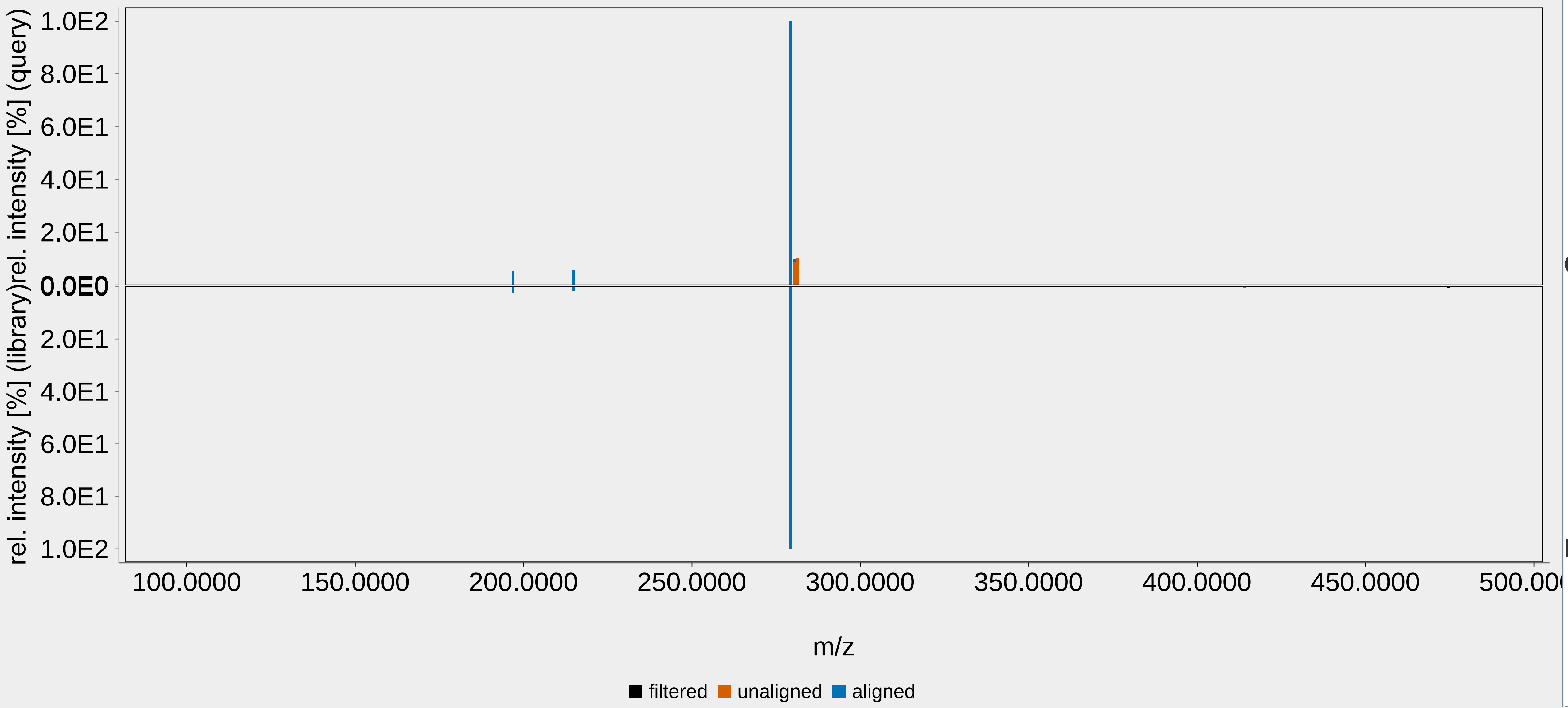

Compound information

Name: PE(18:2/0:0); [M-H]- C23H43N1O7P1

Exact mass: 0.0

Ion type: M-H

Precursor m/z: 476.278

Charge: 1

Polarity: Negative

Instrument information

Instrument type: CID; Lumos

Ion source: LC-ESI

Ms level: 2

Acquisition: Commercial

Database links

Other information

Principal investigator: Thomas Metz

Data collector: Thomas Metz

Entry ID: CCMSLIB00010059801

Guanosine triphosphate

0.779

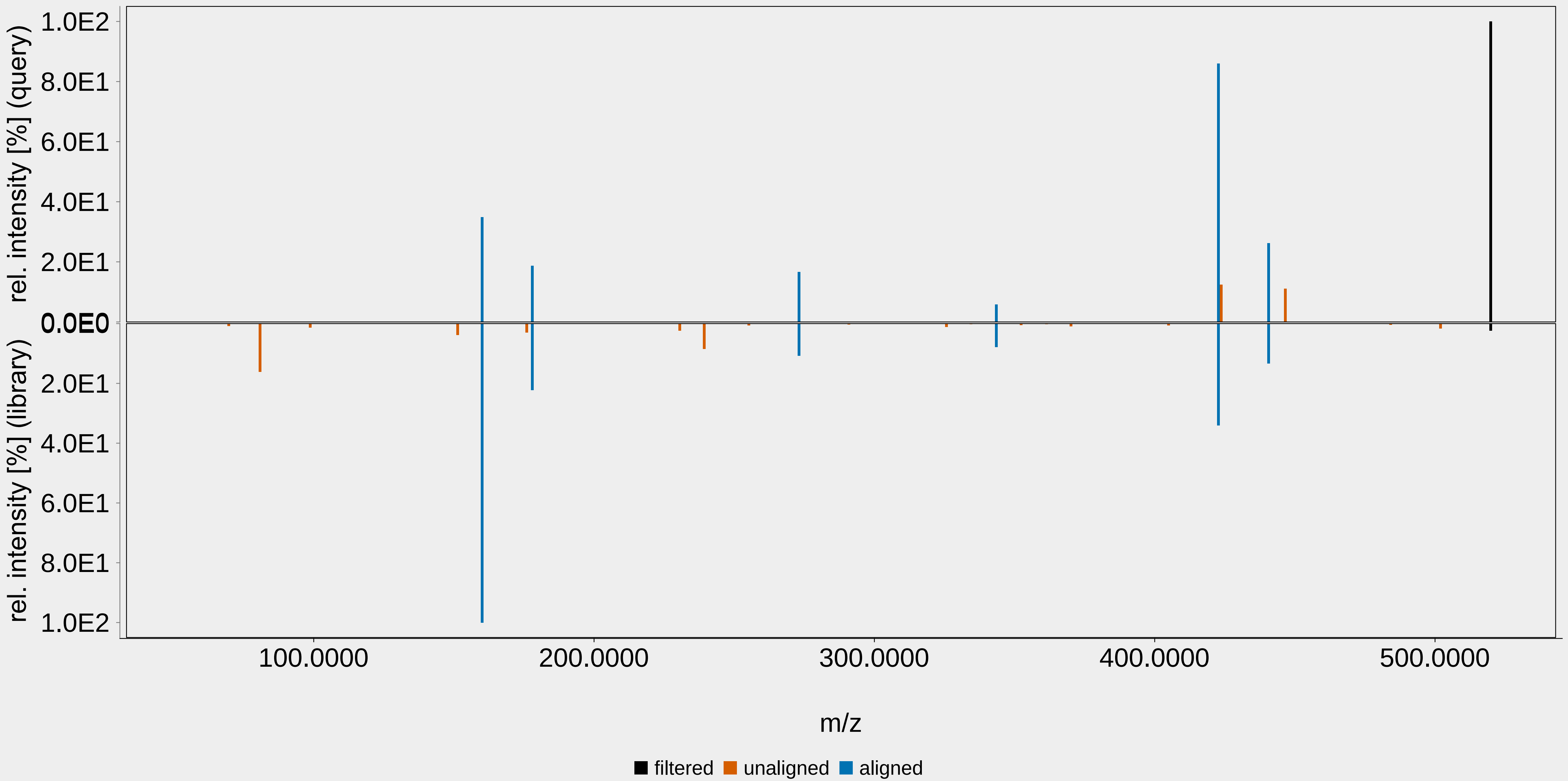

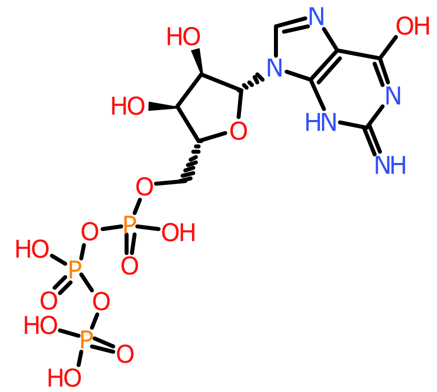

Compound information

Name: Guanosine triphosphate

Formula: C10H16N5O14P3

Ion type: [M-H]-

Precursor m/z: 521.981868542491

RT: 6.6404014

Polarity: negative

InChI:

InChI key:

SMILES:

Instrument information

Instrument type: LC-ESI-QFT

Instrument: Thermo Q-Exactive Plus

Ms level: MS2

Collision energy: 30.0 eV

Database links

Other information

Principal investigator: Prasad Phapale

Data collector: Prasad Phapale

Guanosine triphosphate

0.781

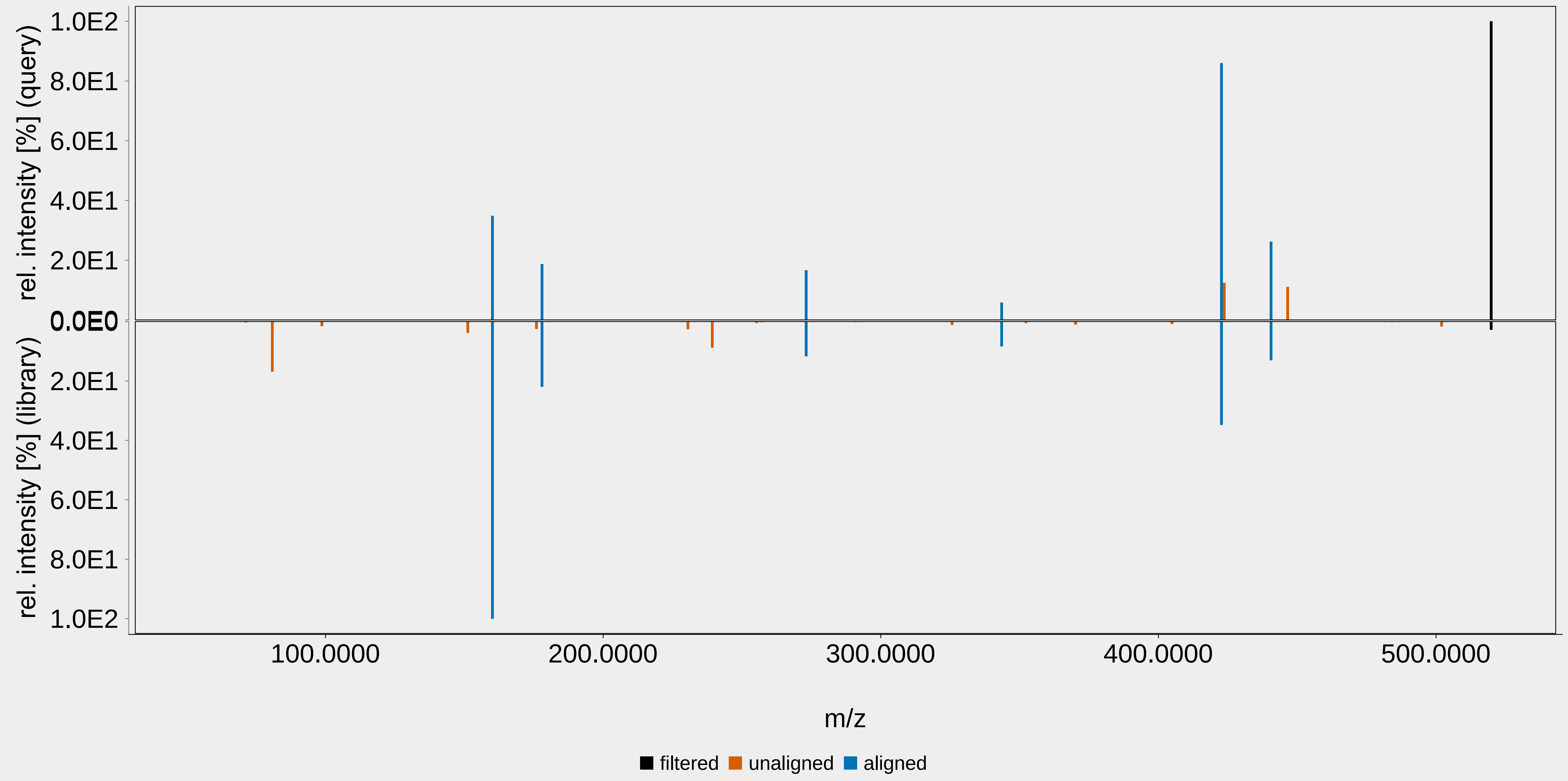

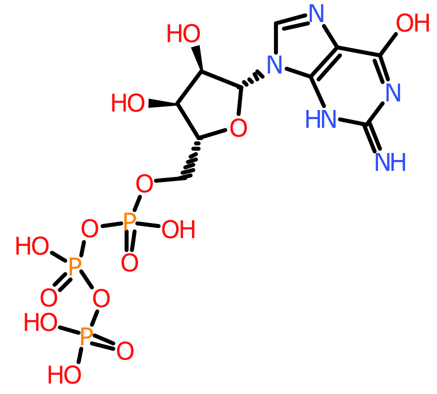

Compound information

Name: Guanosine triphosphate

Formula: C10H16N5O14P3

Ion type: [M-H]-

Precursor m/z: 521.981890480549

RT: 6.6717806

Polarity: negative

InChI:

InChI key:

SMILES:

Instrument information

Instrument type: LC-ESI-QFT

Instrument: Thermo Q-Exactive Plus

Ms level: MS2

Collision energy: 30.0 eV

Database links

Other information

Principal investigator: Prasad Phapale

Data collector: Prasad Phapale

PA 20:4_22:6 0.934

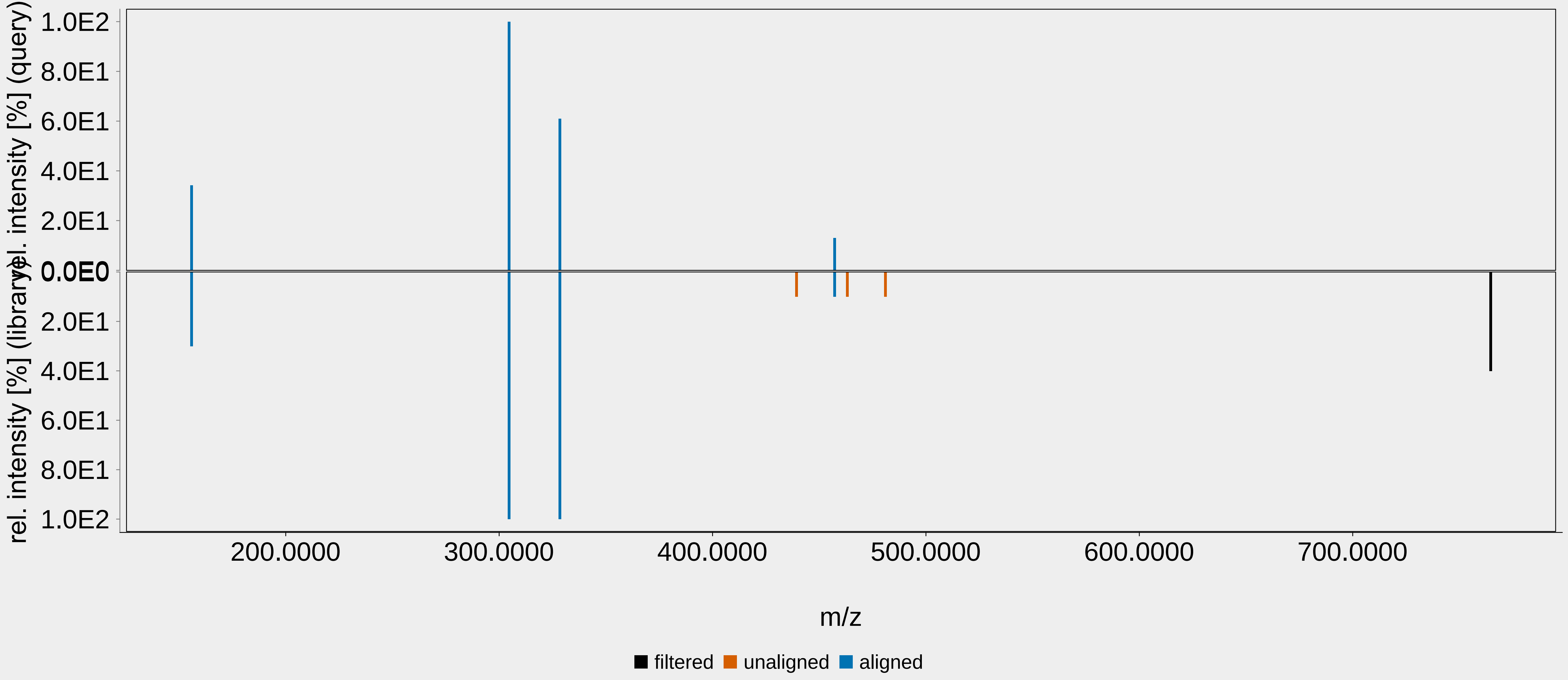

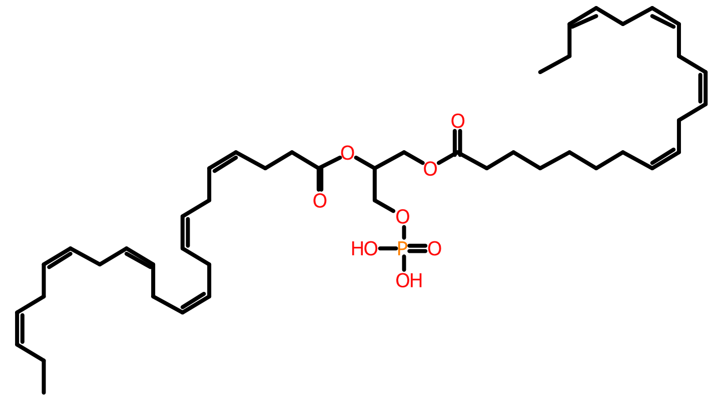

Compound information

Name: PA 20:4_22:6

Formula: C45H69O8P

Ion type: [M-H]-

Precursor m/z: 767.46573

Ccs: 264.96

InChI key:

SMILES:

Num peaks: 8

Instrument information

Database links

Other information

Spectral Match to 1-(9Z-Octadecenoyl)-sn-glycero-3-phospho-(1'-myo-inositol) from NIST14

0.804

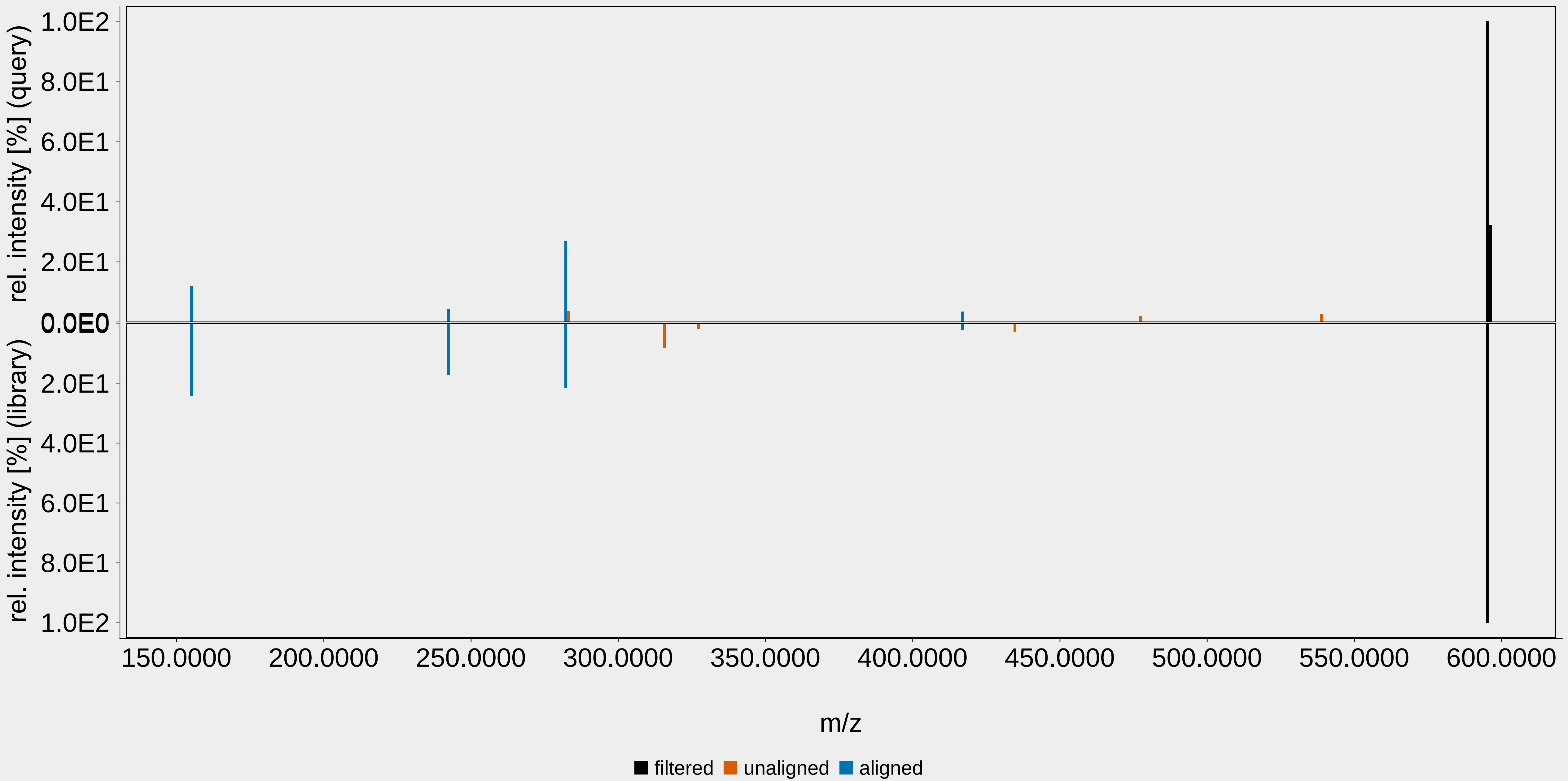

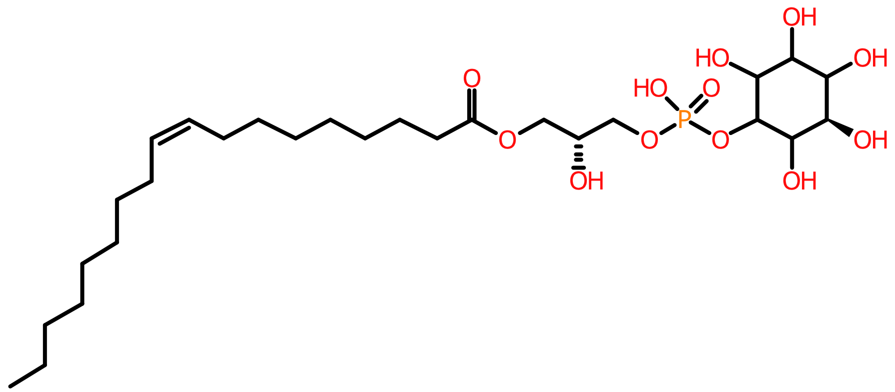

Compound information

Name: Spectral Match to

Exact mass: 598.312

Ion type: M-H

Precursor m/z: 597.3

Charge: 1

Polarity: Negative

InChI:

SMILES:

Instrument information

Instrument type: Ion Trap

Ion source: ESI

Ms level: 2

Acquisition: Isolated

Database links

CAS: 1114770158

Other information

Principal investigator: Data from Pieter

Data collector: Data deposited by lfnothias

Entry ID: CCMSLIB00003134872

LPI 18:1

0.934

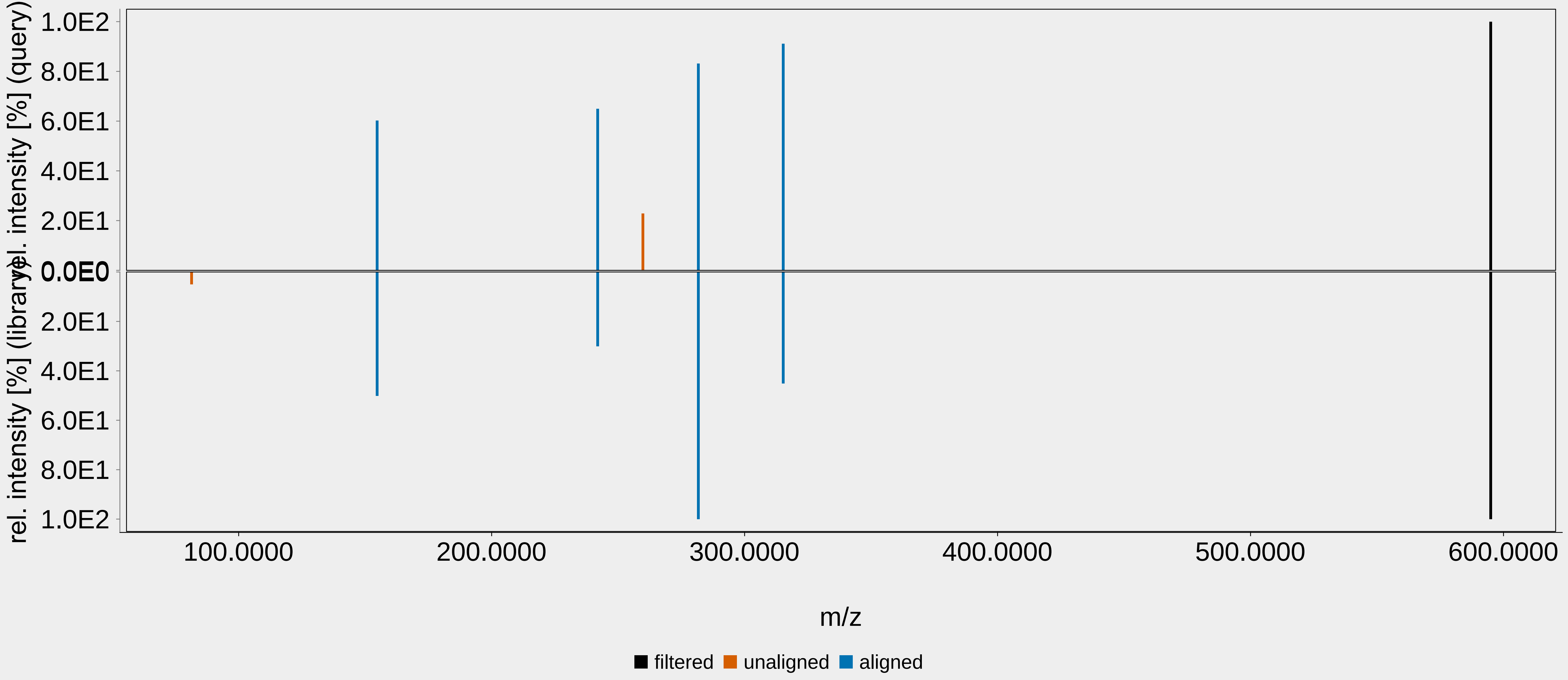

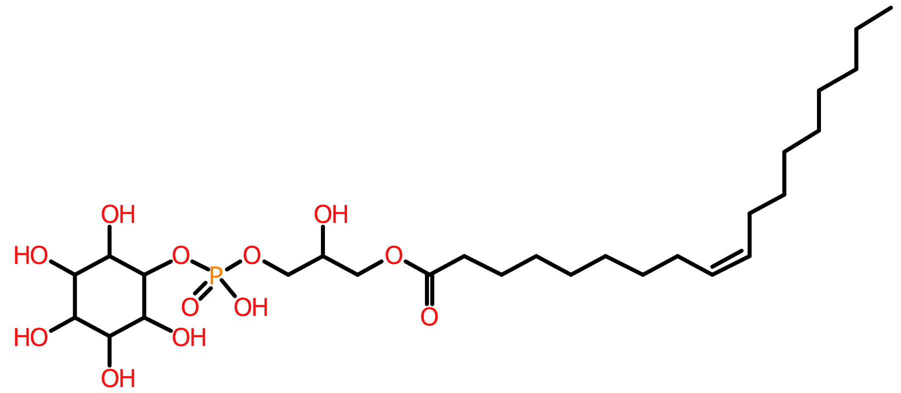

Compound information

Name: LPI 18:1

Formula: C27H51O12P

Ion type: [M-H]-

Precursor m/z: 597.30454

Ccs: 231.61

InChI key:

SMILES:

Num peaks: 6

Instrument information

Database links

Other information

PS 22:0_18:1 **0.798**

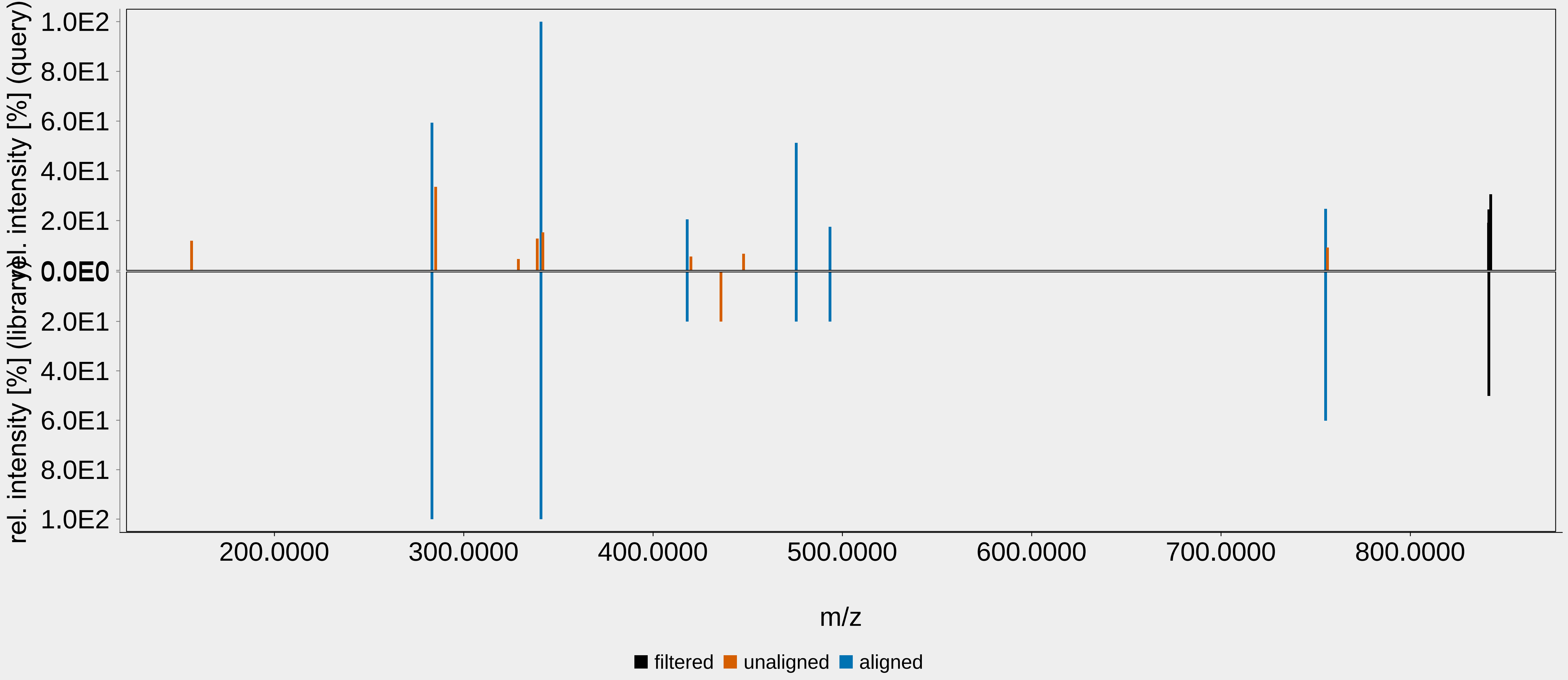

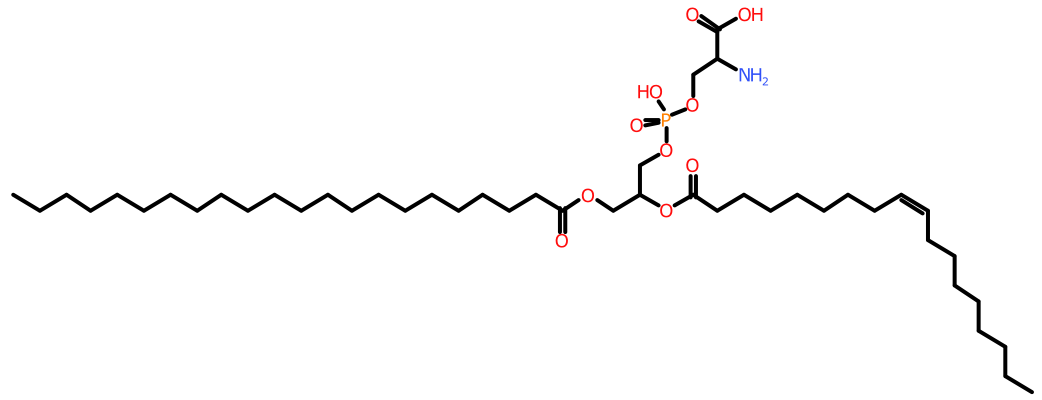

Compound information

Name: PS 22:0_18:1

Formula: C46H88NO10P

Ion type: [M-H]-

Precursor m/z: 844.60731

Ccs: 289.29

InChI key:

SMILES:

Num peaks: 8

Instrument information

Database links

Other information

PE(18:0/20:1); [M-H]- C43H83N1O8P1 0.893

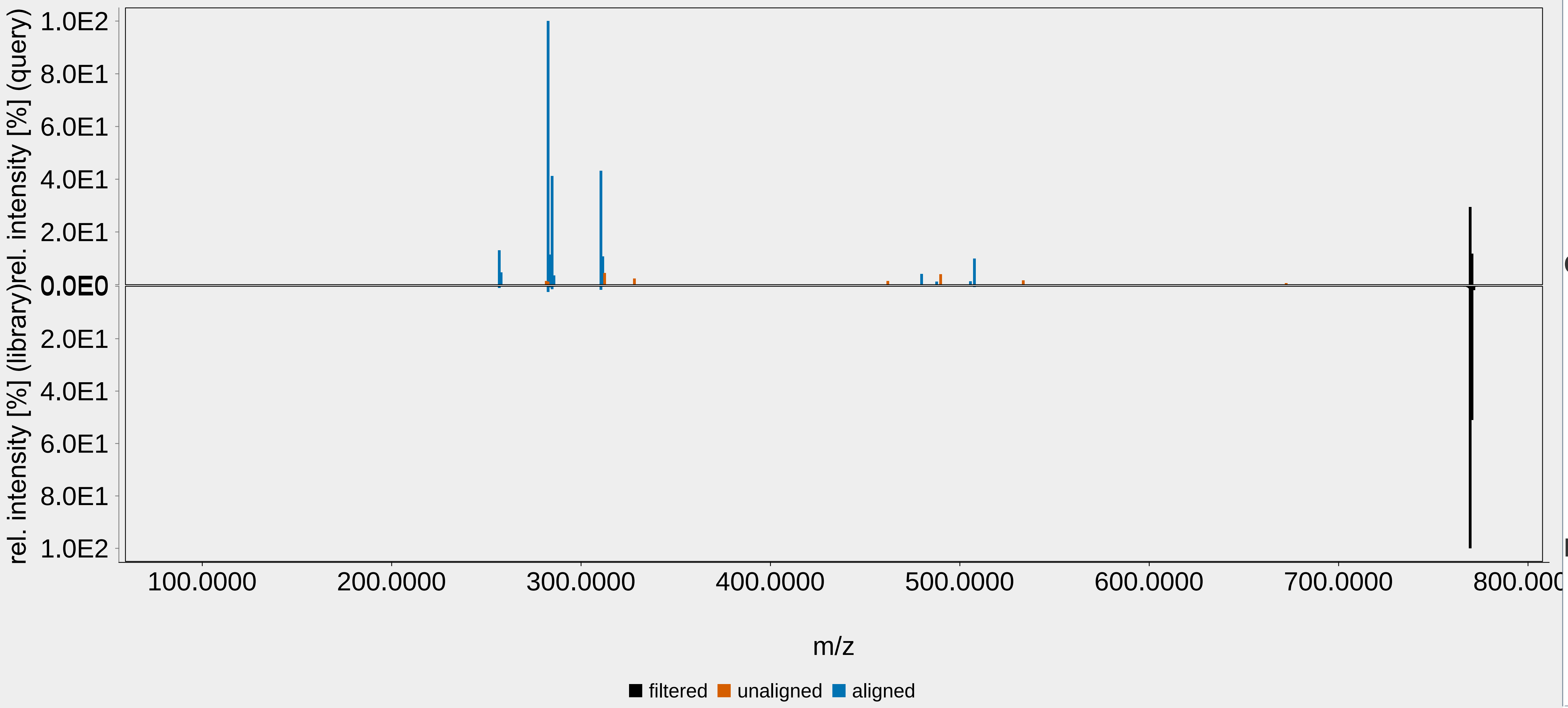

Compound information

Name: PE(18:0/20:1); [M-H]- C43H83N1O8P1

Exact mass: 0.0

Ion type: M-H

Precursor m/z: 772.586

Charge: 1

Polarity: Negative

Instrument information

Instrument type: HCD; Velos

Ion source: LC-ESI

Ms level: 2

Acquisition: Commercial

Database links

Other information

Principal investigator: Thomas Metz

Data collector: Thomas Metz

Entry ID: CCMSLIB00010068369

PE(18:0/20:1); [M-H]- C43H83N1O8P1 0.942

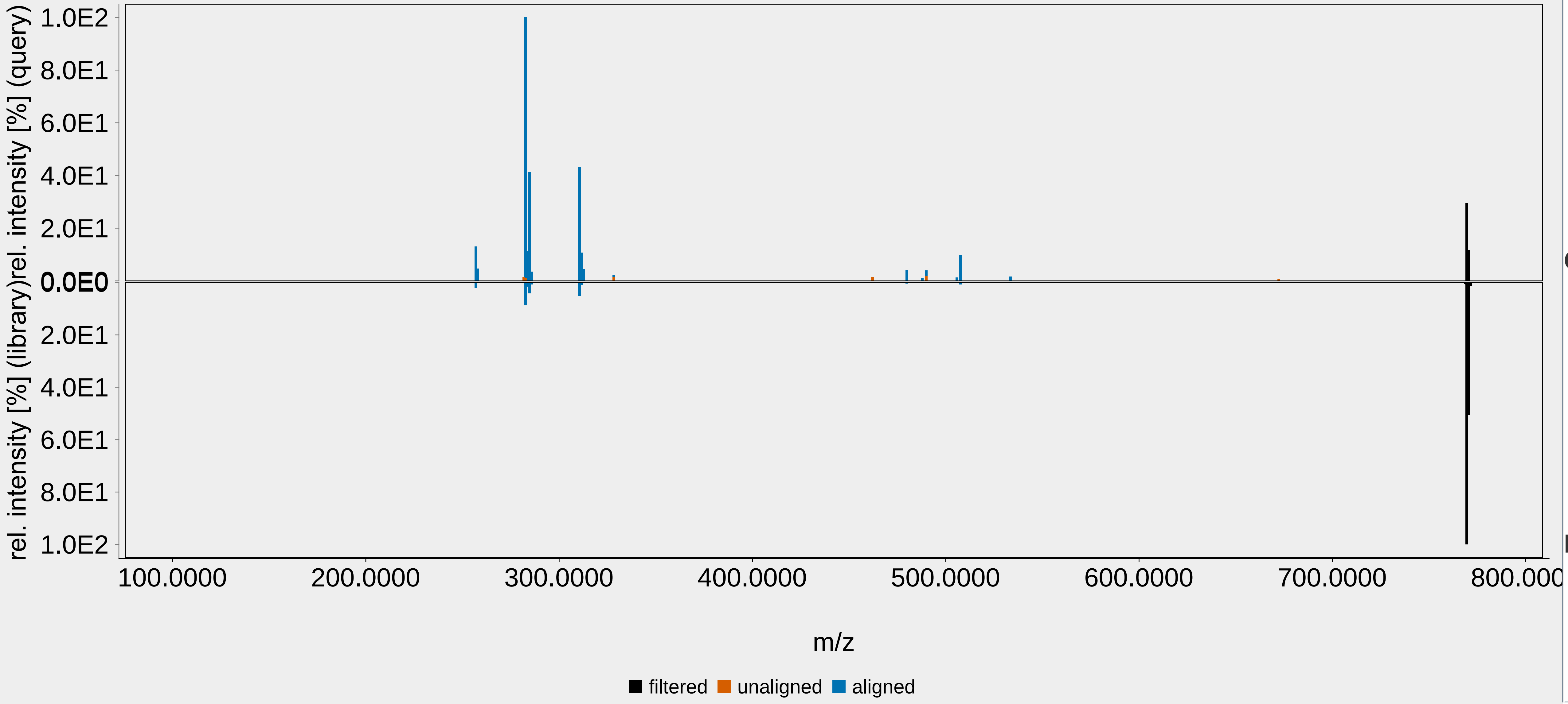

Compound information

Name: PE(18:0/20:1); [M-H]- C43H83N1O8P1

Exact mass: 0.0

Ion type: M-H

Precursor m/z: 772.586

Charge: 1

Polarity: Negative

Instrument information

Instrument type: HCD; Velos

Ion source: LC-ESI

Ms level: 2

Acquisition: Commercial

Database links

Other information

Principal investigator: Thomas Metz

Data collector: Thomas Metz

Entry ID: CCMSLIB00010068147

PI 38:5

0.825

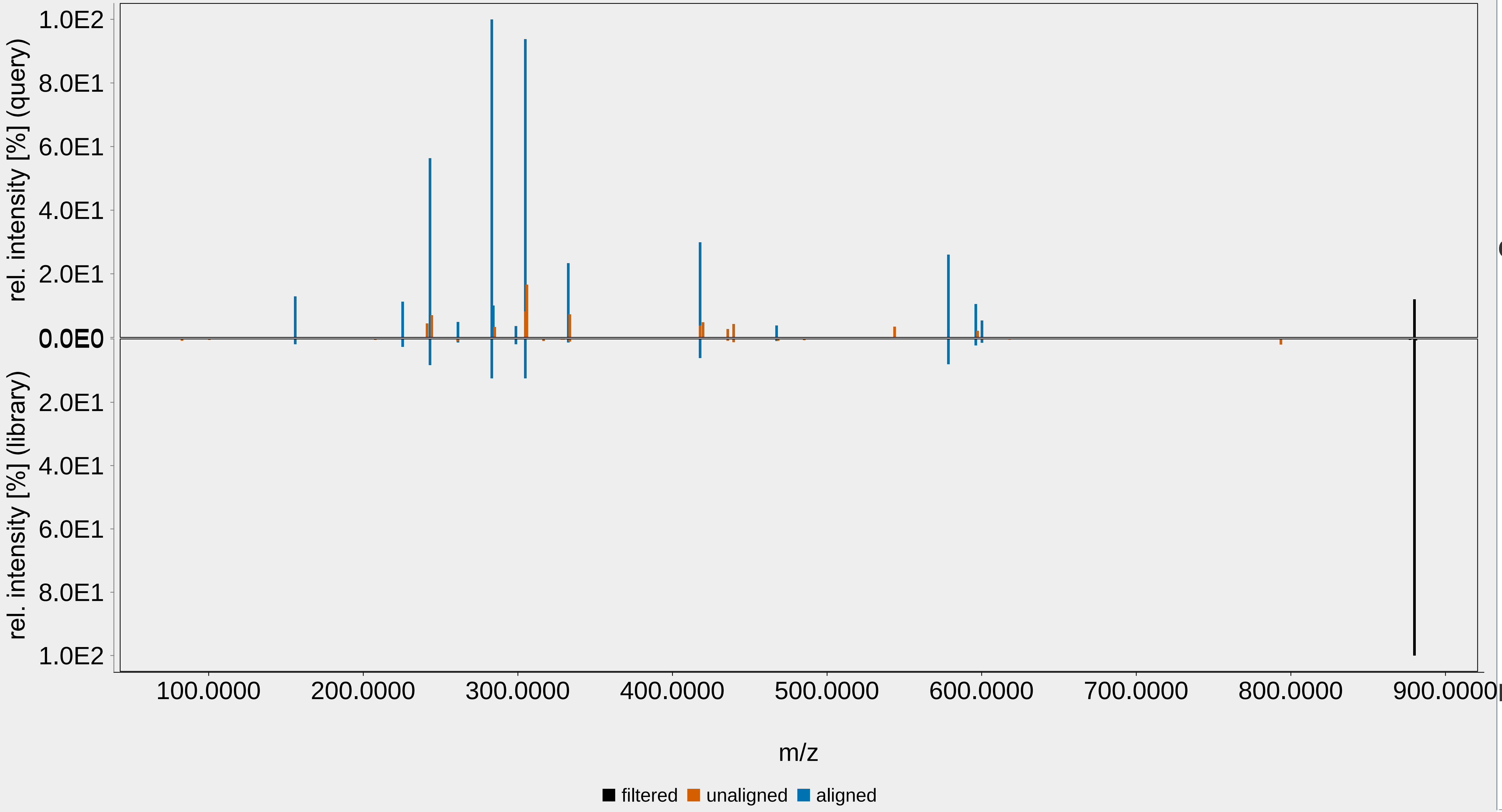

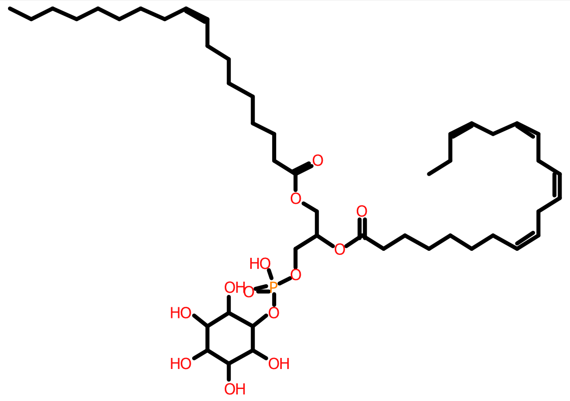

Compound information

Name: PI 38:5

Formula: C47H81O13P

Mol. weight: 885.1279999999999

Exact mass: 885.1279999999999

Ion type: [M-H]-

Precursor m/z: 883.532

RT: 8.353221

Polarity: negative

InChI:

InChI key:

SMILES:

Instrument information

Instrument type: LC-ESI-QTOF

Instrument: AB Sciex TripleTOF 5600+ system

Ms level: MS2

Collision energy: 45V

Database links

Pubchem: 138296016

MoNA ID: LQB00702

Other information

Principal investigator: Kazutaka Ikeda, Hiroshi

Data collector: Kazutaka Ikeda, Hiroshi

PI 38:5

0.825

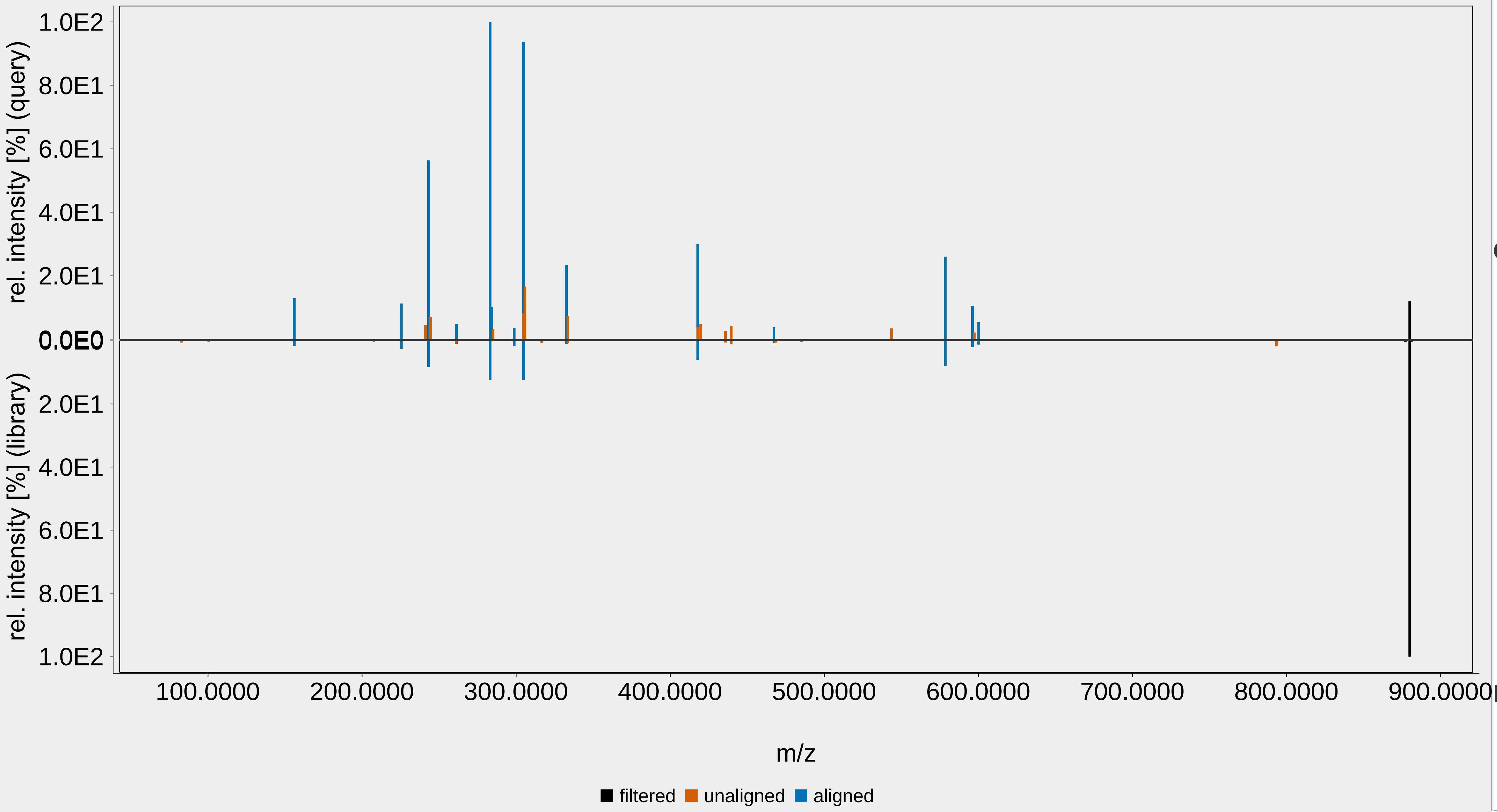

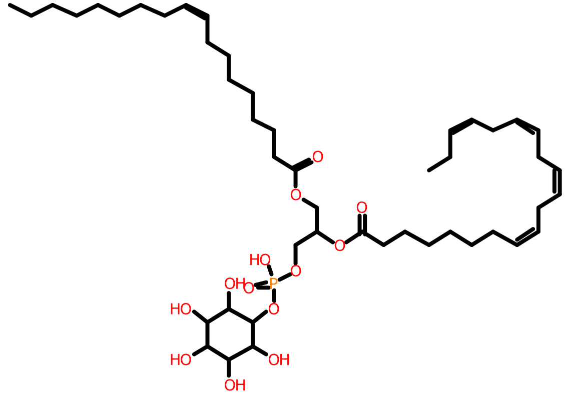

Compound information

Name: PI 38:5

Formula: C47H81O13P

Mol. weight: 885.1279999999999

Exact mass: 885.1279999999999

Ion type: [M-H]-

Precursor m/z: 883.532

RT: 8.353221

Polarity: negative

InChI:

InChI key:

SMILES:

Instrument information

Instrument type: LC-ESI-QTOF

Instrument: AB Sciex TripleTOF 5600+ system

Ms level: MS2

Collision energy: 45V

Database links

Pubchem: 138296016

MoNA ID: LQB00316

Other information

Principal investigator: Kazutaka Ikeda, Hiroshi

Data collector: Kazutaka Ikeda, Hiroshi

PI(18:0_0:0) - [2-hydroxy-3-[hydroxy-[2,3,4,5,6-pentahydroxycyclohexyl]oxyphosphoryl]oxypropyl] octadecanoate **0.778**

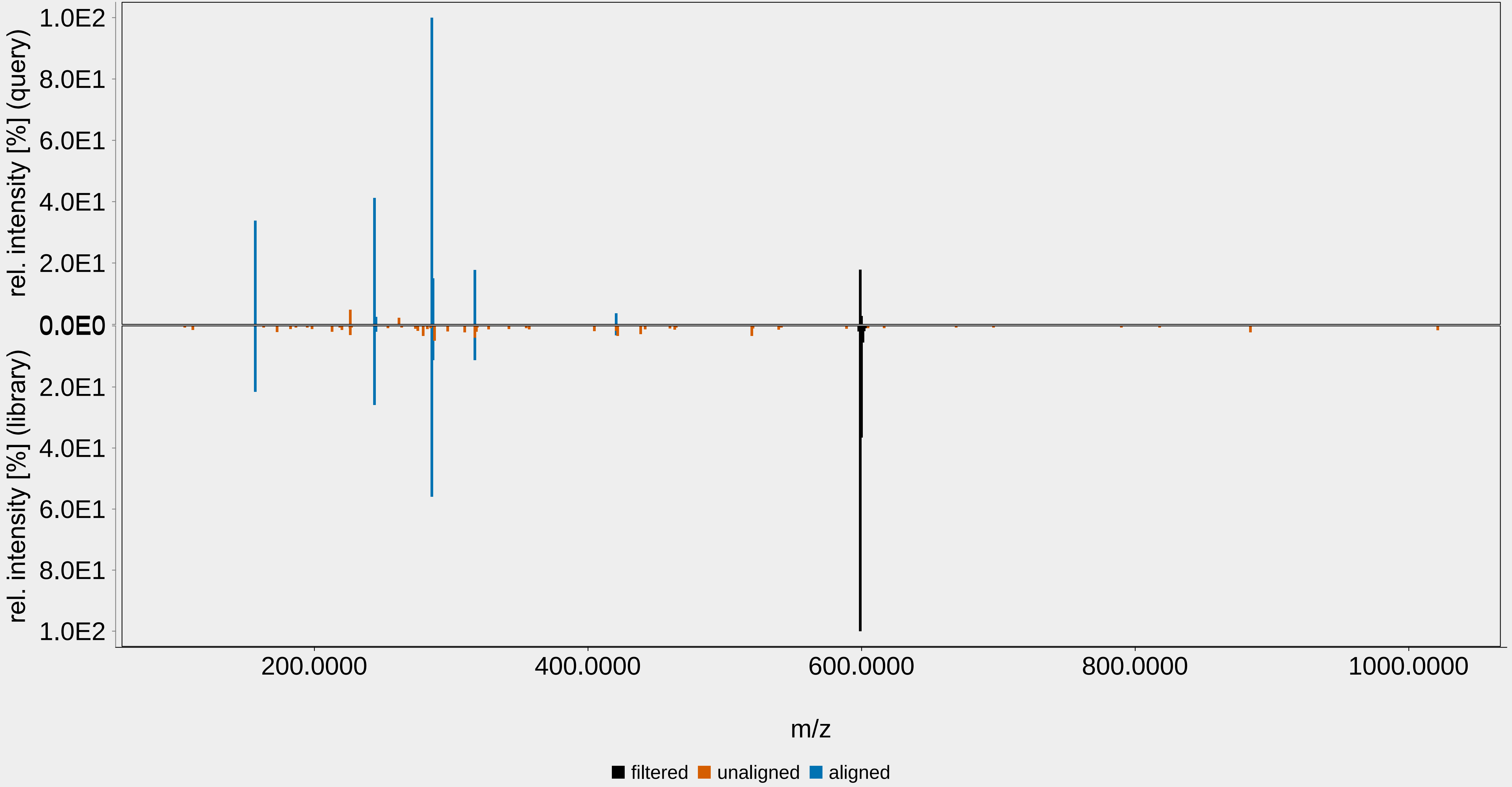

Compound information

Name: PI(18:0_0:0) -

Exact mass: 600.327

Ion type: M-H

Precursor m/z: 599.32

Charge: 1

Polarity: Negative

InChI:

InChI key:

SMILES:

Instrument information

Instrument type: qTof

Ion source: LC-ESI

Ms level: 2

Acquisition: Commercial

Database links

Pubmed:

CAS: 849412-49-3

Other information

Principal investigator: O Laprevote

Data collector: UMR 8038 Universite de Paris

Entry ID: CCMSLIB00005436451

LPI 18:0

0.948

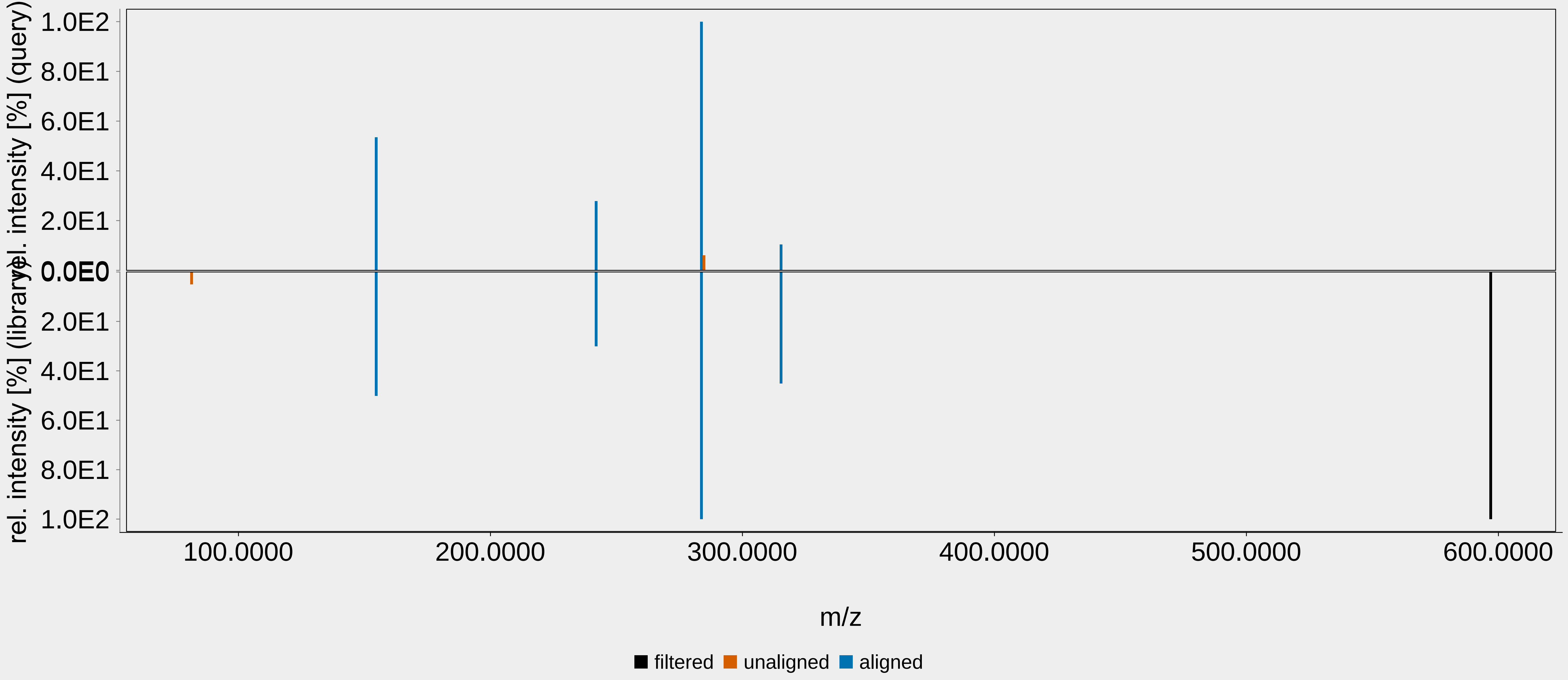

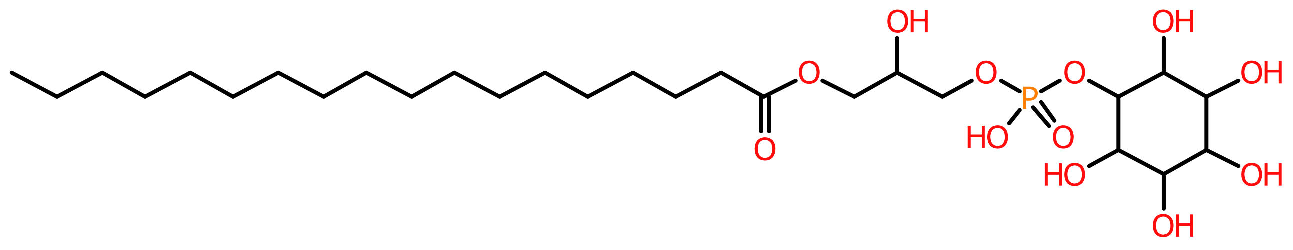

Compound information

Name: LPI 18:0

Formula: C27H53O12P

Ion type: [M-H]-

Precursor m/z: 599.32019

Ccs: 233.47

InChI key: MXAFDFDAIFZFET-UHFFFAOYNA-

SMILES:

Num peaks: 6

Instrument information

Database links

Other information

PE(18:0/18:1); [M-H]- C41H79N1O8P1 0.920

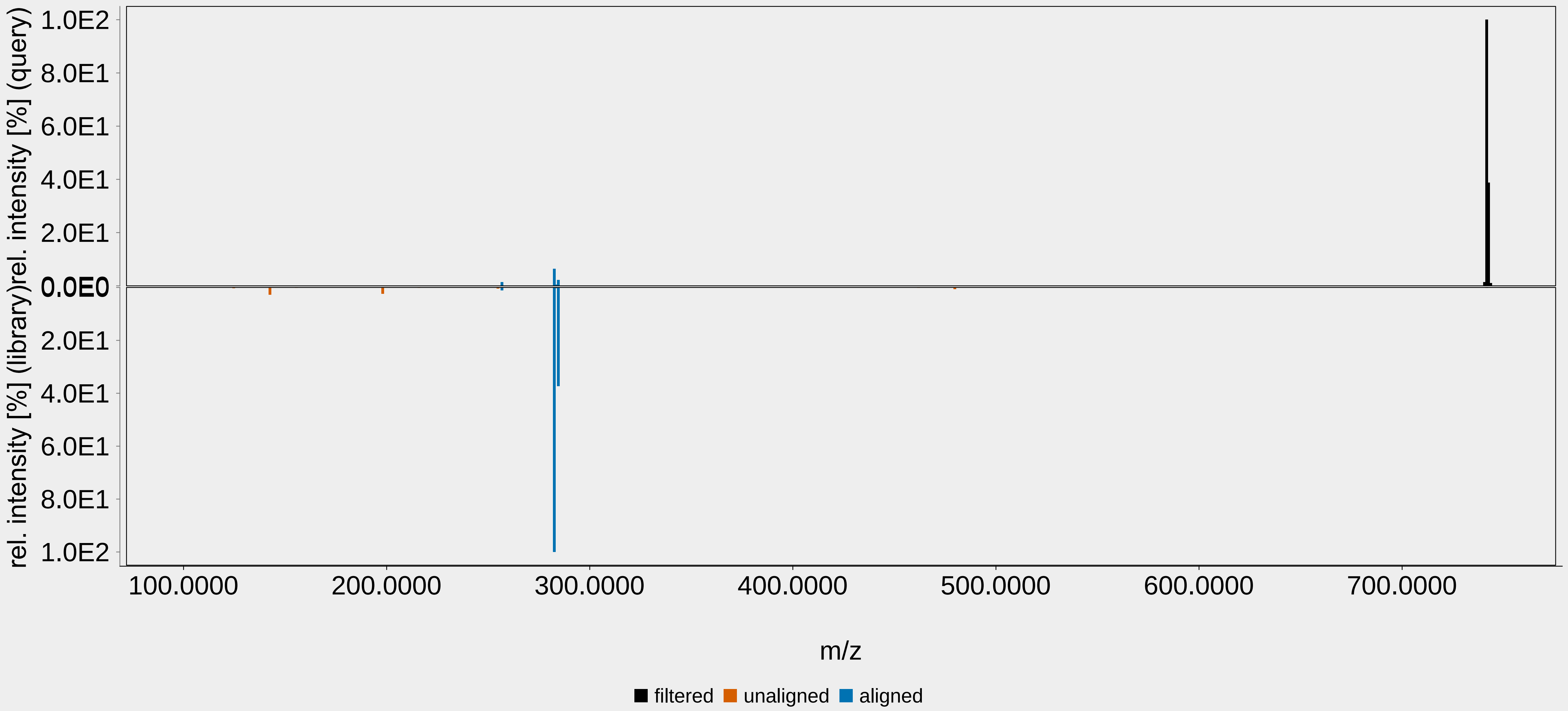

Compound information

Name: PE(18:0/18:1); [M-H]- C41H79N1O8P1

Exact mass: 0.0

Ion type: M-H

Precursor m/z: 744.554

Charge: 1

Polarity: Negative

Instrument information

Instrument type: HCD; Lumos

Ion source: LC-ESI

Ms level: 2

Acquisition: Commercial

Database links

Other information

Principal investigator: Thomas Metz

Data collector: Thomas Metz

Entry ID: CCMSLIB00010059575

PE(18:0/18:1); [M-H]- C41H79N1O8P1

0.930

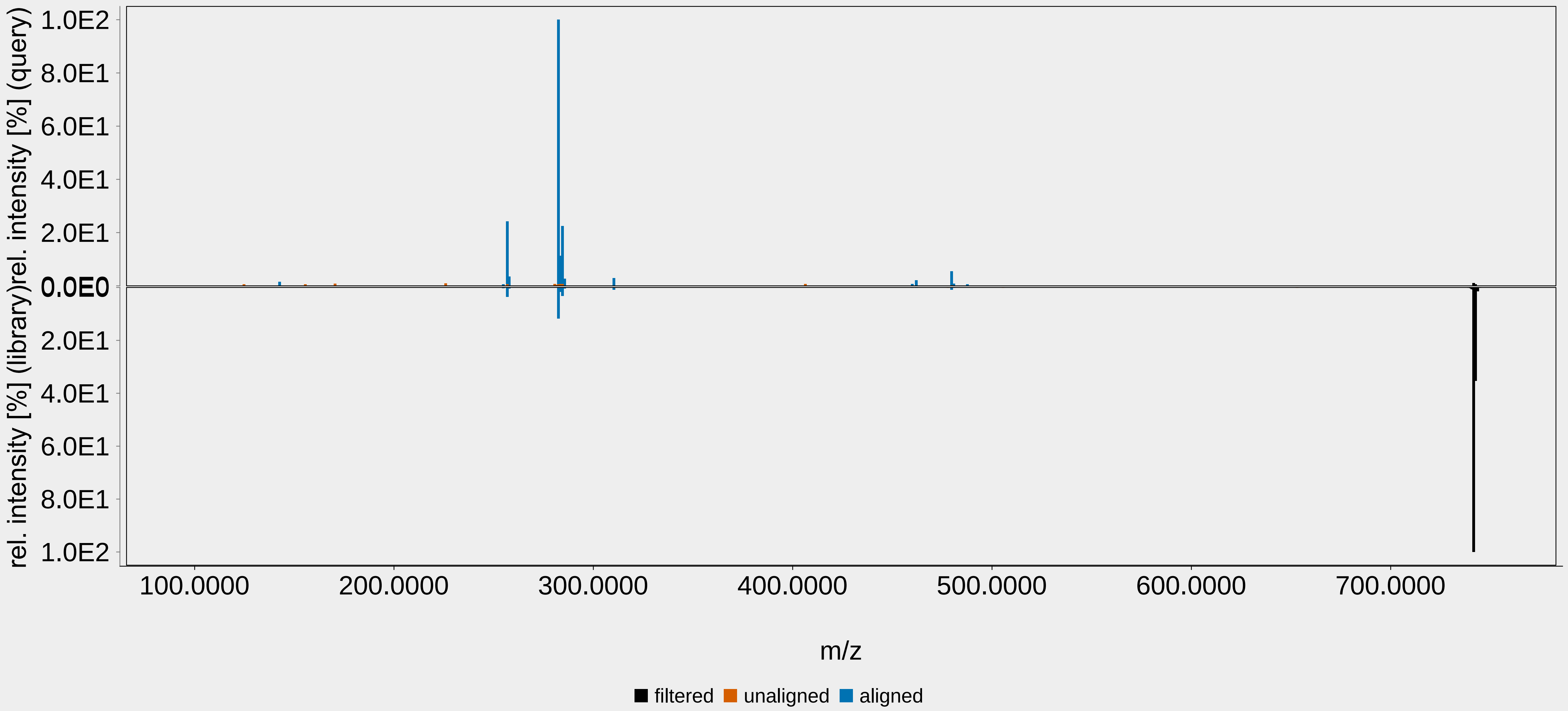

Compound information

Name: PE(18:0/18:1); [M-H]- C41H79N1O8P1

Exact mass: 0.0

Ion type: M-H

Precursor m/z: 744.554

Charge: 1

Polarity: Negative

Instrument information

Instrument type: HCD; Velos

Ion source: LC-ESI

Ms level: 2

Acquisition: Commercial

Database links

Other information

Principal investigator: Thomas Metz

Data collector: Thomas Metz

Entry ID: CCMSLIB00010072259

PE(18:1/22:6); [M-H]- C45H75N1O8P1 0.910

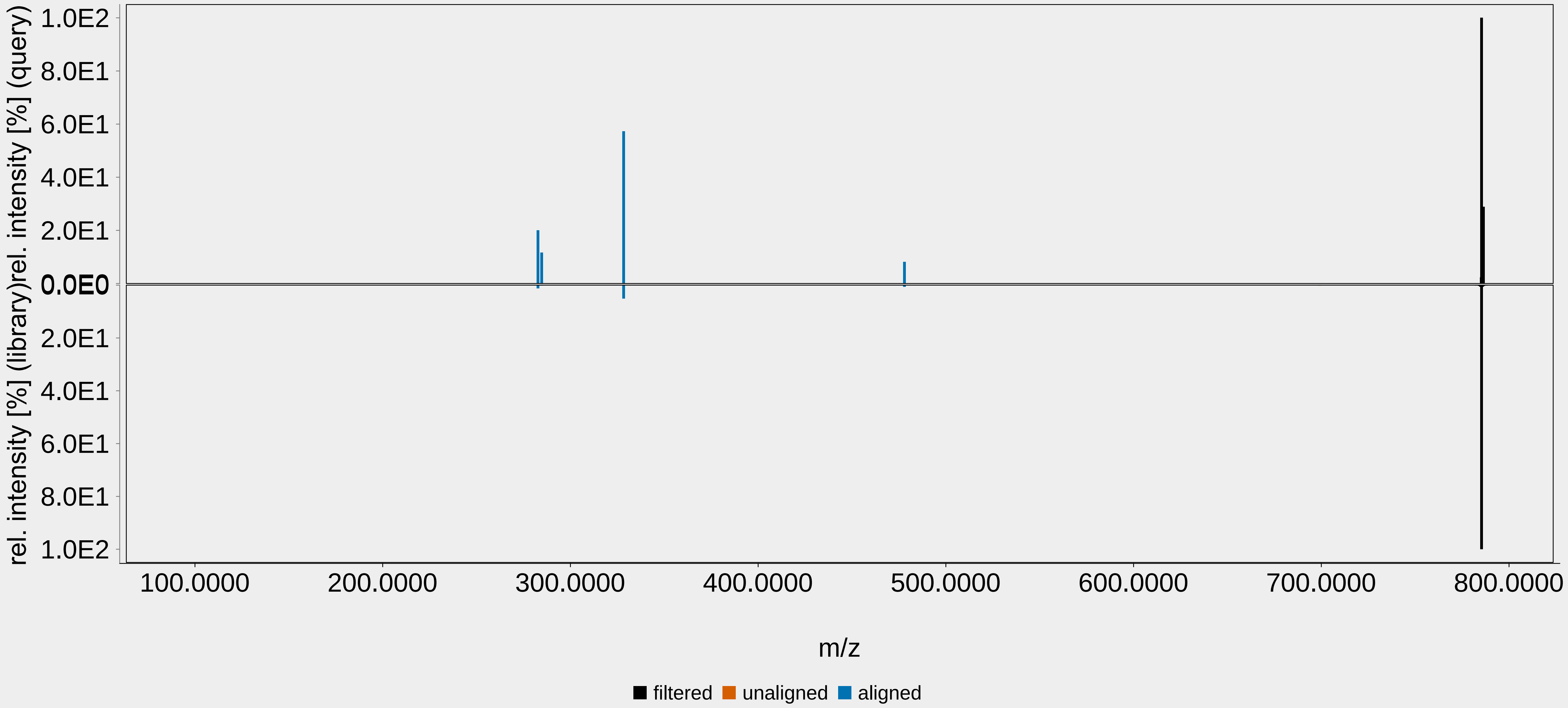

Compound information
Name: PE(18:1/22:6); [M-H]- C45H75N1O8P1

Exact mass: 0.0

Ion type: M-H

Precursor m/z: 788.523

Charge: 1

Polarity: Negative

Instrument information
Instrument type: HCD; Velos

Ion source: LC-ESI

Ms level: 2

Acquisition: Commercial

Database links

Other information
Principal investigator: Thomas Metz

Data collector: Thomas Metz

Entry ID: CCMSLIB00010063779

PE(18:1/22:6); [M-H]- C45H75N1O8P1 **0.923**

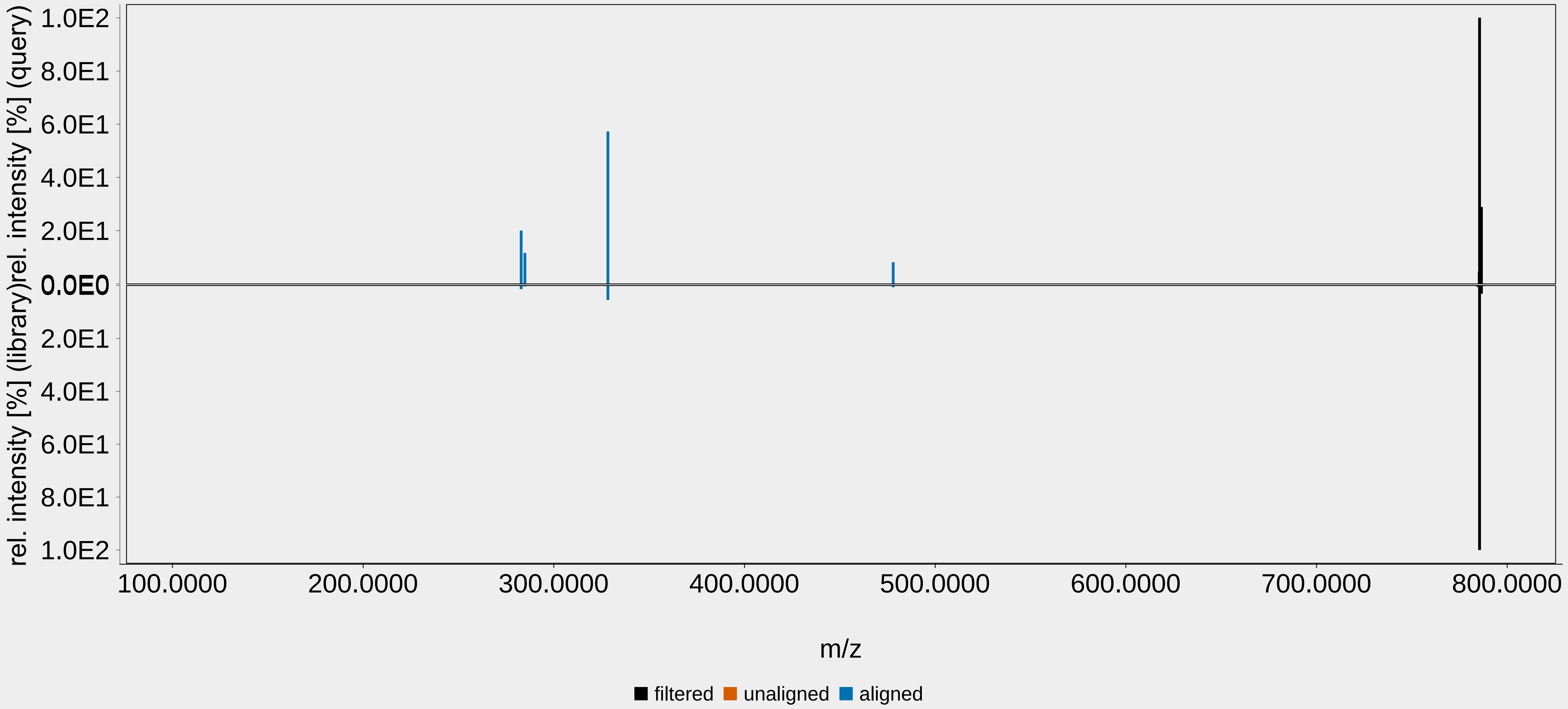

Compound information

Name: PE(18:1/22:6); [M-H]- C45H75N1O8P1

Exact mass: 0.0

Ion type: M-H

Precursor m/z: 788.523

Charge: 1

Polarity: Negative

Instrument information

Instrument type: HCD; Velos

Ion source: LC-ESI

Ms level: 2

Acquisition: Commercial

Database links

Other information

Principal investigator: Thomas Metz

Data collector: Thomas Metz

Entry ID: CCMSLIB00010073275

Adenosine monophosphate - 30.0 eV

0.806

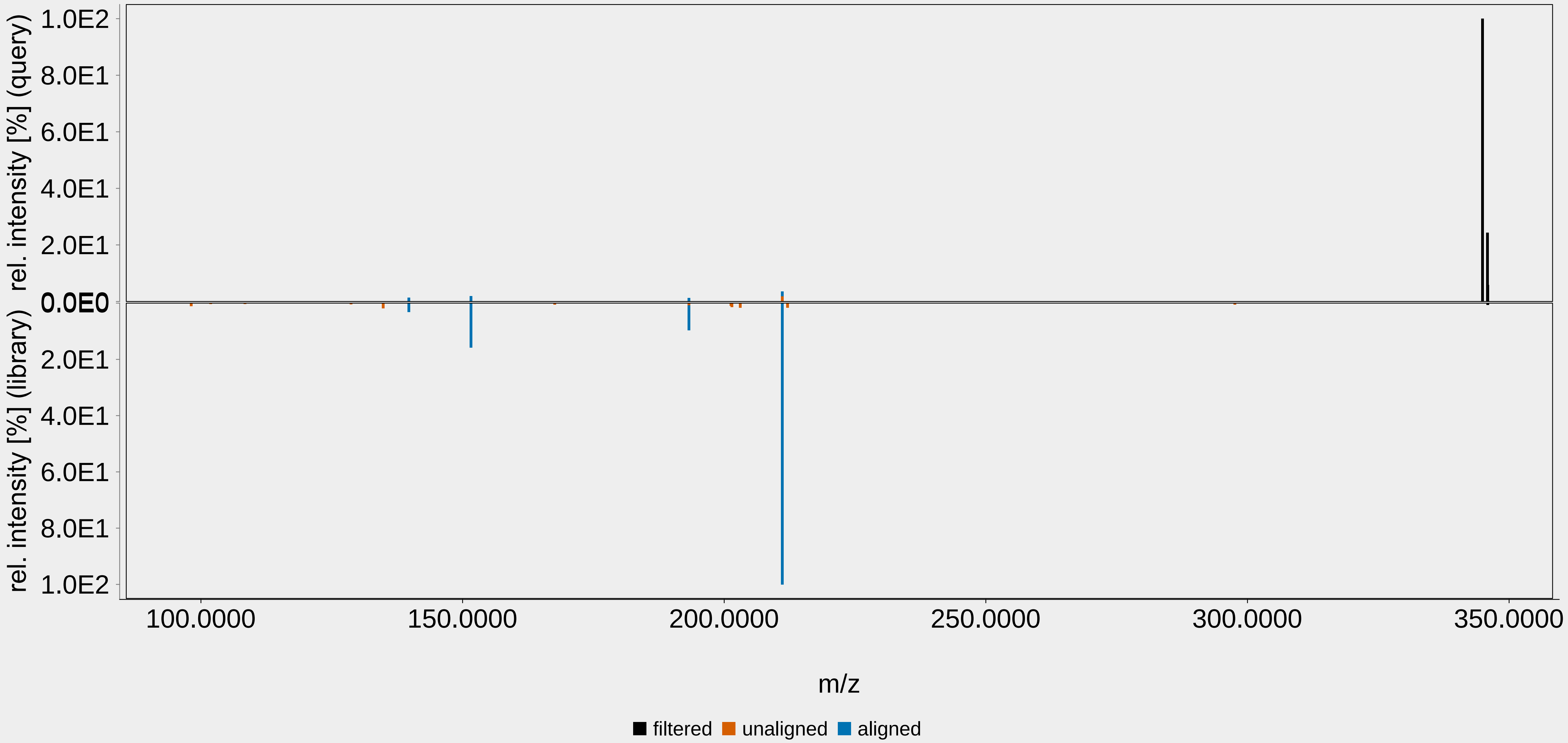

Compound information

Name: Adenosine monophosphate - 30.0 eV

Exact mass: 0.0

Ion type: M-H

Precursor m/z: 346.056

Charge: 1

Polarity: Negative

SMILES:

Instrument information

Instrument type: Orbitrap

Ion source: ESI

Ms level: 2

Acquisition: Isolated

Database links

CAS:

Other information

Principal investigator: PI

Data collector: Data Collector

Entry ID: CCMSLIB00006114160

Adenosine monophosphate - 30.0 eV

0.800

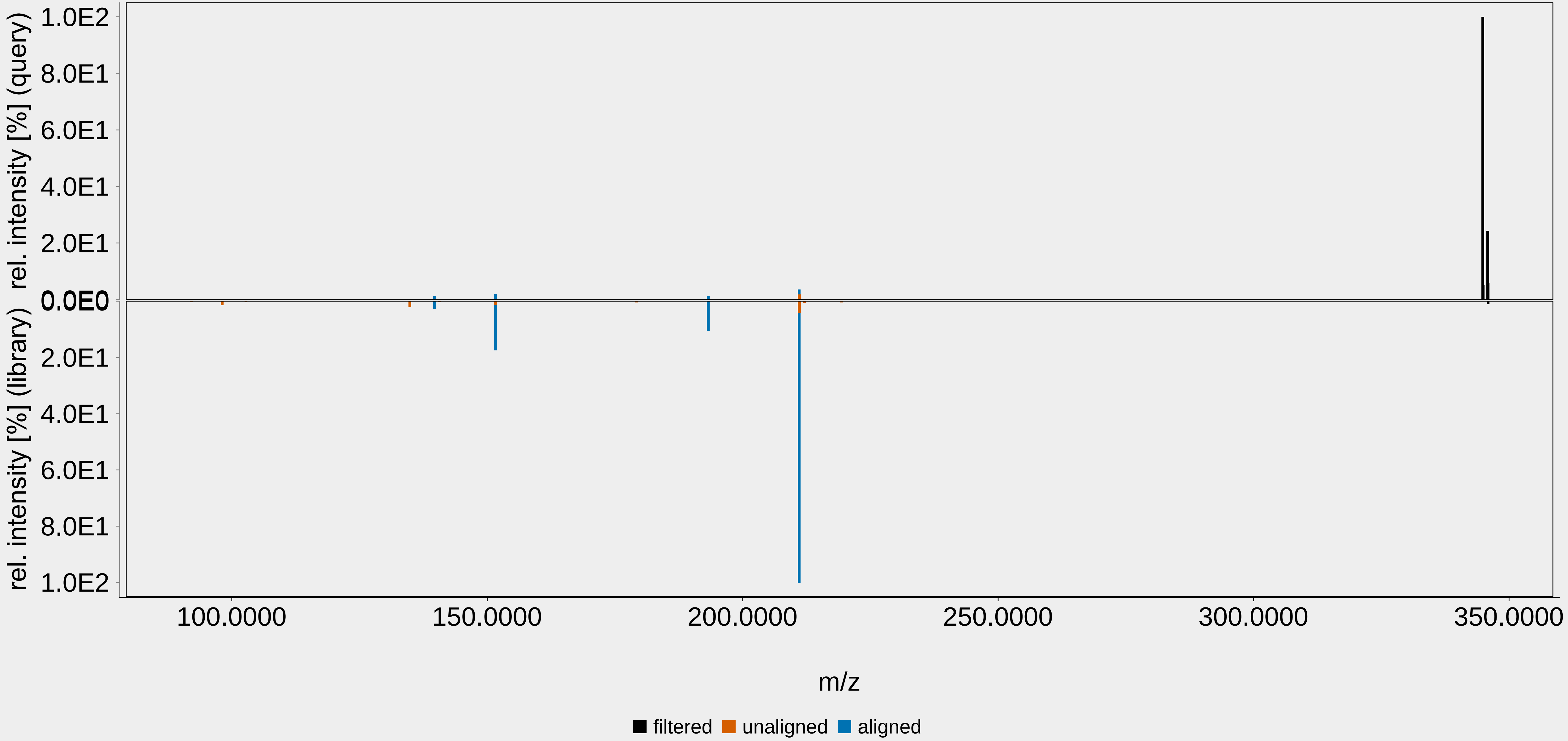

Compound information

Name: Adenosine monophosphate - 30.0 eV

Exact mass: 0.0

Ion type: M-H

Precursor m/z: 346.056

Charge: 1

Polarity: Negative

SMILES:

Instrument information

Instrument type: Orbitrap

Ion source: ESI

Ms level: 2

Acquisition: Isolated

Database links

CAS:

Other information

Principal investigator: PI

Data collector: Data Collector

Entry ID: CCMSLIB00006114141

[5-acetyloxy-3-(hydroxymethyl)-2-oxo-6-propan-2-ylcyclohex-3-en-1-yl] 3-methylbutanoate

0.793

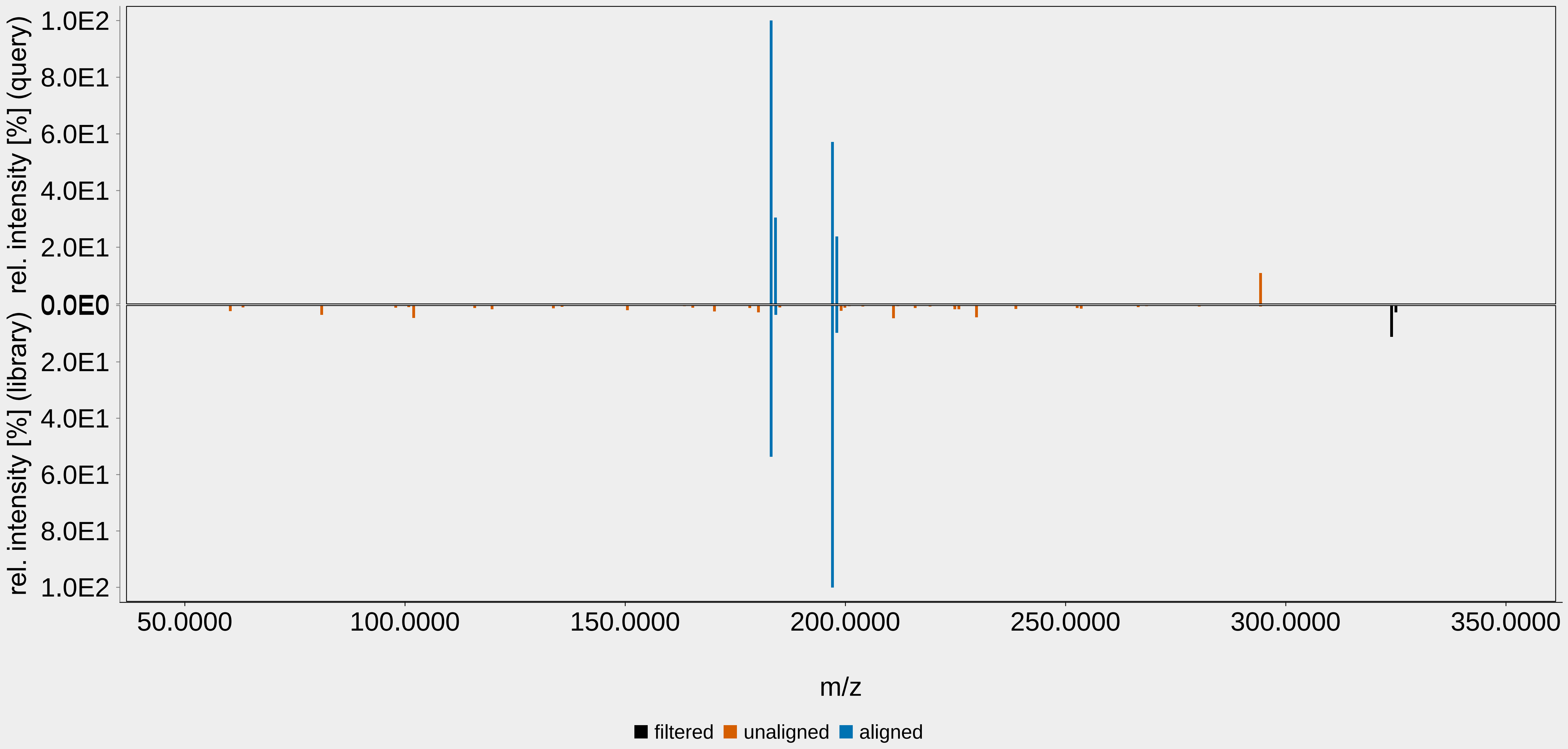

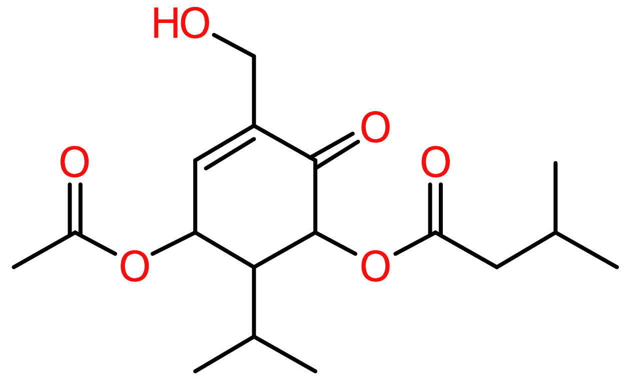

Compound information

Name:

Formula: C17H26O6

Ion type: [M-H]-

Precursor m/z: 325.1656494140625

Polarity: negative

InChI:

InChI key: IVEJMWCGEFICJC-UHFFFAOYSA-

SMILES:

Instrument information

Instrument type: ESI-QFT

Instrument: Thermo Q Exactive HF

Ms level: MS2

Collision energy: 65HCD

Database links

Other information

Principal investigator: Arpana Vaniya

Data collector: Arpana Vaniya

[5-acetyloxy-3-(hydroxymethyl)-2-oxo-6-propan-2-ylcyclohex-3-en-1-yl] 3-methylbutanoate

0.793

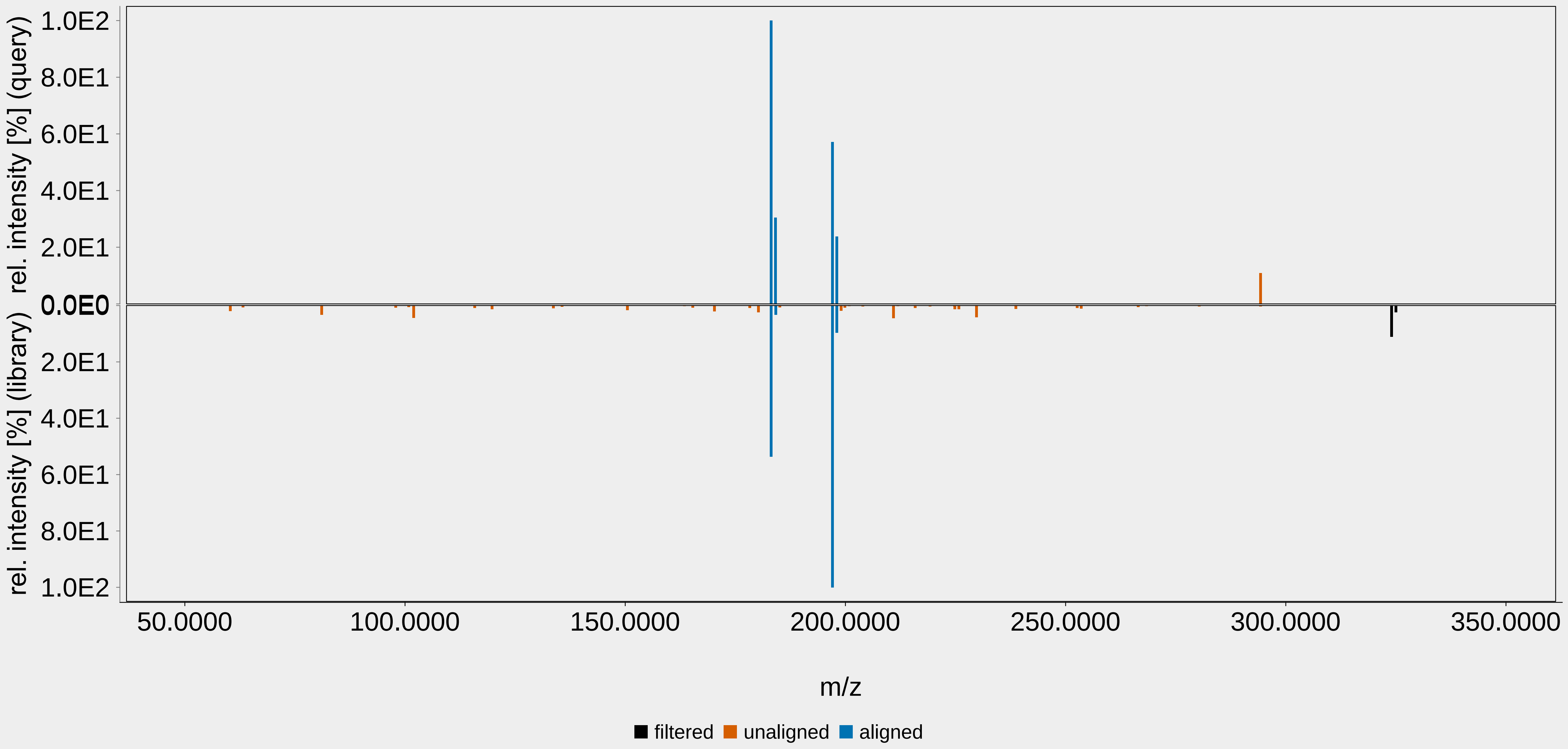

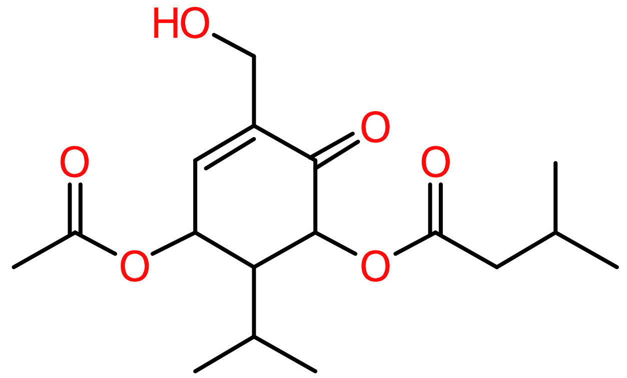

Compound information

Name:

Exact mass: 0.0

Ion type: [M-H]-

Precursor m/z: 325.166

Charge: 1

Polarity: negative

InChI:

Instrument information

Instrument type: ESI-QFT

Ms level: 2

Acquisition: isolated

Database links

Other information

Principal investigator: MoNA

Data collector: MoNA:VF-NPL-QEHF008244

Entry ID: CCMSLIB00004699307

PE(18:1/0:0); [M-H]- C23H45N1O7P1

0.949

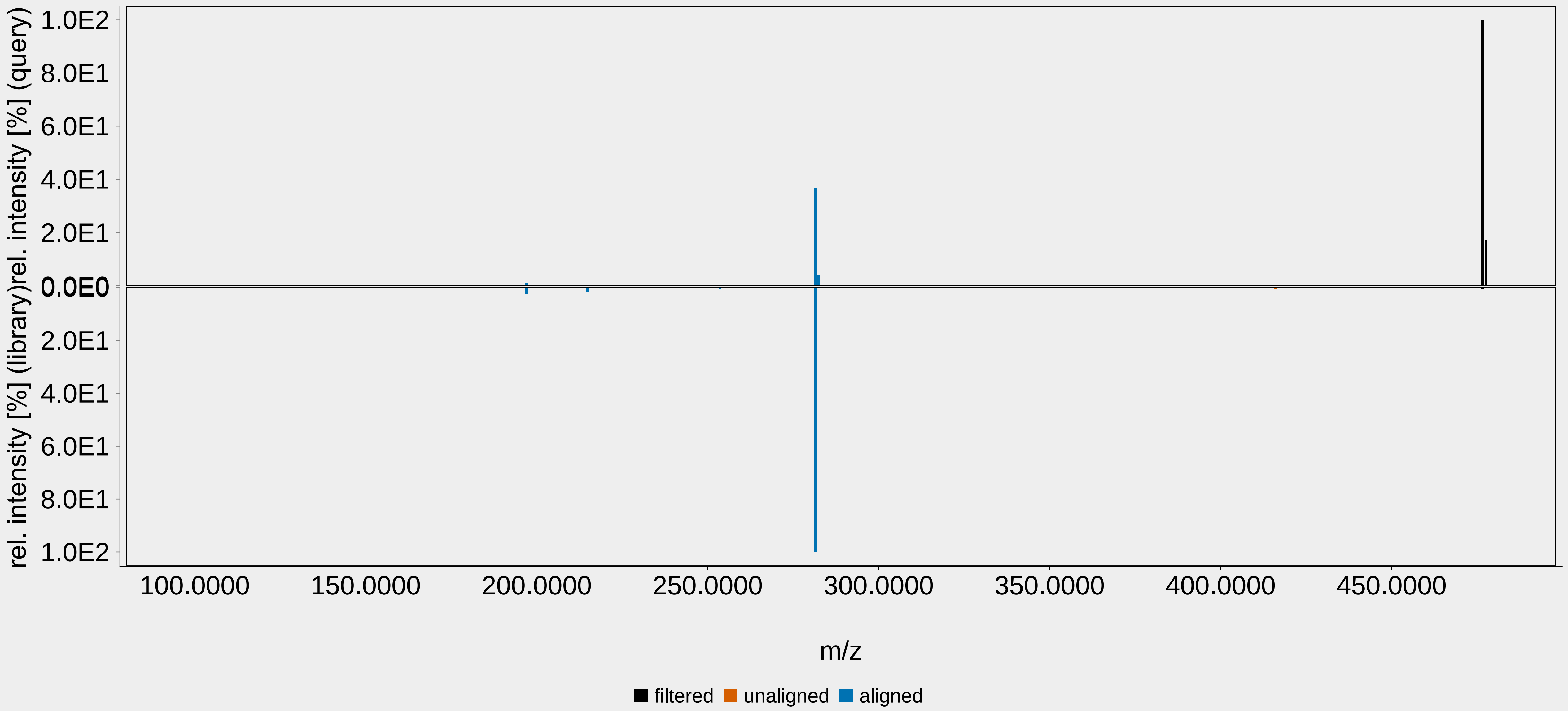

Compound information

Name: PE(18:1/0:0); [M-H]- C23H45N1O7P1

Exact mass: 0.0

Ion type: M-H

Precursor m/z: 478.293

Charge: 1

Polarity: Negative

Instrument information

Instrument type: CID; Lumos

Ion source: LC-ESI

Ms level: 2

Acquisition: Commercial

Database links

Other information

Principal investigator: Thomas Metz

Data collector: Thomas Metz

Entry ID: CCMSLIB00010059278

PE(18:1/0:0); [M-H]- C23H45N1O7P1 **0.950**

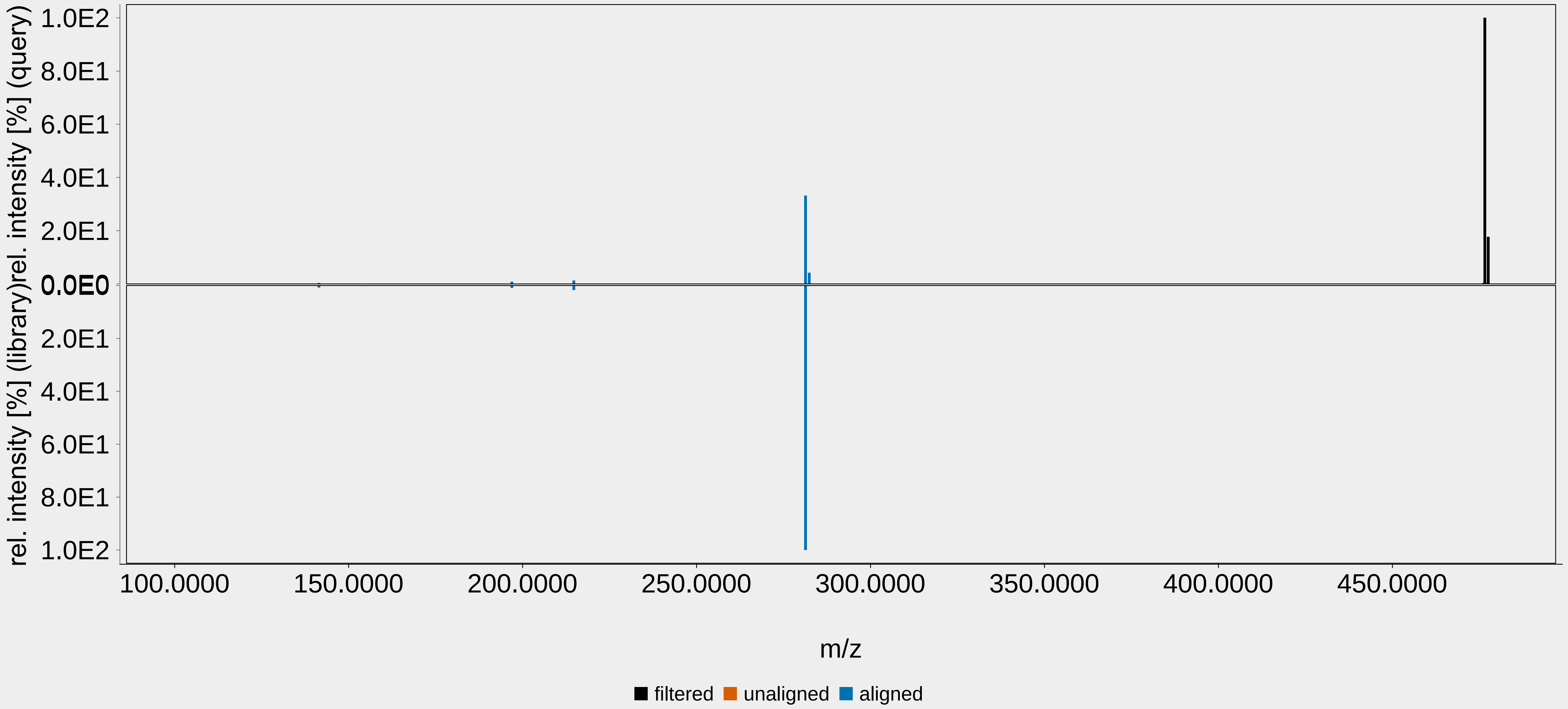

Compound information

Name: PE(18:1/0:0); [M-H]- C23H45N1O7P1

Exact mass: 0.0

Ion type: M-H

Precursor m/z: 478.293

Charge: 1

Polarity: Negative

Instrument information

Instrument type: HCD; Lumos

Ion source: LC-ESI

Ms level: 2

Acquisition: Commercial

Database links

Other information

Principal investigator: Thomas Metz

Data collector: Thomas Metz

Entry ID: CCMSLIB00010059197

C10-LAS (TENTATIVE)

0.899

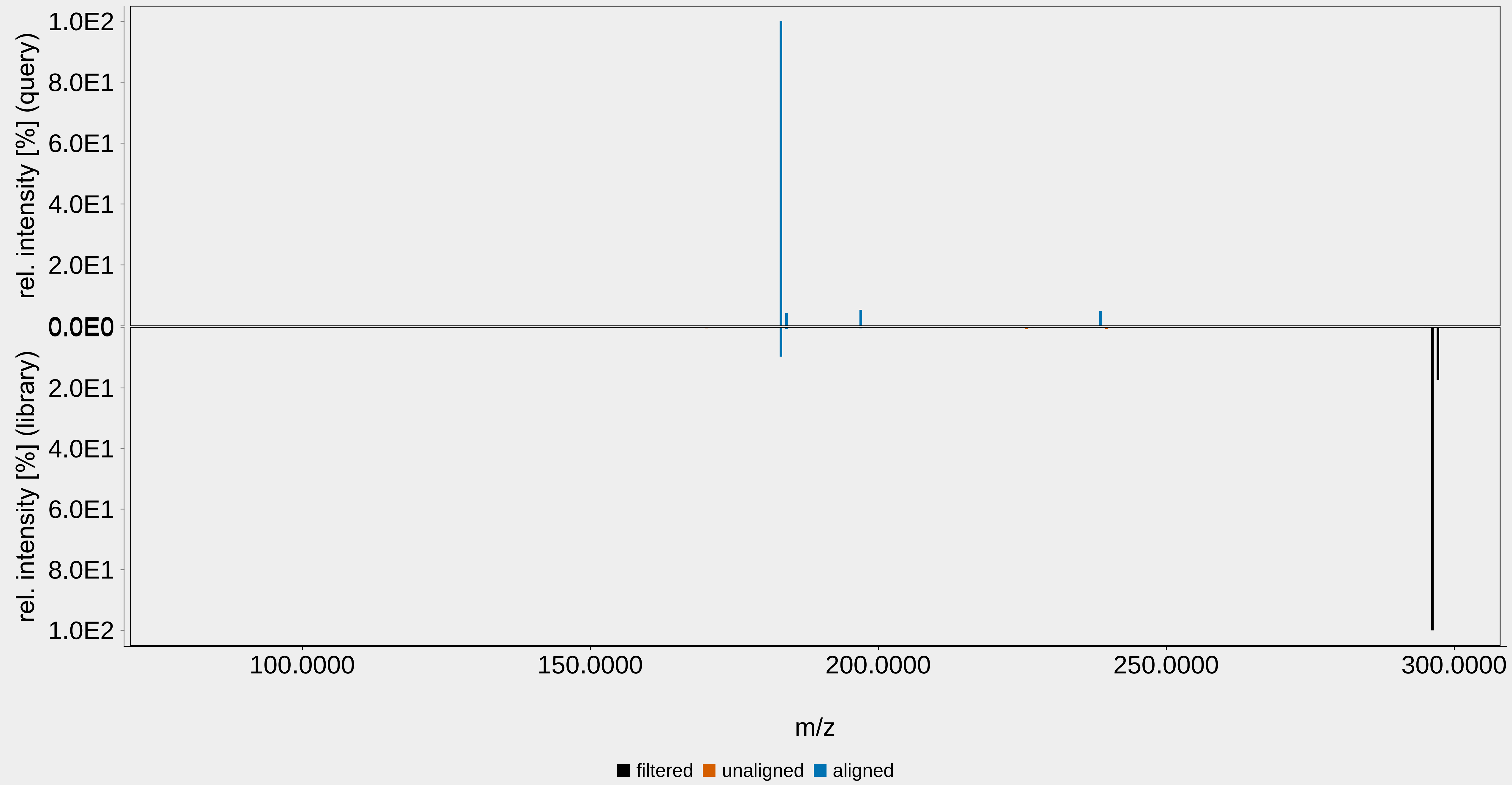

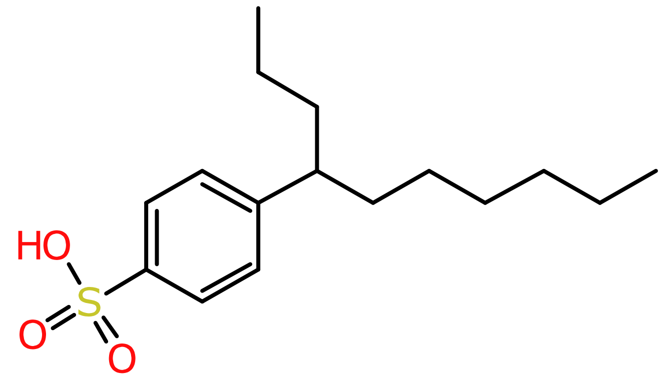

Compound information

Name: C10-LAS (TENTATIVE)

Formula: C16H26O3S

Exact mass: 298.1603

Ion type: [M-H]-

Precursor m/z: 297.1521

Polarity: NEGATIVE

InChI:

InChI key: NANHIUZYPFDGJS-UHFFFAOYSA-

SMILES:

Num peaks: 20

Instrument information

Instrument type: LC-ESI-ITFT

Instrument: LTQ Orbitrap XL Thermo Scientific

Ms level: MS2

Collision energy: 60 NCE

Database links

Other information

Entry ID:

Comment: Parent=297.1521

C10-LAS (TENTATIVE), C10-linear alkylbenzyl sulfonate, 4-(decan-4-yl)benzenesulfonic acid

0.900

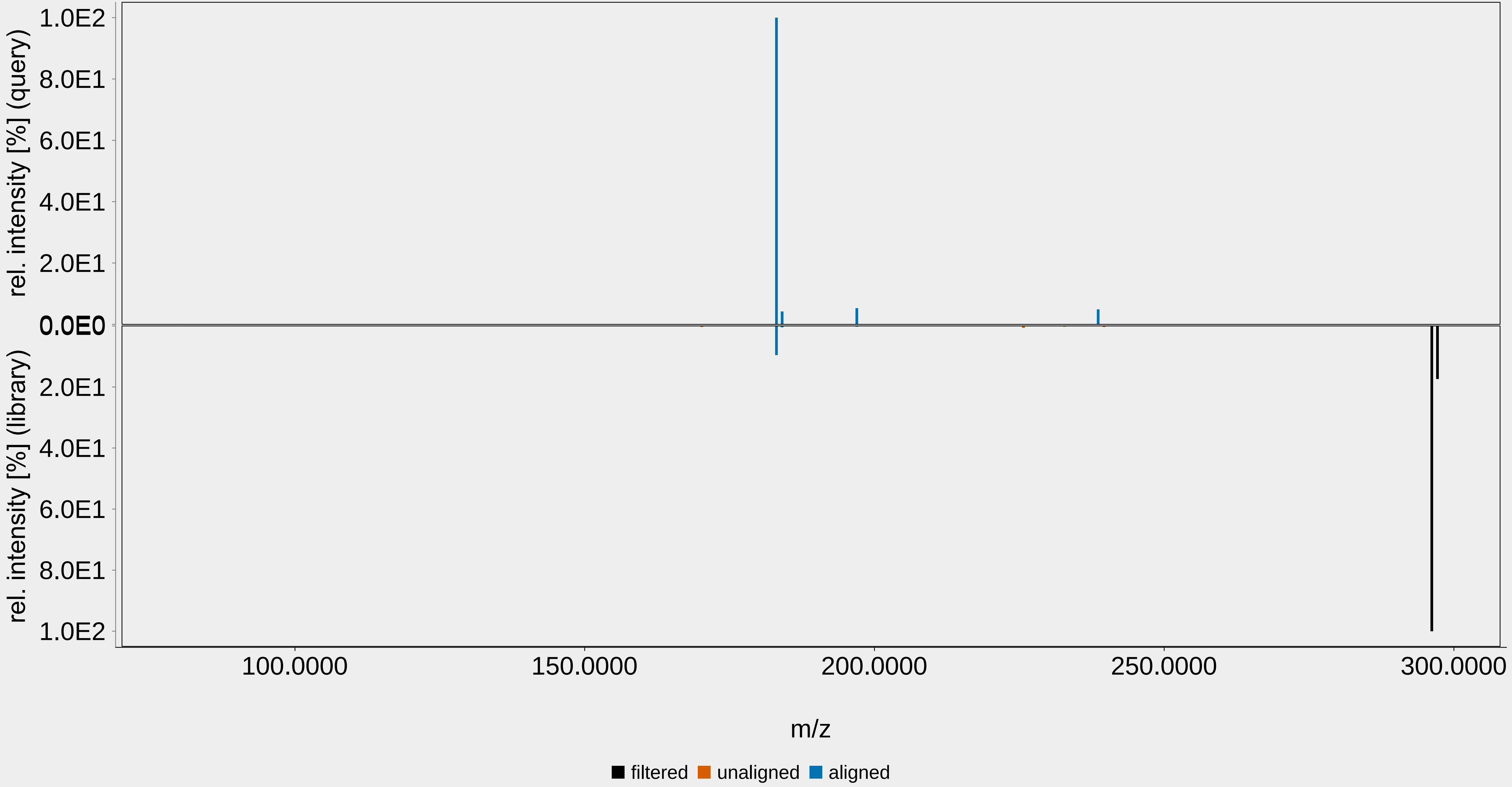

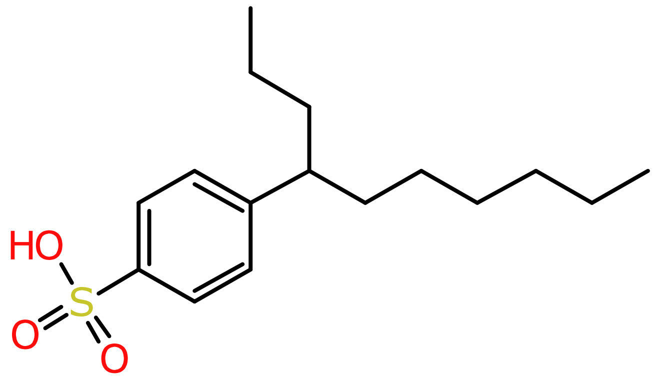

Compound information

Name: C10-LAS (TENTATIVE), C10-linear

Formula: C16H26O3S

Mol. weight: 298.1603

Exact mass: 298.1603

Ion type: [M-H]-

Precursor m/z: 297.1521

Polarity: negative

InChI:

InChI key: NANHIUZYPFDGJS-UHFFFAOYSA-

SMILES:

Instrument information

Instrument type: LC-ESI-ITFT

Instrument: LTQ Orbitrap XL Thermo Scientific

Ion source: ESI

Ms level: MS2

Collision energy: 60 NCE

Database links

Pubchem: 14921167

MoNA ID: ETS00001

Other information

Principal investigator: E. Schymanski, Dept. of

Data collector: E. Schymanski, Dept. of

Crocetin semialdehyde

0.739

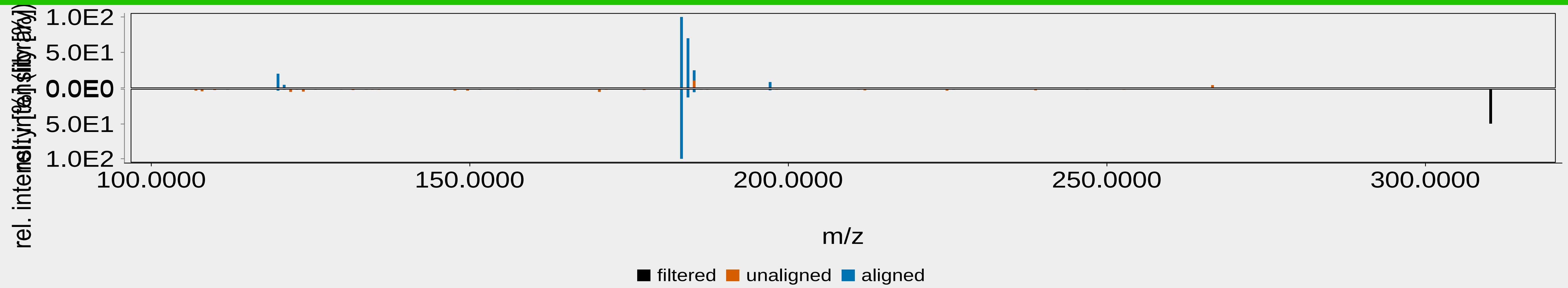

Compound information

Name: Crocetin semialdehyde

Ion type: carotenoid

Precursor m/z: 312.1725

Polarity: negative

Instrument information

Ion source: Electrospray Ionization (ESI)

Ms level: MS2

Database links

Other information

Canrenone 0.912

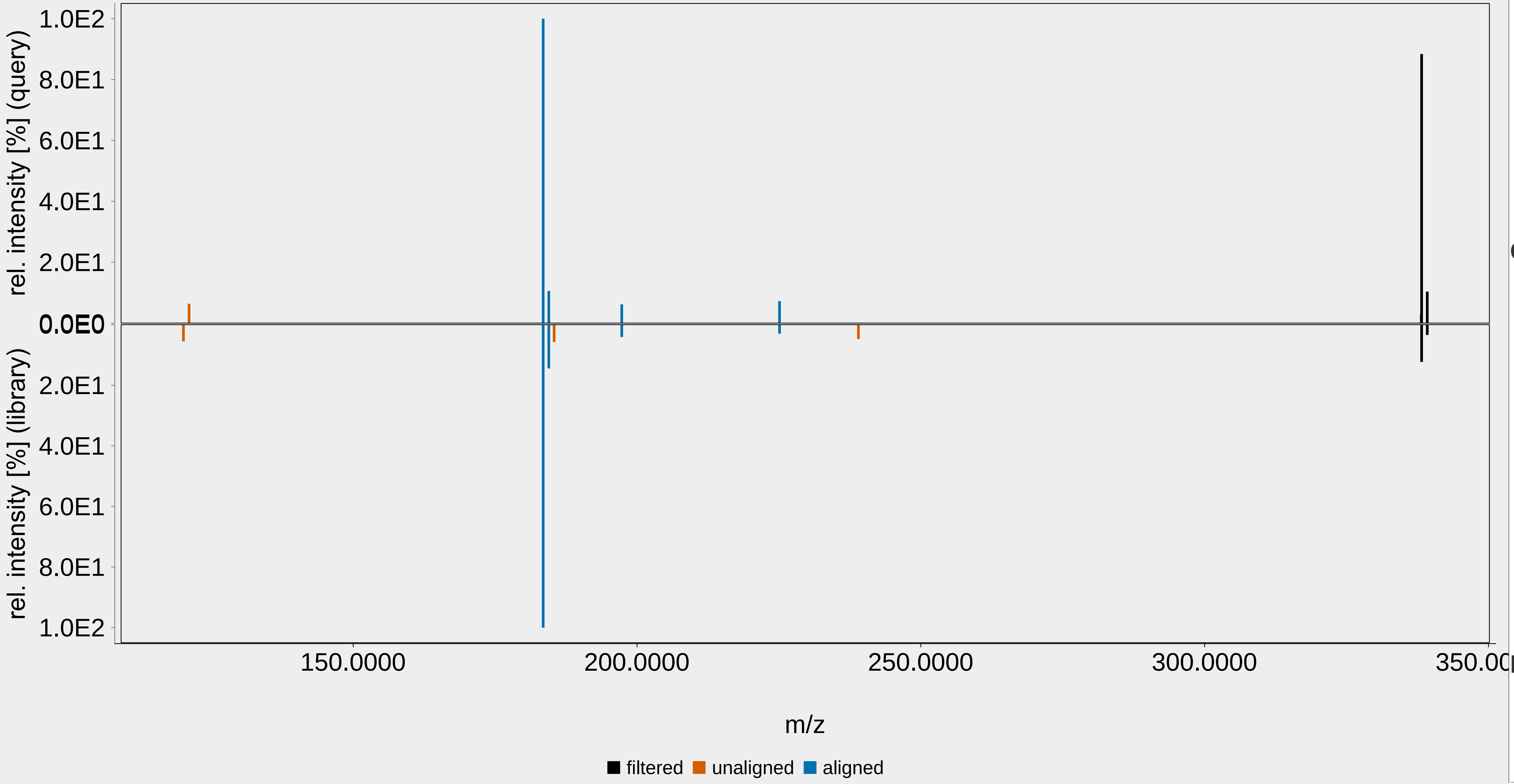

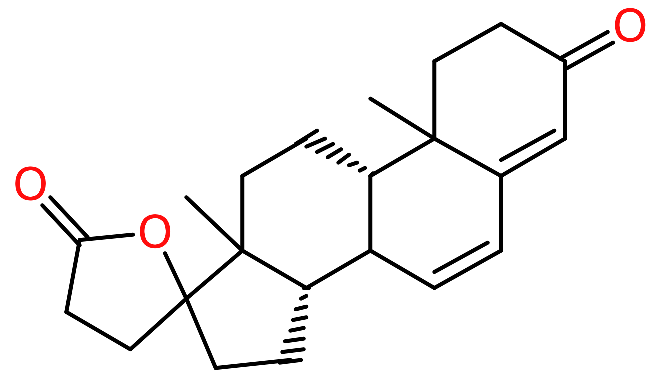

Compound information

Name: Canrenone

Formula: C22H28O3

Exact mass: 340.2038448

Ion type: [M-H]-

Precursor m/z: 339.1966

Polarity: NEGATIVE

InChI:

InChI key:

SMILES:

Num peaks: 9

Instrument information

Instrument type: LC-ESI-QTOF

Instrument: Bruker maXis Impact

Ms level: MS2

Collision energy: 40 eV

Database links

Other information

Entry ID: MSBNK-Athens_Univ-AU239460

Comment: Parent=339.1966

Canrenone, (9S,14S)-10,13-dimethylspiro[2,8,9,11,12,14,15,16-octahydro-1H-cyclopenta[a]phenanthrene-17,5`-oxolane]-2`,3-dione

0.912

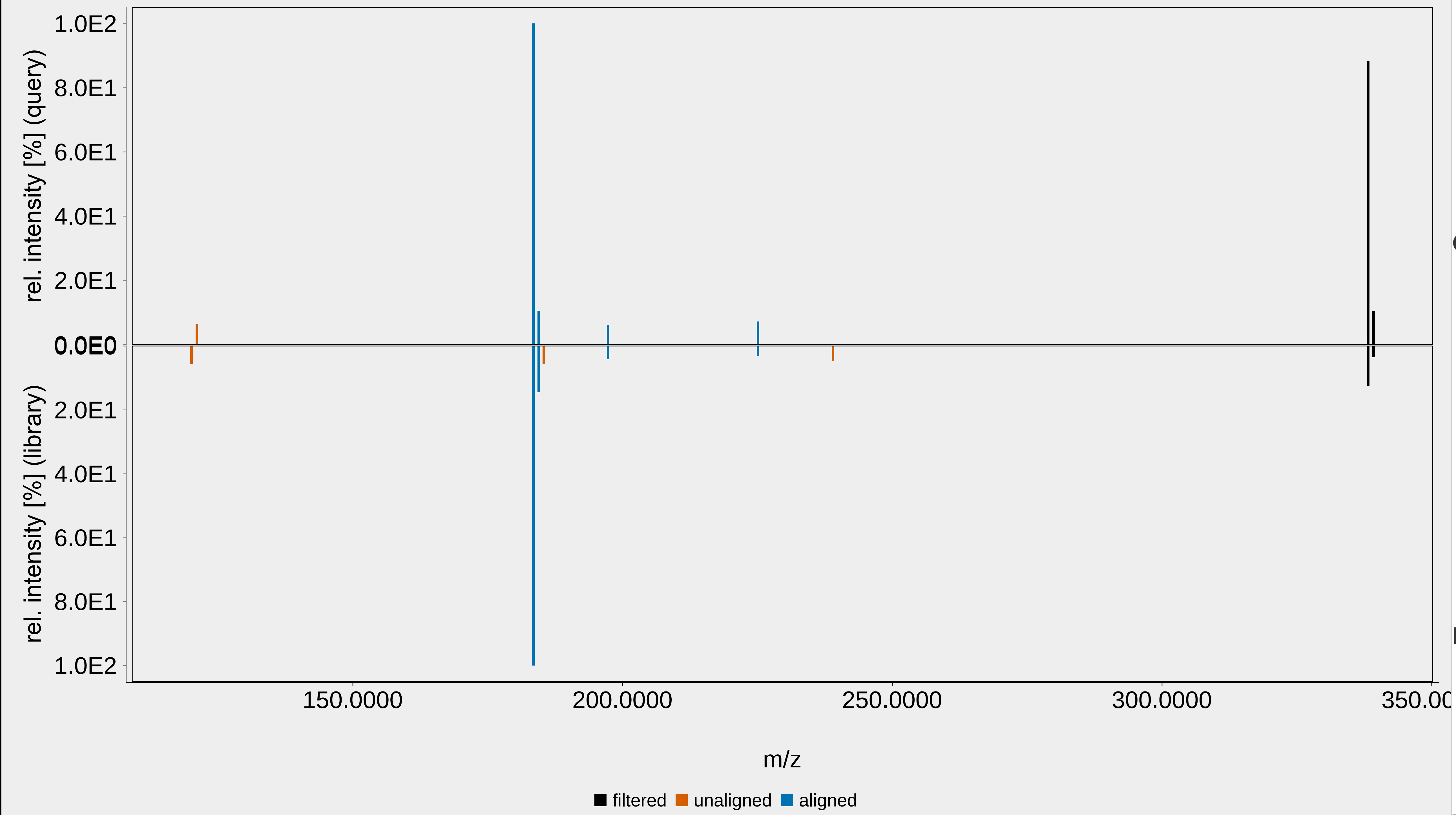

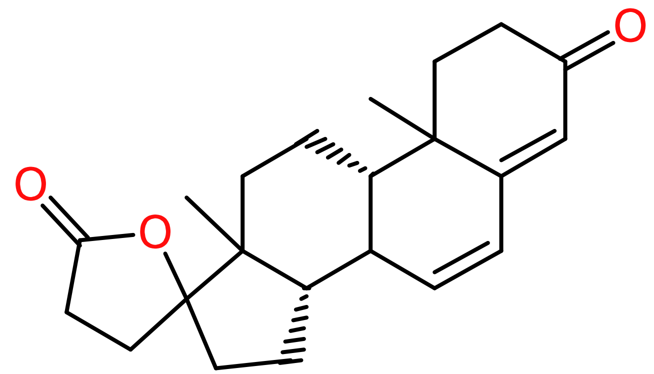

Compound information

Name: Canrenone,

Formula: C22H28O3

Mol. weight: 340.2038448

Exact mass: 340.2038448

Ion type: [M-H]-

Precursor m/z: 339.1966

Polarity: negative

InChI:

InChI key:

SMILES:

Instrument information

Instrument type: LC-ESI-QTOF

Instrument: Bruker maXis Impact

Ion source: ESI

Resolution: 35000

Ms level: MS2

Collision energy: 40 eV

Database links

Pubchem: 24728626

MoNA ID: AU239460

Chemspider: 21112020

CAS: 976-71-6

Other information

Principal investigator: Nikiforos Alygizakis,

Data collector: Nikiforos Alygizakis, Katerina

C10-LAS (TENTATIVE)

0.857

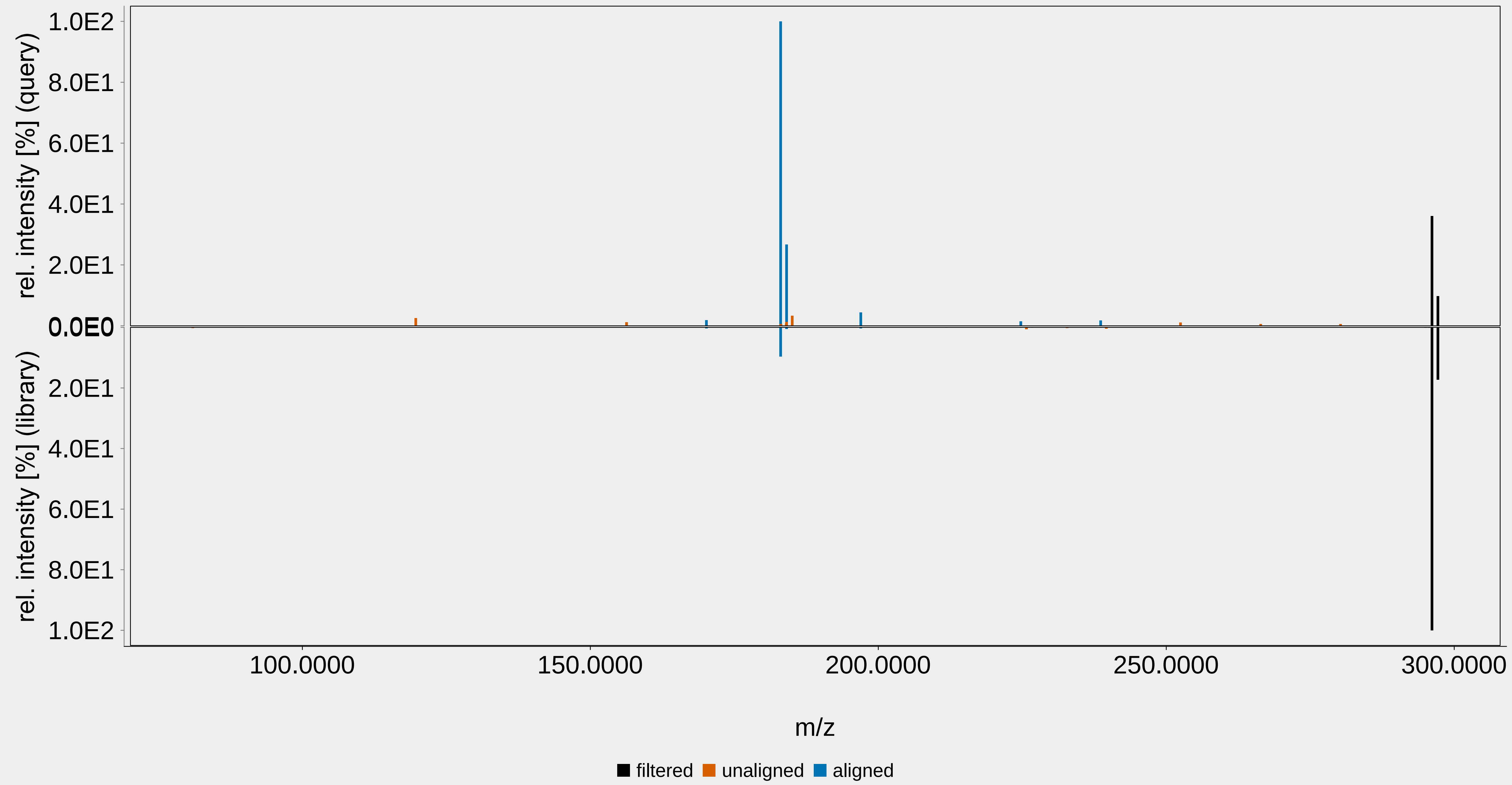

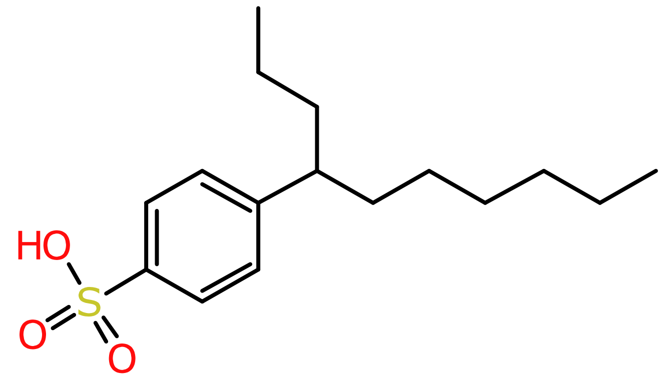

Compound information

Name: C10-LAS (TENTATIVE)

Formula: C16H26O3S

Exact mass: 298.1603

Ion type: [M-H]-

Precursor m/z: 297.1521

Polarity: NEGATIVE

InChI:

InChI key: NANHIUZYPFDGJS-UHFFFAOYSA-

SMILES:

Num peaks: 20

Instrument information

Instrument type: LC-ESI-ITFT

Instrument: LTQ Orbitrap XL Thermo Scientific

Ms level: MS2

Collision energy: 60 NCE

Database links

Other information

Entry ID:

Comment: Parent=297.1521

C10-LAS (TENTATIVE), C10-linear alkylbenzyl sulfonate, 4-(decan-4-yl)benzenesulfonic acid

0.858

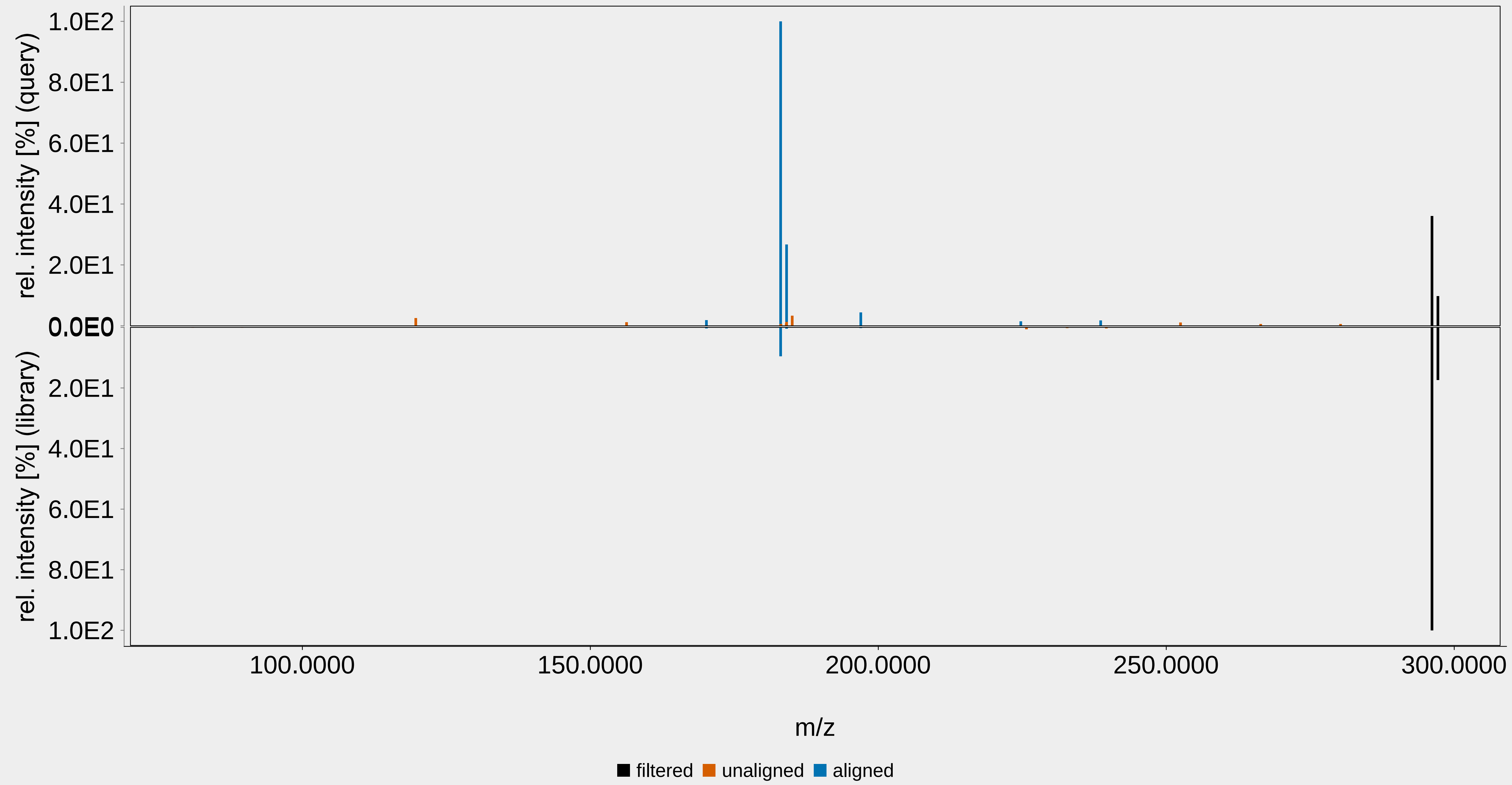

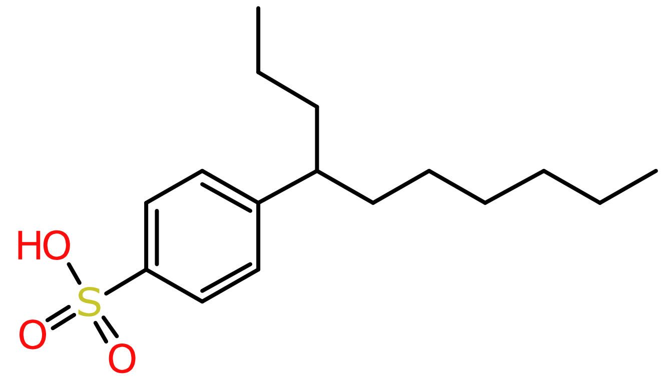

Compound information

Name: C10-LAS (TENTATIVE), C10-linear

Formula: C16H26O3S

Mol. weight: 298.1603

Exact mass: 298.1603

Ion type: [M-H]-

Precursor m/z: 297.1521

Polarity: negative

InChI:

InChI key: NANHIUZYPFDGJS-UHFFFAOYSA-

SMILES:

Instrument information

Instrument type: LC-ESI-ITFT

Instrument: LTQ Orbitrap XL Thermo Scientific

Ion source: ESI

Ms level: MS2

Collision energy: 60 NCE

Database links

Pubchem: 14921167

MoNA ID: ETS00001

Other information

Principal investigator: E. Schymanski, Dept. of

Data collector: E. Schymanski, Dept. of

C11-LAS (STANDARD MIX), C11-linear alkylbenzyl sulfonate, 4-(undecan-5-yl)benzenesulfonic acid

0.862

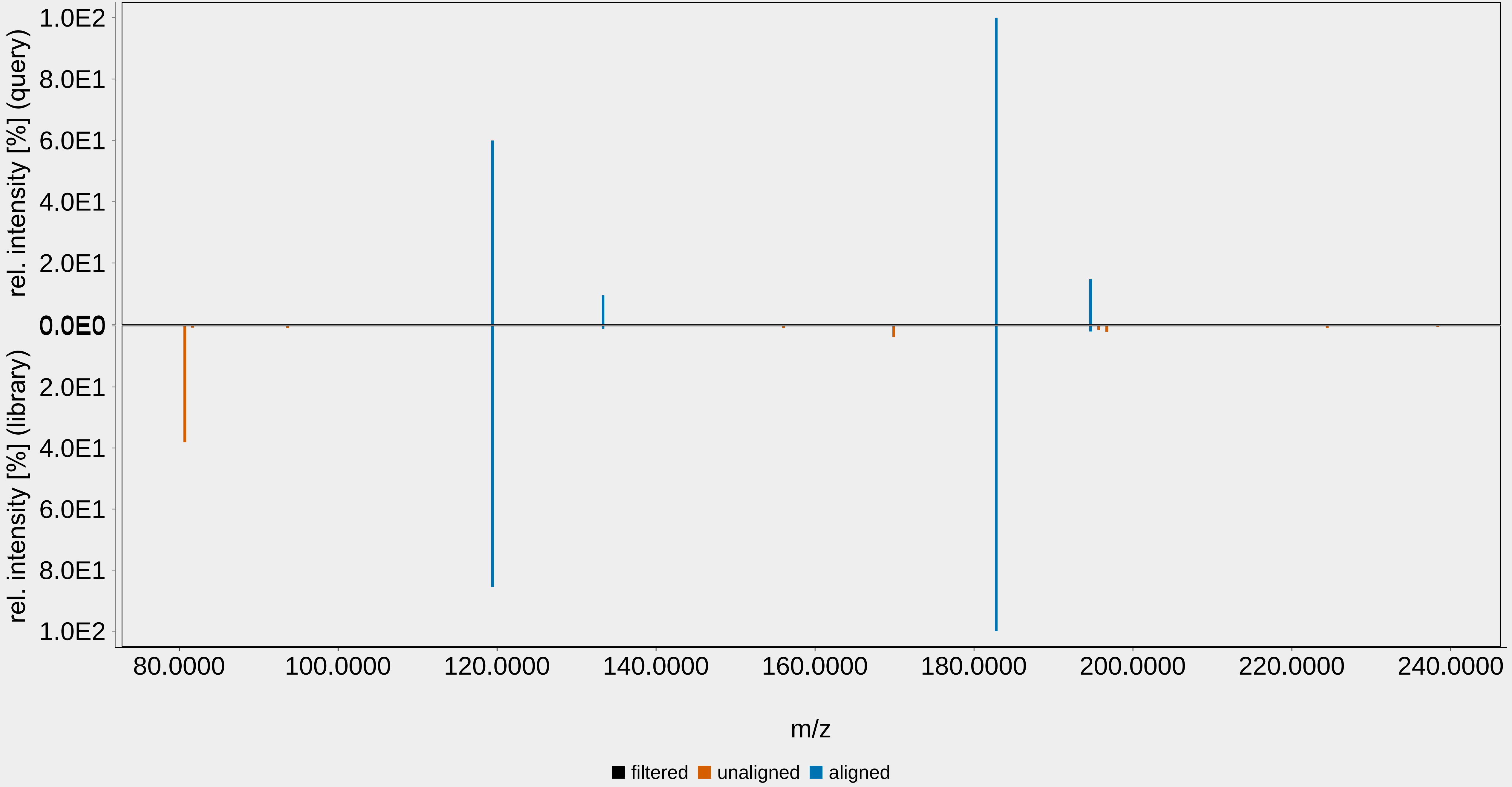

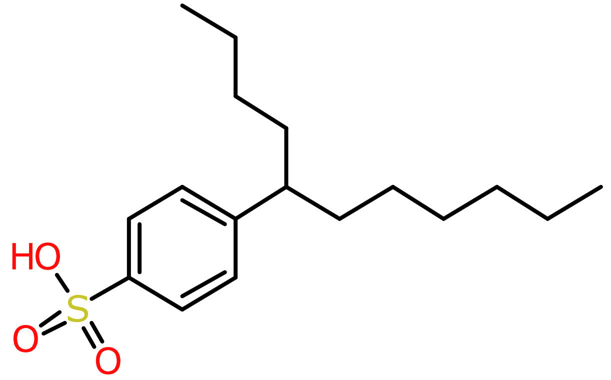

Compound information

Name: C11-LAS (STANDARD MIX), C11-linear

Formula: C17H28O3S

Mol. weight: 312.1759

Exact mass: 312.1759

Ion type: [M-H]-

Precursor m/z: 311.1686

Polarity: negative

InChI:

InChI key:

SMILES:

Instrument information

Instrument type: LC-ESI-QFT

Instrument: Q Exactive Orbitrap Thermo

Ion source: ESI

Ms level: MS2

Collision energy: 90 NCE

Database links

Pubchem: 15593874

MoNA ID: ETS00014

Other information

Principal investigator: E. Schymanski, Dept. of

Data collector: E. Schymanski, Dept. of

C11-LAS (STANDARD MIX)

0.862

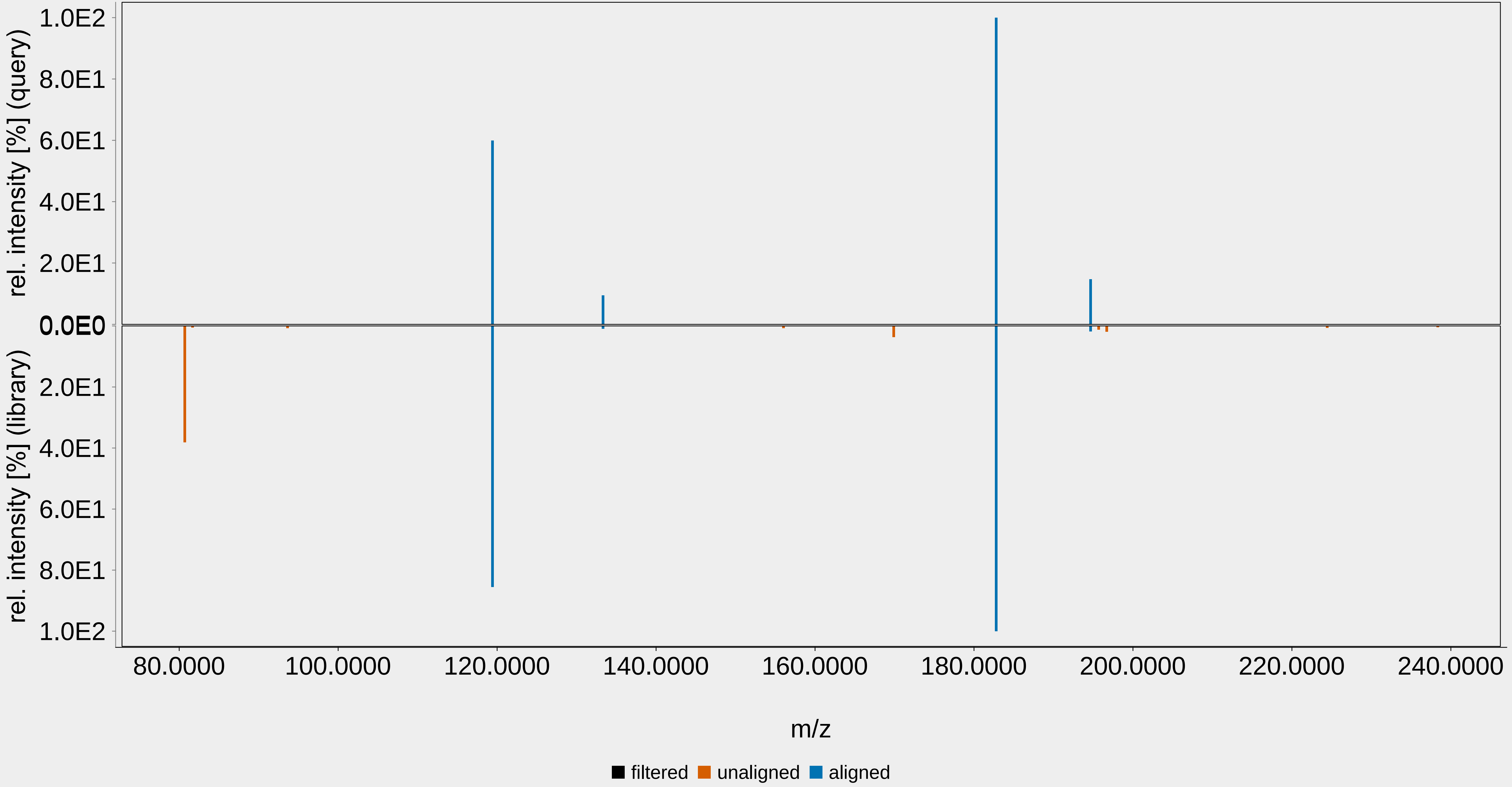

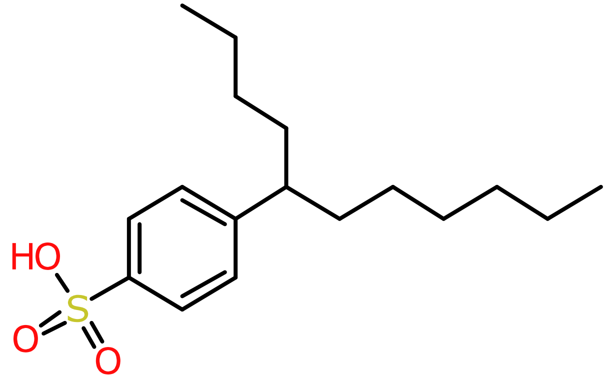

Compound information

Name: C11-LAS (STANDARD MIX)

Formula: C17H28O3S

Exact mass: 312.1759

Ion type: [M-H]-

Precursor m/z: 311.1686

Polarity: NEGATIVE

InChI:

InChI key:

SMILES:

Num peaks: 13

Instrument information

Instrument type: LC-ESI-QFT

Instrument: Q Exactive Orbitrap Thermo

Ms level: MS2

Collision energy: 90 NCE

Database links

Other information

Entry ID:

Comment: Parent=311.1686

Dodecylbenzenesulfonic acid

0.901

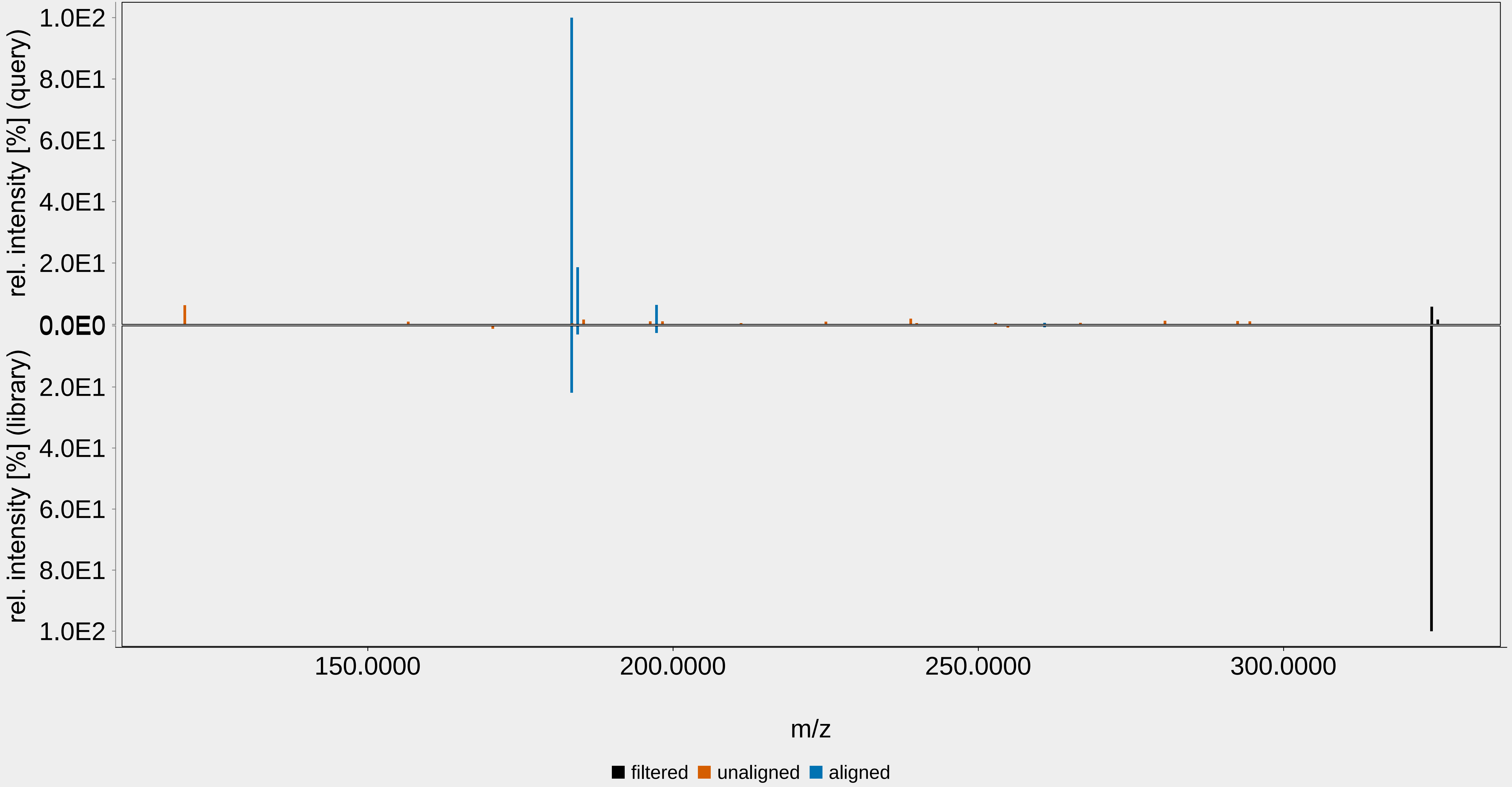

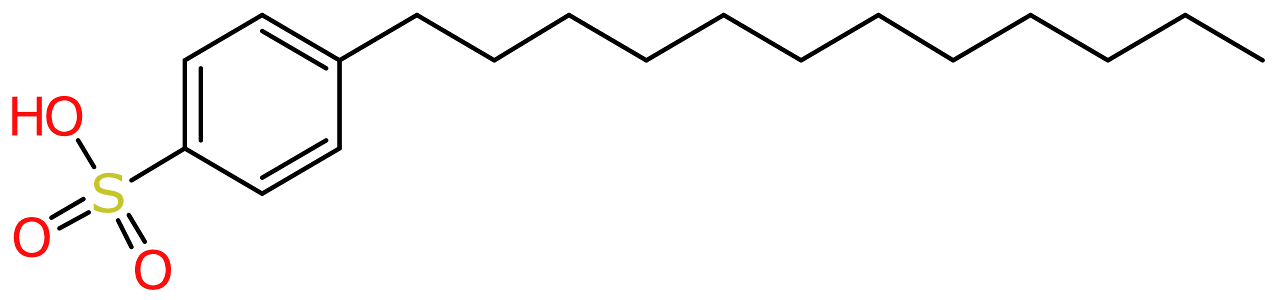

Compound information

Name: Dodecylbenzenesulfonic acid

Formula: C18H30O3S

Exact mass: 326.19157

Ion type: [M-H]-

Precursor m/z: 325.1843

Polarity: NEGATIVE

InChI:

InChI key:

SMILES:

Num peaks: 7

Instrument information

Instrument type: LC-ESI-QFT

Instrument: Q Exactive Plus Orbitrap Thermo

Ms level: MS2

Collision energy: 35 (nominal)

Database links

Other information

Entry ID: MSBNK-CASMI_2016-SM876251

Comment: Parent=325.1843

Dodecylbenzenesulfonic acid

0.900

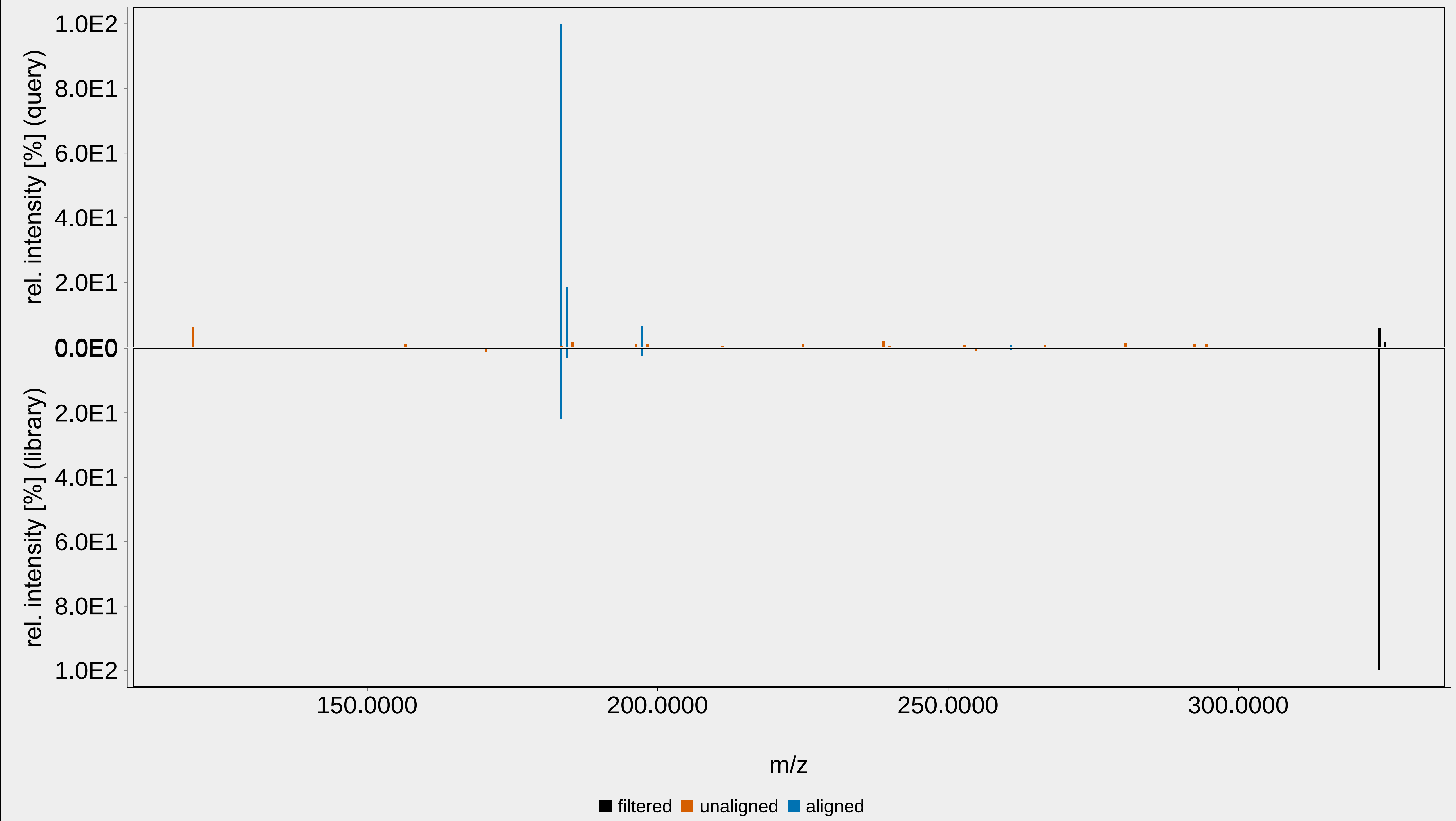

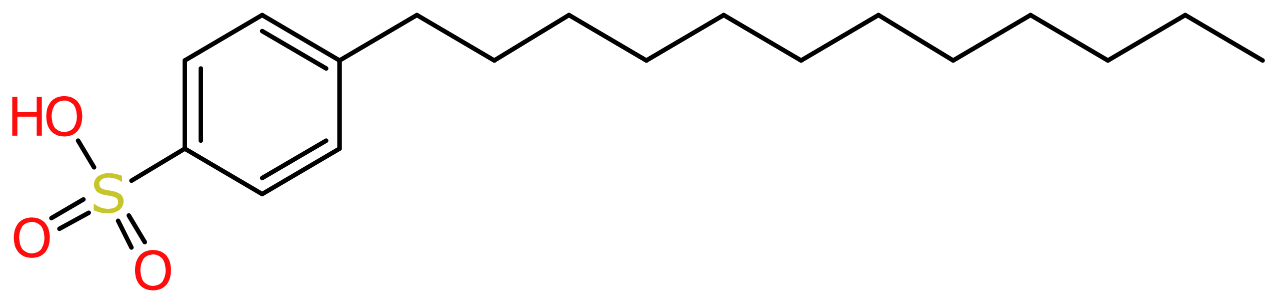

Compound information

Name: Dodecylbenzenesulfonic acid

Formula: C18H30O3S

Mol. weight: 326.19157

Exact mass: 326.19157

Ion type: [M-H]-

Precursor m/z: 325.1843

Polarity: negative

InChI:

InChI key:

SMILES:

Instrument information

Instrument type: LC-ESI-QFT

Instrument: Q Exactive Plus Orbitrap Thermo

Ion source: ESI

Resolution: 35000

Ms level: MS2

Collision energy: 35 (nominal)

Database links

Pubchem: 8485

MoNA ID: SM876251

Chemspider: 8172

CAS: 121-65-3

Other information

Principal investigator: Krauss M, Schymanski

Data collector: Krauss M, Schymanski EL,

Glyceryl phosphate - 30.0 eV

0.985

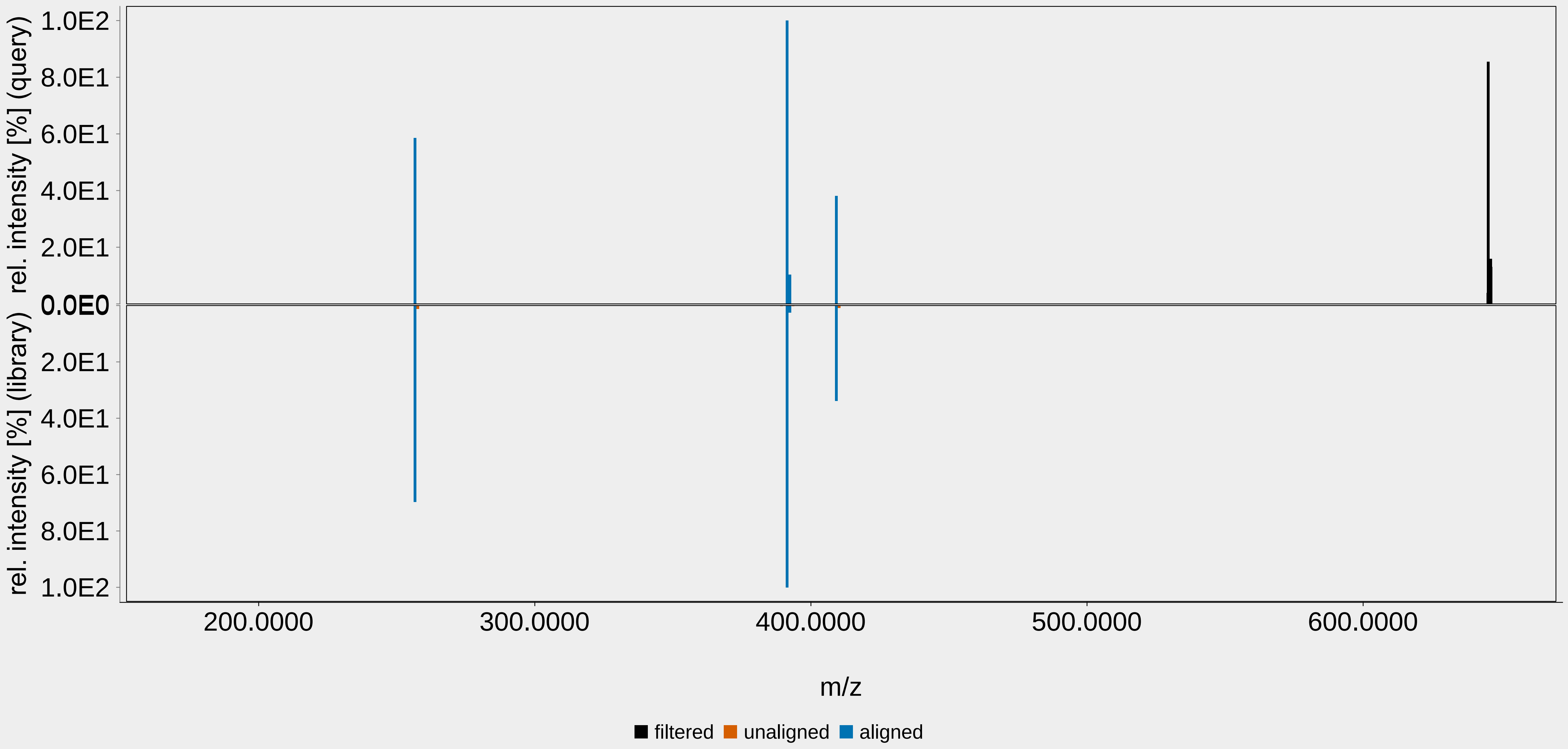

Compound information

Name: Glyceryl phosphate - 30.0 eV

Exact mass: 0.0

Ion type: Unknown

Precursor m/z: 647.466

Charge: 1

Polarity: Negative

SMILES: C([C@H](COP(=O)(O)O)O)O

Instrument information

Instrument type: Orbitrap

Ion source: ESI

Ms level: 2

Acquisition: Isolated

Database links

CAS:

Other information

Principal investigator: PI

Data collector: Data Collector

Entry ID: CCMSLIB00006126789

Glyceryl phosphate - 30.0 eV

0.986

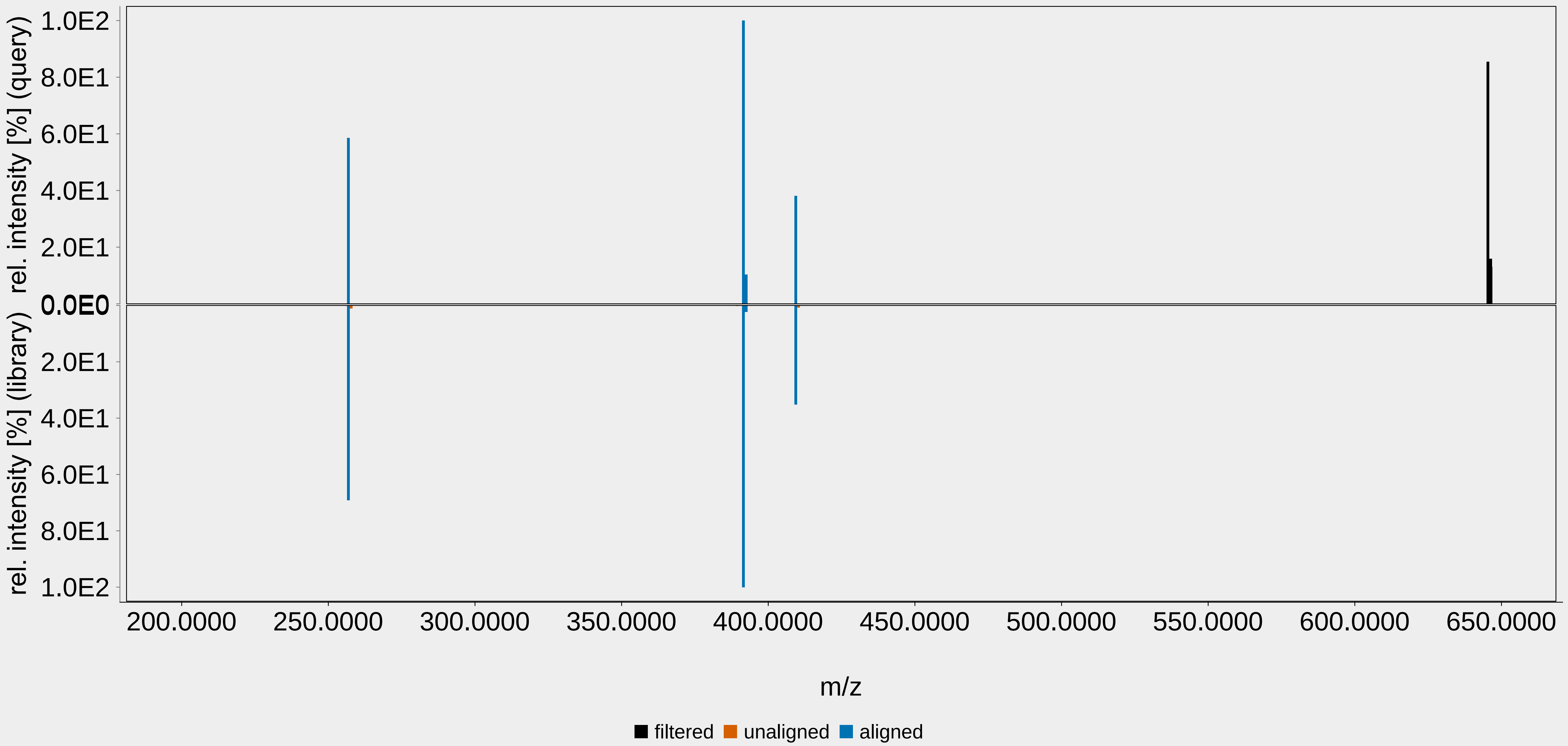

Compound information

Name: Glyceryl phosphate - 30.0 eV

Exact mass: 0.0

Ion type: Unknown

Precursor m/z: 647.466

Charge: 1

Polarity: Negative

SMILES: C([C@H](COP(=O)(O)O)O)O

Instrument information

Instrument type: Orbitrap

Ion source: ESI

Ms level: 2

Acquisition: Isolated

Database links

CAS:

Other information

Principal investigator: PI

Data collector: Data Collector

Entry ID: CCMSLIB00006126790

Dodecylbenzenesulfonic acid

0.939

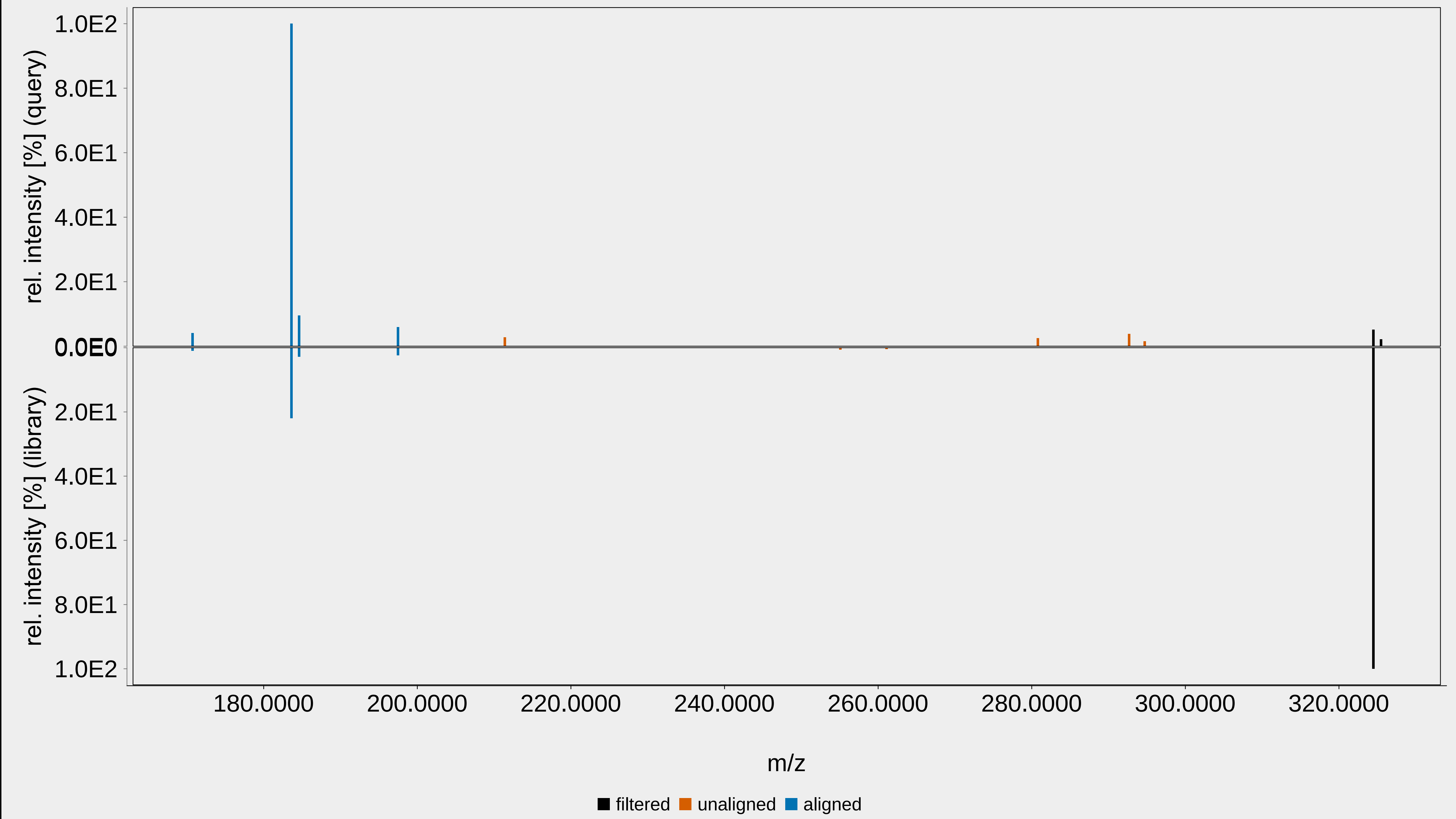

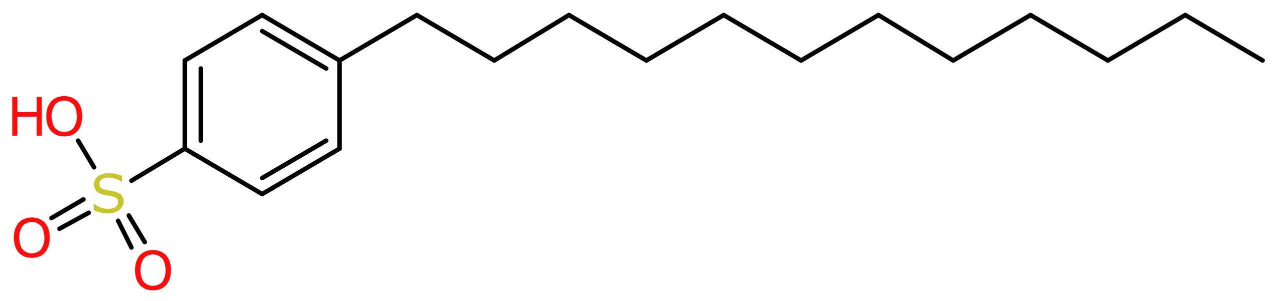

Compound information

Name: Dodecylbenzenesulfonic acid

Formula: C18H30O3S

Mol. weight: 326.19157

Exact mass: 326.19157

Ion type: [M-H]-

Precursor m/z: 325.1843

Polarity: negative

InChI:

InChI key:

SMILES:

Instrument information

Instrument type: LC-ESI-QFT

Instrument: Q Exactive Plus Orbitrap Thermo

Ion source: ESI

Resolution: 35000

Ms level: MS2

Collision energy: 35 (nominal)

Database links

Pubchem: 8485

MoNA ID: SM876251

Chemspider: 8172

CAS: 121-65-3

Other information

Principal investigator: Krauss M, Schymanski

Data collector: Krauss M, Schymanski EL,

Dodecylbenzenesulfonic acid

0.940

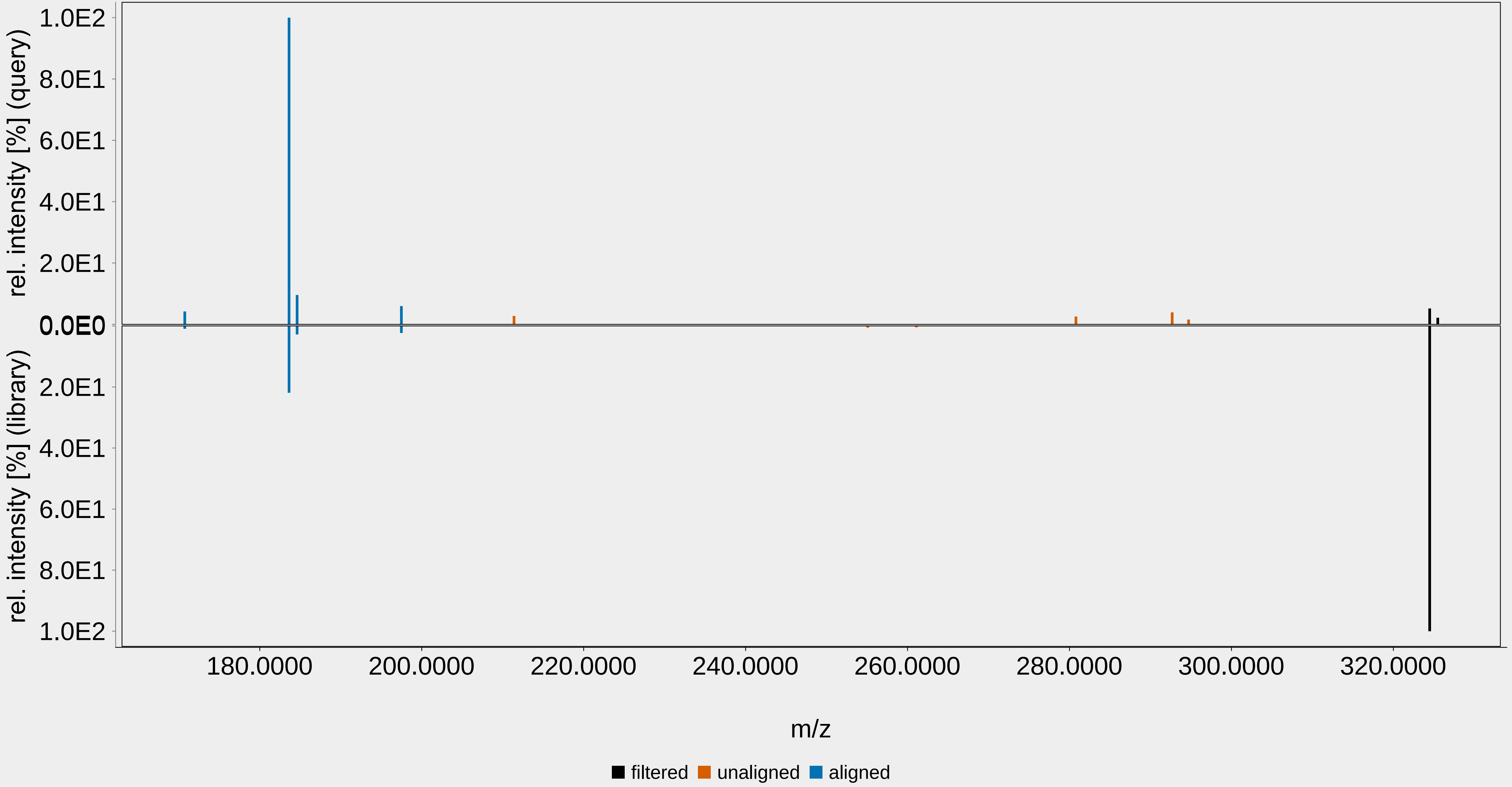

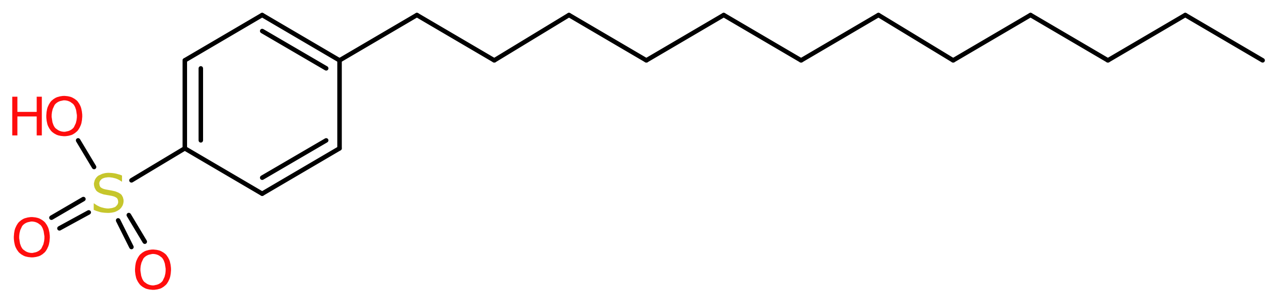

Compound information

Name: Dodecylbenzenesulfonic acid

Formula: C18H30O3S

Exact mass: 326.19157

Ion type: [M-H]-

Precursor m/z: 325.1843

Polarity: NEGATIVE

InChI:

InChI key:

SMILES:

Num peaks: 7

Instrument information

Instrument type: LC-ESI-QFT

Instrument: Q Exactive Plus Orbitrap Thermo

Ms level: MS2

Collision energy: 35 (nominal)

Database links

Other information

Entry ID: MSBNK-CASMI_2016-SM876251

Comment: Parent=325.1843

PS 16:0_22:6 **0.854**

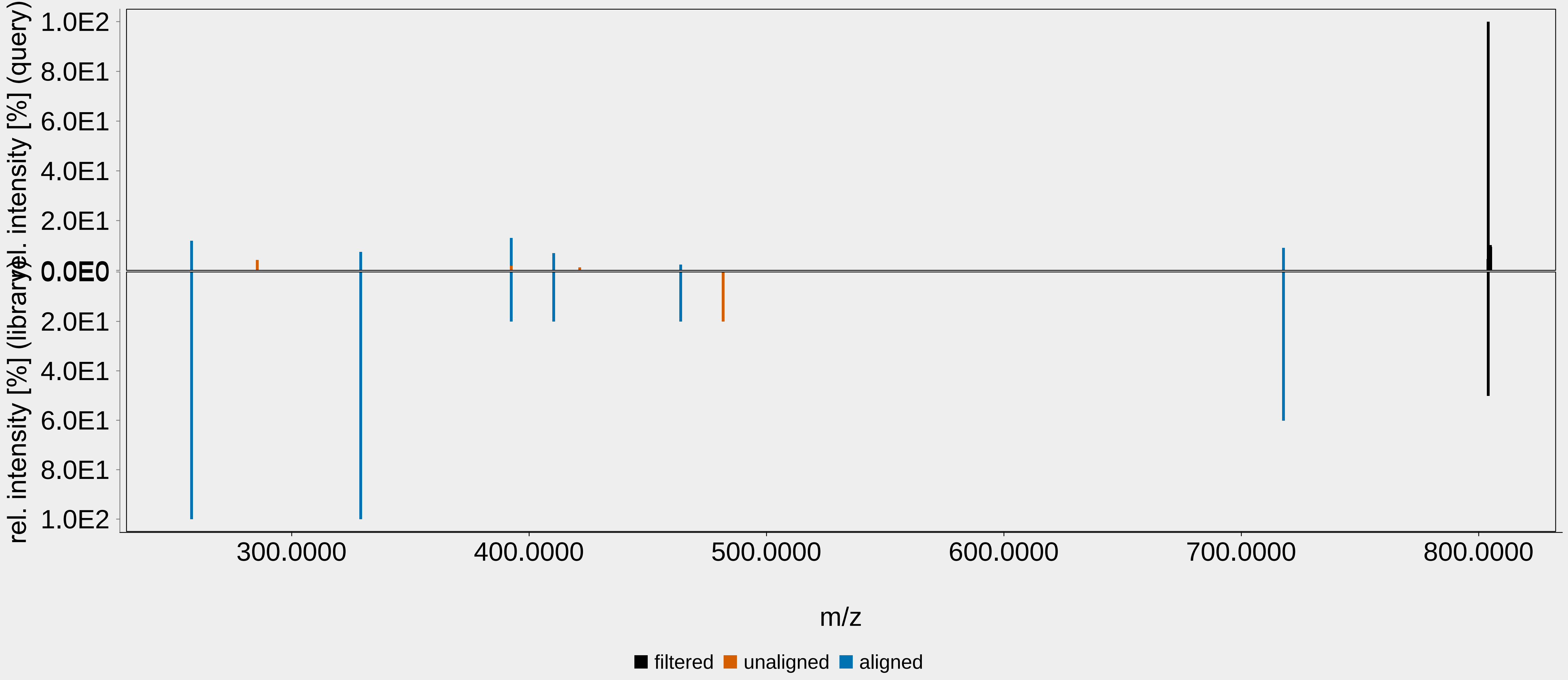

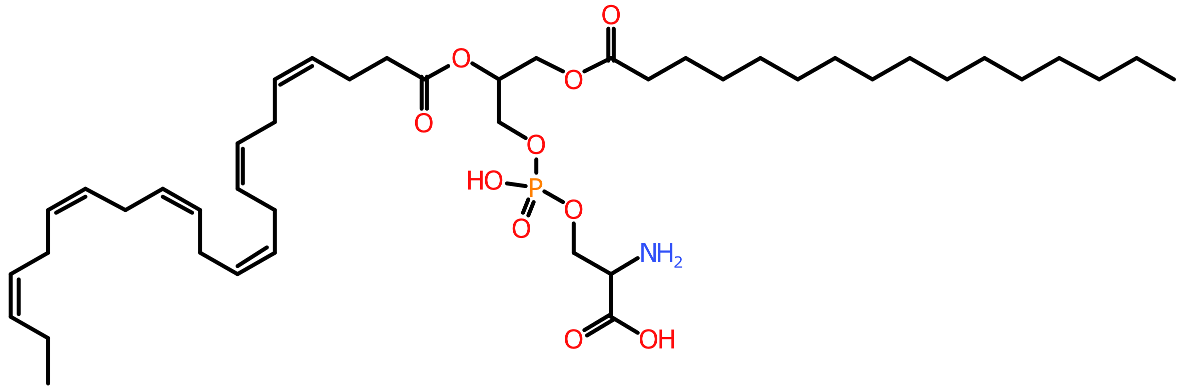

Compound information

Name: PS 16:0_22:6

Formula: C44H74NO10P

Ion type: [M-H]-

Precursor m/z: 806.49776

Ccs: 276.61

InChI key:

SMILES:

Num peaks: 8

Instrument information

Database links

Other information

PS 38:6 **0.850**

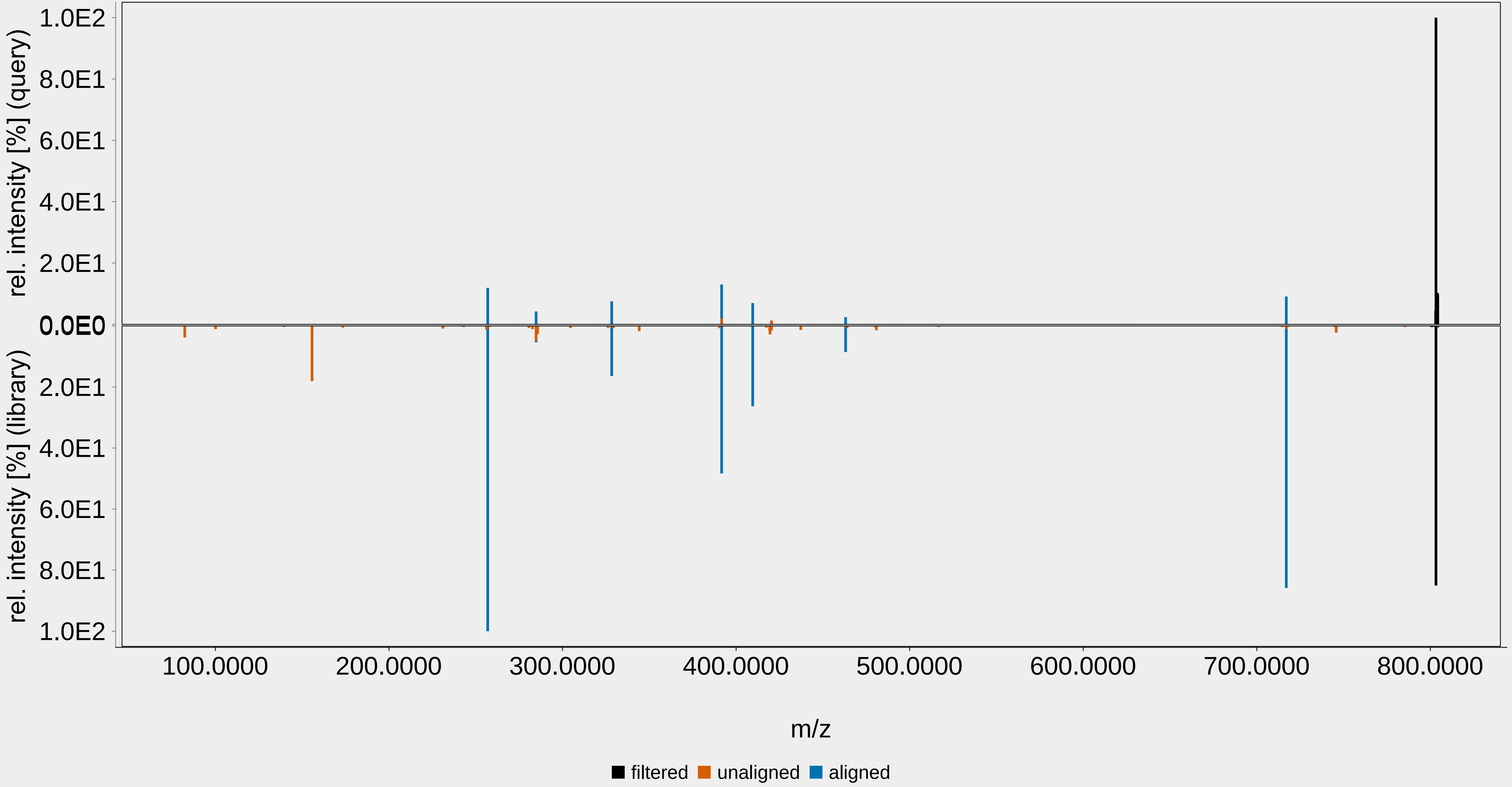

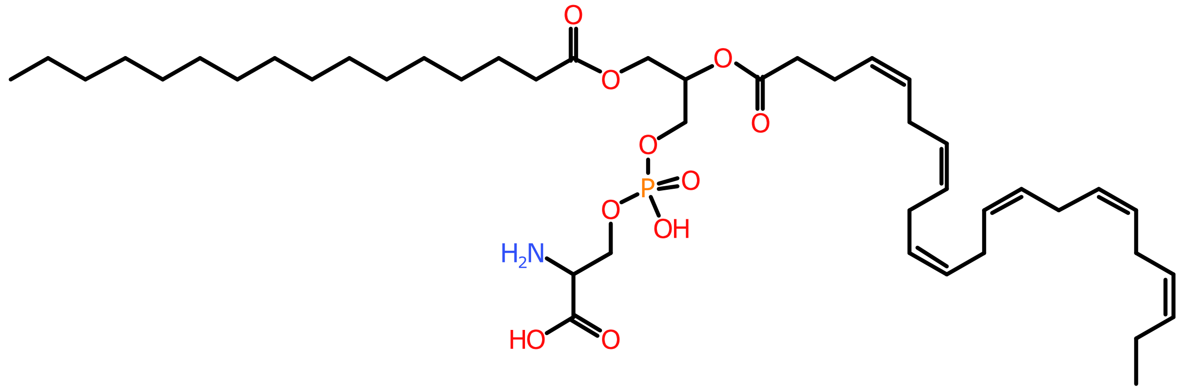

Compound information

Name: PS 38:6

Formula: C44H74NO10P

Exact mass: 808.0489999999999

Ion type: [M-H]-

Precursor m/z: 806.5011

Polarity: NEGATIVE

InChI:

InChI key:

SMILES:

Num peaks: 57

Instrument information

Instrument type: LC-ESI-QTOF

Instrument: AB Sciex TripleTOF 5600+ system

Ms level: MS2

Collision energy: 45V

Database links

Other information

Entry ID: MSBNK-RIKEN_IMS-LQB00330

Comment: Parent=806.5011

PE(16:0/16:0); [M-H]- C37H73N1O8P1 — 0.926

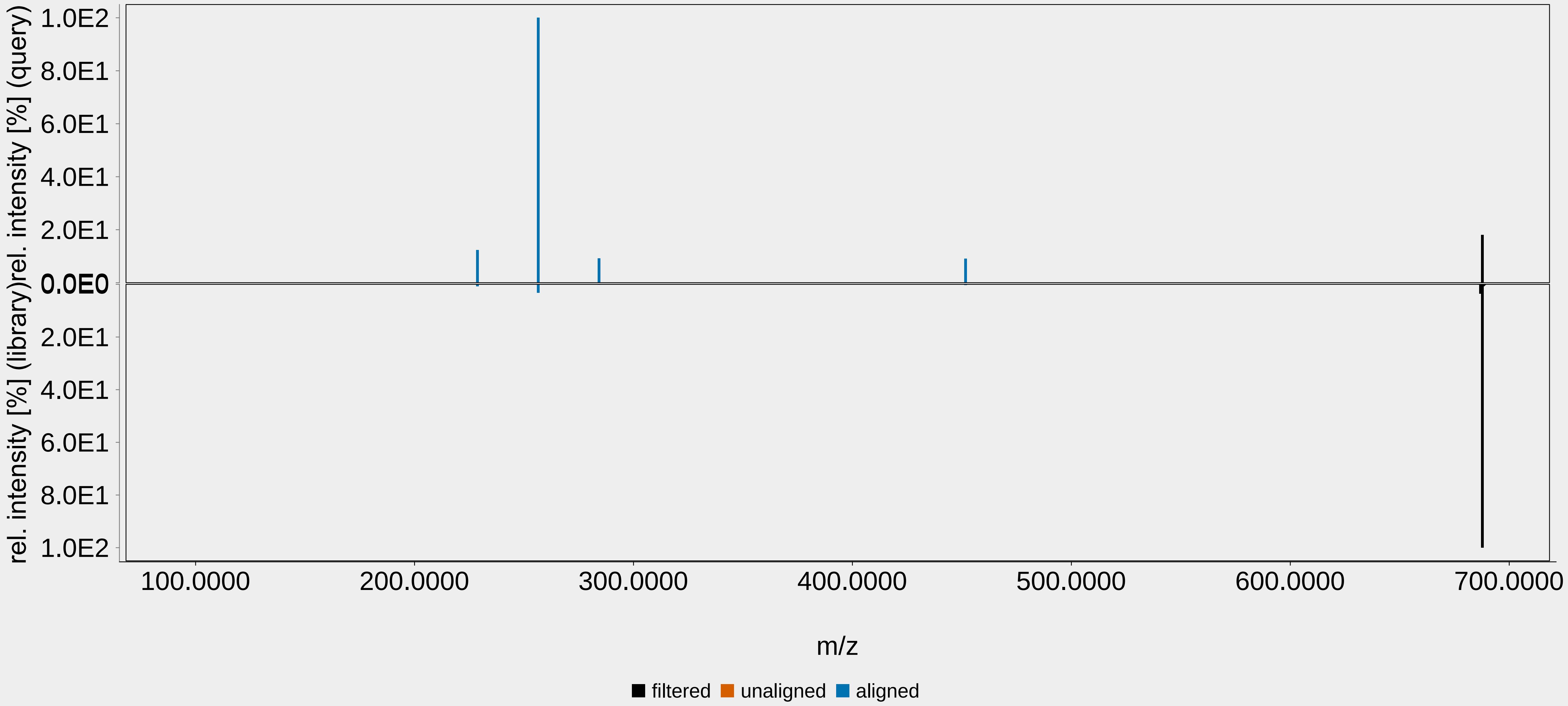

Compound information

Name: PE(16:0/16:0); [M-H]- C37H73N1O8P1

Exact mass: 0.0

Ion type: M-H

Precursor m/z: 690.507

Charge: 1

Polarity: Negative

Instrument information

Instrument type: HCD; Velos

Ion source: LC-ESI

Ms level: 2

Acquisition: Commercial

Database links

Other information

Principal investigator: Thomas Metz

Data collector: Thomas Metz

Entry ID: CCMSLIB00010069794

PE(16:0/16:0); [M-H]- C37H73N1O8P1

0.944

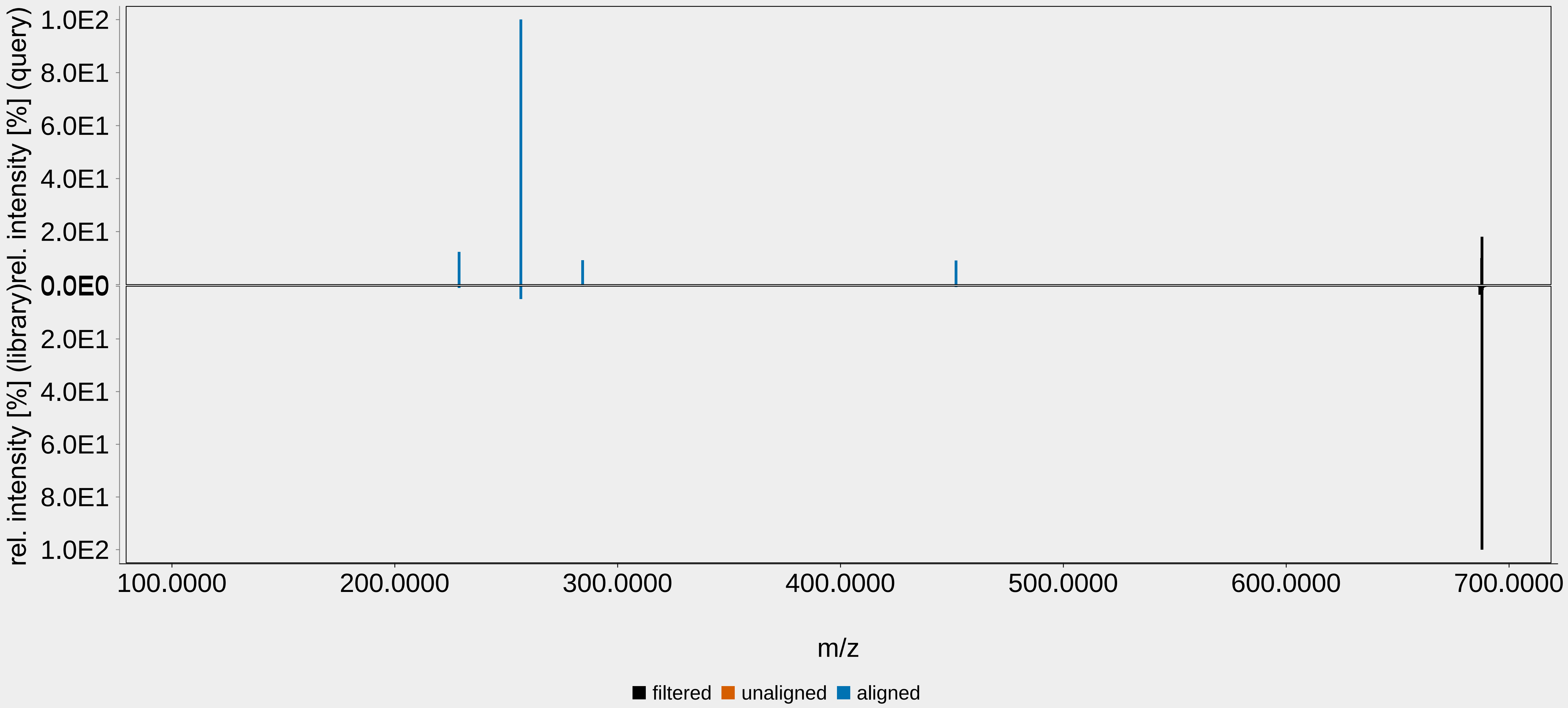

Compound information

Name: PE(16:0/16:0); [M-H]- C37H73N1O8P1

Exact mass: 0.0

Ion type: M-H

Precursor m/z: 690.507

Charge: 1

Polarity: Negative

Instrument information

Instrument type: HCD; Velos

Ion source: LC-ESI

Ms level: 2

Acquisition: Commercial

Database links

Other information

Principal investigator: Thomas Metz

Data collector: Thomas Metz

Entry ID: CCMSLIB00010068325

PA(18:2/18:2); [M-H]- C39H68O8P1 **0.721**

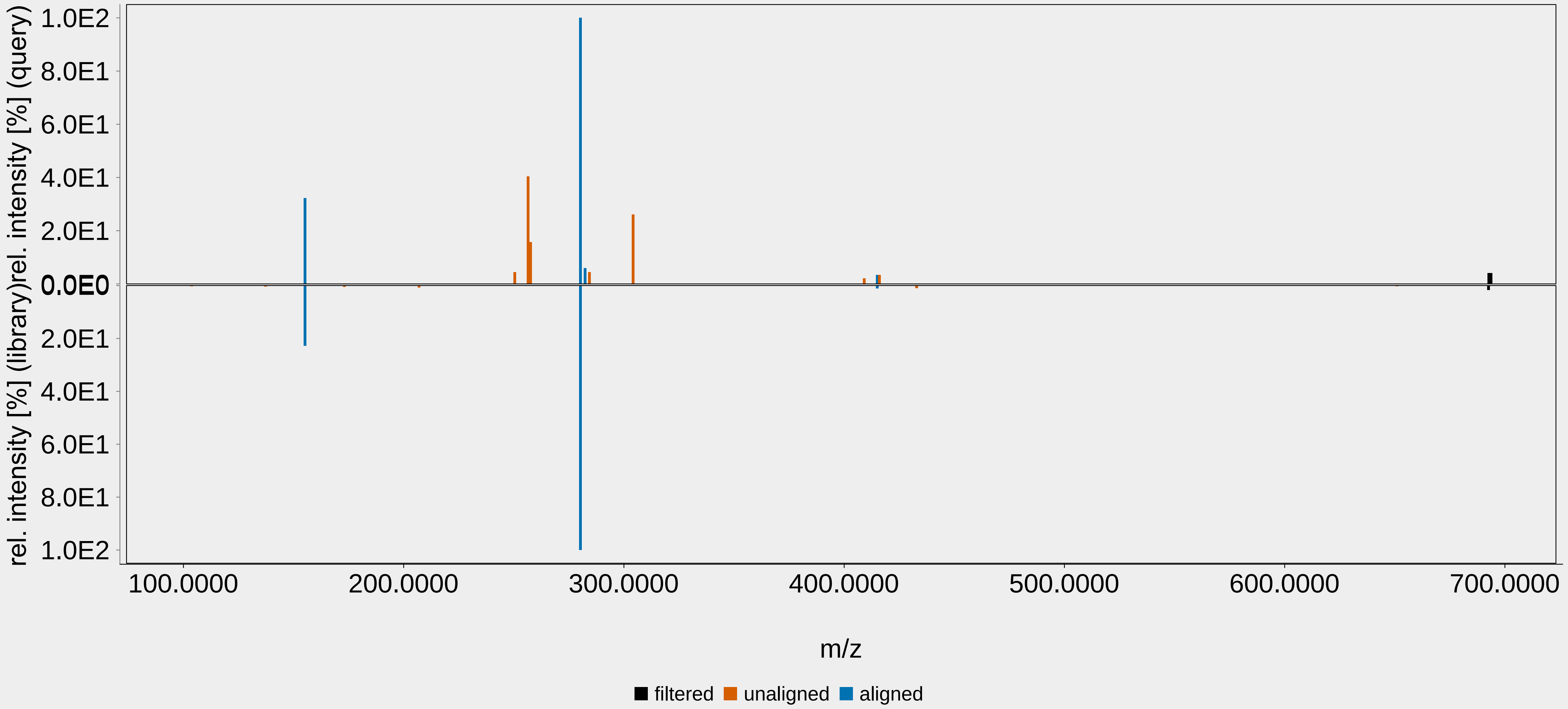

Compound information

Name: PA(18:2/18:2); [M-H]- C39H68O8P1

Exact mass: 0.0

Ion type: M-H

Precursor m/z: 695.465

Charge: 1

Polarity: Negative

Instrument information

Instrument type: HCD; Lumos

Ion source: LC-ESI

Ms level: 2

Acquisition: Commercial

Database links

Other information

Principal investigator: Thomas Metz

Data collector: Thomas Metz

Entry ID: CCMSLIB00010059139

PA 16:0_20:4

0.903

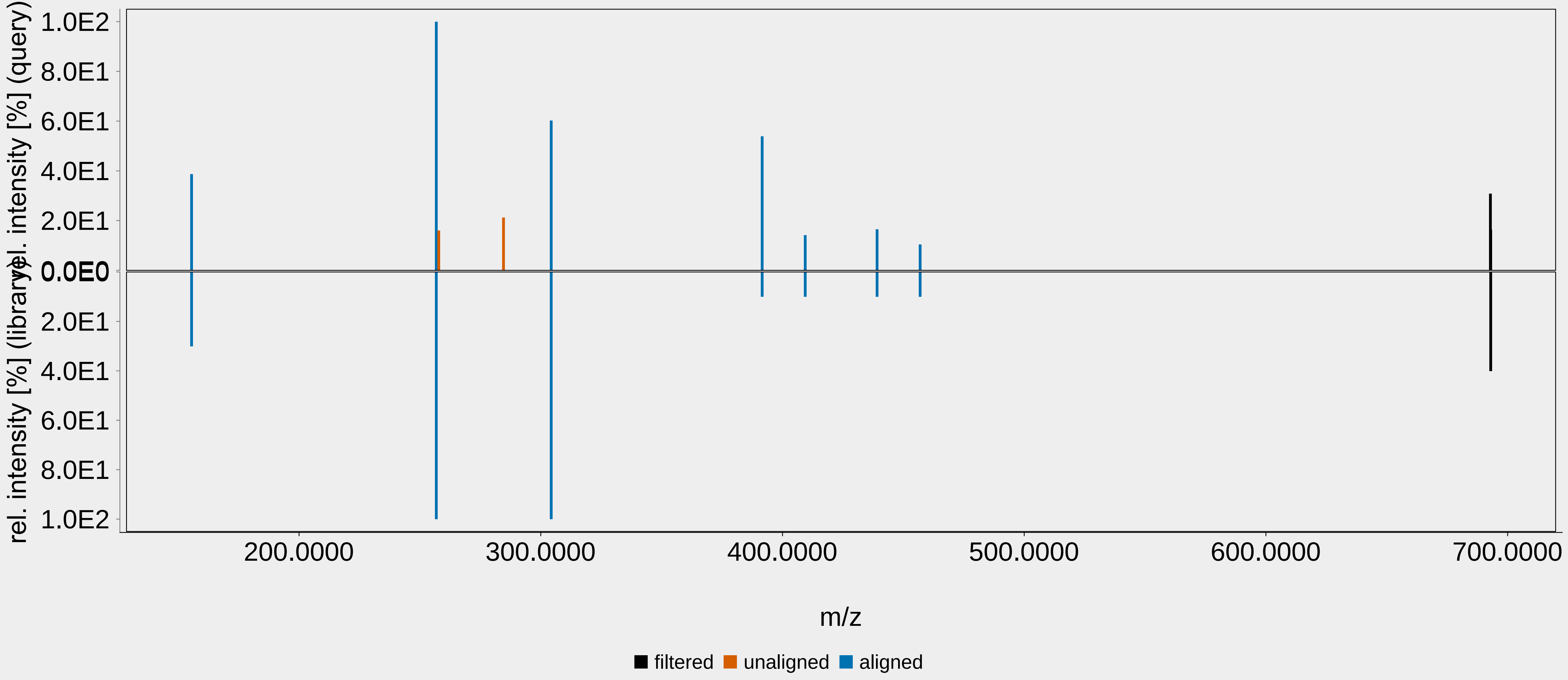

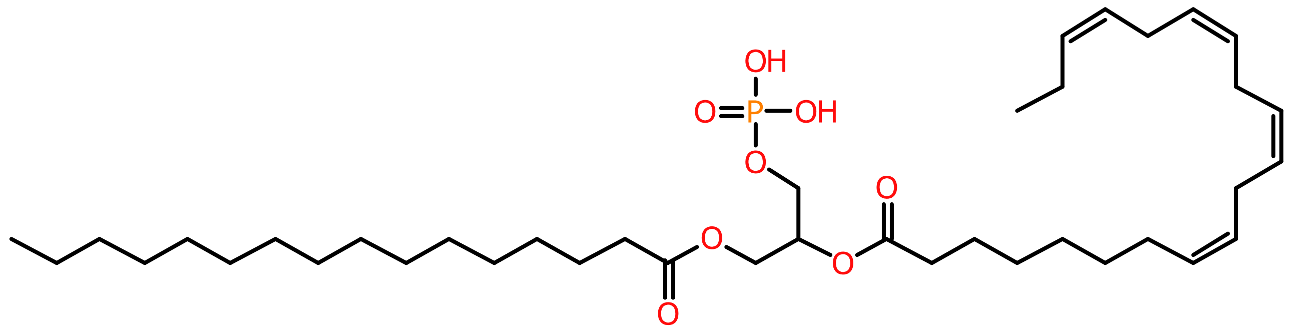

Compound information

Name: PA 16:0_20:4

Formula: C39H69O8P

Ion type: [M-H]-

Precursor m/z: 695.46573

Ccs: 255.1

InChI key:

SMILES:

Num peaks: 8

Instrument information

Database links

Other information

PS(18:1/22:6); [M-H]- C46H75N1O10P1 0.815

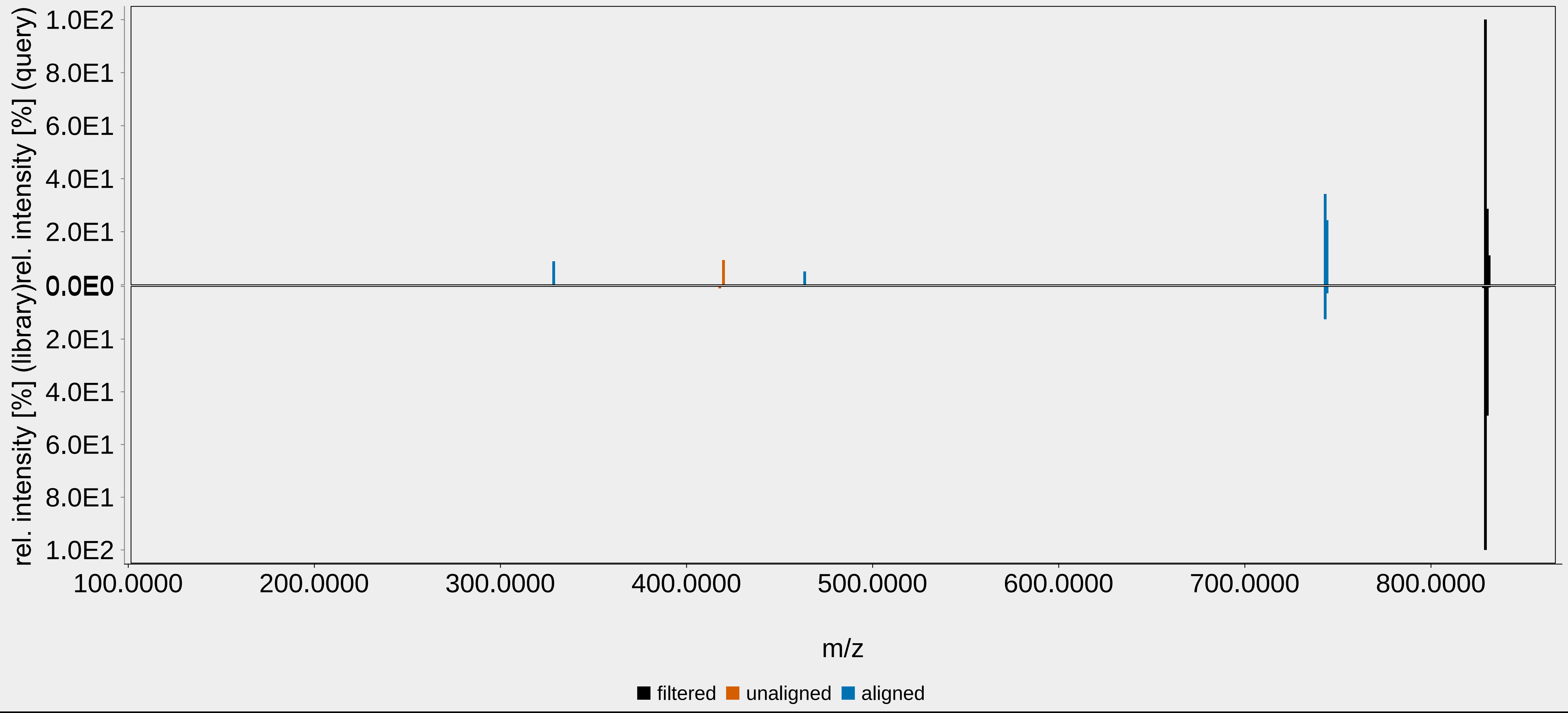

Compound information

Name: PS(18:1/22:6); [M-H]- C46H75N1O10P1

Exact mass: 0.0

Ion type: M-H

Precursor m/z: 832.513

Charge: 1

Polarity: Negative

Instrument information

Instrument type: HCD; Velos

Ion source: LC-ESI

Ms level: 2

Acquisition: Commercial

Database links

Other information

Principal investigator: Thomas Metz

Data collector: Thomas Metz

Entry ID: CCMSLIB00010083738

PS(18:1/22:6); [M-H]- C46H75N1O10P1

0.800

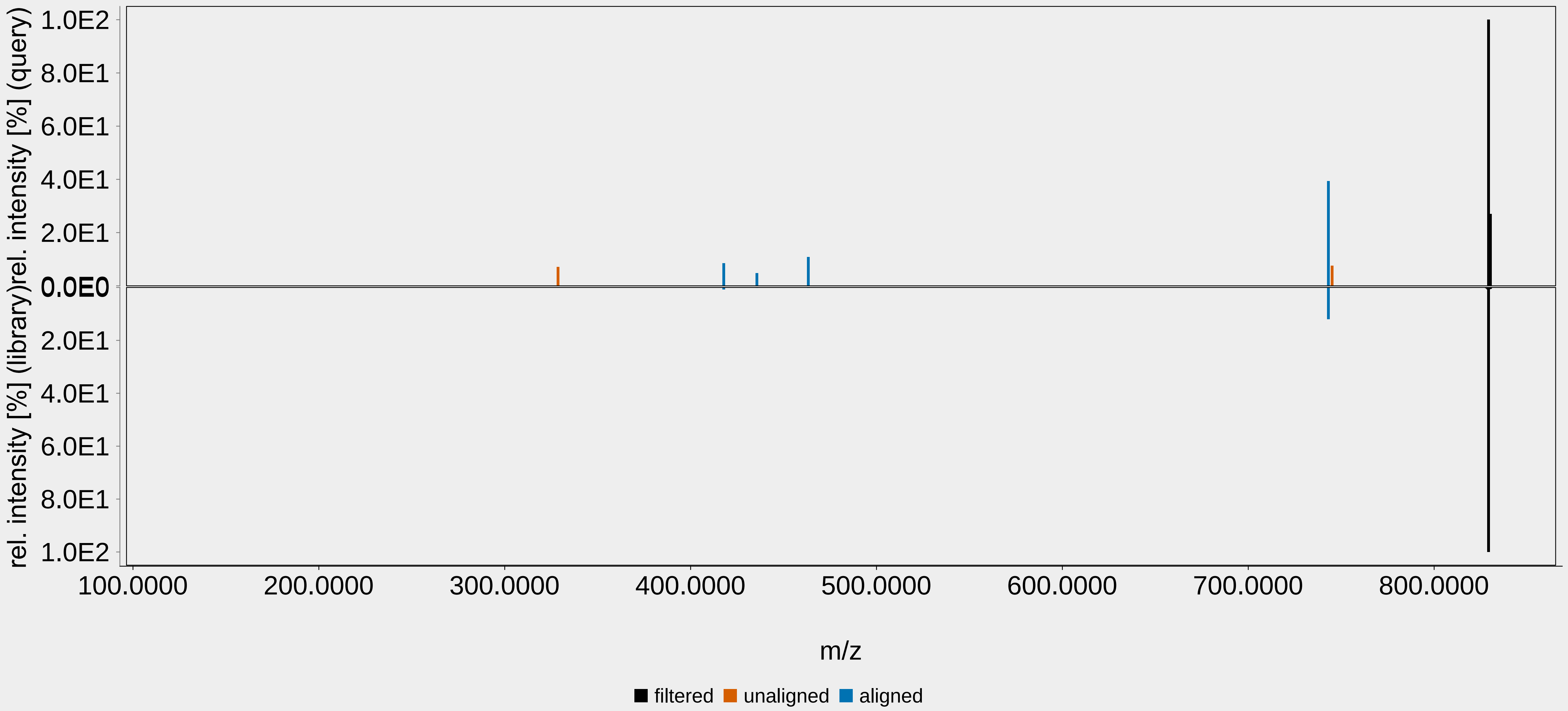

Compound information

Name: PS(18:1/22:6); [M-H]- C46H75N1O10P1

Exact mass: 0.0

Ion type: M-H

Precursor m/z: 832.513

Charge: 1

Polarity: Negative

Instrument information

Instrument type: HCD; Velos

Ion source: LC-ESI

Ms level: 2

Acquisition: Commercial

Database links

Other information

Principal investigator: Thomas Metz

Data collector: Thomas Metz

Entry ID: CCMSLIB00010063314

PS 40:6 0.892

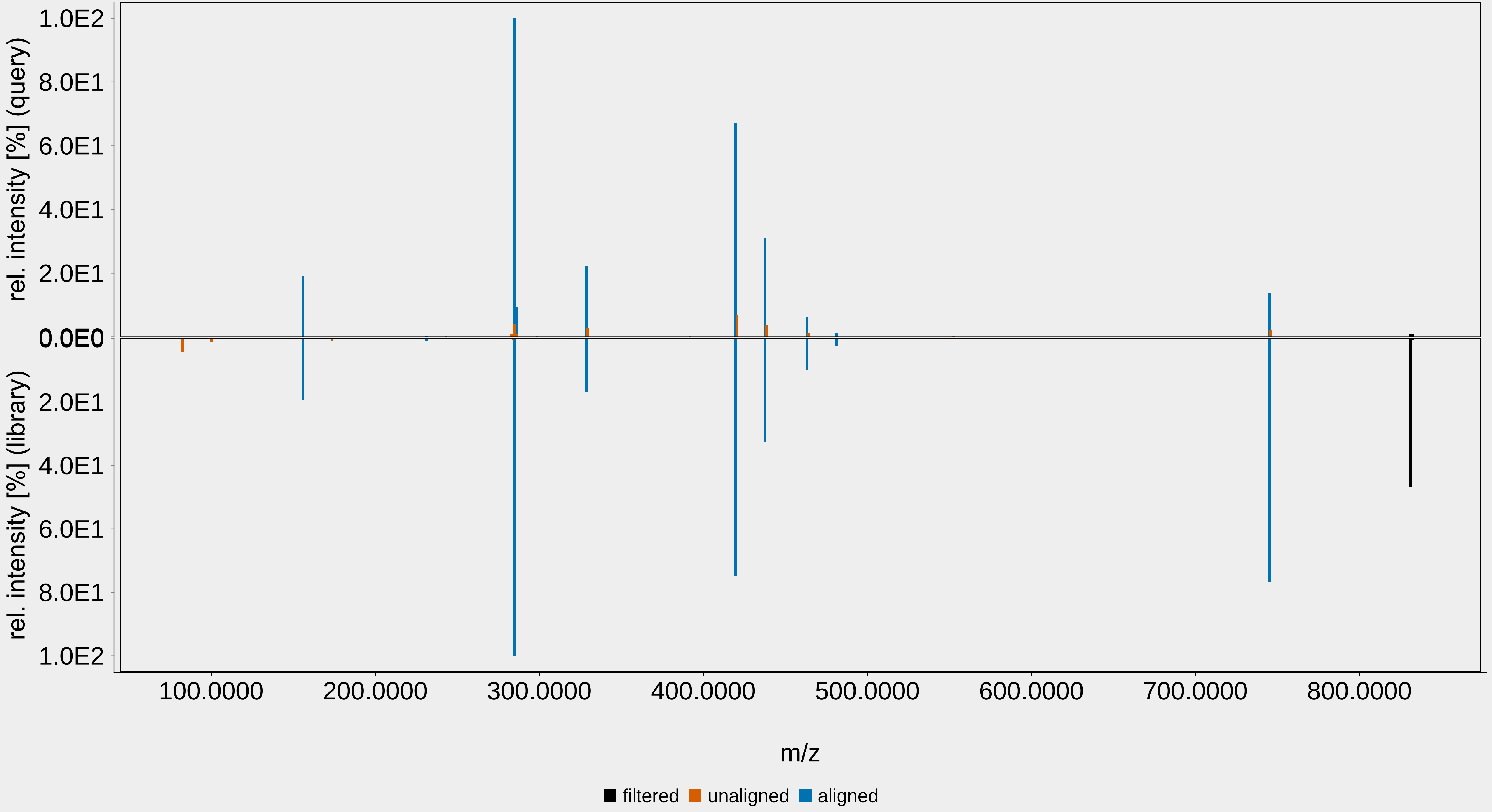

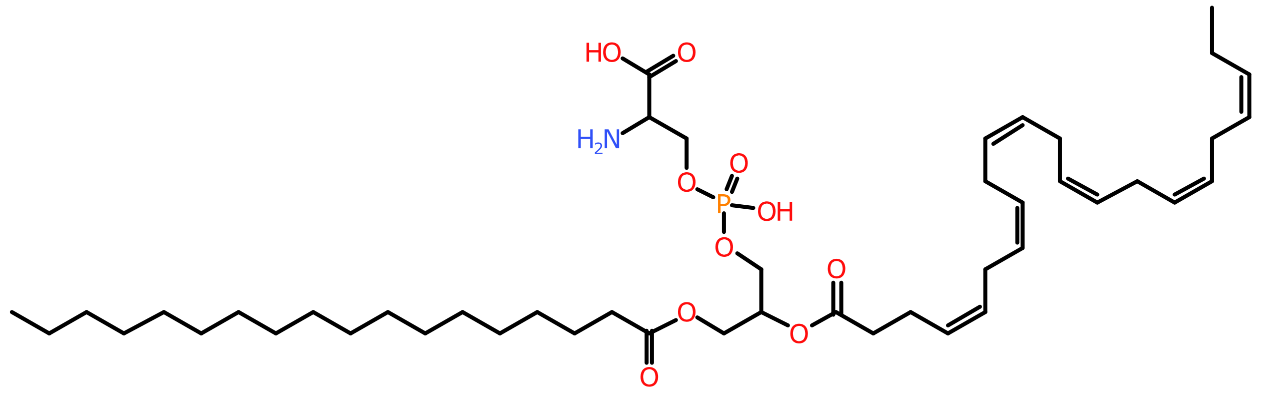

Compound information

Name: PS 40:6

Formula: C46H78NO10P

Mol. weight: 836.103

Exact mass: 836.103

Ion type: [M-H]-

Precursor m/z: 834.5288

RT: 8.68651

Polarity: negative

InChI:

InChI key:

SMILES:

Instrument information

Instrument type: LC-ESI-QTOF

Instrument: AB Sciex TripleTOF 5600+ system

Ms level: MS2

Collision energy: 45V

Database links

Pubchem: 91746251

MoNA ID: LQB00726

Other information

Principal investigator: Kazutaka Ikeda, Hiroshi

Data collector: Kazutaka Ikeda, Hiroshi

PS 40:6 **0.892**

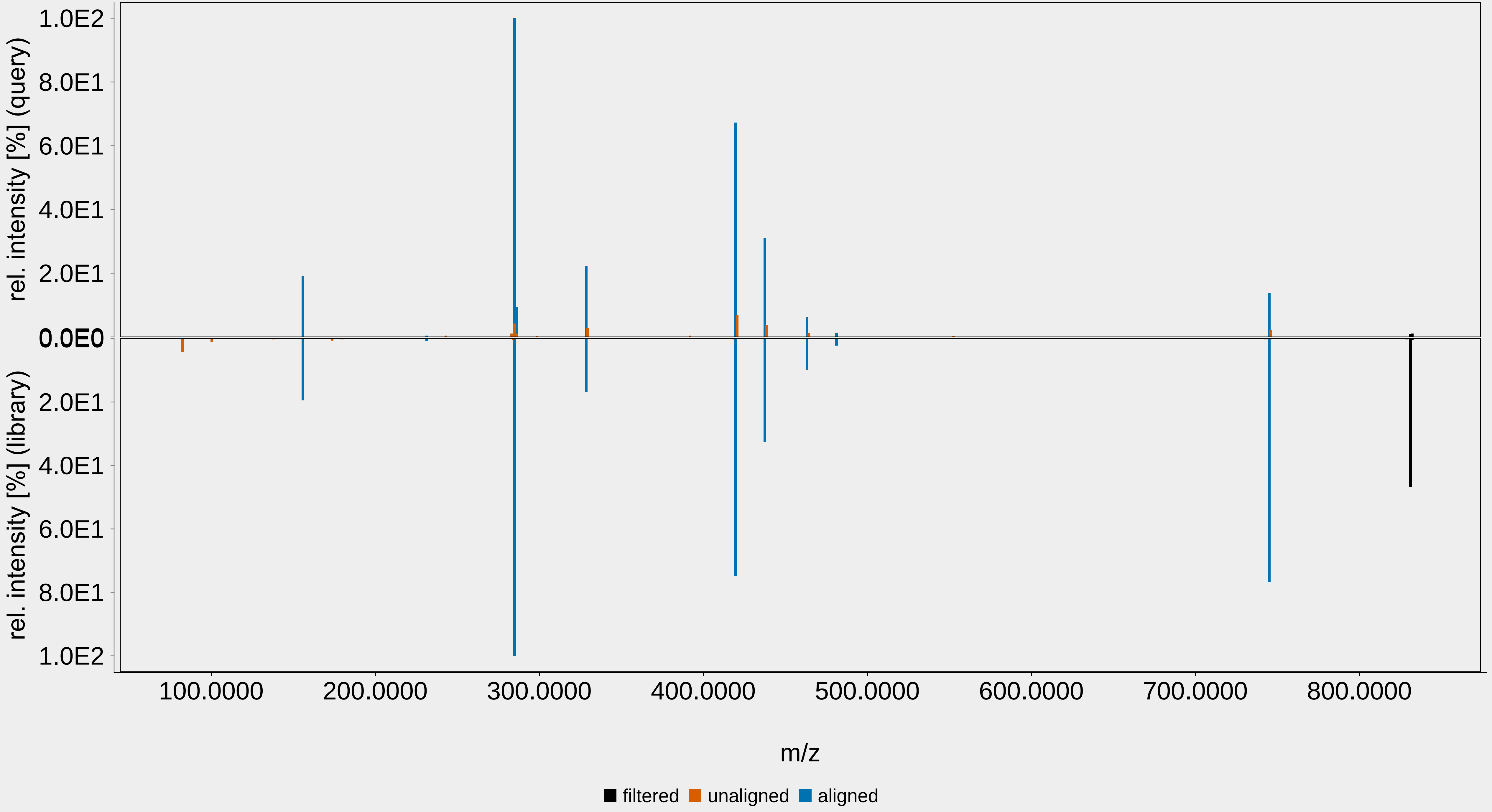

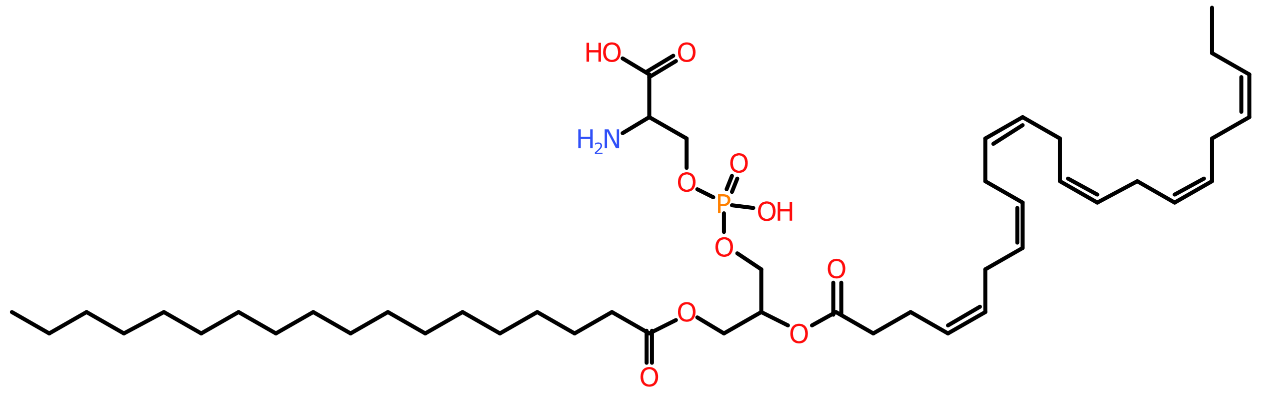

Compound information

Name: PS 40:6

Formula: C46H78NO10P

Mol. weight: 836.103

Exact mass: 836.103

Ion type: [M-H]-

Precursor m/z: 834.5288

RT: 8.68651

Polarity: negative

InChI:

InChI key:

SMILES:

Instrument information

Instrument type: LC-ESI-QTOF

Instrument: AB Sciex TripleTOF 5600+ system

Ms level: MS2

Collision energy: 45V

Database links

Pubchem: 91746251

MoNA ID: LQB00340

Other information

Principal investigator: Kazutaka Ikeda, Hiroshi

Data collector: Kazutaka Ikeda, Hiroshi

PS 18:1_20:4 0.755

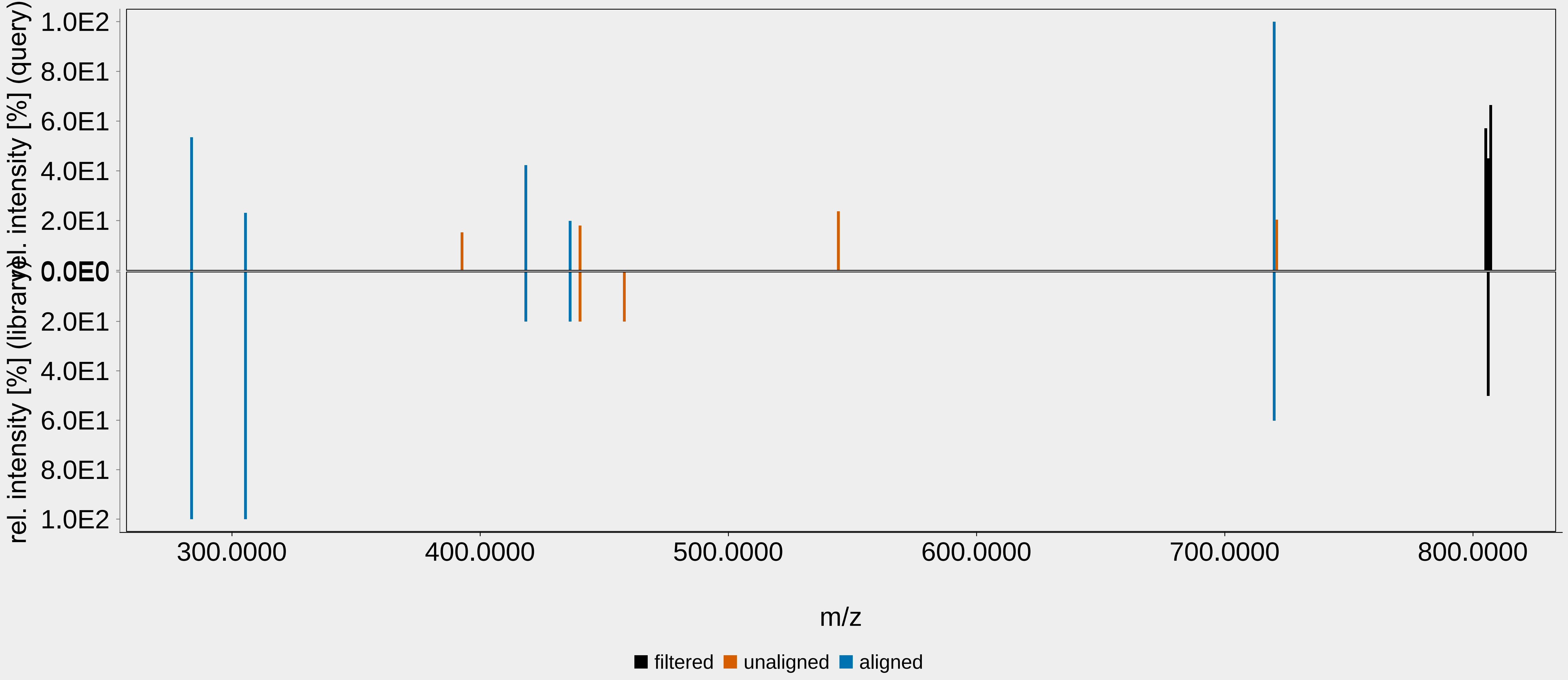

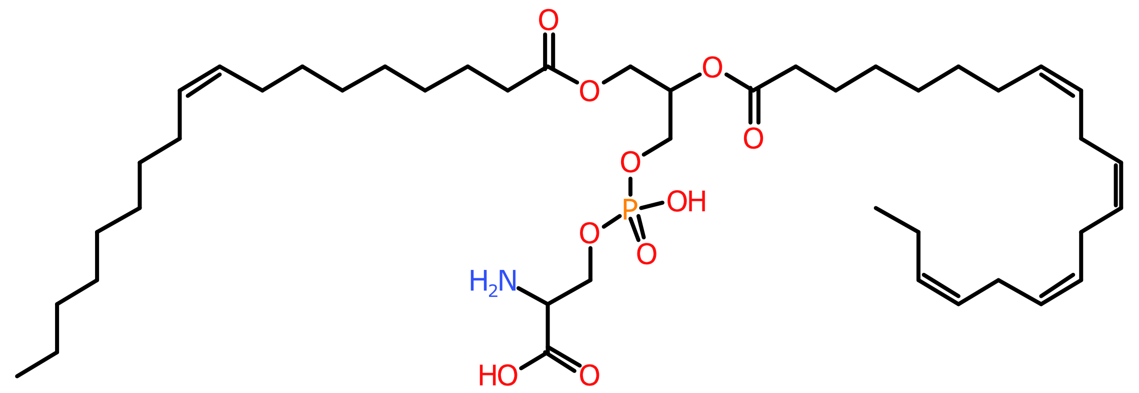

Compound information

Name: PS 18:1_20:4

Formula: C44H76NO10P

Ion type: [M-H]-

Precursor m/z: 808.51341

Ccs: 276.99

InChI key:

SMILES:

Num peaks: 8

Instrument information

Database links

Other information

PE(18:0/20:4); [M-H]- C43H77N1O8P1

0.903

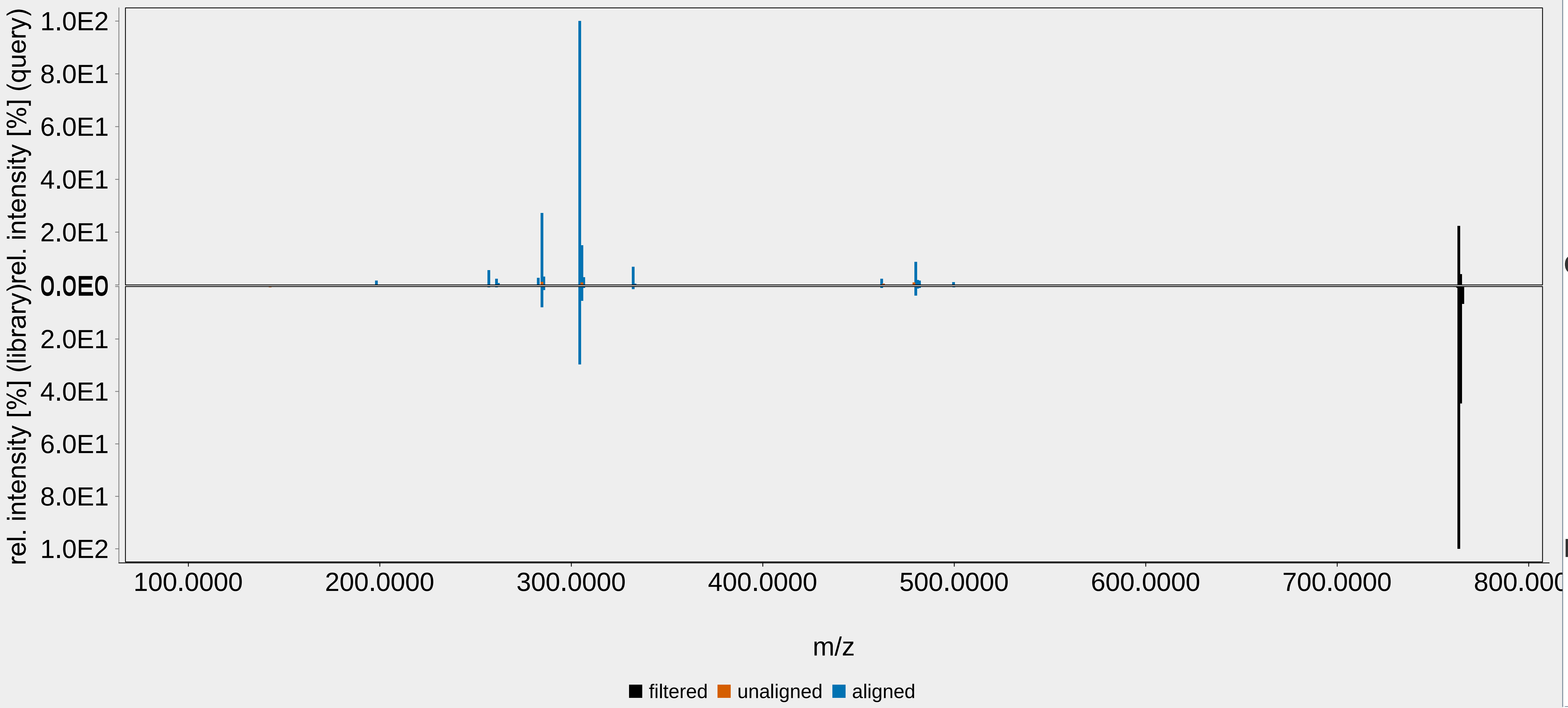

Compound information

Name: PE(18:0/20:4); [M-H]- C43H77N1O8P1

Exact mass: 0.0

Ion type: M-H

Precursor m/z: 766.539

Charge: 1

Polarity: Negative

Instrument information

Instrument type: HCD; Velos

Ion source: LC-ESI

Ms level: 2

Acquisition: Commercial

Database links

Other information

Principal investigator: Thomas Metz

Data collector: Thomas Metz

Entry ID: CCMSLIB00010074407

Spectral Match to 1-Octadecanoyl-2-(5Z,8Z,11Z,14Z-eicosatetraenoyl)-sn-glycero-3-phosphoethanolamine from NIST14

0.904

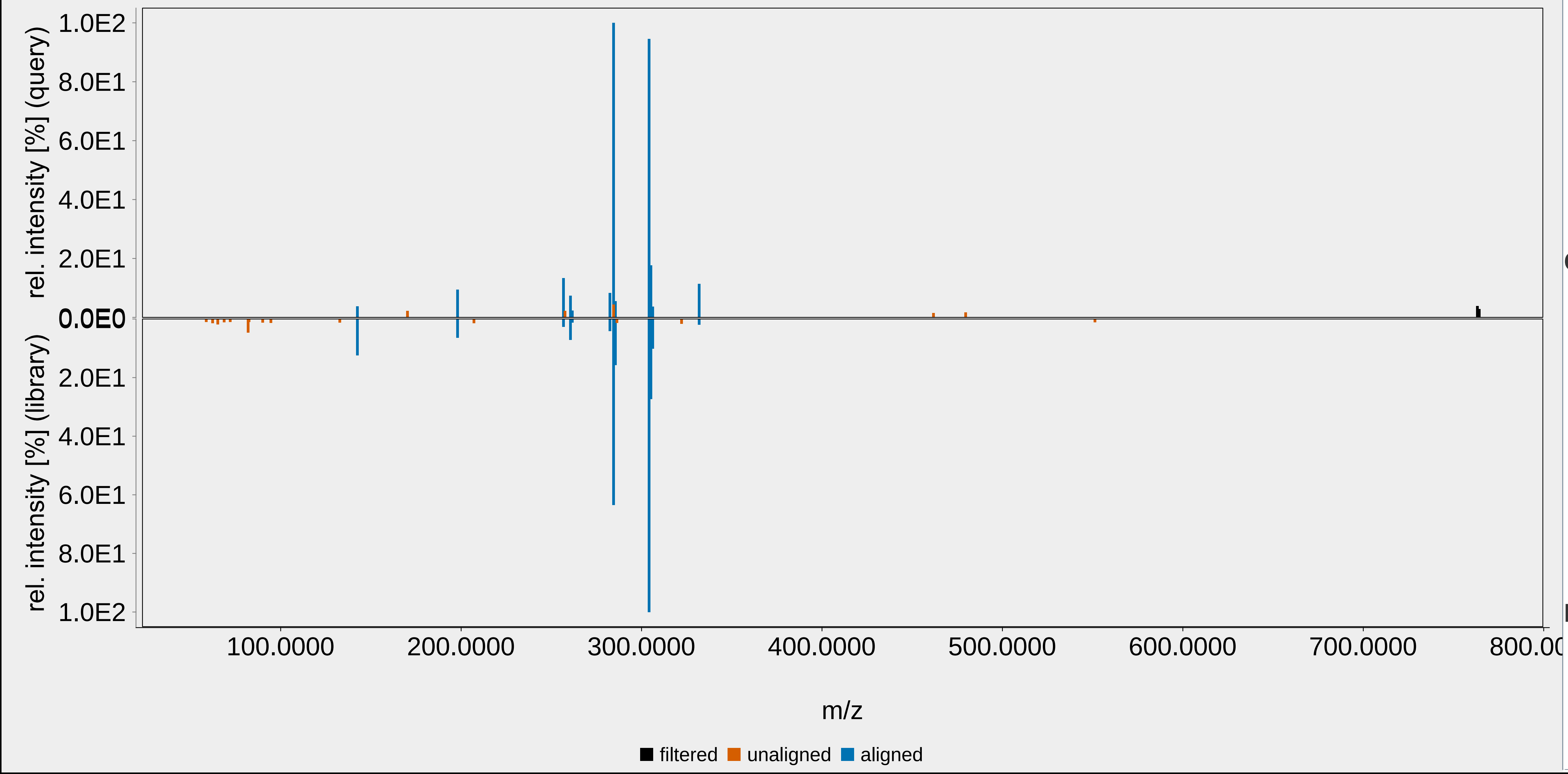

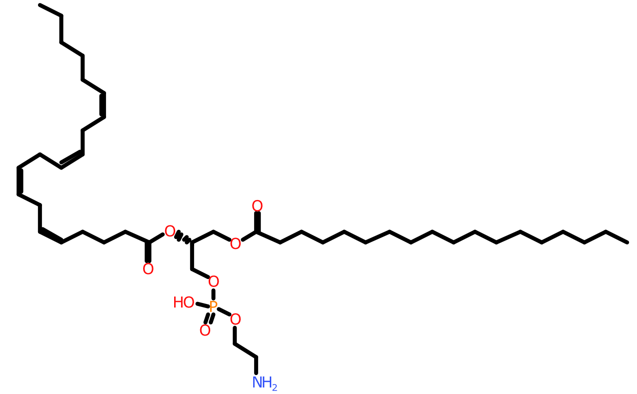

Compound information

Name: Spectral Match to

Exact mass: 767.547

Ion type: M-H

Precursor m/z: 766.541

Charge: 1

Polarity: Negative

InChI:

SMILES:

Instrument information

Instrument type: qTof

Ion source: ESI

Ms level: 2

Acquisition: Isolated

Database links

CAS: 61216624

Other information

Principal investigator: Data from Wiemann

Data collector: Data deposited by pwiemann

Entry ID: CCMSLIB00003139367

PE(16:1/18:1); [M-H]- C39H73N1O8P1 **0.892**

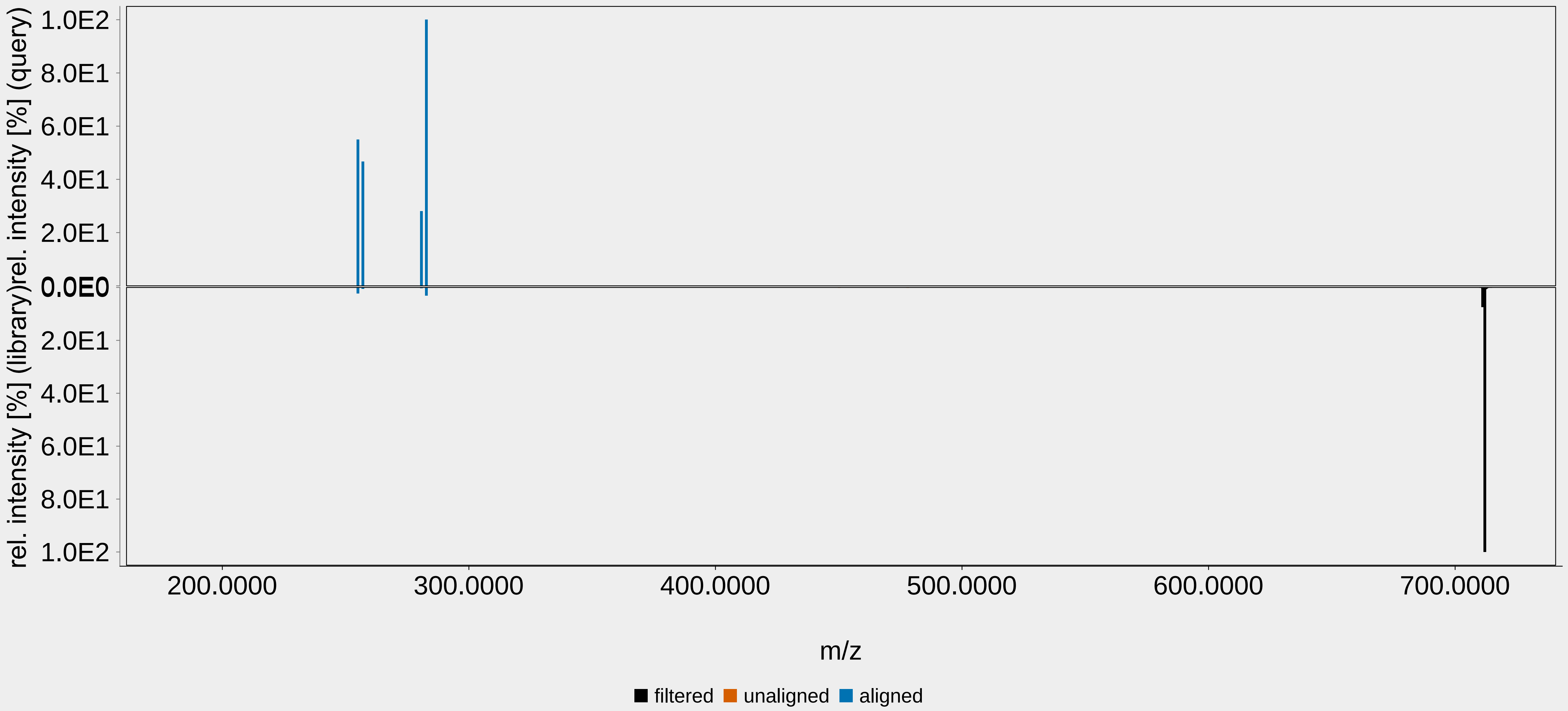

Compound information

Name: PE(16:1/18:1); [M-H]- C39H73N1O8P1

Exact mass: 0.0

Ion type: M-H

Precursor m/z: 714.507

Charge: 1

Polarity: Negative

Instrument information

Instrument type: HCD; Velos

Ion source: LC-ESI

Ms level: 2

Acquisition: Commercial

Database links

Other information

Principal investigator: Thomas Metz

Data collector: Thomas Metz

Entry ID: CCMSLIB00010060068

PE(16:1/18:1); [M-H]- C39H73N1O8P1 0.902

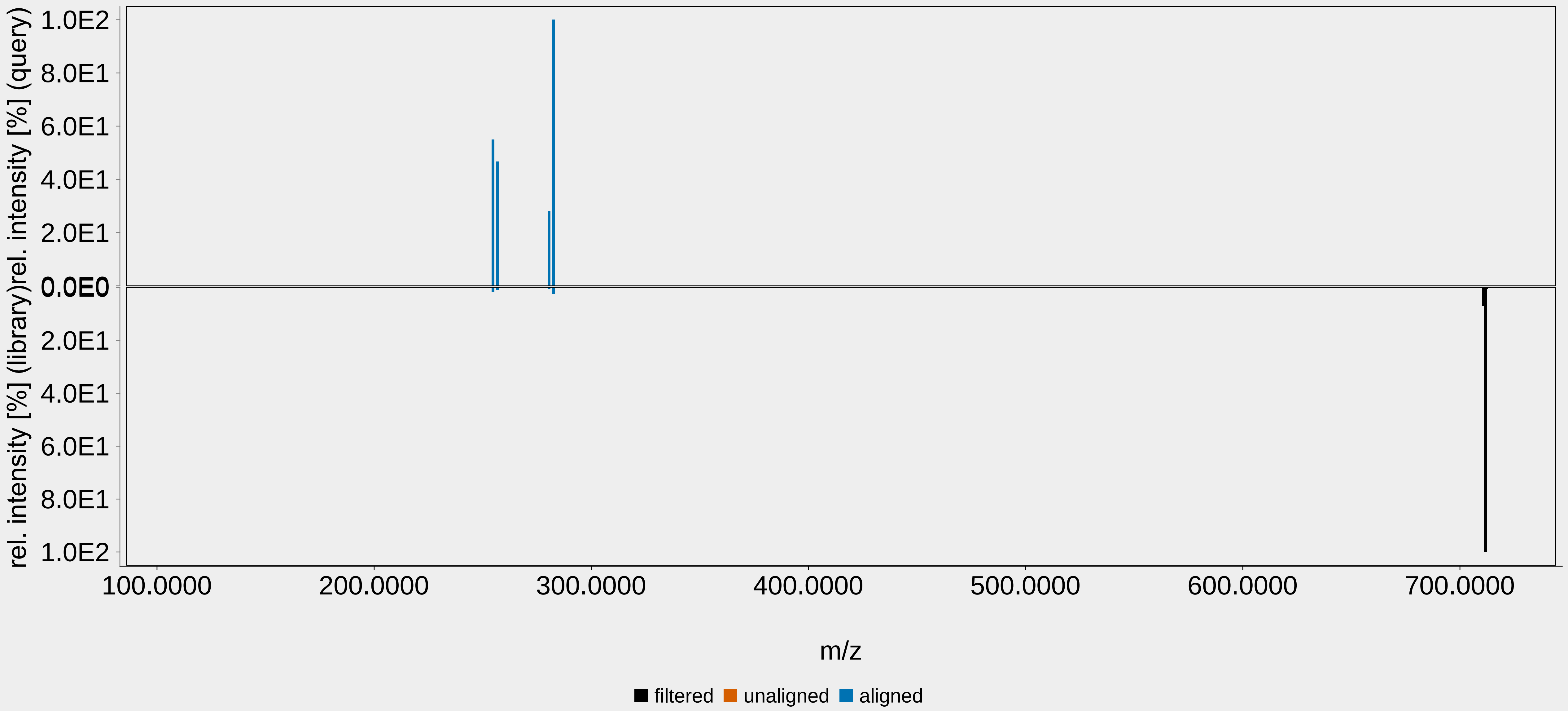

Compound information

Name: PE(16:1/18:1); [M-H]- C39H73N1O8P1

Exact mass: 0.0

Ion type: M-H

Precursor m/z: 714.507

Charge: 1

Polarity: Negative

Instrument information

Instrument type: HCD; Velos

Ion source: LC-ESI

Ms level: 2

Acquisition: Commercial

Database links

Other information

Principal investigator: Thomas Metz

Data collector: Thomas Metz

Entry ID: CCMSLIB00010060185

HYDROQUINIDINE

0.882

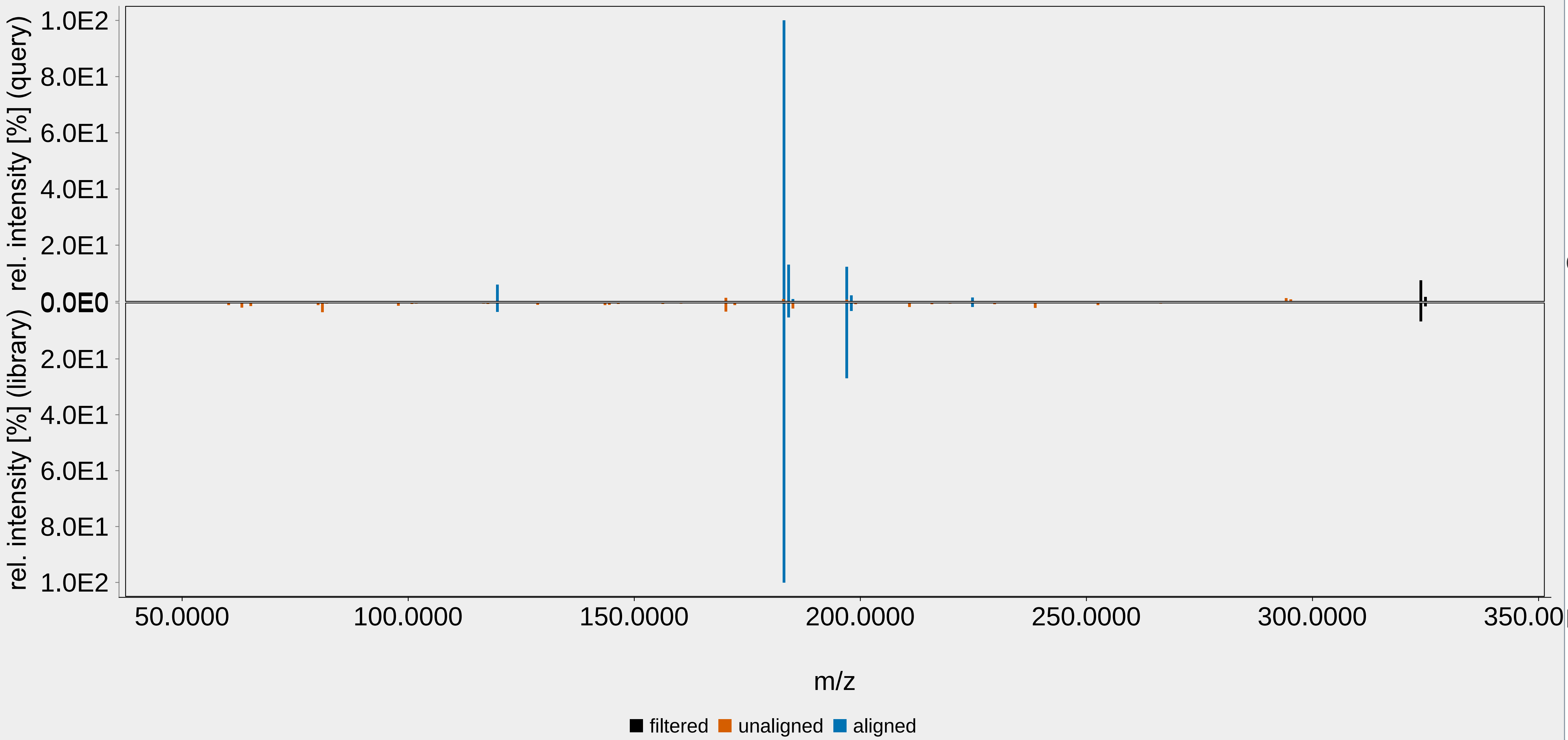

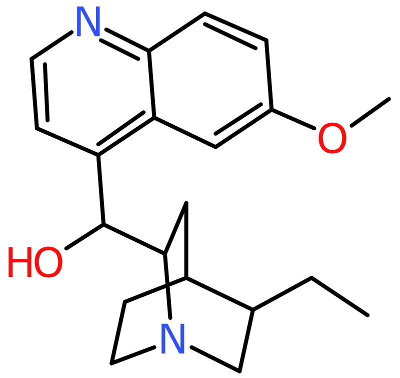

Compound information

Name: HYDROQUINIDINE

Formula: C20H26N2O2

Ion type: [M-H]-

Precursor m/z: 325.192138671875

Polarity: negative

InChI:

InChI key:

SMILES:

Instrument information

Instrument type: ESI-QFT

Instrument: Thermo Q Exactive HF

Ms level: MS2

Collision energy: 65HCD

Database links

Other information

Principal investigator: Arpana Vaniya

Data collector: Arpana Vaniya

HYDROQUINIDINE

0.882

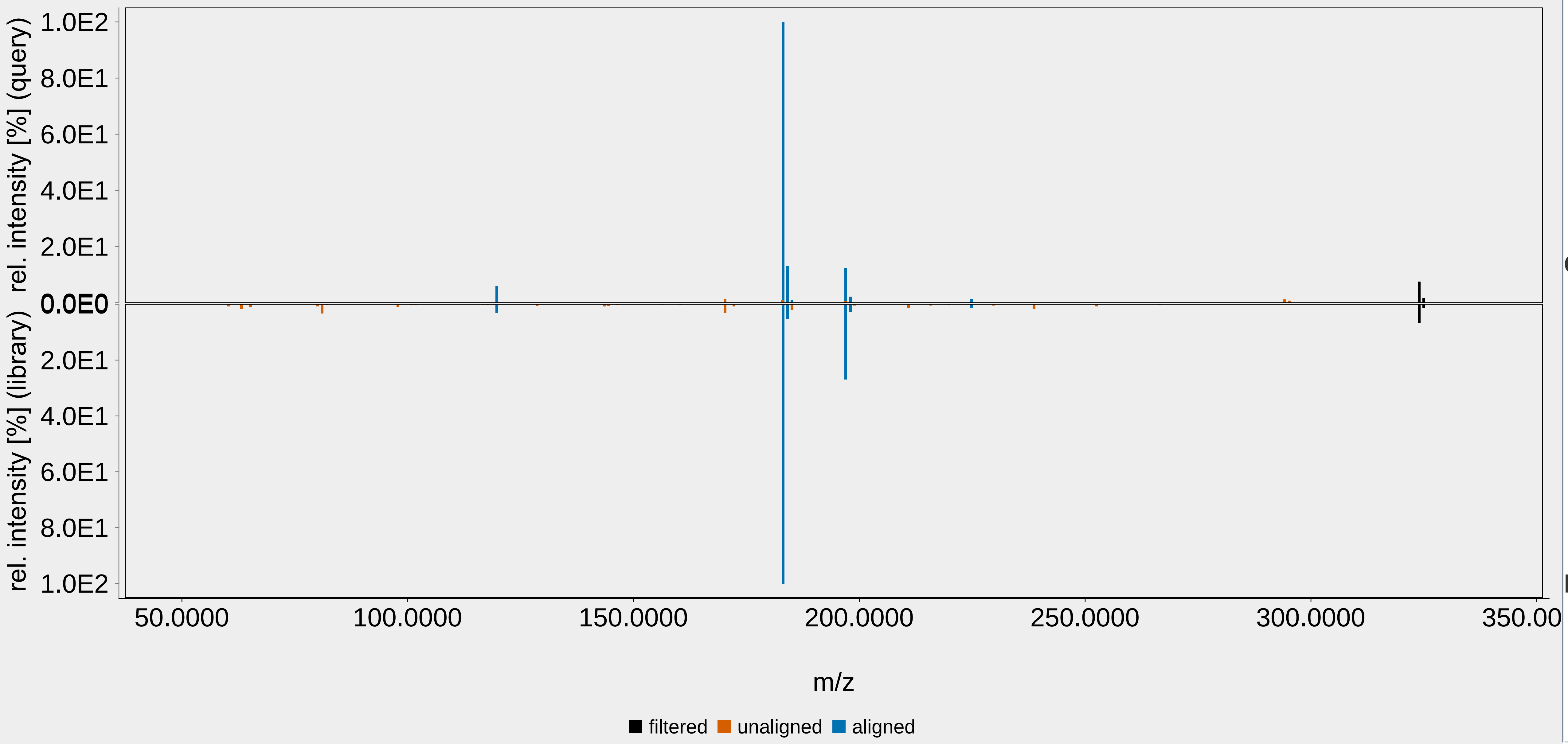

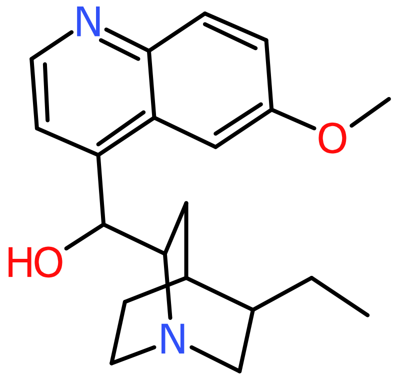

Compound information

Name: HYDROQUINIDINE

Exact mass: 0.0

Ion type: [M-H]-

Precursor m/z: 325.192

Charge: 1

Polarity: negative

InChI:

Instrument information

Instrument type: ESI-QFT

Ms level: 2

Acquisition: isolated

Database links

Other information

Principal investigator: MoNA

Data collector: MoNA:VF-NPL-QEHF003075

Entry ID: CCMSLIB00004694138

Crocetin semialdehyde

0.746

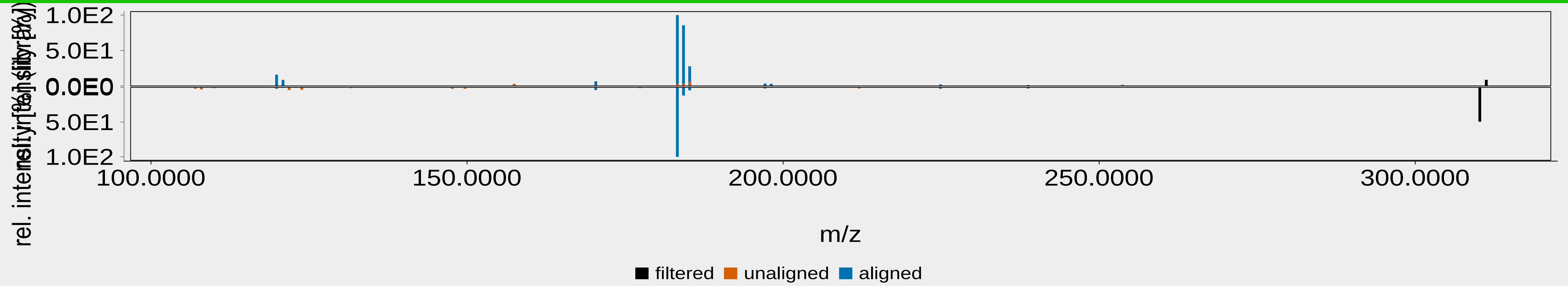

Compound information

Name: Crocetin semialdehyde

Ion type: carotenoid

Precursor m/z: 312.1725

Polarity: negative

Instrument information

Ion source: Electrospray Ionization (ESI)

Ms level: MS2

Database links

Other information

PA 24:0_18:1

0.864

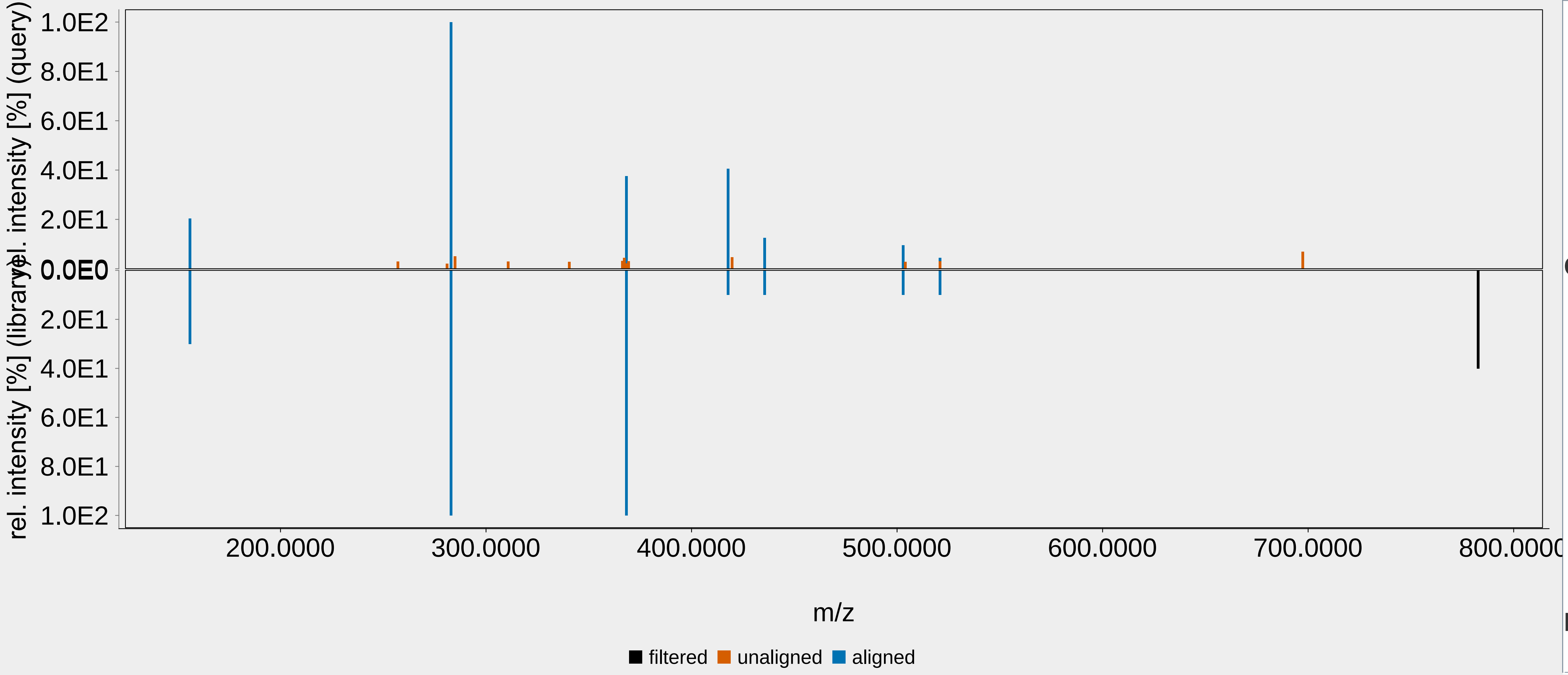

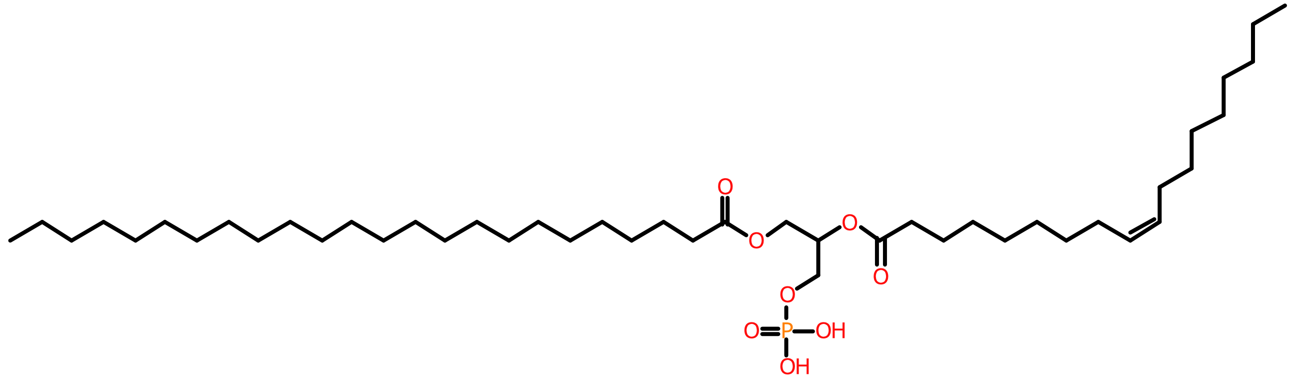

Compound information

Name: PA 24:0_18:1

Formula: C45H87O8P

Ion type: [M-H]-

Precursor m/z: 785.60658

Ccs: 278.64

InChI key:

SMILES:

Num peaks: 8

Instrument information

Database links

Other information

PS 36:1 0.744

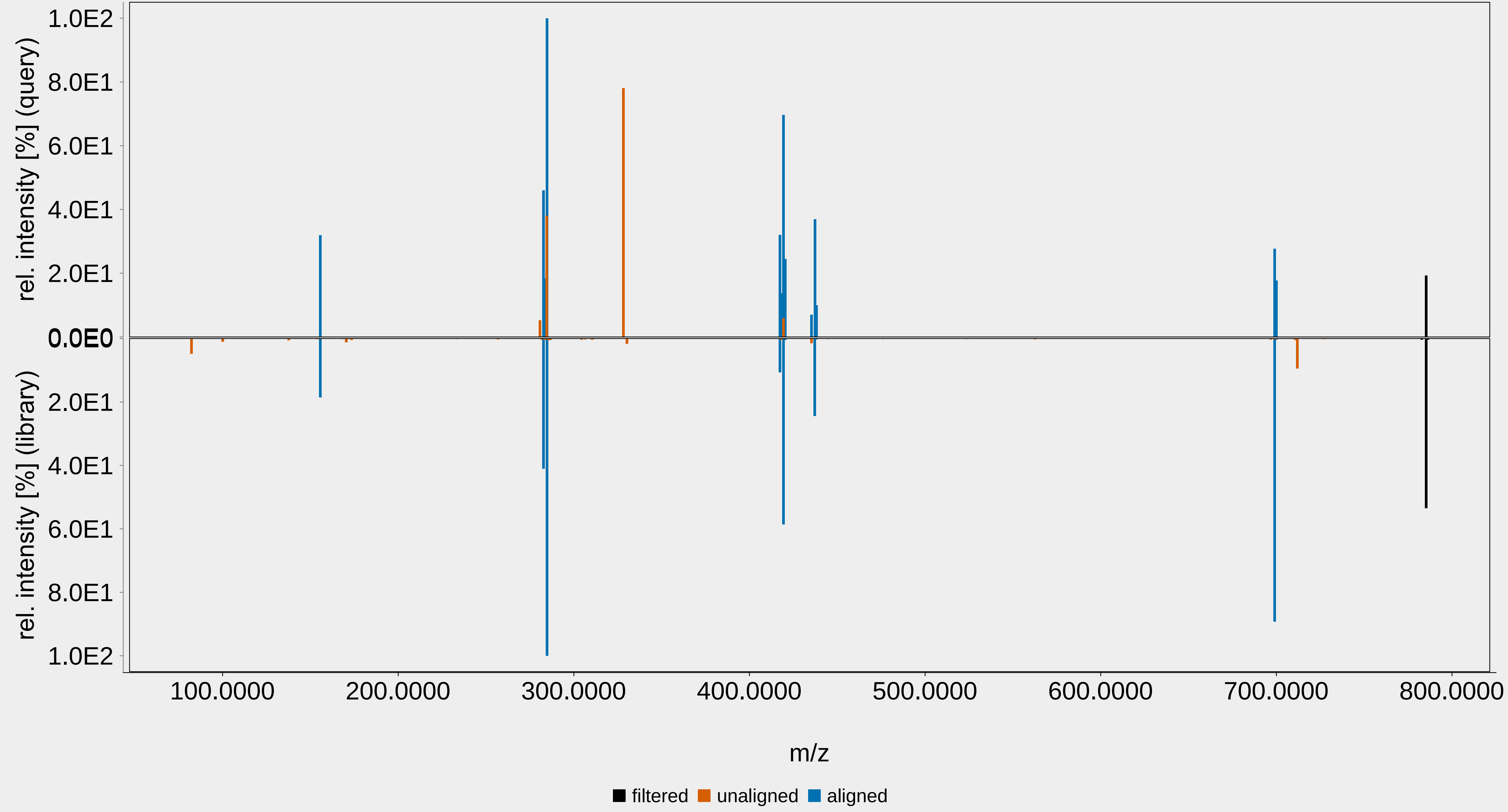

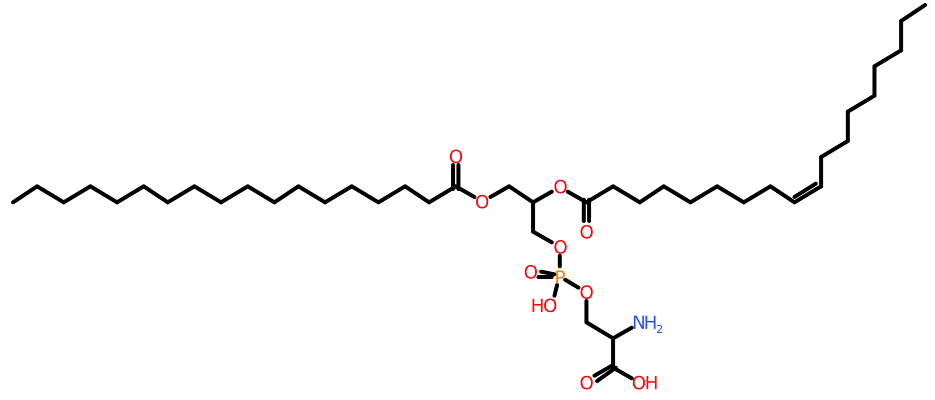

Compound information

Name: PS 36:1

Formula: C42H80NO10P

Mol. weight: 790.0749999999999

Exact mass: 790.0749999999999

Ion type: [M-H]-

Precursor m/z: 788.5428

RT: 9.185796

Polarity: negative

InChI:

InChI key: AJFWREUFUPEYII-ZZEZOPTASA-N

SMILES:

Instrument information

Instrument type: LC-ESI-QTOF

Instrument: AB Sciex TripleTOF 5600+ system

Ms level: MS2

Collision energy: 45V

Database links

Pubchem: 9962404

MoNA ID: LQB00719

Other information

Principal investigator: Kazutaka Ikeda, Hiroshi

Data collector: Kazutaka Ikeda, Hiroshi

PS 36:1 **0.744**

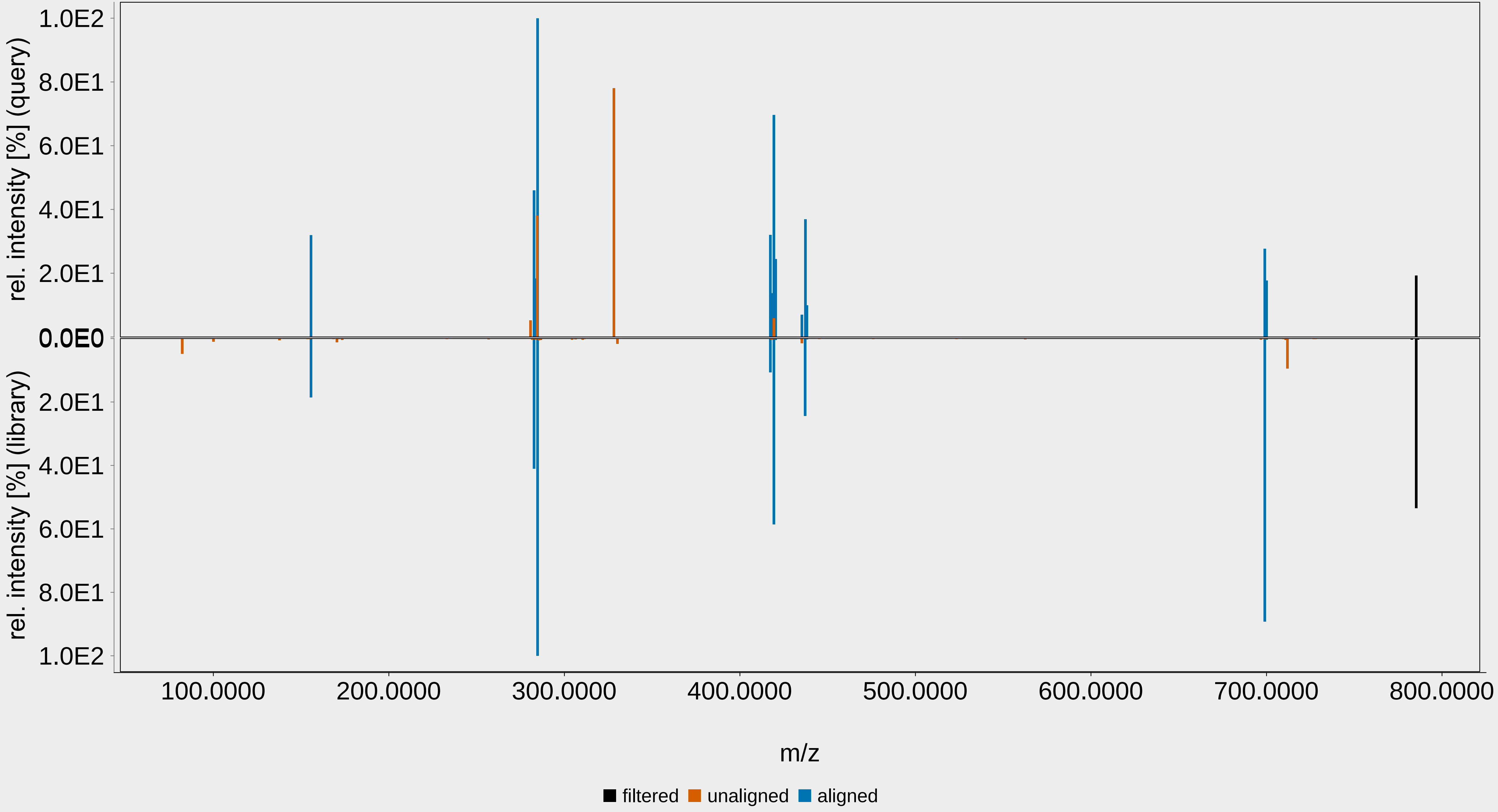

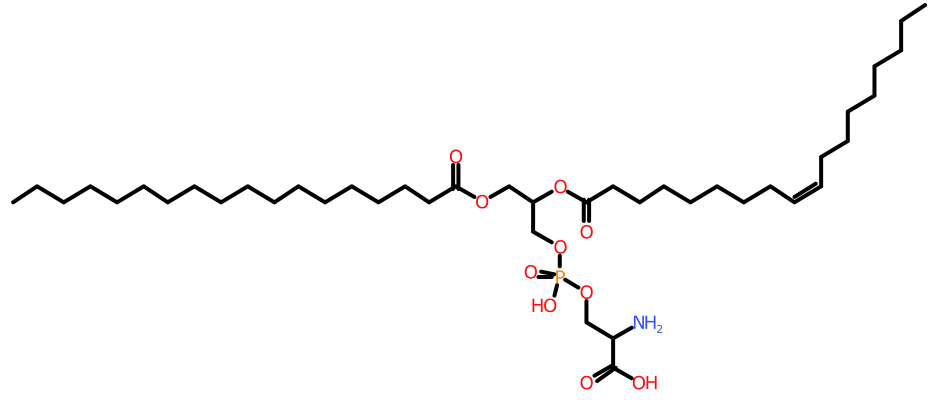

Compound information

Name: PS 36:1

Formula: C42H80NO10P

Mol. weight: 790.0749999999999

Exact mass: 790.0749999999999

Ion type: [M-H]-

Precursor m/z: 788.5428

RT: 9.185796

Polarity: negative

InChI:

InChI key: AJFWREUFUPEYII-ZZEZOPTASA-N

SMILES:

Instrument information

Instrument type: LC-ESI-QTOF

Instrument: AB Sciex TripleTOF 5600+ system

Ms level: MS2

Collision energy: 45V

Database links

Pubchem: 9962404

MoNA ID: LQB00333

Other information

Principal investigator: Kazutaka Ikeda, Hiroshi

Data collector: Kazutaka Ikeda, Hiroshi

PA 17:0_18:1

0.739

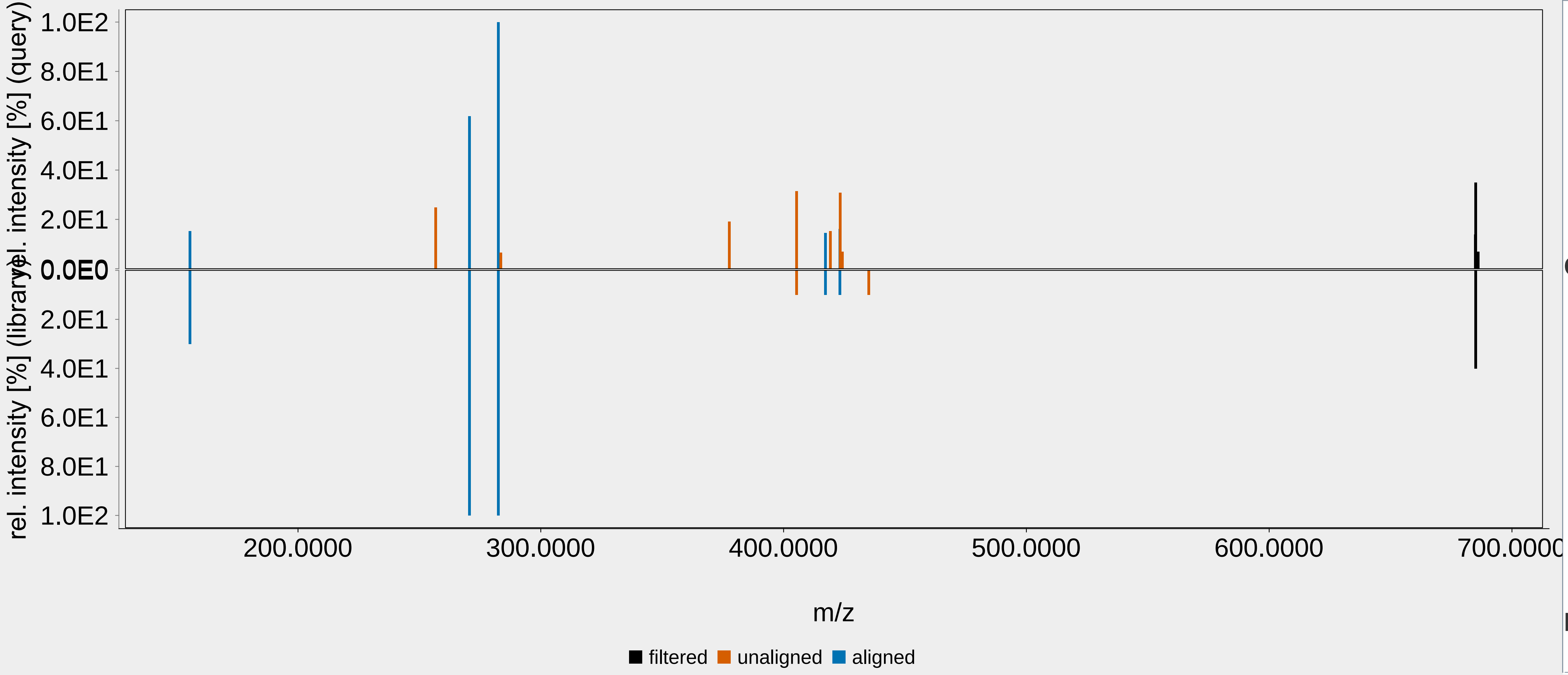

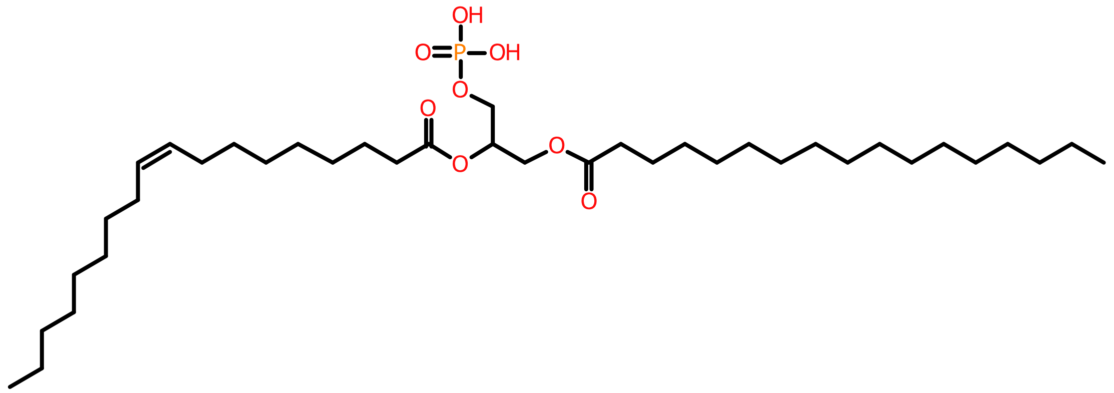

Compound information

Name: PA 17:0_18:1

Formula: C38H73O8P

Ion type: [M-H]-

Precursor m/z: 687.49703

Ccs: 257.18

InChI key:

SMILES:

Num peaks: 8

Instrument information

Database links

Other information

[3-[[2,3-dihydroxypropoxy]-hydroxyphosphoryl]oxy-2-hexadecanoyloxypropyl] hexadecanoate

0.856

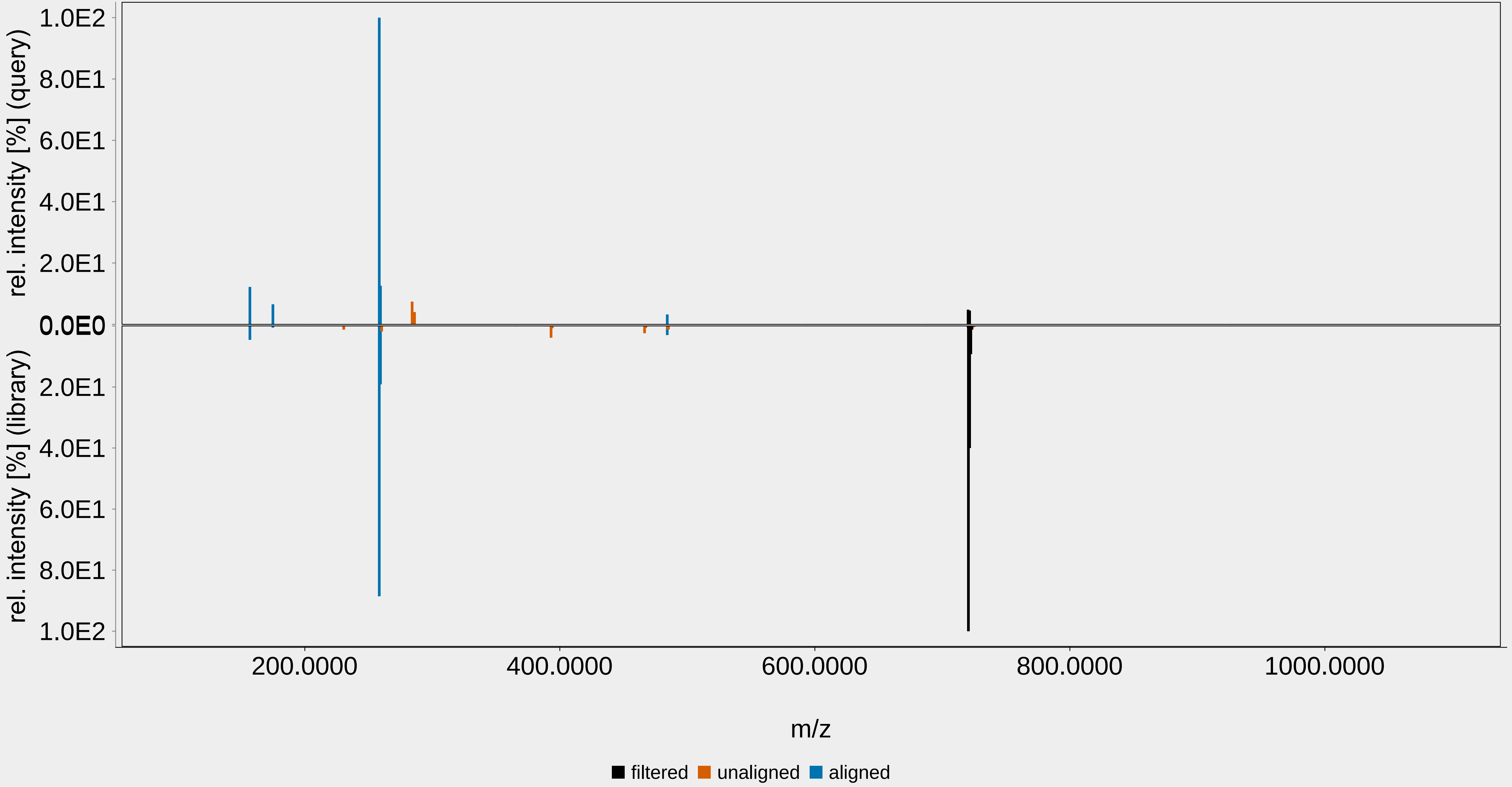

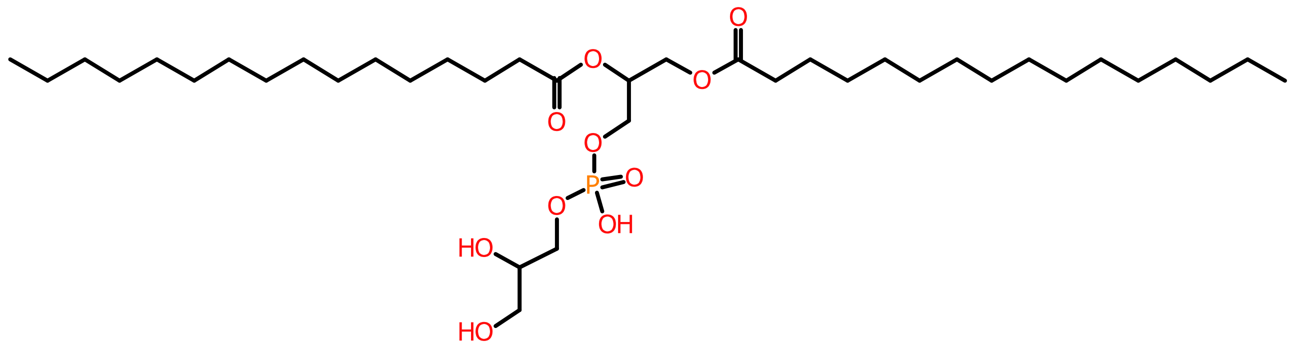

Compound information

Name:

Formula: C38H75O10P

Mol. weight: 722.51

Exact mass: 722.51

Ion type: [M-H]-

Precursor m/z: 721.502

Polarity: negative

InChI:

InChI key:

SMILES:

Instrument information

Instrument: qTof

Ms level: MS2

Database links

Other information

Principal investigator: O Laprevote, UMR 8038

Data collector: O Laprevote, UMR 8038

PG(16:0_16:0) - [3-[[2,3-dihydroxypropoxy]-hydroxyphosphoryl]oxy-2-hexadecanoyloxypropyl] hexadecanoate

0.856

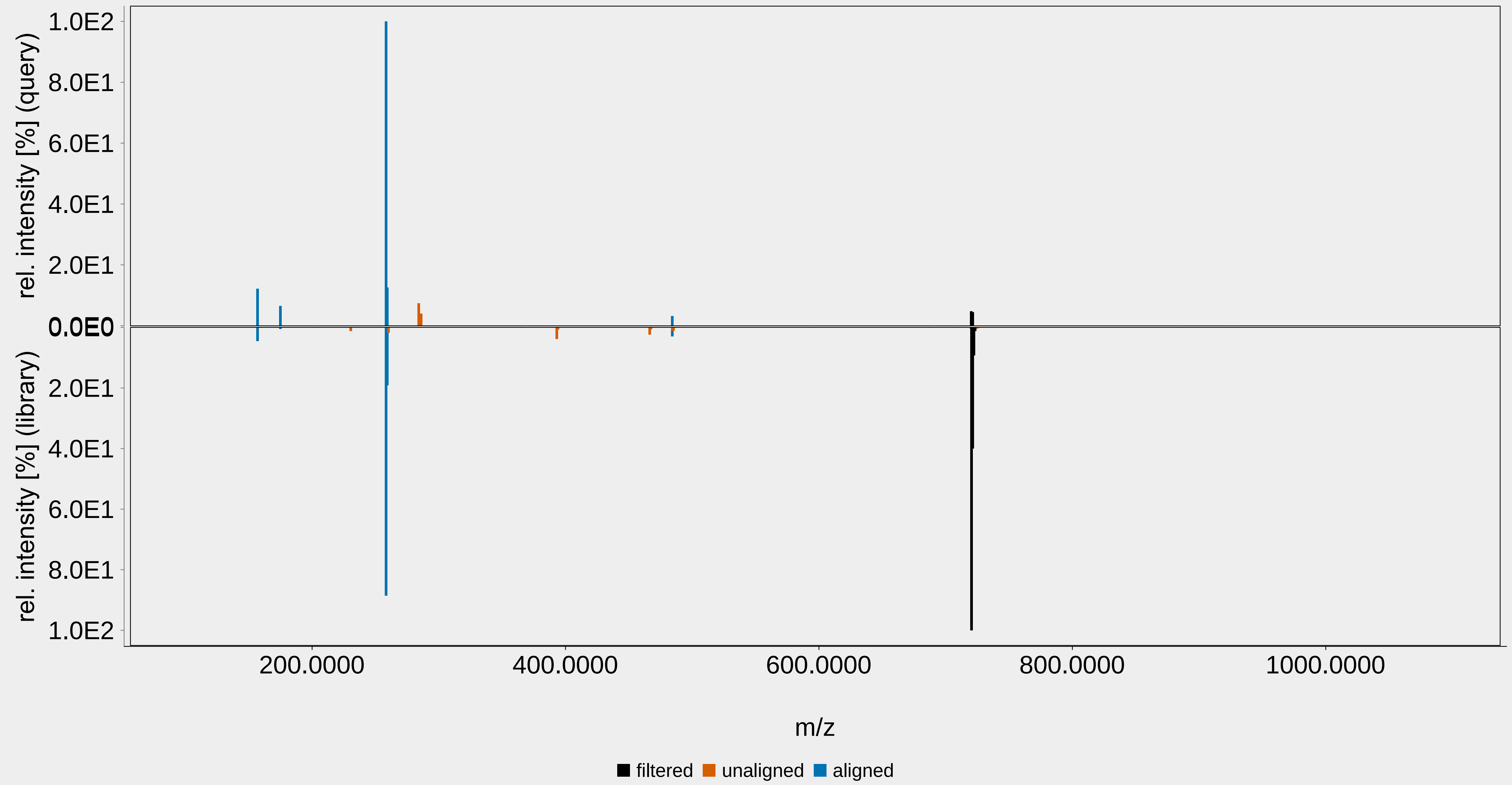

Compound information

Name: PG(16:0_16:0) -

Exact mass: 722.51

Ion type: M-H

Precursor m/z: 721.502

Charge: 1

Polarity: Negative

InChI:

InChI key:

SMILES:

Instrument information

Instrument type: qTof

Ion source: LC-ESI

Ms level: 2

Acquisition: Commercial

Database links

Pubmed:

CAS: 200880-41-7

Other information

Principal investigator: O Laprevote

Data collector: UMR 8038 Universite de Paris

Entry ID: CCMSLIB00005436447

PE(20:3/0:0); [M-H]- C25H45N1O7P1 0.853

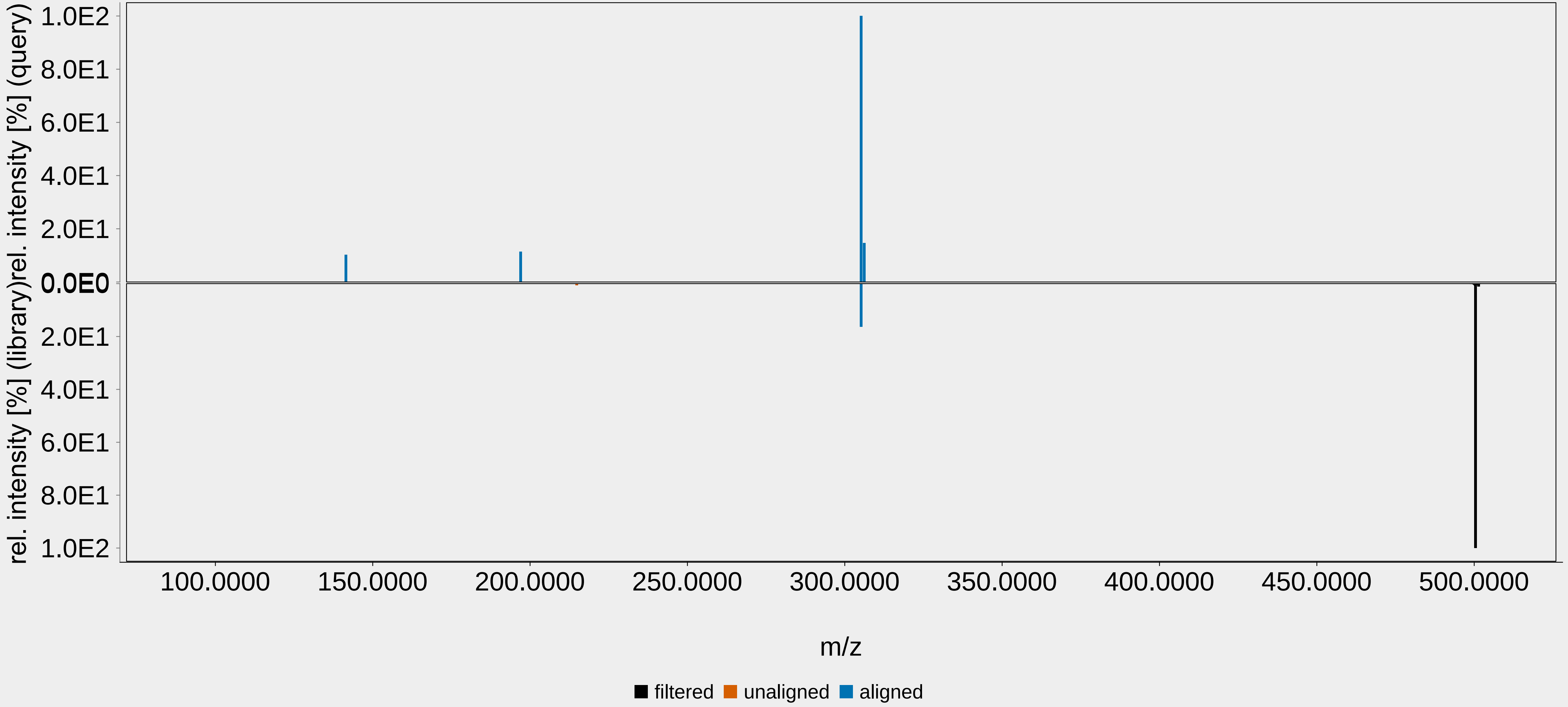

Compound information

Name: PE(20:3/0:0); [M-H]- C25H45N1O7P1

Exact mass: 0.0

Ion type: M-H

Precursor m/z: 502.293

Charge: 1

Polarity: Negative

Instrument information

Instrument type: HCD; Velos

Ion source: LC-ESI

Ms level: 2

Acquisition: Commercial

Database links

Other information

Principal investigator: Thomas Metz

Data collector: Thomas Metz

Entry ID: CCMSLIB00010080527

PE(20:3/0:0); [M-H]- C25H45N1O7P1 **0.868**

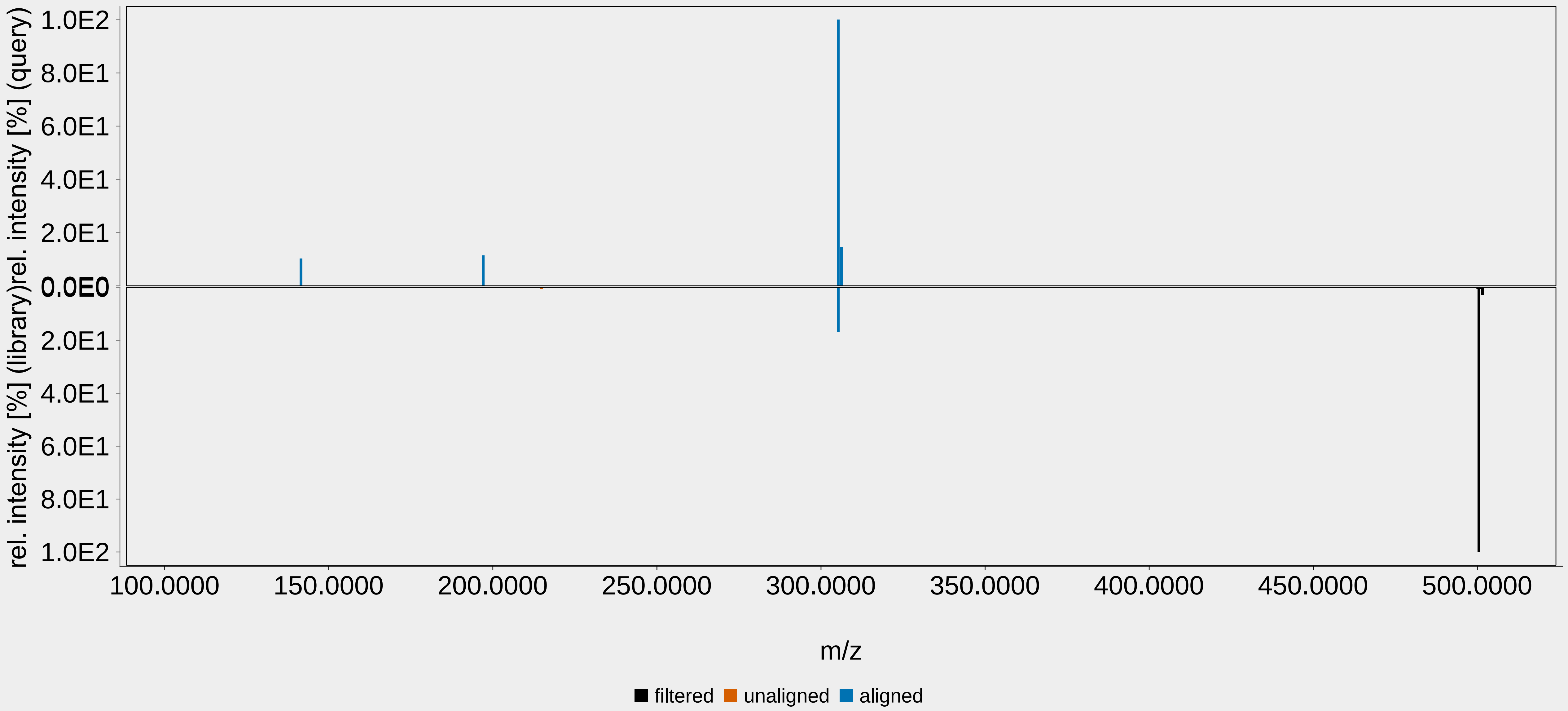

Compound information

Name: PE(20:3/0:0); [M-H]- C25H45N1O7P1

Exact mass: 0.0

Ion type: M-H

Precursor m/z: 502.293

Charge: 1

Polarity: Negative

Instrument information

Instrument type: HCD; Velos

Ion source: LC-ESI

Ms level: 2

Acquisition: Commercial

Database links

Other information

Principal investigator: Thomas Metz

Data collector: Thomas Metz

Entry ID: CCMSLIB00010079788

PE(18:1/18:2); [M-H]- C41H75N1O8P1

0.836

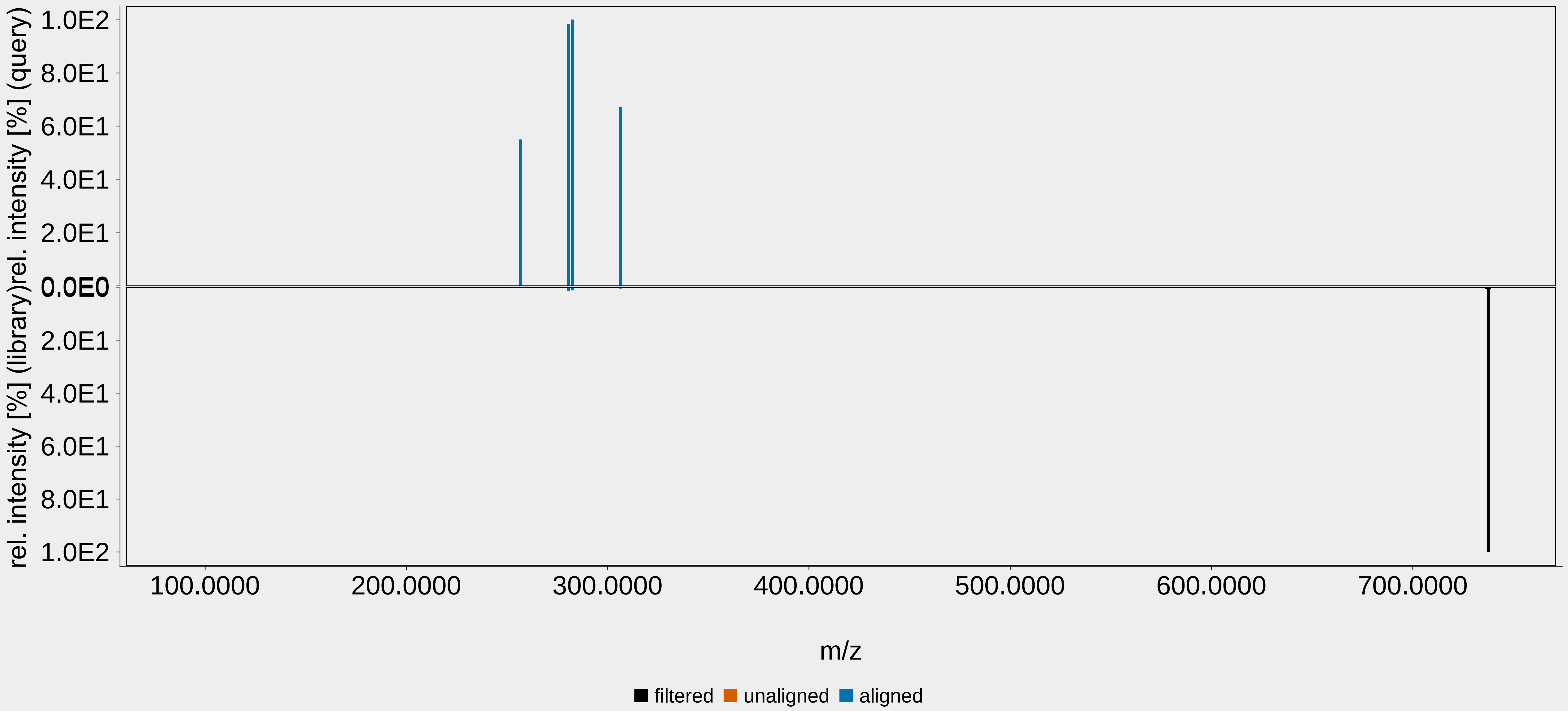

Compound information

Name: PE(18:1/18:2); [M-H]- C41H75N1O8P1

Exact mass: 0.0

Ion type: M-H

Precursor m/z: 740.523

Charge: 1

Polarity: Negative

Instrument information

Instrument type: HCD; Velos

Ion source: LC-ESI

Ms level: 2

Acquisition: Commercial

Database links

Other information

Principal investigator: Thomas Metz

Data collector: Thomas Metz

Entry ID: CCMSLIB00010076756

PE(18:1/18:2); [M-H]- C41H75N1O8P1 **0.880**

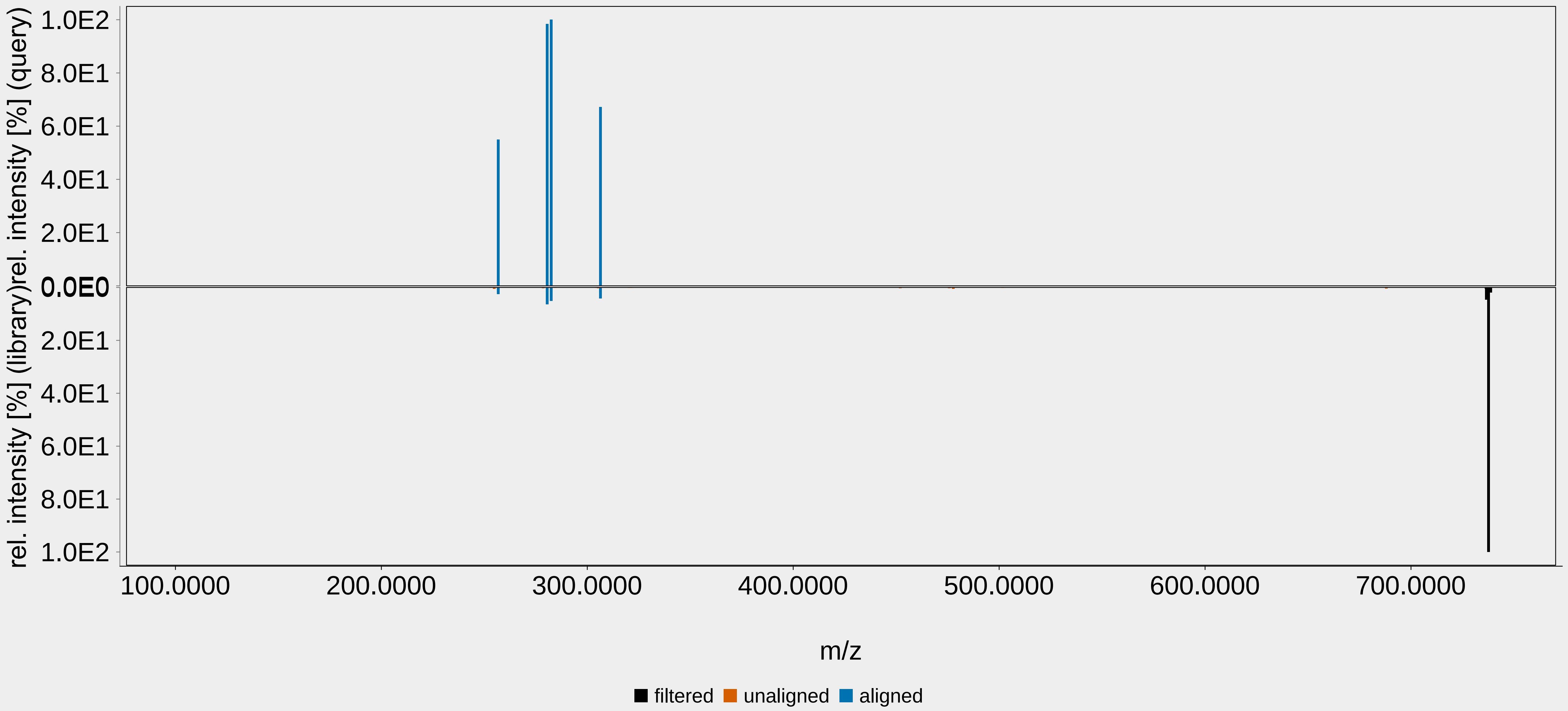

Compound information

Name: PE(18:1/18:2); [M-H]- C41H75N1O8P1

Exact mass: 0.0

Ion type: M-H

Precursor m/z: 740.523

Charge: 1

Polarity: Negative

Instrument information

Instrument type: HCD; Velos

Ion source: LC-ESI

Ms level: 2

Acquisition: Commercial

Database links

Other information

Principal investigator: Thomas Metz

Data collector: Thomas Metz

Entry ID: CCMSLIB00010071000

PS 18:0_20:3 0.753

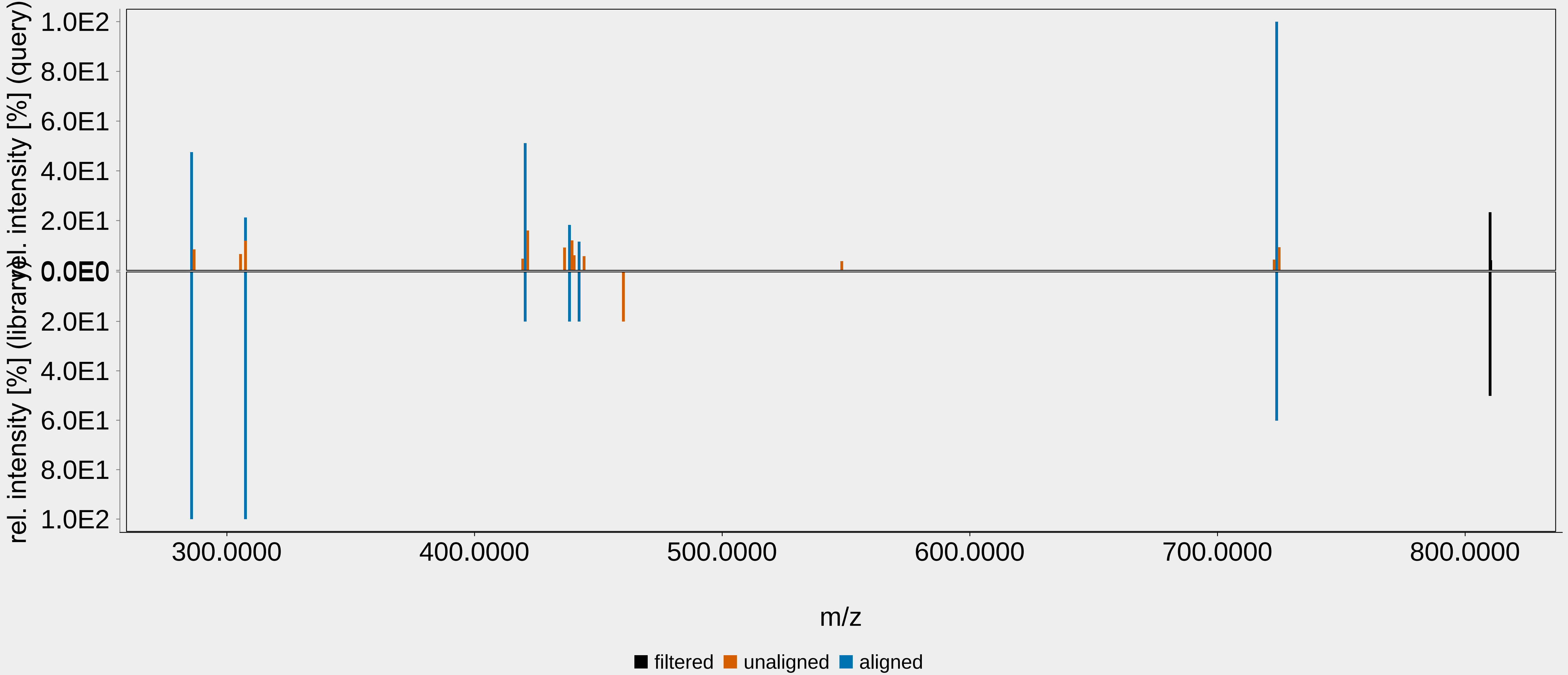

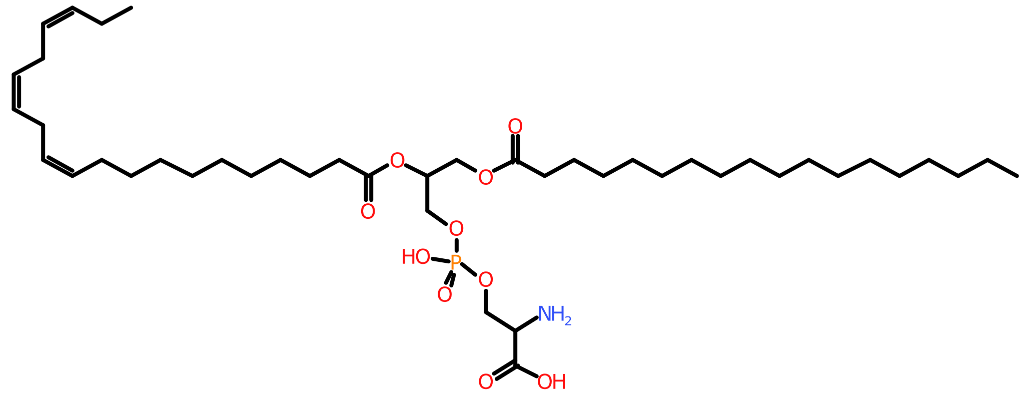

Compound information

Name: PS 18:0_20:3

Formula: C44H80NO10P

Ion type: [M-H]-

Precursor m/z: 812.54471

Ccs: 279.88

InChI key:

SMILES:

Num peaks: 8

Instrument information

Database links

Other information

PS(18:0/20:3); [M-H]- C44H79N1O10P1

0.863

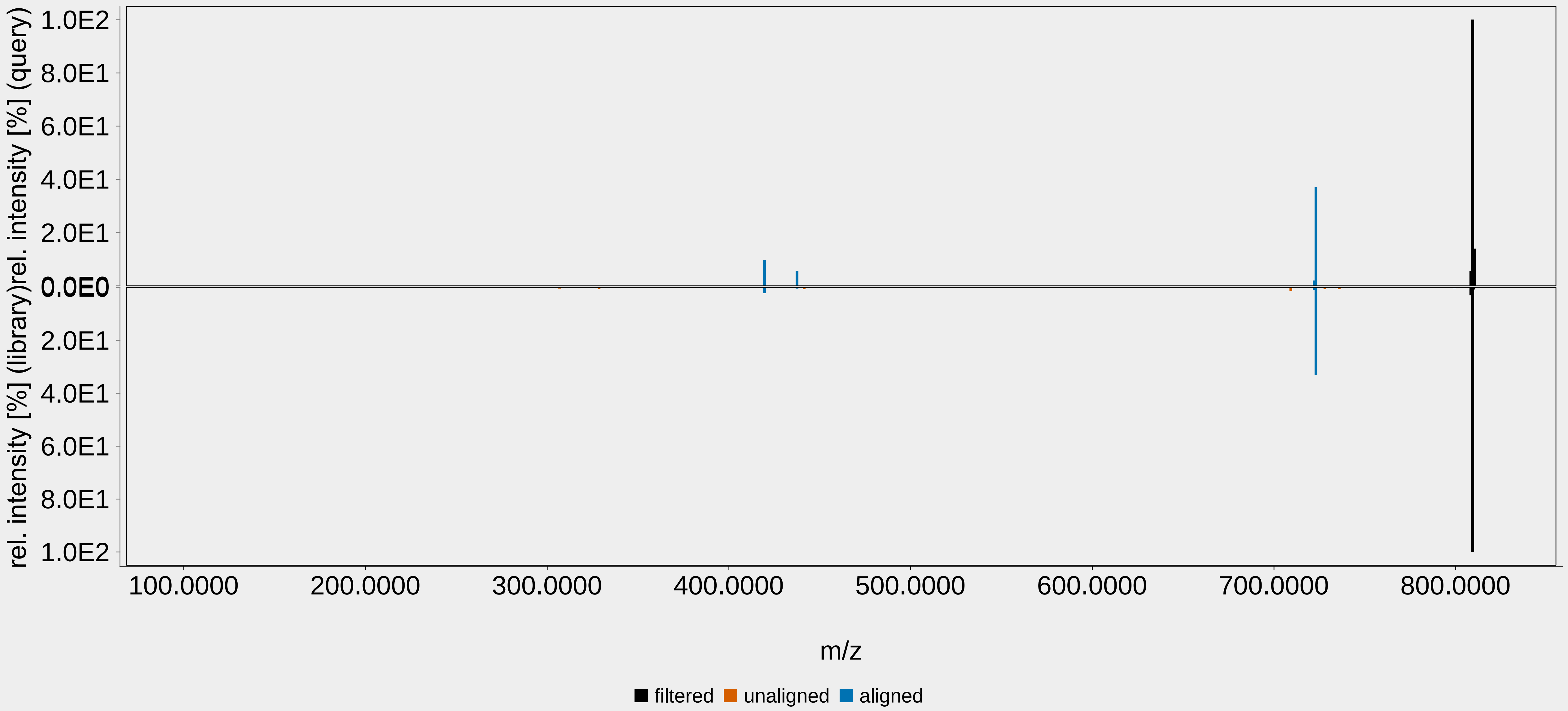

Compound information

Name: PS(18:0/20:3); [M-H]- C44H79N1O10P1

Exact mass: 0.0

Ion type: M-H

Precursor m/z: 812.544

Charge: 1

Polarity: Negative

Instrument information

Instrument type: HCD; Velos

Ion source: LC-ESI

Ms level: 2

Acquisition: Commercial

Database links

Other information

Principal investigator: Thomas Metz

Data collector: Thomas Metz

Entry ID: CCMSLIB00010077483

PE(20:2/0:0); [M-H]- C25H47N1O7P1 **0.925**

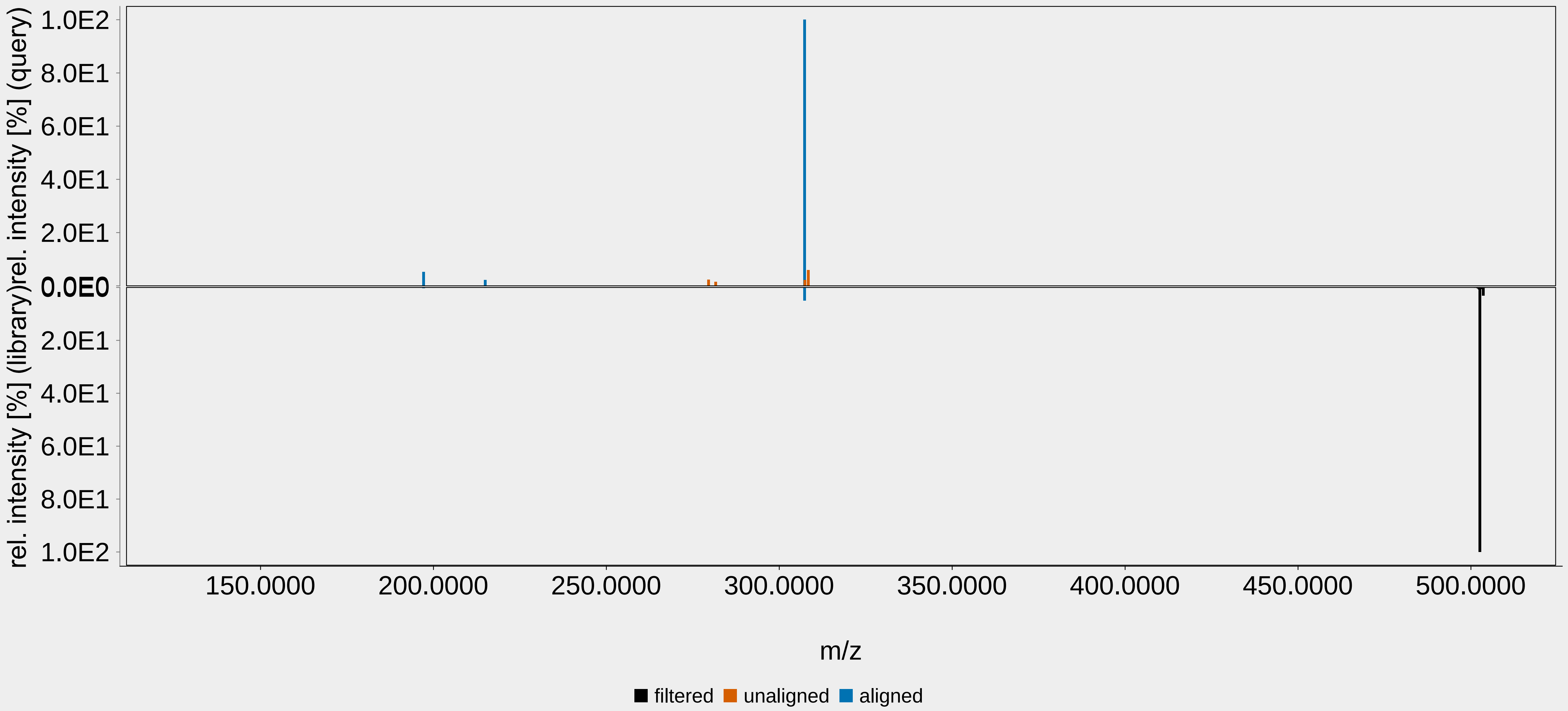

Compound information

Name: PE(20:2/0:0); [M-H]- C25H47N1O7P1

Exact mass: 0.0

Ion type: M-H

Precursor m/z: 504.309

Charge: 1

Polarity: Negative

Instrument information

Instrument type: HCD; Velos

Ion source: LC-ESI

Ms level: 2

Acquisition: Commercial

Database links

Other information

Principal investigator: Thomas Metz

Data collector: Thomas Metz

Entry ID: CCMSLIB00010086312

PS 36:4 **0.759**

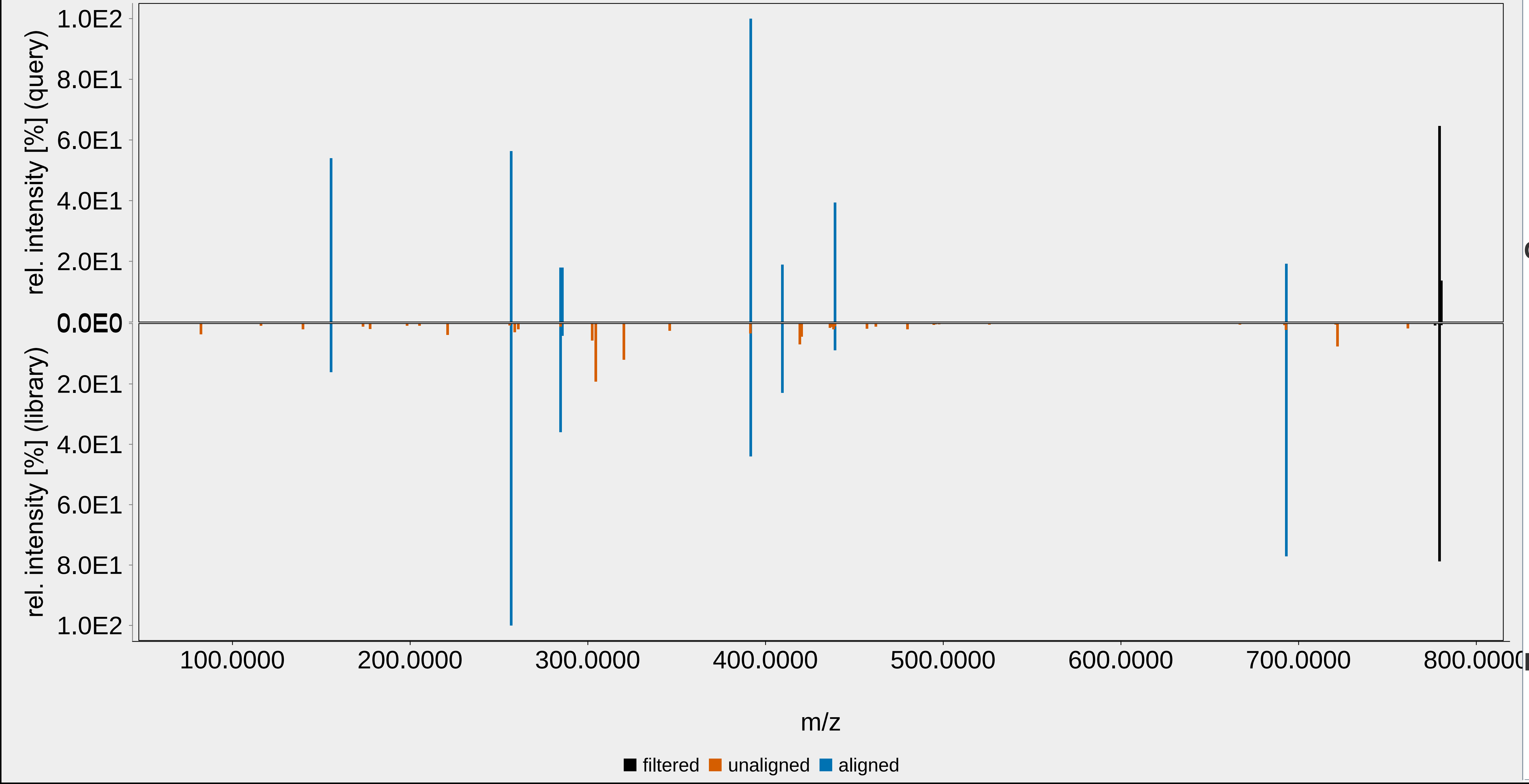

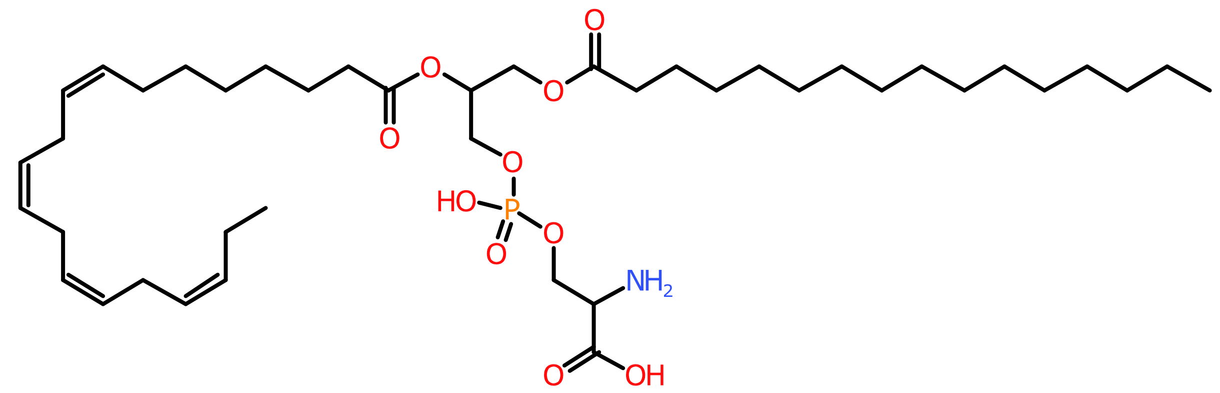

Compound information

Name: PS 36:4

Formula: C42H74NO10P

Exact mass: 784.0269999999999

Ion type: [M-H]-

Precursor m/z: 782.494

Polarity: NEGATIVE

InChI:

InChI key:

SMILES:

Num peaks: 55

Instrument information

Instrument type: LC-ESI-QTOF

Instrument: AB Sciex TripleTOF 5600+ system

Ms level: MS2

Collision energy: 45V

Database links

Other information

Entry ID: MSBNK-RIKEN_IMS-LQB00715

Comment: Parent=782.494

PS 36:4 **0.759**

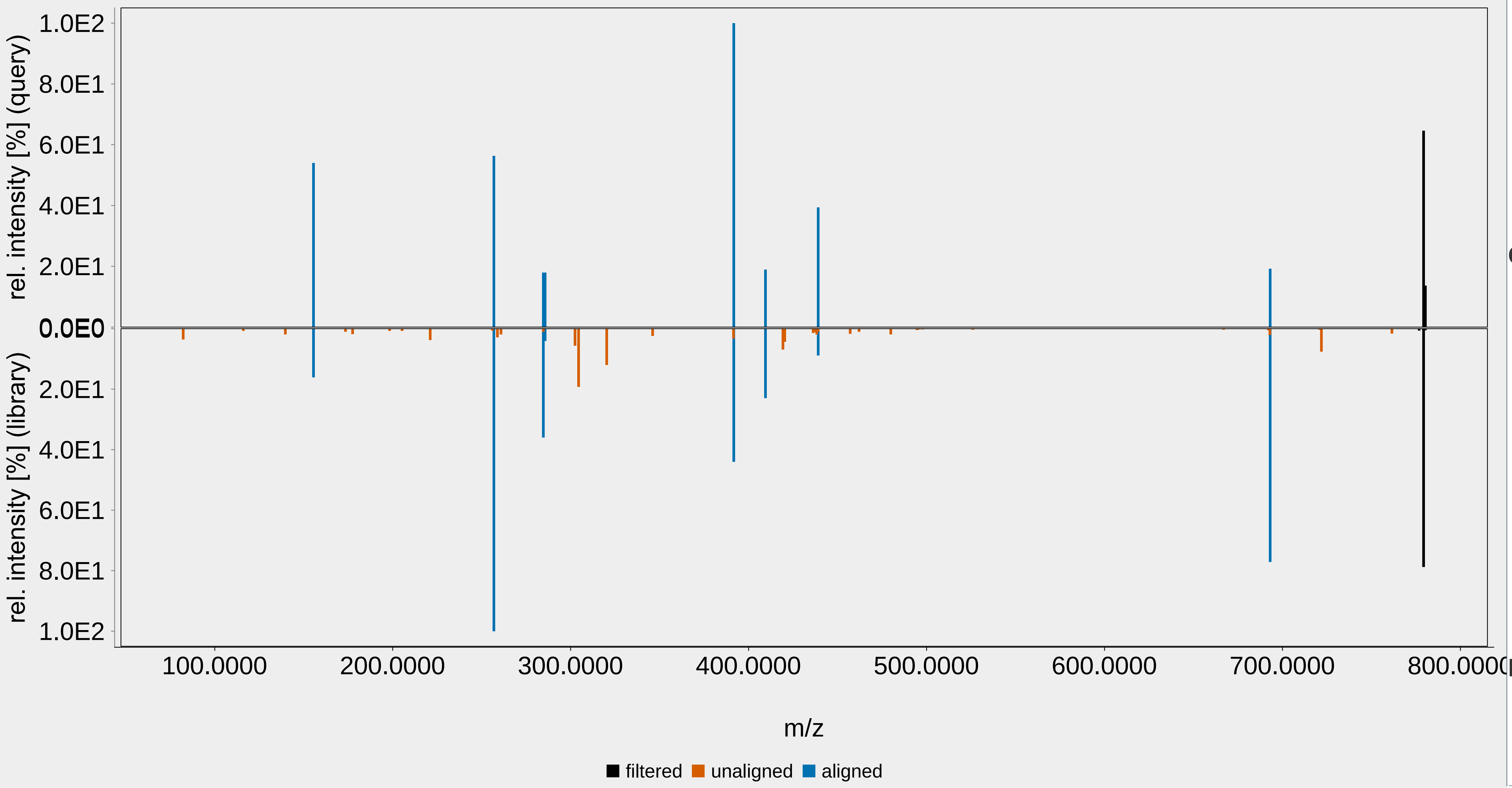

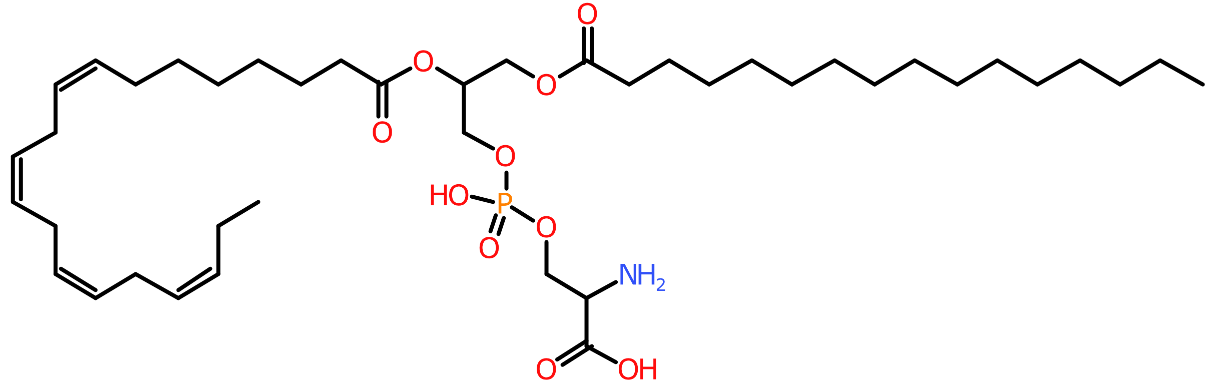

Compound information

Name: PS 36:4

Formula: C42H74NO10P

Exact mass: 784.0269999999999

Ion type: [M-H]-

Precursor m/z: 782.494

Polarity: NEGATIVE

InChI:

InChI key:

SMILES:

Num peaks: 55

Instrument information

Instrument type: LC-ESI-QTOF

Instrument: AB Sciex TripleTOF 5600+ system

Ms level: MS2

Collision energy: 45V

Database links

Other information

Entry ID: MSBNK-RIKEN_IMS-LQB00329

Comment: Parent=782.494

PS(18:0/0:0); [M-H]- C24H47N1O9P1 0.727

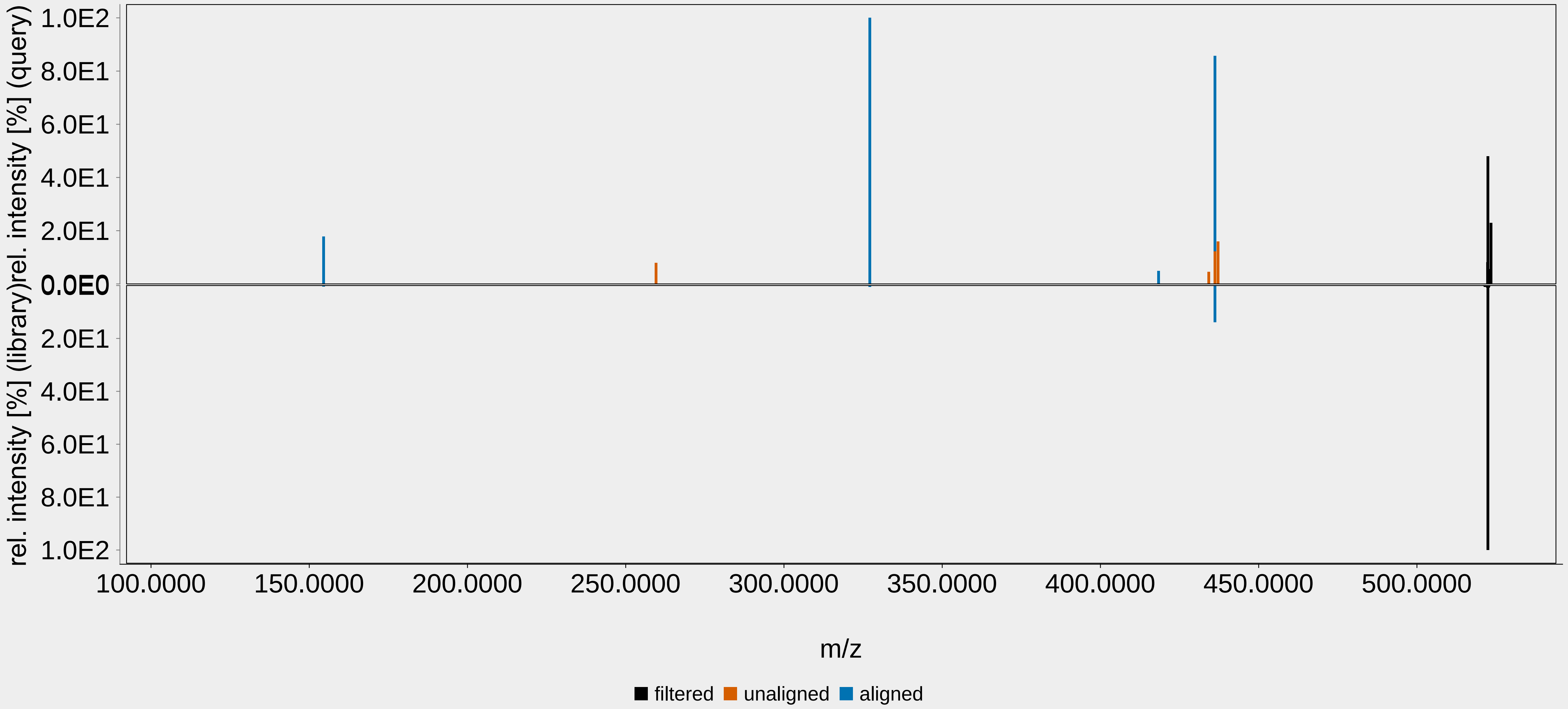

Compound information

Name: PS(18:0/0:0); [M-H]- C24H47N1O9P1

Exact mass: 0.0

Ion type: M-H

Precursor m/z: 524.299

Charge: 1

Polarity: Negative

Instrument information

Instrument type: HCD; Velos

Ion source: LC-ESI

Ms level: 2

Acquisition: Commercial

Database links

Other information

Principal investigator: Thomas Metz

Data collector: Thomas Metz

Entry ID: CCMSLIB00010074162

PE(16:1/0:0); [M-H]- C21H41N1O7P1 **0.825**

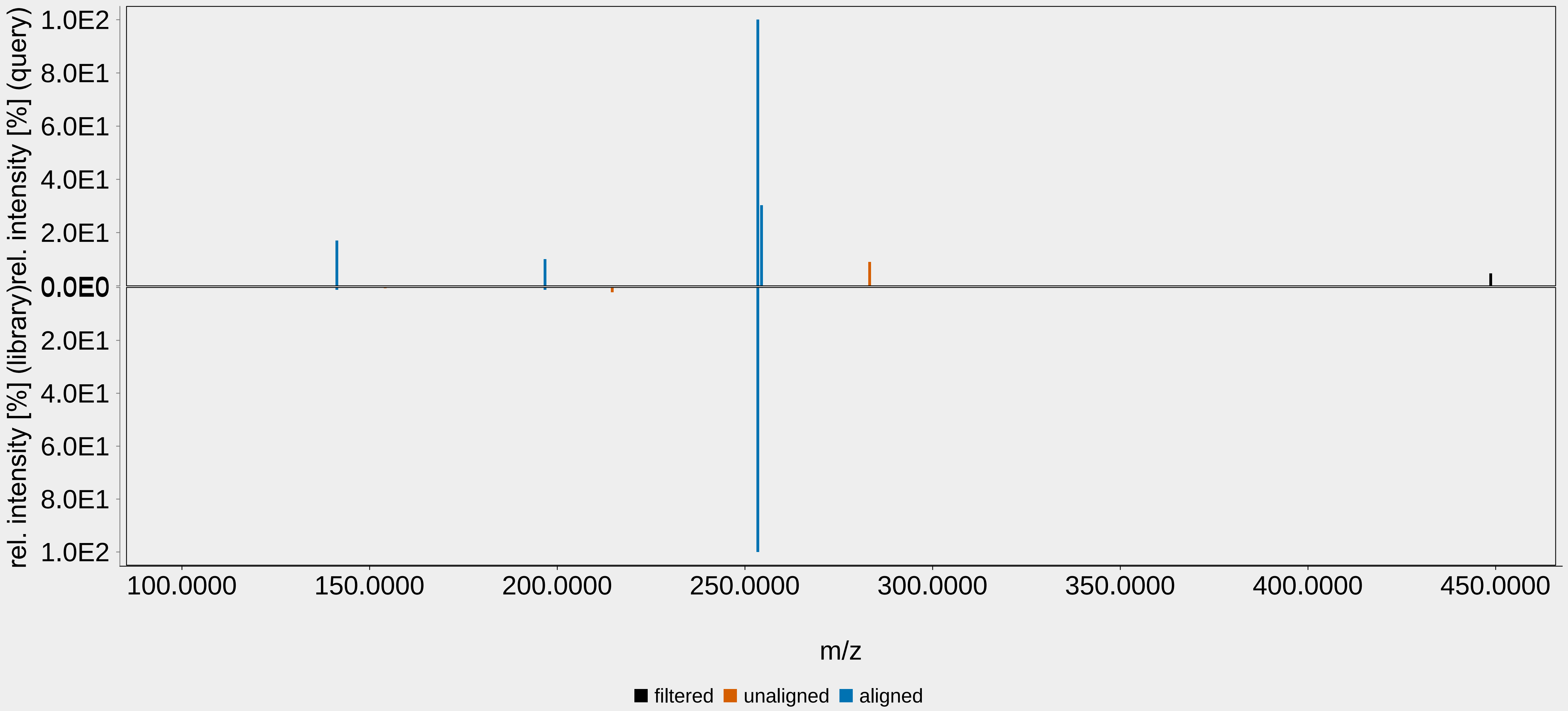

Compound information

Name: PE(16:1/0:0); [M-H]- C21H41N1O7P1

Exact mass: 0.0

Ion type: M-H

Precursor m/z: 450.262

Charge: 1

Polarity: Negative

Instrument information

Instrument type: HCD; Lumos

Ion source: LC-ESI

Ms level: 2

Acquisition: Commercial

Database links

Other information

Principal investigator: Thomas Metz

Data collector: Thomas Metz

Entry ID: CCMSLIB00010059507

PE(16:1/0:0); [M-H]- C21H41N1O7P1 0.848

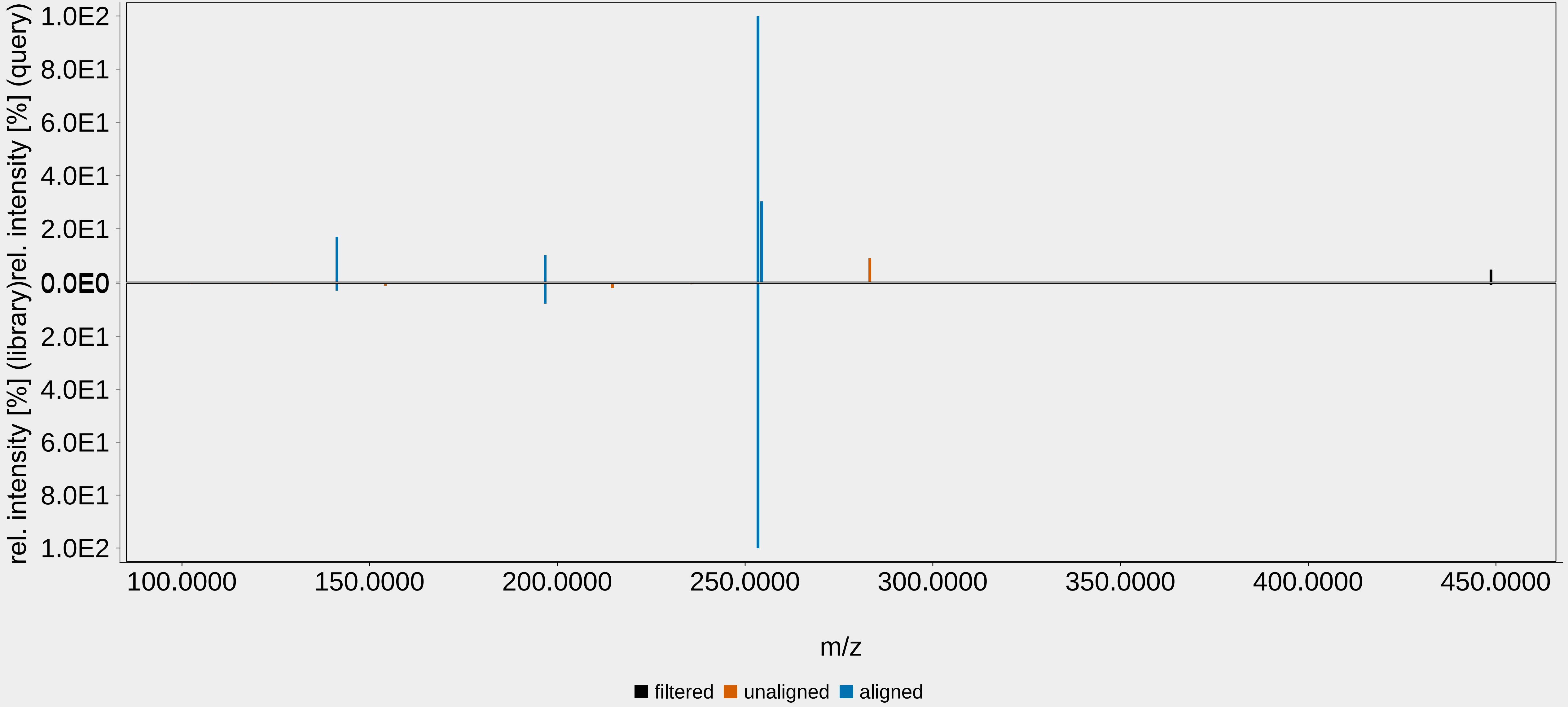

Compound information

Name: PE(16:1/0:0); [M-H]- C21H41N1O7P1

Exact mass: 0.0

Ion type: M-H

Precursor m/z: 450.262

Charge: 1

Polarity: Negative

Instrument information

Instrument type: HCD; Lumos

Ion source: LC-ESI

Ms level: 2

Acquisition: Commercial

Database links

Other information

Principal investigator: Thomas Metz

Data collector: Thomas Metz

Entry ID: CCMSLIB00010059800

PE(20:4/22:4); [M-H]- C47H77N1O8P1 0.732

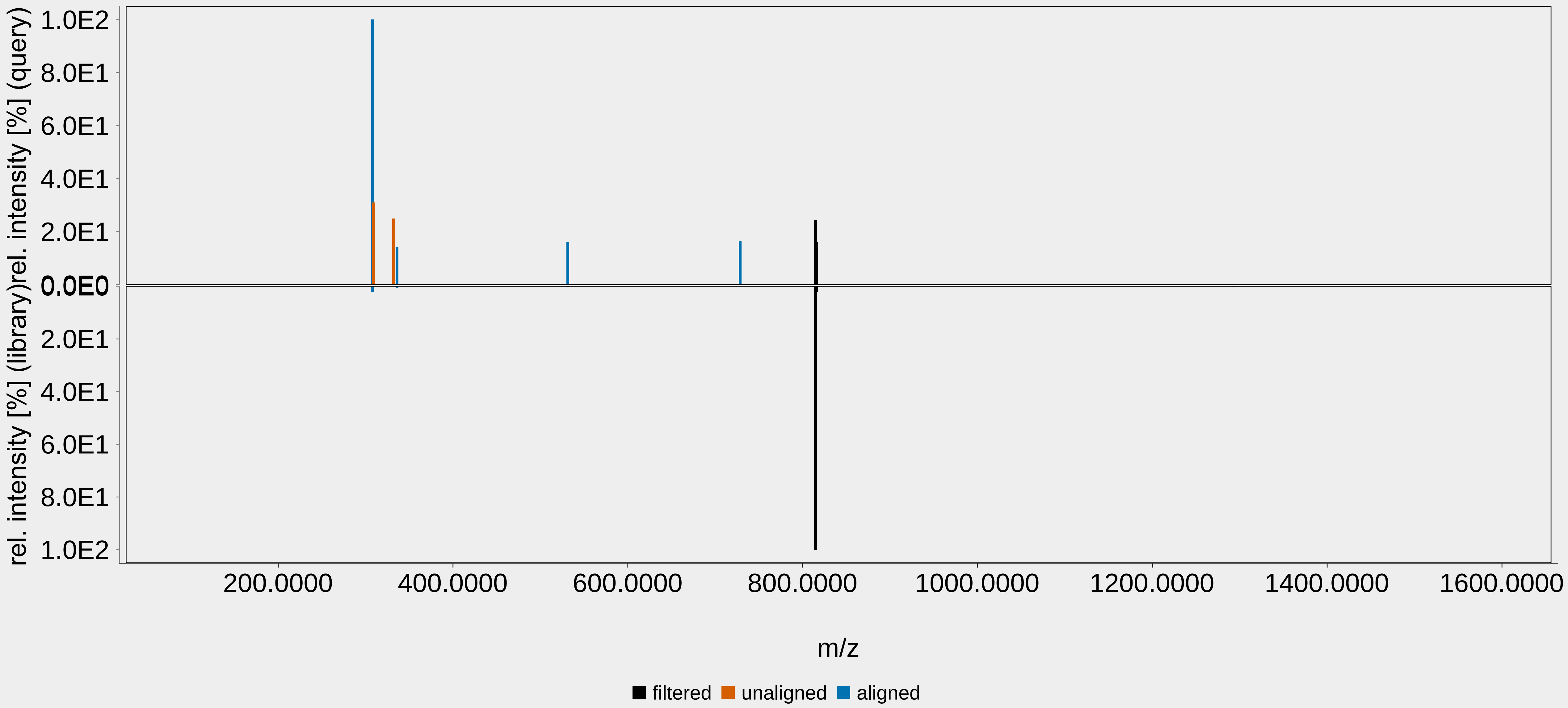

Compound information

Name: PE(20:4/22:4); [M-H]- C47H77N1O8P1

Exact mass: 0.0

Ion type: M-H

Precursor m/z: 814.539

Charge: 1

Polarity: Negative

Instrument information

Instrument type: HCD; Velos

Ion source: LC-ESI

Ms level: 2

Acquisition: Commercial

Database links

Other information

Principal investigator: Thomas Metz

Data collector: Thomas Metz

Entry ID: CCMSLIB00010063417

PE(20:4/22:4); [M-H]- C47H77N1O8P1 **0.860**

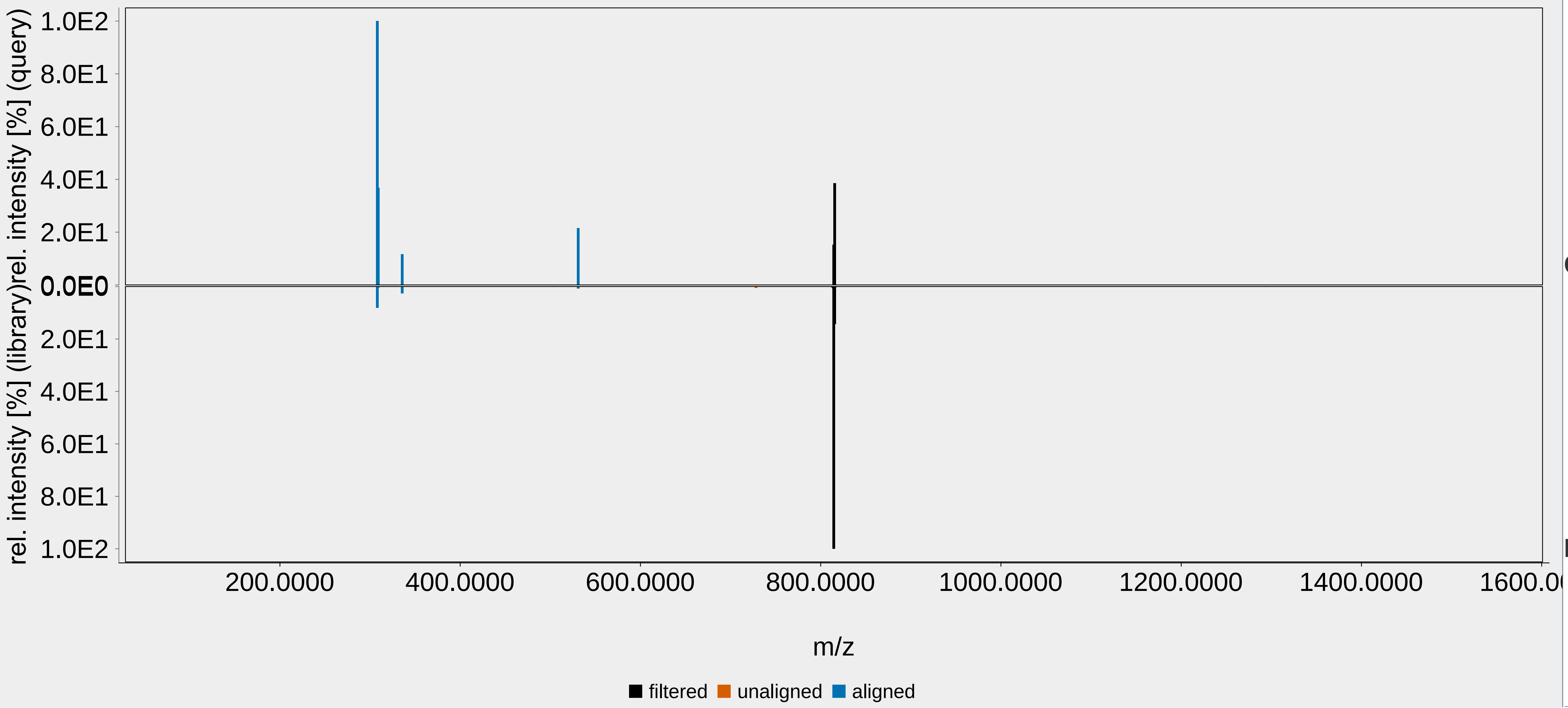

Compound information

Name: PE(20:4/22:4); [M-H]- C47H77N1O8P1

Exact mass: 0.0

Ion type: M-H

Precursor m/z: 814.539

Charge: 1

Polarity: Negative

Instrument information

Instrument type: HCD; Velos

Ion source: LC-ESI

Ms level: 2

Acquisition: Commercial

Database links

Other information

Principal investigator: Thomas Metz

Data collector: Thomas Metz

Entry ID: CCMSLIB00010087014

PA 18:0_20:3

0.742

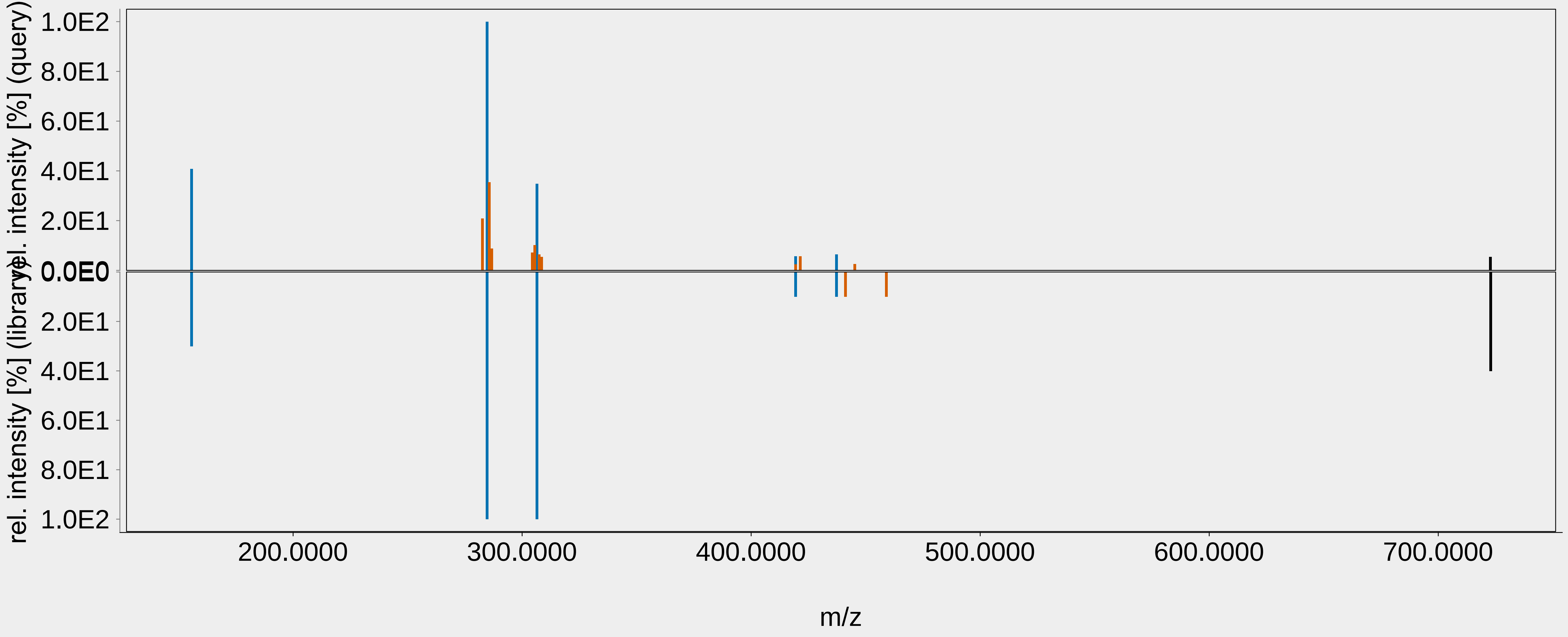

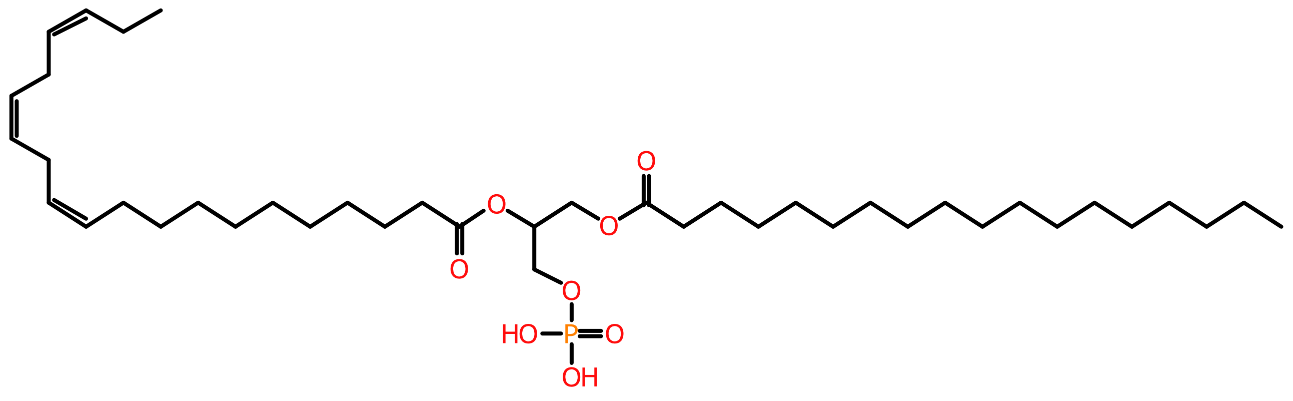

Compound information

Name: PA 18:0_20:3

Formula: C41H75O8P

Ion type: [M-H]-

Precursor m/z: 725.51268

Ccs: 263.04

InChI key: KGDFLTPILJIQMX-JTBMWNAQNA-

SMILES:

Num peaks: 8

Instrument information

Database links

Other information

PE(20:4/0:0); [M-H]- C25H43N1O7P1 **0.917**

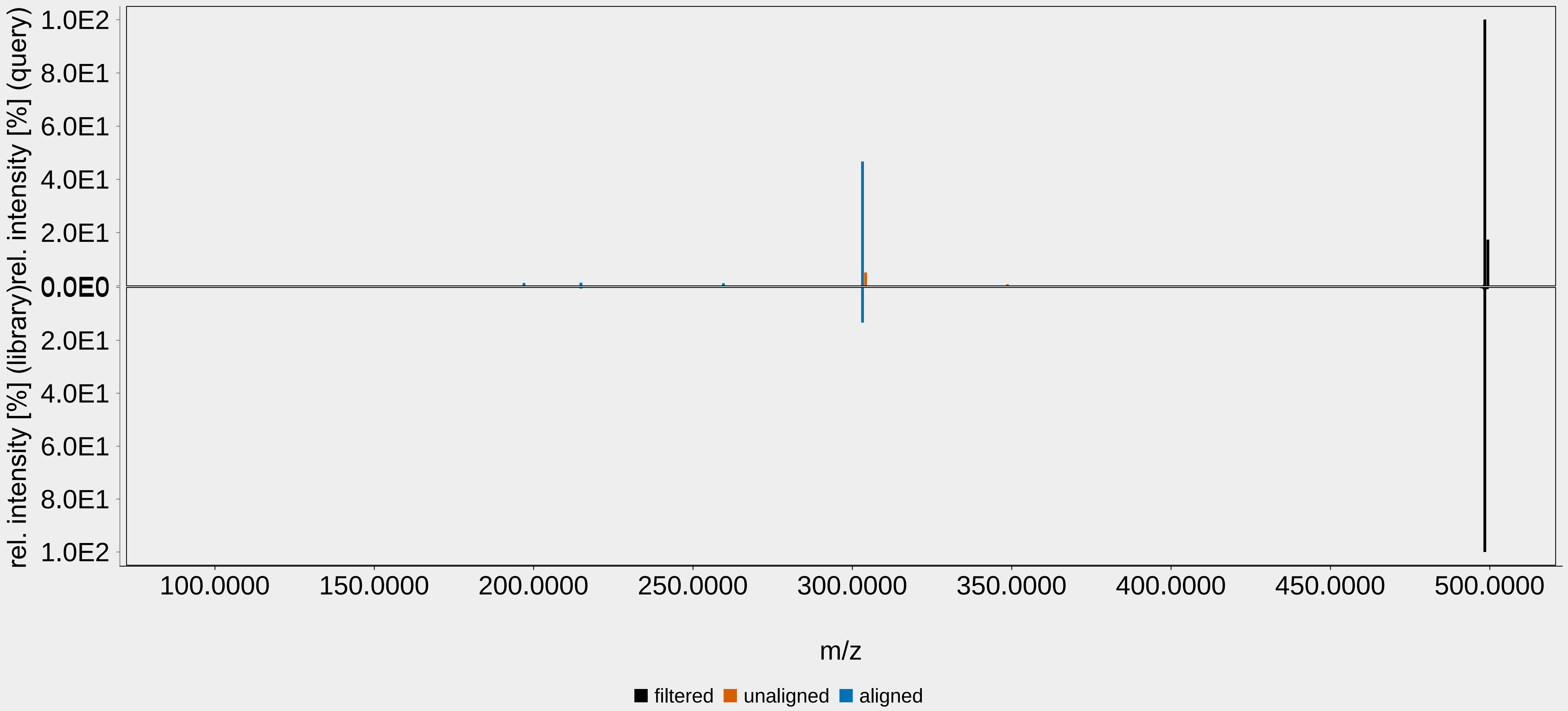

Compound information

Name: PE(20:4/0:0); [M-H]- C25H43N1O7P1

Exact mass: 0.0

Ion type: M-H

Precursor m/z: 500.278

Charge: 1

Polarity: Negative

Instrument information

Instrument type: HCD; Velos

Ion source: LC-ESI

Ms level: 2

Acquisition: Commercial

Database links

Other information

Principal investigator: Thomas Metz

Data collector: Thomas Metz

Entry ID: CCMSLIB00010067367

PE(20:4/0:0); [M-H]- C25H43N1O7P1

0.921

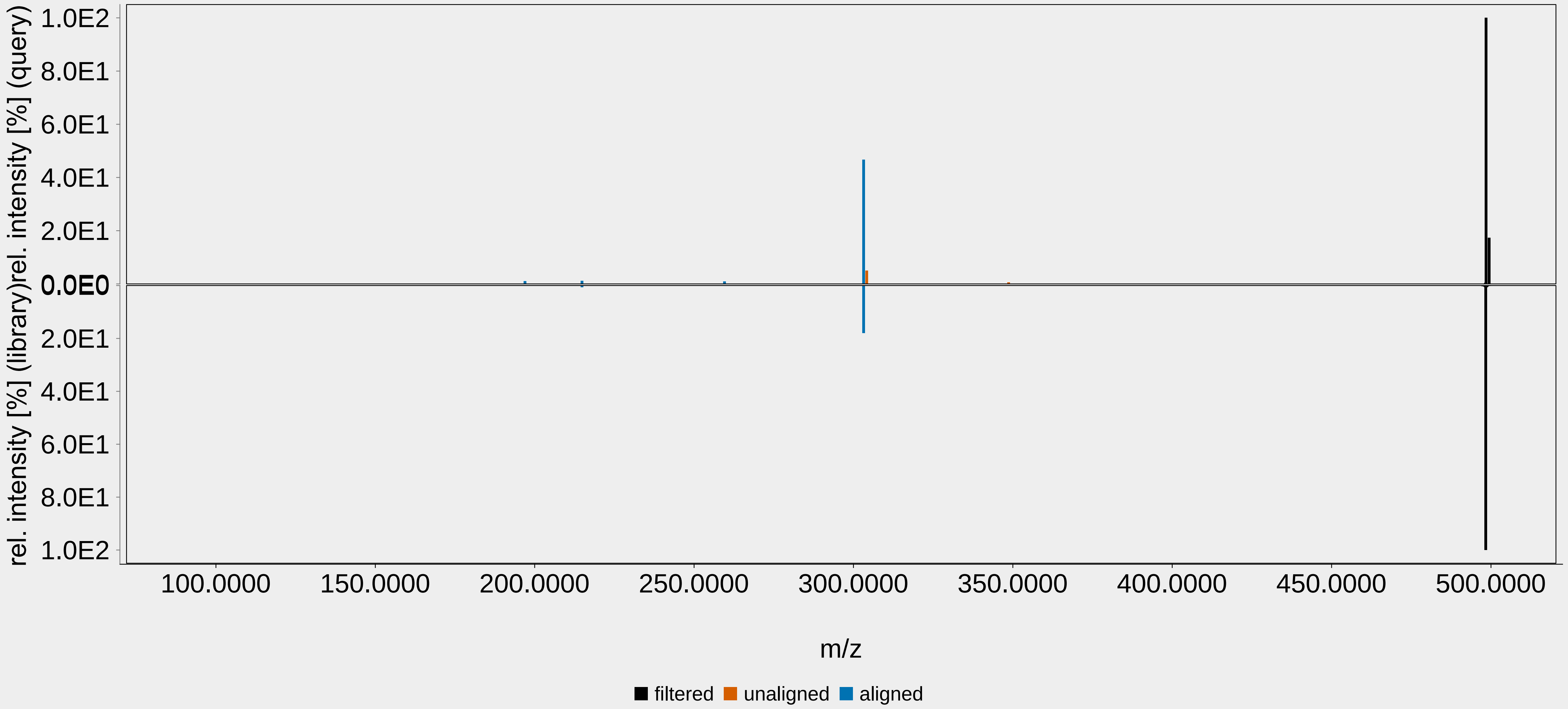

Compound information

Name: PE(20:4/0:0); [M-H]- C25H43N1O7P1

Exact mass: 0.0

Ion type: M-H

Precursor m/z: 500.278

Charge: 1

Polarity: Negative

Instrument information

Instrument type: HCD; Velos

Ion source: LC-ESI

Ms level: 2

Acquisition: Commercial

Database links

Other information

Principal investigator: Thomas Metz

Data collector: Thomas Metz

Entry ID: CCMSLIB00010085243

Dodecylbenzenesulfonic acid

0.965

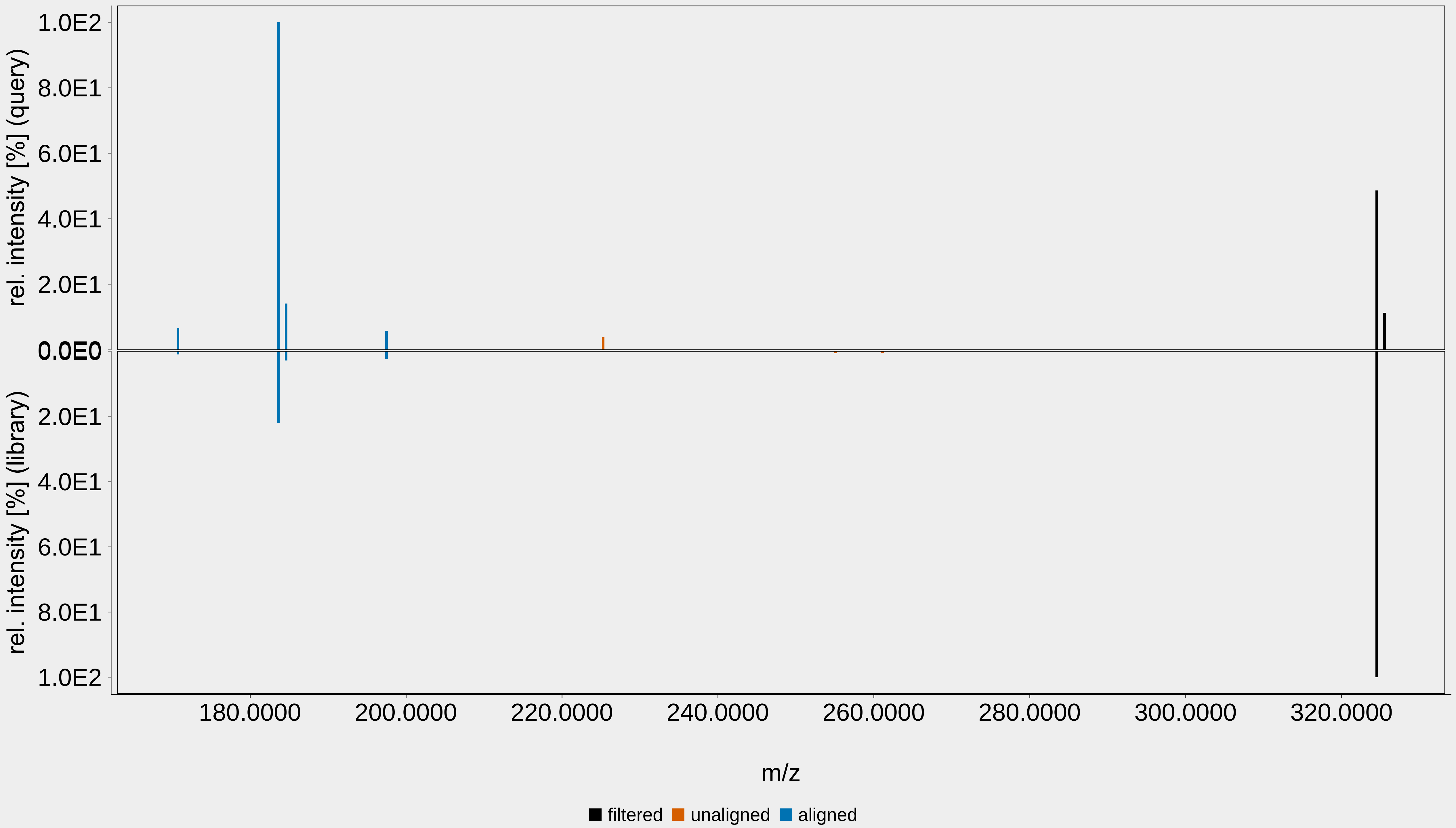

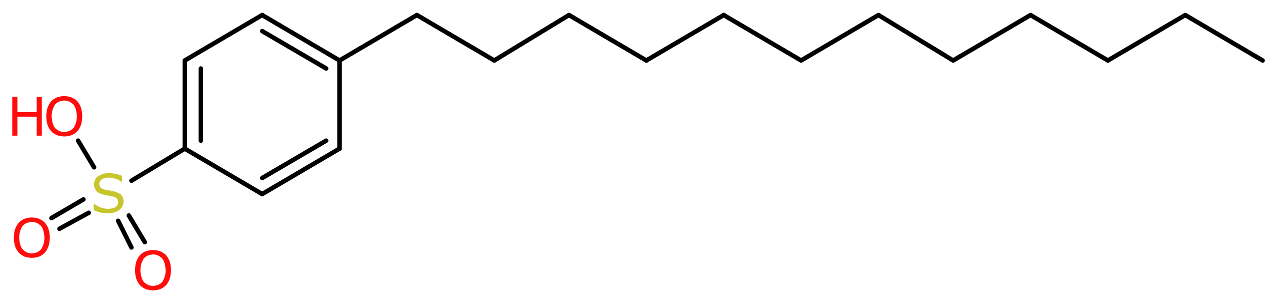

Compound information

Name: Dodecylbenzenesulfonic acid

Formula: C18H30O3S

Mol. weight: 326.19157

Exact mass: 326.19157

Ion type: [M-H]-

Precursor m/z: 325.1843

Polarity: negative

InChI:

InChI key:

SMILES:

Instrument information

Instrument type: LC-ESI-QFT

Instrument: Q Exactive Plus Orbitrap Thermo

Ion source: ESI

Resolution: 35000

Ms level: MS2

Collision energy: 35 (nominal)

Database links

Pubchem: 8485

MoNA ID: SM876251

Chemspider: 8172

CAS: 121-65-3

Other information

Principal investigator: Krauss M, Schymanski

Data collector: Krauss M, Schymanski EL,

Dodecylbenzenesulfonic acid

0.966

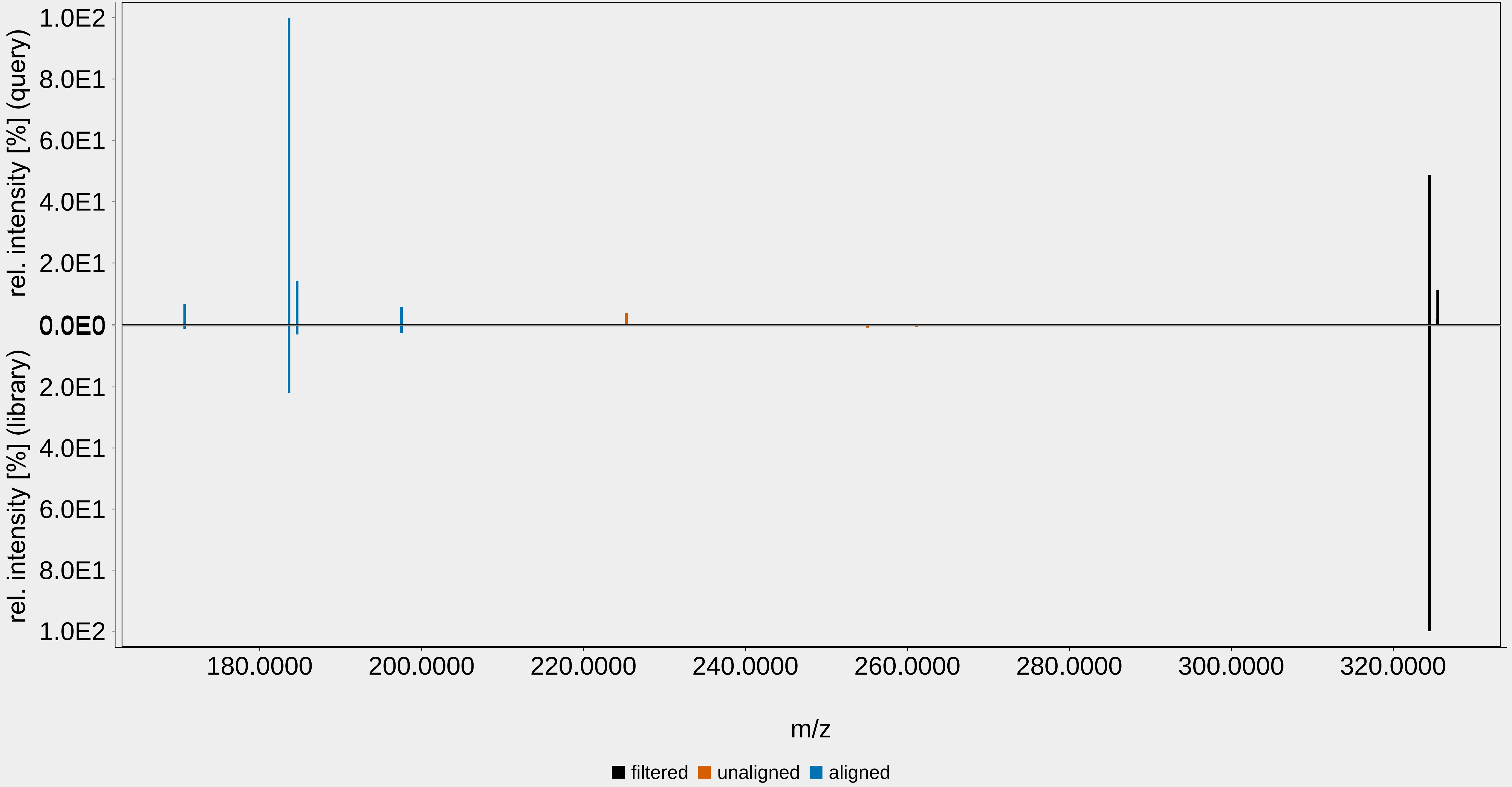

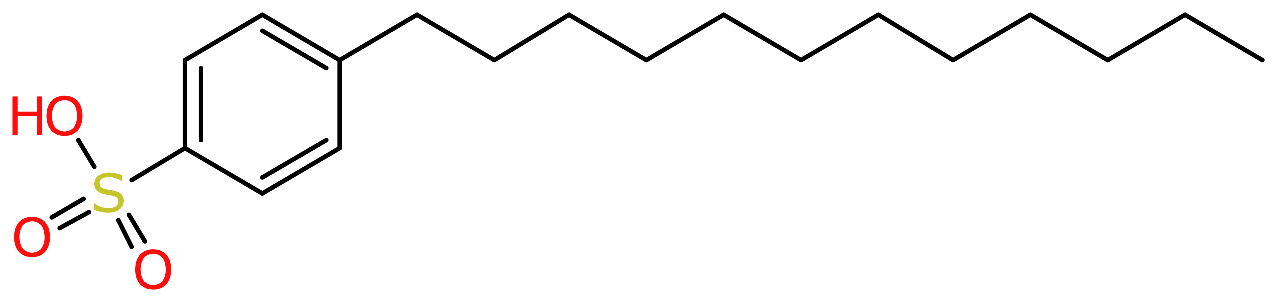

Compound information

Name: Dodecylbenzenesulfonic acid

Formula: C18H30O3S

Exact mass: 326.19157

Ion type: [M-H]-

Precursor m/z: 325.1843

Polarity: NEGATIVE

InChI:

InChI key:

SMILES:

Num peaks: 7

Instrument information

Instrument type: LC-ESI-QFT

Instrument: Q Exactive Plus Orbitrap Thermo

Ms level: MS2

Collision energy: 35 (nominal)

Database links

Other information

Entry ID: MSBNK-CASMI_2016-SM876251

Comment: Parent=325.1843

PS(16:0/18:1); [M-H]- C40H75N1O10P1 0.938

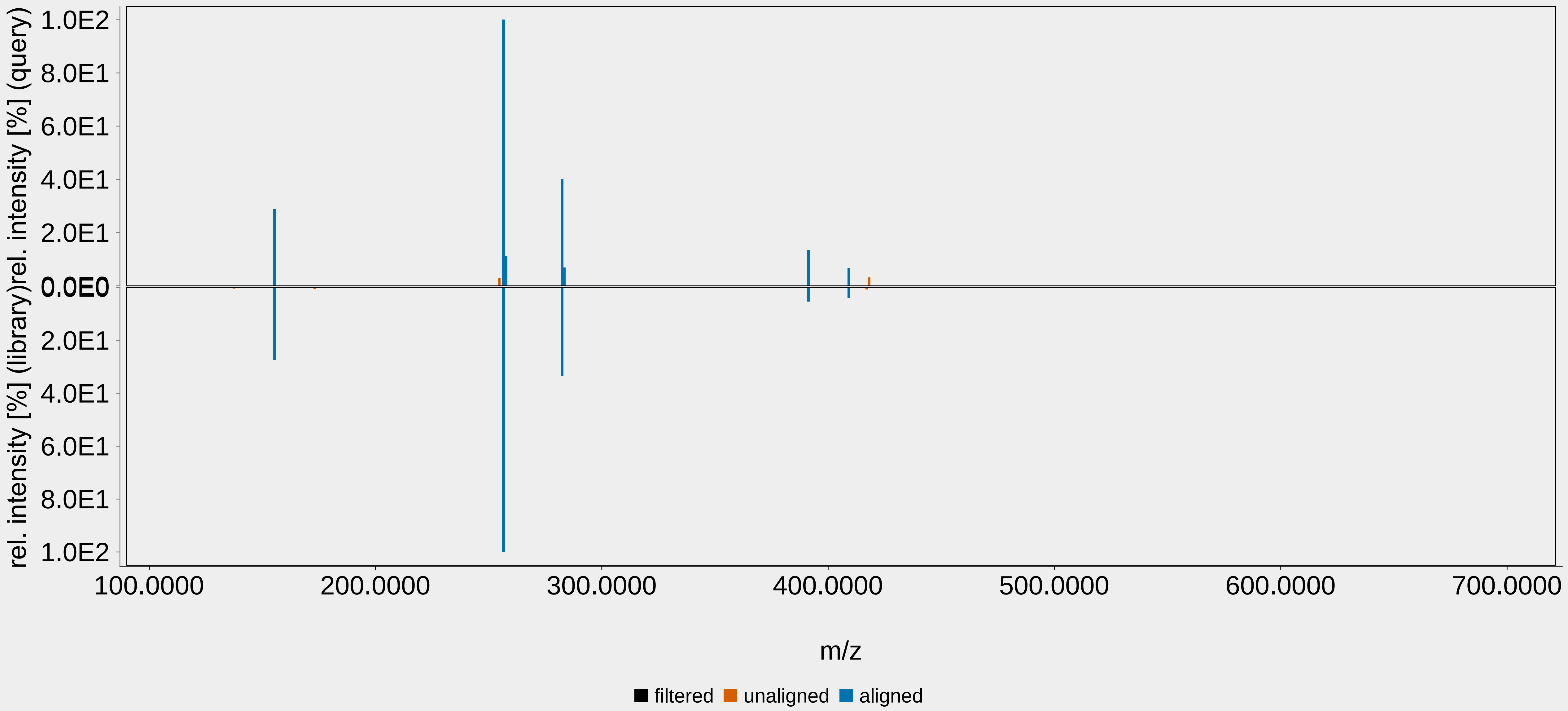

Compound information

Name: PS(16:0/18:1); [M-H]- C40H75N1O10P1

Exact mass: 0.0

Ion type: M-H

Precursor m/z: 760.513

Charge: 1

Polarity: Negative

Instrument information

Instrument type: HCD; Lumos

Ion source: LC-ESI

Ms level: 2

Acquisition: Commercial

Database links

Other information

Principal investigator: Thomas Metz

Data collector: Thomas Metz

Entry ID: CCMSLIB00010059839

PS(16:0/18:1); [M-H]- C40H75N1O10P1 **0.938**

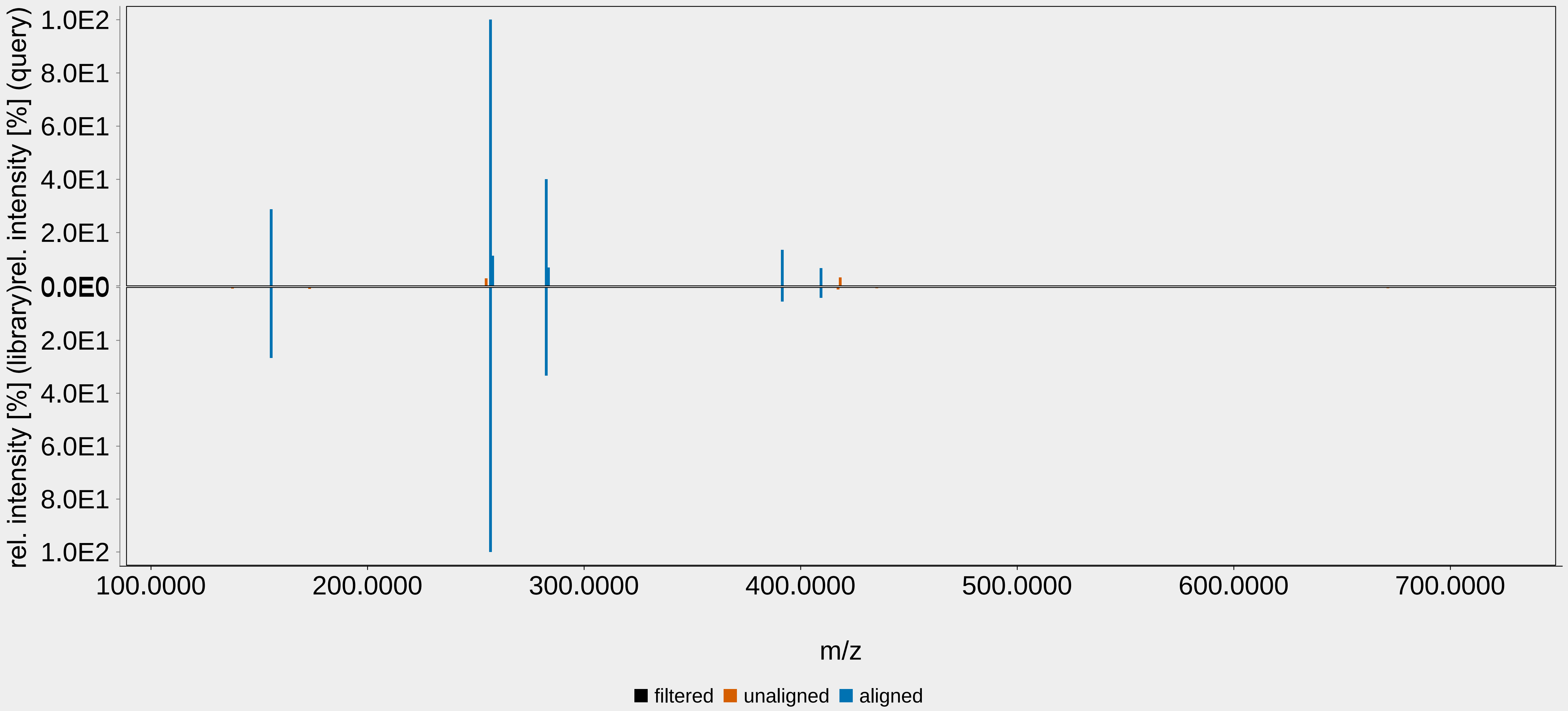

Compound information

Name: PS(16:0/18:1); [M-H]- C40H75N1O10P1

Exact mass: 0.0

Ion type: M-H

Precursor m/z: 760.513

Charge: 1

Polarity: Negative

Instrument information

Instrument type: HCD; Lumos

Ion source: LC-ESI

Ms level: 2

Acquisition: Commercial

Database links

Other information

Principal investigator: Thomas Metz

Data collector: Thomas Metz

Entry ID: CCMSLIB00010059457

PI 36:1 **0.730**

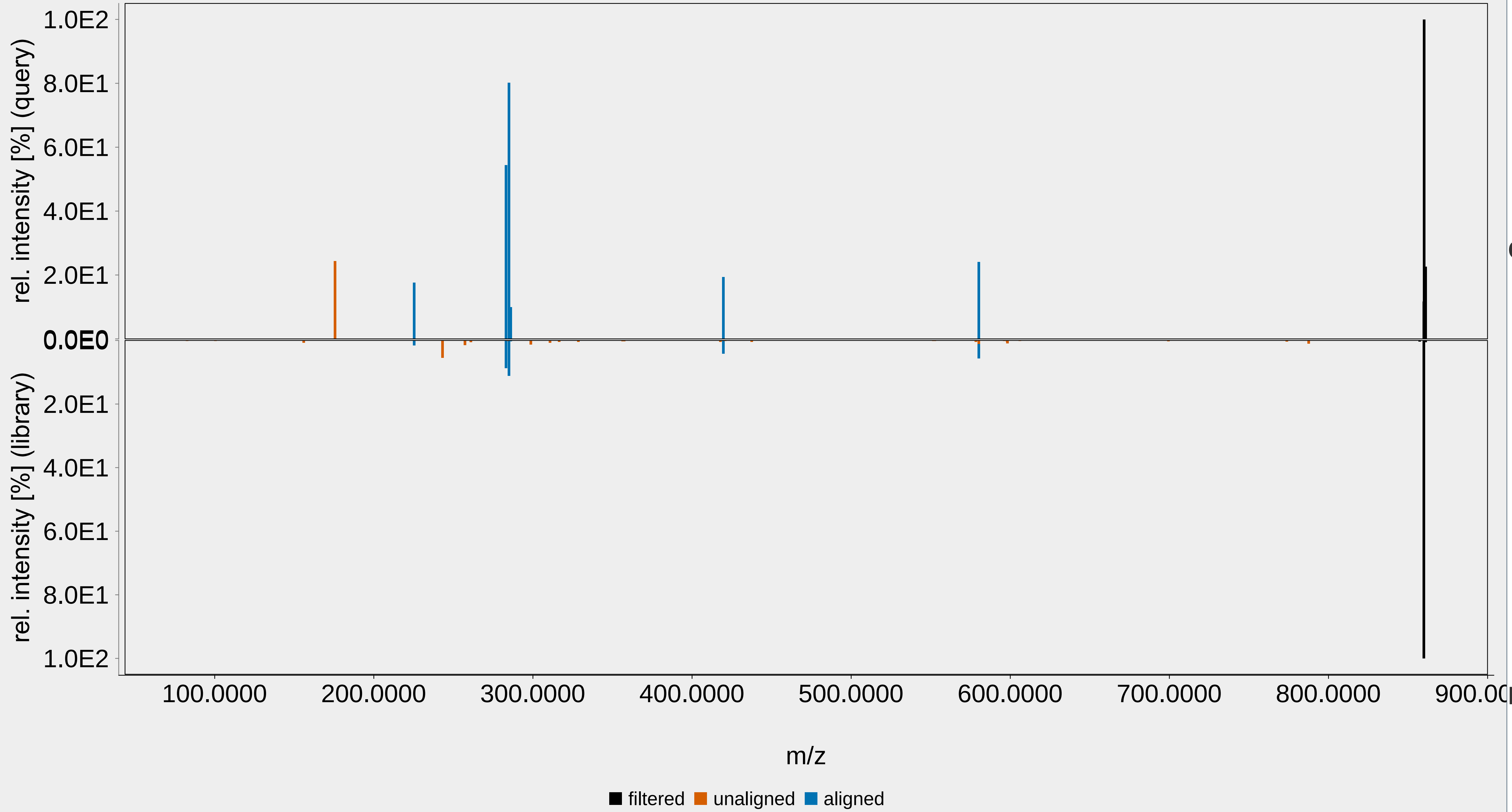

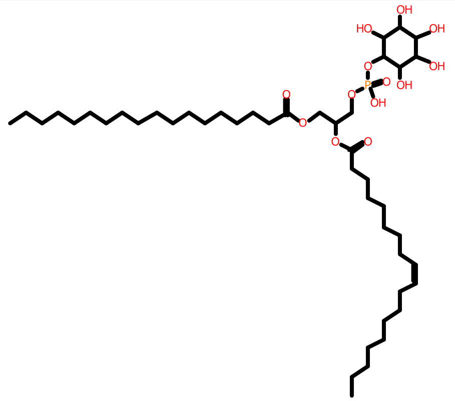

Compound information

Name: PI 36:1

Formula: C45H85O13P

Mol. weight: 865.1379999999999

Exact mass: 865.1379999999999

Ion type: [M-H]-

Precursor m/z: 863.5657

RT: 9.13166

Polarity: negative

InChI:

InChI key: YOBFISPJJWPPTK-ZZEZOPTASA-I

SMILES:

Instrument information

Instrument type: LC-ESI-QTOF

Instrument: AB Sciex TripleTOF 5600+ system

Ms level: MS2

Collision energy: 45V

Database links

Pubchem: 138306742

MoNA ID: LQB00691

Other information

Principal investigator: Kazutaka Ikeda, Hiroshi

Data collector: Kazutaka Ikeda, Hiroshi

PI 36:1 **0.730**

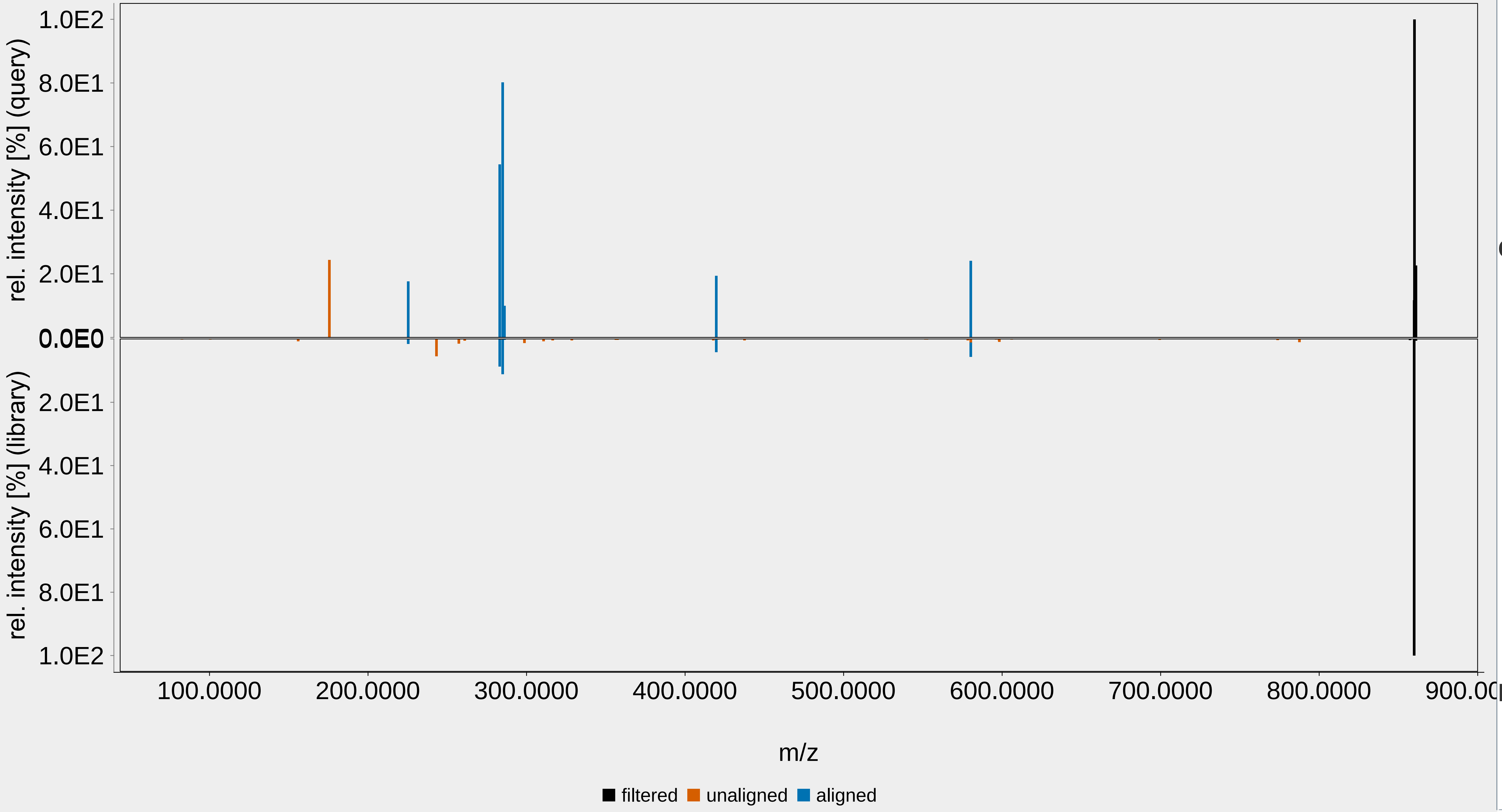

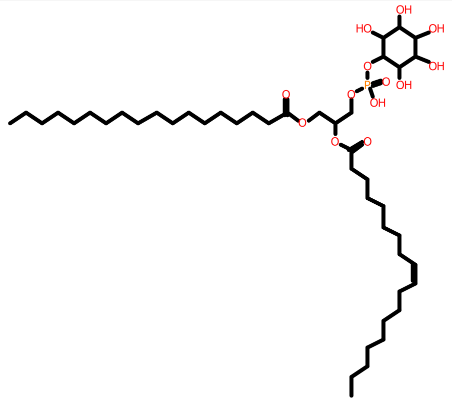

Compound information

Name: PI 36:1

Formula: C45H85O13P

Mol. weight: 865.1379999999999

Exact mass: 865.1379999999999

Ion type: [M-H]-

Precursor m/z: 863.5657

RT: 9.13166

Polarity: negative

InChI:

InChI key: YOBFISPJJWPPTK-ZZEZOPTASA-I

SMILES:

Instrument information

Instrument type: LC-ESI-QTOF

Instrument: AB Sciex TripleTOF 5600+ system

Ms level: MS2

Collision energy: 45V

Database links

Pubchem: 138306742

MoNA ID: LQB00305

Other information

Principal investigator: Kazutaka Ikeda, Hiroshi

Data collector: Kazutaka Ikeda, Hiroshi

PA 16:0_18:0

0.850

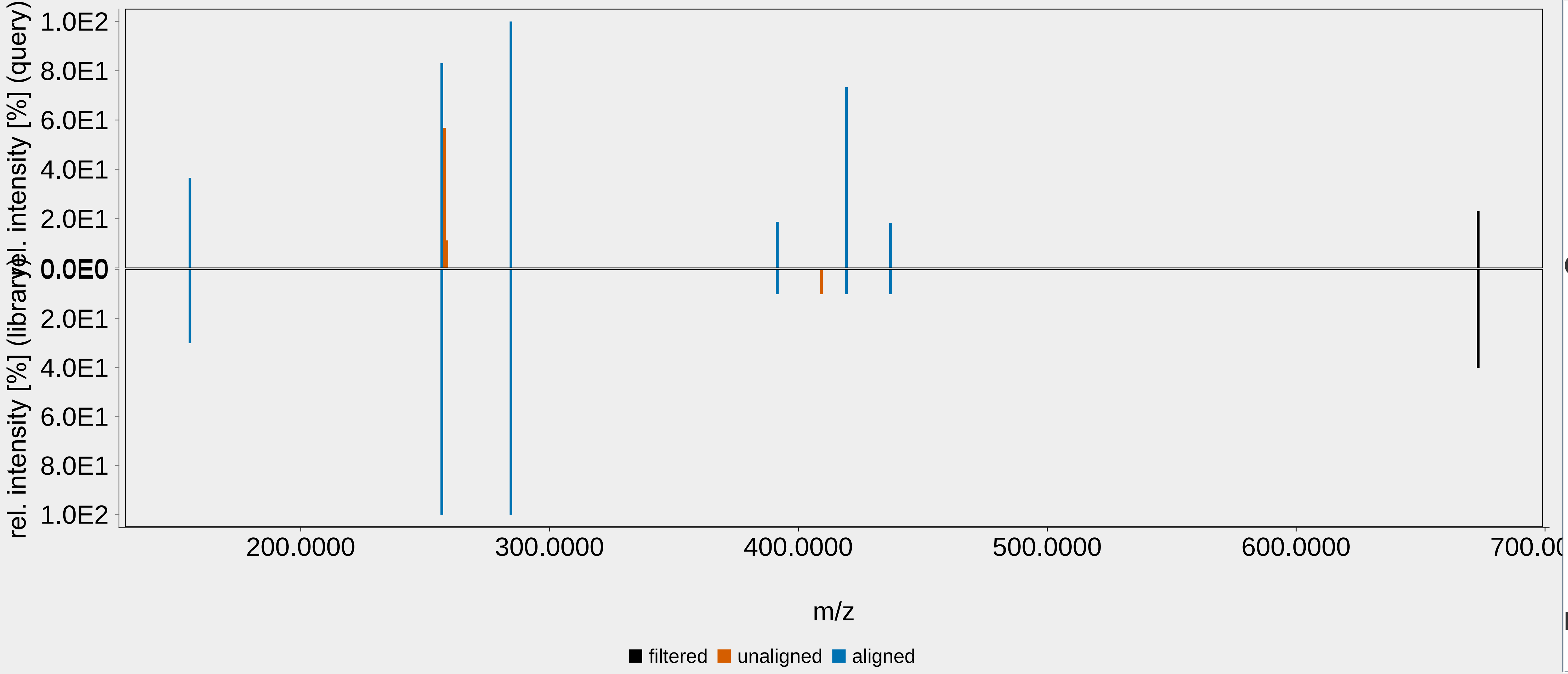

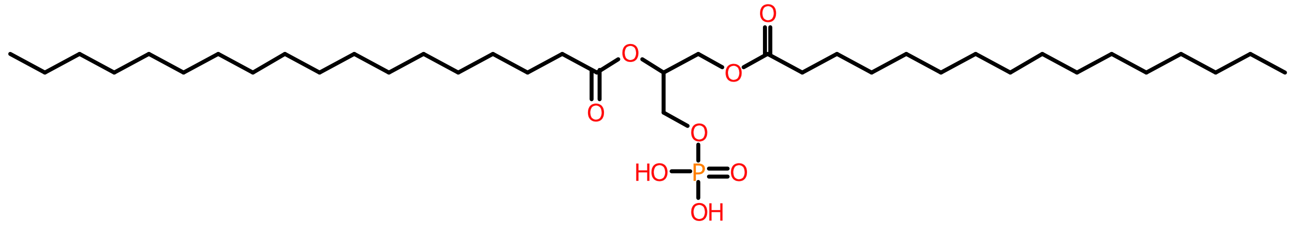

Compound information

Name: PA 16:0_18:0

Formula: C37H73O8P

Ion type: [M-H]-

Precursor m/z: 675.49703

Ccs: 256.25

InChI key: GASPGIDKILJBMM-UHFFFAOYNA-

SMILES:

Num peaks: 8

Instrument information

Database links

Other information

PA(16:1/18:1); [M-H]- C37H68O8P1 0.788

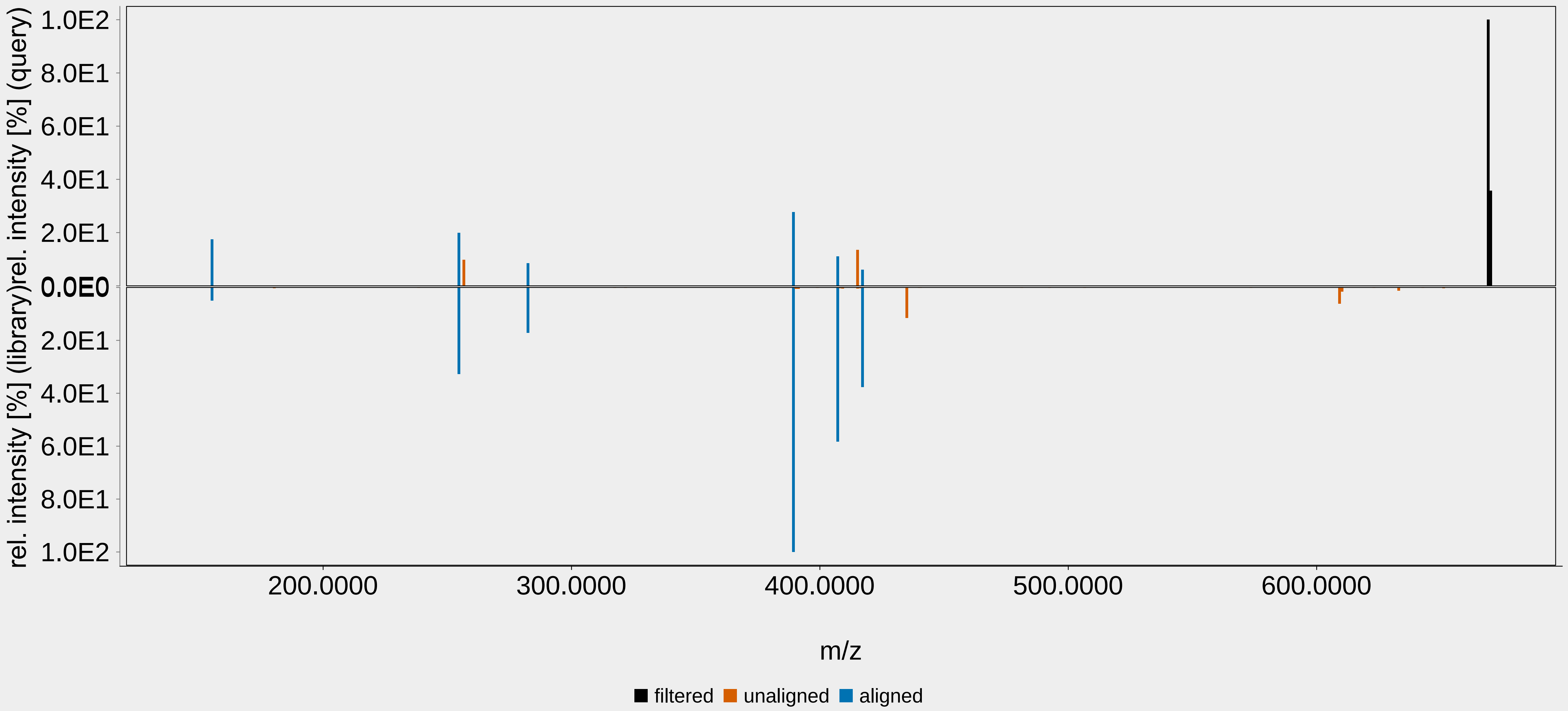

Compound information

Name: PA(16:1/18:1); [M-H]- C37H68O8P1

Exact mass: 0.0

Ion type: M-H

Precursor m/z: 671.465

Charge: 1

Polarity: Negative

Instrument information

Instrument type: CID; Lumos

Ion source: LC-ESI

Ms level: 2

Acquisition: Commercial

Database links

Other information

Principal investigator: Thomas Metz

Data collector: Thomas Metz

Entry ID: CCMSLIB00010059155

PA(16:1/18:1); [M-H]- C37H68O8P1

0.913

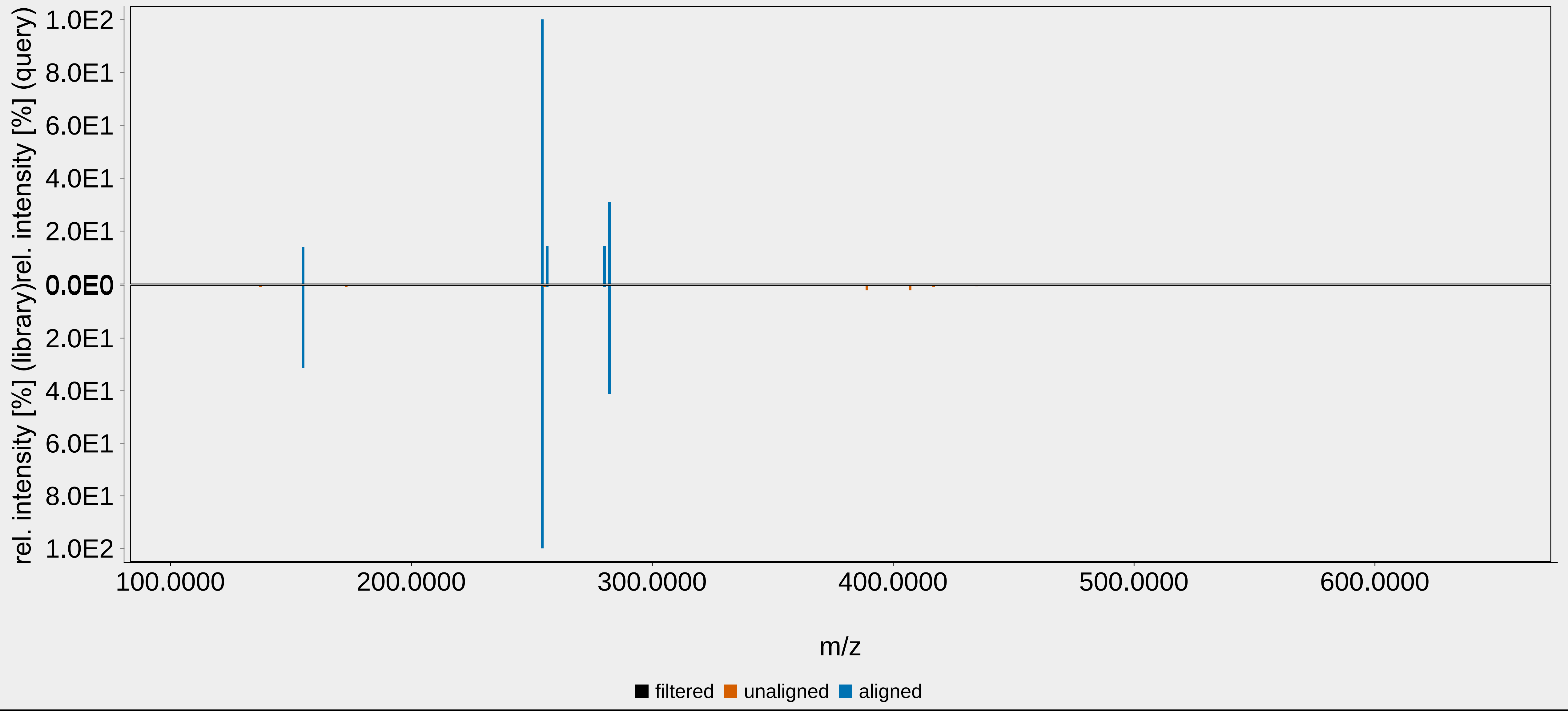

Compound information

Name: PA(16:1/18:1); [M-H]- C37H68O8P1

Exact mass: 0.0

Ion type: M-H

Precursor m/z: 671.465

Charge: 1

Polarity: Negative

Instrument information

Instrument type: HCD; Lumos

Ion source: LC-ESI

Ms level: 2

Acquisition: Commercial

Database links

Other information

Principal investigator: Thomas Metz

Data collector: Thomas Metz

Entry ID: CCMSLIB00010059156

ADENOSINE 5'-TRIPHOSPHATE 0.743

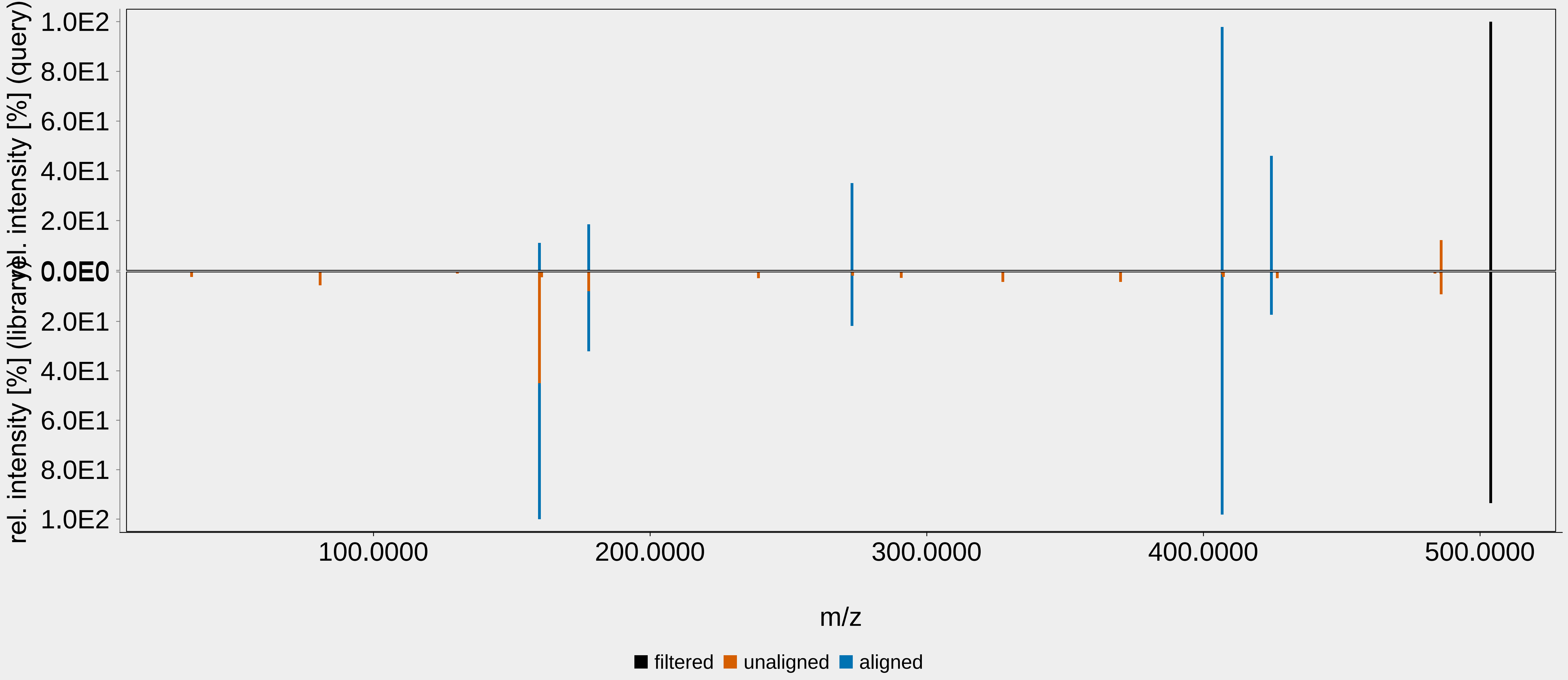

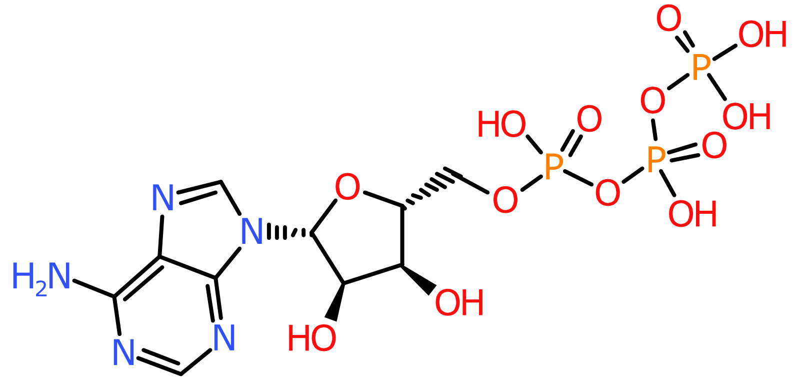

Compound information

Name: ADENOSINE 5'-TRIPHOSPHATE

Formula: C10H16N5O13P3

Ion type: [M-H]-

Precursor m/z: 505.9884695

Polarity: negative

InChI:

InChI key:

SMILES:

Instrument information

Instrument type: LC-ESI-QTOF

Instrument: Agilent qTOF 6545

Ms level: MS2

Collision energy: 20 eV

Database links

Other information

ADENOSINE 5'-TRIPHOSPHATE

0.743

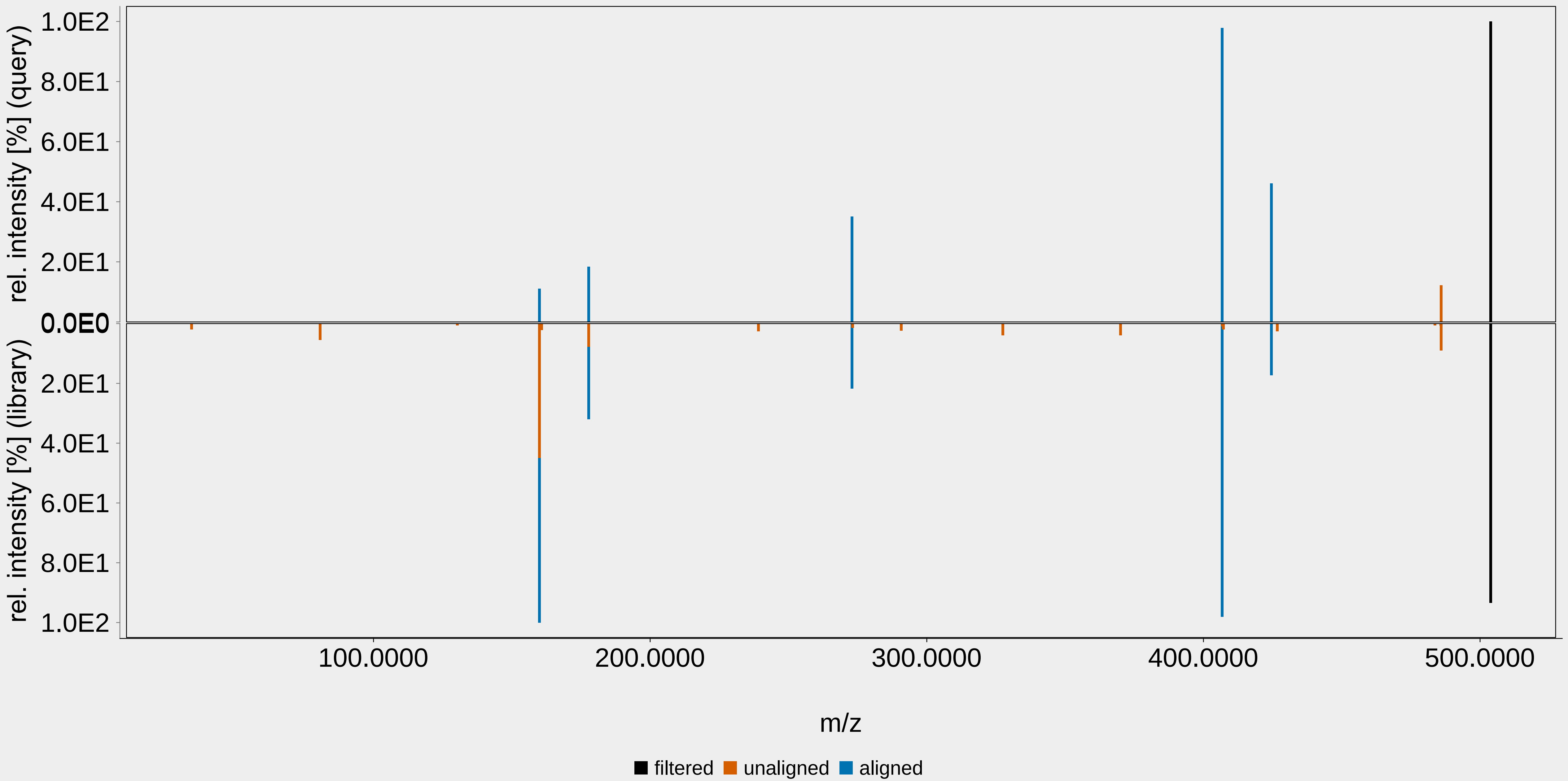

Compound information

Name: ADENOSINE 5'-TRIPHOSPHATE

Exact mass: 0.0

Ion type: M-H

Precursor m/z: 505.988

Charge: 1

Polarity: negative

InChI:

SMILES:

Instrument information

Instrument type: qTof

Ion source: ESI

Ms level: 2

Acquisition: isolated

Database links

Other information

Principal investigator: MoNA

Data collector: MoNA:MoNA034214

Entry ID: CCMSLIB00006685072

PA 18:1_18:2 **0.890**

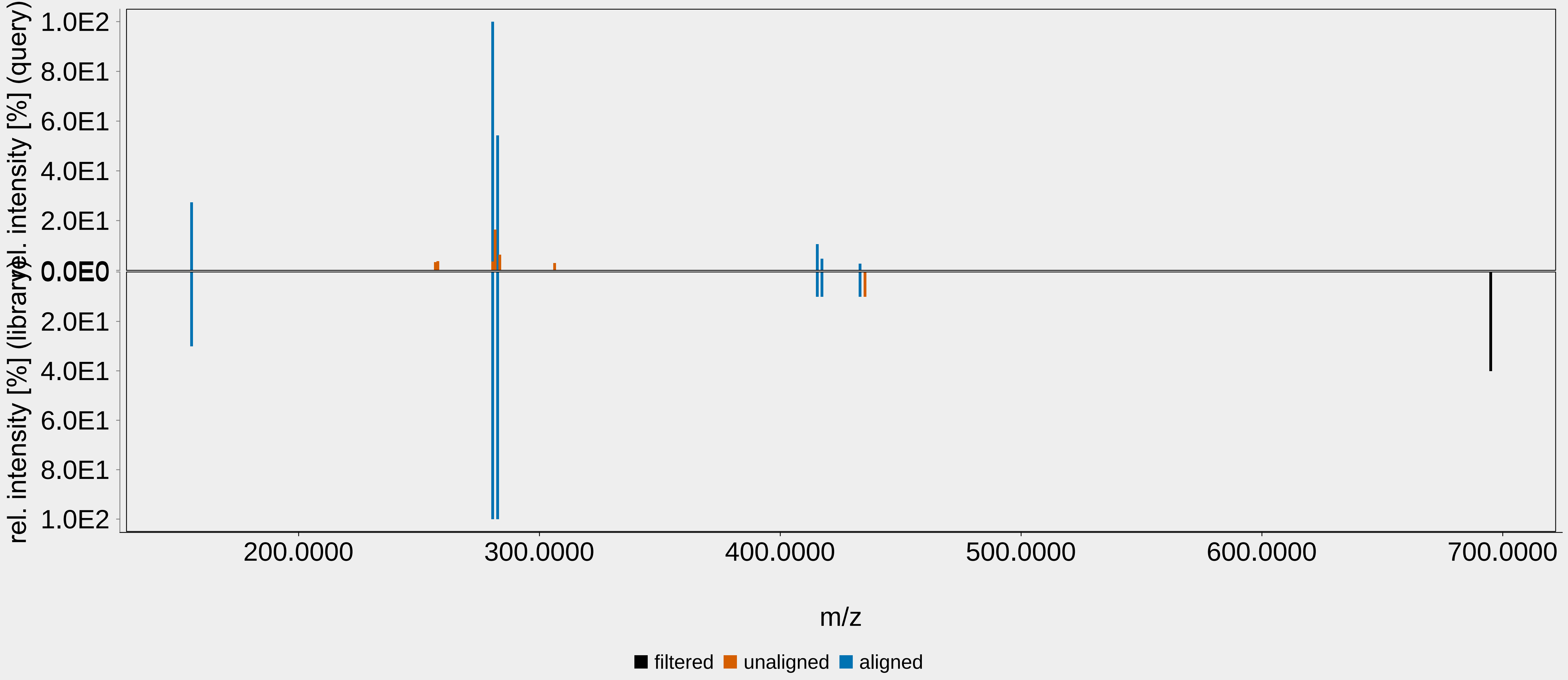

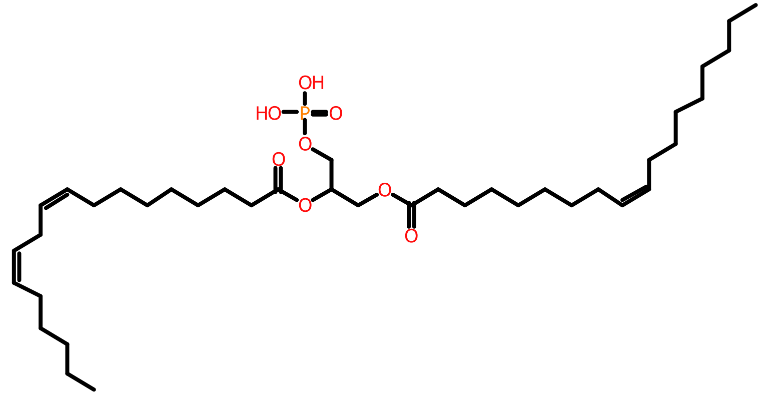

Compound information

Name: PA 18:1_18:2

Formula: C39H71O8P

Ion type: [M-H]-

Precursor m/z: 697.48138

Ccs: 255.37

InChI key:

SMILES:

Num peaks: 8

Instrument information

Database links

Other information

PE(18:1/18:1); [M-H]- C41H77N1O8P1 **0.941**

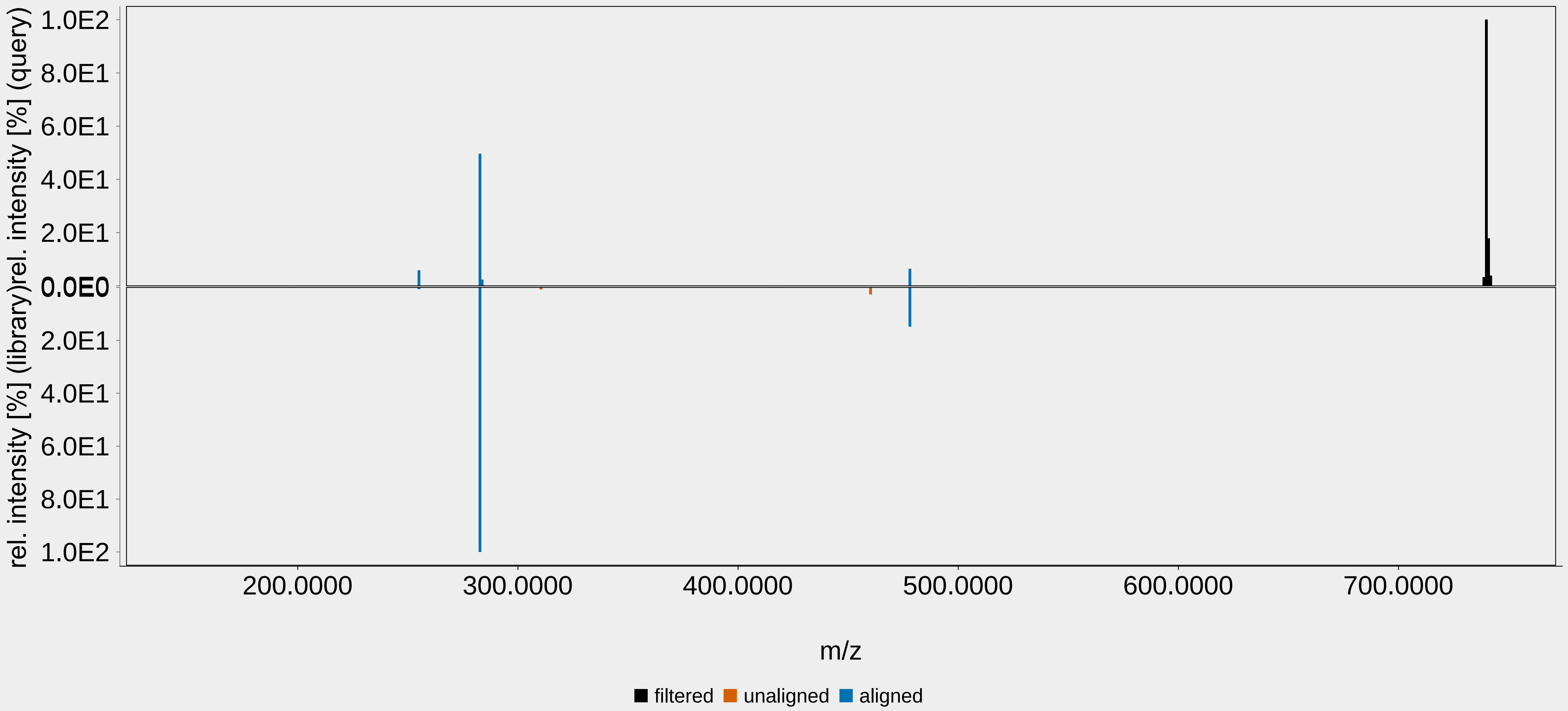

Compound information

Name: PE(18:1/18:1); [M-H]- C41H77N1O8P1

Exact mass: 0.0

Ion type: M-H

Precursor m/z: 742.539

Charge: 1

Polarity: Negative

Instrument information

Instrument type: CID; Lumos

Ion source: LC-ESI

Ms level: 2

Acquisition: Commercial

Database links

Other information

Principal investigator: Thomas Metz

Data collector: Thomas Metz

Entry ID: CCMSLIB00010059182

Spectral Match to 1-Stearoyl-2-linoleoyl-sn-glycero-3-phosphoethanolamine from NIST14

0.957

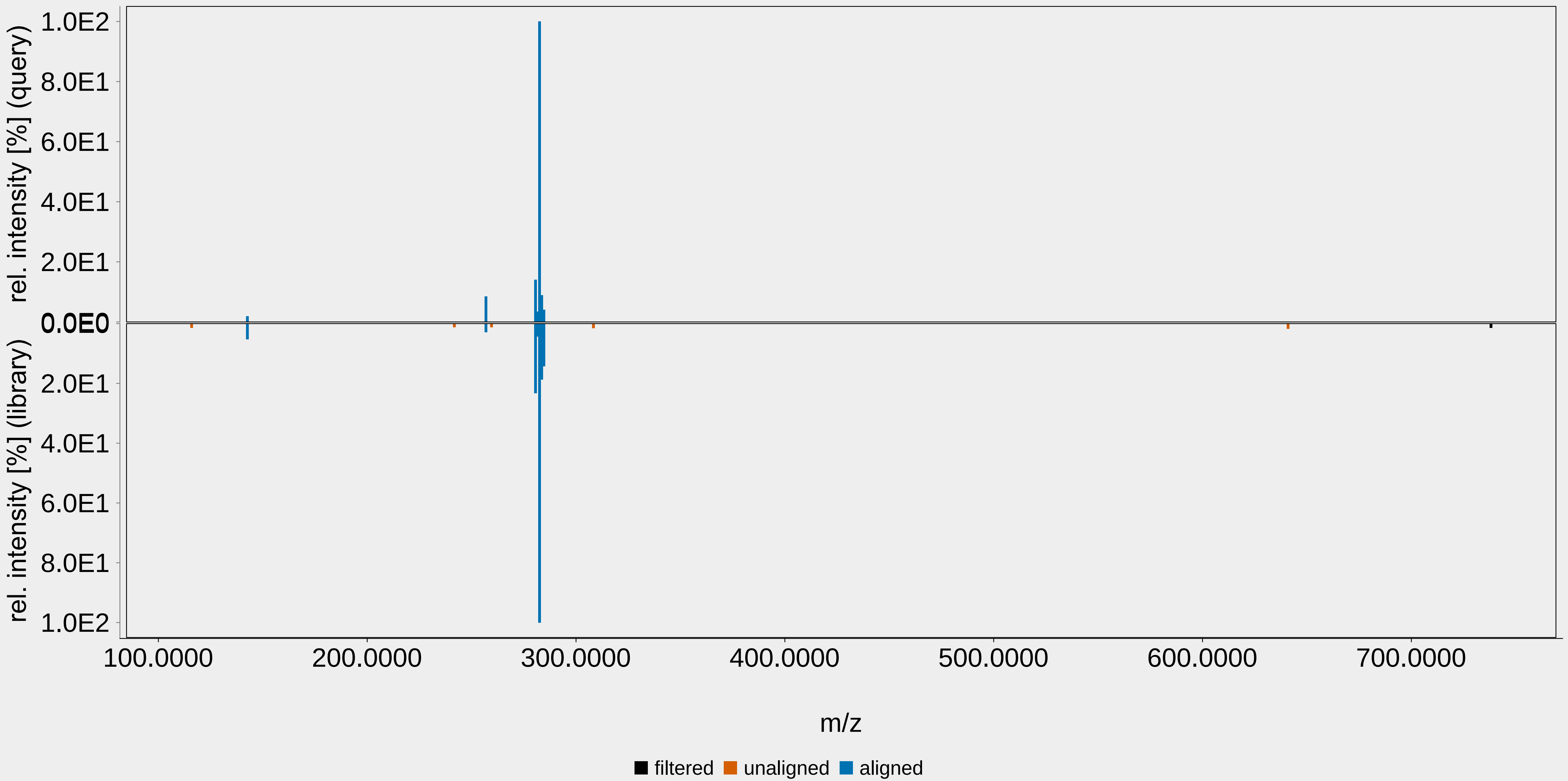

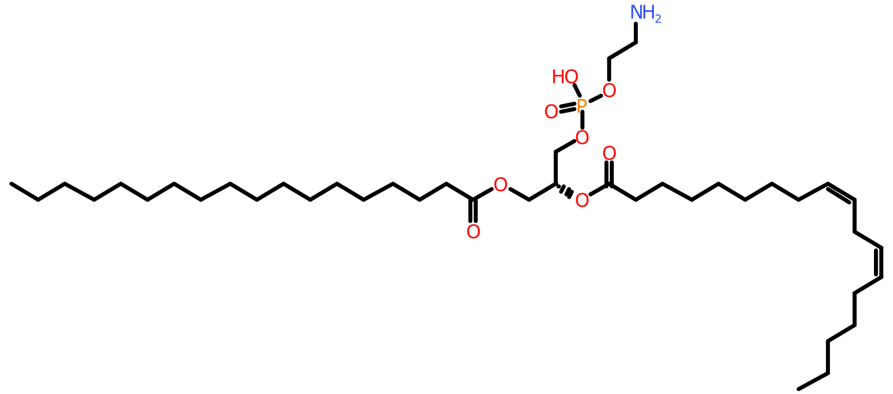

Compound information

Name: Spectral Match to

Exact mass: 743.547

Ion type: M-H

Precursor m/z: 742.541

Charge: 1

Polarity: Negative

InChI:

SMILES:

Instrument information

Instrument type: qTof

Ion source: ESI

Ms level: 2

Acquisition: Isolated

Database links

CAS: 7266537

Other information

Principal investigator: Data from Wiemann

Data collector: Data deposited by pwiemann

Entry ID: CCMSLIB00003136842

PS 18:1_24:1 **0.831**

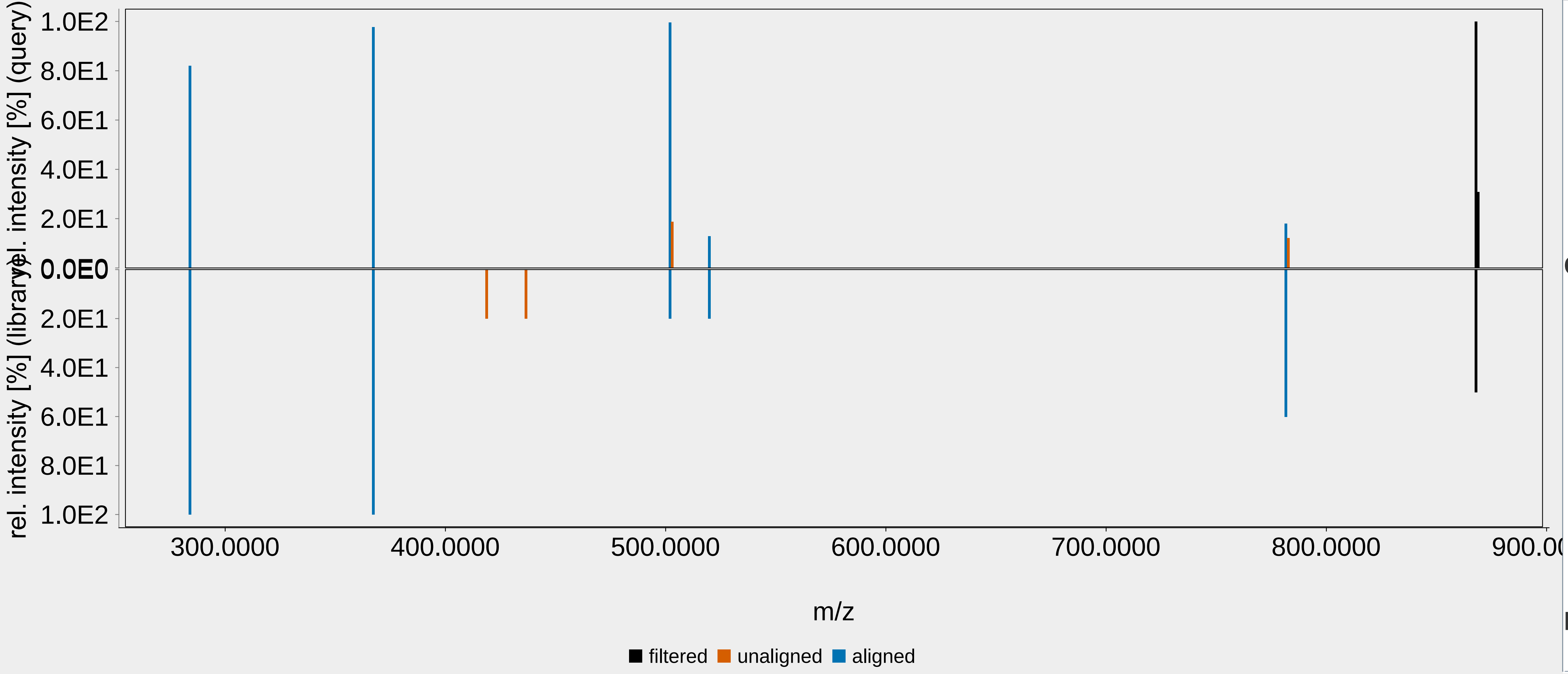

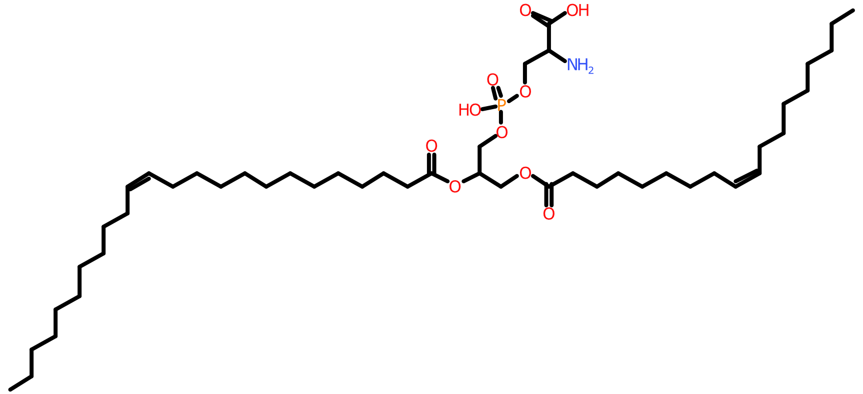

Compound information

Name: PS 18:1_24:1

Formula: C48H90NO10P

Ion type: [M-H]-

Precursor m/z: 870.62296

Ccs: 292.75

InChI key:

SMILES:

Num peaks: 8

Instrument information

Database links

Other information

PE(P-18:0/18:1); [M-H]- C41H79N1O7P1 0.784

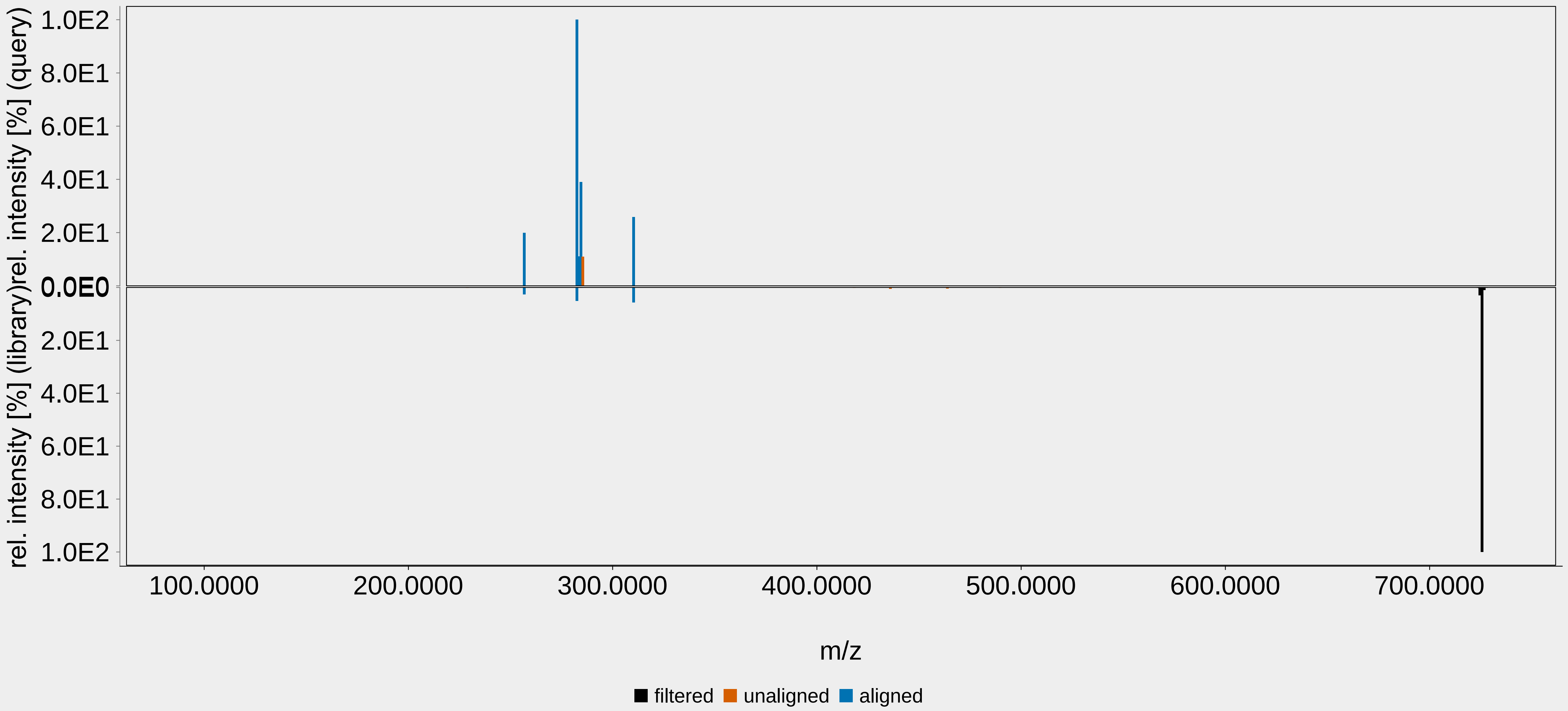

Compound information

Name: PE(P-18:0/18:1); [M-H]-

Exact mass: 0.0

Ion type: M-H

Precursor m/z: 728.559

Charge: 1

Polarity: Negative

Instrument information

Instrument type: HCD; Velos

Ion source: LC-ESI

Ms level: 2

Acquisition: Commercial

Database links

Other information

Principal investigator: Thomas Metz

Data collector: Thomas Metz

Entry ID: CCMSLIB00010072263

PE(P-18:0/18:1); [M-H]- C41H79N1O7P1

0.780

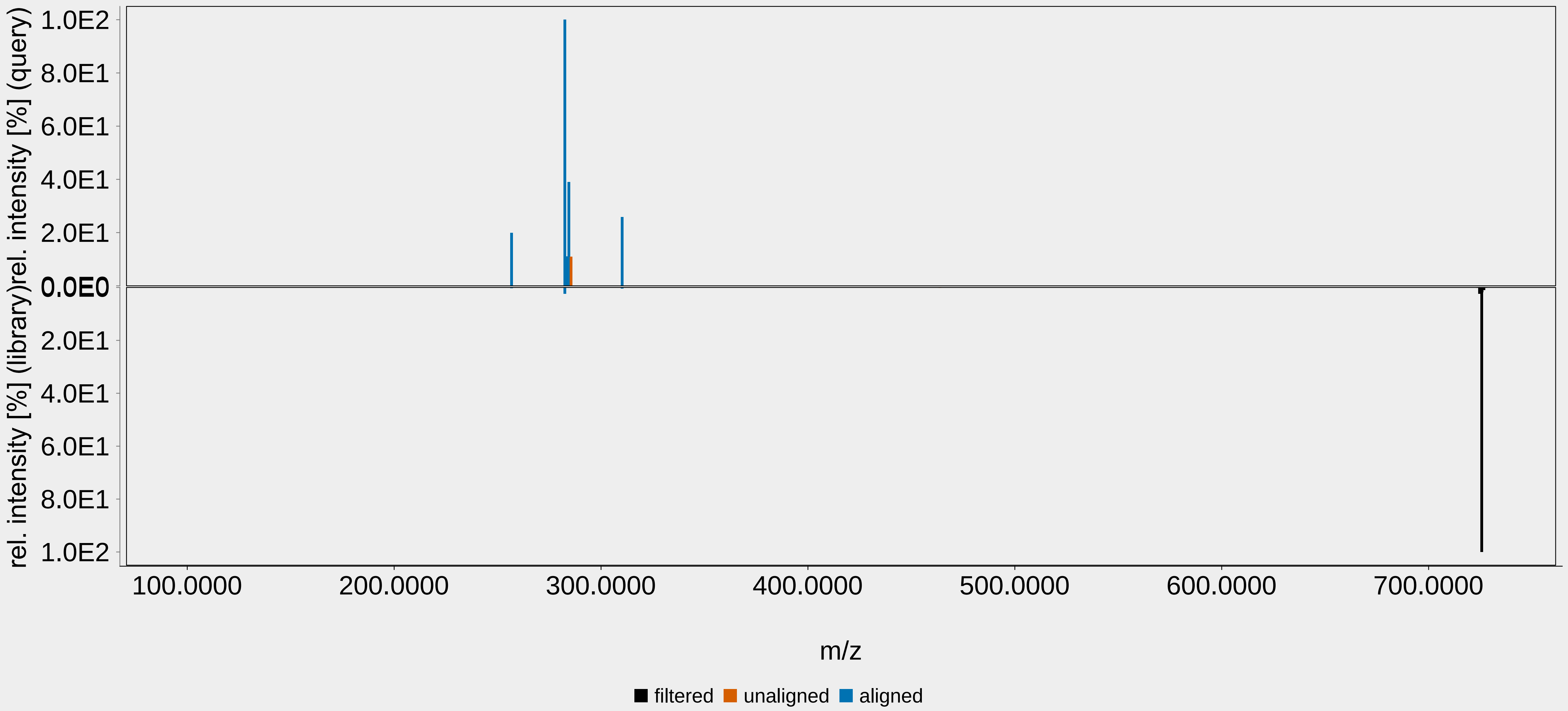

Compound information

Name: PE(P-18:0/18:1); [M-H]-

Exact mass: 0.0

Ion type: M-H

Precursor m/z: 728.559

Charge: 1

Polarity: Negative

Instrument information

Instrument type: HCD; Velos

Ion source: LC-ESI

Ms level: 2

Acquisition: Commercial

Database links

Other information

Principal investigator: Thomas Metz

Data collector: Thomas Metz

Entry ID: CCMSLIB00010068572

Spectral Match to 1-Palmitoyl-2-hydroxy-sn-glycero-3-phosphoethanolamine from NIST14

0.944

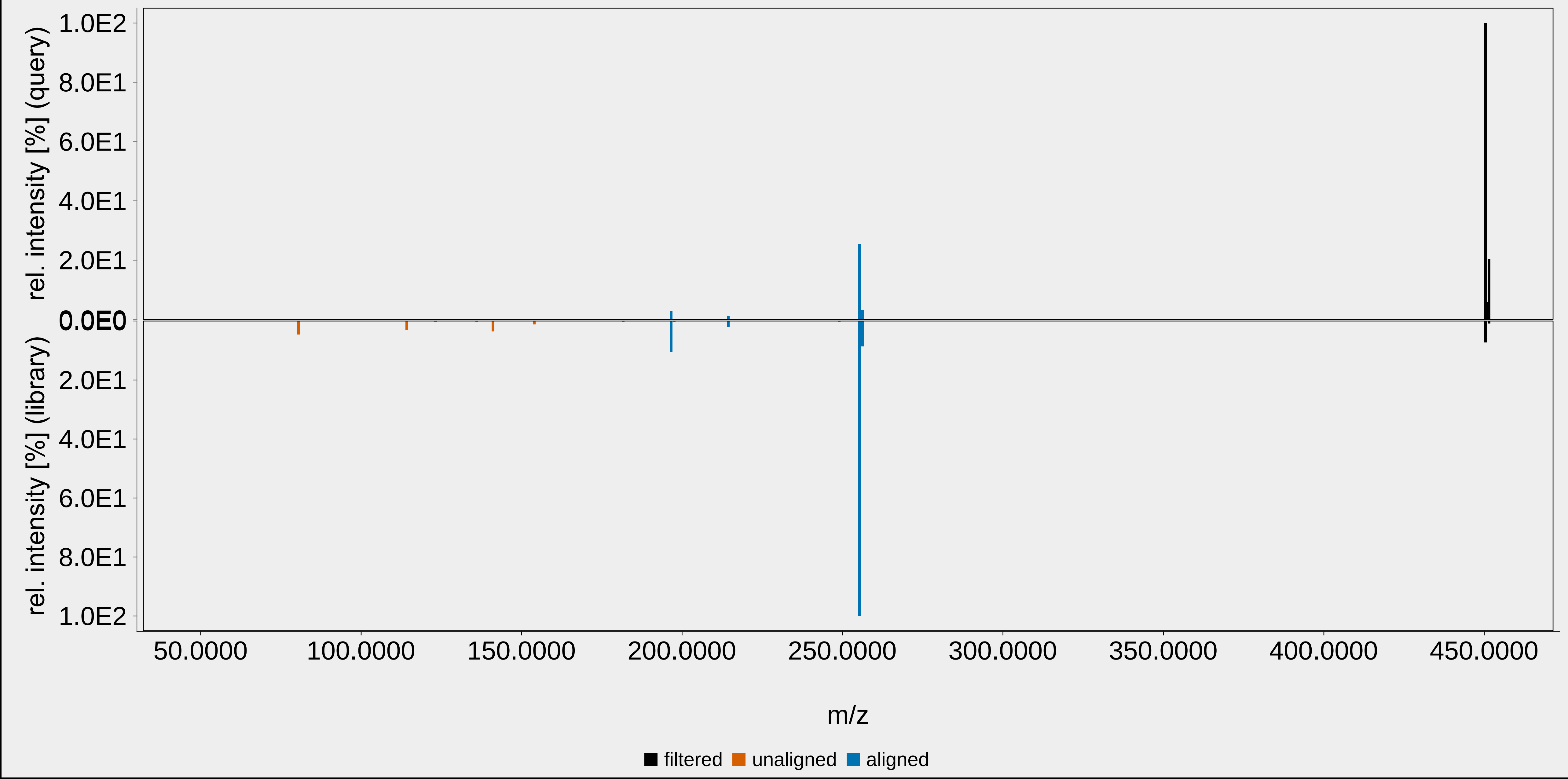

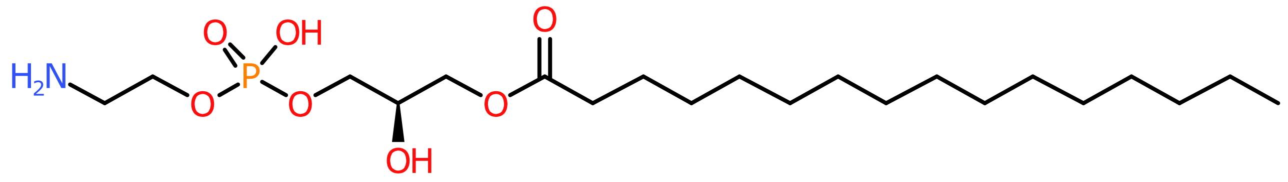

Compound information

Name: Spectral Match to

Exact mass: 453.285

Ion type: M-H

Precursor m/z: 452.279

Charge: 1

Polarity: Negative

InChI:

SMILES:

Instrument information

Instrument type: qTof

Ion source: ESI

Ms level: 2

Acquisition: Isolated

Database links

CAS: 53862354

Other information

Principal investigator: Data from Kevin Bush

Data collector: Data deposited by fevargas

Entry ID: CCMSLIB00003137341

Spectral Match to 1-Palmitoyl-2-hydroxy-sn-glycero-3-phosphoethanolamine from NIST14 0.953

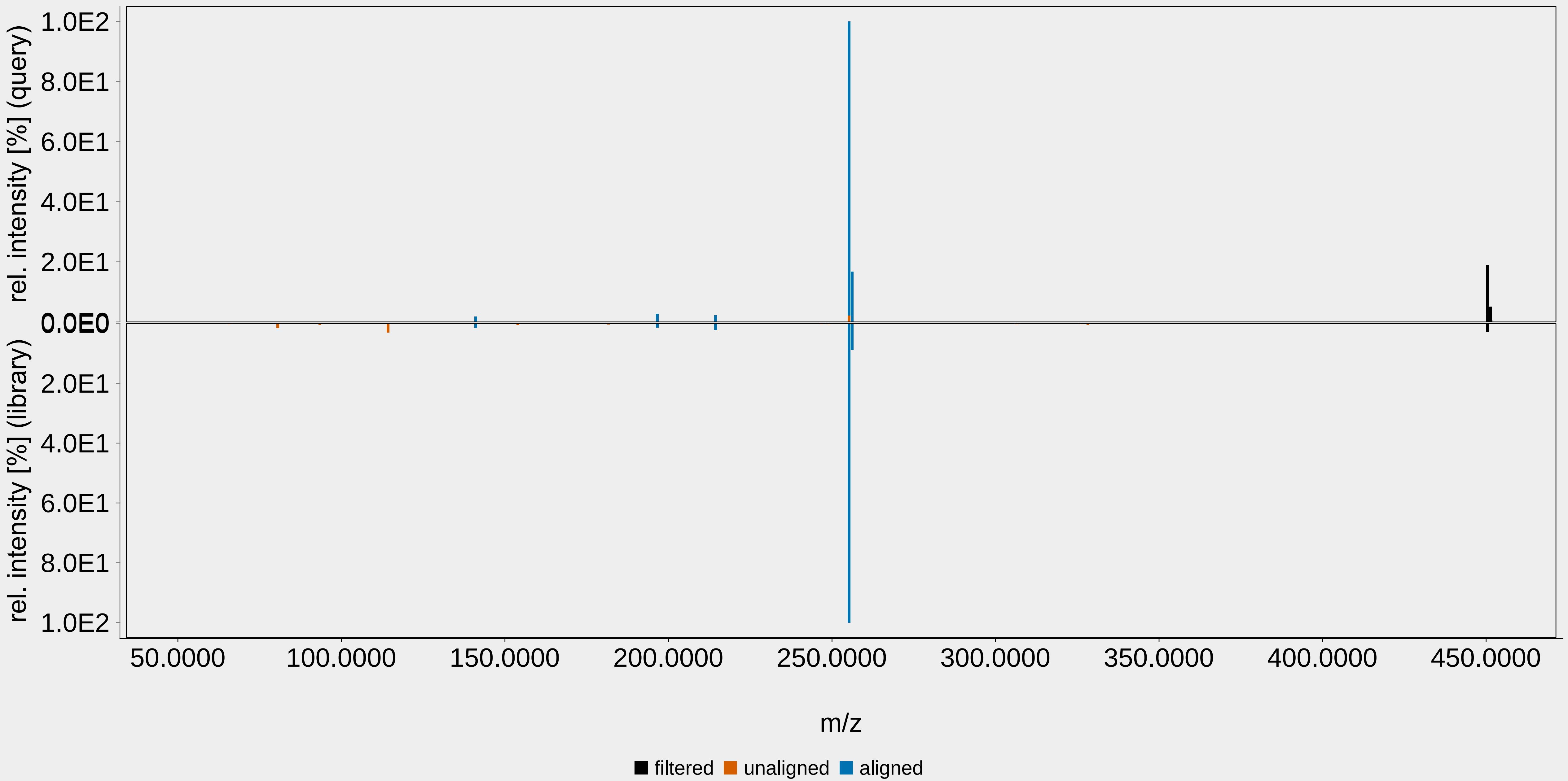

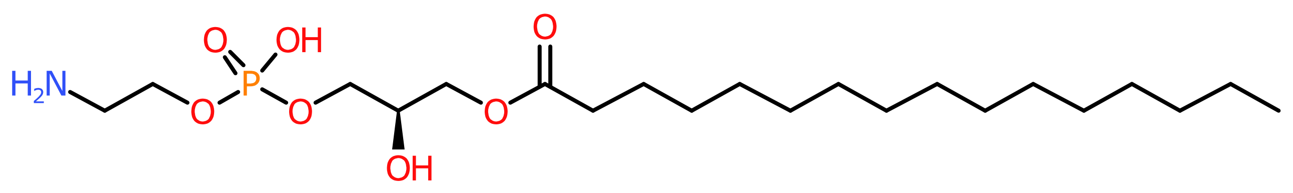

Compound information

Name: Spectral Match to

Exact mass: 453.285

Ion type: M-H

Precursor m/z: 452.279

Charge: 1

Polarity: Negative

InChI:

SMILES:

Instrument information

Instrument type: qTof

Ion source: ESI

Ms level: 2

Acquisition: Isolated

Database links

CAS: 53862354

Other information

Principal investigator: Data from Kevin Bush

Data collector: Data deposited by fevargas

Entry ID: CCMSLIB00003137726

PA 18:1_22:1 **0.808**

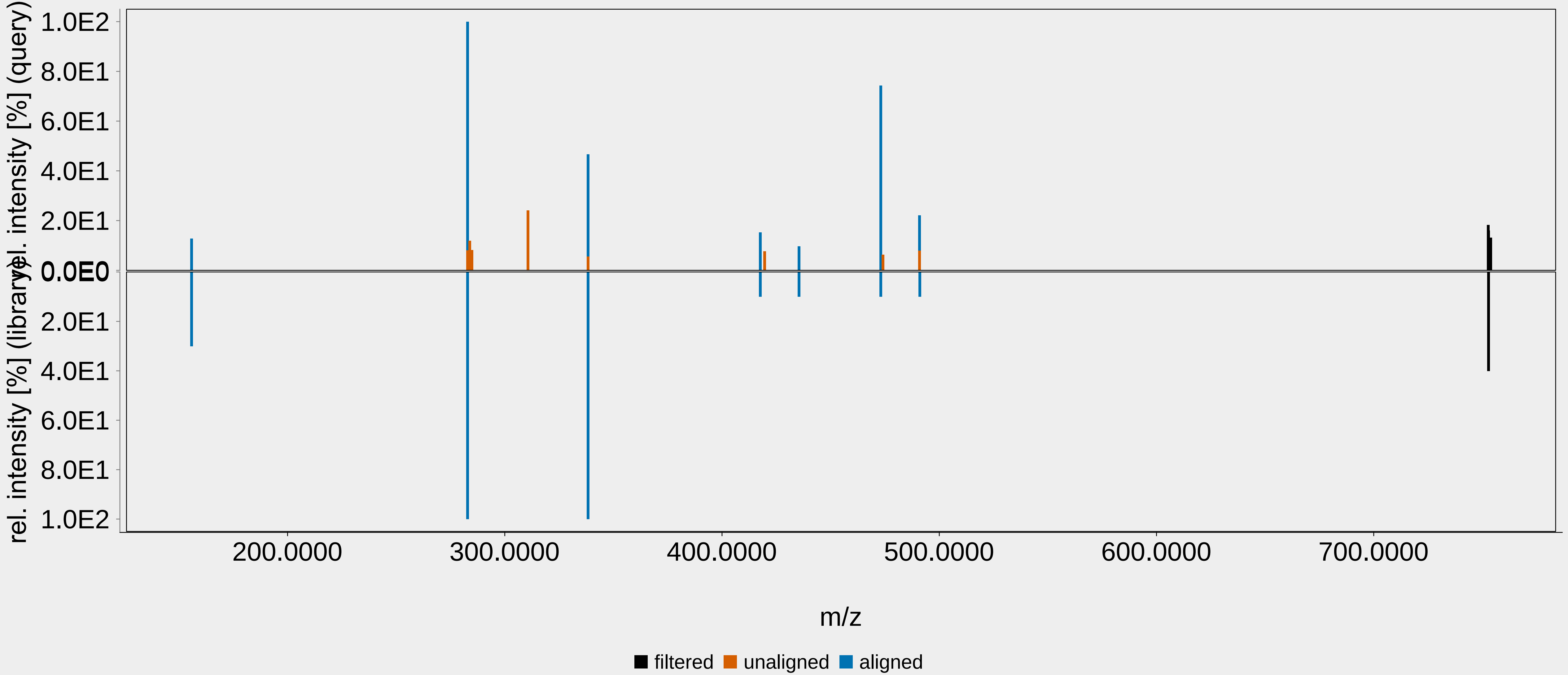

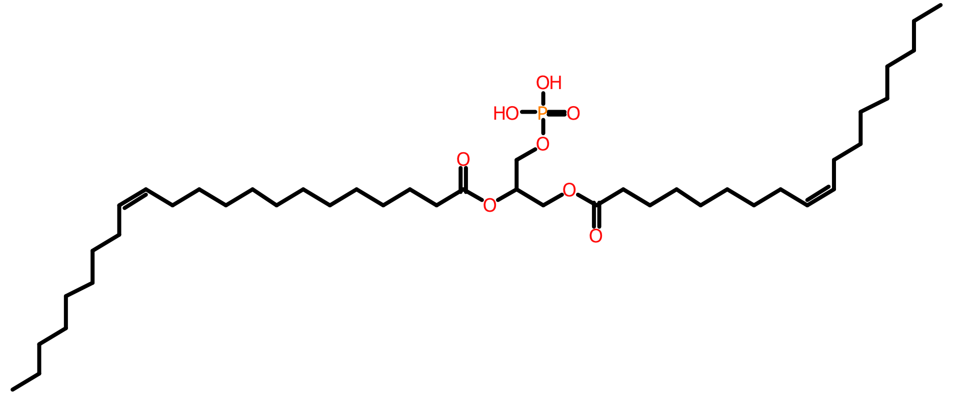

Compound information

Name: PA 18:1_22:1

Formula: C43H81O8P

Ion type: [M-H]-

Precursor m/z: 755.55963

Ccs: 271.45

InChI key:

SMILES:

Num peaks: 8

Instrument information

Database links

Other information

PE(20:1/0:0); [M-H]- C25H49N1O7P1 0.739

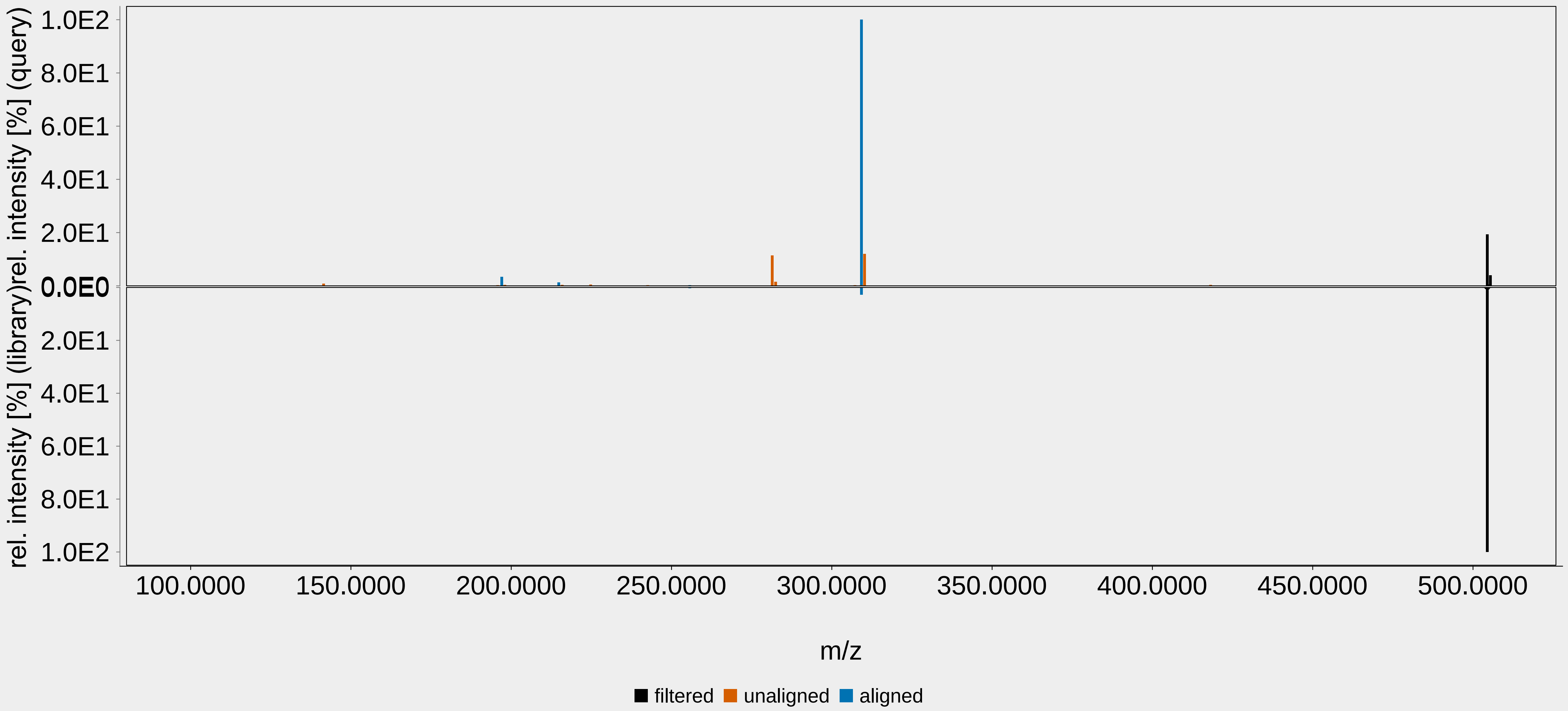

Compound information

Name: PE(20:1/0:0); [M-H]- C25H49N1O7P1

Exact mass: 0.0

Ion type: M-H

Precursor m/z: 506.325

Charge: 1

Polarity: Negative

Instrument information

Instrument type: HCD; Velos

Ion source: LC-ESI

Ms level: 2

Acquisition: Commercial

Database links

Other information

Principal investigator: Thomas Metz

Data collector: Thomas Metz

Entry ID: CCMSLIB00010086765

PE(20:1/0:0); [M-H]- C25H49N1O7P1 **0.757**

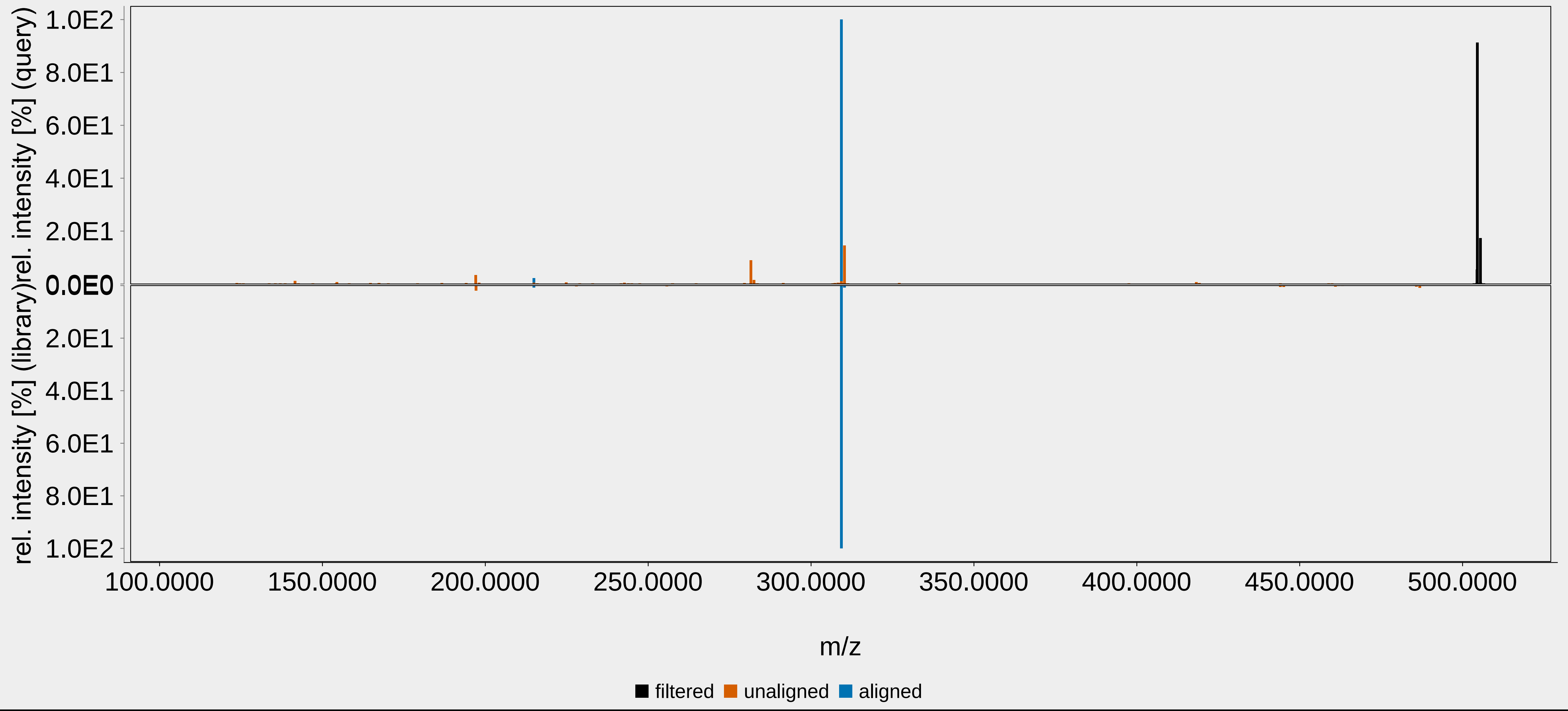

Compound information

Name: PE(20:1/0:0); [M-H]- C25H49N1O7P1

Exact mass: 0.0

Ion type: M-H

Precursor m/z: 506.325

Charge: 1

Polarity: Negative

Instrument information

Instrument type: CID; Velos

Ion source: LC-ESI

Ms level: 2

Acquisition: Commercial

Database links

Other information

Principal investigator: Thomas Metz

Data collector: Thomas Metz

Entry ID: CCMSLIB00010063977

Canrenone

0.939

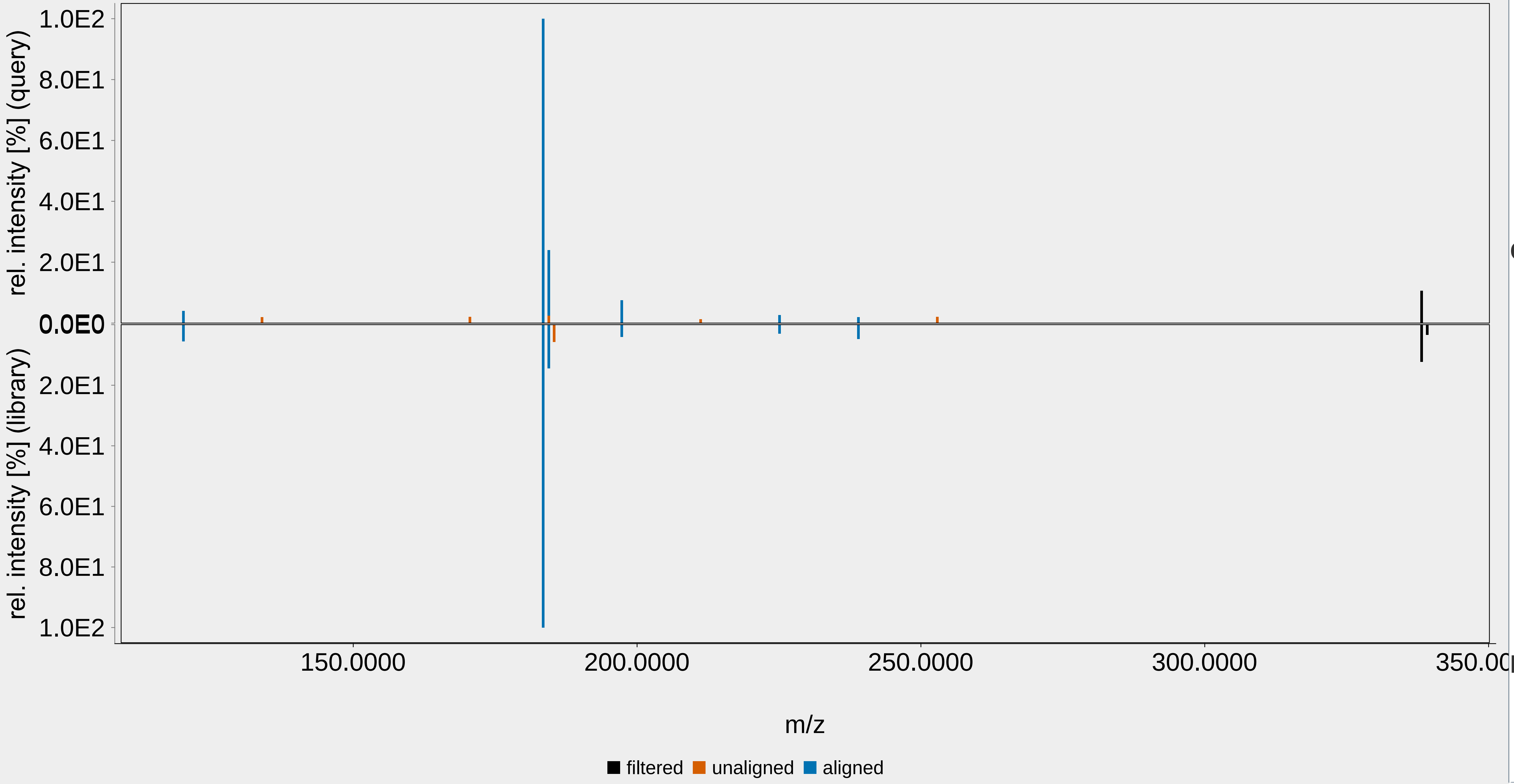

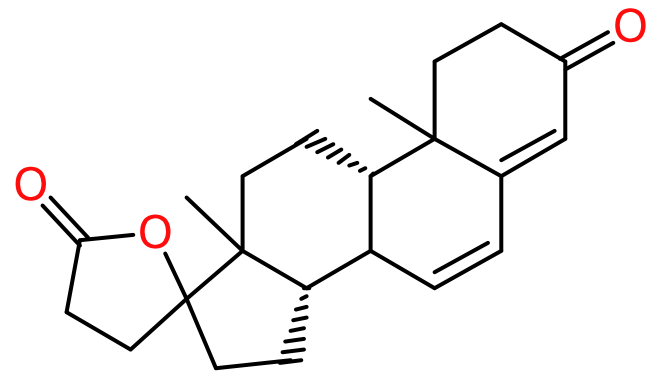

Compound information

Name: Canrenone

Formula: C22H28O3

Exact mass: 340.2038448

Ion type: [M-H]-

Precursor m/z: 339.1966

Polarity: NEGATIVE

InChI:

InChI key:

SMILES:

Num peaks: 9

Instrument information

Instrument type: LC-ESI-QTOF

Instrument: Bruker maXis Impact

Ms level: MS2

Collision energy: 40 eV

Database links

Other information

Entry ID: MSBNK-Athens_Univ-AU239460

Comment: Parent=339.1966

Canrenone, (9S,14S)-10,13-dimethylspiro[2,8,9,11,12,14,15,16-octahydro-1H-cyclopenta[a]phenanthrene-17,5`-oxolane]-2`,3-dione

0.939

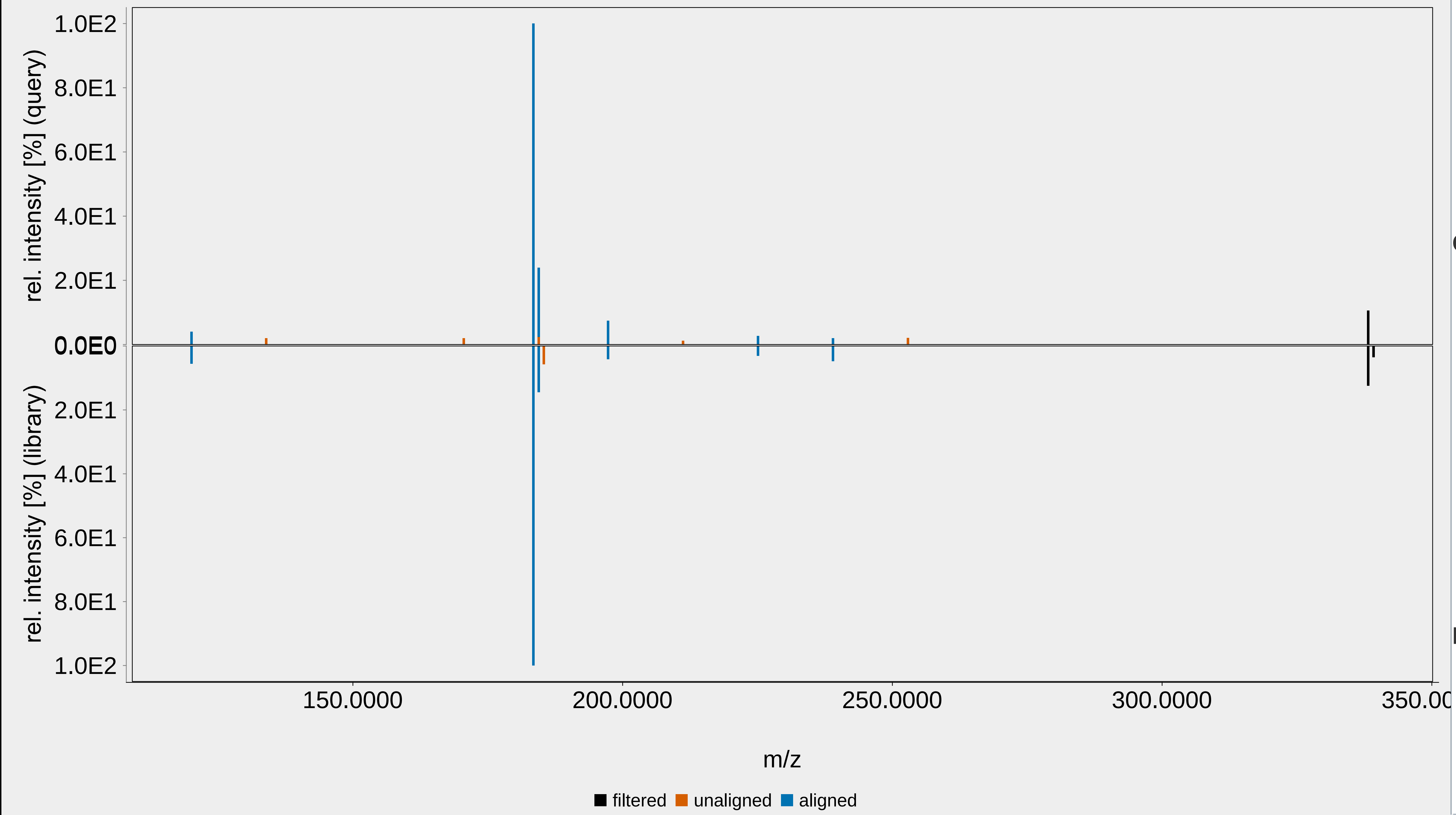

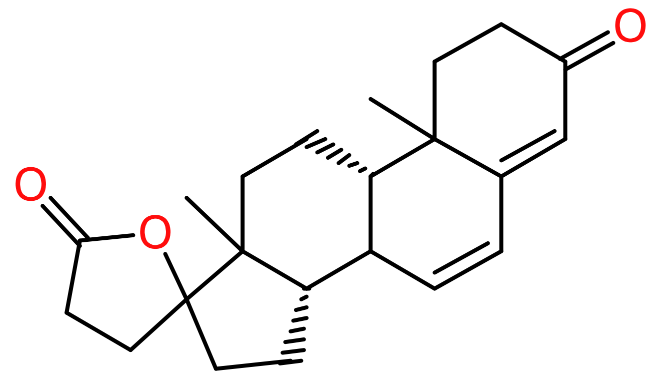

Compound information

Name: Canrenone,

Formula: C22H28O3

Mol. weight: 340.2038448

Exact mass: 340.2038448

Ion type: [M-H]-

Precursor m/z: 339.1966

Polarity: negative

InChI:

InChI key:

SMILES:

Instrument information

Instrument type: LC-ESI-QTOF

Instrument: Bruker maXis Impact

Ion source: ESI

Resolution: 35000

Ms level: MS2

Collision energy: 40 eV

Database links

Pubchem: 24728626

MoNA ID: AU239460

Chemspider: 21112020

CAS: 976-71-6

Other information

Principal investigator: Nikiforos Alygizakis,

Data collector: Nikiforos Alygizakis, Katerina

Guanosine diphosphate - 30.0 eV

0.876

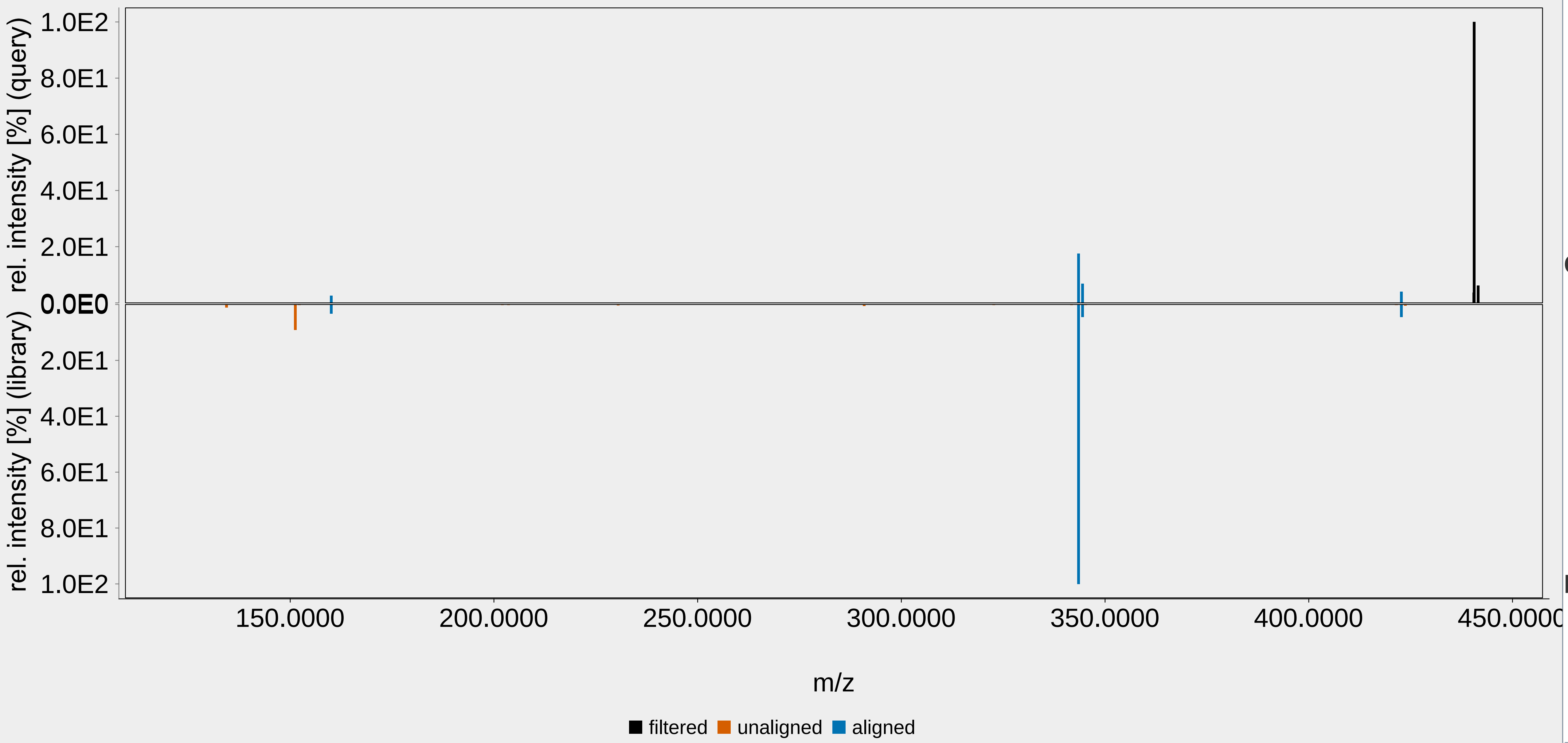

Compound information

Name: Guanosine diphosphate - 30.0 eV

Exact mass: 0.0

Ion type: M-H

Precursor m/z: 442.017

Charge: 1

Polarity: Negative

SMILES:

Instrument information

Instrument type: Orbitrap

Ion source: ESI

Ms level: 2

Acquisition: Isolated

Database links

CAS:

Other information

Principal investigator: PI

Data collector: Data Collector

Entry ID: CCMSLIB00006123856

Guanosine diphosphate - 30.0 eV

0.880

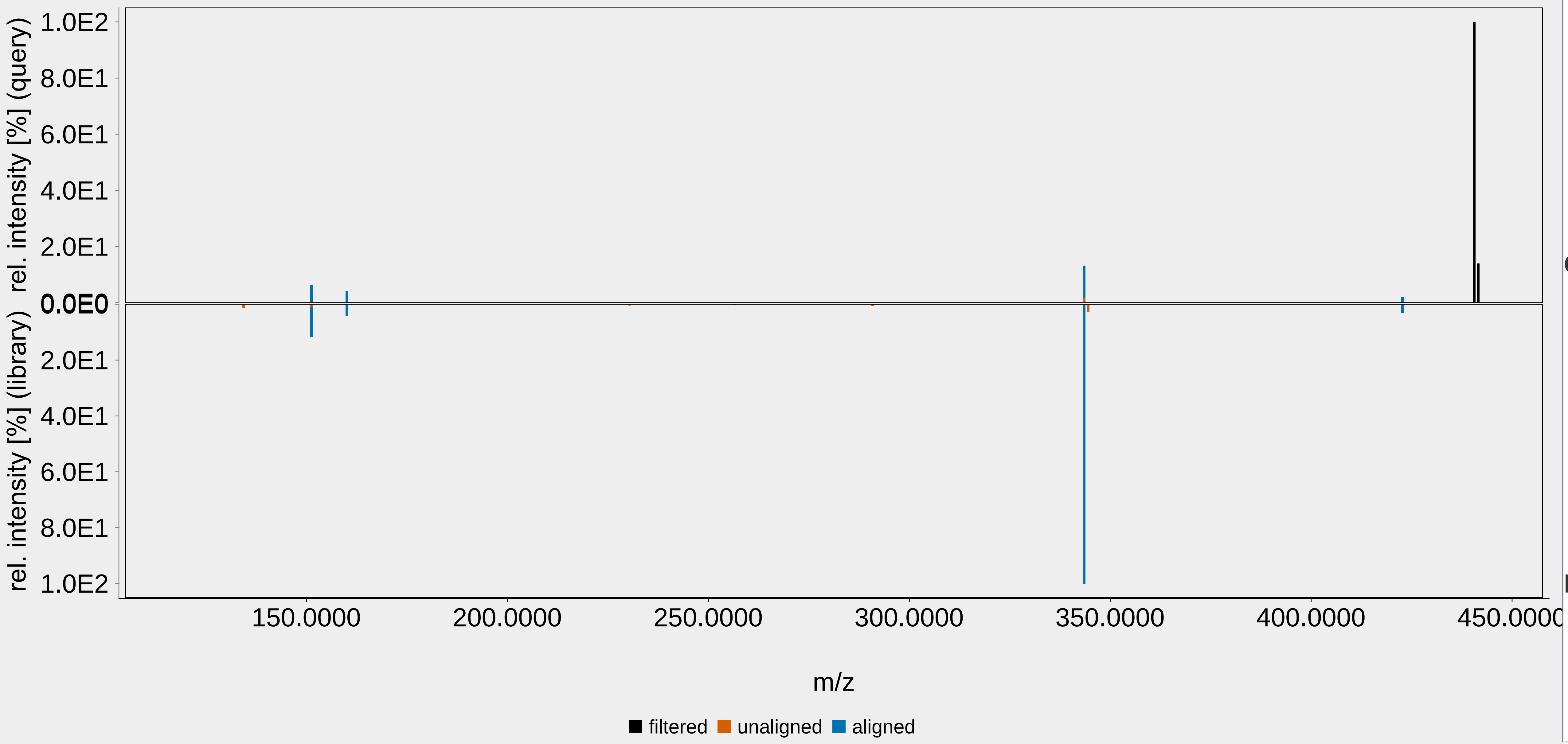

Compound information

Name: Guanosine diphosphate - 30.0 eV

Exact mass: 0.0

Ion type: M-H

Precursor m/z: 442.017

Charge: 1

Polarity: Negative

SMILES:

Instrument information

Instrument type: Orbitrap

Ion source: ESI

Ms level: 2

Acquisition: Isolated

Database links

CAS:

Other information

Principal investigator: PI

Data collector: Data Collector

Entry ID: CCMSLIB00006123838

PS 23:0_18:1

0.841

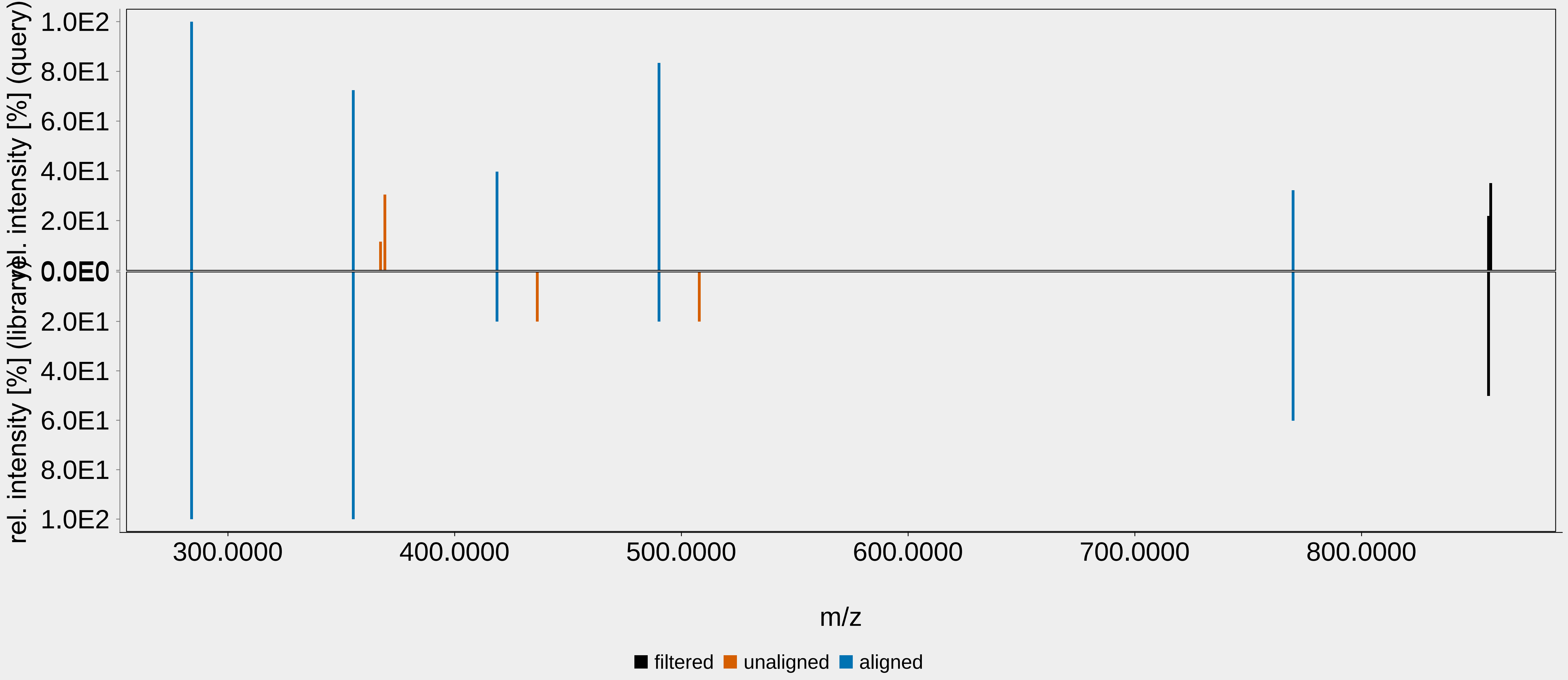

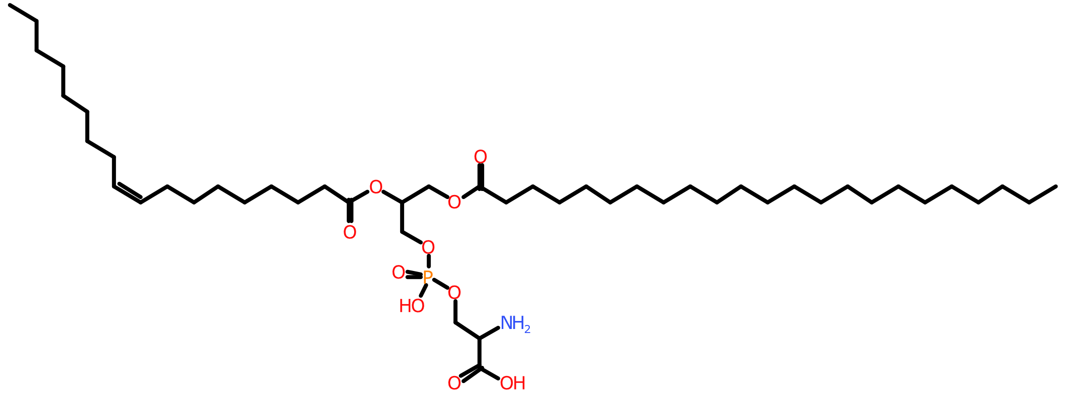

Compound information

Name: PS 23:0_18:1

Formula: C47H90NO10P

Ion type: [M-H]-

Precursor m/z: 858.62296

Ccs: 292.27

InChI key:

SMILES:

Num peaks: 8

Instrument information

Database links

Other information

PA 18:1_18:1 **0.876**

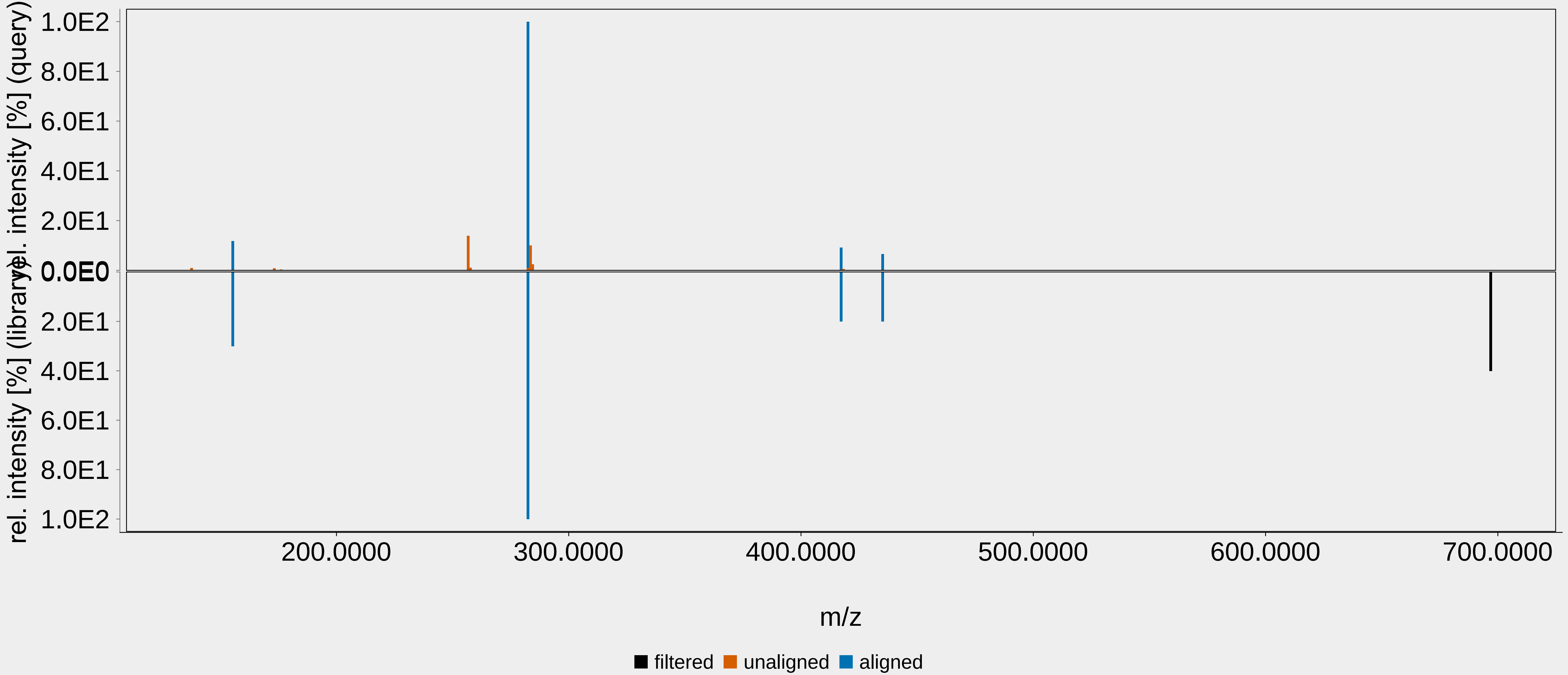

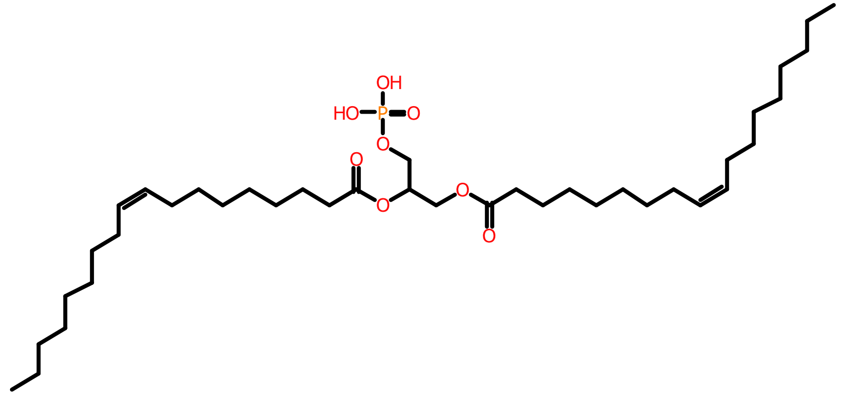

Compound information

Name: PA 18:1_18:1

Formula: C39H73O8P

Ion type: [M-H]-

Precursor m/z: 699.49703

Ccs: 257.54

InChI key: MHUWZNTUIIFHAS-CLFAGFIQNA-l

SMILES:

Num peaks: 5

Instrument information

Database links

Other information

Glutathione - 30.0 eV

0.876

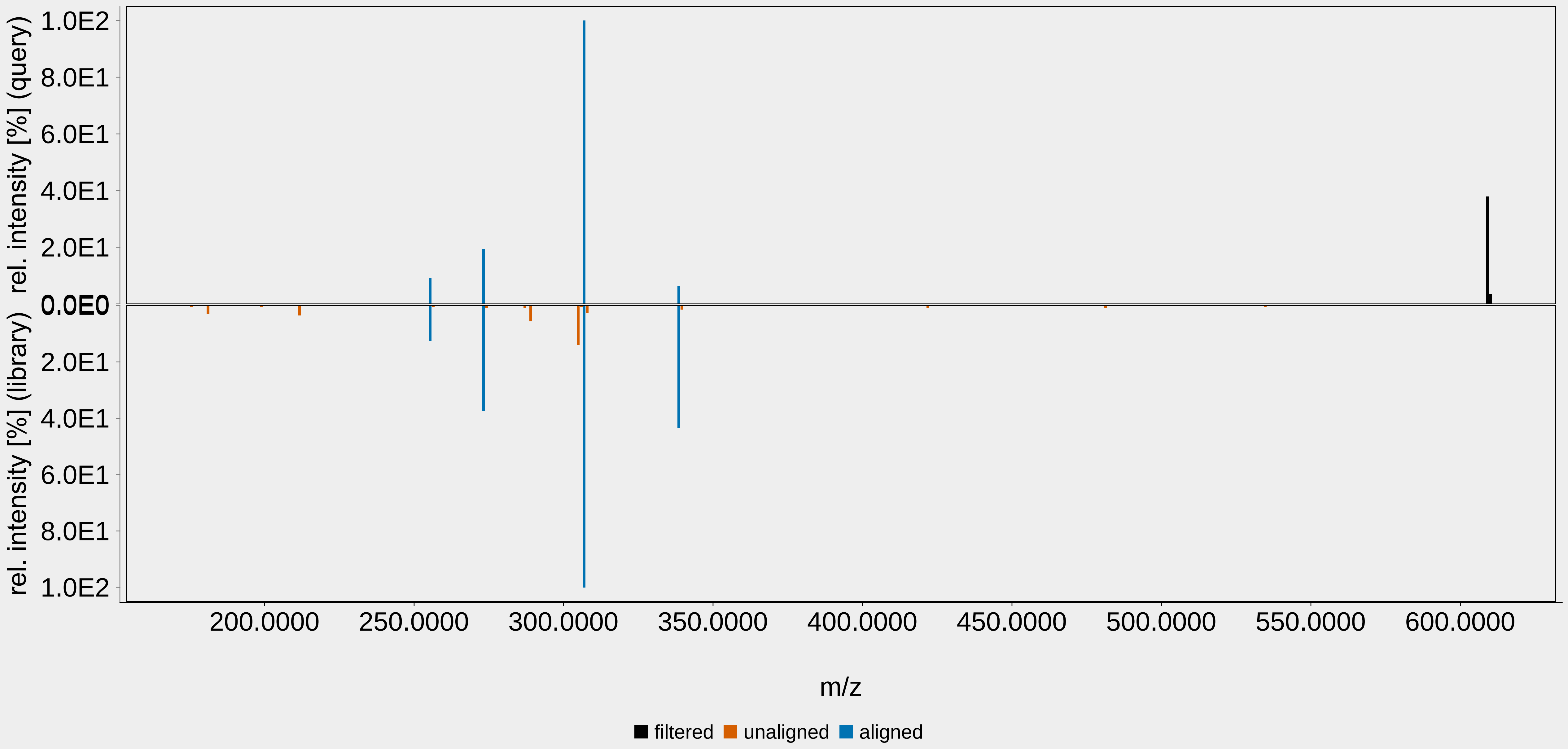

Compound information

Name: Glutathione - 30.0 eV

Exact mass: 0.0

Ion type: M-H

Precursor m/z: 611.145

Charge: 1

Polarity: Negative

SMILES:

Instrument information

Instrument type: Orbitrap

Ion source: ESI

Ms level: 2

Acquisition: Isolated

Database links

CAS:

Other information

Principal investigator: PI

Data collector: Data Collector

Entry ID: CCMSLIB00006123948

Glutathione (oxidized) 0.918

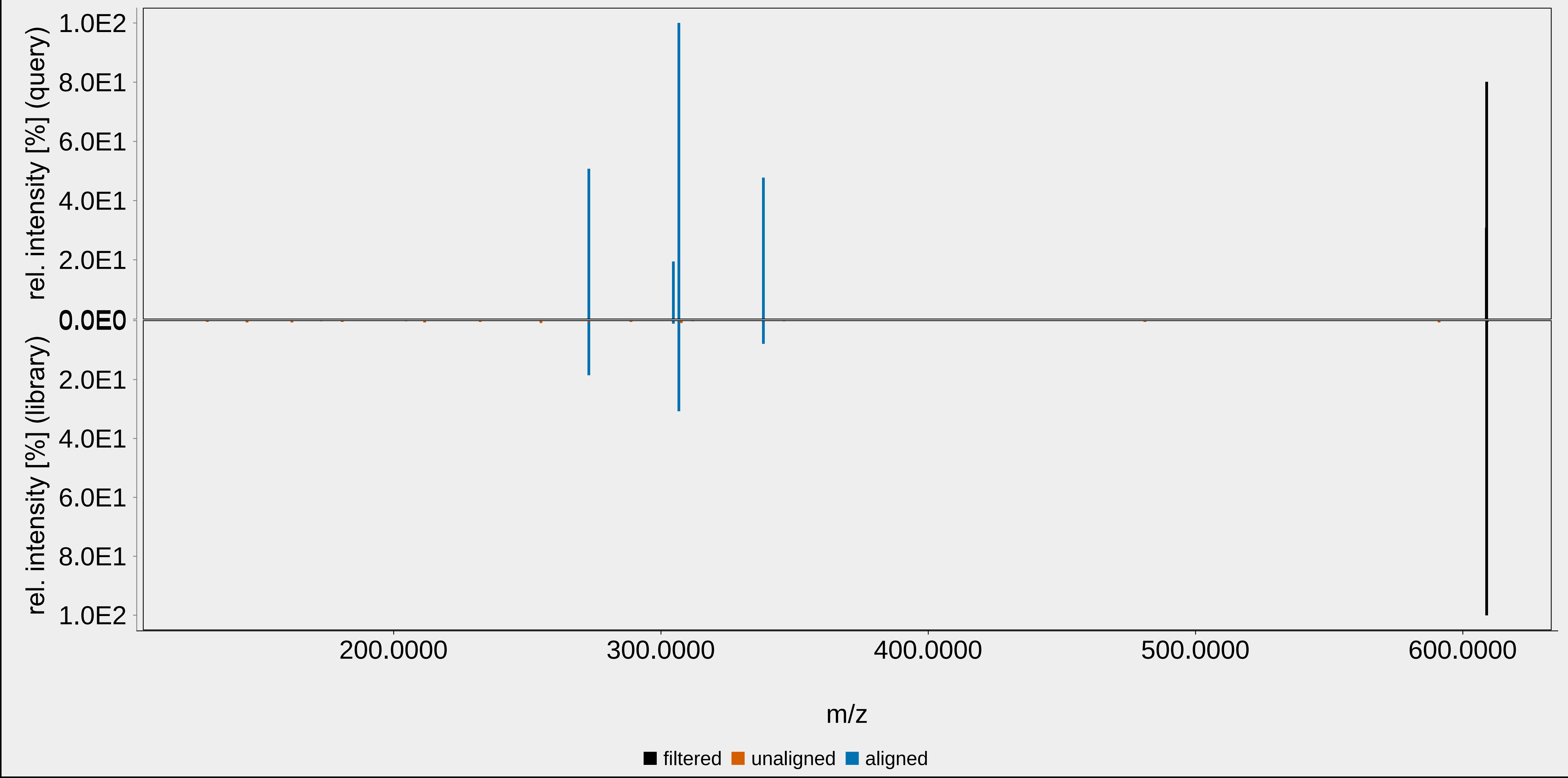

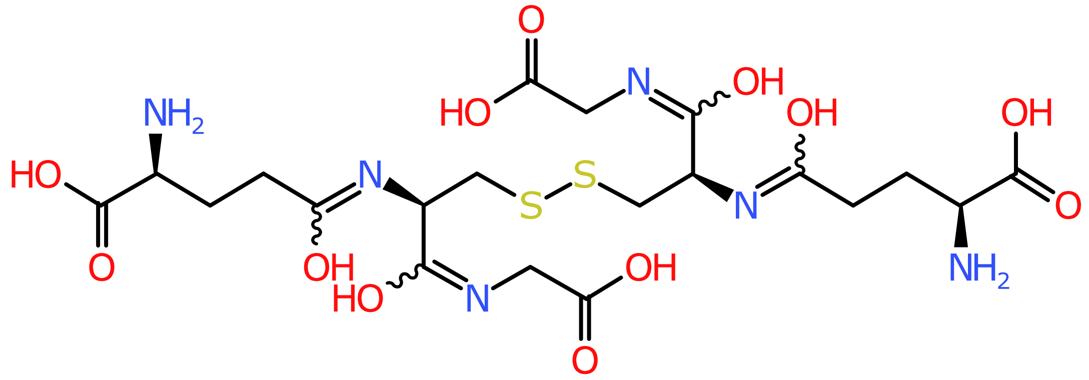

Compound information

Name: Glutathione (oxidized)

Formula: C20H32N6O12S2

Ion type: [M-H]-

Precursor m/z: 611.142

RT: 9.794383

Polarity: negative

InChI:

InChI key:

SMILES:

Instrument information

Instrument type: LC-ESI-QTOF

Instrument: SCIEX TripleTOF 6600

Ms level: MS2

Collision energy: 35 eV

Database links

Other information

Principal investigator: Michael Sa and Megan

Data collector: Michael Sa and Megan

PE(18:1/20:1); [M-H]- C43H81N1O8P1

0.917

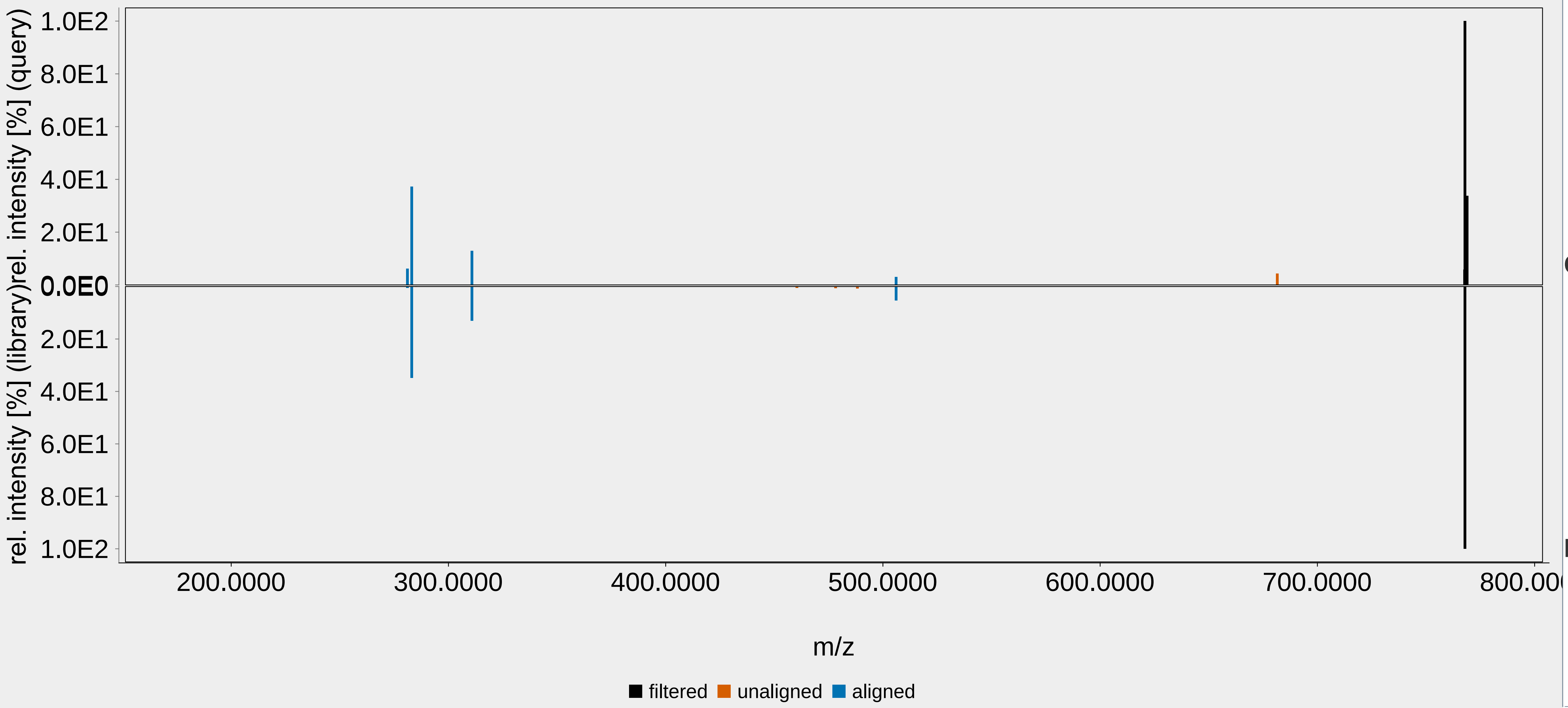

Compound information

Name: PE(18:1/20:1); [M-H]- C43H81N1O8P1

Exact mass: 0.0

Ion type: M-H

Precursor m/z: 770.57

Charge: 1

Polarity: Negative

Instrument information

Instrument type: CID; Lumos

Ion source: LC-ESI

Ms level: 2

Acquisition: Commercial

Database links

Other information

Principal investigator: Thomas Metz

Data collector: Thomas Metz

Entry ID: CCMSLIB00010059697

PE(18:1/20:1); [M-H]- C43H81N1O8P1 **0.918**

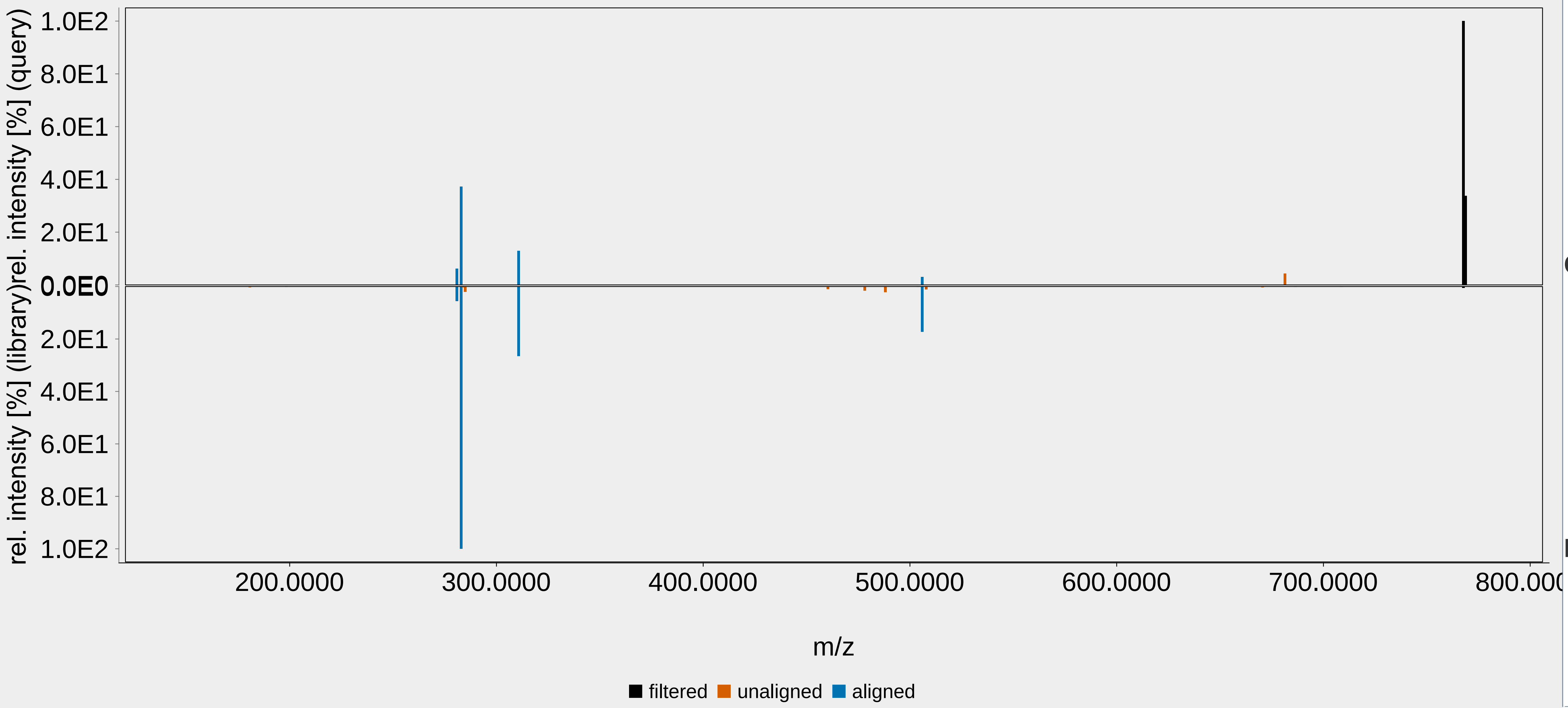

Compound information

Name: PE(18:1/20:1); [M-H]- C43H81N1O8P1

Exact mass: 0.0

Ion type: M-H

Precursor m/z: 770.57

Charge: 1

Polarity: Negative

Instrument information

Instrument type: CID; Lumos

Ion source: LC-ESI

Ms level: 2

Acquisition: Commercial

Database links

Other information

Principal investigator: Thomas Metz

Data collector: Thomas Metz

Entry ID: CCMSLIB00010059418

Docosahexaenoic acid

0.865

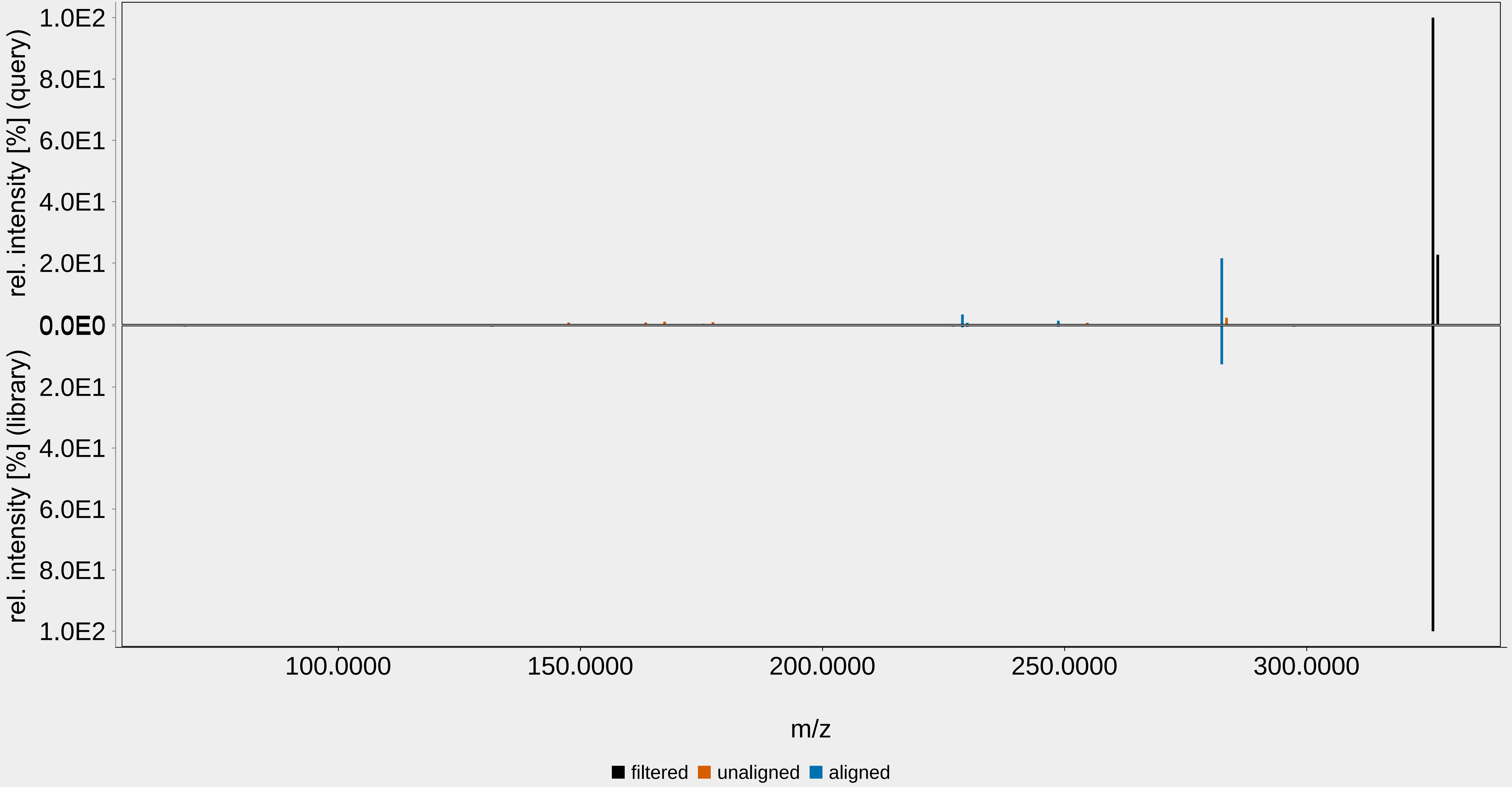

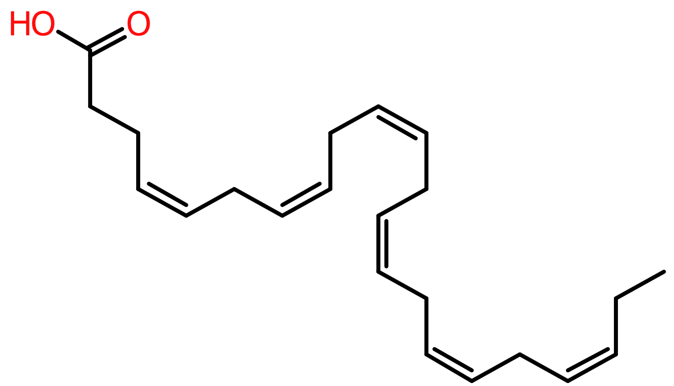

Compound information

Name: Docosahexaenoic acid

Formula: C22H32O2

Exact mass: 328.24023

Ion type: [M-H]-

Precursor m/z: 327.233

Polarity: NEGATIVE

InChI:

InChI key:

SMILES:

Num peaks: 9

Instrument information

Instrument type: LC-ESI-QTOF

Instrument: maXis plus UHR-ToF-MS, Bruker

Ms level: MS2

Collision energy: 10

Database links

Other information

Entry ID: MSBNK-BGC_Munich-RP029611

Comment: Parent=327.233

Spectral Match to cis-4,7,10,13,16,19-Docosahexaenoic acid from NIST14

0.882

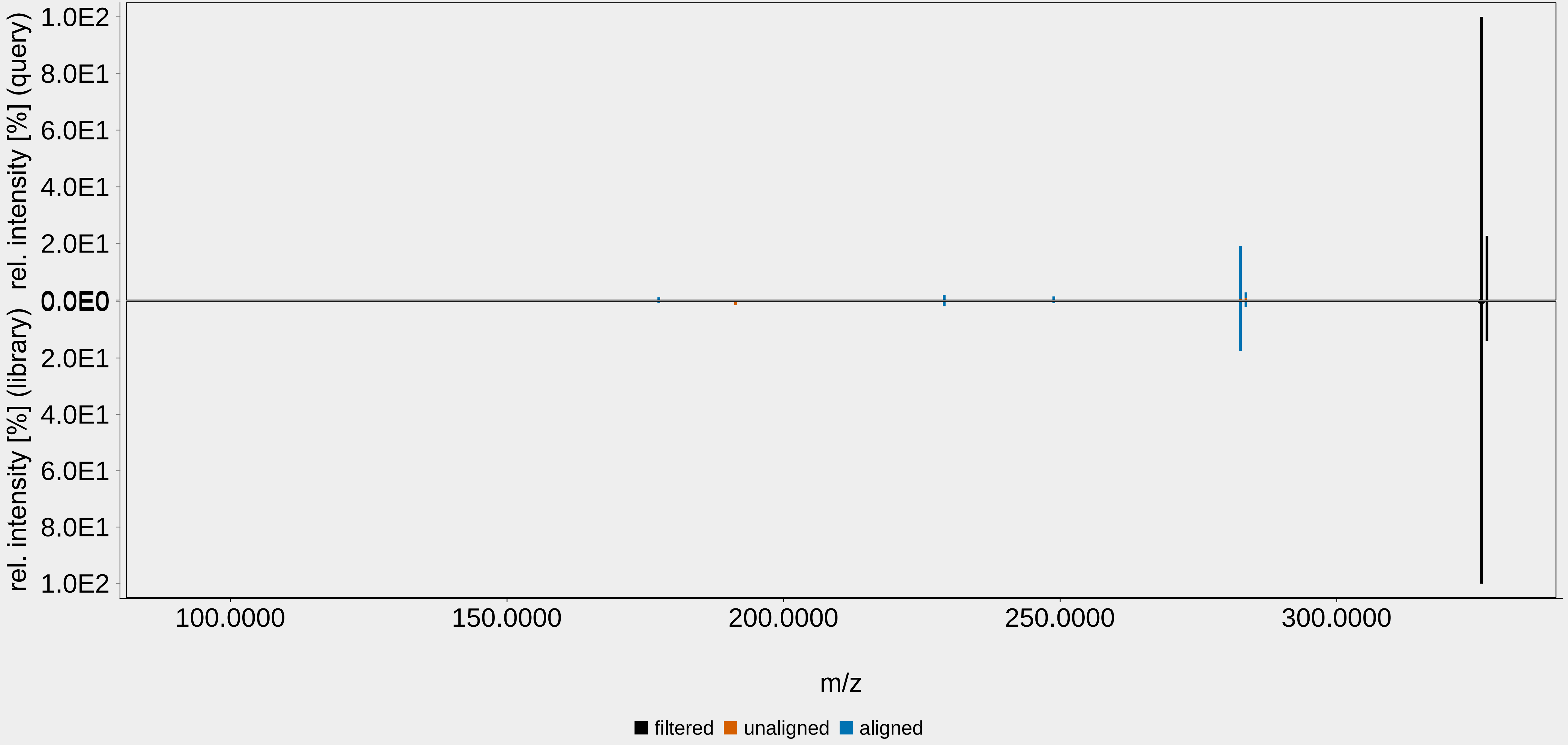

Compound information

Name: Spectral Match to

Exact mass: 0.0

Ion type: M-H

Precursor m/z: 327.232

Charge: 1

Polarity: Negative

SMILES:

Instrument information

Instrument type: qTof

Ion source: LC-ESI

Ms level: 2

Acquisition: Isolated

Database links

CAS: 6217545

Other information

Principal investigator: Data from Charles

Data collector: Data deposited by alchemistmatt

Entry ID: CCMSLIB00003137612

PE(20:0/0:0); [M-H]- C25H51N1O7P1 **0.757**

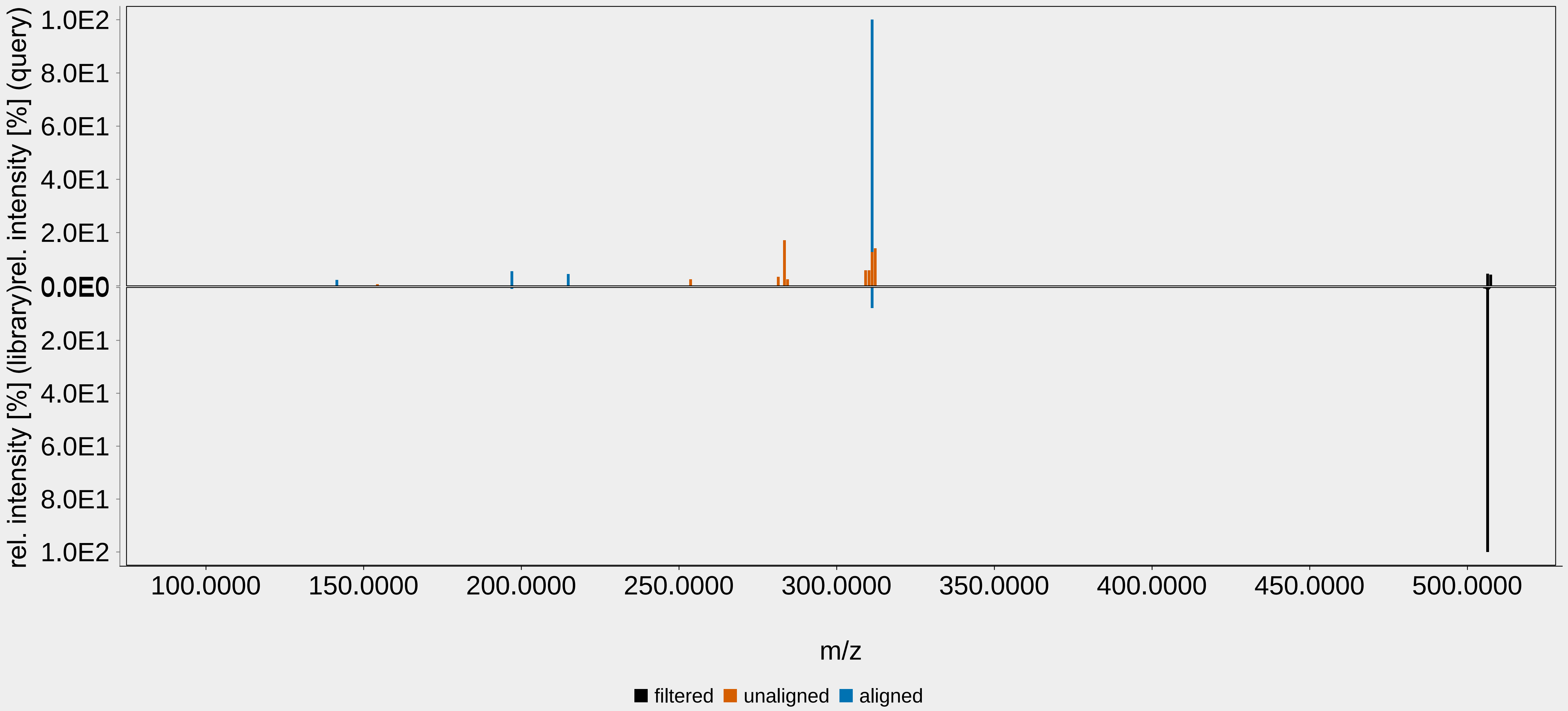

Compound information

Name: PE(20:0/0:0); [M-H]- C25H51N1O7P1

Exact mass: 0.0

Ion type: M-H

Precursor m/z: 508.34

Charge: 1

Polarity: Negative

Instrument information

Instrument type: HCD; Velos

Ion source: LC-ESI

Ms level: 2

Acquisition: Commercial

Database links

Other information

Principal investigator: Thomas Metz

Data collector: Thomas Metz

Entry ID: CCMSLIB00010063231

Adenosine diphosphate - 30.0 eV

0.874

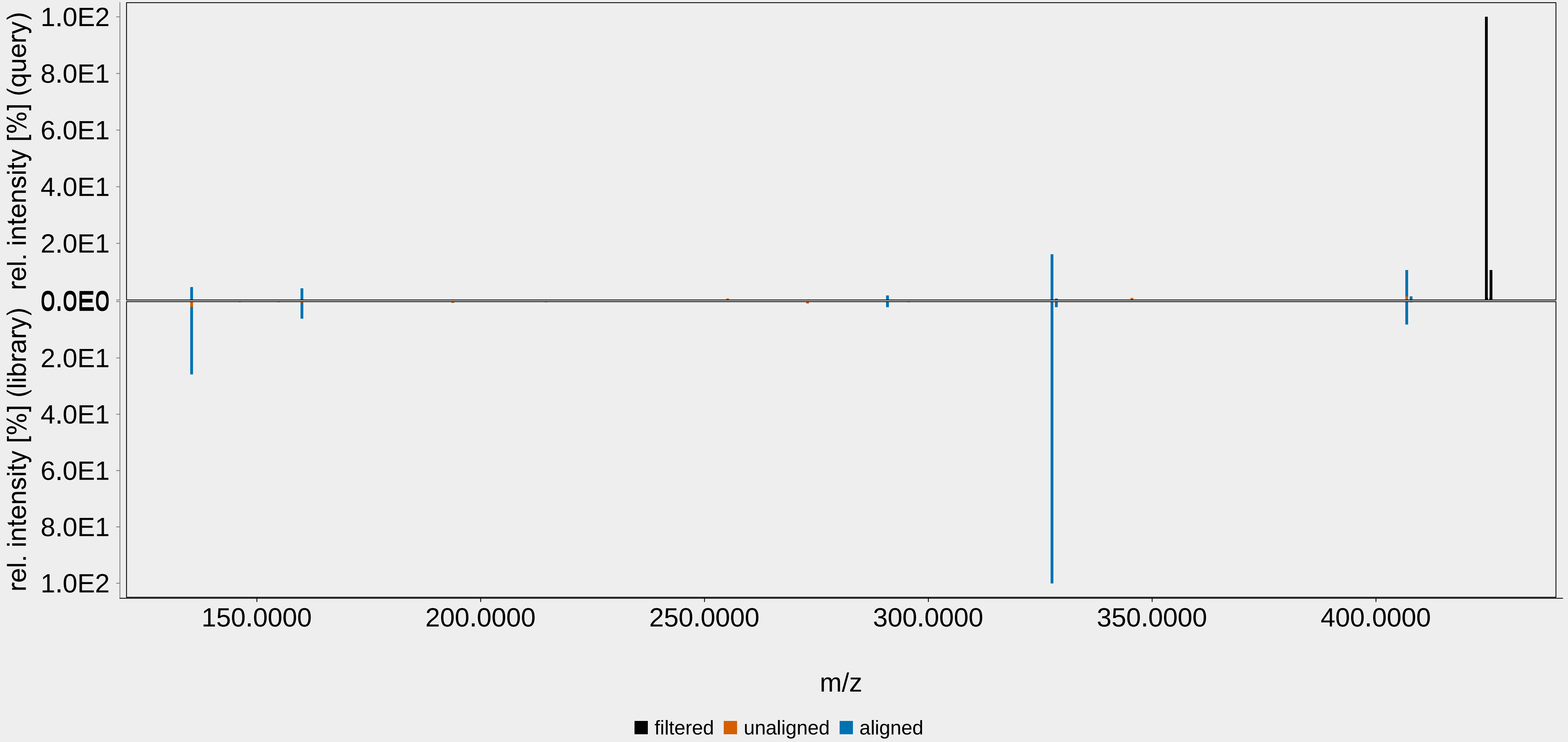

Compound information

Name: Adenosine diphosphate - 30.0 eV

Exact mass: 0.0

Ion type: M-H

Precursor m/z: 426.022

Charge: 1

Polarity: Negative

SMILES:

Instrument information

Instrument type: Orbitrap

Ion source: ESI

Ms level: 2

Acquisition: Isolated

Database links

CAS:

Other information

Principal investigator: PI

Data collector: Data Collector

Entry ID: CCMSLIB00006124443

Adenosine diphosphate - 30.0 eV

0.876

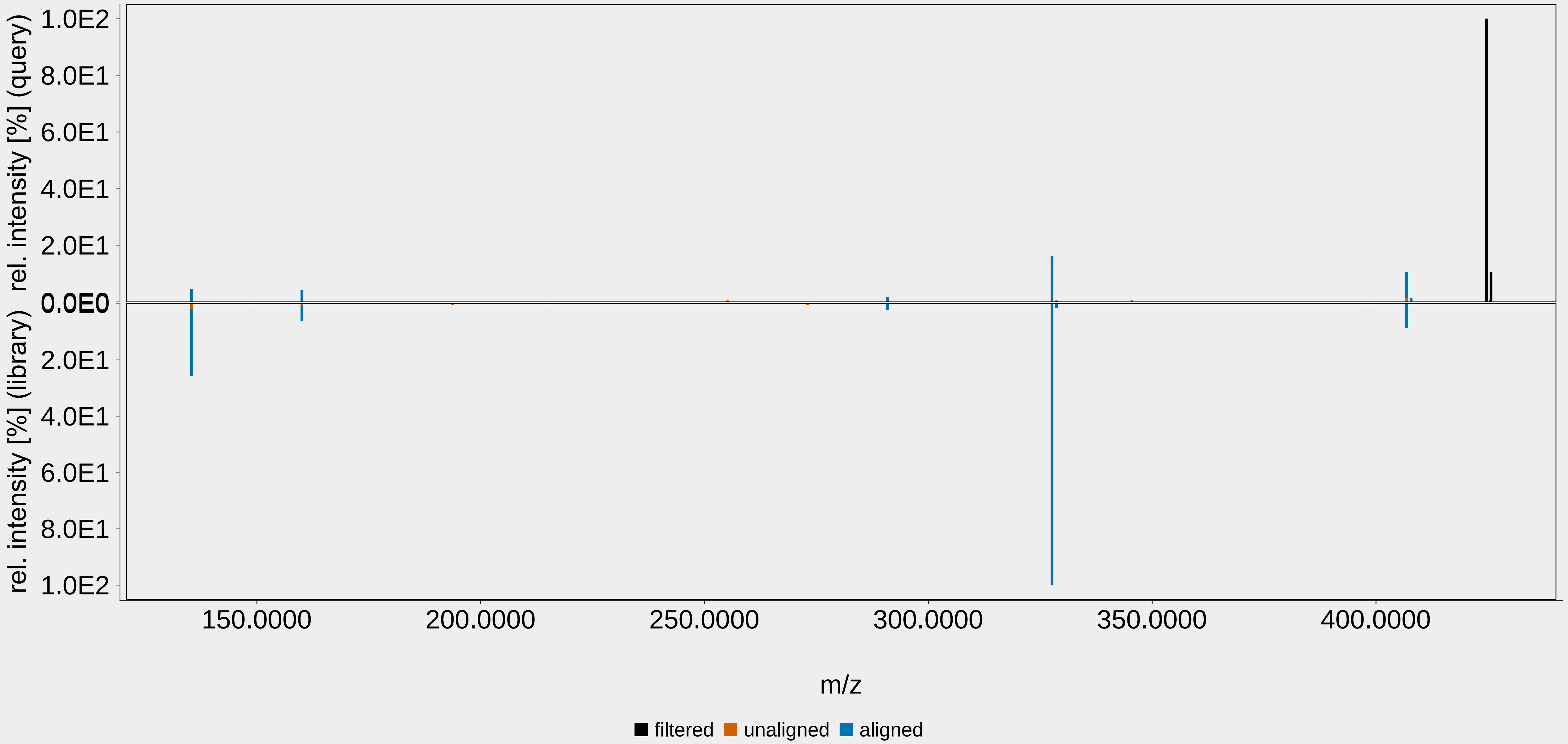

Compound information

Name: Adenosine diphosphate - 30.0 eV

Exact mass: 0.0

Ion type: M-H

Precursor m/z: 426.022

Charge: 1

Polarity: Negative

SMILES:

Instrument information

Instrument type: Orbitrap

Ion source: ESI

Ms level: 2

Acquisition: Isolated

Database links

CAS:

Other information

Principal investigator: PI

Data collector: Data Collector

Entry ID: CCMSLIB00006124450

PG(16:0/18:1); [M-H]- C40H76O10P1 **0.859**

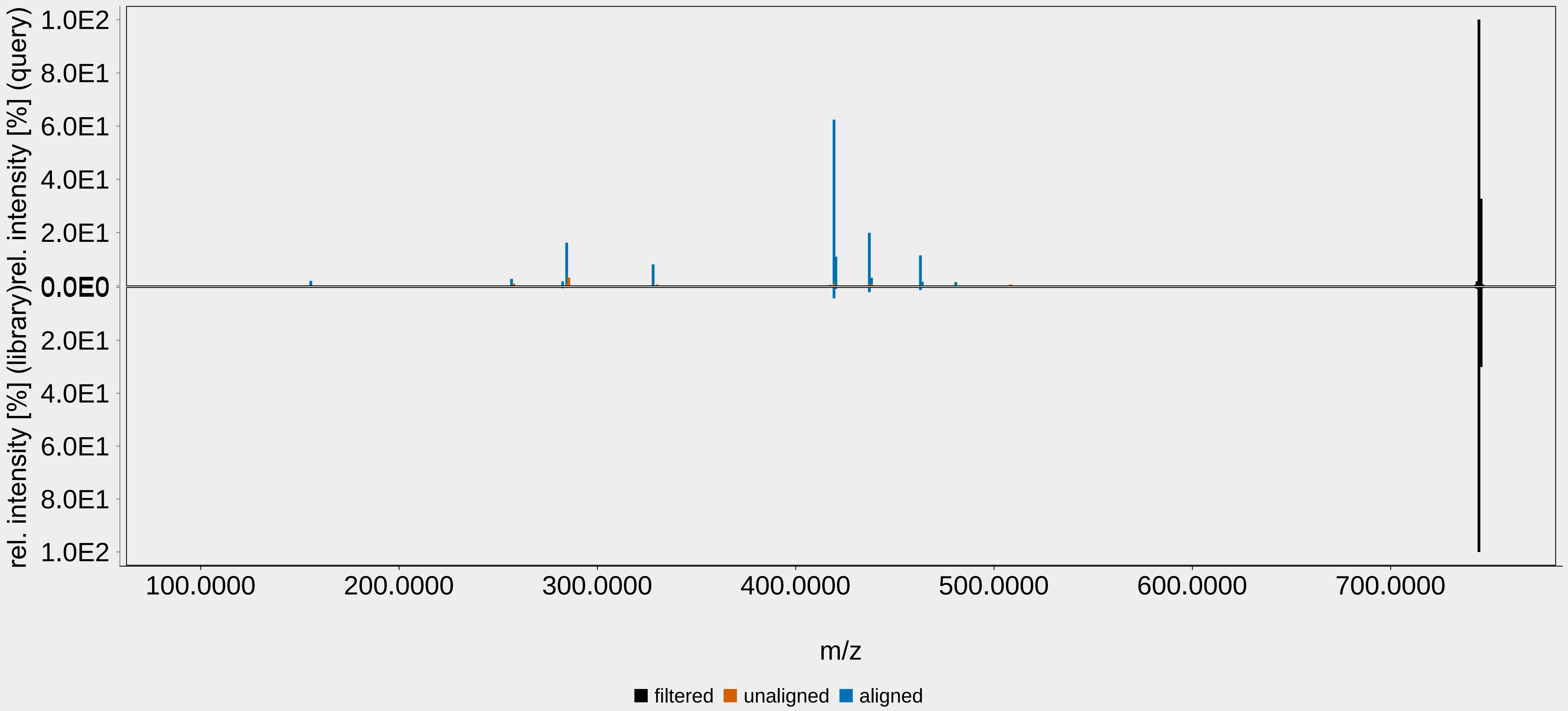

Compound information

Name: PG(16:0/18:1); [M-H]- C40H76O10P1

Exact mass: 0.0

Ion type: M-H

Precursor m/z: 747.518

Charge: 1

Polarity: Negative

Instrument information

Instrument type: HCD; Velos

Ion source: LC-ESI

Ms level: 2

Acquisition: Commercial

Database links

Other information

Principal investigator: Thomas Metz

Data collector: Thomas Metz

Entry ID: CCMSLIB00010063005

PG(16:0/18:1); [M-H]- C40H76O10P1 **0.864**

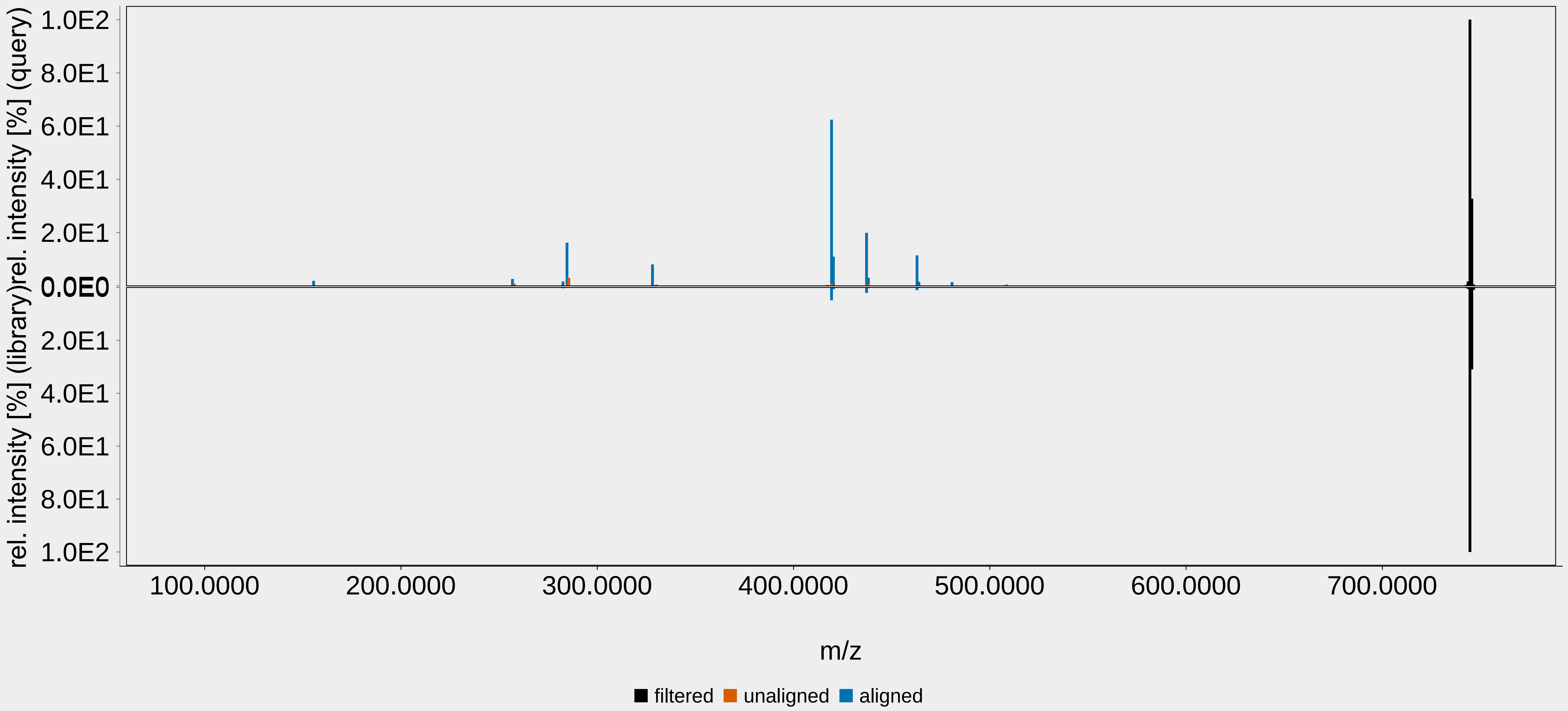

Compound information

Name: PG(16:0/18:1); [M-H]- C40H76O10P1

Exact mass: 0.0

Ion type: M-H

Precursor m/z: 747.518

Charge: 1

Polarity: Negative

Instrument information

Instrument type: HCD; Velos

Ion source: LC-ESI

Ms level: 2

Acquisition: Commercial

Database links

Other information

Principal investigator: Thomas Metz

Data collector: Thomas Metz

Entry ID: CCMSLIB00010063728

C11-LAS (STANDARD MIX), C11-linear alkylbenzyl sulfonate, 4-(undecan-5-yl)benzenesulfonic acid

0.874

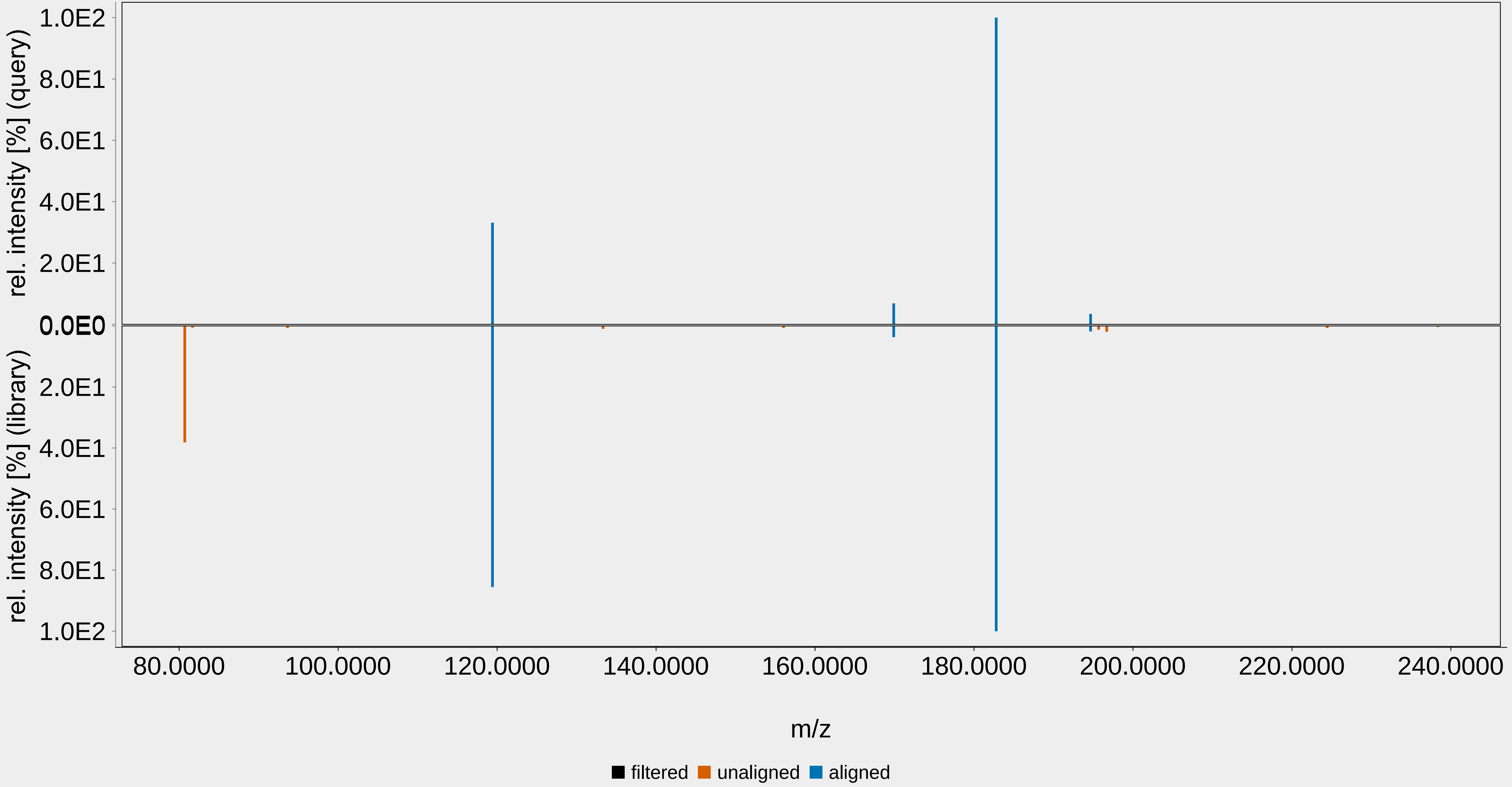

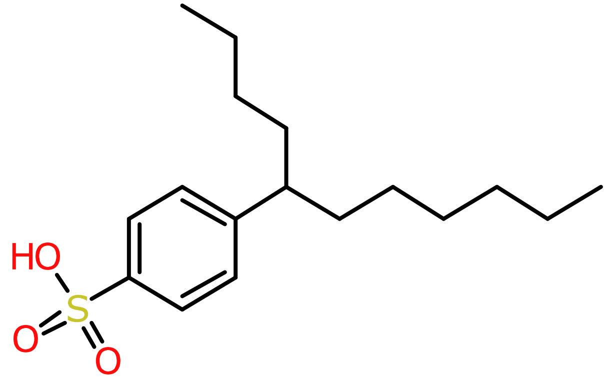

Compound information

Name: C11-LAS (STANDARD MIX), C11-linear

Formula: C17H28O3S

Mol. weight: 312.1759

Exact mass: 312.1759

Ion type: [M-H]-

Precursor m/z: 311.1686

Polarity: negative

InChI:

InChI key:

SMILES:

Instrument information

Instrument type: LC-ESI-QFT

Instrument: Q Exactive Orbitrap Thermo

Ion source: ESI

Ms level: MS2

Collision energy: 90 NCE

Database links

Pubchem: 15593874

MoNA ID: ETS00014

Other information

Principal investigator: E. Schymanski, Dept. of

Data collector: E. Schymanski, Dept. of

C11-LAS (STANDARD MIX) **0.874**

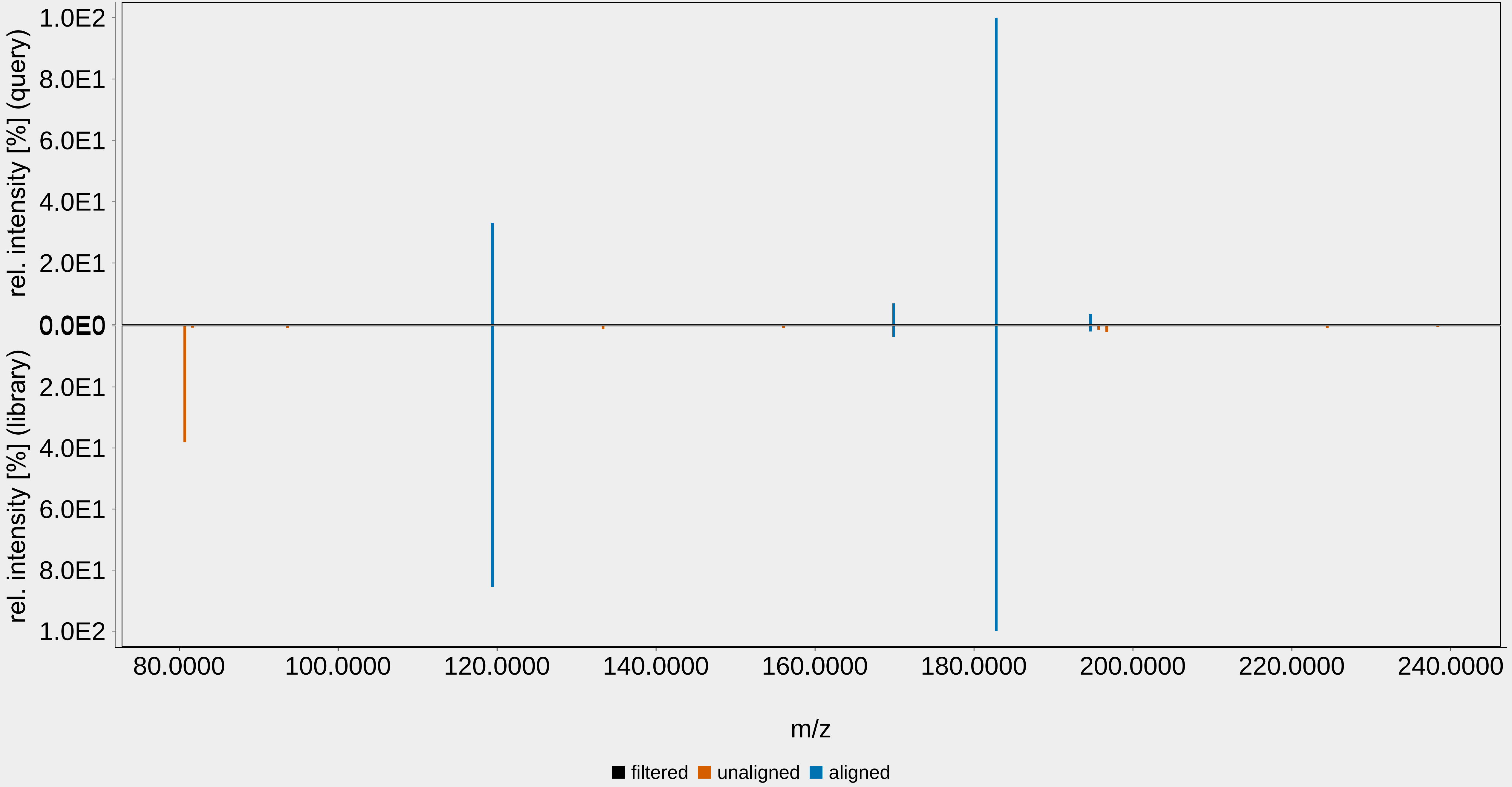

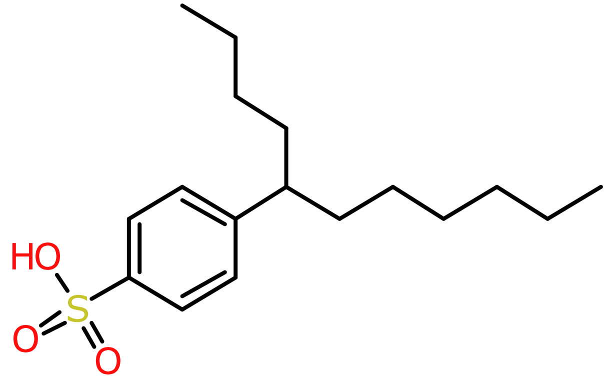

Compound information

Name: C11-LAS (STANDARD MIX)

Formula: C17H28O3S

Exact mass: 312.1759

Ion type: [M-H]-

Precursor m/z: 311.1686

Polarity: NEGATIVE

InChI:

InChI key:

SMILES:

Num peaks: 13

Instrument information

Instrument type: LC-ESI-QFT

Instrument: Q Exactive Orbitrap Thermo

Ms level: MS2

Collision energy: 90 NCE

Database links

Other information

Entry ID:

Comment: Parent=311.1686

PA 18:0_20:4

0.875

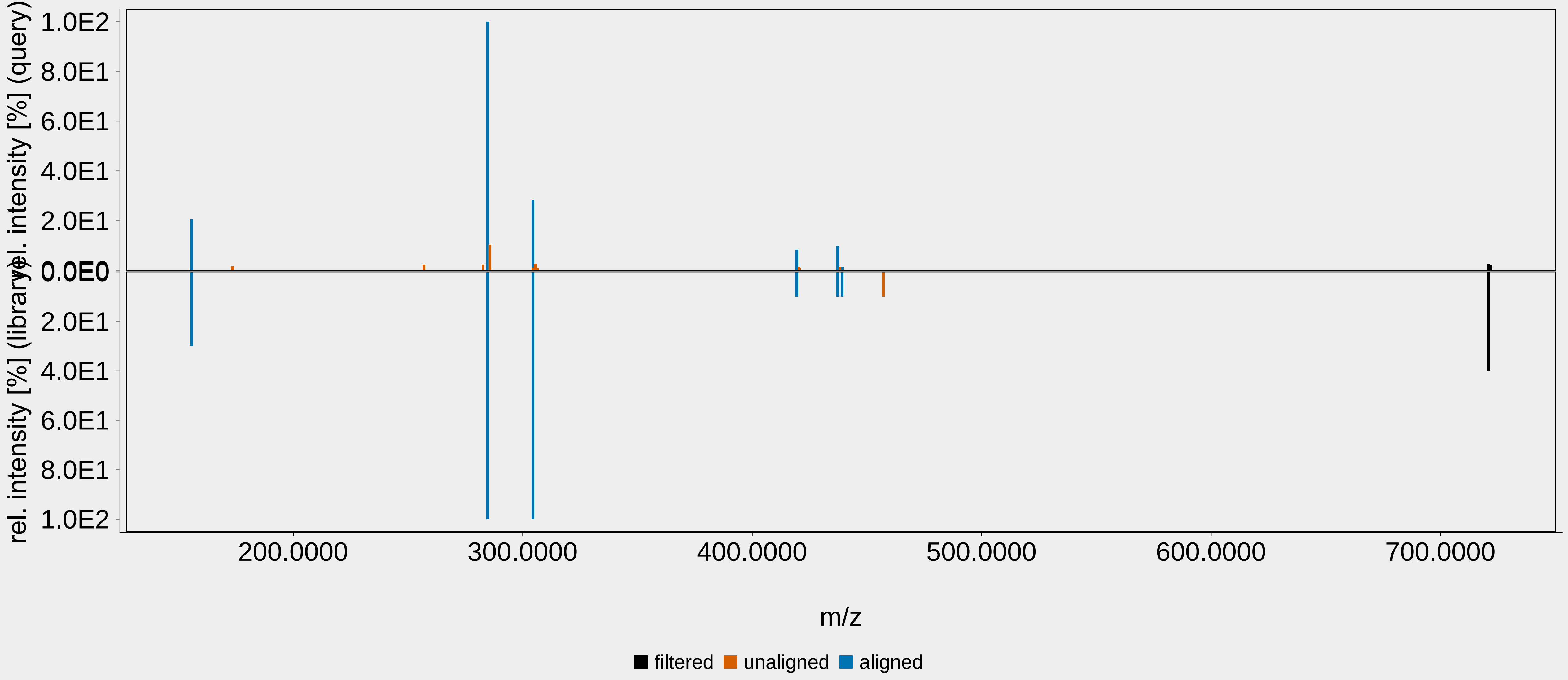

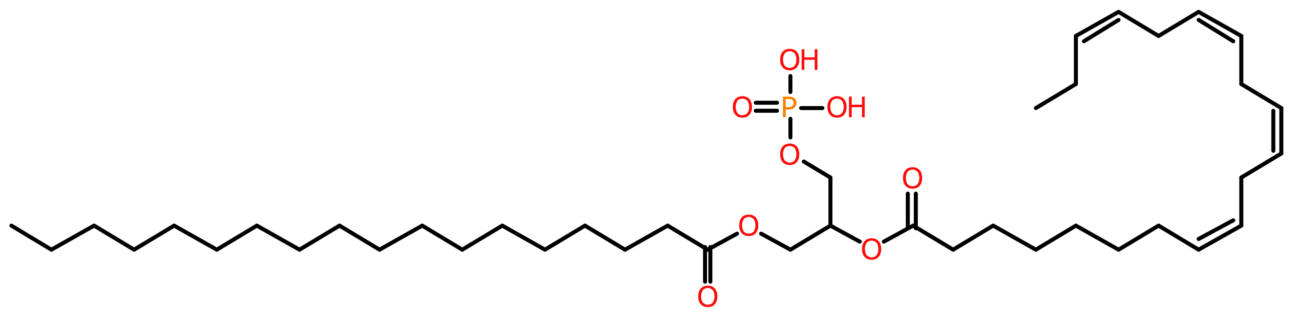

Compound information

Name: PA 18:0_20:4

Formula: C41H73O8P

Ion type: [M-H]-

Precursor m/z: 723.49703

Ccs: 262.1

InChI key:

SMILES:

Num peaks: 8

Instrument information

Database links

Other information

PA(18:0/20:4); [M-H]- C41H72O8P1 0.880

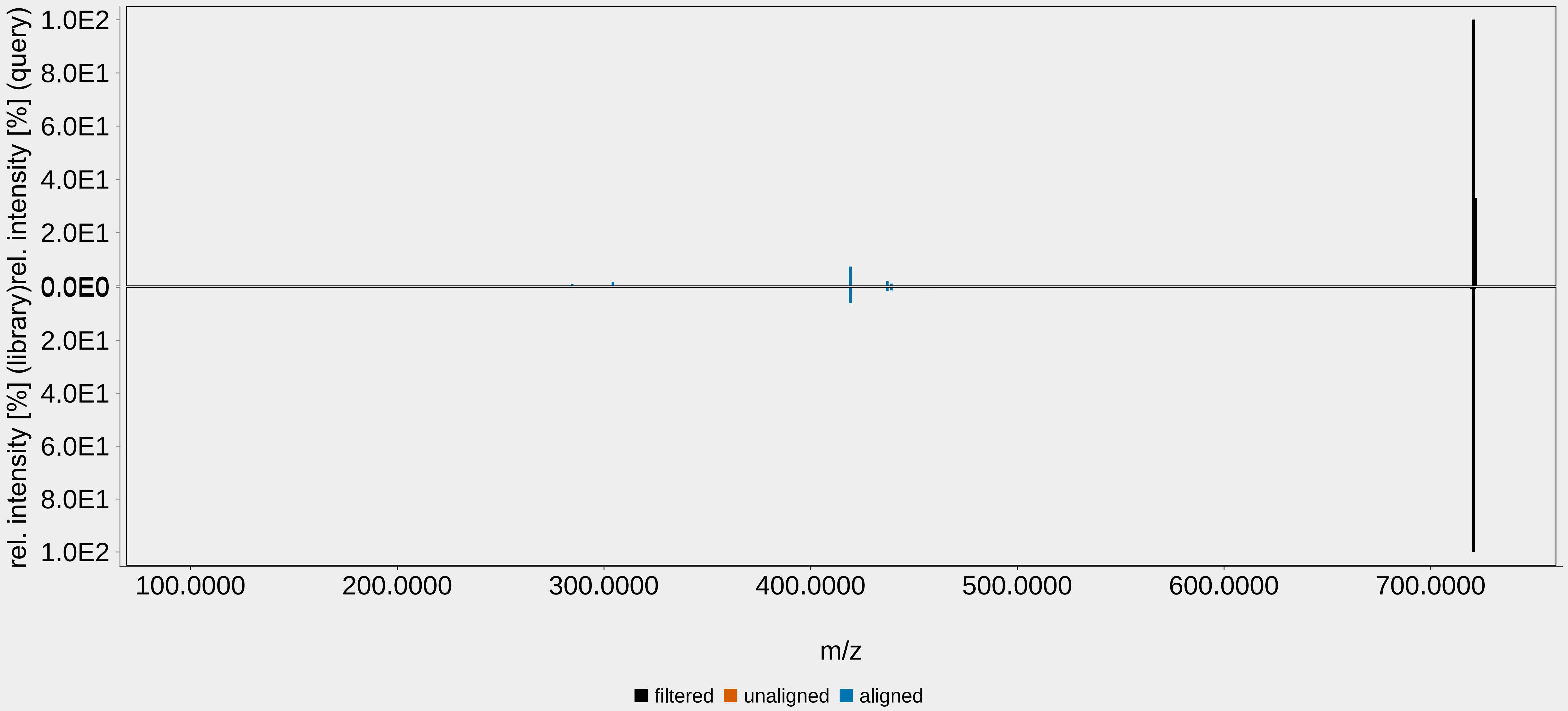

Compound information

Name: PA(18:0/20:4); [M-H]- C41H72O8P1

Exact mass: 0.0

Ion type: M-H

Precursor m/z: 723.496

Charge: 1

Polarity: Negative

Instrument information

Instrument type: HCD; Velos

Ion source: LC-ESI

Ms level: 2

Acquisition: Commercial

Database links

Other information

Principal investigator: Thomas Metz

Data collector: Thomas Metz

Entry ID: CCMSLIB00010065411

[1-hexadecanoyloxy-3-phosphonooxypropan-2-yl]octadec-9-enoate

0.908

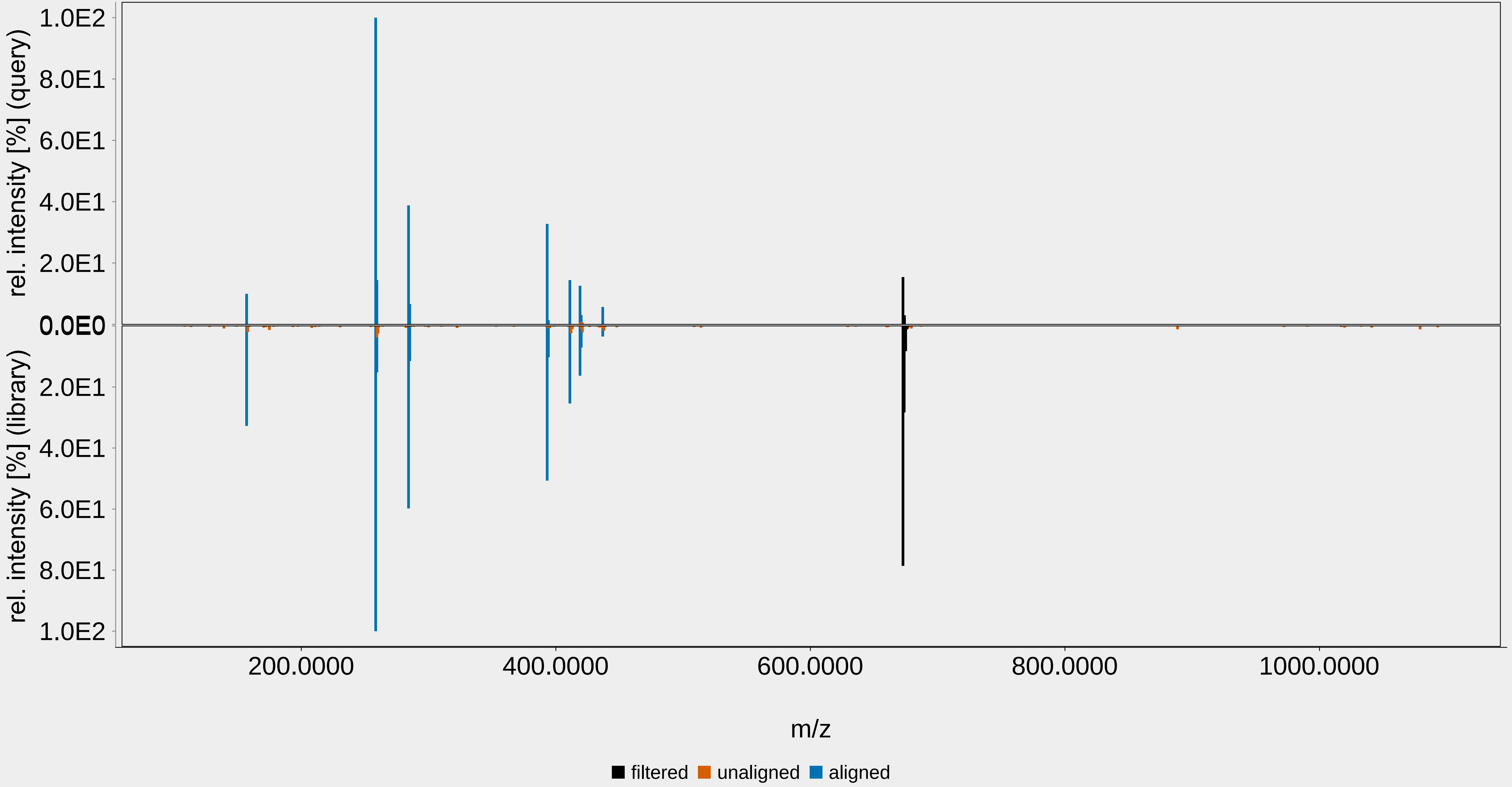

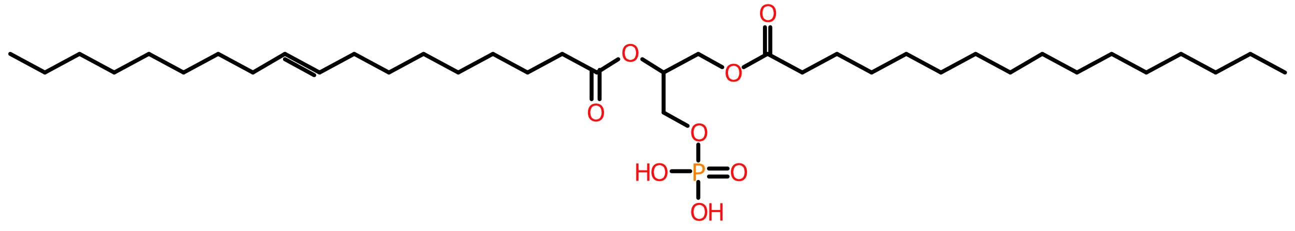

Compound information

Name:

Formula: C37H71O8P

Mol. weight: 674.489

Exact mass: 674.489

Ion type: [M-H]-

Precursor m/z: 673.481

Polarity: negative

InChI:

InChI key:

SMILES:

Instrument information

Instrument: qTof

Ms level: MS2

Database links

Other information

Principal investigator: O Laprevote, UMR 8038

Data collector: O Laprevote, UMR 8038

PA 16:0_18:1

0.935

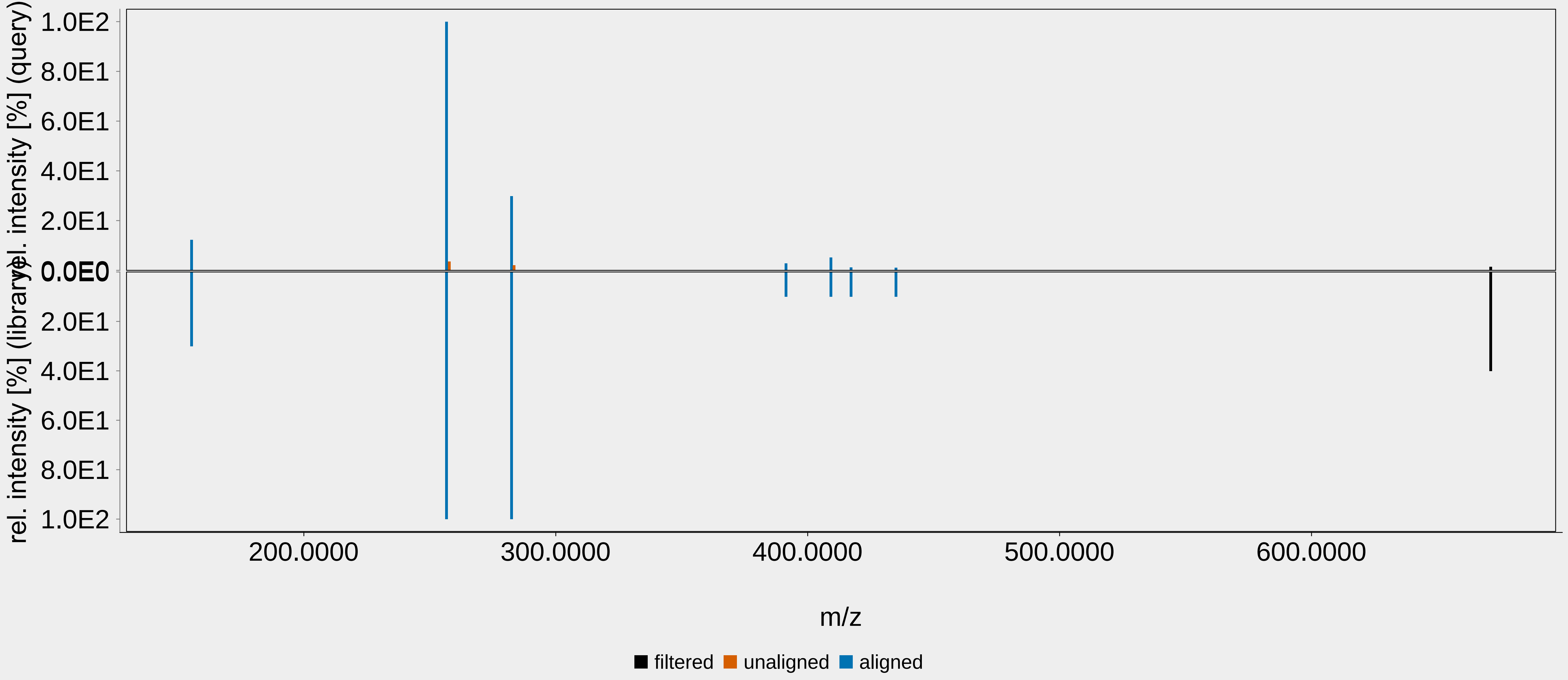

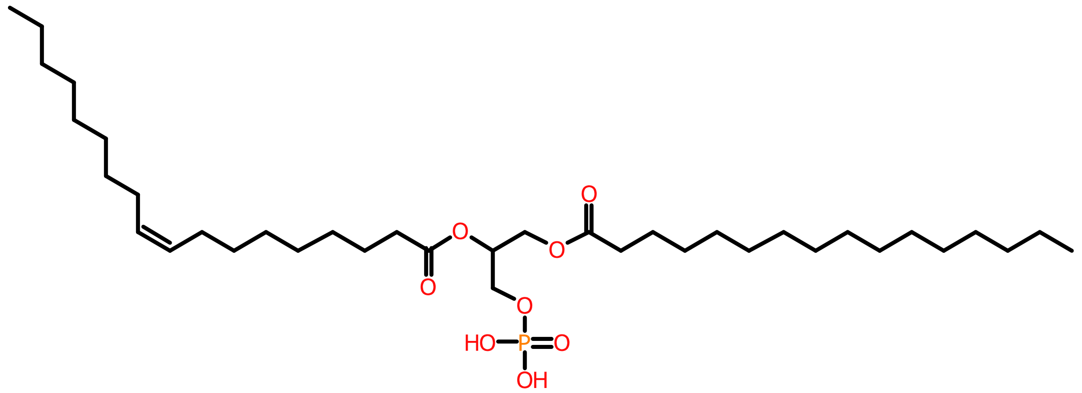

Compound information

Name: PA 16:0_18:1

Formula: C37H71O8P

Ion type: [M-H]-

Precursor m/z: 673.48138

Ccs: 252.18

InChI key:

SMILES:

Num peaks: 8

Instrument information

Database links

Other information

PA 22:4_22:6

0.872

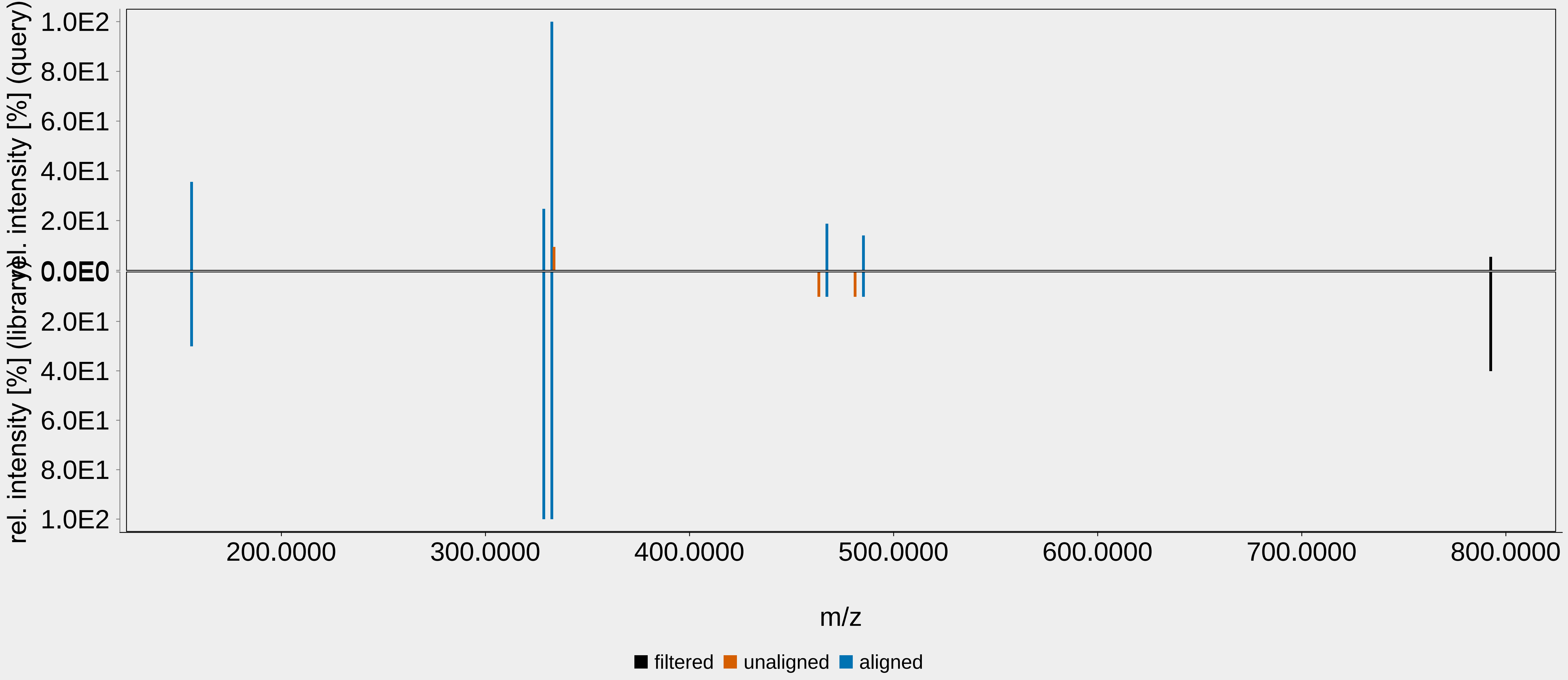

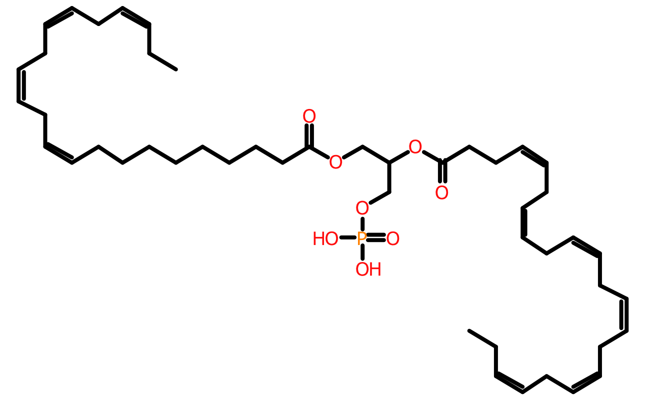

Compound information

Name: PA 22:4_22:6

Formula: C47H73O8P

Ion type: [M-H]-

Precursor m/z: 795.49703

Ccs: 272.68

InChI key:

SMILES:

Num peaks: 8

Instrument information

Database links

Other information

Spectral Match to 1-Oleoyl-L-.alpha.-lysophosphatidic acid from NIST14 **0.830**

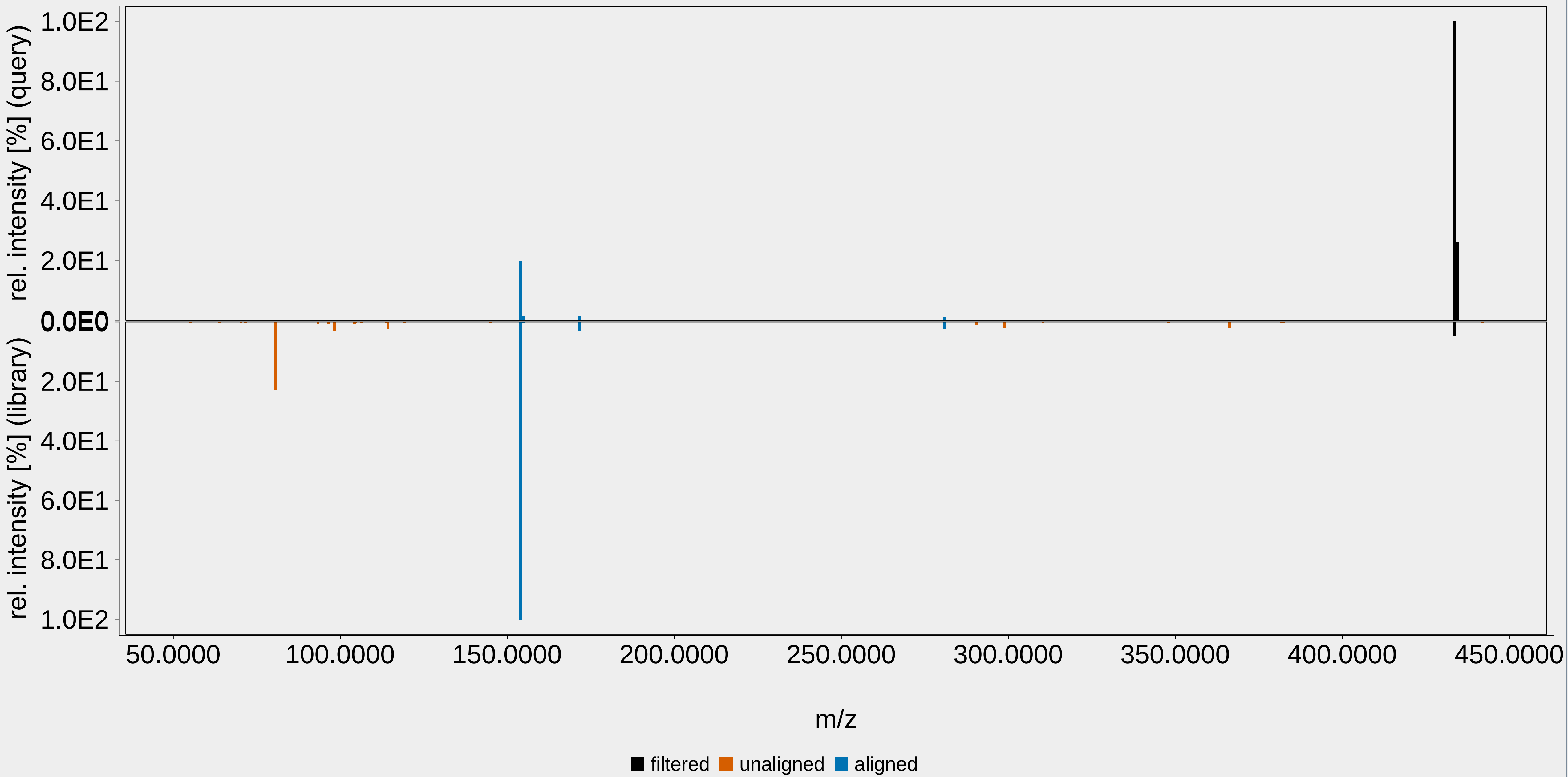

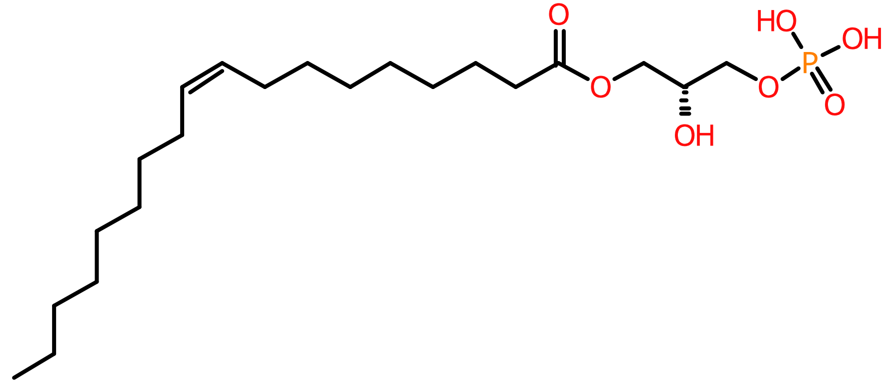

Compound information

Name: Spectral Match to

Exact mass: 436.259

Ion type: M-H

Precursor m/z: 435.252

Charge: 1

Polarity: Negative

InChI:

SMILES:

Instrument information

Instrument type: qTof

Ion source: ESI

Ms level: 2

Acquisition: Isolated

Database links

CAS: 22002875

Other information

Principal investigator: Data from Kevin Bush

Data collector: Data deposited by fevargas

Entry ID: CCMSLIB00003137366

PS 18:1_20:1

0.733

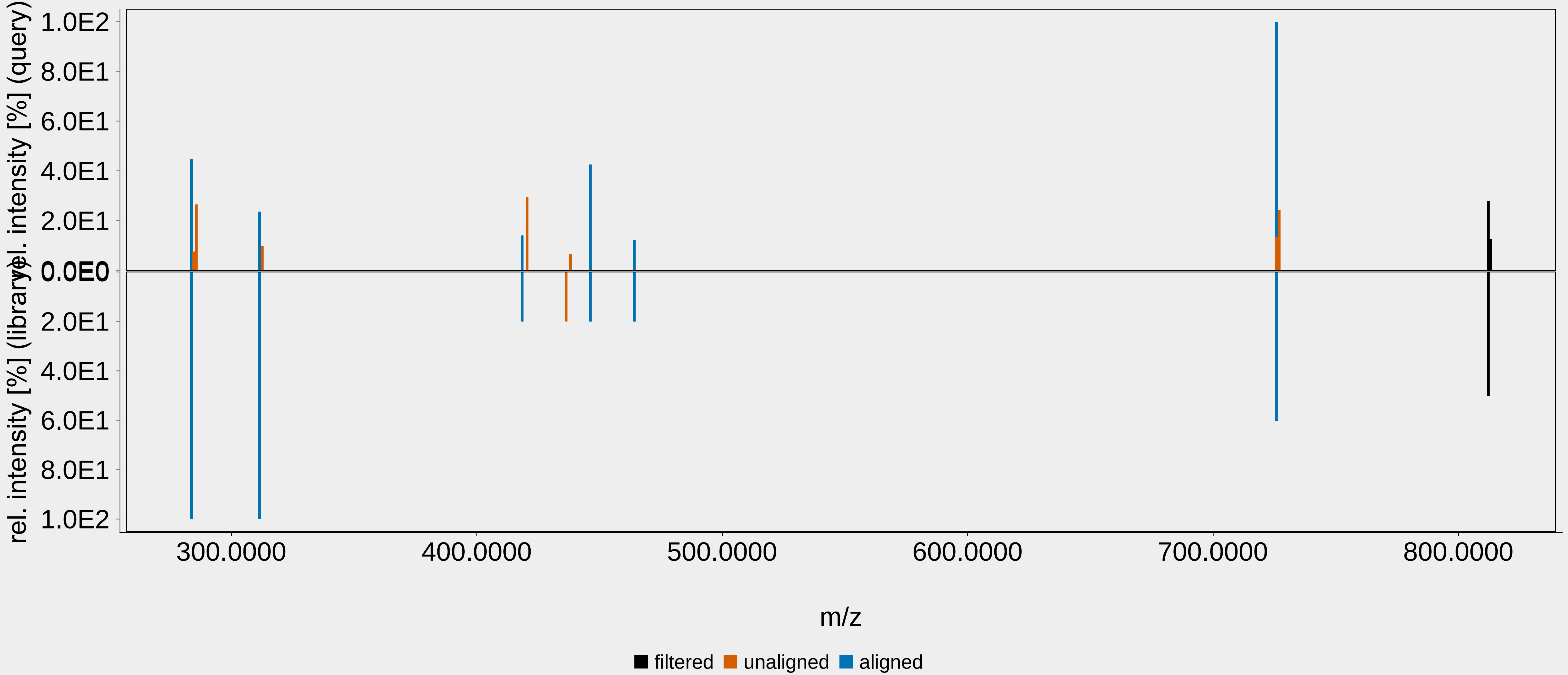

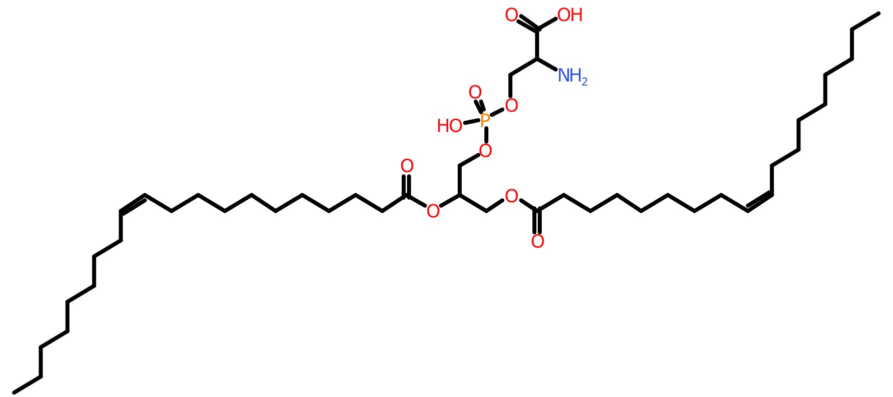

Compound information

Name: PS 18:1_20:1

Formula: C44H82NO10P

Ion type: [M-H]-

Precursor m/z: 814.56036

Ccs: 282.14

InChI key:

SMILES:

Num peaks: 8

Instrument information

Database links

Other information

PS 18:0_20:1 **0.783**

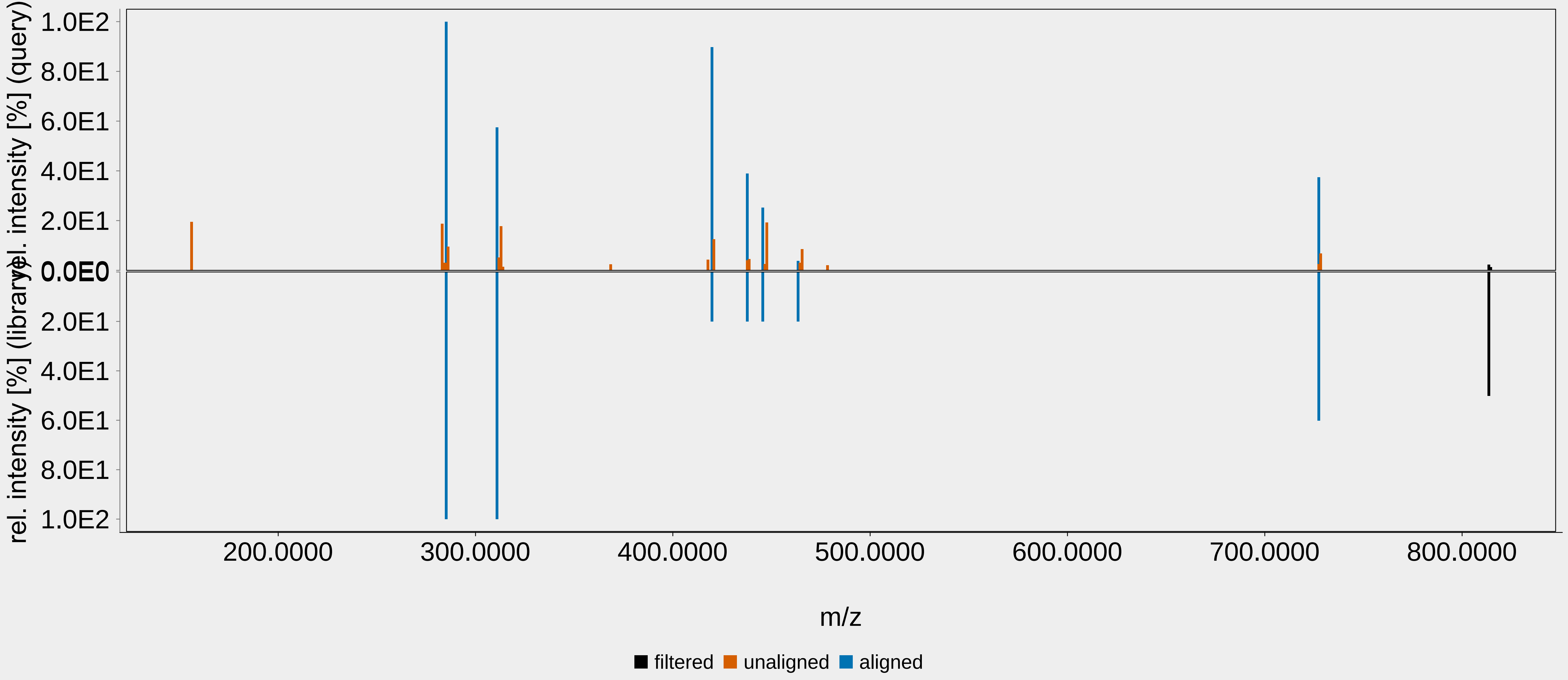

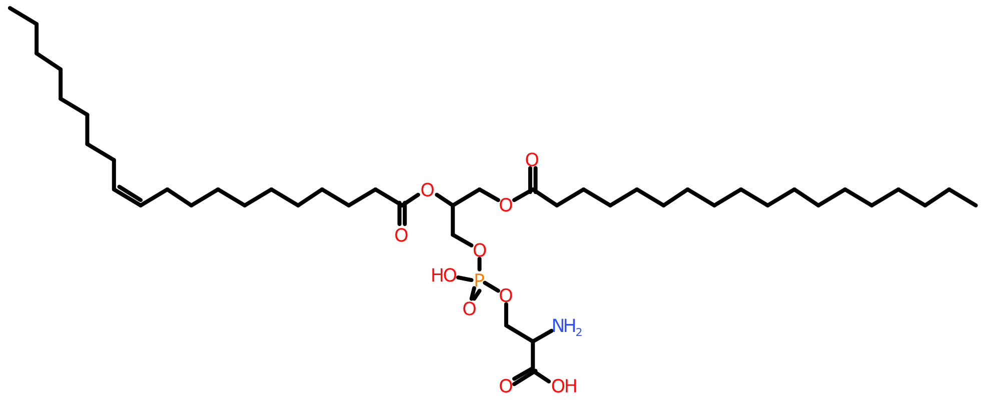

Compound information

Name: PS 18:0_20:1

Formula: C44H84NO10P

Ion type: [M-H]-

Precursor m/z: 816.57601

Ccs: 284.42

InChI key: KVMIIDXBXADXIM-ZPHPHTNENA-N

SMILES:

Num peaks: 8

Instrument information

Database links

Other information

PS(22:6/0:0); [M-H]- C28H43N1O9P1 **0.753**

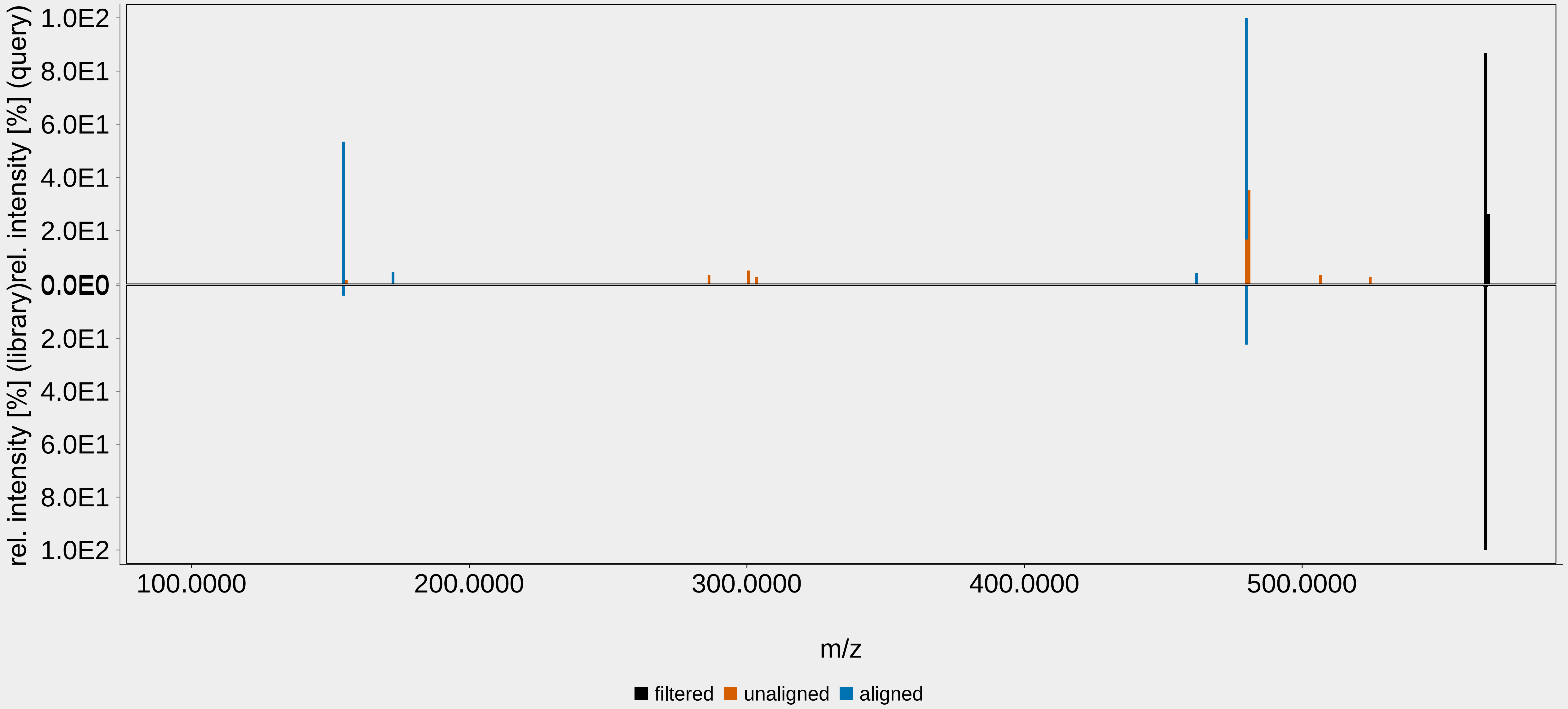

Compound information

Name: PS(22:6/0:0); [M-H]- C28H43N1O9P1

Exact mass: 0.0

Ion type: M-H

Precursor m/z: 568.268

Charge: 1

Polarity: Negative

Instrument information

Instrument type: HCD; Velos

Ion source: LC-ESI

Ms level: 2

Acquisition: Commercial

Database links

Other information

Principal investigator: Thomas Metz

Data collector: Thomas Metz

Entry ID: CCMSLIB00010087165

PE(22:4/22:6); [M-H]- C49H77N1O8P1

0.908

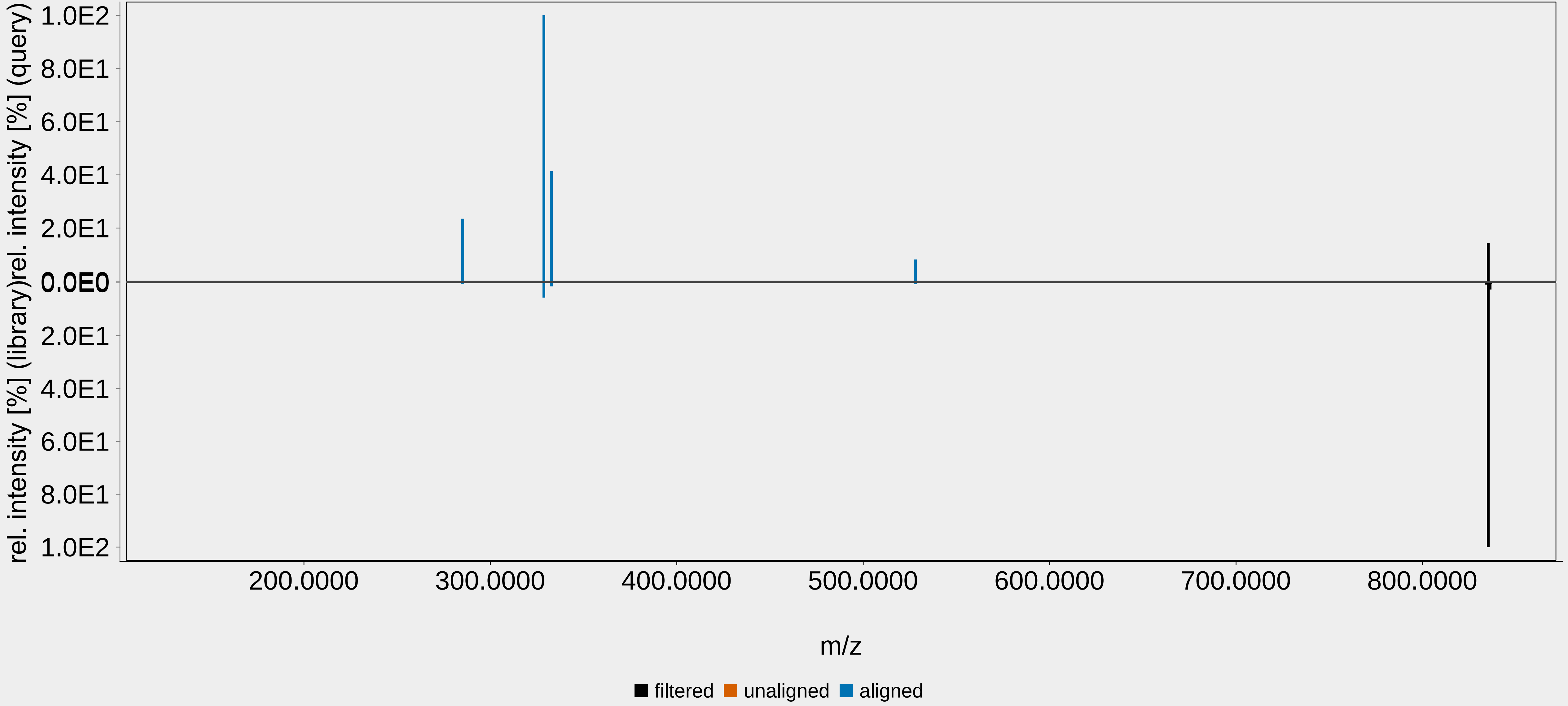

Compound information

Name: PE(22:4/22:6); [M-H]- C49H77N1O8P1

Exact mass: 0.0

Ion type: M-H

Precursor m/z: 838.539

Charge: 1

Polarity: Negative

Instrument information

Instrument type: HCD; Velos

Ion source: LC-ESI

Ms level: 2

Acquisition: Commercial

Database links

Other information

Principal investigator: Thomas Metz

Data collector: Thomas Metz

Entry ID: CCMSLIB00010063765

PE(22:4/22:6); [M-H]- C49H77N1O8P1

0.900

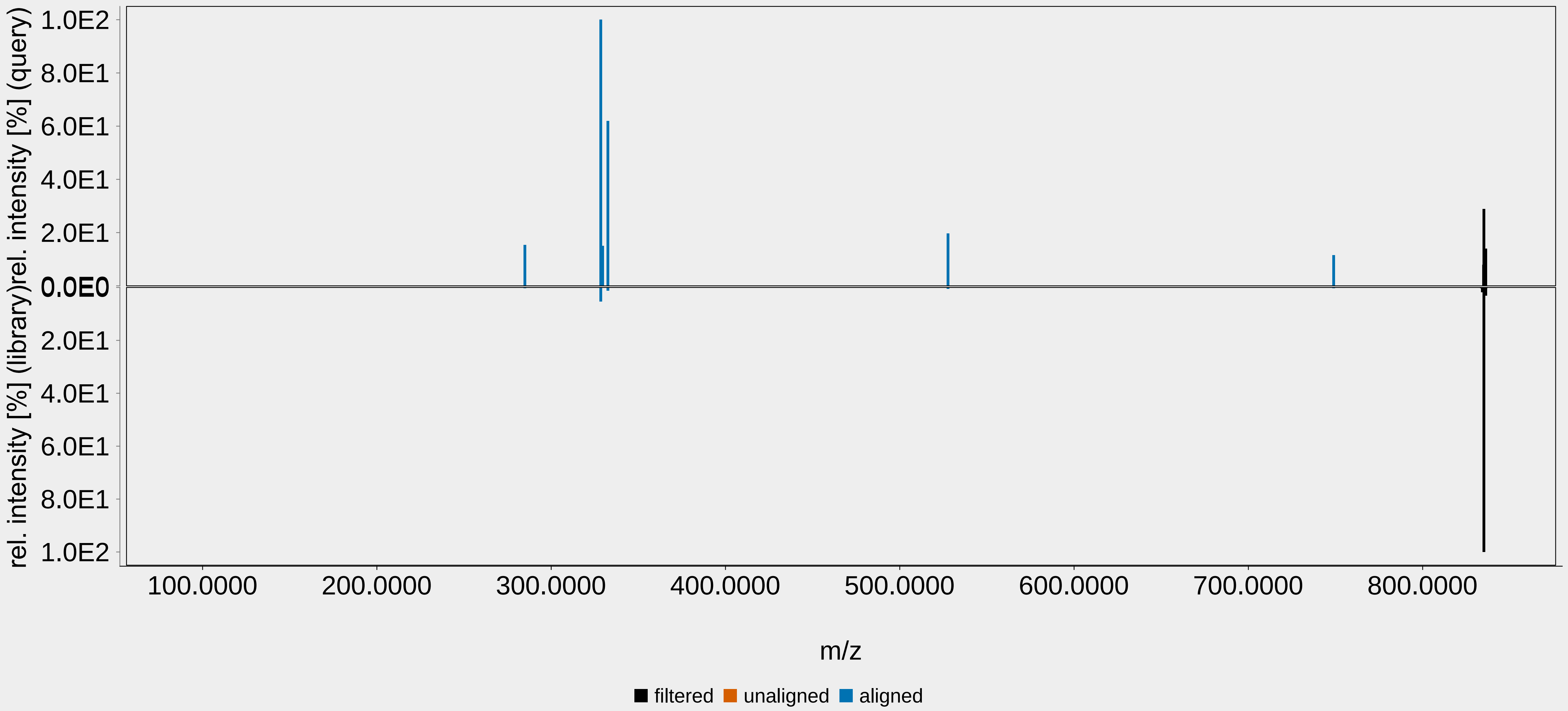

Compound information

Name: PE(22:4/22:6); [M-H]- C49H77N1O8P1

Exact mass: 0.0

Ion type: M-H

Precursor m/z: 838.539

Charge: 1

Polarity: Negative

Instrument information

Instrument type: HCD; Velos

Ion source: LC-ESI

Ms level: 2

Acquisition: Commercial

Database links

Other information

Principal investigator: Thomas Metz

Data collector: Thomas Metz

Entry ID: CCMSLIB00010086991

PI 36:2 0.716

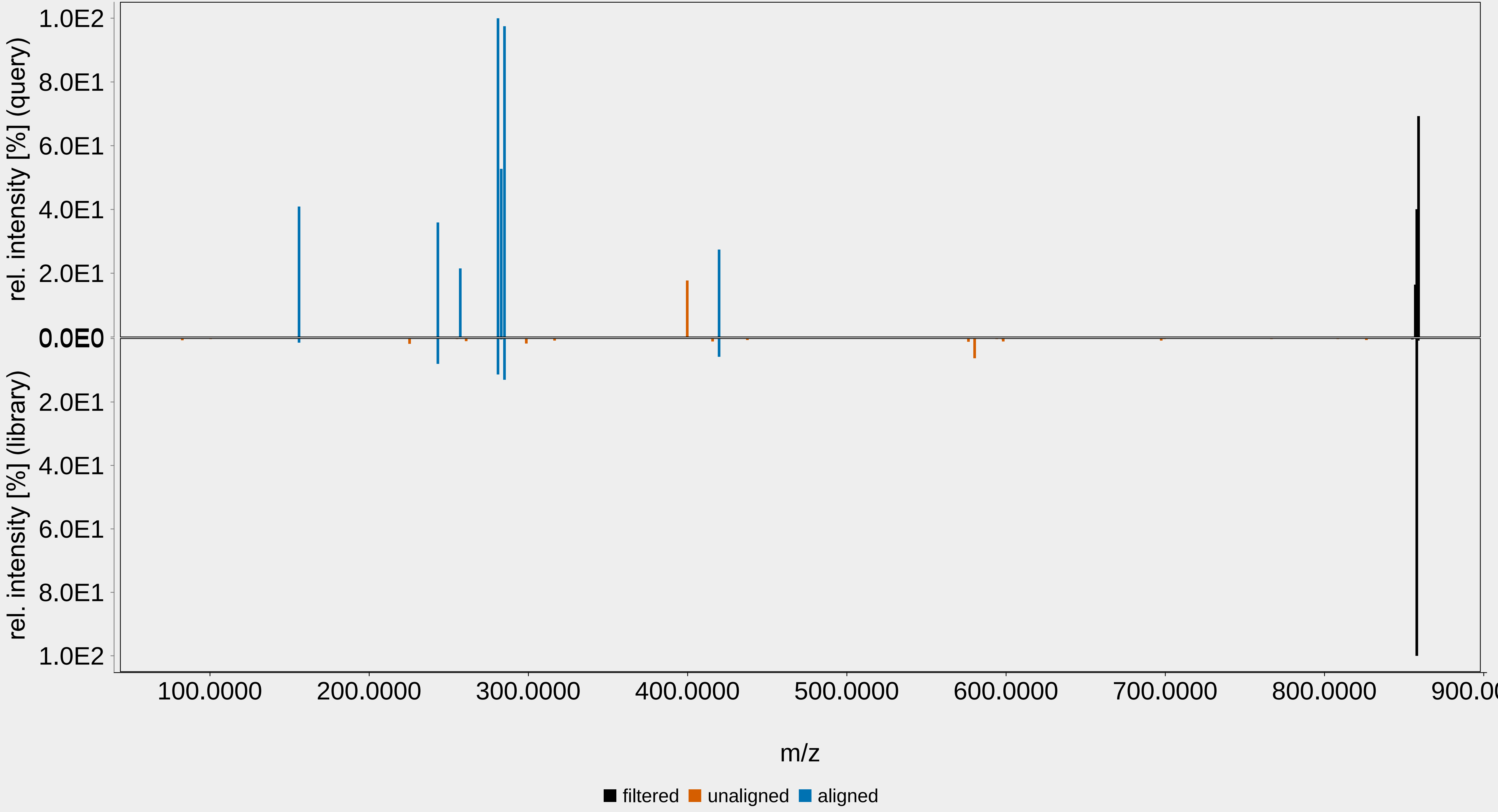

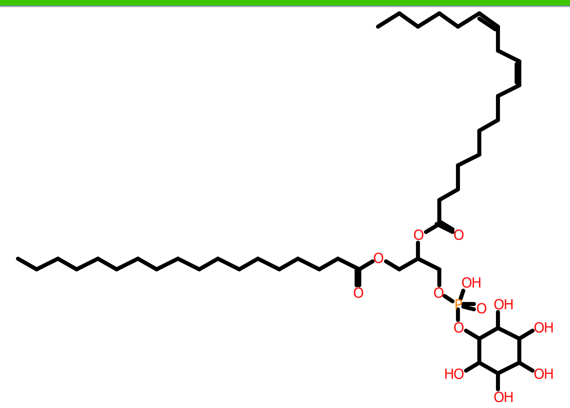

Compound information

Name: PI 36:2

Formula: C45H83O13P

Mol. weight: 863.122

Exact mass: 863.122

Ion type: [M-H]-

Precursor m/z: 861.5504

RT: 8.773114

Polarity: negative

InChI:

InChI key:

SMILES:

Instrument information

Instrument type: LC-ESI-QTOF

Instrument: AB Sciex TripleTOF 5600+ system

Ms level: MS2

Collision energy: 45V

Database links

Pubchem: 138199526

MoNA ID: LQB00692

Other information

Principal investigator: Kazutaka Ikeda, Hiroshi

Data collector: Kazutaka Ikeda, Hiroshi

PI 36:2

0.716

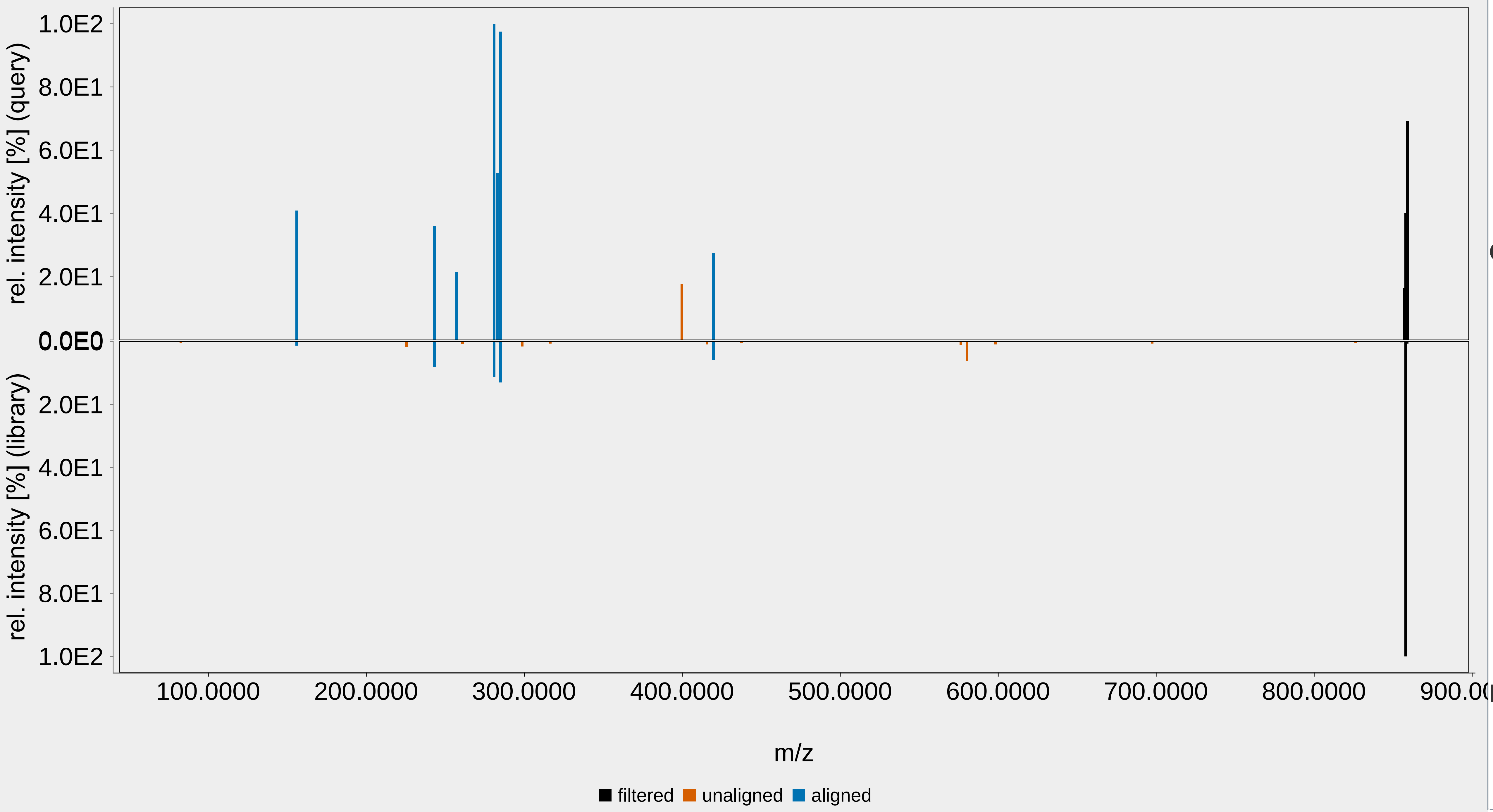

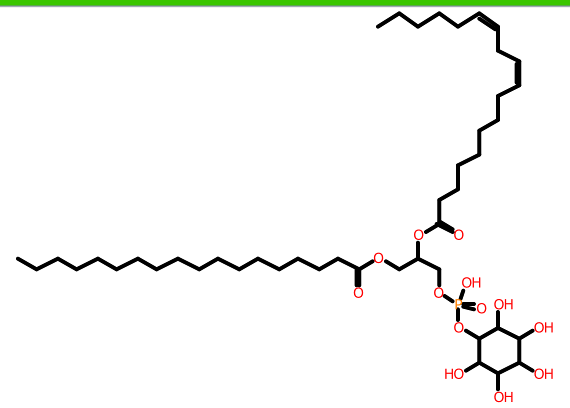

Compound information

Name: PI 36:2

Formula: C45H83O13P

Mol. weight: 863.122

Exact mass: 863.122

Ion type: [M-H]-

Precursor m/z: 861.5504

RT: 8.773114

Polarity: negative

InChI:

InChI key:

SMILES:

Instrument information

Instrument type: LC-ESI-QTOF

Instrument: AB Sciex TripleTOF 5600+ system

Ms level: MS2

Collision energy: 45V

Database links

Pubchem: 138199526

MoNA ID: LQB00306

Other information

Principal investigator: Kazutaka Ikeda, Hiroshi

Data collector: Kazutaka Ikeda, Hiroshi
