# Supplementary material for: On-tissue dataset-dependent MALDI-TIMS-MS2 bioimaging
Source: Nat Commun. 2023 Nov 18;14:7495. doi: 10.1038/s41467-023-43298-9 (PMC10657435; doi:10.1038/s41467-023-43298-9)
Supplement: Supplementary file 3 — Reporting Summary [file 41467_2023_43298_MOESM3_ESM.pdf]

nature portfolio

Corresponding author(s): Robin Schmid

Last updated by author(s): Oct 26, 2023

Reporting Summary

Nature Portfolio wishes to improve the reproducibility of the work that we publish. This form provides structure for consistency and transparency in reporting. For further information on Nature Portfolio policies, see our Editorial Policies and the Editorial Policy Checklist.

Statistics

For all statistical analyses, confirm that the following items are present in the figure legend, table legend, main text, or Methods section.

n/a	Confirmed	
[x]	[ ]	The exact sample size (*n*) for each experimental group/condition, given as a discrete number and unit of measurement
[x]	[ ]	A statement on whether measurements were taken from distinct samples or whether the same sample was measured repeatedly
[x]	[ ]	The statistical test(s) used AND whether they are one- or two-sided<br>*Only common tests should be described solely by name; describe more complex techniques in the Methods section.*
[x]	[ ]	A description of all covariates tested
[x]	[ ]	A description of any assumptions or corrections, such as tests of normality and adjustment for multiple comparisons
[x]	[ ]	A full description of the statistical parameters including central tendency (e.g. means) or other basic estimates (e.g. regression coefficient) AND variation (e.g. standard deviation) or associated estimates of uncertainty (e.g. confidence intervals)
[x]	[ ]	For null hypothesis testing, the test statistic (e.g. *F*, *t*, *r*) with confidence intervals, effect sizes, degrees of freedom and *P* value noted<br>*Give P values as exact values whenever suitable.*
[x]	[ ]	For Bayesian analysis, information on the choice of priors and Markov chain Monte Carlo settings
[x]	[ ]	For hierarchical and complex designs, identification of the appropriate level for tests and full reporting of outcomes
[x]	[ ]	Estimates of effect sizes (e.g. Cohen's *d*, Pearson's *r*), indicating how they were calculated

Our web collection on statistics for biologists contains articles on many of the points above.

Software and code

Policy information about availability of computer code

Data collection	timsControl 4.1 (prototype, Bruker Daltonics GmbH & Co. KG, Bremen, Germany), flexImaging 7.2 (Bruker Daltonics GmbH & Co. KG, Bremen, Germany), BZ-II Viewer Software (Keyence, Osaka, Japan), BZ-II Analyser Software (Keyence, Osaka, Japan)
Data analysis	MZmine 3.8 (https://github.com/mzmine/mzmine3/releases/tag/v3.8.0), SIMSEF 1.0 (https://github.com/SteffenHeu/simsef_py, https://doi.org/10.5281/zenodo.8009939), Python 3.8

For manuscripts utilizing custom algorithms or software that are central to the research but not yet described in published literature, software must be made available to editors and reviewers. We strongly encourage code deposition in a community repository (e.g. GitHub). See the Nature Portfolio guidelines for submitting code & software for further information.

Data

Policy information about availability of data

All manuscripts must include a data availability statement. This statement should provide the following information, where applicable:

- Accession codes, unique identifiers, or web links for publicly available datasets
- A description of any restrictions on data availability
- For clinical datasets or third party data, please ensure that the statement adheres to our policy

The mass spectrometry imaging raw data generated in this study have been deposited in the MassIVE database under accession code MSV000092935 [doi:10.25345/C5S17T379]. GNPS FBMN molecular networks and annotations are available at https://gnps.ucsd.edu/ProteoSAFe/status.jsp?task=9a6f6b34367f4ff69d81c9efe6aedd03.

Research involving human participants, their data, or biological material

Policy information about studies with human participants or human data. See also policy information about sex, gender (identity/presentation), and sexual orientation and race, ethnicity and racism.

Reporting on sex and gender	n/a
Reporting on race, ethnicity, or other socially relevant groupings	n/a
Population characteristics	n/a
Recruitment	n/a
Ethics oversight	n/a

Note that full information on the approval of the study protocol must also be provided in the manuscript.

Field-specific reporting

Please select the one below that is the best fit for your research. If you are not sure, read the appropriate sections before making your selection.

[x] Life sciences [ ] Behavioural & social sciences [ ] Ecological, evolutionary & environmental sciences

For a reference copy of the document with all sections, see nature.com/documents/nr-reporting-summary-flat.pdf

Life sciences study design

All studies must disclose on these points even when the disclosure is negative.

Sample size	No sample size calculation was performed. The sample size of n=4 was chosen to demonstrate the reproducibility of the developed algorithms across multiple measurements. The study itself focuses on the SIMSEF acquisition technique to enhance the annotation confidence in MALDI imaging studies.
Data exclusions	No data was excluded.
Replication	MZmine processing parameters and steps were exported to batch files. The batch files successfully reproduce the obtained results of the data analysis. The obtained SIMSEF schnedules of the same dataset match on every replication attempt. The SIMSEF scheduling algorithm was tested on four tissue sections. For each tissue section, the algorithm reproduces the MS2 schedules if the input parameters are the same. Between the different tissues, comparable results are achieved with regard to annotation numbers.
Randomization	The study focuses on data acquisition techniques and did not require randomization, because no biological meaning is derived in this study. All datasets originate from the same animal and represent consecutive thin-sections.
Blinding	The study focuses on data acquisition techniques and did not require blinding, because no biological meaning is derived in this study. All datasets originate from the same animal and represent consecutive thin-sections.

Reporting for specific materials, systems and methods

We require information from authors about some types of materials, experimental systems and methods used in many studies. Here, indicate whether each material, system or method listed is relevant to your study. If you are not sure if a list item applies to your research, read the appropriate section before selecting a response.

Materials & experimental systems

n/a	Involved in the study
☒	☐ Antibodies
☒	☐ Eukaryotic cell lines
☒	☐ Palaeontology and archaeology
☒	☐ Animals and other organisms
☒	☐ Clinical data
☒	☐ Dual use research of concern
☒	☐ Plants

Methods

n/a	Involved in the study
☒	☐ ChIP-seq
☒	☐ Flow cytometry
☒	☐ MRI-based neuroimaging

Plants

Seed stocks

n/a

Novel plant genotypes

n/a

Authentication

n/a
