# Supplementary material for: On-tissue dataset-dependent MALDI-TIMS-MS2 bioimaging
Source: Nat Commun. 2023 Nov 18;14:7495. doi: 10.1038/s41467-023-43298-9 (PMC10657435; doi:10.1038/s41467-023-43298-9)
Supplement: Supplementary file 1 — Supplementary Information [file 41467_2023_43298_MOESM1_ESM.pdf]

Supplementary information

On-tissue dataset-dependent MALDI-TIMS-MS^2 bioimaging

Steffen Heuckeroth[1], Arne Behrens[2], Carina Wolf[1], Arne Fütterer[2], Ilona D. Nordhorn[1], Katharina Kronenberg[1], Corinna Brungs[3], Ansgar Korf[2], Henning Richter[4], Astrid Jeibmann[5], Uwe Karst[1], Robin Schmid[3,6]*

[1]Institute of Inorganic and Analytical Chemistry, University of Münster, Münster, Germany
[2]Bruker Daltonics GmbH & Co. KG, Bremen, Germany
[3]Institute of Organic Chemistry and Biochemistry of the Czech Academy of Sciences, Prague, Czech Republic
[4]Clinic for Diagnostic Imaging, Diagnostic Imaging Research Unit (DIRU), University of Zurich, Zürich, Switzerland
[5]Institute of Neuropathology, University Hospital Münster, Münster, Germany
[6]Collaborative Mass Spectrometry Innovation Center, University of California San Diego, La Jolla, USA

*corresponding author Robin Schmid, robin.schmid@uochb.cas.cz

Supplementary Note S1: SIMSEF experiment. 2
Supplementary Note S2: Configuring the SIMSEF-MS^2 acquisition. 2
Figure S 1: Flow diagram of the SIMSEF algorithm. 3
Figure S 2: Spectral scheduling procedure of the SIMSEF algorithm. 4
Figure S 3: Distribution of multiple MS^2 spots per precursor and collision energy. 5
Figure S 4: Screenshot of the SIMSEF scheduling dialog from the corresponding MZmine module. 6
Figure S 5: Additional spectral library matches of small metabolites. 7
Figure S 6: The same FBMN from Figure 4, but nodes coloured by their feature's ion mobility. 8
Figure S 7: Imaging specific All MS/MS visualiser in MZmine 3. 9
Figure S 8: SIMSEF data allows recognition of chimeric precursor features by annotating multiple compounds and lipid species in the same MS^2 scans. 10
Figure S 9: Distribution of the cosine similarity of multiple MS^2 spectra assigned to the same TIMS-MS^1 image feature in MZmine (Bin size = 0.02). 12
Figure S 10: Histogram of the TIC distribution of all MS^2 spectra. 12
Figure S 11: Exemplary ion image and MS^2 spectrum of a low intensity feature. 13
Figure S 12: H&E staining of a parasagittal section of rat brain cerebellum 14
Figure S 13: Raster modes for SIMSEF scheduling and acquisition. 14
Table S 1: Overview of time and feature statistics of four SIMSEF measurements on parallel tissue thin sections. 15
Table S 2: Summary of calculated quality criteria across four replicate SIMSEF experiments. BPI describes the base peak intensity. 16
References 16

Supplementary Note S1: SIMSEF experiment.

1. In flexImaging 7.2, create the measurement region and use the "Save imaging run as…" option, to save the geometry.
2. Restart timsControl 4.1 and recalibrate the instrument to make the geometry available for MS^2 acquisition.
3. Acquire the MS^1 data and process it in MZmine.
4. Schedule the SIMSEF experiment. The "Data location" must also exist on the acquisition computer and the schedule must be placed there.
5. Select the geometry of the MS^1 acquisition in timsControl.
6. Activate your MS^2 method.
7. Run the SIMSEF acquisition.

Most up-to-date information for the SIMSEF workflow is captured in the MZmine documentation:
https://mzmine.github.io/mzmine_documentation/workflows/simsef/simsef_workflow.html

Supplementary Note S2: Configuring the SIMSEF-MS^2 acquisition.

Number of MS^2 spectra (N_{MS2}) sets the desired number of MS^2 events to be scheduled per precursor and collision energy. Therefore, the total number of desired MS^2 spectra per feature is $N_{CE} * N_{MS2}$.
Collision energies (CE; defines the number of CE as N_{CE}) lists the collision energies to acquire MS^2 spectra for every precursor.
Minimum distance of MS^2 pixels defines the spatial pixel-based distance between MS^2 events of the same precursor ion and with the same collision energy.
Minimum MS^1 intensity describes a relative and absolute intensity threshold for the precursor in an MS^1 pixel to be selected for MS^2 experiments. Specified as absolute and relative parameter, which will use the larger of the two values regarding the feature.
Minimum purity score defines an exclusion criterion for scheduling MS^2 events in a pixel, in case the quadrupole isolation would lead to chimeric MS^2 spectra composed of multiple precursor ions (Figure S 2).
Quadrupole switch time (ms) defines the assumed switch time of the quadrupole. This influences how close multiple precursors will be scheduled. Low values may lead to co-isolation and overlapping of precursor values.
m/z isolation width sets the quadrupole isolation window in the instrument parameters and influences the purity score calculation.
Minimum mobility window and **maximum mobility window** describe the minimum and maximum length of a precursor isolation width in the mobility dimension. The features detected mobility range will be cropped or extended to be within the minimum and maximum mobility window.
MS2 acquisition mode defines if one or three MS^2 sub-pixels are scheduled per MS^1 pixel (Figure S 13).

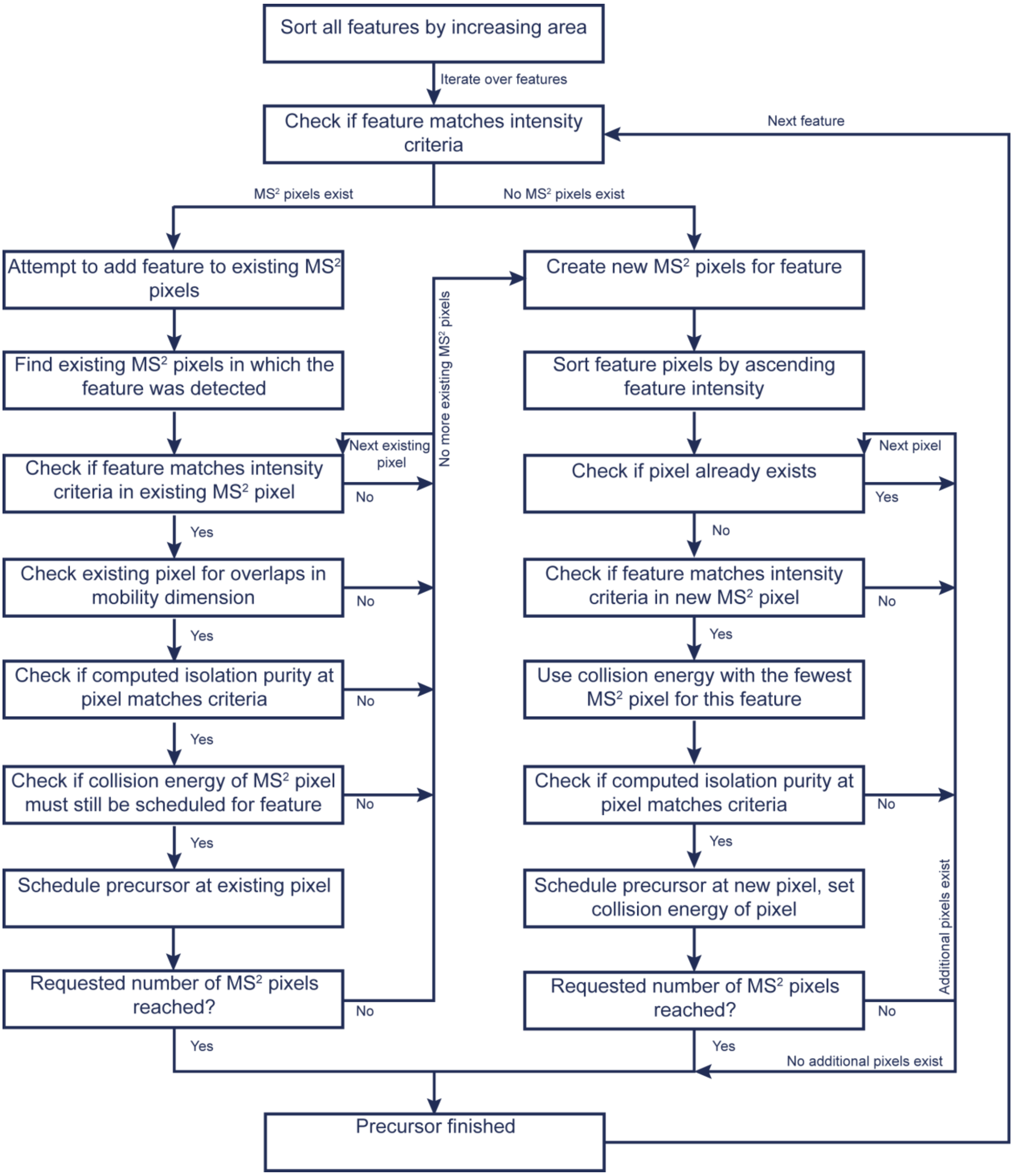

Figure S 1: Flow diagram of the SIMSEF algorithm.

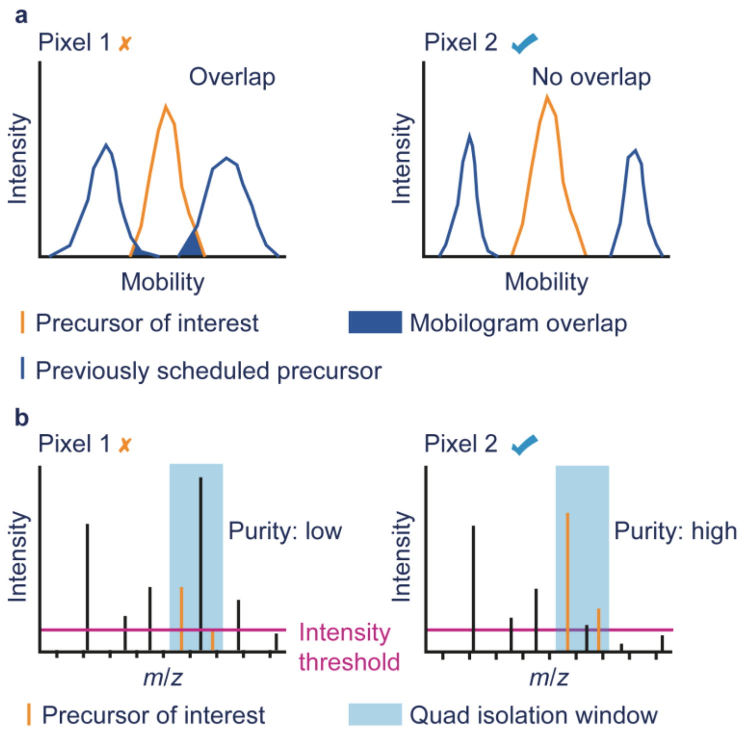

Figure S 2: Spectral scheduling procedure of the SIMSEF algorithm. The SIMSEF scheduler assesses multiple spectral criteria, before scheduling a precursor for MS^2 acquisition in a particular spot. **a**, the mobility window of the precursor is examined regarding overlaps with previously scheduled precursors. In case an overlap is detected, the precursor will not be scheduled at this spot. **b**, the spot selection for precursor ions assesses the expected purity of the quadrupole *m*/*z* isolation window within the mobility range of the precursor. The pixel will only be considered, if the intensity of the precursor exceeds the intensity threshold (magenta) and passes a spectral purity test in the mobility-resolved MS^1 spectra it was detected in.

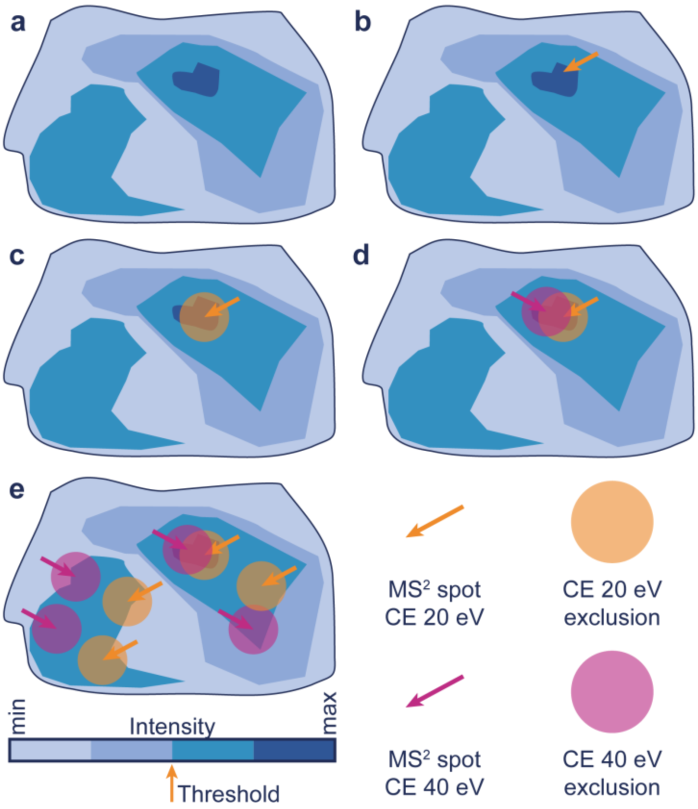

Figure S 3: Distribution of multiple MS^2 spots per precursor and collision energy. Spatial precursor selection aims to schedule spectra from the same precursor across the sample in the most abundant regions, while also distributing it across the tissue. a, in case no MS2 spot has been created previously, the pixels of an ion image are sorted by intensity. b, a precursor spot is selected for a collision energy. c, a spatial exclusion range is created for that respective energy and precursor (orange). d, multiple MS2 events for the same precursor may be scheduled within the same spatial area if the collision energy varies (magenta, orange). e, MS2 events with repeating collision energies are scheduled outside the exclusion ranges to reach the set number of MS2 events per collision energy. Every spot must meet the quality criteria described in Figure S 2.

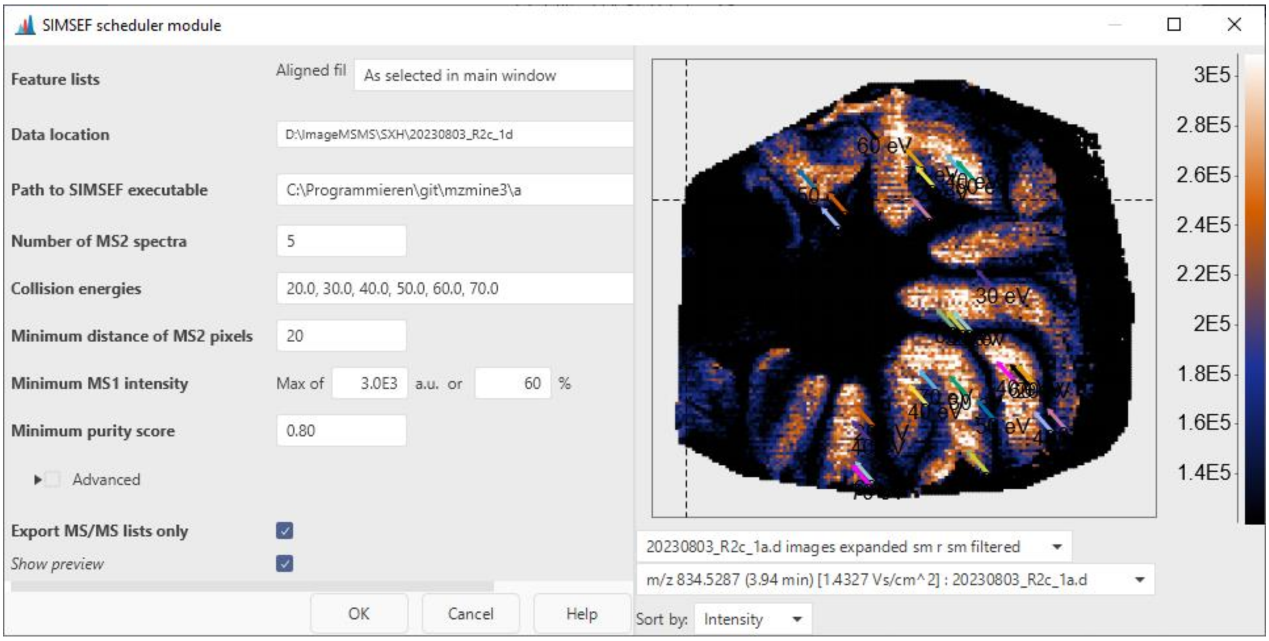

Figure S 4: Screenshot of the SIMSEF scheduling dialog from the corresponding MZmine module. The interactive preview shows the scheduled MS^2 spots for a selected precursor ion with location and fragmentation energy. SMART notation for images: Step size: 50 µm, spot size: 30 µm, resolution: 40,000 FWHM @*m*/*z* 1,221, Time 48 min (R2c_1a).

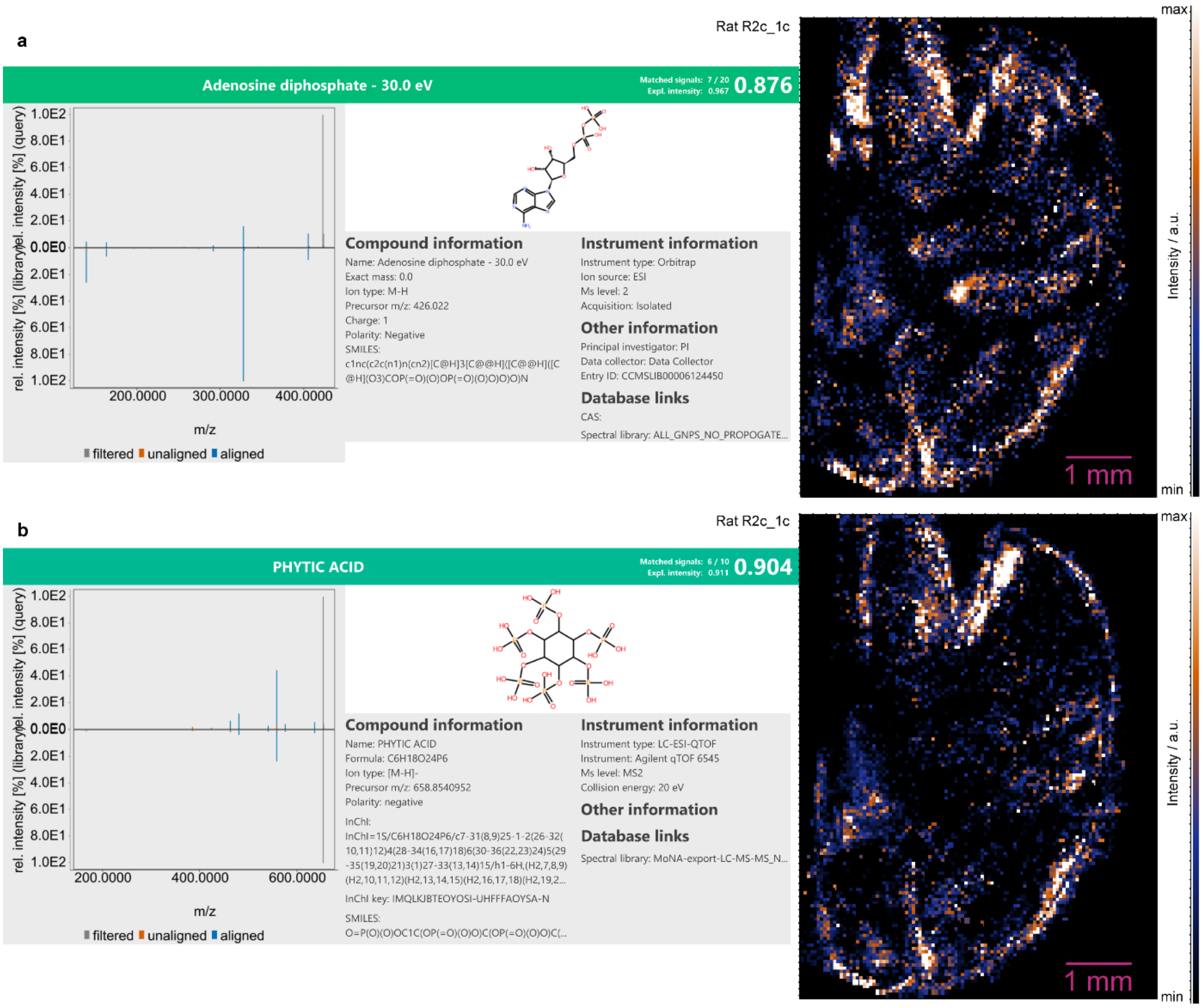

Figure S 5: Additional spectral library matches of small metabolites. **a**, Adenosine diphosphate and **b**, Phytic acid and their spatial distributions. All other annotated features were exported with the batch graphical export module in MZmine. The images, ion mobilogram plots, and spectral matches are collected in the supplementary file *features_summary.zip*. SMART notation for images: Step size: 50 µm, spot size: 30 µm, identification confidence: MSI level 2, resolution: 40,000 FWHM @*m/z* 1,221, Time 40 min (R2c_1c).

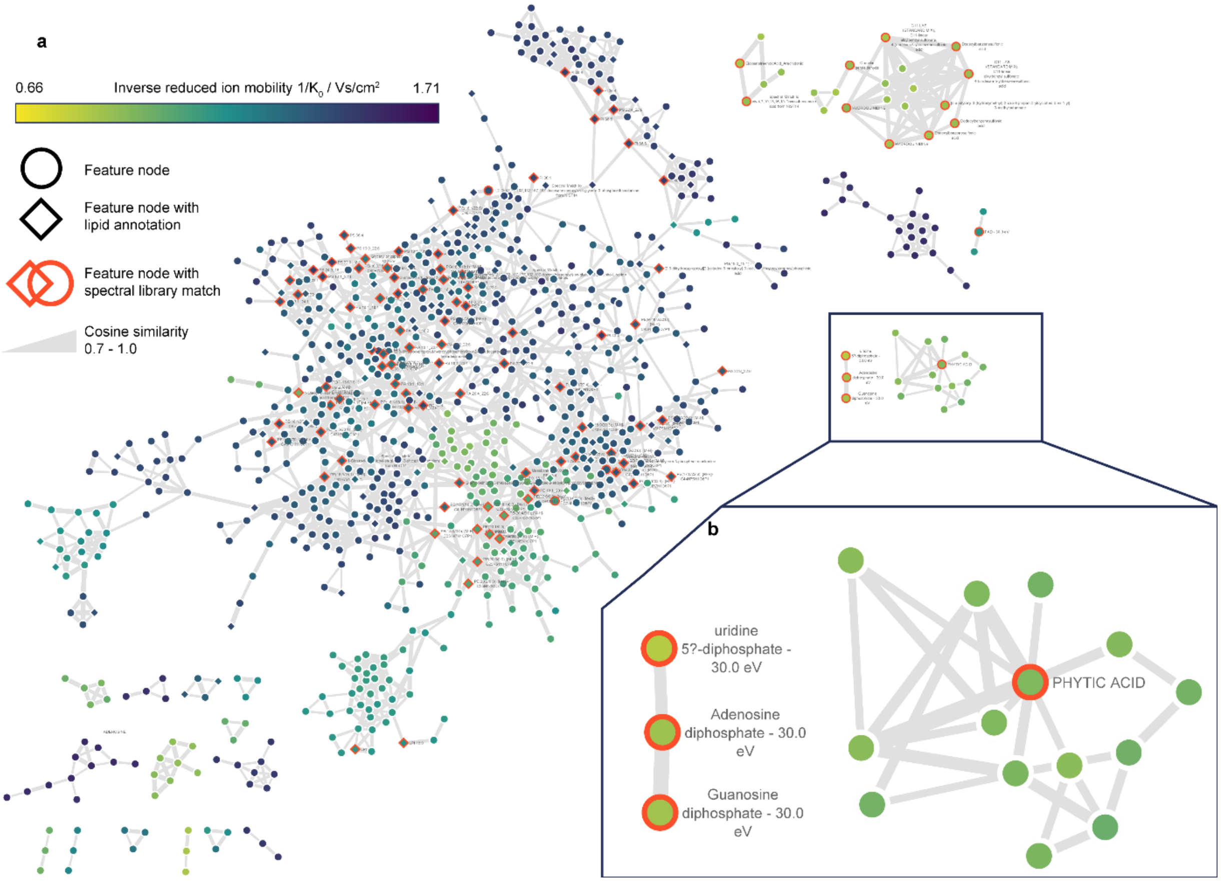

Figure S 6: The same FBMN from Figure 4, but nodes coloured by their feature’s ion mobility. Many subnetworks and communities fall into similar ion mobility ranges, signalling the presence of similar compounds classes, e.g., LPEs and LPAs with lower ion mobilities and PSs, PIs with higher ion mobilities.

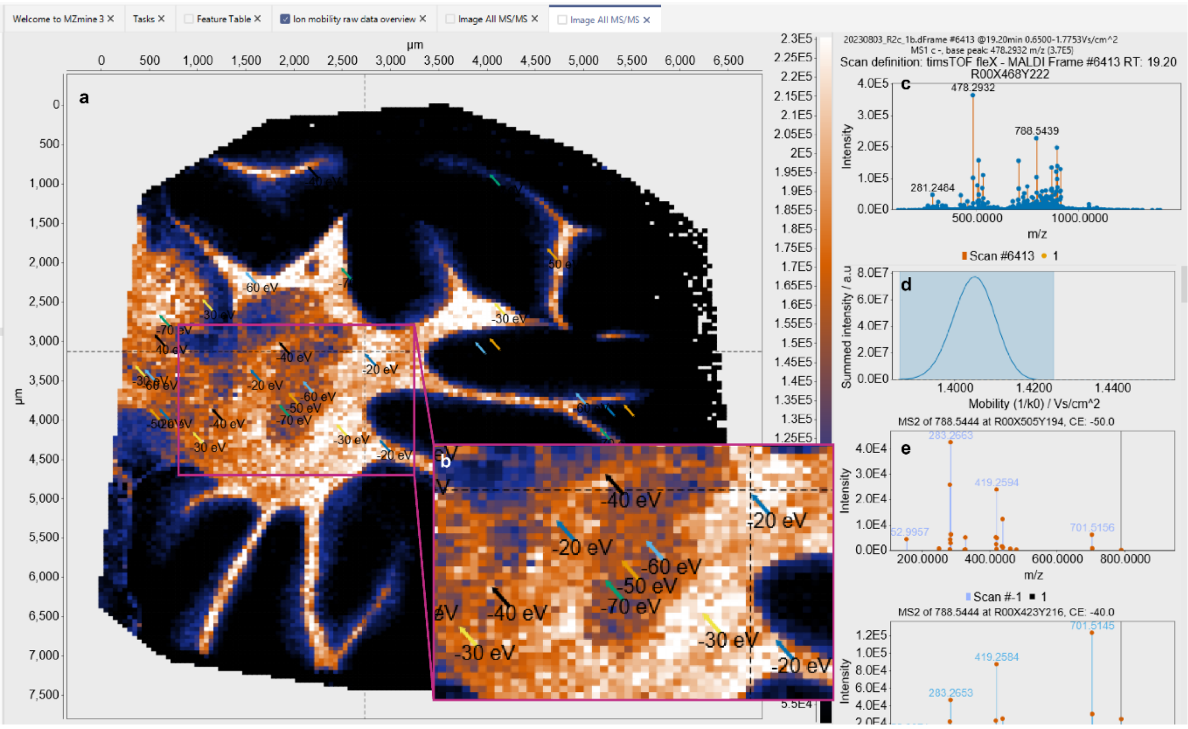

Figure S 7: Imaging specific All MS/MS visualiser in MZmine 3. **a**, the visualiser shows the spatial distribution of an image feature. **b**, the MS^2 spots are indicated by colour-coded arrows, each colour represents a different collision energy. **c**, the MS^1 spectrum at a selected pixel. **d**, the mobilogram of the image feature, and **e**, the assigned MS^2 spectra.

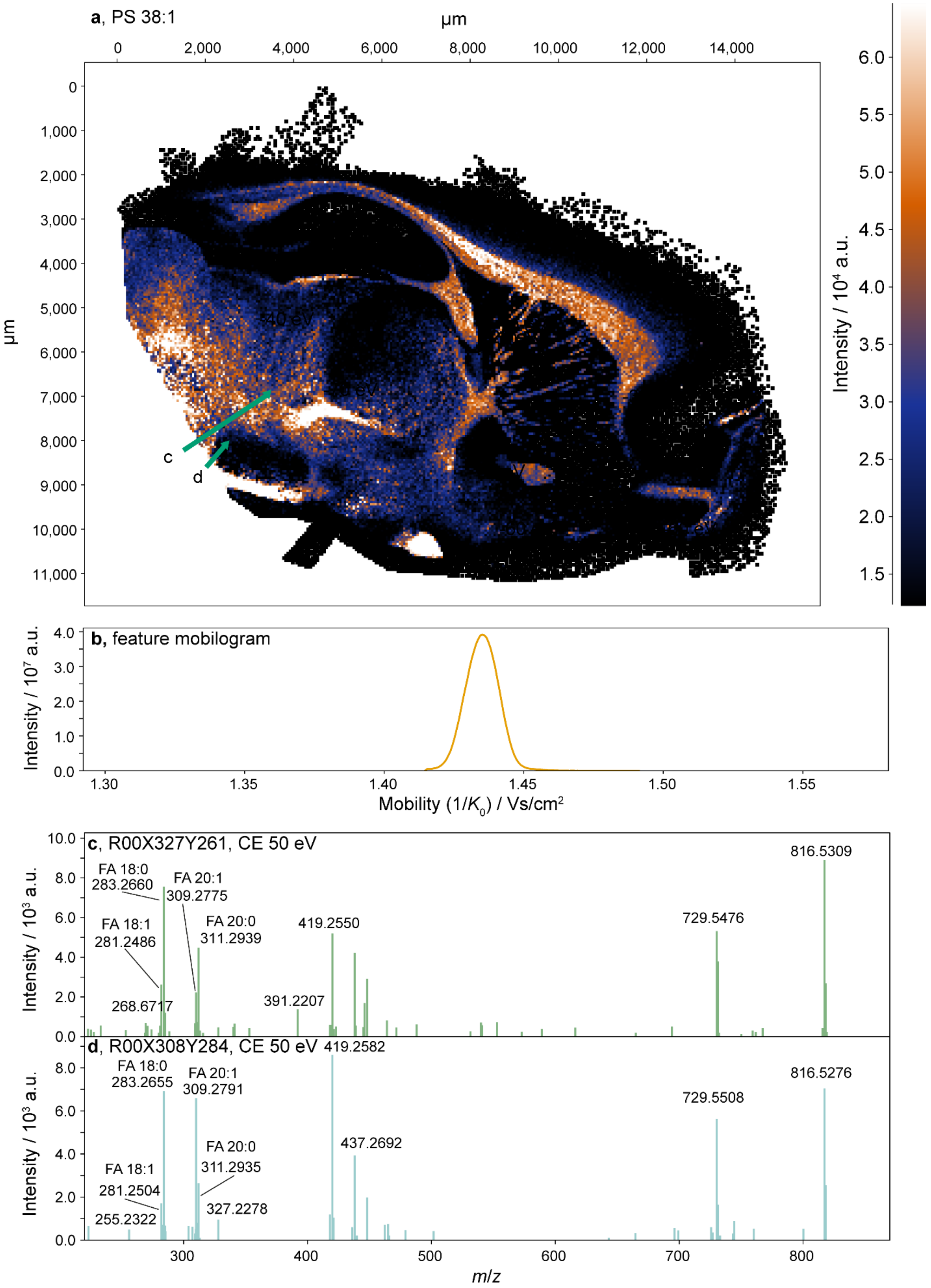

Figure S 8: SIMSEF data allows recognition of chimeric precursor features by annotating multiple compounds and lipid species in the same MS² scans. Spatial distribution of *m/z* 816.5726 ± 0.01, mobilogram of the precursor feature, and two MS² spectra, which were annotated as PS 38:1. **a**, ion image of *m/z* 816.5726 ± 0.01 in frontal sections of rat cerebrum, prepared analogous to the main manuscript. Green arrows indicate the spots of two MS²

spectra acquired with a collision energy of -50 eV (c, d). **b**, the IMS-MS^1 imaging mobilogram of the precursor ion shows no indication for the presence of multiple isomers or other interferences. **c** and **d**, chimeric MS^2 spectra acquired in spots **c** and **d** contain fragment patterns of multiple lipid species. The observed fragment ions imply that multiple isomeric lipids with varying FA composition are present and overlap in their spatial distribution and ion mobility. Replicate MS^2 at different locations may indicate varying compositions of the interfering compounds. The user-defined list of fragmentation energies used by SIMSEF may guide follow-up studies investigating this precursor by full MS^2 image acquisition to better differentiate between the inhomogeneous distribution of the interfering ion species.

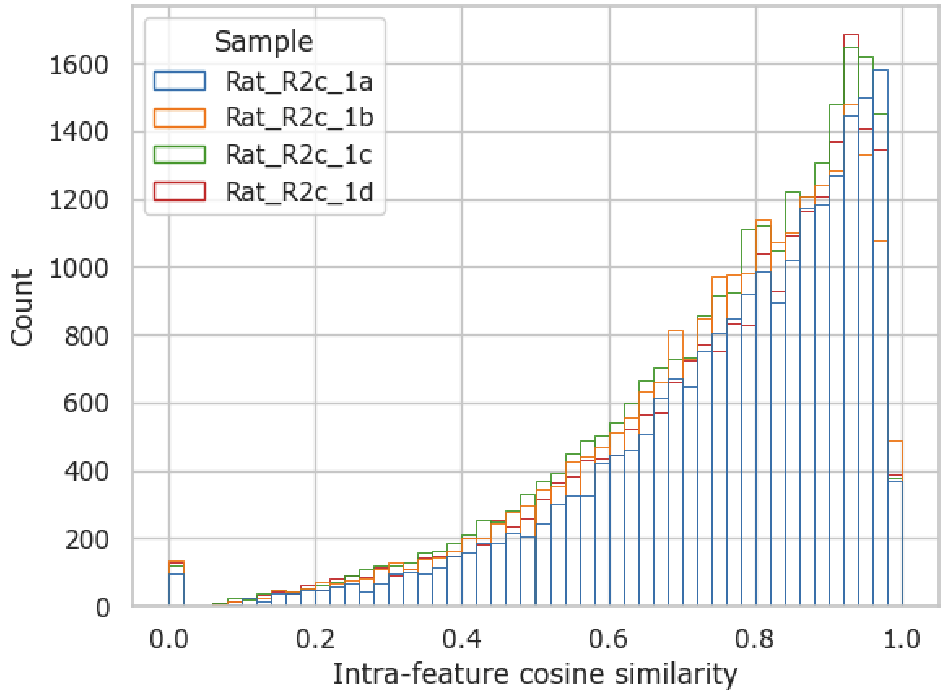

Figure S 9: Distribution of the cosine similarity of multiple MS² spectra assigned to the same TIMS-MS¹ image feature in MZmine (Bin size = 0.02). Only MS² spectra of the same collision energy were scored against each other. Spectra were only scored if at least two spectra of the same collision energy were available. In total, 63,963 of 93,950 MS² pairs had a cosine similarity of ≥0.7 (68 %).

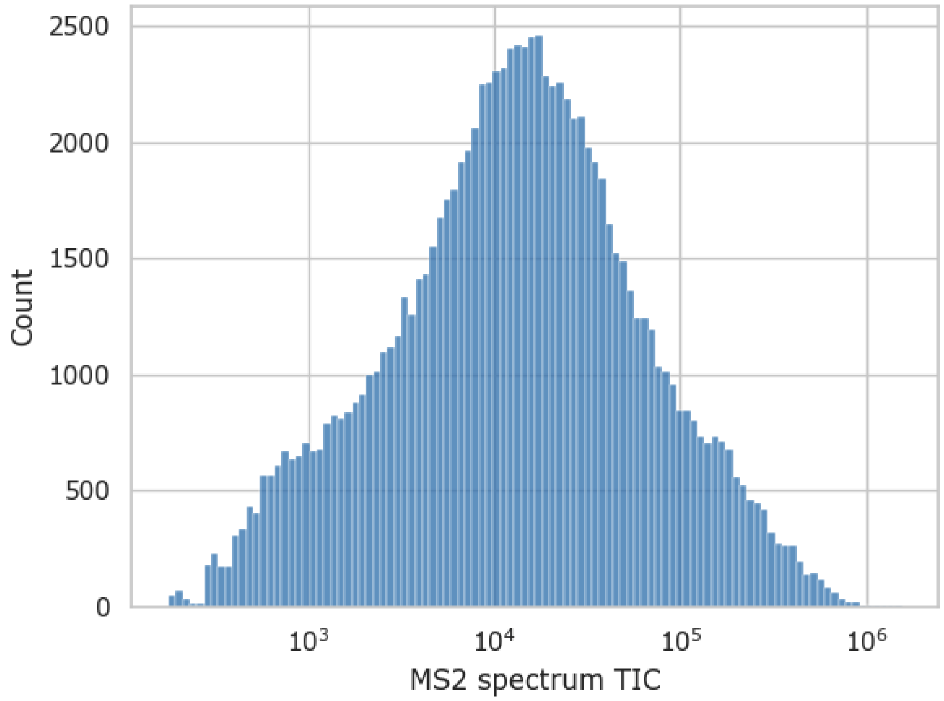

Figure S 10: Histogram of the TIC distribution of all MS² spectra. Signals below the noise level of 100 were removed from MS². An intensity of 1,000 (10x noise level) was used as a quality criterion for the base peak intensity in Supplementary Table S2.

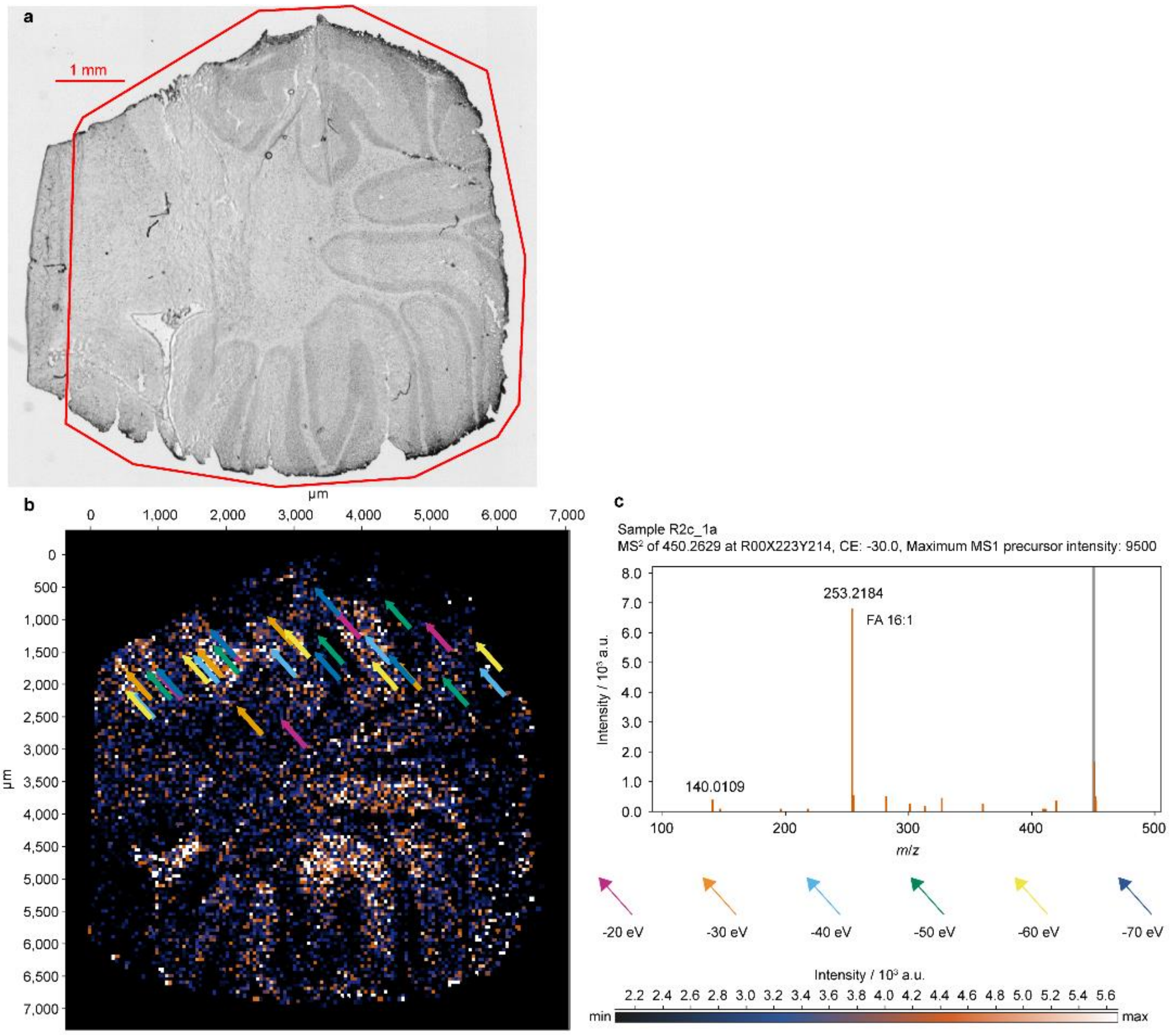

Figure S 11: Exemplary ion image and MS^2 spectrum of a low intensity feature. **a**, bright-field microscopic image of tissue section R2c_1a with the measurement region in red. **b**, distribution of the feature which was annotated as LPE 16:1 by rule-based lipid annotation. The arrows point to pixels in which MS^2 spectra were acquired. **c**, the MS^2 spectrum shows the FA 16:1 fragment signal, indicating the fatty acyl chain. Noise signals below an intensity of 100 were not removed from the spectrum to demonstrate the achieved signal-to-noise ratio. SMART notation for images: Step size: 50 µm, spot size: 30 µm, identification confidence: MSI level 3, resolution: 40,000 FWHM @*m/z* 1,221, Time 48 min (R2c_1a).

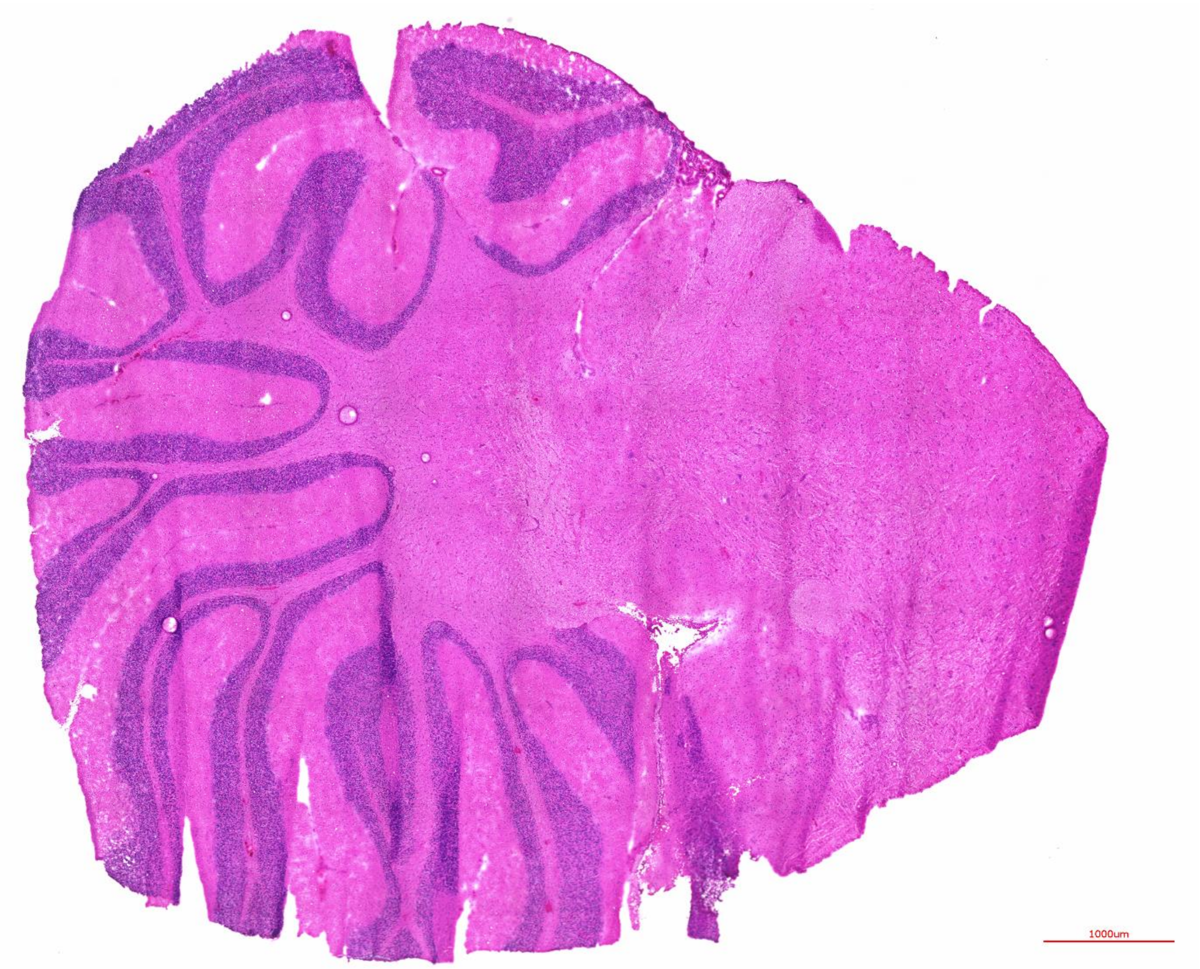

Figure S 12: H&E staining of a parasagittal section of rat brain cerebellum. The section was parallel to the sections used for MALDI-TIMS-MS imaging.

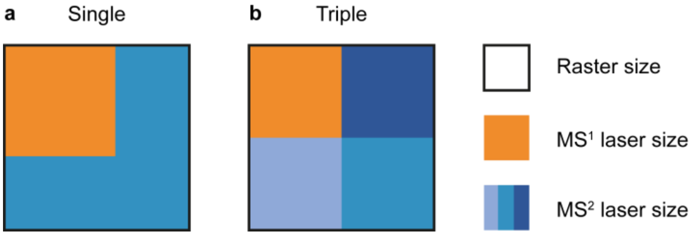

Figure S 13: Raster modes for SIMSEF scheduling and acquisition. In single spot mode **a**, the MS^2 laser spot size corresponds to the raster size of the MS^1 imaging experiment, while the MS^1 laser spot size is smaller than the raster. In triple spot mode **b**, the MS^2 laser size is equivalent to the MS^1 laser size, whilst the laser size is half of the raster size, allowing three MS^2 pixels per MS^1 pixel.

Table S 1: Overview of time and feature statistics of four SIMSEF measurements on parallel tissue thin sections.

	R2c_1a	R2c_1b	R2c_1c	R2c_1d
**MS^1 pixels**	16,161	16,765	13,329	17,984
**Acquisition time / min**	48.3	50.1	39.9	53.7
**Feature detection time / min**	9.3	12.0	9.6	14.3
**MS^1 features**	1,514	1,652	1,722	1,769
**SIMSEF scheduling time / min**	1.0	1.8	1.2	2.3
**MS^2 acquisition time / min**	186.0	206.0	205.0	221.0
**MS^2 pixels**	6,477	6,835	6,722	7,192
**Acquired MS^2 pixels per hour/ 1/h**	2,089.4	1,990.8	1,967.4	1,952.6
**Individual MS^2 spectra per hour / 1/h**	9,327.7	8,667.7	9,180.3	8,733.9

Table S 2: Summary of calculated quality criteria across four replicate SIMSEF experiments. BPI describes the base peak intensity.

	R2c_1a	R2c_1b	R2c_1c	R2c_1d
**Features – total**	1,514	1,652	1,722	1,769
**Features – scheduled (matching SIMSEF criteria)**	1,396	1,514	1,560	1,640
**MS^2 – total**	28,916	29,759	31,366	32,170
**MS^2 – non-empty scans**	23,444	24,813	26,076	25,705
**MS^2 – at least 4 signals and BPI 10x S/N**	17,887	19,126	19,537	19,599
**“good” MS^2 – all the above and BPI / TIC < 0.5**	12,540	13,481	13,555	13,867
**Features – at least one non-empty MS^2**	1,329	1,426	1,556	1,460
**Features – at least one "good" MS^2**	1,105	1,219	1,262	1,236
**Features – “good” MS^2 / Features with non-empty MS^2**	83%	85%	81%	85%
**Spectral library matches**	100	121	118	101
**Lipid annotation**	192	223	208	212
**Annotated features**	208	255	238	232
**Annotation rate**	14%	15%	14%	13%

References

1. Schuhmann, K. *et al.* Quantitative Fragmentation Model for Bottom-Up Shotgun Lipidomics. *Anal. Chem.* **91**, 12085–12093 (2019).
